# Comprehensive analysis of the mouse renal cortex using two-dimensional HPLC – tandem mass spectrometry

**DOI:** 10.1186/1477-5956-6-15

**Published:** 2008-05-23

**Authors:** Yingxin Zhao, Larry Denner, Sigmund J Haidacher, Wanda S LeJeune, Ronald G Tilton

**Affiliations:** 1 Department of Internal Medicine, The University of Texas Medical Branch, Galveston, TX, USA; 2 Stark Diabetes Center, The University of Texas Medical Branch, Galveston, TX, USA; 3 McCoy Diabetes Mass Spectrometry Research Laboratory, The University of Texas Medical Branch, Galveston, TX, USA; 4 Sealy Center for Molecular Science, The University of Texas Medical Branch, Galveston, TX, USA

## Abstract

**Background:**

Proteomic methodologies increasingly have been applied to the kidney to map the renal cortical proteome and to identify global changes in renal proteins induced by diseases such as diabetes. While progress has been made in establishing a renal cortical proteome using 1-D or 2-DE and mass spectrometry, the number of proteins definitively identified by mass spectrometry has remained surprisingly small. Low coverage of the renal cortical proteome as well as our interest in diabetes-induced changes in proteins found in the renal cortex prompted us to perform an in-depth proteomic analysis of mouse renal cortical tissue.

**Results:**

We report a large scale analysis of mouse renal cortical proteome using SCX prefractionation strategy combined with HPLC – tandem mass spectrometry. High-confidence identification of ~2,000 proteins, including cytoplasmic, nuclear, plasma membrane, extracellular and unknown/unclassified proteins, was obtained by separating tryptic peptides of renal cortical proteins into 60 fractions by SCX prior to LC-MS/MS. The identified proteins represented the renal cortical proteome with no discernible bias due to protein physicochemical properties, subcellular distribution, biological processes, or molecular function. The highest ranked molecular functions were characteristic of tubular epithelium, and included binding, catalytic activity, transporter activity, structural molecule activity, and carrier activity. Comparison of this renal cortical proteome with published human urinary proteomes demonstrated enrichment of renal extracellular, plasma membrane, and lysosomal proteins in the urine, with a lack of intracellular proteins. Comparison of the most abundant proteins based on normalized spectral abundance factor (NSAF) in this dataset versus a published glomerular proteome indicated enrichment of mitochondrial proteins in the former and cytoskeletal proteins in the latter.

**Conclusion:**

A whole tissue extract of the mouse kidney cortex was analyzed by an unbiased proteomic approach, yielding a dataset of ~2,000 unique proteins identified with strict criteria to ensure a high level of confidence in protein identification. As a result of extracting all proteins from the renal cortex, we identified an exceptionally wide range of renal proteins in terms of pI, MW, hydrophobicity, abundance, and subcellular location. Many of these proteins, such as low-abundance proteins, membrane proteins and proteins with extreme values in pI or MW are traditionally under-represented in 2-DE-based proteomic analysis.

## Background

The essential role of kidneys in normal physiology, including plasma filtration of metabolic waste products, acid-base balance, regulation of plasma volume, and hormone secretion, is indicated by the large number of diverse, life threatening renal diseases. Plasma filtration and much of the tubular reabsorption takes place in the renal cortex, an important functional component of the kidney between the renal capsule and renal medulla, consisting of glomeruli, proximal and distal tubules. Of all renal pathologies, diabetic nephropathy (DN) has become the most common cause of renal insufficiency culminating in end-stage renal failure in the western world [1]. Although DN has been considered a predominantly glomerular disorder, recent studies have demonstrated the importance of tubulointerstitial changes [2-5]. These findings suggest that previous approaches focused on either glomeruli or tubules are insufficient for a global understanding of the pathophysiology of complicated renal diseases such as DN.

Given the importance of the kidney in normal and disease states, numerous proteomic methodologies increasingly have been applied to the kidney, and novel combinations of research tools are now available to identify global changes in renal protein expression patterns induced by diseases such as diabetes. This is highly relevant since the involvement of proteins and the molecular functions they control is a common denominator in every mechanism invoked to explain diabetes-induced tissue injury [6-9]. The majority of these investigations has focused on the identification and quantification of proteins found in urine [10-15], primarily to identify potential biomarkers of renal disease [16]. This reflects the non-invasive nature of the sample collection, its availability, and the observation that proteins found in urine under pathophysiological conditions will reflect altered glomerular and tubular pathology induced by renal disease [17]. Progress has been made in establishing a renal cortical proteome using 1-D or 2-DE and mass spectrometry [18], including proteomes of renal cortex [19,20], glomerular cells [21-24], tubular epithelial cells [25-27], and collecting duct cells [28]. While thousands of spots can be visualized using 2-DE, the number of proteins definitively identified by mass spectrometry has remained surprisingly small [29].

The coverage of renal cortical proteins has been significantly improved by fractionating renal cortex tissue into functional units comprising glomeruli or tubules [21,30,31]. Using this approach, Miyamoto *et al *[21] identified 6,686 unique proteins (one half were identified with one peptide) representing ~3,000 distinct genes from purified glomeruli obtained from one human kidney. Here, we have employed an alternative fractionation strategy to enhance coverage of the renal proteome. We enzymatically digested proteins extracted from the entire renal cortex, then separated the resulting complex peptide mixture into 60 fractions with strong cation exchange media. Each fraction was further resolved by reversed-phase media prior to sequencing by LC-MS/MS. We elected to use this approach since tissue (or subcellular) fractionation and purification results in the loss of important extracellular and interstitial renal cortical proteins, and our goal was to identify the entire renal cortical proteome rather than to describe what might be unique between different functional components or cell types within the renal cortex. While renal fractionation into functional units has been shown to improve the proteome coverage, it does not diminish the complexity of renal protein mixtures (glomeruli are composed of multiple cell types, including blood cells and plasma, endothelial and vascular smooth muscle cells, mesangial cells, podocytes, infiltrating leukocytes, etc.). Our renal fractionation strategy has been widely used previously [32-34], it can be very complementary to the proteomes generated by fractionating the renal cortex into major functional components, and our more integrative approach using the whole renal cortex can provide important insights into the pathophysiology of disease processes affecting the entire renal cortex

In this study, we elected to identify the proteome of the entire renal cortex tissue by using an unbiased 2D-LC-MS/MS approach. 2D-LC-MS/MS is a well-documented methodology for analyzing the proteome of complex biological systems. Using this approach, Wasburn *et al*. identified 1,484 proteins from the *S. cerevisiae *proteome [35], and Peng *et al*. [36], using offline two-dimensional chromatography coupled with tandem mass spectrometry, identified 1,504 yeast proteins from *S. cerevisiae *from 1 mg of total protein. Here, we have identified ~2,000 proteins from the renal cortex. We used a semi-quantitative approach to determine the relative abundance of the identified proteins, and report a wide dynamic range of 4 orders of magnitude, with many low-abundance, tubule-related proteins present in our dataset. We further demonstrate that the inter-animal variability in identified proteins can be accounted for by the inherent variability in the mass spectrometry instrumentation.

## Methods

### Animal and surgical protocols

Male C57BLKS/J mice were purchased from Jackson Labs (Bar Harbor, Maine) and were housed 2–3/cage in a sterile environment, in a room with a 12 hour light cycle and free access to standard chow and water in the UTMB Animal Resource Center in accordance with its IACUC policies and the Public Health Service Guide for the Care and Use of Laboratory Animals. At 5 months of age, mice were anesthetized (ketamine/xylazine; 70/10 mg/kg i.p.), anti-coagulated (5 units heparin), then exsanguinated prior to rapid aortic perfusion with ice-cold PBS to quickly rinse kidneys free of blood and to deliver protease (10 μg/ml pepstatin A, 10 μg/ml leupeptin, 10 μg/ml STI, 1 mM PMSF, 10 μg/ml aprotinin) and phosphatase (1 mM orthovanadate and 30 mM sodium fluoride) inhibitors, and DTT (0.5 mM). Both kidneys were removed, decapsulated, flash-frozen in liquid nitrogen, then store at -80°C until processed for protein extraction.

### Tissue processing, protein extraction and tryptic digestion

From each mouse, ~50 mg of kidney cortical tissue were separated from the medullary portion of each kidney under magnification with a dissecting microscope and suspended in 20 fold excess (wt/vol) of TRIzol^® ^reagent (Invitrogen, Carlsbad, CA). The tissue was homogenized with 20 stokes of a 1 ml Dounce homogenizer on ice. The proteins were extracted from the lysate according to the manufacturer's instructions, and the protein pellet was dissolved in 250 μl of 8 M guanidinium HCl. The mixture was diluted with 2250 μl of 100 mM ammonium bicarbonate, and digested with 40 μg of trypsin overnight at 37°C. The tryptic peptide mixture was desalted with a Sep-Pak^® ^C18 cartridge (Waters, Milford, MA) following the manufacturer's instructions. Peptides were eluted from the cartridge with 80% acetonitrile and completely dried using a speedvac.

### Two-dimensional liquid chromatography with tandem mass spectrometry (2D LC-MS/MS)

The dried peptide samples were redissolved by adding 20 μl of acetonitrile and diluted with 100 μL of 5 mM ammonium formate, pH 2.7. The peptide mixture was loaded onto a SCX column (4.6 mm × 25 cm; Poly LC, Columbia, MD) and separated with a linear gradient from 100% buffer A (5 mM ammonium formate-20% acetonitrile, pH 2.7) to 25% buffer B (1 M ammonium formate-20% acetonitrile, pH 3.0) over 40 min at a flow rate of 0.8 ml/min and followed by a linear gradient from 25% buffer B to 60% buffer B over 20 min. The eluate was manually collected after the first salt gradient was started. Sixty fractions corresponding to 0.8 ml each were collected and dried using a Speedvac. Each SCX fraction was redissolved in 20 μl of 0.1% TFA and was injected onto a C18 peptide trap (Agilent, Santa Clara, CA), desalted with 0.2% formic acid at a flow rate of 10 μl/min for 180 min. Peptides were eluted from the trap and separated on a reversed phase nano-HPLC column (PicoFritTM, 75 μm × 10 cm; tip ID 15 μm) with a linear gradient of 0–50% mobile phase B (0.1% formic acid-90% acetonitrile) in mobile phase A (0.1% formic acid) over 120 min at 200 nl/min. LC-MS/MS experiments were performed with a LTQ linear ion trap mass spectrometer (ThermoFinnigan, San Jose, CA) equipped with a nanospray source; the mass spectrometer was coupled on-line to a ProteomX^® ^nano-HPLC system (ThermoFinnigan, San Jose, CA). The mass spectrometer was operated in the data-dependent mode using Xcalibur software. The most intense seven ions in each MS survey scan were automatically selected for MS/MS.

### Data processing

The acquired MS/MS spectra were searched with SEQUEST algorithm against a composite target-decoy mouse protein database consisting of the protein sequences (target) downloaded from SWISSPROT mouse protein database (downloaded July 2006) and reversed versions of these sequences (decoy). All SEQUEST searches were performed on the Bioworks 3.2 platform (ThermoFinnigan, San Jose, CA) using the followed parameters: fully tryptic peptide (both tryptic terminus for all peptides), a mass tolerance of ± 2.0 Da for precursor ion and ± 1.0 Da for fragment ion. The output data from these searches were filtered and sorted by the DTASelect software [37]. Only the top one peptide sequence match to each acquired MS/MS spectrum was considered. The criteria used in DTASelect were as follows: First, relatively conservative criteria (Sp ≥ 300; ΔCn ≥ 0.12; Xcorr of 1.9, 2.0 and 3.0 for data from a singly, doubly or triply charged precursor ion, respectively) were applied. Second, proteins that passed these thresholds were separated into two groups: proteins identified with more than one peptide and proteins identified with one peptide. Third, since the majority of the false positive identifications in our dataset were within the group of proteins with one identified peptide, much stricter criteria (Xcorr 2.2, 3.2, or 3.75 for precursor charge states of 1+, 2+, or 3+, respectively) were applied to the peptide hits in this group to increase identification certainty. Forth, if multiple spectra were identified to match precisely the same sequence and charge state, only the spectrum with the highest Xcorr was retained. Finally, proteins that shared all matched peptides with other (homologous) proteins were removed. The false positive rate (FPR) of identification was estimated as described [38-40]. Briefly, the number of false positive identification (FP) was estimated by doubling the passing decoy assignments; the number of true positive identifications (TP) was estimated by subtracting the FP from the total passing assignment. The FPR was estimated by using the followed equation: FPR = FP/(FP+TP).

The normalized spectral abundance factor (NSAF) value for each protein was calculated as described [41],

$${\left(\text{NSAF}\right)}_{k}=\frac{{\left(SpC/L\right)}_{k}}{\sum_{i=1}^{N}{\left(SpC/L\right)}_{i}}$$

in which the total number of tandem MS spectra matching peptides from protein *k *(*SpC*) was divided by the protein length (*L*), then divided by the sum of *SpC/L *for all uniquely identified proteins in the dataset.

## Results and Discussion

### Reproducibility

**Figure 1:**
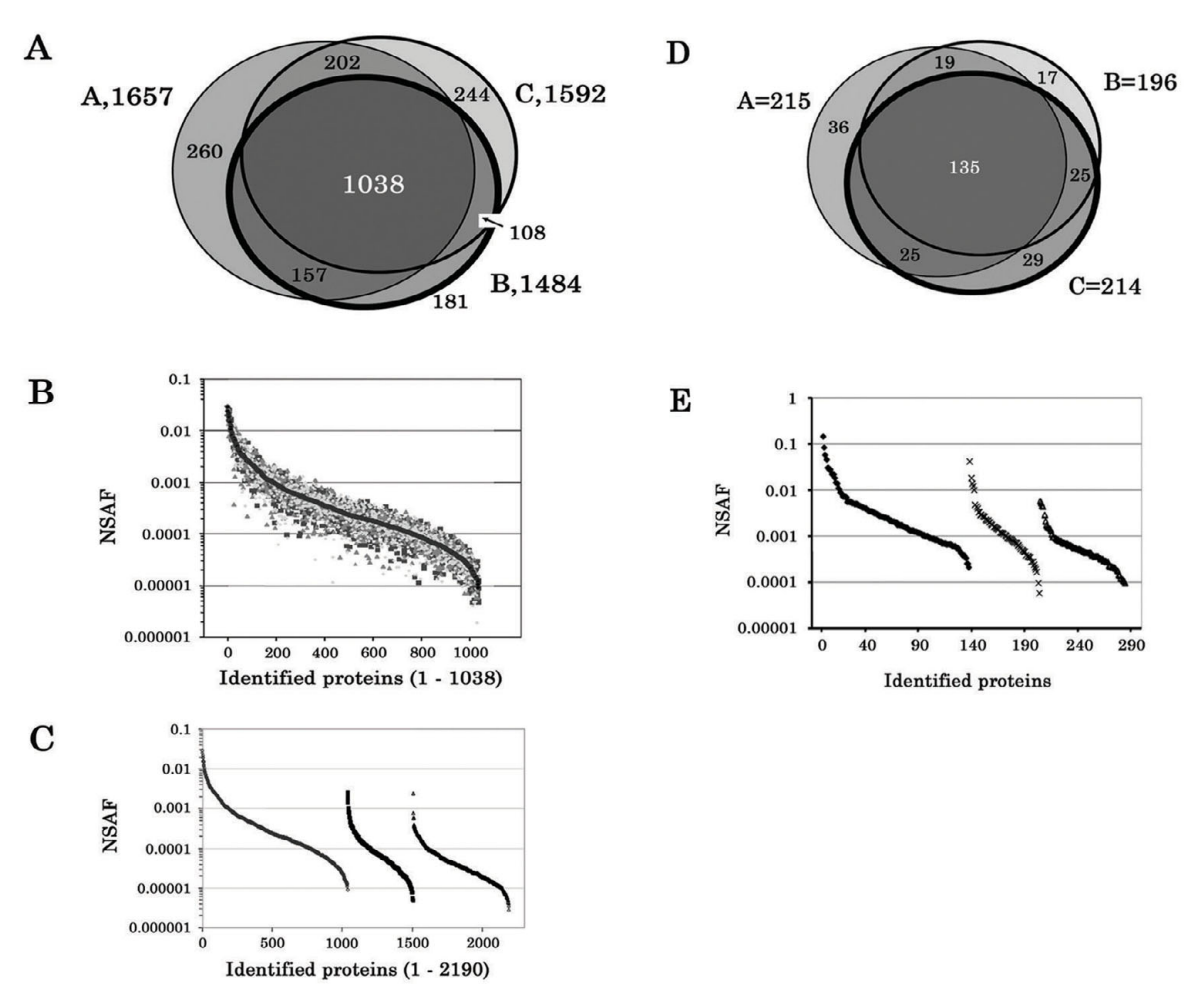
Reproducibility of protein identification between individual mice. **A**. Renal cortical proteins isolated from three mice, were extracted, digested with trypsin, separated by SCX into 60 fractions, and for each mouse, 14 consecutive SCX fractions with retention times between 20 and 34 min were selected for LC-MS/MS analysis. **B**. The NSAF values of the proteins identified as common in all three mice. ■, NSAF values of the proteins identified from mouse A; ▲, NSAF values of the proteins identified from mouse B; ◆, NSAF values of the proteins identified from mouse C; and ●, the mean of the NSAF values. **C**. The NSAF values of the proteins identified from all of the three mice. ◇, the mean of the NSAF values of the proteins identified as common in all three mice; ■, the mean of the NSAF values of the proteins identified in two out of three mice; △, the NSAF values of the protein identified in only one mouse. **D**. To examine reproducibility of the 2D-LC-MS/MS method, we analyzed one SCX fraction by LC-MS/MS three times. **E**. The NSAF values of the proteins identified from all of the three LC-MS/MS runs. ◆, the mean of the NSAF values of the proteins identified as common in all three LC-MS/MS runs; ×, the mean of the NSAF values of the proteins identified in two out of three LC-MS/MS runs; and △, the NSAF values of the protein identified in only one LC-MS/MS run.

First, we examined the reproducibility of protein identification between individual mice. Renal cortical tissue was isolated from three mice, proteins extracted and digested with trypsin, and each of the three tryptic peptide mixtures was separated by SCX into 60 fractions. For each mouse, 14 consecutive SCX fractions with retention times between 20 and 34 min were selected for LC-MS/MS analysis, and the MS data searched against public databases using the SEQUEST algorithm as described above. We estimated the identification false positive rate (FPR) using a composite target-decoy database searching strategy described in the Experimental section. A total of 1,657, 1,484 and 1,592 proteins were identified with ~1% FPR from each mouse. In each dataset, a normalized spectral abundance factor (NSAF) value was calculated for each identified protein. If one protein was identified from more than one mouse, the NSAF values of the protein were averaged and a mean, standard deviation (SD) and coefficient of variation (CV) were calculated. The identified proteins, including SWISSPROT accession number, locus ID, protein name, number of amino acids, theoretical MW and pI, normalized spectral abundance factor (NSAF; including SD and CV of NSAF if the protein was identified in 2 or more animals), and the animal from which each protein was identified are listed [see Additional file 1]. As shown in Figure 1A, overlap between datasets was high, with reproducibility of protein identification between 72 – 82% for pairwise comparisons and 63 – 71% for all three animals. A total of 2,190 unique proteins were identified when all three datasets were combined. Normalized spectral abundance factor (NSAF) values for identified proteins were highly reproducible between individual mice. For example, a total of 1,038 proteins were identified as common in all three mice, and the distribution of their NSAF values is shown in Figure 1B. For the majority (97.6%) of these proteins, the CV of NSAF is less than 1%, indicating that the inter-animal variability in NSAF values is low.

Using protein datasets generated from the 3 mice, we used NSAF values to estimate the relative abundance of proteins that were identified as common in all three mice versus unique to an individual mouse. As shown in Figure 1C, NSAF scores were relatively lower when proteins were identified in only one mouse and relatively higher when proteins were identified in all three mice. A total of 188 proteins with averaged NSAF scores above 0.001 were identified in all 3 mice, decreasing to 6 proteins when identified in 2 out of 3 mice, and 1 protein when identified in only one mouse.

Conversely, only 1 protein identified in all 3 mice had a NSAF score lower than 0.00001, while 13 proteins were in this category when identified in 2 out of 3 mice, and 50 proteins when identified in only one mouse. These results indicate that high abundance proteins with high NSAF scores are more likely to be reproducibly identified in multiple animals.

In order to examine reproducibility of the LC-MS/MS method, we analyzed one SCX fraction by LC-MS/MS three times. As shown in Figure 1D, 135 proteins were common to each MS/MS run. Reproducibility of protein identification was 72 – 82% for pairwise comparisons and 63 – 69% for triplicate analyses. Since this overlap is in the same range for individual animals or different mass spectrometry runs, these results suggest that the variation in reproducibility in protein identifications can mostly be accounted for by the mass spectrometer, and not by variability between mice. We next compared the NSAF values of proteins that were identified as common in all three LC-MS/MS runs versus uniquely identified in individual LC-MS/MS run. As shown in Figure 1E, we found that replicate analyses of the same sample yielded similar trends to those from individual mice (Figure 1C). This further suggests that in our study, the variation in individual mice is minor and the major causes of the variation in reproducibility in protein identification are the sampling speed and the dynamic range of the mass spectrometer. Similar results have been reported by other groups who have shown that peptide mixtures extracted from one gel band and analyzed with LC-MS/MS three times results in reproducibility in peptides identified by LC-MS/MS of 60 – 70% [32,42]. More recently, it has been reported that advanced mass spectrometry methods can unambiguously identify more than 2,000 yeast proteins, the major limitation derived from a combination of dynamic range of the sample and effective sequencing speed of the mass spectrometer [43]. Our results also emphasize the need for more intelligent acquisition software and mass spectrometers with higher dynamic ranges.

### Mouse renal cortical proteome

Using a single mouse in which both kidneys were rapidly perfused with ice-cold PBS and a cocktail of protease and phosphatase inhibitors to minimize blood contamination and to preserve renal cortical protein integrity, peptides in all 60 SCX fractions were further separated and sequenced by nano reversed-phase HPLC with online tandem mass spectrometry as detailed above. Over 600,000 MS/MS spectra were acquired, then used for database searching with the SEQUEST algorithm. A total of 10,593 unique peptides were identified with ≤1% FPR, and the complete list of these sequenced peptides used to identify each protein is found in Additional file 2. The criteria used for determining the certainties of identification in this study are more stringent than those used by other investigators [44,45]. These peptides were mapped to 1,966 proteins, and the identified proteins, including SWISSPROT accession number, locus ID, gene name, protein name, number of unique peptides used in the identification, sequence coverage (%), number of amino acids, theoretical MW and pI, subcellular location, protein family, number of predicted hydrophobic α-helical transmembrane domains, and NSAF, are listed alphabetically by SWISSPROT accession number [see Additional file 3]. Of 1,966 identified proteins, 1,578 (80.3%) proteins were identified with more than one unique peptide. The number of proteins that overlapped with the original 3 datasets containing 2,190 proteins was 76% (data not shown).

We also compared an independent method using ^18^O-labeling technology [46] with data-dependent Zoomscan and MS/MS switching that doubled the duty cycle. Since we showed there was very little inter-animal variability, equal amounts of renal cortical extracts from five control mice were pooled, tryptic peptides labeled using this stable isotope method, then fractionated into 60 SCX fractions. We found 81% overlap with the 1,966 proteins identified above (data not shown). These results indicate that the identification of proteins is highly reproducible at the level of individual animals, pools from multiple animals, and different mass spectrometry methods.

### Relative abundance of identified proteins

**Figure 2:**
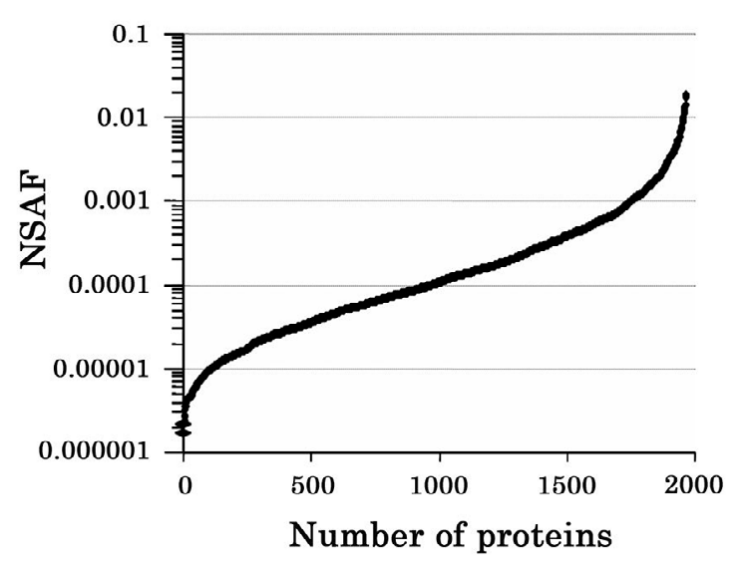
NSAF values for every protein [see Additional file 2] are shown graphically. NSAF values of identified proteins fall within the range from 1.7 × 10^-6 ^to 1.8 × 10^-2^.

We used NSAF to determine the relative abundance of the proteins identified in renal cortex. It has been well documented that abundant proteins are typically identified with multiple unique peptides from a single 2D-LC-MS/MS run and that peptides from abundant proteins are more likely to be re-identified multiple times due to "peak spreading" within the prior in-line chromatography [47]. Liu and Yates found that spectral counting correlated with relative protein abundance and could be used to estimate the abundance of a group of proteins where a large number of peptides with different properties are available [48]. This method has been shown to be an effective quantitative proteomics approach when combined with MudPIT [41,47,49,50]. Using the 1,966 proteins identified from the single mouse, DTASelect sorting and filtering yielded 49,965 MS/MS spectra for further analysis. A calculated NSAF value for each protein is shown in Additional file 3 and all values are shown graphically in Figure 2. NSAF values of identified proteins fall within the range from 1.7 × 10^-6 ^to 1.8 × 10^-2^, indicating that our approach has a wide dynamic range. More than 100 proteins have NSAF values greater than 0.002. The 105 most abundant proteins (5.3% of the total identified proteins) were identified with 24,259 MS/MS spectra, which account for 48.6% of the total of MS/MS spectra that passed DTASelect filtering. Surprisingly, approximately half of the 105 proteins are mitochondrial proteins and one third are cytoplasmic proteins, including enzymes, cytoskeleton proteins, and ribosomal proteins. Of the remaining proteins whose NSAF values are greater than 0.002, most are nuclear proteins. PDZ domain-containing protein 1 (PDZK1), a plasma membrane protein with an NSAF value of 0.0052, is highly expressed in renal proximal tubules and plays a central role in the regulation of polarized tubule cell function and the formation of regulatory complexes that provide spatial and molecular specificity to the intracellular signaling [51,52]. In the dataset reported here, low-abundance proteins such EGF receptor, IGF 2 receptor, MEKK4, MEKK5, and Sp1 have very low NSAF values (< 1 × 10^-5^), demonstrating the value of reduction of sample complexity by our multidimensional tandem mass spectrometry approach.

### Characteristics of identified proteins

**Figure 3:**
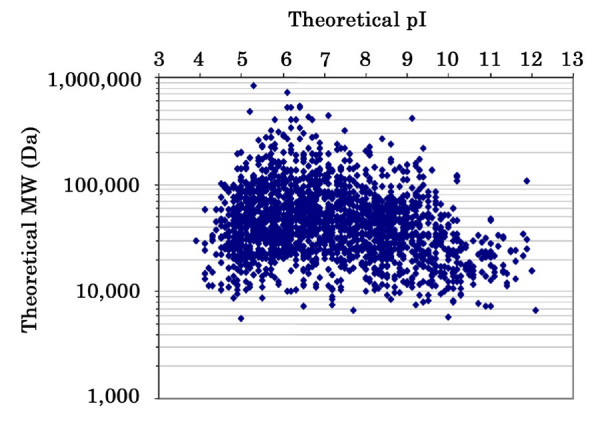
The theoretical pI and MW of identified proteins.

We next evaluated proteins based on their pI, MW, hydrophobicity, and presence of hydrophobic α-helical transmembrane domains. The theoretical pI and MW values of the 1,966 proteins identified from the single mouse renal cortex separated into 60 SCX fractions were calculated by DTASelect software. While ~60% of the identified proteins had theoretical pI values in the pH 5–9 range and theoretical MW between 20,000 to 100,000 Da, a number of proteins with extreme values in pI or MW were present in our dataset (Figure 3). For example, 32 acidic proteins with a theoretical pI of <4.5 and 122 basic proteins with a theoretical pI of >10.0 were identified; 29 small proteins with MW <10,000 Da and 242 proteins with MW > 150,000 Da were identified. These proteins represent a population of proteins that are rarely identified by 2-DE.

A number of proteins with hydrophobic α-helical transmembrane domains were present in our dataset. To locate the α-helical transmembrane domains, the set of identified proteins was searched with the SOSUI transmembrane prediction algorithm [53-55]. A total of 348 proteins contained at least one predicted hydrophobic α-helical transmembrane domain (listed in Additonal file 3). Out of the 348 proteins, 120 were identified as plasma membrane proteins by Ingenuity Pathway Analysis [56] as receptors, transporters, ion channels, etc. A number of integral membrane proteins of subcellular organelles such as mitochondria, endoplasmic reticulum, and Golgi, were present in our dataset.

### Subcellular location of identified proteins

**Figure 4:**
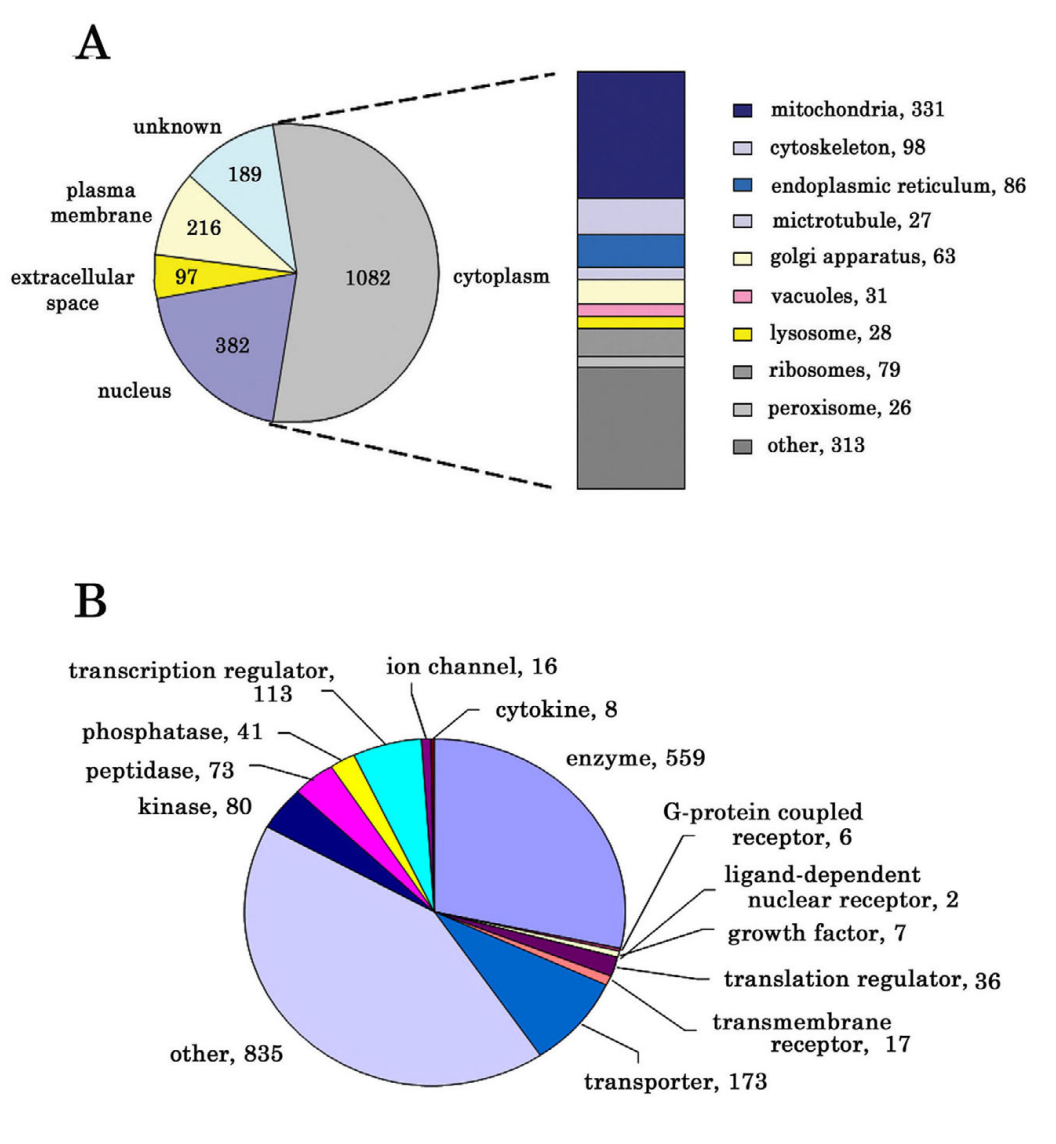
IPA classification of all identified proteins by (**4A**) subcellular location and (**4B**) protein families.

Using IPA to parse identified proteins into subcellular compartments, we found more than one half of all identified proteins were cytoplasmic proteins and Figure 4A (right side) lists the major cytoplasmic compartments represented by these proteins. Almost one third of the cytoplasmic proteins were mitochondrial proteins (331), with the Golgi apparatus, endoplasmic reticulum and ribosomes representing ~20% of total cytoplasmic proteins. IPA classified 216 proteins as plasma membrane proteins with binding activity (159), receptor activity (42), and transporter activity (36) representing major molecular functions. Seventeen plasma membrane proteins were involved in actin binding. Ninety-seven proteins were classified as extracellular proteins, including matrix proteins, cytokines and growth factors. IPA classified 382 proteins as nuclear, and while many of these included structural proteins, numerous low abundance transcription factors were identified, including STAT1, Sp1, and steroid receptor RNA activator 1 (SRA1).

### Renal cortical protein families

The IPA annotation tool for protein families grouped 1,131 identified proteins into 13 protein families while 835 proteins could not be classified (Figure 4B). Many of these protein families are enriched in renal cortex, reflecting its specialized functions of filtration and reabsorption.

#### Transporters and Ion channels

The renal process of reabsorption is accomplished by various transporters and ion channels located on the plasma membranes of tubular epithelial cells. In the present study, 173 transporters and 16 ion channel proteins were identified. Among them, 41 were located on the plasma membranes. As shown in Table 1, these plasma membrane transporters are involved in the selective reabsorption of ions (Na^+^, K^+^, Ca^2+^, Fe^3+ ^and Cl^-^), glucose, carboxylic acid, amino acids and water, which function to keep the concentration of various ions, the volume of water in the body and the pH of the blood constant. Six identified plasma membrane transporters have ATP activities. These proteins provide the essential energy for ions transporting between the cells and external tissue fluid [57]. Nine protein transporters, such as syntaxin 3, 4, and 7, were identified that have been implicated in polarized apical membrane trafficking [58]. In addition to plasma membrane transporters, we also identified 148 intracellular transporters and ion channels.

**Table 1:** Subgroups of renal proteins with transporter and ion channel activity

Sub groups of proteins with transporter activity	# of proteins	Sub groups of proteins with transporter activity	# of proteins
***Plasma membrane transporter***		***Intracellular transporter***	
Cation binding	18	Protein transporter	61
Na^+^, K^+ ^ion	15	P-P-bond-hydrolysis-driven	26
Protein transporter	9	ATPase activity	25
Organic acid	7	H^+^-transporting ATP synthesis activity	21
Carboxylic acid	7	Nucleotide binding	20
Amino acid	6	ATP binding	17
ATPase activity	6	Golgi vesicle transport	12
Chloride	5	Ion channel activity	9
ATP binding	5	Voltage-gated ion channel activity	4
Nucleotide binding	5	Iron ion binding	4
Sugar transporter	3	Chloride transport	3
Ca^2+ ^channel	2	Microtubule motor activity	3
Iron	1		
Water	1		

#### Peptidases

One mechanism for filtered protein reabsorption requires cleavage by peptidases enriched in the microvillar membranes of the brush border of the proximal tubules. We identified 73 peptidases in renal cortex, including 6 aminopeptidases, 5 dipeptidases, 4 cathepsins, 8 α-type and 6 β-type proteasome subunits, 5 protease 26S subunit ATPases and 9 ubiquitin-related peptidases. In our dataset, 13 identified peptidases are located on plasma membranes. Their potential substrates are those that are filtered at the glomerulus, such as neuropeptides and gastric hormones [59,60].

Several identified peptidases play critical roles in regulating vascular function. Kidneys have a major influence on blood pressure, blood volume and systemic vascular resistance which are mainly regulated by the renal renin-angiotensin system (RAS). Several key regulators of RAS were identified from renal cortex tissue in this study, including renin-1, angiotensin-converting enzyme (ACE), aminopeptidase A and aminopeptidase N. Aminopeptidase A (APA) is the principal enzyme that metabolizes angiotensin II to angiotensin III [61]. Aminopeptidase N (APN) is an abundant metallohydrolase in the brush border membranes of kidney proximal tubule cells which degrades angiotensin III to angiotensin IV and, along with dipeptidylaminopeptidase, degrades angiotensin IV [62].

#### Transmembrane receptors

A total of 17 transmembrane receptors were identified from renal cortex, with most having low NSAF values. One exception was heparin binding protein-44 (HBP-44), which is intensely expressed in kidneys in the renal tubular brush border [63]. IGF 1 and 2 receptors have important roles in mediating renal proximal tubular transport processes, glomerular filtration rate and renal plasma flow levels [64]. Cubilin, an albumin binding protein, is heavily expressed in the kidney proximal tubule brush border, and plays a critical role in the uptake of albumin by the proximal tubule [65,66]. We identified β1-integrin, which localizes to the basolateral aspect of epithelial cells and mediates adhesion of renal epithelial cells to the underlying matrix [67].

#### Kinases, phosphatases, and transcription regulators

A total of 80 kinases and 41 phosphatases were identified, and most of these proteins had low NSAF values. We identified 113 transcription regulators including CREB1, JUN, SP1, STAT1 and STAT3. IPA knowledge base indicated that one third of the identified transcription regulators previously have been found to be expressed in mouse kidney, while the remaining two-thirds of the identified transcription regulators, such as AT-binding transcription factor 1 (ATBF1), endothelial differentiation-related factor 1 (EDF1), and steroid receptor RNA activator 1 (SRA1), have not previously been associated with renal tissue.

#### Translation regulator

In this present dataset, there were 36 translation regulators, of which there were 23 translation initiation factors, 9 elongation factors, 2 polyadenylate-binding proteins, 1 poly(rC)-binding protein and 1 ribosomal protein. The identified translation initiation factors accounted for half of the translation initiation factors present in SWISSPROT mouse protein database and included the subunits of all the elF1 to elF6 subfamilies.

#### Enzymes

Enzymes represented the largest group of classified proteins in our dataset. Out of the 559 enzymes, 425 were classified based on their EC numbers. These 425 enzymes included 177 oxidoreductases, 83 transferases, 76 hydrolases, 25 lyases, 32 isomerases and 32 ligases. More than half of identified oxidoreductases were primarily mitochondrial proteins, and included 33 NADH dehydrogenases, 15 cytochrome C oxidase/reductase and 3 superoxide dismutases. We were able to identify ~73% of NADH dehydrogenase proteins that were present in SWISSPROT mouse protein database, indicating that the method we have used provides high proteome coverage.

#### Cytokines, growth factors, and renal disease

Interestingly, several cytokines and growth factors were identified that previously have been implicated in renal pathology. For example, up-regulated renal macrophage migration inhibitory factor (MIF) expression is closely correlated with macrophage accumulation, renal tissue damage and renal function impairment [68,69]. Its expression was an order of magnitude higher than other identified cytokines based on NSAF. Growth-arrested-specific protein 6 (GAS6) and its receptor tyrosine kinase Axl play a fundamental role in inflammatory renal disease [70,71]. EGF is a well known inducer of proliferation of renal epithelial cells, and its expression in the human kidney by far exceeds that in other tissues [72,73]. TGF-β has been implicated in the progression of numerous proliferative renal diseases [74], but our identification of this growth factor from mouse renal cortex is the first using global proteomics methods. Finally, while glia maturation factor beta (GMFB) has been considered a brain-specific growth factor since its expression is largely restricted to this organ, recent studies have reported induction of the GMFB gene in renal proximal tubular cells by proteinuria [75], and GMFB has been implicated in a renal cell tumorogenesis in hypoxia [76]. The observation that these pathology-associated proteins were identified in young, normal wild-type mice suggests that such proteins have a role in normal renal physiology, but can mediate disease processes when expression is out of homeostatic balance.

### GO classification of molecular function

**Figure 5:**
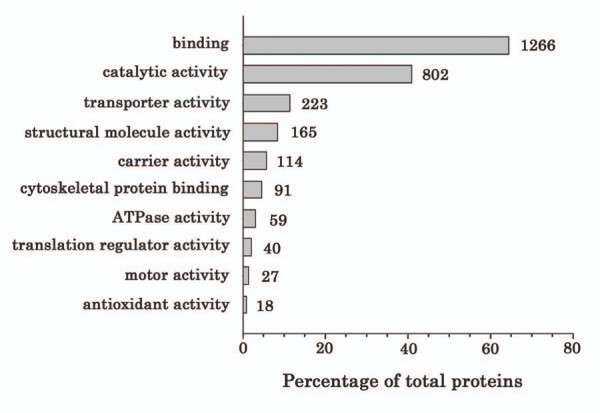
Gene Ontology classification of molecular function. Gene Ontology (GO) analysis using DAVID 2007, hosted by NIAID. GO is a structured, controlled vocabulary that describes gene products in terms of their associated biological processes, cellular components and molecular functions in a species-independent manner. 1,966 Uniprot accession numbers [see Additional file 3] were classified by biological process. Data are presented as a histogram of the relevant biological processes identified and shown as a percentage of the total identified proteins that fall within each category.

We next subjected the protein dataset to GO analysis using DAVID 2007 [77], hosted by the National Institute of Allergy and Infectious Diseases (NIAID). This approach minimizes bias in the classification of proteins in our dataset into molecular functions [78] and differed slightly from the IPA classification. DAVID recognized all 1,966 *Mus musculus *unique Uniprot accession numbers and annotated them to map to the GO molecular function terms shown graphically in Figure 5. Many proteins mapped to multiple functions. Binding activity, defined as the selective interaction of a molecule with one or more specific sites on another molecule, described 65% of all proteins. Major binding categories included protein (588), metal ion (375), nucleotide (349), ATP (189), RNA (159), actin (76), and lipid (51) binding. Catalytic activity involved 41% of all proteins. Transporter activity accounted for 223 proteins, more than the ~200 proteins with transporter activity identified with IPA; carrier activity (transfer of a specific substance or related group of substances from one side of the membrane to the other) accounted for ~52% of all proteins in this category. As shown in Figure 5, 165 proteins were identified with structural molecule activity. Motor activity (catalysis of movement along a polymeric molecule such as a microfilament or microtubule, coupled to the hydrolysis of a nucleoside triphosphate) involved 27 proteins, and included numerous myosin, dynein, kinesin, and dynactin family members. Eighteen proteins were associated with antioxidant activity within the renal cortex (see Additional file 4). This diversity of molecular functions attests to the validity of the unbiased, comprehensive, multidimensional LC-MS approach.

### Comparison of renal cortical and glomerular proteomes

**Figure 6:**
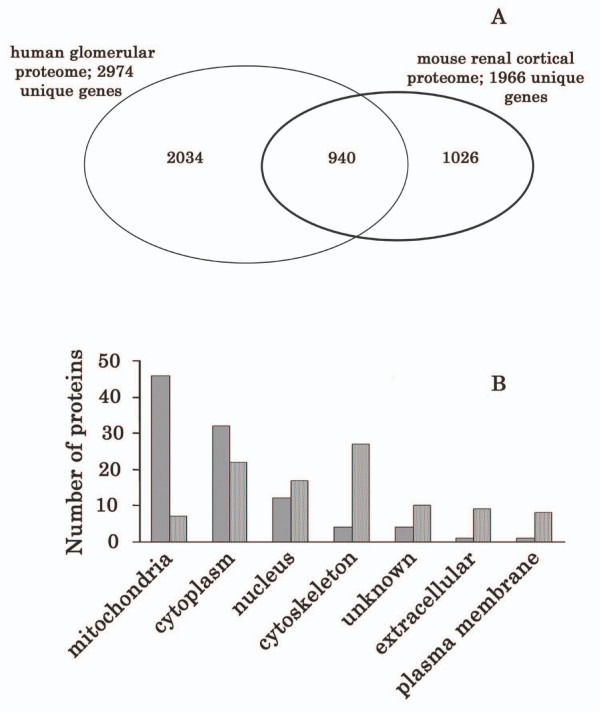
Comparison of the proteins in one published human glomerular proteome dataset with the renal cortical proteome listed in Additional file 3. **A**. the diagram of the overlapping between the two datasets. **B**. Subcellular location distribution patterns of the 100 most abundant proteins in two datasets.

We compared a purified glomerular proteome established by Miyamoto *et al *[21] from one human kidney with our mouse renal cortical proteome. Both approaches used charge prefractionation strategies – solution phase IEF for the human glomerular work and SCX for the mouse cortex – prior to LC-MS/MS. However, Miyamoto *et al *searched their mass spectrometry data against several protein databases, often with redundancy, while we limited our protein identification to SWISSPROT database with the minimum level of redundancy. As shown in Figure 6A, the proteome differs between cortex and glomerulus. About 48% of our renal cortical proteome (940 proteins) was also present in the glomerular proteome, while 1026 proteins were unique to the renal cortex. We calculated NSAF values for the proteins listed in the glomerular dataset, then compared the 100 most abundant (highest NSAF scores) proteins in the two datasets (Figure 6B). While relatively similar numbers of highly abundant proteins were identified in the cytoplasm and nucleus, striking differences in the number of mitochondrial (enriched in the renal cortex) versus cytoskeletal (enriched in the glomerular proteome) proteins were identified. Almost one half of the 100 most abundant cortical proteins were mitochondrial versus less than 10% for the glomerular proteins. It is not surprising that cytoskeletal proteins are enriched in the glomerular proteome since endothelial cells and mesangial cells are two major cell types in glomerulus. Endothelial cells have a complex cytoskeleton and mesangial cells are modified smooth muscle cells which are rich in cytoskeletal proteins. In contrast, tubular epithelial cells are the predominant cell type in the renal cortex and account for more than 80% of renal parenchymal cells [79]. Tubular epithelial cells are richly supplied with mitochondria and are highly dependent on mitochondrial energy production for their physiological roles of absorption and secretion [79,80]. Our findings are consistent with the suggestion that the glomerular proteome is representative of only a small subset of the renal cortical proteome with significant differences in protein families and their expression level. It is noteworthy that we compared the mouse renal cortical proteome identified in this study to a human renal glomerular proteome since the latter is the largest, global map of the glomerular proteome to date. Ultimately, it will be interesting to compare the mouse cortical and glomerular proteomes when a suitable dataset for the latter becomes available. It is important to emphasize that variability from the use of MS can undermine the usefulness of NSAF and the reliability of data among different laboratories or experiments. Therefore, when we performed comparisons of renal cortical and glomerular proteomes, we used the NSAF value to determine the relative abundance of identified proteins within each dataset rather than compare the NSAF value of the same proteins across the two datasets.

#### Comparison of renal cortical and urinary proteomes

**Figure 7:**
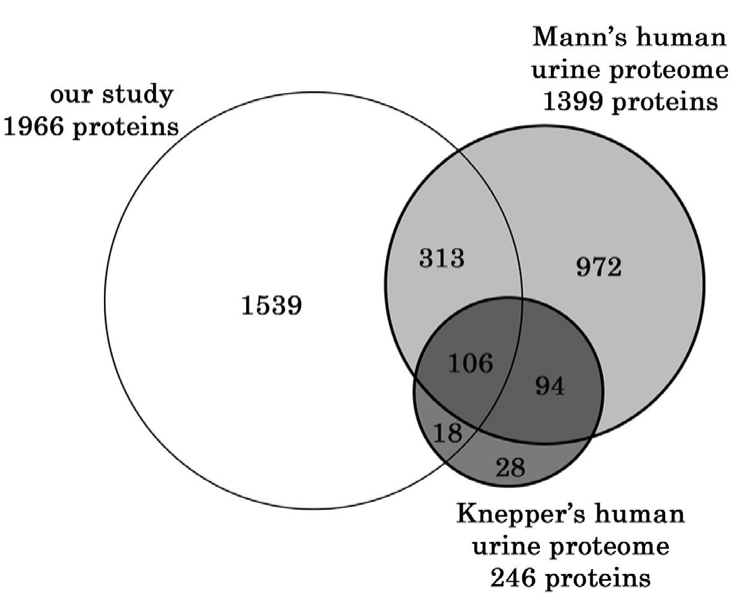
Comparison of the proteins in two urinary proteome datasets with the renal cortical proteome listed in Additional file 2. The total sum of unique gene products reported in these two urinary proteome datasets was 1,435. Of these, 437 (30.4%) were identified in our dataset.

A large number of urinary proteome studies have been performed [10,17,81-83], and one of the most important findings of these studies is that only ~30% of identified proteins are plasma-derived proteins. The remaining ~70% proteins in urinary proteome may be derived from renal cells [17], and the identification of kidney plasma membrane proteins in urine supports this hypothesis [81,83]. Since few studies have directly compared urinary and renal proteomes, we compared our dataset with two large datasets of the urinary proteome obtained from normal human subjects [81,83]. In order to compare the different protein identifiers, the protein IDs in each dataset were converted to gene symbols. While most hypothetical proteins in the two urinary proteomes could not be assigned gene symbols, the total sum of unique gene products reported in these two urinary proteome datasets was 1,435. Of these, 437 (30.4%) were identified in our dataset (see Figure 7 and Additional file 5), indicating that nearly one third of the proteins in the urine are of renal cortex origin.

**Figure 8:**
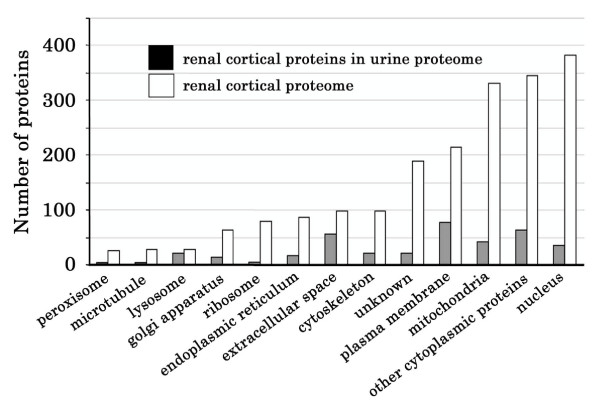
Subcellular location distribution patterns of the renal cortex proteome and the renal proteins that have been identified in urine.

Two possibilities may account for how renal proteins are delivered into the urine, including: 1) whole kidney cells or cell fragments are shed into urine, and 2) these proteins are excreted into urine through exosome formation. In normal kidneys, exosome formation is the dominant excretion pathway and whole cell shedding plays a minor role [83]. This conclusion is supported by our observations. As shown in Figure 8, there are significant differences between the subcellular origin of proteins present in the entire renal cortex compared to the urine. Renal extracellular proteins, plasma membrane proteins, and lysosome proteins are enriched in urine, whereas proteins from other intracellular organelles of kidney cells are not. For example, 20 of the 28 identified renal lysosome proteins (71%) were found in urine, and only 9% (35) of the identified renal nuclear proteins were present in urine. We found that more than one half of the 105 most abundant renal proteins (58) were not present in urine. The majority (78%) of the most abundant renal proteins not found in the urine were nuclear (such as histone H2A and histone H2B) and mitochondrial (such as electron transfer flavoprotein alpha and beta subunit and ubiquinol-cytochrome C reductase) proteins. On the other hand, low-abundance plasma membrane peptidases, receptors and transporters were enriched in urine. In addition, all of the class E vacuolar protein-sorting proteins that were identified in urine samples and found to mediate exosome formation [81] were present in our dataset. Most proteins in urinary vesicles previously identified in exosomes from other cell types [81] are present in our dataset as well.

### Concluding remarks

Using a multiple fractionation strategy, a whole tissue extract of the mouse kidney cortex was analyzed by an unbiased proteomic approach, yielding a dataset of ~2,000 proteins identified with strict criteria to ensure a high level of confidence in the protein identifications. As a result of extracting all proteins from the renal cortex, then using an unbiased fractionation scheme involving prefractionation with SCX followed by LC-MS/MS, we identified an exceptionally wide range of renal proteins in terms of pI, MW, hydrophobicity, abundance, and subcellular location. Many of these proteins, such as low-abundance proteins, membrane proteins and proteins with extreme values in pI or MW are traditionally under-represented in 2-DE-based proteomic analysis. This dataset represents the largest and most confident inventory of proteins present in renal cortical tissue to date, and validates our fractionation strategy for increasing coverage of renal proteins. It not only serves as a useful reference for further experiments characterizing differential expression of renal cortical proteins induced by diseases, but also provides a foundation for the development of systems biology linkages between the dysregulated protein pathways and networks in renal diseases such as diabetic nephropathy.

## List of Abbreviations

*FPR*: false positive rate; *IPA*: Ingenuity Pathways Analysis; *MALDI-TOF*: matrix assisted laser desorption/ionization time-of-flight; *NSAF*: normalized spectral abundance factor; *2D-LC-MS/MS*: two dimensional liquid chromatography-tandem mass spectrometry; *SCX*: strong cation exchange.

## Competing interests

The authors declare that they have no competing interests.

## Authors' contributions

YZ conceived the study, participated in the mass spectrometry, calculated the NSAF values reported in the manuscript, and helped draft the manuscript, LD participated in the design of the study, evaluated the mass spectrometry data, and helped draft the manuscript, SH was responsible for processing the renal cortical proteins and participated in the mass spectrometry, WL was responsible for the animal surgeries necessary to harvest the kidneys, RT was responsible for the implementation of the mouse studies and harvesting the kidneys, participated in the bioinformatics analysis of the mass spectrometry data, and prepared the manuscript. All authors read and approved the final manuscript.

## Supplementary Material

Additional file 1 List of proteins identified from mouse renal cortex. Annotation of proteins identified from three mice used in experiments assessing reproducibility. Click here for file

Additional file 2 List of all peptides. Identified peptides with <1% FPR obtained from one mouse renal cortex separated into 60 fractions using SCX prior to LC-MS/MS. Click here for file

Additional file 3 List of proteins identified from mouse renal cortex. Annotation of all identified proteins from the list of peptides identified in Additional File 2. Click here for file

Additional file 4 Renal proteins with anti-oxidant activity. List of all renal identified proteins with anti-oxidant activity. Click here for file

Additional file 5 Renal proteins identified in published urine proteomes. List of all proteins identified in the mouse renal cortex that have been previously identified in published human urine proteomes. Click here for file
