# Supplementary material for: Comprehensive analysis of the mouse renal cortex using two-dimensional HPLC – tandem mass spectrometry
Source: Proteome Sci. 2008 May 23;6:15. doi: 10.1186/1477-5956-6-15 (PMC2412861; doi:10.1186/1477-5956-6-15)
Supplement: Additional file 1 — List of proteins identified from mouse renal cortex. Annotation of proteins identified from three mice used in experiments assessing reproducibility. [file 1477-5956-6-15-S1.pdf]

**Supplemental Table S1. Proteins identified from three mouse renal cortical tissues.**

*The animal where the proteins were identifed are indicated in the "Animal" column. A, indicates that the protein was identifed from mouse A; B, indicates that the protein was identifed from mouse B; C, indicates that the protein was identifed from mouse C.

| Accession Number | LocusID | Number of amino acid | MW | pI | Description | NSAF | | | Animal* |
|---|---|---|---|---|---|---|---|---|---|
| | | | | | | Mean | Standard Deviation | Coefficient of variation | |
| Q03265 | ATPA_MOUSE | 553 | 59753 | 9.2 | (Q03265) ATP synthase alpha chain, mitochondrial precursor (EC 3.6.3.14) | 0.02901701 | 0.00203309 | 7% | A, B, C |
| P02088 | HBB1_MOUSE | 146 | 15709 | 7.6 | (P02088) Hemoglobin beta-1 subunit (Hemoglobin beta-1 chain) (Beta-1-globin) (Hemoglobin beta-major chain) | 0.024484 | 0.00091548 | 4% | A, B, C |
| P63038 | CH60_MOUSE | 573 | 60956 | 6.2 | (P63038) 60 kDa heat shock protein, mitochondrial precursor (Hsp60) (60 kDa chaperonin) (CPN60) (Heat shock protein 60) (HSP-60) (Mitochondrial matrix protein P1) (HSP-65) | 0.02126995 | 0.00458512 | 22% | A, B, C |
| Q8CGP5 | H2A1F_MOUSE | 129 | 14030 | 11.1 | (Q8CGP5) Histone H2A type 1-F | 0.01963106 | 0.00447135 | 23% | A, B, C |
| Q64475 | H2B1B_MOUSE | 125 | 13821 | 10.3 | (Q64475) Histone H2B type 1-B (h2B-143) | 0.01842853 | 0.00945801 | 51% | A, B, C |
| Q06185 | ATP5I_MOUSE | 70 | 8104 | 9.4 | (Q06185) ATP synthase e chain, mitochondrial (EC 3.6.3.14) | 0.01573326 | 0.00248688 | 16% | A, B, C |
| P52760 | UK114_MOUSE | 134 | 14124 | 8.7 | (P52760) Ribonuclease UK114 (EC 3.1.-.-) (Heat-responsive protein 12) | 0.01571018 | 0.00453299 | 29% | A, B, C |
| Q60932 | VDAC1_MOUSE | 296 | 32351 | 8.4 | (Q60932) Voltage-dependent anion-selective channel protein 1 (VDAC-1) (mVDAC1) (mVDAC5) (Outer mitochondrial membrane protein porin 1) (Plasmalemmal porin) | 0.01570011 | 0.00796399 | 51% | A, B, C |
| P56480 | ATPB_MOUSE | 529 | 56301 | 5.3 | (P56480) ATP synthase beta chain, mitochondrial precursor (EC 3.6.3.14) | 0.01553484 | 0.0014145 | 9% | A, B, C |
| P17182 | ENOA_MOUSE | 433 | 47010 | 6.8 | (P17182) Alpha-enolase (EC 4.2.1.11) (2-phospho-D-glycerate hydro-lyase) (Non-neural enolase) (NNE) (Enolase 1) | 0.01480272 | 0.00076962 | 5% | A, B, C |
| P62806 | H4_MOUSE | 102 | 11236 | 11.4 | (P62806) Histone H4 | 0.01271294 | 0.00478084 | 38% | A, B, C |
| P99029 | PRDX5_MOUSE | 210 | 21897 | 8.9 | (P99029) Peroxiredoxin-5, mitochondrial precursor (EC 1.11.1.15) (Prx-V) (Peroxisomal antioxidant enzyme) (PLP) (Thioredoxin reductase) (Thioredoxin peroxidase PMP20) (Antioxidant enzyme B166) (AOEB166) (Liver tissue 2D-page spot 2D-0014IV) | 0.01241915 | 0.0071178 | 57% | A, B, C |
| Q99LC5 | ETFA_MOUSE | 333 | 35039 | 8.4 | (Q99LC5) Electron transfer flavoprotein subunit alpha, mitochondrial precursor (Alpha-ETF) | 0.01170696 | 0.00479819 | 41% | A, B, C |
| P17742 | PPIA_MOUSE | 163 | 17840 | 7.9 | (P17742) Peptidyl-prolyl cis-trans isomerase A (EC 5.2.1.8) (PPIase A) (Rotamase A) (Cyclophilin A) (Cyclosporin A-binding protein) (SP18) | 0.01035478 | 0.00484014 | 47% | A, B, C |
| P17751 | TPIS_MOUSE | 248 | 26581 | 7.3 | (P17751) Triosephosphate isomerase (EC 5.3.1.1) (TIM) (Triose-phosphate isomerase) | 0.00947044 | 0.00165881 | 18% | A, B, C |
| P16858 | G3P_MOUSE | 332 | 35679 | 8.2 | (P16858) Glyceraldehyde-3-phosphate dehydrogenase (EC 1.2.1.12) (GAPDH) | 0.0090097 | 0.00219925 | 24% | A, B, C |
| Q8BWT1 | THIM_MOUSE | 397 | 41858 | 8.1 | (Q8BWT1) 3-ketoacyl-CoA thiolase, mitochondrial (EC 2.3.1.16) (Beta-ketothiolase) (Acetyl-CoA acyltransferase) (Mitochondrial 3-oxoacyl-CoA thiolase) | 0.0088786 | 0.00170153 | 19% | A, B, C |
| P08249 | MDHM_MOUSE | 338 | 35596 | 8.6 | (P08249) Malate dehydrogenase, mitochondrial precursor (EC 1.1.1.37) | 0.00845112 | 0.00157557 | 19% | A, B, C |
| Q8QZT1 | THIL_MOUSE | 424 | 44816 | 8.5 | (Q8QZT1) Acetyl-CoA acetyltransferase, mitochondrial precursor (EC 2.3.1.9) (Acetoacetyl-CoA thiolase) | 0.00824283 | 0.00084024 | 10% | A, B, C |

| | | | | | | | | | |
|---|---|---|---|---|---|---|---|---|---|
| Q8CHT0 | AL4A1_MOUSE | 562 | 61811 | 8.4 | (Q8CHT0) Delta-1-pyrroline-5-carboxylate dehydrogenase, mitochondrial precursor (EC 1.5.1.12) (P5C dehydrogenase) (Aldehyde dehydrogenase 4A1) | 0.00764763 | 0.00295288 | 39% | A, B, C |
| Q64433 | CH10_MOUSE | 101 | 10831 | 8.3 | (Q64433) 10 kDa heat shock protein, mitochondrial (Hsp10) (10 kDa chaperonin) (CPN10) | 0.0075216 | 0.00373016 | 50% | A, B, C |
| P51881 | ADT2_MOUSE | 297 | 32800 | 9.7 | (P51881) ADP/ATP translocase 2 (Adenine nucleotide translocator 2) (ANT 2) (ADP,ATP carrier protein 2) (Solute carrier family 25 member 5) | 0.00732297 | 0.00195528 | 27% | A, B, C |
| Q9D0K2 | SCOT_MOUSE | 520 | 55989 | 8.5 | (Q9D0K2) Succinyl-CoA:3-ketoacid-coenzyme A transferase 1, mitochondrial precursor (EC 2.8.3.5) (Somatic-type succinyl CoA:3-oxoacid CoA-transferase) (Scot-S) | 0.00731079 | 0.00131809 | 18% | A, B, C |
| P18760 | COF1_MOUSE | 165 | 18428 | 8.1 | (P18760) Cofilin-1 (Cofilin, non-muscle isoform) | 0.00718626 | 0.00464231 | 65% | A, B, C |
| P97816 | S100G_MOUSE | 78 | 8839 | 4.8 | (P97816) Protein S100-G (S100 calcium-binding protein G) (Vitamin D-dependent calcium-binding protein, intestinal) (CABP) (Calbindin D9K) | 0.00713807 | 0.00672537 | 94% | A, B, C |
| Q91Y97 | ALDOB_MOUSE | 363 | 39376 | 8.3 | (Q91Y97) Fructose-bisphosphate aldolase B (EC 4.1.2.13) (Liver type aldolase) (Aldolase 2) | 0.00670852 | 0.00365975 | 55% | A, B, C |
| Q9QXD6 | F16P1_MOUSE | 337 | 36781 | 6.6 | (Q9QXD6) Fructose-1,6-bisphosphatase 1 (EC 3.1.3.11) (D-fructose-1,6-bisphosphate 1-phosphohydrolase 1) (FBPase 1) | 0.00646889 | 0.00340227 | 53% | A, B, C |
| P09411 | PGK1_MOUSE | 416 | 44405 | 7.6 | (P09411) Phosphoglycerate kinase 1 (EC 2.7.2.3) | 0.00636975 | 0.00495981 | 78% | A, B, C |
| Q99LB2 | DHRS4_MOUSE | 260 | 27754 | 9.1 | (Q99LB2) Dehydrogenase/reductase SDR family member 4 (EC 1.1.1.184) (NADPH-dependent carbonyl reductase/NADP-retinol dehydrogenase) (CR) (PHCR) (Peroxisomal short-chain alcohol dehydrogenase) (NADPH-dependent retinol dehydrogenase/reductase) | 0.00636562 | 0.00595613 | 94% | A, B, C |
| P14094 | AT1B1_MOUSE | 304 | 35195 | 8.6 | (P14094) Sodium/potassium-transporting ATPase subunit beta-1 (Sodium/potassium-dependent ATPase beta-1 subunit) | 0.00603348 | 0.00157999 | 26% | A, B, C |
| P11352 | GPX1_MOUSE | 201 | 22282 | 7.2 | (P11352) Glutathione peroxidase 1 (EC 1.11.1.9) (GSHPx-1) (GPx-1) (Cellular glutathione peroxidase) | 0.0059792 | 0.00352256 | 59% | A, B, C |
| P35505 | FAAA_MOUSE | 419 | 46104 | 7.4 | (P35505) Fumarylacetoacetase (EC 3.7.1.2) (Fumarylacetoacetate hydrolase) (Beta-diketonase) (FAA) | 0.00585275 | 0.00275695 | 47% | A, B, C |
| P62204 | CALM_MOUSE | 148 | 16706 | 4.2 | (P62204) Calmodulin (CaM) | 0.00563834 | 0.00448503 | 80% | A, B, C |
| Q64522 | H2A2B_MOUSE | 129 | 13882 | 10.9 | (Q64522) Histone H2A type 2-B (H2a-613A) | 0.00558962 | 0.00200813 | 36% | A, B, C |
| Q8VDN2 | AT1A1_MOUSE | 1023 | 112982 | 5.5 | (Q8VDN2) Sodium/potassium-transporting ATPase alpha-1 chain precursor (EC 3.6.3.9) (Sodium pump 1) (Na+/K+ ATPase 1) | 0.0054646 | 0.00014726 | 3% | A, B, C |
| O88844 | IDHC_MOUSE | 414 | 46660 | 6.9 | (O88844) Isocitrate dehydrogenase [NADP] cytoplasmic (EC 1.1.1.42) (Cytosolic NADP-isocitrate dehydrogenase) (Oxalosuccinate decarboxylase) (IDH) (NADP(+)-specific ICDH) (IDP) | 0.00525304 | 0.00292751 | 56% | A, B, C |
| P07724 | ALBU_MOUSE | 608 | 68693 | 6.1 | (P07724) Serum albumin precursor | 0.00522162 | 0.00114039 | 22% | A, B, C |
| P47199 | QOR_MOUSE | 331 | 35269 | 8.1 | (P47199) Quinone oxidoreductase (EC 1.6.5.5) (NADPH:quinone reductase) (Zeta-crystallin) | 0.00520954 | 0.00180006 | 35% | A, B, C |
| P67778 | PHB_MOUSE | 272 | 29820 | 5.8 | (P67778) Prohibitin (B-cell receptor-associated protein 32) (BAP 32) | 0.00520614 | 0.0022151 | 43% | A, B, C |
| Q9DCX2 | ATP5H_MOUSE | 160 | 18618 | 5.7 | (Q9DCX2) ATP synthase D chain, mitochondrial (EC 3.6.3.14) | 0.00498868 | 0.00271414 | 54% | A, B, C |

| | | | | | | | | | |
|---|---|---|---|---|---|---|---|---|---|
| Q9WTP6 | KAD2_MOUSE | 231 | 25474 | 7.4 | (Q9WTP6) Adenylate kinase isoenzyme 2, mitochondrial (EC 2.7.4.3) (ATP-AMP transphosphorylase) | 0.00467063 | 0.00172665 | 37% | A, B, C |
| P09103 | PDIA1_MOUSE | 509 | 57144 | 4.9 | (P09103) Protein disulfide-isomerase precursor (EC 5.3.4.1) (PDI) (Prolyl 4-hydroxylase subunit beta) (Cellular thyroid hormone-binding protein) (p55) (Erp59) | 0.00450812 | 0.00117845 | 26% | A, B, C |
| P12787 | COX5A_MOUSE | 145 | 16030 | 6.5 | (P12787) Cytochrome c oxidase polypeptide Va, mitochondrial precursor (EC 1.9.3.1) | 0.00439849 | 0.00045975 | 10% | A, B, C |
| P62843 | RS15_MOUSE | 144 | 16909 | 10.4 | (P62843) 40S ribosomal protein S15 (RIG protein) | 0.00427214 | 0.00217757 | 51% | A, B, C |
| Q9WUM5 | SUCA_MOUSE | 333 | 34994 | 9.4 | (Q9WUM5) Succinyl-CoA ligase [GDP-forming] alpha-chain, mitochondrial precursor (EC 6.2.1.4) (Succinyl-CoA synthetase, alpha chain) (SCS-alpha) | 0.00426323 | 0.00083301 | 20% | A, B, C |
| P62908 | RS3_MOUSE | 243 | 26674 | 9.7 | (P62908) 40S ribosomal protein S3 | 0.00417053 | 0.00388132 | 93% | A, B, C |
| P99027 | RLA2_MOUSE | 115 | 11651 | 4.5 | (P99027) 60S acidic ribosomal protein P2 | 0.00413544 | 0.00042077 | 10% | A, B, C |
| P16460 | ASSY_MOUSE | 412 | 46585 | 8.2 | (P16460) Argininosuccinate synthase (EC 6.3.4.5) (Citrulline--aspartate ligase) | 0.00412928 | 0.00154464 | 37% | A, B, C |
| Q9JII6 | AK1A1_MOUSE | 324 | 36456 | 7.4 | (Q9JII6) Alcohol dehydrogenase [NADP+] (EC 1.1.1.2) (Aldehyde reductase) (Aldo-keto reductase family 1 member A1) | 0.00393121 | 0.00153444 | 39% | A, B, C |
| O88569 | ROA2_MOUSE | 341 | 35993 | 8.6 | (O88569) Heterogeneous nuclear ribonucleoproteins A2/B1 (hnRNP A2 / hnRNP B1) | 0.00389255 | 0.00202825 | 52% | A, B, C |
| Q9DCW4 | ETFB_MOUSE | 254 | 27492 | 8.1 | (Q9DCW4) Electron transfer flavoprotein subunit beta (Beta-ETF) | 0.00382758 | 0.00041616 | 11% | A, B, C |
| P10922 | H10_MOUSE | 193 | 20730 | 10.9 | (P10922) Histone H1' (H1.0) (H1(0)) | 0.00377418 | 0.00153379 | 41% | A, B, C |
| P63242 | IF5A1_MOUSE | 153 | 16701 | 5.2 | (P63242) Eukaryotic translation initiation factor 5A-1 (eIF-5A-1) (eIF-5A1) (Eukaryotic initiation factor 5A isoform 1) (eIF-5A) (eIF-4D) | 0.00357492 | 0.00181921 | 51% | A, B, C |
| P38647 | GRP75_MOUSE | 679 | 73528 | 6.2 | (P38647) Stress-70 protein, mitochondrial precursor (75 kDa glucose-regulated protein) (GRP 75) (Peptide-binding protein 74) (PBP74) (P66 MOT) (Mortalin) | 0.0035529 | 0.00088921 | 25% | A, B, C |
| P14152 | MDHC_MOUSE | 333 | 36380 | 6.6 | (P14152) Malate dehydrogenase, cytoplasmic (EC 1.1.1.37) (Cytosolic malate dehydrogenase) | 0.00352434 | 0.00124606 | 35% | A, B, C |
| P62897 | CYC_MOUSE | 104 | 11474 | 9.6 | (P62897) Cytochrome c, somatic | 0.00348482 | 0.00334614 | 96% | A, B, C |
| Q9DCS9 | NDUBA_MOUSE | 175 | 20893 | 8 | (Q9DCS9) NADH dehydrogenase [ubiquinone] 1 beta subcomplex subunit 10 (EC 1.6.5.3) (EC 1.6.99.3) (NADH-ubiquinone oxidoreductase PDSW subunit) (Complex I-PDSW) (CI-PDSW) | 0.0034313 | 0.00059937 | 17% | A, B, C |
| P43024 | CX6A1_MOUSE | 111 | 12352 | 10 | (P43024) Cytochrome c oxidase polypeptide VIa-liver, mitochondrial precursor (EC 1.9.3.1) | 0.00342903 | 0.00184429 | 54% | A, B, C |
| P12658 | CALB1_MOUSE | 260 | 29863 | 4.8 | (P12658) Calbindin (Vitamin D-dependent calcium-binding protein, avian-type) (Calbindin D28) (D-28K) (Spot 35 protein) (PCD-29) | 0.00342501 | 0.00277813 | 81% | A, B, C |
| P10126 | EF1A1_MOUSE | 462 | 50114 | 9 | (P10126) Elongation factor 1-alpha 1 (EF-1-alpha-1) (Elongation factor 1 A-1) (eEF1A-1) (Elongation factor Tu) (EF-Tu) | 0.00341895 | 0.00275932 | 81% | A, B, C |
| Q99KI0 | ACON_MOUSE | 780 | 85464 | 7.9 | (Q99KI0) Aconitate hydratase, mitochondrial precursor (EC 4.2.1.3) (Citrate hydro-lyase) (Aconitase) | 0.00337204 | 0.00097322 | 29% | A, B, C |
| P50543 | S10AB_MOUSE | 98 | 11083 | 5.5 | (P50543) Protein S100-A11 (S100 calcium-binding protein A11) (Protein S100C) (Calgizzarin) (Endothelial monocyte-activating polypeptide) (EMAP) | 0.0033716 | 0.002262 | 67% | A, B, C |

| | | | | | | | | | |
|---|---|---|---|---|---|---|---|---|---|
| Q62425 | NDUA4_MOUSE | 82 | 9327 | 9.5 | (Q62425) NADH dehydrogenase [ubiquinone] 1 alpha subcomplex subunit 4 (EC 1.6.5.3) (EC 1.6.99.3) (NADH-ubiquinone oxidoreductase MLRQ subunit) (Complex I-MLRQ) (CI-MLRQ) | 0.00334905 | 0.00111288 | 33% | A, B, C |
| Q8K2B3 | DHSA_MOUSE | 664 | 72585 | 7.4 | (Q8K2B3) Succinate dehydrogenase [ubiquinone] flavoprotein subunit, mitochondrial precursor (EC 1.3.5.1) (Fp) (Flavoprotein subunit of complex II) | 0.00319019 | 0.00066319 | 21% | A, B, C |
| P97328 | KHK_MOUSE | 298 | 32750 | 6.2 | (P97328) Ketohexokinase (EC 2.7.1.3) (Hepatic fructokinase) | 0.00318936 | 0.00244076 | 77% | A, B, C |
| O35129 | PHB2_MOUSE | 299 | 33296 | 9.8 | (O35129) Prohibitin-2 (B-cell receptor-associated protein BAP37) (Repressor of estrogen receptor activity) | 0.00316372 | 0.00061278 | 19% | A, B, C |
| O09174 | AMACR_MOUSE | 380 | 41587 | 7.4 | (O09174) Alpha-methylacyl-CoA racemase (EC 5.1.99.4) (2-methylacyl-CoA racemase) | 0.00314434 | 0.00142679 | 45% | A, B, C |
| O35488 | S27A2_MOUSE | 620 | 70367 | 8.8 | (O35488) Very-long-chain acyl-CoA synthetase (EC 6.2.1.-) (VLCS) (Very-long-chain-fatty-acid-CoA ligase) (VLACS) (THCA-CoA ligase) (Fatty-acid-coenzyme A ligase, very long-chain 1) (Long-chain-fatty-acid--CoA ligase) (EC 6.2.1.3) (Fatty acid | 0.00313054 | 0.00025382 | 8% | A, B, C |
| P16125 | LDHB_MOUSE | 333 | 36441 | 6.1 | (P16125) L-lactate dehydrogenase B chain (EC 1.1.1.27) (LDH-B) (LDH heart subunit) (LDH-H) | 0.00310182 | 0.00278814 | 90% | A, B, C |
| P24270 | CATA_MOUSE | 526 | 59634 | 7.9 | (P24270) Catalase (EC 1.11.1.6) | 0.00301726 | 0.00281793 | 93% | A, B, C |
| P34914 | HYES_MOUSE | 554 | 62515 | 6.2 | (P34914) Epoxide hydrolase 2 (EC 3.3.2.3) (Soluble epoxide hydrolase) (SEH) (Epoxide hydratase) (Cytosolic epoxide hydrolase) (CEH) | 0.00298247 | 0.00183571 | 62% | A, B, C |
| Q99L13 | 3HIDH_MOUSE | 335 | 35440 | 8.1 | (Q99L13) 3-hydroxyisobutyrate dehydrogenase, mitochondrial precursor (EC 1.1.1.31) (HIBADH) | 0.00297012 | 0.00048311 | 16% | A, B, C |
| P62962 | PROF1_MOUSE | 139 | 14826 | 8.3 | (P62962) Profilin-1 (Profilin I) | 0.00294919 | 0.00217184 | 74% | A, B, C |
| Q9D0S9 | HINT2_MOUSE | 163 | 17320 | 9.8 | (Q9D0S9) Histidine triad nucleotide-binding protein 2 (EC 3.-.-.-) (HINT-2) (HINT-3) | 0.00291376 | 0.00342813 | 118% | A, B, C |
| Q8BH95 | ECHM_MOUSE | 290 | 31474 | 8.5 | (Q8BH95) Enoyl-CoA hydratase, mitochondrial precursor (EC 4.2.1.17) (Short chain enoyl-CoA hydratase) (SCEH) (Enoyl-CoA hydratase 1) | 0.0028256 | 0.00068844 | 24% | A, B, C |
| P04117 | FABPA_MOUSE | 131 | 14519 | 8.4 | (P04117) Fatty acid-binding protein, adipocyte (AFABP) (Adipocyte lipid-binding protein) (ALBP) (A-FABP) (P2 adipocyte protein) (Myelin P2 protein homolog) (3T3-L1 lipid-binding protein) (422 protein) (P15) | 0.00274298 | 0.00164437 | 60% | A, B, C |
| O88338 | CAD16_MOUSE | 830 | 89860 | 4.7 | (O88338) Cadherin-16 precursor (Kidney-specific cadherin) (Ksp-cadherin) | 0.00267708 | 0.00067975 | 25% | A, B, C |
| Q6GSS7 | H2A2A_MOUSE | 129 | 13964 | 10.9 | (Q6GSS7) Histone H2A type 2-A (H2A.2) (H2a-614) (H2a-615) | 0.00266716 | 0.00112984 | 42% | A, B, C |
| Q9CPY7 | AMPL_MOUSE | 487 | 52619 | 7 | (Q9CPY7) Cytosol aminopeptidase (EC 3.4.11.1) (Leucine aminopeptidase) (LAP) (Leucyl aminopeptidase) (Proline aminopeptidase) (EC 3.4.11.5) (Prolyl aminopeptidase) | 0.00266047 | 0.00119597 | 45% | A, B, C |
| Q60928 | GGT1_MOUSE | 568 | 61563 | 7.2 | (Q60928) Gamma-glutamyltranspeptidase 1 precursor (EC 2.3.2.2) (Gamma-glutamyltransferase 1) (GGT 1) (CD224 antigen) [Contains: Gamma-glutamyltranspeptidase 1 heavy chain; Gamma-glutamyltranspeptidase 1 light chain] | 0.00266014 | 0.00064662 | 24% | A, B, C |
| Q61425 | HCDH_MOUSE | 314 | 34464 | 8.6 | (Q61425) Short chain 3-hydroxyacyl-CoA dehydrogenase, mitochondrial precursor (EC 1.1.1.35) (HCDH) (Medium and short chain L-3-hydroxyacyl-coenzyme A dehydrogenase) | 0.00265583 | 0.00172765 | 65% | A, B, C |

| | | | | | | | | | |
|---|---|---|---|---|---|---|---|---|---|
| P62849 | RS24_MOUSE | 133 | 15423 | 10.8 | (P62849) 40S ribosomal protein S24 | 0.00265028 | 0.00303075 | 114% | A, B, C |
| P26040 | EZRI_MOUSE | 585 | 69276 | 6.1 | (P26040) Ezrin (p81) (Cytovillin) (Villin-2) | 0.00264386 | 0.00193246 | 73% | A, B, C |
| P63276 | RS17_MOUSE | 134 | 15393 | 9.8 | (P63276) 40S ribosomal protein S17 | 0.00263475 | 0.00088986 | 34% | A, B, C |
| Q91VR2 | ATPG_MOUSE | 298 | 32886 | 9 | (Q91VR2) ATP synthase gamma chain, mitochondrial precursor (EC 3.6.3.14) | 0.00253713 | 0.00121452 | 48% | A, B, C |
| Q921H8 | THIKA_MOUSE | 424 | 43953 | 8.4 | (Q921H8) 3-ketoacyl-CoA thiolase A, peroxisomal precursor (EC 2.3.1.16) (Beta-ketothiolase A) (Acetyl-CoA acyltransferase A) (Peroxisomal 3-oxoacyl-CoA thiolase A) | 0.00253585 | 0.00109031 | 43% | A, B, C |
| P17563 | SBP1_MOUSE | 472 | 52352 | 6.4 | (P17563) Selenium-binding protein 1 (56 kDa selenium-binding protein) (SP56) | 0.00249265 | 0.00167475 | 67% | A, B, C |
| Q9Z2I8 | SUCB2_MOUSE | 433 | 46840 | 7 | (Q9Z2I8) Succinyl-CoA ligase [GDP-forming] beta-chain, mitochondrial precursor (EC 6.2.1.4) (Succinyl-CoA synthetase, betaG chain) (SCS-betaG) (GTP-specific succinyl-CoA synthetase subunit beta) | 0.00249018 | 0.0004349 | 17% | A, B, C |
| Q9DB20 | ATPO_MOUSE | 213 | 23364 | 10 | (Q9DB20) ATP synthase O subunit, mitochondrial precursor (EC 3.6.3.14) (Oligomycin sensitivity conferral protein) (OSCP) | 0.00243013 | 0.00078072 | 32% | A, B, C |
| P01942 | HBA_MOUSE | 141 | 14954 | 8.2 | (P01942) Hemoglobin subunit alpha (Hemoglobin alpha chain) (Alpha-globin) | 0.00240173 | 0.00050211 | 21% | A, B, C |
| P62737 | ACTA_MOUSE | 377 | 42009 | 5.4 | (P62737) Actin, aortic smooth muscle (Alpha-actin-2) | 0.00240073 | 0.00100311 | 42% | A, B, C |
| Q62468 | VILI_MOUSE | 826 | 92670 | 6 | (Q62468) Villin-1 | 0.00235496 | 0.00017757 | 8% | A, B, C |
| O08709 | PRDX6_MOUSE | 223 | 24739 | 6 | (O08709) Peroxiredoxin-6 (EC 1.11.1.15) (Antioxidant protein 2) (1-Cys peroxiredoxin) (1-Cys PRX) (Acidic calcium-independent phospholipase A2) (EC 3.1.1.-) (aiPLA2) (Non-selenium glutathione peroxidase) (EC 1.11.1.7) (NSGPx) | 0.00234921 | 0.00084178 | 36% | A, B, C |
| Q64442 | DHSO_MOUSE | 375 | 40091 | 7 | (Q64442) Sorbitol dehydrogenase (EC 1.1.1.14) (L-iditol 2-dehydrogenase) (Fragment) | 0.00231192 | 0.00110087 | 48% | A, B, C |
| P35700 | PRDX1_MOUSE | 199 | 22176 | 8.1 | (P35700) Peroxiredoxin-1 (EC 1.11.1.15) (Thioredoxin peroxidase 2) (Thioredoxin-dependent peroxide reductase 2) (Osteoblast-specific factor 3) (OSF-3) (Macrophage 23 kDa stress protein) | 0.00231008 | 0.00053049 | 23% | A, B, C |
| Q8VC30 | DAK_MOUSE | 578 | 59691 | 6.9 | (Q8VC30) Dihydroxyacetone kinase (EC 2.7.1.29) (Glycerone kinase) (DHA kinase) | 0.00230504 | 0.00143721 | 62% | A, B, C |
| O08749 | DLDH_MOUSE | 509 | 54212 | 7.9 | (O08749) Dihydrolipoyl dehydrogenase, mitochondrial precursor (EC 1.8.1.4) (Dihydrolipoamide dehydrogenase) | 0.00229857 | 0.00048606 | 21% | A, B, C |
| P10639 | THIO_MOUSE | 104 | 11544 | 4.9 | (P10639) Thioredoxin (ATL-derived factor) (ADF) | 0.0022803 | 0.00087485 | 38% | A, B, C |
| P70441 | NHERF_MOUSE | 354 | 38469 | 5.9 | (P70441) Ezrin-radixin-moesin-binding phosphoprotein 50 (EBP50) (Na(+)/H(+) exchange regulatory cofactor NHE-RF) (NHERF-1) (Regulatory cofactor of Na(+)/H(+) exchanger) (Sodium-hydrogen exchanger regulatory factor 1) (Solute carrier family 9 | 0.0022688 | 0.00091604 | 40% | A, B, C |
| Q8K3J1 | NUIM_MOUSE | 212 | 24038 | 6.2 | (Q8K3J1) NADH-ubiquinone oxidoreductase 23 kDa subunit, mitochondrial precursor (EC 1.6.5.3) (EC 1.6.99.3) (Complex I-23KD) (CI-23KD) (TYKY subunit) | 0.0022602 | 0.00039593 | 18% | A, B, C |
| Q9WTP7 | KAD3_MOUSE | 226 | 25295 | 8.8 | (Q9WTP7) GTP:AMP phosphotransferase mitochondrial (EC 2.7.4.10) (Adenylate kinase 3) (AK3) (Adenylate kinase 3 alpha-like 1) | 0.00221864 | 0.00204977 | 92% | A, B, C |
| P68372 | TBB2C_MOUSE | 445 | 49831 | 4.9 | (P68372) Tubulin beta-2C chain | 0.0022164 | 0.00102482 | 46% | A, B, C |
| P68369 | TBA1_MOUSE | 451 | 50136 | 5.1 | (P68369) Tubulin alpha-1 chain (Alpha-tubulin 1) (Alpha-tubulin isotype M-alpha-1) | 0.0021961 | 0.00136087 | 62% | A, B, C |

| | | | | | | | | | |
|---|---|---|---|---|---|---|---|---|---|
| P47738 | ALDH2_MOUSE | 519 | 56538 | 7.6 | (P47738) Aldehyde dehydrogenase, mitochondrial precursor (EC 1.2.1.3) (ALDH class 2) (AHD-M1) (ALDHI) (ALDH-E2) | 0.00218838 | 0.00047383 | 22% | A, B, C |
| P68433 | H31_MOUSE | 135 | 15273 | 11.1 | (P68433) Histone H3.1 | 0.00217481 | 0.00205587 | 95% | A, B, C |
| P06745 | G6PI_MOUSE | 557 | 62637 | 7.9 | (P06745) Glucose-6-phosphate isomerase (EC 5.3.1.9) (GPI) (Phosphoglucose isomerase) (PGI) (Phosphohexose isomerase) (PHI) (Neuroleukin) (NLK) | 0.00211186 | 0.00096155 | 46% | A, B, C |
| P20108 | PRDX3_MOUSE | 257 | 28127 | 7.6 | (P20108) Thioredoxin-dependent peroxide reductase, mitochondrial precursor (EC 1.11.1.15) (Perioredoxin-3) (PRX III) (Antioxidant protein 1) (AOP-1) (Protein MER5) | 0.00209646 | 0.00131338 | 63% | A, B, C |
| P20029 | GRP78_MOUSE | 655 | 72422 | 5.2 | (P20029) 78 kDa glucose-regulated protein precursor (GRP 78) (Immunoglobulin heavy chain-binding protein) (BiP) | 0.00208008 | 0.00034157 | 16% | A, B, C |
| Q9D3D9 | ATPD_MOUSE | 168 | 17600 | 5.1 | (Q9D3D9) ATP synthase delta chain, mitochondrial precursor (EC 3.6.3.14) | 0.00204292 | 0.00184216 | 90% | A, B, C |
| Q60930 | VDAC2_MOUSE | 295 | 31733 | 7.5 | (Q60930) Voltage-dependent anion-selective channel protein 2 (VDAC-2) (mVDAC2) (mVDAC6) (Outer mitochondrial membrane protein porin 2) | 0.00202604 | 0.00155372 | 77% | A, B, C |
| Q9DB77 | UQCR2_MOUSE | 453 | 48235 | 9.3 | (Q9DB77) Ubiquinol-cytochrome-c reductase complex core protein 2, mitochondrial precursor (EC 1.10.2.2) (Complex III subunit II) | 0.00201318 | 7.4318E-05 | 4% | A, B, C |
| P63017 | HSP7C_MOUSE | 646 | 70871 | 5.5 | (P63017) Heat shock cognate 71 kDa protein (Heat shock 70 kDa protein 8) | 0.00194735 | 0.00016197 | 8% | A, B, C |
| P11588 | MUP1_MOUSE | 180 | 20648 | 5.1 | (P11588) Major urinary protein 1 precursor (MUP 1) | 0.00191624 | 0.00194586 | 102% | A, B, C |
| Q9DBJ1 | PGAM1_MOUSE | 253 | 28701 | 7.2 | (Q9DBJ1) Phosphoglycerate mutase 1 (EC 5.4.2.1) (EC 5.4.2.4) (EC 3.1.3.13) (Phosphoglycerate mutase isozyme B) (PGAM-B) (BPG-dependent PGAM 1) | 0.00189827 | 0.00082124 | 43% | A, B, C |
| P60710 | ACTB_MOUSE | 375 | 41737 | 5.5 | (P60710) Actin, cytoplasmic 1 (Beta-actin) | 0.00189076 | 0.00165225 | 87% | A, B, C |
| P27773 | PDIA3_MOUSE | 504 | 56621 | 6.4 | (P27773) Protein disulfide-isomerase A3 precursor (EC 5.3.4.1) (Disulfide isomerase ER-60) (ERp60) (58 kDa microsomal protein) (p58) (ERp57) | 0.00188164 | 0.00027331 | 15% | A, B, C |
| Q9D051 | ODPB_MOUSE | 359 | 38937 | 6.9 | (Q9D051) Pyruvate dehydrogenase E1 component subunit beta, mitochondrial precursor (EC 1.2.4.1) (PDHE1-B) | 0.00187398 | 0.001037 | 55% | A, B, C |
| Q9D1K2 | VATF_MOUSE | 119 | 13370 | 5.8 | (Q9D1K2) Vacuolar ATP synthase subunit F (EC 3.6.3.14) (V-ATPase F subunit) (Vacuolar proton pump F subunit) (V-ATPase 14 kDa subunit) | 0.00183394 | 0.00180165 | 98% | A, B, C |
| Q9D1A2 | CPGL1_MOUSE | 475 | 52767 | 5.7 | (Q9D1A2) Cytosolic nonspecific dipeptidase (Glutamate carboxypeptidase-like protein 1) (CNDP dipeptidase 2) | 0.00181927 | 0.00053191 | 29% | A, B, C |
| P05064 | ALDOA_MOUSE | 363 | 39225 | 8.1 | (P05064) Fructose-bisphosphate aldolase A (EC 4.1.2.13) (Muscle-type aldolase) (Aldolase 1) | 0.00180327 | 0.00099091 | 55% | A, B, C |
| Q9D6J6 | NUHM_MOUSE | 248 | 27315 | 7.4 | (Q9D6J6) NADH-ubiquinone oxidoreductase 24 kDa subunit, mitochondrial precursor (EC 1.6.5.3) (EC 1.6.99.3) | 0.00177458 | 0.0005936 | 33% | A, B, C |
| P62814 | VATB2_MOUSE | 511 | 56551 | 5.8 | (P62814) Vacuolar ATP synthase subunit B, brain isoform (EC 3.6.3.14) (V-ATPase B2 subunit) (Vacuolar proton pump B isoform 2) (Endomembrane proton pump 58 kDa subunit) | 0.00175967 | 0.00024953 | 14% | A, B, C |
| Q05920 | PYC_MOUSE | 1178 | 129685 | 6.7 | (Q05920) Pyruvate carboxylase, mitochondrial precursor (EC 6.4.1.1) (Pyruvic carboxylase) (PCB) | 0.00173049 | 0.00040149 | 23% | A, B, C |
| P24369 | PPIB_MOUSE | 208 | 22713 | 9.5 | (P24369) Peptidyl-prolyl cis-trans isomerase B precursor (EC 5.2.1.8) (PPIase) (Rotamase) (Cyclophilin B) (S-cyclophilin) (SCYLP) (CYP-S1) | 0.00172098 | 0.00121851 | 71% | A, B, C |

| | | | | | | | | | |
|---|---|---|---|---|---|---|---|---|---|
| P08113 | ENPL_MOUSE | 802 | 92476 | 4.8 | (P08113) Endoplasmin precursor (Heat shock protein 90 kDa beta member 1) (94 kDa glucose-regulated protein) (GRP94) (ERP99) (Polymorphic tumor rejection antigen 1) (Tumor rejection antigen gp96) | 0.001694 | 0.00050099 | 30% | A, B, C |
| Q8BG05 | ROA3_MOUSE | 379 | 39652 | 9 | (Q8BG05) Heterogeneous nuclear ribonucleoprotein A3 (hnRNP A3) | 0.00168952 | 0.0006399 | 38% | A, B, C |
| P62264 | RS14_MOUSE | 150 | 16141 | 10.1 | (P62264) 40S ribosomal protein S14 | 0.00168704 | 0.00101378 | 60% | A, B, C |
| P62075 | TIM13_MOUSE | 95 | 10458 | 8.2 | (P62075) Mitochondrial import inner membrane translocase subunit Tim13 | 0.00163343 | 0.00120886 | 74% | A, B, C |
| P34884 | MIF_MOUSE | 114 | 12373 | 7.3 | (P34884) Macrophage migration inhibitory factor (MIF) (Phenylpyruvate tautomerase) (EC 5.3.2.1) (Glycosylation-inhibiting factor) (GIF) (Delayed early response protein 6) (DER6) | 0.0016279 | 0.00057125 | 35% | A, B, C |
| P97450 | ATP5J_MOUSE | 108 | 12496 | 9.4 | (P97450) ATP synthase coupling factor 6, mitochondrial precursor (EC 3.6.3.14) (ATPase subunit F6) | 0.00162373 | 0.00075955 | 47% | A, B, C |
| Q8BVI4 | DHPR_MOUSE | 241 | 25570 | 7.8 | (Q8BVI4) Dihydropteridine reductase (EC 1.5.1.34) (HDHPR) (Quinoid dihydropteridine reductase) | 0.00162353 | 0.00067992 | 42% | A, B, C |
| Q9CWS0 | DDAH1_MOUSE | 284 | 31250 | 6 | (Q9CWS0) NG,NG-dimethylarginine dimethylaminohydrolase 1 (EC 3.5.3.18) (Dimethylargininase-1) (Dimethylarginine dimethylaminohydrolase 1) (DDAHI) (DDAH-1) | 0.00161866 | 0.0012234 | 76% | A, B, C |
| P19783 | COX41_MOUSE | 169 | 19530 | 9.2 | (P19783) Cytochrome c oxidase subunit 4 isoform 1, mitochondrial precursor (EC 1.9.3.1) (Cytochrome c oxidase subunit IV isoform 1) (COX IV-1) (Cytochrome c oxidase polypeptide IV) | 0.00161138 | 0.00106074 | 66% | A, B, C |
| P63101 | 1433Z_MOUSE | 245 | 27771 | 4.8 | (P63101) 14-3-3 protein zeta/delta (Protein kinase C inhibitor protein 1) (KCIP-1) (SEZ-2) | 0.00159858 | 0.00103023 | 64% | A, B, C |
| Q9CQ19 | MLRN_MOUSE | 171 | 19723 | 4.9 | (Q9CQ19) Myosin regulatory light chain 2, smooth muscle isoform (Myosin RLC) (Myosin regulatory light chain 9) | 0.00158382 | 0.00026445 | 17% | A, B, C |
| P19157 | GSTP1_MOUSE | 209 | 23478 | 7.8 | (P19157) Glutathione S-transferase P 1 (EC 2.5.1.18) (GST YF-YF) (GST-piB) (GST class-pi) (Gst P1) (Preadipocyte growth factor) | 0.00158352 | 0.00074276 | 47% | A, B, C |
| P51410 | RL9_MOUSE | 192 | 21881 | 10 | (P51410) 60S ribosomal protein L9 | 0.00157879 | 0.00088244 | 56% | A, B, C |
| P97807 | FUMH_MOUSE | 507 | 54371 | 9 | (P97807) Fumarate hydratase, mitochondrial precursor (EC 4.2.1.2) (Fumarase) (EF-3) | 0.00153827 | 0.00075792 | 49% | A, B, C |
| Q9D2G2 | ODO2_MOUSE | 454 | 48995 | 9 | (Q9D2G2) Dihydrolipoyllysine-residue succinyltransferase component of 2-oxoglutarate dehydrogenase complex, mitochondrial precursor (EC 2.3.1.61) (Dihydrolipoamide succinyltransferase component of 2-oxoglutarate dehydrogenase complex) (E2) (E2 | 0.00149864 | 0.00056429 | 38% | A, B, C |
| P40142 | TKT_MOUSE | 623 | 67631 | 7.5 | (P40142) Transketolase (EC 2.2.1.1) (TK) (P68) | 0.0014552 | 0.00062158 | 43% | A, B, C |
| Q9R0H0 | ACOX1_MOUSE | 661 | 74634 | 8.6 | (Q9R0H0) Acyl-coenzyme A oxidase 1, peroxisomal (EC 1.3.3.6) (Palmitoyl-CoA oxidase) (AOX) | 0.00141945 | 0.00027807 | 20% | A, B, C |
| Q91XE4 | ACY3_MOUSE | 318 | 35286 | 5.5 | (Q91XE4) Aspartoacylase-2 (EC 3.5.1.15) (Aminoacylase-3) (ACY-3) (Acylase III) (Hepatitis C virus core-binding protein 1) (HCBP1) | 0.00139101 | 0.00108552 | 78% | A, B, C |
| Q9JHI5 | IVD_MOUSE | 424 | 46325 | 8.3 | (Q9JHI5) Isovaleryl-CoA dehydrogenase, mitochondrial precursor (EC 1.3.99.10) (IVD) | 0.00138081 | 0.00032061 | 23% | A, B, C |
| Q9D6R2 | IDH3A_MOUSE | 366 | 39639 | 6.7 | (Q9D6R2) Isocitrate dehydrogenase [NAD] subunit alpha, mitochondrial precursor (EC 1.1.1.41) (Isocitric dehydrogenase) (NAD(+)-specific ICDH) | 0.00137856 | 0.00024087 | 17% | A, B, C |

| | | | | | | | | | |
|---|---|---|---|---|---|---|---|---|---|
| P45952 | ACADM_MOUSE | 421 | 46481 | 8.4 | (P45952) Medium-chain specific acyl-CoA dehydrogenase, mitochondrial precursor (EC 1.3.99.3) (MCAD) | 0.00134177 | 0.00080242 | 60% | A, B, C |
| P10649 | GSTM1_MOUSE | 217 | 25839 | 8 | (P10649) Glutathione S-transferase Mu 1 (EC 2.5.1.18) (GST class-mu 1) (Glutathione S-transferase GT8.7) (pmGT10) (GST 1-1) | 0.00133476 | 0.00018258 | 14% | A, B, C |
| P62821 | RAB1A_MOUSE | 204 | 22547 | 6.2 | (P62821) Ras-related protein Rab-1A (YPT1-related protein) | 0.00132345 | 0.00112962 | 85% | A, B, C |
| P62315 | SMD1_MOUSE | 119 | 13282 | 11.6 | (P62315) Small nuclear ribonucleoprotein Sm D1 (snRNP core protein D1) (Sm-D1) (Sm-D autoantigen) | 0.00128352 | 0.00092478 | 72% | A, B, C |
| P62259 | 1433E_MOUSE | 255 | 29174 | 4.7 | (P62259) 14-3-3 protein epsilon (14-3-3E) | 0.00128042 | 0.00053564 | 42% | A, B, C |
| P56389 | CDD_MOUSE | 146 | 16131 | 5.6 | (P56389) Cytidine deaminase (EC 3.5.4.5) (Cytidine aminohydrolase) | 0.00127465 | 0.00029873 | 23% | A, B, C |
| P11862 | GAS2_MOUSE | 314 | 34901 | 8.6 | (P11862) Growth-arrest-specific protein 2 (GAS-2) | 0.00126846 | 0.00048912 | 39% | A, B, C |
| Q8VCT4 | CES3_MOUSE | 565 | 61788 | 6.6 | (Q8VCT4) Carboxylesterase 3 precursor (EC 3.1.1.1) (Triacylglycerol hydrolase) (TGH) | 0.00126368 | 0.00077482 | 61% | A, B, C |
| Q9CZ13 | UQCR1_MOUSE | 480 | 52769 | 6.1 | (Q9CZ13) Ubiquinol-cytochrome-c reductase complex core protein I, mitochondrial precursor (EC 1.10.2.2) | 0.00123736 | 0.00027017 | 22% | A, B, C |
| P19536 | COX5B_MOUSE | 128 | 13813 | 8.4 | (P19536) Cytochrome c oxidase polypeptide Vb, mitochondrial precursor (EC 1.9.3.1) | 0.00121549 | 0.00077651 | 64% | A, B, C |
| P51150 | RAB7_MOUSE | 207 | 23490 | 6.7 | (P51150) Ras-related protein Rab-7 | 0.00120966 | 0.00054444 | 45% | A, B, C |
| Q9JIL4 | PDZK1_MOUSE | 519 | 56499 | 5.4 | (Q9JIL4) PDZ domain-containing protein 1 (CFTR-associated protein of 70 kDa) (Na/Pi cotransporter C-terminal-associated protein) (NaPi-Cap1) (Na(+)/H(+) exchanger regulatory factor 3) (Sodium-hydrogen exchanger regulatory factor 3) | 0.00120323 | 0.0002071 | 17% | A, B, C |
| Q91WS0 | CJ070_MOUSE | 108 | 12097 | 9.1 | (Q91WS0) Protein C10orf70 homolog | 0.00120203 | 0.00098107 | 82% | A, B, C |
| P70296 | PEBP1_MOUSE | 186 | 20699 | 5.4 | (P70296) Phosphatidylethanolamine-binding protein 1 (PEBP-1) (HCNPpp) [Contains: Hippocampal cholinergic neurostimulating peptide (HCNP)] | 0.00119192 | 0.00072658 | 61% | A, B, C |
| P26443 | DHE3_MOUSE | 558 | 61337 | 8 | (P26443) Glutamate dehydrogenase 1, mitochondrial precursor (EC 1.4.1.3) (GDH) | 0.00118834 | 0.00012382 | 10% | A, B, C |
| P56135 | ATPK_MOUSE | 87 | 10213 | 9.9 | (P56135) ATP synthase f chain, mitochondrial (EC 3.6.3.14) | 0.00116048 | 0.00035624 | 31% | A, B, C |
| P47963 | RL13_MOUSE | 210 | 24174 | 11.5 | (P47963) 60S ribosomal protein L13 (A52) | 0.0011547 | 0.00051703 | 45% | A, B, C |
| P50516 | VATA1_MOUSE | 617 | 68268 | 5.9 | (P50516) Vacuolar ATP synthase catalytic subunit A, ubiquitous isoform (EC 3.6.3.14) (V-ATPase subunit A 1) (Vacuolar proton pump alpha subunit 1) (V-ATPase 69 kDa subunit 1) | 0.00115424 | 0.0003695 | 32% | A, B, C |
| Q01853 | TERA_MOUSE | 805 | 89177 | 5.3 | (Q01853) Transitional endoplasmic reticulum ATPase (TER ATPase) (15S Mg(2+)-ATPase p97 subunit) (Valosin-containing protein) (VCP) | 0.00115124 | 0.00025729 | 22% | A, B, C |
| Q9CQQ7 | AT5F1_MOUSE | 256 | 28949 | 9.1 | (Q9CQQ7) ATP synthase B chain, mitochondrial precursor (EC 3.6.3.14) | 0.00114744 | 0.00086236 | 75% | A, B, C |
| Q9Z1P6 | NDUA7_MOUSE | 112 | 12444 | 10.2 | (Q9Z1P6) NADH dehydrogenase [ubiquinone] 1 alpha subcomplex subunit 7 (EC 1.6.5.3) (EC 1.6.99.3) (NADH-ubiquinone oxidoreductase subunit B14.5a) (Complex I-B14.5a) (CI-B14.5a) | 0.00113601 | 0.00059028 | 52% | A, B, C |
| Q9DCS3 | MECR_MOUSE | 373 | 40343 | 9.1 | (Q9DCS3) Trans-2-enoyl-CoA reductase, mitochondrial precursor (EC 1.3.1.38) | 0.0011303 | 0.00064288 | 57% | A, B, C |
| P35278 | RAB5C_MOUSE | 216 | 23412 | 8.4 | (P35278) Ras-related protein Rab-5C | 0.00112453 | 0.00034758 | 31% | A, B, C |
| Q99LX0 | PARK7_MOUSE | 189 | 20021 | 6.8 | (Q99LX0) Protein DJ-1 | 0.00112434 | 0.00052566 | 47% | A, B, C |
| O35215 | DOPD_MOUSE | 117 | 12946 | 6.5 | (O35215) D-dopachrome decarboxylase (EC 4.1.1.84) (D-dopachrome tautomerase) | 0.00110707 | 0.00049152 | 44% | A, B, C |

| | | | | | | | | | |
|---|---|---|---|---|---|---|---|---|---|
| Q04646 | ATNG_MOUSE | 70 | 7520 | 7.2 | (Q04646) Sodium/potassium-transporting ATPase gamma chain (Sodium pump gamma chain) (Na+/K+ ATPase subunit gamma) (FXYD domain-containing ion transport regulator 2) | 0.00110554 | 0.00021014 | 19% | A, B, C |
| Q9WVA4 | TAGL2_MOUSE | 211 | 23466 | 7.1 | (Q9WVA4) Transgelin-2 | 0.00110377 | 0.00043417 | 39% | A, B, C |
| Q8VCR7 | AB14B_MOUSE | 210 | 22451 | 6.3 | (Q8VCR7) Abhydrolase domain-containing protein 14B (CCG1-interacting factor B) | 0.00110288 | 0.00078053 | 71% | A, B, C |
| Q9DBM2 | ECHP_MOUSE | 717 | 78112 | 9.2 | (Q9DBM2) Peroxisomal bifunctional enzyme (PBE) (PBFE) [Includes: Enoyl-CoA hydratase (EC 4.2.1.17); 3,2-trans-enoyl-CoA isomerase (EC 5.3.3.8); 3-hydroxyacyl-CoA dehydrogenase (EC 1.1.1.35)] | 0.00109489 | 0.00016668 | 15% | A, B, C |
| P48036 | ANXA5_MOUSE | 319 | 35752 | 5 | (P48036) Annexin A5 (Annexin V) (Lipocortin V) (Endonexin II) (Calphobindin I) (CBP-I) (Placental anticoagulant protein I) (PAP-I) (PP4) (Thromboplastin inhibitor) (Vascular anticoagulant-alpha) (VAC-alpha) (Anchorin CII) | 0.00109366 | 0.00072612 | 66% | A, B, C |
| Q9CR61 | NDUB7_MOUSE | 136 | 16200 | 8.2 | (Q9CR61) NADH dehydrogenase [ubiquinone] 1 beta subcomplex subunit 7 (EC 1.6.5.3) (EC 1.6.99.3) (NADH-ubiquinone oxidoreductase B18 subunit) (Complex I-B18) (CI-B18) | 0.00107903 | 0.00036262 | 34% | A, B, C |
| O09044 | SNP23_MOUSE | 210 | 23261 | 5 | (O09044) Synaptosomal-associated protein 23 (SNAP-23) (Vesicle-membrane fusion protein SNAP-23) (Syndet) | 0.00107622 | 0.00041284 | 38% | A, B, C |
| P16546 | SPTA2_MOUSE | 1458 | 167552 | 5.4 | (P16546) Spectrin alpha chain, brain (Spectrin, non-erythroid alpha chain) (Alpha-II spectrin) (Fodrin alpha chain) (Fragment) | 0.00106935 | 0.00036766 | 34% | A, B, C |
| P50544 | ACADV_MOUSE | 656 | 70876 | 8.7 | (P50544) Very-long-chain specific acyl-CoA dehydrogenase, mitochondrial precursor (EC 1.3.99.-) (VLCAD) (MVLCAD) | 0.00106872 | 0.00021229 | 20% | A, B, C |
| P52480 | KPYM_MOUSE | 530 | 57756 | 7.5 | (P52480) Pyruvate kinase isozyme M2 (EC 2.7.1.40) | 0.00106721 | 0.00070304 | 66% | A, B, C |
| Q9Z2Y8 | PROSC_MOUSE | 274 | 30049 | 8.3 | (Q9Z2Y8) Proline synthetase co-transcribed bacterial homolog protein | 0.0010659 | 0.00043587 | 41% | A, B, C |
| P28271 | IREB1_MOUSE | 889 | 98179 | 7.5 | (P28271) Iron-responsive element-binding protein 1 (IRE-BP 1) (Iron regulatory protein 1) (IRP1) (Ferritin repressor protein) (Aconitate hydratase) (EC 4.2.1.3) (Citrate hydro-lyase) (Aconitase) | 0.00105918 | 0.00011156 | 11% | A, B, C |
| Q9CQA3 | DHSB_MOUSE | 282 | 31814 | 8.7 | (Q9CQA3) Succinate dehydrogenase [ubiquinone] iron-sulfur protein, mitochondrial precursor (EC 1.3.5.1) (Ip) (Iron-sulfur subunit of complex II) | 0.00105621 | 0.00059116 | 56% | A, B, C |
| Q9JLJ2 | AL9A1_MOUSE | 494 | 53515 | 7 | (Q9JLJ2) 4-trimethylaminobutyraldehyde dehydrogenase (EC 1.2.1.47) (TMABADH) (Aldehyde dehydrogenase 9A1) (EC 1.2.1.3) | 0.0010467 | 0.00076236 | 73% | A, B, C |
| Q9CQR4 | THEM2_MOUSE | 140 | 15183 | 8.8 | (Q9CQR4) Thioesterase superfamily member 2 | 0.00102563 | 0.00025309 | 25% | A, B, C |
| Q9DBP5 | KCY_MOUSE | 196 | 22165 | 5.8 | (Q9DBP5) UMP-CMP kinase (EC 2.7.4.14) (Cytidylate kinase) (Deoxycytidylate kinase) (Cytidine monophosphate kinase) (Uridine monophosphate/cytidine monophosphate kinase) (UMP/CMP kinase) (UMP/CMPK) (Uridine monophosphate kinase) | 0.00100777 | 0.00036536 | 36% | A, B, C |
| Q62093 | SFRS2_MOUSE | 220 | 25345 | 11.9 | (Q62093) Splicing factor, arginine/serine-rich 2 (Splicing factor SC35) (SC-35) (Splicing component, 35 kDa) (Protein PR264) (Putative myelin regulatory factor 1) (MRF-1) | 0.00100726 | 0.00050425 | 50% | A, B, C |

| | | | | | | | | | |
|---|---|---|---|---|---|---|---|---|---|
| Q61171 | PRDX2_MOUSE | 197 | 21647 | 5.4 | (Q61171) Peroxiredoxin-2 (EC 1.11.1.15) (Thioredoxin peroxidase 1) (Thioredoxin-dependent peroxide reductase 1) (Thiol-specific antioxidant protein) (TSA) | 0.00100556 | 0.00053416 | 53% | A, B, C |
| Q63886 | UD11_MOUSE | 535 | 60124 | 8.6 | (Q63886) UDP-glucuronosyltransferase 1-1 precursor (EC 2.4.1.17) (UDPGT) (UGT1*1) (UGT1-01) (UGT1.1) (UGT1A1) (UGTBR1) | 0.00100527 | 0.00078046 | 78% | A, B, C |
| Q01768 | NDKB_MOUSE | 152 | 17363 | 7.5 | (Q01768) Nucleoside diphosphate kinase B (EC 2.7.4.6) (NDK B) (NDP kinase B) (nm23-M2) (P18) | 0.0009931 | 0.00076148 | 77% | A, B, C |
| Q8CAQ8 | IMMT_MOUSE | 757 | 83900 | 6.6 | (Q8CAQ8) Mitochondrial inner membrane protein (Mitofilin) | 0.000985 | 0.00031738 | 32% | A, B, C |
| P35980 | RL18_MOUSE | 187 | 21513 | 11.8 | (P35980) 60S ribosomal protein L18 | 0.00097498 | 0.00095482 | 98% | A, B, C |
| Q9Z2V4 | PPCKC_MOUSE | 622 | 69355 | 6.6 | (Q9Z2V4) Phosphoenolpyruvate carboxykinase, cytosolic [GTP] (EC 4.1.1.32) (Phosphoenolpyruvate carboxylase) (PEPCK-C) | 0.00096573 | 0.00018846 | 20% | A, B, C |
| Q99JR1 | SFXN1_MOUSE | 321 | 35518 | 9.2 | (Q99JR1) Sideroflexin-1 | 0.00096331 | 0.0010734 | 111% | A, B, C |
| O08756 | HCD2_MOUSE | 260 | 27287 | 8.4 | (O08756) 3-hydroxyacyl-CoA dehydrogenase type-2 (EC 1.1.1.35) (3-hydroxyacyl-CoA dehydrogenase type II) (Type II HADH) (3-hydroxy-2-methylbutyryl-CoA dehydrogenase) (EC 1.1.1.178) (Endoplasmic reticulum-associated amyloid beta-peptide-binding | 0.00096132 | 0.00017073 | 18% | A, B, C |
| P62855 | RS26_MOUSE | 114 | 12884 | 11 | (P62855) 40S ribosomal protein S26 | 0.00095268 | 0.00058302 | 61% | A, B, C |
| Q60605 | MYL6_MOUSE | 150 | 16799 | 4.7 | (Q60605) Myosin light polypeptide 6 (Smooth muscle and nonmuscle myosin light chain alkali 6) (Myosin light chain alkali 3) (Myosin light chain 3) (MLC-3) (LC17) | 0.00094738 | 0.00093083 | 98% | A, B, C |
| Q921G7 | ETFD_MOUSE | 616 | 68091 | 7.6 | (Q921G7) Electron transfer flavoprotein-ubiquinone oxidoreductase, mitochondrial precursor (EC 1.5.5.1) (ETF-QO) (ETF-ubiquinone oxidoreductase) (ETF dehydrogenase) (Electron-transferring-flavoprotein dehydrogenase) | 0.00094671 | 0.00073106 | 77% | A, B, C |
| P61979 | HNRPK_MOUSE | 463 | 50976 | 5.5 | (P61979) Heterogeneous nuclear ribonucleoprotein K | 0.00094441 | 0.00037835 | 40% | A, B, C |
| Q91VD9 | NUAM_MOUSE | 727 | 79749 | 5.7 | (Q91VD9) NADH-ubiquinone oxidoreductase 75 kDa subunit, mitochondrial precursor (EC 1.6.5.3) (EC 1.6.99.3) (Complex I-75Kd) (CI-75Kd) | 0.00093817 | 0.00011039 | 12% | A, B, C |
| P62991 | UBIQ_MOUSE | 76 | 8565 | 7.2 | (P62991) Ubiquitin | 0.00093614 | 0.00057146 | 61% | A, B, C |
| P57780 | ACTN4_MOUSE | 912 | 104977 | 5.4 | (P57780) Alpha-actinin-4 (Non-muscle alpha-actinin 4) (F-actin cross linking protein) | 0.00092544 | 0.00021055 | 23% | A, B, C |
| P13707 | GPDA_MOUSE | 348 | 37442 | 7.2 | (P13707) Glycerol-3-phosphate dehydrogenase [NAD+], cytoplasmic (EC 1.1.1.8) (GPD-C) (GPDH-C) | 0.00092308 | 0.00093265 | 101% | A, B, C |
| P62918 | RL8_MOUSE | 256 | 27893 | 11 | (P62918) 60S ribosomal protein L8 | 0.0009063 | 0.00054467 | 60% | A, B, C |
| P62242 | RS8_MOUSE | 207 | 24074 | 10.3 | (P62242) 40S ribosomal protein S8 | 0.00090113 | 0.00043144 | 48% | A, B, C |
| Q9Z2I9 | SUCB1_MOUSE | 463 | 50114 | 7 | (Q9Z2I9) Succinyl-CoA ligase [ADP-forming] beta-chain, mitochondrial precursor (EC 6.2.1.5) (Succinyl-CoA synthetase, betaA chain) (SCS-betaA) (ATP-specific succinyl-CoA synthetase subunit beta) | 0.00088747 | 0.00062538 | 70% | A, B, C |
| P62082 | RS7_MOUSE | 194 | 22127 | 10.1 | (P62082) 40S ribosomal protein S7 | 0.00087975 | 0.00059028 | 67% | A, B, C |
| Q99K67 | AASS_MOUSE | 926 | 102975 | 6.9 | (Q99K67) Alpha-aminoadipic semialdehyde synthase, mitochondrial precursor (LKR/SDH) [Includes: Lysine ketoglutarate reductase (EC 1.5.1.8) (LOR) (LKR); Saccharopine dehydrogenase (EC 1.5.1.9) (SDH)] | 0.00087554 | 0.0001842 | 21% | A, B, C |
| Q8BFR5 | EFTU_MOUSE | 452 | 49508 | 7.6 | (Q8BFR5) Elongation factor Tu, mitochondrial precursor | 0.00087152 | 0.00042853 | 49% | A, B, C |
| P19324 | HSP47_MOUSE | 417 | 46590 | 8.8 | (P19324) 47 kDa heat shock protein precursor (Collagen-binding protein 1) (Serine protease inhibitor J6) | 0.00086321 | 0.00039479 | 46% | A, B, C |

| | | | | | | | | | |
|---|---|---|---|---|---|---|---|---|---|
| P50518 | VATE_MOUSE | 228 | 26588 | 9.2 | (P50518) Vacuolar ATP synthase subunit E (EC 3.6.3.14) (V-ATPase E subunit) (Vacuolar proton pump E subunit) (V-ATPase 31 kDa subunit) (P31) | 0.00085965 | 0.00082363 | 96% | A, B, C |
| Q64727 | VINC_MOUSE | 1065 | 116586 | 6 | (Q64727) Vinculin (Metavinculin) | 0.00084116 | 6.3863E-05 | 8% | A, B, C |
| Q9WUK2 | IF4H_MOUSE | 247 | 27210 | 7.2 | (Q9WUK2) Eukaryotic translation initiation factor 4H (eIF-4H) (Williams-Beuren syndrome chromosome region 1 protein homolog) | 0.00083746 | 0.00016954 | 20% | A, B, C |
| Q60597 | ODO1_MOUSE | 1019 | 116118 | 7 | (Q60597) 2-oxoglutarate dehydrogenase E1 component, mitochondrial precursor (EC 1.2.4.2) (Alpha-ketoglutarate dehydrogenase) | 0.00082799 | 0.00054689 | 66% | A, B, C |
| P40124 | CAP1_MOUSE | 473 | 51444 | 7.5 | (P40124) Adenylyl cyclase-associated protein 1 (CAP 1) | 0.0008257 | 0.00034165 | 41% | A, B, C |
| Q9DB15 | RM12_MOUSE | 201 | 21708 | 9.3 | (Q9DB15) 39S ribosomal protein L12, mitochondrial precursor (L12mt) (MRP-L12) | 0.00081999 | 0.00019618 | 24% | A, B, C |
| P62305 | RUXE_MOUSE | 92 | 10804 | 9.4 | (P62305) Small nuclear ribonucleoprotein E (snRNP-E) (Sm protein E) (Sm-E) (SmE) | 0.00081921 | 0.00078058 | 95% | A, B, C |
| Q9DCT2 | NUGM_MOUSE | 263 | 30207 | 6.9 | (Q9DCT2) NADH-ubiquinone oxidoreductase 30 kDa subunit, mitochondrial precursor (EC 1.6.5.3) (EC 1.6.99.3) (Complex I-30KD) (CI-30KD) | 0.00081919 | 0.00018116 | 22% | A, B, C |
| Q9QXX4 | CMC2_MOUSE | 676 | 74467 | 8.6 | (Q9QXX4) Calcium-binding mitochondrial carrier protein Aralar2 (Mitochondrial aspartate glutamate carrier 2) (Solute carrier family 25 member 13) (Citrin) | 0.00081684 | 0.00013826 | 17% | A, B, C |
| P32020 | NLTP_MOUSE | 547 | 59126 | 7.4 | (P32020) Nonspecific lipid-transfer protein (EC 2.3.1.176) (Propanoyl-CoA C-acyltransferase) (NSL-TP) (Sterol carrier protein 2) (SCP-2) (Sterol carrier protein X) (SCP-X) (SCP-chi) (SCPX) | 0.00081273 | 0.00061068 | 75% | A, B, C |
| Q9Z0X1 | PDCD8_MOUSE | 612 | 66766 | 9.2 | (Q9Z0X1) Programmed cell death protein 8, mitochondrial precursor (EC 1.-.-.-) (Apoptosis-inducing factor) | 0.00080973 | 0.00021456 | 26% | A, B, C |
| Q9DC69 | NDUA9_MOUSE | 377 | 42509 | 9.7 | (Q9DC69) NADH dehydrogenase [ubiquinone] 1 alpha subcomplex subunit 9, mitochondrial precursor (EC 1.6.5.3) (EC 1.6.99.3) (NADH-ubiquinone oxidoreductase 39 kDa subunit) (Complex I-39KD) (CI-39KD) | 0.00080039 | 0.00040234 | 50% | A, B, C |
| P14211 | CRTC_MOUSE | 416 | 47995 | 4.5 | (P14211) Calreticulin precursor (CRP55) (Calregulin) (HACBP) (ERp60) | 0.00079894 | 0.00074382 | 93% | A, B, C |
| Q9CR51 | VATG1_MOUSE | 117 | 13593 | 8 | (Q9CR51) Vacuolar ATP synthase subunit G 1 (EC 3.6.3.14) (V-ATPase G subunit 1) (Vacuolar proton pump G subunit 1) (V-ATPase 13 kDa subunit 1) | 0.00079627 | 0.00052659 | 66% | A, B, C |
| Q3UIU2 | NDUB6_MOUSE | 127 | 15384 | 9.8 | (Q3UIU2) NADH dehydrogenase [ubiquinone] 1 beta subcomplex subunit 6 (EC 1.6.5.3) (EC 1.6.99.3) (NADH-ubiquinone oxidoreductase B17 subunit) (Complex I-B17) (CI-B17) | 0.00079174 | 0.00046459 | 59% | A, B, C |
| Q99KP3 | CRYL1_MOUSE | 318 | 35078 | 5.9 | (Q99KP3) Lambda-crystallin homolog | 0.00079019 | 0.00052376 | 66% | A, B, C |
| P80314 | TCPB_MOUSE | 534 | 57346 | 6.4 | (P80314) T-complex protein 1 subunit beta (TCP-1-beta) (CCT-beta) | 0.00077269 | 0.00027321 | 35% | A, B, C |
| Q9CZR8 | EFTS_MOUSE | 324 | 35334 | 7.1 | (Q9CZR8) Elongation factor Ts, mitochondrial precursor (EF-Ts) (EF-TsMt) | 0.00077214 | 0.00067969 | 88% | A, B, C |
| P48962 | ADT1_MOUSE | 297 | 32773 | 9.7 | (P48962) ADP/ATP translocase 1 (Adenine nucleotide translocator 1) (ANT 1) (ADP,ATP carrier protein 1) (Solute carrier family 25 member 4) (ADP,ATP carrier protein, heart/skeletal muscle isoform T1) (mANC1) | 0.00076811 | 0.00067135 | 87% | A, B, C |

| | | | | | | | | | |
|---|---|---|---|---|---|---|---|---|---|
| P00405 | COX2_MOUSE | 227 | 25976 | 4.7 | (P00405) Cytochrome c oxidase subunit 2 (EC 1.9.3.1) (Cytochrome c oxidase polypeptide II) | 0.00076561 | 0.00041011 | 54% | A, B, C |
| Q9ERS2 | NDUAD_MOUSE | 143 | 16728 | 9.5 | (Q9ERS2) NADH dehydrogenase [ubiquinone] 1 alpha subcomplex subunit 13 (EC 1.6.5.3) (EC 1.6.99.3) (NADH-ubiquinone oxidoreductase B16.6 subunit) (Complex I-B16.6) (CI B16.6) (Gene associated with retinoic-interferon-induced mortality 19 prote | 0.00075631 | 0.00031678 | 42% | A, B, C |
| Q8CG76 | ARK72_MOUSE | 367 | 40598 | 8.1 | (Q8CG76) Aflatoxin B1 aldehyde reductase member 2 (EC 1.-.-.-) | 0.0007548 | 0.00063153 | 84% | A, B, C |
| Q99J99 | THTM_MOUSE | 296 | 32892 | 6.6 | (Q99J99) 3-mercaptopyruvate sulfurtransferase (EC 2.8.1.2) (MST) | 0.00074857 | 0.00052011 | 69% | A, B, C |
| P09671 | SODM_MOUSE | 222 | 24603 | 8.6 | (P09671) Superoxide dismutase [Mn], mitochondrial precursor (EC 1.15.1.1) | 0.00073818 | 0.00035864 | 49% | A, B, C |
| Q9D0M3 | CY1_MOUSE | 325 | 35328 | 9.2 | (Q9D0M3) Cytochrome c1, heme protein, mitochondrial precursor (Cytochrome c-1) | 0.00073778 | 0.000165 | 22% | A, B, C |
| Q99LT0 | DPY30_MOUSE | 99 | 11213 | 4.9 | (Q99LT0) Dpy-30-like protein | 0.00072944 | 0.00048714 | 67% | A, B, C |
| Q9D939 | ST1C2_MOUSE | 296 | 34953 | 7.8 | (Q9D939) Sulfotransferase 1C2 (EC 2.8.2.-) | 0.00072202 | 0.00058934 | 82% | A, B, C |
| P61089 | UBE2N_MOUSE | 152 | 17138 | 6.6 | (P61089) Ubiquitin-conjugating enzyme E2 N (EC 6.3.2.19) (Ubiquitin-protein ligase N) (Ubiquitin carrier protein N) (Ubc13) (Bendless-like ubiquitin-conjugating enzyme) | 0.00072105 | 7.3347E-05 | 10% | A, B, C |
| P99028 | UCRH_MOUSE | 89 | 10435 | 4.9 | (P99028) Ubiquinol-cytochrome c reductase complex 11 kDa protein, mitochondrial precursor (EC 1.10.2.2) (Mitochondrial hinge protein) (Cytochrome C1, nonheme 11 kDa protein) (Complex III subunit VIII) | 0.0007164 | 0.00073912 | 103% | A, B, C |
| Q9WUR9 | KAD4_MOUSE | 223 | 25062 | 7.5 | (Q9WUR9) Adenylate kinase isoenzyme 4, mitochondrial (EC 2.7.4.3) (Adenylate kinase 3-like 1) (ATP-AMP transphosphorylase) | 0.00071527 | 0.00038809 | 54% | A, B, C |
| P62900 | RL31_MOUSE | 125 | 14463 | 10.5 | (P62900) 60S ribosomal protein L31 | 0.00071175 | 0.00047495 | 67% | A, B, C |
| P62492 | RB11A_MOUSE | 215 | 24262 | 6.5 | (P62492) Ras-related protein Rab-11A (Rab-11) | 0.00070902 | 0.00040518 | 57% | A, B, C |
| Q8VEK0 | CC50A_MOUSE | 364 | 41061 | 8.4 | (Q8VEK0) Cell cycle control protein 50A (Transmembrane protein 30A) | 0.00070532 | 0.00065891 | 93% | A, B, C |
| O54983 | CRYM_MOUSE | 313 | 33523 | 5.7 | (O54983) Mu-crystallin homolog | 0.00069925 | 0.00062388 | 89% | A, B, C |
| Q64105 | SPRE_MOUSE | 261 | 27883 | 5.7 | (Q64105) Sepiapterin reductase (EC 1.1.1.153) (SPR) | 0.00069862 | 0.00018397 | 26% | A, B, C |
| Q9WUR2 | PECI_MOUSE | 358 | 39479 | 8 | (Q9WUR2) Peroxisomal 3,2-trans-enoyl-CoA isomerase (EC 5.3.3.8) (Dodecenoyl-CoA isomerase) (Delta(3),delta(2)-enoyl-CoA isomerase) (D3,D2-enoyl-CoA isomerase) | 0.00069762 | 0.0002228 | 32% | A, B, C |
| Q99LC3 | NDUAA_MOUSE | 355 | 40603 | 7.8 | (Q99LC3) NADH dehydrogenase [ubiquinone] 1 alpha subcomplex subunit 10, mitochondrial precursor (EC 1.6.5.3) (EC 1.6.99.3) (NADH-ubiquinone oxidoreductase 42 kDa subunit) (Complex I-42KD) (CI-42KD) | 0.00069414 | 0.0002261 | 33% | A, B, C |
| Q9Z2I0 | LETM1_MOUSE | 738 | 82989 | 6.5 | (Q9Z2I0) Leucine zipper-EF-hand-containing transmembrane protein 1, mitochondrial precursor | 0.00068655 | 8.7035E-05 | 13% | A, B, C |
| Q76MZ3 | 2AAA_MOUSE | 588 | 65192 | 5.1 | (Q76MZ3) Serine/threonine-protein phosphatase 2A 65 kDa regulatory subunit A alpha isoform (PP2A, subunit A, PR65-alpha isoform) (PP2A, subunit A, R1-alpha isoform) | 0.0006726 | 0.00085643 | 127% | A, B, C |
| P42669 | PURA_MOUSE | 321 | 34884 | 6.4 | (P42669) Transcriptional activator protein Pur-alpha (Purine-rich single-stranded DNA-binding protein alpha) | 0.00067185 | 0.0002063 | 31% | A, B, C |
| Q9CXZ1 | NUYM_MOUSE | 175 | 19785 | 10 | (Q9CXZ1) NADH-ubiquinone oxidoreductase 18 kDa subunit, mitochondrial precursor (EC 1.6.5.3) (EC 1.6.99.3) (Complex I-18 kDa) (CI-18 kDa) (Complex I-AQDQ) (CI-AQDQ) | 0.00066206 | 0.00037077 | 56% | A, B, C |

| | | | | | | | | | |
|---|---|---|---|---|---|---|---|---|---|
| Q61696 | HS70A_MOUSE | 641 | 70079 | 5.7 | (Q61696) Heat shock 70 kDa protein 1A (Heat shock 70 kDa protein 3) (HSP70.3) (Hsp68) | 0.00066063 | 0.0002497 | 38% | A, B, C |
| Q9Z204 | HNRPC_MOUSE | 313 | 34385 | 5 | (Q9Z204) Heterogeneous nuclear ribonucleoproteins C1/C2 (hnRNP C1 / hnRNP C2) | 0.00065939 | 0.00030596 | 46% | A, B, C |
| Q8BJ64 | CHDH_MOUSE | 596 | 66415 | 8.5 | (Q8BJ64) Choline dehydrogenase, mitochondrial precursor (EC 1.1.99.1) (CHD) (CDH) | 0.00065934 | 0.00033055 | 50% | A, B, C |
| P09405 | NUCL_MOUSE | 706 | 76592 | 4.8 | (P09405) Nucleolin (Protein C23) | 0.00065641 | 0.00045455 | 69% | A, B, C |
| Q9DCJ5 | NDUA8_MOUSE | 171 | 19861 | 8.5 | (Q9DCJ5) NADH dehydrogenase [ubiquinone] 1 alpha subcomplex subunit 8 (EC 1.6.5.3) (EC 1.6.99.3) (NADH-ubiquinone oxidoreductase 19 kDa subunit) (Complex I-19KD) (CI-19KD) (Complex I-PGIV) (CI-PGIV) | 0.00065398 | 0.00042157 | 64% | A, B, C |
| Q99MZ7 | PECR_MOUSE | 303 | 32410 | 8.3 | (Q99MZ7) Peroxisomal trans-2-enoyl-CoA reductase (EC 1.3.1.38) | 0.00065204 | 0.00016335 | 25% | A, B, C |
| Q8VDD5 | MYH9_MOUSE | 1959 | 226224 | 5.7 | (Q8VDD5) Myosin-9 (Myosin heavy chain, nonmuscle IIa) (Nonmuscle myosin heavy chain IIa) (NMMHC II-a) (NMMHC-IIA) (Cellular myosin heavy chain, type A) (Nonmuscle myosin heavy chain-A) (NMMHC-A) | 0.00064588 | 9.4216E-05 | 15% | A, B, C |
| O54962 | BAF_MOUSE | 89 | 10103 | 6.1 | (O54962) Barrier-to-autointegration factor (Breakpoint cluster region protein 1) (LAP2-binding protein 1) | 0.00064097 | 0.00017965 | 28% | A, B, C |
| Q9D1G1 | RAB1B_MOUSE | 201 | 22187 | 5.7 | (Q9D1G1) Ras-related protein Rab-1B | 0.00063661 | 0.00034102 | 54% | A, B, C |
| O70251 | EF1B_MOUSE | 224 | 24562 | 4.7 | (O70251) Elongation factor 1-beta (EF-1-beta) | 0.000634 | 3.46E-05 | 5% | A, B, C |
| Q922D8 | C1TC_MOUSE | 934 | 101124 | 7.1 | (Q922D8) C-1-tetrahydrofolate synthase, cytoplasmic (C1-THF synthase) [Includes: Methylenetetrahydrofolate dehydrogenase (EC 1.5.1.5); Methenyltetrahydrofolate cyclohydrolase (EC 3.5.4.9); Formyltetrahydrofolate synthetase (EC 6.3.4.3)] | 0.000633 | 0.00017923 | 28% | A, B, C |
| P61922 | GABT_MOUSE | 500 | 56452 | 8.1 | (P61922) 4-aminobutyrate aminotransferase, mitochondrial precursor (EC 2.6.1.19) ((S)-3-amino-2-methylpropionate transaminase) (EC 2.6.1.22) (Gamma-amino-N-butyrate transaminase) (GABA transaminase) (GABA aminotransferase) (GABA-AT) (GABA-T) ( | 0.00063245 | 0.00010316 | 16% | A, B, C |
| Q62433 | NDRG1_MOUSE | 394 | 43009 | 6.1 | (Q62433) Protein NDRG1 (N-myc downstream-regulated gene 1 protein) (Protein Ndr1) | 0.00063018 | 0.0002322 | 37% | A, B, C |
| Q99NB1 | ACS2L_MOUSE | 682 | 74623 | 7 | (Q99NB1) Acetyl-coenzyme A synthetase 2-like, mitochondrial precursor (EC 6.2.1.1) (Acetate--CoA ligase 2) (Acetyl-CoA synthetase 2) (AceCS2) (Acyl-CoA synthetase short-chain family member 1) | 0.00062809 | 0.00028725 | 46% | A, B, C |
| P28474 | ADHX_MOUSE | 373 | 39502 | 7.5 | (P28474) Alcohol dehydrogenase class 3 (EC 1.1.1.1) (Alcohol dehydrogenase class III) (Alcohol dehydrogenase 2) (S-(hydroxymethyl)glutathione dehydrogenase) (EC 1.1.1.284) (Glutathione-dependent formaldehyde dehydrogenase) (FDH) (FALDH) (Alcoh | 0.00062786 | 0.00034561 | 55% | A, B, C |
| Q78JT3 | 3HAO_MOUSE | 286 | 32804 | 6.5 | (Q78JT3) 3-hydroxyanthranilate 3,4-dioxygenase (EC 1.13.11.6) (3-HAO) (3-hydroxyanthranilic acid dioxygenase) (3-hydroxyanthranilate oxygenase) | 0.00062772 | 0.00024381 | 39% | A, B, C |
| Q99JY0 | ECHB_MOUSE | 475 | 51386 | 9.4 | (Q99JY0) Trifunctional enzyme subunit beta, mitochondrial precursor (TP-beta) [Includes: 3-ketoacyl-CoA thiolase (EC 2.3.1.16) (Acetyl-CoA acyltransferase) (Beta-ketothiolase)] | 0.00062519 | 0.00013651 | 22% | A, B, C |
| Q8VEM8 | MPCP_MOUSE | 357 | 39632 | 9.3 | (Q8VEM8) Phosphate carrier protein, mitochondrial precursor (PTP) (Solute carrier family 25 member 3) | 0.00062019 | 0.00030866 | 50% | A, B, C |

| | | | | | | | | | |
|---|---|---|---|---|---|---|---|---|---|
| P56391 | CX6B1_MOUSE | 85 | 9940 | 8.7 | (P56391) Cytochrome c oxidase subunit VIb isoform 1 (EC 1.9.3.1) (COX VIb-1) | 0.00061672 | 4.343E-05 | 7% | A, B, C |
| P18242 | CATD_MOUSE | 410 | 44954 | 7.1 | (P18242) Cathepsin D precursor (EC 3.4.23.5) | 0.00061636 | 0.00045084 | 73% | A, B, C |
| Q8JZV9 | DHRS6_MOUSE | 245 | 26753 | 8 | (Q8JZV9) Dehydrogenase/reductase SDR family member 6 precursor (EC 1.1.-.-) | 0.00061417 | 0.00058365 | 95% | A, B, C |
| Q91Z53 | GRHPR_MOUSE | 328 | 35329 | 7.6 | (Q91Z53) Glyoxylate reductase/hydroxypyruvate reductase (EC 1.1.1.79) | 0.00061157 | 0.00024363 | 40% | A, B, C |
| P15532 | NDKA_MOUSE | 152 | 17208 | 7.4 | (P15532) Nucleoside diphosphate kinase A (EC 2.7.4.6) (NDK A) (NDP kinase A) (Tumor metastatic process-associated protein) (Metastasis inhibition factor NM23) (NDPK-A) (nm23-M1) | 0.00060748 | 0.00015677 | 26% | A, B, C |
| P17225 | PTBP1_MOUSE | 527 | 56478 | 8.3 | (P17225) Polypyrimidine tract-binding protein 1 (PTB) (Heterogeneous nuclear ribonucleoprotein I) (hnRNP I) | 0.00060646 | 7.4044E-05 | 12% | A, B, C |
| Q9Z1Q5 | CLIC1_MOUSE | 240 | 26882 | 5.2 | (Q9Z1Q5) Chloride intracellular channel protein 1 (Nuclear chloride ion channel 27) (NCC27) | 0.00060306 | 0.00022014 | 37% | A, B, C |
| Q9WVM8 | AADAT_MOUSE | 425 | 47598 | 8.2 | (Q9WVM8) Kynurenine/alpha-aminoadipate aminotransferase mitochondrial precursor (KAT/AadAT) (Kynurenine--oxoglutarate transaminase II) (EC 2.6.1.7) (Kynurenine aminotransferase II) (Kynurenine--oxoglutarate aminotransferase II) (2-aminoadipat | 0.00060266 | 3.3966E-05 | 6% | A, B, C |
| Q62393 | TPD52_MOUSE | 185 | 20059 | 4.9 | (Q62393) Tumor protein D52 (mD52) | 0.00060014 | 0.00012315 | 21% | A, B, C |
| P16332 | MUTA_MOUSE | 748 | 82965 | 7.1 | (P16332) Methylmalonyl-CoA mutase, mitochondrial precursor (EC 5.4.99.2) (MCM) (Methylmalonyl-CoA isomerase) | 0.00059662 | 0.00023227 | 39% | A, B, C |
| Q9CRB9 | CHCH3_MOUSE | 227 | 26335 | 8.4 | (Q9CRB9) Coiled-coil-helix-coiled-coil-helix domain-containing protein 3 | 0.00059486 | 0.00022956 | 39% | A, B, C |
| Q61937 | NPM_MOUSE | 292 | 32560 | 4.8 | (Q61937) Nucleophosmin (NPM) (Nucleolar phosphoprotein B23) (Numatrin) (Nucleolar protein NO38) | 0.00059389 | 0.00033905 | 57% | A, B, C |
| P97429 | ANXA4_MOUSE | 318 | 35859 | 5.6 | (P97429) Annexin A4 (Annexin IV) | 0.00059341 | 0.00029076 | 49% | A, B, C |
| P13745 | GSTA1_MOUSE | 222 | 25477 | 9 | (P13745) Glutathione S-transferase Ya chain (EC 2.5.1.18) (GST class-alpha) (Ya1) | 0.00059182 | 0.00053078 | 90% | A, B, C |
| P62754 | RS6_MOUSE | 249 | 28681 | 10.8 | (P62754) 40S ribosomal protein S6 (Phosphoprotein NP33) | 0.00059136 | 0.00054634 | 92% | A, B, C |
| P10852 | 4F2_MOUSE | 526 | 58337 | 5.9 | (P10852) 4F2 cell-surface antigen heavy chain (4F2hc) | 0.00059044 | 0.00015392 | 26% | A, B, C |
| Q922R8 | PDIA6_MOUSE | 440 | 48100 | 5.1 | (Q922R8) Protein disulfide-isomerase A6 precursor (EC 5.3.4.1) (Thioredoxin domain-containing protein 7) | 0.00058806 | 0.00014498 | 25% | A, B, C |
| Q9WVL0 | MAAI_MOUSE | 216 | 24275 | 7.9 | (Q9WVL0) Maleylacetoacetate isomerase (EC 5.2.1.2) (MAAI) (Glutathione S-transferase zeta 1) (EC 2.5.1.18) (GSTZ1-1) | 0.00058684 | 0.00021215 | 36% | A, B, C |
| O35459 | ECH1_MOUSE | 327 | 36118 | 7.7 | (O35459) Delta3,5-delta2,4-dienoyl-CoA isomerase, mitochondrial precursor (EC 5.3.3.-) | 0.0005843 | 0.0003504 | 60% | A, B, C |
| Q9CQV8 | 1433B_MOUSE | 245 | 27955 | 4.8 | (Q9CQV8) 14-3-3 protein beta/alpha (Protein kinase C inhibitor protein 1) (KCIP-1) | 0.00058075 | 0.00034283 | 59% | A, B, C |
| P57746 | VATD_MOUSE | 247 | 28369 | 9.4 | (P57746) Vacuolar ATP synthase subunit D (EC 3.6.3.14) (V-ATPase D subunit) (Vacuolar proton pump D subunit) (V-ATPase 28 kDa accessory protein) | 0.00057681 | 0.00025209 | 44% | A, B, C |
| P26039 | TLN1_MOUSE | 2541 | 269831 | 6.1 | (P26039) Talin-1 | 0.00057563 | 4.5116E-05 | 8% | A, B, C |
| P20152 | VIME_MOUSE | 465 | 53557 | 5.1 | (P20152) Vimentin | 0.00057454 | 0.00014358 | 25% | A, B, C |
| Q8R0Y6 | FTHFD_MOUSE | 902 | 98709 | 5.9 | (Q8R0Y6) 10-formyltetrahydrofolate dehydrogenase (EC 1.5.1.6) (10-FTHFDH) (Aldehyde dehydrogenase 1 family member L1) | 0.00057338 | 9.7834E-05 | 17% | A, B, C |

| | | | | | | | | | |
|---|---|---|---|---|---|---|---|---|---|
| Q9D6J5 | NDUB8_MOUSE | 186 | 21876 | 6.6 | (Q9D6J5) NADH dehydrogenase [ubiquinone] 1 beta subcomplex subunit 8, mitochondrial precursor (EC 1.6.5.3) (EC 1.6.99.3) (NADH-ubiquinone oxidoreductase ASHI subunit) (Complex I-ASHI) (CI-ASHI) | 0.00057224 | 0.00034315 | 60% | A, B, C |
| O09167 | RL21_MOUSE | 159 | 18431 | 10.5 | (O09167) 60S ribosomal protein L21 | 0.00057047 | 4.024E-05 | 7% | A, B, C |
| Q99JB2 | STML2_MOUSE | 353 | 38385 | 8.9 | (Q99JB2) Stomatin-like protein 2 (SLP-2) | 0.00056716 | 0.00016096 | 28% | A, B, C |
| Q91WD5 | NUCM_MOUSE | 463 | 52626 | 7 | (Q91WD5) NADH-ubiquinone oxidoreductase 49 kDa subunit, mitochondrial precursor (EC 1.6.5.3) (EC 1.6.99.3) (Complex I-49KD) (CI-49KD) | 0.00056271 | 0.00054206 | 96% | A, B, C |
| P57759 | ERP29_MOUSE | 262 | 28823 | 6.1 | (P57759) Endoplasmic reticulum protein ERp29 precursor | 0.00055644 | 0.00015984 | 29% | A, B, C |
| P68254 | 1433T_MOUSE | 245 | 27778 | 4.8 | (P68254) 14-3-3 protein theta (14-3-3 protein tau) | 0.00055621 | 0.00028464 | 51% | A, B, C |
| Q9QXT0 | MSAP_MOUSE | 182 | 20767 | 5.1 | (Q9QXT0) MIR-interacting saposin-like protein precursor (Transmembrane protein 4) (Putative secreted protein ZSIG9) | 0.00055367 | 0.00015918 | 29% | A, B, C |
| P35564 | CALX_MOUSE | 591 | 67278 | 4.6 | (P35564) Calnexin precursor | 0.00055292 | 0.00060049 | 109% | A, B, C |
| Q9CPU0 | LGUL_MOUSE | 183 | 20678 | 5.5 | (Q9CPU0) Lactoylglutathione lyase (EC 4.4.1.5) (Methylglyoxalase) (Aldoketomutase) (Glyoxalase I) (Glx I) (Ketone-aldehyde mutase) (S-D-lactoylglutathione methylglyoxal lyase) | 0.00055272 | 0.00024252 | 44% | A, B, C |
| Q9CQH0 | PDZ1I_MOUSE | 114 | 12298 | 4.8 | (Q9CQH0) PDZK1-interacting protein 1 (17 kDa membrane-associated protein) | 0.00055065 | 0.00032187 | 58% | A, B, C |
| Q62261 | SPTB2_MOUSE | 2363 | 274221 | 5.6 | (Q62261) Spectrin beta chain, brain 1 (Spectrin, non-erythroid beta chain 1) (Beta-II spectrin) (Fodrin beta chain) | 0.00055057 | 0.00010665 | 19% | A, B, C |
| Q99MR8 | MCCA_MOUSE | 717 | 79344 | 7.8 | (Q99MR8) Methylcrotonoyl-CoA carboxylase subunit alpha, mitochondrial precursor (EC 6.4.1.4) (3-methylcrotonyl-CoA carboxylase 1) (MCCase alpha subunit) (3-methylcrotonyl-CoA:carbon dioxide ligase subunit alpha) (3-methylcrotonyl-CoA carboxyla | 0.00054947 | 5.7529E-05 | 10% | A, B, C |
| Q91YI0 | ARLY_MOUSE | 464 | 51739 | 7 | (Q91YI0) Argininosuccinate lyase (EC 4.3.2.1) (Arginosuccinase) (ASAL) | 0.00054591 | 0.00021 | 38% | A, B, C |
| Q9NYQ2 | HAOX2_MOUSE | 353 | 38700 | 7.6 | (Q9NYQ2) Hydroxyacid oxidase 2 (EC 1.1.3.15) (HAOX2) ((S)-2-hydroxy-acid oxidase, peroxisomal) (Medium chain alpha-hydroxy acid oxidase) (Medium-chain L-2-hydroxy acid oxidase) | 0.00054232 | 0.00030549 | 56% | A, B, C |
| Q99KR7 | PPIF_MOUSE | 206 | 21737 | 9.2 | (Q99KR7) Peptidyl-prolyl cis-trans isomerase, mitochondrial precursor (EC 5.2.1.8) (PPIase) (Rotamase) (Cyclophilin F) | 0.00053364 | 0.00015731 | 29% | A, B, C |
| Q61598 | GDIB_MOUSE | 445 | 50537 | 6.2 | (Q61598) Rab GDP dissociation inhibitor beta (Rab GDI beta) (Guanosine diphosphate dissociation inhibitor 2) (GDI-2) (GDI-3) | 0.00053354 | 0.00026884 | 50% | A, B, C |
| O08547 | SC22B_MOUSE | 214 | 24609 | 8.5 | (O08547) Vesicle-trafficking protein SEC22b (SEC22 vesicle-trafficking protein-like 1) | 0.00053343 | 0.00020026 | 38% | A, B, C |
| Q07417 | ACADS_MOUSE | 412 | 44947 | 8.8 | (Q07417) Short-chain specific acyl-CoA dehydrogenase, mitochondrial precursor (EC 1.3.99.2) (SCAD) (Butyryl-CoA dehydrogenase) | 0.00053228 | 0.000187 | 35% | A, B, C |
| P45878 | FKBP2_MOUSE | 140 | 15344 | 8.9 | (P45878) FK506-binding protein 2 precursor (EC 5.2.1.8) (Peptidyl-prolyl cis-trans isomerase) (PPIase) (Rotamase) (13 kDa FKBP) (FKBP-13) | 0.00052856 | 0.00016629 | 31% | A, B, C |
| P45591 | COF2_MOUSE | 166 | 18710 | 7.9 | (P45591) Cofilin-2 (Cofilin, muscle isoform) | 0.00052772 | 0.00067607 | 128% | A, B, C |
| Q9CZ44 | NSF1C_MOUSE | 370 | 40710 | 5.1 | (Q9CZ44) NSFL1 cofactor p47 (p97 cofactor p47) | 0.00052464 | 0.00019158 | 37% | A, B, C |
| P29341 | PABP1_MOUSE | 636 | 70643 | 9.5 | (P29341) Polyadenylate-binding protein 1 (Poly(A)-binding protein 1) (PABP 1) | 0.00051655 | 0.00022466 | 43% | A, B, C |

| | | | | | | | | |
|---|---|---|---|---|---|---|---|---|
| Q9CQ60 | 6PGL_MOUSE | 257 | 27254 | 5.8 | (Q9CQ60) 6-phosphogluconolactonase (EC 3.1.1.31) (6PGL) | 0.00050637 | 0.00033257 | 66% | A, B, C |
| Q99K48 | NONO_MOUSE | 473 | 54541 | 8.9 | (Q99K48) Non-POU domain-containing octamer-binding protein (NonO protein) | 0.00050396 | 0.0002177 | 43% | A, B, C |
| Q9QZQ8 | H2AY_MOUSE | 371 | 39604 | 9.8 | (Q9QZQ8) Core histone macro-H2A.1 (Histone macroH2A1) (mH2A1) (H2A.y) (H2A/y) | 0.00049811 | 0.00012508 | 25% | A, B, C |
| Q9DCM2 | GSTK1_MOUSE | 225 | 25573 | 8.9 | (Q9DCM2) Glutathione S-transferase kappa 1 (EC 2.5.1.18) (GST 13-13) (Glutathione S-transferase subunit 13) (GST class-kappa) (GSTK1-1) (mGSTK1) | 0.00049731 | 0.0002398 | 48% | A, B, C |
| P62869 | ELOB_MOUSE | 118 | 13170 | 5 | (P62869) Transcription elongation factor B polypeptide 2 (RNA polymerase II transcription factor SIII subunit B) (SIII p18) (Elongin B) (EloB) (Elongin 18 kDa subunit) | 0.00049212 | 0.00044796 | 91% | A, B, C |
| P37804 | TAGL_MOUSE | 200 | 22445 | 8.8 | (P37804) Transgelin (Smooth muscle protein 22-alpha) (SM22-alpha) (Actin-associated protein p27) | 0.00049108 | 0.0002772 | 56% | A, B, C |
| P40936 | INMT_MOUSE | 264 | 29460 | 6.4 | (P40936) Indolethylamine N-methyltransferase (EC 2.1.1.49) (Aromatic alkylamine N-methyltransferase) (Indolamine N-methyltransferase) (Arylamine N-methyltransferase) (Amine N-methyltransferase) | 0.00048894 | 0.00022924 | 47% | A, B, C |
| P49443 | PP2CA_MOUSE | 382 | 42433 | 5.4 | (P49443) Protein phosphatase 2C isoform alpha (EC 3.1.3.16) (PP2C-alpha) (IA) (Protein phosphatase 1A) | 0.00048672 | 0.00037862 | 78% | A, B, C |
| P08003 | PDIA4_MOUSE | 638 | 71973 | 5.3 | (P08003) Protein disulfide-isomerase A4 precursor (EC 5.3.4.1) (Protein ERp-72) (ERp72) | 0.00048636 | 2.8442E-06 | 1% | A, B, C |
| Q99KJ8 | DCTN2_MOUSE | 401 | 43986 | 5.3 | (Q99KJ8) Dynactin subunit 2 (Dynactin complex 50 kDa subunit) (50 kDa dynein-associated polypeptide) (p50 dynamitin) (DCTN-50) (Growth cone membrane protein 23-48K) (GMP23-48K) | 0.00048423 | 5.3028E-05 | 11% | A, B, C |
| Q6IRU2 | TPM4_MOUSE | 247 | 28337 | 4.7 | (Q6IRU2) Tropomyosin alpha-4 chain (Tropomyosin-4) | 0.00048169 | 0.00016274 | 34% | A, B, C |
| P62270 | RS18_MOUSE | 152 | 17719 | 11 | (P62270) 40S ribosomal protein S18 (Ke-3) (Ke3) | 0.00047957 | 0.00023953 | 50% | A, B, C |
| P00329 | ADH1_MOUSE | 374 | 39640 | 8.1 | (P00329) Alcohol dehydrogenase 1 (EC 1.1.1.1) (Alcohol dehydrogenase A subunit) (ADH-A2) | 0.0004795 | 0.00031204 | 65% | A, B, C |
| P31428 | DPEP1_MOUSE | 410 | 45682 | 6.4 | (P31428) Dipeptidase 1 precursor (EC 3.4.13.19) (Microsomal dipeptidase) (Renal dipeptidase) (Membrane-bound dipeptidase 1) (MBD-1) | 0.00047741 | 0.00023191 | 49% | A, B, C |
| Q99PT1 | GDIR_MOUSE | 203 | 23276 | 5.2 | (Q99PT1) Rho GDP-dissociation inhibitor 1 (Rho GDI 1) (Rho-GDI alpha) (GDI-1) | 0.00047715 | 0.00011881 | 25% | A, B, C |
| P12970 | RL7A_MOUSE | 265 | 29845 | 10.6 | (P12970) 60S ribosomal protein L7a (Surfeit locus protein 3) | 0.00047573 | 0.00032101 | 67% | A, B, C |
| P97314 | CSRP2_MOUSE | 192 | 20795 | 8.6 | (P97314) Cysteine and glycine-rich protein 2 (Cysteine-rich protein 2) (CRP2) (Double LIM protein 1) (DLP-1) | 0.00047414 | 0.00015969 | 34% | A, B, C |
| P49312 | ROA1_MOUSE | 319 | 34065 | 9.2 | (P49312) Heterogeneous nuclear ribonucleoprotein A1 (Helix-destabilizing protein) (Single-strand-binding protein) (hnRNP core protein A1) (HDP-1) (Topoisomerase-inhibitor suppressed) | 0.00047345 | 0.0003362 | 71% | A, B, C |
| P68368 | TBA4_MOUSE | 448 | 49924 | 5.1 | (P68368) Tubulin alpha-4 chain (Alpha-tubulin 4) (Alpha-tubulin isotype M-alpha-4) | 0.00047294 | 0.00026902 | 57% | A, B, C |
| P00920 | CAH2_MOUSE | 259 | 28960 | 7 | (P00920) Carbonic anhydrase 2 (EC 4.2.1.1) (Carbonic anhydrase II) (Carbonate dehydratase II) (CA-II) | 0.00047142 | 0.0002237 | 47% | A, B, C |
| Q99KB8 | GLO2_MOUSE | 260 | 28901 | 7 | (Q99KB8) Hydroxyacylglutathione hydrolase (EC 3.1.2.6) (Glyoxalase II) (Glx II) | 0.00046878 | 0.00010274 | 22% | A, B, C |
| P26883 | FKB1A_MOUSE | 107 | 11791 | 8.2 | (P26883) FK506-binding protein 1A (EC 5.2.1.8) (Peptidyl-prolyl cis-trans isomerase) (PPIase) (Rotamase) (12 kDa FKBP) (FKBP-12) (Immunophilin FKBP12) | 0.00046768 | 0.00015551 | 33% | A, B, C |

| | | | | | | | | | |
|---|---|---|---|---|---|---|---|---|---|
| Q60931 | VDAC3_MOUSE | 283 | 30753 | 8.8 | (Q60931) Voltage-dependent anion-selective channel protein 3 (VDAC-3) (mVDAC3) (Outer mitochondrial membrane protein porin 3) | 0.00046488 | 0.00011841 | 25% | A, B, C |
| P07901 | HS90A_MOUSE | 732 | 84657 | 5 | (P07901) Heat shock protein HSP 90-alpha (HSP 86) (Tumor-specific transplantation 86 kDa antigen) (TSTA) | 0.00046418 | 5.6594E-05 | 12% | A, B, C |
| Q8R164 | BPHL_MOUSE | 291 | 32851 | 8.9 | (Q8R164) Valacyclovir hydrolase precursor (EC 3.1.-.-) (VACVase) (Biphenyl hydrolase-like protein) | 0.00046375 | 0.00010608 | 23% | A, B, C |
| Q4FZG7 | TI8AB_MOUSE | 97 | 11283 | 6.5 | (Q4FZG7) Putative mitochondrial import inner membrane translocase subunit Tim8 A-B | 0.00046063 | 7.166E-05 | 16% | A, B, C |
| O88343 | S4A4_MOUSE | 1079 | 121483 | 6.8 | (O88343) Electrogenic sodium bicarbonate cotransporter 1 (Sodium bicarbonate cotransporter) (Na(+)/HCO3(-) cotransporter) (Solute carrier family 4 member 4) | 0.00046034 | 0.00026713 | 58% | A, B, C |
| Q80X90 | FLNB_MOUSE | 2602 | 277750 | 5.7 | (Q80X90) Filamin-B (FLN-B) (Beta-filamin) (Actin-binding-like protein) (ABP-280-like protein) | 0.00045914 | 3.0481E-05 | 7% | A, B, C |
| Q78IK4 | CX033_MOUSE | 265 | 29261 | 9.3 | (Q78IK4) Protein CXorf33 homolog precursor | 0.00045904 | 0.0001437 | 31% | A, B, C |
| Q9DC50 | OCTC_MOUSE | 612 | 70264 | 6.7 | (Q9DC50) Peroxisomal carnitine O-octanoyltransferase (EC 2.3.1.137) (COT) | 0.00045887 | 0.00010014 | 22% | A, B, C |
| Q9Z0P4 | PALM_MOUSE | 383 | 41614 | 4.8 | (Q9Z0P4) Paralemmin | 0.00045679 | 1.7587E-05 | 4% | A, B, C |
| P60335 | PCBP1_MOUSE | 356 | 37498 | 7.1 | (P60335) Poly(rC)-binding protein 1 (Alpha-CP1) (hnRNP-E1) | 0.0004558 | 0.00026345 | 58% | A, B, C |
| Q60648 | SAP3_MOUSE | 193 | 20824 | 5.9 | (Q60648) Ganglioside GM2 activator precursor (GM2-AP) (Cerebroside sulfate activator protein) (Shingolipid activator protein 3) (SAP-3) | 0.00045482 | 0.00026149 | 57% | A, B, C |
| P07758 | A1AT1_MOUSE | 413 | 46003 | 5.7 | (P07758) Alpha-1-antitrypsin 1-1 precursor (Serine protease inhibitor 1-1) (Alpha-1 protease inhibitor 1) (Alpha-1-antiproteinase) (AAT) | 0.00045307 | 0.00023174 | 51% | A, B, C |
| Q7M6Y3 | PICA_MOUSE | 660 | 71543 | 7.9 | (Q7M6Y3) Phosphatidylinositol-binding clathrin assembly protein (Clathrin assembly lymphoid myeloid leukemia) (CALM) | 0.0004522 | 0.00010788 | 24% | A, B, C |
| P47740 | AL3A2_MOUSE | 484 | 53943 | 8.4 | (P47740) Fatty aldehyde dehydrogenase (EC 1.2.1.3) (Aldehyde dehydrogenase, microsomal) (Aldehyde dehydrogenase family 3 member A2) (Aldehyde dehydrogenase 10) | 0.00045063 | 6.2381E-05 | 14% | A, B, C |
| P62960 | YBOX1_MOUSE | 321 | 35599 | 9.9 | (P62960) Nuclease sensitive element-binding protein 1 (Y-box-binding protein 1) (Y-box transcription factor) (YB-1) (CCAAT-binding transcription factor I subunit A) (CBF-A) (Enhancer factor I subunit A) (EFI-A) (DNA-binding protein B) (DBPB) | 0.00044933 | 0.00018533 | 41% | A, B, C |
| Q9R0Y5 | KAD1_MOUSE | 194 | 21540 | 5.8 | (Q9R0Y5) Adenylate kinase isoenzyme 1 (EC 2.7.4.3) (ATP-AMP transphosphorylase) (AK1) (Myokinase) | 0.00044172 | 0.00018385 | 42% | A, B, C |
| Q9WUM4 | COR1C_MOUSE | 474 | 53121 | 7.1 | (Q9WUM4) Coronin-1C (Coronin-3) | 0.00044066 | 0.00045695 | 104% | A, B, C |
| P61961 | UFM1_MOUSE | 85 | 9118 | 9.3 | (P61961) Ubiquitin-fold modifier 1 precursor | 0.00043851 | 0.00018234 | 42% | A, B, C |
| Q9CR62 | M2OM_MOUSE | 313 | 34024 | 9.9 | (Q9CR62) Mitochondrial 2-oxoglutarate/malate carrier protein (OGCP) (Solute carrier family 25 member 11) | 0.0004378 | 0.00033053 | 75% | A, B, C |
| Q9WVK4 | EHD1_MOUSE | 534 | 60603 | 6.8 | (Q9WVK4) EH-domain-containing protein 1 (mPAST1) | 0.00043364 | 0.00011299 | 26% | A, B, C |
| Q01730 | RSU1_MOUSE | 276 | 31419 | 8.9 | (Q01730) Ras suppressor protein 1 (Rsu-1) (RSP-1) | 0.00043363 | 0.00019466 | 45% | A, B, C |
| P50247 | SAHH_MOUSE | 431 | 47557 | 6.5 | (P50247) Adenosylhomocysteinase (EC 3.3.1.1) (S-adenosyl-L-homocysteine hydrolase) (AdoHcyase) (Liver copper-binding protein) (CUBP) | 0.00043077 | 0.00035961 | 83% | A, B, C |
| Q923D4 | SF3B5_MOUSE | 86 | 10119 | 6.4 | (Q923D4) Splicing factor 3B subunit 5 (SF3b5) (Pre-mRNA-splicing factor SF3b 10 kDa subunit) | 0.00042935 | 0.00027302 | 64% | A, B, C |

| | | | | | | | | | |
|---|---|---|---|---|---|---|---|---|---|
| P97449 | AMPN_MOUSE | 965 | 109520 | 5.9 | (P97449) Aminopeptidase N (EC 3.4.11.2) (mAPN) (Alanyl aminopeptidase) (Microsomal aminopeptidase) (Aminopeptidase M) (Membrane protein p161) (CD13 antigen) | 0.00042866 | 0.00041461 | 97% | A, B, C |
| Q64467 | G3PT_MOUSE | 440 | 47657 | 7.9 | (Q64467) Glyceraldehyde-3-phosphate dehydrogenase, testis-specific (EC 1.2.1.12) (Spermatogenic cell-specific glyceraldehyde 3-phosphate dehydrogenase 2) (GAPDH-2) | 0.00042509 | 0.00059193 | 139% | A, B, C |
| Q9DCN2 | NCB5R_MOUSE | 300 | 33996 | 8.4 | (Q9DCN2) NADH-cytochrome b5 reductase (EC 1.6.2.2) (B5R) (Diaphorase-1) (Cytochrome b5 reductase 3) [Contains: NADH-cytochrome b5 reductase membrane-bound form; NADH-cytochrome b5 reductase soluble form] | 0.0004248 | 0.0001336 | 31% | A, B, C |
| P15864 | H12_MOUSE | 211 | 21135 | 11 | (P15864) Histone H1.2 (H1 VAR.1) (H1c) | 0.00042474 | 0.00019895 | 47% | A, B, C |
| P05202 | AATM_MOUSE | 430 | 47411 | 9 | (P05202) Aspartate aminotransferase, mitochondrial precursor (EC 2.6.1.1) (Transaminase A) (Glutamate oxaloacetate transaminase 2) | 0.0004237 | 0.00011027 | 26% | A, B, C |
| Q07813 | BAXA_MOUSE | 192 | 21395 | 5 | (Q07813) Apoptosis regulator BAX, membrane isoform alpha | 0.00041985 | 0.00014433 | 34% | A, B, C |
| Q9R112 | SQRD_MOUSE | 450 | 50340 | 9 | (Q9R112) Sulfide:quinone oxidoreductase, mitochondrial precursor (EC 1.-.-.-) | 0.00041555 | 6.7298E-05 | 16% | A, B, C |
| Q9CZY3 | UB2V1_MOUSE | 147 | 16355 | 8 | (Q9CZY3) Ubiquitin-conjugating enzyme E2 variant 1 (UEV-1) (CROC-1) | 0.00041512 | 0.00020374 | 49% | A, B, C |
| P14131 | RS16_MOUSE | 145 | 16314 | 10.2 | (P14131) 40S ribosomal protein S16 | 0.00041469 | 0.00039722 | 96% | A, B, C |
| P08228 | SODC_MOUSE | 153 | 15811 | 6.5 | (P08228) Superoxide dismutase [Cu-Zn] (EC 1.15.1.1) | 0.00041375 | 0.00012463 | 30% | A, B, C |
| Q9CPQ3 | TOM22_MOUSE | 141 | 15406 | 4.3 | (Q9CPQ3) Mitochondrial import receptor subunit TOM22 homolog (Translocase of outer membrane 22 kDa subunit homolog) | 0.00041125 | 0.00018725 | 46% | A, B, C |
| P62774 | MTPN_MOUSE | 117 | 12730 | 5.5 | (P62774) Myotrophin (Protein V-1) (Granule cell differentiation protein) | 0.00040865 | 0.00017902 | 44% | A, B, C |
| Q91ZA3 | PCCA_MOUSE | 724 | 79922 | 7.2 | (Q91ZA3) Propionyl-CoA carboxylase alpha chain, mitochondrial precursor (EC 6.4.1.3) (PCCase subunit alpha) (Propanoyl-CoA:carbon dioxide ligase subunit alpha) | 0.00040678 | 8.7567E-05 | 22% | A, B, C |
| Q9CYR0 | SSB_MOUSE | 152 | 17319 | 9.9 | (Q9CYR0) Single-stranded DNA-binding protein, mitochondrial precursor (Mt-SSB) (MtSSB) | 0.00040501 | 4.0187E-05 | 10% | A, B, C |
| Q9DCT8 | CRIP2_MOUSE | 208 | 22727 | 8.6 | (Q9DCT8) Cysteine-rich protein 2 (CRP2) (Heart LIM protein) | 0.00040315 | 0.00010626 | 26% | A, B, C |
| Q9R0P3 | ESTD_MOUSE | 282 | 31320 | 7.1 | (Q9R0P3) Esterase D (EC 3.1.1.1) (Esterase 10) (Sid 478) | 0.00040283 | 0.00010195 | 25% | A, B, C |
| Q6PDM2 | SFRS1_MOUSE | 247 | 27613 | 10.4 | (Q6PDM2) Splicing factor, arginine/serine-rich 1 | 0.00039696 | 0.00017817 | 45% | A, B, C |
| P84104 | SFRS3_MOUSE | 164 | 19330 | 11.6 | (P84104) Splicing factor, arginine/serine-rich 3 (Pre-mRNA-splicing factor SRP20) (X16 protein) | 0.00039634 | 0.00029622 | 75% | A, B, C |
| Q8VCH0 | THIKB_MOUSE | 424 | 43995 | 8.5 | (Q8VCH0) 3-ketoacyl-CoA thiolase B, peroxisomal precursor (EC 2.3.1.16) (Beta-ketothiolase B) (Acetyl-CoA acyltransferase B) (Peroxisomal 3-oxoacyl-CoA thiolase B) | 0.00039406 | 0.00019608 | 50% | A, B, C |
| P51660 | DHB4_MOUSE | 734 | 79351 | 8.6 | (P51660) Peroxisomal multifunctional enzyme type 2 (MFE-2) (D-bifunctional protein) (DBP) (17-beta-hydroxysteroid dehydrogenase 4) (17-beta-HSD 4) [Includes: D-3-hydroxyacyl-CoA dehydratase (EC 4.2.1.107) (3-alpha,7-alpha,12-alpha-trihydroxy-5 | 0.00039402 | 4.6308E-05 | 12% | A, B, C |
| Q61081 | CDC37_MOUSE | 379 | 44593 | 5.3 | (Q61081) Hsp90 co-chaperone Cdc37 (Hsp90 chaperone protein kinase-targeting subunit) (p50Cdc37) | 0.00039167 | 9.3049E-05 | 24% | A, B, C |
| P62267 | RS23_MOUSE | 142 | 15676 | 10.5 | (P62267) 40S ribosomal protein S23 | 0.00039016 | 0.00019257 | 49% | A, B, C |

| | | | | | | | | | |
|---|---|---|---|---|---|---|---|---|---|
| P97447 | FHL1_MOUSE | 280 | 31889 | 8.4 | (P97447) Four and a half LIM domains protein 1 (FHL-1) (Skeletal muscle LIM-protein 1) (SLIM 1) (SLIM) (KyoT) (RBP-associated molecule 14-1) (RAM14-1) | 0.00038903 | 0.00012411 | 32% | A, B, C |
| P63037 | DNJA1_MOUSE | 397 | 44868 | 7.1 | (P63037) DnaJ homolog subfamily A member 1 (Heat shock 40 kDa protein 4) (DnaJ protein homolog 2) (HSJ-2) | 0.00038786 | 0.00025299 | 65% | A, B, C |
| Q9CXS4 | PRR6_MOUSE | 252 | 27541 | 9.8 | (Q9CXS4) Proline-rich protein 6 | 0.00038782 | 0.00016928 | 44% | A, B, C |
| Q9R0P5 | DEST_MOUSE | 164 | 18390 | 8 | (Q9R0P5) Destrin (Actin-depolymerizing factor) (ADF) (Sid 23) | 0.00038646 | 0.00013971 | 36% | A, B, C |
| Q99MN9 | PCCB_MOUSE | 541 | 58394 | 7.5 | (Q99MN9) Propionyl-CoA carboxylase beta chain, mitochondrial precursor (EC 6.4.1.3) (PCCase subunit beta) (Propanoyl-CoA:carbon dioxide ligase subunit beta) | 0.00038151 | 6.5801E-05 | 17% | A, B, C |
| P55258 | RAB8A_MOUSE | 207 | 23668 | 9.1 | (P55258) Ras-related protein Rab-8A (Oncogene c-mel) | 0.00038114 | 0.00024872 | 65% | A, B, C |
| P70670 | NACAM_MOUSE | 2187 | 220599 | 9.4 | (P70670) Nascent polypeptide-associated complex subunit alpha, muscle-specific form (Alpha-NAC, muscle-specific form) | 0.00038093 | 0.00034634 | 91% | A, B, C |
| Q64010 | CRK_MOUSE | 304 | 33815 | 5.6 | (Q64010) Proto-oncogene C-crk (P38) (Adapter molecule crk) | 0.00037588 | 5.9073E-05 | 16% | A, B, C |
| Q9D964 | GATM_MOUSE | 423 | 48297 | 7.9 | (Q9D964) Glycine amidinotransferase, mitochondrial precursor (EC 2.1.4.1) (L-arginine:glycine amidinotransferase) (Transamidinase) (AT) | 0.00037542 | 0.00017641 | 47% | A, B, C |
| Q9D0F9 | PGM1_MOUSE | 561 | 61386 | 6.8 | (Q9D0F9) Phosphoglucomutase-1 (EC 5.4.2.2) (Glucose phosphomutase 1) (PGM 1) | 0.00037397 | 9.852E-05 | 26% | A, B, C |
| Q9EQP2 | EHD4_MOUSE | 541 | 61481 | 6.8 | (Q9EQP2) EH-domain-containing protein 4 (mPAST2) | 0.00037061 | 0.00010506 | 28% | A, B, C |
| P42125 | D3D2_MOUSE | 289 | 32078 | 8.7 | (P42125) 3,2-trans-enoyl-CoA isomerase, mitochondrial precursor (EC 5.3.3.8) (Dodecenoyl-CoA isomerase) (Delta(3),delta(2)-enoyl-CoA isomerase) (D3,D2-enoyl-CoA isomerase) | 0.00036636 | 0.00018903 | 52% | A, B, C |
| P47955 | RLA1_MOUSE | 114 | 11475 | 4.3 | (P47955) 60S acidic ribosomal protein P1 | 0.00036464 | 1.6961E-05 | 5% | A, B, C |
| P84089 | ERH_MOUSE | 104 | 12259 | 5.9 | (P84089) Enhancer of rudimentary homolog | 0.0003643 | 9.6441E-05 | 26% | A, B, C |
| P05201 | AATC_MOUSE | 412 | 46100 | 7.2 | (P05201) Aspartate aminotransferase, cytoplasmic (EC 2.6.1.1) (Transaminase A) (Glutamate oxaloacetate transaminase 1) | 0.0003617 | 1.0747E-05 | 3% | A, B, C |
| Q9DBL7 | COASY_MOUSE | 563 | 62023 | 7.1 | (Q9DBL7) Bifunctional coenzyme A synthase (CoA synthase) [Includes: Phosphopantetheine adenylyltransferase (EC 2.7.7.3) (Pantetheine-phosphate adenylyltransferase) (PPAT) (Dephospho-CoA pyrophosphorylase); Dephospho-CoA kinase (EC 2.7.1.24) ( | 0.00035941 | 0.00010469 | 29% | A, B, C |
| Q9CQE8 | CN166_MOUSE | 244 | 28152 | 6.9 | (Q9CQE8) Protein C14orf166 homolog | 0.00035756 | 0.00016345 | 46% | A, B, C |
| P29391 | FRIL1_MOUSE | 182 | 20671 | 6 | (P29391) Ferritin light chain 1 (Ferritin L subunit 1) | 0.0003575 | 0.00018807 | 53% | A, B, C |
| Q9D0E1 | HNRPM_MOUSE | 728 | 77517 | 8.6 | (Q9D0E1) Heterogeneous nuclear ribonucleoprotein M (hnRNP M) | 0.00035519 | 0.00013527 | 38% | A, B, C |
| P62317 | SMD2_MOUSE | 118 | 13527 | 9.9 | (P62317) Small nuclear ribonucleoprotein Sm D2 (snRNP core protein D2) (Sm-D2) | 0.00035295 | 0.00018145 | 51% | A, B, C |
| Q8VE22 | RT23_MOUSE | 177 | 20348 | 8.6 | (Q8VE22) Mitochondrial ribosomal protein S23 (S23mt) (MRP-S23) | 0.00035294 | 8.1969E-05 | 23% | A, B, C |
| Q9CQJ8 | NDUB9_MOUSE | 178 | 21853 | 7.8 | (Q9CQJ8) NADH dehydrogenase [ubiquinone] 1 beta subcomplex subunit 9 (EC 1.6.5.3) (EC 1.6.99.3) (NADH-ubiquinone oxidoreductase B22 subunit) (Complex I-B22) (CI-B22) | 0.00035183 | 6.7999E-05 | 19% | A, B, C |

| | | | | | | | | | |
|---|---|---|---|---|---|---|---|---|---|
| Q9WTX5 | SKP1_MOUSE | 162 | 18541 | 4.5 | (Q9WTX5) S-phase kinase-associated protein 1A (Cyclin A/CDK2-associated protein p19) (p19A) (p19skp1) | 0.00035118 | 8.2846E-05 | 24% | A, B, C |
| P46414 | CDN1B_MOUSE | 197 | 22210 | 7 | (P46414) Cyclin-dependent kinase inhibitor 1B (Cyclin-dependent kinase inhibitor p27) (p27Kip1) | 0.00035042 | 0.00031364 | 90% | A, B, C |
| Q60866 | PTER_MOUSE | 349 | 39218 | 6.7 | (Q60866) Phosphotriesterase-related protein (Parathion hydrolase-related protein) | 0.00034994 | 0.00023378 | 67% | A, B, C |
| P49722 | PSA2_MOUSE | 233 | 25794 | 8.3 | (P49722) Proteasome subunit alpha type 2 (EC 3.4.25.1) (Proteasome component C3) (Macropain subunit C3) (Multicatalytic endopeptidase complex subunit C3) | 0.00034991 | 0.00023482 | 67% | A, B, C |
| P80317 | TCPZ_MOUSE | 530 | 57873 | 7.1 | (P80317) T-complex protein 1 subunit zeta (TCP-1-zeta) (CCT-zeta) (CCT-zeta-1) | 0.00034965 | 0.00019059 | 55% | A, B, C |
| Q91XV3 | BASP_MOUSE | 225 | 21955 | 4.5 | (Q91XV3) Brain acid soluble protein 1 (BASP1 protein) (Neuronal axonal membrane protein NAP-22) (22 kDa neuronal tissue-enriched acidic protein) | 0.00034789 | 0.00011436 | 33% | A, B, C |
| P27659 | RL3_MOUSE | 402 | 45993 | 10.2 | (P27659) 60S ribosomal protein L3 (J1 protein) | 0.00034721 | 0.00023171 | 67% | A, B, C |
| Q61941 | NNTM_MOUSE | 1086 | 113838 | 7.6 | (Q61941) NAD(P) transhydrogenase, mitochondrial precursor (EC 1.6.1.2) (Pyridine nucleotide transhydrogenase) (Nicotinamide nucleotide transhydrogenase) | 0.00034592 | 0.00020959 | 61% | A, B, C |
| P12367 | KAP2_MOUSE | 400 | 45258 | 4.9 | (P12367) cAMP-dependent protein kinase type II-alpha regulatory subunit | 0.00034095 | 0.0003469 | 102% | A, B, C |
| Q3TNA1 | XYLB_MOUSE | 551 | 59544 | 6.9 | (Q3TNA1) Xylulose kinase (EC 2.7.1.17) (Xylulokinase) | 0.00034026 | 0.00011455 | 34% | A, B, C |
| P31001 | DESM_MOUSE | 468 | 53367 | 5.3 | (P31001) Desmin | 0.0003401 | 0.00017368 | 51% | A, B, C |
| Q8BP67 | RL24_MOUSE | 157 | 17779 | 11.3 | (Q8BP67) 60S ribosomal protein L24 | 0.00033921 | 0.00013614 | 40% | A, B, C |
| Q9CQ62 | DECR_MOUSE | 335 | 36214 | 8.9 | (Q9CQ62) 2,4-dienoyl-CoA reductase, mitochondrial precursor (EC 1.3.1.34) (2,4-dienoyl-CoA reductase [NADPH]) (4-enoyl-CoA reductase [NADPH]) | 0.0003383 | 0.00015127 | 45% | A, B, C |
| P38060 | HMGCL_MOUSE | 325 | 34161 | 8.3 | (P38060) Hydroxymethylglutaryl-CoA lyase, mitochondrial precursor (EC 4.1.3.4) (HMG-CoA lyase) (HL) (3-hydroxy-3-methylglutarate-CoA lyase) | 0.00033716 | 0.00016374 | 49% | A, B, C |
| Q8BKZ9 | ODPX_MOUSE | 501 | 53999 | 7.8 | (Q8BKZ9) Pyruvate dehydrogenase protein X component, mitochondrial precursor (Dihydrolipoamide dehydrogenase-binding protein of pyruvate dehydrogenase complex) (Lipoyl-containing pyruvate dehydrogenase complex component X) | 0.00033318 | 0.00019754 | 59% | A, B, C |
| Q60864 | STIP1_MOUSE | 543 | 62582 | 6.8 | (Q60864) Stress-induced-phosphoprotein 1 (STI1) (Hsc70/Hsp90-organizing protein) (Hop) (mSTI1) | 0.00033239 | 0.00015452 | 46% | A, B, C |
| P14206 | RSSA_MOUSE | 294 | 32588 | 4.8 | (P14206) 40S ribosomal protein SA (p40) (34/67 kDa laminin receptor) | 0.00033129 | 0.00014371 | 43% | A, B, C |
| P10518 | HEM2_MOUSE | 330 | 36024 | 6.8 | (P10518) Delta-aminolevulinic acid dehydratase (EC 4.2.1.24) (Porphobilinogen synthase) (ALADH) | 0.00033127 | 0.00019167 | 58% | A, B, C |
| P63325 | RS10_MOUSE | 165 | 18916 | 10.2 | (P63325) 40S ribosomal protein S10 | 0.00033064 | 0.00010508 | 32% | A, B, C |
| Q80UU9 | PGRC2_MOUSE | 214 | 23061 | 5.1 | (Q80UU9) Membrane-associated progesterone receptor component 2 (Fragment) | 0.0003289 | 6.9856E-05 | 21% | A, B, C |
| O88696 | CLPP_MOUSE | 272 | 29800 | 7.5 | (O88696) Putative ATP-dependent Clp protease proteolytic subunit, mitochondrial precursor (EC 3.4.21.92) (Endopeptidase Clp) | 0.00032622 | 3.66E-05 | 11% | A, B, C |
| P47941 | CRKL_MOUSE | 303 | 33830 | 6.7 | (P47941) Crk-like protein | 0.00032281 | 0.00036017 | 112% | A, B, C |
| O88685 | PRS6A_MOUSE | 442 | 49493 | 5.2 | (O88685) 26S protease regulatory subunit 6A (TAT-binding protein 1) (TBP-1) | 0.00032256 | 0.00011043 | 34% | A, B, C |
| P61027 | RAB10_MOUSE | 200 | 22541 | 8.4 | (P61027) Ras-related protein Rab-10 | 0.00032183 | 0.00015243 | 47% | A, B, C |
| O70439 | STX7_MOUSE | 260 | 29690 | 5.8 | (O70439) Syntaxin-7 | 0.00032097 | 0.00018907 | 59% | A, B, C |

| | | | | | | | | | |
|---|---|---|---|---|---|---|---|---|---|
| P56375 | ACYP2_MOUSE | 97 | 10895 | 8.7 | (P56375) Acylphosphatase, muscle type isozyme (EC 3.6.1.7) (Acylphosphate phosphohydrolase) | 0.00032056 | 0.00022298 | 70% | A, B, C |
| P52825 | CPT2_MOUSE | 658 | 73927 | 8.2 | (P52825) Carnitine O-palmitoyltransferase 2, mitochondrial precursor (EC 2.3.1.21) (Carnitine palmitoyltransferase II) (CPT II) | 0.00031984 | 7.7337E-05 | 24% | A, B, C |
| P84078 | ARF1_MOUSE | 180 | 20566 | 6.8 | (P84078) ADP-ribosylation factor 1 | 0.00031893 | 4.2675E-05 | 13% | A, B, C |
| P98078 | DAB2_MOUSE | 766 | 82312 | 6.1 | (P98078) Disabled homolog 2 (DOC-2) (Mitogen-responsive phosphoprotein) | 0.00031859 | 5.9912E-05 | 19% | A, B, C |
| P09242 | PPBT_MOUSE | 524 | 57455 | 6.9 | (P09242) Alkaline phosphatase, tissue-nonspecific isozyme precursor (EC 3.1.3.1) (AP-TNAP) (TNSALP) | 0.00031757 | 0.00015148 | 48% | A, B, C |
| Q9QYR9 | ACOT2_MOUSE | 453 | 49652 | 7.4 | (Q9QYR9) Acyl-coenzyme A thioesterase 2, mitochondrial precursor (EC 3.1.2.2) (Acyl-CoA thioesterase 2) (Acyl coenzyme A thioester hydrolase) (Very-long-chain acyl-CoA thioesterase) (MTE-I) | 0.00031348 | 7.9418E-05 | 25% | A, B, C |
| Q9D7N9 | APMAP_MOUSE | 415 | 46434 | 6.3 | (Q9D7N9) Adipocyte plasma membrane-associated protein (Protein DD16) | 0.00031151 | 9.9859E-05 | 32% | A, B, C |
| Q9JM14 | NT5C_MOUSE | 200 | 23076 | 5.5 | (Q9JM14) 5'(3')-deoxyribonucleotidase, cytosolic type (EC 3.1.3.-) (Cytosolic 5',3'-pyrimidine nucleotidase) (Deoxy-5'-nucleotidase 1) (dNT-1) | 0.0003108 | 0.00033333 | 107% | A, B, C |
| Q8BGD9 | IF4B_MOUSE | 611 | 68840 | 5.7 | (Q8BGD9) Eukaryotic translation initiation factor 4B (eIF-4B) | 0.00030812 | 0.00029159 | 95% | A, B, C |
| P62835 | RAP1A_MOUSE | 184 | 20987 | 6.6 | (P62835) Ras-related protein Rap-1A precursor (Ras-related protein Krev-1) | 0.00030658 | 0.00018403 | 60% | A, B, C |
| P50396 | GDIA_MOUSE | 447 | 50522 | 5.1 | (P50396) Rab GDP dissociation inhibitor alpha (Rab GDI alpha) (Guanosine diphosphate dissociation inhibitor 1) (GDI-1) | 0.00030296 | 0.00028078 | 93% | A, B, C |
| P14869 | RLA0_MOUSE | 317 | 34216 | 6.2 | (P14869) 60S acidic ribosomal protein P0 (L10E) | 0.00030145 | 4.8418E-05 | 16% | A, B, C |
| Q9R257 | HEBP1_MOUSE | 190 | 21053 | 5.3 | (Q9R257) Heme-binding protein 1 (p22HBP) | 0.0003004 | 0.00011278 | 38% | A, B, C |
| Q8C0I1 | ADAS_MOUSE | 645 | 71684 | 7.5 | (Q8C0I1) Alkyldihydroxyacetonephosphate synthase, peroxisomal precursor (EC 2.5.1.26) (Alkyl-DHAP synthase) (Alkylglycerone-phosphate synthase) | 0.00029975 | 0.00012299 | 41% | A, B, C |
| P06151 | LDHA_MOUSE | 331 | 36367 | 7.7 | (P06151) L-lactate dehydrogenase A chain (EC 1.1.1.27) (LDH-A) (LDH muscle subunit) (LDH-M) | 0.00029691 | 0.00011438 | 39% | A, B, C |
| P28825 | MEP1A_MOUSE | 747 | 84197 | 6.2 | (P28825) Meprin A subunit alpha precursor (EC 3.4.24.18) (Endopeptidase-2) (MEP-1) | 0.00029547 | 0.00015431 | 52% | A, B, C |
| P48678 | LMNA_MOUSE | 665 | 74238 | 7 | (P48678) Lamin-A/C | 0.00029504 | 0.00013112 | 44% | A, B, C |
| P58252 | EF2_MOUSE | 857 | 95183 | 6.8 | (P58252) Elongation factor 2 (EF-2) | 0.00029471 | 6.3785E-05 | 22% | A, B, C |
| Q68FD5 | CLH_MOUSE | 1675 | 191555 | 5.7 | (Q68FD5) Clathrin heavy chain | 0.00029236 | 5.2757E-05 | 18% | A, B, C |
| P62702 | RS4X_MOUSE | 262 | 29467 | 10.2 | (P62702) 40S ribosomal protein S4, X isoform | 0.00029202 | 6.9762E-05 | 24% | A, B, C |
| O88487 | DC1I2_MOUSE | 612 | 68394 | 5.3 | (O88487) Cytoplasmic dynein 1 intermediate chain 2 (Dynein intermediate chain 2, cytosolic) (DH IC-2) (Cytoplasmic dynein intermediate chain 2) | 0.00029174 | 7.7069E-05 | 26% | A, B, C |
| Q9CZX8 | RS19_MOUSE | 144 | 15954 | 10.4 | (Q9CZX8) 40S ribosomal protein S19 | 0.00029127 | 6.1274E-05 | 21% | A, B, C |
| P54071 | IDHP_MOUSE | 523 | 58749 | 8.7 | (P54071) Isocitrate dehydrogenase [NADP], mitochondrial precursor (EC 1.1.1.42) (Oxalosuccinate decarboxylase) (IDH) (NADP(+)-specific ICDH) (IDP) (ICD-M) | 0.0002883 | 7.5155E-05 | 26% | A, B, C |
| Q9DBG6 | RPN2_MOUSE | 631 | 69063 | 5.8 | (Q9DBG6) Dolichyl-diphosphooligosaccharide--protein glycosyltransferase 63 kDa subunit precursor (EC 2.4.1.119) (Ribophorin II) (RPN-II) | 0.00028823 | 0.00013795 | 48% | A, B, C |
| P62301 | RS13_MOUSE | 150 | 17091 | 10.5 | (P62301) 40S ribosomal protein S13 | 0.00028568 | 8.6311E-05 | 30% | A, B, C |

| | | | | | | | | | |
|---|---|---|---|---|---|---|---|---|---|
| O88428 | PAPS2_MOUSE | 621 | 70291 | 7.6 | (O88428) Bifunctional 3'-phosphoadenosine 5'-phosphosulfate synthetase 2 (PAPS synthetase 2) (PAPSS 2) (Sulfurylase kinase 2) (SK2) (SK 2) [Includes: Sulfate adenylyltransferase (EC 2.7.7.4) (Sulfate adenylate transferase) (SAT) (ATP-sulfuryl | 0.00028548 | 5.9823E-06 | 2% | A, B, C |
| Q9DAW9 | CNN3_MOUSE | 330 | 36429 | 5.7 | (Q9DAW9) Calponin-3 (Calponin, acidic isoform) | 0.00028466 | 0.00013486 | 47% | A, B, C |
| Q9CZG9 | PDK11_MOUSE | 140 | 16182 | 7.1 | (Q9CZG9) PDZ domain-containing protein 11 | 0.0002845 | 0.00016266 | 57% | A, B, C |
| Q8R086 | SUOX_MOUSE | 488 | 54048 | 6.1 | (Q8R086) Sulfite oxidase, mitochondrial precursor (EC 1.8.3.1) | 0.00028433 | 9.7435E-05 | 34% | A, B, C |
| Q64516 | GLPK_MOUSE | 524 | 57458 | 5.6 | (Q64516) Glycerol kinase (EC 2.7.1.30) (ATP:glycerol 3-phosphotransferase) (Glycerokinase) (GK) | 0.00028319 | 0.00012958 | 46% | A, B, C |
| Q9CQ40 | RM49_MOUSE | 166 | 19133 | 9.5 | (Q9CQ40) Mitochondrial 39S ribosomal protein L49 (L49mt) (MRP-L49) | 0.00028298 | 1.9553E-05 | 7% | A, B, C |
| P55096 | ABCD3_MOUSE | 659 | 75483 | 9.3 | (P55096) ATP-binding cassette sub-family D member 3 (70 kDa peroxisomal membrane protein) (PMP70) (PMP68) | 0.00028226 | 0.00017176 | 61% | A, B, C |
| Q8BFZ9 | SPFH2_MOUSE | 340 | 37873 | 5.5 | (Q8BFZ9) SPFH domain-containing protein 2 precursor | 0.00028212 | 2.5795E-05 | 9% | A, B, C |
| P50285 | FMO1_MOUSE | 532 | 59915 | 8.5 | (P50285) Dimethylaniline monooxygenase [N-oxide-forming] 1 (EC 1.14.13.8) (Hepatic flavin-containing monooxygenase 1) (FMO 1) (Dimethylaniline oxidase 1) | 0.00027958 | 0.00019017 | 68% | A, B, C |
| P35293 | RAB18_MOUSE | 206 | 23035 | 5.4 | (P35293) Ras-related protein Rab-18 | 0.00027953 | 9.6798E-05 | 35% | A, B, C |
| P57776 | EF1D_MOUSE | 280 | 31162 | 5 | (P57776) Elongation factor 1-delta (EF-1-delta) | 0.0002795 | 0.00024506 | 88% | A, B, C |
| P70290 | EM55_MOUSE | 466 | 52227 | 7.2 | (P70290) 55 kDa erythrocyte membrane protein (p55) (Membrane protein, palmitoylated 1) (Palmitoylated protein p55) | 0.00027891 | 6.5911E-05 | 24% | A, B, C |
| P09803 | CADH1_MOUSE | 884 | 98256 | 4.8 | (P09803) Epithelial-cadherin precursor (E-cadherin) (Uvomorulin) (Cadherin-1) (ARC-1) (CD324 antigen) [Contains: E-Cad/CTF1; E-Cad/CTF2; E-Cad/CTF3] | 0.00027834 | 7.3441E-05 | 26% | A, B, C |
| Q9WU79 | PROD_MOUSE | 497 | 56774 | 6.8 | (Q9WU79) Proline oxidase, mitochondrial precursor (EC 1.5.3.-) (Proline dehydrogenase) | 0.00027781 | 4.8868E-05 | 18% | A, B, C |
| Q9CR68 | UCRI_MOUSE | 274 | 29368 | 8.7 | (Q9CR68) Ubiquinol-cytochrome c reductase iron-sulfur subunit, mitochondrial precursor (EC 1.10.2.2) (Rieske iron-sulfur protein) (RISP) | 0.00027558 | 0.00024972 | 91% | A, B, C |
| Q61316 | HSP74_MOUSE | 841 | 94133 | 5.2 | (Q61316) Heat shock 70 kDa protein 4 (Heat shock 70-related protein APG-2) | 0.00026897 | 0.00013696 | 51% | A, B, C |
| Q9D2M8 | UB2V2_MOUSE | 144 | 16236 | 8 | (Q9D2M8) Ubiquitin-conjugating enzyme E2 variant 2 (Ubc-like protein MMS2) | 0.00026874 | 8.1808E-05 | 30% | A, B, C |
| P47962 | RL5_MOUSE | 296 | 34269 | 9.8 | (P47962) 60S ribosomal protein L5 | 0.00026689 | 3.8901E-05 | 15% | A, B, C |
| Q9D8E6 | RL4_MOUSE | 418 | 47023 | 11 | (Q9D8E6) 60S ribosomal protein L4 (L1) | 0.00026638 | 0.00014768 | 55% | A, B, C |
| Q60759 | GCDH_MOUSE | 438 | 48647 | 8.6 | (Q60759) Glutaryl-CoA dehydrogenase, mitochondrial precursor (EC 1.3.99.7) (GCD) | 0.00026525 | 0.00016588 | 63% | A, B, C |
| Q64012 | RALY_MOUSE | 312 | 33158 | 8.8 | (Q64012) RNA-binding protein Raly (hnRNP associated with lethal yellow protein) (Maternally expressed hnRNP C-related protein) | 0.00026469 | 0.0001212 | 46% | A, B, C |
| Q8BWF0 | SSDH_MOUSE | 523 | 55968 | 8.3 | (Q8BWF0) Succinate semialdehyde dehydrogenase, mitochondrial precursor (EC 1.2.1.24) (NAD(+)-dependent succinic semialdehyde dehydrogenase) | 0.0002619 | 5.1108E-05 | 20% | A, B, C |
| Q9CWK8 | SNX2_MOUSE | 519 | 58471 | 5.1 | (Q9CWK8) Sorting nexin-2 | 0.00026157 | 0.00013599 | 52% | A, B, C |
| Q80XN0 | BDH_MOUSE | 343 | 38285 | 9 | (Q80XN0) D-beta-hydroxybutyrate dehydrogenase, mitochondrial precursor (EC 1.1.1.30) (BDH) (3-hydroxybutyrate dehydrogenase) | 0.0002586 | 7.7734E-05 | 30% | A, B, C |

| | | | | | | | | | |
|---|---|---|---|---|---|---|---|---|---|
| P31253 | UBE1X_MOUSE | 450 | 50986 | 6.8 | (P31253) Ubiquitin-activating enzyme E1 X (Fragment) | 0.00025636 | 3.6682E-05 | 14% | A, B, C |
| Q02819 | NUCB1_MOUSE | 459 | 53409 | 5.1 | (Q02819) Nucleobindin-1 precursor (CALNUC) | 0.00025602 | 2.9958E-05 | 12% | A, B, C |
| P26041 | MOES_MOUSE | 576 | 67636 | 6.6 | (P26041) Moesin (Membrane-organizing extension spike protein) | 0.00025508 | 0.00017025 | 67% | A, B, C |
| P19253 | RL13A_MOUSE | 202 | 23333 | 11 | (P19253) 60S ribosomal protein L13a (Transplantation antigen P198) (Tum-P198 antigen) | 0.00025402 | 0.00020061 | 79% | A, B, C |
| P53395 | ODB2_MOUSE | 482 | 53160 | 8.7 | (P53395) Lipoamide acyltransferase component of branched-chain alpha-keto acid dehydrogenase complex, mitochondrial precursor (EC 2.3.1.168) (Dihydrolipoyllysine-residue (2-methylpropanoyl)transferase) (E2) (Dihydrolipoamide branched chain tra | 0.00025274 | 5.1658E-05 | 20% | A, B, C |
| O70475 | UGDH_MOUSE | 493 | 54832 | 7.6 | (O70475) UDP-glucose 6-dehydrogenase (EC 1.1.1.22) (UDP-Glc dehydrogenase) (UDP-GlcDH) (UDPGDH) | 0.00025226 | 3.6336E-05 | 14% | A, B, C |
| P23492 | PNPH_MOUSE | 289 | 32277 | 6.2 | (P23492) Purine nucleoside phosphorylase (EC 2.4.2.1) (Inosine phosphorylase) (PNP) | 0.00025087 | 0.00012299 | 49% | A, B, C |
| Q7TMF3 | NDUAC_MOUSE | 145 | 17086 | 9.4 | (Q7TMF3) NADH dehydrogenase [ubiquinone] 1 alpha subcomplex subunit 12 (EC 1.6.5.3) (EC 1.6.99.3) (NADH-ubiquinone oxidoreductase subunit B17.2) (Complex I-B17.2) (CI B17.2) (CIB17.2) | 0.00024989 | 0.00016153 | 65% | A, B, C |
| Q61599 | GDIS_MOUSE | 199 | 22720 | 5.1 | (Q61599) Rho GDP-dissociation inhibitor 2 (Rho GDI 2) (Rho-GDI beta) (D4) | 0.00024705 | 0.00014274 | 58% | A, B, C |
| Q80X50 | UBP2L_MOUSE | 1107 | 116799 | 7.1 | (Q80X50) Ubiquitin-associated protein 2-like | 0.00024651 | 5.0377E-05 | 20% | A, B, C |
| P52196 | THTR_MOUSE | 296 | 33335 | 7.9 | (P52196) Thiosulfate sulfurtransferase (EC 2.8.1.1) (Rhodanese) | 0.00024623 | 0.00014108 | 57% | A, B, C |
| Q9EQU5 | SET_MOUSE | 289 | 33378 | 4.3 | (Q9EQU5) Protein SET (Phosphatase 2A inhibitor I2PP2A) (I-2PP2A) (Template-activating factor I) (TAF-I) | 0.00024513 | 0.00023165 | 94% | A, B, C |
| Q9ERR7 | SEP15_MOUSE | 162 | 17731 | 5.4 | (Q9ERR7) 15 kDa selenoprotein precursor | 0.00024449 | 7.3734E-05 | 30% | A, B, C |
| Q99020 | ROAA_MOUSE | 285 | 30831 | 7.9 | (Q99020) Heterogeneous nuclear ribonucleoprotein A/B (hnRNP A/B) (CArG-binding factor-A) (CBF-A) | 0.0002438 | 0.00010495 | 43% | A, B, C |
| Q8BH59 | CMC1_MOUSE | 677 | 74570 | 8.3 | (Q8BH59) Calcium-binding mitochondrial carrier protein Aralar1 (Mitochondrial aspartate glutamate carrier 1) (Solute carrier family 25 member 12) | 0.00024264 | 0.00015986 | 66% | A, B, C |
| Q9DBG9 | TX1B3_MOUSE | 124 | 13723 | 8.5 | (Q9DBG9) Tax1-binding protein 3 (Tax interaction protein 1) (TIP 1) | 0.00024138 | 7.1285E-05 | 30% | A, B, C |
| Q8JZN5 | ACAD9_MOUSE | 625 | 68707 | 7.9 | (Q8JZN5) Acyl-CoA dehydrogenase family member 9, mitochondrial precursor (EC 1.3.99.-) (ACAD-9) | 0.00024094 | 0.00019215 | 80% | A, B, C |
| Q920R6 | VPP4_MOUSE | 833 | 95605 | 6.4 | (Q920R6) Vacuolar proton translocating ATPase 116 kDa subunit a isoform 4 (V-ATPase 116 kDa isoform a4) (Vacuolar proton translocating ATPase 116 kDa subunit a kidney isoform) | 0.00024018 | 8.4188E-05 | 35% | A, B, C |
| Q9JKB3 | DBPA_MOUSE | 361 | 38814 | 9.7 | (Q9JKB3) DNA-binding protein A (Cold shock domain-containing protein A) (Y-box protein 3) | 0.00023873 | 9.3134E-05 | 39% | A, B, C |
| Q04447 | KCRB_MOUSE | 381 | 42713 | 5.7 | (Q04447) Creatine kinase B-type (EC 2.7.3.2) (Creatine kinase B chain) (B-CK) | 0.00023861 | 8.1577E-05 | 34% | A, B, C |
| P61161 | ARP2_MOUSE | 394 | 44761 | 6.7 | (P61161) Actin-like protein 2 (Actin-related protein 2) | 0.00023836 | 0.0002587 | 109% | A, B, C |
| O88587 | COMT_MOUSE | 265 | 29496 | 5.8 | (O88587) Catechol O-methyltransferase (EC 2.1.1.6) | 0.00023824 | 0.0001501 | 63% | A, B, C |
| Q9ERB0 | SNP29_MOUSE | 260 | 29572 | 5.4 | (Q9ERB0) Synaptosomal-associated protein 29 (SNAP-29) (Vesicle-membrane fusion protein SNAP-29) (Soluble 29 kDa NSF attachment protein) (Golgi SNARE of 32 kDa) (Gs32) | 0.00023749 | 5.6476E-05 | 24% | A, B, C |

| | | | | | | | | | |
|---|---|---|---|---|---|---|---|---|---|
| O35295 | PURB_MOUSE | 323 | 33770 | 5.4 | (O35295) Transcriptional activator protein Pur-beta (Purine-rich element-binding protein B) (Vascular actin single-stranded DNA-binding factor 2 p44 component) | 0.00023726 | 0.00010018 | 42% | A, B, C |
| Q9Z2D6 | MECP2_MOUSE | 484 | 52308 | 10 | (Q9Z2D6) Methyl-CpG-binding protein 2 (MeCP-2 protein) (MeCP2) | 0.00023394 | 0.00010051 | 43% | A, B, C |
| Q8VDJ3 | VIGLN_MOUSE | 1268 | 141742 | 6.9 | (Q8VDJ3) Vigilin (High density lipoprotein-binding protein) (HDL-binding protein) | 0.00023382 | 5.2557E-05 | 22% | A, B, C |
| P62192 | PRS4_MOUSE | 440 | 49185 | 6.2 | (P62192) 26S protease regulatory subunit 4 (P26s4) (Proteasome 26S subunit ATPase 1) | 0.00023183 | 0.00010866 | 47% | A, B, C |
| Q8K1E0 | STX5_MOUSE | 301 | 34103 | 8.5 | (Q8K1E0) Syntaxin-5 | 0.00023145 | 0.00019469 | 84% | A, B, C |
| Q6P1B1 | XPP1_MOUSE | 623 | 69591 | 5.5 | (Q6P1B1) Xaa-Pro aminopeptidase 1 (EC 3.4.11.9) (X-Pro aminopeptidase 1) (X-prolyl aminopeptidase 1, soluble) (Cytosolic aminopeptidase P) (Soluble aminopeptidase P) (sAmp) (Aminoacylproline aminopeptidase) | 0.00023087 | 3.7464E-05 | 16% | A, B, C |
| Q9WTI7 | MYO1C_MOUSE | 1028 | 118156 | 9.4 | (Q9WTI7) Myosin Ic (Myosin I beta) (MMIb) | 0.00023041 | 0.00016164 | 70% | A, B, C |
| Q62418 | DBNL_MOUSE | 436 | 48700 | 4.9 | (Q62418) Drebrin-like protein (SH3 domain-containing protein 7) (Actin-binding protein 1) | 0.00022834 | 5.7404E-05 | 25% | A, B, C |
| Q9CQJ6 | DENR_MOUSE | 198 | 22166 | 5.3 | (Q9CQJ6) Density-regulated protein (DRP) | 0.00022821 | 4.5193E-05 | 20% | A, B, C |
| P17156 | HSP72_MOUSE | 633 | 69741 | 5.8 | (P17156) Heat shock-related 70 kDa protein 2 (Heat shock protein 70.2) | 0.00022756 | 0.00015809 | 69% | A, B, C |
| Q8K310 | MATR3_MOUSE | 846 | 94630 | 6.3 | (Q8K310) Matrin-3 | 0.00022731 | 0.0001319 | 58% | A, B, C |
| Q9DBT9 | M2GD_MOUSE | 869 | 97255 | 7.9 | (Q9DBT9) Dimethylglycine dehydrogenase, mitochondrial precursor (EC 1.5.99.2) (ME2GLYDH) | 0.00022698 | 3.1888E-05 | 14% | A, B, C |
| Q8K157 | GALM_MOUSE | 342 | 37799 | 6.7 | (Q8K157) Aldose 1-epimerase (EC 5.1.3.3) (Galactose mutarotase) | 0.00022697 | 0.00017482 | 77% | A, B, C |
| O35643 | AP1B1_MOUSE | 943 | 103979 | 5.1 | (O35643) AP-1 complex subunit beta-1 (Adapter-related protein complex 1 beta-1 subunit) (Beta-adaptin 1) (Adaptor protein complex AP-1 beta-1 subunit) (Golgi adaptor HA1/AP1 adaptin beta subunit) (Clathrin assembly protein complex 1 beta larg | 0.00022652 | 3.3717E-05 | 15% | A, B, C |
| P28352 | APEX1_MOUSE | 316 | 35359 | 7.9 | (P28352) DNA-(apurinic or apyrimidinic site) lyase (EC 4.2.99.18) (AP endonuclease 1) (APEX nuclease) (APEN) | 0.00022591 | 0.00017286 | 77% | A, B, C |
| Q8VDQ1 | ZADH1_MOUSE | 351 | 38054 | 5.5 | (Q8VDQ1) Zinc-binding alcohol dehydrogenase domain-containing protein 1 (EC 1.-.-.-) | 0.00022575 | 0.00012288 | 54% | A, B, C |
| Q9CU62 | SMC1A_MOUSE | 1233 | 143215 | 7.4 | (Q9CU62) Structural maintenance of chromosome 1-like 1 protein (SMC1alpha protein) (Chromosome segregation protein SmcB) (Sb1.8) | 0.00022539 | 0.00011512 | 51% | A, B, C |
| P55302 | AMRP_MOUSE | 360 | 42215 | 7.9 | (P55302) Alpha-2-macroglobulin receptor-associated protein precursor (Alpha-2-MRAP) (Low density lipoprotein receptor-related protein-associated protein 1) (RAP) (Heparin-binding protein 44) (HBP-44) | 0.00022349 | 1.4498E-05 | 6% | A, B, C |
| P11499 | HS90B_MOUSE | 723 | 83194 | 5 | (P11499) Heat shock protein HSP 90-beta (HSP 84) (Tumor-specific transplantation 84 kDa antigen) (TSTA) | 0.00022248 | 6.4864E-05 | 29% | A, B, C |
| Q7TPR4 | ACTN1_MOUSE | 892 | 103068 | 5.4 | (Q7TPR4) Alpha-actinin-1 (Alpha-actinin cytoskeletal isoform) (Non-muscle alpha-actinin-1) (F-actin cross linking protein) | 0.00022242 | 6.2297E-05 | 28% | A, B, C |
| Q9D1D4 | TMEDA_MOUSE | 219 | 24911 | 6.7 | (Q9D1D4) Transmembrane emp24 domain-containing protein 10 precursor (Transmembrane protein Tmp21) (21 kDa transmembrane-trafficking protein) | 0.00022216 | 0.00012595 | 57% | A, B, C |

| | | | | | | | | | |
|---|---|---|---|---|---|---|---|---|---|
| Q9CQN1 | TRAP1_MOUSE | 706 | 80209 | 6.7 | (Q9CQN1) Heat shock protein 75 kDa, mitochondrial precursor (HSP 75) (Tumor necrosis factor type 1 receptor-associated protein) (TRAP-1) (TNFR-associated protein 1) | 0.00022154 | 9.1011E-05 | 41% | A, B, C |
| Q91V41 | RAB14_MOUSE | 214 | 23766 | 6.2 | (Q91V41) Ras-related protein Rab-14 | 0.00022037 | 5.4572E-05 | 25% | A, B, C |
| P05784 | K1C18_MOUSE | 422 | 47373 | 5.3 | (P05784) Keratin, type I cytoskeletal 18 (Cytokeratin-18) (CK-18) (Keratin-18) (K18) (Cytokeratin endo B) (Keratin D) | 0.00021881 | 5.6468E-05 | 26% | A, B, C |
| Q91YT0 | NUBM_MOUSE | 464 | 50834 | 8.2 | (Q91YT0) NADH-ubiquinone oxidoreductase 51 kDa subunit, mitochondrial precursor (EC 1.6.5.3) (EC 1.6.99.3) (Complex I-51KD) (CI-51KD) (NADH dehydrogenase flavoprotein 1) | 0.0002183 | 8.3121E-05 | 38% | A, B, C |
| P30416 | FKBP4_MOUSE | 457 | 51441 | 5.7 | (P30416) FK506-binding protein 4 (EC 5.2.1.8) (Peptidyl-prolyl cis-trans isomerase) (PPIase) (Rotamase) (p59 protein) (HSP-binding immunophilin) (HBI) (FKBP52 protein) (52 kDa FK506-binding protein) (FKBP59) | 0.00021785 | 5.4766E-05 | 25% | A, B, C |
| Q9CWW6 | PIN4_MOUSE | 131 | 13815 | 9.8 | (Q9CWW6) Peptidyl-prolyl cis-trans isomerase NIMA-interacting 4 (EC 5.2.1.8) (Rotamase Pin4) (PPIase Pin4) | 0.00021784 | 0.00015584 | 72% | A, B, C |
| Q99L47 | F10A1_MOUSE | 371 | 41656 | 5.3 | (Q99L47) Hsc70-interacting protein (Hip) (Protein ST13 homolog) (Protein FAM10A1) | 0.00021723 | 0.0001945 | 90% | A, B, C |
| Q9D967 | MGDP1_MOUSE | 164 | 18582 | 6.8 | (Q9D967) Magnesium-dependent phosphatase 1 (EC 3.1.3.-) (EC 3.1.3.48) (MDP-1) | 0.00021641 | 0.0001144 | 53% | A, B, C |
| P07356 | ANXA2_MOUSE | 338 | 38545 | 7.7 | (P07356) Annexin A2 (Annexin II) (Lipocortin II) (Calpactin I heavy chain) (Chromobindin-8) (p36) (Protein I) (Placental anticoagulant protein IV) (PAP-IV) | 0.00021462 | 3.6455E-05 | 17% | A, B, C |
| Q62167 | DDX3X_MOUSE | 661 | 72970 | 7.2 | (Q62167) ATP-dependent RNA helicase DDX3X (EC 3.6.1.-) (DEAD box protein 3, X-chromosomal) (DEAD box RNA helicase DEAD3) (mDEAD3) (Embryonic RNA helicase) (D1Pas1-related sequence 2) | 0.00021435 | 0.00017994 | 84% | A, B, C |
| P48758 | DHCA_MOUSE | 276 | 30597 | 7.8 | (P48758) Carbonyl reductase [NADPH] 1 (EC 1.1.1.184) (NADPH-dependent carbonyl reductase 1) | 0.00021395 | 8.6856E-05 | 41% | A, B, C |
| Q925I1 | ATAD3_MOUSE | 591 | 66742 | 9.3 | (Q925I1) ATPase family AAA domain-containing protein 3 (AAA-ATPase TOB3) | 0.00021363 | 0.00014494 | 68% | A, B, C |
| Q8VBV7 | CSN8_MOUSE | 209 | 23256 | 5.2 | (Q8VBV7) COP9 signalosome complex subunit 8 (Signalosome subunit 8) (SGN8) (JAB1-containing signalosome subunit 8) (COP9 homolog) | 0.00021358 | 1.69E-05 | 8% | A, B, C |
| P14733 | LMNB1_MOUSE | 587 | 66654 | 5.2 | (P14733) Lamin-B1 | 0.00021286 | 6.8376E-05 | 32% | A, B, C |
| P56376 | ACYP1_MOUSE | 98 | 11110 | 9.1 | (P56376) Acylphosphatase, organ-common type isozyme (EC 3.6.1.7) (Acylphosphate phosphohydrolase) | 0.00021136 | 0.00012227 | 58% | A, B, C |
| P29758 | OAT_MOUSE | 439 | 48355 | 6.6 | (P29758) Ornithine aminotransferase, mitochondrial precursor (EC 2.6.1.13) (Ornithine--oxo-acid aminotransferase) | 0.00021029 | 4.7753E-05 | 23% | A, B, C |
| Q99LB7 | SARDH_MOUSE | 919 | 101682 | 6.7 | (Q99LB7) Sarcosine dehydrogenase, mitochondrial precursor (EC 1.5.99.1) (SarDH) | 0.00020984 | 8.9535E-05 | 43% | A, B, C |
| Q99KQ4 | NAMPT_MOUSE | 491 | 55447 | 7.2 | (Q99KQ4) Nicotinamide phosphoribosyltransferase (EC 2.4.2.12) (NAmPRTase) (Nampt) (Pre-B-cell colony-enhancing factor 1 homolog) (PBEF) (Visfatin) | 0.00020901 | 5.7628E-05 | 28% | A, B, C |
| P28656 | NP1L1_MOUSE | 391 | 45345 | 4.5 | (P28656) Nucleosome assembly protein 1-like 1 (NAP-1-related protein) (Brain protein DN38) | 0.00020894 | 3.7915E-05 | 18% | A, B, C |
| Q9CQM9 | TXNL2_MOUSE | 337 | 37778 | 5.6 | (Q9CQM9) Thioredoxin-like protein 2 (PKC-interacting cousin of thioredoxin) (PKC-theta-interacting protein) (PKCq-interacting protein) | 0.00020815 | 0.00012299 | 59% | A, B, C |

| | | | | | | | | | |
|---|---|---|---|---|---|---|---|---|---|
| Q68FL4 | SAHH3_MOUSE | 613 | 66899 | 7.4 | (Q68FL4) Putative adenosylhomocysteinase 3 (EC 3.3.1.1) (S-adenosyl-L-homocysteine hydrolase 3) (AdoHcyase 3) | 0.00020756 | 8.3383E-05 | 40% | A, B, C |
| Q9D172 | ES1_MOUSE | 266 | 28090 | 8.8 | (Q9D172) ES1 protein homolog, mitochondrial precursor | 0.00020716 | 8.8205E-05 | 43% | A, B, C |
| Q91WN4 | KMO_MOUSE | 479 | 54532 | 8.9 | (Q91WN4) Kynurenine 3-monooxygenase (EC 1.14.13.9) (Kynurenine 3-hydroxylase) | 0.00020597 | 7.405E-05 | 36% | A, B, C |
| Q60668 | HNRPD_MOUSE | 355 | 38354 | 7.8 | (Q60668) Heterogeneous nuclear ribonucleoprotein D0 (hnRNP D0) (AU-rich element RNA-binding protein 1) | 0.00020571 | 0.00011731 | 57% | A, B, C |
| O35387 | HAX1_MOUSE | 280 | 31654 | 4.9 | (O35387) HS1-associating protein X-1 (HAX-1) (HS1-binding protein) | 0.00020482 | 0.00021473 | 105% | A, B, C |
| Q9EP89 | LACTB_MOUSE | 551 | 60706 | 8.9 | (Q9EP89) Serine beta-lactamase-like protein LACTB | 0.00020393 | 9.8472E-05 | 48% | A, B, C |
| P21981 | TGM2_MOUSE | 686 | 77046 | 5.1 | (P21981) Protein-glutamine gamma-glutamyltransferase 2 (EC 2.3.2.13) (Tissue transglutaminase) (TGase C) (TGC) (TG(C)) (Transglutaminase-2) | 0.0002037 | 0.00018449 | 91% | A, B, C |
| Q8BH86 | CN159_MOUSE | 617 | 66366 | 7.1 | (Q8BH86) Protein C14orf159 homolog, mitochondrial precursor | 0.00020355 | 9.4685E-05 | 47% | A, B, C |
| P46938 | YAP1_MOUSE | 472 | 50703 | 5 | (P46938) 65 kDa Yes-associated protein (YAP65) | 0.0002025 | 6.1304E-05 | 30% | A, B, C |
| Q93092 | TALDO_MOUSE | 337 | 37387 | 7 | (Q93092) Transaldolase (EC 2.2.1.2) | 0.00020241 | 0.00012417 | 61% | A, B, C |
| Q80Y14 | GLRX5_MOUSE | 152 | 16292 | 6.5 | (Q80Y14) Glutaredoxin-related protein 5 | 0.00020217 | 2.2566E-05 | 11% | A, B, C |
| Q9EPL9 | ACOX3_MOUSE | 700 | 78539 | 7.4 | (Q9EPL9) Acyl-coenzyme A oxidase 3, peroxisomal (EC 1.3.3.6) (Pristanoyl-CoA oxidase) (Branched-chain acyl-CoA oxidase) (BRCACox) | 0.00020194 | 0.00016369 | 81% | A, B, C |
| P22315 | HEMH_MOUSE | 420 | 47130 | 8.9 | (P22315) Ferrochelatase, mitochondrial precursor (EC 4.99.1.1) (Protoheme ferro-lyase) (Heme synthetase) | 0.00020189 | 4.8812E-05 | 24% | A, B, C |
| P16331 | PH4H_MOUSE | 452 | 51798 | 6.4 | (P16331) Phenylalanine-4-hydroxylase (EC 1.14.16.1) (PAH) (Phe-4-monooxygenase) | 0.00020117 | 0.00012576 | 63% | A, B, C |
| Q3THW5 | H2AV_MOUSE | 127 | 13377 | 10.6 | (Q3THW5) Histone H2AV (H2A.F/Z) | 0.00020057 | 0.00016346 | 81% | A, B, C |
| P99024 | TBB5_MOUSE | 444 | 49671 | 4.9 | (P99024) Tubulin beta-5 chain | 0.00019908 | 9.7545E-05 | 49% | A, B, C |
| Q8BTM8 | FLNA_MOUSE | 2646 | 281060 | 6 | (Q8BTM8) Filamin-A (Alpha-filamin) (Filamin-1) (Endothelial actin-binding protein) (Actin-binding protein 280) (ABP-280) (Nonmuscle filamin) | 0.00019858 | 2.8512E-05 | 14% | A, B, C |
| Q91VC4 | PLVAP_MOUSE | 438 | 49933 | 8.4 | (Q91VC4) Plasmalemma vesicle-associated protein (Plasmalemma vesicle protein 1) (PV-1) (MECA-32 antigen) | 0.00019815 | 8.2382E-05 | 42% | A, B, C |
| P61164 | ACTZ_MOUSE | 376 | 42614 | 6.6 | (P61164) Alpha-centractin (Centractin) (Centrosome-associated actin homolog) (Actin-RPV) (ARP1) | 0.00019752 | 0.00014173 | 72% | A, B, C |
| Q9CZU6 | CISY_MOUSE | 464 | 51737 | 8.6 | (Q9CZU6) Citrate synthase, mitochondrial precursor (EC 2.3.3.1) | 0.00019743 | 0.00012176 | 62% | A, B, C |
| P80315 | TCPD_MOUSE | 538 | 57935 | 8 | (P80315) T-complex protein 1 subunit delta (TCP-1-delta) (CCT-delta) (A45) | 0.00019728 | 7.7618E-05 | 39% | A, B, C |
| Q9WV80 | SNX1_MOUSE | 522 | 58952 | 5.2 | (Q9WV80) Sorting nexin-1 | 0.00019714 | 0.00010661 | 54% | A, B, C |
| P49935 | CATH_MOUSE | 333 | 37184 | 8.4 | (P49935) Cathepsin H precursor (EC 3.4.22.16) (Cathepsin B3) (Cathepsin BA) [Contains: Cathepsin H mini chain; Cathepsin H heavy chain; Cathepsin H light chain] | 0.00019659 | 0.00010467 | 53% | A, B, C |
| P30275 | KCRU_MOUSE | 418 | 47004 | 8.2 | (P30275) Creatine kinase, ubiquitous mitochondrial precursor (EC 2.7.3.2) (U-MtCK) (Mia-CK) (Acidic-type mitochondrial creatine kinase) | 0.00019572 | 0.00016881 | 86% | A, B, C |
| P30999 | CTND1_MOUSE | 911 | 101731 | 6.9 | (P30999) Catenin delta-1 (p120 catenin) (p120(ctn)) (Cadherin-associated Src substrate) (CAS) (p120(cas)) | 0.00019557 | 1.7565E-05 | 9% | A, B, C |
| Q9QUH0 | GLRX1_MOUSE | 106 | 11740 | 8.4 | (Q9QUH0) Glutaredoxin-1 (Thioltransferase-1) (TTase-1) | 0.00019553 | 0.00012195 | 62% | A, B, C |
| Q91YP0 | L2HDH_MOUSE | 464 | 50899 | 8.3 | (Q91YP0) L-2-hydroxyglutarate dehydrogenase, mitochondrial precursor (EC 1.1.99.2) (Duranin) | 0.00019519 | 0.00013795 | 71% | A, B, C |

| | | | | | | | | | |
|---|---|---|---|---|---|---|---|---|---|
| P37040 | NCPR_MOUSE | 677 | 76913 | 5.5 | (P37040) NADPH--cytochrome P450 reductase (EC 1.6.2.4) (CPR) (P450R) | 0.00019517 | 2.6269E-05 | 13% | A, B, C |
| P26043 | RADI_MOUSE | 583 | 68601 | 6.1 | (P26043) Radixin (ESP10) | 0.00019459 | 0.00017514 | 90% | A, B, C |
| Q8VI36 | PAXI_MOUSE | 591 | 64476 | 6 | (Q8VI36) Paxillin | 0.00019398 | 5.8852E-05 | 30% | A, B, C |
| P45376 | ALDR_MOUSE | 315 | 35601 | 7.2 | (P45376) Aldose reductase (EC 1.1.1.21) (AR) (Aldehyde reductase) | 0.00019369 | 2.6377E-05 | 14% | A, B, C |
| P59017 | B2L13_MOUSE | 434 | 46669 | 4.6 | (P59017) Bcl-2-like 13 protein (Protein Mil1) (Bcl-rambo) | 0.00019252 | 9.1395E-05 | 47% | A, B, C |
| Q61879 | MYH10_MOUSE | 1976 | 228994 | 5.5 | (Q61879) Myosin-10 (Myosin heavy chain, nonmuscle IIb) (Nonmuscle myosin heavy chain IIb) (NMMHC II-b) (NMMHC-IIB) (Cellular myosin heavy chain, type B) (Nonmuscle myosin heavy chain-B) (NMMHC-B) | 0.00019216 | 6.3653E-05 | 33% | A, B, C |
| Q925B0 | PAWR_MOUSE | 197 | 22686 | 5.5 | (Q925B0) PRKC apoptosis WT1 regulator protein (Prostate apoptosis response 4 protein) (Par-4) (Fragment) | 0.00019155 | 5.126E-05 | 27% | A, B, C |
| Q02053 | UBE1_MOUSE | 1058 | 117809 | 5.7 | (Q02053) Ubiquitin-activating enzyme E1 1 | 0.00019058 | 4.2424E-05 | 22% | A, B, C |
| Q66JS6 | IF31_MOUSE | 263 | 29486 | 4.8 | (Q66JS6) Eukaryotic translation initiation factor 3 subunit 1 (eIF-3 alpha) | 0.00019047 | 0.00020806 | 109% | A, B, C |
| O09061 | PSB1_MOUSE | 240 | 26372 | 7.8 | (O09061) Proteasome subunit beta type 1 (EC 3.4.25.1) (Proteasome component C5) (Macropain subunit C5) (Multicatalytic endopeptidase complex subunit C5) (Proteasome gamma chain) | 0.00019043 | 6.0197E-05 | 32% | A, B, C |
| Q61233 | PLSL_MOUSE | 626 | 70018 | 5.3 | (Q61233) Plastin-2 (L-plastin) (Lymphocyte cytosolic protein 1) (LCP-1) (65 kDa macrophage protein) (pp65) | 0.00018975 | 2.079E-05 | 11% | A, B, C |
| Q9DCQ2 | ASPD_MOUSE | 287 | 30270 | 7 | (Q9DCQ2) Putative L-aspartate dehydrogenase (EC 1.4.1.21) | 0.00018945 | 7.0888E-05 | 37% | A, B, C |
| P61982 | 1433G_MOUSE | 246 | 28171 | 4.9 | (P61982) 14-3-3 protein gamma | 0.00018938 | 0.00010002 | 53% | A, B, C |
| P47911 | RL6_MOUSE | 295 | 33378 | 10.7 | (P47911) 60S ribosomal protein L6 (TAX-responsive enhancer element-binding protein 107) (TAXREB107) | 0.00018848 | 0.00016174 | 86% | A, B, C |
| Q91X52 | DCXR_MOUSE | 244 | 25746 | 7.3 | (Q91X52) L-xylulose reductase (EC 1.1.1.10) (XR) (Dicarbonyl/L-xylulose reductase) | 0.00018822 | 0.00016197 | 86% | A, B, C |
| Q9CYZ2 | TPD54_MOUSE | 220 | 24043 | 6.1 | (Q9CYZ2) Tumor protein D54 (Tumor protein D52-like 2) | 0.00018776 | 0.00011507 | 61% | A, B, C |
| Q8BVE3 | VATH_MOUSE | 483 | 55855 | 6.6 | (Q8BVE3) Vacuolar ATP synthase subunit H (EC 3.6.3.14) (V-ATPase H subunit) (Vacuolar proton pump H subunit) | 0.00018681 | 0.00010771 | 58% | A, B, C |
| Q8K1M6 | DNM1L_MOUSE | 742 | 82658 | 7.1 | (Q8K1M6) Dynamin-1-like protein (EC 3.6.5.5) (Dynamin-related protein 1) (Dynamin family member proline-rich carboxyl-terminal domain less) (Dymple) | 0.00018655 | 8.55E-05 | 46% | A, B, C |
| Q8BXK9 | CLIC5_MOUSE | 251 | 28287 | 5.9 | (Q8BXK9) Chloride intracellular channel protein 5 | 0.0001864 | 2.5947E-05 | 14% | A, B, C |
| Q8R1Q8 | DC1L1_MOUSE | 523 | 56614 | 6.4 | (Q8R1Q8) Cytoplasmic dynein 1 light intermediate chain 1 (Dynein light intermediate chain 1, cytosolic) (Dynein light chain A) (DLC-A) | 0.00018619 | 1.214E-05 | 7% | A, B, C |
| Q8VIJ6 | SFPQ_MOUSE | 699 | 75442 | 9.4 | (Q8VIJ6) Splicing factor, proline- and glutamine-rich (Polypyrimidine tract-binding protein-associated-splicing factor) (PTB-associated-splicing factor) (PSF) (DNA-binding p52/p100 complex, 100 kDa subunit) | 0.00018561 | 0.00015161 | 82% | A, B, C |
| Q99M87 | DNJA3_MOUSE | 480 | 52443 | 9.2 | (Q99M87) DnaJ homolog subfamily A member 3, mitochondrial precursor (Tumorous imaginal discs protein Tid56 homolog) (DnaJ protein Tid-1) (mTid-1) | 0.0001849 | 0.00013857 | 75% | A, B, C |
| Q922Y1 | U33K_MOUSE | 297 | 33573 | 5.3 | (Q922Y1) UBA/UBX 33.3 kDa protein | 0.00018408 | 0.0001371 | 74% | A, B, C |
| P35979 | RL12_MOUSE | 165 | 17805 | 9.4 | (P35979) 60S ribosomal protein L12 | 0.00018324 | 0.00015225 | 83% | A, B, C |
| Q8CDN6 | TXNL1_MOUSE | 288 | 32106 | 5 | (Q8CDN6) Thioredoxin-like protein 1 (32 kDa thioredoxin-related protein) | 0.00018247 | 5.2678E-05 | 29% | A, B, C |

| | | | | | | | | | |
|---|---|---|---|---|---|---|---|---|---|
| Q8R3F5 | FABD_MOUSE | 381 | 41928 | 8.1 | (Q8R3F5) Malonyl CoA-acyl carrier protein transacylase, mitochondrial precursor (EC 2.3.1.39) (MCT) (Mitochondrial malonyltransferase) | 0.00018235 | 2.2776E-05 | 12% | A, B, C |
| P80318 | TCPG_MOUSE | 545 | 60630 | 6.7 | (P80318) T-complex protein 1 subunit gamma (TCP-1-gamma) (CCT-gamma) (Matricin) (mTRiC-P5) | 0.00018224 | 5.3641E-05 | 29% | A, B, C |
| Q9BCZ4 | SELS_MOUSE | 190 | 21462 | 9.3 | (Q9BCZ4) Selenoprotein S (VCP-interacting membrane protein) (Minor histocompatibility antigen H47) | 0.00018211 | 7.7153E-05 | 42% | A, B, C |
| P27546 | MAP4_MOUSE | 1125 | 117675 | 5 | (P27546) Microtubule-associated protein 4 (MAP 4) | 0.00018157 | 1.7523E-05 | 10% | A, B, C |
| O35621 | PMM1_MOUSE | 262 | 29775 | 5.5 | (O35621) Phosphomannomutase 1 (EC 5.4.2.8) (PMM 1) | 0.00018138 | 1.9172E-05 | 11% | A, B, C |
| P97371 | PSME1_MOUSE | 249 | 28673 | 6 | (P97371) Proteasome activator complex subunit 1 (Proteasome activator 28-alpha subunit) (PA28alpha) (PA28a) (Activator of multicatalytic protease subunit 1) (11S regulator complex subunit alpha) (REG-alpha) | 0.0001813 | 9.0756E-05 | 50% | A, B, C |
| Q8BG32 | PSD11_MOUSE | 421 | 47306 | 6.5 | (Q8BG32) 26S proteasome non-ATPase regulatory subunit 11 (26S proteasome regulatory subunit S9) (26S proteasome regulatory subunit p44.5) | 0.00018043 | 4.5358E-05 | 25% | A, B, C |
| Q9WV85 | NDK3_MOUSE | 169 | 19099 | 6.7 | (Q9WV85) Nucleoside diphosphate kinase 3 (EC 2.7.4.6) (NDK 3) (NDP kinase 3) (Nucleoside diphosphate kinase C) (NDPKC) (nm23-M3) (DR-nm23) | 0.00018042 | 6.7449E-05 | 37% | A, B, C |
| Q78PY7 | SND1_MOUSE | 910 | 102088 | 7.4 | (Q78PY7) Staphylococcal nuclease domain-containing protein 1 (p100 co-activator) (100 kDa coactivator) | 0.00017994 | 7.4037E-05 | 41% | A, B, C |
| P42932 | TCPQ_MOUSE | 547 | 59424 | 5.6 | (P42932) T-complex protein 1 subunit theta (TCP-1-theta) (CCT-theta) | 0.00017963 | 2.2932E-05 | 13% | A, B, C |
| P40630 | TFAM_MOUSE | 243 | 27988 | 9.7 | (P40630) Transcription factor A, mitochondrial precursor (mtTFA) (Testis-specific high mobility group protein) (TS-HMG) | 0.00017936 | 0.00013234 | 74% | A, B, C |
| Q9CQ65 | MTAP_MOUSE | 283 | 31062 | 7.1 | (Q9CQ65) S-methyl-5-thioadenosine phosphorylase (EC 2.4.2.28) (5'-methylthioadenosine phosphorylase) (MTA phosphorylase) (MTAPase) | 0.00017924 | 0.00016766 | 94% | A, B, C |
| O08795 | GLU2B_MOUSE | 521 | 58793 | 4.5 | (O08795) Glucosidase 2 subunit beta precursor (Glucosidase II subunit beta) (Protein kinase C substrate, 60.1 kDa protein, heavy chain) (PKCSH) (80K-H protein) | 0.00017892 | 5.4552E-05 | 30% | A, B, C |
| Q91X78 | SPFH1_MOUSE | 346 | 38937 | 7.2 | (Q91X78) SPFH domain-containing protein 1 precursor (KE04 protein homolog) | 0.0001773 | 8.1944E-05 | 46% | A, B, C |
| Q9Z0S1 | BPNT1_MOUSE | 308 | 33196 | 5.8 | (Q9Z0S1) 3'(2'),5'-bisphosphate nucleotidase 1 (EC 3.1.3.7) (Bisphosphate 3'-nucleotidase 1) (PAP-inositol-1,4-phosphatase) (PIP) | 0.00017633 | 0.00012482 | 71% | A, B, C |
| Q922Q8 | LRC59_MOUSE | 307 | 34877 | 9.5 | (Q922Q8) Leucine-rich repeat-containing protein 59 | 0.00017583 | 4.6177E-05 | 26% | A, B, C |
| P26638 | SYS_MOUSE | 511 | 58258 | 6.3 | (P26638) Seryl-tRNA synthetase (EC 6.1.1.11) (Serine--tRNA ligase) (SerRS) | 0.00017572 | 2.7222E-05 | 15% | A, B, C |
| P97855 | G3BP_MOUSE | 465 | 51829 | 5.6 | (P97855) Ras-GTPase-activating protein-binding protein 1 (EC 3.6.1.-) (ATP-dependent DNA helicase VIII) (GAP SH3-domain-binding protein 1) (G3BP-1) (HDH-VIII) | 0.00017427 | 7.2975E-05 | 42% | A, B, C |
| Q91ZJ5 | UGPA2_MOUSE | 507 | 56848 | 7.6 | (Q91ZJ5) UTP--glucose-1-phosphate uridylyltransferase 2 (EC 2.7.7.9) (UDP-glucose pyrophosphorylase 2) (UDPGP 2) (UGPase 2) | 0.00017387 | 6.7003E-05 | 39% | A, B, C |
| Q9CY64 | BIEA_MOUSE | 295 | 33525 | 7 | (Q9CY64) Biliverdin reductase A precursor (EC 1.3.1.24) (Biliverdin-IX alpha-reductase) (BVR A) | 0.00017384 | 4.091E-05 | 24% | A, B, C |
| O08553 | DPYL2_MOUSE | 572 | 62278 | 6.4 | (O08553) Dihydropyrimidinase-related protein 2 (DRP-2) (ULIP 2 protein) | 0.00017309 | 4.169E-05 | 24% | A, B, C |

| | | | | | | | | | |
|---|---|---|---|---|---|---|---|---|---|
| P27601 | GNA13_MOUSE | 377 | 44055 | 8.2 | (P27601) Guanine nucleotide-binding protein alpha-13 subunit (G alpha-13) | 0.0001728 | 6.4045E-05 | 37% | A, B, C |
| Q91Z38 | TTC1_MOUSE | 292 | 33263 | 5 | (Q91Z38) Tetratricopeptide repeat protein 1 (TPR repeat protein 1) | 0.00017261 | 3.2095E-05 | 19% | A, B, C |
| Q9CYR6 | AGM1_MOUSE | 542 | 59453 | 6.2 | (Q9CYR6) Phosphoacetylglucosamine mutase (EC 5.4.2.3) (PAGM) (Acetylglucosamine phosphomutase) (N-acetylglucosamine-phosphate mutase) (Phosphoglucomutase 3) | 0.00017247 | 0.00012674 | 73% | A, B, C |
| P97384 | ANX11_MOUSE | 503 | 54111 | 7.7 | (P97384) Annexin A11 (Annexin XI) (Calcyclin-associated annexin 50) (CAP-50) | 0.0001696 | 3.549E-05 | 21% | A, B, C |
| P61924 | COPZ1_MOUSE | 177 | 20198 | 4.8 | (P61924) Coatomer subunit zeta-1 (Zeta-1 coat protein) (Zeta-1 COP) | 0.00016895 | 0.00010382 | 61% | A, B, C |
| Q9D8U8 | SNX5_MOUSE | 404 | 46797 | 6.6 | (Q9D8U8) Sorting nexin-5 | 0.00016884 | 0.00010358 | 61% | A, B, C |
| Q04857 | CO6A1_MOUSE | 1025 | 108489 | 5.4 | (Q04857) Collagen alpha-1(VI) chain precursor | 0.00016704 | 4.4593E-05 | 27% | A, B, C |
| P10637 | TAU_MOUSE | 732 | 76112 | 6.8 | (P10637) Microtubule-associated protein tau (Neurofibrillary tangle protein) (Paired helical filament-tau) (PHF-tau) | 0.00016698 | 0.00010211 | 61% | A, B, C |
| Q9CYH2 | CJ058_MOUSE | 218 | 24395 | 9.2 | (Q9CYH2) Protein C10orf58 homolog precursor | 0.00016596 | 1.0963E-05 | 7% | A, B, C |
| P43274 | H14_MOUSE | 218 | 21846 | 11.1 | (P43274) Histone H1.4 (H1 VAR.2) (H1e) | 0.00016489 | 3.1514E-05 | 19% | A, B, C |
| Q8VE37 | RCC1_MOUSE | 421 | 44931 | 8.1 | (Q8VE37) Regulator of chromosome condensation (Chromosome condensation protein 1) | 0.00016471 | 0.00013379 | 81% | A, B, C |
| O08638 | MYH11_MOUSE | 1972 | 227026 | 5.5 | (O08638) Myosin-11 (Myosin heavy chain, smooth muscle isoform) (SMMHC) | 0.00016436 | 1.6091E-05 | 10% | A, B, C |
| Q7TNE1 | CG010_MOUSE | 436 | 47674 | 8.7 | (Q7TNE1) Protein C7orf10 homolog | 0.00016338 | 8.2965E-05 | 51% | A, B, C |
| Q91YR1 | TWF1_MOUSE | 350 | 40079 | 6.7 | (Q91YR1) Twinfilin-1 (Protein A6) | 0.00016308 | 6.1222E-05 | 38% | A, B, C |
| Q9WV92 | E41L3_MOUSE | 929 | 103338 | 5.3 | (Q9WV92) Band 4.1-like protein 3 (4.1B) (Differentially expressed in adenocarcinoma of the lung protein 1) (DAL-1) (DAL1P) (mDAL-1) | 0.00016279 | 4.461E-05 | 27% | A, B, C |
| Q9DC70 | NUKM_MOUSE | 224 | 24683 | 9.9 | (Q9DC70) NADH-ubiquinone oxidoreductase 20 kDa subunit, mitochondrial precursor (EC 1.6.5.3) (EC 1.6.99.3) (Complex I-20KD) (CI-20KD) (PSST subunit) | 0.00016234 | 6.0041E-05 | 37% | A, B, C |
| P06801 | MAOX_MOUSE | 572 | 63999 | 7.4 | (P06801) NADP-dependent malic enzyme (EC 1.1.1.40) (NADP-ME) (Malic enzyme 1) | 0.00016217 | 0.00011622 | 72% | A, B, C |
| O35593 | PSDE_MOUSE | 310 | 34577 | 6.5 | (O35593) 26S proteasome non-ATPase regulatory subunit 14 (26S proteasome regulatory subunit rpn11) (MAD1) | 0.00016149 | 0.00010535 | 65% | A, B, C |
| Q9WU78 | PDC6I_MOUSE | 869 | 96010 | 6.5 | (Q9WU78) Programmed cell death 6-interacting protein (ALG-2-interacting protein X) (ALG-2-interacting protein 1) (E2F1-inducible protein) (Eig2) | 0.00016029 | 2.2804E-05 | 14% | A, B, C |
| O88544 | CSN4_MOUSE | 406 | 46285 | 5.8 | (O88544) COP9 signalosome complex subunit 4 (Signalosome subunit 4) (SGN4) (JAB1-containing signalosome subunit 4) | 0.00015969 | 8.5842E-05 | 54% | A, B, C |
| P35486 | ODPA_MOUSE | 390 | 43232 | 8.2 | (P35486) Pyruvate dehydrogenase E1 component alpha subunit, somatic form, mitochondrial precursor (EC 1.2.4.1) (PDHE1-A type I) | 0.00015965 | 5.2771E-05 | 33% | A, B, C |
| Q9JHJ0 | TMOD3_MOUSE | 352 | 39503 | 5.1 | (Q9JHJ0) Tropomodulin-3 (Ubiquitous tropomodulin) (U-Tmod) | 0.00015913 | 8.6986E-05 | 55% | A, B, C |
| O55135 | IF6_MOUSE | 245 | 26511 | 4.7 | (O55135) Eukaryotic translation initiation factor 6 (eIF-6) (B4 integrin interactor) (CAB) (p27(BBP)) | 0.0001588 | 0.00011148 | 70% | A, B, C |
| Q64314 | CD34_MOUSE | 382 | 40983 | 5.3 | (Q64314) Hematopoietic progenitor cell antigen CD34 precursor | 0.00015702 | 0.0001141 | 73% | A, B, C |
| Q99JY9 | ARP3_MOUSE | 417 | 47226 | 5.9 | (Q99JY9) Actin-like protein 3 (Actin-related protein 3) | 0.00015541 | 2.7627E-05 | 18% | A, B, C |

| | | | | | | | | | |
|---|---|---|---|---|---|---|---|---|---|
| Q91VR5 | DDX1_MOUSE | 740 | 82500 | 7.2 | (Q91VR5) ATP-dependent RNA helicase DDX1 (EC 3.6.1.-) (DEAD box protein 1) | 0.0001551 | 6.7459E-05 | 43% | A, B, C |
| P26231 | CTN1_MOUSE | 906 | 100106 | 6.2 | (P26231) Alpha-1 catenin (102 kDa cadherin-associated protein) (CAP102) (Alpha E-catenin) | 0.00015497 | 2.401E-05 | 15% | A, B, C |
| Q03734 | SPA3M_MOUSE | 418 | 47004 | 5.5 | (Q03734) Serine protease inhibitor A3M precursor (Serpin A3M) | 0.00015406 | 0.00016183 | 105% | A, B, C |
| Q9WVE8 | PACN2_MOUSE | 486 | 55833 | 5.2 | (Q9WVE8) Protein kinase C and casein kinase substrate in neurons protein 2 | 0.00015371 | 6.8795E-05 | 45% | A, B, C |
| O09111 | NDUBB_MOUSE | 151 | 17444 | 5.2 | (O09111) NADH dehydrogenase [ubiquinone] 1 beta subcomplex subunit 11, mitochondrial precursor (EC 1.6.5.3) (EC 1.6.99.3) (NADH-ubiquinone oxidoreductase ESSS subunit) (Complex I-ESSS) (CI-ESSS) (Neuronal protein 15.6) (p15.6) (Np15.6) | 0.0001535 | 5.3955E-05 | 35% | A, B, C |
| Q921F2 | TADBP_MOUSE | 414 | 44548 | 6.7 | (Q921F2) TAR DNA-binding protein 43 (TDP-43) | 0.00015274 | 0.00011797 | 77% | A, B, C |
| Q99PL5 | RRBP1_MOUSE | 1605 | 172878 | 9.3 | (Q99PL5) Ribosome-binding protein 1 (Ribosome receptor protein) (mRRp) | 0.00015242 | 2.2458E-05 | 15% | A, B, C |
| Q80WJ7 | LYRIC_MOUSE | 579 | 63846 | 9.3 | (Q80WJ7) Protein LYRIC (Lysine-rich CEACAM1 co-isolated protein) (3D3/LYRIC) (Metastasis adhesion protein) (Metadherin) | 0.000152 | 1.5587E-05 | 10% | A, B, C |
| P70333 | HNRH2_MOUSE | 449 | 49280 | 6.3 | (P70333) Heterogeneous nuclear ribonucleoprotein H' (hnRNP H') | 0.00015195 | 8.1309E-05 | 54% | A, B, C |
| Q8BHN3 | GANAB_MOUSE | 944 | 106911 | 6.1 | (Q8BHN3) Neutral alpha-glucosidase AB precursor (EC 3.2.1.84) (Glucosidase II subunit alpha) (Alpha glucosidase 2) | 0.00015159 | 3.0101E-05 | 20% | A, B, C |
| P06797 | CATL_MOUSE | 334 | 37547 | 6.8 | (P06797) Cathepsin L precursor (EC 3.4.22.15) (Major excreted protein) (MEP) (p39 cysteine proteinase) [Contains: Cathepsin L heavy chain; Cathepsin L light chain] | 0.00015156 | 2.7899E-05 | 18% | A, B, C |
| P13020 | GELS_MOUSE | 780 | 85942 | 6.2 | (P13020) Gelsolin precursor (Actin-depolymerizing factor) (ADF) (Brevin) | 0.00015147 | 0.00016756 | 111% | A, B, C |
| Q61792 | LASP1_MOUSE | 263 | 29994 | 7 | (Q61792) LIM and SH3 domain protein 1 (LASP-1) (MLN 50) | 0.00015127 | 6.3653E-05 | 42% | A, B, C |
| Q8R1U2 | CGRE1_MOUSE | 281 | 30847 | 4.3 | (Q8R1U2) Cell growth regulator with EF hand domain 1 (Cell growth regulatory gene 11 protein) | 0.00015121 | 5.8875E-05 | 39% | A, B, C |
| O88986 | KBL_MOUSE | 416 | 44931 | 7.3 | (O88986) 2-amino-3-ketobutyrate coenzyme A ligase, mitochondrial precursor (EC 2.3.1.29) (AKB ligase) (Glycine acetyltransferase) | 0.00015104 | 5.1643E-05 | 34% | A, B, C |
| Q9CQC9 | SAR1B_MOUSE | 198 | 22382 | 6.1 | (Q9CQC9) GTP-binding protein SAR1b | 0.000151 | 0.0001249 | 83% | A, B, C |
| Q9QYB5 | ADDG_MOUSE | 706 | 78763 | 5.9 | (Q9QYB5) Gamma-adducin (Adducin-like protein 70) | 0.00015013 | 3.4548E-05 | 23% | A, B, C |
| Q9QXY6 | EHD3_MOUSE | 535 | 60869 | 6.4 | (Q9QXY6) EH-domain-containing protein 3 | 0.00014604 | 8.3757E-05 | 57% | A, B, C |
| Q9CZW5 | TOM70_MOUSE | 611 | 67521 | 7.3 | (Q9CZW5) Mitochondrial precursor proteins import receptor (Translocase of outer membrane TOM70) | 0.00014588 | 6.0065E-05 | 41% | A, B, C |
| Q9QYB1 | CLIC4_MOUSE | 252 | 28598 | 5.6 | (Q9QYB1) Chloride intracellular channel protein 4 (mc3s5/mtCLIC) | 0.00014574 | 3.112E-05 | 21% | A, B, C |
| Q9R0X4 | ACOT9_MOUSE | 439 | 50560 | 8.6 | (Q9R0X4) Acyl-coenzyme A thioesterase 9, mitochondrial precursor (EC 3.1.2.-) (Acyl-CoA thioesterase 9) (Acyl-CoA thioester hydrolase 9) (Acyl coenzyme A thioester hydrolase 2) (48 kDa acyl-CoA thioester hydrolase) (p48) (Mt-ACT48) (Protein U | 0.00014472 | 1.2319E-05 | 9% | A, B, C |
| P62830 | RL23_MOUSE | 140 | 14865 | 10.5 | (P62830) 60S ribosomal protein L23 | 0.00014459 | 5.6784E-05 | 39% | A, B, C |
| Q9WUM3 | COR1B_MOUSE | 484 | 53912 | 5.8 | (Q9WUM3) Coronin-1B (Coronin-2) | 0.00014455 | 8.8898E-05 | 62% | A, B, C |

| | | | | | | | | | |
|---|---|---|---|---|---|---|---|---|---|
| Q9DB05 | SNAA_MOUSE | 295 | 33190 | 5.4 | (Q9DB05) Alpha-soluble NSF attachment protein (SNAP-alpha) (N-ethylmaleimide-sensitive factor attachment protein, alpha) | 0.00014423 | 6.7369E-05 | 47% | A, B, C |
| P53986 | MOT1_MOUSE | 493 | 53267 | 7.5 | (P53986) Monocarboxylate transporter 1 (MCT 1) | 0.0001432 | 7.5605E-05 | 53% | A, B, C |
| Q64521 | GPDM_MOUSE | 727 | 80900 | 6.6 | (Q64521) Glycerol-3-phosphate dehydrogenase, mitochondrial precursor (EC 1.1.99.5) (GPD-M) (GPDH-M) | 0.00014216 | 4.553E-05 | 32% | A, B, C |
| Q9EPC1 | PARVA_MOUSE | 372 | 42330 | 5.9 | (Q9EPC1) Alpha-parvin (Actopaxin) | 0.00014213 | 0.00010413 | 73% | A, B, C |
| P56399 | UBP5_MOUSE | 858 | 95833 | 5 | (P56399) Ubiquitin carboxyl-terminal hydrolase 5 (EC 3.1.2.15) (Ubiquitin thioesterase 5) (Ubiquitin-specific-processing protease 5) (Deubiquitinating enzyme 5) (Isopeptidase T) | 0.00014197 | 4.8767E-05 | 34% | A, B, C |
| Q99LD8 | DDAH2_MOUSE | 285 | 29646 | 6 | (Q99LD8) NG,NG-dimethylarginine dimethylaminohydrolase 2 (EC 3.5.3.18) (Dimethylargininase-2) (Dimethylarginine dimethylaminohydrolase 2) (DDAHII) | 0.00014181 | 5.2393E-05 | 37% | A, B, C |
| Q64213 | SF01_MOUSE | 652 | 70277 | 8.8 | (Q64213) Splicing factor 1 (Zinc finger protein 162) (Transcription factor ZFM1) (mZFM) (Zinc finger gene in MEN1 locus) (Mammalian branch point-binding protein mBBP) (BBP) (CW17) | 0.0001408 | 1.6113E-05 | 11% | A, B, C |
| O35737 | HNRH1_MOUSE | 448 | 49068 | 6.3 | (O35737) Heterogeneous nuclear ribonucleoprotein H (hnRNP H) | 0.00014032 | 0.00013639 | 97% | A, B, C |
| P46471 | PRS7_MOUSE | 432 | 48517 | 5.9 | (P46471) 26S protease regulatory subunit 7 (Protein MSS1) | 0.00013956 | 2.4375E-05 | 17% | A, B, C |
| P26645 | MARCS_MOUSE | 308 | 29530 | 4.3 | (P26645) Myristoylated alanine-rich C-kinase substrate (MARCKS) | 0.00013853 | 4.4216E-05 | 32% | A, B, C |
| P10107 | ANXA1_MOUSE | 345 | 38603 | 7.4 | (P10107) Annexin A1 (Annexin I) (Lipocortin I) (Calpactin II) (Chromobindin-9) (p35) (Phospholipase A2 inhibitory protein) | 0.00013839 | 8.6302E-05 | 62% | A, B, C |
| Q9QZD8 | DIC_MOUSE | 287 | 31715 | 9.3 | (Q9QZD8) Mitochondrial dicarboxylate carrier (Solute carrier family 25 member 10) | 0.00013811 | 0.00010367 | 75% | A, B, C |
| Q80VD1 | FA98B_MOUSE | 429 | 45349 | 8.5 | (Q80VD1) Protein FAM98B | 0.00013765 | 5.5879E-05 | 41% | A, B, C |
| Q8BP40 | PPA6_MOUSE | 418 | 47625 | 7.7 | (Q8BP40) Lysophosphatidic acid phosphatase type 6 precursor (EC 3.1.3.2) (Acid phosphatase 6, lysophosphatidic) (Acid phosphatase-like protein 1) (PACPL1) | 0.00013692 | 6.8549E-05 | 50% | A, B, C |
| Q61735 | CD47_MOUSE | 303 | 33098 | 8.6 | (Q61735) Leukocyte surface antigen CD47 precursor (Integrin-associated protein) (IAP) | 0.00013616 | 0.0001041 | 76% | A, B, C |
| Q7TMK9 | HNRPQ_MOUSE | 623 | 69633 | 8.6 | (Q7TMK9) Heterogeneous nuclear ribonucleoprotein Q (hnRNP Q) (hnRNP-Q) (Synaptotagmin-binding, cytoplasmic RNA-interacting protein) (Glycine- and tyrosine-rich RNA-binding protein) (GRY-RBP) (NS1-associated protein 1) (pp68) | 0.00013581 | 2.2038E-05 | 16% | A, B, C |
| P60843 | IF4A1_MOUSE | 406 | 46154 | 5.5 | (P60843) Eukaryotic initiation factor 4A-I (EC 3.6.1.-) (ATP-dependent RNA helicase eIF4A-1) (eIF4A-I) (eIF-4A-I) | 0.00013572 | 5.779E-05 | 43% | A, B, C |
| Q8VE47 | UE1D1_MOUSE | 403 | 44786 | 5 | (Q8VE47) Ubiquitin-activating enzyme E1 domain-containing protein 1 (UFM1-activating enzyme) | 0.0001353 | 1.873E-05 | 14% | A, B, C |
| Q62417 | SRBS1_MOUSE | 1290 | 143070 | 8.2 | (Q62417) Sorbin and SH3 domain-containing protein 1 (Ponsin) (c-Cbl-associated protein) (CAP) (SH3 domain protein 5) (SH3P12) | 0.00013501 | 4.1911E-05 | 31% | A, B, C |
| P01837 | KAC_MOUSE | 106 | 11778 | 5.4 | (P01837) Ig kappa chain C region | 0.00013461 | 9.6299E-05 | 72% | A, B, C |

| | | | | | | | | | |
|---|---|---|---|---|---|---|---|---|---|
| Q64191 | ASPG_MOUSE | 346 | 37022 | 6.4 | (Q64191) N(4)-(beta-N-acetylglucosaminyl)-L-asparaginase precursor (EC 3.5.1.26) (Glycosylasparaginase) (Aspartylglucosaminidase) (N4-(N-acetyl-beta-glucosaminyl)-L-asparagine amidase) (AGA) [Contains: Glycosylasparaginase alpha chain; Glycosy | 0.00013418 | 3.1497E-05 | 23% | A, B, C |
| Q60865 | GP137_MOUSE | 656 | 73548 | 5.4 | (Q60865) GPI-anchored protein p137 (p137GPI) | 0.00013388 | 1.5434E-05 | 12% | A, B, C |
| Q9CR57 | RL14_MOUSE | 216 | 23433 | 11 | (Q9CR57) 60S ribosomal protein L14 | 0.00013387 | 4.3956E-05 | 33% | A, B, C |
| Q91YJ2 | SNX4_MOUSE | 450 | 51778 | 5.8 | (Q91YJ2) Sorting nexin-4 | 0.00013276 | 2.3812E-05 | 18% | A, B, C |
| Q02248 | CTNB1_MOUSE | 781 | 85471 | 5.9 | (Q02248) Beta-catenin | 0.00013222 | 6.1769E-05 | 47% | A, B, C |
| Q8VDM4 | PSD2_MOUSE | 908 | 100203 | 5.2 | (Q8VDM4) 26S proteasome non-ATPase regulatory subunit 2 (26S proteasome regulatory subunit RPN1) (26S proteasome regulatory subunit S2) (26S proteasome subunit p97) | 0.00013212 | 9.0151E-05 | 68% | A, B, C |
| Q9DBL1 | ACDSB_MOUSE | 432 | 47874 | 7.9 | (Q9DBL1) Short/branched chain specific acyl-CoA dehydrogenase, mitochondrial precursor (EC 1.3.99.-) (SBCAD) (2-methyl branched chain acyl-CoA dehydrogenase) (2-MEBCAD) (2-methylbutyryl-coenzyme A dehydrogenase) (2-methylbutyryl-CoA dehydroge | 0.00013162 | 2.6693E-05 | 20% | A, B, C |
| Q91YQ5 | RIB1_MOUSE | 608 | 68528 | 6.5 | (Q91YQ5) Dolichyl-diphosphooligosaccharide--protein glycosyltransferase 67 kDa subunit precursor (EC 2.4.1.119) (Ribophorin I) (RPN-I) | 0.00013162 | 9.7521E-05 | 74% | A, B, C |
| Q9D892 | ITPA_MOUSE | 198 | 21897 | 5.9 | (Q9D892) Inosine triphosphate pyrophosphatase (EC 3.6.1.19) (ITPase) (Inosine triphosphatase) | 0.00013096 | 5.3888E-05 | 41% | A, B, C |
| P11984 | TCPA1_MOUSE | 556 | 60341 | 6.1 | (P11984) T-complex protein 1 subunit alpha A (TCP-1-alpha) (CCT-alpha) (Tailless complex polypeptide 1A) (TCP-1-A) | 0.00013079 | 2.4687E-05 | 19% | A, B, C |
| Q9JHW4 | SELB_MOUSE | 583 | 63417 | 8.3 | (Q9JHW4) Selenocysteine-specific elongation factor (Elongation factor sec) (Eukaryotic elongation factor, selenocysteine-tRNA-specific) (mSelB) | 0.00013038 | 0.00013295 | 102% | A, B, C |
| Q9JHS4 | CLPX_MOUSE | 634 | 69314 | 7.9 | (Q9JHS4) ATP-dependent Clp protease ATP-binding subunit ClpX-like, mitochondrial precursor | 0.00013035 | 5.7173E-05 | 44% | A, B, C |
| Q9JMH6 | TRXR1_MOUSE | 499 | 54497 | 6.3 | (Q9JMH6) Thioredoxin reductase 1, cytoplasmic (EC 1.8.1.9) (TR) (TR1) | 0.00012987 | 2.3087E-05 | 18% | A, B, C |
| Q99LP6 | GRPE1_MOUSE | 217 | 24307 | 8.4 | (Q99LP6) GrpE protein homolog 1, mitochondrial precursor (Mt-GrpE#1) | 0.00012975 | 4.932E-05 | 38% | A, B, C |
| Q9CY58 | PAIRB_MOUSE | 407 | 44714 | 8.5 | (Q9CY58) Plasminogen activator inhibitor 1 RNA-binding protein (PAI1 RNA-binding protein 1) (PAI-RBP1) (SERPINE1 mRNA-binding protein 1) | 0.00012912 | 2.0042E-05 | 16% | A, B, C |
| P62196 | PRS8_MOUSE | 406 | 45626 | 7.5 | (P62196) 26S protease regulatory subunit 8 (Proteasome subunit p45) (p45/SUG) (Proteasome 26S subunit ATPase 5) (mSUG1) | 0.0001291 | 9.0916E-05 | 70% | A, B, C |
| Q6NVF9 | CPSF6_MOUSE | 551 | 59153 | 7.2 | (Q6NVF9) Cleavage and polyadenylation specificity factor 6 | 0.00012905 | 5.928E-05 | 46% | A, B, C |
| Q8BKX1 | BAIP2_MOUSE | 535 | 59237 | 9 | (Q8BKX1) Brain-specific angiogenesis inhibitor 1-associated protein 2 (BAI1-associated protein 2) (BAI-associated protein 2) (Insulin receptor substrate p53) (IRSp53) (Insulin receptor substrate protein of 53 kDa) (Insulin receptor tyrosine k | 0.00012878 | 6.0242E-05 | 47% | A, B, C |
| O89079 | COPE_MOUSE | 307 | 34436 | 5.1 | (O89079) Coatomer subunit epsilon (Epsilon-coat protein) (Epsilon-COP) | 0.00012851 | 0.00010011 | 78% | A, B, C |
| Q921I1 | TRFE_MOUSE | 697 | 76724 | 7.2 | (Q921I1) Serotransferrin precursor (Transferrin) (Siderophilin) (Beta-1-metal-binding globulin) | 0.00012815 | 6.9127E-05 | 54% | A, B, C |
| Q6ZWV3 | RL10_MOUSE | 213 | 24473 | 10.1 | (Q6ZWV3) 60S ribosomal protein L10 (QM protein homolog) | 0.00012681 | 3.6034E-05 | 28% | A, B, C |

| | | | | | | | | | |
|---|---|---|---|---|---|---|---|---|---|
| Q7TMS5 | ABCG2_MOUSE | 657 | 72978 | 8.5 | (Q7TMS5) ATP-binding cassette sub-family G member 2 (Breast cancer resistance protein 1 homolog) | 0.00012656 | 7.5269E-06 | 6% | A, B, C |
| P51174 | ACADL_MOUSE | 430 | 47908 | 8.3 | (P51174) Long-chain specific acyl-CoA dehydrogenase, mitochondrial precursor (EC 1.3.99.13) (LCAD) | 0.00012553 | 3.2968E-05 | 26% | A, B, C |
| Q7TNC4 | LC7L2_MOUSE | 392 | 46583 | 10.1 | (Q7TNC4) Putative RNA-binding protein Luc7-like 2 (CGI-74 homolog) | 0.00012539 | 6.3405E-05 | 51% | A, B, C |
| Q9JKB1 | UCHL3_MOUSE | 230 | 26152 | 5 | (Q9JKB1) Ubiquitin carboxyl-terminal hydrolase isozyme L3 (EC 3.4.19.12) (UCH-L3) (Ubiquitin thioesterase L3) | 0.00012527 | 6.3545E-05 | 51% | A, B, C |
| Q8VIM4 | BSND_MOUSE | 307 | 33814 | 4.6 | (Q8VIM4) Barttin | 0.00012498 | 0.00010126 | 81% | A, B, C |
| P14148 | RL7_MOUSE | 270 | 31420 | 10.9 | (P14148) 60S ribosomal protein L7 | 0.00012469 | 0.00010154 | 81% | A, B, C |
| P84084 | ARF5_MOUSE | 179 | 20398 | 6.8 | (P84084) ADP-ribosylation factor 5 | 0.00012417 | 1.8325E-05 | 15% | A, B, C |
| Q924T2 | RT02_MOUSE | 291 | 32313 | 9.1 | (Q924T2) Mitochondrial 28S ribosomal protein S2 (S2mt) (MRP-S2) | 0.00012308 | 2.4235E-05 | 20% | A, B, C |
| P58281 | OPA1_MOUSE | 960 | 111339 | 7.5 | (P58281) Dynamin-like 120 kDa protein, mitochondrial precursor (Large GTP-binding protein) (LargeG) | 0.00012296 | 5.4416E-05 | 44% | A, B, C |
| Q60967 | PAPS1_MOUSE | 624 | 70794 | 6.8 | (Q60967) Bifunctional 3'-phosphoadenosine 5'-phosphosulfate synthetase 1 (PAPS synthetase 1) (PAPSS 1) (Sulfurylase kinase 1) (SK1) (SK 1) [Includes: Sulfate adenylyltransferase (EC 2.7.7.4) (Sulfate adenylate transferase) (SAT) (ATP-sulfuryl | 0.00012266 | 6.5534E-05 | 53% | A, B, C |
| P00375 | DYR_MOUSE | 186 | 21475 | 8.6 | (P00375) Dihydrofolate reductase (EC 1.5.1.3) | 0.00012244 | 5.7107E-05 | 47% | A, B, C |
| P36552 | HEM6_MOUSE | 443 | 49715 | 8.5 | (P36552) Coproporphyrinogen III oxidase, mitochondrial precursor (EC 1.3.3.3) (Coproporphyrinogenase) (Coprogen oxidase) (COX) | 0.00012233 | 8.1026E-05 | 66% | A, B, C |
| Q64331 | MYO6_MOUSE | 1265 | 146409 | 8.8 | (Q64331) Myosin-6 (Myosin VI) | 0.00012142 | 7.8598E-05 | 65% | A, B, C |
| O88643 | PAK1_MOUSE | 545 | 60737 | 5.7 | (O88643) Serine/threonine-protein kinase PAK 1 (EC 2.7.11.1) (p21-activated kinase 1) (PAK-1) (P65-PAK) (Alpha-PAK) (CDC42/RAC effector kinase PAK-A) | 0.0001209 | 6.8297E-05 | 56% | A, B, C |
| Q61029 | LAP2B_MOUSE | 451 | 50163 | 9.4 | (Q61029) Lamina-associated polypeptide 2 isoforms beta/delta/epsilon/gamma (Thymopoietin isoforms beta/delta/epsilon/gamma) (TP beta/delta/epsilon/gamma) | 0.00012049 | 4.0074E-05 | 33% | A, B, C |
| P21619 | LMNB2_MOUSE | 596 | 67318 | 5.5 | (P21619) Lamin-B2 | 0.00011993 | 3.3629E-05 | 28% | A, B, C |
| P70404 | IDH3G_MOUSE | 393 | 42785 | 9 | (P70404) Isocitrate dehydrogenase [NAD] subunit gamma, mitochondrial precursor (EC 1.1.1.41) (Isocitric dehydrogenase) (NAD(+)-specific ICDH) | 0.00011916 | 8.1282E-05 | 68% | A, B, C |
| P43277 | H13_MOUSE | 220 | 21968 | 11 | (P43277) Histone H1.3 (H1 VAR.4) (H1d) | 0.00011915 | 4.9618E-05 | 42% | A, B, C |
| P04228 | HA2D_MOUSE | 256 | 28243 | 5 | (P04228) H-2 class II histocompatibility antigen, A-D alpha chain precursor | 0.00011846 | 4.6573E-05 | 39% | A, B, C |
| P07759 | SPA3K_MOUSE | 418 | 46880 | 5.2 | (P07759) Serine protease inhibitor A3K precursor (Contrapsin) (Serpin A3K) | 0.00011796 | 1.5375E-05 | 13% | A, B, C |
| Q8K0D5 | EFG1_MOUSE | 751 | 83550 | 6.9 | (Q8K0D5) Elongation factor G 1, mitochondrial precursor (mEF-G 1) (Elongation factor G1) | 0.00011675 | 3.2865E-05 | 28% | A, B, C |
| Q9Z2H5 | E41L1_MOUSE | 879 | 98315 | 5.7 | (Q9Z2H5) Band 4.1-like protein 1 (Neuronal protein 4.1) (4.1N) | 0.00011674 | 4.2865E-05 | 37% | A, B, C |
| P40336 | VPS26_MOUSE | 327 | 38114 | 6.5 | (P40336) Vacuolar protein sorting 26 (Vesicle protein sorting 26) (H<beta 58 protein) (H beta 58) | 0.00011664 | 3.5479E-05 | 30% | A, B, C |
| P47753 | CAZA1_MOUSE | 285 | 32809 | 5.6 | (P47753) F-actin capping protein alpha-1 subunit (CapZ alpha-1) | 0.00011631 | 7.5014E-05 | 64% | A, B, C |
| Q9EQK5 | MVP_MOUSE | 860 | 95821 | 5.6 | (Q9EQK5) Major vault protein (MVP) | 0.00011526 | 6.0911E-05 | 53% | A, B, C |

| | | | | | | | | | |
|---|---|---|---|---|---|---|---|---|---|
| P09055 | ITB1_MOUSE | 798 | 88231 | 5.9 | (P09055) Integrin beta-1 precursor (Fibronectin receptor subunit beta) (Integrin VLA-4 subunit beta) (CD29 antigen) | 0.00011524 | 7.923E-05 | 69% | A, B, C |
| Q68EF0 | RAB3I_MOUSE | 428 | 47134 | 6.7 | (Q68EF0) RAB3A-interacting protein (Rabin-3) (SSX2-interacting protein) | 0.00011488 | 8.8372E-05 | 77% | A, B, C |
| Q6WVG3 | KCD12_MOUSE | 327 | 35892 | 5.8 | (Q6WVG3) Potassium channel tetramerization domain-containing protein 12 (Pfetin) (Predominantly fetal expressed T1 domain) | 0.00011487 | 5.5218E-05 | 48% | A, B, C |
| Q60598 | SRC8_MOUSE | 546 | 61260 | 5.4 | (Q60598) Src substrate cortactin | 0.0001147 | 2.2168E-05 | 19% | A, B, C |
| P09925 | SURF1_MOUSE | 306 | 34798 | 9.7 | (P09925) Surfeit locus protein 1 | 0.00011408 | 4.3754E-05 | 38% | A, B, C |
| Q61847 | MEP1B_MOUSE | 704 | 79549 | 5.9 | (Q61847) Meprin A subunit beta precursor (EC 3.4.24.18) (Endopeptidase-2) | 0.00011377 | 1.9531E-05 | 17% | A, B, C |
| Q923I7 | SC5A2_MOUSE | 670 | 73008 | 7.7 | (Q923I7) Sodium/glucose cotransporter 2 (Na(+)/glucose cotransporter 2) (Low affinity sodium-glucose cotransporter) | 0.00011284 | 1.5504E-05 | 14% | A, B, C |
| Q62422 | OSTF1_MOUSE | 215 | 23783 | 5.7 | (Q62422) Osteoclast-stimulating factor 1 (SH3 domain protein 3) | 0.00011205 | 4.4335E-05 | 40% | A, B, C |
| P97742 | CPT1A_MOUSE | 773 | 88252 | 8.6 | (P97742) Carnitine O-palmitoyltransferase I, liver isoform (EC 2.3.1.21) (CPT I) (CPTI-L) (Carnitine palmitoyltransferase 1A) | 0.000112 | 4.2279E-05 | 38% | A, B, C |
| Q8VDP4 | K1967_MOUSE | 922 | 103002 | 5.3 | (Q8VDP4) Protein KIAA1967 homolog | 0.00011178 | 0.00013331 | 119% | A, B, C |
| Q501J6 | DDX17_MOUSE | 650 | 72400 | 8.6 | (Q501J6) Probable ATP-dependent RNA helicase DDX17 (EC 3.6.1.-) (DEAD box protein 17) | 0.00011156 | 3.3374E-05 | 30% | A, B, C |
| Q99L45 | IF2B_MOUSE | 331 | 38092 | 5.8 | (Q99L45) Eukaryotic translation initiation factor 2 subunit 2 (Eukaryotic translation initiation factor 2 subunit beta) (eIF-2-beta) | 0.00011095 | 2.3692E-05 | 21% | A, B, C |
| O70194 | IF37_MOUSE | 547 | 63558 | 6 | (O70194) Eukaryotic translation initiation factor 3 subunit 7 (eIF-3 zeta) (eIF3 p66) (eIF3d) | 0.00011054 | 2.7342E-05 | 25% | A, B, C |
| P46460 | NSF_MOUSE | 744 | 82565 | 6.9 | (P46460) Vesicle-fusing ATPase (EC 3.6.4.6) (Vesicular-fusion protein NSF) (N-ethylmaleimide sensitive fusion protein) (NEM-sensitive fusion protein) (SKD2 protein) | 0.00011035 | 3.8202E-05 | 35% | A, B, C |
| Q9EQH3 | VPS35_MOUSE | 796 | 91713 | 5.4 | (Q9EQH3) Vacuolar protein sorting 35 (Vesicle protein sorting 35) (Maternal-embryonic 3) | 0.00010964 | 2.2551E-05 | 21% | A, B, C |
| Q8VBZ3 | CLPT1_MOUSE | 664 | 75291 | 6.3 | (Q8VBZ3) Cleft lip and palate transmembrane protein 1 homolog (Thymic epithelial cell surface antigen) | 0.00010952 | 2.0672E-05 | 19% | A, B, C |
| Q8CI51 | PDLI5_MOUSE | 590 | 63198 | 8.2 | (Q8CI51) PDZ and LIM domain protein 5 (Enigma homolog) (Enigma-like PDZ and LIM domains protein) | 0.00010777 | 9.4264E-05 | 87% | A, B, C |
| P23506 | PIMT_MOUSE | 226 | 24503 | 7.6 | (P23506) Protein-L-isoaspartate(D-aspartate) O-methyltransferase (EC 2.1.1.77) (Protein-beta-aspartate methyltransferase) (PIMT) (Protein L-isoaspartyl/D-aspartyl methyltransferase) (L-isoaspartyl protein carboxyl methyltransferase) | 0.00010764 | 6.1022E-05 | 57% | A, B, C |
| P70122 | SBDS_MOUSE | 249 | 28649 | 8.8 | (P70122) Shwachman-Bodian-Diamond syndrome protein homolog (Protein 22A3) | 0.00010735 | 7.6592E-05 | 71% | A, B, C |
| Q8K3G5 | VRK3_MOUSE | 453 | 50830 | 8.6 | (Q8K3G5) Serine/threonine-protein kinase VRK3 (EC 2.7.11.1) (Vaccinia-related kinase 3) | 0.0001073 | 5.8664E-05 | 55% | A, B, C |
| Q8VCH8 | UBXD2_MOUSE | 506 | 56472 | 6.6 | (Q8VCH8) UBX domain-containing protein 2 | 0.00010668 | 2.8017E-05 | 26% | A, B, C |
| O88746 | TOM1_MOUSE | 492 | 54325 | 4.9 | (O88746) Target of Myb protein 1 | 0.00010646 | 3.1296E-05 | 29% | A, B, C |
| P42208 | SEPT2_MOUSE | 361 | 41526 | 6.5 | (P42208) Septin-2 (Protein NEDD5) (Neural precursor cell expressed developmentally down-regulated protein 5) | 0.00010628 | 8.6115E-05 | 81% | A, B, C |
| Q8CIN4 | PAK2_MOUSE | 524 | 57930 | 5.8 | (Q8CIN4) Serine/threonine-protein kinase PAK 2 (EC 2.7.11.1) (p21-activated kinase 2) (PAK-2) | 0.00010623 | 5.5889E-06 | 5% | A, B, C |

| | | | | | | | | | |
|---|---|---|---|---|---|---|---|---|---|
| Q9DCX8 | IYD1_MOUSE | 285 | 32814 | 6.4 | (Q9DCX8) Iodotyrosine dehalogenase 1 precursor (EC 1.-.-.-) (IYD-1) | 0.0001061 | 4.2242E-05 | 40% | A, B, C |
| Q9DBF1 | AL7A1_MOUSE | 510 | 55514 | 6.4 | (Q9DBF1) Aldehyde dehydrogenase family 7 member A1 (EC 1.2.1.3) (Antiquitin-1) | 0.0001059 | 2.5837E-05 | 24% | A, B, C |
| P80316 | TCPE_MOUSE | 541 | 59624 | 6 | (P80316) T-complex protein 1 subunit epsilon (TCP-1-epsilon) (CCT-epsilon) | 0.00010415 | 1.9035E-05 | 18% | A, B, C |
| O35841 | API5_MOUSE | 504 | 56771 | 5.8 | (O35841) Apoptosis inhibitor 5 (API-5) (AAC-11) | 0.00010392 | 3.0551E-05 | 29% | A, B, C |
| O55143 | AT2A2_MOUSE | 1044 | 114858 | 5.3 | (O55143) Sarcoplasmic/endoplasmic reticulum calcium ATPase 2 (EC 3.6.3.8) (Calcium pump 2) (SERCA2) (SR Ca(2+)-ATPase 2) (Calcium-transporting ATPase sarcoplasmic reticulum type, slow twitch skeletal muscle isoform) (Endoplasmic reticulum cla | 0.00010316 | 5.9034E-05 | 57% | A, B, C |
| Q91W39 | NCOA5_MOUSE | 579 | 65319 | 9.8 | (Q91W39) Nuclear receptor coactivator 5 (NCoA-5) (Coactivator independent of AF-2) (CIA) | 0.00010293 | 5.4408E-05 | 53% | A, B, C |
| Q8C5W0 | CLMN_MOUSE | 1052 | 117227 | 5 | (Q8C5W0) Calmin | 0.00010044 | 2.6954E-05 | 27% | A, B, C |
| Q06890 | CLUS_MOUSE | 448 | 51656 | 5.7 | (Q06890) Clusterin precursor (Sulfated glycoprotein 2) (SGP-2) (Clustrin) (Apolipoprotein J) (Apo-J) [Contains: Clusterin beta chain; Clusterin alpha chain] | 0.00010006 | 3.0661E-05 | 31% | A, B, C |
| Q8K4K6 | PANK1_MOUSE | 548 | 60092 | 8 | (Q8K4K6) Pantothenate kinase 1 (EC 2.7.1.33) (Pantothenic acid kinase 1) (mPank1) (mPank) | 9.9932E-05 | 8.6823E-05 | 87% | A, B, C |
| Q8BL66 | EEA1_MOUSE | 1411 | 160914 | 5.8 | (Q8BL66) Early endosome antigen 1 | 9.9404E-05 | 3.02E-05 | 30% | A, B, C |
| Q9Z0V7 | TI17B_MOUSE | 172 | 18352 | 9 | (Q9Z0V7) Mitochondrial import inner membrane translocase subunit Tim17-B | 9.9333E-05 | 1.6758E-05 | 17% | A, B, C |
| Q3UM45 | PP1R7_MOUSE | 361 | 41292 | 4.9 | (Q3UM45) Protein phosphatase 1 regulatory subunit 7 (Protein phosphatase 1 regulatory subunit 22) | 9.9251E-05 | 7.3716E-05 | 74% | A, B, C |
| Q61699 | HS105_MOUSE | 858 | 96407 | 5.5 | (Q61699) Heat-shock protein 105 kDa (Heat shock-related 100 kDa protein E7I) (HSP-E7I) (Heat shock 110 kDa protein) (42 degrees C-HSP) | 9.9203E-05 | 4.2291E-05 | 43% | A, B, C |
| Q9D7S9 | CHMP5_MOUSE | 219 | 24576 | 4.8 | (Q9D7S9) Charged multivesicular body protein 5 (Chromatin-modifying protein 5) (SNF7 domain-containing protein 2) | 9.8941E-05 | 2.55E-05 | 26% | A, B, C |
| P27612 | PLAP_MOUSE | 794 | 87235 | 6.1 | (P27612) Phospholipase A-2-activating protein (PLAP) | 9.8833E-05 | 5.2116E-05 | 53% | A, B, C |
| Q8VHF2 | MUCDL_MOUSE | 831 | 88208 | 5.1 | (Q8VHF2) Mucin and cadherin-like protein precursor (Mu-protocadherin) | 9.8746E-05 | 2.3309E-05 | 24% | A, B, C |
| P46664 | PURA2_MOUSE | 456 | 50021 | 6.4 | (P46664) Adenylosuccinate synthetase isozyme 2 (EC 6.3.4.4) (Adenylosuccinate synthetase, non-muscle isozyme) (Adenylosuccinate synthetase, acidic isozyme) (IMP--aspartate ligase 2) (AdSS 2) (AMPSase 2) | 9.8447E-05 | 5.6035E-05 | 57% | A, B, C |
| Q62448 | IF4G2_MOUSE | 906 | 102105 | 7.1 | (Q62448) Eukaryotic translation initiation factor 4 gamma 2 (eIF-4-gamma 2) (eIF-4G 2) (eIF4G 2) (p97) (Novel APOBEC-1 target 1) (Translation repressor NAT1) | 9.8347E-05 | 2.2278E-05 | 23% | A, B, C |
| O89017 | LGMN_MOUSE | 435 | 49373 | 6.4 | (O89017) Legumain precursor (EC 3.4.22.34) (Asparaginyl endopeptidase) (Protease, cysteine 1) | 9.8153E-05 | 8.1442E-05 | 83% | A, B, C |
| Q64324 | STXB2_MOUSE | 593 | 66358 | 6.7 | (Q64324) Syntaxin-binding protein 2 (UNC-18 homolog 2) (UNC-18B) (MUSEC1) | 9.7971E-05 | 0.0001026 | 105% | A, B, C |
| P54775 | PRS6B_MOUSE | 418 | 47281 | 5.3 | (P54775) 26S protease regulatory subunit 6B (MIP224) (MB67-interacting protein) (TAT-binding protein 7) (TBP-7) (CIP21) | 9.7723E-05 | 1.7733E-05 | 18% | A, B, C |
| Q62465 | VAT1_MOUSE | 406 | 43097 | 6.4 | (Q62465) Synaptic vesicle membrane protein VAT-1 homolog (EC 1.-.-.-) | 9.7695E-05 | 4.5363E-05 | 46% | A, B, C |
| Q9ERD7 | TBB3_MOUSE | 450 | 50419 | 4.9 | (Q9ERD7) Tubulin beta-3 chain | 9.6374E-05 | 2.746E-05 | 28% | A, B, C |

| | | | | | | | | | |
|---|---|---|---|---|---|---|---|---|---|
| Q9D6M3 | GHC1_MOUSE | 323 | 34670 | 9.1 | (Q9D6M3) Mitochondrial glutamate carrier 1 (GC-1) (Glutamate/H(+) symporter 1) (Solute carrier family 25 member 22) | 9.5856E-05 | 2.1597E-05 | 23% | A, B, C |
| O55137 | ACOT1_MOUSE | 419 | 46136 | 6.6 | (O55137) Acyl-coenzyme A thioesterase 1 (EC 3.1.2.2) (Acyl-CoA thioesterase 1) (Inducible cytosolic acyl-coenzyme A thioester hydrolase) (Long chain acyl-CoA thioester hydrolase) (Long chain acyl-CoA hydrolase) (CTE-I) | 9.5763E-05 | 4.284E-05 | 45% | A, B, C |
| Q8R1G6 | PDLI2_MOUSE | 349 | 37703 | 8.7 | (Q8R1G6) PDZ and LIM domain protein 2 (PDZ-LIM protein mystique) | 9.5405E-05 | 1.9093E-05 | 20% | A, B, C |
| Q9WVT6 | CAH14_MOUSE | 337 | 37505 | 6.4 | (Q9WVT6) Carbonic anhydrase 14 precursor (EC 4.2.1.1) (Carbonic anhydrase XIV) (Carbonate dehydratase XIV) (CA-XIV) | 9.5031E-05 | 3.4119E-05 | 36% | A, B, C |
| Q8VH51 | RNPC2_MOUSE | 530 | 59494 | 10.1 | (Q8VH51) RNA-binding region-containing protein 2 (Coactivator of activating protein 1 and estrogen receptors) (Coactivator of AP-1 and ERs) (Transcription coactivator CAPER) | 9.48E-05 | 8.9776E-05 | 95% | A, B, C |
| P50136 | ODBA_MOUSE | 442 | 50371 | 8.1 | (P50136) 2-oxoisovalerate dehydrogenase subunit alpha, mitochondrial precursor (EC 1.2.4.4) (Branched-chain alpha-keto acid dehydrogenase E1 component alpha chain) (BCKDH E1-alpha) | 9.4143E-05 | 7.1667E-05 | 76% | A, B, C |
| P39654 | LX12L_MOUSE | 662 | 75286 | 6 | (P39654) Arachidonate 12-lipoxygenase, leukocyte-type (EC 1.13.11.31) (12-LOX) | 9.3773E-05 | 2.154E-05 | 23% | A, B, C |
| Q80UG5 | SEPT9_MOUSE | 583 | 65575 | 8.9 | (Q80UG5) Septin-9 (SL3-3 integration site 1 protein) | 9.3579E-05 | 2.9739E-05 | 32% | A, B, C |
| P14576 | SRP54_MOUSE | 504 | 55721 | 8.8 | (P14576) Signal recognition particle 54 kDa protein (SRP54) | 9.3062E-05 | 3.5507E-05 | 38% | A, B, C |
| Q91YW3 | DNJC3_MOUSE | 504 | 57464 | 5.8 | (Q91YW3) DnaJ homolog subfamily C member 3 (Interferon-induced, double-stranded RNA-activated protein kinase inhibitor) (Protein kinase inhibitor p58) (Protein kinase inhibitor of 58 kDa) | 9.2853E-05 | 4.0498E-05 | 44% | A, B, C |
| Q80VP1 | EPN1_MOUSE | 566 | 59165 | 4.7 | (Q80VP1) Epsin-1 (EPS-15-interacting protein 1) (Intersectin-EH-binding protein 1) (Ibp1) | 9.2796E-05 | 4.8399E-05 | 52% | A, B, C |
| Q6EJB6 | UT14B_MOUSE | 756 | 85928 | 8.8 | (Q6EJB6) U3 small nucleolar RNA-associated protein 14 homolog B (Juvenile spermatogonial depletion protein) | 9.0528E-05 | 7.7898E-05 | 86% | A, B, C |
| O35409 | FOLH1_MOUSE | 752 | 84635 | 7.5 | (O35409) Glutamate carboxypeptidase 2 (EC 3.4.17.21) (Glutamate carboxypeptidase II) (Membrane glutamate carboxypeptidase) (mGCP) (N-acetylated-alpha-linked acidic dipeptidase I) (NAALADase I) (Pteroylpoly-gamma-glutamate carboxypeptidase) (F | 9.0462E-05 | 3.7429E-05 | 41% | A, B, C |
| Q64176 | EST22_MOUSE | 562 | 61582 | 6.1 | (Q64176) Liver carboxylesterase 22 precursor (EC 3.1.1.1) (Egasyn) (Esterase-22) (Es-22) | 9.0346E-05 | 6.8329E-05 | 76% | A, B, C |
| P97494 | GSH1_MOUSE | 636 | 72440 | 5.9 | (P97494) Glutamate--cysteine ligase catalytic subunit (EC 6.3.2.2) (Gamma-glutamylcysteine synthetase) (Gamma-ECS) (GCS heavy chain) | 9.002E-05 | 2.91E-05 | 32% | A, B, C |
| P32261 | ANT3_MOUSE | 465 | 52004 | 6.5 | (P32261) Antithrombin-III precursor (ATIII) | 8.98E-05 | 1.514E-05 | 17% | A, B, C |
| P01027 | CO3_MOUSE | 1663 | 186482 | 6.8 | (P01027) Complement C3 precursor (HSE-MSF) [Contains: Complement C3 beta chain; Complement C3 alpha chain; C3a anaphylatoxin; Complement C3b alpha' chain; Complement C3c fragment; Complement C3dg fragment; Complement C3g fragment; Complement C3 | 8.9093E-05 | 4.8975E-05 | 55% | A, B, C |
| Q62241 | RU1C_MOUSE | 159 | 17364 | 9.7 | (Q62241) U1 small nuclear ribonucleoprotein C (U1 snRNP protein C) (U1C protein) (U1-C) | 8.9087E-05 | 3.4484E-05 | 39% | A, B, C |

| | | | | | | | | | |
|---|---|---|---|---|---|---|---|---|---|
| Q9Z2W0 | DNPEP_MOUSE | 473 | 52167 | 7.1 | (Q9Z2W0) Aspartyl aminopeptidase (EC 3.4.11.21) | 8.8674E-05 | 1.8654E-05 | 21% | A, B, C |
| P57016 | LAD1_MOUSE | 528 | 58864 | 9.7 | (P57016) Ladinin 1 (Lad-1) (Linear IgA disease autoantigen) | 8.8615E-05 | 3.4269E-05 | 39% | A, B, C |
| P14246 | GTR2_MOUSE | 523 | 57107 | 6.7 | (P14246) Solute carrier family 2, facilitated glucose transporter member 2 (Glucose transporter type 2, liver) | 8.8155E-05 | 8.2437E-05 | 94% | A, B, C |
| Q8JZK9 | HMCS1_MOUSE | 520 | 57569 | 6 | (Q8JZK9) Hydroxymethylglutaryl-CoA synthase, cytoplasmic (EC 2.3.3.10) (HMG-CoA synthase) (3-hydroxy-3-methylglutaryl coenzyme A synthase) | 8.8093E-05 | 6.9047E-05 | 78% | A, B, C |
| O55222 | ILK_MOUSE | 452 | 51347 | 8.1 | (O55222) Integrin-linked protein kinase (EC 2.7.11.1) | 8.756E-05 | 4.1059E-05 | 47% | A, B, C |
| Q9CQY6 | CF125_MOUSE | 136 | 16321 | 9.2 | (Q9CQY6) Protein C6orf125 homolog | 8.6985E-05 | 5.068E-05 | 58% | A, B, C |
| Q9CXW2 | RT22_MOUSE | 359 | 41192 | 8.6 | (Q9CXW2) Mitochondrial 28S ribosomal protein S22 (S22mt) (MRP-S22) | 8.676E-05 | 3.1301E-05 | 36% | A, B, C |
| O88322 | NID2_MOUSE | 1403 | 154249 | 5.4 | (O88322) Nidogen-2 precursor (NID-2) (Entactin-2) | 8.6313E-05 | 2.6875E-05 | 31% | A, B, C |
| Q8R146 | APEH_MOUSE | 732 | 81522 | 5.5 | (Q8R146) Acylamino-acid-releasing enzyme (EC 3.4.19.1) (AARE) (Acyl-peptide hydrolase) (APH) (Acylaminoacyl-peptidase) | 8.6301E-05 | 1.9919E-05 | 23% | A, B, C |
| Q9CR16 | PPID_MOUSE | 369 | 40611 | 7.4 | (Q9CR16) 40 kDa peptidyl-prolyl cis-trans isomerase (EC 5.2.1.8) (PPIase) (Rotamase) (Cyclophilin-40) (CYP-40) | 8.6287E-05 | 4.3453E-05 | 50% | A, B, C |
| P06728 | APOA4_MOUSE | 395 | 45029 | 5.6 | (P06728) Apolipoprotein A-IV precursor (Apo-AIV) (ApoA-IV) | 8.615E-05 | 4.0482E-05 | 47% | A, B, C |
| Q99KP6 | PRP19_MOUSE | 504 | 55239 | 6.6 | (Q99KP6) Pre-mRNA-splicing factor 19 (PRP19/PSO4 homolog) (Nuclear matrix protein 200) (Nuclear matrix protein SNEV) | 8.6049E-05 | 2.4518E-05 | 28% | A, B, C |
| Q8BRF7 | SCFD1_MOUSE | 639 | 72323 | 6.4 | (Q8BRF7) Sec1 family domain-containing protein 1 (Syntaxin-binding protein 1-like 2) | 8.4812E-05 | 2.7319E-05 | 32% | A, B, C |
| P47758 | SRPRB_MOUSE | 269 | 29579 | 9.3 | (P47758) Signal recognition particle receptor subunit beta (SR-beta) | 8.4658E-05 | 3.9486E-05 | 47% | A, B, C |
| P63011 | RAB3A_MOUSE | 220 | 24970 | 5 | (P63011) Ras-related protein Rab-3A | 8.3944E-05 | 4.1432E-05 | 49% | A, B, C |
| Q99JX3 | GORS2_MOUSE | 450 | 46907 | 4.8 | (Q99JX3) Golgi reassembly-stacking protein 2 (GRS2) (Golgi reassembly-stacking protein of 55 kDa) (GRASP55) | 8.2954E-05 | 3.4489E-05 | 42% | A, B, C |
| Q8CBY8 | DCTN4_MOUSE | 467 | 53057 | 7.7 | (Q8CBY8) Dynactin subunit 4 (Dynactin subunit p62) | 8.2867E-05 | 2.5226E-05 | 30% | A, B, C |
| Q9Z1Z2 | STRAP_MOUSE | 351 | 38513 | 5.1 | (Q9Z1Z2) Serine-threonine kinase receptor-associated protein (UNR-interacting protein) | 8.261E-05 | 5.7368E-05 | 69% | A, B, C |
| Q8VCN5 | CGL_MOUSE | 398 | 43567 | 7.6 | (Q8VCN5) Cystathionine gamma-lyase (EC 4.4.1.1) (Gamma-cystathionase) | 8.1005E-05 | 5.2642E-05 | 65% | A, B, C |
| Q62186 | SSRD_MOUSE | 172 | 18937 | 5.8 | (Q62186) Translocon-associated protein subunit delta precursor (TRAP-delta) (Signal sequence receptor subunit delta) (SSR-delta) | 8.022E-05 | 2.2295E-05 | 28% | A, B, C |
| Q78ZA7 | NP1L4_MOUSE | 375 | 42679 | 4.7 | (Q78ZA7) Nucleosome assembly protein 1-like 4 | 8.014E-05 | 1.2272E-05 | 15% | A, B, C |
| P08226 | APOE_MOUSE | 311 | 35867 | 5.7 | (P08226) Apolipoprotein E precursor (Apo-E) | 8.0103E-05 | 2.0663E-05 | 26% | A, B, C |
| O08788 | DYNA_MOUSE | 1281 | 141727 | 6 | (O08788) Dynactin-1 (150 kDa dynein-associated polypeptide) (DP-150) (DAP-150) (p150-glued) | 7.9976E-05 | 3.295E-05 | 41% | A, B, C |
| Q61733 | RT31_MOUSE | 384 | 43881 | 8.5 | (Q61733) 28S ribosomal protein S31, mitochondrial precursor (S31mt) (MRP-S31) (Imogen 38) | 7.9687E-05 | 9.1196E-06 | 11% | A, B, C |
| P21279 | GNAQ_MOUSE | 353 | 41483 | 5.8 | (P21279) Guanine nucleotide-binding protein G(q) subunit alpha (Guanine nucleotide-binding protein alpha-q) | 7.9292E-05 | 3.7439E-05 | 47% | A, B, C |
| Q08509 | EPS8_MOUSE | 821 | 91738 | 7.5 | (Q08509) Epidermal growth factor receptor kinase substrate 8 | 7.9186E-05 | 5.4835E-05 | 69% | A, B, C |
| Q99LG1 | TMM51_MOUSE | 249 | 27398 | 7 | (Q99LG1) Transmembrane protein 51 | 7.8871E-05 | 5.4227E-05 | 69% | A, B, C |
| Q8K4Z5 | SF3A1_MOUSE | 791 | 88545 | 5.2 | (Q8K4Z5) Splicing factor 3 subunit 1 (SF3a120) | 7.8703E-05 | 3.1923E-05 | 41% | A, B, C |

| | | | | | | | | | |
|---|---|---|---|---|---|---|---|---|---|
| P49586 | PCY1A_MOUSE | 367 | 41667 | 7 | (P49586) Choline-phosphate cytidylyltransferase A (EC 2.7.7.15) (Phosphorylcholine transferase A) (CTP:phosphocholine cytidylyltransferase A) (CT A) (CCT A) (CCT-alpha) | 7.8542E-05 | 4.5882E-05 | 58% | A, B, C |
| Q9DBG5 | M6PBP_MOUSE | 437 | 47262 | 5.6 | (Q9DBG5) Mannose-6-phosphate receptor-binding protein 1 (Cargo selection protein TIP47) | 7.8493E-05 | 6.9413E-05 | 88% | A, B, C |
| P63094 | GNAS_MOUSE | 394 | 45664 | 6 | (P63094) Guanine nucleotide-binding protein G(s) subunit alpha (Adenylate cyclase-stimulating G alpha protein) | 7.8356E-05 | 4.6202E-05 | 59% | A, B, C |
| P54823 | DDX6_MOUSE | 483 | 54192 | 8.7 | (P54823) Probable ATP-dependent RNA helicase DDX6 (EC 3.6.1.-) (DEAD box protein 6) (ATP-dependent RNA helicase p54) (Oncogene RCK homolog) | 7.824E-05 | 3.4406E-05 | 44% | A, B, C |
| Q9CX34 | SUGT1_MOUSE | 335 | 38028 | 5.4 | (Q9CX34) Suppressor of G2 allele of SKP1 homolog | 7.7403E-05 | 3.185E-05 | 41% | A, B, C |
| P70372 | ELAV1_MOUSE | 326 | 36069 | 9.2 | (P70372) ELAV-like protein 1 (Hu-antigen R) (HuR) (Elav-like generic protein) (MelG) | 7.6751E-05 | 2.5521E-05 | 33% | A, B, C |
| Q9JLT2 | TREA_MOUSE | 576 | 65401 | 5.6 | (Q9JLT2) Trehalase precursor (EC 3.2.1.28) (Alpha,alpha-trehalase) (Alpha,alpha-trehalose glucohydrolase) | 7.6496E-05 | 4.968E-05 | 65% | A, B, C |
| Q61739 | ITA6_MOUSE | 1091 | 122148 | 7 | (Q61739) Integrin alpha-6 precursor (VLA-6) (CD49f antigen) [Contains: Integrin alpha-6 heavy chain; Integrin alpha-6 light chain] | 7.5913E-05 | 2.1285E-05 | 28% | A, B, C |
| Q8CGC7 | SYEP_MOUSE | 1512 | 169936 | 7.6 | (Q8CGC7) Bifunctional aminoacyl-tRNA synthetase [Includes: Glutamyl-tRNA synthetase (EC 6.1.1.17) (Glutamate--tRNA ligase); Prolyl-tRNA synthetase (EC 6.1.1.15) (Proline--tRNA ligase)] | 7.3785E-05 | 2.0809E-05 | 28% | A, B, C |
| Q9Z2U0 | PSA7_MOUSE | 248 | 27855 | 8.5 | (Q9Z2U0) Proteasome subunit alpha type 7 (EC 3.4.25.1) (Proteasome subunit RC6-1) | 7.3511E-05 | 7.2357E-05 | 98% | A, B, C |
| Q9D358 | PPAC_MOUSE | 157 | 18061 | 6.8 | (Q9D358) Low molecular weight phosphotyrosine protein phosphatase (EC 3.1.3.48) (LMW-PTP) (Low molecular weight cytosolic acid phosphatase) (EC 3.1.3.2) (PTPase) | 7.3013E-05 | 3.4642E-05 | 47% | A, B, C |
| O55125 | NIPS1_MOUSE | 284 | 33363 | 9.4 | (O55125) Protein NipSnap1 | 7.2978E-05 | 4.5518E-05 | 62% | A, B, C |
| P51885 | LUM_MOUSE | 338 | 38265 | 6.4 | (P51885) Lumican precursor (Keratan sulfate proteoglycan lumican) (KSPG lumican) | 7.2664E-05 | 1.2768E-05 | 18% | A, B, C |
| Q9CWZ7 | SNAG_MOUSE | 312 | 34732 | 5.4 | (Q9CWZ7) Gamma-soluble NSF attachment protein (SNAP-gamma) (N-ethylmaleimide-sensitive factor attachment protein, gamma) | 7.2362E-05 | 1.9202E-05 | 27% | A, B, C |
| Q8R361 | RFIP5_MOUSE | 645 | 69553 | 9.1 | (Q8R361) Rab11 family-interacting protein 5 (Rab11-FIP5) (Rab11-interacting protein Rip11) | 7.2027E-05 | 3.1388E-05 | 44% | A, B, C |
| Q71LX4 | TLN2_MOUSE | 2375 | 253554 | 5.8 | (Q71LX4) Talin-2 | 7.1966E-05 | 3.9507E-05 | 55% | A, B, C |
| P47754 | CAZA2_MOUSE | 285 | 32836 | 5.8 | (P47754) F-actin capping protein alpha-2 subunit (CapZ alpha-2) | 7.1482E-05 | 2.8667E-05 | 40% | A, B, C |
| Q99L43 | CDS2_MOUSE | 444 | 51314 | 7 | (Q99L43) Phosphatidate cytidylyltransferase 2 (EC 2.7.7.41) (CDP-diglyceride synthetase 2) (CDP-diglyceride pyrophosphorylase 2) (CDP-diacylglycerol synthase 2) (CDS 2) (CTP:phosphatidate cytidylyltransferase 2) (CDP-DAG synthase 2) (CDP-DG sy | 7.0124E-05 | 1.6382E-05 | 23% | A, B, C |
| Q9JHU4 | DYHC_MOUSE | 4644 | 532030 | 6.4 | (Q9JHU4) Dynein heavy chain, cytosolic (DYHC) (Cytoplasmic dynein heavy chain 1) (DHC1) (Dynein heavy chain 1, cytoplasmic 1) | 6.9245E-05 | 2.1856E-05 | 32% | A, B, C |
| Q9Z1D1 | IF34_MOUSE | 320 | 35638 | 5.9 | (Q9Z1D1) Eukaryotic translation initiation factor 3 subunit 4 (eIF-3 delta) (eIF3 p44) (eIF-3 RNA-binding subunit) (eIF3 p42) (Eif3p42) (eIF3g) | 6.9184E-05 | 1.0567E-05 | 15% | A, B, C |

| | | | | | | | | | |
|---|---|---|---|---|---|---|---|---|---|
| Q8R317 | UBQL1_MOUSE | 582 | 61976 | 4.9 | (Q8R317) Ubiquilin-1 (Protein linking IAP with cytoskeleton 1) (PLIC-1) | 6.8624E-05 | 2.4397E-05 | 36% | A, B, C |
| Q8VDC0 | SYLM_MOUSE | 902 | 101480 | 8.2 | (Q8VDC0) Probable leucyl-tRNA synthetase, mitochondrial precursor (EC 6.1.1.4) (Leucine--tRNA ligase) (LeuRS) | 6.8044E-05 | 1.9257E-05 | 28% | A, B, C |
| Q8BJY1 | PSD5_MOUSE | 503 | 55841 | 5.2 | (Q8BJY1) 26S proteasome non-ATPase regulatory subunit 5 (26S proteasome subunit S5B) (26S protease subunit S5 basic) | 6.8002E-05 | 2.4969E-05 | 37% | A, B, C |
| P97434 | MRIP_MOUSE | 1024 | 116408 | 6.2 | (P97434) Myosin phosphatase Rho-interacting protein (Rho-interacting protein 3) (p116Rip) (RIP3) | 6.7968E-05 | 4.4554E-05 | 66% | A, B, C |
| P17427 | AP2A2_MOUSE | 938 | 104101 | 6.9 | (P17427) AP-2 complex subunit alpha-2 (Adapter-related protein complex 2 alpha-2 subunit) (Alpha-adaptin C) (Adaptor protein complex AP-2 alpha-2 subunit) (Clathrin assembly protein complex 2 alpha-C large chain) (100 kDa coated vesicle prote | 6.7766E-05 | 2.6352E-05 | 39% | A, B, C |
| Q8K2L8 | TTC15_MOUSE | 797 | 87724 | 4.8 | (Q8K2L8) Tetratricopeptide repeat protein 15 (TPR repeat protein 15) | 6.7533E-05 | 2.7638E-05 | 41% | A, B, C |
| Q9D2V7 | CORO7_MOUSE | 922 | 100812 | 5.8 | (Q9D2V7) Coronin-7 (70 kDa WD repeat tumor rejection antigen homolog) | 6.7521E-05 | 2.7387E-05 | 41% | A, B, C |
| P17426 | AP2A1_MOUSE | 977 | 107664 | 7 | (P17426) AP-2 complex subunit alpha-1 (Adapter-related protein complex 2 alpha-1 subunit) (Alpha-adaptin A) (Adaptor protein complex AP-2 alpha-1 subunit) (Clathrin assembly protein complex 2 alpha-A large chain) (100 kDa coated vesicle prote | 6.7071E-05 | 2.8929E-05 | 43% | A, B, C |
| Q9JMA1 | UBP14_MOUSE | 492 | 55871 | 5.2 | (Q9JMA1) Ubiquitin carboxyl-terminal hydrolase 14 (EC 3.1.2.15) (Ubiquitin thioesterase 14) (Ubiquitin-specific-processing protease 14) (Deubiquitinating enzyme 14) | 6.694E-05 | 6.5977E-06 | 10% | A, B, C |
| P46935 | NEDD4_MOUSE | 887 | 102706 | 5.3 | (P46935) E3 ubiquitin-protein ligase NEDD4 (EC 6.3.2.-) (Neural precursor cell expressed developmentally down-regulated protein 4) | 6.6737E-05 | 1.7527E-05 | 26% | A, B, C |
| Q91WQ3 | SYYC_MOUSE | 527 | 58974 | 7 | (Q91WQ3) Tyrosyl-tRNA synthetase, cytoplasmic (EC 6.1.1.1) (Tyrosyl--tRNA ligase) (TyrRS) | 6.6296E-05 | 3.6522E-05 | 55% | A, B, C |
| Q61033 | LAP2A_MOUSE | 692 | 75200 | 8.3 | (Q61033) Lamina-associated polypeptide 2 isoforms alpha/zeta (Thymopoietin isoforms alpha/zeta) (TP alpha/zeta) | 6.6045E-05 | 2.0877E-05 | 32% | A, B, C |
| Q9WVL3 | S12A7_MOUSE | 1083 | 119481 | 6.5 | (Q9WVL3) Solute carrier family 12 member 7 (Electroneutral potassium-chloride cotransporter 4) (K-Cl cotransporter 4) | 6.5686E-05 | 4.4958E-05 | 68% | A, B, C |
| Q9D024 | CC47_MOUSE | 483 | 55844 | 4.8 | (Q9D024) Coiled-coil domain-containing protein 47 precursor (Adipocyte-specific protein 4) | 6.5679E-05 | 5.1803E-05 | 79% | A, B, C |
| Q8VD04 | GRAP1_MOUSE | 806 | 92715 | 5.2 | (Q8VD04) GRIP1-associated protein 1 (GRASP-1) (HCMV-interacting protein) | 6.5662E-05 | 2.6817E-05 | 41% | A, B, C |
| Q61768 | KINH_MOUSE | 963 | 109549 | 6.3 | (Q61768) Kinesin heavy chain (Ubiquitous kinesin heavy chain) (UKHC) | 6.5568E-05 | 3.2575E-05 | 50% | A, B, C |
| Q99LI8 | HGS_MOUSE | 775 | 86015 | 6.2 | (Q99LI8) Hepatocyte growth factor-regulated tyrosine kinase substrate | 6.5504E-05 | 1.0629E-05 | 16% | A, B, C |
| Q05793 | PGBM_MOUSE | 3707 | 398295 | 6.3 | (Q05793) Basement membrane-specific heparan sulfate proteoglycan core protein precursor (HSPG) (Perlecan) (PLC) | 6.5446E-05 | 2.5124E-06 | 4% | A, B, C |

| | | | | | | | | | |
|---|---|---|---|---|---|---|---|---|---|
| Q8CDG3 | VCIP1_MOUSE | 1220 | 134503 | 7.2 | (Q8CDG3) Deubiquitinating protein VCIP135 (EC 3.4.22.-) (Valosin-containing protein p97/p47 complex-interacting protein p135) (Valosin-containing protein p97/p47 complex-interacting protein 1) | 6.5441E-05 | 5.6682E-05 | 87% | A, B, C |
| P41216 | ACSL1_MOUSE | 699 | 77923 | 7.1 | (P41216) Long-chain-fatty-acid--CoA ligase 1 (EC 6.2.1.3) (Long-chain acyl-CoA synthetase 1) (LACS 1) | 6.5384E-05 | 2.0668E-05 | 32% | A, B, C |
| Q9JLZ8 | SIGIR_MOUSE | 409 | 46159 | 5.7 | (Q9JLZ8) Single Ig IL-1-related receptor (Single Ig IL-1R-related molecule) (Single immunoglobulin domain-containing IL1R-related protein) (Toll/interleukin-1 receptor 8) (TIR8) | 6.4768E-05 | 3.5213E-05 | 54% | A, B, C |
| Q9CWJ9 | PUR9_MOUSE | 592 | 64157 | 6.8 | (Q9CWJ9) Bifunctional purine biosynthesis protein PURH [Includes: Phosphoribosylaminoimidazolecarboxamide formyltransferase (EC 2.1.2.3) (AICAR transformylase); IMP cyclohydrolase (EC 3.5.4.10) (Inosinicase) (IMP synthetase) (ATIC)] | 6.4132E-05 | 1.9554E-05 | 30% | A, B, C |
| P56959 | FUS_MOUSE | 518 | 52673 | 9.4 | (P56959) RNA-binding protein FUS (Pigpen protein) | 6.3639E-05 | 4.3717E-05 | 69% | A, B, C |
| P39447 | ZO1_MOUSE | 1745 | 194710 | 6.7 | (P39447) Tight junction protein ZO-1 (Zonula occludens 1 protein) (Zona occludens 1 protein) (Tight junction protein 1) | 6.3501E-05 | 2.8545E-05 | 45% | A, B, C |
| Q3TXS7 | PSD1_MOUSE | 953 | 105730 | 5.4 | (Q3TXS7) 26S proteasome non-ATPase regulatory subunit 1 (26S proteasome regulatory subunit RPN2) (26S proteasome regulatory subunit S1) | 6.3264E-05 | 1.1049E-05 | 17% | A, B, C |
| Q9DCT1 | AK1E1_MOUSE | 301 | 34461 | 7.3 | (Q9DCT1) Aldo-keto reductase family 1 member E1 (EC 1.1.1.-) | 6.2918E-05 | 2.7366E-05 | 43% | A, B, C |
| P29621 | SPA3C_MOUSE | 417 | 46766 | 7.9 | (P29621) Serine protease inhibitor A3C precursor (Kallikrein-binding protein) (KBP) (Serpin A3C) | 6.2862E-05 | 2.6177E-05 | 42% | A, B, C |
| Q02788 | CO6A2_MOUSE | 1029 | 109812 | 6.3 | (Q02788) Collagen alpha-2(VI) chain precursor | 6.2778E-05 | 2.1609E-05 | 34% | A, B, C |
| Q569Z5 | DDX46_MOUSE | 1032 | 117448 | 9.3 | (Q569Z5) Probable ATP-dependent RNA helicase DDX46 (EC 3.6.1.-) (DEAD box protein 46) | 6.2295E-05 | 3.0345E-05 | 49% | A, B, C |
| P42567 | EP15_MOUSE | 897 | 98471 | 4.6 | (P42567) Epidermal growth factor receptor substrate 15 (Protein Eps15) (AF-1p protein) | 6.218E-05 | 3.5325E-05 | 57% | A, B, C |
| Q80WC7 | HRBL_MOUSE | 479 | 48968 | 9.1 | (Q80WC7) HIV-1 Rev-binding protein-like protein (Rev/Rex activation domain-binding protein related) (RAB-R) | 6.2048E-05 | 4.1316E-05 | 67% | A, B, C |
| P11152 | LIPL_MOUSE | 474 | 53127 | 8 | (P11152) Lipoprotein lipase precursor (EC 3.1.1.34) (LPL) | 6.1871E-05 | 2.8284E-05 | 46% | A, B, C |
| Q9QWR8 | NAGAB_MOUSE | 415 | 47235 | 6.4 | (Q9QWR8) Alpha-N-acetylgalactosaminidase precursor (EC 3.2.1.49) (Alpha-galactosidase B) | 6.182E-05 | 3.7321E-05 | 60% | A, B, C |
| Q91YD9 | WASL_MOUSE | 501 | 54274 | 7.9 | (Q91YD9) Neural Wiskott-Aldrich syndrome protein (N-WASP) | 6.1505E-05 | 4.0476E-05 | 66% | A, B, C |
| Q8C2Q3 | RBM14_MOUSE | 669 | 69449 | 9.7 | (Q8C2Q3) RNA-binding protein 14 (RNA-binding motif protein 14) | 6.1336E-05 | 1.7002E-05 | 28% | A, B, C |
| P24549 | AL1A1_MOUSE | 500 | 54337 | 7.8 | (P24549) Retinal dehydrogenase 1 (EC 1.2.1.36) (RalDH1) (RALDH 1) (Aldehyde dehydrogenase family 1 member A1) (Aldehyde dehydrogenase, cytosolic) (ALHDII) (ALDH-E1) | 6.1225E-05 | 4.6879E-05 | 77% | A, B, C |
| Q8BWM0 | PGES2_MOUSE | 384 | 43308 | 9 | (Q8BWM0) Prostaglandin E synthase 2 (EC 5.3.99.3) (Microsomal prostaglandin E synthase 2) (mPGES-2) (GATE-binding factor 1) (GBF-1) [Contains: Prostaglandin E synthase 2 truncated form] | 6.1162E-05 | 3.5121E-05 | 57% | A, B, C |
| P17710 | HXK1_MOUSE | 974 | 108302 | 6.9 | (P17710) Hexokinase-1 (EC 2.7.1.1) (Hexokinase type I) (HK I) (Hexokinase, tumor isozyme) | 6.0988E-05 | 2.7371E-05 | 45% | A, B, C |
| P54728 | RD23B_MOUSE | 416 | 43517 | 4.8 | (P54728) UV excision repair protein RAD23 homolog B (mHR23B) (XP-C repair-complementing complex 58 kDa protein) (p58) | 6.0697E-05 | 5.5659E-05 | 92% | A, B, C |

| | | | | | | | | | |
|---|---|---|---|---|---|---|---|---|---|
| P29351 | PTN6_MOUSE | 595 | 67559 | 7.8 | (P29351) Tyrosine-protein phosphatase non-receptor type 6 (EC 3.1.3.48) (Protein-tyrosine phosphatase 1C) (PTP-1C) (Hematopoietic cell protein-tyrosine phosphatase) (70Z-SHP) (SH-PTP1) (SHP-1) (PTPTY-42) | 6.0218E-05 | 4.4725E-05 | 74% | A, B, C |
| Q99JG3 | ANX13_MOUSE | 316 | 35791 | 6 | (Q99JG3) Annexin A13 (Annexin XIII) | 5.9931E-05 | 2.6067E-05 | 43% | A, B, C |
| Q9DBR7 | MYPT1_MOUSE | 1004 | 111809 | 5.6 | (Q9DBR7) Protein phosphatase 1 regulatory subunit 12A (Myosin phosphatase-targeting subunit 1) (Myosin phosphatase target subunit 1) | 5.9865E-05 | 9.1674E-06 | 15% | A, B, C |
| P62334 | PRS10_MOUSE | 389 | 44173 | 7.5 | (P62334) 26S protease regulatory subunit S10B (Proteasome subunit p42) (Proteasome 26S subunit ATPase 6) | 5.9317E-05 | 3.9708E-05 | 67% | A, B, C |
| Q6ZQF0 | TOPB1_MOUSE | 1515 | 168859 | 6.7 | (Q6ZQF0) DNA topoisomerase II-binding protein 1 (DNA topoisomerase IIbeta-binding protein 1) (TopBP1) | 5.8651E-05 | 3.0829E-05 | 53% | A, B, C |
| O08914 | FAAH_MOUSE | 579 | 63221 | 7.9 | (O08914) Fatty-acid amide hydrolase (EC 3.1.-.-) (Oleamide hydrolase) (Anandamide amidohydrolase) | 5.7559E-05 | 4.4414E-05 | 77% | A, B, C |
| Q61391 | NEP_MOUSE | 749 | 85571 | 5.8 | (Q61391) Neprilysin (EC 3.4.24.11) (Neutral endopeptidase) (NEP) (Enkephalinase) (Neutral endopeptidase 24.11) (Atriopeptidase) (CD10 antigen) | 5.746E-05 | 3.2498E-05 | 57% | A, B, C |
| P16406 | AMPE_MOUSE | 945 | 107956 | 5.4 | (P16406) Glutamyl aminopeptidase (EC 3.4.11.7) (EAP) (Aminopeptidase A) (APA) (BP-1/6C3 antigen) | 5.6924E-05 | 2.7369E-05 | 48% | A, B, C |
| P58871 | TB182_MOUSE | 909 | 97079 | 4.8 | (P58871) 182 kDa tankyrase 1-binding protein (Fragment) | 5.6052E-05 | 1.891E-05 | 34% | A, B, C |
| Q80XI3 | IF4G3_MOUSE | 1579 | 174889 | 5.5 | (Q80XI3) Eukaryotic translation initiation factor 4 gamma 3 (eIF-4-gamma 3) (eIF-4G 3) (eIF4G 3) (eIF-4-gamma II) (eIF4GII) | 5.5842E-05 | 3.4438E-05 | 62% | A, B, C |
| Q8BWY3 | ERF1_MOUSE | 436 | 48900 | 5.7 | (Q8BWY3) Eukaryotic peptide chain release factor subunit 1 (eRF1) (Eukaryotic release factor 1) | 5.5402E-05 | 2.9373E-05 | 53% | A, B, C |
| P80313 | TCPH_MOUSE | 544 | 59652 | 7.8 | (P80313) T-complex protein 1 subunit eta (TCP-1-eta) (CCT-eta) | 5.5243E-05 | 8.4596E-06 | 15% | A, B, C |
| Q8K2C6 | SIRT5_MOUSE | 310 | 34134 | 8.5 | (Q8K2C6) NAD-dependent deacetylase sirtuin-5 (EC 3.5.1.-) (SIR2-like protein 5) | 5.5114E-05 | 9.2979E-06 | 17% | A, B, C |
| O08810 | U5S1_MOUSE | 971 | 109361 | 5 | (O08810) 116 kDa U5 small nuclear ribonucleoprotein component (U5 snRNP-specific protein, 116 kDa) (U5-116 kDa) (Elongation factor Tu GTP-binding domain protein 2) | 5.4899E-05 | 3.9397E-05 | 72% | A, B, C |
| Q9JIX8 | ACINU_MOUSE | 1338 | 150691 | 5.9 | (Q9JIX8) Apoptotic chromatin condensation inducer in the nucleus (Acinus) | 5.4896E-05 | 1.1722E-05 | 21% | A, B, C |
| Q91V92 | ACLY_MOUSE | 1091 | 119728 | 7.4 | (Q91V92) ATP-citrate synthase (EC 2.3.3.8) (ATP-citrate (pro-S-)-lyase) (Citrate cleavage enzyme) | 5.4788E-05 | 1.4095E-05 | 26% | A, B, C |
| Q9JKF1 | IQGA1_MOUSE | 1657 | 188755 | 6.5 | (Q9JKF1) Ras GTPase-activating-like protein IQGAP1 | 5.3225E-05 | 1.3536E-05 | 25% | A, B, C |
| Q91ZR2 | SNX18_MOUSE | 614 | 67904 | 6.7 | (Q91ZR2) Sorting nexin-18 (Sorting nexin-associated Golgi protein 1) | 5.3192E-05 | 1.9432E-05 | 37% | A, B, C |
| Q6PDN3 | MYLK_MOUSE | 1941 | 212924 | 6.2 | (Q6PDN3) Myosin light chain kinase, smooth muscle (EC 2.7.11.18) (MLCK) (Telokin) (Kinase-related protein) (KRP) | 5.3063E-05 | 2.5782E-05 | 49% | A, B, C |
| P63328 | PP2BA_MOUSE | 521 | 58644 | 5.9 | (P63328) Serine/threonine-protein phosphatase 2B catalytic subunit alpha isoform (EC 3.1.3.16) (Calmodulin-dependent calcineurin A subunit alpha isoform) (CAM-PRP catalytic subunit) | 5.2967E-05 | 1.4721E-05 | 28% | A, B, C |
| P15092 | IFI4_MOUSE | 640 | 71648 | 8.8 | (P15092) Interferon-activable protein 204 (Ifi-204) (Interferon-inducible protein p204) | 5.288E-05 | 8.5809E-06 | 16% | A, B, C |
| Q6PDQ2 | CHD4_MOUSE | 1915 | 217749 | 5.8 | (Q6PDQ2) Chromodomain helicase-DNA-binding protein 4 (CHD 4) | 5.2371E-05 | 1.6882E-05 | 32% | A, B, C |

| | | | | | | | | | |
|---|---|---|---|---|---|---|---|---|---|
| Q8CIB5 | PKHC1_MOUSE | 680 | 77800 | 6.7 | (Q8CIB5) Pleckstrin homology domain-containing family C member 1 | 5.1911E-05 | 2.7407E-05 | 53% | A, B, C |
| P70297 | STAM1_MOUSE | 547 | 59640 | 4.8 | (P70297) Signal transducing adapter molecule 1 (STAM-1) | 5.1672E-05 | 1.2493E-05 | 24% | A, B, C |
| Q8R081 | HNRPL_MOUSE | 555 | 60123 | 7.1 | (Q8R081) Heterogeneous nuclear ribonucleoprotein L (hnRNP L) | 5.1579E-05 | 1.6673E-05 | 32% | A, B, C |
| Q9JIY5 | HTRA2_MOUSE | 458 | 49348 | 9.6 | (Q9JIY5) Serine protease HTRA2, mitochondrial precursor (EC 3.4.21.-) (High temperature requirement protein A2) (HtrA2) (Omi stress-regulated endoprotease) (Serine proteinase OMI) | 4.9723E-05 | 2.3192E-05 | 47% | A, B, C |
| P54726 | RD23A_MOUSE | 363 | 39770 | 4.6 | (P54726) UV excision repair protein RAD23 homolog A (mHR23A) | 4.969E-05 | 3.1128E-05 | 63% | A, B, C |
| Q7TPV4 | MBB1A_MOUSE | 1344 | 152036 | 9 | (Q7TPV4) Myb-binding protein 1A (Myb-binding protein of 160 kDa) | 4.9686E-05 | 2.4394E-06 | 5% | A, B, C |
| Q8K4G5 | ABLM1_MOUSE | 861 | 96805 | 8.6 | (Q8K4G5) Actin-binding LIM protein 1 (Actin-binding LIM protein family member 1) (abLIM-1) | 4.935E-05 | 1.9215E-05 | 39% | A, B, C |
| Q9JLB4 | CUBN_MOUSE | 3623 | 399070 | 5.8 | (Q9JLB4) Cubilin precursor (Intrinsic factor-cobalamin receptor) | 4.9184E-05 | 2.4541E-06 | 5% | A, B, C |
| Q9DCL9 | PUR6_MOUSE | 424 | 46939 | 7.2 | (Q9DCL9) Multifunctional protein ADE2 [Includes: Phosphoribosylaminoimidazole-succinocarboxamide synthase (EC 6.3.2.6) (SAICAR synthetase); Phosphoribosylaminoimidazole carboxylase (EC 4.1.1.21) (AIR carboxylase) (AIRC)] | 4.9062E-05 | 3.0418E-05 | 62% | A, B, C |
| P98203 | ARVC_MOUSE | 962 | 105066 | 6.6 | (P98203) Armadillo repeat protein deleted in velo-cardio-facial syndrome homolog | 4.883E-05 | 3.3741E-06 | 7% | A, B, C |
| Q8VCF0 | MAVS_MOUSE | 503 | 53399 | 6.4 | (Q8VCF0) Mitochondrial antiviral signaling protein (Interferon-beta promoter stimulator protein 1) (IPS-1) (Virus-induced signaling adapter) (CARD adapter inducing interferon-beta) (Cardif) | 4.8828E-05 | 8.58E-06 | 18% | A, B, C |
| Q8CC35 | SYNPO_MOUSE | 929 | 99552 | 9.4 | (Q8CC35) Synaptopodin | 4.844E-05 | 1.3108E-05 | 27% | A, B, C |
| Q00898 | A1AT5_MOUSE | 413 | 45891 | 5.7 | (Q00898) Alpha-1-antitrypsin 1-5 precursor (Serine protease inhibitor 1-5) (Alpha-1 protease inhibitor 5) | 4.844E-05 | 1.4872E-05 | 31% | A, B, C |
| Q9D6Z1 | NOP56_MOUSE | 580 | 64464 | 9.1 | (Q9D6Z1) Nucleolar protein Nop56 (Nucleolar protein 5A) | 4.8412E-05 | 1.865E-05 | 39% | A, B, C |
| P10493 | NID1_MOUSE | 1245 | 136623 | 5.5 | (P10493) Nidogen-1 precursor (Entactin) | 4.8284E-05 | 2.6624E-05 | 55% | A, B, C |
| Q9JMH9 | MY18A_MOUSE | 2035 | 230906 | 6.2 | (Q9JMH9) Myosin-18A (Myosin XVIIIa) (Myosin containing PDZ domain) (Molecule associated with JAK3 N-terminus) (MAJN) | 4.778E-05 | 2.0223E-05 | 42% | A, B, C |
| P26369 | U2AF2_MOUSE | 475 | 53517 | 9.1 | (P26369) Splicing factor U2AF 65 kDa subunit (U2 auxiliary factor 65 kDa subunit) (U2 snRNP auxiliary factor large subunit) | 4.7493E-05 | 3.8984E-05 | 82% | A, B, C |
| O35874 | SATT_MOUSE | 532 | 56062 | 5.9 | (O35874) Neutral amino acid transporter A (SATT) (Alanine/serine/cysteine/ threonine transporter) (ASCT1) | 4.7195E-05 | 1.5477E-05 | 33% | A, B, C |
| Q8VCT3 | AMPB_MOUSE | 650 | 72343 | 5.3 | (Q8VCT3) Aminopeptidase B (EC 3.4.11.6) (Ap-B) (Arginyl aminopeptidase) (Arginine aminopeptidase) (Cytosol aminopeptidase IV) | 4.6791E-05 | 1.7738E-05 | 38% | A, B, C |
| O54749 | CP2J5_MOUSE | 501 | 57784 | 8.7 | (O54749) Cytochrome P450 2J5 (EC 1.14.14.1) (CYPIIJ5) (Arachidonic acid epoxygenase) | 4.6004E-05 | 1.5958E-05 | 35% | A, B, C |
| Q6P9R2 | OXSR1_MOUSE | 527 | 58214 | 6.4 | (Q6P9R2) Serine/threonine-protein kinase OSR1 (EC 2.7.11.1) (Oxidative stress-responsive 1 protein) | 4.5711E-05 | 1.8087E-05 | 40% | A, B, C |
| P48193 | 41_MOUSE | 858 | 95990 | 5.6 | (P48193) Protein 4.1 (Band 4.1) (P4.1) (4.1R) | 4.5591E-05 | 2.5654E-05 | 56% | A, B, C |

| | | | | | | | | | |
|---|---|---|---|---|---|---|---|---|---|
| O88543 | CSN3_MOUSE | 422 | 47701 | 6.7 | (O88543) COP9 signalosome complex subunit 3 (Signalosome subunit 3) (SGN3) (JAB1-containing signalosome subunit 3) | 4.4631E-05 | 2.2889E-05 | 51% | A, B, C |
| Q8BMS4 | COQ3_MOUSE | 370 | 40957 | 7.9 | (Q8BMS4) Hexaprenyldihydroxybenzoate methyltransferase, mitochondrial precursor (EC 2.1.1.114) (Dihydroxyhexaprenylbenzoate methyltransferase) (3,4-dihydroxy-5-hexaprenylbenzoate methyltransferase) (DHHB methyltransferase) (DHHB-MT) (DHHB-MTas | 4.4594E-05 | 1.8196E-05 | 41% | A, B, C |
| P22892 | AP1G1_MOUSE | 821 | 91219 | 6.8 | (P22892) AP-1 complex subunit gamma-1 (Adapter-related protein complex 1 gamma-1 subunit) (Gamma-adaptin) (Adaptor protein complex AP-1 gamma-1 subunit) (Golgi adaptor HA1/AP1 adaptin subunit gamma-1) (Clathrin assembly protein complex 1 gamm | 4.4507E-05 | 1.3928E-05 | 31% | A, B, C |
| Q9D0F3 | LMAN1_MOUSE | 517 | 57789 | 6.3 | (Q9D0F3) ERGIC-53 protein precursor (ER-Golgi intermediate compartment 53 kDa protein) (Lectin, mannose-binding 1) (p58) | 4.4344E-05 | 2.104E-05 | 47% | A, B, C |
| Q9WTM5 | RUVB2_MOUSE | 462 | 50981 | 5.6 | (Q9WTM5) RuvB-like 2 (EC 3.6.1.-) (p47 protein) | 4.3648E-05 | 1.7294E-05 | 40% | A, B, C |
| Q99PG0 | AAAD_MOUSE | 397 | 45119 | 7.5 | (Q99PG0) Arylacetamide deacetylase (EC 3.1.1.-) (AADAC) | 4.3201E-05 | 3.8203E-05 | 88% | A, B, C |
| Q00623 | APOA1_MOUSE | 264 | 30587 | 5.9 | (Q00623) Apolipoprotein A-I precursor (Apo-AI) (ApoA-I) | 4.3131E-05 | 2.0117E-05 | 47% | A, B, C |
| P14685 | PSD3_MOUSE | 530 | 60699 | 8.2 | (P14685) 26S proteasome non-ATPase regulatory subunit 3 (26S proteasome regulatory subunit S3) (Proteasome subunit p58) (Transplantation antigen P91A) (Tum-P91A antigen) | 4.2968E-05 | 2.0041E-05 | 47% | A, B, C |
| Q80X80 | TMM24_MOUSE | 706 | 76357 | 7.2 | (Q80X80) Transmembrane protein 24 | 4.2567E-05 | 6.5185E-06 | 15% | A, B, C |
| Q08122 | TLE3_MOUSE | 771 | 83212 | 7.1 | (Q08122) Transducin-like enhancer protein 3 (ESG) (Grg-3) | 4.1905E-05 | 2.9383E-05 | 70% | A, B, C |
| Q6NZF1 | ZC11A_MOUSE | 792 | 86492 | 8.1 | (Q6NZF1) Zinc finger CCCH domain-containing protein 11A | 4.1777E-05 | 4.2567E-06 | 10% | A, B, C |
| P46061 | RGP1_MOUSE | 589 | 63616 | 4.7 | (P46061) Ran GTPase-activating protein 1 | 4.1699E-05 | 7.3272E-06 | 18% | A, B, C |
| P47934 | CACP_MOUSE | 626 | 70925 | 8.3 | (P47934) Carnitine O-acetyltransferase (EC 2.3.1.7) (Carnitine acetylase) (CAT) (Carnitine acetyltransferase) (CrAT) | 4.1525E-05 | 1.8819E-05 | 45% | A, B, C |
| Q6URW6 | MYH14_MOUSE | 2000 | 228583 | 5.6 | (Q6URW6) Myosin-14 (Myosin heavy chain, nonmuscle IIc) (Nonmuscle myosin heavy chain IIc) (NMHC II-C) | 4.1411E-05 | 1.1611E-05 | 28% | A, B, C |
| Q7TQG1 | PKHA6_MOUSE | 1173 | 131427 | 9 | (Q7TQG1) Pleckstrin homology domain-containing family A member 6 (Phosphoinositol 3-phosphate-binding protein 3) (PEPP-3) | 4.1305E-05 | 4.3291E-05 | 105% | A, B, C |
| Q8K202 | RPF53_MOUSE | 482 | 54034 | 8.2 | (Q8K202) DNA-directed RNA polymerase I-associated factor 53 kDa subunit (EC 2.7.7.6) (RNA polymerase I-associated factor 1) | 4.1052E-05 | 2.1714E-05 | 53% | A, B, C |
| Q91X17 | UROM_MOUSE | 642 | 70845 | 4.9 | (Q91X17) Uromodulin precursor (Tamm-Horsfall urinary glycoprotein) (THP) | 4.0953E-05 | 1.9412E-05 | 47% | A, B, C |
| P39061 | COIA1_MOUSE | 1774 | 182230 | 5.6 | (P39061) Collagen alpha-1(XVIII) chain precursor [Contains: Endostatin] | 4.0897E-05 | 2.8042E-05 | 69% | A, B, C |
| P70699 | LYAG_MOUSE | 953 | 106248 | 5.8 | (P70699) Lysosomal alpha-glucosidase precursor (EC 3.2.1.20) (Acid maltase) | 4.0744E-05 | 1.2197E-05 | 30% | A, B, C |
| Q9D687 | S6A19_MOUSE | 634 | 71367 | 5.1 | (Q9D687) Sodium-dependent neutral amino acid transporter B(0) (System B(0) neutral amino acid transporter) (B(0)AT1) (Solute carrier family 6 member 19) | 4.0177E-05 | 3.2744E-05 | 81% | A, B, C |
| Q61838 | A2MG_MOUSE | 1495 | 165827 | 6.7 | (Q61838) Alpha-2-macroglobulin precursor (Alpha-2-M) [Contains: Alpha-2-macroglobulin 165 kDa subunit; Alpha-2-macroglobulin 35 kDa subunit] | 4.008E-05 | 1.4976E-05 | 37% | A, B, C |

| | | | | | | | | | |
|---|---|---|---|---|---|---|---|---|---|
| P06537 | GCR_MOUSE | 783 | 86053 | 6.4 | (P06537) Glucocorticoid receptor (GR) | 3.908E-05 | 4.4725E-06 | 11% | A, B, C |
| Q8R1N0 | CCD16_MOUSE | 363 | 40658 | 5.4 | (Q8R1N0) Coiled-coil domain-containing protein 16 (Ovus mutant candidate gene 1 protein) | 3.9021E-05 | 1.5104E-05 | 39% | A, B, C |
| P33175 | KIF5A_MOUSE | 1027 | 117019 | 5.9 | (P33175) Kinesin heavy chain isoform 5A (Neuronal kinesin heavy chain) (NKHC) (Kinesin heavy chain neuron-specific 1) | 3.7681E-05 | 1.1471E-05 | 30% | A, B, C |
| Q61136 | PRP4B_MOUSE | 1007 | 116948 | 10.2 | (Q61136) Serine/threonine-protein kinase PRP4 homolog (EC 2.7.11.1) (PRP4 pre-mRNA-processing factor 4 homolog) (Pre-mRNA protein kinase) | 3.7613E-05 | 1.636E-05 | 43% | A, B, C |
| Q9QXD1 | ACOX2_MOUSE | 681 | 76893 | 7.3 | (Q9QXD1) Acyl-coenzyme A oxidase 2, peroxisomal (EC 1.17.99.3) (3-alpha,7-alpha,12-alpha-trihydroxy-5-beta-cholestanoyl-CoA 24-hydroxylase) (3-alpha,7-alpha,12-alpha-trihydroxy-5-beta-cholestanoyl-CoA oxidase) (Trihydroxycoprostanoyl-CoA oxid | 3.7273E-05 | 6.0482E-06 | 16% | A, B, C |
| Q80WT5 | AFTIN_MOUSE | 931 | 101130 | 4.5 | (Q80WT5) Aftiphilin | 3.7139E-05 | 2.5478E-05 | 69% | A, B, C |
| Q8VDM6 | HNRL1_MOUSE | 859 | 96002 | 6.6 | (Q8VDM6) Heterogeneous nuclear ribonucleoprotein U-like protein 1 | 3.688E-05 | 1.7906E-05 | 49% | A, B, C |
| Q9JIK5 | DDX21_MOUSE | 851 | 93582 | 9.1 | (Q9JIK5) Nucleolar RNA helicase 2 (EC 3.6.1.-) (Nucleolar RNA helicase II) (Nucleolar RNA helicase Gu) (RH II/Gu) (Gu-alpha) (DEAD box protein 21) | 3.66E-05 | 1.3205E-05 | 36% | A, B, C |
| Q61545 | EWS_MOUSE | 655 | 68418 | 9.3 | (Q61545) RNA-binding protein EWS | 3.6023E-05 | 2.2318E-05 | 62% | A, B, C |
| P02469 | LAMB1_MOUSE | 1786 | 196904 | 4.9 | (P02469) Laminin beta-1 chain precursor (Laminin B1 chain) | 3.5759E-05 | 1.3217E-05 | 37% | A, B, C |
| Q99MZ3 | WBS14_MOUSE | 864 | 94875 | 7.9 | (Q99MZ3) Williams-Beuren syndrome chromosome region 14 protein homolog (Mlx interactor) (MLX-interacting protein-like) | 3.5416E-05 | 4.0532E-06 | 11% | A, B, C |
| P55014 | S12A1_MOUSE | 1095 | 120355 | 7.8 | (P55014) Solute carrier family 12 member 1 (Bumetanide-sensitive sodium-(potassium)-chloride cotransporter 2) (BSC1) (Kidney-specific Na-K-Cl symporter) | 3.5341E-05 | 1.0758E-05 | 30% | A, B, C |
| Q99KK7 | DPP3_MOUSE | 738 | 82911 | 5.3 | (Q99KK7) Dipeptidyl-peptidase 3 (EC 3.4.14.4) (Dipeptidyl-peptidase III) (DPP III) (Dipeptidyl aminopeptidase III) (Dipeptidyl arylamidase III) | 3.5309E-05 | 2.1794E-05 | 62% | A, B, C |
| O70570 | PIGR_MOUSE | 771 | 84999 | 5.4 | (O70570) Polymeric-immunoglobulin receptor precursor (Poly-Ig receptor) (PIGR) [Contains: Secretory component] | 3.5089E-05 | 3.5362E-05 | 101% | A, B, C |
| Q99MI1 | RB6I2_MOUSE | 1120 | 128330 | 5.9 | (Q99MI1) RAB6-interacting protein 2 (ERC protein 1) (ERC1) (CAZ-associated structural protein 2) (CAST2) | 3.5041E-05 | 1.6462E-05 | 47% | A, B, C |
| Q61146 | OCLN_MOUSE | 521 | 59000 | 6.5 | (Q61146) Occludin | 3.4621E-05 | 2.1688E-05 | 63% | A, B, C |
| Q8BGQ7 | SYA_MOUSE | 968 | 106908 | 5.7 | (Q8BGQ7) Alanyl-tRNA synthetase (EC 6.1.1.7) (Alanine--tRNA ligase) (AlaRS) | 3.4589E-05 | 8.7445E-06 | 25% | A, B, C |
| Q9ERU9 | RBP2_MOUSE | 3053 | 341092 | 6.2 | (Q9ERU9) Ran-binding protein 2 (RanBP2) | 3.4203E-05 | 1.4809E-05 | 43% | A, B, C |
| P97427 | DPYL1_MOUSE | 572 | 62168 | 7.1 | (P97427) Dihydropyrimidinase-related protein 1 (DRP-1) (Collapsin response mediator protein 1) (CRMP-1) (ULIP3 protein) | 3.3951E-05 | 3.0861E-06 | 9% | A, B, C |
| P49710 | HCLS1_MOUSE | 486 | 54212 | 4.8 | (P49710) Hematopoietic lineage cell-specific protein (Hematopoietic cell-specific LYN substrate 1) (LckBP1) | 3.395E-05 | 1.3853E-05 | 41% | A, B, C |
| P97496 | SMRC1_MOUSE | 1104 | 122890 | 5.7 | (P97496) SWI/SNF-related matrix-associated actin-dependent regulator of chromatin subfamily C member 1 (SWI/SNF complex 155 kDa subunit) (BRG1-associated factor 155) (SWI3-related protein) | 3.3762E-05 | 1.4039E-05 | 42% | A, B, C |

| | | | | | | | | | |
|---|---|---|---|---|---|---|---|---|---|
| Q8BTY1 | KAT1_MOUSE | 424 | 47564 | 6.9 | (Q8BTY1) Kynurenine--oxoglutarate transaminase 1 (EC 2.6.1.7) (Kynurenine--oxoglutarate transaminase I) (Kynurenine aminotransferase I) (KATI) (Glutamine--phenylpyruvate transaminase) (EC 2.6.1.64) (Glutamine transaminase K) (GTK) (Cysteine-S- | 3.3407E-05 | 1.2931E-05 | 39% | A, B, C |
| Q64462 | CP4B1_MOUSE | 511 | 58900 | 8.4 | (Q64462) Cytochrome P450 4B1 (EC 1.14.14.1) (CYPIVB1) | 3.2492E-05 | 1.6334E-05 | 50% | A, B, C |
| O55131 | SEPT7_MOUSE | 436 | 50550 | 8.6 | (O55131) Septin-7 (CDC10 protein homolog) | 3.2488E-05 | 1.2575E-05 | 39% | A, B, C |
| Q9ESZ8 | GTF2I_MOUSE | 998 | 112265 | 6.6 | (Q9ESZ8) General transcription factor II-I (GTFII-I) (TFII-I) (Bruton tyrosine kinase-associated protein 135) (BTK-associated protein 135) (BAP-135) | 3.2297E-05 | 2.2775E-05 | 71% | A, B, C |
| Q91YJ5 | IF2M_MOUSE | 727 | 81347 | 7.1 | (Q91YJ5) Translation initiation factor IF-2, mitochondrial precursor (IF-2Mt) (IF-2(Mt)) | 3.2221E-05 | 2.5747E-05 | 80% | A, B, C |
| Q7TSC1 | BAT2_MOUSE | 2158 | 229200 | 9.4 | (Q7TSC1) Large proline-rich protein BAT2 (HLA-B-associated transcript 2) | 3.1543E-05 | 2.7611E-05 | 88% | A, B, C |
| P02468 | LAMC1_MOUSE | 1607 | 177298 | 5.2 | (P02468) Laminin gamma-1 chain precursor (Laminin B2 chain) | 3.114E-05 | 1.0354E-05 | 33% | A, B, C |
| Q8CFX1 | G6PE_MOUSE | 789 | 88911 | 6.9 | (Q8CFX1) GDH/6PGL endoplasmic bifunctional protein precursor [Includes: Glucose 1-dehydrogenase (EC 1.1.1.47) (Hexose-6-phosphate dehydrogenase); 6-phosphogluconolactonase (EC 3.1.1.31) (6PGL)] | 3.1019E-05 | 5.6552E-06 | 18% | A, B, C |
| Q8K010 | OPLA_MOUSE | 1288 | 137611 | 6.3 | (Q8K010) 5-oxoprolinase (EC 3.5.2.9) (5-oxo-L-prolinase) (Pyroglutamase) (5-OPase) | 3.1009E-05 | 1.1024E-05 | 36% | A, B, C |
| O55029 | COPB2_MOUSE | 905 | 102449 | 5.3 | (O55029) Coatomer subunit beta' (Beta'-coat protein) (Beta'-COP) (p102) | 3.0928E-05 | 1.4603E-05 | 47% | A, B, C |
| P70168 | IMB1_MOUSE | 876 | 97152 | 4.8 | (P70168) Importin beta-1 subunit (Karyopherin beta-1 subunit) (Nuclear factor P97) (Pore targeting complex 97 kDa subunit) (PTAC97) (SCG) | 3.0514E-05 | 2.1771E-05 | 71% | A, B, C |
| P17717 | UDB5_MOUSE | 530 | 60856 | 7.9 | (P17717) UDP-glucuronosyltransferase 2B5 precursor (EC 2.4.1.17) (UDPGT) (M-1) | 3.0439E-05 | 1.0763E-05 | 35% | A, B, C |
| Q922J3 | REST_MOUSE | 1391 | 155813 | 5.2 | (Q922J3) Restin | 3.0296E-05 | 2.0162E-05 | 67% | A, B, C |
| Q99MR6 | ARS2_MOUSE | 875 | 100452 | 6 | (Q99MR6) Arsenite-resistance protein 2 | 3.0112E-05 | 1.5581E-05 | 52% | A, B, C |
| Q9Z1K5 | ARI1_MOUSE | 555 | 64017 | 5.1 | (Q9Z1K5) Protein ariadne-1 homolog (ARI-1) (Ubiquitin-conjugating enzyme E2-binding protein 1) (UbcH7-binding protein) (UbcM4-interacting protein 77) | 2.9916E-05 | 1.5039E-05 | 50% | A, B, C |
| Q8BIQ5 | CSTF2_MOUSE | 580 | 61341 | 6.8 | (Q8BIQ5) Cleavage stimulation factor 64 kDa subunit (CSTF 64 kDa subunit) (CF-1 64 kDa subunit) (CstF-64) | 2.957E-05 | 2.6149E-05 | 88% | A, B, C |
| Q8CHC4 | SYNJ1_MOUSE | 1574 | 172616 | 6.9 | (Q8CHC4) Synaptojanin-1 (EC 3.1.3.36) (Synaptic inositol-1,4,5-trisphosphate 5-phosphatase 1) | 2.9515E-05 | 1.2862E-05 | 44% | A, B, C |
| Q9QWI6 | SNIP_MOUSE | 1250 | 134858 | 9.3 | (Q9QWI6) p130Cas-associated protein (p140Cap) (SNAP-25-interacting protein) (SNIP) | 2.9381E-05 | 6.2737E-06 | 21% | A, B, C |
| Q9JLB0 | MPP6_MOUSE | 553 | 62631 | 6.4 | (Q9JLB0) MAGUK p55 subfamily member 6 (Protein associated with Lin-7 2) (Dlgh4 protein) (P55T protein) | 2.9035E-05 | 1.0267E-05 | 35% | A, B, C |
| Q52KI8 | SRRM1_MOUSE | 946 | 106892 | 11.9 | (Q52KI8) Serine/arginine repetitive matrix protein 1 (Plenty-of-prolines 101) | 2.8431E-05 | 8.1334E-06 | 29% | A, B, C |
| Q3TJZ6 | FA98A_MOUSE | 515 | 55055 | 9 | (Q3TJZ6) Protein FAM98A | 2.7504E-05 | 1.0646E-05 | 39% | A, B, C |
| Q8C863 | ITCH_MOUSE | 864 | 98994 | 6.3 | (Q8C863) Itchy E3 ubiquitin protein ligase (EC 6.3.2.-) | 2.7309E-05 | 1.692E-05 | 62% | A, B, C |
| Q7TQK5 | CCD93_MOUSE | 629 | 72603 | 8.3 | (Q7TQK5) Coiled-coil domain-containing protein 93 | 2.7162E-05 | 4.5824E-06 | 17% | A, B, C |

| | | | | | | | | | |
|---|---|---|---|---|---|---|---|---|---|
| O08529 | CAN2_MOUSE | 699 | 79741 | 5 | (O08529) Calpain-2 catalytic subunit precursor (EC 3.4.22.53) (Calpain-2 large subunit) (Calcium-activated neutral proteinase 2) (CANP 2) (Calpain M-type) (M-calpain) (Millimolar-calpain) (80 kDa M-calpain subunit) (CALP80) | 2.642E-05 | 1.304E-05 | 49% | A, B, C |
| Q99KY4 | GAK_MOUSE | 1305 | 143640 | 5.7 | (Q99KY4) Cyclin G-associated kinase (EC 2.7.11.1) | 2.6353E-05 | 9.0293E-06 | 34% | A, B, C |
| Q99KU1 | DHDDS_MOUSE | 333 | 38509 | 8.3 | (Q99KU1) Dehydrodolichyl diphosphate synthase (EC 2.5.1.-) (Dedol-PP synthase) | 2.5653E-05 | 4.3279E-06 | 17% | A, B, C |
| Q61292 | LAMB2_MOUSE | 1799 | 196352 | 6.7 | (Q61292) Laminin beta-2 chain precursor (S-laminin) (S-LAM) | 2.5508E-05 | 1.3898E-05 | 54% | A, B, C |
| O70133 | DHX9_MOUSE | 1380 | 149474 | 6.8 | (O70133) ATP-dependent RNA helicase A (EC 3.6.1.-) (Nuclear DNA helicase II) (NDH II) (DEAH box protein 9) (mHEL-5) | 2.5065E-05 | 1.5923E-05 | 64% | A, B, C |
| Q8BIL5 | HOOK1_MOUSE | 728 | 84439 | 5.2 | (Q8BIL5) Hook homolog 1 | 2.4598E-05 | 1.5247E-05 | 62% | A, B, C |
| Q61191 | HCFC1_MOUSE | 2045 | 210535 | 7.3 | (Q61191) Host cell factor (HCF) (HCF-1) (C1 factor) [Contains: HCF N-terminal chain 1; HCF N-terminal chain 2; HCF N-terminal chain 3; HCF N-terminal chain 4; HCF N-terminal chain 5; HCF N-terminal chain 6; HCF C-terminal chain 1; HCF C-termi | 2.4561E-05 | 1.5412E-05 | 63% | A, B, C |
| P14824 | ANXA6_MOUSE | 672 | 75755 | 5.5 | (P14824) Annexin A6 (Annexin VI) (Lipocortin VI) (P68) (P70) (Protein III) (Chromobindin-20) (67 kDa calelectrin) (Calphobindin-II) (CPB-II) | 2.4424E-05 | 9.9278E-06 | 41% | A, B, C |
| Q8BP47 | SYNC_MOUSE | 547 | 63066 | 6 | (Q8BP47) Asparaginyl-tRNA synthetase, cytoplasmic (EC 6.1.1.22) (Asparagine--tRNA ligase) (AsnRS) | 2.4154E-05 | 1.2392E-05 | 51% | A, B, C |
| P11276 | FINC_MOUSE | 2477 | 272487 | 5.6 | (P11276) Fibronectin precursor (FN) | 2.3989E-05 | 2.1549E-05 | 90% | A, B, C |
| O35286 | DHX15_MOUSE | 795 | 91007 | 7.5 | (O35286) Putative pre-mRNA-splicing factor ATP-dependent RNA helicase DHX15 (EC 3.6.1.-) (DEAH box protein 15) | 2.3966E-05 | 1.0547E-05 | 44% | A, B, C |
| Q8BMJ2 | SYLC_MOUSE | 1178 | 134192 | 7.1 | (Q8BMJ2) Leucyl-tRNA synthetase, cytoplasmic (EC 6.1.1.4) (Leucine--tRNA ligase) (LeuRS) | 2.3917E-05 | 2.1006E-05 | 88% | A, B, C |
| Q61165 | SL9A1_MOUSE | 820 | 91468 | 7.1 | (Q61165) Sodium/hydrogen exchanger 1 (Na(+)/H(+) exchanger 1) (NHE-1) (Solute carrier family 9 member 1) | 2.3236E-05 | 1.0225E-05 | 44% | A, B, C |
| Q9WV27 | AT1A4_MOUSE | 1032 | 114816 | 5.7 | (Q9WV27) Sodium/potassium-transporting ATPase alpha-4 chain (EC 3.6.3.9) (Sodium pump 4) (Na+/K+ ATPase 4) | 2.3071E-05 | 2.1007E-05 | 91% | A, B, C |
| Q80VC9 | K1543_MOUSE | 1252 | 135175 | 8.4 | (Q80VC9) Protein KIAA1543 | 2.2865E-05 | 7.3911E-06 | 32% | A, B, C |
| Q9CQF9 | PCYOX_MOUSE | 505 | 56495 | 6.9 | (Q9CQF9) Prenylcysteine oxidase precursor (EC 1.8.3.5) | 2.2547E-05 | 1.0517E-05 | 47% | A, B, C |
| Q9JLB2 | MPP5_MOUSE | 675 | 77230 | 6.1 | (Q9JLB2) MAGUK p55 subfamily member 5 (Protein associated with Lin-7 1) | 2.2396E-05 | 1.8609E-05 | 83% | A, B, C |
| Q9JJG0 | TACC2_MOUSE | 1035 | 112716 | 5 | (Q9JJG0) Transforming acidic coiled-coil-containing protein 2 | 2.1501E-05 | 3.4545E-06 | 16% | A, B, C |
| P59808 | SASH1_MOUSE | 1230 | 135591 | 6.2 | (P59808) SAM and SH3 domain-containing protein 1 | 2.1256E-05 | 1.6616E-05 | 78% | A, B, C |
| P28738 | KIF5C_MOUSE | 956 | 109240 | 6.2 | (P28738) Kinesin heavy chain isoform 5C (Kinesin heavy chain neuron-specific 2) | 2.0234E-05 | 1.9763E-06 | 10% | A, B, C |
| Q9Z1X4 | ILF3_MOUSE | 898 | 96021 | 8.8 | (Q9Z1X4) Interleukin enhancer-binding factor 3 | 1.9913E-05 | 1.6542E-05 | 83% | A, B, C |
| Q63ZW7 | INADL_MOUSE | 1834 | 198515 | 5 | (Q63ZW7) InaD-like protein (Inadl protein) (Pals1-associated tight junction protein) (Protein associated to tight junctions) (Channel-interacting PDZ domain-containing protein) | 1.963E-05 | 1.7014E-05 | 87% | A, B, C |
| Q99P72 | RTN4_MOUSE | 1162 | 126613 | 4.5 | (Q99P72) Reticulon-4 (Neurite outgrowth inhibitor) (Nogo protein) | 1.891E-05 | 1.086E-05 | 57% | A, B, C |
| Q6P5E4 | UGGG1_MOUSE | 1527 | 173909 | 5.6 | (Q6P5E4) UDP-glucose:glycoprotein glucosyltransferase 1 precursor (EC 2.4.1.-) (UDP-glucose ceramide glucosyltransferase-like 1) (UDP--Glc:glycoprotein glucosyltransferase) | 1.851E-05 | 4.4752E-06 | 24% | A, B, C |

| | | | | | | | | | |
|---|---|---|---|---|---|---|---|---|---|
| P70336 | ROCK2_MOUSE | 1388 | 160585 | 6 | (P70336) Rho-associated protein kinase 2 (EC 2.7.11.1) (Rho-associated, coiled-coil-containing protein kinase 2) (p164 ROCK-2) | 1.8352E-05 | 1.4957E-05 | 81% | A, B, C |
| P01029 | CO4B_MOUSE | 1738 | 192870 | 7.6 | (P01029) Complement C4-B precursor [Contains: Complement C4 beta chain; Complement C4 alpha chain; C4a anaphylatoxin; Complement C4 gamma chain] | 1.7309E-05 | 1.2191E-05 | 70% | A, B, C |
| Q7TMY8 | HUWE1_MOUSE | 4377 | 482668 | 5.2 | (Q7TMY8) HECT, UBA and WWE domain-containing protein 1 (EC 6.3.2.-) (E3 ubiquitin protein ligase URE-B1) (E3Histone) | 1.6788E-05 | 8.9988E-06 | 54% | A, B, C |
| Q6NZJ6 | IF4G1_MOUSE | 1600 | 176076 | 5.4 | (Q6NZJ6) Eukaryotic translation initiation factor 4 gamma 1 (eIF-4-gamma 1) (eIF-4G1) (eIF-4G 1) | 1.6663E-05 | 1.0486E-05 | 63% | A, B, C |
| Q61026 | NCOA2_MOUSE | 1462 | 158511 | 6.7 | (Q61026) Nuclear receptor coactivator 2 (NCoA-2) (Transcriptional intermediary factor 2) (Glucocorticoid receptor-interacting protein 1) (GRIP-1) | 1.6634E-05 | 1.4841E-05 | 89% | A, B, C |
| Q8BY87 | UBP47_MOUSE | 1376 | 157455 | 5.1 | (Q8BY87) Ubiquitin carboxyl-terminal hydrolase 47 (EC 3.1.2.15) (Ubiquitin thioesterase 47) (Ubiquitin-specific-processing protease 47) (Deubiquitinating enzyme 47) | 1.6152E-05 | 2.3839E-06 | 15% | A, B, C |
| P55937 | GOGA3_MOUSE | 1487 | 167219 | 5.4 | (P55937) Golgin subfamily A member 3 (Golgin-160) (Male-enhanced antigen 2) (MEA-2) | 1.5868E-05 | 9.8308E-06 | 62% | A, B, C |
| Q9CZU3 | SK2L2_MOUSE | 1040 | 117636 | 6.4 | (Q9CZU3) Superkiller viralicidic activity 2-like 2 (EC 3.6.1.-) (ATP-dependent helicase SKIV2L2) | 1.5781E-05 | 6.4149E-06 | 41% | A, B, C |
| Q68FL6 | SYM_MOUSE | 902 | 101431 | 7.1 | (Q68FL6) Methionyl-tRNA synthetase (EC 6.1.1.10) (Methionine-tRNA ligase) (MetRS) | 1.5297E-05 | 4.2514E-06 | 28% | A, B, C |
| Q80UJ7 | RB3GP_MOUSE | 980 | 109967 | 5.7 | (Q80UJ7) Rab3 GTPase-activating protein catalytic subunit (RAB3 GTPase-activating protein 130 kDa subunit) (Rab3-GAP p130) (Rab3-GAP) | 1.5051E-05 | 6.9365E-06 | 46% | A, B, C |
| Q99NB9 | SF3B1_MOUSE | 1304 | 145816 | 7.1 | (Q99NB9) Splicing factor 3B subunit 1 (Spliceosome-associated protein 155) (SAP 155) (SF3b155) (Pre-mRNA-splicing factor SF3b 155 kDa subunit) | 1.4826E-05 | 1.3414E-06 | 9% | A, B, C |
| Q62165 | DAG1_MOUSE | 893 | 96905 | 8.4 | (Q62165) Dystroglycan precursor (Dystrophin-associated glycoprotein 1) [Contains: Alpha-dystroglycan (Alpha-DG); Beta-dystroglycan (Beta-DG)] | 1.4795E-05 | 7.5908E-06 | 51% | A, B, C |
| Q9QXL2 | KI21A_MOUSE | 1672 | 186535 | 6.3 | (Q9QXL2) Kinesin family member 21A | 1.4774E-05 | 1.016E-05 | 69% | A, B, C |
| P28665 | MUG1_MOUSE | 1476 | 165139 | 6.5 | (P28665) Murinoglobulin-1 precursor (MuG1) | 1.433E-05 | 1.1021E-05 | 77% | A, B, C |
| Q6P9Q4 | FHOD1_MOUSE | 1196 | 129469 | 6.3 | (Q6P9Q4) FH1/FH2 domain-containing protein (Formin homolog overexpressed in spleen) (FHOS) (Formin homology 2 domain-containing protein 1) | 1.4285E-05 | 2.41E-06 | 17% | A, B, C |
| Q9QX47 | SON_MOUSE | 2404 | 261428 | 5.6 | (Q9QX47) SON protein | 1.4155E-05 | 6.6516E-06 | 47% | A, B, C |
| Q91VS8 | FARP2_MOUSE | 1065 | 121297 | 8.4 | (Q91VS8) FERM, RhoGEF and pleckstrin domain-containing protein 2 (FERM domain including RhoGEF) (FIR) | 1.3398E-05 | 9.5847E-06 | 72% | A, B, C |
| Q61001 | LAMA5_MOUSE | 3718 | 404013 | 6.7 | (Q61001) Laminin alpha-5 chain precursor | 1.3323E-05 | 1.4945E-05 | 112% | A, B, C |
| P11881 | ITPR1_MOUSE | 2749 | 313196 | 6.1 | (P11881) Inositol 1,4,5-trisphosphate receptor type 1 (Type 1 inositol 1,4,5-trisphosphate receptor) (Type 1 InsP3 receptor) (IP3 receptor isoform 1) (InsP3R1) (Inositol 1,4,5-trisphosphate-binding protein P400) (Purkinje cell protein 1) (Pro | 1.3309E-05 | 8.6003E-06 | 65% | A, B, C |
| Q68FH0 | PKP4_MOUSE | 1190 | 131551 | 8.9 | (Q68FH0) Plakophilin-4 (Armadillo-related protein) | 1.3065E-05 | 9.0876E-06 | 70% | A, B, C |
| Q9QXZ0 | MACF1_MOUSE | 5327 | 607984 | 5.5 | (Q9QXZ0) Microtubule-actin crosslinking factor 1 (Actin cross-linking family 7) | 1.2974E-05 | 1.809E-07 | 1% | A, B, C |
| P11531 | DMD_MOUSE | 3678 | 425819 | 5.9 | (P11531) Dystrophin | 1.2593E-05 | 6.8095E-06 | 54% | A, B, C |

| | | | | | | | | | |
|---|---|---|---|---|---|---|---|---|---|
| Q91ZU6 | BPA1_MOUSE | 7389 | 833654 | 5.3 | (Q91ZU6) Bullous pemphigoid antigen 1, isoforms 1/2/3/4 (BPA) (Hemidesmosomal plaque protein) (Dystonia musculorum protein) (Dystonin) | 1.1714E-05 | 2.5002E-06 | 21% | A, B, C |
| Q6PIC6 | AT1A3_MOUSE | 1013 | 111691 | 5.4 | (Q6PIC6) Sodium/potassium-transporting ATPase alpha-3 chain (EC 3.6.3.9) (Sodium pump 3) (Na+/K+ ATPase 3) (Alpha(III)) | 1.0738E-05 | 2.7319E-06 | 25% | A, B, C |
| Q6PB44 | PTN23_MOUSE | 1692 | 185215 | 6.8 | (Q6PB44) Tyrosine-protein phosphatase non-receptor type 23 (EC 3.1.3.48) | 9.1886E-06 | 6.3914E-06 | 70% | A, B, C |
| Q8R0F8 | FAHD1_MOUSE | 227 | 25158 | 7.7 | (Q8R0F8) Fumarylacetoacetate hydrolase domain-containing protein 1 (EC 3.-.-.-) | 0.00195053 | 0.00227365 | 117% | A, B |
| P32848 | PRVA_MOUSE | 109 | 11799 | 5.2 | (P32848) Parvalbumin alpha | 0.00144279 | 0.00166163 | 115% | A, B |
| P56395 | CYB5_MOUSE | 133 | 15110 | 5.1 | (P56395) Cytochrome b5 | 0.00102004 | 0.0003043 | 30% | A, B |
| Q91V76 | CK054_MOUSE | 315 | 34996 | 6.3 | (Q91V76) Ester hydrolase C11orf54 homolog (EC 3.1.-.-) | 0.00085297 | 0.00093455 | 110% | A, B |
| Q8K1Z0 | COQ9_MOUSE | 313 | 35083 | 5.9 | (Q8K1Z0) Ubiquinone biosynthesis protein COQ9, mitochondrial precursor | 0.00075464 | 0.00014385 | 19% | A, B |
| Q8CAY6 | THIC_MOUSE | 397 | 41298 | 7.5 | (Q8CAY6) Acetyl-CoA acetyltransferase, cytosolic (EC 2.3.1.9) (Cytosolic acetoacetyl-CoA thiolase) | 0.00051464 | 0.00010381 | 20% | A, B |
| Q9CQX8 | RT36_MOUSE | 102 | 11101 | 10 | (Q9CQX8) Mitochondrial 28S ribosomal protein S36 (S36mt) (MRP-S36) | 0.00049815 | 0.00010508 | 21% | A, B |
| Q9QYJ0 | DNJA2_MOUSE | 412 | 45746 | 6.5 | (Q9QYJ0) DnaJ homolog subfamily A member 2 (mDj3) | 0.00045134 | 0.00016341 | 36% | A, B |
| P63028 | TCTP_MOUSE | 172 | 19462 | 4.9 | (P63028) Translationally-controlled tumor protein (TCTP) (p23) (21 kDa polypeptide) (p21) | 0.00044143 | 0.00029993 | 68% | A, B |
| Q9DCM0 | ETHE1_MOUSE | 254 | 27739 | 7.2 | (Q9DCM0) ETHE1 protein, mitochondrial precursor (EC 3.-.-.-) (Ethylmalonic encephalopathy protein 1 homolog) (Hepatoma subtracted clone one protein) | 0.00040649 | 0.00023788 | 59% | A, B |
| Q9CQC7 | NDUB4_MOUSE | 128 | 14950 | 9.9 | (Q9CQC7) NADH dehydrogenase [ubiquinone] 1 beta subcomplex subunit 4 (EC 1.6.5.3) (EC 1.6.99.3) (NADH-ubiquinone oxidoreductase B15 subunit) (Complex I-B15) (CI-B15) | 0.00037635 | 0.00043544 | 116% | A, B |
| P51855 | GSHB_MOUSE | 474 | 52247 | 5.8 | (P51855) Glutathione synthetase (EC 6.3.2.3) (Glutathione synthase) (GSH synthetase) (GSH-S) | 0.00037406 | 0.00016784 | 45% | A, B |
| P68037 | UB2L3_MOUSE | 154 | 17862 | 8.5 | (P68037) Ubiquitin-conjugating enzyme E2 L3 (EC 6.3.2.19) (Ubiquitin-protein ligase L3) (Ubiquitin carrier protein L3) (UbcM4) | 0.00036025 | 2.673E-05 | 7% | A, B |
| P63024 | VAMP3_MOUSE | 103 | 11480 | 8.5 | (P63024) Vesicle-associated membrane protein 3 (VAMP-3) (Synaptobrevin-3) (Cellubrevin) (CEB) | 0.00034759 | 0.00022075 | 64% | A, B |
| P47791 | GSHR_MOUSE | 500 | 53663 | 8 | (P47791) Glutathione reductase, mitochondrial precursor (EC 1.8.1.7) (GR) (GRase) | 0.00032752 | 0.0002705 | 83% | A, B |
| Q9D855 | UCR6_MOUSE | 110 | 13396 | 9.1 | (Q9D855) Ubiquinol-cytochrome c reductase complex 14 kDa protein (EC 1.10.2.2) (Complex III subunit VI) | 0.00032428 | 0.00020838 | 64% | A, B |
| Q9CWH6 | PSA7L_MOUSE | 250 | 27866 | 8.7 | (Q9CWH6) Proteasome subunit alpha type 7-like (EC 3.4.25.1) | 0.00030483 | 0.00015585 | 51% | A, B |
| Q6ZWN5 | RS9_MOUSE | 193 | 22460 | 10.7 | (Q6ZWN5) 40S ribosomal protein S9 | 0.00027195 | 0.00031233 | 115% | A, B |
| Q8VCC2 | EST1_MOUSE | 565 | 62680 | 6 | (Q8VCC2) Liver carboxylesterase 1 precursor (EC 3.1.1.1) (Acyl coenzyme A:cholesterol acyltransferase) (ES-x) | 0.00026661 | 0.00022894 | 86% | A, B |
| P28667 | MRP_MOUSE | 199 | 20034 | 4.6 | (P28667) MARCKS-related protein (MARCKS-like protein 1) (Macrophage myristoylated alanine-rich C kinase substrate) (Mac-MARCKS) (MacMARCKS) (Brain protein F52) | 0.00026653 | 0.00031466 | 118% | A, B |

| | | | | | | | | | |
|---|---|---|---|---|---|---|---|---|---|
| O35683 | NDUA1_MOUSE | 70 | 8139 | 9.5 | (O35683) NADH dehydrogenase [ubiquinone] 1 alpha subcomplex subunit 1 (EC 1.6.5.3) (EC 1.6.99.3) (NADH-ubiquinone oxidoreductase MWFE subunit) (Complex I-MWFE) (CI-MWFE) | 0.00026605 | 2.2235E-05 | 8% | A, B |
| Q9D1J3 | HCC1_MOUSE | 209 | 23401 | 6.7 | (Q9D1J3) Nuclear protein Hcc-1 | 0.0002612 | 4.0151E-05 | 15% | A, B |
| P61967 | AP1S1_MOUSE | 158 | 18733 | 5.7 | (P61967) AP-1 complex subunit sigma-1A (Adapter-related protein complex 1 sigma-1A subunit) (Sigma-adaptin 1A) (Adaptor protein complex AP-1 sigma-1A subunit) (Golgi adaptor HA1/AP1 adaptin sigma-1A subunit) (Clathrin assembly protein complex | 0.00026 | 1.9271E-05 | 7% | A, B |
| P56873 | SSA27_MOUSE | 199 | 21336 | 5.1 | (P56873) Sjoegren syndrome/scleroderma autoantigen 1 homolog (Autoantigen p27 homolog) (Protein C184L) | 0.00024988 | 7.6747E-05 | 31% | A, B |
| Q9ET26 | ZN313_MOUSE | 229 | 25745 | 7 | (Q9ET26) Zinc finger protein 313 | 0.00024808 | 0.00028993 | 117% | A, B |
| Q9CR88 | RT14_MOUSE | 128 | 14920 | 11.4 | (Q9CR88) Mitochondrial 28S ribosomal protein S14 (S14mt) (MRP-S14) | 0.0002449 | 0.00013131 | 54% | A, B |
| Q8K1L5 | PP1RB_MOUSE | 131 | 14544 | 6.1 | (Q8K1L5) Protein phosphatase 1 regulatory subunit 11 (T-complex testis expressed protein 5) (Tctex-5) | 0.00024248 | 0.00012999 | 54% | A, B |
| P52503 | NUMM_MOUSE | 116 | 13020 | 8.7 | (P52503) NADH-ubiquinone oxidoreductase 13 kDa-A subunit, mitochondrial precursor (EC 1.6.5.3) (EC 1.6.99.3) (Complex I-13KD-A) (CI-13KD-A) | 0.00023409 | 9.0595E-05 | 39% | A, B |
| P61082 | UBC12_MOUSE | 183 | 20900 | 7.7 | (P61082) NEDD8-conjugating enzyme Ubc12 (EC 6.3.2.-) (Ubiquitin-conjugating enzyme E2 M) (NEDD8 protein ligase) (NEDD8 carrier protein) | 0.00022258 | 8.614E-05 | 39% | A, B |
| Q9WV96 | TIM9B_MOUSE | 100 | 11314 | 7.1 | (Q9WV96) Mitochondrial import inner membrane translocase subunit Tim9 B (TIMM10B) (Tim10b) | 0.00022224 | 0.00017482 | 79% | A, B |
| Q9CQD1 | RAB5A_MOUSE | 215 | 23599 | 8.1 | (Q9CQD1) Ras-related protein Rab-5A | 0.00022087 | 0.00014173 | 64% | A, B |
| P22599 | A1AT2_MOUSE | 413 | 45975 | 5.5 | (P22599) Alpha-1-antitrypsin 1-2 precursor (Serine protease inhibitor 1-2) (Alpha-1 protease inhibitor 2) (Alpha-1-antiproteinase) (AAT) | 0.00021465 | 9.6313E-05 | 45% | A, B |
| P84099 | RL19_MOUSE | 196 | 23466 | 11.5 | (P84099) 60S ribosomal protein L19 | 0.00021353 | 4.911E-05 | 23% | A, B |
| Q9D7X3 | DUS3_MOUSE | 185 | 20472 | 6.5 | (Q9D7X3) Dual specificity protein phosphatase 3 (EC 3.1.3.48) (EC 3.1.3.16) (T-DSP11) | 0.00020133 | 1.6827E-05 | 8% | A, B |
| O35465 | FKBP8_MOUSE | 355 | 38615 | 7 | (O35465) 38 kDa FK506-binding protein homolog (FKBPR38) (FK506-binding protein 8) (muFKBP38) | 0.0001949 | 3.4514E-05 | 18% | A, B |
| Q9WVA2 | TIM8A_MOUSE | 97 | 11042 | 5.2 | (Q9WVA2) Mitochondrial import inner membrane translocase subunit Tim8 A (Deafness dystonia protein 1 homolog) | 0.00019199 | 1.6046E-05 | 8% | A, B |
| Q9D1J1 | NECP2_MOUSE | 266 | 28598 | 8 | (Q9D1J1) Adaptin ear-binding coat-associated protein 2 (NECAP-2) | 0.00019102 | 4.0292E-05 | 21% | A, B |
| Q8BK64 | AHSA1_MOUSE | 338 | 38117 | 5.5 | (Q8BK64) Activator of 90 kDa heat shock protein ATPase homolog 1 (AHA1) | 0.0001727 | 8.1363E-05 | 47% | A, B |
| P53810 | PIPNA_MOUSE | 270 | 31762 | 6.4 | (P53810) Phosphatidylinositol transfer protein alpha isoform (PtdIns transfer protein alpha) (PtdInsTP) (PI-TP-alpha) | 0.00015616 | 8.4984E-05 | 54% | A, B |
| P21614 | VTDB_MOUSE | 476 | 53600 | 5.5 | (P21614) Vitamin D-binding protein precursor (DBP) (Group-specific component) (Gc-globulin) (VDB) | 0.00015568 | 0.00019086 | 123% | A, B |
| Q9DCZ1 | GMPR1_MOUSE | 345 | 37482 | 7.1 | (Q9DCZ1) GMP reductase 1 (EC 1.7.1.7) (Guanosine 5'-monophosphate oxidoreductase 1) (Guanosine monophosphate reductase 1) | 0.00015213 | 0.00017473 | 115% | A, B |
| Q8BL97 | SFRS7_MOUSE | 267 | 30818 | 11.9 | (Q8BL97) Splicing factor, arginine/serine-rich 7 | 0.00014979 | 0.00010875 | 73% | A, B |
| Q9D1H6 | CF066_MOUSE | 173 | 20082 | 9.4 | (Q9D1H6) UPF0240 protein C6orf66 homolog | 0.00013615 | 4.9306E-05 | 36% | A, B |
| P62858 | RS28_MOUSE | 69 | 7841 | 10.7 | (P62858) 40S ribosomal protein S28 | 0.00013495 | 1.1279E-05 | 8% | A, B |

| | | | | | | | | | |
|---|---|---|---|---|---|---|---|---|---|
| Q9EQS3 | MYCBP_MOUSE | 102 | 11824 | 5.9 | (Q9EQS3) C-Myc-binding protein (Associate of Myc 1) (AMY-1) | 0.00013424 | 5.3107E-05 | 40% | A, B |
| Q99K30 | ES8L2_MOUSE | 729 | 82229 | 7.2 | (Q99K30) Epidermal growth factor receptor kinase substrate 8-like protein 2 (Epidermal growth factor receptor pathway substrate 8-related protein 2) (EPS8-like protein 2) | 0.00012605 | 1.0525E-05 | 8% | A, B |
| P11930 | NUD19_MOUSE | 357 | 40368 | 6.7 | (P11930) Nucleoside diphosphate-linked moiety X motif 19 (EC 3.-.-.-) (Nudix motif 19) (Testosterone-regulated RP2 protein) (Androgen-regulated protein RP2) | 0.00012566 | 6.2056E-05 | 49% | A, B |
| Q91VI7 | RINI_MOUSE | 456 | 49817 | 4.8 | (Q91VI7) Ribonuclease inhibitor (Ribonuclease/angiogenin inhibitor 1) | 0.00012329 | 6.7093E-05 | 54% | A, B |
| Q8VCM7 | FIBG_MOUSE | 436 | 49391 | 5.9 | (Q8VCM7) Fibrinogen gamma chain precursor | 0.00012272 | 7.8858E-05 | 64% | A, B |
| O35435 | PYRD_MOUSE | 395 | 42700 | 9.6 | (O35435) Dihydroorotate dehydrogenase, mitochondrial precursor (EC 1.3.3.1) (Dihydroorotate oxidase) (DHOdehase) | 0.00011769 | 4.2612E-05 | 36% | A, B |
| Q9JJK5 | HERPU_MOUSE | 391 | 43907 | 5.5 | (Q9JJK5) Homocysteine-responsive endoplasmic reticulum-resident ubiquitin-like domain member 1 protein | 0.00011767 | 2.3727E-05 | 20% | A, B |
| Q64737 | PUR2_MOUSE | 1010 | 107395 | 6.7 | (Q64737) Trifunctional purine biosynthetic protein adenosine-3 [Includes: Phosphoribosylamine--glycine ligase (EC 6.3.4.13) (GARS) (Glycinamide ribonucleotide synthetase) (Phosphoribosylglycinamide synthetase); Phosphoribosylformylglycinamidin | 0.00011668 | 8.0262E-05 | 69% | A, B |
| Q61337 | BAD_MOUSE | 204 | 22080 | 9.2 | (Q61337) Bcl2 antagonist of cell death (BAD) (Bcl-2-binding component 6) (Bcl-xL/Bcl-2-associated death promoter) | 0.00011546 | 4.1813E-05 | 36% | A, B |
| O08600 | NUCG_MOUSE | 294 | 32191 | 9.5 | (O08600) Endonuclease G, mitochondrial precursor (EC 3.1.30.-) (Endo G) | 0.00010847 | 1.2966E-05 | 12% | A, B |
| Q6NZB0 | DNJC8_MOUSE | 253 | 29813 | 9.1 | (Q6NZB0) DnaJ homolog subfamily C member 8 | 0.00010824 | 4.2822E-05 | 40% | A, B |
| Q9DBS5 | KLC4_MOUSE | 619 | 68613 | 6.1 | (Q9DBS5) Kinesin light chain 4 (KLC 4) (Kinesin-like protein 8) | 0.00010742 | 9.2653E-05 | 86% | A, B |
| P97765 | WBP2_MOUSE | 261 | 28032 | 6.3 | (P97765) WW domain-binding protein 2 (WBP-2) | 0.00010703 | 8.9453E-06 | 8% | A, B |
| P08752 | GNAI2_MOUSE | 354 | 40340 | 5.5 | (P08752) Guanine nucleotide-binding protein G(i), alpha-2 subunit (Adenylate cyclase-inhibiting G alpha protein) | 0.00010522 | 8.7937E-06 | 8% | A, B |
| Q61550 | RAD21_MOUSE | 635 | 72023 | 4.6 | (Q61550) Double-strand-break repair protein rad21 homolog (Pokeweed agglutinin-binding protein 29) (PW29) (SCC1 homolog) | 0.00010386 | 6.9853E-05 | 67% | A, B |
| Q8K183 | PDXK_MOUSE | 312 | 35015 | 6.3 | (Q8K183) Pyridoxal kinase (EC 2.7.1.35) (Pyridoxine kinase) | 9.8943E-05 | 9.5226E-05 | 96% | A, B |
| Q8BTV2 | CPSF7_MOUSE | 471 | 52011 | 8 | (Q8BTV2) Cleavage and polyadenylation specificity factor 7 | 9.87E-05 | 3.5736E-05 | 36% | A, B |
| Q7TNV0 | DEK_MOUSE | 380 | 43159 | 6.9 | (Q7TNV0) Protein DEK | 9.8608E-05 | 4.1634E-05 | 42% | A, B |
| P23198 | CBX3_MOUSE | 183 | 20855 | 5.2 | (P23198) Chromobox protein homolog 3 (Heterochromatin protein 1 homolog gamma) (HP1 gamma) (Modifier 2 protein) (M32) | 9.7818E-05 | 6.2123E-05 | 64% | A, B |
| Q60692 | PSB6_MOUSE | 238 | 25379 | 5.1 | (Q60692) Proteasome subunit beta type 6 precursor (EC 3.4.25.1) (Proteasome delta chain) (Macropain delta chain) (Multicatalytic endopeptidase complex delta chain) (Proteasome subunit Y) | 9.7663E-05 | 3.5361E-05 | 36% | A, B |
| Q01147 | CREB1_MOUSE | 341 | 36674 | 5.6 | (Q01147) cAMP response element-binding protein (CREB) | 9.5111E-05 | 2.693E-05 | 28% | A, B |
| Q9JHL1 | NHRF2_MOUSE | 337 | 37393 | 7.6 | (Q9JHL1) Na(+)/H(+) exchange regulatory cofactor NHE-RF2 (NHERF-2) (Tyrosine kinase activator protein 1) (TKA-1) (SRY-interacting protein 1) (SIP-1) (Solute carrier family 9 isoform A3 regulatory factor 2) (NHE3 kinase A regulatory protein E3 | 9.463E-05 | 1.1312E-05 | 12% | A, B |

| | | | | | | | | | |
|---|---|---|---|---|---|---|---|---|---|
| Q9JHH9 | COPZ2_MOUSE | 205 | 22934 | 5.2 | (Q9JHH9) Coatomer subunit zeta-2 (Zeta-2 coat protein) (Zeta-2 COP) | 9.0845E-05 | 7.5926E-06 | 8% | A, B |
| Q07076 | ANXA7_MOUSE | 463 | 49939 | 6.2 | (Q07076) Annexin A7 (Annexin VII) (Synexin) | 9.0392E-05 | 2.079E-05 | 23% | A, B |
| Q8WTY4 | CPIN1_MOUSE | 309 | 33429 | 5.2 | (Q8WTY4) Anamorsin (Cytokine-induced apoptosis inhibitor 1) | 8.9214E-05 | 7.4495E-06 | 8% | A, B |
| Q8K3X6 | ANS4B_MOUSE | 423 | 47975 | 5 | (Q8K3X6) Ankyrin repeat and SAM domain-containing protein 4B (Harmonin-interacting ankyrin repeat-containing protein) (Harp) | 8.8584E-05 | 3.7401E-05 | 42% | A, B |
| O08583 | THOC4_MOUSE | 254 | 26809 | 11.2 | (O08583) THO complex subunit 4 (Tho4) (RNA and export factor binding protein 1) (REF1-I) (Ally of AML-1 and LEF-1) (Aly/REF) | 8.84E-05 | 7.0108E-05 | 79% | A, B |
| O35887 | CALU_MOUSE | 315 | 37064 | 4.7 | (O35887) Calumenin precursor (Crocalbin) | 8.7515E-05 | 7.3076E-06 | 8% | A, B |
| P15626 | GSTM2_MOUSE | 217 | 25585 | 7.4 | (P15626) Glutathione S-transferase Mu 2 (EC 2.5.1.18) (GST class-mu 2) (Glutathione S-transferase pmGT2) (GST 5-5) | 8.7456E-05 | 6.6584E-05 | 76% | A, B |
| Q8BMP6 | GCP60_MOUSE | 524 | 60050 | 5.1 | (Q8BMP6) Golgi resident protein GCP60 (Acyl-CoA-binding domain-containing protein 3) (Golgi phosphoprotein 1) (GOLPH1) (Golgi complex-associated protein 1) (GOCAP1) (PBR- and PKA-associated protein 7) (Peripheral benzodiazepine receptor-assoc | 8.6646E-05 | 1.7479E-05 | 20% | A, B |
| O35083 | PLCA_MOUSE | 285 | 31709 | 9.1 | (O35083) 1-acyl-sn-glycerol-3-phosphate acyltransferase alpha (EC 2.3.1.51) (1-AGP acyltransferase 1) (1-AGPAT 1) (Lysophosphatidic acid acyltransferase-alpha) (LPAAT-alpha) (1-acylglycerol-3-phosphate O-acyltransferase 1) | 8.3663E-05 | 7.4842E-05 | 89% | A, B |
| Q8VBT0 | TXND1_MOUSE | 278 | 31396 | 5.3 | (Q8VBT0) Thioredoxin domain-containing protein 1 precursor | 8.2748E-05 | 1.6686E-05 | 20% | A, B |
| Q59J78 | MIMIT_MOUSE | 168 | 19628 | 8.2 | (Q59J78) Mimitin, mitochondrial precursor (Myc-induced mitochondrial protein) (MMTN) | 8.1502E-05 | 3.2244E-05 | 40% | A, B |
| Q9CZ30 | PTD4_MOUSE | 396 | 44730 | 7.8 | (Q9CZ30) Putative GTP-binding protein PTD004 homolog | 8.0215E-05 | 4.3003E-05 | 54% | A, B |
| Q8K0Z7 | CCD44_MOUSE | 294 | 32314 | 8.1 | (Q8K0Z7) Coiled-coil domain-containing protein 44 | 8.0116E-05 | 2.9013E-05 | 36% | A, B |
| Q9D273 | MMAB_MOUSE | 237 | 26273 | 9.2 | (Q9D273) Cob(I)yrinic acid a,c-diamide adenosyltransferase, mitochondrial precursor (EC 2.5.1.17) (Cob(I)alamin adenosyltransferase) (Methylmalonic aciduria type B homolog) | 7.8579E-05 | 6.5674E-06 | 8% | A, B |
| P21550 | ENOB_MOUSE | 433 | 46894 | 7.2 | (P21550) Beta-enolase (EC 4.2.1.11) (2-phospho-D-glycerate hydro-lyase) (Muscle-specific enolase) (MSE) (Skeletal muscle enolase) (Enolase 3) | 7.7173E-05 | 5.1909E-05 | 67% | A, B |
| Q8BYK6 | YTHD3_MOUSE | 585 | 63961 | 9 | (Q8BYK6) YTH domain protein 3 | 7.6684E-05 | 3.787E-05 | 49% | A, B |
| Q8VEH3 | ARL8A_MOUSE | 186 | 21390 | 7.8 | (Q8VEH3) ADP-ribosylation factor-like protein 8A (ADP-ribosylation factor-like protein 10B) (Novel small G protein indispensable for equal chromosome segregation 2) | 7.3614E-05 | 2.9123E-05 | 40% | A, B |
| O88531 | PPT1_MOUSE | 306 | 34490 | 8 | (O88531) Palmitoyl-protein thioesterase 1 precursor (EC 3.1.2.22) (Palmitoyl-protein hydrolase 1) | 7.2627E-05 | 5.7132E-05 | 79% | A, B |
| Q05421 | CP2E1_MOUSE | 493 | 56805 | 8.4 | (Q05421) Cytochrome P450 2E1 (EC 1.14.14.1) (CYPIIE1) (P450-J) (P450-ALC) | 7.2619E-05 | 4.612E-05 | 64% | A, B |
| Q60749 | SAM68_MOUSE | 443 | 48371 | 8.7 | (Q60749) KH domain-containing, RNA-binding, signal transduction-associated protein 1 (p21 Ras GTPase-activating protein-associated p62) (GAP-associated tyrosine phosphoprotein p62) (Src-associated in mitosis 68 kDa protein) (Sam68) (p68) | 7.2428E-05 | 7.9811E-06 | 11% | A, B |

| | | | | | | | | | |
|---|---|---|---|---|---|---|---|---|---|
| O88630 | GOSR1_MOUSE | 250 | 28429 | 9.3 | (O88630) Golgi SNAP receptor complex member 1 (28 kDa Golgi SNARE protein) (28 kDa cis-Golgi SNARE p28) (GOS-28) | 7.1603E-05 | 4.5474E-05 | 64% | A, B |
| Q9D110 | MTHFS_MOUSE | 202 | 23070 | 7 | (Q9D110) 5-formyltetrahydrofolate cyclo-ligase (EC 6.3.3.2) (5,10-methenyl-tetrahydrofolate synthetase) (Methenyl-THF synthetase) (MTHFS) | 7.0508E-05 | 3.8375E-05 | 54% | A, B |
| O35479 | HNRPG_MOUSE | 388 | 42234 | 10 | (O35479) Heterogeneous nuclear ribonucleoprotein G (hnRNP G) (RNA-binding motif protein, X chromosome) | 6.9987E-05 | 2.7085E-05 | 39% | A, B |
| P10605 | CATB_MOUSE | 339 | 37280 | 5.9 | (P10605) Cathepsin B precursor (EC 3.4.22.1) (Cathepsin B1) [Contains: Cathepsin B light chain; Cathepsin B heavy chain] | 6.9481E-05 | 2.5162E-05 | 36% | A, B |
| P61759 | PFD3_MOUSE | 196 | 22436 | 6.3 | (P61759) Prefoldin subunit 3 (Von Hippel-Lindau-binding protein 1) (VHL-binding protein 1) (VBP-1) | 6.9273E-05 | 2.6809E-05 | 39% | A, B |
| Q61166 | MARE1_MOUSE | 267 | 29885 | 5.2 | (Q61166) Microtubule-associated protein RP/EB family member 1 (APC-binding protein EB1) (End-binding protein 1) (EB1) | 6.7689E-05 | 4.3491E-05 | 64% | A, B |
| Q9WV60 | GSK3B_MOUSE | 420 | 46710 | 8.8 | (Q9WV60) Glycogen synthase kinase-3 beta (EC 2.7.11.26) (GSK-3 beta) | 6.6101E-05 | 6.1389E-06 | 9% | A, B |
| P32921 | SYW_MOUSE | 481 | 54358 | 6.9 | (P32921) Tryptophanyl-tRNA synthetase (EC 6.1.1.2) (Tryptophan--tRNA ligase) (TrpRS) | 6.5443E-05 | 3.4559E-05 | 53% | A, B |
| Q9CYI4 | LUC7L_MOUSE | 371 | 43934 | 9.9 | (Q9CYI4) Putative RNA-binding protein Luc7-like 1 | 6.3488E-05 | 2.2992E-05 | 36% | A, B |
| P16054 | KPCE_MOUSE | 737 | 83561 | 7 | (P16054) Protein kinase C epsilon type (EC 2.7.11.13) (nPKC-epsilon) | 6.3077E-05 | 2.2838E-05 | 36% | A, B |
| P98197 | AT11A_MOUSE | 1187 | 135502 | 6.5 | (P98197) Probable phospholipid-transporting ATPase IH (EC 3.6.3.1) (ATPase class I type 11A) (ATPase IS) | 6.056E-05 | 2.7673E-05 | 46% | A, B |
| O54724 | PTRF_MOUSE | 392 | 43954 | 5.5 | (O54724) Polymerase I and transcript release factor | 5.9296E-05 | 2.1469E-05 | 36% | A, B |
| P23591 | FCL_MOUSE | 321 | 35878 | 6.7 | (P23591) GDP-L-fucose synthetase (EC 1.1.1.271) (Protein FX) (Red cell NADP(H)-binding protein) (GDP-4-keto-6-deoxy-D-mannose-3,5-epimerase-4-reductase) (Transplantation antigen P35B) (Tum-P35B antigen) | 5.9122E-05 | 4.5011E-05 | 76% | A, B |
| Q99PG2 | OGFR_MOUSE | 633 | 70679 | 4.8 | (Q99PG2) Opioid growth factor receptor (OGFr) (Zeta-type opioid receptor) | 5.8067E-05 | 4.8486E-06 | 8% | A, B |
| O88888 | APBA3_MOUSE | 571 | 60718 | 5.1 | (O88888) Amyloid beta A4 precursor protein-binding family A member 3 (Neuron-specific X11L2 protein) (Neuronal Munc18-1-interacting protein 3) (Mint-3) (Adapter protein X11gamma) | 5.7951E-05 | 3.8557E-05 | 67% | A, B |
| Q8VDG5 | PPCS_MOUSE | 311 | 33794 | 6.6 | (Q8VDG5) Phosphopantothenate--cysteine ligase (EC 6.3.2.5) (Phosphopantothenoylcysteine synthetase) (PPC synthetase) | 5.7558E-05 | 3.6555E-05 | 64% | A, B |
| P11679 | K2C8_MOUSE | 489 | 54434 | 5.8 | (P11679) Keratin, type II cytoskeletal 8 (Cytokeratin-8) (CK-8) (Keratin-8) (K8) (Cytokeratin endo A) | 5.7484E-05 | 3.1282E-05 | 54% | A, B |
| Q8R001 | MARE2_MOUSE | 326 | 36946 | 5.4 | (Q8R001) Microtubule-associated protein RP/EB family member 2 (APC-binding protein EB2) (End-binding protein 2) (EB2) | 5.5438E-05 | 3.562E-05 | 64% | A, B |
| Q63918 | SDPR_MOUSE | 417 | 46633 | 5.2 | (Q63918) Serum deprivation-response protein (Phosphatidylserine-binding protein) | 5.4752E-05 | 1.054E-05 | 19% | A, B |
| Q00612 | G6PD1_MOUSE | 514 | 59131 | 6.5 | (Q00612) Glucose-6-phosphate 1-dehydrogenase X (EC 1.1.1.49) (G6PD) | 5.4688E-05 | 2.9761E-05 | 54% | A, B |
| Q9JKK7 | TMOD2_MOUSE | 351 | 39510 | 5.4 | (Q9JKK7) Tropomodulin-2 (Neuronal tropomodulin) (N-Tmod) | 5.4625E-05 | 4.1952E-05 | 77% | A, B |
| Q64FW2 | RETST_MOUSE | 609 | 67464 | 9 | (Q64FW2) All-trans-retinol 13,14-reductase precursor (EC 1.3.99.23) (All-trans-13,14-dihydroretinol saturase) (RetSat) | 5.2365E-05 | 6.2596E-06 | 12% | A, B |

| | | | | | | | | | |
|---|---|---|---|---|---|---|---|---|---|
| Q5YD48 | ACF_MOUSE | 595 | 65725 | 8.7 | (Q5YD48) APOBEC1 complementation factor (APOBEC1-stimulating protein) | 5.1883E-05 | 4.9933E-05 | 96% | A, B |
| Q9D394 | RUFY3_MOUSE | 469 | 53007 | 5.5 | (Q9D394) Protein RUFY3 (Rap2-interacting protein x) (RIPx) | 4.956E-05 | 1.7944E-05 | 36% | A, B |
| Q8C0D4 | RHG12_MOUSE | 838 | 95352 | 7.6 | (Q8C0D4) Rho-GTPase-activating protein 12 | 4.9021E-05 | 3.6334E-06 | 7% | A, B |
| Q9DCN1 | NUD12_MOUSE | 462 | 51511 | 7.1 | (Q9DCN1) Peroxisomal NADH pyrophosphatase NUDT12 (EC 3.6.1.22) (Nucleoside diphosphate-linked moiety X motif 12) (Nudix motif 12) | 4.8104E-05 | 3.7841E-05 | 79% | A, B |
| Q8R519 | ACMSD_MOUSE | 194 | 22391 | 7.4 | (Q8R519) 2-amino-3-carboxymuconate-6-semialdehyde decarboxylase (EC 4.1.1.45) (Fragment) | 4.7998E-05 | 4.0115E-06 | 8% | A, B |
| Q9D662 | SC23B_MOUSE | 767 | 86437 | 7 | (Q9D662) Protein transport protein Sec23B (SEC23-related protein B) | 4.7215E-05 | 1.2941E-05 | 27% | A, B |
| P49615 | CDK5_MOUSE | 292 | 33288 | 7.7 | (P49615) Cell division protein kinase 5 (EC 2.7.11.22) (Tau protein kinase II catalytic subunit) (TPKII catalytic subunit) (Serine/threonine-protein kinase PSSALRE) (CRK6) | 4.6891E-05 | 1.8551E-05 | 40% | A, B |
| Q505F5 | LRC47_MOUSE | 581 | 63590 | 8.1 | (Q505F5) Leucine-rich repeat-containing protein 47 | 4.6738E-05 | 1.8088E-05 | 39% | A, B |
| Q8R5F7 | IFIH1_MOUSE | 1025 | 115971 | 6.2 | (Q8R5F7) Interferon-induced helicase C domain-containing protein 1 (EC 3.6.1.-) (Interferon induced with helicase C domain protein 1) (Helicase with 2 CARD domains) (Helicard) (Melanoma differentiation-associated protein 5) (MDA-5) | 4.6525E-05 | 4.1619E-05 | 89% | A, B |
| Q9CRA5 | GOLP3_MOUSE | 298 | 33752 | 6.4 | (Q9CRA5) Golgi phosphoprotein 3 (Coat-protein GPP34) | 4.5947E-05 | 1.8178E-05 | 40% | A, B |
| P54103 | ZRF1_MOUSE | 514 | 59511 | 8.6 | (P54103) Zuotin-related factor 1 | 4.5221E-05 | 1.6373E-05 | 36% | A, B |
| P59158 | S12A3_MOUSE | 1002 | 110694 | 7.6 | (P59158) Solute carrier family 12 member 3 (Thiazide-sensitive sodium-chloride cotransporter) (Na-Cl symporter) | 4.477E-05 | 2.211E-05 | 49% | A, B |
| Q8BIJ7 | RUFY1_MOUSE | 712 | 80376 | 5.7 | (Q8BIJ7) RUN and FYVE domain-containing protein 1 (Rab4-interacting protein) | 4.3357E-05 | 4.1728E-05 | 96% | A, B |
| Q61074 | PP2CG_MOUSE | 542 | 58728 | 4.4 | (Q61074) Protein phosphatase 2C isoform gamma (EC 3.1.3.16) (PP2C-gamma) (Protein phosphatase magnesium-dependent 1 gamma) (Protein phosphatase 1C) (Fibroblast growth factor-inducible protein 13) (FIN13) | 4.1884E-05 | 8.449E-06 | 20% | A, B |
| Q91W43 | GCSP_MOUSE | 1025 | 113267 | 7.6 | (Q91W43) Glycine dehydrogenase [decarboxylating], mitochondrial precursor (EC 1.4.4.2) (Glycine decarboxylase) (Glycine cleavage system P-protein) | 4.0077E-05 | 2.9705E-06 | 7% | A, B |
| P68134 | ACTS_MOUSE | 377 | 42051 | 5.4 | (P68134) Actin, alpha skeletal muscle (Alpha-actin-1) | 3.7433E-05 | 2.0073E-05 | 54% | A, B |
| Q8QZR5 | ALAT_MOUSE | 495 | 55011 | 6.6 | (Q8QZR5) Alanine aminotransferase (EC 2.6.1.2) (Glutamic--pyruvic transaminase) (GPT) (Glutamic--alanine transaminase) | 3.7391E-05 | 3.4725E-06 | 9% | A, B |
| O54931 | AKAP2_MOUSE | 885 | 97501 | 5.4 | (O54931) A-kinase anchor protein 2 (Protein kinase A-anchoring protein 2) (PRKA2) (AKAP-2) (AKAP expressed in kidney and lung) (AKAP-KL) | 3.4882E-05 | 3.3571E-05 | 96% | A, B |
| P82343 | RENBP_MOUSE | 419 | 48510 | 6.2 | (P82343) N-acylglucosamine 2-epimerase (EC 5.1.3.8) (GlcNAc 2-epimerase) (N-acetyl-D-glucosamine 2-epimerase) (AGE) (Renin-binding protein) (RnBP) | 3.3992E-05 | 1.85E-05 | 54% | A, B |
| Q8K2I3 | FMO2_MOUSE | 534 | 60843 | 8.5 | (Q8K2I3) Dimethylaniline monooxygenase [N-oxide-forming] 2 (EC 1.14.13.8) (Pulmonary flavin-containing monooxygenase 2) (FMO 2) (Dimethylaniline oxidase 2) | 3.3522E-05 | 2.1289E-05 | 64% | A, B |
| O55028 | BCKD_MOUSE | 412 | 46588 | 8.9 | (O55028) [3-methyl-2-oxobutanoate dehydrogenase [lipoamide]] kinase, mitochondrial precursor (EC 2.7.11.4) (Branched-chain alpha-ketoacid dehydrogenase kinase) (BCKDHKIN) (BCKD-kinase) | 3.3234E-05 | 1.3148E-05 | 40% | A, B |

| | | | | | | | | |
|---|---|---|---|---|---|---|---|---|
| Q9Z1T6 | FYV1_MOUSE | 2052 | 233050 | 6.8 | (Q9Z1T6) FYVE finger-containing phosphoinositide kinase (EC 2.7.1.68) (1-phosphatidylinositol-4-phosphate 5-kinase) (PIP5K) (PtdIns(4)P-5-kinase) (PIKfyve) (p235) | 3.2404E-05 | 2.7949E-05 | 86% | A, B |
| Q8CI96 | RSNL2_MOUSE | 704 | 75789 | 8.6 | (Q8CI96) Restin-like protein 2 | 3.1894E-05 | 2.5294E-05 | 79% | A, B |
| Q7TN29 | SMP1L_MOUSE | 428 | 46578 | 8.9 | (Q7TN29) Stromal membrane-associated protein 1-like (Stromal membrane-associated protein 2) | 3.1723E-05 | 1.2277E-05 | 39% | A, B |
| P15331 | PERI_MOUSE | 475 | 54268 | 5.5 | (P15331) Peripherin | 2.8826E-05 | 1.1404E-05 | 40% | A, B |
| Q5SV85 | SYNG_MOUSE | 1306 | 139616 | 5 | (Q5SV85) AP1 subunit gamma-binding protein 1 (Gamma-synergin) | 2.8344E-05 | 2.6323E-06 | 9% | A, B |
| Q91Z96 | BMP2K_MOUSE | 1138 | 126185 | 6.8 | (Q91Z96) BMP-2-inducible protein kinase (EC 2.7.11.1) (BIKe) | 2.8023E-05 | 3.3498E-06 | 12% | A, B |
| P35822 | PTPRK_MOUSE | 1457 | 164185 | 5.9 | (P35822) Receptor-type tyrosine-protein phosphatase kappa precursor (EC 3.1.3.48) (Protein-tyrosine phosphatase kappa) (R-PTP-kappa) | 2.7822E-05 | 1.1009E-05 | 40% | A, B |
| Q61249 | IGBP1_MOUSE | 340 | 38971 | 6.2 | (Q61249) Immunoglobulin-binding protein 1 (CD79a-binding protein 1) (Alpha4 phosphoprotein) (Lymphocyte signal transduction molecule alpha 4) (p52) | 2.7387E-05 | 2.2889E-06 | 8% | A, B |
| P13597 | ICAM1_MOUSE | 537 | 58844 | 6.1 | (P13597) Intercellular adhesion molecule 1 precursor (ICAM-1) (MALA-2) | 2.5498E-05 | 1.0087E-05 | 40% | A, B |
| Q8R3S6 | EXOC1_MOUSE | 893 | 101746 | 6.5 | (Q8R3S6) Exocyst complex component 1 (Exocyst complex component Sec3) | 2.5421E-05 | 5.1281E-06 | 20% | A, B |
| Q9JLI8 | SART3_MOUSE | 962 | 109619 | 5.2 | (Q9JLI8) Squamous cell carcinoma antigen recognized by T-cells 3 (SART-3) (mSART-3) (Tumor-rejection antigen SART3) | 2.4281E-05 | 8.5795E-06 | 35% | A, B |
| Q3U4I7 | CJ033_MOUSE | 580 | 62685 | 8 | (Q3U4I7) Probable oxidoreductase C10orf33 homolog (EC 1.-.-.-) | 2.3409E-05 | 9.0595E-06 | 39% | A, B |
| Q8R0H9 | GGA1_MOUSE | 635 | 69972 | 5.3 | (Q8R0H9) ADP-ribosylation factor-binding protein GGA1 (Golgi-localized, gamma ear-containing, ARF-binding protein 1) (Gamma-adaptin-related protein 1) | 2.2429E-05 | 1.2207E-05 | 54% | A, B |
| Q6ZQ38 | CAND1_MOUSE | 1230 | 136331 | 5.8 | (Q6ZQ38) Cullin-associated NEDD8-dissociated protein 1 (Cullin associated and neddylation-dissociated protein 1) (p120 CAND1) | 2.2077E-05 | 8.5439E-06 | 39% | A, B |
| Q9R190 | MTA2_MOUSE | 668 | 75030 | 9.7 | (Q9R190) Metastasis-associated protein MTA2 (Metastasis-associated 1-like 1) | 2.1321E-05 | 1.1604E-05 | 54% | A, B |
| Q91VW5 | GOGA4_MOUSE | 2238 | 257561 | 5.4 | (Q91VW5) Golgin subfamily A member 4 (tGolgin-1) | 2.0772E-05 | 7.5209E-06 | 36% | A, B |
| Q8CBW3 | ABI1_MOUSE | 480 | 52156 | 7.6 | (Q8CBW3) Abl interactor 1 (Abelson interactor 1) (Abi-1) (Spectrin SH3 domain-binding protein 1) (Eps8 SH3 domain-binding protein) (Eps8-binding protein) (e3B1) (Ablphilin-1) | 1.9399E-05 | 1.6213E-06 | 8% | A, B |
| Q80YR5 | SAFB2_MOUSE | 991 | 111839 | 6.4 | (Q80YR5) Scaffold attachment factor B2 | 1.8792E-05 | 1.5706E-06 | 8% | A, B |
| Q62351 | TFR1_MOUSE | 763 | 85731 | 6.6 | (Q62351) Transferrin receptor protein 1 (TfR1) (TR) (TfR) (Trfr) (CD71 antigen) | 1.8667E-05 | 1.0159E-05 | 54% | A, B |
| Q9Z1Q9 | SYV_MOUSE | 1263 | 140215 | 7.8 | (Q9Z1Q9) Valyl-tRNA synthetase (EC 6.1.1.9) (Valine--tRNA ligase) (ValRS) (Protein G7a) | 1.8404E-05 | 6.6634E-06 | 36% | A, B |
| P45481 | CBP_MOUSE | 2441 | 265471 | 8.4 | (P45481) CREB-binding protein (EC 2.3.1.48) | 1.5502E-05 | 1.1905E-05 | 77% | A, B |
| O08919 | NUMBL_MOUSE | 604 | 64190 | 8.8 | (O08919) Numb-like protein | 1.5417E-05 | 1.2885E-06 | 8% | A, B |
| P70398 | USP9X_MOUSE | 2559 | 290543 | 6 | (P70398) Probable ubiquitin carboxyl-terminal hydrolase FAF-X (EC 3.1.2.15) (Ubiquitin thioesterase FAF-X) (Ubiquitin-specific-processing protease FAF-X) (Deubiquitinating enzyme FAF-X) (Fat facets protein-related, X-linked) (Ubiquitin-specif | 1.4363E-05 | 1.1994E-06 | 8% | A, B |

| | | | | | | | | | |
|---|---|---|---|---|---|---|---|---|---|
| Q01405 | SC23A_MOUSE | 765 | 86162 | 7.1 | (Q01405) Protein transport protein Sec23A (SEC23-related protein A) | 1.2172E-05 | 1.0173E-06 | 8% | A, B |
| Q80UE6 | WNK4_MOUSE | 1222 | 132410 | 5.5 | (Q80UE6) Serine/threonine-protein kinase WNK4 (EC 2.7.11.1) (Protein kinase with no lysine 4) (Protein kinase, lysine-deficient 4) | 1.1655E-05 | 6.3434E-06 | 54% | A, B |
| Q80XJ3 | K1043_MOUSE | 1691 | 183214 | 6.8 | (Q80XJ3) TPR repeat-containing protein KIAA1043 | 1.0586E-05 | 6.7229E-06 | 64% | A, B |
| Q91ZU8 | BPAEA_MOUSE | 2611 | 301691 | 6.5 | (Q91ZU8) Bullous pemphigoid antigen 1, isoform 5 (BPA) (Hemidesmosomal plaque protein) (Dystonia musculorum protein) (Dystonin) | 5.2001E-06 | 2.0125E-06 | 39% | A, B |
| Q61037 | TSC2_MOUSE | 1814 | 202070 | 7 | (Q61037) Tuberin (Tuberous sclerosis 2 homolog protein) | 5.1332E-06 | 4.2902E-07 | 8% | A, B |
| Q9D1I5 | MCEE_MOUSE | 178 | 19017 | 9.1 | (Q9D1I5) Methylmalonyl-CoA epimerase, mitochondrial precursor (EC 5.1.99.1) (DL-methylmalonyl-CoA racemase) | 0.00161866 | 0.00082735 | 51% | A, C |
| P97351 | RS3A_MOUSE | 263 | 29754 | 9.7 | (P97351) 40S ribosomal protein S3a | 0.00102123 | 0.00051435 | 50% | A, C |
| Q9CQM5 | TXNL5_MOUSE | 123 | 14015 | 4.8 | (Q9CQM5) Thioredoxin-like protein 5 (14 kDa thioredoxin-related protein) (TRP14) (Protein 42-9-9) | 0.00090583 | 8.8072E-05 | 10% | A, C |
| P70696 | H2B1A_MOUSE | 126 | 14105 | 10.3 | (P70696) Histone H2B type 1-A (Histone H2B, testis) (Testis-specific histone H2B) | 0.00079741 | 0.00081323 | 102% | A, C |
| O89086 | RBM3_MOUSE | 153 | 16605 | 7.5 | (O89086) Putative RNA-binding protein 3 (RNA-binding motif protein 3) | 0.00079013 | 0.0006642 | 84% | A, C |
| Q9CQ75 | NDUA2_MOUSE | 98 | 10784 | 10 | (Q9CQ75) NADH dehydrogenase [ubiquinone] 1 alpha subcomplex subunit 2 (EC 1.6.5.3) (EC 1.6.99.3) (NADH-ubiquinone oxidoreductase B8 subunit) (Complex I-B8) (CI-B8) | 0.00060788 | 0.00065751 | 108% | A, C |
| Q9CQH3 | NDUB5_MOUSE | 189 | 21710 | 9.4 | (Q9CQH3) NADH dehydrogenase [ubiquinone] 1 beta subcomplex subunit 5, mitochondrial precursor (EC 1.6.5.3) (EC 1.6.99.3) (NADH-ubiquinone oxidoreductase SGDH subunit) (Complex I-SGDH) (CI-SGDH) | 0.00060689 | 0.0001769 | 29% | A, C |
| P46412 | GPX3_MOUSE | 226 | 25377 | 8.1 | (P46412) Glutathione peroxidase 3 precursor (EC 1.11.1.9) (GSHPx-3) (GPx-3) (Plasma glutathione peroxidase) (GSHPx-P) | 0.00059228 | 0.0001363 | 23% | A, C |
| Q6ZQI3 | K0152_MOUSE | 291 | 32342 | 6 | (Q6ZQI3) Protein KIAA0152 precursor | 0.00057169 | 0.00053616 | 94% | A, C |
| O35972 | RM23_MOUSE | 146 | 17122 | 9.7 | (O35972) Mitochondrial 39S ribosomal protein L23 (L23mt) (MRP-L23) (L23 mitochondrial-related protein) | 0.00051201 | 0.00011344 | 22% | A, C |
| P63044 | VAMP2_MOUSE | 115 | 12560 | 8.1 | (P63044) Vesicle-associated membrane protein 2 (VAMP-2) (Synaptobrevin-2) | 0.00049775 | 0.00035937 | 72% | A, C |
| P59999 | ARPC4_MOUSE | 167 | 19536 | 8.4 | (P59999) Actin-related protein 2/3 complex subunit 4 (ARP2/3 complex 20 kDa subunit) (p20-ARC) | 0.0003811 | 0.00017285 | 45% | A, C |
| P62852 | RS25_MOUSE | 125 | 13742 | 10.1 | (P62852) 40S ribosomal protein S25 | 0.00037562 | 5.5728E-05 | 15% | A, C |
| P62751 | RL23A_MOUSE | 156 | 17695 | 10.4 | (P62751) 60S ribosomal protein L23a | 0.00036833 | 5.0596E-05 | 14% | A, C |
| P58771 | TPM1_MOUSE | 284 | 32681 | 4.7 | (P58771) Tropomyosin 1 alpha chain (Alpha-tropomyosin) | 0.00035744 | 0.00022646 | 63% | A, C |
| Q91VM9 | IPYR2_MOUSE | 330 | 38115 | 7 | (Q91VM9) Inorganic pyrophosphatase 2, mitochondrial precursor (EC 3.6.1.1) (PPase 2) | 0.00033697 | 0.00011633 | 35% | A, C |
| Q3THS6 | METK2_MOUSE | 395 | 43689 | 6.5 | (Q3THS6) S-adenosylmethionine synthetase isoform type-2 (EC 2.5.1.6) (Methionine adenosyltransferase 2) (AdoMet synthetase 2) | 0.0003149 | 1.901E-05 | 6% | A, C |
| P32067 | LA_MOUSE | 415 | 47756 | 9.8 | (P32067) Lupus La protein homolog (La ribonucleoprotein) (La autoantigen homolog) | 0.00030263 | 0.00030864 | 102% | A, C |
| P05213 | TBA2_MOUSE | 451 | 50152 | 5.1 | (P05213) Tubulin alpha-2 chain (Alpha-tubulin 2) (Alpha-tubulin isotype M-alpha-2) | 0.00028735 | 0.00029648 | 103% | A, C |

| | | | | | | | | | |
|---|---|---|---|---|---|---|---|---|---|
| Q8VCI5 | PEX19_MOUSE | 299 | 32733 | 4.3 | (Q8VCI5) Peroxisomal biogenesis factor 19 (Peroxin-19) (Peroxisomal farnesylated protein) (PxF) | 0.00027271 | 0.00018688 | 69% | A, C |
| Q08093 | CNN2_MOUSE | 305 | 33156 | 7.6 | (Q08093) Calponin-2 (Calponin H2, smooth muscle) (Neutral calponin) | 0.00024312 | 0.00014336 | 59% | A, C |
| O55022 | PGRC1_MOUSE | 194 | 21563 | 4.7 | (O55022) Membrane-associated progesterone receptor component 1 | 0.00023927 | 1.9046E-05 | 8% | A, C |
| P53994 | RAB2A_MOUSE | 212 | 23548 | 6.5 | (P53994) Ras-related protein Rab-2A | 0.00022535 | 8.5061E-05 | 38% | A, C |
| Q9CXW4 | RL11_MOUSE | 177 | 20121 | 9.6 | (Q9CXW4) 60S ribosomal protein L11 | 0.00022106 | 3.2797E-05 | 15% | A, C |
| P11031 | TCP4_MOUSE | 126 | 14296 | 9.6 | (P11031) Activated RNA polymerase II transcriptional coactivator p15 precursor (SUB1 homolog) (Positive cofactor 4) (PC4) (p14) (Single-stranded DNA-binding protein p9) | 0.00021412 | 1.1667E-05 | 5% | A, C |
| P61022 | CHP1_MOUSE | 194 | 22301 | 5.1 | (P61022) Calcium-binding protein p22 (Calcium-binding protein CHP) (Calcineurin homologous protein) (Sid 470) | 0.00021128 | 0.0001097 | 52% | A, C |
| O70250 | PGAM2_MOUSE | 252 | 28696 | 8.5 | (O70250) Phosphoglycerate mutase 2 (EC 5.4.2.1) (EC 5.4.2.4) (EC 3.1.3.13) (Phosphoglycerate mutase isozyme M) (PGAM-M) (BPG-dependent PGAM 2) (Muscle-specific phosphoglycerate mutase) | 0.00021086 | 5.5583E-05 | 26% | A, C |
| P53026 | RL10A_MOUSE | 216 | 24785 | 10 | (P53026) 60S ribosomal protein L10a (CSA-19) (Protein NEDD6) (Neural precursor cell expressed developmentally down-regulated protein 6) | 0.00020276 | 5.7436E-05 | 28% | A, C |
| Q8BJZ4 | RT35_MOUSE | 320 | 35975 | 8.6 | (Q8BJZ4) 28S ribosomal protein S35, mitochondrial precursor (S35mt) (MRP-S35) | 0.00019564 | 2.9025E-05 | 15% | A, C |
| O88456 | CPNS1_MOUSE | 269 | 28463 | 5.6 | (O88456) Calpain small subunit 1 (CSS1) (Calcium-dependent protease small subunit 1) (Calcium-dependent protease small subunit) (CDPS) (Calpain regulatory subunit) (Calcium-activated neutral proteinase small subunit) (CANP small subunit) | 0.00019448 | 9.3211E-05 | 48% | A, C |
| Q9QZ23 | HIRP5_MOUSE | 199 | 22140 | 4.4 | (Q9QZ23) HIRA-interacting protein 5 (mHIRIP5) | 0.00018837 | 8.2055E-05 | 44% | A, C |
| O55023 | IMPA1_MOUSE | 277 | 30436 | 5.2 | (O55023) Inositol monophosphatase (EC 3.1.3.25) (IMPase) (IMP) (Inositol-1(or 4)-monophosphatase) (Lithium-sensitive myo-inositol monophosphatase A1) | 0.00018215 | 7.2672E-06 | 4% | A, C |
| Q9JM76 | ARPC3_MOUSE | 177 | 20393 | 8.6 | (Q9JM76) Actin-related protein 2/3 complex subunit 3 (ARP2/3 complex 21 kDa subunit) (p21-ARC) | 0.00017685 | 2.6237E-05 | 15% | A, C |
| Q9D7B6 | ACAD8_MOUSE | 413 | 45020 | 8.1 | (Q9D7B6) Acyl-CoA dehydrogenase family member 8, mitochondrial precursor (EC 1.3.99.-) (ACAD-8) (Isobutyryl-CoA dehydrogenase) | 0.00017199 | 0.00017128 | 100% | A, C |
| Q9D8Y0 | EFHD2_MOUSE | 240 | 26791 | 5.1 | (Q9D8Y0) EF-hand domain-containing protein 2 (Swiprosin-1) | 0.00016987 | 0.00011641 | 69% | A, C |
| P97478 | COQ7_MOUSE | 217 | 24042 | 7.2 | (P97478) Ubiquinone biosynthesis protein COQ7 homolog (Coenzyme Q biosynthesis protein 7 homolog) (Timing protein clk-1 homolog) | 0.00016417 | 4.9576E-05 | 30% | A, C |
| O70493 | SNX12_MOUSE | 165 | 19116 | 7.3 | (O70493) Sorting nexin-12 (SDP8 protein) | 0.00016351 | 8.909E-06 | 5% | A, C |
| Q8K4M5 | COMD1_MOUSE | 188 | 20996 | 7.6 | (Q8K4M5) COMM domain-containing protein 1 (Protein Murr1) | 0.00016213 | 3.4165E-05 | 21% | A, C |
| O88958 | GNPI_MOUSE | 289 | 32550 | 6.5 | (O88958) Glucosamine-6-phosphate isomerase (EC 3.5.99.6) (Glucosamine-6-phosphate deaminase) (GNPDA) (GlcN6P deaminase) (Oscillin) | 0.00015963 | 1.419E-05 | 9% | A, C |
| Q9Z2U1 | PSA5_MOUSE | 241 | 26411 | 4.8 | (Q9Z2U1) Proteasome subunit alpha type 5 (EC 3.4.25.1) (Proteasome zeta chain) (Macropain zeta chain) (Multicatalytic endopeptidase complex zeta chain) | 0.00015895 | 2.1834E-05 | 14% | A, C |

| | | | | | | | | | |
|---|---|---|---|---|---|---|---|---|---|
| Q9CQX2 | CYB5B_MOUSE | 146 | 16318 | 4.9 | (Q9CQX2) Cytochrome b5 type B precursor (Cytochrome b5 outer mitochondrial membrane isoform) | 0.00015596 | 5.0832E-05 | 33% | A, C |
| P35282 | RAB21_MOUSE | 221 | 23975 | 7.9 | (P35282) Ras-related protein Rab-21 (Rab-12) | 0.00015377 | 5.1475E-05 | 33% | A, C |
| Q920A5 | RISC_MOUSE | 452 | 50967 | 5.8 | (Q920A5) Retinoid-inducible serine carboxypeptidase precursor (EC 3.4.16.-) (Serine carboxypeptidase 1) | 0.00014759 | 0.00014297 | 97% | A, C |
| Q9QXN5 | MIOX_MOUSE | 285 | 33164 | 5.3 | (Q9QXN5) Inositol oxygenase (EC 1.13.99.1) (Myo-inositol oxygenase) (Aldehyde reductase-like 6) (Renal-specific oxidoreductase) | 0.0001467 | 3.5842E-05 | 24% | A, C |
| P47757 | CAPZB_MOUSE | 276 | 31214 | 5.7 | (P47757) F-actin capping protein subunit beta (CapZ beta) | 0.00014553 | 0.00011721 | 81% | A, C |
| Q9QYC0 | ADDA_MOUSE | 735 | 80647 | 5.9 | (Q9QYC0) Alpha-adducin (Erythrocyte adducin subunit alpha) | 0.000144 | 9.582E-05 | 67% | A, C |
| Q7TQI3 | OTUB1_MOUSE | 271 | 31270 | 4.9 | (Q7TQI3) Ubiquitin thioesterase protein OTUB1 (EC 3.4.-.-) (Otubain 1) (OTU domain-containing ubiquitin aldehyde-binding protein 1) (Ubiquitin-specific-processing protease OTUB1) (Deubiquitinating enzyme OTUB1) | 0.0001422 | 1.8218E-05 | 13% | A, C |
| P50580 | PA2G4_MOUSE | 393 | 43568 | 6.9 | (P50580) Proliferation-associated protein 2G4 (Proliferation-associated protein 1) (Protein p38-2G4) (Mpp1) (IRES-specific cellular trans-acting factor 45 kDa) (ITAF45) | 0.0001373 | 7.4808E-06 | 5% | A, C |
| Q61990 | PCBP2_MOUSE | 362 | 38222 | 6.8 | (Q61990) Poly(rC)-binding protein 2 (Alpha-CP2) (Putative heterogeneous nuclear ribonucleoprotein X) (hnRNP X) (CTBP) (CBP) | 0.0001297 | 1.9243E-05 | 15% | A, C |
| Q9CQ89 | CUTA_MOUSE | 177 | 18865 | 6.8 | (Q9CQ89) Protein CutA precursor (Brain acetylcholinesterase putative membrane anchor) | 0.00012865 | 4.193E-05 | 33% | A, C |
| P68510 | 1433F_MOUSE | 245 | 28081 | 4.9 | (P68510) 14-3-3 protein eta | 0.00012776 | 1.8955E-05 | 15% | A, C |
| Q9D880 | TIM50_MOUSE | 353 | 39776 | 8.1 | (Q9D880) Import inner membrane translocase subunit TIM50, mitochondrial precursor | 0.00012542 | 6.5116E-05 | 52% | A, C |
| Q03958 | PFD6_MOUSE | 127 | 14455 | 8.9 | (Q03958) Prefoldin subunit 6 (Protein Ke2) | 0.00012414 | 1.9562E-05 | 16% | A, C |
| Q9WUQ2 | PREB_MOUSE | 417 | 45437 | 8.8 | (Q9WUQ2) Prolactin regulatory element-binding protein (Mammalian guanine nucleotide exchange factor mSec12) | 0.00012244 | 0.0001494 | 122% | A, C |
| P43276 | H15_MOUSE | 222 | 22445 | 10.9 | (P43276) Histone H1.5 (H1 VAR.5) (H1b) | 0.00012153 | 6.6216E-06 | 5% | A, C |
| Q9WUL7 | ARL3_MOUSE | 182 | 20487 | 7.2 | (Q9WUL7) ADP-ribosylation factor-like protein 3 | 0.00012029 | 0.00010203 | 85% | A, C |
| P59325 | IF5_MOUSE | 429 | 48968 | 5.5 | (P59325) Eukaryotic translation initiation factor 5 (eIF-5) | 0.00011953 | 3.0489E-05 | 26% | A, C |
| P10923 | OSTP_MOUSE | 294 | 32459 | 4.5 | (P10923) Osteopontin precursor (Bone sialoprotein-1) (Secreted phosphoprotein 1) (SPP-1) (Minopontin) (Early T-lymphocyte activation 1 protein) (2AR) (Calcium oxalate crystal growth inhibitor protein) | 0.00011838 | 1.051E-06 | 1% | A, C |
| Q8VDW0 | DDX39_MOUSE | 427 | 49067 | 5.7 | (Q8VDW0) ATP-dependent RNA helicase DDX39 (EC 3.6.1.-) (DEAD box protein 39) | 0.00011816 | 4.7143E-06 | 4% | A, C |
| P51863 | VA0D_MOUSE | 351 | 40301 | 5 | (P51863) Vacuolar ATP synthase subunit d (EC 3.6.3.14) (V-ATPase d subunit) (Vacuolar proton pump d subunit) (V-ATPase AC39 subunit) (V-ATPase 40 kDa accessory protein) (P39) (Physophilin) | 0.00011615 | 7.9598E-05 | 69% | A, C |
| Q9DCG6 | MAWB2_MOUSE | 288 | 32048 | 7 | (Q9DCG6) Probable isomerase MAWBP-2 (EC 5.1.-.-) | 0.00011515 | 7.7927E-05 | 68% | A, C |
| Q9WUZ9 | ENP5_MOUSE | 427 | 47102 | 5.3 | (Q9WUZ9) Ectonucleoside triphosphate diphosphohydrolase 5 precursor (EC 3.6.1.6) (NTPDase5) (Nucleoside diphosphatase) (CD39 antigen-like 4) (ER-UDPase) | 0.00011381 | 6.815E-05 | 60% | A, C |
| Q6PFR5 | TRA2A_MOUSE | 281 | 32316 | 11.3 | (Q6PFR5) Transformer-2 protein homolog (TRA-2 alpha) | 0.0001114 | 1.6527E-05 | 15% | A, C |
| Q61133 | GSTT2_MOUSE | 243 | 27549 | 7.6 | (Q61133) Glutathione S-transferase theta-2 (EC 2.5.1.18) (GST class-theta-2) | 0.00010812 | 5.0924E-05 | 47% | A, C |

| | | | | | | | | | |
|---|---|---|---|---|---|---|---|---|---|
| O35609 | SCAM3_MOUSE | 349 | 38398 | 7.6 | (O35609) Secretory carrier-associated membrane protein 3 (Secretory carrier membrane protein 3) | 0.0001079 | 4.609E-05 | 43% | A, C |
| Q9ESG4 | TMM27_MOUSE | 222 | 25070 | 5.8 | (Q9ESG4) Collectrin precursor (Transmembrane protein 27) | 0.00010257 | 3.343E-05 | 33% | A, C |
| Q9D404 | OXSM_MOUSE | 459 | 48628 | 7.1 | (Q9D404) 3-oxoacyl-[acyl-carrier-protein] synthase, mitochondrial precursor (EC 2.3.1.41) (Beta-ketoacyl synthase) | 0.00010229 | 1.5176E-05 | 15% | A, C |
| Q91VN4 | CHCH6_MOUSE | 273 | 29799 | 8.2 | (Q91VN4) Coiled-coil-helix-coiled-coil-helix domain-containing protein 6 | 9.9455E-05 | 4.4921E-06 | 5% | A, C |
| Q9CRC3 | U235_MOUSE | 126 | 13189 | 9.1 | (Q9CRC3) UPF0235 protein C15orf40 homolog | 9.733E-05 | 5.9027E-05 | 61% | A, C |
| P67984 | RL22_MOUSE | 127 | 14628 | 9.2 | (P67984) 60S ribosomal protein L22 (Heparin-binding protein HBp15) | 9.6564E-05 | 5.8562E-05 | 61% | A, C |
| Q8BFW7 | LPP_MOUSE | 613 | 65891 | 7.4 | (Q8BFW7) Lipoma-preferred partner homolog | 9.6414E-05 | 2.3232E-05 | 24% | A, C |
| Q9CR24 | NUDT8_MOUSE | 210 | 23253 | 6.5 | (Q9CR24) Nucleoside diphosphate-linked moiety X motif 8, mitochondrial precursor (EC 3.6.1.-) (Nudix motif 8) | 9.5116E-05 | 4.0171E-05 | 42% | A, C |
| Q9CXN7 | MAWB1_MOUSE | 288 | 31983 | 5.3 | (Q9CXN7) Probable isomerase MAWBP-1 (EC 5.1.-.-) | 9.3676E-05 | 5.1041E-06 | 5% | A, C |
| O88967 | YMEL1_MOUSE | 715 | 80028 | 9 | (O88967) ATP-dependent metalloprotease YME1L1 (EC 3.4.24.-) (YME1-like protein 1) (ATP-dependent metalloprotease FtsH1) | 9.016E-05 | 2.4894E-05 | 28% | A, C |
| Q8VED2 | CNO_MOUSE | 215 | 23112 | 5.1 | (Q8VED2) Protein cappuccino | 8.9619E-05 | 1.1482E-05 | 13% | A, C |
| P97822 | AN32E_MOUSE | 260 | 29622 | 3.9 | (P97822) Acidic leucine-rich nuclear phosphoprotein 32 family member E (LANP-like protein) (LANP-L) (Cerebellar postnatal development protein 1) | 8.7579E-05 | 2.8544E-05 | 33% | A, C |
| Q9CQI3 | GMFB_MOUSE | 141 | 16592 | 5.2 | (Q9CQI3) Glia maturation factor beta (GMF-beta) | 8.6976E-05 | 5.2747E-05 | 61% | A, C |
| O54946 | DNJB6_MOUSE | 242 | 26978 | 7.6 | (O54946) DnaJ homolog subfamily B member 6 (Heat shock protein J2) (HSJ-2) (MRJ) (mDj4) | 8.688E-05 | 8.1933E-05 | 94% | A, C |
| P16045 | LEG1_MOUSE | 134 | 14735 | 5.5 | (P16045) Galectin-1 (Lectin galactoside-binding soluble 1) (Beta-galactoside-binding lectin L-14-I) (Lactose-binding lectin 1) (S-Lac lectin 1) (Galaptin) (14 kDa lectin) | 8.4964E-05 | 2.7692E-05 | 33% | A, C |
| Q00915 | RET1_MOUSE | 134 | 15715 | 5.2 | (Q00915) Retinol-binding protein I, cellular (Cellular retinol-binding protein) (CRBP) (mCRBPI) | 8.4964E-05 | 2.7692E-05 | 33% | A, C |
| P31230 | MCA1_MOUSE | 310 | 33997 | 8.4 | (P31230) Multisynthetase complex auxiliary component p43 [Contains: Endothelial monocyte-activating polypeptide 2 (EMAP-II) (Small inducible cytokine subfamily E member 1)] | 8.4751E-05 | 3.9918E-05 | 47% | A, C |
| Q9JLQ0 | CD2AP_MOUSE | 637 | 70432 | 6.4 | (Q9JLQ0) CD2-associated protein (Mesenchyme-to-epithelium transition protein with SH3 domains 1) (METS-1) | 8.4706E-05 | 4.6153E-06 | 5% | A, C |
| Q9R1T2 | ULE1A_MOUSE | 350 | 38620 | 5.4 | (Q9R1T2) Ubiquitin-like 1-activating enzyme E1A (SUMO-1-activating enzyme subunit 1) | 8.4119E-05 | 9.066E-05 | 108% | A, C |
| Q9DCH4 | IF35_MOUSE | 361 | 38000 | 5.6 | (Q9DCH4) Eukaryotic translation initiation factor 3 subunit 5 (eIF-3 epsilon) (eIF3 p47 subunit) (eIF3f) | 8.2479E-05 | 4.7999E-05 | 58% | A, C |
| Q8BUK6 | HOOK3_MOUSE | 718 | 83218 | 5.2 | (Q8BUK6) Hook homolog 3 | 8.2315E-05 | 1.9834E-05 | 24% | A, C |
| P24668 | MPRD_MOUSE | 278 | 31172 | 5.4 | (P24668) Cation-dependent mannose-6-phosphate receptor precursor (CD Man-6-P receptor) (CD-MPR) (46 kDa mannose 6-phosphate receptor) (MPR 46) | 8.1908E-05 | 2.6696E-05 | 33% | A, C |
| P07361 | A1AG2_MOUSE | 207 | 23843 | 5.5 | (P07361) Alpha-1-acid glycoprotein 2 precursor (AGP 2) (Orosomucoid-2) (OMD 2) | 8.0407E-05 | 6.5858E-05 | 82% | A, C |
| Q9WV69 | DEMA_MOUSE | 405 | 45468 | 8.4 | (Q9WV69) Dematin (Erythrocyte membrane protein band 4.9) | 7.7289E-05 | 1.1467E-05 | 15% | A, C |
| Q9CR26 | CF055_MOUSE | 309 | 33913 | 6.1 | (Q9CR26) Protein C6orf55 homolog | 7.3691E-05 | 2.4018E-05 | 33% | A, C |
| P47802 | MTX1_MOUSE | 317 | 35624 | 6.2 | (P47802) Metaxin-1 | 7.1831E-05 | 2.3412E-05 | 33% | A, C |

| | | | | | | | | | |
|---|---|---|---|---|---|---|---|---|---|
| Q8BH43 | WASF2_MOUSE | 497 | 54074 | 5.5 | (Q8BH43) Wiskott-Aldrich syndrome protein family member 2 (WASP-family protein member 2) (Protein WAVE-2) | 7.1681E-05 | 2.1646E-05 | 30% | A, C |
| Q9CXU9 | EIF1B_MOUSE | 113 | 12824 | 7.4 | (Q9CXU9) Eukaryotic translation initiation factor 1b (eIF1b) | 6.9761E-05 | 1.0993E-05 | 16% | A, C |
| Q9DC51 | GNAI3_MOUSE | 353 | 40407 | 5.7 | (Q9DC51) Guanine nucleotide-binding protein G(k) subunit alpha (G(i) alpha-3) | 6.9482E-05 | 4.2138E-05 | 61% | A, C |
| P62996 | TRA2B_MOUSE | 288 | 33666 | 11.2 | (P62996) Arginine/serine-rich-splicing factor 10 (Transformer-2-beta) (HTRA2-beta) (Transformer 2 protein homolog) (Silica-induced gene 41 protein) (SIG-41) | 6.9355E-05 | 2.9292E-05 | 42% | A, C |
| Q9D819 | IPYR_MOUSE | 289 | 32667 | 5.6 | (Q9D819) Inorganic pyrophosphatase (EC 3.6.1.1) (Pyrophosphate phospho-hydrolase) (PPase) | 6.9115E-05 | 2.919E-05 | 42% | A, C |
| P27048 | RSMB_MOUSE | 231 | 23656 | 10.9 | (P27048) Small nuclear ribonucleoprotein-associated protein B (snRNP-B) (Sm protein B) (Sm-B) (SmB) | 6.8251E-05 | 1.0755E-05 | 16% | A, C |
| Q811D0 | DLG1_MOUSE | 905 | 100120 | 5.8 | (Q811D0) Disks large homolog 1 (Synapse-associated protein 97) (SAP-97) (Embryo-dlg/synapse-associated protein 97) (E-dlg/SAP97) | 6.8027E-05 | 5.2422E-05 | 77% | A, C |
| Q9CT10 | RANB3_MOUSE | 491 | 52573 | 5.1 | (Q9CT10) Ran-binding protein 3 (RanBP3) | 6.7541E-05 | 4.5709E-05 | 68% | A, C |
| Q60739 | BAG1_MOUSE | 355 | 39740 | 8.5 | (Q60739) BAG family molecular chaperone regulator 1 (BCL-2-binding athanogene-1) (BAG-1) | 6.6131E-05 | 9.8112E-06 | 15% | A, C |
| Q9QYA2 | TOM40_MOUSE | 359 | 38001 | 8.4 | (Q9QYA2) Probable mitochondrial import receptor subunit TOM40 homolog (Translocase of outer membrane 40 kDa subunit homolog) | 6.5394E-05 | 9.7019E-06 | 15% | A, C |
| Q9WV55 | VAPA_MOUSE | 242 | 27280 | 8.4 | (Q9WV55) Vesicle-associated membrane protein-associated protein A (VAMP-associated protein A) (VAMP-A) (VAP-A) (33 kDa Vamp-associated protein) (VAP-33) | 6.5148E-05 | 1.0266E-05 | 16% | A, C |
| Q9D5V6 | SYAP1_MOUSE | 365 | 41350 | 4.5 | (Q9D5V6) Synapse-associated protein 1 | 6.4791E-05 | 1.021E-05 | 16% | A, C |
| P97315 | CSRP1_MOUSE | 192 | 20452 | 8.6 | (P97315) Cysteine and glycine-rich protein 1 (Cysteine-rich protein 1) (CRP1) (CRP) | 6.3873E-05 | 3.8736E-05 | 61% | A, C |
| P50431 | GLYC_MOUSE | 478 | 52585 | 6.9 | (P50431) Serine hydroxymethyltransferase, cytosolic (EC 2.1.2.1) (Serine methylase) (Glycine hydroxymethyltransferase) (SHMT) | 6.3768E-05 | 1.3437E-05 | 21% | A, C |
| P51125 | ICAL_MOUSE | 788 | 84922 | 5.5 | (P51125) Calpastatin (Calpain inhibitor) | 5.9585E-05 | 8.8401E-06 | 15% | A, C |
| P19091 | ANDR_MOUSE | 899 | 98194 | 6.8 | (P19091) Androgen receptor (Dihydrotestosterone receptor) | 5.9106E-05 | 1.5581E-05 | 26% | A, C |
| Q9D0I9 | SYR_MOUSE | 660 | 75674 | 7.6 | (Q9D0I9) Arginyl-tRNA synthetase (EC 6.1.1.19) (Arginine--tRNA ligase) (ArgRS) | 5.8388E-05 | 7.4805E-06 | 13% | A, C |
| Q9JIF7 | COPB_MOUSE | 953 | 107066 | 6 | (Q9JIF7) Coatomer subunit beta (Beta-coat protein) (Beta-COP) | 5.7709E-05 | 4.312E-05 | 75% | A, C |
| Q60902 | EP15R_MOUSE | 907 | 99295 | 5 | (Q60902) Epidermal growth factor receptor substrate 15-like 1 (Eps15-related protein) (Eps15R) (Epidermal growth factor receptor pathway substrate 15-related sequence) (Eps15-rs) | 5.768E-05 | 2.7645E-05 | 48% | A, C |
| Q8C854 | MYEF2_MOUSE | 591 | 63295 | 8.9 | (Q8C854) Myelin expression factor 2 (MyEF-2) (MEF-2) | 5.7502E-05 | 1.9249E-05 | 33% | A, C |
| Q99KV1 | DNJBB_MOUSE | 358 | 40555 | 6.3 | (Q99KV1) DnaJ homolog subfamily B member 11 precursor | 5.6275E-05 | 2.4245E-05 | 43% | A, C |
| Q8K0E8 | FIBB_MOUSE | 481 | 54753 | 7.1 | (Q8K0E8) Fibrinogen beta chain precursor [Contains: Fibrinopeptide B] | 5.6089E-05 | 3.0561E-06 | 5% | A, C |
| P35585 | AP1M1_MOUSE | 422 | 48412 | 7.3 | (P35585) AP-1 complex subunit mu-1 (Adaptor-related protein complex 1 mu-1 subunit) (Mu-adaptin 1) (Adaptor protein complex AP-1 mu-1 subunit) (Golgi adaptor HA1/AP1 adaptin mu-1 subunit) (Clathrin assembly protein assembly protein complex 1 | 5.5632E-05 | 8.2535E-06 | 15% | A, C |
| O70324 | MOT8_MOUSE | 565 | 62209 | 5.2 | (O70324) Monocarboxylate transporter 8 (MCT 8) (X-linked PEST-containing transporter) | 5.5402E-05 | 8.2194E-06 | 15% | A, C |

| | | | | | | | | | |
|---|---|---|---|---|---|---|---|---|---|
| P10711 | TCEA1_MOUSE | 301 | 33880 | 8.4 | (P10711) Transcription elongation factor A protein 1 (Transcription elongation factor S-II protein 1) (Transcription elongation factor TFIIS.o) | 5.4724E-05 | 4.4482E-05 | 81% | A, C |
| Q8R429 | AT2A1_MOUSE | 994 | 109425 | 5.2 | (Q8R429) Sarcoplasmic/endoplasmic reticulum calcium ATPase 1 (EC 3.6.3.8) (Calcium pump 1) (SERCA1) (SR Ca(2+)-ATPase 1) (Calcium-transporting ATPase sarcoplasmic reticulum type, fast twitch skeletal muscle isoform) (Endoplasmic reticulum cla | 5.4064E-05 | 4.656E-05 | 86% | A, C |
| Q920Q6 | MSI2H_MOUSE | 346 | 36939 | 8.5 | (Q920Q6) RNA-binding protein Musashi homolog 2 (Musashi-2) | 5.315E-05 | 3.9355E-05 | 74% | A, C |
| Q8C1B7 | SEP11_MOUSE | 430 | 49564 | 6.7 | (Q8C1B7) Septin-11 | 5.2687E-05 | 1.7637E-05 | 33% | A, C |
| P03958 | ADA_MOUSE | 351 | 39860 | 5.7 | (P03958) Adenosine deaminase (EC 3.5.4.4) (Adenosine aminohydrolase) | 5.2393E-05 | 3.8794E-05 | 74% | A, C |
| Q9Z315 | SNUT1_MOUSE | 806 | 90885 | 5.8 | (Q9Z315) U4/U6.U5 tri-snRNP-associated protein 1 (Squamous cell carcinoma antigen recognized by T-cells 1) (SART-1) (mSART-1) (Hypoxia-associated factor) | 4.9564E-05 | 2.0933E-05 | 42% | A, C |
| Q60936 | CABC1_MOUSE | 645 | 71743 | 6.5 | (Q60936) Chaperone-activity of bc1 complex-like, mitochondrial precursor (Chaperone-ABC1-like) | 4.7258E-05 | 9.9581E-06 | 21% | A, C |
| Q9CSN1 | SNW1_MOUSE | 536 | 61476 | 9.5 | (Q9CSN1) SNW domain-containing protein 1 (Nuclear protein SkiP) (Ski-interacting protein) | 4.5331E-05 | 2.7145E-05 | 60% | A, C |
| Q6P8X1 | SNX6_MOUSE | 406 | 46635 | 6 | (Q6P8X1) Sorting nexin-6 | 4.5295E-05 | 3.3539E-05 | 74% | A, C |
| Q60770 | STXB3_MOUSE | 592 | 67943 | 8 | (Q60770) Syntaxin-binding protein 3 (UNC-18 homolog 3) (UNC-18C) (MUNC-18-3) | 4.438E-05 | 2.0903E-05 | 47% | A, C |
| Q9R061 | NUBP2_MOUSE | 275 | 29518 | 6.5 | (Q9R061) Nucleotide-binding protein 2 (NBP 2) | 4.4177E-05 | 2.6454E-05 | 60% | A, C |
| P60762 | MO4L1_MOUSE | 362 | 41493 | 9.3 | (P60762) Mortality factor 4-like protein 1 (MORF-related gene 15 protein) (Transcription factor-like protein MRG15) (Testis-expressed gene 189 protein) | 4.3552E-05 | 6.863E-06 | 16% | A, C |
| Q60634 | FLOT2_MOUSE | 379 | 41659 | 5.2 | (Q60634) Flotillin-2 (Epidermal surface antigen) (ESA) | 4.3462E-05 | 3.5327E-05 | 81% | A, C |
| Q91VZ6 | SMAP1_MOUSE | 440 | 47660 | 8.5 | (Q91VZ6) Stromal membrane-associated protein 1 | 4.1795E-05 | 3.0947E-05 | 74% | A, C |
| Q923D5 | WBP11_MOUSE | 641 | 69875 | 8.4 | (Q923D5) WW domain-binding protein 11 (WBP-11) | 4.0808E-05 | 1.9558E-05 | 48% | A, C |
| Q8JZQ2 | AF3G2_MOUSE | 802 | 89519 | 8.6 | (Q8JZQ2) AFG3-like protein 2 (EC 3.4.24.-) | 4.0054E-05 | 1.959E-05 | 49% | A, C |
| Q8VDZ4 | ZDHC5_MOUSE | 715 | 77501 | 9 | (Q8VDZ4) Probable palmitoyltransferase ZDHHC5 (EC 2.3.1.-) (Zinc finger DHHC domain-containing protein 5) (DHHC-5) | 4.0029E-05 | 2.8901E-05 | 72% | A, C |
| Q99J21 | MCLN1_MOUSE | 580 | 65506 | 7.6 | (Q99J21) Mucolipin-1 (Mucolipidin) | 3.9259E-05 | 1.2796E-05 | 33% | A, C |
| Q9DBS9 | OSBL3_MOUSE | 855 | 96967 | 6.5 | (Q9DBS9) Oxysterol-binding protein-related protein 3 (OSBP-related protein 3) (ORP-3) | 3.6611E-05 | 5.4316E-06 | 15% | A, C |
| Q5SUF2 | CROP_MOUSE | 432 | 51450 | 9.8 | (Q5SUF2) Cisplatin resistance-associated overexpressed protein | 3.6495E-05 | 5.751E-06 | 16% | A, C |
| Q9D8N0 | EF1G_MOUSE | 436 | 49930 | 6.7 | (Q9D8N0) Elongation factor 1-gamma (EF-1-gamma) (eEF-1B gamma) | 3.616E-05 | 5.6982E-06 | 16% | A, C |
| Q8CH72 | TRI32_MOUSE | 655 | 72057 | 6.9 | (Q8CH72) Tripartite motif-containing protein 32 (EC 6.3.2.-) | 3.5842E-05 | 5.3175E-06 | 15% | A, C |
| Q9QZQ1 | AFAD_MOUSE | 885 | 100531 | 6.9 | (Q9QZQ1) Afadin (Protein Af-6) (Fragment) | 3.537E-05 | 5.2474E-06 | 15% | A, C |
| Q9DBH5 | LMAN2_MOUSE | 358 | 40416 | 7 | (Q9DBH5) Vesicular integral-membrane protein VIP36 precursor (Lectin, mannose-binding 2) | 3.4256E-05 | 2.0775E-05 | 61% | A, C |
| Q11011 | PSA_MOUSE | 920 | 103351 | 5.9 | (Q11011) Puromycin-sensitive aminopeptidase (EC 3.4.11.-) (PSA) | 3.3132E-05 | 6.9815E-06 | 21% | A, C |
| Q99LM2 | CK5P3_MOUSE | 503 | 56991 | 4.8 | (Q99LM2) CDK5 regulatory subunit-associated protein 3 | 3.309E-05 | 2.7103E-05 | 82% | A, C |
| P23116 | IF3A_MOUSE | 1344 | 161950 | 6.8 | (P23116) Eukaryotic translation initiation factor 3 subunit 10 (eIF-3 theta) (eIF3 p167) (eIF3 p180) (eIF3 p185) (eIF3a) (p162 protein) (Centrosomin) | 3.294E-05 | 1.7103E-05 | 52% | A, C |

| | | | | | | | | | |
|---|---|---|---|---|---|---|---|---|---|
| P35235 | PTN11_MOUSE | 597 | 68460 | 7.3 | (P35235) Tyrosine-protein phosphatase non-receptor type 11 (EC 3.1.3.48) (Protein-tyrosine phosphatase SYP) (SH-PTP2) (SHP-2) (Shp2) | 3.2275E-05 | 4.1349E-06 | 13% | A, C |
| Q9JIG7 | CCD22_MOUSE | 627 | 70844 | 6 | (Q9JIG7) Coiled-coil domain-containing protein 22 | 3.1857E-05 | 1.3454E-05 | 42% | A, C |
| Q6PDL0 | DC1L2_MOUSE | 492 | 54218 | 6.5 | (Q6PDL0) Cytoplasmic dynein 1 light intermediate chain 2 (Dynein light intermediate chain 2, cytosolic) | 3.1811E-05 | 4.7195E-06 | 15% | A, C |
| Q8CBE3 | WDR37_MOUSE | 496 | 55046 | 7.2 | (Q8CBE3) WD repeat protein 37 | 3.1786E-05 | 5.0089E-06 | 16% | A, C |
| O35551 | RABE1_MOUSE | 862 | 99552 | 5 | (O35551) Rab GTPase-binding effector protein 1 (Rabaptin-5) (Rabaptin-5alpha) | 3.1298E-05 | 1.7053E-06 | 5% | A, C |
| Q9CXT8 | MPPB_MOUSE | 489 | 54615 | 7 | (Q9CXT8) Mitochondrial-processing peptidase subunit beta, mitochondrial precursor (EC 3.4.24.64) (Beta-MPP) (P-52) | 3.0445E-05 | 1.7717E-05 | 58% | A, C |
| Q9JK81 | MYG1_MOUSE | 380 | 42723 | 7 | (Q9JK81) Protein MYG1 (Protein Gamm1) | 2.9961E-05 | 9.7652E-06 | 33% | A, C |
| Q8BH04 | PPCKM_MOUSE | 640 | 70528 | 7.3 | (Q8BH04) Phosphoenolpyruvate carboxykinase [GTP], mitochondrial precursor (EC 4.1.1.32) (Phosphoenolpyruvate carboxylase) (PEPCK-M) | 2.9927E-05 | 4.1109E-06 | 14% | A, C |
| O54941 | SMCE1_MOUSE | 411 | 46638 | 4.9 | (O54941) SWI/SNF-related matrix-associated actin-dependent regulator chromatin subfamily E member 1 (BRG1-associated factor 57) | 2.9838E-05 | 1.8096E-05 | 61% | A, C |
| Q9CY18 | SNX7_MOUSE | 387 | 45000 | 5.1 | (Q9CY18) Sorting nexin-7 | 2.9419E-05 | 9.5885E-06 | 33% | A, C |
| O70585 | DTNB_MOUSE | 700 | 78356 | 8.4 | (O70585) Dystrobrevin beta (Beta-dystrobrevin) (DTN-B) (MDTN-B) | 2.8535E-05 | 1.2051E-05 | 42% | A, C |
| Q8C754 | VPS52_MOUSE | 723 | 82044 | 5.9 | (Q8C754) Vacuolar protein sorting protein 52 | 2.7627E-05 | 1.1668E-05 | 42% | A, C |
| Q6R5P0 | TLR11_MOUSE | 926 | 105873 | 7.3 | (Q6R5P0) Toll-like receptor 11 precursor | 2.7424E-05 | 2.5402E-05 | 93% | A, C |
| P60670 | NPL4_MOUSE | 607 | 67886 | 6.5 | (P60670) Nuclear protein localization protein 4 homolog (Protein NPL4) | 2.5973E-05 | 4.0929E-06 | 16% | A, C |
| Q9D6J3 | CCD94_MOUSE | 314 | 35988 | 6.1 | (Q9D6J3) Coiled-coil domain-containing protein 94 | 2.5105E-05 | 3.9561E-06 | 16% | A, C |
| Q8C8U0 | LIPB1_MOUSE | 969 | 108540 | 5.5 | (Q8C8U0) Liprin-beta-1 (Protein tyrosine phosphatase receptor type f polypeptide-interacting protein-binding protein 1) (PTPRF-interacting protein-binding protein 1) | 2.5075E-05 | 1.5015E-05 | 60% | A, C |
| Q80W68 | KIRR1_MOUSE | 789 | 87176 | 5.9 | (Q80W68) Kin of IRRE-like protein 1 precursor (Kin of irregular chiasm-like protein 1) (Nephrin-like protein 1) | 2.4421E-05 | 3.1287E-06 | 13% | A, C |
| Q8R066 | C1QT4_MOUSE | 326 | 35058 | 9 | (Q8R066) Complement C1q tumor necrosis factor-related protein 4 precursor | 2.4181E-05 | 3.8105E-06 | 16% | A, C |
| Q80X19 | COEA1_MOUSE | 1797 | 193113 | 5.1 | (Q80X19) Collagen alpha-1(XIV) chain precursor | 2.3266E-05 | 5.6844E-06 | 24% | A, C |
| Q91YD6 | VILL_MOUSE | 859 | 96509 | 6.3 | (Q91YD6) Villin-like protein (EF-6) | 2.3253E-05 | 9.8207E-06 | 42% | A, C |
| Q61035 | SYH_MOUSE | 509 | 57416 | 5.8 | (Q61035) Histidyl-tRNA synthetase (EC 6.1.1.21) (Histidine--tRNA ligase) (HisRS) | 2.2368E-05 | 7.2903E-06 | 33% | A, C |
| Q9JI39 | ABCBA_MOUSE | 715 | 77188 | 9.6 | (Q9JI39) ATP-binding cassette sub-family B member 10, mitochondrial precursor (ATP-binding cassette transporter 10) (ABC transporter 10 protein) (ABC-mitochondrial erythroid protein) (ABC-me protein) | 2.189E-05 | 3.2475E-06 | 15% | A, C |
| P12023 | A4_MOUSE | 770 | 86722 | 4.8 | (P12023) Amyloid beta A4 protein precursor (APP) (ABPP) (Alzheimer disease amyloid protein homolog) (Amyloidogenic glycoprotein) (AG) [Contains: Soluble APP-alpha (S-APP-alpha); Soluble APP-beta (S-APP-beta); C99 (APP-C99); Beta-amyloid protein | 2.1616E-05 | 1.7705E-05 | 82% | A, C |
| Q61140 | BCAR1_MOUSE | 874 | 94257 | 5.7 | (Q61140) Breast cancer anti-estrogen resistance protein 1 (CRK-associated substrate) (p130cas) | 2.1041E-05 | 1.558E-05 | 74% | A, C |
| Q9ERG0 | LIMA1_MOUSE | 753 | 84090 | 6.6 | (Q9ERG0) LIM domain and actin-binding protein 1 (Epithelial protein lost in neoplasm) (mEPLIN) | 2.0937E-05 | 3.2994E-06 | 16% | A, C |

| | | | | | | | | | |
|---|---|---|---|---|---|---|---|---|---|
| Q9JLV5 | CUL3_MOUSE | 768 | 88948 | 8.4 | (Q9JLV5) Cullin-3 (CUL-3) | 2.0528E-05 | 3.2349E-06 | 16% | A, C |
| Q924X2 | CPT1B_MOUSE | 772 | 88217 | 8.4 | (Q924X2) Carnitine O-palmitoyltransferase I, muscle isoform (EC 2.3.1.21) (CPT I) (CPTI-M) (Carnitine palmitoyltransferase 1B) | 2.0422E-05 | 3.2182E-06 | 16% | A, C |
| Q8CHU3 | EPN2_MOUSE | 595 | 63472 | 8.2 | (Q8CHU3) Epsin-2 (EPS-15-interacting protein 2) (Intersectin-EH-binding protein 2) (Ibp2) | 2.0418E-05 | 1.2227E-05 | 60% | A, C |
| Q8BXR9 | OSBL6_MOUSE | 959 | 108920 | 7.3 | (Q8BXR9) Oxysterol-binding protein-related protein 6 (OSBP-related protein 6) (ORP-6) | 1.9972E-05 | 2.7435E-06 | 14% | A, C |
| P49442 | INPP_MOUSE | 396 | 43346 | 5 | (P49442) Inositol polyphosphate 1-phosphatase (EC 3.1.3.57) (IPPase) (IPP) | 1.9906E-05 | 3.1369E-06 | 16% | A, C |
| Q8CH18 | CCAR1_MOUSE | 1146 | 132060 | 5.8 | (Q8CH18) Cell division cycle and apoptosis regulator protein 1 (Cell cycle and apoptosis regulatory protein 1) (CARP-1) | 1.987E-05 | 6.476E-06 | 33% | A, C |
| P27046 | MA2A1_MOUSE | 1150 | 131589 | 8 | (P27046) Alpha-mannosidase 2 (EC 3.2.1.114) (Alpha-mannosidase II) (Mannosyl-oligosaccharide 1,3-1,6-alpha-mannosidase) (MAN II) (Golgi alpha-mannosidase II) (Mannosidase alpha class 2A member 1) (AMAN II) | 1.9701E-05 | 6.5947E-06 | 33% | A, C |
| Q6Y685 | TACC1_MOUSE | 774 | 83952 | 5 | (Q6Y685) Transforming acidic coiled-coil-containing protein 1 | 1.9235E-05 | 1.1193E-05 | 58% | A, C |
| Q9R0K7 | AT2B2_MOUSE | 1198 | 132587 | 6 | (Q9R0K7) Plasma membrane calcium-transporting ATPase 2 (EC 3.6.3.8) (PMCA2) (Plasma membrane calcium pump isoform 2) (Plasma membrane calcium ATPase isoform 2) | 1.9007E-05 | 6.1949E-06 | 33% | A, C |
| Q9DBE9 | RRMJ3_MOUSE | 838 | 95532 | 8.4 | (Q9DBE9) Putative rRNA methyltransferase 3 (EC 2.1.1.-) (rRNA (uridine-2'-O-)-methyltransferase 3) | 1.8677E-05 | 2.7709E-06 | 15% | A, C |
| Q6ZPS6 | AKIB1_MOUSE | 1087 | 122118 | 5.2 | (Q6ZPS6) Ankyrin repeat and IBR domain-containing protein 1 (Fragment) | 1.8376E-05 | 7.7608E-06 | 42% | A, C |
| Q9Z1T1 | AP3B1_MOUSE | 1105 | 122870 | 5.7 | (Q9Z1T1) AP-3 complex subunit beta-1 (Adapter-related protein complex 3 beta-1 subunit) (Beta3A-adaptin) (Adaptor protein complex AP-3 beta-1 subunit) (Clathrin assembly protein complex 3 beta-1 large chain) | 1.8076E-05 | 7.6344E-06 | 42% | A, C |
| Q8R180 | ERO1A_MOUSE | 464 | 54038 | 6.5 | (Q8R180) ERO1-like protein alpha precursor (EC 1.8.4.-) (ERO1-Lalpha) (Oxidoreductin-1-Lalpha) (Endoplasmic oxidoreductin-1-like protein) (ERO1-L) | 1.6989E-05 | 2.6772E-06 | 16% | A, C |
| Q8C5Q4 | GRSF1_MOUSE | 479 | 53076 | 6.7 | (Q8C5Q4) G-rich sequence factor 1 (GRSF-1) | 1.6457E-05 | 2.5933E-06 | 16% | A, C |
| Q9Z0R6 | ITSN2_MOUSE | 1658 | 188775 | 8.1 | (Q9Z0R6) Intersectin-2 (SH3 domain-containing protein 1B) (EH and SH3 domains protein 2) (EH domain and SH3 domain regulator of endocytosis 2) | 1.6272E-05 | 8.866E-07 | 5% | A, C |
| Q80TZ3 | AUXI_MOUSE | 938 | 102299 | 7.2 | (Q80TZ3) Putative tyrosine-protein phosphatase auxilin (EC 3.1.3.48) (DnaJ homolog subfamily C member 6) | 1.5872E-05 | 9.2364E-06 | 58% | A, C |
| Q9R0M4 | PODXL_MOUSE | 503 | 53389 | 5 | (Q9R0M4) Podocalyxin-like protein 1 precursor | 1.5672E-05 | 2.4696E-06 | 16% | A, C |
| Q03173 | ENAH_MOUSE | 802 | 85844 | 7.7 | (Q03173) Protein enabled homolog (NPC-derived proline-rich protein 1) (NDPP-1) | 1.5291E-05 | 9.2736E-06 | 61% | A, C |
| P12382 | K6PL_MOUSE | 779 | 85170 | 7.1 | (P12382) 6-phosphofructokinase, liver type (EC 2.7.1.11) (Phosphofructokinase 1) (Phosphohexokinase) (Phosphofructo-1 kinase isozyme B) (PFK-B) | 1.4615E-05 | 4.7635E-06 | 33% | A, C |
| Q3UHD1 | BAI1_MOUSE | 1582 | 173295 | 7.6 | (Q3UHD1) Brain-specific angiogenesis inhibitor 1 precursor | 1.4393E-05 | 4.6912E-06 | 33% | A, C |
| Q9D824 | FIP1_MOUSE | 581 | 64959 | 5.8 | (Q9D824) Pre-mRNA 3'-end-processing factor FIP1 (FIP1-like 1) | 1.3568E-05 | 2.1381E-06 | 16% | A, C |
| Q01279 | EGFR_MOUSE | 1210 | 134853 | 6.9 | (Q01279) Epidermal growth factor receptor precursor (EC 2.7.10.1) | 1.303E-05 | 2.0532E-06 | 16% | A, C |

| | | | | | | | | | |
|---|---|---|---|---|---|---|---|---|---|
| Q9Z160 | COG1_MOUSE | 980 | 109050 | 7.2 | (Q9Z160) Conserved oligomeric Golgi complex component 1 (Low density lipoprotein receptor defect B-complementing protein) | 1.2397E-05 | 7.4234E-06 | 60% | A, C |
| P51432 | PLCB3_MOUSE | 1234 | 139492 | 6 | (P51432) 1-phosphatidylinositol-4,5-bisphosphate phosphodiesterase beta 3 (EC 3.1.4.11) (Phosphoinositide phospholipase C) (Phospholipase C-beta-3) (PLC-beta-3) | 1.2064E-05 | 7.0209E-06 | 58% | A, C |
| P81122 | IRS2_MOUSE | 1321 | 136527 | 8.7 | (P81122) Insulin receptor substrate 2 (IRS-2) (4PS) | 1.1935E-05 | 1.8807E-06 | 16% | A, C |
| Q61595 | KTN1_MOUSE | 1327 | 152592 | 5.9 | (Q61595) Kinectin | 1.1794E-05 | 1.7498E-06 | 15% | A, C |
| Q64127 | TIF1A_MOUSE | 1051 | 116657 | 7.1 | (Q64127) Transcription intermediary factor 1-alpha (TIF1-alpha) (Tripartite motif-containing protein 24) | 1.1669E-05 | 7.0765E-06 | 61% | A, C |
| P97927 | LAMA4_MOUSE | 1816 | 201818 | 6.2 | (P97927) Laminin alpha-4 chain precursor | 1.061E-05 | 1.3593E-06 | 13% | A, C |
| Q8R4H2 | ARHGC_MOUSE | 1543 | 172321 | 5.7 | (Q8R4H2) Rho guanine nucleotide exchange factor 12 (Leukemia-associated RhoGEF) | 9.6484E-06 | 5.6149E-06 | 58% | A, C |
| Q61645 | HAIR_MOUSE | 1182 | 127193 | 7.4 | (Q61645) Protein hairless | 9.6322E-06 | 3.1394E-06 | 33% | A, C |
| O35927 | CTND2_MOUSE | 1247 | 134999 | 7.7 | (O35927) Catenin delta-2 (Neural plakophilin-related ARM-repeat protein) (NPRAP) (Neurojungin) | 9.1301E-06 | 2.9758E-06 | 33% | A, C |
| O35206 | COFA1_MOUSE | 1367 | 140525 | 4.9 | (O35206) Collagen alpha-1(XV) chain precursor [Contains: Endostatin (Endostatin-XV)] | 8.9712E-06 | 5.4407E-06 | 61% | A, C |
| Q3TJ91 | L2GL2_MOUSE | 1027 | 114323 | 7.5 | (Q3TJ91) Lethal(2) giant larvae protein homolog 2 (Lethal giant larvae-like protein 2) | 7.6757E-06 | 1.2096E-06 | 16% | A, C |
| Q8VDC1 | FYCO1_MOUSE | 1437 | 162336 | 5 | (Q8VDC1) FYVE and coiled-coil domain-containing protein 1 | 5.4857E-06 | 8.6445E-07 | 16% | A, C |
| Q9ESK9 | RBCC1_MOUSE | 1588 | 182364 | 5.5 | (Q9ESK9) RB1-inducible coiled-coil protein 1 (Coiled-coil-forming protein 1) (LaXp180 protein) | 4.9641E-06 | 7.8225E-07 | 16% | A, C |
| Q8R0W0 | EPIPL_MOUSE | 6548 | 724679 | 6.1 | (Q8R0W0) Epiplakin | 4.9058E-06 | 2.3993E-06 | 49% | A, C |
| P56565 | S10A1_MOUSE | 93 | 10374 | 4.5 | (P56565) Protein S100-A1 (S100 calcium-binding protein A1) (S-100 protein alpha subunit) (S-100 protein alpha chain) | 0.00250074 | 0.00193884 | 78% | B, C |
| Q9CY16 | RT28_MOUSE | 186 | 20520 | 8.9 | (Q9CY16) Mitochondrial 28S ribosomal protein S28 (S28mt) (MRP-S28) | 0.00044647 | 0.00041837 | 94% | B, C |
| Q9CPQ8 | ATP5L_MOUSE | 103 | 11424 | 9.7 | (Q9CPQ8) ATP synthase subunit g, mitochondrial (EC 3.6.3.14) (ATPase subunit g) | 0.00037221 | 0.00014169 | 38% | B, C |
| P05533 | LY6A_MOUSE | 134 | 14377 | 4.8 | (P05533) Lymphocyte antigen Ly-6A.2/Ly-6E.1 precursor (T-cell-activating protein) (TAP) | 0.00034855 | 0.0001233 | 35% | B, C |
| Q99L04 | DHRS1_MOUSE | 313 | 34005 | 8.4 | (Q99L04) Dehydrogenase/reductase SDR family member 1 (EC 1.1.-.-) | 0.0003233 | 1.7514E-05 | 5% | B, C |
| Q9D6Y7 | MSRA_MOUSE | 233 | 25988 | 8.4 | (Q9D6Y7) Peptide methionine sulfoxide reductase (EC 1.8.4.6) (Protein-methionine-S-oxide reductase) (PMSR) (Peptide Met(O) reductase) | 0.00031658 | 0.00032016 | 101% | B, C |
| Q91Y63 | S13A3_MOUSE | 600 | 66143 | 7.7 | (Q91Y63) Solute carrier family 13 member 3 (Sodium-dependent high-affinity dicarboxylate transporter 2) (Na(+)/dicarboxylate cotransporter 3) (NaDC-3) (mNaDC3) | 0.00026788 | 0.00014947 | 56% | B, C |
| Q9JJU8 | SH3L1_MOUSE | 114 | 12811 | 4.9 | (Q9JJU8) SH3 domain-binding glutamic acid-rich-like protein | 0.00022758 | 0.00019951 | 88% | B, C |
| Q9CPP6 | NDUA5_MOUSE | 115 | 13229 | 8.1 | (Q9CPP6) NADH dehydrogenase [ubiquinone] 1 alpha subcomplex subunit 5 (EC 1.6.5.3) (EC 1.6.99.3) (NADH-ubiquinone oxidoreductase 13 kDa-B subunit) (Complex I-13Kd-B) (CI-13Kd-B) (Complex I subunit B13) | 0.0002183 | 5.0303E-05 | 23% | B, C |
| P46638 | RB11B_MOUSE | 217 | 24358 | 5.9 | (P46638) Ras-related protein Rab-11B | 0.00019552 | 0.00023086 | 118% | B, C |
| O08997 | ATOX1_MOUSE | 68 | 7338 | 6.5 | (O08997) Copper transport protein ATOX1 (Metal transport protein ATX1) | 0.00017552 | 4.3125E-05 | 25% | B, C |
| O35309 | NMI_MOUSE | 314 | 35236 | 5 | (O35309) N-myc-interactor (Nmi) (N-myc and STAT interactor) | 0.0001599 | 3.6846E-05 | 23% | B, C |

| | | | | | | | | | |
|---|---|---|---|---|---|---|---|---|---|
| P70279 | SURF6_MOUSE | 355 | 41235 | 10.5 | (P70279) Surfeit locus protein 6 | 0.00015951 | 0.00010927 | 69% | B, C |
| Q11136 | PEPD_MOUSE | 492 | 54898 | 5.8 | (Q11136) Xaa-Pro dipeptidase (EC 3.4.13.9) (X-Pro dipeptidase) (Proline dipeptidase) (Prolidase) (Imidodipeptidase) (Peptidase 4) | 0.00015345 | 0.00010513 | 69% | B, C |
| Q9D7P6 | NIFUN_MOUSE | 168 | 18098 | 9.3 | (Q9D7P6) NifU-like N-terminal domain-containing protein, mitochondrial precursor (NifU-like protein) (Iron-sulfur cluster assembly enzyme ISCU) | 0.00014943 | 3.4434E-05 | 23% | B, C |
| Q9D6S7 | RRFM_MOUSE | 262 | 29051 | 9.8 | (Q9D6S7) Ribosome recycling factor, mitochondrial precursor | 0.00014633 | 5.5704E-05 | 38% | B, C |
| Q9Z1J3 | NFS1_MOUSE | 451 | 50001 | 8.5 | (Q9Z1J3) Cysteine desulfurase, mitochondrial precursor (EC 2.8.1.7) (m-Nfs1) | 0.00014239 | 1.8275E-05 | 13% | B, C |
| O35988 | SDC4_MOUSE | 198 | 21482 | 4.4 | (O35988) Syndecan-4 precursor (SYND4) (Ryudocan core protein) | 0.00013825 | 5.4637E-05 | 40% | B, C |
| Q91WU5 | AS3MT_MOUSE | 376 | 41793 | 6.1 | (Q91WU5) Arsenite methyltransferase (EC 2.1.1.137) (S-adenosyl-L-methionine:arsenic(III) methyltransferase) (Methylarsonite methyltransferase) | 0.00013197 | 7.6824E-05 | 58% | B, C |
| Q9JIK9 | RT34_MOUSE | 218 | 25827 | 10.4 | (Q9JIK9) Mitochondrial 28S ribosomal protein S34 (S34mt) (MRP-S34) (T-complex expressed gene 2 protein) | 0.00012767 | 0.00013511 | 106% | B, C |
| P03930 | ATP8_MOUSE | 67 | 7766 | 9.9 | (P03930) ATP synthase protein 8 (EC 3.6.3.14) (ATPase subunit 8) (A6L) | 0.00012587 | 3.0156E-05 | 24% | B, C |
| Q99JY3 | GIMA4_MOUSE | 219 | 24554 | 7 | (Q99JY3) GTPase, IMAP family member 4 (Immunity-associated protein 4) (Immunity-associated nucleotide 1 protein) | 0.00012499 | 4.9397E-05 | 40% | B, C |
| P30115 | GSTA3_MOUSE | 220 | 25229 | 8.7 | (P30115) Glutathione S-transferase Yc (EC 2.5.1.18) (GST class alpha) (Ya3) | 0.00012149 | 8.1762E-05 | 67% | B, C |
| O35639 | ANXA3_MOUSE | 322 | 36240 | 5.5 | (O35639) Annexin A3 (Annexin III) (Lipocortin III) (Placental anticoagulant protein III) (PAP-III) (35-alpha calcimedin) | 0.0001132 | 0.00011678 | 103% | B, C |
| Q9QZ73 | DCNL1_MOUSE | 259 | 30097 | 5.3 | (Q9QZ73) DCN1-like protein 1 (Defective in cullin neddylation protein 1-like protein 1) (DCUN1 domain-containing protein 1) (Testis-specific protein 3) | 0.00011045 | 3.212E-06 | 3% | B, C |
| Q9QYJ3 | DNJB1_MOUSE | 339 | 38036 | 8.6 | (Q9QYJ3) DnaJ homolog subfamily B member 1 (Heat shock 40 kDa protein 1) (Heat shock protein 40) (HSP40) | 0.00010907 | 8.1422E-06 | 7% | B, C |
| Q8R311 | CTGE5_MOUSE | 779 | 87719 | 5.1 | (Q8R311) Cutaneous T-cell lymphoma-associated antigen 5 homolog (cTAGE-5 protein) (Meningioma-expressed antigen 6) | 0.00010893 | 0.00012862 | 118% | B, C |
| Q9ESX5 | DKC1_MOUSE | 508 | 57371 | 9.2 | (Q9ESX5) H/ACA ribonucleoprotein complex subunit 4 (EC 5.4.99.-) (Dyskerin) (Nucleolar protein family A member 4) (snoRNP protein DKC1) (Nopp140-associated protein of 57 kDa) (Nucleolar protein NAP57) | 8.5047E-05 | 4.2275E-05 | 50% | B, C |
| Q9DCV4 | FA82B_MOUSE | 305 | 35000 | 8.7 | (Q9DCV4) Protein FAM82B | 8.2309E-05 | 1.8967E-05 | 23% | B, C |
| Q62189 | SNRPA_MOUSE | 286 | 31704 | 9.8 | (Q62189) U1 small nuclear ribonucleoprotein A (U1 snRNP protein A) (U1A protein) (U1-A) | 8.0299E-05 | 7.8923E-05 | 98% | B, C |
| P61021 | RAB5B_MOUSE | 215 | 23707 | 8.1 | (P61021) Ras-related protein Rab-5B | 7.7843E-05 | 1.7938E-05 | 23% | B, C |
| Q9R0A0 | PEX14_MOUSE | 376 | 41208 | 5.1 | (Q9R0A0) Peroxisomal membrane protein PEX14 (Peroxin-14) (Peroxisomal membrane anchor protein PEX14) (PTS1 receptor docking protein) | 7.6601E-05 | 2.9478E-06 | 4% | B, C |
| Q8CCS6 | PABP2_MOUSE | 301 | 32165 | 5.2 | (Q8CCS6) Polyadenylate-binding protein 2 (Poly(A)-binding protein 2) (Poly(A)-binding protein II) (PABII) (Polyadenylate-binding nuclear protein 1) (Nuclear poly(A)-binding protein 1) | 7.6297E-05 | 7.499E-05 | 98% | B, C |

| | | | | | | | | | |
|---|---|---|---|---|---|---|---|---|---|
| Q9D8B3 | CHM4B_MOUSE | 224 | 24936 | 4.8 | (Q9D8B3) Charged multivesicular body protein 4b (Chromatin-modifying protein 4b) (CHMP4b) | 7.5297E-05 | 1.804E-05 | 24% | B, C |
| Q8K0Q5 | RHG18_MOUSE | 663 | 74930 | 6.7 | (Q8K0Q5) Rho-GTPase-activating protein 18 | 7.2503E-05 | 4.2771E-05 | 59% | B, C |
| Q9QUI0 | RHOA_MOUSE | 193 | 21782 | 6.1 | (Q9QUI0) Transforming protein RhoA precursor | 6.9245E-05 | 4.66E-05 | 67% | B, C |
| Q91WK2 | IF33_MOUSE | 352 | 39832 | 6.7 | (Q91WK2) Eukaryotic translation initiation factor 3 subunit 3 (eIF-3 gamma) (eIF3 p40 subunit) (eIF3h) | 6.7815E-05 | 1.6662E-05 | 25% | B, C |
| O54734 | OST48_MOUSE | 441 | 49014 | 5.8 | (O54734) Dolichyl-diphosphooligosaccharide--protein glycosyltransferase 48 kDa subunit precursor (EC 2.4.1.119) (Oligosaccharyl transferase 48 kDa subunit) (DDOST 48 kDa subunit) | 6.6773E-05 | 6.2806E-05 | 94% | B, C |
| P18894 | OXDA_MOUSE | 346 | 38714 | 7.3 | (P18894) D-amino-acid oxidase (EC 1.4.3.3) (DAMOX) (DAO) (DAAO) | 6.6374E-05 | 6.5237E-05 | 98% | B, C |
| Q9EQI8 | RM46_MOUSE | 283 | 32132 | 7.4 | (Q9EQI8) 39S ribosomal protein L46, mitochondrial precursor (L46mt) (MRP-L46) | 6.3956E-05 | 5.5445E-05 | 87% | B, C |
| P58137 | ACOT8_MOUSE | 320 | 35827 | 7.6 | (P58137) Acyl-coenzyme A thioesterase 8 (EC 3.1.2.2) (Acyl-CoA thioesterase 8) (Peroxisomal acyl-coenzyme A thioester hydrolase 1) (PTE-1) (Peroxisomal long-chain acyl-coA thioesterase 1) (Peroxisomal acyl-CoA thioesterase 2) (PTE-2) | 6.3652E-05 | 2.8502E-06 | 4% | B, C |
| O88342 | WDR1_MOUSE | 605 | 66276 | 6.6 | (O88342) WD repeat protein 1 (Actin-interacting protein 1) (AIP1) | 6.1115E-05 | 4.5623E-06 | 7% | B, C |
| Q64337 | SQSTM_MOUSE | 442 | 48163 | 5.2 | (Q64337) Sequestosome-1 (Ubiquitin-binding protein p62) (STONE14) | 6.0471E-05 | 4.0696E-05 | 67% | B, C |
| Q99KH8 | STK24_MOUSE | 431 | 47954 | 5.4 | (Q99KH8) Serine/threonine-protein kinase 24 (EC 2.7.11.1) | 6.0196E-05 | 5.2771E-05 | 88% | B, C |
| Q8VCE2 | XAB1_MOUSE | 372 | 41598 | 4.9 | (Q8VCE2) XPA-binding protein 1 | 5.807E-05 | 2.8865E-05 | 50% | B, C |
| Q62318 | TIF1B_MOUSE | 833 | 88716 | 5.8 | (Q62318) Transcription intermediary factor 1-beta (TIF1-beta) (Tripartite motif-containing protein 28) (KRAB-A-interacting protein) (KRIP-1) | 5.7931E-05 | 4.8884E-05 | 84% | B, C |
| Q9Z2A0 | PDPK1_MOUSE | 559 | 63759 | 7.4 | (Q9Z2A0) 3-phosphoinositide-dependent protein kinase 1 (EC 2.7.11.1) (mPDK1) | 5.744E-05 | 7.3722E-06 | 13% | B, C |
| Q91WL5 | CP4AC_MOUSE | 508 | 58332 | 9.1 | (Q91WL5) Cytochrome P450 4A12 (EC 1.14.14.1) (CYPIVA12) | 5.6312E-05 | 1.6376E-06 | 3% | B, C |
| O70591 | PFD2_MOUSE | 154 | 16534 | 6.6 | (O70591) Prefoldin subunit 2 | 5.4761E-05 | 1.312E-05 | 24% | B, C |
| Q9Z1R2 | BAT3_MOUSE | 1154 | 121037 | 5.7 | (Q9Z1R2) Large proline-rich protein BAT3 (HLA-B-associated transcript 3) | 5.4304E-05 | 4.2462E-05 | 78% | B, C |
| Q9DBG3 | AP2B1_MOUSE | 937 | 104583 | 5.4 | (Q9DBG3) AP-2 complex subunit beta-1 (Adapter-related protein complex 2 beta-1 subunit) (Beta-adaptin) (Plasma membrane adaptor HA2/AP2 adaptin beta subunit) (Clathrin assembly protein complex 2 beta large chain) (AP105B) | 5.2956E-05 | 3.0828E-05 | 58% | B, C |
| O88983 | STX8_MOUSE | 236 | 26925 | 5 | (O88983) Syntaxin-8 (Syntaxin-like protein 3I35) | 5.0574E-05 | 1.2426E-05 | 25% | B, C |
| Q80XA6 | REPS2_MOUSE | 521 | 57750 | 7.5 | (Q80XA6) RalBP1-associated Eps domain-containing protein 2 (RalBP1-interacting protein 2) (Partner of RalBP1) | 4.856E-05 | 1.1634E-05 | 24% | B, C |
| Q61235 | SNTB2_MOUSE | 520 | 56382 | 8.7 | (Q61235) Beta-2-syntrophin (59 kDa dystrophin-associated protein A1 basic component 2) (Syntrophin 3) (SNT3) (Syntrophin-like) (SNTL) | 4.8277E-05 | 1.1125E-05 | 23% | B, C |
| Q91W50 | CSDE1_MOUSE | 798 | 88791 | 6.4 | (Q91W50) Cold shock domain-containing protein E1 | 4.5679E-05 | 4.7122E-05 | 103% | B, C |
| Q9CZA6 | NDE1_MOUSE | 344 | 38523 | 5.3 | (Q9CZA6) Nuclear distribution protein nudE homolog 1 (NudE) (mNudE) | 4.4877E-05 | 2.2923E-05 | 51% | B, C |

| | | | | | | | | | |
|---|---|---|---|---|---|---|---|---|---|
| P31809 | CEAM1_MOUSE | 521 | 57016 | 5.6 | (P31809) Carcinoembryonic antigen-related cell adhesion molecule 1 precursor (Biliary glycoprotein 1) (BGP-1) (Murine hepatitis virus receptor) (MHV-R) (Biliary glycoprotein D) | 4.458E-05 | 4.4032E-05 | 99% | B, C |
| Q61646 | HPT_MOUSE | 347 | 38752 | 6.3 | (Q61646) Haptoglobin precursor [Contains: Haptoglobin alpha chain; Haptoglobin beta chain] | 4.4489E-05 | 2.2725E-05 | 51% | B, C |
| Q08857 | CD36_MOUSE | 471 | 52567 | 8.3 | (Q08857) Platelet glycoprotein 4 (Platelet glycoprotein IV) (GPIV) (GPIIIB) (CD36 antigen) (PAS IV) (PAS-4 protein) | 4.3246E-05 | 1.9365E-06 | 4% | B, C |
| Q9ER88 | RT29_MOUSE | 391 | 44699 | 8.9 | (Q9ER88) Mitochondrial 28S ribosomal protein S29 (S29mt) (MRP-S29) (Death-associated protein 3) (DAP-3) | 4.3137E-05 | 1.0335E-05 | 24% | B, C |
| P62915 | TF2B_MOUSE | 316 | 34819 | 8.4 | (P62915) Transcription initiation factor IIB (General transcription factor TFIIB) (RNA polymerase II alpha initiation factor) | 4.2292E-05 | 2.8462E-05 | 67% | B, C |
| Q8C0C0 | ZHX2_MOUSE | 836 | 92259 | 7.5 | (Q8C0C0) Zinc fingers and homeoboxes protein 2 (Zinc finger and homeodomain protein 2) (Alpha-fetoprotein regulator 1) (AFP regulator 1) (Regulator of AFP) | 4.167E-05 | 2.3382E-05 | 56% | B, C |
| Q8VEJ9 | VPS4A_MOUSE | 437 | 48907 | 7.8 | (Q8VEJ9) Vacuolar sorting protein 4a | 4.1418E-05 | 3.5906E-05 | 87% | B, C |
| Q922B2 | SYD_MOUSE | 501 | 57117 | 6.5 | (Q922B2) Aspartyl-tRNA synthetase (EC 6.1.1.12) (Aspartate--tRNA ligase) (AspRS) | 4.0656E-05 | 1.8205E-06 | 4% | B, C |
| P28741 | KIF3A_MOUSE | 701 | 80168 | 6.5 | (P28741) Kinesin-like protein KIF3A (Microtubule plus end-directed kinesin motor 3A) | 3.9049E-05 | 1.5432E-05 | 40% | B, C |
| P27889 | HNF1B_MOUSE | 558 | 61588 | 7.8 | (P27889) Hepatocyte nuclear factor 1-beta (HNF-1beta) (HNF-1B) (Variant hepatic nuclear factor 1) (VHNF1) (Homeoprotein LFB3) | 3.8713E-05 | 1.9243E-05 | 50% | B, C |
| Q9WUH1 | TM115_MOUSE | 350 | 38099 | 7.5 | (Q9WUH1) Transmembrane protein 115 (Protein PL6 homolog) | 3.8183E-05 | 2.5697E-05 | 67% | B, C |
| Q62384 | ZPR1_MOUSE | 459 | 50715 | 4.8 | (Q62384) Zinc-finger protein ZPR1 (Zinc finger protein 259) | 3.6746E-05 | 8.8037E-06 | 24% | B, C |
| P28653 | PGS1_MOUSE | 369 | 41639 | 7.3 | (P28653) Biglycan precursor (Bone/cartilage proteoglycan I) (PG-S1) | 3.5864E-05 | 2.3874E-05 | 67% | B, C |
| P42227 | STAT3_MOUSE | 770 | 88054 | 6.3 | (P42227) Signal transducer and activator of transcription 3 (Acute-phase response factor) | 3.555E-05 | 1.4049E-05 | 40% | B, C |
| Q8VI47 | MRP2_MOUSE | 1543 | 173698 | 8.7 | (Q8VI47) Canalicular multispecific organic anion transporter 1 (ATP-binding cassette sub-family C member 2) | 3.4428E-05 | 2.1931E-05 | 64% | B, C |
| Q62394 | ZN185_MOUSE | 352 | 38322 | 5.1 | (Q62394) Zinc finger protein 185 (LIM-domain protein Zfp185) (P1-A) | 3.3908E-05 | 8.331E-06 | 25% | B, C |
| Q9CW03 | SMC3_MOUSE | 1217 | 141555 | 7.2 | (Q9CW03) Structural maintenance of chromosome 3 (Chondroitin sulfate proteoglycan 6) (Chromosome segregation protein SmcD) (Bamacan) (Basement membrane-associated chondroitin proteoglycan) (Mad member-interacting protein 1) | 3.2623E-05 | 2.1716E-05 | 67% | B, C |
| Q99P65 | S29A3_MOUSE | 475 | 51720 | 7.6 | (Q99P65) Equilibrative nucleoside transporter 3 (Solute carrier family 29 member 3) | 3.2501E-05 | 1.6601E-05 | 51% | B, C |
| P51163 | HEM4_MOUSE | 265 | 28504 | 6.5 | (P51163) Uroporphyrinogen-III synthase (EC 4.2.1.75) (UROS) (Uroporphyrinogen-III cosynthetase) (Hydroxymethylbilane hydrolyase [cyclizing]) (UROIIIS) | 3.1824E-05 | 7.6243E-06 | 24% | B, C |
| Q9DBC7 | KAP0_MOUSE | 380 | 43054 | 5.3 | (Q9DBC7) cAMP-dependent protein kinase type I-alpha regulatory subunit | 3.1409E-05 | 7.7172E-06 | 25% | B, C |
| Q60855 | RIPK1_MOUSE | 656 | 74854 | 6.5 | (Q60855) Receptor-interacting serine/threonine-protein kinase 1 (EC 2.7.11.1) (Serine/threonine-protein kinase RIP) (Cell death protein RIP) (Receptor-interacting protein) | 3.105E-05 | 1.3904E-06 | 4% | B, C |
| Q9QXS6 | DREB_MOUSE | 705 | 77156 | 4.5 | (Q9QXS6) Drebrin (Developmentally-regulated brain protein) | 3.0641E-05 | 1.5231E-05 | 50% | B, C |

| | | | | | | | | | |
|---|---|---|---|---|---|---|---|---|---|
| Q80U87 | UBP8_MOUSE | 1080 | 122610 | 8.5 | (Q80U87) Ubiquitin carboxyl-terminal hydrolase 8 (EC 3.1.2.15) (Ubiquitin thioesterase 8) (Ubiquitin-specific-processing protease 8) (Deubiquitinating enzyme 8) (mUBPy) | 2.973E-05 | 3.8158E-06 | 13% | B, C |
| Q07643 | CO9A2_MOUSE | 688 | 65321 | 9.4 | (Q07643) Collagen alpha-2(IX) chain precursor | 2.9416E-05 | 1.5936E-06 | 5% | B, C |
| Q6ZQ88 | LSD1_MOUSE | 853 | 92851 | 6.5 | (Q6ZQ88) Lysine-specific histone demethylase 1 (EC 1.-.-.-) (Amine oxidase flavin-containing domain protein 2) (Protein AOF2) (BRAF35-HDAC complex protein BHC110) | 2.631E-05 | 2.0858E-05 | 79% | B, C |
| Q91VU6 | WDR23_MOUSE | 549 | 61992 | 6.4 | (Q91VU6) WD repeat protein 23 | 2.4105E-05 | 1.6047E-05 | 67% | B, C |
| Q99JT1 | GATB_MOUSE | 557 | 62118 | 8.6 | (Q99JT1) Probable glutamyl-tRNA(Gln) amidotransferase subunit B, mitochondrial precursor (EC 6.3.5.-) (Glu-ADT subunit B) (Cytochrome oxidase assembly factor PET112 homolog) | 2.3759E-05 | 1.5816E-05 | 67% | B, C |
| Q91WJ8 | FUBP1_MOUSE | 651 | 68540 | 7.9 | (Q91WJ8) Far upstream element-binding protein 1 (FUSE-binding protein 1) (FBP) | 2.3714E-05 | 1.2113E-05 | 51% | B, C |
| Q58A65 | JIP4_MOUSE | 1321 | 146219 | 5.1 | (Q58A65) C-jun-amino-terminal kinase-interacting protein 4 (JNK interacting protein 4) (JIP-4) (JNK-associated leucine-zipper protein) (JLP) (Sperm-associated antigen 9) (Mitogen-activated protein kinase 8-interacting protein 4) (JNK/SAPK-asso | 2.2687E-05 | 9.5883E-06 | 42% | B, C |
| Q6PDG5 | SMRC2_MOUSE | 1213 | 132604 | 5.6 | (Q6PDG5) SWI/SNF-related matrix-associated actin-dependent regulator of chromatin subfamily C member 2 (SWI/SNF complex 170 kDa subunit) (BRG1-associated factor 170) | 2.182E-05 | 1.4525E-05 | 67% | B, C |
| Q91W92 | BORG5_MOUSE | 409 | 43096 | 7.2 | (Q91W92) Cdc42 effector protein 1 (Binder of Rho GTPases 5) | 2.0619E-05 | 4.94E-06 | 24% | B, C |
| P42225 | STAT1_MOUSE | 749 | 87197 | 5.6 | (P42225) Signal transducer and activator of transcription 1 | 2.0611E-05 | 1.0528E-05 | 51% | B, C |
| P59997 | JHD1A_MOUSE | 1161 | 132679 | 7.5 | (P59997) JmjC domain-containing histone demethylation protein 1A (EC 1.14.11.-) (F-box/LRR-repeat protein 11) (F-box and leucine-rich repeat protein 11) | 1.9781E-05 | 1.9442E-05 | 98% | B, C |
| O70318 | E41L2_MOUSE | 988 | 109833 | 5.5 | (O70318) Band 4.1-like protein 2 (Generally expressed protein 4.1) (4.1G) | 1.917E-05 | 1.2994E-05 | 68% | B, C |
| Q61543 | GSLG1_MOUSE | 1175 | 133734 | 6.8 | (Q61543) Golgi apparatus protein 1 precursor (Golgi sialoglycoprotein MG-160) (E-selectin ligand 1) (ESL-1) (Selel) | 1.91E-05 | 1.5142E-05 | 79% | B, C |
| O70481 | UBR1_MOUSE | 1757 | 200215 | 6 | (O70481) Ubiquitin-protein ligase E3 component N-recognin-1 (EC 6.-.-.-) (Ubiquitin-protein ligase E3-alpha-1) (Ubiquitin-protein ligase E3-alpha-I) | 1.9051E-05 | 4.39E-06 | 23% | B, C |
| Q8C7E9 | CSTFT_MOUSE | 632 | 65862 | 7.3 | (Q8C7E9) Cleavage stimulation factor 64 kDa subunit, tau variant (CSTF 64 kDa subunit, tau variant) (CF-1 64 kDa subunit, tau variant) (TauCstF-64) | 1.8885E-05 | 4.6401E-06 | 25% | B, C |
| Q00519 | XDH_MOUSE | 1334 | 146387 | 7.6 | (Q00519) Xanthine dehydrogenase/oxidase [Includes: Xanthine dehydrogenase (EC 1.17.1.4) (XD); Xanthine oxidase (EC 1.17.3.2) (XO) (Xanthine oxidoreductase)] | 1.8819E-05 | 4.3365E-06 | 23% | B, C |
| Q9JL26 | FMNL_MOUSE | 1094 | 122059 | 5.8 | (Q9JL26) Formin-like 1 protein (Formin-related protein) | 1.8619E-05 | 8.3371E-07 | 4% | B, C |
| Q9Z1G4 | VPP1_MOUSE | 839 | 96501 | 6.8 | (Q9Z1G4) Vacuolar proton translocating ATPase 116 kDa subunit a isoform 1 (V-ATPase 116 kDa isoform a1) (Clathrin-coated vesicle/synaptic vesicle proton pump 116 kDa subunit) (Vacuolar proton pump subunit 1) (Vacuolar adenosine triphosphatase | 1.5773E-05 | 1.05E-05 | 67% | B, C |

| | | | | | | | | | |
|---|---|---|---|---|---|---|---|---|---|
| Q9JKY5 | HIP1R_MOUSE | 1068 | 119485 | 6.6 | (Q9JKY5) Huntingtin-interacting protein 1-related protein (Hip1-related) | 1.5671E-05 | 3.611E-06 | 23% | B, C |
| O54754 | ADO_MOUSE | 1333 | 146677 | 7 | (O54754) Aldehyde oxidase (EC 1.2.3.1) (Retinal oxidase) | 1.5183E-05 | 8.225E-07 | 5% | B, C |
| Q8R0I0 | ACE2_MOUSE | 805 | 92368 | 5.5 | (Q8R0I0) Angiotensin-converting enzyme 2 precursor (EC 3.4.17.-) (ACE-related carboxypeptidase) | 1.4827E-05 | 3.6429E-06 | 25% | B, C |
| Q9WTK5 | NFKB2_MOUSE | 899 | 96832 | 6.4 | (Q9WTK5) Nuclear factor NF-kappa-B p100 subunit (DNA-binding factor KBF2) [Contains: Nuclear factor NF-kappa-B p52 subunit] | 1.4721E-05 | 9.7993E-06 | 67% | B, C |
| Q9D2G5 | SYNJ2_MOUSE | 1434 | 158479 | 7.5 | (Q9D2G5) Synaptojanin-2 (EC 3.1.3.36) (Synaptic inositol-1,4,5-trisphosphate 5-phosphatase 2) | 1.4204E-05 | 6.3604E-07 | 4% | B, C |
| Q80TH2 | LAP2_MOUSE | 1402 | 157232 | 5.7 | (Q80TH2) Protein LAP2 (Erbb2-interacting protein) (Erbin) (Densin-180-like protein) | 1.3049E-05 | 1.1389E-05 | 87% | B, C |
| Q9EPE9 | AT131_MOUSE | 1200 | 132378 | 8 | (Q9EPE9) Probable cation-transporting ATPase 13A1 (EC 3.6.3.-) (CATP) | 1.1137E-05 | 7.4949E-06 | 67% | B, C |
| Q9Z2V5 | HDAC6_MOUSE | 1149 | 125704 | 5.8 | (Q9Z2V5) Histone deacetylase 6 (HD6) (Histone deacetylase mHDA2) | 1.0388E-05 | 2.5522E-06 | 25% | B, C |
| Q8CJ40 | CROCC_MOUSE | 2009 | 226912 | 5.6 | (Q8CJ40) Rootletin (Ciliary rootlet coiled-coil protein) | 1.0074E-05 | 5.4574E-07 | 5% | B, C |
| Q8K212 | PACS1_MOUSE | 961 | 104829 | 7.7 | (Q8K212) Phosphofurin acidic cluster sorting protein 1 (PACS-1) | 8.7755E-06 | 2.1024E-06 | 24% | B, C |
| Q9JL70 | FANCA_MOUSE | 1439 | 161161 | 7.1 | (Q9JL70) Fanconi anemia group A protein homolog (Protein FACA) | 8.2943E-06 | 2.0379E-06 | 25% | B, C |
| P34152 | FAK1_MOUSE | 1090 | 123511 | 6.7 | (P34152) Focal adhesion kinase 1 (EC 2.7.10.2) (FADK 1) (pp125FAK) | 7.7369E-06 | 1.8536E-06 | 24% | B, C |
| O70404 | VAMP8_MOUSE | 101 | 11451 | 8.2 | (O70404) Vesicle-associated membrane protein 8 (VAMP-8) (Endobrevin) (Edb) | 0.00077048 | n/a | n/a | A |
| Q9CQZ6 | NDUB3_MOUSE | 104 | 11692 | 9 | (Q9CQZ6) NADH dehydrogenase [ubiquinone] 1 beta subcomplex subunit 3 (EC 1.6.5.3) (EC 1.6.99.3) (NADH-ubiquinone oxidoreductase B12 subunit) (Complex I-B12) (CI-B12) | 0.00058197 | n/a | n/a | A |
| Q9CQZ1 | HSBP1_MOUSE | 76 | 8611 | 4.3 | (Q9CQZ1) Heat shock factor-binding protein 1 | 0.00056885 | n/a | n/a | A |
| O55013 | TPPC3_MOUSE | 180 | 20302 | 5 | (O55013) Trafficking protein particle complex subunit 3 (BET3 homolog) | 0.00038429 | n/a | n/a | A |
| Q9CQF4 | CF203_MOUSE | 240 | 27847 | 9.4 | (Q9CQF4) Protein C6orf203 homolog | 0.00036027 | n/a | n/a | A |
| Q61335 | BAP31_MOUSE | 244 | 27791 | 8.7 | (Q61335) B-cell receptor-associated protein 31 (BCR-associated protein Bap31) (p28 Bap31) | 0.00035436 | n/a | n/a | A |
| P60766 | CDC42_MOUSE | 191 | 21311 | 6 | (P60766) Cell division control protein 42 homolog precursor (G25K GTP-binding protein) | 0.0003211 | n/a | n/a | A |
| Q9CQ45 | NENF_MOUSE | 171 | 18904 | 5.3 | (Q9CQ45) Neudesin precursor (Neuron-derived neurotrophic factor) (Secreted protein of unknown function) (SPUF protein) | 0.00030338 | n/a | n/a | A |
| P14602 | HSPB1_MOUSE | 209 | 23014 | 6.5 | (P14602) Heat-shock protein beta-1 (HspB1) (Heat shock 27 kDa protein) (HSP 27) (Growth-related 25 kDa protein) (P25) (HSP25) | 0.00028959 | n/a | n/a | A |
| P56394 | COX17_MOUSE | 62 | 6653 | 7.7 | (P56394) Cytochrome c oxidase copper chaperone | 0.00028262 | n/a | n/a | A |
| Q99JR6 | NMNA3_MOUSE | 245 | 27703 | 8.5 | (Q99JR6) Nicotinamide mononucleotide adenylyltransferase 3 (EC 2.7.7.1) (NMN adenylyltransferase 3) | 0.00028233 | n/a | n/a | A |
| Q9DAK9 | PHP14_MOUSE | 124 | 13997 | 5.5 | (Q9DAK9) 14 kDa phosphohistidine phosphatase (EC 3.1.3.-) (Phosphohistidine phosphatase 1) | 0.00027892 | n/a | n/a | A |
| O70492 | SNX3_MOUSE | 161 | 18626 | 8.7 | (O70492) Sorting nexin-3 (SDP3 protein) | 0.00026852 | n/a | n/a | A |
| P55264 | ADK_MOUSE | 361 | 40149 | 6.2 | (P55264) Adenosine kinase (EC 2.7.1.20) (AK) (Adenosine 5'-phosphotransferase) | 0.00026347 | n/a | n/a | A |

| | | | | | | | | | |
|---|---|---|---|---|---|---|---|---|---|
| P01843 | LAC1_MOUSE | 105 | 11575 | 6.3 | (P01843) Ig lambda-1 chain C region | 0.00024704 | n/a | n/a | A |
| P61255 | RL26_MOUSE | 145 | 17258 | 10.6 | (P61255) 60S ribosomal protein L26 (Silica-induced gene 20 protein) (SIG-20) | 0.00023852 | n/a | n/a | A |
| P25444 | RS2_MOUSE | 293 | 31231 | 10.2 | (P25444) 40S ribosomal protein S2 (S4) (LLRep3 protein) | 0.00023608 | n/a | n/a | A |
| P07309 | TTHY_MOUSE | 147 | 15776 | 6.2 | (P07309) Transthyretin precursor (Prealbumin) | 0.00023528 | n/a | n/a | A |
| P17047 | LAMP2_MOUSE | 415 | 45647 | 7.4 | (P17047) Lysosome-associated membrane glycoprotein 2 precursor (LAMP-2) (Lysosomal membrane glycoprotein type B) (LGP-B) (CD107b antigen) | 0.00022918 | n/a | n/a | A |
| P97461 | RS5_MOUSE | 203 | 22758 | 9.7 | (P97461) 40S ribosomal protein S5 | 0.00021297 | n/a | n/a | A |
| P55041 | GEM_MOUSE | 295 | 33725 | 8.4 | (P55041) GTP-binding protein GEM (GTP-binding mitogen-induced T-cell protein) (RAS-like protein KIR) | 0.00020517 | n/a | n/a | A |
| Q91XF0 | PNPO_MOUSE | 261 | 30114 | 8.2 | (Q91XF0) Pyridoxine-5'-phosphate oxidase (EC 1.4.3.5) (Pyridoxamine-phosphate oxidase) | 0.00019877 | n/a | n/a | A |
| Q9R0U0 | FUSIP_MOUSE | 262 | 31301 | 11.3 | (Q9R0U0) FUS-interacting serine-arginine-rich protein 1 (TLS-associated protein with Ser-Arg repeats) (TLS-associated protein with SR repeats) (TASR) (TLS-associated serine-arginine protein) (TLS-associated SR protein) (Neural-specific SR pro | 0.00019801 | n/a | n/a | A |
| P63323 | RS12_MOUSE | 131 | 14394 | 7.2 | (P63323) 40S ribosomal protein S12 | 0.00019801 | n/a | n/a | A |
| Q9R1P4 | PSA1_MOUSE | 263 | 29547 | 6.4 | (Q9R1P4) Proteasome subunit alpha type 1 (EC 3.4.25.1) (Proteasome component C2) (Macropain subunit C2) (Multicatalytic endopeptidase complex subunit C2) (Proteasome nu chain) | 0.00019726 | n/a | n/a | A |
| P62073 | TIM10_MOUSE | 90 | 10333 | 6.3 | (P62073) Mitochondrial import inner membrane translocase subunit Tim10 | 0.0001947 | n/a | n/a | A |
| Q9CR23 | TMEM9_MOUSE | 183 | 20633 | 7 | (Q9CR23) Transmembrane protein 9 precursor | 0.00018899 | n/a | n/a | A |
| P21460 | CYTC_MOUSE | 140 | 15531 | 9 | (P21460) Cystatin C precursor (Cystatin 3) | 0.00018528 | n/a | n/a | A |
| Q9D517 | PLCC_MOUSE | 376 | 43296 | 8.5 | (Q9D517) 1-acyl-sn-glycerol-3-phosphate acyltransferase gamma (EC 2.3.1.51) (1-AGP acyltransferase 3) (1-AGPAT 3) (Lysophosphatidic acid acyltransferase-gamma) (LPAAT-gamma) (1-acylglycerol-3-phosphate O-acyltransferase 3) | 0.00018397 | n/a | n/a | A |
| P12815 | PDCD6_MOUSE | 191 | 21867 | 5.4 | (P12815) Programmed cell death protein 6 (Probable calcium-binding protein ALG-2) (PMP41) (ALG-257) | 0.00018108 | n/a | n/a | A |
| P34022 | RANG_MOUSE | 203 | 23596 | 5.2 | (P34022) Ran-specific GTPase-activating protein (Ran-binding protein 1) (RANBP1) (HpaII tiny fragments locus 9a protein) | 0.00017037 | n/a | n/a | A |
| P61458 | PHS_MOUSE | 103 | 11854 | 6.8 | (P61458) Pterin-4-alpha-carbinolamine dehydratase (EC 4.2.1.96) (PHS) (4-alpha-hydroxy-tetrahydropterin dehydratase) (Phenylalanine hydroxylase-stimulating protein) (Pterin carbinolamine dehydratase) (PCD) (Dimerization cofactor of hepatocyte n | 0.00016789 | n/a | n/a | A |
| Q8BVI5 | STX16_MOUSE | 326 | 37052 | 5.9 | (Q8BVI5) Syntaxin-16 | 0.00015914 | n/a | n/a | A |
| Q9ERK9 | P2RY6_MOUSE | 328 | 36721 | 9.8 | (Q9ERK9) P2Y purinoceptor 6 (P2Y6) | 0.00015817 | n/a | n/a | A |
| Q9ER00 | STX12_MOUSE | 274 | 31195 | 5.4 | (Q9ER00) Syntaxin-12 | 0.00015778 | n/a | n/a | A |
| Q9CQS2 | NOLA3_MOUSE | 64 | 7706 | 10 | (Q9CQS2) H/ACA ribonucleoprotein complex subunit 3 (Nucleolar protein family A member 3) (snoRNP protein NOP10) | 0.0001369 | n/a | n/a | A |
| Q60829 | IPPD_MOUSE | 194 | 21781 | 4.7 | (Q60829) Dopamine- and cAMP-regulated neuronal phosphoprotein (DARPP-32) | 0.00013548 | n/a | n/a | A |
| P25233 | NECD_MOUSE | 325 | 36832 | 8.5 | (P25233) Necdin | 0.00013302 | n/a | n/a | A |

| | | | | | | | | | |
|---|---|---|---|---|---|---|---|---|---|
| Q9CSU0 | CT077_MOUSE | 326 | 36884 | 6 | (Q9CSU0) Uncharacterized protein C20orf77 homolog | 0.00013261 | n/a | n/a | A |
| P23953 | ESTN_MOUSE | 554 | 61140 | 5.2 | (P23953) Liver carboxylesterase N precursor (EC 3.1.1.1) (PES-N) (Lung surfactant convertase) | 0.00012652 | n/a | n/a | A |
| Q9QZ06 | TOLIP_MOUSE | 274 | 30345 | 5.2 | (Q9QZ06) Toll-interacting protein | 0.00012623 | n/a | n/a | A |
| Q99KF1 | TMED9_MOUSE | 214 | 25010 | 7.2 | (Q99KF1) Transmembrane emp24 domain-containing protein 9 precursor (Glycoprotein 25L2) | 0.00012282 | n/a | n/a | A |
| P50172 | DHI1_MOUSE | 291 | 32233 | 8.5 | (P50172) Corticosteroid 11-beta-dehydrogenase isozyme 1 (EC 1.1.1.146) (11-DH) (11-beta-hydroxysteroid dehydrogenase 1) (11-beta-HSD1) (11beta-HSD1A) | 0.00011885 | n/a | n/a | A |
| Q9D8W5 | PSD12_MOUSE | 455 | 52746 | 7.1 | (Q9D8W5) 26S proteasome non-ATPase regulatory subunit 12 (26S proteasome regulatory subunit p55) | 0.00011402 | n/a | n/a | A |
| Q08619 | IFI5_MOUSE | 425 | 47046 | 8.1 | (Q08619) Interferon-activable protein 205 (IFI-205) (D3 protein) | 0.00010172 | n/a | n/a | A |
| Q9ERT9 | IPP1_MOUSE | 171 | 18718 | 5.3 | (Q9ERT9) Protein phosphatase inhibitor 1 (IPP-1) (I-1) | 0.00010113 | n/a | n/a | A |
| Q9D1B9 | RM28_MOUSE | 257 | 30170 | 9.3 | (Q9D1B9) 39S ribosomal protein L28, mitochondrial precursor (L28mt) (MRP-L28) | 0.00010093 | n/a | n/a | A |
| O35326 | SFRS5_MOUSE | 270 | 30945 | 11.4 | (O35326) Splicing factor, arginine/serine-rich 5 (Pre-mRNA-splicing factor SRP40) (Delayed-early protein HRS) | 9.7348E-05 | n/a | n/a | A |
| Q8K330 | SSH3_MOUSE | 649 | 72227 | 5.6 | (Q8K330) Protein phosphatase Slingshot homolog 3 (EC 3.1.3.48) (EC 3.1.3.16) (SSH-3L) (mSSH-3L) | 9.3259E-05 | n/a | n/a | A |
| Q61411 | RASH_MOUSE | 189 | 21348 | 5.3 | (Q61411) GTPase HRas precursor (Transforming protein p21) (p21ras) (H-Ras-1) (c-H-ras) | 9.2712E-05 | n/a | n/a | A |
| P58774 | TPM2_MOUSE | 284 | 32837 | 4.7 | (P58774) Tropomyosin beta chain (Tropomyosin 2) (Beta-tropomyosin) | 9.1336E-05 | n/a | n/a | A |
| P63001 | RAC1_MOUSE | 192 | 21450 | 8.5 | (P63001) Ras-related C3 botulinum toxin substrate 1 precursor (p21-Rac1) | 9.1264E-05 | n/a | n/a | A |
| Q9CQ85 | TIM22_MOUSE | 194 | 20114 | 8.2 | (Q9CQ85) Mitochondrial import inner membrane translocase subunit Tim22 | 9.0323E-05 | n/a | n/a | A |
| Q3T9Z9 | CF113_MOUSE | 577 | 65593 | 6.9 | (Q3T9Z9) Zinc finger protein C6orf113 homolog | 8.9911E-05 | n/a | n/a | A |
| Q63829 | COMD3_MOUSE | 195 | 22037 | 5.6 | (Q63829) COMM domain-containing protein 3 (Bmi-1 upstream gene protein) (Bup protein) | 8.986E-05 | n/a | n/a | A |
| Q64704 | STX3_MOUSE | 289 | 33243 | 5.4 | (Q64704) Syntaxin-3 | 8.9756E-05 | n/a | n/a | A |
| Q64520 | KGUA_MOUSE | 197 | 21787 | 6.5 | (Q64520) Guanylate kinase (EC 2.7.4.8) (GMP kinase) | 8.8947E-05 | n/a | n/a | A |
| P61087 | UBC1_MOUSE | 199 | 22275 | 5.4 | (P61087) Ubiquitin-conjugating enzyme E2-25 kDa (EC 6.3.2.19) (Ubiquitin-protein ligase) (Ubiquitin carrier protein) (E2(25K)) (Huntingtin-interacting protein 2) (HIP-2) | 8.8053E-05 | n/a | n/a | A |
| Q9D0J8 | PTMS_MOUSE | 100 | 11299 | 4.2 | (Q9D0J8) Parathymosin | 8.7613E-05 | n/a | n/a | A |
| Q923D2 | BLVRB_MOUSE | 205 | 22066 | 7 | (Q923D2) Flavin reductase (EC 1.5.1.30) (FR) (NADPH-dependent diaphorase) (NADPH-flavin reductase) (FLR) (Biliverdin reductase B) (EC 1.3.1.24) (BVR-B) (Biliverdin-IX beta-reductase) | 8.5476E-05 | n/a | n/a | A |
| P35279 | RAB6A_MOUSE | 207 | 23459 | 5.5 | (P35279) Ras-related protein Rab-6A (Rab-6) | 8.465E-05 | n/a | n/a | A |
| Q9CYG7 | OM34_MOUSE | 309 | 34278 | 9.1 | (Q9CYG7) Mitochondrial import receptor subunit TOM34 (Translocase of outer membrane 34 kDa subunit) | 8.3946E-05 | n/a | n/a | A |
| P43275 | H11_MOUSE | 212 | 21654 | 10.9 | (P43275) Histone H1.1 (H1 VAR.3) (H1a) | 8.2654E-05 | n/a | n/a | A |
| Q6ZWX6 | IF2A_MOUSE | 314 | 35977 | 5.1 | (Q6ZWX6) Eukaryotic translation initiation factor 2 subunit 1 (Eukaryotic translation initiation factor 2 subunit alpha) (eIF-2-alpha) (EIF-2alpha) (EIF-2A) | 8.261E-05 | n/a | n/a | A |
| O88968 | TCO2_MOUSE | 430 | 47586 | 6.3 | (O88968) Transcobalamin-2 precursor (Transcobalamin II) (TCII) (TC II) | 8.1501E-05 | n/a | n/a | A |

| | | | | | | | | | |
|---|---|---|---|---|---|---|---|---|---|
| P20060 | HEXB_MOUSE | 536 | 61116 | 8.1 | (P20060) Beta-hexosaminidase beta chain precursor (EC 3.2.1.52) (N-acetyl-beta-glucosaminidase) (Beta-N-acetylhexosaminidase) (Hexosaminidase B) | 8.0657E-05 | n/a | n/a | A |
| Q9CXI0 | COQ5_MOUSE | 327 | 37336 | 7.5 | (Q9CXI0) Ubiquinone biosynthesis methyltransferase COQ5, mitochondrial precursor (EC 2.1.1.-) | 8.0379E-05 | n/a | n/a | A |
| Q9WU40 | MAN1_MOUSE | 331 | 34594 | 5.9 | (Q9WU40) Inner nuclear membrane protein Man1 (LEM domain-containing protein 3) (Fragment) | 7.9408E-05 | n/a | n/a | A |
| Q9CZT8 | RAB3B_MOUSE | 219 | 24757 | 5.1 | (Q9CZT8) Ras-related protein Rab-3B | 7.8963E-05 | n/a | n/a | A |
| Q9CR59 | G45IP_MOUSE | 222 | 25820 | 10.3 | (Q9CR59) Growth arrest and DNA-damage-inducible proteins-interacting protein 1 | 7.8931E-05 | n/a | n/a | A |
| P62137 | PP1A_MOUSE | 330 | 37540 | 6.3 | (P62137) Serine/threonine-protein phosphatase PP1-alpha catalytic subunit (EC 3.1.3.16) (PP-1A) | 7.8604E-05 | n/a | n/a | A |
| Q8CCI5 | RYBP_MOUSE | 228 | 24776 | 9.6 | (Q8CCI5) RING1 and YY1-binding protein (Death effector domain-associated factor) (DED-associated factor) | 7.6854E-05 | n/a | n/a | A |
| Q9QYS9 | QKI_MOUSE | 341 | 37671 | 8.5 | (Q9QYS9) Quaking protein (qkI) | 7.6069E-05 | n/a | n/a | A |
| Q8R3G1 | PP1R8_MOUSE | 351 | 38528 | 7.4 | (Q8R3G1) Nuclear inhibitor of protein phosphatase 1 (NIPP-1) (Protein phosphatase 1 regulatory inhibitor subunit 8) | 7.4883E-05 | n/a | n/a | A |
| P70445 | 4EBP2_MOUSE | 120 | 12898 | 6.5 | (P70445) Eukaryotic translation initiation factor 4E-binding protein 2 (4E-BP2) (eIF4E-binding protein 2) (Phosphorylated heat- and acid-stable protein regulated by insulin 2) (PHAS-II) | 7.3011E-05 | n/a | n/a | A |
| P60840 | ENSA_MOUSE | 121 | 13335 | 7.2 | (P60840) Alpha-endosulfine (ARPP-19e) | 7.2408E-05 | n/a | n/a | A |
| Q8BH50 | CR025_MOUSE | 245 | 26446 | 5.2 | (Q8BH50) Uncharacterized protein C18orf25 homolog | 7.1521E-05 | n/a | n/a | A |
| Q9D1E6 | TBCB_MOUSE | 244 | 27386 | 5.2 | (Q9D1E6) Tubulin-specific chaperone B (Tubulin folding cofactor B) (Cytoskeleton-associated protein 1) (Cytoskeleton-associated protein CKAPI) | 7.0873E-05 | n/a | n/a | A |
| P26149 | 3BHS2_MOUSE | 372 | 41864 | 6.5 | (P26149) 3 beta-hydroxysteroid dehydrogenase/delta 5-- 4-isomerase type II (3Beta-HSD II) [Includes: 3-beta-hydroxy-delta(5)-steroid dehydrogenase (EC 1.1.1.145) (3-beta-hydroxy-5-ene steroid dehydrogenase) (Progesterone reductase); Steroid d | 7.0656E-05 | n/a | n/a | A |
| Q9CY57 | CA077_MOUSE | 249 | 26585 | 12.2 | (Q9CY57) Protein C1orf77 homolog | 7.0372E-05 | n/a | n/a | A |
| Q9DCG9 | U315_MOUSE | 125 | 14141 | 5.3 | (Q9DCG9) UPF0315 protein | 7.0091E-05 | n/a | n/a | A |
| Q9CQV1 | TIM16_MOUSE | 125 | 13785 | 9.6 | (Q9CQV1) Mitochondrial import inner membrane translocase subunit TIM16 (Mitochondria-associated granulocyte macrophage CSF signaling molecule) | 7.0091E-05 | n/a | n/a | A |
| Q61578 | ADRO_MOUSE | 494 | 54202 | 8.7 | (Q61578) NADPH:adrenodoxin oxidoreductase, mitochondrial precursor (EC 1.18.1.2) (Adrenodoxin reductase) (AR) (Ferredoxin reductase) (Ferredoxin--NADP(+) reductase) | 7.0012E-05 | n/a | n/a | A |
| Q9D4H8 | CUL2_MOUSE | 745 | 86877 | 7 | (Q9D4H8) Cullin-2 (CUL-2) | 6.9636E-05 | n/a | n/a | A |
| Q60710 | SAMH1_MOUSE | 627 | 72650 | 8 | (Q60710) SAM domain and HD domain-containing protein 1 (Interferon-gamma-inducible protein Mg11) | 6.8951E-05 | n/a | n/a | A |
| Q8K2Y7 | RM47_MOUSE | 252 | 29726 | 10.2 | (Q8K2Y7) 39S ribosomal protein L47, mitochondrial precursor (L47mt) (MRP-L47) | 6.8623E-05 | n/a | n/a | A |
| Q5XJY5 | COPD_MOUSE | 511 | 57217 | 6.2 | (Q5XJY5) Coatomer subunit delta (Delta-coat protein) (Delta-COP) (Archain) | 6.7683E-05 | n/a | n/a | A |
| Q791T5 | MTCH1_MOUSE | 389 | 41565 | 9.3 | (Q791T5) Mitochondrial carrier homolog 1 (Mitochondrial carrier-like protein 1) | 6.6682E-05 | n/a | n/a | A |

| | | | | | | | | | |
|---|---|---|---|---|---|---|---|---|---|
| Q8R0X7 | SGPL1_MOUSE | 568 | 63677 | 9.1 | (Q8R0X7) Sphingosine-1-phosphate lyase 1 (EC 4.1.2.27) (SP-lyase) (mSPL) (Sphingosine-1-phosphate aldolase) | 6.1699E-05 | n/a | n/a | A |
| Q8K2K6 | NUPL_MOUSE | 561 | 58043 | 8.6 | (Q8K2K6) Nucleoporin-like protein RIP (HIV-1 Rev-binding protein homolog) | 6.165E-05 | n/a | n/a | A |
| P53657 | KPYR_MOUSE | 574 | 62309 | 7.1 | (P53657) Pyruvate kinase isozymes R/L (EC 2.7.1.40) (L-PK) | 6.0254E-05 | n/a | n/a | A |
| Q9D338 | RM19_MOUSE | 292 | 33578 | 9.4 | (Q9D338) 39S ribosomal protein L19, mitochondrial precursor (L19mt) (MRP-L19) | 5.9222E-05 | n/a | n/a | A |
| Q9JLC6 | TEF_MOUSE | 301 | 33145 | 5.9 | (Q9JLC6) Thyrotroph embryonic factor | 5.7452E-05 | n/a | n/a | A |
| Q8BJU0 | SGTA_MOUSE | 315 | 34322 | 5.1 | (Q8BJU0) Small glutamine-rich tetratricopeptide repeat-containing protein A | 5.5627E-05 | n/a | n/a | A |
| Q80W54 | FACE1_MOUSE | 475 | 54735 | 6.9 | (Q80W54) CAAX prenyl protease 1 homolog (EC 3.4.24.84) (Prenyl protein-specific endoprotease 1) (Farnesylated proteins-converting enzyme 1) (FACE-1) (Zinc metalloproteinase Ste24 homolog) | 5.4609E-05 | n/a | n/a | A |
| Q60779 | GAS8_MOUSE | 478 | 56264 | 7.9 | (Q60779) Growth-arrest-specific protein 8 (Growth arrest-specific 11) | 5.4267E-05 | n/a | n/a | A |
| P97379 | G3B2_MOUSE | 482 | 54088 | 5.6 | (P97379) Ras-GTPase-activating protein-binding protein 2 (GAP SH3-domain-binding protein 2) (G3BP-2) | 5.3816E-05 | n/a | n/a | A |
| O70400 | PDLI1_MOUSE | 326 | 35643 | 6.8 | (O70400) PDZ and LIM domain protein 1 (Elfin) (LIM domain protein CLP-36) (C-terminal LIM domain protein 1) | 5.3046E-05 | n/a | n/a | A |
| Q9CPT4 | CS010_MOUSE | 166 | 17982 | 6.8 | (Q9CPT4) Uncharacterized protein C19orf10 homolog precursor (Stromal cell-derived growth factor SF20) (Interleukin-25) (IL-25) | 5.2779E-05 | n/a | n/a | A |
| Q8BFQ9 | KLDC5_MOUSE | 493 | 55599 | 6.3 | (Q8BFQ9) Kelch domain-containing protein 5 | 5.2615E-05 | n/a | n/a | A |
| Q8C0L0 | TXD13_MOUSE | 335 | 37131 | 4.4 | (Q8C0L0) Thioredoxin domain-containing protein 13 precursor | 5.2306E-05 | n/a | n/a | A |
| Q9DAR7 | DCPS_MOUSE | 338 | 38988 | 6.5 | (Q9DAR7) Scavenger mRNA decapping enzyme DcpS (EC 3.-.-.-) (DCS-1) (Hint-related 7meGMP-directed hydrolase) (Histidine triad protein member 5) (HINT-5) | 5.1163E-05 | n/a | n/a | A |
| Q9R0C0 | PLDN_MOUSE | 172 | 19682 | 6.3 | (Q9R0C0) Pallidin (Pallid protein) (Syntaxin 13-interacting protein) | 5.0938E-05 | n/a | n/a | A |
| Q9D787 | PPIL2_MOUSE | 521 | 59065 | 8.4 | (Q9D787) Peptidyl-prolyl cis-trans isomerase-like 2 (EC 5.2.1.8) (PPIase) (Rotamase) (Cyclophilin-60) (Cyclophilin-like protein Cyp-60) | 4.9788E-05 | n/a | n/a | A |
| Q9CZH7 | TMAP1_MOUSE | 178 | 19417 | 4.3 | (Q9CZH7) Transmembrane anchor protein 1 | 4.9221E-05 | n/a | n/a | A |
| Q9R0P6 | SPC18_MOUSE | 179 | 20626 | 9.3 | (Q9R0P6) Microsomal signal peptidase 18 kDa subunit (EC 3.4.-.-) (SPase 18 kDa subunit) (SPC18) (Endopeptidase SP18) (SEC11-like 1) (Sid 2895) | 4.8946E-05 | n/a | n/a | A |
| Q60867 | NDF1_MOUSE | 357 | 39999 | 5.3 | (Q60867) Neurogenic differentiation factor 1 (NeuroD1) | 4.844E-05 | n/a | n/a | A |
| Q9QZ88 | VPS29_MOUSE | 182 | 20496 | 6.8 | (Q9QZ88) Vacuolar protein sorting 29 (Vesicle protein sorting 29) | 4.8139E-05 | n/a | n/a | A |
| P01900 | HA12_MOUSE | 365 | 41110 | 6.7 | (P01900) H-2 class I histocompatibility antigen, D-D alpha chain precursor (H-2D(D)) | 4.8007E-05 | n/a | n/a | A |
| Q6P9J9 | TM16F_MOUSE | 911 | 106255 | 6.7 | (Q6P9J9) Transmembrane protein 16F | 4.7456E-05 | n/a | n/a | A |
| Q8BZZ3 | WWP1_MOUSE | 918 | 104694 | 6.4 | (Q8BZZ3) NEDD4-like E3 ubiquitin-protein ligase WWP1 (EC 6.3.2.-) (WW domain-containing protein 1) | 4.7094E-05 | n/a | n/a | A |
| Q9Z2Q6 | SEPT5_MOUSE | 369 | 42748 | 6.7 | (Q9Z2Q6) Septin-5 (Peanut-like protein 1) (Cell division control-related protein 1) (CDCrel-1) | 4.6864E-05 | n/a | n/a | A |

| | | | | | | | | | |
|---|---|---|---|---|---|---|---|---|---|
| Q9D8V0 | HM13_MOUSE | 378 | 41748 | 6 | (Q9D8V0) Minor histocompatibility antigen H13 (EC 3.4.99.-) (Signal peptide peptidase) (Presenilin-like protein 3) | 4.6356E-05 | n/a | n/a | A |
| Q01063 | PDE4D_MOUSE | 747 | 84563 | 4.9 | (Q01063) cAMP-specific 3',5'-cyclic phosphodiesterase 4D (EC 3.1.4.17) (DPDE3) | 4.63E-05 | n/a | n/a | A |
| P84096 | RHOG_MOUSE | 191 | 21308 | 8.1 | (P84096) Rho-related GTP-binding protein RhoG precursor (Sid 10750) | 4.5871E-05 | n/a | n/a | A |
| Q9DCE5 | PK1IP_MOUSE | 382 | 42116 | 8.5 | (Q9DCE5) p21-activated protein kinase-interacting protein 1 (PAK1-interacting protein 1) (Putative PAK inhibitor Skb15) | 4.5871E-05 | n/a | n/a | A |
| Q8R2V5 | CENA2_MOUSE | 381 | 43989 | 9.1 | (Q8R2V5) Centaurin-alpha 2 | 4.5388E-05 | n/a | n/a | A |
| Q9CXY6 | ILF2_MOUSE | 390 | 43062 | 5.3 | (Q9CXY6) Interleukin enhancer-binding factor 2 (Nuclear factor of activated T-cells 45 kDa) | 4.493E-05 | n/a | n/a | A |
| O88291 | ZN326_MOUSE | 580 | 65225 | 5.2 | (O88291) Zinc finger protein 326 (Zinc finger protein-associated with nuclear matrix of 75 kDa) | 4.4723E-05 | n/a | n/a | A |
| Q9R1Q8 | TAGL3_MOUSE | 199 | 22471 | 7.3 | (Q9R1Q8) Transgelin-3 (Neuronal protein NP25) | 4.4027E-05 | n/a | n/a | A |
| Q91YE3 | EGLN1_MOUSE | 400 | 43111 | 8.3 | (Q91YE3) Egl nine homolog 1 (EC 1.14.11.-) (Hypoxia-inducible factor prolyl hydroxylase 2) (HIF-prolyl hydroxylase 2) (HIF-PH2) (HPH-2) (SM-20) | 4.3807E-05 | n/a | n/a | A |
| Q8VHR5 | P66B_MOUSE | 594 | 65411 | 9.7 | (Q8VHR5) Transcriptional repressor p66 beta (p66/p68) (GATA zinc finger domain-containing protein 2B) | 4.3669E-05 | n/a | n/a | A |
| Q8K1I3 | SPP24_MOUSE | 203 | 23136 | 8.4 | (Q8K1I3) Secreted phosphoprotein 24 precursor (Spp-24) (Secreted phosphoprotein 2) | 4.3159E-05 | n/a | n/a | A |
| Q00897 | A1AT4_MOUSE | 413 | 45998 | 5.4 | (Q00897) Alpha-1-antitrypsin 1-4 precursor (Serine protease inhibitor 1-4) (Alpha-1 protease inhibitor 4) | 4.1872E-05 | n/a | n/a | A |
| Q91WM2 | CECR5_MOUSE | 419 | 46306 | 7.9 | (Q91WM2) Cat eye syndrome critical region protein 5 homolog precursor | 4.182E-05 | n/a | n/a | A |
| Q8C147 | DOCK8_MOUSE | 1037 | 118563 | 7.1 | (Q8C147) Dedicator of cytokinesis protein 8 | 4.169E-05 | n/a | n/a | A |
| Q91W90 | TXND5_MOUSE | 417 | 46416 | 5.8 | (Q91W90) Thioredoxin domain-containing protein 5 precursor (Thioredoxin-like protein p46) (Endoplasmic reticulum protein ERp46) (Plasma cell-specific thioredoxin-related protein) (PC-TRP) | 4.147E-05 | n/a | n/a | A |
| Q8BJF9 | CHM2B_MOUSE | 213 | 23935 | 8.8 | (Q8BJF9) Charged multivesicular body protein 2b (Chromatin-modifying protein 2b) (CHMP2b) | 4.1133E-05 | n/a | n/a | A |
| P56371 | RAB4A_MOUSE | 213 | 23938 | 6.1 | (P56371) Ras-related protein Rab-4A | 4.1133E-05 | n/a | n/a | A |
| Q9EPQ7 | STAR5_MOUSE | 213 | 23922 | 6.4 | (Q9EPQ7) StAR-related lipid transfer protein 5 (StARD5) (START domain-containing protein 5) | 4.1133E-05 | n/a | n/a | A |
| Q61510 | TRI25_MOUSE | 634 | 71772 | 8.3 | (Q61510) Tripartite motif-containing protein 25 (Zinc finger protein 147) (Estrogen-responsive finger protein) (Efp) | 4.0914E-05 | n/a | n/a | A |
| Q7TT50 | MRCKB_MOUSE | 1713 | 194779 | 6.5 | (Q7TT50) Serine/threonine-protein kinase MRCK beta (EC 2.7.11.1) (CDC42-binding protein kinase beta) (Myotonic dystrophy kinase-related CDC42-binding kinase beta) (Myotonic dystrophy protein kinase-like beta) (MRCK beta) (DMPK-like beta) | 4.038E-05 | n/a | n/a | A |
| Q9JKX6 | NUDT5_MOUSE | 218 | 23984 | 5.5 | (Q9JKX6) ADP-sugar pyrophosphatase (EC 3.6.1.13) (EC 3.6.1.-) (Nucleoside diphosphate-linked moiety X motif 5) (Nudix motif 5) | 4.019E-05 | n/a | n/a | A |
| Q80Y44 | DDX10_MOUSE | 875 | 100738 | 7.7 | (Q80Y44) Probable ATP-dependent RNA helicase DDX10 (EC 3.6.1.-) (DEAD box protein 10) | 3.9527E-05 | n/a | n/a | A |
| Q8BTZ5 | ANR46_MOUSE | 228 | 25223 | 5.7 | (Q8BTZ5) Ankyrin repeat domain-containing protein 46 (Ankyrin repeat small protein) (ANK-S) | 3.8427E-05 | n/a | n/a | A |

| | | | | | | | | | |
|---|---|---|---|---|---|---|---|---|---|
| Q3UEI1 | PDE4C_MOUSE | 686 | 76090 | 5 | (Q3UEI1) cAMP-specific 3',5'-cyclic phosphodiesterase 4C (EC 3.1.4.17) | 3.8315E-05 | n/a | n/a | A |
| P49452 | CENPC_MOUSE | 906 | 102226 | 9.7 | (P49452) Centromere protein C 1 (CENP-C) (Centromere autoantigen C) | 3.8174E-05 | n/a | n/a | A |
| Q8BNU0 | ARMC6_MOUSE | 468 | 50683 | 6 | (Q8BNU0) Armadillo repeat-containing protein 6 | 3.7442E-05 | n/a | n/a | A |
| Q9JI44 | DMAP1_MOUSE | 468 | 53130 | 9.5 | (Q9JI44) DNA methyltransferase 1-associated protein 1 (DNMT1 associated protein 1) (DNMAP1) (MAT1-mediated transcriptional repressor) | 3.7442E-05 | n/a | n/a | A |
| Q61189 | ICLN_MOUSE | 236 | 26021 | 4.1 | (Q61189) Methylosome subunit pICln (Chloride conductance regulatory protein ICln) (I(Cln)) (Chloride channel, nucleotide sensitive 1A) (Chloride ion current inducer protein) (ClCI) | 3.7124E-05 | n/a | n/a | A |
| P41183 | BCL6_MOUSE | 707 | 78982 | 8 | (P41183) B-cell lymphoma 6 protein homolog | 3.6689E-05 | n/a | n/a | A |
| O88533 | DDC_MOUSE | 480 | 53874 | 6.6 | (O88533) Aromatic-L-amino-acid decarboxylase (EC 4.1.1.28) (AADC) (DOPA decarboxylase) (DDC) | 3.6027E-05 | n/a | n/a | A |
| Q9Z1B3 | PLCB1_MOUSE | 1216 | 138325 | 5.9 | (Q9Z1B3) 1-phosphatidylinositol-4,5-bisphosphate phosphodiesterase beta 1 (EC 3.1.4.11) (Phosphoinositide phospholipase C) (Phospholipase C-beta-1) (PLC-beta-1) (PLC-I) (PLC-154) | 3.5553E-05 | n/a | n/a | A |
| Q9DBE0 | CSAD_MOUSE | 493 | 55145 | 6.6 | (Q9DBE0) Cysteine sulfinic acid decarboxylase (EC 4.1.1.29) (Sulfinoalanine decarboxylase) (Cysteine-sulfinate decarboxylase) | 3.5543E-05 | n/a | n/a | A |
| Q9Z108 | STAU1_MOUSE | 487 | 53925 | 9.5 | (Q9Z108) Double-stranded RNA-binding protein Staufen homolog 1 | 3.5509E-05 | n/a | n/a | A |
| Q99N84 | RT18B_MOUSE | 254 | 28703 | 8.4 | (Q99N84) 28S ribosomal protein S18b, mitochondrial precursor (MRP-S18-b) (Mrps18b) (MRP-S18-2) | 3.4493E-05 | n/a | n/a | A |
| P11714 | CP2D9_MOUSE | 504 | 56949 | 6.3 | (P11714) Cytochrome P450 2D9 (EC 1.14.14.1) (CYPIID9) (P450-16-alpha) (CA) (Testosterone 16-alpha hydroxylase) | 3.4311E-05 | n/a | n/a | A |
| P50586 | TUB_MOUSE | 505 | 55362 | 8.7 | (P50586) Tubby protein | 3.4243E-05 | n/a | n/a | A |
| P51791 | CLCN3_MOUSE | 760 | 84475 | 7.2 | (P51791) Chloride channel protein 3 (ClC-3) | 3.4131E-05 | n/a | n/a | A |
| P35831 | PTN12_MOUSE | 775 | 86992 | 6.2 | (P35831) Tyrosine-protein phosphatase non-receptor type 12 (EC 3.1.3.48) (Protein-tyrosine phosphatase P19) (P19-PTP) (MPTP-PEST) | 3.347E-05 | n/a | n/a | A |
| P99026 | PSB4_MOUSE | 264 | 29116 | 5.7 | (P99026) Proteasome subunit beta type 4 precursor (EC 3.4.25.1) (Proteasome beta chain) (Macropain beta chain) (Multicatalytic endopeptidase complex beta chain) (Proteasome chain 3) | 3.3187E-05 | n/a | n/a | A |
| P60954 | NOL4_MOUSE | 523 | 57963 | 5.2 | (P60954) Nucleolar protein 4 | 3.3065E-05 | n/a | n/a | A |
| Q99KC8 | LHR2A_MOUSE | 793 | 87143 | 6.6 | (Q99KC8) Loss of heterozygosity 11 chromosomal region 2 gene A protein homolog | 3.271E-05 | n/a | n/a | A |
| Q9R078 | AAKB1_MOUSE | 269 | 30177 | 6.2 | (Q9R078) 5'-AMP-activated protein kinase subunit beta-1 (AMPK beta-1 chain) (AMPKb) | 3.257E-05 | n/a | n/a | A |
| Q8K4G2 | CONA1_MOUSE | 532 | 51476 | 6.9 | (Q8K4G2) Collagen alpha-1(XXIII) chain | 3.2506E-05 | n/a | n/a | A |
| Q64669 | NQO1_MOUSE | 273 | 30828 | 8.7 | (Q64669) NAD(P)H dehydrogenase [quinone] 1 (EC 1.6.5.2) (Quinone reductase 1) (NAD(P)H:quinone oxidoreductase 1) (QR1) (DT-diaphorase) (DTD) (Azoreductase) (Phylloquinone reductase) (Menadione reductase) | 3.2093E-05 | n/a | n/a | A |
| P08551 | NFL_MOUSE | 542 | 61377 | 4.6 | (P08551) Neurofilament triplet L protein (68 kDa neurofilament protein) (Neurofilament light polypeptide) (NF-L) | 3.1906E-05 | n/a | n/a | A |

| | | | | | | | | | |
|---|---|---|---|---|---|---|---|---|---|
| Q5SSI6 | UTP18_MOUSE | 552 | 61218 | 8.8 | (Q5SSI6) U3 small nucleolar RNA-associated protein 18 homolog (WD repeat protein 50) | 3.1744E-05 | n/a | n/a | A |
| Q9DBY8 | NVL_MOUSE | 855 | 94476 | 6.4 | (Q9DBY8) Nuclear valosin-containing protein-like (Nuclear VCP-like protein) (NVLp) | 3.0741E-05 | n/a | n/a | A |
| Q8CGB3 | UACA_MOUSE | 1411 | 160812 | 7.2 | (Q8CGB3) Uveal autoantigen with coiled-coil domains and ankyrin repeats protein (Nucling) (Nuclear membrane-binding protein) | 3.0639E-05 | n/a | n/a | A |
| P39053 | DYN1_MOUSE | 867 | 97803 | 7.7 | (P39053) Dynamin-1 (EC 3.6.5.5) | 2.9919E-05 | n/a | n/a | A |
| O08759 | UBE3A_MOUSE | 885 | 101176 | 5.1 | (O08759) Ubiquitin-protein ligase E3A (EC 6.3.2.-) (Oncogenic protein-associated protein E6-AP) | 2.931E-05 | n/a | n/a | A |
| Q9DBX2 | PHLP_MOUSE | 301 | 34407 | 4.9 | (Q9DBX2) Phosducin-like protein (PHLP) | 2.9107E-05 | n/a | n/a | A |
| O08856 | ELL_MOUSE | 602 | 67120 | 9.2 | (O08856) RNA polymerase II elongation factor ELL (Eleven-nineteen lysine-rich leukemia protein) | 2.8726E-05 | n/a | n/a | A |
| P07607 | TYSY_MOUSE | 307 | 34958 | 6.5 | (P07607) Thymidylate synthase (EC 2.1.1.45) (TS) (TSase) | 2.8538E-05 | n/a | n/a | A |
| P63330 | PP2AA_MOUSE | 309 | 35608 | 5.5 | (P63330) Serine/threonine-protein phosphatase 2A catalytic subunit alpha isoform (EC 3.1.3.16) (PP2A-alpha) | 2.8354E-05 | n/a | n/a | A |
| Q7TSV4 | PGM2_MOUSE | 620 | 68748 | 6.1 | (Q7TSV4) Phosphoglucomutase-2 (EC 5.4.2.2) (Glucose phosphomutase 2) (PGM 2) | 2.7892E-05 | n/a | n/a | A |
| Q8BP92 | RCN2_MOUSE | 320 | 37271 | 4.4 | (Q8BP92) Reticulocalbin-2 precursor (Taipoxin-associated calcium-binding protein 49) (TCBP-49) | 2.7379E-05 | n/a | n/a | A |
| Q6P9R1 | DDX51_MOUSE | 639 | 70368 | 9.5 | (Q6P9R1) ATP-dependent RNA helicase DDX51 (EC 3.6.1.-) (DEAD box protein 51) | 2.7062E-05 | n/a | n/a | A |
| O70305 | ATX2_MOUSE | 1285 | 136485 | 9.6 | (O70305) Ataxin-2 (Spinocerebellar ataxia type 2 protein homolog) | 2.6915E-05 | n/a | n/a | A |
| Q9R0X5 | RPGR_MOUSE | 1001 | 111856 | 4.6 | (Q9R0X5) X-linked retinitis pigmentosa GTPase regulator (mRpgr) | 2.6258E-05 | n/a | n/a | A |
| Q8CEE7 | RDH13_MOUSE | 334 | 36464 | 8.9 | (Q8CEE7) Retinol dehydrogenase 13 (EC 1.1.1.-) | 2.6231E-05 | n/a | n/a | A |
| P70695 | F16P2_MOUSE | 339 | 36947 | 6.2 | (P70695) Fructose-1,6-bisphosphatase isozyme 2 (EC 3.1.3.11) (D-fructose-1,6-bisphosphate 1-phosphohydrolase 2) (FBPase 2) (RAE-30) | 2.5845E-05 | n/a | n/a | A |
| Q9D9K3 | AVEN_MOUSE | 342 | 37195 | 5 | (Q9D9K3) Cell death regulator Aven | 2.5618E-05 | n/a | n/a | A |
| Q9D6Y9 | GLGB_MOUSE | 702 | 80364 | 6.4 | (Q9D6Y9) 1,4-alpha-glucan branching enzyme (EC 2.4.1.18) (Glycogen branching enzyme) (Brancher enzyme) | 2.4961E-05 | n/a | n/a | A |
| P24452 | CAPG_MOUSE | 352 | 39240 | 7.2 | (P24452) Macrophage capping protein (Myc basic motif homolog 1) (Actin-capping protein GCAP39) | 2.489E-05 | n/a | n/a | A |
| O88623 | UBP2_MOUSE | 353 | 40581 | 8.5 | (O88623) Ubiquitin carboxyl-terminal hydrolase 2 (EC 3.1.2.15) (Ubiquitin thioesterase 2) (Ubiquitin-specific-processing protease 2) (Deubiquitinating enzyme 2) (41 kDa ubiquitin-specific protease) | 2.482E-05 | n/a | n/a | A |
| Q99LI7 | CSTF3_MOUSE | 717 | 82877 | 8.1 | (Q99LI7) Cleavage stimulation factor 77 kDa subunit (CSTF 77 kDa subunit) (CF-1 77 kDa subunit) (CstF-77) | 2.4118E-05 | n/a | n/a | A |
| Q6ZQ58 | LARP1_MOUSE | 364 | 41971 | 9.7 | (Q6ZQ58) La-related protein 1 (La ribonucleoprotein domain family member 1) (Fragment) | 2.407E-05 | n/a | n/a | A |
| Q8BZT5 | LRC19_MOUSE | 364 | 41573 | 4.9 | (Q8BZT5) Leucine-rich repeat-containing protein 19 precursor | 2.407E-05 | n/a | n/a | A |
| P97792 | CXAR_MOUSE | 365 | 39948 | 7 | (P97792) Coxsackievirus and adenovirus receptor homolog precursor (CAR) (mCAR) | 2.4004E-05 | n/a | n/a | A |
| Q9JK48 | SHLB1_MOUSE | 365 | 40855 | 6 | (Q9JK48) SH3 domain GRB2-like protein B1 (EC 2.3.1.-) (Endophilin B1) | 2.4004E-05 | n/a | n/a | A |
| Q5ND29 | RILP_MOUSE | 369 | 41139 | 5.3 | (Q5ND29) Rab-interacting lysosomal protein | 2.3743E-05 | n/a | n/a | A |

| | | | | | | | | | |
|---|---|---|---|---|---|---|---|---|---|
| Q9R0Q6 | ARC1A_MOUSE | 370 | 41626 | 8.2 | (Q9R0Q6) Actin-related protein 2/3 complex subunit 1A (SOP2-like protein) (Sid 329) | 2.3679E-05 | n/a | n/a | A |
| P27641 | KU86_MOUSE | 731 | 83192 | 5.3 | (P27641) ATP-dependent DNA helicase 2 subunit 2 (EC 3.6.1.-) (ATP-dependent DNA helicase II 80 kDa subunit) (Ku autoantigen protein p86 homolog) (Ku80) (CTC box-binding factor 85 kDa subunit) (CTCBF) (CTC85) (Nuclear factor IV) (DNA-repair pro | 2.3657E-05 | n/a | n/a | A |
| P97452 | BOP1_MOUSE | 732 | 82546 | 6.3 | (P97452) Ribosome biogenesis protein BOP1 (Block of proliferation 1 protein) | 2.3624E-05 | n/a | n/a | A |
| Q02257 | PLAK_MOUSE | 745 | 81801 | 6.1 | (Q02257) Junction plakoglobin (Desmoplakin-3) (Desmoplakin III) | 2.352E-05 | n/a | n/a | A |
| P70460 | VASP_MOUSE | 374 | 39535 | 8.5 | (P70460) Vasodilator-stimulated phosphoprotein (VASP) | 2.3426E-05 | n/a | n/a | A |
| P18654 | KS6A3_MOUSE | 740 | 83694 | 6.9 | (P18654) Ribosomal protein S6 kinase alpha-3 (EC 2.7.11.1) (S6K-alpha 3) (90 kDa ribosomal protein S6 kinase 3) (p90-RSK 3) (Ribosomal S6 kinase 2) (RSK-2) (pp90RSK2) (MAP kinase-activated protein kinase 1b) (MAPKAPK1B) | 2.3369E-05 | n/a | n/a | A |
| Q71FD7 | FBLI1_MOUSE | 375 | 41069 | 6.6 | (Q71FD7) Filamin-binding LIM protein 1 (CSX-associated LIM) | 2.3364E-05 | n/a | n/a | A |
| P19426 | NELFE_MOUSE | 375 | 42554 | 9.4 | (P19426) Negative elongation factor E (NELF-E) (RD protein) | 2.3364E-05 | n/a | n/a | A |
| Q9R1M5 | NALP5_MOUSE | 1111 | 125502 | 5.8 | (Q9R1M5) NACHT, LRR and PYD-containing protein 5 (Maternal antigen that embryos require) (Mater protein) (Ooplasm-specific protein 1) (OP1) | 2.3348E-05 | n/a | n/a | A |
| O88856 | TPS2_MOUSE | 376 | 42067 | 9.3 | (O88856) Protein-tyrosine sulfotransferase 2 (EC 2.8.2.20) (Tyrosylprotein sulfotransferase-2) (TPST-2) | 2.3301E-05 | n/a | n/a | A |
| P53564 | CUTL1_MOUSE | 1515 | 165595 | 6.3 | (P53564) Homeobox protein cut-like 1 (CCAAT displacement protein) (CDP) (Homeobox protein Cux) | 2.2829E-05 | n/a | n/a | A |
| P63005 | LIS1_MOUSE | 409 | 46539 | 7.4 | (P63005) Platelet-activating factor acetylhydrolase IB subunit alpha (PAF acetylhydrolase 45 kDa subunit) (PAF-AH 45 kDa subunit) (PAF-AH alpha) (PAFAH alpha) (Lissencephaly-1 protein) (LIS-1) | 2.1421E-05 | n/a | n/a | A |
| Q8C9X6 | EPC1_MOUSE | 813 | 90411 | 8.3 | (Q8C9X6) Enhancer of polycomb homolog 1 | 2.1271E-05 | n/a | n/a | A |
| Q9EPJ9 | ARFG1_MOUSE | 414 | 45288 | 5.6 | (Q9EPJ9) ADP-ribosylation factor GTPase-activating protein 1 (ADP-ribosylation factor 1 GTPase-activating protein) (ARF1 GAP) (ARF1-directed GTPase-activating protein) (GAP protein) | 2.1163E-05 | n/a | n/a | A |
| P18052 | PTPRA_MOUSE | 829 | 93698 | 7.1 | (P18052) Receptor-type tyrosine-protein phosphatase alpha precursor (EC 3.1.3.48) (Protein-tyrosine phosphatase alpha) (R-PTP-alpha) (LCA-related phosphatase) (PTPTY-28) | 2.1137E-05 | n/a | n/a | A |
| O08917 | FLOT1_MOUSE | 428 | 47513 | 7.2 | (O08917) Flotillin-1 | 2.047E-05 | n/a | n/a | A |
| P41778 | PBX1_MOUSE | 430 | 46626 | 7 | (P41778) Pre-B-cell leukemia transcription factor 1 (Homeobox protein PBX1) | 2.0375E-05 | n/a | n/a | A |
| P06795 | MDR1_MOUSE | 1276 | 140993 | 8.3 | (P06795) Multidrug resistance protein 1 (EC 3.6.3.44) (P-glycoprotein 1) (CD243 antigen) | 2.0329E-05 | n/a | n/a | A |
| Q9DCD2 | XAB2_MOUSE | 855 | 99988 | 6.2 | (Q9DCD2) XPA-binding protein 2 | 2.0226E-05 | n/a | n/a | A |
| Q6PAL8 | RA6I1_MOUSE | 1287 | 146653 | 6.7 | (Q6PAL8) Rab6-interacting protein 1 (Rab6IP1) | 2.0155E-05 | n/a | n/a | A |
| Q6PEM6 | GRAM3_MOUSE | 445 | 49287 | 8.1 | (Q6PEM6) GRAM domain-containing protein 3 | 1.9688E-05 | n/a | n/a | A |
| P60229 | IF36_MOUSE | 445 | 52221 | 6 | (P60229) Eukaryotic translation initiation factor 3 subunit 6 (eIF-3 p48) (eIF3e) (Mammary tumor-associated protein INT-6) (Viral integration site protein INT-6) (MMTV integration site 6) | 1.9688E-05 | n/a | n/a | A |

| | | | | | | | | | |
|---|---|---|---|---|---|---|---|---|---|
| Q8BJL0 | SMAL1_MOUSE | 910 | 100840 | 8.9 | (Q8BJL0) SWI/SNF-related matrix-associated actin-dependent regulator of chromatin subfamily A-like protein 1 (EC 3.6.1.-) (Sucrose nonfermenting protein 2-like 1) (HepA-related protein) (mharp) | 1.9256E-05 | n/a | n/a | A |
| Q99JR5 | TINAL_MOUSE | 466 | 52665 | 6.8 | (Q99JR5) Tubulointerstitial nephritis antigen-like precursor (Androgen-regulated gene 1 protein) (Adrenocortical zonation factor 1) (AZ-1) (Tubulointerstitial nephritis antigen-related protein) (TARP) | 1.8801E-05 | n/a | n/a | A |
| Q9Z0N1 | IF2G_MOUSE | 471 | 50934 | 8.4 | (Q9Z0N1) Eukaryotic translation initiation factor 2 subunit 3, X-linked (Eukaryotic translation initiation factor 2 subunit gamma, X linked) (eIF-2-gamma X) | 1.8602E-05 | n/a | n/a | A |
| O88199 | CHST3_MOUSE | 472 | 53997 | 9.1 | (O88199) Carbohydrate sulfotransferase 3 (EC 2.8.2.17) (Chondroitin 6-O-sulfotransferase 1) (C6ST-1) (Galactose/N-acetylglucosamine/N-acetylglucosamine 6-O-sulfotransferase 0) (GST-0) | 1.8562E-05 | n/a | n/a | A |
| P48377 | RFX1_MOUSE | 963 | 103693 | 6.3 | (P48377) DNA-binding protein RFX1 | 1.8196E-05 | n/a | n/a | A |
| Q6NXI6 | K0460_MOUSE | 1469 | 156585 | 7.6 | (Q6NXI6) Protein KIAA0460 | 1.7658E-05 | n/a | n/a | A |
| Q8VDM1 | ZGPAT_MOUSE | 511 | 56411 | 5.3 | (Q8VDM1) Zinc finger CCCH-type with G patch domain protein | 1.7145E-05 | n/a | n/a | A |
| Q9CS84 | NRX1A_MOUSE | 1514 | 166168 | 6.1 | (Q9CS84) Neurexin-1-alpha precursor (Neurexin I-alpha) | 1.7133E-05 | n/a | n/a | A |
| O09159 | MA2B1_MOUSE | 1013 | 114604 | 8.1 | (O09159) Lysosomal alpha-mannosidase precursor (EC 3.2.1.24) (Mannosidase, alpha B) (Lysosomal acid alpha-mannosidase) (Laman) (Mannosidase alpha class 2B member 1) | 1.7071E-05 | n/a | n/a | A |
| Q8CIF4 | BTD_MOUSE | 524 | 58601 | 5.8 | (Q8CIF4) Biotinidase precursor (EC 3.5.1.12) | 1.672E-05 | n/a | n/a | A |
| Q9Z0H8 | CYLN2_MOUSE | 1047 | 115910 | 6.5 | (Q9Z0H8) Cytoplasmic linker protein 2 (Cytoplasmic linker protein 115) (CLIP-115) | 1.6517E-05 | n/a | n/a | A |
| Q61753 | SERA_MOUSE | 532 | 56454 | 6.5 | (Q61753) D-3-phosphoglycerate dehydrogenase (EC 1.1.1.95) (3-PGDH) (A10) | 1.6469E-05 | n/a | n/a | A |
| Q62470 | ITA3_MOUSE | 1053 | 116745 | 6.6 | (Q62470) Integrin alpha-3 precursor (Galactoprotein B3) (GAPB3) (VLA-3 alpha chain) (CD49c antigen) [Contains: Integrin alpha-3 heavy chain; Integrin alpha-3 light chain] | 1.6423E-05 | n/a | n/a | A |
| Q8CGV9 | TSH3_MOUSE | 1081 | 118626 | 7 | (Q8CGV9) Teashirt homolog 3 (Zinc finger protein 537) | 1.5997E-05 | n/a | n/a | A |
| Q9EPU0 | RENT1_MOUSE | 1113 | 122657 | 6.7 | (Q9EPU0) Regulator of nonsense transcripts 1 (EC 3.6.1.-) (ATP dependent helicase RENT1) (Nonsense mRNA reducing factor 1) (NORF1) (Up-frameshift suppressor 1 homolog) (mUpf1) | 1.5744E-05 | n/a | n/a | A |
| Q61508 | ECM1_MOUSE | 559 | 62775 | 6.7 | (Q61508) Extracellular matrix protein 1 precursor (Secretory component p85) | 1.5673E-05 | n/a | n/a | A |
| O55134 | PCD12_MOUSE | 1180 | 128674 | 5.1 | (O55134) Protocadherin-12 precursor (Vascular cadherin-2) (Vascular endothelial cadherin-2) (VE-cadherin-2) (VE-cad-2) | 1.485E-05 | n/a | n/a | A |
| Q810B6 | ANFY1_MOUSE | 1169 | 128604 | 5.9 | (Q810B6) Ankyrin repeat and FYVE domain-containing protein 1 (Ankyrin repeats hooked to a zinc finger motif) | 1.4793E-05 | n/a | n/a | A |
| Q91VH2 | SNX9_MOUSE | 595 | 66546 | 5.5 | (Q91VH2) Sorting nexin-9 | 1.4725E-05 | n/a | n/a | A |
| Q8CG48 | SMC2_MOUSE | 1191 | 134273 | 8.4 | (Q8CG48) Structural maintenance of chromosome 2-like 1 protein (Chromosome-associated protein E) (XCAP-E homolog) (FGF-inducible protein 16) | 1.452E-05 | n/a | n/a | A |
| P04627 | ARAF_MOUSE | 604 | 67581 | 9.1 | (P04627) A-Raf proto-oncogene serine/threonine-protein kinase (EC 2.7.11.1) | 1.4505E-05 | n/a | n/a | A |

| | | | | | | | | | |
|---|---|---|---|---|---|---|---|---|---|
| P01132 | EGF_MOUSE | 1217 | 133144 | 6.5 | (P01132) Pro-epidermal growth factor precursor (EGF) [Contains: Epidermal growth factor] | 1.4398E-05 | n/a | n/a | A |
| P59509 | ATS19_MOUSE | 1210 | 134561 | 7.6 | (P59509) ADAMTS-19 precursor (EC 3.4.24.-) (A disintegrin and metalloproteinase with thrombospondin motifs 19) (ADAM-TS 19) (ADAM-TS19) | 1.4292E-05 | n/a | n/a | A |
| P35922 | FMR1_MOUSE | 614 | 68989 | 7.6 | (P35922) Fragile X mental retardation protein 1 homolog (Protein FMR-1) (FMRP) (mFmr1p) | 1.4269E-05 | n/a | n/a | A |
| Q99PU8 | DHX30_MOUSE | 1217 | 136668 | 8.7 | (Q99PU8) Putative ATP-dependent RNA helicase DHX30 (EC 3.6.1.-) (DEAH box protein 30) | 1.4209E-05 | n/a | n/a | A |
| P08775 | RPB1_MOUSE | 1970 | 217174 | 7.4 | (P08775) DNA-directed RNA polymerase II largest subunit (EC 2.7.7.6) (RPB1) | 1.3342E-05 | n/a | n/a | A |
| P70399 | TP53B_MOUSE | 1957 | 211338 | 4.6 | (P70399) Tumor suppressor p53-binding protein 1 (p53-binding protein 1) (p53BP1) (53BP1) | 1.3255E-05 | n/a | n/a | A |
| Q69ZS7 | HBS1L_MOUSE | 682 | 75101 | 6.5 | (Q69ZS7) HBS1-like protein | 1.2847E-05 | n/a | n/a | A |
| Q61102 | ABCB7_MOUSE | 694 | 76418 | 9.1 | (Q61102) ATP-binding cassette sub-family B member 7, mitochondrial (ATP-binding cassette transporter 7) (ABC transporter 7 protein) (Fragment) | 1.2624E-05 | n/a | n/a | A |
| Q8K3W3 | CASC3_MOUSE | 697 | 75673 | 6.4 | (Q8K3W3) Protein CASC3 (Cancer susceptibility candidate gene 3 protein homolog) (Metastatic lymph node protein 51 homolog) (MLN 51 protein homolog) (Barentsz protein) (Btz) | 1.257E-05 | n/a | n/a | A |
| Q8BJS4 | UN84B_MOUSE | 699 | 78195 | 7 | (Q8BJS4) Sad1/unc-84-like protein 2 | 1.2534E-05 | n/a | n/a | A |
| P57716 | NICA_MOUSE | 708 | 78490 | 6.1 | (P57716) Nicastrin precursor | 1.2375E-05 | n/a | n/a | A |
| Q571K4 | TAB3_MOUSE | 716 | 79029 | 8.5 | (Q571K4) Mitogen-activated protein kinase kinase kinase 7-interacting protein 3 (TAK1-binding protein 3) | 1.2236E-05 | n/a | n/a | A |
| Q91WD2 | TRPV6_MOUSE | 727 | 83195 | 6.8 | (Q91WD2) Transient receptor potential cation channel subfamily V member 6 (TrpV6) (Epithelial calcium channel 2) (ECaC2) (Calcium transport protein 1) (CaT1) | 1.2051E-05 | n/a | n/a | A |
| Q9CZD3 | SYG_MOUSE | 729 | 81878 | 6.7 | (Q9CZD3) Glycyl-tRNA synthetase (EC 6.1.1.14) (Glycine--tRNA ligase) (GlyRS) | 1.2018E-05 | n/a | n/a | A |
| Q9CZR2 | NALD2_MOUSE | 740 | 82801 | 8.4 | (Q9CZR2) N-acetylated-alpha-linked acidic dipeptidase 2 (EC 3.4.17.21) (N-acetylated-alpha-linked acidic dipeptidase II) (NAALADase II) (Glutamate carboxypeptidase III) (GCPIII) (N-acetylaspartylglutamate peptidase II) (NAAG-peptidase II) | 1.184E-05 | n/a | n/a | A |
| Q9R1B9 | SLIT2_MOUSE | 1521 | 168770 | 7.1 | (Q9R1B9) Slit homolog 2 protein precursor (Slit-2) [Contains: Slit homolog 2 protein N-product; Slit homolog 2 protein C-product] | 1.152E-05 | n/a | n/a | A |
| Q8BX17 | GEMI5_MOUSE | 1502 | 166561 | 6.7 | (Q8BX17) Gem-associated protein 5 (Gemin5) | 1.1513E-05 | n/a | n/a | A |
| Q8C1D8 | IWS1_MOUSE | 766 | 85248 | 4.7 | (Q8C1D8) IWS1 homolog (IWS1-like protein) | 1.1438E-05 | n/a | n/a | A |
| Q03142 | FGFR4_MOUSE | 799 | 88661 | 6.5 | (Q03142) Fibroblast growth factor receptor 4 precursor (EC 2.7.10.1) (FGFR-4) (Protein-tyrosine kinase receptor MPK-11) | 1.0965E-05 | n/a | n/a | A |
| O70496 | CLCN7_MOUSE | 803 | 88713 | 7.3 | (O70496) Chloride channel protein 7 (ClC-7) | 1.0911E-05 | n/a | n/a | A |
| Q80XQ2 | TBCD5_MOUSE | 815 | 91853 | 6.8 | (Q80XQ2) TBC1 domain family member 5 | 1.075E-05 | n/a | n/a | A |
| Q8BX02 | ANR25_MOUSE | 843 | 90245 | 5.6 | (Q8BX02) Ankyrin repeat domain-containing protein 25 | 1.0393E-05 | n/a | n/a | A |
| P56677 | ST14_MOUSE | 855 | 94655 | 6.8 | (P56677) Suppressor of tumorigenicity protein 14 (EC 3.4.21.-) (Serine protease 14) (Epithin) | 1.0247E-05 | n/a | n/a | A |
| Q8K3I4 | MYRIP_MOUSE | 856 | 94924 | 6.1 | (Q8K3I4) Rab effector MyRIP (Myosin-VIIa- and Rab-interacting protein) (Exophilin-8) (Slp homolog lacking C2 domains c) | 1.0235E-05 | n/a | n/a | A |

| | | | | | | | | | |
|---|---|---|---|---|---|---|---|---|---|
| Q9ESV0 | DDX24_MOUSE | 857 | 96471 | 9.4 | (Q9ESV0) ATP-dependent RNA helicase DDX24 (EC 3.6.1.-) (DEAD box protein 24) | 1.0223E-05 | n/a | n/a | A |
| Q99PP2 | ZN318_MOUSE | 2025 | 223867 | 6 | (Q99PP2) Zinc finger protein 318 (Testicular zinc finger protein) | 8.5397E-06 | n/a | n/a | A |
| Q9EQU3 | TLR9_MOUSE | 1032 | 116348 | 8.7 | (Q9EQU3) Toll-like receptor 9 precursor (CD289 antigen) | 8.4896E-06 | n/a | n/a | A |
| Q9JI57 | GT2D1_MOUSE | 1104 | 123483 | 7.1 | (Q9JI57) General transcription factor II-I repeat domain-containing protein 1 (GTF2I repeat domain-containing protein 1) (Binding factor for early enhancer) | 7.936E-06 | n/a | n/a | A |
| Q9ES00 | UBE4B_MOUSE | 1173 | 133303 | 6.1 | (Q9ES00) Ubiquitin conjugation factor E4 B (Ubiquitin-fusion degradation protein 2) (Ufd2a) | 7.4692E-06 | n/a | n/a | A |
| Q8BYR5 | CAPS2_MOUSE | 1297 | 147840 | 6.1 | (Q8BYR5) Calcium-dependent secretion activator 2 (Calcium-dependent activator protein for secretion 2) (CAPS-2) | 6.7551E-06 | n/a | n/a | A |
| Q04690 | NF1_MOUSE | 2841 | 319595 | 7.4 | (Q04690) Neurofibromin (Neurofibromatosis-related protein NF-1) | 6.0869E-06 | n/a | n/a | A |
| P97393 | RHG05_MOUSE | 1501 | 171926 | 6.4 | (P97393) Rho-GTPase-activating protein 5 (p190-B) | 5.837E-06 | n/a | n/a | A |
| Q9ESD7 | DYSF_MOUSE | 2083 | 237127 | 5.8 | (Q9ESD7) Dysferlin (Dystrophy-associated fer-1-like protein) (Fer-1-like protein 1) | 4.2061E-06 | n/a | n/a | A |
| Q64512 | PTN13_MOUSE | 2453 | 270332 | 6.4 | (Q64512) Tyrosine-protein phosphatase non-receptor type 13 (EC 3.1.3.48) (Protein tyrosine phosphatase PTP-BL) (Protein-tyrosine phosphatase RIP) (protein tyrosine phosphatase DPZPTP) (PTP36) | 3.5717E-06 | n/a | n/a | A |
| Q9DCC8 | TOM20_MOUSE | 145 | 16284 | 8.6 | (Q9DCC8) Mitochondrial import receptor subunit TOM20 homolog (Mitochondrial 20 kDa outer membrane protein) (Outer mitochondrial membrane receptor Tom20) | 0.00244848 | n/a | n/a | B |
| Q9CZL5 | PHS2_MOUSE | 103 | 11740 | 6.8 | (Q9CZL5) Pterin-4-alpha-carbinolamine dehydratase 2 (EC 4.2.1.96) (PHS 2) (4-alpha-hydroxy-tetrahydropterin dehydratase 2) (DcoH-like protein DCoHm) (Dimerization cofactor of hepatocyte nuclear factor 1 from muscle) (HNF1-alpha dimerization co | 0.00037793 | n/a | n/a | B |
| Q8VHB5 | CAH9_MOUSE | 437 | 47265 | 4.8 | (Q8VHB5) Carbonic anhydrase 9 precursor (EC 4.2.1.1) (Carbonic anhydrase IX) (Carbonate dehydratase IX) (CA-IX) (CAIX) (Membrane antigen MN homolog) | 0.00036108 | n/a | n/a | B |
| O55142 | RL35A_MOUSE | 110 | 12554 | 10.9 | (O55142) 60S ribosomal protein L35a | 0.00026541 | n/a | n/a | B |
| Q9R022 | DJC12_MOUSE | 198 | 22853 | 6.2 | (Q9R022) DnaJ homolog subfamily C member 12 (J domain-containing protein 1) | 0.00024575 | n/a | n/a | B |
| Q9Z1N5 | UAP56_MOUSE | 428 | 49035 | 5.7 | (Q9Z1N5) Spliceosome RNA helicase Bat1 (EC 3.6.1.-) (DEAD box protein UAP56) (56 kDa U2AF65-associated protein) (HLA-B-associated transcript 1) | 0.00022737 | n/a | n/a | B |
| Q62446 | FKBP3_MOUSE | 224 | 25148 | 9.3 | (Q62446) FK506-binding protein 3 (EC 5.2.1.8) (Peptidyl-prolyl cis-trans isomerase) (PPIase) (Rotamase) (25 kDa FKBP) (FKBP-25) (Rapamycin-selective 25 kDa immunophilin) | 0.00021722 | n/a | n/a | B |
| P58802 | TBC10_MOUSE | 500 | 56202 | 7.9 | (P58802) TBC1 domain family member 10A (EBP50-PDX interactor of 64 kDa) (EPI64 protein) | 0.00021409 | n/a | n/a | B |
| P61620 | S61A1_MOUSE | 475 | 52134 | 8.1 | (P61620) Protein transport protein Sec61 subunit alpha isoform 1 (Sec61 alpha-1) | 0.00020762 | n/a | n/a | B |
| P36536 | SAR1A_MOUSE | 198 | 22371 | 6.9 | (P36536) GTP-binding protein SAR1a | 0.0001966 | n/a | n/a | B |
| Q64152 | BTF3_MOUSE | 204 | 22031 | 9.5 | (Q64152) Transcription factor BTF3 (RNA polymerase B transcription factor 3) | 0.00019082 | n/a | n/a | B |

| | | | | | | | | | |
|---|---|---|---|---|---|---|---|---|---|
| Q99N94 | RM09_MOUSE | 265 | 30244 | 10.1 | (Q99N94) 39S ribosomal protein L9, mitochondrial precursor (L9mt) (MRP-L9) | 0.00018361 | n/a | n/a | B |
| P62274 | RS29_MOUSE | 55 | 6546 | 10.1 | (P62274) 40S ribosomal protein S29 | 0.00017931 | n/a | n/a | B |
| P62746 | RHOB_MOUSE | 196 | 22123 | 5.2 | (P62746) Rho-related GTP-binding protein RhoB precursor | 0.00015095 | n/a | n/a | B |
| P84244 | H33_MOUSE | 135 | 15197 | 11.3 | (P84244) Histone H3.3 | 0.0001461 | n/a | n/a | B |
| P63158 | HMGB1_MOUSE | 214 | 24763 | 5.7 | (P63158) High mobility group protein B1 (High mobility group protein 1) (HMG-1) | 0.00013642 | n/a | n/a | B |
| Q9CPW4 | ARPC5_MOUSE | 150 | 16157 | 5.7 | (Q9CPW4) Actin-related protein 2/3 complex subunit 5 (ARP2/3 complex 16 kDa subunit) (p16-ARC) | 0.00012975 | n/a | n/a | B |
| Q7TN05 | AP1S3_MOUSE | 154 | 18440 | 6.3 | (Q7TN05) AP-1 complex subunit sigma-3 (Adapter-related protein complex 1 sigma-1C subunit) (Sigma-adaptin 1C) (Adaptor protein complex AP-1 sigma-1C subunit) (Golgi adaptor HA1/AP1 adaptin sigma-1C subunit) (Clathrin assembly protein complex | 0.00012808 | n/a | n/a | B |
| P61750 | ARF4_MOUSE | 179 | 20265 | 7.2 | (P61750) ADP-ribosylation factor 4 | 0.00011019 | n/a | n/a | B |
| Q9CR95 | NECP1_MOUSE | 275 | 29639 | 6.4 | (Q9CR95) Adaptin ear-binding coat-associated protein 1 (NECAP-1) | 0.00010616 | n/a | n/a | B |
| O35226 | PSD4_MOUSE | 376 | 40704 | 4.8 | (O35226) 26S proteasome non-ATPase regulatory subunit 4 (26S proteasome regulatory subunit S5A) (Rpn10) (Multiubiquitin chain-binding protein) | 0.00010353 | n/a | n/a | B |
| Q99JY4 | YV03_MOUSE | 376 | 42189 | 8.4 | (Q99JY4) Protein PP2447 homolog | 0.00010353 | n/a | n/a | B |
| P0C0A3 | CHMP6_MOUSE | 199 | 23284 | 5.4 | (P0C0A3) Charged multivesicular body protein 6 (Chromatin-modifying protein 6) | 9.9115E-05 | n/a | n/a | B |
| Q6P069 | SORCN_MOUSE | 198 | 21627 | 5.6 | (Q6P069) Sorcin | 9.8299E-05 | n/a | n/a | B |
| Q8CJ61 | CKLF4_MOUSE | 208 | 22921 | 5.6 | (Q8CJ61) CKLF-like MARVEL transmembrane domain-containing protein 4 (Chemokine-like factor superfamily member 4) | 9.4826E-05 | n/a | n/a | B |
| Q9ESW8 | PGPI_MOUSE | 209 | 22934 | 5.4 | (Q9ESW8) Pyroglutamyl-peptidase 1 (EC 3.4.19.3) (Pyroglutamyl-peptidase I) (Pyrrolidone-carboxylate peptidase) (5-oxoprolyl-peptidase) (PGP-I) | 9.4372E-05 | n/a | n/a | B |
| Q9JK45 | KCNQ5_MOUSE | 878 | 97038 | 9.4 | (Q9JK45) Potassium voltage-gated channel subfamily KQT member 5 (Voltage-gated potassium channel subunit Kv7.5) (Potassium channel subunit alpha KvLQT5) (KQT-like 5) (Fragment) | 8.867E-05 | n/a | n/a | B |
| P83940 | ELOC_MOUSE | 112 | 12473 | 4.8 | (P83940) Transcription elongation factor B polypeptide 1 (RNA polymerase II transcription factor SIII subunit C) (SIII p15) (Elongin-C) (EloC) (Elongin 15 kDa subunit) (Stromal membrane-associated protein SMAP1B homolog) | 8.8053E-05 | n/a | n/a | B |
| P70158 | ASM3A_MOUSE | 445 | 49843 | 6.3 | (P70158) Acid sphingomyelinase-like phosphodiesterase 3a precursor (EC 3.1.4.-) (ASM-like phosphodiesterase 3a) | 8.7475E-05 | n/a | n/a | B |
| O35386 | PAHX_MOUSE | 338 | 38607 | 7.5 | (O35386) Phytanoyl-CoA dioxygenase, peroxisomal precursor (EC 1.14.11.18) (Phytanoyl-CoA alpha-hydroxylase) (PhyH) (Phytanic acid oxidase) (Lupus nephritis-associated peptide 1) | 8.6375E-05 | n/a | n/a | B |
| Q922Z0 | OXDD_MOUSE | 341 | 37546 | 8.5 | (Q922Z0) D-aspartate oxidase (EC 1.4.3.1) (DASOX) (DDO) | 8.5615E-05 | n/a | n/a | B |
| Q7TND5 | RPF1_MOUSE | 349 | 40037 | 10 | (Q7TND5) Ribosome production factor 1 (Ribosome biogenesis protein RPF1) (Brix domain-containing protein 5) | 8.3653E-05 | n/a | n/a | B |
| Q9CQ36 | DPOE4_MOUSE | 118 | 12240 | 4.9 | (Q9CQ36) DNA polymerase epsilon subunit 4 (EC 2.7.7.7) (DNA polymerase II subunit 4) (DNA polymerase epsilon subunit p12) | 8.3576E-05 | n/a | n/a | B |

| | | | | | | | | | |
|---|---|---|---|---|---|---|---|---|---|
| P51175 | PPOX_MOUSE | 477 | 50871 | 8.8 | (P51175) Protoporphyrinogen oxidase (EC 1.3.3.4) (PPO) | 8.2699E-05 | n/a | n/a | B |
| Q9JME5 | AP3B2_MOUSE | 1082 | 119192 | 5.6 | (Q9JME5) AP-3 complex subunit beta-2 (Adapter-related protein complex 3 beta-2 subunit) (Beta3B-adaptin) (Adaptor protein complex AP-3 beta-2 subunit) (Clathrin assembly protein complex 3 beta-2 large chain) | 8.0947E-05 | n/a | n/a | B |
| Q8R104 | SIRT3_MOUSE | 257 | 28822 | 6.2 | (Q8R104) NAD-dependent deacetylase sirtuin-3 (EC 3.5.1.-) (SIR2-like protein 3) (mSIR2L3) | 7.6746E-05 | n/a | n/a | B |
| P31938 | MP2K1_MOUSE | 392 | 43343 | 6.7 | (P31938) Dual specificity mitogen-activated protein kinase kinase 1 (EC 2.7.12.2) (MAP kinase kinase 1) (MAPKK 1) (ERK activator kinase 1) (MAPK/ERK kinase 1) (MEK1) | 7.4476E-05 | n/a | n/a | B |
| P97298 | PEDF_MOUSE | 417 | 46234 | 7 | (P97298) Pigment epithelium-derived factor precursor (PEDF) (Stromal cell-derived factor 3) (SDF-3) (Caspin) | 7.0011E-05 | n/a | n/a | B |
| Q9D7A8 | ARMC1_MOUSE | 282 | 31247 | 5.6 | (Q9D7A8) Armadillo repeat-containing protein 1 | 6.9943E-05 | n/a | n/a | B |
| Q9R059 | FHL3_MOUSE | 289 | 31818 | 6.1 | (Q9R059) Four and a half LIM domains protein 3 (FHL-3) (Skeletal muscle LIM-protein 2) (SLIM 2) | 6.8249E-05 | n/a | n/a | B |
| Q9QYE6 | GOGA5_MOUSE | 729 | 82368 | 6.2 | (Q9QYE6) Golgin subfamily A member 5 (Golgin-84) (Sumiko protein) (Ret-II protein) | 6.6746E-05 | n/a | n/a | B |
| Q91VF2 | HNMT_MOUSE | 295 | 33665 | 5.1 | (Q91VF2) Histamine N-methyltransferase (EC 2.1.1.8) (HMT) | 6.5977E-05 | n/a | n/a | B |
| O88792 | JAM1_MOUSE | 300 | 32369 | 6.4 | (O88792) Junctional adhesion molecule A precursor (JAM-A) (Junctional adhesion molecule 1) (JAM-1) (CD321 antigen) | 6.5746E-05 | n/a | n/a | B |
| Q791V5 | MTCH2_MOUSE | 303 | 33499 | 8.3 | (Q791V5) Mitochondrial carrier homolog 2 | 6.5095E-05 | n/a | n/a | B |
| Q99KN9 | EPN4_MOUSE | 631 | 68513 | 6.3 | (Q99KN9) Epsin-4 (Epsin-related protein) (EpsinR) (Enthoprotin) | 6.169E-05 | n/a | n/a | B |
| Q91V61 | SFXN3_MOUSE | 321 | 35406 | 9.5 | (Q91V61) Sideroflexin-3 | 6.0633E-05 | n/a | n/a | B |
| P24529 | TY3H_MOUSE | 498 | 55993 | 6.1 | (P24529) Tyrosine 3-monooxygenase (EC 1.14.16.2) (Tyrosine 3 hydroxylase) (TH) | 5.8624E-05 | n/a | n/a | B |
| Q8K021 | SCAM1_MOUSE | 338 | 38029 | 7.7 | (Q8K021) Secretory carrier-associated membrane protein 1 (Secretory carrier membrane protein 1) | 5.8355E-05 | n/a | n/a | B |
| Q922U1 | PRPF3_MOUSE | 683 | 77455 | 9.5 | (Q922U1) U4/U6 small nuclear ribonucleoprotein Prp3 (Pre-mRNA-splicing factor 3) | 5.6993E-05 | n/a | n/a | B |
| Q9JM96 | BORG4_MOUSE | 349 | 37869 | 5.4 | (Q9JM96) Cdc42 effector protein 4 (Binder of Rho GTPases 4) | 5.6515E-05 | n/a | n/a | B |
| P29268 | CTGF_MOUSE | 348 | 37794 | 7.8 | (P29268) Connective tissue growth factor precursor (Protein FISP-12) (Hypertrophic chondrocyte-specific protein 24) | 5.5929E-05 | n/a | n/a | B |
| Q6P8I4 | PCNP_MOUSE | 178 | 18963 | 7.5 | (Q6P8I4) PEST-containing nuclear protein (PCNP) | 5.5404E-05 | n/a | n/a | B |
| P21278 | GNA11_MOUSE | 359 | 42024 | 6 | (P21278) Guanine nucleotide-binding protein alpha-11 subunit | 5.4941E-05 | n/a | n/a | B |
| Q3UHX2 | HAP28_MOUSE | 181 | 20605 | 7.4 | (Q3UHX2) 28 kDa heat- and acid-stable phosphoprotein (PDGF-associated protein) (PAP) (PDGFA-associated protein 1) (PAP1) | 5.4486E-05 | n/a | n/a | B |
| P09528 | FRIH_MOUSE | 181 | 20935 | 5.9 | (P09528) Ferritin heavy chain (EC 1.16.3.1) (Ferritin H subunit) | 5.4486E-05 | n/a | n/a | B |
| Q9WV32 | ARC1B_MOUSE | 371 | 41018 | 8.4 | (Q9WV32) Actin-related protein 2/3 complex subunit 1B (ARP2/3 complex 41 kDa subunit) (p41-ARC) | 5.3164E-05 | n/a | n/a | B |
| P70444 | BID_MOUSE | 195 | 21952 | 4.8 | (P70444) BH3-interacting domain death agonist (BID) (p22 BID) [Contains: BH3-interacting domain death agonist p15 (p15 BID); BH3-interacting domain death agonist p13 (p13 BID); BH3-interacting domain death agonist p11 (p11 BID)] | 5.0574E-05 | n/a | n/a | B |
| Q9CQU3 | RER1_MOUSE | 196 | 22988 | 9.5 | (Q9CQU3) RER1 protein | 5.0316E-05 | n/a | n/a | B |

| | | | | | | | | | |
|---|---|---|---|---|---|---|---|---|---|
| Q9D4H2 | GCC1_MOUSE | 778 | 87678 | 5.5 | (Q9D4H2) GRIP and coiled-coil domain-containing protein 1 (Golgi coiled coil protein 1) | 5.0034E-05 | n/a | n/a | B |
| Q61187 | TS101_MOUSE | 391 | 44124 | 6.7 | (Q61187) Tumor susceptibility gene 101 protein | 4.9778E-05 | n/a | n/a | B |
| Q9D1M7 | FKB11_MOUSE | 201 | 22137 | 9.4 | (Q9D1M7) FK506-binding protein 11 precursor (EC 5.2.1.8) (Peptidyl-prolyl cis-trans isomerase) (PPIase) (Rotamase) (19 kDa FK506-binding protein) (FKBP-19) | 4.9064E-05 | n/a | n/a | B |
| Q8JZY2 | COMDA_MOUSE | 202 | 22812 | 6.7 | (Q8JZY2) COMM domain-containing protein 10 (Down regulated in W/WV mouse stomach 2) (mDRWMS2) | 4.8821E-05 | n/a | n/a | B |
| Q9D1Q6 | TXND4_MOUSE | 406 | 46853 | 5.3 | (Q9D1Q6) Thioredoxin domain-containing protein 4 precursor (Endoplasmic reticulum resident protein ERp44) | 4.8581E-05 | n/a | n/a | B |
| Q9ERI5 | PTDSR_MOUSE | 403 | 46557 | 8.9 | (Q9ERI5) Protein PTDSR (Phosphatidylserine receptor) (Apoptotic cell clearance receptor PtdSerR) | 4.8296E-05 | n/a | n/a | B |
| P54729 | NUB1_MOUSE | 614 | 70307 | 5.9 | (P54729) NEDD8 ultimate buster 1 (Protein BS4) | 4.7548E-05 | n/a | n/a | B |
| Q921S7 | RM37_MOUSE | 423 | 48341 | 8.8 | (Q921S7) 39S ribosomal protein L37, mitochondrial precursor (L37mt) (MRP-L37) | 4.6628E-05 | n/a | n/a | B |
| P30412 | PPIC_MOUSE | 212 | 22794 | 7.5 | (P30412) Peptidyl-prolyl cis-trans isomerase C (EC 5.2.1.8) (PPIase) (Rotamase) (Cyclophilin C) | 4.6518E-05 | n/a | n/a | B |
| Q9QZM0 | UBQL2_MOUSE | 638 | 67379 | 5.3 | (Q9QZM0) Ubiquilin-2 (Protein linking IAP with cytoskeleton 2) (PLIC-2) (Ubiquitin-like product Chap1/Dsk2) (DSK2 homolog) (Chap1) | 4.576E-05 | n/a | n/a | B |
| Q8K0H5 | TAF10_MOUSE | 218 | 21841 | 6.5 | (Q8K0H5) Transcription initiation factor TFIID subunit 10 (Transcription initiation factor TFIID 30 kDa subunit) (TAF(II)30) (TAFII-30) (mTAFII30) | 4.5238E-05 | n/a | n/a | B |
| Q9DBZ5 | IF3C_MOUSE | 218 | 25087 | 4.9 | (Q9DBZ5) Eukaryotic translation initiation factor 3 subunit 12 (eIF-3 p25) (eIF3k) | 4.5238E-05 | n/a | n/a | B |
| Q8R2Y2 | MUC18_MOUSE | 648 | 71546 | 5.8 | (Q8R2Y2) Cell surface glycoprotein MUC18 precursor (Melanoma-associated antigen MUC18) (Melanoma cell adhesion molecule) (Gicerin) | 4.5054E-05 | n/a | n/a | B |
| Q9D6Z0 | ALKB7_MOUSE | 221 | 24970 | 6.5 | (Q9D6Z0) Alkylated repair protein alkB homolog 7 precursor | 4.4624E-05 | n/a | n/a | B |
| Q9ESP1 | SDF2L_MOUSE | 221 | 23648 | 7.4 | (Q9ESP1) Stromal cell-derived factor 2-like protein 1 precursor (SDF2-like protein 1) | 4.4624E-05 | n/a | n/a | B |
| Q6PEV3 | WIRE_MOUSE | 440 | 46298 | 11 | (Q6PEV3) WIP-related protein (WASP-interacting protein-related protein) | 4.4234E-05 | n/a | n/a | B |
| Q8K363 | DDX18_MOUSE | 660 | 74181 | 9.5 | (Q8K363) ATP-dependent RNA helicase DDX18 (EC 3.6.1.-) (DEAD box protein 18) | 4.4234E-05 | n/a | n/a | B |
| Q7TNE3 | SPAG7_MOUSE | 227 | 25921 | 7.4 | (Q7TNE3) Sperm-associated antigen 7 | 4.3445E-05 | n/a | n/a | B |
| P58044 | IDI1_MOUSE | 227 | 26289 | 6.2 | (P58044) Isopentenyl-diphosphate delta-isomerase 1 (EC 5.3.3.2) (IPP isomerase 1) (Isopentenyl pyrophosphate isomerase 1) (IPPI1) | 4.3445E-05 | n/a | n/a | B |
| Q6QD59 | SEC20_MOUSE | 228 | 26175 | 8.8 | (Q6QD59) Vesicle transport protein SEC20 | 4.3254E-05 | n/a | n/a | B |
| Q8BXZ1 | TXD10_MOUSE | 456 | 51848 | 5.2 | (Q8BXZ1) Protein disulfide-isomerase TXNDC10 precursor (EC 5.3.4.1) (Thioredoxin domain-containing protein 10) | 4.2682E-05 | n/a | n/a | B |
| P49698 | HNF4A_MOUSE | 465 | 51755 | 7.1 | (P49698) Hepatocyte nuclear factor 4-alpha (HNF-4-alpha) (Transcription factor HNF-4) (Transcription factor 14) | 4.2417E-05 | n/a | n/a | B |
| Q9R1C7 | PRP40_MOUSE | 953 | 108481 | 7.7 | (Q9R1C7) Pre-mRNA-processing factor 40 homolog A (Formin-binding protein 3) (Formin-binding protein 11) (FBP 11) | 4.0846E-05 | n/a | n/a | B |

| | | | | | | | | | |
|---|---|---|---|---|---|---|---|---|---|
| Q6P9Z1 | SMRD3_MOUSE | 483 | 54986 | 9.3 | (Q6P9Z1) SWI/SNF-related matrix-associated actin-dependent regulator of chromatin subfamily D member 3 (60 kDa BRG-1/Brm-associated factor subunit C) (BRG1-associated factor 60C) (mBAF60c) | 4.0836E-05 | n/a | n/a | B |
| Q61247 | A2AP_MOUSE | 491 | 54972 | 6.3 | (Q61247) Alpha-2-antiplasmin precursor (Alpha-2-plasmin inhibitor) (Alpha-2-PI) (Alpha-2-AP) | 3.964E-05 | n/a | n/a | B |
| Q8R092 | CA043_MOUSE | 253 | 28691 | 9.4 | (Q8R092) Protein C1orf43 homolog | 3.898E-05 | n/a | n/a | B |
| P11103 | PARP1_MOUSE | 1012 | 112969 | 9 | (P11103) Poly [ADP-ribose] polymerase 1 (EC 2.4.2.30) (PARP-1) (ADPRT) (NAD(+) ADP-ribosyltransferase 1) (Poly[ADP-ribose] synthetase 1) (msPARP) | 3.8465E-05 | n/a | n/a | B |
| P16015 | CAH3_MOUSE | 259 | 29235 | 7.4 | (P16015) Carbonic anhydrase 3 (EC 4.2.1.1) (Carbonic anhydrase III) (Carbonate dehydratase III) (CA-III) | 3.8077E-05 | n/a | n/a | B |
| O08579 | EMD_MOUSE | 259 | 29436 | 5 | (O08579) Emerin | 3.8077E-05 | n/a | n/a | B |
| P30658 | CBX2_MOUSE | 519 | 54890 | 9.9 | (P30658) Chromobox protein homolog 2 (Modifier 3 protein) (M33) | 3.7501E-05 | n/a | n/a | B |
| O88441 | MTX2_MOUSE | 263 | 29758 | 5.6 | (O88441) Metaxin-2 | 3.7498E-05 | n/a | n/a | B |
| Q91W89 | MA2C1_MOUSE | 1039 | 115688 | 6.5 | (Q91W89) Alpha-mannosidase 2C1 (EC 3.2.1.24) (Alpha-D-mannoside mannohydrolase) (Mannosidase alpha class 2C member 1) | 3.7465E-05 | n/a | n/a | B |
| P14483 | HB2A_MOUSE | 265 | 30128 | 7.2 | (P14483) H-2 class II histocompatibility antigen, A beta chain precursor | 3.7215E-05 | n/a | n/a | B |
| P52479 | UBP10_MOUSE | 792 | 87056 | 5.2 | (P52479) Ubiquitin carboxyl-terminal hydrolase 10 (EC 3.1.2.15) (Ubiquitin thioesterase 10) (Ubiquitin-specific-processing protease 10) (Deubiquitinating enzyme 10) | 3.6862E-05 | n/a | n/a | B |
| P81269 | ATF1_MOUSE | 269 | 29238 | 8.4 | (P81269) Cyclic AMP-dependent transcription factor ATF-1 (Activating transcription factor 1) (TCR-ATF1) | 3.6661E-05 | n/a | n/a | B |
| O88502 | PDE8A_MOUSE | 823 | 93171 | 5.8 | (O88502) High-affinity cAMP-specific and IBMX-insensitive 3',5'-cyclic phosphodiesterase 8A (EC 3.1.4.17) (MMPDE8) | 3.5474E-05 | n/a | n/a | B |
| P59326 | YTHD1_MOUSE | 559 | 60879 | 9 | (P59326) YTH domain protein 1 (Dermatomyositis associated with cancer putative autoantigen 1 homolog) (DACA-1 homolog) | 3.5284E-05 | n/a | n/a | B |
| O70362 | PHLD_MOUSE | 837 | 93255 | 7.1 | (O70362) Phosphatidylinositol-glycan-specific phospholipase D 1 precursor (EC 3.1.4.50) (PI-G PLD) (Glycoprotein phospholipase D) (Glycosyl-phosphatidylinositol-specific phospholipase D) | 3.488E-05 | n/a | n/a | B |
| O35098 | DPYL4_MOUSE | 572 | 61962 | 7 | (O35098) Dihydropyrimidinase-related protein 4 (DRP-4) (Collapsin response mediator protein 3) (CRMP-3) (UNC33-like phosphoprotein 4) (ULIP4 protein) | 3.4482E-05 | n/a | n/a | B |
| Q64362 | FTS1_MOUSE | 292 | 32942 | 9.3 | (Q64362) Fused toes protein (FT1) | 3.3774E-05 | n/a | n/a | B |
| Q9WUA2 | SYFB_MOUSE | 589 | 65670 | 7.1 | (Q9WUA2) Phenylalanyl-tRNA synthetase beta chain (EC 6.1.1.20) (Phenylalanine--tRNA ligase beta chain) (PheRS) | 3.3487E-05 | n/a | n/a | B |
| Q99MP8 | BRAP_MOUSE | 591 | 66991 | 5.6 | (Q99MP8) BRCA1-associated protein (EC 6.3.2.-) (BRAP2) (Impedes mitogenic signal propagation) (IMP) | 3.2933E-05 | n/a | n/a | B |
| Q9D903 | EBP2_MOUSE | 306 | 34703 | 10.1 | (Q9D903) Probable rRNA-processing protein EBP2 | 3.2228E-05 | n/a | n/a | B |
| Q99JX7 | NXF1_MOUSE | 618 | 70301 | 8.6 | (Q99JX7) Nuclear RNA export factor 1 (Tip-associating protein) (Tip-associated protein) (mRNA export factor TAP) | 3.1916E-05 | n/a | n/a | B |
| Q8K1A6 | CCD1A_MOUSE | 943 | 103698 | 7.8 | (Q8K1A6) Coiled-coil and C2 domain-containing protein 1A (Five repressor element under dual repression-binding protein 1) (FRE under dual repression-binding protein 1) (Freud-1) | 3.0959E-05 | n/a | n/a | B |

| | | | | | | | | | |
|---|---|---|---|---|---|---|---|---|---|
| Q91XL9 | OSBL1_MOUSE | 950 | 107795 | 6.4 | (Q91XL9) Oxysterol-binding protein-related protein 1 (OSBP-related protein 1) (ORP-1) | 3.0731E-05 | n/a | n/a | B |
| Q8VDK1 | NIT1_MOUSE | 323 | 35695 | 7.9 | (Q8VDK1) Nitrilase homolog 1 (EC 3.5.-.-) | 3.0532E-05 | n/a | n/a | B |
| P25916 | PCGF4_MOUSE | 324 | 36708 | 8.6 | (P25916) Polycomb group RING finger protein 4 (Polycomb complex protein BMI-1) | 3.0438E-05 | n/a | n/a | B |
| P05132 | KAPCA_MOUSE | 350 | 40439 | 8.8 | (P05132) cAMP-dependent protein kinase, alpha-catalytic subunit (EC 2.7.11.11) (PKA C-alpha) | 2.8177E-05 | n/a | n/a | B |
| Q3U5C8 | ARHGG_MOUSE | 713 | 80395 | 7.5 | (Q3U5C8) Rho guanine nucleotide exchange factor 16 | 2.7663E-05 | n/a | n/a | B |
| P58735 | S26A1_MOUSE | 704 | 75788 | 8.1 | (P58735) Sulfate anion transporter 1 (SAT-1) (Solute carrier family 26 member 1) | 2.7647E-05 | n/a | n/a | B |
| P46978 | STT3A_MOUSE | 705 | 80598 | 8.1 | (P46978) Dolichyl-diphosphooligosaccharide--protein glycosyltransferase subunit STT3A (EC 2.4.1.119) (Oligosaccharyl transferase subunit STT3A) (STT3-A) (B5) (Integral membrane protein 1) | 2.7607E-05 | n/a | n/a | B |
| Q7TMK6 | HOOK2_MOUSE | 716 | 83384 | 5.4 | (Q7TMK6) Hook homolog 2 | 2.7547E-05 | n/a | n/a | B |
| Q91XA5 | SNPC2_MOUSE | 359 | 38370 | 6.8 | (Q91XA5) snRNA-activating protein complex subunit 2 (SNAPc subunit 2) (snRNA-activating protein complex 45 kDa subunit) (SNAPc 45 kDa subunit) (Small nuclear RNA-activating complex polypeptide 2) | 2.7471E-05 | n/a | n/a | B |
| Q61112 | CAB45_MOUSE | 361 | 42064 | 5 | (Q61112) 45 kDa calcium-binding protein precursor (Cab45) (Stromal cell-derived factor 4) (SDF-4) | 2.7318E-05 | n/a | n/a | B |
| Q6PGF7 | EXOC8_MOUSE | 716 | 81035 | 5.4 | (Q6PGF7) Exocyst complex component 8 (Exocyst complex 84 kDa subunit) | 2.7183E-05 | n/a | n/a | B |
| Q8BWW4 | LARP4_MOUSE | 719 | 79763 | 6.4 | (Q8BWW4) La-related protein 4 (La ribonucleoprotein domain family member 4) | 2.707E-05 | n/a | n/a | B |
| P70365 | NCOA1_MOUSE | 1447 | 157015 | 6.1 | (P70365) Nuclear receptor coactivator 1 (EC 2.3.1.48) (NCoA-1) (Steroid receptor coactivator 1) (SRC-1) (Nuclear receptor coactivator protein 1) (mNRC-1) | 2.6901E-05 | n/a | n/a | B |
| Q62419 | SH3G1_MOUSE | 368 | 41518 | 5.7 | (Q62419) SH3-containing GRB2-like protein 1 (SH3 domain protein 2B) (SH3p8) | 2.6799E-05 | n/a | n/a | B |
| Q8C0D9 | CEP68_MOUSE | 733 | 78749 | 5.5 | (Q8C0D9) Centrosomal protein of 68 kDa (Cep68 protein) | 2.6553E-05 | n/a | n/a | B |
| Q8VE62 | PAIP1_MOUSE | 400 | 45702 | 4.5 | (Q8VE62) Polyadenylate-binding protein-interacting protein 1 (Poly(A)-binding protein-interacting protein 1) (PABP-interacting protein 1) (PAIP-1) | 2.4655E-05 | n/a | n/a | B |
| P70295 | AUP1_MOUSE | 410 | 46121 | 8.4 | (P70295) Ancient ubiquitous protein 1 precursor | 2.4053E-05 | n/a | n/a | B |
| Q00896 | A1AT3_MOUSE | 413 | 45854 | 5.5 | (Q00896) Alpha-1-antitrypsin 1-3 precursor (Serine protease inhibitor 1-3) (Alpha-1 protease inhibitor 3) | 2.3879E-05 | n/a | n/a | B |
| Q5DTU0 | K1914_MOUSE | 825 | 92175 | 5.4 | (Q5DTU0) Protein KIAA1914 | 2.3592E-05 | n/a | n/a | B |
| Q6P542 | ABCF1_MOUSE | 837 | 94945 | 6.5 | (Q6P542) ATP-binding cassette sub-family F member 1 | 2.3565E-05 | n/a | n/a | B |
| Q9CYC6 | DCP2_MOUSE | 422 | 48396 | 8 | (Q9CYC6) mRNA decapping enzyme 2 (EC 3.-.-.-) | 2.3369E-05 | n/a | n/a | B |
| Q922D4 | SAPS3_MOUSE | 844 | 94653 | 4.6 | (Q922D4) SAPS domain family member 3 | 2.3369E-05 | n/a | n/a | B |
| P59222 | SREC2_MOUSE | 833 | 87871 | 8.2 | (P59222) Scavenger receptor class F member 2 precursor (Scavenger receptor expressed by endothelial cells 2 protein) (SREC-II) | 2.3365E-05 | n/a | n/a | B |
| O08808 | DIAP1_MOUSE | 1255 | 139343 | 5.6 | (O08808) Protein diaphanous homolog 1 (Diaphanous-related formin-1) (DRF1) (mDIA1) (p140mDIA) | 2.3263E-05 | n/a | n/a | B |
| O09126 | SEM4D_MOUSE | 861 | 95714 | 7.8 | (O09126) Semaphorin-4D precursor (Semaphorin J) (Sema J) (Semaphorin C-like 2) (M-Sema G) | 2.2605E-05 | n/a | n/a | B |
| Q3TDN2 | UBXD8_MOUSE | 445 | 52471 | 5.5 | (Q3TDN2) UBX domain-containing protein 8 | 2.2162E-05 | n/a | n/a | B |

| | | | | | | | | | |
|---|---|---|---|---|---|---|---|---|---|
| O08784 | TCOF_MOUSE | 1320 | 135001 | 9.3 | (O08784) Treacle protein (Treacher Collins syndrome protein homolog) | 2.2117E-05 | n/a | n/a | B |
| Q9D4H9 | PHF14_MOUSE | 881 | 99105 | 5.3 | (Q9D4H9) PHD finger protein 14 | 2.2092E-05 | n/a | n/a | B |
| Q8C033 | ARHGA_MOUSE | 1345 | 147945 | 5.7 | (Q8C033) Rho guanine nucleotide exchange factor 10 | 2.1706E-05 | n/a | n/a | B |
| Q9D6X5 | CT054_MOUSE | 460 | 49605 | 6.7 | (Q9D6X5) Protein C20orf54 homolog precursor | 2.1439E-05 | n/a | n/a | B |
| Q9ESU6 | BRD4_MOUSE | 1400 | 155923 | 9.2 | (Q9ESU6) Bromodomain-containing protein 4 (Mitotic chromosome-associated protein) (MCAP) | 2.0853E-05 | n/a | n/a | B |
| Q61466 | SMRD1_MOUSE | 476 | 54929 | 9.1 | (Q61466) SWI/SNF-related matrix-associated actin-dependent regulator of chromatin subfamily D member 1 (SWI/SNF complex 60 kDa subunit) (60 kDa BRG-1/Brm-associated factor subunit A) (BRG1-associated factor 60A) (D15KZ1 protein) | 2.0718E-05 | n/a | n/a | B |
| Q9D7B1 | DUS2L_MOUSE | 493 | 55325 | 6.3 | (Q9D7B1) tRNA-dihydrouridine synthase 2-like (EC 1.-.-.-) | 2.0004E-05 | n/a | n/a | B |
| P15539 | C11B2_MOUSE | 500 | 57315 | 9.9 | (P15539) Cytochrome P450 11B2, mitochondrial precursor (EC 1.14.15.4) (EC 1.14.15.5) (CYPXIB2) (P450C11) (Steroid 11-beta-hydroxylase) (Aldosterone synthase) | 1.9724E-05 | n/a | n/a | B |
| Q61666 | HIRA_MOUSE | 1015 | 111616 | 8 | (Q61666) HIRA protein (TUP1-like enhancer of split protein 1) | 1.9432E-05 | n/a | n/a | B |
| Q9DC61 | MPPA_MOUSE | 524 | 58279 | 6.8 | (Q9DC61) Mitochondrial-processing peptidase alpha subunit, mitochondrial precursor (EC 3.4.24.64) (Alpha-MPP) (P-55) | 1.882E-05 | n/a | n/a | B |
| P38532 | HSF1_MOUSE | 525 | 57224 | 5.3 | (P38532) Heat shock factor protein 1 (HSF 1) (Heat shock transcription factor 1) (HSTF 1) | 1.8785E-05 | n/a | n/a | B |
| Q6NS82 | CB017_MOUSE | 541 | 57542 | 4.5 | (Q6NS82) Protein C2orf17 homolog | 1.8229E-05 | n/a | n/a | B |
| Q6P5F9 | XPO1_MOUSE | 1071 | 123093 | 6.1 | (Q6P5F9) Exportin-1 (Exp1) (Chromosome region maintenance 1 protein homolog) | 1.8173E-05 | n/a | n/a | B |
| P97363 | LCB2_MOUSE | 560 | 62982 | 8.2 | (P97363) Serine palmitoyltransferase 2 (EC 2.3.1.50) (Long chain base biosynthesis protein 2) (LCB 2) (Serine-palmitoyl-CoA transferase 2) (SPT 2) | 1.7611E-05 | n/a | n/a | B |
| Q61315 | APC_MOUSE | 2845 | 311089 | 7.6 | (Q61315) Adenomatous polyposis coli protein (Protein APC) (mAPC) | 1.7103E-05 | n/a | n/a | B |
| Q8K182 | CO8A_MOUSE | 587 | 66080 | 6.5 | (Q8K182) Complement component C8 alpha chain precursor (Complement component 8 subunit alpha) | 1.6801E-05 | n/a | n/a | B |
| P48318 | DCE1_MOUSE | 593 | 66648 | 7.2 | (P48318) Glutamate decarboxylase 1 (EC 4.1.1.15) (Glutamate decarboxylase, 67 kDa isoform) (GAD-67) (67 kDa glutamic acid decarboxylase) | 1.6631E-05 | n/a | n/a | B |
| Q8CFI7 | RPB2_MOUSE | 1174 | 133910 | 6.9 | (Q8CFI7) DNA-directed RNA polymerase II 140 kDa polypeptide (EC 2.7.7.6) (RNA polymerase II subunit 2) (RPB2) | 1.6579E-05 | n/a | n/a | B |
| P24527 | LKHA4_MOUSE | 610 | 68890 | 6.4 | (P24527) Leukotriene A-4 hydrolase (EC 3.3.2.6) (LTA-4 hydrolase) (Leukotriene A(4) hydrolase) | 1.6167E-05 | n/a | n/a | B |
| Q7TNF8 | RIMB1_MOUSE | 1846 | 199927 | 5.3 | (Q7TNF8) Peripheral-type benzodiazepine receptor-associated protein 1 (PRAX-1) (Peripheral benzodiazepine receptor-interacting protein) (PBR-IP) (RIM-binding protein 1) (RIM-BP1) | 1.5815E-05 | n/a | n/a | B |
| Q9Z148 | EHMT2_MOUSE | 1263 | 138039 | 5.8 | (Q9Z148) Histone-lysine N-methyltransferase, H3 lysine-9 specific 3 (EC 2.1.1.43) (Histone H3-K9 methyltransferase 3) (H3-K9-HMTase 3) (Euchromatic histone-lysine N-methyltransferase 2) (HLA-B-associated transcript 8) (Protein G9a) | 1.5617E-05 | n/a | n/a | B |
| Q920F6 | SM1L2_MOUSE | 1248 | 144513 | 7.1 | (Q920F6) Structural maintenance of chromosomes 1-like 2 protein (SMC1beta protein) | 1.5595E-05 | n/a | n/a | B |

| | | | | | | | | | |
|---|---|---|---|---|---|---|---|---|---|
| Q9DBG7 | SRPR_MOUSE | 636 | 69623 | 9 | (Q9DBG7) Signal recognition particle receptor subunit alpha (SR-alpha) (Docking protein alpha) (DP-alpha) | 1.5506E-05 | n/a | n/a | B |
| Q8CH02 | SF04_MOUSE | 643 | 72649 | 7.6 | (Q8CH02) Splicing factor 4 | 1.5337E-05 | n/a | n/a | B |
| Q6P9P0 | CJ006_MOUSE | 1278 | 143970 | 9.3 | (Q6P9P0) Protein C10orf6 homolog | 1.5229E-05 | n/a | n/a | B |
| Q63961 | EGLN_MOUSE | 653 | 70037 | 6.1 | (Q63961) Endoglin precursor (Cell surface MJ7/18 antigen) | 1.5102E-05 | n/a | n/a | B |
| P29121 | PCSK4_MOUSE | 655 | 73214 | 8.2 | (P29121) Proprotein convertase subtilisin/kexin type 4 precursor (EC 3.4.21.-) (PC4) (Neuroendocrine convertase 3) (NEC 3) (Prohormone convertase 3) (KEX2-like endoprotease 3) | 1.5056E-05 | n/a | n/a | B |
| O70161 | PI51C_MOUSE | 661 | 72408 | 5.6 | (O70161) Phosphatidylinositol-4-phosphate 5-kinase type-1 gamma (EC 2.7.1.68) (Phosphatidylinositol-4-phosphate 5-kinase type I gamma) (PtdIns(4)P-5-kinase gamma) (PtdInsPKIgamma) (PIP5KIgamma) | 1.492E-05 | n/a | n/a | B |
| P70388 | RAD50_MOUSE | 1312 | 153487 | 6.9 | (P70388) DNA repair protein RAD50 (EC 3.6.-.-) (mRad50) | 1.4835E-05 | n/a | n/a | B |
| P26450 | P85A_MOUSE | 724 | 83414 | 6.4 | (P26450) Phosphatidylinositol 3-kinase regulatory subunit alpha (PI3-kinase p85-subunit alpha) (PtdIns-3-kinase p85-alpha) (PI3K) | 1.3621E-05 | n/a | n/a | B |
| Q08481 | PECA1_MOUSE | 727 | 81263 | 6.7 | (Q08481) Platelet endothelial cell adhesion molecule precursor (PECAM-1) (CD31 antigen) | 1.3565E-05 | n/a | n/a | B |
| Q9WUN2 | TBK1_MOUSE | 729 | 83425 | 6.9 | (Q9WUN2) Serine/threonine-protein kinase TBK1 (EC 2.7.11.1) (TANK-binding kinase 1) (T2K) | 1.3528E-05 | n/a | n/a | B |
| Q64446 | ATP7B_MOUSE | 1462 | 157297 | 6.7 | (Q64446) Copper-transporting ATPase 2 (EC 3.6.3.4) (Copper pump 2) (Wilson disease-associated protein homolog) | 1.3313E-05 | n/a | n/a | B |
| Q60680 | IKKA_MOUSE | 745 | 84729 | 6.8 | (Q60680) Inhibitor of nuclear factor kappa-B kinase alpha subunit (EC 2.7.11.10) (I kappa-B kinase alpha) (IkBKA) (IKK-alpha) (IKK-A) (IkappaB kinase) (I-kappa-B kinase 1) (IKK1) (Conserved helix-loop-helix ubiquitous kinase) (Nuclear factor N | 1.3237E-05 | n/a | n/a | B |
| Q62441 | TLE4_MOUSE | 766 | 82943 | 7.4 | (Q62441) Transducin-like enhancer protein 4 (Groucho-related protein 4) (Grg-4) | 1.2875E-05 | n/a | n/a | B |
| Q9CS00 | CS029_MOUSE | 772 | 90662 | 9.8 | (Q9CS00) Uncharacterized protein C19orf29 homolog | 1.2774E-05 | n/a | n/a | B |
| O55106 | STRN_MOUSE | 780 | 86014 | 5.3 | (O55106) Striatin | 1.2643E-05 | n/a | n/a | B |
| Q91WG7 | DGKG_MOUSE | 788 | 88523 | 6.8 | (Q91WG7) Diacylglycerol kinase gamma (EC 2.7.1.107) (Diglyceride kinase gamma) (DGK-gamma) (DAG kinase gamma) (88 kDa diacylglycerol kinase) | 1.2515E-05 | n/a | n/a | B |
| Q80Y81 | RNZ2_MOUSE | 831 | 92719 | 7.3 | (Q80Y81) Zinc phosphodiesterase ELAC protein 2 (EC 3.1.26.11) (Ribonuclease Z 2) (RNase Z 2) (tRNase Z 2) (tRNA 3 endonuclease 2) (ElaC homolog protein 2) | 1.1868E-05 | n/a | n/a | B |
| Q8BPM2 | M4K5_MOUSE | 847 | 95045 | 7.8 | (Q8BPM2) Mitogen-activated protein kinase kinase kinase kinase 5 (EC 2.7.11.1) (MAPK/ERK kinase kinase kinase 5) (MEK kinase kinase 5) (MEKKK 5) | 1.1643E-05 | n/a | n/a | B |
| Q6GQX6 | ANKS6_MOUSE | 883 | 93474 | 7.6 | (Q6GQX6) Ankyrin repeat and SAM domain-containing protein 6 (Sterile alpha motif domain-containing protein 6) | 1.1169E-05 | n/a | n/a | B |
| Q99104 | MYO5A_MOUSE | 1853 | 215593 | 8.7 | (Q99104) Myosin-5A (Myosin Va) (Dilute myosin heavy chain, non-muscle) | 1.0504E-05 | n/a | n/a | B |
| Q9Z1Z0 | VDP_MOUSE | 959 | 106983 | 4.9 | (Q9Z1Z0) General vesicular transport factor p115 (Transcytosis-associated protein) (TAP) (Vesicle docking protein) | 1.0284E-05 | n/a | n/a | B |

| | | | | | | | | | |
|---|---|---|---|---|---|---|---|---|---|
| O54782 | MA2B2_MOUSE | 1018 | 115609 | 7.4 | (O54782) Epididymis-specific alpha-mannosidase precursor (EC 3.2.1.24) (Mannosidase alpha class 2B member 2) | 9.6875E-06 | n/a | n/a | B |
| Q8BLK9 | KS6C1_MOUSE | 1056 | 115712 | 4.9 | (Q8BLK9) Ribosomal protein S6 kinase delta-1 (EC 2.7.11.1) (52 kDa ribosomal protein S6 kinase) | 9.3389E-06 | n/a | n/a | B |
| O08523 | TECTA_MOUSE | 2155 | 239534 | 5.4 | (O08523) Alpha-tectorin precursor | 9.0316E-06 | n/a | n/a | B |
| Q9EPU4 | CPSF1_MOUSE | 1441 | 160818 | 6.4 | (Q9EPU4) Cleavage and polyadenylation specificity factor, 160 kDa subunit (CPSF 160 kDa subunit) | 6.8438E-06 | n/a | n/a | B |
| Q9EPN1 | NBEA_MOUSE | 2936 | 326742 | 6.2 | (Q9EPN1) Protein neurobeachin (Lysosomal-trafficking regulator 2) | 6.6291E-06 | n/a | n/a | B |
| Q64430 | ATP7A_MOUSE | 1491 | 161909 | 6.7 | (Q64430) Copper-transporting ATPase 1 (EC 3.6.3.4) (Copper pump 1) (Menkes disease-associated protein homolog) | 6.6143E-06 | n/a | n/a | B |
| Q8R420 | ABCA3_MOUSE | 1704 | 192000 | 7.1 | (Q8R420) ATP-binding cassette sub-family A member 3 | 5.7875E-06 | n/a | n/a | B |
| Q9JLC8 | SACS_MOUSE | 3830 | 436758 | 7.1 | (Q9JLC8) Sacsin | 5.0818E-06 | n/a | n/a | B |
| Q61043 | NIN_MOUSE | 2035 | 234772 | 5 | (Q61043) Ninein | 4.8461E-06 | n/a | n/a | B |
| P61514 | RL37A_MOUSE | 91 | 10144 | 10.4 | (P61514) 60S ribosomal protein L37a | 0.00076973 | n/a | n/a | C |
| Q9CQF0 | RM11_MOUSE | 192 | 20680 | 9.7 | (Q9CQF0) 39S ribosomal protein L11, mitochondrial precursor (L11mt) (MRP-L11) | 0.00036482 | n/a | n/a | C |
| Q6ZWY3 | RS27L_MOUSE | 83 | 9346 | 9.5 | (Q6ZWY3) 40S ribosomal protein S27-like protein | 0.00033757 | n/a | n/a | C |
| Q9JHS3 | MAPIP_MOUSE | 125 | 13480 | 5.4 | (Q9JHS3) Mitogen-activated protein-binding protein-interacting protein (Late endosomal/lysosomal Mp1-interacting protein) (p14) | 0.00033622 | n/a | n/a | C |
| Q8R5C5 | ACTY_MOUSE | 376 | 42281 | 6.4 | (Q8R5C5) Beta-centractin (Actin-related protein 1B) (ARP1B) | 0.00029807 | n/a | n/a | C |
| P51859 | HDGF_MOUSE | 237 | 26269 | 4.8 | (P51859) Hepatoma-derived growth factor (HDGF) | 0.00029555 | n/a | n/a | C |
| Q9CQ92 | FIS1_MOUSE | 152 | 17009 | 8.5 | (Q9CQ92) Mitochondrial fission 1 protein (Fis1 homolog) (Tetratricopeptide repeat protein 11) (TPR repeat protein 11) | 0.0002765 | n/a | n/a | C |
| P58059 | RT21_MOUSE | 87 | 10561 | 10.3 | (P58059) Mitochondrial 28S ribosomal protein S21 (S21mt) (MRP-S21) | 0.00024154 | n/a | n/a | C |
| P70349 | HINT1_MOUSE | 125 | 13646 | 6.9 | (P70349) Histidine triad nucleotide-binding protein 1 (Adenosine 5'-monophosphoramidase) (Protein kinase C inhibitor 1) (Protein kinase C-interacting protein 1) (PKCI-1) | 0.00022415 | n/a | n/a | C |
| Q99JT2 | MST4_MOUSE | 416 | 46614 | 5.2 | (Q99JT2) Serine/threonine-protein kinase MST4 (EC 2.7.11.1) (STE20-like kinase MST4) (MST-4) (Mammalian STE20-like protein kinase 4) | 0.00021889 | n/a | n/a | C |
| P97352 | S10AD_MOUSE | 98 | 11158 | 6.2 | (P97352) Protein S100-A13 (S100 calcium-binding protein A13) | 0.00021443 | n/a | n/a | C |
| Q8BIG7 | CMTD1_MOUSE | 262 | 28961 | 8.3 | (Q8BIG7) Catechol-O-methyltransferase domain-containing protein 1 (EC 2.1.1.-) | 0.00021388 | n/a | n/a | C |
| O35381 | AN32A_MOUSE | 247 | 28538 | 4.1 | (O35381) Acidic leucine-rich nuclear phosphoprotein 32 family member A (Potent heat-stable protein phosphatase 2A inhibitor I1PP2A) (Acidic nuclear phosphoprotein pp32) (Leucine-rich acidic nuclear protein) | 0.00019851 | n/a | n/a | C |
| P56812 | PDCD5_MOUSE | 125 | 14144 | 5.7 | (P56812) Programmed cell death protein 5 (Protein TFAR19) (TF-1 cell apoptosis-related gene 19 protein) | 0.00016811 | n/a | n/a | C |
| Q9DC71 | RT15_MOUSE | 258 | 29464 | 10.1 | (Q9DC71) 28S ribosomal protein S15, mitochondrial precursor (S15mt) (MRP-S15) | 0.0001629 | n/a | n/a | C |
| P50171 | DHB8_MOUSE | 260 | 26645 | 6.5 | (P50171) Estradiol 17-beta-dehydrogenase 8 (EC 1.1.1.62) (17-beta-HSD 8) (17-beta-hydroxysteroid dehydrogenase 8) (Protein Ke6) (Ke-6) | 0.00016164 | n/a | n/a | C |
| Q9D1X0 | NOL3_MOUSE | 220 | 24568 | 4.1 | (Q9D1X0) Nucleolar protein 3 | 0.00015919 | n/a | n/a | C |

| | | | | | | | | | |
|---|---|---|---|---|---|---|---|---|---|
| Q9QUR6 | PPCE_MOUSE | 710 | 80752 | 5.7 | (Q9QUR6) Prolyl endopeptidase (EC 3.4.21.26) (Post-proline cleaving enzyme) (PE) | 0.00015785 | n/a | n/a | C |
| P58389 | PTPA_MOUSE | 323 | 36710 | 6.4 | (P58389) Serine/threonine-protein phosphatase 2A regulatory subunit B' (PP2A, subunit B', PR53 isoform) (Phosphotyrosyl phosphatase activator) (PTPA) | 0.0001518 | n/a | n/a | C |
| Q9D7X8 | CG024_MOUSE | 188 | 21166 | 5.7 | (Q9D7X8) Protein C7orf24 homolog | 0.00014903 | n/a | n/a | C |
| Q9D0G0 | RT30_MOUSE | 442 | 49939 | 9.4 | (Q9D0G0) Mitochondrial 28S ribosomal protein S30 (S30mt) (MRP-S30) | 0.00014263 | n/a | n/a | C |
| O70325 | GPX41_MOUSE | 197 | 22182 | 8.4 | (O70325) Phospholipid hydroperoxide glutathione peroxidase, mitochondrial precursor (EC 1.11.1.12) (PHGPx) (GPX-4) | 0.00014222 | n/a | n/a | C |
| P08905 | LYSCM_MOUSE | 148 | 16689 | 8.8 | (P08905) Lysozyme C type M precursor (EC 3.2.1.17) (1,4-beta-N-acetylmuramidase C) | 0.00014198 | n/a | n/a | C |
| Q9D2R8 | RT33_MOUSE | 106 | 12459 | 10.3 | (Q9D2R8) Mitochondrial 28S ribosomal protein S33 (S33mt) (MRP-S33) (Ganglioside-induced differentiation-associated-protein 3) | 0.00013216 | n/a | n/a | C |
| P24472 | GSTA4_MOUSE | 222 | 25564 | 7.4 | (P24472) Glutathione S-transferase 5.7 (EC 2.5.1.18) (GST 5.7) (GST class-alpha) (GST A4-4) (GSTA4-4) | 0.00012621 | n/a | n/a | C |
| P51807 | DYLT1_MOUSE | 113 | 12483 | 5.1 | (P51807) Dynein light chain Tctex-type 1 (T-complex testis-specific protein 1) (TCTEX-1) | 0.00012397 | n/a | n/a | C |
| P97823 | LYPA1_MOUSE | 230 | 24688 | 6.6 | (P97823) Acyl-protein thioesterase 1 (EC 3.1.2.-) (Lysophospholipase 1) (Lysophospholipase I) (LysoPLA I) | 0.00012182 | n/a | n/a | C |
| P04235 | CD3D_MOUSE | 173 | 19032 | 6.9 | (P04235) T-cell surface glycoprotein CD3 delta chain precursor (T-cell receptor T3 delta chain) | 0.00012147 | n/a | n/a | C |
| O55060 | TPMT_MOUSE | 240 | 27586 | 6.4 | (O55060) Thiopurine S-methyltransferase (EC 2.1.1.67) (Thiopurine methyltransferase) | 0.00011674 | n/a | n/a | C |
| Q9CWM4 | PFD1_MOUSE | 122 | 14255 | 8.3 | (Q9CWM4) Prefoldin subunit 1 | 0.00011483 | n/a | n/a | C |
| P15392 | CP2A4_MOUSE | 494 | 56595 | 9.1 | (P15392) Cytochrome P450 2A4 (EC 1.14.14.1) (CYPIIA4) (Testosterone 15-alpha-hydroxylase) (P450-15-alpha) (P450-IIA3.1) | 0.00011343 | n/a | n/a | C |
| Q9D0T1 | NHPX_MOUSE | 127 | 14042 | 8.5 | (Q9D0T1) NHP2-like protein 1 (High mobility group-like nuclear protein 2 homolog 1) (U4/U6.U5 tri-snRNP 15.5 kDa protein) (Sperm-specific antigen 1) (Fertilization antigen 1) (FA-1) | 0.00011031 | n/a | n/a | C |
| Q3TGF2 | F107B_MOUSE | 131 | 15572 | 8.3 | (Q3TGF2) Protein FAM107B | 0.00010694 | n/a | n/a | C |
| Q9DCD0 | 6PGD_MOUSE | 482 | 53116 | 7.2 | (Q9DCD0) 6-phosphogluconate dehydrogenase, decarboxylating (EC 1.1.1.44) | 0.00010173 | n/a | n/a | C |
| Q8CG50 | RAB43_MOUSE | 210 | 23263 | 5.9 | (Q8CG50) Ras-related protein Rab-43 | 0.00010007 | n/a | n/a | C |
| O55126 | NIPS2_MOUSE | 281 | 32933 | 9.3 | (O55126) Protein NipSnap2 (Glioblastoma amplified sequence) | 9.9709E-05 | n/a | n/a | C |
| P21107 | TPM3_MOUSE | 284 | 32863 | 4.7 | (P21107) Tropomyosin alpha-3 chain (Tropomyosin-3) (Tropomyosin gamma) | 9.8656E-05 | n/a | n/a | C |
| Q9Z2C8 | YBOX2_MOUSE | 360 | 38271 | 10.9 | (Q9Z2C8) Y-box-binding protein 2 (Germ cell-specific Y-box-binding protein) (FRGY2 homolog) | 9.7286E-05 | n/a | n/a | C |
| Q62376 | RU17_MOUSE | 448 | 51992 | 9.9 | (Q62376) U1 small nuclear ribonucleoprotein 70 kDa (U1 SNRNP 70 kDa) (snRNP70) | 9.3811E-05 | n/a | n/a | C |
| Q99JB7 | AMNLS_MOUSE | 458 | 48696 | 5.6 | (Q99JB7) Amnionless protein precursor | 9.1763E-05 | n/a | n/a | C |
| Q9ER69 | WTAP_MOUSE | 396 | 44219 | 5.2 | (Q9ER69) Wilms' tumor 1-associating protein (WT1-associated protein) (Putative pre-mRNA-splicing regulator female-lethal(2D) homolog) | 8.8442E-05 | n/a | n/a | C |

| | | | | | | | | | |
|---|---|---|---|---|---|---|---|---|---|
| Q9QZA0 | CAH5B_MOUSE | 317 | 36623 | 7.3 | (Q9QZA0) Carbonic anhydrase 5B, mitochondrial precursor (EC 4.2.1.1) (Carbonic anhydrase VB) (Carbonate dehydratase VB) (CA-VB) | 8.8386E-05 | n/a | n/a | C |
| Q922Q4 | P5CR2_MOUSE | 320 | 33659 | 7.8 | (Q922Q4) Pyrroline-5-carboxylate reductase 2 (EC 1.5.1.2) (P5CR 2) (P5C reductase 2) | 8.7557E-05 | n/a | n/a | C |
| Q8CFA2 | GCST_MOUSE | 403 | 44009 | 8.7 | (Q8CFA2) Aminomethyltransferase, mitochondrial precursor (EC 2.1.2.10) (Glycine cleavage system T protein) (GCVT) | 8.6905E-05 | n/a | n/a | C |
| P62077 | TIM8B_MOUSE | 83 | 9286 | 5.1 | (P62077) Mitochondrial import inner membrane translocase subunit Tim8 B (Deafness dystonia protein 2 homolog) | 8.4392E-05 | n/a | n/a | C |
| Q9DAT5 | TRMU_MOUSE | 417 | 47240 | 8.4 | (Q9DAT5) tRNA (5-methylaminomethyl-2-thiouridylate)-methyltransferase (EC 2.1.1.61) | 8.3988E-05 | n/a | n/a | C |
| P05627 | JUN_MOUSE | 334 | 35944 | 8.7 | (P05627) Transcription factor AP-1 (Activator protein 1) (AP1) (Proto-oncogene c-jun) (V-jun avian sarcoma virus 17 oncogene homolog) (Jun A) (AH119) | 8.3887E-05 | n/a | n/a | C |
| O09164 | SODE_MOUSE | 251 | 27392 | 6.8 | (O09164) Extracellular superoxide dismutase [Cu-Zn] precursor (EC 1.15.1.1) (EC-SOD) | 8.372E-05 | n/a | n/a | C |
| Q63810 | CANB1_MOUSE | 169 | 19142 | 4.8 | (Q63810) Calcineurin subunit B isoform 1 (Protein phosphatase 2B regulatory subunit 1) (Protein phosphatase 3 regulatory subunit B alpha isoform 1) | 8.2894E-05 | n/a | n/a | C |
| Q9CZ28 | SNF8_MOUSE | 258 | 28886 | 6.7 | (Q9CZ28) Vacuolar sorting protein SNF8 | 8.1448E-05 | n/a | n/a | C |
| Q922B9 | SSFA2_MOUSE | 264 | 28729 | 4.9 | (Q922B9) Sperm-specific antigen 2 | 7.9597E-05 | n/a | n/a | C |
| Q8K3J9 | GPC5C_MOUSE | 440 | 48422 | 7.6 | (Q8K3J9) G-protein coupled receptor family C group 5 member C precursor (Retinoic acid-induced gene 3 protein) (RAIG-3) | 7.9597E-05 | n/a | n/a | C |
| P04939 | MUP3_MOUSE | 184 | 21465 | 4.8 | (P04939) Major urinary protein 3 precursor (MUP 3) (Non-group 1/group 2 MUP15) | 7.6137E-05 | n/a | n/a | C |
| Q6PAM1 | TXLNA_MOUSE | 554 | 62369 | 6.7 | (Q6PAM1) Alpha-taxilin | 7.5862E-05 | n/a | n/a | C |
| O09114 | PTGDS_MOUSE | 189 | 21066 | 8.3 | (O09114) Prostaglandin-H2 D-isomerase precursor (EC 5.3.99.2) (Lipocalin-type prostaglandin-D synthase) (Glutathione-independent PGD synthetase) (Prostaglandin-D2 synthase) (PGD2 synthase) (PGDS2) (PGDS) | 7.4122E-05 | n/a | n/a | C |
| O35900 | LSM2_MOUSE | 95 | 10835 | 6.5 | (O35900) U6 snRNA-associated Sm-like protein LSm2 (snRNP core Sm-like protein Sm-x5) (Protein G7b) | 7.3732E-05 | n/a | n/a | C |
| O88952 | LIN7C_MOUSE | 197 | 21834 | 8.4 | (O88952) LIN-7 homolog C (LIN-7C) (mLin7C) (Mammalian LIN-seven protein 3) (MALS-3) (Vertebrate LIN 7 homolog 3) (Veli-3 protein) | 7.1112E-05 | n/a | n/a | C |
| Q80YV2 | NIPA_MOUSE | 501 | 55196 | 5.4 | (Q80YV2) Nuclear-interacting partner of ALK (Nuclear-interacting partner of anaplastic lymphoma kinase) (mNIPA) (Zinc finger C3HC-type protein 1) | 6.9906E-05 | n/a | n/a | C |
| Q9D6K9 | LASS5_MOUSE | 414 | 48167 | 8.1 | (Q9D6K9) LAG1 longevity assurance homolog 5 (Translocating chain-associating membrane protein homolog 4) (TRAM homolog 4) | 6.7677E-05 | n/a | n/a | C |
| Q9DBE8 | ALG2_MOUSE | 415 | 47405 | 8 | (Q9DBE8) Alpha-1,3-mannosyltransferase ALG2 (EC 2.4.1.-) (GDP-Man:Man(1)GlcNAc(2)-PP-dolichol mannosyltransferase) | 6.7514E-05 | n/a | n/a | C |
| Q8R3P0 | ACY2_MOUSE | 312 | 35345 | 6.6 | (Q8R3P0) Aspartoacylase (EC 3.5.1.15) (Aminoacylase-2) (ACY-2) | 6.7352E-05 | n/a | n/a | C |
| P35292 | RAB17_MOUSE | 214 | 23640 | 5.4 | (P35292) Ras-related protein Rab-17 | 6.5463E-05 | n/a | n/a | C |
| Q9D287 | BCAS2_MOUSE | 225 | 26131 | 5.6 | (Q9D287) Breast carcinoma amplified sequence 2 homolog (DNA amplified in mammary carcinoma 1 protein) | 6.2263E-05 | n/a | n/a | C |

| | | | | | | | | | |
|---|---|---|---|---|---|---|---|---|---|
| O09131 | GSTO1_MOUSE | 240 | 27498 | 7.4 | (O09131) Glutathione transferase omega-1 (EC 2.5.1.18) (GSTO 1-1) (p28) | 5.8371E-05 | n/a | n/a | C |
| Q9QXJ4 | ARL10_MOUSE | 243 | 27361 | 4.8 | (Q9QXJ4) ADP-ribosylation factor-like protein 10 (ADP-ribosylation factor-like membrane-associated protein) | 5.7651E-05 | n/a | n/a | C |
| P32233 | DRG1_MOUSE | 367 | 40512 | 8.9 | (P32233) Developmentally-regulated GTP-binding protein 1 (DRG 1) (Protein NEDD3) (Neural precursor cell expressed developmentally down-regulated protein 3) | 5.7258E-05 | n/a | n/a | C |
| Q61767 | 3BHS4_MOUSE | 372 | 41635 | 8.6 | (Q61767) 3 beta-hydroxysteroid dehydrogenase type 4 (3 beta-hydroxysteroid dehydrogenase type IV) (3Beta-HSD IV) (NADPH dependent 3-beta-hydroxy-delta(5)-steroid dehydrogenase) (EC 1.1.1.-) (3-beta-hydroxy-5-ene steroid dehydrogenase) (Proges | 5.6488E-05 | n/a | n/a | C |
| Q60676 | PPP5_MOUSE | 499 | 56847 | 6.2 | (Q60676) Serine/threonine-protein phosphatase 5 (EC 3.1.3.16) (PP5) (Protein phosphatase T) (PPT) | 5.6149E-05 | n/a | n/a | C |
| O88351 | IKKB_MOUSE | 757 | 86690 | 6.4 | (O88351) Inhibitor of nuclear factor kappa B kinase subunit beta (EC 2.7.11.10) (I-kappa-B-kinase beta) (IkBKB) (IKK-beta) (IKK-B) (I-kappa-B kinase 2) (IKK2) (Nuclear factor NF-kappa-B inhibitor kinase beta) (NFKBIKB) | 5.5518E-05 | n/a | n/a | C |
| Q8BW75 | AOFB_MOUSE | 519 | 58413 | 8.3 | (Q8BW75) Amine oxidase [flavin-containing] B (EC 1.4.3.4) (Monoamine oxidase type B) (MAO-B) | 5.3985E-05 | n/a | n/a | C |
| Q99LX5 | MMTA2_MOUSE | 260 | 29299 | 9.9 | (Q99LX5) Multiple myeloma tumor-associated protein 2 homolog | 5.3881E-05 | n/a | n/a | C |
| Q924D0 | RT4I1_MOUSE | 396 | 43371 | 9.2 | (Q924D0) Reticulon-4-interacting protein 1, mitochondrial precursor (NOGO-interacting mitochondrial protein) | 5.3065E-05 | n/a | n/a | C |
| P13864 | DNMT1_MOUSE | 1620 | 183188 | 7.7 | (P13864) DNA (cytosine-5)-methyltransferase 1 (EC 2.1.1.37) (Dnmt1) (DNA methyltransferase MmuI) (DNA MTase MmuI) (MCMT) (M.MmuI) (Met-1) | 5.1886E-05 | n/a | n/a | C |
| Q9Z266 | S25BP_MOUSE | 136 | 14904 | 9.3 | (Q9Z266) SNARE-associated protein Snapin (Synaptosomal-associated protein 25-binding protein) (SNAP-associated protein) | 5.1504E-05 | n/a | n/a | C |
| Q9QXA5 | LSM4_MOUSE | 137 | 15076 | 10.1 | (Q9QXA5) U6 snRNA-associated Sm-like protein LSm4 | 5.1128E-05 | n/a | n/a | C |
| Q8VEB4 | LYPA3_MOUSE | 412 | 47307 | 6.5 | (Q8VEB4) 1-O-acylceramide synthase precursor (EC 2.3.1.-) (ACS) (Lysosomal phospholipase A2) (Lysophospholipase 3) (LCAT-like lysophospholipase) (LLPL) | 5.1004E-05 | n/a | n/a | C |
| Q924M7 | MANA_MOUSE | 423 | 46575 | 6 | (Q924M7) Mannose-6-phosphate isomerase (EC 5.3.1.8) (Phosphomannose isomerase) (PMI) (Phosphohexomutase) | 4.9678E-05 | n/a | n/a | C |
| Q99N92 | RM27_MOUSE | 148 | 15945 | 10.2 | (Q99N92) Mitochondrial 39S ribosomal protein L27 (L27mt) (MRP-L27) | 4.7328E-05 | n/a | n/a | C |
| Q80ZM8 | CRLS1_MOUSE | 303 | 32502 | 9.9 | (Q80ZM8) Cardiolipin synthetase (EC 2.7.8.-) (Cardiolipin synthase) (CLS) | 4.6235E-05 | n/a | n/a | C |
| Q64378 | FKBP5_MOUSE | 456 | 50966 | 7.8 | (Q64378) FK506-binding protein 5 (EC 5.2.1.8) (Peptidyl-prolyl cis-trans isomerase) (PPIase) (Rotamase) (51 kDa FK506-binding protein) (FKBP-51) | 4.6083E-05 | n/a | n/a | C |
| P59016 | VP33B_MOUSE | 617 | 70526 | 6.9 | (P59016) Vacuolar protein sorting 33B | 4.541E-05 | n/a | n/a | C |
| Q9CPX6 | ATG3_MOUSE | 314 | 35796 | 4.7 | (Q9CPX6) Autophagy-related protein 3 (APG3-like) | 4.4615E-05 | n/a | n/a | C |
| Q61655 | DD19A_MOUSE | 478 | 53889 | 6.7 | (Q61655) ATP-dependent RNA helicase DDX19A (EC 3.6.1.-) (DEAD box protein 19A) (DEAD box RNA helicase DEAD5) (mDEAD5) (Eukaryotic translation initiation factor 4A-related sequence 1) | 4.3962E-05 | n/a | n/a | C |

| | | | | | | | | | |
|---|---|---|---|---|---|---|---|---|---|
| P09470 | ACE_MOUSE | 1312 | 150918 | 6.6 | (P09470) Angiotensin-converting enzyme, somatic isoform precursor (EC 3.4.15.1) (Dipeptidyl carboxypeptidase I) (Kininase II) [Contains: Angiotensin-converting enzyme, somatic isoform, soluble form] | 4.2711E-05 | n/a | n/a | C |
| Q9ERN0 | SCAM2_MOUSE | 329 | 36465 | 6.3 | (Q9ERN0) Secretory carrier-associated membrane protein 2 (Secretory carrier membrane protein 2) | 4.2581E-05 | n/a | n/a | C |
| Q9D0W5 | PPIL1_MOUSE | 166 | 18237 | 8 | (Q9D0W5) Peptidyl-prolyl cis-trans isomerase-like 1 (EC 5.2.1.8) (PPIase) (Rotamase) | 4.2196E-05 | n/a | n/a | C |
| Q9QXD8 | LIMD1_MOUSE | 668 | 71422 | 6.3 | (Q9QXD8) LIM domains-containing protein 1 | 4.1944E-05 | n/a | n/a | C |
| Q9R1K9 | CETN2_MOUSE | 172 | 19796 | 5 | (Q9R1K9) Centrin-2 (Caltractin isoform 1) | 4.0724E-05 | n/a | n/a | C |
| Q99LD9 | EI2BB_MOUSE | 351 | 38898 | 6.2 | (Q99LD9) Translation initiation factor eIF-2B subunit beta (eIF-2B GDP-GTP exchange factor subunit beta) | 3.9912E-05 | n/a | n/a | C |
| Q99P31 | HPBP1_MOUSE | 357 | 39167 | 5.4 | (Q99P31) Hsp70-binding protein 1 (HspBP1) (Heat shock protein-binding protein 1) (Hsp70-interacting protein 1) | 3.9241E-05 | n/a | n/a | C |
| Q9CYK1 | SYWM_MOUSE | 360 | 40167 | 8.8 | (Q9CYK1) Tryptophanyl-tRNA synthetase, mitochondrial precursor (EC 6.1.1.2) (Tryptophan--tRNA ligase) (TrpRS) ((Mt)TrpRS) | 3.8914E-05 | n/a | n/a | C |
| P22907 | HEM3_MOUSE | 361 | 39302 | 7.1 | (P22907) Porphobilinogen deaminase (EC 2.5.1.61) (Hydroxymethylbilane synthase) (HMBS) (Pre-uroporphyrinogen synthase) (PBG-D) | 3.8806E-05 | n/a | n/a | C |
| Q9D5T0 | ATAD1_MOUSE | 361 | 40744 | 6.9 | (Q9D5T0) ATPase family AAA domain-containing protein 1 | 3.8806E-05 | n/a | n/a | C |
| P05063 | ALDOC_MOUSE | 362 | 39264 | 7.1 | (P05063) Fructose-bisphosphate aldolase C (EC 4.1.2.13) (Brain type aldolase) (Aldolase 3) (Zebrin II) (Scrapie-responsive protein 2) | 3.8699E-05 | n/a | n/a | C |
| Q9ES64 | USH1C_MOUSE | 910 | 102285 | 7.1 | (Q9ES64) Harmonin (Usher syndrome type-1C protein homolog) (PDZ domain-containing protein) | 3.8487E-05 | n/a | n/a | C |
| P83917 | CBX1_MOUSE | 185 | 21418 | 4.9 | (P83917) Chromobox protein homolog 1 (Heterochromatin protein 1 homolog beta) (HP1 beta) (Modifier 1 protein) (M31) (Heterochromatin protein p25) | 3.7863E-05 | n/a | n/a | C |
| Q99K85 | SERC_MOUSE | 370 | 40473 | 8 | (Q99K85) Phosphoserine aminotransferase (EC 2.6.1.52) (PSAT) (Endometrial progesterone-induced protein) (EPIP) | 3.7863E-05 | n/a | n/a | C |
| Q9QYG0 | NDRG2_MOUSE | 371 | 40789 | 5.4 | (Q9QYG0) Protein NDRG2 (Protein Ndr2) | 3.776E-05 | n/a | n/a | C |
| P51655 | GPC4_MOUSE | 557 | 62586 | 6.3 | (P51655) Glypican-4 precursor (K-glypican) | 3.7727E-05 | n/a | n/a | C |
| Q9WTS2 | FUT8_MOUSE | 575 | 66556 | 7.5 | (Q9WTS2) Alpha-(1,6)-fucosyltransferase (EC 2.4.1.68) (Glycoprotein 6-alpha-L-fucosyltransferase) (GDP-fucose--glycoprotein fucosyltransferase) (GDP-L-Fuc:N-acetyl-beta-D-glucosaminide alpha1,6-fucosyltransferase) (alpha1-6FucT) (Fucosyltransf | 3.6546E-05 | n/a | n/a | C |
| Q9D7N3 | RT09_MOUSE | 390 | 44886 | 8.7 | (Q9D7N3) 28S ribosomal protein S9, mitochondrial precursor (S9mt) (MRP-S9) | 3.5921E-05 | n/a | n/a | C |
| Q8BWW9 | PKN2_MOUSE | 983 | 111630 | 6.4 | (Q8BWW9) Serine/threonine-protein kinase N2 (EC 2.7.11.13) (Protein kinase C-like 2) (Protein-kinase C-related kinase 2) | 3.5629E-05 | n/a | n/a | C |
| Q8R0A5 | TCAL3_MOUSE | 200 | 22468 | 5.4 | (Q8R0A5) Transcription elongation factor A protein-like 3 (TCEA-like protein 3) (Transcription elongation factor S-II protein-like 3) | 3.5023E-05 | n/a | n/a | C |
| Q9R1P3 | PSB2_MOUSE | 201 | 22906 | 7 | (Q9R1P3) Proteasome subunit beta type 2 (EC 3.4.25.1) (Proteasome component C7-I) (Macropain subunit C7-I) (Multicatalytic endopeptidase complex subunit C7-I) | 3.4849E-05 | n/a | n/a | C |

| | | | | | | | | | |
|---|---|---|---|---|---|---|---|---|---|
| Q6R891 | NEB2_MOUSE | 817 | 89520 | 4.9 | (Q6R891) Neurabin-2 (Neurabin-II) (Spinophilin) (Protein phosphatase 1 regulatory subunit 9B) | 3.4294E-05 | n/a | n/a | C |
| P63321 | RALA_MOUSE | 206 | 23553 | 7.1 | (P63321) Ras-related protein Ral-A | 3.4003E-05 | n/a | n/a | C |
| Q7TQH0 | ATX2L_MOUSE | 1049 | 110649 | 8.9 | (Q7TQH0) Ataxin-2-like protein | 3.3387E-05 | n/a | n/a | C |
| Q99JB8 | PACN3_MOUSE | 424 | 48585 | 6.1 | (Q99JB8) Protein kinase C and casein kinase II substrate protein 3 | 3.304E-05 | n/a | n/a | C |
| Q99N87 | RT05_MOUSE | 432 | 48207 | 10.1 | (Q99N87) Mitochondrial 28S ribosomal protein S5 (S5mt) (MRP-S5) | 3.2429E-05 | n/a | n/a | C |
| Q8BYW1 | RHG25_MOUSE | 648 | 73383 | 6.4 | (Q8BYW1) Rho-GTPase-activating protein 25 | 3.2429E-05 | n/a | n/a | C |
| O55201 | SPT5H_MOUSE | 1082 | 120664 | 5 | (O55201) Transcription elongation factor SPT5 (DRB sensitivity-inducing factor large subunit) (DSIF large subunit) | 3.2369E-05 | n/a | n/a | C |
| Q9D8L5 | CCD91_MOUSE | 442 | 50007 | 5.1 | (Q9D8L5) Coiled-coil domain-containing protein 91 (GGA-binding partner) | 3.1695E-05 | n/a | n/a | C |
| Q9CR00 | PSD9_MOUSE | 222 | 24720 | 6.4 | (Q9CR00) 26S proteasome non-ATPase regulatory subunit 9 (26S proteasome regulatory subunit p27) | 3.1552E-05 | n/a | n/a | C |
| Q8K1Y2 | KPCD3_MOUSE | 889 | 100078 | 6.8 | (Q8K1Y2) Serine/threonine-protein kinase D3 (EC 2.7.11.13) (Protein kinase C nu type) (nPKC-nu) | 3.1517E-05 | n/a | n/a | C |
| Q8JZL3 | THTPA_MOUSE | 223 | 24133 | 4.7 | (Q8JZL3) Thiamine-triphosphatase (EC 3.6.1.28) (ThTPase) | 3.1411E-05 | n/a | n/a | C |
| Q60649 | CLPB_MOUSE | 677 | 76004 | 8.5 | (Q60649) Caseinolytic peptidase B protein homolog (Suppressor of potassium transport defect 3) | 3.1039E-05 | n/a | n/a | C |
| Q9QZR0 | RNF25_MOUSE | 456 | 51227 | 6.3 | (Q9QZR0) RING finger protein 25 (EC 6.3.2.-) (RING finger protein AO7) | 3.0722E-05 | n/a | n/a | C |
| O35963 | RB33B_MOUSE | 229 | 25767 | 7.7 | (O35963) Ras-related protein Rab-33B | 3.0588E-05 | n/a | n/a | C |
| Q61206 | PA1B2_MOUSE | 229 | 25492 | 6.2 | (Q61206) Platelet-activating factor acetylhydrolase IB subunit beta (EC 3.1.1.47) (PAF acetylhydrolase 30 kDa subunit) (PAF-AH 30 kDa subunit) (PAF-AH subunit beta) (PAFAH subunit beta) | 3.0588E-05 | n/a | n/a | C |
| Q9EQH2 | ARTS1_MOUSE | 930 | 106599 | 6.1 | (Q9EQH2) Adipocyte-derived leucine aminopeptidase precursor (EC 3.4.11.-) (A-LAP) (ARTS-1) (Aminopeptidase PILS) (Puromycin-insensitive leucyl-specific aminopeptidase) (PILS-AP) (VEGF-induced aminopeptidase) | 3.0127E-05 | n/a | n/a | C |
| P28658 | ATX10_MOUSE | 475 | 53707 | 5.2 | (P28658) Ataxin-10 (Spinocerebellar ataxia type 10 protein homolog) (Brain protein E46) | 2.9493E-05 | n/a | n/a | C |
| P28661 | SEPT4_MOUSE | 478 | 54936 | 5.9 | (P28661) Septin-4 (Peanut-like protein 2) (Brain protein H5) | 2.9308E-05 | n/a | n/a | C |
| Q9QY76 | VAPB_MOUSE | 242 | 26815 | 7.8 | (Q9QY76) Vesicle-associated membrane protein-associated protein B (VAMP-associated protein B) (VAMP-associated protein 33b) (VAMP-B) (VAP-B) | 2.8944E-05 | n/a | n/a | C |
| Q8K0T4 | KATL1_MOUSE | 488 | 55165 | 7.1 | (Q8K0T4) Katanin p60 ATPase-containing subunit A-like 1 (EC 3.6.4.3) (Katanin p60 subunit A-like 1) (p60 katanin-like 1) | 2.8707E-05 | n/a | n/a | C |
| Q9EP71 | RAI14_MOUSE | 979 | 108852 | 6.3 | (Q9EP71) Ankycorbin (Ankyrin repeat and coiled-coil structure-containing protein) (Retinoic acid-induced protein 14) (Novel retinal pigment epithelial cell protein) (p125) | 2.8619E-05 | n/a | n/a | C |
| P56656 | CP239_MOUSE | 490 | 55903 | 7.9 | (P56656) Cytochrome P450 2C39 (EC 1.14.14.1) (CYPIIC39) | 2.859E-05 | n/a | n/a | C |
| Q62283 | TSN7_MOUSE | 249 | 27544 | 7.2 | (Q62283) Tetraspanin-7 (Tspan-7) (Transmembrane 4 superfamily member 2) (Cell surface glycoprotein A15) (PE31) (TALLA homolog) (CD231 antigen) | 2.8131E-05 | n/a | n/a | C |

| | | | | | | | | | |
|---|---|---|---|---|---|---|---|---|---|
| Q8C180 | FRS2_MOUSE | 507 | 56663 | 6.2 | (Q8C180) Fibroblast growth factor receptor substrate 2 (FGFR substrate 2) (Suc1-associated neurotrophic factor target 1) (SNT-1) (FGFR signaling adaptor SNT) (FRS2 alpha) | 2.7631E-05 | n/a | n/a | C |
| Q04750 | TOP1_MOUSE | 767 | 90790 | 9.3 | (Q04750) DNA topoisomerase 1 (EC 5.99.1.2) (DNA topoisomerase I) | 2.7397E-05 | n/a | n/a | C |
| Q9JLT4 | TRXR2_MOUSE | 524 | 56556 | 8.4 | (Q9JLT4) Thioredoxin reductase 2, mitochondrial precursor (EC 1.8.1.9) (TR3) | 2.6735E-05 | n/a | n/a | C |
| Q91Z31 | PTBP2_MOUSE | 531 | 57489 | 8.7 | (Q91Z31) Polypyrimidine tract-binding protein 2 (Brain-enriched polypyrimidine tract-binding protein) (Brain-enriched PTB) (RRM-type RNA-binding protein brPTB) (Neural polypyrimidine tract-binding protein) | 2.6383E-05 | n/a | n/a | C |
| Q6A068 | CDC5L_MOUSE | 802 | 92190 | 8 | (Q6A068) Cell division cycle 5-related protein (Cdc5-like protein) | 2.6202E-05 | n/a | n/a | C |
| O09000 | NCOA3_MOUSE | 1398 | 151573 | 7.8 | (O09000) Nuclear receptor coactivator 3 (EC 2.3.1.48) (NCoA-3) (Thyroid hormone receptor activator molecule 1) (TRAM-1) (ACTR) (Receptor-associated coactivator 3) (RAC-3) (Amplified in breast cancer-1 protein homolog) (AIB-1) (Steroid recepto | 2.5052E-05 | n/a | n/a | C |
| P97714 | ADA1D_MOUSE | 562 | 59858 | 9.8 | (P97714) Alpha-1D adrenergic receptor (Alpha 1D-adrenoceptor) (Alpha 1D-adrenoreceptor) (Alpha-1A adrenergic receptor) | 2.4927E-05 | n/a | n/a | C |
| Q9JHK4 | PGTA_MOUSE | 567 | 64990 | 5.8 | (Q9JHK4) Geranylgeranyl transferase type-2 alpha subunit (EC 2.5.1.60) (Geranylgeranyl transferase type II alpha subunit) (Rab geranylgeranyltransferase alpha subunit) (Rab geranyl-geranyltransferase alpha subunit) (Rab GG transferase alpha) ( | 2.4707E-05 | n/a | n/a | C |
| P39054 | DYN2_MOUSE | 870 | 98145 | 7.4 | (P39054) Dynamin-2 (EC 3.6.5.5) (Dynamin UDNM) | 2.4154E-05 | n/a | n/a | C |
| Q9Z0U1 | ZO2_MOUSE | 1167 | 131280 | 6.8 | (Q9Z0U1) Tight junction protein ZO-2 (Zonula occludens 2 protein) (Zona occludens 2 protein) (Tight junction protein 2) | 2.4009E-05 | n/a | n/a | C |
| Q9Z0E6 | GBP2_MOUSE | 589 | 66740 | 5.7 | (Q9Z0E6) Interferon-induced guanylate-binding protein 2 (GTP-binding protein 2) (Guanine nucleotide-binding protein 2) (mGBP2) (mGBP-2) | 2.3785E-05 | n/a | n/a | C |
| O35075 | DSCR3_MOUSE | 297 | 32970 | 7.7 | (O35075) Down syndrome critical region protein 3 homolog (Down syndrome critical region protein A homolog) | 2.3584E-05 | n/a | n/a | C |
| Q9CRD2 | T103_MOUSE | 297 | 34935 | 6.8 | (Q9CRD2) Tetratricopeptide repeat protein KIAA0103 | 2.3584E-05 | n/a | n/a | C |
| Q9EQ06 | DHRS8_MOUSE | 298 | 32881 | 8.7 | (Q9EQ06) Dehydrogenase/reductase SDR family member 8 precursor (EC 1.1.1.-) (17-beta-hydroxysteroid dehydrogenase 11) (17-beta-HSD 11) (17-beta-HSD XI) (17betaHSDXI) (17bHSD11) (17betaHSD11) | 2.3505E-05 | n/a | n/a | C |
| Q8BMF3 | MAON_MOUSE | 604 | 67180 | 7.7 | (Q8BMF3) NADP-dependent malic enzyme, mitochondrial precursor (EC 1.1.1.40) (NADP-ME) (Malic enzyme 3) | 2.3194E-05 | n/a | n/a | C |
| Q3UZB0 | ARMX5_MOUSE | 606 | 67896 | 9 | (Q3UZB0) Armadillo repeat-containing X-linked protein 5 | 2.3117E-05 | n/a | n/a | C |
| Q9D4H1 | EXOC2_MOUSE | 924 | 103959 | 7.2 | (Q9D4H1) Exocyst complex component 2 (Exocyst complex component Sec5) | 2.2742E-05 | n/a | n/a | C |
| Q99K51 | PLST_MOUSE | 627 | 70367 | 5.8 | (Q99K51) Plastin-3 (T-plastin) | 2.2343E-05 | n/a | n/a | C |
| Q91YR7 | PRP6_MOUSE | 941 | 106722 | 8.1 | (Q91YR7) Pre-mRNA-processing factor 6 homolog (U5 snRNP-associated 102 kDa protein) (U5-102 kDa protein) | 2.2331E-05 | n/a | n/a | C |
| Q80WW9 | CT116_MOUSE | 315 | 35977 | 5.3 | (Q80WW9) Uncharacterized protein C20orf116 homolog precursor | 2.2237E-05 | n/a | n/a | C |

| | | | | | | | | | |
|---|---|---|---|---|---|---|---|---|---|
| Q9WVA3 | BUB3_MOUSE | 326 | 36985 | 6.7 | (Q9WVA3) Mitotic checkpoint protein BUB3 (WD repeat type I transmembrane protein A72.5) | 2.1486E-05 | n/a | n/a | C |
| Q91VB4 | HPS3_MOUSE | 1002 | 113152 | 5.6 | (Q91VB4) Hermansky-Pudlak syndrome 3 protein homolog (Cocoa protein) | 2.0972E-05 | n/a | n/a | C |
| Q8K0U4 | HS12A_MOUSE | 675 | 74871 | 6.8 | (Q8K0U4) Heat shock 70 kDa protein 12A | 2.0754E-05 | n/a | n/a | C |
| P62874 | GBB1_MOUSE | 339 | 37246 | 6 | (P62874) Guanine nucleotide-binding protein G(I)/G(S)/G(T) subunit beta 1 (Transducin beta chain 1) | 2.0662E-05 | n/a | n/a | C |
| P43406 | ITAV_MOUSE | 1044 | 115278 | 5.7 | (P43406) Integrin alpha-V precursor (Vitronectin receptor subunit alpha) (CD51 antigen) [Contains: Integrin alpha-V heavy chain; Integrin alpha-V light chain] | 2.0128E-05 | n/a | n/a | C |
| Q8VDY9 | CI082_MOUSE | 356 | 37823 | 4.7 | (Q8VDY9) Protein C9orf82 homolog | 1.9676E-05 | n/a | n/a | C |
| Q8K4B0 | MTA1_MOUSE | 715 | 80798 | 9.3 | (Q8K4B0) Metastasis-associated protein MTA1 | 1.9593E-05 | n/a | n/a | C |
| P28740 | KIF2_MOUSE | 716 | 80946 | 6.3 | (P28740) Kinesin-like protein KIF2 | 1.9566E-05 | n/a | n/a | C |
| Q8R574 | KPRB_MOUSE | 369 | 40881 | 7.2 | (Q8R574) Phosphoribosyl pyrophosphate synthetase-associated protein 2 (PRPP synthetase-associated protein 2) (41 kDa phosphoribosypyrophosphate synthetase-associated protein) (PAP41) | 1.8983E-05 | n/a | n/a | C |
| P15105 | GLNA_MOUSE | 372 | 42014 | 6.9 | (P15105) Glutamine synthetase (EC 6.3.1.2) (Glutamate--ammonia ligase) (GS) | 1.8829E-05 | n/a | n/a | C |
| Q99PM3 | TF2AA_MOUSE | 378 | 41614 | 4.5 | (Q99PM3) Transcription initiation factor IIA subunit 1 (General transcription factor IIA1) [Contains: Transcription initiation factor IIA alpha chain (TFIIA p35 subunit); Transcription initiation factor IIA beta chain (TFIIA p19 subunit)] | 1.8531E-05 | n/a | n/a | C |
| P28843 | DPP4_MOUSE | 760 | 87437 | 6.4 | (P28843) Dipeptidyl peptidase 4 (EC 3.4.14.5) (Dipeptidyl peptidase IV) (DPP IV) (T-cell activation antigen CD26) (Thymocyte-activating molecule) (THAM) [Contains: Dipeptidyl peptidase 4 membrane form (Dipeptidyl peptidase IV membrane form); D | 1.8433E-05 | n/a | n/a | C |
| Q9Z1G3 | VATC_MOUSE | 381 | 43729 | 6.8 | (Q9Z1G3) Vacuolar ATP synthase subunit C (EC 3.6.3.14) (V-ATPase C subunit) (Vacuolar proton pump C subunit) | 1.8385E-05 | n/a | n/a | C |
| Q8C1F5 | TTC16_MOUSE | 767 | 86945 | 8.7 | (Q8C1F5) Tetratricopeptide repeat protein 16 (TPR repeat protein 16) | 1.8265E-05 | n/a | n/a | C |
| Q8CGC4 | FA61B_MOUSE | 385 | 42340 | 9.6 | (Q8CGC4) Protein FAM61B | 1.8194E-05 | n/a | n/a | C |
| P47857 | K6PF_MOUSE | 779 | 85137 | 8 | (P47857) 6-phosphofructokinase, muscle type (EC 2.7.1.11) (Phosphofructokinase 1) (Phosphohexokinase) (Phosphofructo-1 kinase isozyme A) (PFK-A) (Phosphofructokinase-M) | 1.7983E-05 | n/a | n/a | C |
| P36993 | PP2CB_MOUSE | 390 | 42795 | 5.2 | (P36993) Protein phosphatase 2C isoform beta (EC 3.1.3.16) (PP2C-beta) (IA) (Protein phosphatase 1B) | 1.796E-05 | n/a | n/a | C |
| O35855 | BCAT2_MOUSE | 393 | 44127 | 8.3 | (O35855) Branched-chain-amino-acid aminotransferase, mitochondrial precursor (EC 2.6.1.42) (BCAT(m)) | 1.7823E-05 | n/a | n/a | C |
| Q7TT16 | IPMK_MOUSE | 396 | 44453 | 8.7 | (Q7TT16) Inositol polyphosphate multikinase (EC 2.7.1.151) (Inositol 1,3,4,6-tetrakisphosphate 5-kinase) | 1.7688E-05 | n/a | n/a | C |
| Q8BKC8 | PI4KB_MOUSE | 801 | 89958 | 6.5 | (Q8BKC8) Phosphatidylinositol 4-kinase beta (EC 2.7.1.67) (PtdIns 4-kinase beta) (PI4Kbeta) (PI4K-beta) | 1.749E-05 | n/a | n/a | C |
| Q63932 | MP2K2_MOUSE | 401 | 44436 | 7 | (Q63932) Dual specificity mitogen-activated protein kinase kinase 2 (EC 2.7.12.2) (MAP kinase kinase 2) (MAPKK 2) (ERK activator kinase 2) (MAPK/ERK kinase 2) (MEK2) | 1.7468E-05 | n/a | n/a | C |

| | | | | | | | | | |
|---|---|---|---|---|---|---|---|---|---|
| O35730 | RING1_MOUSE | 406 | 42631 | 5.7 | (O35730) Polycomb complex protein RING1 (RING finger protein 1) (Transcription repressor Ring1A) | 1.7253E-05 | n/a | n/a | C |
| Q9JLF6 | TGM1_MOUSE | 815 | 89826 | 6.5 | (Q9JLF6) Protein-glutamine gamma-glutamyltransferase K (EC 2.3.2.13) (Transglutaminase K) (TGase K) (TGK) (TG(K)) (Transglutaminase-1) (Epidermal TGase) | 1.7189E-05 | n/a | n/a | C |
| Q9DC28 | KC1D_MOUSE | 415 | 47316 | 9.7 | (Q9DC28) Casein kinase I isoform delta (EC 2.7.11.1) (CKI-delta) (CKId) | 1.6878E-05 | n/a | n/a | C |
| O09043 | NAPSA_MOUSE | 419 | 45544 | 7.5 | (O09043) Napsin-A precursor (EC 3.4.23.-) (Kidney-derived aspartic protease-like protein) (KDAP-1) (KAP) | 1.6717E-05 | n/a | n/a | C |
| Q91WS2 | NALP6_MOUSE | 843 | 94592 | 8.2 | (Q91WS2) NACHT, LRR and PYD-containing protein 6 (PYRIN-containing APAF1-like protein 5-like) | 1.6618E-05 | n/a | n/a | C |
| Q8K1J6 | TRNT1_MOUSE | 434 | 49895 | 8.6 | (Q8K1J6) tRNA-nucleotidyltransferase 1, mitochondrial precursor (EC 2.7.7.25) (mitochondrial tRNA nucleotidyl transferase, CCA-adding) (mt tRNA adenylyltransferase) (mt tRNA CCA-pyrophosphorylase) (mt tRNA CCA-diphosphorylase) (mt CCA-adding | 1.614E-05 | n/a | n/a | C |
| Q8BL74 | TF3C2_MOUSE | 907 | 100274 | 6.6 | (Q8BL74) General transcription factor 3C polypeptide 2 (Transcription factor IIIC-subunit beta) (TF3C-beta) (TFIIIC 110 kDa subunit) (TFIIIC110) | 1.5446E-05 | n/a | n/a | C |
| P60122 | RUVB1_MOUSE | 456 | 50214 | 6.4 | (P60122) RuvB-like 1 (EC 3.6.1.-) (49 kDa TATA box-binding protein-interacting protein) (49 kDa TBP-interacting protein) (TIP49a) (Pontin 52) (DNA helicase p50) | 1.5361E-05 | n/a | n/a | C |
| Q99KR8 | FUCO2_MOUSE | 461 | 53645 | 6.6 | (Q99KR8) Plasma alpha-L-fucosidase precursor (EC 3.2.1.51) (Alpha-L-fucosidase 2) (Alpha-L-fucoside fucohydrolase 2) | 1.5194E-05 | n/a | n/a | C |
| Q8BYL4 | SYYM_MOUSE | 472 | 52598 | 9.2 | (Q8BYL4) Probable tyrosyl-tRNA synthetase, mitochondrial precursor (EC 6.1.1.1) (Tyrosine--tRNA ligase) (TyrRS) | 1.484E-05 | n/a | n/a | C |
| P01873 | MUCM_MOUSE | 475 | 52527 | 6.4 | (P01873) Ig mu chain C region membrane-bound form | 1.4746E-05 | n/a | n/a | C |
| Q8K2V1 | PP4R1_MOUSE | 951 | 106298 | 4.8 | (Q8K2V1) Serine/threonine-protein phosphatase 4 regulatory subunit 1 | 1.4731E-05 | n/a | n/a | C |
| O08663 | AMPM2_MOUSE | 478 | 52922 | 5.8 | (O08663) Methionine aminopeptidase 2 (EC 3.4.11.18) (MetAP 2) (Peptidase M 2) (Initiation factor 2-associated 67 kDa glycoprotein) (p67) (p67eIF2) | 1.4654E-05 | n/a | n/a | C |
| P54822 | PUR8_MOUSE | 484 | 54808 | 7.3 | (P54822) Adenylosuccinate lyase (EC 4.3.2.2) (Adenylosuccinase) (ASL) (ASASE) | 1.4472E-05 | n/a | n/a | C |
| Q5SPW0 | VPS54_MOUSE | 977 | 110397 | 6.5 | (Q5SPW0) Vacuolar protein sorting protein 54 (Tumor antigen SLP-8p homolog) | 1.4339E-05 | n/a | n/a | C |
| O70310 | NMT1_MOUSE | 496 | 56888 | 8 | (O70310) Glycylpeptide N-tetradecanoyltransferase 1 (EC 2.3.1.97) (Peptide N-myristoyltransferase 1) (Myristoyl-CoA:protein N-myristoyltransferase 1) (NMT 1) (Type I N-myristoyltransferase) | 1.4122E-05 | n/a | n/a | C |
| P97386 | DNL3_MOUSE | 1015 | 113018 | 9 | (P97386) DNA ligase 3 (EC 6.5.1.1) (DNA ligase III) (Polydeoxyribonucleotide synthase [ATP] 3) | 1.3802E-05 | n/a | n/a | C |
| Q8BIE6 | FRM4A_MOUSE | 1020 | 113879 | 8.9 | (Q8BIE6) FERM domain-containing protein 4A | 1.3734E-05 | n/a | n/a | C |
| Q9Z0K8 | VNN1_MOUSE | 512 | 57061 | 5.9 | (Q9Z0K8) Pantetheinase precursor (EC 3.5.1.92) (Pantetheine hydrolase) (Vascular non-inflammatory molecule 1) (Vanin-1) | 1.3681E-05 | n/a | n/a | C |
| Q61749 | EI2BD_MOUSE | 524 | 57596 | 9.2 | (Q61749) Translation initiation factor eIF-2B subunit delta (eIF-2B GDP-GTP exchange factor subunit delta) | 1.3367E-05 | n/a | n/a | C |

| | | | | | | | | | |
|---|---|---|---|---|---|---|---|---|---|
| Q64511 | TOP2B_MOUSE | 1612 | 181908 | 8.3 | (Q64511) DNA topoisomerase 2-beta (EC 5.99.1.3) (DNA topoisomerase II, beta isozyme) | 1.3036E-05 | n/a | n/a | C |
| O70361 | PER3_MOUSE | 1113 | 120939 | 6.5 | (O70361) Period circadian protein 3 (mPER3) | 1.2587E-05 | n/a | n/a | C |
| Q9Z2V6 | HDAC5_MOUSE | 1113 | 120942 | 6.2 | (Q9Z2V6) Histone deacetylase 5 (HD5) (Histone deacetylase mHDA1) | 1.2587E-05 | n/a | n/a | C |
| Q8CHG3 | GCC2_MOUSE | 1679 | 194443 | 5.1 | (Q8CHG3) GRIP and coiled-coil domain-containing protein 2 (Golgi coiled coil protein GCC185) | 1.2516E-05 | n/a | n/a | C |
| Q91XQ5 | ST4S6_MOUSE | 561 | 64986 | 8 | (Q91XQ5) N-acetylgalactosamine 4-sulfate 6-O-sulfotransferase (EC 2.8.2.33) (GalNAc4S-6ST) (B-cell RAG-associated gene protein) | 1.2486E-05 | n/a | n/a | C |
| Q61503 | 5NTD_MOUSE | 576 | 63864 | 6.7 | (Q61503) 5'-nucleotidase precursor (EC 3.1.3.5) (Ecto-5'-nucleotidase) (5'-NT) (CD73 antigen) | 1.2161E-05 | n/a | n/a | C |
| P59242 | CING_MOUSE | 1191 | 136447 | 5.9 | (P59242) Cingulin | 1.1762E-05 | n/a | n/a | C |
| Q8BGZ4 | CDC23_MOUSE | 597 | 68562 | 7.2 | (Q8BGZ4) Cell division cycle protein 23 homolog (Anaphase-promoting complex subunit 8) (APC8) (Cyclosome subunit 8) | 1.1733E-05 | n/a | n/a | C |
| Q9CRC8 | LRC40_MOUSE | 602 | 68076 | 6.9 | (Q9CRC8) Leucine-rich repeat-containing protein 40 | 1.1635E-05 | n/a | n/a | C |
| Q6PNC0 | DMXL1_MOUSE | 3013 | 336009 | 6.4 | (Q6PNC0) Protein DmX-like 1 (X-like 1 protein) | 1.1624E-05 | n/a | n/a | C |
| Q9Z2J0 | S23A1_MOUSE | 605 | 65554 | 7.1 | (Q9Z2J0) Solute carrier family 23 member 1 (Sodium-dependent vitamin C transporter 1) (Na(+)/L-ascorbic acid transporter 1) (Yolk sac permease-like molecule 3) | 1.1578E-05 | n/a | n/a | C |
| P33174 | KIF4A_MOUSE | 1231 | 139551 | 6.7 | (P33174) Chromosome-associated kinesin KIF4A (Chromokinesin) | 1.138E-05 | n/a | n/a | C |
| Q61687 | ATRX_MOUSE | 2476 | 278602 | 6.7 | (Q61687) Transcriptional regulator ATRX (EC 3.6.1.-) (ATP-dependent helicase ATRX) (X-linked nuclear protein) (Heterochromatin protein 2) (HP1 alpha-interacting protein) (HP1 BP38 protein) | 1.1316E-05 | n/a | n/a | C |
| Q9QZS7 | NPHN_MOUSE | 1242 | 134890 | 5.6 | (Q9QZS7) Nephrin precursor (Renal glomerulus-specific cell adhesion receptor) | 1.1279E-05 | n/a | n/a | C |
| Q8K1N2 | PHLB2_MOUSE | 1249 | 141485 | 7.7 | (Q8K1N2) Pleckstrin homology-like domain family B member 2 (Protein LL5-beta) | 1.1216E-05 | n/a | n/a | C |
| Q3U9G9 | LBR_MOUSE | 626 | 71440 | 9.4 | (Q3U9G9) Lamin-B receptor (Integral nuclear envelope inner membrane protein) | 1.1189E-05 | n/a | n/a | C |
| Q64487 | PTPRD_MOUSE | 1894 | 212193 | 6.6 | (Q64487) Receptor-type tyrosine-protein phosphatase delta precursor (EC 3.1.3.48) (Protein-tyrosine phosphatase delta) (R-PTP-delta) | 1.1095E-05 | n/a | n/a | C |
| Q9R1E0 | FOXO1_MOUSE | 652 | 69502 | 6.9 | (Q9R1E0) Forkhead box protein O1A (Forkhead in rhabdomyosarcoma) | 1.0743E-05 | n/a | n/a | C |
| Q99PE8 | ABCG5_MOUSE | 652 | 73244 | 8.1 | (Q99PE8) ATP-binding cassette sub-family G member 5 (Sterolin-1) | 1.0743E-05 | n/a | n/a | C |
| P45700 | MA1A1_MOUSE | 655 | 73276 | 6.8 | (P45700) Mannosyl-oligosaccharide 1,2-alpha-mannosidase IA (EC 3.2.1.113) (Processing alpha-1,2-mannosidase IA) (Alpha-1,2-mannosidase IA) (Mannosidase alpha class 1A member 1) (Man(9)-alpha-mannosidase) (Man9-mannosidase) | 1.0694E-05 | n/a | n/a | C |
| Q62245 | SOS1_MOUSE | 1319 | 150883 | 6.9 | (Q62245) Son of sevenless homolog 1 (SOS-1) (mSOS-1) | 1.0621E-05 | n/a | n/a | C |
| Q9QXZ6 | SO1A1_MOUSE | 670 | 74396 | 8.2 | (Q9QXZ6) Solute carrier organic anion transporter family member 1A1 (Solute carrier family 21 member 1) (Sodium-independent organic anion-transporting polypeptide 1) (OATP1) | 1.0455E-05 | n/a | n/a | C |

| | | | | | | | | | |
|---|---|---|---|---|---|---|---|---|---|
| Q61584 | FXR1_MOUSE | 677 | 76222 | 7 | (Q61584) Fragile X mental retardation syndrome-related protein 1 (mFxr1p) | 1.0346E-05 | n/a | n/a | C |
| Q6PGC1 | DHX29_MOUSE | 1365 | 153975 | 7.9 | (Q6PGC1) Putative ATP-dependent RNA helicase DHX29 (EC 3.6.1.-) (DEAH box protein 29) | 1.0263E-05 | n/a | n/a | C |
| Q8K3H0 | DP13A_MOUSE | 707 | 79328 | 5.4 | (Q8K3H0) DCC-interacting protein 13 alpha (Dip13 alpha) (Adapter protein containing PH domain, PTB domain and leucine zipper motif 1) | 9.9074E-06 | n/a | n/a | C |
| Q60604 | ADSV_MOUSE | 715 | 80294 | 5.9 | (Q60604) Adseverin (Scinderin) (Gelsolin-like protein) | 9.7966E-06 | n/a | n/a | C |
| Q9R1X5 | MRP5_MOUSE | 1436 | 161129 | 8.7 | (Q9R1X5) Multidrug resistance-associated protein 5 (ABC transporter MOAT-C) (SMRP) | 9.7557E-06 | n/a | n/a | C |
| Q9D0R2 | SYTC_MOUSE | 722 | 83356 | 7.4 | (Q9D0R2) Threonyl-tRNA synthetase, cytoplasmic (EC 6.1.1.3) (Threonine--tRNA ligase) (ThrRS) | 9.7016E-06 | n/a | n/a | C |
| Q8VHE0 | SEC63_MOUSE | 759 | 87711 | 5.4 | (Q8VHE0) Translocation protein SEC63 homolog | 9.2287E-06 | n/a | n/a | C |
| Q3TCH7 | CUL4A_MOUSE | 759 | 87753 | 8.3 | (Q3TCH7) Cullin-4A (CUL-4A) | 9.2287E-06 | n/a | n/a | C |
| Q9EQZ7 | RIMS2_MOUSE | 1530 | 172862 | 9.2 | (Q9EQZ7) Regulating synaptic membrane exocytosis protein 2 (Rab3-interacting molecule 2) (RIM 2) (Rab3-interacting protein 2) | 9.1563E-06 | n/a | n/a | C |
| Q9Z103 | ADNP_MOUSE | 828 | 92063 | 6.1 | (Q9Z103) Activity-dependent neuroprotector (Activity-dependent neuroprotective protein) | 8.4596E-06 | n/a | n/a | C |
| Q9CQ33 | LZTR1_MOUSE | 837 | 94476 | 6.5 | (Q9CQ33) Leucine-zipper-like transcriptional regulator 1 (LZTR-1) | 8.3687E-06 | n/a | n/a | C |
| Q9JLN9 | FRAP_MOUSE | 2549 | 288734 | 7.1 | (Q9JLN9) FKBP12-rapamycin complex-associated protein (FK506-binding protein 12-rapamycin complex-associated protein 1) (Rapamycin target protein) (RAPT1) (Mammalian target of rapamycin) (mTOR) | 8.2439E-06 | n/a | n/a | C |
| P08122 | CO4A2_MOUSE | 1707 | 167391 | 8.6 | (P08122) Collagen alpha-2(IV) chain precursor | 8.2069E-06 | n/a | n/a | C |
| O54990 | PROM1_MOUSE | 867 | 97113 | 6.7 | (O54990) Prominin-1 precursor (Prominin-like protein 1) (Antigen AC133 homolog) | 8.0791E-06 | n/a | n/a | C |
| Q921M4 | GOGA2_MOUSE | 888 | 101357 | 4.8 | (Q921M4) Golgin subfamily A member 2 (Cis-Golgi matrix protein GM130) | 7.888E-06 | n/a | n/a | C |
| Q8VIM5 | MYCD_MOUSE | 935 | 101373 | 6.4 | (Q8VIM5) Myocardin (SRF cofactor protein) (Basic SAP coiled-coil transcription activator 2) | 7.4915E-06 | n/a | n/a | C |
| P70268 | PKN1_MOUSE | 946 | 104411 | 6.3 | (P70268) Serine/threonine-protein kinase N1 (EC 2.7.11.13) (Protein kinase C-like 1) (Protein-kinase C-related kinase 1) (Protein kinase C-like PKN) (Serine-threonine protein kinase N) | 7.4044E-06 | n/a | n/a | C |
| P35123 | UBP4_MOUSE | 962 | 108281 | 5.6 | (P35123) Ubiquitin carboxyl-terminal hydrolase 4 (EC 3.1.2.15) (Ubiquitin thioesterase 4) (Ubiquitin-specific-processing protease 4) (Deubiquitinating enzyme 4) (Ubiquitous nuclear protein) | 7.2813E-06 | n/a | n/a | C |
| Q8C9B9 | DIDO1_MOUSE | 1956 | 214444 | 8.8 | (Q8C9B9) Death-inducer obliterator 1 (DIO-1) (Death-associated transcription factor 1) (DATF-1) (Fragments) | 7.1621E-06 | n/a | n/a | C |
| Q61464 | ZN638_MOUSE | 1960 | 218132 | 6.9 | (Q61464) Zinc finger protein 638 (Nuclear protein 220) (Zinc-finger matrin-like protein) | 7.1475E-06 | n/a | n/a | C |
| Q9QVP9 | FAK2_MOUSE | 1009 | 115822 | 6.4 | (Q9QVP9) Protein tyrosine kinase 2 beta (EC 2.7.10.2) (Focal adhesion kinase 2) (FADK 2) (Proline-rich tyrosine kinase 2) (Cell adhesion kinase beta) (CAK beta) (Calcium-dependent tyrosine kinase) (CADTK) (Related adhesion focal tyrosine kinas | 6.9421E-06 | n/a | n/a | C |
| Q6P9J5 | ANR38_MOUSE | 1016 | 110404 | 4.9 | (Q6P9J5) Ankyrin repeat domain-containing protein 38 | 6.8943E-06 | n/a | n/a | C |

| | | | | | | | | | |
|---|---|---|---|---|---|---|---|---|---|
| Q8BUR4 | DOCK1_MOUSE | 1027 | 118534 | 7.5 | (Q8BUR4) Dedicator of cytokinesis protein 1 (180 kDa protein downstream of CRK) (DOCK180) (Fragments) | 6.8204E-06 | n/a | n/a | C |
| Q9WTZ2 | MBTP1_MOUSE | 1052 | 117457 | 8.8 | (Q9WTZ2) Membrane-bound transcription factor site 1 protease precursor (EC 3.4.21.-) (S1P endopeptidase) (Site-1 protease) (Subtilisin/kexin isozyme 1) (SKI-1) (Sterol-regulated luminal protease) | 6.6583E-06 | n/a | n/a | C |
| Q8VHY0 | CSPG4_MOUSE | 2327 | 252403 | 5.4 | (Q8VHY0) Chondroitin sulfate proteoglycan 4 precursor (Chondroitin sulfate proteoglycan NG2) (AN2 proteoglycan) | 6.0203E-06 | n/a | n/a | C |
| Q923L3 | CSMD1_MOUSE | 3564 | 387872 | 6.2 | (Q923L3) CUB and sushi domain-containing protein 1 precursor (CUB and sushi multiple domains protein 1) | 5.8961E-06 | n/a | n/a | C |
| P08032 | SPTA1_MOUSE | 2415 | 279993 | 5 | (P08032) Spectrin alpha chain, erythrocyte (Erythroid alpha-spectrin) | 5.8009E-06 | n/a | n/a | C |
| O54988 | SLK_MOUSE | 1233 | 141457 | 5.1 | (O54988) STE20-like serine/threonine-protein kinase (EC 2.7.11.1) (STE20-like kinase) (STE20-related serine/threonine-protein kinase) (STE20-related kinase) (mSLK) (Serine/threonine protein kinase 2) (STE20-related kinase SMAK) (Etk4) | 5.6809E-06 | n/a | n/a | C |
| Q925H1 | TRPS1_MOUSE | 1281 | 141034 | 7.6 | (Q925H1) Zinc finger transcription factor Trps1 | 5.468E-06 | n/a | n/a | C |
| P35917 | VGFR3_MOUSE | 1363 | 153016 | 6.3 | (P35917) Vascular endothelial growth factor receptor 3 precursor (EC 2.7.10.1) (VEGFR-3) (Tyrosine-protein kinase receptor FLT4) | 5.1391E-06 | n/a | n/a | C |
| P11087 | CO1A1_MOUSE | 1453 | 138033 | 5.8 | (P11087) Collagen alpha-1(I) chain precursor | 4.8208E-06 | n/a | n/a | C |
| P42859 | HD_MOUSE | 3119 | 344689 | 6.3 | (P42859) Huntingtin (Huntington disease protein homolog) (HD protein) | 4.4915E-06 | n/a | n/a | C |
| Q8CJ27 | ASPM_MOUSE | 3122 | 364121 | 10.6 | (Q8CJ27) Abnormal spindle-like microcephaly-associated protein homolog (Calmodulin-binding protein 1) (Spindle and hydroxyurea checkpoint abnormal protein) (Calmodulin-binding protein Sha1) | 4.4872E-06 | n/a | n/a | C |
| P19096 | FAS_MOUSE | 2504 | 272426 | 6.6 | (P19096) Fatty acid synthase (EC 2.3.1.85) [Includes: [Acyl-carrier-protein] S-acetyltransferase (EC 2.3.1.38); [Acyl-carrier-protein] S-malonyltransferase (EC 2.3.1.39); 3-oxoacyl-[acyl-carrier-protein] synthase (EC 2.3.1.41); 3-oxoacyl-[acyl- | 2.7974E-06 | n/a | n/a | C |
