# Supplementary material for: Comprehensive analysis of the mouse renal cortex using two-dimensional HPLC – tandem mass spectrometry
Source: Proteome Sci. 2008 May 23;6:15. doi: 10.1186/1477-5956-6-15 (PMC2412861; doi:10.1186/1477-5956-6-15)
Supplement: Additional file 2 — List of all peptides. Identified peptides with <1% FPR obtained from one mouse renal cortex separated into 60 fractions using SCX prior to LC-MS/MS. [file 1477-5956-6-15-S2.pdf]

Supplemental Table S2. The identified peptides.

LocusID	MS/MS Scan Number	Charge State	XCorr	DeltCN	SpR	SpScore	Ion Proportion	
1433B_MOUSE	MK_SCX_17.7815.7815.2	2	3.311	0.567	1	465.5	52.77778	K.TAFDEAIAELDTLNEESYK.D
1433B_MOUSE	MK_SCX_19.4539.4539.2	2	5.468	0.595	1	1490.3	66.66667	K.QTTVSNSQQAYQEAFEISK.K
1433B_MOUSE	MK_SCX_21.4012.4012.2	2	2.65	0.258	1	365.6	46.153847	K.AVTEQGHELSNEER.N
1433B_MOUSE	MK_SCX_23.5450.5450.3	3	5.952	0.592	1	941.7	31.034481	R.YLSEVASGENKQTTVSNSQQAYQEAFEISK.K
1433B_MOUSE	MK_SCX_25.4282.4282.2	2	2.482	0.263	1	485.6	85.71429	R.NLLSVAYK.N
1433B_MOUSE	MK_SCX_30.4259.4259.3	3	4.748	0.623	1	1451.2	58.333332	K.LAEQAERYDDMAAAMK.A
1433B_MOUSE	MK_SCX_36.6188.6188.2	2	3.743	0.541	1	949.1	73.07692	R.NLLSVAYKNVVGAR.R
1433E_MOUSE	MK_SCX_16.5470.5470.2	2	4.616	0.459	1	1658.7	66.66667	R.DNLTLWTSDMQGDGEEQNK.E
1433E_MOUSE	MK_SCX_16.7879.7879.2	2	6.76	0.628	1	2769.4	77.77778	K.AAFDDAIAELDTLSEESYK.D
1433E_MOUSE	MK_SCX_19.3992.3992.2	2	4.612	0.495	1	1951.4	87.5	K.VAGM*DVELTVEER.N
1433E_MOUSE	MK_SCX_21.3725.3725.2	2	2.59	0.474	1	614.5	70	K.EAAENSLVAYK.A
1433E_MOUSE	MK_SCX_29.4895.4895.3	3	3.481	0.458	1	925.9	42.1875	K.AASDIAMTELPPTHPIR.L
1433E_MOUSE	MK_SCX_30.4947.4947.3	3	4.585	0.508	1	1345.4	51.666664	K.LAEQAERYDEMVESMK.K
1433E_MOUSE	MK_SCX_32.13778.13778.3	3	5.858	0.43	1	2239.2	46.739132	K.LICCDILDVLDKHLIPAANTGESK.V
1433E_MOUSE	MK_SCX_36.6745.6745.2	2	2.729	0.238	1	720.8	61.538464	R.NLLSVAYKNVIGAR.R
1433E_MOUSE	MK_SCX_41.5020.5020.3	3	5.065	0.535	1	1400.4	42.045452	R.YLAEFATGNDRKEAAENSLVAYK.A
1433E_MOUSE	MK_SCX_44.4635.4635.3	3	5.489	0.393	1	1734.8	51.5625	K.LAEQAERYDEMVESMKK.V
1433E_MOUSE	MK_SCX_44.4689.4689.2	2	4.215	0.476	1	989.8	53.125	K.LAEQAERYDEMVESMKK.V
1433F_MOUSE	MK_SCX_25.6287.6287.3	3	4.544	0.528	1	811.1	34.523808	K.AVTELNEPLSNEDRNLLSVAYK.N
1433F_MOUSE	MK_SCX_25.6299.6299.2	2	4.011	0.481	1	971.4	52.380955	K.AVTELNEPLSNEDRNLLSVAYK.N
1433G_MOUSE	MK_SCX_20_1.3983.3983.2	2	4.766	0.511	1	927.3	73.07692	K.NVTELNEPLSNEER.N
1433G_MOUSE	MK_SCX_2201.3129.3129.2	2	3.683	0.365	1	1038.1	88.88889	R.YLAEVATGEK.R
1433T_MOUSE	MK_SCX_14.3680.3680.2	2	3.671	0.404	1	1838.2	95	R.YLAEVACGDDR.K
1433T_MOUSE	MK_SCX_21.3202.3202.2	2	5.157	0.547	1	2041.3	84.61539	K.AVTEQGAELSNEER.N
1433T_MOUSE	MK_SCX_21.3262.3262.3	3	3.989	0.326	1	940.5	50	K.AVTEQGAELSNEER.N
1433T_MOUSE	MK_SCX_37.6731.6731.2	2	2.591	0.359	1	308	50	R.NLLSVAYKNVVGGR.R
1433T_MOUSE	MK_SCX_46.4367.4367.3	3	5.294	0.497	1	1681.6	48.684208	R.KQTIENSQGAYQEAFDISKK.E
1433Z_MOUSE	MK_SCX_17.8433.8433.2	2	5.762	0.596	1	1901.3	66.66667	K.TAFDEAIAELDTLSEESYK.D
1433Z_MOUSE	MK_SCX_17.8477.8477.3	3	3.789	0.546	1	691.3	36.11111	K.TAFDEAIAELDTLSEESYK.D
1433Z_MOUSE	MK_SCX_18.5998.5998.2	2	6.23	0.669	1	3320.5	76.47059	K.GIVDQSQQAYQEAFEISK.K
1433Z_MOUSE	MK_SCX_18.9619.9619.2	2	2.868	0.259	1	574.7	47.058823	K.IETELRDICNDVLSLLEK.F
1433Z_MOUSE	MK_SCX_21.3314.3314.3	3	5.011	0.467	1	1852.3	57.692307	K.SVTEQGAELSNEER.N
1433Z_MOUSE	MK_SCX_21.3371.3371.2	2	3.782	0.41	1	991	80	R.YLAEVAAGDDK.K
1433Z_MOUSE	MK_SCX_21.3452.3452.2	2	5.43	0.495	1	1991.8	80.769226	K.SVTEQGAELSNEER.N
1433Z_MOUSE	MK_SCX_21.4285.4285.2	2	2.98	0.252	1	420.6	68.181816	K.FLIPNASQPESK.V
1433Z_MOUSE	MK_SCX_27.6046.6046.2	2	4.817	0.498	1	1292.5	58.333332	K.GIVDQSQQAYQEAFEISKK.E
1433Z_MOUSE	MK_SCX_27.6077.6077.3	3	3.156	0.538	1	528.4	37.5	K.GIVDQSQQAYQEAFEISKK.E
1433Z_MOUSE	MK_SCX_30.6464.6464.3	3	6.316	0.568	1	2892.9	37.068966	R.YLAEVAAGDDKKGIVDQSQQAYQEAFEISK.K
1433Z_MOUSE	MK_SCX_31.5676.5676.2	2	7.108	0.576	1	3870	77.77778	K.KGIVDQSQQAYQEAFEISK.K
1433Z_MOUSE	MK_SCX_31.5778.5778.3	3	5.777	0.418	1	2712.4	48.61111	K.KGIVDQSQQAYQEAFEISK.K
1433Z_MOUSE	MK_SCX_33.2866.2866.3	3	3.547	0.477	1	926.2	59.090908	R.YLAEVAAGDDKK.G
1433Z_MOUSE	MK_SCX_44.5395.5395.3	3	6.874	0.534	1	2527.8	50	K.KGIVDQSQQAYQEAFEISKK.E
1433Z_MOUSE	MK_SCX_44.5423.5423.2	2	6.749	0.556	1	3374.7	73.68421	K.KGIVDQSQQAYQEAFEISKK.E
3BHS2_MOUSE	MK_SCX_17.6179.6179.2	2	4.488	0.631	1	1156.7	80.769226	R.DLGYEPLVSWEEAK.Q
3BHS3_MOUSE	MK_SCX_13.7990.7990.2	2	5.072	0.611	1	1822.4	81.25	-.PGWSCLVTGAGGFLGQR.I
3BHS4_MOUSE	MK_SCX_21.5030.5030.2	2	3.68	0.362	1	1880.7	86.36364	R.MLVQEEELQEIR.A
3HAO_MOUSE	MK_SCX_19.5055.5055.2	2	3.558	0.463	1	1700.9	83.33333	R.YYVGDTEDVLFEK.W
3HAO_MOUSE	MK_SCX_21.4055.4055.2	2	3.872	0.502	1	1176.2	71.42857	R.AQGSVALSVTQDPAR.K
3HAO_MOUSE	MK_SCX_2201.4993.4993.2	2	3.858	0.315	1	869.8	80	R.FANTMGLVIER.R

3HAO_MOUSE	MK_SCX_23.10068.10068.2	2	5.201	0.541	1	814.3	60.526318	K.DLGTQLAPIIQEFFHSEQYR.T
3HAO_MOUSE	MK_SCX_23.10227.10227.3	3	4.063	0.501	1	697.3	35.526314	K.DLGTQLAPIIQEFFHSEQYR.T
3HAO_MOUSE	MK_SCX_25.4175.4175.2	2	2.439	0.133	1	719.6	72.22222	K.IMFVGGPNTR.K
3HAO_MOUSE	MK_SCX_57.9007.9007.3	3	3.02	0.31	1	758	52.499996	R.RRLESELDGLR.Y
3HIDH_MOUSE	MK_SCX_15.6389.6389.2	2	3.899	0.477	1	1272.6	56.81818	R.IITM*LPSSM*NAVEVYSGANGILK.K
3HIDH_MOUSE	MK_SCX_15.6860.6860.2	2	3.882	0.144	1	1072.7	54.545456	R.IITMLPSSM*NAVEVYSGANGILK.K
3HIDH_MOUSE	MK_SCX_15.7050.7050.2	2	4.119	0.223	1	1647.3	63.636364	R.IITM*LPSSMNAVEVYSGANGILK.K
3HIDH_MOUSE	MK_SCX_15.7410.7410.2	2	4.411	0.479	1	1621.5	65.909096	R.IITMLPSSMNAVEVYSGANGILK.K
3HIDH_MOUSE	MK_SCX_15.7498.7498.3	3	4.119	0.464	1	305.2	35.227272	R.IITMLPSSMNAVEVYSGANGILK.K
3HIDH_MOUSE	MK_SCX_18.3354.3354.2	2	3.949	0.532	1	1436.8	83.33333	K.DLGLAQDSATSTK.T
3HIDH_MOUSE	MK_SCX_18.5059.5059.2	2	5.083	0.58	1	2135.9	80	K.GSLLIDSSTIDPSVSK.E
3HIDH_MOUSE	MK_SCX_19.3205.3205.2	2	4.842	0.422	1	945.4	63.333332	K.EAGEQVASSPAEVAEK.A
3HIDH_MOUSE	MK_SCX_19.4115.4115.2	2	5.076	0.506	1	2303.3	79.411766	K.M*GAVFM*DAPVSGGVGAAR.S
3HIDH_MOUSE	MK_SCX_19.4408.4408.2	2	5.395	0.224	1	2101.8	82.35294	K.MGAVFM*DAPVSGGVGAAR.S
3HIDH_MOUSE	MK_SCX_19.4476.4476.2	2	5.691	0.224	1	2388.6	79.411766	K.M*GAVFMDAPVSGGVGAAR.S
3HIDH_MOUSE	MK_SCX_19.4759.4759.2	2	5.605	0.636	1	2190.9	85.29411	K.MGAVFMDAPVSGGVGAAR.S
3HIDH_MOUSE	MK_SCX_19.7122.7122.2	2	3.419	0.55	1	954.5	83.33333	K.DFSSVFQYLR.E
3HIDH_MOUSE	MK_SCX_20_1.4880.4880.2	2	4.744	0.554	1	952.2	62.5	K.TPVGFIGLGNM*GNPM*AK.N
3HIDH_MOUSE	MK_SCX_20_1.5771.5771.3	3	3.013	0.175	1	446.4	34.375	K.TPVGFIGLGNM*GNPMAK.N
3HIDH_MOUSE	MK_SCX_20_1.5857.5857.2	2	4.485	0.157	1	1609.7	71.875	K.TPVGFIGLGNM*GNPMAK.N
3HIDH_MOUSE	MK_SCX_20_1.6048.6048.2	2	5.059	0.219	1	1629.9	71.875	K.TPVGFIGLGNMGNPM*AK.N
3HIDH_MOUSE	MK_SCX_20_1.6081.6081.3	3	3.957	0.291	1	683.8	37.5	K.TPVGFIGLGNMGNPM*AK.N
3HIDH_MOUSE	MK_SCX_20_1.6890.6890.3	3	3.817	0.354	1	973.9	42.1875	K.TPVGFIGLGNMGNPMAK.N
3HIDH_MOUSE	MK_SCX_20_1.6960.6960.2	2	4.848	0.535	1	1633	71.875	K.TPVGFIGLGNMGNPMAK.N
3HIDH_MOUSE	MK_SCX_2201.7204.7204.3	3	3.845	0.168	1	377.9	32.608696	R.IITM*LPSSMNAVEVYSGANGILKK.V
3HIDH_MOUSE	MK_SCX_23.7269.7269.2	2	2.929	0.483	1	577.5	45.652176	R.IITMLPSSMNAVEVYSGANGILKK.V
3HIDH_MOUSE	MK_SCX_25.5936.5936.2	2	6.042	0.498	1	913	65.789474	K.GSLLIDSSTIDPSVSKELAK.E
3HIDH_MOUSE	MK_SCX_25.6040.6040.3	3	4.192	0.485	1	681.2	39.473686	K.GSLLIDSSTIDPSVSKELAK.E
3HIDH_MOUSE	MK_SCX_27.3782.3782.3	3	3.238	0.367	1	665.4	45.833336	K.EAGEQVASSPAEVAEKADR.I
3HIDH_MOUSE	MK_SCX_28.3721.3721.2	2	4.941	0.617	1	1693.9	66.66667	K.EAGEQVASSPAEVAEKADR.I
3HIDH_MOUSE	MK_SCX_28.3883.3883.2	2	5.853	0.625	1	1368.2	63.88889	K.EFKEAGEQVASSPAEVAEK.A
3HIDH_MOUSE	MK_SCX_28.3894.3894.3	3	4.115	0.439	1	1338.5	43.055553	K.EFKEAGEQVASSPAEVAEK.A
3HIDH_MOUSE	MK_SCX_30.7412.7412.3	3	7.621	0.636	1	3771.9	48.076923	K.DLGLAQDSATSTKTPILLGSLAHQIYR.M
3HIDH_MOUSE	MK_SCX_30.7416.7416.2	2	5.29	0.625	1	1076.2	48.076923	K.DLGLAQDSATSTKTPILLGSLAHQIYR.M
3HIDH_MOUSE	MK_SCX_31.4759.4759.2	2	5.299	0.609	1	1471.6	84.375	K.KGSLLIDSSTIDPSVSK.E
3HIDH_MOUSE	MK_SCX_31.4762.4762.3	3	3.27	0.294	1	301.8	42.1875	K.KGSLLIDSSTIDPSVSK.E
3HIDH_MOUSE	MK_SCX_31.9452.9452.3	3	3.752	0.321	1	721.6	34.72222	K.HGYPLILYDVFPDVCKEFK.E
3HIDH_MOUSE	MK_SCX_33.6963.6963.3	3	5.078	0.457	1	787.6	33.695652	K.GSLLIDSSTIDPSVSKELAKEVEK.M
3HIDH_MOUSE	MK_SCX_34.6195.6195.2	2	4.624	0.435	1	1157	73.07692	K.TPILLGSLAHQIYR.M
3HIDH_MOUSE	MK_SCX_34.6359.6359.3	3	4.353	0.495	1	1570.9	61.538464	K.TPILLGSLAHQIYR.M
3HIDH_MOUSE	MK_SCX_40.5944.5944.3	3	5.642	0.562	1	2206.4	48.75	K.KGSLLIDSSTIDPSVSKELAK.E
3HIDH_MOUSE	MK_SCX_40.5970.5970.2	2	5.655	0.647	1	1246	67.5	K.KGSLLIDSSTIDPSVSKELAK.E
3HIDH_MOUSE	MK_SCX_40.6854.6854.3	3	3.208	0.247	1	805.8	50	K.KDFSSVFQYLR.E
3HIDH_MOUSE	MK_SCX_40.6886.6886.2	2	3.631	0.433	1	1390.8	90	K.KDFSSVFQYLR.E
3HIDH_MOUSE	MK_SCX_48.4451.4451.3	3	5.542	0.506	1	1826.7	48.61111	K.VKKGSLLIDSSTIDPSVSK.E
3HIDH_MOUSE	MK_SCX_48.4472.4472.2	2	5.831	0.608	1	1354.6	77.77778	K.VKKGSLLIDSSTIDPSVSK.E
41_MOUSE	MK_SCX_17.8241.8241.2	2	2.247	0.17	1	567.9	44.11765	R.HSNLM*LEDLDKSQEEIKK.H
41_MOUSE	MK_SCX_20_1.3811.3811.2	2	3.299	0.386	1	742.1	73.07692	K.TQTVTISDTANAVK.S
41_MOUSE	MK_SCX_21.3868.3868.2	2	2.612	0.121	1	991.3	63.636364	K.FRYSGRTQAQTR.Q
41_MOUSE	MK_SCX_23.6222.6222.2	2	5.171	0.468	1	3192.6	71.05263	R.IVITGDADIDHDQVLVQAIK.E
41_MOUSE	MK_SCX_23.6271.6271.3	3	3.573	0.527	1	433.2	36.842106	R.IVITGDADIDHDQVLVQAIK.E

41_MOUSE	MK_SCX_31.3948.3948.3	3	3.798	0.413	1	813.8	36.764706	K.RGEEPAEPAEPEPTEAWK.V
41_MOUSE	MK_SCX_41.3899.3899.3	3	5.476	0.582	1	1610.2	53.333336	K.SEIPTKDVPIVHTETK.T
41_MOUSE	MK_SCX_42.3842.3842.2	2	3.889	0.492	1	953.1	63.333332	K.SEIPTKDVPIVHTETK.T
4F2_MOUSE	MK_SCX_19.4922.4922.2	2	4.196	0.511	1	583.2	62.5	R.LGASNLPAGISLPASAK.L
4F2_MOUSE	MK_SCX_19.8048.8048.2	2	4.611	0.48	1	1757.1	68.75	K.EALSSWLQDGVDGFQFR.D
4F2_MOUSE	MK_SCX_2201.4562.4562.2	2	3.024	0.344	1	660.3	72.22222	R.IGDLQAFVGR.D
4F2_MOUSE	MK_SCX_33.8491.8491.3	3	3.956	0.458	1	925.3	41.666664	K.MKEALSSWLQDGVDGFQFR.D
4F2_MOUSE	MK_SCX_35.3085.3085.3	3	3.342	0.316	1	917.8	45.833336	K.IKVAEDETEAGVK.F
4F2_MOUSE	MK_SCX_41.3916.3916.2	2	2.73	0.55	1	873.6	83.33333	K.SHLEYLSTLK.V
4F2_MOUSE	MK_SCX_41.7706.7706.3	3	4.32	0.563	1	1234	37.5	R.SLLHGDFHALSSSPDLFSYIR.H
4F2_MOUSE	MK_SCX_41.7711.7711.2	2	4.775	0.285	1	1791.8	57.5	R.SLLHGDFHALSSSPDLFSYIR.H
5NT3_MOUSE	MK_SCX_33.4731.4731.3	3	3.64	0.425	1	871.4	56.25	R.MADGVANVEHILK.I
5NT3_MOUSE	MK_SCX_33.4751.4751.2	2	3.183	0.348	1	798.8	75	R.MADGVANVEHILK.I
5NTD_MOUSE	MK_SCX_14.8369.8369.2	2	4.189	0.58	1	978	42.307693	K.ETPFLSNPGTNLVFEDEISALQPEVDK.L
5NTD_MOUSE	MK_SCX_28.4789.4789.3	3	3.33	0.469	1	699	39.0625	K.EVPAGKYPFIVTADDGR.Q
5NTD_MOUSE	MK_SCX_29.5216.5216.3	3	3.341	0.312	1	931	37.5	K.HDSGDQDISVVSEYISK.M
5NTD_MOUSE	MK_SCX_29.5227.5227.2	2	4.365	0.462	1	1772.7	71.875	K.HDSGDQDISVVSEYISK.M
5NTD_MOUSE	MK_SCX_29.5674.5674.3	3	3.149	0.247	1	400.3	42.307693	R.VPIYEPLEMDKVYK.V
5NTD_MOUSE	MK_SCX_38.5675.5675.2	2	2.448	0.168	1	355.1	54.545456	R.NVKFPILSANIK.A
5NTD_MOUSE	MK_SCX_40.3707.3707.3	3	3.481	0.322	1	1581.7	55	K.MKVVYPAVEGR.I
5NTD_MOUSE	MK_SCX_42.6001.6001.3	3	4.178	0.472	1	742.5	41.17647	K.IIALGHSGFEMDKLIAQK.V
5NTD_MOUSE	MK_SCX_52.5148.5148.3	3	3.715	0.397	1	900	37.5	K.ARGPLAHQISGLFLPSK.V
6PGL_MOUSE	MK_SCX_14.7972.7972.3	3	4.48	0.518	1	1054.2	34	K.LPIPDSQVLTINPALPVEDAAEDYAR.K
6PGL_MOUSE	MK_SCX_14.7991.7991.2	2	5.703	0.6	1	1048.9	48	K.LPIPDSQVLTINPALPVEDAAEDYAR.K
6PGL_MOUSE	MK_SCX_17.4643.4643.2	2	3.749	0.569	1	1092.4	76.666664	R.DLPAAAAPAGPASFAR.W
6PGL_MOUSE	MK_SCX_28.5113.5113.2	2	5.138	0.583	1	1313	71.875	R.ILEDKEGTLPAALVQPR.T
6PGL_MOUSE	MK_SCX_31.5405.5405.2	2	3.926	0.611	1	687.1	78.57143	R.LVPFDHAESTYGLYR.T
6PGL_MOUSE	MK_SCX_45.3699.3699.3	3	3.041	0.498	1	429	31.25	R.EKIVAPISDSPKPPPQR.V
A1AT1_MOUSE	MK_SCX_16.5949.5949.2	2	3.932	0.408	1	2376.6	66.66667	R.IFNNGADLSGITEENAPLK.L
A1AT1_MOUSE	MK_SCX_23.4202.4202.2	2	3.054	0.474	1	984.8	88.88889	R.LSISGEYNLK.T
A1AT1_MOUSE	MK_SCX_23.7799.7799.3	3	4.951	0.585	1	2703.8	47.61905	K.DQSPASHEIATNLGDFAISLYR.E
A1AT1_MOUSE	MK_SCX_58.9984.9984.2	2	2.73	0.22	1	458.8	75	R.RLAQIHFPR.L
A1AT2_MOUSE	MK_SCX_23.4060.4060.2	2	2.5	0.273	1	979.3	83.33333	R.LSISGDYNLK.T
A1AT2_MOUSE	MK_SCX_44.4252.4252.3	3	4.749	0.516	1	631.9	45.3125	K.KPFDPENTEEAEFHVDK.S
A1AT2_MOUSE	MK_SCX_50.4097.4097.3	3	3.819	0.428	1	1000.9	46.42857	K.MQHLEQTLNKELISK.I
A1AT5_MOUSE	MK_SCX_18.5543.5543.2	2	3.663	0.331	1	642.5	58.333332	R.IFNSGADLSGITEENAPLK.L
A1AT5_MOUSE	MK_SCX_34.6547.6547.3	3	6.025	0.528	1	1271.4	38	R.IFNSGADLSGITEENAPLKLSQAVHK.A
A2MG_MOUSE	MK_SCX_27.6937.6937.2	2	2.139	0.13	1	417.1	46.42857	K.LTEVPALVHKDTVVK.S
A2MG_MOUSE	MK_SCX_28.6624.6624.3	3	4.208	0.349	1	1274.8	41.25	K.VNTNYRPGLPFSGQVLLVDEK.G
AAAD_MOUSE	MK_SCX_21.8557.8557.3	3	3.78	0.509	1	359.5	24.13793	K.VQALIYPALQALDTNVPSQQEGSHFPVLTR.S
AAAD_MOUSE	MK_SCX_2201.6348.6348.2	2	3.186	0.32	1	1122.1	88.88889	R.WFLQEDVLEK.Y
AADAT_MOUSE	MK_SCX_15.8555.8555.2	2	3.38	0.509	1	674.3	57.14286	R.EVLLVPGNGFFIDGSAPTSFFR.A
AADAT_MOUSE	MK_SCX_17.5135.5135.2	2	3.262	0.404	1	529.3	57.894737	K.FLYTVPNGNNPTGNSLTGDR.K
AADAT_MOUSE	MK_SCX_18.6700.6700.2	2	3.69	0.53	1	430.7	50	K.TLISLAPGSPNPSM*FPFK.S
AADAT_MOUSE	MK_SCX_18.7358.7358.2	2	3.882	0.424	1	345.5	47.058823	K.TLISLAPGSPNPSMFPFK.S
AADAT_MOUSE	MK_SCX_18.9810.9810.2	2	4.533	0.567	1	1129.1	67.64706	R.ALQYSPSYGIPELLSWLK.Q
AADAT_MOUSE	MK_SCX_19.4656.4656.2	2	3.007	0.396	1	398	43.75	R.ASFSLATPAQM*DTAFQR.L
AADAT_MOUSE	MK_SCX_19.5623.5623.2	2	4.611	0.593	1	1068.7	71.875	R.ASFSLATPAQMDTAFQR.L
AADAT_MOUSE	MK_SCX_45.3608.3608.3	3	3.644	0.45	1	860.6	53.571426	K.ILSQWKPEDSKDPTK.K
AASS_MOUSE	MK_SCX_14.10297.10297.3	3	5.033	0.52	1	1034.2	32.575756	K.LGGDNTQLEAAEWLGLLGDEQVPQAESIVDAFSK.H
AASS_MOUSE	MK_SCX_15.7028.7028.2	2	5.653	0.624	1	2086.7	67.5	R.DNNIEITLGSDMTNQMQQLSK.K

AASS_MOUSE	MK_SCX_19.7064.7064.2	2	3.025	0.22	1	366.1	43.75	K.SIGPLTFVFTGTGNVSK.G
AASS_MOUSE	MK_SCX_21.11059.11059.3	3	4.918	0.466	1	1152.5	37.5	K.LQSLVESQDLVISLLPYVLHPVVAK.A
AASS_MOUSE	MK_SCX_21.4391.4391.2	2	2.678	0.401	1	957.5	88.88889	K.MLLDGEIEAK.G
AASS_MOUSE	MK_SCX_24.4517.4517.2	2	3.12	0.359	1	815	64.28571	R.DAVITSNGLLTDKYK.Y
AASS_MOUSE	MK_SCX_28.6393.6393.3	3	3.258	0.309	1	432.1	33.82353	R.VNMVTASYITPAMKELEK.S
AASS_MOUSE	MK_SCX_39.5025.5025.2	2	3.402	0.404	1	1381.2	83.33333	K.KYNINPVSLTVGK.Q
AASS_MOUSE	MK_SCX_44.4232.4232.3	3	3.589	0.438	1	739.5	37.5	R.KTDGVYDPVEYEKYPER.Y
AASS_MOUSE	MK_SCX_51.4122.4122.3	3	6.031	0.494	1	1064.7	50	K.KYNINPVSLTVGKQEAK.L
AASS_MOUSE	MK_SCX_57.3179.3179.3	3	3.032	0.401	1	431.5	35.714287	R.DSFGIRHPSGHLENK.T
AATC_MOUSE	MK_SCX_17.6770.6770.2	2	5.751	0.55	1	1262.4	72.22222	-.APPSVFAQVPQAPPVLVFK.L
AATC_MOUSE	MK_SCX_28.6515.6515.2	2	3.977	0.464	1	628.1	63.333332	R.IVAATLSDPELFKEWK.G
AATM_MOUSE	MK_SCX_20_1.5068.5068.2	2	3.672	0.302	1	1262.5	67.85714	K.ASAELALGENNEVLK.S
AATM_MOUSE	MK_SCX_20_1.5168.5168.2	2	4.344	0.468	1	1645.2	86.36364	R.IAATILTSPDLR.K
AATM_MOUSE	MK_SCX_21.5076.5076.2	2	5.038	0.509	1	2092.6	84.61539	R.FVTVQTISGTGALR.V
AATM_MOUSE	MK_SCX_27.4028.4028.2	2	2.515	0.299	1	500	75	K.MNLGVGAYR.D
AATM_MOUSE	MK_SCX_30.5186.5186.3	3	3.456	0.204	1	837.2	40	K.ILIRPLYSNPPLNGAR.I
AATM_MOUSE	MK_SCX_31.5478.5478.2	2	2.749	0.205	1	361.2	50	K.ILIRPLYSNPPLNGAR.I
AATM_MOUSE	MK_SCX_32.4281.4281.3	3	3.082	0.334	1	300.4	39.583336	R.IAATILTSPDLRK.Q
AATM_MOUSE	MK_SCX_37.4175.4175.3	3	3.474	0.326	1	842.6	50	R.LTKEFSVYMTK.D
AATM_MOUSE	MK_SCX_38.5971.5971.3	3	4.226	0.359	1	1344.6	38.095238	K.MNLGVGAYRDDNGKPYVLPSVR.K
AATM_MOUSE	MK_SCX_43.6645.6645.3	3	3.335	0.476	1	652.6	39.0625	R.DVFLPKPSWGNHTPIFR.D
AATM_MOUSE	MK_SCX_43.6663.6663.2	2	3.688	0.469	1	944	68.75	R.DVFLPKPSWGNHTPIFR.D
AATM_MOUSE	MK_SCX_52.4616.4616.3	3	3.571	0.311	1	972.7	36.363636	K.KM*NLGVGAYRDDNGKPYVLPSVR.K
AATM_MOUSE	MK_SCX_52.5124.5124.2	2	2.968	0.305	1	581.2	45.454548	K.KMNLGVGAYRDDNGKPYVLPSVR.K
AATM_MOUSE	MK_SCX_53.5435.5435.3	3	4.902	0.347	1	1403.4	38.636364	K.KMNLGVGAYRDDNGKPYVLPSVR.K
AB14B_MOUSE	MK_SCX_21.5483.5483.2	2	3.086	0.163	1	957	75	R.AVAIDLPGLGR.S
AB14B_MOUSE	MK_SCX_2201.5304.5304.2	2	5.106	0.56	1	1545.7	76.92308	R.FSSETWQNLGTLQR.L
AB14B_MOUSE	MK_SCX_26.5157.5157.3	3	5.898	0.605	1	1720.1	44.04762	K.TPALIVYGDQDPM*GSSSFQHLK.Q
AB14B_MOUSE	MK_SCX_26.5809.5809.2	2	5.896	0.631	1	1305.9	59.523808	K.TPALIVYGDQDPMGSSSFQHLK.Q
AB14B_MOUSE	MK_SCX_26.5841.5841.3	3	5.885	0.525	1	1863.7	44.04762	K.TPALIVYGDQDPMGSSSFQHLK.Q
AB14B_MOUSE	MK_SCX_39.6885.6885.2	2	2.909	0.401	1	1034.4	83.33333	R.FSVLLLHGIR.F
ABCA3_MOUSE	MK_SCX_26.4471.4471.3	3	4.385	0.191	1	777.7	32.894737	R.YHANTSAQQLFQKLM*VITKR.F
ABCB9_MOUSE	MK_SCX_18.7294.7294.2	2	2.566	0.181	1	346.2	50	R.HTVLIIAHRLSTVER.A
ABCB9_MOUSE	MK_SCX_31.6442.6442.2	2	2.023	0.246	1	325.7	55	R.AHLIVVLDKGR.V
ABCD3_MOUSE	MK_SCX_16.6848.6848.2	2	3.926	0.615	1	548.2	47.916664	K.GIEGAQASPLVPGAGEIINTDNIIK.F
ABCD3_MOUSE	MK_SCX_18.9000.9000.2	2	4.407	0.36	1	983.3	52.77778	R.EGGWDSVQDWMDVLSGGEK.Q
ABCD3_MOUSE	MK_SCX_31.6120.6120.2	2	2.595	0.176	1	355.5	50	K.EYLDNVQLGHILER.E
ABCD3_MOUSE	MK_SCX_36.10384.10384.3	3	4.223	0.392	1	795.4	34.523808	R.GISDQVLKEYLDNVQLGHILER.E
ABCF1_MOUSE	MK_SCX_27.3900.3900.3	3	3.975	0.501	1	545.4	45.588234	R.LQGQLEQGDDTAAEKLEK.V
ABCG2_MOUSE	MK_SCX_20_1.3721.3721.2	2	2.568	0.138	1	864.8	86.36364	K.AELDQLPGAQEK.K
ABCG2_MOUSE	MK_SCX_2201.6054.6054.2	2	3.392	0.33	1	1951.6	94.44444	K.SSLLDVLAAR.K
ABCG2_MOUSE	MK_SCX_23.4328.4328.2	2	2.486	0.415	1	845.8	77.77778	R.ENLQFSAALR.L
ABCG2_MOUSE	MK_SCX_23.8663.8663.3	3	3.051	0.246	1	851.2	27.083334	K.EILSDINGIMKPGLNAILGPTGGGK.S
ABCG2_MOUSE	MK_SCX_28.4974.4974.2	2	3.242	0.325	1	1002.4	58.823532	K.GLSGDVLINGAPQPAHFK.C
ABCG2_MOUSE	MK_SCX_35.6159.6159.2	2	3.126	0.148	1	424.3	72.72727	R.INTIIKELGLEK.V
ABI1_MOUSE	MK_SCX_2201.4334.4334.2	2	2.745	0.307	1	1437.8	80	R.ALIESYQNLTR.V
ABI1_MOUSE	MK_SCX_46.4955.4955.3	3	4.958	0.524	1	1707.5	55	R.KPIDYTVLDDVGHGVK.W
ABLM1_MOUSE	MK_SCX_25.6281.6281.3	3	4.154	0.409	1	999.3	46.666668	K.VDNEILDYKDLAAIPK.V
ABLM1_MOUSE	MK_SCX_31.4877.4877.3	3	3.871	0.344	1	990.7	46.875	K.FSKFPAAQAPDPNEIPK.I
ACAD8_MOUSE	MK_SCX_23.4878.4878.2	2	3.583	0.422	1	1551.8	81.818184	K.AAQLGFGGVYVR.T
ACAD8_MOUSE	MK_SCX_24.8276.8276.3	3	3.517	0.365	1	751.4	31.25	R.EMAPNMAEWDQKELFPVDVMR.K

ACAD8_MOUSE	MK_SCX_38.3665.3665.3	3	4.525	0.462	1	1241.6	52.083332	R.VHQILEGSNEVMR.M
ACAD8_MOUSE	MK_SCX_38.3668.3668.2	2	4.079	0.48	1	2310.7	87.5	R.VHQILEGSNEVMR.M
ACAD9_MOUSE	MK_SCX_23.9458.9458.3	3	4.341	0.419	1	674.4	33.333336	K.GVFPFPEVSQHELSEINQFVGPLEK.F
ACAD9_MOUSE	MK_SCX_47.3903.3903.3	3	5.277	0.501	1	1375.2	55.35714	R.KIDQEGKIPVDTLEK.L
ACADL_MOUSE	MK_SCX_13.5789.5789.2	2	4.344	0.373	1	832	57.894737	K.CIGAIAMTEPGAGSDLQGVR.T
ACADL_MOUSE	MK_SCX_20_1.6820.6820.2	2	3.857	0.396	1	1250.2	79.16667	K.AQDTAELFFEDVR.L
ACADL_MOUSE	MK_SCX_21.4027.4027.2	2	2.872	0.206	1	474.9	66.66667	R.VQPIYGGTNEIM*K.E
ACADL_MOUSE	MK_SCX_23.8712.8712.3	3	3.556	0.385	1	670.9	30.208334	K.AQDTAELFFEDVRLPANALLGEENK.G
ACADL_MOUSE	MK_SCX_25.8856.8856.3	3	4.771	0.428	1	1276.6	38.541664	R.LPANALLGEENKGFYYLMQELPQER.L
ACADL_MOUSE	MK_SCX_29.7519.7519.2	2	4.351	0.464	1	1321.7	73.52941	R.VQPIYGGTNEIMKELIAR.Q
ACADL_MOUSE	MK_SCX_37.4587.4587.3	3	3.866	0.245	1	1206.8	48.076923	K.AGKQGLLGINIAEK.H
ACADL_MOUSE	MK_SCX_37.4599.4599.2	2	4.374	0.465	1	1750	80.769226	K.AGKQGLLGINIAEK.H
ACADL_MOUSE	MK_SCX_38.4123.4123.2	2	3.207	0.365	1	1011.4	83.33333	R.IFSSEHDIFR.E
ACADL_MOUSE	MK_SCX_48.4787.4787.3	3	3.405	0.389	1	1162.9	50	K.FFQEEVIPHHTEWEK.A
ACADL_MOUSE	MK_SCX_48.4874.4874.2	2	5.454	0.447	1	923.7	75	K.FFQEEVIPHHTEWEK.A
ACADL_MOUSE	MK_SCX_51.4339.4339.2	2	2.857	0.367	1	583.4	57.692307	R.IFSSEHDIFRESVR.K
ACADL_MOUSE	MK_SCX_56.3790.3790.2	2	3.939	0.377	1	975.7	85	R.RIFSSEHDIFR.E
ACADL_MOUSE	MK_SCX_56.3806.3806.3	3	3.673	0.413	1	1149.9	57.5	R.RIFSSEHDIFR.E
ACADL_MOUSE	MK_SCX_56.4579.4579.3	3	4.742	0.433	1	1534	51.666664	R.KFFQEEVIPHHTEWEK.A
ACADL_MOUSE	MK_SCX_56.4613.4613.2	2	5.198	0.602	1	1141	73.333336	R.KFFQEEVIPHHTEWEK.A
ACADM_MOUSE	MK_SCX_15.10348.10348.2	2	4.686	0.589	1	1103.7	40	K.AFAGDIANQLATDAVQIFGGYGFNTEYPVEK.L
ACADM_MOUSE	MK_SCX_15.10562.10562.3	3	6.461	0.627	1	1972.8	35.833332	K.AFAGDIANQLATDAVQIFGGYGFNTEYPVEK.L
ACADM_MOUSE	MK_SCX_16.5189.5189.2	2	3.923	0.364	1	343.8	37.5	R.M*TEQPM*M*CAYCVTEPSAGSDVAAIK.T
ACADM_MOUSE	MK_SCX_16.6011.6011.2	2	3.408	0.334	1	515.4	45.833336	R.MTEQPMMCAYCVTEPSAGSDVAAIK.T
ACADM_MOUSE	MK_SCX_16.6019.6019.3	3	4.794	0.395	1	1238.8	37.5	R.MTEQPMMCAYCVTEPSAGSDVAAIK.T
ACADM_MOUSE	MK_SCX_18.4557.4557.2	2	3.225	0.459	1	635.1	86.36364	R.EEIIPVAPEYDK.S
ACADM_MOUSE	MK_SCX_21.17058.17058.3	3	3.74	0.405	1	1047	58.333332	K.IYQIYEGTAQIQR.L
ACADM_MOUSE	MK_SCX_21.4531.4531.2	2	4.856	0.483	1	2298.3	87.5	K.IYQIYEGTAQIQR.L
ACADM_MOUSE	MK_SCX_2201.4246.4246.2	2	3.457	0.252	1	777	77.27273	K.ENVLIGEGAGFK.I
ACADM_MOUSE	MK_SCX_2201.5183.5183.2	2	2.746	0.406	1	642.2	77.77778	K.SGEYPFPLIK.R
ACADM_MOUSE	MK_SCX_2201.7774.7774.2	2	4.065	0.611	1	389.2	45.238094	R.EEIIPVAPEYDKSGEYPFPLIK.R
ACADM_MOUSE	MK_SCX_23.4842.4842.3	3	4.961	0.177	1	537.4	32.692307	R.MTEQPM*M*CAYCVTEPSAGSDVAAIKTK.A
ACADM_MOUSE	MK_SCX_23.5658.5658.3	3	3.484	0.443	1	472.3	29.807693	R.MTEQPMMCAYCVTEPSAGSDVAAIKTK.A
ACADM_MOUSE	MK_SCX_23.5695.5695.2	2	3.552	0.534	1	335.2	36.53846	R.MTEQPMMCAYCVTEPSAGSDVAAIKTK.A
ACADM_MOUSE	MK_SCX_26.6577.6577.2	2	3.87	0.319	1	1476.6	93.75	K.ANWYFLLAR.S
ACADM_MOUSE	MK_SCX_33.3817.3817.3	3	3.203	0.421	1	422.2	40.909092	R.ALDEATKYALDR.K
ACADM_MOUSE	MK_SCX_35.4517.4517.2	2	4.839	0.561	1	1552.6	75	R.VPKENVLIGEGAGFK.I
ACADM_MOUSE	MK_SCX_35.4608.4608.3	3	3.43	0.267	1	1165.2	46.42857	R.VPKENVLIGEGAGFK.I
ACADM_MOUSE	MK_SCX_36.4272.4272.2	2	2.809	0.21	1	1066.2	80	R.GIAFEDVRVPK.E
ACADM_MOUSE	MK_SCX_36.4392.4392.2	2	3.096	0.365	1	643.5	80	K.SGEYPFPLIKR.A
ACADM_MOUSE	MK_SCX_38.9610.9610.3	3	4.221	0.411	1	969.9	32.954548	R.GIAFEDVRVPKENVLIGEGAGFK.I
ACADM_MOUSE	MK_SCX_39.3550.3550.3	3	4.537	0.468	1	915.9	42.857143	R.TRPTVAAGAVGLAQR.A
ACADM_MOUSE	MK_SCX_39.3563.3563.2	2	5.022	0.572	1	1383.1	82.14286	R.TRPTVAAGAVGLAQR.A
ACADM_MOUSE	MK_SCX_43.6045.6045.2	2	5.931	0.594	1	1425.3	61.11111	K.AFTGFIVEADTPGIHIGKK.E
ACADM_MOUSE	MK_SCX_43.6050.6050.3	3	3.644	0.601	1	988.9	44.444447	K.AFTGFIVEADTPGIHIGKK.E
ACADM_MOUSE	MK_SCX_48.5321.5321.3	3	3.573	0.476	1	563.3	35.526314	K.AAHKQEPGLGFSFELTEQQK.E
ACADM_MOUSE	MK_SCX_52.5688.5688.3	3	5.748	0.621	1	1296.5	39.423077	K.AAHKQEPGLGFSFELTEQQKEFQATAR.K
ACADS_MOUSE	MK_SCX_17.6605.6605.2	2	4.159	0.433	1	946.2	67.64706	K.GISAFLVPM*PTPGLTLGK.K
ACADS_MOUSE	MK_SCX_21.5224.5224.2	2	3.317	0.407	1	910.1	68.181816	K.LADMALALESAR.L
ACADS_MOUSE	MK_SCX_36.7406.7406.3	3	3.683	0.412	1	457.4	35	K.ELVPIAAQLDREHLFPTAQVK.K
ACADS_MOUSE	MK_SCX_45.5382.5382.3	3	5.095	0.553	1	756.3	38.235294	R.LHTVYQSVELPETHQMLR.Q

ACADS_MOUSE	MK_SCX_45.5420.5420.2	2	6.157	0.644	1	1633.8	73.52941	R.LHTVYQSVELPETHQMLR.Q
ACADS_MOUSE	MK_SCX_46.4666.4666.3	3	5.067	0.567	1	1461.3	45.588234	R.LHTVYQSVELPETHQM*LR.Q
ACADS_MOUSE	MK_SCX_49.6660.6660.3	3	3.387	0.342	1	396.9	30.952381	K.ELVPIAAQLDREHLFPTAQVKK.M
ACADV_MOUSE	MK_SCX_16.7924.7924.2	2	5.159	0.581	1	687.9	50	K.ELGAFGLQVPSELGGLGLSNTQYAR.L
ACADV_MOUSE	MK_SCX_16.7944.7944.3	3	5.063	0.507	1	1323.5	37.5	K.ELGAFGLQVPSELGGLGLSNTQYAR.L
ACADV_MOUSE	MK_SCX_16.8428.8428.2	2	3.994	0.538	1	337.7	34.782608	K.GQLTIDQVFPYPSVLSEEQAQFLK.E
ACADV_MOUSE	MK_SCX_16.8451.8451.3	3	4.367	0.558	1	879.1	34.782608	K.GQLTIDQVFPYPSVLSEEQAQFLK.E
ACADV_MOUSE	MK_SCX_17.12635.12635.2	2	4.389	0.48	1	301.5	52.77778	R.SGELAVQALDQFATVVEAK.L
ACADV_MOUSE	MK_SCX_17.9751.9751.2	2	3.084	0.325	1	1249.6	62.5	K.IWISNGGLADIFTVFAK.T
ACADV_MOUSE	MK_SCX_19.9741.9741.3	3	6.136	0.545	1	1414.7	33.088234	K.VEDDTLQGLKELGAFGLQVPSELGGLGLSNTQYAR.L
ACADV_MOUSE	MK_SCX_2201.5209.5209.2	2	3.637	0.228	1	1353.8	75	K.GIVNEQFLLQR.L
ACADV_MOUSE	MK_SCX_23.3998.3998.2	2	2.768	0.296	1	1155.3	77.27273	R.FGMAATLAGTM*K.S
ACADV_MOUSE	MK_SCX_23.4552.4552.2	2	2.702	0.314	1	464.2	83.33333	R.IFEGANDILR.L
ACADV_MOUSE	MK_SCX_25.4128.4128.3	3	5.024	0.454	1	1363.2	42.105263	R.EATQAVLDKPETLSSDASTR.E
ACADV_MOUSE	MK_SCX_25.4147.4147.2	2	5.388	0.574	1	666	52.63158	R.EATQAVLDKPETLSSDASTR.E
ACADV_MOUSE	MK_SCX_28.4370.4370.2	2	5.365	0.474	1	1325.3	80	R.FFEEVNDPAKNDALEK.V
ACADV_MOUSE	MK_SCX_29.6545.6545.3	3	4.702	0.534	1	1873.3	48.61111	R.TGIGSGLSLSGIVHPELSR.S
ACADV_MOUSE	MK_SCX_40.4938.4938.2	2	3.506	0.352	1	1405.1	81.818184	K.KGIVNEQFLLQR.L
ACADV_MOUSE	MK_SCX_49.6124.6124.3	3	3.601	0.221	1	684.9	34.210526	R.RTGIGSGLSLSGIVHPELSR.S
ACADV_MOUSE	MK_SCX_50.4266.4266.3	3	5.222	0.545	1	1769.9	47.22222	R.IRENMASLQSSPQHQELFR.N
ACADV_MOUSE	MK_SCX_50.4316.4316.2	2	5.74	0.636	1	1011.2	61.11111	R.IRENMASLQSSPQHQELFR.N
ACBP_MOUSE	MK_SCX_25.5175.5175.3	3	3.287	0.451	1	1009.8	44.11765	K.QATVGDVNTDRPGLLDLK.G
ACBP_MOUSE	MK_SCX_28.4869.4869.2	2	4.39	0.521	1	651.1	61.764706	K.QATVGDVNTDRPGLLDLK.G
ACBP_MOUSE	MK_SCX_28.4915.4915.1	1	2.17	0.311	1	312.9	55.88235	K.QATVGDVNTDRPGLLDLK.G
ACBP_MOUSE	MK_SCX_28.5912.5912.2	2	4.836	0.654	1	1027	73.333336	K.TQPTDEEM*LFIYSHFK.Q
ACBP_MOUSE	MK_SCX_28.7165.7165.2	2	5.172	0.595	1	1212.7	73.333336	K.TQPTDEEMLFIYSHFK.Q
ACBP_MOUSE	MK_SCX_40.4560.4560.3	3	3.789	0.53	1	608.8	36.842106	K.QATVGDVNTDRPGLLDLKGK.A
ACBP_MOUSE	MK_SCX_40.4722.4722.2	2	3.554	0.484	1	430.2	55.263157	K.QATVGDVNTDRPGLLDLKGK.A
ACBP_MOUSE	MK_SCX_42.6634.6634.3	3	5.641	0.491	1	1328.6	48.52941	R.LKTQPTDEEMLFIYSHFK.Q
ACBP_MOUSE	MK_SCX_42.6647.6647.2	2	5.723	0.575	1	1421.5	67.64706	R.LKTQPTDEEMLFIYSHFK.Q
ACBP_MOUSE	MK_SCX_43.5835.5835.3	3	4.853	0.479	1	1290.3	48.52941	R.LKTQPTDEEM*LFIYSHFK.Q
ACBP_MOUSE	MK_SCX_43.5860.5860.2	2	5.207	0.575	1	931	58.823532	R.LKTQPTDEEM*LFIYSHFK.Q
ACDSB_MOUSE	MK_SCX_14.8795.8795.2	2	2.352	0.16	1	318.7	25	K.SSQPEALVSLTNNAVAFAPLQTLTDEEIM*M*K.Q
ACDSB_MOUSE	MK_SCX_14.9463.9463.2	2	4.866	0.679	1	740.8	38.333332	K.SSQPEALVSLTNNAVAFAPLQTLTDEEIMMK.Q
ACDSB_MOUSE	MK_SCX_18.5444.5444.2	2	4.371	0.308	1	1554.7	61.764706	K.IGTIYEGASNIQLNTIAK.H
ACDSB_MOUSE	MK_SCX_26.9255.9255.3	3	5.685	0.523	1	1569.5	38.095238	R.IFDFQGLQHQVAQVATQLEATR.L
ACDSB_MOUSE	MK_SCX_26.9265.9265.2	2	5.395	0.667	1	1920.6	59.523808	R.IFDFQGLQHQVAQVATQLEATR.L
ACDSB_MOUSE	MK_SCX_29.4378.4378.3	3	3.775	0.448	1	1165.9	44.11765	K.FAQEHVAPLVSSMDENSK.M
ACDSB_MOUSE	MK_SCX_29.4409.4409.2	2	5.258	0.548	1	1700.8	73.52941	K.FAQEHVAPLVSSMDENSK.M
ACDSB_MOUSE	MK_SCX_48.3477.3477.3	3	6.158	0.53	1	3362.6	61.11111	K.KFAQEHVAPLVSSM*DENSK.M
ACDSB_MOUSE	MK_SCX_48.4137.4137.3	3	5.444	0.522	1	3377.8	56.944443	K.KFAQEHVAPLVSSMDENSK.M
ACE_MOUSE	MK_SCX_18.8881.8881.2	2	5.558	0.603	1	1545.8	69.44444	K.ALLEYFQPVSQWLEEQNQR.N
ACF_MOUSE	MK_SCX_12.9243.9243.2	2	2.208	0.122	1	318.9	46.666668	-.MESNHKSGDGLSGTQK.E
ACF_MOUSE	MK_SCX_38.3798.3798.3	3	3.088	0.313	1	1413.1	56.666668	R.KYGGPPPGWDSTPPER.G
ACINU_MOUSE	MK_SCX_15.7011.7011.2	2	3.584	0.561	1	510.6	43.18182	K.APVVLQPEQIVSEEETPPPLLTK.E
ACINU_MOUSE	MK_SCX_18.5932.5932.2	2	3.424	0.557	1	429.6	60.714287	K.SAPLPLTVEEFAPAK.G
ACINU_MOUSE	MK_SCX_29.3851.3851.3	3	6.148	0.403	1	1830.6	45	K.SKLPEYSQTAEEEEDQETPSR.N
ACLY_MOUSE	MK_SCX_14.3837.3837.2	2	2.301	0.146	1	622.5	75	K.YICTTSAIQNR.F
ACLY_MOUSE	MK_SCX_41.3627.3627.3	3	3.747	0.353	1	1166.9	50	K.LYRPGSVAYVSR.S
ACO12_MOUSE	MK_SCX_53.5793.5793.3	3	3.851	0.32	1	493.7	30.681818	K.VHLKPVLLQTEQEQVEHNLASER.R
ACON_MOUSE	MK_SCX_13.5010.5010.2	2	4.664	0.666	1	1463.7	75	R.VGLIGSCTNSSYEDMGR.S

ACON_MOUSE	MK_SCX_16.5996.5996.2	2	5.606	0.666	1	2841.3	69.047615	R.VAM*QDATAQM*AM*LQFISSGLPK.V
ACON_MOUSE	MK_SCX_16.6664.6664.2	2	3.653	0.176	1	554.5	47.61905	R.VAM*QDATAQMAM*LQFISSGLPK.V
ACON_MOUSE	MK_SCX_16.7205.7205.2	2	3.368	0.344	1	1447.3	57.894737	R.DVGGIVLANACGPCIGQWDR.K
ACON_MOUSE	MK_SCX_16.7312.7312.3	3	3.749	0.135	1	456.3	35.714287	R.VAMQDATAQMAM*LQFISSGLPK.V
ACON_MOUSE	MK_SCX_16.7327.7327.2	2	3.156	0.14	1	559.5	42.857143	R.VAMQDATAQMAM*LQFISSGLPK.V
ACON_MOUSE	MK_SCX_16.8140.8140.2	2	5.368	0.148	1	1643.2	61.904762	R.VAMQDATAQM*AMLQFISSGLPK.V
ACON_MOUSE	MK_SCX_16.8573.8573.3	3	4.954	0.427	1	640.3	38.095238	R.VAMQDATAQMAMLQFISSGLPK.V
ACON_MOUSE	MK_SCX_17.7756.7756.1	1	3.712	0.512	1	651.4	66.66667	K.DINQEVYNFLATAGAK.Y
ACON_MOUSE	MK_SCX_18.6648.6648.2	2	3.337	0.424	1	789	60.000004	K.QGLLPLTFADPSDYNK.I
ACON_MOUSE	MK_SCX_18.9139.9139.2	2	5.866	0.68	1	2197.7	66.66667	R.VAMQDATAQMAMLQFISSGLPK.V
ACON_MOUSE	MK_SCX_18.9139.9139.3	3	3.072	0.412	1	349.1	18.333334	K.TYLRLRPDRVAMQDATAQMAMLQFISSGLPK.V
ACON_MOUSE	MK_SCX_20_1.4528.4528.2	2	4.668	0.14	1	1479.8	78.57143	R.NAVTQEFGPVPDTAR.Y
ACON_MOUSE	MK_SCX_21.4064.4064.2	2	2.314	0.282	1	1032.9	92.85714	R.DGYAQILR.D
ACON_MOUSE	MK_SCX_21.4707.4707.2	2	5.254	0.613	1	2034	89.28571	R.WVVIGDENYGEGSSR.E
ACON_MOUSE	MK_SCX_2201.4191.4191.2	2	3.971	0.612	1	1077.9	83.33333	K.SQFTITPGSEQIR.A
ACON_MOUSE	MK_SCX_2201.9290.9290.3	3	5.718	0.526	1	1479.5	35.576923	R.NDANPETHAFVTSPEIVTALAIAGTLK.F
ACON_MOUSE	MK_SCX_2201.9305.9305.2	2	4.48	0.52	1	1085.3	44.230766	R.NDANPETHAFVTSPEIVTALAIAGTLK.F
ACON_MOUSE	MK_SCX_24.4886.4886.2	2	2.337	0.368	1	463.7	78.57143	K.EGWPLDIR.V
ACON_MOUSE	MK_SCX_29.4199.4199.2	2	5.414	0.542	1	1394.7	80	K.IVYGHLDDPANQEIER.G
ACON_MOUSE	MK_SCX_30.4066.4066.3	3	4.97	0.507	1	1608.2	48.333332	K.IVYGHLDDPANQEIER.G
ACON_MOUSE	MK_SCX_31.6215.6215.3	3	4.026	0.435	1	1956.9	48.4375	K.KQGLLPLTFADPSDYNK.I
ACON_MOUSE	MK_SCX_31.6357.6357.2	2	5.343	0.539	1	1547.9	75	K.KQGLLPLTFADPSDYNK.I
ACON_MOUSE	MK_SCX_32.4580.4580.2	2	4.104	0.587	1	916.6	84.61539	K.FNPETDFLTGKDGK.K
ACON_MOUSE	MK_SCX_32.7103.7103.2	2	6.197	0.639	1	3068.4	82.35294	R.AKDINQEVYNFLATAGAK.Y
ACON_MOUSE	MK_SCX_32.7361.7361.3	3	4.177	0.477	1	1293.5	42.647057	R.AKDINQEVYNFLATAGAK.Y
ACON_MOUSE	MK_SCX_33.5076.5076.2	2	4.29	0.48	1	1960.3	91.66667	K.FKLEAPDADELPR.S
ACON_MOUSE	MK_SCX_33.5123.5123.3	3	4.931	0.431	1	2423.3	60.416668	K.FKLEAPDADELPR.S
ACON_MOUSE	MK_SCX_35.4215.4215.2	2	3.776	0.519	1	549.2	66.66667	K.VAM*SHFEPSEYIR.Y
ACON_MOUSE	MK_SCX_35.4235.4235.3	3	4.645	0.478	1	1168.9	54.166668	K.VAM*SHFEPSEYIR.Y
ACON_MOUSE	MK_SCX_35.5279.5279.3	3	4.021	0.383	1	631.9	47.916664	K.VAMSHFEPSEYIR.Y
ACON_MOUSE	MK_SCX_35.5352.5352.2	2	3.862	0.49	1	847	66.66667	K.VAMSHFEPSEYIR.Y
ACON_MOUSE	MK_SCX_40.3269.3269.3	3	3.542	0.518	1	612.3	41.25	R.SDFDPGQDTYQHPPKDSSGQR.V
ACON_MOUSE	MK_SCX_42.5001.5001.3	3	4.418	0.457	1	633.8	36.363636	R.WVVIGDENYGEGSSREHAALEPR.H
ACON_MOUSE	MK_SCX_48.4189.4189.2	2	4.002	0.497	1	944.3	78.57143	K.FNPETDFLTGKDGKK.F
ACON_MOUSE	MK_SCX_50.6272.6272.3	3	3.078	0.148	1	455.2	27.272728	K.KQGLLPLTFADPSDYNKIHPVDK.L
ACON_MOUSE	MK_SCX_52.4514.4514.3	3	4.432	0.375	1	1974.6	59.615387	K.KFKLEAPDADELPR.S
ACOT1_MOUSE	MK_SCX_24.11121.11121.3	3	3.48	0.135	1	487.1	25.925926	R.TPALGGSFSGLEPM*GLLWAMEPDRPFWR.L
ACOT1_MOUSE	MK_SCX_34.7018.7018.3	3	4.754	0.245	1	2035.8	48.52941	K.DGLKDVVDALQSPLVDKK.S
ACOT2_MOUSE	MK_SCX_2201.3388.3388.2	2	3.518	0.323	1	820.1	95	R.ADAGGELNLAR.A
ACOT2_MOUSE	MK_SCX_2201.8944.8944.2	2	4.996	0.388	1	1524.3	61.764706	K.DGLLDVVEALQSPLVDKK.S
ACOT2_MOUSE	MK_SCX_24.11946.11946.3	3	4.461	0.6	1	721.8	27.777779	R.APALGGSFSGLEPMGLLWAMEPERPLWR.L
ACOT2_MOUSE	MK_SCX_41.3618.3618.3	3	3.582	0.297	1	1001.1	45.833336	R.YRADAGGELNLAR.A
ACOT2_MOUSE	MK_SCX_41.3636.3636.2	2	3.594	0.459	1	1244.4	79.16667	R.YRADAGGELNLAR.A
ACOT8_MOUSE	MK_SCX_19.6038.6038.2	2	3.307	0.272	1	439.6	53.333336	K.VVNPPTLTQLQALEPK.Q
ACOX1_MOUSE	MK_SCX_15.9010.9010.2	2	3.407	0.457	1	336	40	R.IQPQQVAVWPTLVDINSLDSLTEAYK.L
ACOX1_MOUSE	MK_SCX_18.5202.5202.2	2	5.084	0.502	1	1634.6	80	R.SEPEPQILDFQTQQYK.L
ACOX1_MOUSE	MK_SCX_18.6222.6222.2	2	3.814	0.223	1	601.9	45	K.GGDFLEGNIITGAQMSQVNSR.I
ACOX1_MOUSE	MK_SCX_18.8065.8065.2	2	3.425	0.497	1	941.2	69.230774	R.EFGIADPEEIMWFK.K
ACOX1_MOUSE	MK_SCX_19.4534.4534.2	2	2.912	0.37	1	1806.2	86.36364	K.FGYEEMDNGYLK.M
ACOX1_MOUSE	MK_SCX_20_1.8323.8323.2	2	4.023	0.399	1	783.1	69.230774	R.YDGNVYENLFEWAK.K
ACOX1_MOUSE	MK_SCX_21.3420.3420.2	2	2.995	0.444	1	1149.9	85	R.GLETTATYDPK.T

ACOX1_MOUSE	MK_SCX_2201.8076.8076.3	3	4.298	0.446	1	627.3	35.714287	R.EIENLILNDPDFQHEDYNFLTR.S
ACOX1_MOUSE	MK_SCX_2201.8144.8144.2	2	5.001	0.559	1	653.6	50	R.EIENLILNDPDFQHEDYNFLTR.S
ACOX1_MOUSE	MK_SCX_23.9502.9502.3	3	4.824	0.254	1	832	30.645163	K.LHMVNFVEPVGLNYSM*FIPTLLNQGTTAQQEK.W
ACOX1_MOUSE	MK_SCX_23.9739.9739.3	3	5.885	0.667	1	4364.4	52.380955	R.ILELLTVTRPNAVALVDAFDFK.D
ACOX1_MOUSE	MK_SCX_23.9890.9890.3	3	6.192	0.596	1	1289.1	34.677418	K.LHMVNFVEPVGLNYSMFIPTLLNQGTTAQQEK.W
ACOX1_MOUSE	MK_SCX_33.8187.8187.3	3	3.852	0.438	1	1283.3	45	K.MREFGIADPEEIMWFK.K
ACOX1_MOUSE	MK_SCX_43.4996.4996.3	3	4.201	0.557	1	1016.8	38.88889	R.EIGTHKPLPGITVGDIGPK.F
ACOX1_MOUSE	MK_SCX_46.3634.3634.3	3	4.826	0.58	1	1221.6	46.875	K.YAQVKPDGTYVKPLSNK.L
ACOX1_MOUSE	MK_SCX_53.7709.7709.3	3	4.802	0.479	1	804.5	34.782608	R.RREIENLILNDPDFQHEDYNFLTR.S
ACOX1_MOUSE	MK_SCX_57.3426.3426.3	3	3.057	0.378	1	515.5	41.07143	K.KSPLNKTEVHQSYYK.H
ACOX3_MOUSE	MK_SCX_20_1.8281.8281.2	2	3.922	0.467	1	1465.5	76.92308	K.TIFSTLENDPLFAR.S
ACOX3_MOUSE	MK_SCX_31.4373.4373.2	2	3.551	0.515	1	743.1	50	R.TGNITSEGTYNSPFKDVR.Q
ACOX3_MOUSE	MK_SCX_32.4272.4272.3	3	3.396	0.402	1	625.7	32.352943	R.TGNITSEGTYNSPFKDVR.Q
ACOX3_MOUSE	MK_SCX_52.3440.3440.3	3	3.773	0.454	1	1119.9	38.88889	R.GIQECREACGGHGYLAMNR.F
ACPM_MOUSE	MK_SCX_13.8227.8227.2	2	3.865	0.412	1	1329.8	84.61539	K.LMCPQEIVDYIADK.K
ACPM_MOUSE	MK_SCX_53.4904.4904.3	3	5.783	0.527	1	1565.9	50	K.LYDKIDPEKLSVNSHFM*K.D
ACPM_MOUSE	MK_SCX_53.4946.4946.2	2	4.383	0.589	1	674.5	64.70589	K.LYDKIDPEKLSVNSHFM*K.D
ACPM_MOUSE	MK_SCX_53.5401.5401.2	2	5.131	0.51	1	983	67.64706	K.LYDKIDPEKLSVNSHFMK.D
ACPM_MOUSE	MK_SCX_53.5424.5424.3	3	5.613	0.498	1	1292.6	47.058823	K.LYDKIDPEKLSVNSHFMK.D
ACS2L_MOUSE	MK_SCX_14.10006.10006.2	2	5.566	0.642	1	1655.7	58.333332	R.GQDLGDTTTLEDPSVITEILSAFQK.Y
ACS2L_MOUSE	MK_SCX_14.10046.10046.3	3	5.821	0.556	1	2097.8	39.583336	R.GQDLGDTTTLEDPSVITEILSAFQK.Y
ACSL1_MOUSE	MK_SCX_20_1.15639.15639.2	2	2.795	0.147	1	638.3	53.571426	K.DINKAILDDLLKLGK.E
ACSL1_MOUSE	MK_SCX_41.4754.4754.3	3	4.973	0.522	1	2122.7	52.941177	R.TAEALDKDGWLHTGDIGK.W
ACTA_MOUSE	MK_SCX_15.6290.6290.2	2	4.316	0.479	1	1217	55	K.DLYANNVLSGGTTMYPGIADR.M
ACTA_MOUSE	MK_SCX_16.5863.5863.2	2	4.303	0.603	1	1277.9	57.5	K.DLYANNVLSGGTTM*YPGIADR.M
ACTA_MOUSE	MK_SCX_18.2180.2180.2	2	3.087	0.476	1	1160.1	85	K.DSYVGDEAQSK.R
ACTA_MOUSE	MK_SCX_20_1.6271.6271.3	3	4.687	0.425	1	1912.4	55	K.SYELPDGQVITIGNER.F
ACTA_MOUSE	MK_SCX_20_1.6399.6399.2	2	4.99	0.542	1	1659.3	83.33333	K.SYELPDGQVITIGNER.F
ACTA_MOUSE	MK_SCX_21.3742.3742.2	2	2.41	0.361	1	330.8	65	K.EITALAPSTM*K.I
ACTA_MOUSE	MK_SCX_21.4147.4147.2	2	3.069	0.367	1	977	85	K.EITALAPSTMK.I
ACTA_MOUSE	MK_SCX_2201.2496.2496.2	2	3.47	0.435	1	1256.6	88.88889	K.AGFAGDDAPR.A
ACTA_MOUSE	MK_SCX_26.4512.4512.2	2	4.747	0.55	1	850.9	61.764706	R.VAPEEHPTLLTEAPLNPK.A
ACTA_MOUSE	MK_SCX_26.5661.5661.3	3	3.371	0.232	1	501.3	32.142857	R.KDLYANNVLSGGTTMYPGIADR.M
ACTA_MOUSE	MK_SCX_27.4811.4811.3	3	3.963	0.51	1	559.4	38.235294	R.VAPEEHPTLLTEAPLNPK.A
ACTA_MOUSE	MK_SCX_29.4898.4898.2	2	4.306	0.509	1	432.8	56.666668	K.YPIEHGIITNWDDM*EK.I
ACTA_MOUSE	MK_SCX_29.6032.6032.3	3	3.14	0.296	1	770.8	48.076923	R.LDLAGRDLTDYLM*K.I
ACTA_MOUSE	MK_SCX_29.6050.6050.2	2	2.879	0.457	1	495.3	61.538464	R.LDLAGRDLTDYLM*K.I
ACTA_MOUSE	MK_SCX_29.6766.6766.2	2	3.129	0.348	1	617.9	61.538464	R.LDLAGRDLTDYLMK.I
ACTA_MOUSE	MK_SCX_29.6801.6801.3	3	3.14	0.271	1	457.8	42.307693	R.LDLAGRDLTDYLMK.I
ACTA_MOUSE	MK_SCX_30.4747.4747.3	3	3.349	0.357	1	1000.2	40	K.YPIEHGIITNWDDM*EK.I
ACTA_MOUSE	MK_SCX_30.5282.5282.3	3	4.773	0.37	1	1678.1	48.333332	K.YPIEHGIITNWDDMEK.I
ACTA_MOUSE	MK_SCX_30.5290.5290.2	2	5.039	0.457	1	1390.3	66.66667	K.YPIEHGIITNWDDMEK.I
ACTA_MOUSE	MK_SCX_32.2673.2673.2	2	2.092	0.417	1	432	54.545456	K.DSYVGDEAQSKR.G
ACTA_MOUSE	MK_SCX_32.3706.3706.2	2	2.597	0.166	1	909.8	66.66667	K.QEYDEAGPSIVHR.K
ACTA_MOUSE	MK_SCX_34.3594.3594.3	3	3.455	0.352	1	522.5	42.307693	R.MQKEITALAPSTM*K.I
ACTA_MOUSE	MK_SCX_34.3751.3751.2	2	5.022	0.469	1	2999.7	88.46153	R.MQKEITALAPSTMK.I
ACTA_MOUSE	MK_SCX_34.3767.3767.3	3	4.019	0.418	1	977.9	50	R.MQKEITALAPSTMK.I
ACTA_MOUSE	MK_SCX_35.3396.3396.2	2	3.368	0.338	1	823.9	61.538464	K.QEYDEAGPSIVHRK.C
ACTA_MOUSE	MK_SCX_36.7494.7494.3	3	4.455	0.499	1	1051	36.904762	R.GILTLKYPIEHGIITNWDDM*EK.I
ACTA_MOUSE	MK_SCX_36.8834.8834.3	3	4.348	0.471	1	1055.6	35.714287	R.GILTLKYPIEHGIITNWDDMEK.I
ACTA_MOUSE	MK_SCX_39.4318.4318.2	2	3.023	0.42	1	715	80	R.AVFPSIVGRPR.H

ACTA_MOUSE	MK_SCX_46.3099.3099.3	3	4.67	0.639	1	920.5	40.476192	R.HQGVM*VGM*GQKDSYVGDEAQSK.R
ACTA_MOUSE	MK_SCX_46.3232.3232.2	2	4.233	0.201	1	507.4	52.380955	R.HQGVMVGM*GQKDSYVGDEAQSK.R
ACTA_MOUSE	MK_SCX_46.3408.3408.3	3	4.975	0.138	1	1437.6	44.04762	R.HQGVM*VGMGQKDSYVGDEAQSK.R
ACTA_MOUSE	MK_SCX_46.3675.3675.2	2	5.895	0.679	1	2855.5	69.047615	R.HQGVMVGMGQKDSYVGDEAQSK.R
ACTA_MOUSE	MK_SCX_46.3694.3694.3	3	5.341	0.628	1	1133.8	41.666664	R.HQGVMVGMGQKDSYVGDEAQSK.R
ACTA_MOUSE	MK_SCX_48.3859.3859.3	3	3.173	0.209	1	1112.8	48.333332	R.MQKEITALAPSTM*KIK.I
ACTA_MOUSE	MK_SCX_52.6157.6157.3	3	3.387	0.361	1	673	34.090908	K.RGILTLKYPIEHGIITNWDDM*EK.I
ACTA_MOUSE	MK_SCX_52.6183.6183.2	2	3.799	0.479	1	337.1	43.18182	K.RGILTLKYPIEHGIITNWDDM*EK.I
ACTA_MOUSE	MK_SCX_52.6773.6773.2	2	3.926	0.449	1	370.5	36.363636	K.RGILTLKYPIEHGIITNWDDMEK.I
ACTA_MOUSE	MK_SCX_52.6819.6819.3	3	5.258	0.404	1	1219.4	36.363636	K.RGILTLKYPIEHGIITNWDDMEK.I
ACTA_MOUSE	MK_SCX_54.3089.3089.3	3	5.245	0.144	1	732.3	34.090908	R.HQGVMVGM*GQKDSYVGDEAQSKR.G
ACTA_MOUSE	MK_SCX_54.3274.3274.3	3	5.587	0.153	1	683.1	34.090908	R.HQGVM*VGMGQKDSYVGDEAQSKR.G
ACTA_MOUSE	MK_SCX_54.3407.3407.3	3	5.821	0.591	1	1105.5	40.909092	R.HQGVMVGMGQKDSYVGDEAQSKR.G
ACTB_MOUSE	MK_SCX_15.5931.5931.2	2	5.954	0.718	1	2098.4	62.5	K.DLYANTVLSGGTTM*YPGIADR.M
ACTB_MOUSE	MK_SCX_15.6019.6019.3	3	3.367	0.265	1	490.1	32.5	K.DLYANTVLSGGTTM*YPGIADR.M
ACTB_MOUSE	MK_SCX_15.7066.7066.2	2	6.292	0.623	1	2128.2	70	K.DLYANTVLSGGTTMYPGIADR.M
ACTB_MOUSE	MK_SCX_24.3521.3521.2	2	3.255	0.433	1	1147.2	88.88889	R.GYSFTTTAER.E
ACTB_MOUSE	MK_SCX_26.4527.4527.2	2	4.715	0.263	1	1080.2	67.64706	R.VAPEEHPVLLTEAPLNPK.A
ACTB_MOUSE	MK_SCX_26.4743.4743.3	3	4.408	0.524	1	1014.7	45.588234	R.VAPEEHPVLLTEAPLNPK.A
ACTB_MOUSE	MK_SCX_28.5367.5367.2	2	5.931	0.597	1	1563.9	61.904762	R.KDLYANTVLSGGTTM*YPGIADR.M
ACTB_MOUSE	MK_SCX_28.5393.5393.3	3	6.084	0.59	1	1620	47.61905	R.KDLYANTVLSGGTTM*YPGIADR.M
ACTB_MOUSE	MK_SCX_28.5909.5909.2	2	6.75	0.684	1	2577.3	69.047615	R.KDLYANTVLSGGTTMYPGIADR.M
ACTB_MOUSE	MK_SCX_28.5917.5917.3	3	6.492	0.527	1	1272.2	45.238094	R.KDLYANTVLSGGTTMYPGIADR.M
ACTB_MOUSE	MK_SCX_29.7193.7193.3	3	6.042	0.575	1	2229.8	37.931034	R.TTGIVM*DSGDGVTHTVPIYEGYALPHAILR.L
ACTB_MOUSE	MK_SCX_29.7831.7831.3	3	7.016	0.625	1	2040.4	36.206894	R.TTGIVMDSGDGVTHTVPIYEGYALPHAILR.L
ACTB_MOUSE	MK_SCX_32.3370.3370.3	3	3.065	0.493	1	1172.1	50	K.QEYDESGPSIVHR.K
ACTB_MOUSE	MK_SCX_52.6818.6818.3	3	3.9	0.386	1	734	32.954548	K.RGILTLKYPIEHGIVTNWDDM*EK.I
ACTB_MOUSE	MK_SCX_57.10040.10040.2	2	3.419	0.514	1	1065.4	80	K.IWHHTFYNELR.V
ACTB_MOUSE	MK_SCX_57.9975.9975.3	3	3.343	0.397	1	1027.1	55	K.IWHHTFYNELR.V
ACTN1_MOUSE	MK_SCX_17.3956.3956.2	2	3.706	0.562	1	434.4	56.666668	R.ETADTDTADQVM*ASFK.I
ACTN1_MOUSE	MK_SCX_21.4674.4674.2	2	3.164	0.327	1	605.7	68.181816	R.TINEVENQILTR.D
ACTN1_MOUSE	MK_SCX_2201.7658.7658.2	2	3.642	0.238	1	1008.8	83.33333	K.LASDLLEWIR.R
ACTN1_MOUSE	MK_SCX_2201.8470.8470.2	2	4.504	0.483	1	1684.2	86.36364	R.VGWEQLLTTIAR.T
ACTN1_MOUSE	MK_SCX_39.3191.3191.3	3	3.07	0.265	1	818.3	47.916664	R.LSNRPAFM*PSEGR.M
ACTN1_MOUSE	MK_SCX_39.3608.3608.2	2	3.35	0.373	1	465.2	54.166668	R.LSNRPAFMPSEGR.M
ACTN4_MOUSE	MK_SCX_17.5223.5223.2	2	5.601	0.681	1	2166.9	80	R.ETTDTDTADQVIASFK.V
ACTN4_MOUSE	MK_SCX_18.4310.4310.2	2	3.423	0.418	1	1381.1	85	R.DYETATLSDIK.A
ACTN4_MOUSE	MK_SCX_19.4118.4118.2	2	5.117	0.535	1	1912.7	81.25	R.M*APYQGPDAAPGALDYK.S
ACTN4_MOUSE	MK_SCX_19.4293.4293.2	2	5.341	0.535	1	1071.6	78.125	R.MAPYQGPDAAPGALDYK.S
ACTN4_MOUSE	MK_SCX_19.4597.4597.2	2	5.757	0.549	1	1876.2	70.588234	K.LSGSNPYTTVTPQIINSK.W
ACTN4_MOUSE	MK_SCX_2201.3057.3057.2	2	2.925	0.408	1	620.6	65	K.GISQEQM*QEFR.A
ACTN4_MOUSE	MK_SCX_23.7963.7963.3	3	6.327	0.648	1	1179.5	34.375	R.VEQIAAIAQELNELDYYDSHNVNTR.C
ACTN4_MOUSE	MK_SCX_30.5208.5208.3	3	5.143	0.498	1	1509	50	R.KDDPVTNLNNAFEVAEK.Y
ACTN4_MOUSE	MK_SCX_30.5229.5229.2	2	5.349	0.576	1	2281.1	84.375	R.KDDPVTNLNNAFEVAEK.Y
ACTN4_MOUSE	MK_SCX_32.4841.4841.2	2	4.644	0.5	1	1848.8	78.57143	K.VLAGDKNFITAEELR.R
ACTN4_MOUSE	MK_SCX_32.4895.4895.3	3	4.533	0.366	1	1516.4	50	K.VLAGDKNFITAEELR.R
ACTN4_MOUSE	MK_SCX_37.3845.3845.2	2	3.33	0.191	1	712.2	75	K.QRDYETATLSDIK.A
ACTN4_MOUSE	MK_SCX_39.6021.6021.3	3	3.234	0.41	1	788.6	40.625	R.RQFASQANMVGPWIQTK.M
ACTN4_MOUSE	MK_SCX_49.4468.4468.3	3	3.269	0.361	1	1477.3	50	K.ASIHEAWTDGKEAMLK.Q
ACTN4_MOUSE	MK_SCX_49.4509.4509.2	2	4.749	0.475	1	1652.4	70	K.ASIHEAWTDGKEAMLK.Q
ACTN4_MOUSE	MK_SCX_51.5067.5067.3	3	4.189	0.235	1	965.5	45.588234	R.CQKICDQWDNLGSLTHSR.R

ACTN4_MOUSE	MK_SCX_59.12097.12097.3	3	3.705	0.427	1	969.5	52.272724	R.HRPELIEYDKLR.K
ACTZ_MOUSE	MK_SCX_19.6545.6545.2	2	3.326	0.269	2	1048.9	75	R.VMAGALEGDIFIGPK.A
ACTZ_MOUSE	MK_SCX_21.6946.6946.3	3	6.354	0.616	1	2254.7	45.238094	K.DQLQTFSEEHPVLLTEAPLNPR.K
ACTZ_MOUSE	MK_SCX_32.9960.9960.2	2	2.047	0.13	2	449.1	53.571426	R.VM*AGALEGDIFIGPK.A
ACY2_MOUSE	MK_SCX_20_1.3976.3976.2	2	2.96	0.438	1	342.9	60.000004	K.EM*SEDLPYEVR.R
ACY2_MOUSE	MK_SCX_32.4911.4911.3	3	4.546	0.547	1	1319.4	53.333336	K.YPVGIEVGPQPHGVLR.A
ACY2_MOUSE	MK_SCX_32.4964.4964.2	2	4.59	0.491	1	1495	76.666664	K.YPVGIEVGPQPHGVLR.A
ACY2_MOUSE	MK_SCX_34.4618.4618.2	2	4.121	0.422	1	726.2	79.16667	R.AGLDVKPFITNPR.A
ACY2_MOUSE	MK_SCX_34.4625.4625.3	3	4.153	0.363	1	1031.6	47.916664	R.AGLDVKPFITNPR.A
ACY2_MOUSE	MK_SCX_44.5083.5083.3	3	6.54	0.498	1	3085.9	56.578945	R.SIAKYPVGIEVGPQPHGVLR.A
ACY3_MOUSE	MK_SCX_06.5808.5808.2	2	2.885	0.298	1	629.5	63.333332	K.NGICLEM*GPQPQGVLR.A
ACY3_MOUSE	MK_SCX_11.6755.6755.2	2	4.717	0.593	1	805.6	55.263157	R.SCTLTFLGSTATPDDPYEVK.R
ACY3_MOUSE	MK_SCX_14.10919.10919.3	3	5.032	0.587	1	961.1	32.407406	K.LFSGEDVLYEGDSIVYPVFINEAAYYEK.H
ACY3_MOUSE	MK_SCX_14.9386.9386.2	2	5.414	0.644	1	608.6	42.592594	K.LFSGEDVLYEGDSIVYPVFINEAAYYEK.H
ACY3_MOUSE	MK_SCX_17.6560.6560.2	2	6.546	0.669	1	1604.8	83.33333	R.LFLYEPAGTETFSVESISK.N
ACY3_MOUSE	MK_SCX_2201.3823.3823.2	2	2.324	0.346	1	711.3	87.5	R.ELNQLLGPK.G
ACY3_MOUSE	MK_SCX_23.7210.7210.3	3	5.777	0.574	1	1072.7	31.666666	R.YWLQNPGELQRPSFSAM*PVLANPAATAACCR.Y
ACY3_MOUSE	MK_SCX_23.8019.8019.2	2	2.453	0.344	1	316.3	26.666668	R.YWLQNPGELQRPSFSAMPVLANPAATAACCR.Y
ACY3_MOUSE	MK_SCX_24.8693.8693.3	3	6.944	0.586	1	1892.3	36.666668	R.YWLQNPGELQRPSFSAMPVLANPAATAACCR.Y
ACY3_MOUSE	MK_SCX_25.4303.4303.2	2	2.36	0.383	1	623.5	81.25	R.NLGSVDFPR.T
ACY3_MOUSE	MK_SCX_39.7467.7467.3	3	6.429	0.626	1	1205.3	29.310345	R.TADGDLAGTVHPQLQDHDFEPLRPGEPIFK.L
ACY3_MOUSE	MK_SCX_43.5452.5452.2	2	2.761	0.329	1	721.5	72.22222	K.IRVTVPALLR.L
ACYP1_MOUSE	MK_SCX_2201.5409.5409.2	2	2.224	0.294	1	567.4	63.636364	K.LGLVGWVQNTDR.G
ACYP1_MOUSE	MK_SCX_25.4999.4999.2	2	2.904	0.342	1	835.7	87.5	R.FMQQWLETR.G
ACYP2_MOUSE	MK_SCX_21.2679.2679.2	2	3.12	0.384	1	803.6	62.5	K.GTVTGQVQGPEEK.V
ACYP2_MOUSE	MK_SCX_28.3913.3913.2	2	4.406	0.603	1	1601.5	70.588234	K.GTVTGQVQGPEEKVDAMK.S
ADA10_MOUSE	MK_SCX_19.5201.5201.2	2	2.229	0.263	1	402.2	46.42857	K.AIDTIYQTTDFSGIR.N
ADA10_MOUSE	MK_SCX_31.6856.6856.2	2	2.374	0.156	1	377.1	40.625	K.KM*APSTCASTGSLQWSK.Q
ADA10_MOUSE	MK_SCX_32.3910.3910.3	3	3.11	0.486	1	606.1	44.642857	K.NLGQKENGNYIMYAR.A
ADA10_MOUSE	MK_SCX_32.3935.3935.2	2	3.076	0.334	1	1290.9	67.85714	K.NLGQKENGNYIMYAR.A
ADA10_MOUSE	MK_SCX_57.2496.2496.3	3	3.853	0.438	1	965	54.545456	R.RRPPQPIQQPPR.Q
ADAS_MOUSE	MK_SCX_16.7212.7212.2	2	3.724	0.406	1	604.7	52.380955	K.TSINPSEAPPSIVNEDFLQELK.E
ADAS_MOUSE	MK_SCX_18.3898.3898.2	2	3.515	0.324	1	435.1	40.909092	R.AASAAGASPAATPAAPESGTIPK.K
ADAS_MOUSE	MK_SCX_18.5863.5863.2	2	2.855	0.228	1	307	36.666668	K.WNGWGYNDSKFLLNKK.G
ADAS_MOUSE	MK_SCX_32.3587.3587.3	3	5.596	0.515	1	1480.6	42.391304	R.RAASAAGASPAATPAAPESGTIPK.K
ADAS_MOUSE	MK_SCX_32.7724.7724.2	2	2.6	0.202	1	364.4	50	K.NIYGNIEDLVVHMK.M
ADAS_MOUSE	MK_SCX_34.5359.5359.3	3	4.05	0.464	1	1542.3	53.571426	K.SVKEYVDPSNIFGNR.N
ADAS_MOUSE	MK_SCX_34.5386.5386.2	2	3.489	0.492	1	748.2	64.28571	K.SVKEYVDPSNIFGNR.N
ADCY5_MOUSE	MK_SCX_21.7571.7571.3	3	4.003	0.228	1	521.8	29.545454	R.LLNEIIADFDEIISEDRFRQLEK.I
ADDA_MOUSE	MK_SCX_18.3882.3882.2	2	4.97	0.636	1	895.7	70.588234	K.SPPDQSAVPNTPPSTPVK.L
ADDA_MOUSE	MK_SCX_19.5500.5500.2	2	3.356	0.371	1	475.8	50	R.TSTSAVPNLFVPLNTNPK.E
ADDA_MOUSE	MK_SCX_20_1.5892.5892.3	3	5.166	0.619	1	573.6	29.310345	K.SPPDQSAVPNTPPSTPVKLEEDLPQEPTSR.D
ADDA_MOUSE	MK_SCX_26.6107.6107.3	3	4.797	0.54	1	2365.6	44.04762	K.EYQPHVIVSTTGPNPFNTLTDR.E
ADDA_MOUSE	MK_SCX_26.7202.7202.3	3	4.374	0.581	1	428.4	27.777779	R.SPGTPAGEGSGSPPKWQIGEQEFEALMR.M
ADDA_MOUSE	MK_SCX_29.3123.3123.3	3	3.164	0.445	1	468.3	37.5	R.AAVVTSPPPTTAPHK.E
ADDA_MOUSE	MK_SCX_34.4252.4252.2	2	4.053	0.41	1	1194.6	65.38461	R.YFDRVDENNPEYLR.E
ADDA_MOUSE	MK_SCX_41.3973.3973.2	2	2.065	0.19	1	358.6	55	R.TGYPYRYPALR.E
ADDA_MOUSE	MK_SCX_41.6998.6998.3	3	6.312	0.617	1	1377.2	33.62069	K.SRSPGTPAGEGSGSPPKWQIGEQEFEALMR.M
ADDA_MOUSE	MK_SCX_50.4017.4017.3	3	3.279	0.36	1	590.3	35	R.YFDRVDENNPEYLRER.N
ADDG_MOUSE	MK_SCX_15.7690.7690.2	2	4.351	0.682	1	1702.3	54.166668	K.SDVEIPATVTAFSFEDDSAPLSPLK.F
ADDG_MOUSE	MK_SCX_21.7578.7578.3	3	3.394	0.293	1	417.7	25	K.VSSGTPIKIEDPNQFVPLNTNPTEVLEK.R

ADDG_MOUSE	MK_SCX_25.4434.4434.2	2	2.904	0.461	1	388.2	66.66667	R.WLNSPNTYMK.V
ADDG_MOUSE	MK_SCX_26.7457.7457.3	3	4.194	0.436	1	916.5	30.555555	K.SFTSMDAPVMIMNGKDEMHDVEDELAQR.V
ADDG_MOUSE	MK_SCX_30.7024.7024.3	3	5.3	0.53	1	862.6	33.92857	K.VSSGTPIKIEDPNQFVPLNTNPTEVLEKR.N
ADDG_MOUSE	MK_SCX_34.4732.4732.3	3	3.239	0.191	1	941.7	48.076923	R.YFDRINESDPEYLR.E
ADDG_MOUSE	MK_SCX_38.12614.12614.3	3	3.167	0.388	1	416	26.923079	R.HKSDVEIPATVTAFSFEDDSAPLSPLK.F
ADH1_MOUSE	MK_SCX_21.6108.6108.2	2	2.387	0.233	1	849.9	53.125	K.VCLIGCGFSTGYGSAVK.V
ADH1_MOUSE	MK_SCX_2201.3320.3320.2	2	2.592	0.371	1	334.1	78.57143	K.LVADFM*AK.K
ADH1_MOUSE	MK_SCX_23.5163.5163.2	2	3.547	0.305	1	1330.5	93.75	K.INEAFDLLR.S
ADH1_MOUSE	MK_SCX_24.7606.7606.2	2	3.742	0.428	1	435.9	38	K.IDGASPLDKVCLIGCGFSTGYGSAVK.V
ADH1_MOUSE	MK_SCX_24.7636.7636.3	3	5.754	0.464	1	954.5	34	K.IDGASPLDKVCLIGCGFSTGYGSAVK.V
ADH1_MOUSE	MK_SCX_35.11404.11404.3	3	4.28	0.475	1	1055.2	34.523808	K.AAVLWELHKPFTIEDIEVAPPK.A
ADH1_MOUSE	MK_SCX_44.7901.7901.3	3	3.717	0.42	1	1114.7	51.666664	K.KFPLDPLITHVLPFEK.I
ADH1_MOUSE	MK_SCX_44.7923.7923.2	2	3.788	0.402	1	1475.5	83.33333	K.KFPLDPLITHVLPFEK.I
ADH1_MOUSE	MK_SCX_47.10939.10939.3	3	3.996	0.431	1	608.1	29.166666	K.KFPLDPLITHVLPFEKINEAFDLLR.S
ADHX_MOUSE	MK_SCX_18.6020.6020.2	2	2.774	0.186	1	333.3	43.75	K.AGDTVIPLYIPQCGECK.F
ADHX_MOUSE	MK_SCX_19.5498.5498.2	2	2.924	0.204	1	1036	55.263157	K.VCLLGCGISTGYGAAVNTAK.V
ADHX_MOUSE	MK_SCX_21.7844.7844.3	3	3.885	0.399	1	1412.7	29.464287	K.IDPSAPLDKVCLLGCGISTGYGAAVNTAK.V
ADHX_MOUSE	MK_SCX_21.8394.8394.3	3	3.025	0.266	1	481.1	25.925926	K.VDEFVTGNLSFDQINQAFDLM*HSGDSIR.T
ADHX_MOUSE	MK_SCX_21.9236.9236.3	3	6.383	0.613	1	1125.2	37.962963	K.VDEFVTGNLSFDQINQAFDLMHSGDSIR.T
ADHX_MOUSE	MK_SCX_25.6621.6621.2	2	5.926	0.547	1	1482.6	66.66667	K.AAVAWEAGKPLSIEEIEVAPPK.A
ADHX_MOUSE	MK_SCX_48.6187.6187.3	3	6.96	0.584	1	1331.7	36.53846	K.AAVAWEAGKPLSIEEIEVAPPKAHEVR.I
ADHX_MOUSE	MK_SCX_9.6912.6912.3	3	3.077	0.142	1	440.7	22.321428	R.DPEGCFPVILGHEGAGIVESVGEGVTKLK.A
ADK_MOUSE	MK_SCX_35.4206.4206.2	2	3.307	0.459	1	1197.1	75	K.VAQWLIQEPHK.A
ADK_MOUSE	MK_SCX_51.4135.4135.3	3	6.808	0.483	1	1080.3	33.333336	R.KAADAHVDAHYYEQNEQPTGTCAACITGGNR.S
ADT1_MOUSE	MK_SCX_2201.7275.7275.3	3	3.658	0.206	1	775.5	50	R.YFPTQALNFAFK.D
ADT1_MOUSE	MK_SCX_2201.7390.7390.2	2	4.07	0.357	1	949	90.909096	R.YFPTQALNFAFK.D
ADT1_MOUSE	MK_SCX_24.2486.2486.2	2	2.001	0.284	1	598.6	71.42857	K.TAVAPIER.V
ADT1_MOUSE	MK_SCX_25.6594.6594.2	2	2.478	0.457	1	637.4	75	K.EQGFLSFWR.G
ADT1_MOUSE	MK_SCX_33.6708.6708.2	2	4.513	0.479	1	532.3	76.92308	R.YFPTQALNFAFKDK.Y
ADT1_MOUSE	MK_SCX_49.6640.6640.2	2	5.113	0.497	1	1648.9	73.333336	R.YFPTQALNFAFKDKYK.Q
ADT1_MOUSE	MK_SCX_51.1418.1418.2	2	2.651	0.35	1	440.1	92.85714	R.RMMMQSGR.K
ADT2_MOUSE	MK_SCX_18.5416.5416.2	2	4.571	0.242	1	2623.6	87.5	K.DFLAGGVAAAISK.T
ADT2_MOUSE	MK_SCX_18.7275.7275.1	1	2.989	0.395	1	877.3	66.66667	K.DFLAGGVAAAISK.T
ADT2_MOUSE	MK_SCX_2201.4347.4347.2	2	2.488	0.35	1	761.9	65	R.AAYFGIYDTAK.G
ADT2_MOUSE	MK_SCX_25.5575.5575.2	2	2.874	0.347	1	651	81.25	K.EQGVLSFWR.G
ADT2_MOUSE	MK_SCX_41.5413.5413.3	3	3.347	0.221	1	1471.1	56.81818	R.IPKEQGVLSFWR.G
ADT2_MOUSE	MK_SCX_41.5443.5443.2	2	3.008	0.585	1	1071.1	77.27273	R.IPKEQGVLSFWR.G
ADX_MOUSE	MK_SCX_15.9084.9084.2	2	6.13	0.611	1	2828.2	67.5	K.LDAITDEENDMLDLAFGLTDR.S
ADX_MOUSE	MK_SCX_2201.3127.3127.2	2	3.03	0.497	1	967.4	93.75	R.VPEAVADVR.Q
ADX_MOUSE	MK_SCX_29.4912.4912.3	3	3.289	0.174	1	1130.4	40.625	K.AM*DNMTVRVPEAVADVR.Q
ADX_MOUSE	MK_SCX_29.5400.5400.3	3	4.032	0.447	1	1522.8	46.875	K.AMDNMTVRVPEAVADVR.Q
AFTIN_MOUSE	MK_SCX_17.3535.3535.2	2	3.246	0.472	1	391.8	50	K.SPDPDPTGQNALDDSAASM*K.N
AFTIN_MOUSE	MK_SCX_19.7998.7998.3	3	5.405	0.499	1	1067.8	33.035713	K.TLDPSIDGMESLEDLDKVVVQGPSTGQLR.S
AFTIN_MOUSE	MK_SCX_28.4832.4832.3	3	4.154	0.516	1	776.9	34.523808	K.GAVASGHLQEPGTSVQTALLNR.L
AHSA1_MOUSE	MK_SCX_14.7090.7090.2	2	2.681	0.318	1	479.8	40	K.TEFTQGMILPTVNGESVDPVGQPALK.T
AHSA1_MOUSE	MK_SCX_25.8778.8778.3	3	3.239	0.261	1	646.9	32.142857	R.VFTTQELVQAFTHAPAALEADR.G
AINX_MOUSE	MK_SCX_55.4428.4428.3	3	4.87	0.32	1	1488.6	59.090908	R.HLREYQDLLNVK.M
AIP_MOUSE	MK_SCX_17.4762.4762.2	2	5.203	0.695	1	928.4	63.88889	K.VESPGTYQQDPWAMTDEEK.A
AIP_MOUSE	MK_SCX_18.5843.5843.2	2	2.646	0.331	1	549.4	53.846157	K.VLELDPALAPVVSR.E
AK1A1_MOUSE	MK_SCX_17.7631.7631.2	2	5.25	0.633	1	1786.1	64.70589	R.ILQNIQVFDFTFSPEEM*K.Q
AK1A1_MOUSE	MK_SCX_17.8175.8175.2	2	6.104	0.603	1	2248.8	73.52941	R.ILQNIQVFDFTFSPEEMK.Q

AK1A1_MOUSE	MK_SCX_20_1.5135.5135.2	2	2.572	0.456	1	557.1	80	R.YIVPM*ITVDGK.R
AK1A1_MOUSE	MK_SCX_20_1.5184.5184.2	2	5.235	0.572	1	1852.3	82.14286	R.GLEVTAYSPLGSSDR.A
AK1A1_MOUSE	MK_SCX_20_1.5959.5959.2	2	3.344	0.39	1	759.2	90	R.YIVPMITVDGK.R
AK1A1_MOUSE	MK_SCX_23.8412.8412.3	3	4.521	0.283	1	914.9	33.333336	R.ILQNIQVFDFTFSPEEM*KQLDALNK.N
AK1A1_MOUSE	MK_SCX_23.9152.9152.2	2	4.522	0.517	1	662	43.75	R.ILQNIQVFDFTFSPEEMKQLDALNK.N
AK1A1_MOUSE	MK_SCX_23.9202.9202.3	3	4.866	0.23	1	580.7	30.208334	R.ILQNIQVFDFTFSPEEMKQLDALNK.N
AK1A1_MOUSE	MK_SCX_24.4972.4972.2	2	2.773	0.178	1	888.5	83.33333	K.M*PLIGLGTWK.S
AK1A1_MOUSE	MK_SCX_24.6092.6092.2	2	3.059	0.201	1	1218.6	88.88889	K.MPLIGLGTWK.S
AK1A1_MOUSE	MK_SCX_26.6477.6477.3	3	6.83	0.552	1	794.8	37	R.HIDCASVYGNETEIGEALKESVGSGK.A
AK1A1_MOUSE	MK_SCX_26.7639.7639.2	2	4.837	0.615	1	1470.2	69.44444	R.HPDEPVLLEEPVVLALAEK.H
AK1A1_MOUSE	MK_SCX_26.7751.7751.3	3	6.497	0.518	1	2488.2	51.38889	R.HPDEPVLLEEPVVLALAEK.H
AK1A1_MOUSE	MK_SCX_31.5803.5803.3	3	4.237	0.489	1	951.1	48.4375	K.MPLIGLGTWKSEPGQVK.A
AK1A1_MOUSE	MK_SCX_33.4926.4926.2	2	3.044	0.356	1	826.5	86.36364	R.YIVPMITVDGKR.V
AK1A1_MOUSE	MK_SCX_35.3853.3853.2	2	2.947	0.213	1	814.7	68.181816	K.AVPREELFVTSK.L
AK1A1_MOUSE	MK_SCX_41.7764.7764.2	2	5.813	0.657	1	1530.4	57.14286	R.AWRHPDEPVLLEEPVVLALAEK.H
AK1A1_MOUSE	MK_SCX_42.7850.7850.3	3	6.631	0.59	1	4051.7	57.14286	R.AWRHPDEPVLLEEPVVLALAEK.H
AK1A1_MOUSE	MK_SCX_48.5700.5700.3	3	3.956	0.457	1	922.3	42.1875	K.AVPREELFVTSKLWNTK.H
AK1A1_MOUSE	MK_SCX_50.1649.1649.2	2	2.43	0.124	1	874	100	K.HALSAGYR.H
AK1A1_MOUSE	MK_SCX_54.2802.2802.3	3	3.09	0.203	1	727.7	52.499996	K.HHPEDVEPALR.K
AK1A1_MOUSE	MK_SCX_55.2792.2792.3	3	3.224	0.38	1	897.1	47.727272	K.AAIKHALSAGYR.H
AK1A1_MOUSE	MK_SCX_56.3407.3407.3	3	3.557	0.302	1	1363.9	44.11765	K.NADGTVRYDSTHYKETWK.A
AK1A1_MOUSE	MK_SCX_57.11020.11020.3	3	3.688	0.426	1	755.3	52.499996	K.HGRSPAQILLR.W
AK1A1_MOUSE	MK_SCX_59.2983.2983.3	3	3.942	0.217	1	1017.7	52.272724	K.HHPEDVEPALRK.T
AK1A1_MOUSE	MK_SCX_59.3757.3757.2	2	2.673	0.141	1	367.9	68.181816	K.HHPEDVEPALRK.T
AKA10_MOUSE	MK_SCX_16.5214.5214.2	2	3.548	0.573	1	477.7	47.61905	R.LGDSSSAPLLVTQSEGTDLSSR.T
AKAP2_MOUSE	MK_SCX_13.3324.3324.2	2	5.749	0.705	1	1202.9	59.523808	K.APCVSESQSAGAGPANAATQGK.E
AKAP2_MOUSE	MK_SCX_17.6637.6637.2	2	5.192	0.618	1	612.2	62.5	R.SVNVSLTQEELDSGLDELSVR.S
AKAP2_MOUSE	MK_SCX_2201.4271.4271.2	2	4.581	0.564	1	1356	90.909096	K.LWAEDGEFTSAR.A
AKAP2_MOUSE	MK_SCX_2201.4420.4420.2	2	2.473	0.271	1	477.6	72.22222	R.TLSM*IEEEIR.A
AKAP2_MOUSE	MK_SCX_23.4669.4669.3	3	3.216	0.462	1	490	31.25	K.GPSQPPTAAQPSGPVNMEETRPEGGYFSK.Y
AKAP2_MOUSE	MK_SCX_24.4674.4674.2	2	4.383	0.593	1	387.7	39.285713	K.GPSQPPTAAQPSGPVNMEETRPEGGYFSK.Y
AKAP2_MOUSE	MK_SCX_38.7114.7114.3	3	4.488	0.507	1	1938.3	44.444447	R.AVLTVVKDEDHGILDQFSR.S
AKAP2_MOUSE	MK_SCX_38.7132.7132.2	2	2.376	0.486	1	614	63.636364	K.DEDHGILDQFSR.S
AKAP2_MOUSE	MK_SCX_42.3811.3811.3	3	3.126	0.416	1	379.2	35.714287	K.TIEEQLDEEHLESHR.R
AKAP2_MOUSE	MK_SCX_52.3741.3741.3	3	3.225	0.333	1	353.2	38.46154	K.VRPSEEMIELEKER.R
AKAP8_MOUSE	MK_SCX_13.9327.9327.3	3	5.264	0.619	1	749.5	27.941175	R.NEAAMPTADAGSTLPVIAIPGIMEDELEQTGAEAK.D
AKAP8_MOUSE	MK_SCX_29.7796.7796.3	3	3.213	0.302	1	712.6	33.333336	K.LPDKTVEFLQEYIINR.N
AKAP8_MOUSE	MK_SCX_40.3659.3659.3	3	4.009	0.285	1	2468.2	65	K.FRSFEDEEIQK.H
AKIP_MOUSE	MK_SCX_35.3683.3683.3	3	3.958	0.133	1	1361.8	51.923077	K.AGLKEAPENWQTPK.I
AKT2_MOUSE	MK_SCX_29.4348.4348.2	2	2.273	0.184	1	340	46.153847	K.DPKQRLGGGPSDAK.E
AKT2_MOUSE	MK_SCX_55.16623.16623.2	2	2.019	0.141	1	333.5	61.11111	K.EGISDGATM*K.T
AL1A1_MOUSE	MK_SCX_18.7265.7265.2	2	5.713	0.546	1	1525	84.375	K.GFFVQPTVFSNVTDEMR.I
AL1A1_MOUSE	MK_SCX_31.6051.6051.3	3	4.461	0.33	1	1531.3	51.785713	R.IAKEEIFGPVQQIMK.F
AL3A2_MOUSE	MK_SCX_17.6791.6791.2	2	4.182	0.574	1	989.8	59.375	K.VM*QEEIFGPILPIVSVK.N
AL3A2_MOUSE	MK_SCX_17.7832.7832.2	2	4.828	0.525	1	1358.2	65.625	K.VMQEEIFGPILPIVSVK.N
AL3A2_MOUSE	MK_SCX_18.5429.5429.2	2	4.533	0.48	1	2960.3	89.28571	R.YLAPTILTDVDPNSK.V
AL3A2_MOUSE	MK_SCX_21.4242.4242.2	2	3.366	0.537	1	1142.5	86.36364	K.IAFGGEMDEATR.Y
AL3A2_MOUSE	MK_SCX_21.5248.5248.2	2	3.377	0.445	1	1303.2	81.818184	K.NVDEAINFINDR.E
AL4A1_MOUSE	MK_SCX_15.3981.3981.2	2	2.828	0.36	1	1564.3	85	R.NAAGNFYINDK.S
AL4A1_MOUSE	MK_SCX_16.8815.8815.2	2	5.749	0.598	1	2312	72.22222	K.VGDPAEDFGTFFSAVIDAK.A
AL4A1_MOUSE	MK_SCX_17.15695.15695.2	2	6.004	0.656	1	2760	77.77778	K.LVDSTTSYGLTGAVFAQDK.A

AL4A1_MOUSE	MK_SCX_20_1.5009.5009.3	3	5.164	0.449	1	2267.7	53.333336	K.VANEPILAFSQGSPER.D
AL4A1_MOUSE	MK_SCX_20_1.5176.5176.2	2	5.782	0.56	1	1751.3	73.333336	K.VANEPILAFSQGSPER.D
AL4A1_MOUSE	MK_SCX_2201.3153.3153.2	2	3.064	0.305	1	1058.8	93.75	R.AIDAALAAR.K
AL4A1_MOUSE	MK_SCX_2201.3737.3737.2	2	3.671	0.467	1	936.5	67.85714	K.STGSVVGQQPFGGAR.A
AL4A1_MOUSE	MK_SCX_25.5972.5972.3	3	4.32	0.382	1	916.1	38.636364	K.FAVELEGEQPISVPPSTNHTVYR.G
AL4A1_MOUSE	MK_SCX_26.5178.5178.2	2	4.786	0.554	1	1967.3	60.000004	K.VANEPILAFSQGSPERDALQK.A
AL4A1_MOUSE	MK_SCX_26.5208.5208.3	3	3.254	0.337	1	350.1	30.000002	K.VANEPILAFSQGSPERDALQK.A
AL4A1_MOUSE	MK_SCX_26.9202.9202.2	2	4.126	0.653	1	1072.8	65	R.IKVGDPAEDFGTFFSAVIDAK.A
AL4A1_MOUSE	MK_SCX_26.9415.9415.3	3	5.547	0.466	1	1379	41.25	R.IKVGDPAEDFGTFFSAVIDAK.A
AL4A1_MOUSE	MK_SCX_51.3053.3053.3	3	3.299	0.308	1	503.7	38.333332	R.ASGTNDKPGGPHYILR.W
AL4A1_MOUSE	MK_SCX_56.4881.4881.2	2	4.889	0.588	1	974.3	58.823532	R.WTSPQVIKETHKPLGDWR.Y
AL7A1_MOUSE	MK_SCX_13.4760.4760.2	2	3.886	0.534	1	913.7	62.5	R.GEVITTYCPANNEPIAR.V
AL7A1_MOUSE	MK_SCX_29.6574.6574.2	2	4.592	0.447	1	808.7	65.625	R.VGNPWDPNILYGPLHTK.Q
AL7A1_MOUSE	MK_SCX_29.6625.6625.3	3	4.884	0.436	1	1736.3	54.6875	R.VGNPWDPNILYGPLHTK.Q
AL7A1_MOUSE	MK_SCX_46.7452.7452.3	3	5.412	0.514	1	1137	41.666664	-.STLLIHHPQYAWLQDLGLR.E
AL7A1_MOUSE	MK_SCX_52.3604.3604.3	3	3.191	0.348	1	499.1	39.285713	R.VRQASLKDYEETIGK.A
AL7A1_MOUSE	MK_SCX_52.3640.3640.2	2	4.647	0.576	1	1483.1	78.57143	R.VRQASLKDYEETIGK.A
AL9A1_MOUSE	MK_SCX_20_1.13522.13522.2	2	2.335	0.188	1	410.5	60.714287	R.ANDTTFGLAAGVFTR.D
AL9A1_MOUSE	MK_SCX_20_1.4191.4191.2	2	3.71	0.314	1	1526.3	94.44444	K.LGDPLLEDTR.M
AL9A1_MOUSE	MK_SCX_2201.4279.4279.2	2	3.288	0.373	1	762.2	72.72727	K.ISFTGSVPTGVK.I
AL9A1_MOUSE	MK_SCX_33.5295.5295.2	2	4.101	0.455	1	830.3	81.818184	K.EIADKFINEVVK.Q
AL9A1_MOUSE	MK_SCX_44.6764.6764.3	3	5.105	0.54	1	1937.4	54.6875	R.VFVQKEIADKFINEVVK.Q
ALBU_MOUSE	MK_SCX_13.8579.8579.3	3	3.266	0.289	1	430.4	25	K.CCAEANPPACYGTVLAEFQPLVEEPK.N
ALBU_MOUSE	MK_SCX_13.9152.9152.2	2	4.492	0.543	1	1677.1	84.61539	R.LPCVEDYLSAILNR.V
ALBU_MOUSE	MK_SCX_14.3459.3459.2	2	4.105	0.544	1	1162.2	86.36364	K.YMCENQATISSK.L
ALBU_MOUSE	MK_SCX_17.5624.5624.2	2	3.018	0.426	1	553.1	71.42857	R.LSQTFPNADFAEITK.L
ALBU_MOUSE	MK_SCX_18.9735.9735.2	2	3.28	0.294	1	1937.6	75	K.TVMDDFAQFLDTCCK.A
ALBU_MOUSE	MK_SCX_20_1.4263.4263.2	2	3.557	0.503	1	1089.2	76.92308	K.APQVSTPTLVEAAR.N
ALBU_MOUSE	MK_SCX_21.4276.4276.2	2	3.445	0.18	1	1199	83.33333	K.LVQEVTDFAK.T
ALBU_MOUSE	MK_SCX_21.6186.6186.2	2	4.247	0.467	1	1736.2	79.16667	K.LGEYGFQNAILVR.Y
ALBU_MOUSE	MK_SCX_25.5415.5415.3	3	3.381	0.374	1	460.9	28.40909	K.LVQEVTDFAKTCVADESAANCDK.S
ALBU_MOUSE	MK_SCX_31.4275.4275.2	2	5.201	0.637	1	913.7	73.52941	R.YTQKAPQVSTPTLVEAAR.N
ALBU_MOUSE	MK_SCX_31.4314.4314.3	3	4.355	0.529	1	1024.2	47.058823	R.YTQKAPQVSTPTLVEAAR.N
ALBU_MOUSE	MK_SCX_49.6041.6041.2	2	5.12	0.563	1	1665	76.666664	K.ENPTTFMGHYLHEVAR.R
ALBU_MOUSE	MK_SCX_55.4793.4793.2	2	3.138	0.364	1	765.8	72.72727	R.RHPDYSVSLLLR.L
ALBU_MOUSE	MK_SCX_55.4932.4932.3	3	4.407	0.253	1	2111	65.909096	R.RHPDYSVSLLLR.L
ALDH2_MOUSE	MK_SCX_18.7566.7566.2	2	5.094	0.578	1	1114.2	71.875	K.VAEQTPLTALYVANLIK.E
ALDH2_MOUSE	MK_SCX_19.4826.4826.2	2	4.785	0.587	1	1895.2	80.769226	R.ELGEYGLQAYTEVK.T
ALDH2_MOUSE	MK_SCX_19.6106.6106.2	2	2.378	0.166	1	526.1	59.090908	K.EEIFGPVM*QILK.F
ALDH2_MOUSE	MK_SCX_19.7186.7186.2	2	3.873	0.376	1	1383.7	81.818184	K.EEIFGPVMQILK.F
ALDH2_MOUSE	MK_SCX_20_1.4458.4458.2	2	3.339	0.573	1	1465.4	73.07692	K.LGPALATGNVVVM*K.V
ALDH2_MOUSE	MK_SCX_20_1.6680.6680.2	2	3.967	0.322	1	1027.2	79.16667	R.GYFIQPTVFGDVK.D
ALDH2_MOUSE	MK_SCX_23.4506.4506.2	2	3.734	0.534	1	1511.7	88.88889	K.YGLAAAVFTK.D
ALDH2_MOUSE	MK_SCX_23.8322.8322.3	3	3.866	0.457	1	642.7	36.11111	K.DGM*TIAKEEIFGPVMQILK.F
ALDH2_MOUSE	MK_SCX_23.8352.8352.2	2	5.078	0.549	1	919.9	61.11111	K.DGM*TIAKEEIFGPVMQILK.F
ALDH2_MOUSE	MK_SCX_23.8466.8466.3	3	3.361	0.239	1	570.1	37.5	K.DGMTIAKEEIFGPVM*QILK.F
ALDH2_MOUSE	MK_SCX_23.8566.8566.2	2	3.621	0.468	1	759.1	55.555557	K.DGMTIAKEEIFGPVM*QILK.F
ALDH2_MOUSE	MK_SCX_23.9365.9365.2	2	5.586	0.447	1	2573.3	75	K.DGMTIAKEEIFGPVMQILK.F
ALDH2_MOUSE	MK_SCX_25.5159.5159.2	2	3.363	0.409	1	1216.4	88.88889	R.AAFQLGSPWR.R
ALDH2_MOUSE	MK_SCX_40.4049.4049.2	2	3.074	0.478	1	1022.2	94.44444	K.FKTIEEVVGR.A
ALDOA_MOUSE	MK_SCX_15.5714.5714.2	2	3.933	0.595	1	951.7	56.81818	K.GVVPLAGTNGETTTQGLDGLSER.C

ALDOA_MOUSE	MK_SCX_20_1.3929.3929.2	2	4.24	0.55	1	1799.3	76.92308	K.GILAADESTGSIAK.R
ALDOA_MOUSE	MK_SCX_2201.5529.5529.3	3	4.794	0.544	1	580.4	28	K.VDKGVVPLAGTNGETTTQGLDGLSER.C
ALDOA_MOUSE	MK_SCX_23.5343.5343.2	2	3.74	0.447	1	588.7	44	K.VDKGVVPLAGTNGETTTQGLDGLSER.C
ALDOA_MOUSE	MK_SCX_28.4543.4543.2	2	5.252	0.655	1	2010.1	76.31579	R.IVAPGKGILAADESTGSIAK.R
ALDOA_MOUSE	MK_SCX_29.16422.16422.3	3	5.085	0.32	1	1035.9	39.473686	K.IGEHTPSALAIMENANVLAR.Y
ALDOA_MOUSE	MK_SCX_29.16478.16478.3	3	4.867	0.312	1	1329.3	39.473686	K.IGEHTPSALAIM*ENANVLAR.Y
ALDOA_MOUSE	MK_SCX_29.4710.4710.3	3	5.705	0.641	1	1775.6	53.947372	R.IVAPGKGILAADESTGSIAK.R
ALDOA_MOUSE	MK_SCX_31.7072.7072.2	2	5.711	0.524	1	1629.5	73.68421	K.IGEHTPSALAIMENANVLAR.Y
ALDOA_MOUSE	MK_SCX_33.3610.3610.2	2	4.471	0.571	1	1684.3	75	K.GILAADESTGSIAKR.L
ALDOA_MOUSE	MK_SCX_43.4501.4501.2	2	5.418	0.563	1	1236	62.5	R.IVAPGKGILAADESTGSIAKR.L
ALDOA_MOUSE	MK_SCX_52.3183.3183.3	3	3.3	0.436	1	751	47.916664	K.ENLKAAQEEYIKR.A
ALDOB_MOUSE	MK_SCX_13.5416.5416.3	3	4.189	0.424	1	1069.6	44.736843	R.IADQCPSSLAIQENANALAR.Y
ALDOB_MOUSE	MK_SCX_13.5437.5437.2	2	6.219	0.455	1	2632.8	73.68421	R.IADQCPSSLAIQENANALAR.Y
ALDOB_MOUSE	MK_SCX_20_1.4488.4488.2	2	3.967	0.518	1	1324.3	79.16667	K.ETTIQGLDGLSER.C
ALDOB_MOUSE	MK_SCX_21.15654.15654.2	2	4.683	0.602	1	1838.4	78.57143	K.GILAADESVGTMGNR.L
ALDOB_MOUSE	MK_SCX_21.2804.2804.2	2	3.963	0.473	1	1299.4	83.33333	K.LDQGGAPLAGTNK.E
ALDOB_MOUSE	MK_SCX_21.3930.3930.2	2	5.463	0.575	1	1368.7	75	K.GILAADESVGTM*GNR.L
ALDOB_MOUSE	MK_SCX_2201.2675.2675.2	2	2.277	0.234	1	613.3	85.71429	K.ELSEIAQR.I
ALDOB_MOUSE	MK_SCX_2201.2742.2742.2	2	2.225	0.275	1	484.8	85.71429	K.DSQGNLFR.N
ALDOB_MOUSE	MK_SCX_2201.3788.3788.2	2	2.309	0.17	1	1022.3	87.5	R.FPALTPEQK.K
ALDOB_MOUSE	MK_SCX_2201.4502.4502.2	2	4.981	0.483	1	2964.4	87.5	R.ALQASALAAWGGK.A
ALDOB_MOUSE	MK_SCX_2201.4520.4520.3	3	4.924	0.355	1	2622.2	60.416668	R.ALQASALAAWGGK.A
ALDOB_MOUSE	MK_SCX_23.4914.4914.2	2	5.662	0.658	1	897.3	46	K.LDQGGAPLAGTNKETTIQGLDGLSER.C
ALDOB_MOUSE	MK_SCX_23.5186.5186.3	3	6.274	0.579	1	1186.5	39	K.LDQGGAPLAGTNKETTIQGLDGLSER.C
ALDOB_MOUSE	MK_SCX_26.4351.4351.2	2	3.896	0.259	1	614.7	47.5	R.IVANGKGILAADESVGTM*GNR.L
ALDOB_MOUSE	MK_SCX_26.4360.4360.3	3	3.969	0.235	1	872.6	37.5	R.IVANGKGILAADESVGTM*GNR.L
ALDOB_MOUSE	MK_SCX_26.4688.4688.3	3	3.991	0.449	1	1082.2	36.25	R.IVANGKGILAADESVGTMGNR.L
ALDOB_MOUSE	MK_SCX_28.4847.4847.2	2	4.922	0.591	1	1216.7	57.5	R.IVANGKGILAADESVGTMGNR.L
ALDOB_MOUSE	MK_SCX_28.5416.5416.3	3	3.337	0.403	1	578.4	34.210526	K.GIVVGIKLDQGGAPLAGTNK.E
ALDOB_MOUSE	MK_SCX_29.6024.6024.2	2	4.869	0.663	1	951.9	73.333336	K.YTPEQVAM*ATVTALHR.T
ALDOB_MOUSE	MK_SCX_29.7070.7070.2	2	5.113	0.677	1	1233.3	76.666664	K.YTPEQVAMATVTALHR.T
ALDOB_MOUSE	MK_SCX_37.4179.4179.2	2	2.788	0.394	1	546	87.5	K.DGVDFGKWR.A
ALDOB_MOUSE	MK_SCX_48.5046.5046.2	2	5.333	0.567	1	922.9	81.25	K.KYTPEQVAM*ATVTALHR.T
ALDOB_MOUSE	MK_SCX_48.5056.5056.3	3	5.907	0.63	1	1653	51.5625	K.KYTPEQVAM*ATVTALHR.T
ALDOB_MOUSE	MK_SCX_48.5872.5872.3	3	5.942	0.626	1	1640	51.5625	K.KYTPEQVAMATVTALHR.T
ALDOB_MOUSE	MK_SCX_48.5882.5882.2	2	6.227	0.642	1	2641.6	90.625	K.KYTPEQVAMATVTALHR.T
ALDR_MOUSE	MK_SCX_17.12539.12539.2	2	3.887	0.42	1	749.4	61.11111	K.VFDFEVSSEDMATLLSYNR.N
ALDR_MOUSE	MK_SCX_21.6378.6378.2	2	3.884	0.585	1	1264.3	73.07692	K.TIGVSNFNPLQIER.I
ALDR_MOUSE	MK_SCX_21.6467.6467.3	3	4.446	0.415	1	1864.3	57.692307	K.TIGVSNFNPLQIER.I
ALDR_MOUSE	MK_SCX_21.8979.8979.2	2	2.255	0.196	1	309.6	63.636364	R.FPIQRNLVVIPK.S
ALDR_MOUSE	MK_SCX_24.5260.5260.2	2	2.78	0.354	1	1127.2	83.33333	K.MPTLGLGTWK.S
ALDR_MOUSE	MK_SCX_24.5281.5281.1	1	2.419	0.495	1	322.4	66.66667	K.MPTLGLGTWK.S
ALG5_MOUSE	MK_SCX_34.3641.3641.3	3	3.021	0.22	1	369.6	28.947369	K.ILM*ADADGATKFPDVEKLEK.G
ALG5_MOUSE	MK_SCX_49.7933.7933.3	3	4.899	0.58	1	2366.9	53.333336	K.RLPVMMDEALNYLEKR.Q
AMACR_MOUSE	MK_SCX_16.10312.10312.2	2	4.763	0.431	1	1007.4	52.380955	K.TADGEFMAVGAIEPQFYALLLK.G
AMACR_MOUSE	MK_SCX_16.8585.8585.2	2	3.888	0.47	1	793.3	47.61905	K.TADGEFM*AVGAIEPQFYALLLK.G
AMACR_MOUSE	MK_SCX_17.5349.5349.2	2	3.891	0.571	1	806.4	52.380955	K.GLGLESEELPSQM*SSADWPEM*K.K
AMACR_MOUSE	MK_SCX_17.5900.5900.2	2	4.585	0.463	1	894	54.761906	K.GLGLESEELPSQM*SSADWPEMK.K
AMACR_MOUSE	MK_SCX_17.6440.6440.2	2	5.472	0.614	1	1279.5	64.28571	K.GLGLESEELPSQMSSADWPEMK.K
AMACR_MOUSE	MK_SCX_20_1.6111.6111.2	2	5.685	0.525	1	1848.3	80	R.GQNILDGGAPFYTTYK.T
AMACR_MOUSE	MK_SCX_20_1.6119.6119.2	2	4.875	0.474	1	2018	84.61539	K.LQLGPETLLQDNPK.L

AMACR_MOUSE	MK_SCX_2201.3884.3884.2	2	2.85	0.23	1	620.4	80	R.LGSTGENFLAR.G
AMACR_MOUSE	MK_SCX_30.6043.6043.3	3	4.035	0.321	1	911.3	35.9375	K.VAGHDINYLALSGVLSK.I
AMACR_MOUSE	MK_SCX_30.6068.6068.2	2	5.814	0.566	1	2861.2	81.25	K.VAGHDINYLALSGVLSK.I
AMACR_MOUSE	MK_SCX_50.8919.8919.3	3	4.561	0.458	1	594.4	24.13793	K.AEWCQIFDGTDACVTPVLTFEEALHHQHNR.E
AMACR_MOUSE	MK_SCX_51.3410.3410.3	3	4.429	0.459	1	1810.2	55.76923	K.RDPSVGEHTVEVLR.E
AMACR_MOUSE	MK_SCX_51.3431.3431.2	2	4.315	0.507	1	1321.1	92.30769	K.RDPSVGEHTVEVLR.E
AMNLS_MOUSE	MK_SCX_2201.6944.6944.2	2	2.912	0.394	1	679.8	77.77778	R.NPIFDAIVFK.Q
AMNLS_MOUSE	MK_SCX_37.3830.3830.2	2	3.126	0.533	1	876.9	70.83333	R.VALGPGPNPVHVR.S
AMPE_MOUSE	MK_SCX_16.9670.9670.2	2	4.177	0.43	1	917.8	43.18182	R.IVTIAEPFNTELQLWQMQSFFAK.Y
AMPE_MOUSE	MK_SCX_17.5992.5992.2	2	5.279	0.598	1	1047.7	66.66667	K.ADPSQPPSELGYTWNIPVR.W
AMPE_MOUSE	MK_SCX_17.8123.8123.2	2	3.247	0.354	1	577.9	47.368423	K.IAIPDFGTGAMENWGLVTYR.E
AMPE_MOUSE	MK_SCX_18.5897.5897.2	2	5.237	0.618	1	1774.9	70.588234	R.ETNLLYDPLLSASSNQQR.V
AMPE_MOUSE	MK_SCX_21.4067.4067.2	2	3.054	0.412	1	757.4	72.72727	K.EYSALSNMPEEK.S
AMPE_MOUSE	MK_SCX_21.7418.7418.2	2	2.901	0.546	1	852.7	72.72727	R.SSFIDDAFALAR.A
AMPE_MOUSE	MK_SCX_21.7496.7496.1	1	2.768	0.399	1	312	54.545456	R.SSFIDDAFALAR.A
AMPE_MOUSE	MK_SCX_2201.3258.3258.2	2	3.126	0.469	1	1143.7	88.88889	K.TTYMEDGQIR.S
AMPE_MOUSE	MK_SCX_23.7356.7356.3	3	5.307	0.567	1	1034.1	35.416664	R.FLLDSKADPSQPPSELGYTWNIPVR.W
AMPE_MOUSE	MK_SCX_25.4780.4780.2	2	3.346	0.366	1	1273.4	83.33333	K.NNIEWLNVNR.Q
AMPE_MOUSE	MK_SCX_28.6929.6929.2	2	3.665	0.244	1	1333.4	67.85714	R.LPDFINPVHYDLEVK.A
AMPE_MOUSE	MK_SCX_29.7431.7431.3	3	3.666	0.436	1	522.3	36.764706	R.EQVLETVKNNIEWLNVNR.Q
AMPE_MOUSE	MK_SCX_34.4587.4587.2	2	2.6	0.407	1	742.8	72.72727	K.LLYGLASVKDVK.L
AMPE_MOUSE	MK_SCX_43.8104.8104.3	3	3.847	0.179	1	835	39.705883	K.NFRLPDFINPVHYDLEVK.A
AMPL_MOUSE	MK_SCX_13.8954.8954.2	2	4.903	0.469	1	1682	67.5	K.LNLPINIIGLAPLCENMPSGK.A
AMPL_MOUSE	MK_SCX_21.2814.2814.2	2	2.769	0.423	1	722.4	68.181816	K.TIQVDNTDAEGR.L
AMPL_MOUSE	MK_SCX_21.5235.5235.2	2	3.175	0.523	1	1662.8	86.36364	K.GITFDSGGISIK.A
AMPL_MOUSE	MK_SCX_21.8451.8451.3	3	3.909	0.429	1	526.8	30.357143	K.GSEEPPVFLEIHYM*GSPNATEAPLVFVGK.G
AMPL_MOUSE	MK_SCX_21.8884.8884.2	2	5.393	0.69	1	746.4	39.285713	K.GSEEPPVFLEIHYMGSPNATEAPLVFVGK.G
AMPL_MOUSE	MK_SCX_23.4130.4130.2	2	3.175	0.307	1	986.4	72.72727	K.GVLFASGQNLAR.H
AMPL_MOUSE	MK_SCX_23.6998.6998.2	2	3.213	0.317	1	730.8	92.85714	R.TLIEFLLR.F
AMPL_MOUSE	MK_SCX_24.3475.3475.2	2	2.679	0.391	1	1057.7	87.5	K.ASANMDLMR.A
AMPL_MOUSE	MK_SCX_25.4928.4928.2	2	6.156	0.559	1	1464.3	76.47059	K.DKDDDLPQFTSAGESFNK.L
AMPL_MOUSE	MK_SCX_25.4978.4978.3	3	5.354	0.6	1	1052	44.11765	K.DKDDDLPQFTSAGESFNK.L
AMPL_MOUSE	MK_SCX_28.7369.7369.2	2	5.512	0.583	1	2563.6	69.44444	R.TFYGLHQDFPSVVVVGLGK.R
AMPL_MOUSE	MK_SCX_28.7477.7477.3	3	3.658	0.552	1	1149.7	37.5	R.TFYGLHQDFPSVVVVGLGK.R
AMPL_MOUSE	MK_SCX_32.4370.4370.2	2	4.159	0.438	1	956.6	73.07692	R.EMLNISGPPLKAGK.T
AMPL_MOUSE	MK_SCX_33.6976.6976.2	2	4.451	0.58	1	980.1	52.272724	K.DKDDDLPQFTSAGESFNKLVSGK.L
AMPL_MOUSE	MK_SCX_33.7011.7011.3	3	6.706	0.63	1	2025.9	47.727272	K.DKDDDLPQFTSAGESFNKLVSGK.L
AMPL_MOUSE	MK_SCX_37.2679.2679.3	3	4.242	0.342	1	945.5	53.846157	R.HLM*ESPANEM*TPTR.F
AMPL_MOUSE	MK_SCX_37.3144.3144.3	3	4.654	0.386	1	944	50	R.HLMESPANEM*TPTR.F
AMPL_MOUSE	MK_SCX_37.3152.3152.2	2	3.822	0.557	1	1284.1	80.769226	R.HLMESPANEM*TPTR.F
AMPL_MOUSE	MK_SCX_37.3188.3188.3	3	5.075	0.419	1	1776.1	53.846157	R.HLMESPANEMTPTR.F
AMPL_MOUSE	MK_SCX_37.3262.3262.3	3	3.857	0.418	1	949	50	R.HLM*ESPANEMTPTR.F
AMPL_MOUSE	MK_SCX_37.3537.3537.2	2	4.591	0.599	1	1274.3	80.769226	R.HLMESPANEMTPTR.F
AMPL_MOUSE	MK_SCX_37.5545.5545.2	2	3.593	0.482	1	846.8	79.16667	K.LREMLNISGPPLK.A
AMPL_MOUSE	MK_SCX_38.5237.5237.3	3	3.073	0.31	1	804.3	50	K.LREMLNISGPPLK.A
AMPL_MOUSE	MK_SCX_40.3716.3716.3	3	3.466	0.208	1	821.8	45.454548	K.LHGSGDLEAWEK.G
AMPL_MOUSE	MK_SCX_40.3785.3785.2	2	4	0.622	1	1026.2	86.36364	K.LHGSGDLEAWEK.G
AMPL_MOUSE	MK_SCX_40.4338.4338.2	2	2.191	0.149	1	587.6	70	-.TKGLVLGIYAK.D
AMPL_MOUSE	MK_SCX_43.6958.6958.2	2	5.44	0.451	1	1362	65.789474	R.TFYGLHQDFPSVVVVGLGKR.S
AMPL_MOUSE	MK_SCX_43.6984.6984.3	3	6.005	0.624	1	2831.9	51.315792	R.TFYGLHQDFPSVVVVGLGKR.S
AMPL_MOUSE	MK_SCX_44.7380.7380.3	3	4.109	0.362	1	796.6	40.789474	K.WAHLDIAGVMTNKDEIPYLR.K

AMPL_MOUSE	MK_SCX_45.7066.7066.3	3	4.202	0.35	1	1832.7	42.5	K.TRTFYGLHQDFPSVVVVGLGK.R
AMPL_MOUSE	MK_SCX_48.3218.3218.2	2	5.042	0.453	1	1343	61.764706	R.SAGVDDQENWHEGKENIR.A
AMPL_MOUSE	MK_SCX_48.3267.3267.3	3	4.566	0.505	1	807	48.52941	R.SAGVDDQENWHEGKENIR.A
AMPL_MOUSE	MK_SCX_51.4588.4588.2	2	4.109	0.493	1	1320.4	73.333336	K.LREMLNISGPPLKAGK.T
AMPL_MOUSE	MK_SCX_51.4618.4618.3	3	5.227	0.534	1	1439.4	48.333332	K.LREMLNISGPPLKAGK.T
AMPM2_MOUSE	MK_SCX_24.15437.15437.2	2	2.149	0.306	1	301.9	46.153847	R.EEGTSSTAEEAAKK.K
AMPM2_MOUSE	MK_SCX_25.7224.7224.3	3	4.069	0.527	1	1427.9	38.636364	K.GAVSAVQQELDKESGALVDEVAK.Q
AMPN_MOUSE	MK_SCX_17.12557.12557.2	2	5.067	0.579	1	1936.7	62.5	R.QYEMDSQFQGELADDLAGFYR.S
AMPN_MOUSE	MK_SCX_17.7205.7205.2	2	5.261	0.6	1	1257.8	59.523808	R.WILQM*GFPVITVNTNTGEISQK.H
AMPN_MOUSE	MK_SCX_17.8401.8401.2	2	4.678	0.554	1	1303.4	59.523808	R.WILQMGFPVITVNTNTGEISQK.H
AMPN_MOUSE	MK_SCX_18.6663.6663.2	2	5.473	0.608	1	1560	78.125	K.LQNQLQTDLSVIPVINR.A
AMPN_MOUSE	MK_SCX_18.8777.8777.2	2	4.33	0.418	1	1525.8	70	K.TPDQIMELFDSITYSK.G
AMPN_MOUSE	MK_SCX_18.8876.8876.2	2	5.317	0.642	1	1340.6	57.5	K.LFENYGGGSFSFANLIQGVTR.R
AMPN_MOUSE	MK_SCX_18.9047.9047.2	2	5.302	0.525	1	2227.7	80	K.M*IPITLALDNTLFLVK.E
AMPN_MOUSE	MK_SCX_18.9632.9632.2	2	3.258	0.36	1	473.8	53.333336	K.MIPITLALDNTLFLVK.E
AMPN_MOUSE	MK_SCX_20_1.7951.7951.2	2	3.155	0.52	1	1775.7	90.909096	R.M*LSSFLTEDLFK.K
AMPN_MOUSE	MK_SCX_21.3152.3152.2	2	4.671	0.544	1	2218.5	83.33333	K.VVATTQMQAADAR.K
AMPN_MOUSE	MK_SCX_21.3728.3728.3	3	3.666	0.204	1	1517	50	K.AVNQQTAVQPPATVR.T
AMPN_MOUSE	MK_SCX_21.3756.3756.2	2	4.127	0.295	1	1362.7	78.57143	K.AVNQQTAVQPPATVR.T
AMPN_MOUSE	MK_SCX_21.7407.7407.2	2	4.91	0.307	1	2682.2	80.769226	R.FSSEFELQQLEQFK.A
AMPN_MOUSE	MK_SCX_2201.2548.2548.2	2	2.321	0.247	1	643.2	81.25	R.SEYMEGDVK.K
AMPN_MOUSE	MK_SCX_2201.5492.5492.2	2	4.016	0.434	1	599	63.636364	R.YLSYTLNPDYIR.K
AMPN_MOUSE	MK_SCX_23.10071.10071.3	3	4.113	0.46	1	617.7	28.125	K.QDTTSTIISIASNVAGHPLVWDFVR.S
AMPN_MOUSE	MK_SCX_25.15156.15156.3	3	5.151	0.553	1	1471.9	33.653847	R.FSSEFELQQLEQFKADNSATGFGTGTR.A
AMPN_MOUSE	MK_SCX_25.5748.5748.3	3	3.331	0.379	1	310.4	27.5	R.VMAVDALASSHPLSSPADEIK.T
AMPN_MOUSE	MK_SCX_26.4440.4440.2	2	4.497	0.554	1	984.8	61.764706	R.TLDGTPAPNIDKTELVER.T
AMPN_MOUSE	MK_SCX_32.6864.6864.2	2	6.246	0.449	1	1740.2	73.52941	K.KLQNQLQTDLSVIPVINR.A
AMPN_MOUSE	MK_SCX_33.4363.4363.3	3	4.286	0.476	1	925.6	51.923077	R.AQIIHDSFNLASAK.M
AMPN_MOUSE	MK_SCX_34.4180.4180.2	2	3.86	0.504	1	1315.1	76.92308	R.AQIIHDSFNLASAK.M
AMPN_MOUSE	MK_SCX_38.2926.2926.3	3	3.889	0.237	1	1521.2	50	K.KVVATTQMQAADAR.K
AMPN_MOUSE	MK_SCX_41.6660.6660.3	3	3.226	0.357	1	557.5	32.142857	R.RFSSEFELQQLEQFK.A
AMPN_MOUSE	MK_SCX_43.10584.10584.3	3	4.189	0.436	1	365.3	24.074074	R.RFSSEFELQQLEQFKADNSATGFGTGTR.A
AMPN_MOUSE	MK_SCX_49.4696.4696.3	3	3.912	0.497	1	763.3	44.642857	R.ANIDWVKENKDAVFK.W
AMPN_MOUSE	MK_SCX_49.4724.4724.2	2	4.419	0.544	1	1787	71.42857	R.ANIDWVKENKDAVFK.W
AMRP_MOUSE	MK_SCX_20_1.7201.7201.2	2	5.039	0.449	1	2278	87.5	K.IQEYNVLLDTLSR.A
AMRP_MOUSE	MK_SCX_20_1.7377.7377.3	3	3.246	0.194	1	726.6	45.833336	K.IQEYNVLLDTLSR.A
AMRP_MOUSE	MK_SCX_23.3874.3874.3	3	5.344	0.34	1	628.1	35.714287	K.DAQM*VHSNALNEDTQDELGDPR.L
AMRP_MOUSE	MK_SCX_23.4243.4243.3	3	6.175	0.516	1	1486	46.42857	K.DAQMVHSNALNEDTQDELGDPR.L
AMRP_MOUSE	MK_SCX_23.4251.4251.2	2	6.167	0.673	1	2585.6	64.28571	K.DAQMVHSNALNEDTQDELGDPR.L
AMRP_MOUSE	MK_SCX_27.4819.4819.3	3	3.929	0.435	1	877.6	45.588234	R.AEEGYENLLSPSDM*AHIK.S
AMRP_MOUSE	MK_SCX_27.5875.5875.2	2	5.309	0.416	1	964.3	61.764706	R.AEEGYENLLSPSDMAHIK.S
AMRP_MOUSE	MK_SCX_27.5996.5996.3	3	4.069	0.496	1	831.8	44.11765	R.AEEGYENLLSPSDMAHIK.S
AMRP_MOUSE	MK_SCX_36.3484.3484.3	3	3.905	0.44	1	1215.7	55	K.VSHQGYGSTTEFEEPR.V
AMRP_MOUSE	MK_SCX_36.3505.3505.2	2	5.288	0.615	1	1587.7	73.333336	K.VSHQGYGSTTEFEEPR.V
AMRP_MOUSE	MK_SCX_37.6134.6134.2	2	3.585	0.39	1	962.5	80	K.FSSEELDKLWR.E
AMRP_MOUSE	MK_SCX_39.5385.5385.3	3	3.478	0.293	1	663.9	47.5	K.LIHNLNVILAR.Y
AMRP_MOUSE	MK_SCX_39.5463.5463.2	2	2.901	0.375	1	550	75	K.LIHNLNVILAR.Y
AMRP_MOUSE	MK_SCX_41.3656.3656.3	3	4.064	0.446	1	434.4	39.772728	R.KDAQM*VHSNALNEDTQDELGDPR.L
AMRP_MOUSE	MK_SCX_41.3998.3998.3	3	5.256	0.622	1	810.6	43.18182	R.KDAQMVHSNALNEDTQDELGDPR.L
AMRP_MOUSE	MK_SCX_41.4235.4235.3	3	3.663	0.253	1	877.7	63.88889	R.MEKLNQLWEK.A
AMRP_MOUSE	MK_SCX_41.4238.4238.2	2	3.307	0.342	1	1102.1	83.33333	R.MEKLNQLWEK.A

AMRP_MOUSE	MK_SCX_52.3016.3016.3	3	4.202	0.584	1	1338.1	56.25	K.HVESIGDPEHISR.N
AMRP_MOUSE	MK_SCX_52.4934.4934.2	2	4.072	0.532	1	1793.1	75	K.TSGKFSSEELDKLWR.E
AMRP_MOUSE	MK_SCX_53.3157.3157.3	3	4.719	0.592	1	1567.4	54.6875	R.KVSHQGYGSTTEFEEPR.V
AMRP_MOUSE	MK_SCX_53.3173.3173.2	2	5.008	0.656	1	2468.6	81.25	R.KVSHQGYGSTTEFEEPR.V
AMRP_MOUSE	MK_SCX_56.3161.3161.3	3	3.847	0.398	1	1124.2	53.571426	K.LKHVESIGDPEHISR.N
AMRP_MOUSE	MK_SCX_59.7427.7427.2	2	2.235	0.284	1	311.1	78.57143	K.RLHLSPVR.L
AN32A_MOUSE	MK_SCX_35.4232.4232.3	3	3.266	0.205	1	335.4	52.499996	K.IKDLSTIEPLK.K
AN32A_MOUSE	MK_SCX_49.1421.1421.2	2	2.161	0.228	1	537	78.57143	K.HLNLSGNK.I
AN32E_MOUSE	MK_SCX_34.5178.5178.3	3	3.59	0.204	1	1472.9	50	K.IKDLSTVEALQNLK.N
AN32E_MOUSE	MK_SCX_34.5216.5216.2	2	5.307	0.506	1	2634.8	84.61539	K.IKDLSTVEALQNLK.N
ANRE_MOUSE	MK_SCX_14.7046.7046.2	2	5.408	0.58	1	1389.5	46.153847	K.APLEDYTDDDLSTDSEQIMDFTPAANK.Q
ANRE_MOUSE	MK_SCX_14.7055.7055.3	3	3.641	0.422	1	330.9	21.153847	K.APLEDYTDDDLSTDSEQIMDFTPAANK.Q
ANS4B_MOUSE	MK_SCX_36.5219.5219.2	2	3.02	0.356	1	461.7	69.230774	R.YHQAASDSYLELLK.E
ANS4B_MOUSE	MK_SCX_47.2984.2984.3	3	5.209	0.641	1	1868.8	48.61111	R.TYSKEDSGTISSSHSTLSR.S
ANT3_MOUSE	MK_SCX_31.4101.4101.2	2	3.68	0.475	1	744.9	73.07692	K.LQPLDFKENPEQSR.V
ANX11_MOUSE	MK_SCX_48.3744.3744.2	2	4.755	0.508	1	2056.3	73.333336	K.SLYHDITGDTSGDYRK.I
ANX13_MOUSE	MK_SCX_19.6087.6087.2	2	2.084	0.201	1	425.9	50	K.GM*GTDEAAIIEVLSSR.T
ANX13_MOUSE	MK_SCX_23.3685.3685.3	3	3.949	0.265	1	1014.6	45	R.DEEDTVDKELAGQDAK.D
ANX13_MOUSE	MK_SCX_31.3960.3960.2	2	3.886	0.381	1	490.3	53.571426	R.SLESDVKEDTSGNLR.K
ANX13_MOUSE	MK_SCX_33.4680.4680.3	3	3.389	0.399	1	778.9	41.07143	K.TALALLDRPNEYAAR.Q
ANX13_MOUSE	MK_SCX_33.4713.4713.2	2	3.561	0.394	1	542.4	53.571426	K.TALALLDRPNEYAAR.Q
ANX13_MOUSE	MK_SCX_45.4241.4241.3	3	3.91	0.586	1	440.1	46.666668	K.SLSDMVHSDTSGDFRK.L
ANX13_MOUSE	MK_SCX_45.6762.6762.3	3	4.797	0.53	1	1174.6	40.27778	K.YGKDLEEVLNSELSGNFKK.T
ANXA1_MOUSE	MK_SCX_18.8228.8228.2	2	3.597	0.435	1	673.7	56.666668	K.GLGTDEDTLIEILTTR.S
ANXA1_MOUSE	MK_SCX_20_1.4869.4869.2	2	3.267	0.316	1	301.8	57.692307	R.FLENQEQEYVQAVK.S
ANXA1_MOUSE	MK_SCX_29.7700.7700.2	2	2.734	0.319	1	468.6	53.846157	K.GVDEATIIDILTKR.T
ANXA1_MOUSE	MK_SCX_32.4645.4645.3	3	3.188	0.293	1	510.6	37.5	K.TPAQFDADELRGAMK.G
ANXA2_MOUSE	MK_SCX_17.5335.5335.2	2	4.657	0.481	1	1339.8	62.5	R.AEDGSVIDYELIDQDAR.E
ANXA2_MOUSE	MK_SCX_19.6781.6781.2	2	3.335	0.19	1	662.1	61.538464	K.GVDEVTIVNILTNR.S
ANXA2_MOUSE	MK_SCX_2201.4474.4474.2	2	3.569	0.433	1	1699.1	85	K.SLYYYIQQDTK.G
ANXA2_MOUSE	MK_SCX_27.5791.5791.2	2	4.658	0.56	1	1638.2	73.333336	K.TDLEKDIISDTSGDFR.K
ANXA2_MOUSE	MK_SCX_27.5931.5931.3	3	3.647	0.442	1	1240.3	43.333332	K.TDLEKDIISDTSGDFR.K
ANXA2_MOUSE	MK_SCX_29.5202.5202.2	2	5.306	0.492	1	1890.6	73.52941	R.RAEDGSVIDYELIDQDAR.E
ANXA2_MOUSE	MK_SCX_29.5243.5243.3	3	4.103	0.375	1	1942.9	48.52941	R.RAEDGSVIDYELIDQDAR.E
ANXA2_MOUSE	MK_SCX_32.6720.6720.3	3	4.026	0.427	1	2234.5	50	K.TKGVDEVTIVNILTNR.S
ANXA2_MOUSE	MK_SCX_41.5130.5130.3	3	4.552	0.482	1	2201.1	53.125	K.TDLEKDIISDTSGDFRK.L
ANXA2_MOUSE	MK_SCX_41.5167.5167.2	2	5.068	0.621	1	2120.4	71.875	K.TDLEKDIISDTSGDFRK.L
ANXA3_MOUSE	MK_SCX_18.7260.7260.2	2	2.702	0.222	1	542.2	66.66667	K.GTGTDEDALIEILTTR.S
ANXA3_MOUSE	MK_SCX_29.5198.5198.3	3	3.456	0.376	1	693.1	37.5	R.GTIKDYPGFSPSVDAEAIR.K
ANXA3_MOUSE	MK_SCX_30.4141.4141.2	2	3.397	0.489	1	556.2	63.333332	K.SLGDDISSETSGDFRK.A
ANXA3_MOUSE	MK_SCX_42.4824.4824.3	3	4.509	0.518	1	1271.4	42.105263	R.GTIKDYPGFSPSVDAEAIRK.A
ANXA4_MOUSE	MK_SCX_13.7216.7216.2	2	4.013	0.303	1	737.3	60.000004	K.GAGTDEGCLIEILASR.T
ANXA4_MOUSE	MK_SCX_17.4962.4962.1	1	3.124	0.439	1	432.9	59.090908	R.DEGNYLDDALMK.Q
ANXA4_MOUSE	MK_SCX_18.3872.3872.2	2	3.754	0.475	1	1373.6	71.42857	K.AASGFNATEDAQTLR.K
ANXA4_MOUSE	MK_SCX_18.7534.7534.2	2	5.112	0.606	1	2158.7	76.666664	K.GLGTDEDAIIGILAYR.N
ANXA4_MOUSE	MK_SCX_19.7738.7738.2	2	4.304	0.443	1	1335.1	70	K.SETSGSFEDALLAIVK.C
ANXA4_MOUSE	MK_SCX_33.3575.3575.2	2	5.221	0.465	1	1002.3	63.333332	K.AASGFNATEDAQTLRK.A
ANXA4_MOUSE	MK_SCX_38.3691.3691.3	3	4.366	0.342	1	1028.6	70	R.ISQKDIEQSIK.S
ANXA4_MOUSE	MK_SCX_38.3705.3705.2	2	3.615	0.332	1	1559.3	90	R.ISQKDIEQSIK.S
ANXA5_MOUSE	MK_SCX_16.6083.6083.2	2	6.215	0.689	1	1532.3	56.25	K.QVYEEEYGSNLEDDVVGDTSGYYQR.M
ANXA5_MOUSE	MK_SCX_16.6089.6089.3	3	4.916	0.494	1	978	34.375	K.QVYEEEYGSNLEDDVVGDTSGYYQR.M

ANXA5_MOUSE	MK_SCX_18.7766.7766.2	2	5.856	0.565	1	2262.6	80	K.GLGTDEDSILNLLTSR.S
ANXA5_MOUSE	MK_SCX_18.7898.7898.2	2	2.306	0.136	1	326.8	41.17647	R.ETSGNLEQLLLAVVKSIR.S
ANXA5_MOUSE	MK_SCX_21.4631.4631.2	2	2.073	0.348	1	397.7	59.090908	R.GTVTDFPGFDGR.A
ANXA5_MOUSE	MK_SCX_21.8046.8046.3	3	6.217	0.683	1	2450.7	35.60606	R.TPEELSAIKQVYEEEYGSNLEDDVVGDTSGYYQR.M
ANXA5_MOUSE	MK_SCX_2201.3422.3422.2	2	2.528	0.265	1	483.8	87.5	R.TPEELSAIK.Q
ANXA5_MOUSE	MK_SCX_23.6955.6955.2	2	2.87	0.323	1	897.3	70	K.NFATSLYSMIK.G
ANXA5_MOUSE	MK_SCX_25.7005.7005.2	2	4.193	0.473	1	1785.3	83.33333	R.DLVDDLKSELTGK.F
ANXA5_MOUSE	MK_SCX_32.7304.7304.3	3	3.781	0.477	1	991.4	42.857143	K.WGTDEEKFITIFGTR.S
ANXA5_MOUSE	MK_SCX_35.11400.11400.3	3	4.267	0.564	1	494.7	43.333332	R.DLVDDLKSELTGKFEK.L
ANXA5_MOUSE	MK_SCX_36.8216.8216.2	2	4.369	0.51	1	920.3	66.66667	R.DLVDDLKSELTGKFEK.L
ANXA5_MOUSE	MK_SCX_37.3884.3884.2	2	2.901	0.452	1	1349.1	94.44444	K.LIVAMMKPSR.L
ANXA5_MOUSE	MK_SCX_42.5764.5764.2	2	3.982	0.383	1	1201	81.818184	R.KNFATSLYSMIK.G
ANXA5_MOUSE	MK_SCX_42.7931.7931.3	3	4.129	0.439	1	1349	44.11765	K.TLFGRDLVDDLKSELTGK.F
ANXA6_MOUSE	MK_SCX_25.7247.7247.3	3	4.325	0.47	1	1030.1	32.954548	R.GSVHDFPEFDANQDAEALYTAMK.G
ANXA6_MOUSE	MK_SCX_28.6416.6416.3	3	3.532	0.463	1	450.7	35.714287	R.LILGLMMPPAHYDAK.Q
ANXA6_MOUSE	MK_SCX_31.7383.7383.2	2	3.89	0.468	1	1671.5	76.666664	K.GFGSDKESILELITSR.S
ANXA6_MOUSE	MK_SCX_33.4239.4239.2	2	5.079	0.578	1	1931.9	76.666664	K.SLHQAIEGDTSGDFMK.A
ANXA6_MOUSE	MK_SCX_43.8317.8317.3	3	3.307	0.489	1	531.4	32.352943	K.SLYGKDLIEDLKYELTGK.F
ANXA7_MOUSE	MK_SCX_19.5751.5751.2	2	4.69	0.516	1	2133.3	73.333336	K.GFGTDEQAIVDVVSNR.S
AOC3_MOUSE	MK_SCX_26.10117.10117.3	3	4.32	0.536	1	894.5	31	R.THPSQSQPFADLSPEELTAVMSFLTK.H
AP1B1_MOUSE	MK_SCX_15.9266.9266.2	2	5.463	0.638	1	1650	46.551723	R.LGAPISSGLSDLFDLTSGVGTLSGSYVAPK.A
AP1B1_MOUSE	MK_SCX_15.9402.9402.3	3	5.554	0.597	1	934.1	30.172413	R.LGAPISSGLSDLFDLTSGVGTLSGSYVAPK.A
AP1B1_MOUSE	MK_SCX_19.9781.9781.3	3	5.171	0.462	1	337.5	22.916668	R.NSFGLAPAAPLQVHVPLSPNQTVEISLPLNTVGSVLK.M
AP1G1_MOUSE	MK_SCX_15.8630.8630.3	3	4.506	0.387	1	1086	30.769232	K.TFQLQLLSPSSSVVPAFNTGTITQVIK.V
AP1G1_MOUSE	MK_SCX_16.8664.8664.2	2	5.232	0.655	1	853.2	48.076923	K.TFQLQLLSPSSSVVPAFNTGTITQVIK.V
AP1M1_MOUSE	MK_SCX_20_1.8053.8053.2	2	2.936	0.407	1	802.3	69.230774	K.FEIPYFTTSGIQVR.Y
AP1M1_MOUSE	MK_SCX_2201.5466.5466.2	2	2.048	0.149	1	1300.7	88.88889	R.VFLSGMPELR.L
AP1M1_MOUSE	MK_SCX_25.5049.5049.2	2	3.233	0.34	1	585.1	77.77778	K.SGYQALPWVR.Y
AP1S1_MOUSE	MK_SCX_18.4433.4433.2	2	5.503	0.582	1	2155.6	73.333336	K.AIEQADLLQEEDESPR.S
AP1S1_MOUSE	MK_SCX_25.5853.5853.2	2	2.244	0.298	1	708.2	91.66667	R.FMLLFSR.Q
AP2M1_MOUSE	MK_SCX_31.7432.7432.3	3	4.297	0.346	1	696.3	35.526314	K.WARPPISM*NFEVPFAPSGLK.V
AP2M1_MOUSE	MK_SCX_31.8358.8358.2	2	3.94	0.469	1	750.7	55.263157	K.WARPPISMNFEVPFAPSGLK.V
AP2M1_MOUSE	MK_SCX_31.8434.8434.3	3	4.672	0.34	1	1232.2	43.421055	K.WARPPISMNFEVPFAPSGLK.V
AP2M1_MOUSE	MK_SCX_43.3811.3811.2	2	2.653	0.313	1	631.6	70	K.YKASENAIVWK.I
AP2M1_MOUSE	MK_SCX_50.7608.7608.3	3	3.506	0.229	1	704.6	35	K.KWARPPISMNFEVPFAPSGLK.V
AP3B1_MOUSE	MK_SCX_13.7387.7387.3	3	3.193	0.384	1	409.9	29.347824	R.VPIIVPVM*M*LAIKEASADLSPYVR.K
AP3B1_MOUSE	MK_SCX_2201.2768.2768.2	2	2.228	0.207	1	320.4	62.5	R.NVEVIESAK.E
AP3B1_MOUSE	MK_SCX_25.7528.7528.3	3	3.162	0.206	1	312	17.5	K.KEQGTLTGM*NETSATLIAAPQNFTPSMILQK.V
AP3B1_MOUSE	MK_SCX_28.4342.4342.3	3	4.308	0.52	1	1282	48.61111	K.VVNVANLGAVPSSQDNVHR.F
AP3D1_MOUSE	MK_SCX_33.6173.6173.3	3	4.828	0.503	1	972.5	35.526314	R.ALDIDLDKPLADSEKLPVQK.H
AP3D1_MOUSE	MK_SCX_38.8175.8175.3	3	3.231	0.151	1	340.5	24	K.AIRKFAVSQMSSLLDSAHLVASSTQR.N
AP3D1_MOUSE	MK_SCX_45.3497.3497.3	3	5.13	0.5	1	1180.7	42.647057	R.NAEAVKSPEKEGVLGVEK.K
AP3M1_MOUSE	MK_SCX_26.5778.5778.3	3	5.442	0.531	1	1176.1	37.5	K.GLVNLQSGAPKPEENPNLNIQFK.I
APBA3_MOUSE	MK_SCX_15.3762.3762.2	2	4.607	0.602	1	435	52.63158	R.DEASSPAQSVPQDPAQTAPR.L
APEX1_MOUSE	MK_SCX_17.3836.3836.2	2	4.627	0.587	1	1208.5	68.75	K.EAAGEGPVLYEDPPDQK.T
APEX1_MOUSE	MK_SCX_19.8266.8266.2	2	4.809	0.575	1	633.6	62.5	R.QGFGELLQAVPLADSFR.H
APEX1_MOUSE	MK_SCX_24.3883.3883.3	3	3.68	0.568	1	790	37.5	K.EAAGEGPVLYEDPPDQKTSPSGK.S
APEX1_MOUSE	MK_SCX_27.3892.3892.3	3	3.751	0.342	1	655.2	34.210526	K.TEKEAAGEGPVLYEDPPDQK.T
APEX1_MOUSE	MK_SCX_44.3531.3531.3	3	4.305	0.412	1	585.4	41.25	K.KTEKEAAGEGPVLYEDPPDQK.T
API5_MOUSE	MK_SCX_19.9005.9005.2	2	5.439	0.537	1	2084	70.588234	K.GTLGGLFSQILQGEDIVR.E
API5_MOUSE	MK_SCX_30.4780.4780.2	2	2.301	0.232	1	505.8	63.636364	K.TEENKIKVVALK.I

APMAP_MOUSE	MK_SCX_15.9042.9042.2	2	5.014	0.63	1	1072.8	46.153847	R.LFENQLSGPESIVNIGDVLFTGTADGR.V
APMAP_MOUSE	MK_SCX_15.9050.9050.3	3	5.481	0.693	1	1306.7	35.576923	R.LFENQLSGPESIVNIGDVLFTGTADGR.V
APMAP_MOUSE	MK_SCX_20_1.4410.4410.2	2	2.377	0.23	1	525.4	72.72727	K.LLLSSETPIEGK.K
APMAP_MOUSE	MK_SCX_51.3909.3909.3	3	3.81	0.455	1	1059.1	42.647057	R.RPLRPQVVTDDGQVPEVK.E
APOA1_MOUSE	MK_SCX_35.3943.3943.2	2	3.214	0.32	1	509	85	K.VQPYLDEFQKK.W
APOA1_MOUSE	MK_SCX_36.4330.4330.3	3	3.198	0.29	1	505.1	39.583336	K.VKDFANVYVDAVK.D
APOA1_MOUSE	MK_SCX_38.4158.4158.2	2	2.91	0.197	1	581.8	75	R.HSLM*PMLETLK.T
APOA1_MOUSE	MK_SCX_38.4715.4715.3	3	3.309	0.407	1	946	60.000004	R.HSLMPM*LETLK.T
APOA1_MOUSE	MK_SCX_38.4719.4719.2	2	2.689	0.243	1	309.2	70	R.HSLMPM*LETLK.T
APOA1_MOUSE	MK_SCX_38.5762.5762.3	3	3.89	0.199	1	827	52.499996	R.HSLMPMLETLK.T
APOA1_MOUSE	MK_SCX_38.5826.5826.2	2	3.006	0.481	1	364.6	75	R.HSLMPMLETLK.T
APOA1_MOUSE	MK_SCX_43.4125.4125.3	3	3.611	0.184	1	797.9	53.125	K.WKEDVELYR.Q
APOA1_MOUSE	MK_SCX_43.4170.4170.2	2	3.047	0.46	1	738.1	93.75	K.WKEDVELYR.Q
APOC3_MOUSE	MK_SCX_17.15716.15716.2	2	4.032	0.432	1	1228.3	61.11111	K.TVQDALSSVQESDIAVVAR.G
APOE_MOUSE	MK_SCX_34.3947.3947.3	3	3.419	0.29	1	371.5	37.5	R.NEVHTMLGQSTEEIR.A
APOE_MOUSE	MK_SCX_49.4335.4335.3	3	4.636	0.455	1	1650.8	47.058823	K.AYKKELEEQLGPVAEETR.A
AQP1_MOUSE	MK_SCX_17.5307.5307.2	2	6.632	0.585	1	3229.7	71.05263	K.VWTSGQVEEYDLDADDINSR.V
ARC1B_MOUSE	MK_SCX_15.4531.4531.2	2	3.542	0.358	1	1972.9	83.33333	K.NSVSQISVLSGGK.A
ARC1B_MOUSE	MK_SCX_38.4454.4454.2	2	2.44	0.233	1	524.4	77.77778	K.SLESALKDLK.I
ARF1_MOUSE	MK_SCX_2201.3494.3494.2	2	2.753	0.338	1	616.9	92.85714	R.MLAEDELR.D
ARF1_MOUSE	MK_SCX_26.9066.9066.3	3	4.064	0.235	1	1897.9	47.058823	R.M*LAEDELRDAVLLVFANK.Q
ARFG1_MOUSE	MK_SCX_19.4948.4948.2	2	5.468	0.582	1	2814.2	89.28571	R.IFDDVSSGVSQLASK.V
ARFG1_MOUSE	MK_SCX_25.6200.6200.3	3	4.736	0.589	1	565.8	30.000002	K.VATLAEGKEWSLESSPAQNWTPPQPK.T
ARFG1_MOUSE	MK_SCX_43.4184.4184.3	3	4.947	0.523	1	1834	45.833336	K.ASELGHSLNENVLKPAQEK.V
ARFG3_MOUSE	MK_SCX_53.3318.3318.3	3	3.936	0.432	1	1800.3	53.125	R.RKYQEDPEDSYFSSSSK.W
ARFG3_MOUSE	MK_SCX_57.2619.2619.3	3	4.677	0.54	1	1828.5	51.5625	R.RKPEYEPIGSTDEAQKK.F
ARHGC_MOUSE	MK_SCX_18.4145.4145.2	2	3.971	0.447	1	1152	61.11111	R.SEGVQDAEPQSLVGSPSTR.G
ARHGC_MOUSE	MK_SCX_27.3130.3130.3	3	3.209	0.364	1	592.6	35.526314	R.TDWSSGDASRPSSDSADSPK.S
ARHGC_MOUSE	MK_SCX_38.4012.4012.3	3	3.85	0.147	1	1084.5	50	R.SSTMQYVILM*YM*K.Y
ARHGC_MOUSE	MK_SCX_49.4181.4181.2	2	2.194	0.377	1	593.4	72.72727	K.RRGFPSILGPPR.R
ARHGG_MOUSE	MK_SCX_17.6371.6371.3	3	5.308	0.61	1	1383.4	29.605263	R.ANAAFEPDASEPLPPPPSPEDEEPPRPIVLSTQSPAALK.M
ARI2_MOUSE	MK_SCX_36.3304.3304.3	3	4.999	0.42	1	2932.9	56.25	R.YKENPDIVNQSQQAQAR.E
ARK72_MOUSE	MK_SCX_20_1.7340.7340.2	2	4.606	0.549	1	1558.7	85.71429	R.FYAYNPLAGGLLTGK.Y
ARK72_MOUSE	MK_SCX_2201.2538.2538.2	2	4.082	0.551	1	1962	95	R.MDASASAASVR.A
ARK72_MOUSE	MK_SCX_2201.3095.3095.2	2	3.529	0.515	1	1100.9	81.818184	K.ALQTTYGTNAPR.M
ARK72_MOUSE	MK_SCX_24.11729.11729.3	3	4.224	0.476	1	559.2	21.323528	R.GHSELDTAFMYCDGQSENILGGLGLGLGSGDCTVK.I
ARK72_MOUSE	MK_SCX_26.5064.5064.2	2	2.347	0.49	1	394.9	70	R.FFGNNWAETYR.N
ARK72_MOUSE	MK_SCX_29.5665.5665.2	2	4.052	0.531	1	695.2	50	R.AASGAPLRPATVLGTMEMGR.R
ARK72_MOUSE	MK_SCX_29.5689.5689.3	3	4.601	0.587	1	2761.8	53.947372	R.AASGAPLRPATVLGTMEMGR.R
ARK72_MOUSE	MK_SCX_35.10380.10380.3	3	3.859	0.345	1	525.2	32.894737	R.VDLFYLHAPDHSTPVEETLR.A
ARK72_MOUSE	MK_SCX_46.5325.5325.3	3	3.068	0.227	1	365.6	32.5	R.AASGAPLRPATVLGTMEMGRR.M
ARL1_MOUSE	MK_SCX_18.10306.10306.2	2	3.306	0.391	1	543.2	56.666668	K.GTGLDEAMEWLVETLK.S
ARL3_MOUSE	MK_SCX_27.5788.5788.3	3	3.205	0.417	1	339.1	27.777779	K.QLASEDISHITPTQGFNIK.S
ARL3_MOUSE	MK_SCX_27.5814.5814.2	2	4.725	0.577	1	558.4	50	K.QLASEDISHITPTQGFNIK.S
ARL6_MOUSE	MK_SCX_29.6049.6049.3	3	3.636	0.363	1	435.5	32.5	K.LKPSNAQSQDIVPTIGFSIEK.F
ARL6_MOUSE	MK_SCX_44.6009.6009.2	2	2.773	0.253	1	849.9	72.22222	R.RIPILFFANK.M
ARL8A_MOUSE	MK_SCX_25.5101.5101.2	2	4.145	0.448	1	2277.7	84.61539	R.DLAGALDEKELIEK.M
ARL8A_MOUSE	MK_SCX_51.7709.7709.3	3	4.673	0.535	1	830.7	35.869564	K.NELHNLLDKPQLQGIPVLVLGNKR.D
ARLY_MOUSE	MK_SCX_17.9176.9176.2	2	4.988	0.576	1	1474.9	64.70589	K.QALSPDM*LATDLAYYLVR.K
ARLY_MOUSE	MK_SCX_17.9377.9377.2	2	5.129	0.575	1	2408.5	79.411766	K.QALSPDMLATDLAYYLVR.K
ARLY_MOUSE	MK_SCX_18.6794.6794.2	2	6.01	0.638	1	1734.3	73.68421	R.INVLPLGSGAIAGNPLGVDR.E

ARLY_MOUSE	MK_SCX_21.5510.5510.2	2	4.394	0.402	1	2289.6	86.36364	K.AEMQQILQGLDK.V
ARLY_MOUSE	MK_SCX_25.7879.7879.3	3	3.411	0.32	1	399.8	29.347824	R.INVLPLGSGAIAGNPLGVDRELLR.A
ARLY_MOUSE	MK_SCX_45.3855.3855.2	2	3.948	0.359	1	1103.8	90	R.HLWNVDVQGSK.A
ARLY_MOUSE	MK_SCX_46.4779.4779.3	3	3.274	0.136	1	669.5	40.625	R.AEAERDVLFPGYTHLQR.A
ARLY_MOUSE	MK_SCX_55.3266.3266.3	3	3.164	0.212	1	442.6	38.636364	R.RLKELIGEAAGK.L
ARMC1_MOUSE	MK_SCX_21.4040.4040.2	2	2.47	0.412	1	532.3	72.72727	K.AEALASAIASTK.V
ARMC1_MOUSE	MK_SCX_31.7878.7878.2	2	5.094	0.486	1	1745.4	72.5	R.VGSHPEGGASWLSTAANFLSR.S
ARMET_MOUSE	MK_SCX_54.3874.3874.3	3	4.11	0.472	1	829.7	42.1875	K.IINEVSKPLAHHIPVEK.I
ARP2_MOUSE	MK_SCX_27.6663.6663.3	3	3.256	0.27	1	1019	38.88889	K.VGNIEIKDLMVGDEASELR.S
ARP2_MOUSE	MK_SCX_40.5609.5609.2	2	2.821	0.386	1	613.5	70	K.HLWDYTFGPEK.L
ARP3_MOUSE	MK_SCX_24.6199.6199.3	3	3.582	0.321	1	657.9	33.75	R.AEPEDHYFLLTEPPLNTPENR.E
ARP3_MOUSE	MK_SCX_41.3639.3639.2	2	3.333	0.432	1	1017.9	90	R.LKLSEELSGGR.L
ARPC2_MOUSE	MK_SCX_36.3623.3623.3	3	3.671	0.351	1	610.6	46.153847	K.YFQFQEEGKEGENR.A
ARPC2_MOUSE	MK_SCX_46.3560.3560.3	3	3.323	0.343	1	795.4	40	R.AVIHYRDDETM*YVESK.K
ARPC3_MOUSE	MK_SCX_2201.5200.5200.2	2	2.309	0.297	1	659.2	70	K.LIGNM*ALLPLR.S
ARPC3_MOUSE	MK_SCX_2201.6718.6718.2	2	3.481	0.482	1	1383.3	90	K.LIGNMALLPLR.S
ARPC4_MOUSE	MK_SCX_20_1.5490.5490.2	2	4.04	0.324	1	584.3	70	K.ELLLQPVTISR.N
ARPC4_MOUSE	MK_SCX_23.4029.4029.2	2	3.395	0.394	1	1265.6	90	K.VLIEGSINSVR.V
ARPC5_MOUSE	MK_SCX_18.5350.5350.2	2	3.376	0.404	1	1026.6	60.526318	R.QGNMTAALQAALKNPPINTK.S
ARPC5_MOUSE	MK_SCX_28.5151.5151.2	2	2.95	0.204	1	1780.3	71.42857	K.AVQSLDKNGVDLLMK.Y
ARPC5_MOUSE	MK_SCX_30.5996.5996.3	3	3.567	0.395	1	515.2	35.526314	R.QGNMTAALQAALKNPPINTK.S
ARS2_MOUSE	MK_SCX_16.6879.6879.2	2	4.391	0.63	1	759.2	50	R.TQLWASEPGTPPVPTSLPSQNPILK.N
ARY2_MOUSE	MK_SCX_25.8525.8525.3	3	3.805	0.426	1	833.3	30.434782	R.SYQMWEPLELTSGKDQPQVPAIFR.L
AS3MT_MOUSE	MK_SCX_14.3482.3482.2	2	3.019	0.336	1	995.8	75	K.TSADLQTNACVTR.A
AS3MT_MOUSE	MK_SCX_17.15719.15719.2	2	3.718	0.382	1	766.9	60.000004	K.EGEAVAVDEETAAVLK.N
AS3MT_MOUSE	MK_SCX_17.7463.7463.2	2	4.677	0.574	1	1583.9	65.789474	R.FAPDFLFTPVDASLPAPQGR.S
AS3MT_MOUSE	MK_SCX_36.5321.5321.3	3	4.32	0.437	1	943.7	39.705883	R.DADEIHKDVQNYYGNVLK.T
AS3MT_MOUSE	MK_SCX_36.6524.6524.2	2	3.636	0.583	1	816.3	73.07692	K.FGFQAPNVTFLHGR.I
AS3MT_MOUSE	MK_SCX_36.6534.6534.3	3	4.078	0.503	1	924.5	48.076923	K.FGFQAPNVTFLHGR.I
AS3MT_MOUSE	MK_SCX_46.5255.5255.3	3	3.14	0.315	1	382.6	37.5	K.VLIRDPFKLAEDSDK.M
ASCL3_MOUSE	MK_SCX_18.4480.4480.2	2	3.535	0.255	1	317.3	41.17647	K.YISYLQSLLYPDESETKK.N
ASM3A_MOUSE	MK_SCX_25.7401.7401.3	3	4.578	0.26	1	1364.9	35.714287	K.VYXIAHVPVGYLPYATDTPAIR.Q
ASM3A_MOUSE	MK_SCX_25.7508.7508.2	2	4.309	0.671	1	611.1	54.761906	K.VYXIAHVPVGYLPYATDTPAIR.Q
ASM3A_MOUSE	MK_SCX_25.9052.9052.2	2	4.671	0.494	1	569.6	50	K.VYSAVADLWKPWLGEEAISTLK.K
ASM3A_MOUSE	MK_SCX_26.8676.8676.3	3	5.536	0.528	1	1280.9	42.857143	K.VYSAVADLWKPWLGEEAISTLK.K
ASM3A_MOUSE	MK_SCX_44.7209.7209.3	3	6.17	0.579	1	1410	40.625	R.APAVGQFWHVTDLHLDPTYHITDDR.T
ASPG_MOUSE	MK_SCX_14.6215.6215.3	3	4.051	0.478	1	654.6	28.333334	R.VGDSPIPGAGAYADDTAGAAAATGDGDTLLR.F
ASPG_MOUSE	MK_SCX_14.6223.6223.2	2	6.846	0.575	1	1638.8	51.666664	R.VGDSPIPGAGAYADDTAGAAAATGDGDTLLR.F
ASPG_MOUSE	MK_SCX_19.4471.4471.2	2	4.347	0.598	1	1646.3	78.57143	K.FAESMGFTNEDLSTK.T
ASPG_MOUSE	MK_SCX_2201.4619.4619.2	2	3.659	0.515	1	472.3	77.27273	R.FLPSYQAVEYM*R.G
ASPG_MOUSE	MK_SCX_2201.5769.5769.2	2	3.454	0.527	1	414.3	72.72727	R.FLPSYQAVEYMR.G
ASPG_MOUSE	MK_SCX_45.3696.3696.3	3	4.822	0.498	1	1417.8	48.4375	R.VLEHTTHTLLVGDSATK.F
ASSY_MOUSE	MK_SCX_13.6477.6477.2	2	2.43	0.366	1	304.6	54.166668	R.FELTCYSLAPQIK.V
ASSY_MOUSE	MK_SCX_16.8117.8117.2	2	4.95	0.572	1	1417.8	68.42105	K.EFVEEFIWPAVQSSALYEDR.Y
ASSY_MOUSE	MK_SCX_16.8350.8350.3	3	4.406	0.526	1	770.2	34.210526	K.EFVEEFIWPAVQSSALYEDR.Y
ASSY_MOUSE	MK_SCX_19.11230.11230.2	2	5.228	0.536	1	2255.6	76.666664	R.TTSLELFMYLNEVAGK.H
ASSY_MOUSE	MK_SCX_19.7805.7805.2	2	4.366	0.491	1	1748.4	70	R.TTSLELFM*YLNEVAGK.H
ASSY_MOUSE	MK_SCX_20_1.6019.6019.2	2	4.193	0.538	1	966.8	75	K.APNSPDVLEIEFK.K
ASSY_MOUSE	MK_SCX_2201.2683.2683.2	2	2.651	0.307	1	892.6	92.85714	R.NDLM*EYAK.Q
ASSY_MOUSE	MK_SCX_2201.2828.2828.2	2	2.412	0.218	1	735.3	91.66667	R.IDIVENR.F
ASSY_MOUSE	MK_SCX_2201.5692.5692.2	2	2.592	0.292	1	509.3	53.846157	R.YLLGTSLARPCIAR.R

ASSY_MOUSE	MK_SCX_25.5442.5442.3	3	5.55	0.651	1	1165.2	48.61111	K.TQDPAKAPNSPDVLEIEFK.K
ASSY_MOUSE	MK_SCX_26.5351.5351.2	2	5.87	0.57	1	1292.4	69.44444	K.TQDPAKAPNSPDVLEIEFK.K
ASSY_MOUSE	MK_SCX_26.7188.7188.3	3	6.545	0.123	1	2648.3	43.18182	K.SPWSM*DENLMHISYEAGILENPK.N
ASSY_MOUSE	MK_SCX_26.7205.7205.2	2	4.987	0.218	1	473.8	47.727272	K.SPWSM*DENLMHISYEAGILENPK.N
ASSY_MOUSE	MK_SCX_26.7244.7244.2	2	5.232	0.144	1	498.2	47.727272	K.SPWSMDENLM*HISYEAGILENPK.N
ASSY_MOUSE	MK_SCX_26.7827.7827.2	2	5.2	0.649	1	1121.2	50	K.SPWSMDENLMHISYEAGILENPK.N
ASSY_MOUSE	MK_SCX_26.7857.7857.3	3	7.603	0.704	1	4316.8	51.136364	K.SPWSMDENLMHISYEAGILENPK.N
ASSY_MOUSE	MK_SCX_27.3708.3708.2	2	2.392	0.353	1	643	91.66667	R.MPEFYNR.F
ASSY_MOUSE	MK_SCX_30.4781.4781.3	3	4.318	0.574	1	1180.8	59.615387	K.APNSPDVLEIEFKK.G
ASSY_MOUSE	MK_SCX_30.6911.6911.3	3	6.217	0.6	1	601.1	30.46875	K.SPWSM*DENLM*HISYEAGILENPKNQAPPGLYTK.T
ASSY_MOUSE	MK_SCX_30.8855.8855.3	3	7.482	0.713	1	2524.9	35.15625	K.SPWSMDENLMHISYEAGILENPKNQAPPGLYTK.T
ASSY_MOUSE	MK_SCX_31.5018.5018.2	2	4.335	0.481	1	1113.5	73.07692	K.APNSPDVLEIEFKK.G
ASSY_MOUSE	MK_SCX_37.5175.5175.2	2	5.277	0.566	1	1090.9	65.789474	K.TQDPAKAPNSPDVLEIEFKK.G
ASSY_MOUSE	MK_SCX_37.5191.5191.3	3	6.109	0.632	1	1573	50	K.TQDPAKAPNSPDVLEIEFKK.G
ASSY_MOUSE	MK_SCX_42.6610.6610.3	3	3.881	0.424	1	873.1	38.88889	R.NDLMEYAKQHGIPIPVTPK.S
ASSY_MOUSE	MK_SCX_49.1676.1676.2	2	3.118	0.255	1	1015.9	100	R.RQVEIAQR.E
ASSY_MOUSE	MK_SCX_53.7738.7738.3	3	4.386	0.509	1	885.1	38.04348	K.FAELVYTGFWHSPECEFVRHCIQK.S
ASSY_MOUSE	MK_SCX_55.3469.3469.3	3	3.184	0.339	1	579.1	50	R.FKGRNDLMEYAK.Q
ASSY_MOUSE	MK_SCX_55.3496.3496.2	2	3.534	0.379	1	1670.7	86.36364	R.FKGRNDLMEYAK.Q
AT1A1_MOUSE	MK_SCX_16.10064.10064.2	2	3.518	0.45	1	318.2	38.636364	K.QAADMILLDDNFASIVTGVEEGR.L
AT1A1_MOUSE	MK_SCX_16.4267.4267.2	2	3.845	0.232	1	763.4	45.454548	R.IISANGCKVDNSSLTGESEPQTR.S
AT1A1_MOUSE	MK_SCX_17.5365.5365.2	2	4.223	0.489	1	1375.8	86.36364	K.DM*TSEELDDILR.Y
AT1A1_MOUSE	MK_SCX_17.5915.5915.2	2	4.335	0.474	1	1594.6	86.36364	K.DMTSEELDDILR.Y
AT1A1_MOUSE	MK_SCX_18.15914.15914.2	2	2.497	0.395	1	307.5	46.666668	K.ADIGVAMGIVGSDVSK.Q
AT1A1_MOUSE	MK_SCX_18.4566.4566.2	2	5.426	0.632	1	1873.3	69.44444	R.QGAIVAVTGDGVNDSPALK.K
AT1A1_MOUSE	MK_SCX_18.5514.5514.2	2	5.333	0.607	1	1933.6	73.52941	K.GVGIISEGNETVEDIAAR.L
AT1A1_MOUSE	MK_SCX_18.6411.6411.2	2	4.102	0.419	1	1201.8	62.5	K.M*SINAEDVVVGDLVEVK.G
AT1A1_MOUSE	MK_SCX_18.7227.7227.2	2	5.257	0.501	1	2759.9	75	K.MSINAEDVVVGDLVEVK.G
AT1A1_MOUSE	MK_SCX_20_1.3047.3047.2	2	4.72	0.52	1	2001.3	82.14286	K.VDNSSLTGESEPQTR.S
AT1A1_MOUSE	MK_SCX_20_1.3730.3730.2	2	4.408	0.583	1	1696.7	83.33333	R.AVAGDASESALLK.C
AT1A1_MOUSE	MK_SCX_20_1.4520.4520.2	2	4.73	0.589	1	1827.1	83.33333	R.SPDFTNENPLETR.N
AT1A1_MOUSE	MK_SCX_20_1.5789.5789.2	2	3.961	0.419	1	1412.8	80.769226	R.AVFQANQENLPILK.R
AT1A1_MOUSE	MK_SCX_2201.10358.10358.3	3	4.485	0.456	1	1133.3	29.166666	K.EQPLDEELKDAFQNAYLELGGLGER.V
AT1A1_MOUSE	MK_SCX_23.4043.4043.2	2	3.296	0.571	1	872.3	90	R.LNIPVNQVNPR.D
AT1A1_MOUSE	MK_SCX_25.4944.4944.2	2	3.83	0.487	1	1544.7	94.44444	K.TSATWFALSR.I
AT1A1_MOUSE	MK_SCX_27.4302.4302.3	3	3.938	0.554	1	813.3	40.789474	R.QGAIVAVTGDGVNDSPALKK.A
AT1A1_MOUSE	MK_SCX_27.4320.4320.2	2	4.842	0.597	1	1194.8	57.894737	R.QGAIVAVTGDGVNDSPALKK.A
AT1A1_MOUSE	MK_SCX_31.5220.5220.3	3	3.233	0.344	1	568	32.8125	K.KADIGVAMGIVGSDVSK.Q
AT1A1_MOUSE	MK_SCX_31.5327.5327.2	2	4.659	0.346	1	2242.8	71.875	K.KADIGVAMGIVGSDVSK.Q
AT1A1_MOUSE	MK_SCX_33.5075.5075.2	2	4.737	0.473	1	1593.2	75	R.AVFQANQENLPILKR.A
AT1A1_MOUSE	MK_SCX_43.3848.3848.2	2	2.456	0.328	1	500.2	93.75	R.YHTEIVFAR.T
AT1A1_MOUSE	MK_SCX_51.5144.5144.3	3	4.125	0.518	1	484.7	32	K.IVEIPFNSTNKYQLSIHKNPNASEPK.H
AT1A4_MOUSE	MK_SCX_12.5828.5828.2	2	3.011	0.268	1	946.3	58.333332	R.LGAIVAVTGDGVNDSPALK.K
AT1A4_MOUSE	MK_SCX_27.5231.5231.2	2	3.574	0.55	1	919.7	50	R.LGAIVAVTGDGVNDSPALKK.A
AT1A4_MOUSE	MK_SCX_29.3791.3791.2	2	4.409	0.411	1	1031.9	75	K.NM*VPQQALVIRDGEK.M
AT1A4_MOUSE	MK_SCX_29.4208.4208.2	2	4.618	0.417	1	948.7	71.42857	K.NMVPQQALVIRDGEK.M
AT1B1_MOUSE	MK_SCX_14.4515.4515.2	2	2.438	0.392	1	397.6	54.166668	K.YNPNVLPVQCTGK.R
AT1B1_MOUSE	MK_SCX_16.7941.7941.3	3	5.095	0.583	1	1805.6	37.5	K.VGNIEYFGM*GGYYGFPLQYYPYYGK.L
AT1B1_MOUSE	MK_SCX_16.7964.7964.2	2	4.519	0.621	1	739.5	43.75	K.VGNIEYFGM*GGYYGFPLQYYPYYGK.L
AT1B1_MOUSE	MK_SCX_16.8411.8411.3	3	5.419	0.606	1	1616.8	36.458336	K.VGNIEYFGMGGYYGFPLQYYPYYGK.L
AT1B1_MOUSE	MK_SCX_16.8533.8533.2	2	5.496	0.665	1	1100.5	47.916664	K.VGNIEYFGMGGYYGFPLQYYPYYGK.L

AT1B1_MOUSE	MK_SCX_19.4687.4687.2	2	2.745	0.414	1	484.8	80.769226	R.VAPPGLTQIPQIQK.T
AT1B1_MOUSE	MK_SCX_2201.3263.3263.2	2	3.615	0.505	1	1054.4	85	K.AYGENIGYSEK.D
AT1B1_MOUSE	MK_SCX_2201.7135.7135.3	3	4.411	0.288	1	1205.7	55	K.SYEAYVLNIIR.F
AT1B1_MOUSE	MK_SCX_23.6738.6738.2	2	4.648	0.454	1	1828.5	85	K.SYEAYVLNIIR.F
AT1B1_MOUSE	MK_SCX_26.7962.7962.3	3	4.887	0.612	1	1172.1	31.666666	R.DEDKDKVGNIEYFGM*GGYYGFPLQYYPYYGK.L
AT1B1_MOUSE	MK_SCX_26.8489.8489.3	3	5.221	0.522	1	1390.9	32.5	R.DEDKDKVGNIEYFGMGGYYGFPLQYYPYYGK.L
AT1B1_MOUSE	MK_SCX_36.3219.3219.2	2	2.13	0.219	1	433.1	54.166668	K.AYGENIGYSEKDR.F
AT1B1_MOUSE	MK_SCX_38.12975.12975.3	3	3.067	0.267	1	311.8	28.57143	K.TEISFRPNDPKSYEAYVLNIIR.F
AT1B1_MOUSE	MK_SCX_51.3548.3548.2	2	3.649	0.368	1	402.4	40.625	K.AYGENIGYSEKDRFQGR.F
AT2A2_MOUSE	MK_SCX_21.8220.8220.2	2	4.235	0.643	1	1104.7	57.5	K.DIVPGDIVEIAVGDKVPADIR.L
AT5F1_MOUSE	MK_SCX_18.10462.10462.2	2	4.926	0.626	1	771.1	75	R.LGLIPEEFFQFLYPK.T
AT5F1_MOUSE	MK_SCX_21.3235.3235.2	2	2.276	0.415	1	345	72.22222	K.QIQDAIDM*EK.A
AT5F1_MOUSE	MK_SCX_21.3923.3923.2	2	2.694	0.478	1	364.3	77.77778	K.QIQDAIDMEK.A
AT5F1_MOUSE	MK_SCX_2201.13747.13747.2	2	2.435	0.373	1	391.7	63.636364	R.NNIALALEVTYR.E
AT5F1_MOUSE	MK_SCX_34.3595.3595.3	3	3.06	0.427	1	397.9	39.583336	K.IAQLEEVKQSSMK.Q
AT5F1_MOUSE	MK_SCX_35.9258.9258.3	3	3.231	0.289	1	460.6	40.909092	R.LDYHISVQNMMR.R
AT5F1_MOUSE	MK_SCX_40.4265.4265.3	3	3.996	0.496	1	863.2	43.055553	K.QIQDAIDM*EKAQQALVQKR.H
AT5F1_MOUSE	MK_SCX_47.4002.4002.2	2	2.723	0.214	1	1086.4	92.85714	R.HYLFDVQR.N
AT5F1_MOUSE	MK_SCX_54.4933.4933.2	2	3.905	0.572	1	1232.4	84.61539	K.NRLDYHISVQNMMR.R
AT5F1_MOUSE	MK_SCX_54.4936.4936.3	3	3.314	0.131	1	1384.1	50	K.NRLDYHISVQNMMR.R
ATAD3_MOUSE	MK_SCX_17.4746.4746.2	2	4.575	0.591	1	802.7	54.545456	K.GEGTGPPLPLPPAQPGAEGGGDR.G
ATAD3_MOUSE	MK_SCX_36.4048.4048.3	3	4.395	0.144	1	1553.9	56.81818	K.LKEYEAAVEQLK.S
ATAD3_MOUSE	MK_SCX_36.4076.4076.2	2	2.882	0.223	1	938.8	77.27273	K.LKEYEAAVEQLK.S
ATAD3_MOUSE	MK_SCX_46.6414.6414.3	3	5.018	0.584	1	1603.1	38.095238	R.RLVSRPQDALEGVILSPSLEAR.V
ATAD3_MOUSE	MK_SCX_48.4953.4953.3	3	5.066	0.545	1	590.8	42.1875	K.LKEYEAAVEQLKSEQIR.V
ATBF1_MOUSE	MK_SCX_10.8874.8874.2	2	2.442	0.268	1	353.8	50	R.LSVRDHIFSQQHISK.V
ATBF1_MOUSE	MK_SCX_13.7195.7195.2	2	2.919	0.208	1	493.7	44.444447	K.EKKDLAPGGGSEGTMLPPR.I
ATF1_MOUSE	MK_SCX_16.6472.6472.2	2	3.469	0.603	1	338.7	34	R.TTPSATSLPQTVVMTSPVTLASQTTK.T
ATG3_MOUSE	MK_SCX_20_1.3685.3685.2	2	4.409	0.373	1	1633.7	83.33333	K.ADAGGEDAILQTR.T
ATG3_MOUSE	MK_SCX_27.6958.6958.3	3	3.785	0.46	1	361.5	38.333332	K.ALEVAEYLTPVLKESK.F
ATG3_MOUSE	MK_SCX_32.4657.4657.2	2	3.311	0.121	1	1059.1	79.16667	K.AYLPTDKQFLVTK.N
ATNG_MOUSE	MK_SCX_14.3770.3770.2	2	4.503	0.448	1	1356.5	68.75	-.MAGEISDLSANSGGSAK.G
ATNG_MOUSE	MK_SCX_20_1.5418.5418.2	2	5.245	0.509	1	1322.6	76.92308	K.GTENPFEYDYETVR.K
ATNG_MOUSE	MK_SCX_31.4675.4675.2	2	4.336	0.525	1	1337.7	67.85714	K.GTENPFEYDYETVRK.G
ATOX1_MOUSE	MK_SCX_32.5335.5335.3	3	3.354	0.439	1	1091.9	51.923077	K.LGGVEFNIDLPNKK.V
ATOX1_MOUSE	MK_SCX_32.5451.5451.2	2	3.646	0.408	1	1240.2	61.538464	K.LGGVEFNIDLPNKK.V
ATP5E_MOUSE	MK_SCX_14.2834.2834.1	1	2.07	0.195	1	309.6	75	R.FSQICAK.A
ATP5E_MOUSE	MK_SCX_34.2754.2754.2	2	2.064	0.138	1	341.7	64.28571	R.DALKTEFK.A
ATP5E_MOUSE	MK_SCX_47.3065.3065.3	3	3.224	0.404	1	1204.6	52.083332	R.DALKTEFKANAEK.T
ATP5H_MOUSE	MK_SCX_14.4319.4319.2	2	3.697	0.376	1	1078.2	68.181816	K.SCAEFVSGSQLR.I
ATP5H_MOUSE	MK_SCX_16.8479.8479.3	3	3.928	0.463	1	1381.5	41.25	R.NIIPFDQM*TIDDLNEIFPETK.L
ATP5H_MOUSE	MK_SCX_16.8619.8619.2	2	5.535	0.436	1	837.2	57.5	R.NIIPFDQM*TIDDLNEIFPETK.L
ATP5H_MOUSE	MK_SCX_16.9037.9037.3	3	4.79	0.546	1	1355.2	41.25	R.NIIPFDQMTIDDLNEIFPETK.L
ATP5H_MOUSE	MK_SCX_16.9227.9227.2	2	5.354	0.498	1	1262.3	57.5	R.NIIPFDQMTIDDLNEIFPETK.L
ATP5H_MOUSE	MK_SCX_19.6752.6752.2	2	4.97	0.406	1	1480.4	70	K.TIDWVSFVEVM*PQNQK.A
ATP5H_MOUSE	MK_SCX_19.7741.7741.2	2	5.11	0.25	1	1356	70	K.TIDWVSFVEVMPQNQK.A
ATP5H_MOUSE	MK_SCX_27.11544.11544.3	3	4.878	0.169	1	1116.6	37.5	K.MRNIIPFDQM*TIDDLNEIFPETK.L
ATP5H_MOUSE	MK_SCX_27.12693.12693.3	3	5.126	0.531	1	949.6	36.363636	K.MRNIIPFDQMTIDDLNEIFPETK.L
ATP5H_MOUSE	MK_SCX_27.5150.5150.3	3	4.403	0.504	1	1071.4	50	K.IPVPEDKYTALVDQEEK.E
ATP5H_MOUSE	MK_SCX_30.3383.3383.3	3	3.149	0.355	1	549	38.46154	K.YTALVDQEEKEDVK.S
ATP5H_MOUSE	MK_SCX_30.4197.4197.2	2	2.43	0.185	1	438.2	53.846157	K.YTALVDQEEKEDVK.S

ATP5H_MOUSE	MK_SCX_30.6098.6098.2	2	4.594	0.435	1	609.2	65.625	R.LASLSEKPPAIDWAYYR.A
ATP5H_MOUSE	MK_SCX_30.6266.6266.3	3	5.32	0.619	1	1369.8	48.4375	R.LASLSEKPPAIDWAYYR.A
ATP5H_MOUSE	MK_SCX_32.3849.3849.3	3	3.04	0.458	1	706.5	46.153847	R.ANVAKPGLVDDFEK.K
ATP5H_MOUSE	MK_SCX_32.4025.4025.2	2	4.466	0.508	1	671.9	73.07692	R.ANVAKPGLVDDFEK.K
ATP5H_MOUSE	MK_SCX_34.4295.4295.3	3	4.206	0.267	1	1242.5	56.81818	K.YNALKIPVPEDK.Y
ATP5H_MOUSE	MK_SCX_34.4303.4303.2	2	3.827	0.366	1	1487.5	86.36364	K.YNALKIPVPEDK.Y
ATP5H_MOUSE	MK_SCX_34.5377.5377.3	3	5.056	0.417	1	805.6	40	K.IPVPEDKYTALVDQEEKEDVK.S
ATP5H_MOUSE	MK_SCX_37.6819.6819.3	3	4.56	0.568	1	1196.7	40.476192	K.YNALKIPVPEDKYTALVDQEEK.E
ATP5H_MOUSE	MK_SCX_45.5612.5612.3	3	3.299	0.429	1	535	45	K.AIGNALKSWNETFHAR.L
ATP5H_MOUSE	MK_SCX_46.3747.3747.3	3	4.588	0.473	1	1064.9	48.214287	R.ANVAKPGLVDDFEKK.Y
ATP5H_MOUSE	MK_SCX_47.3870.3870.2	2	5.004	0.555	1	1060.9	78.57143	R.ANVAKPGLVDDFEKK.Y
ATP5H_MOUSE	MK_SCX_48.4285.4285.2	2	2.98	0.339	1	1135.8	79.16667	K.KYNALKIPVPEDK.Y
ATP5H_MOUSE	MK_SCX_52.3786.3786.3	3	4.168	0.24	1	984	52.083332	K.KYNALKIPVPEDK.Y
ATP5I_MOUSE	MK_SCX_21.16259.16259.2	2	2.53	0.22	1	332	50	R.YSALIIGMAYGAK.R
ATP5I_MOUSE	MK_SCX_21.4685.4685.2	2	3.887	0.552	1	615.2	80	-.VPPVQVSPLIK.F
ATP5J_MOUSE	MK_SCX_18.4681.4681.2	2	4.304	0.604	1	1119.8	69.44444	R.QASGGPVDIGPEYQQDLDR.E
ATP5J_MOUSE	MK_SCX_19.4339.4339.2	2	3.736	0.267	1	549.7	78.57143	K.QMYGKGEM*DTFPTFK.F
ATP5J_MOUSE	MK_SCX_19.4858.4858.2	2	4.301	0.56	1	1027.6	82.14286	K.QMYGKGEMDTFPTFK.F
ATP5J_MOUSE	MK_SCX_24.5812.5812.2	2	3.354	0.48	1	370.4	36.363636	R.QASGGPVDIGPEYQQDLDRELYK.L
ATP5J_MOUSE	MK_SCX_25.5959.5959.3	3	4.058	0.519	1	702.6	29.545454	R.QASGGPVDIGPEYQQDLDRELYK.L
ATP5J_MOUSE	MK_SCX_28.5745.5745.3	3	3.248	0.492	1	488.3	39.285713	K.GEMDTFPTFKFDDPK.F
ATP5J_MOUSE	MK_SCX_28.5880.5880.2	2	2.152	0.244	1	327.3	50	K.GEMDTFPTFKFDDPK.F
ATP5J_MOUSE	MK_SCX_32.4353.4353.3	3	5.412	0.499	1	2069.6	48.684208	K.RQASGGPVDIGPEYQQDLDR.E
ATP5J_MOUSE	MK_SCX_33.4417.4417.2	2	5.524	0.572	1	972.6	68.42105	K.RQASGGPVDIGPEYQQDLDR.E
ATP5J_MOUSE	MK_SCX_34.4568.4568.3	3	3.206	0.278	1	657.5	48.214287	K.QM*YGKGEMDTFPTFK.F
ATP5J_MOUSE	MK_SCX_34.4586.4586.2	2	2.935	0.409	1	310.9	53.571426	K.QM*YGKGEMDTFPTFK.F
ATP5J_MOUSE	MK_SCX_34.6834.6834.3	3	3.686	0.506	1	453.9	26.041666	R.QASGGPVDIGPEYQQDLDRELYKLK.Q
ATP5J_MOUSE	MK_SCX_41.5723.5723.3	3	6.137	0.615	1	1374.9	39.130436	K.RQASGGPVDIGPEYQQDLDRELYK.L
ATP5J_MOUSE	MK_SCX_42.5544.5544.2	2	3.337	0.467	1	476.2	41.304348	K.RQASGGPVDIGPEYQQDLDRELYK.L
ATP5J_MOUSE	MK_SCX_50.4237.4237.3	3	3.16	0.355	1	459.4	39.0625	K.LKQMYGKGEM*DTFPTFK.F
ATP5J_MOUSE	MK_SCX_50.4443.4443.3	3	3.331	0.428	1	513	39.0625	K.LKQM*YGKGEMDTFPTFK.F
ATP5J_MOUSE	MK_SCX_50.4861.4861.2	2	5.253	0.535	1	1947.1	75	K.LKQMYGKGEMDTFPTFK.F
ATP5J_MOUSE	MK_SCX_50.4883.4883.3	3	4.151	0.504	1	864.2	50	K.LKQMYGKGEMDTFPTFK.F
ATP5L_MOUSE	MK_SCX_25.6461.6461.2	2	3.952	0.506	1	398	50	K.VELVPPTPAEIPTAIQSVKK.I
ATP5L_MOUSE	MK_SCX_33.4131.4131.3	3	4.19	0.567	1	686.1	42.857143	K.APSMVAAAVTYSKPR.L
ATP5L_MOUSE	MK_SCX_33.4184.4184.2	2	4.737	0.616	1	2401.4	85.71429	K.APSMVAAAVTYSKPR.L
ATP5L_MOUSE	MK_SCX_43.4468.4468.2	2	3.294	0.569	1	703.2	81.25	R.LATFWHYAK.V
ATP5L_MOUSE	MK_SCX_45.4764.4764.3	3	5.415	0.605	1	1925.5	50	R.NFAEKAPSM*VAAAVTYSKPR.L
ATP5L_MOUSE	MK_SCX_45.5758.5758.3	3	5.49	0.665	1	1826.6	44.736843	R.NFAEKAPSMVAAAVTYSKPR.L
ATPA_MOUSE	MK_SCX_16.9043.9043.2	2	7.446	0.586	1	3260.1	71.42857	R.EVAAFAQFGSDLDAATQQLLSR.G
ATPA_MOUSE	MK_SCX_16.9083.9083.3	3	5.933	0.49	1	1924.3	46.42857	R.EVAAFAQFGSDLDAATQQLLSR.G
ATPA_MOUSE	MK_SCX_17.6282.6282.2	2	6.349	0.587	1	1040.4	63.15789	K.GM*SLNLEPDNVGVVVFGNDK.L
ATPA_MOUSE	MK_SCX_17.6744.6744.2	2	5.949	0.573	1	1215.2	68.42105	K.GMSLNLEPDNVGVVVFGNDK.L
ATPA_MOUSE	MK_SCX_18.4680.4680.1	1	3.152	0.465	1	422.9	60.714287	R.ILGADTSVDLEETGR.V
ATPA_MOUSE	MK_SCX_18.4922.4922.2	2	5.333	0.544	1	2918.1	92.85714	R.ILGADTSVDLEETGR.V
ATPA_MOUSE	MK_SCX_18.6334.6334.2	2	4.883	0.561	1	1304.8	80	R.TGAIVDVPVGEELLGR.V
ATPA_MOUSE	MK_SCX_18.7809.7809.2	2	4.127	0.59	1	1696.3	60.000004	K.QGQYSPM*AIEEQVAVIYAGVR.G
ATPA_MOUSE	MK_SCX_18.8376.8376.2	2	5.226	0.618	1	2262.9	70	K.QGQYSPMAIEEQVAVIYAGVR.G
ATPA_MOUSE	MK_SCX_20_1.15751.15751.3	3	4.609	0.325	1	1716.5	50	R.NVQAEEM*VEFSSGLK.G
ATPA_MOUSE	MK_SCX_20_1.15791.15791.3	3	4.413	0.467	1	1307.3	46.42857	R.NVQAEEMVEFSSGLK.G
ATPA_MOUSE	MK_SCX_20_1.15833.15833.2	2	5.459	0.574	1	2768.5	82.14286	R.NVQAEEMVEFSSGLK.G

ATPA_MOUSE	MK_SCX_20_1.4001.4001.2	2	4.29	0.629	1	1725.5	90.909096	R.VVDALGNAIDGK.G
ATPA_MOUSE	MK_SCX_20_1.7685.7685.2	2	5.122	0.593	1	2840.9	82.14286	R.NVQAEEM*VEFSSGLK.G
ATPA_MOUSE	MK_SCX_21.3759.3759.2	2	3.906	0.463	1	1300.3	75	K.TGTAEM*SSILEER.I
ATPA_MOUSE	MK_SCX_21.4555.4555.2	2	3.709	0.49	1	1268.1	70.83333	K.TGTAEMSSILEER.I
ATPA_MOUSE	MK_SCX_21.5122.5122.2	2	3.527	0.374	1	1357.1	77.27273	K.TSIAIDTIINQK.R
ATPA_MOUSE	MK_SCX_2201.4007.4007.2	2	2.42	0.281	1	622	83.33333	R.VLSIGDGIAR.V
ATPA_MOUSE	MK_SCX_23.7493.7493.3	3	3.336	0.484	1	335.7	27.083334	R.ILGADTSVDLEETGRVLSIGDGIAR.V
ATPA_MOUSE	MK_SCX_25.7415.7415.3	3	4.084	0.272	1	576	28.40909	K.GM*SLNLEPDNVGVVVFGNDKLIK.E
ATPA_MOUSE	MK_SCX_25.8024.8024.3	3	3.815	0.369	1	702.9	31.818182	K.GMSLNLEPDNVGVVVFGNDKLIK.E
ATPA_MOUSE	MK_SCX_25.8051.8051.2	2	4.557	0.525	1	948.3	56.81818	K.GMSLNLEPDNVGVVVFGNDKLIK.E
ATPA_MOUSE	MK_SCX_27.4352.4352.3	3	3.099	0.39	1	714.8	41.17647	R.VVDALGNAIDGKGPIGSK.T
ATPA_MOUSE	MK_SCX_29.4638.4638.2	2	5.359	0.612	1	1108.5	61.764706	R.VVDALGNAIDGKGPIGSK.T
ATPA_MOUSE	MK_SCX_32.6128.6128.2	2	5.498	0.545	1	1293.7	75	K.RTGAIVDVPVGEELLGR.V
ATPA_MOUSE	MK_SCX_33.4637.4637.3	3	3.03	0.274	1	676.5	43.333332	R.QTGKTSIAIDTIINQK.R
ATPA_MOUSE	MK_SCX_33.4739.4739.2	2	3.845	0.611	1	1143.6	83.33333	R.EAYPGDVFYLHSR.L
ATPA_MOUSE	MK_SCX_33.6157.6157.2	2	2.831	0.17	1	461.7	46.666668	R.LQKTGTAEM*SSILEER.I
ATPA_MOUSE	MK_SCX_33.6419.6419.3	3	4.243	0.464	1	1562	56.25	K.RTGAIVDVPVGEELLGR.V
ATPA_MOUSE	MK_SCX_34.4640.4640.2	2	3.755	0.381	1	1319.1	79.16667	K.TSIAIDTIINQKR.F
ATPA_MOUSE	MK_SCX_36.4273.4273.2	2	3.299	0.566	1	1636.3	90	K.HALIIYDDLSK.Q
ATPA_MOUSE	MK_SCX_38.3664.3664.3	3	3.685	0.412	1	465.4	50	R.ISVREPMQTGIK.A
ATPA_MOUSE	MK_SCX_38.3688.3688.2	2	3.4	0.412	1	691.7	63.636364	R.ISVREPMQTGIK.A
ATPA_MOUSE	MK_SCX_38.4108.4108.2	2	3.234	0.481	1	695.7	80	R.VGLKAPGIIPR.I
ATPA_MOUSE	MK_SCX_38.5599.5599.2	2	3.067	0.449	1	655.8	65.38461	K.GIRPAINVGLSVSR.V
ATPA_MOUSE	MK_SCX_38.6830.6830.3	3	3.127	0.337	1	637.4	42.307693	K.GIRPAINVGLSVSR.V
ATPA_MOUSE	MK_SCX_44.7384.7384.2	2	6.51	0.686	1	1978.7	60.000004	K.FENAFLSHVISQHQSLLGNIR.S
ATPA_MOUSE	MK_SCX_44.7459.7459.3	3	6.249	0.611	1	2315.5	47.5	K.FENAFLSHVISQHQSLLGNIR.S
ATPA_MOUSE	MK_SCX_50.4105.4105.3	3	3.143	0.38	1	676.9	50	R.GYLDKLEPSKITK.F
ATPA_MOUSE	MK_SCX_50.4143.4143.2	2	4.74	0.23	1	1547	83.33333	R.GYLDKLEPSKITK.F
ATPA_MOUSE	MK_SCX_53.8014.8014.3	3	3.358	0.409	1	391.7	28.260868	K.ITKFENAFLSHVISQHQSLLGNIR.S
ATPA_MOUSE	MK_SCX_55.3733.3733.3	3	3.393	0.226	1	899.3	52.272724	R.RVGLKAPGIIPR.I
ATPA_MOUSE	MK_SCX_55.3761.3761.2	2	3.166	0.497	1	960.6	86.36364	R.RVGLKAPGIIPR.I
ATPA_MOUSE	MK_SCX_57.12293.12293.3	3	3.426	0.458	1	453.2	32.352943	R.RPPGREAYPGDVFYLHSR.L
ATPB_MOUSE	MK_SCX_16.10155.10155.2	2	6.185	0.578	1	2411.2	83.33333	R.DQEGQDVLLFIDNIFR.F
ATPB_MOUSE	MK_SCX_16.6539.6539.2	2	4.544	0.655	1	861.2	63.88889	R.AIAELGIYPAVDPLDSTSR.I
ATPB_MOUSE	MK_SCX_16.6633.6633.3	3	4.097	0.386	1	819.4	38.88889	R.AIAELGIYPAVDPLDSTSR.I
ATPB_MOUSE	MK_SCX_17.4493.4493.2	2	5.149	0.474	1	724.3	52.499996	R.IPSAVGYQPTLATDM*GTM*QER.I
ATPB_MOUSE	MK_SCX_17.4549.4549.3	3	4.563	0.502	1	1503	40	R.IPSAVGYQPTLATDM*GTM*QER.I
ATPB_MOUSE	MK_SCX_17.4805.4805.2	2	4.26	0.153	1	646.7	52.499996	R.IPSAVGYQPTLATDM*GTMQER.I
ATPB_MOUSE	MK_SCX_17.5018.5018.2	2	5.295	0.144	1	669.6	52.499996	R.IPSAVGYQPTLATDMGTM*QER.I
ATPB_MOUSE	MK_SCX_17.5069.5069.3	3	4.409	0.405	1	1172.8	37.5	R.IPSAVGYQPTLATDMGTMQER.I
ATPB_MOUSE	MK_SCX_17.5073.5073.3	3	4.99	0.136	1	948	36.25	R.IPSAVGYQPTLATDMGTM*QER.I
ATPB_MOUSE	MK_SCX_17.5407.5407.2	2	4.463	0.53	1	789.6	57.5	R.IPSAVGYQPTLATDMGTMQER.I
ATPB_MOUSE	MK_SCX_20_1.3904.3904.2	2	4.805	0.4	1	999.3	71.42857	K.VALVYGQM*NEPPGAR.A
ATPB_MOUSE	MK_SCX_20_1.4184.4184.2	2	4.327	0.427	1	1455.6	86.36364	R.IM*NVIGEPIDER.G
ATPB_MOUSE	MK_SCX_20_1.7968.7968.2	2	3.955	0.43	1	2023.6	75	K.TVLIM*ELINNVAK.A
ATPB_MOUSE	MK_SCX_21.10175.10175.3	3	3.724	0.467	1	578.5	29.347824	K.SLQDIIAILGMDELSEEDKLTVSR.A
ATPB_MOUSE	MK_SCX_21.10562.10562.2	2	4.902	0.51	1	661.8	39.655174	K.GFQQILAGEYDHLPEQAFYMVGPIEEAVAK.A
ATPB_MOUSE	MK_SCX_21.12410.12410.3	3	5.188	0.644	1	690.1	33.695652	K.SLQDIIAILGM*DELSEEDKLTVSR.A
ATPB_MOUSE	MK_SCX_21.3850.3850.2	2	2.813	0.415	1	1108	81.818184	R.TIAM*DGTEGLVR.G
ATPB_MOUSE	MK_SCX_21.4302.4302.2	2	3.615	0.525	1	1135.9	81.818184	R.TIAMDGTEGLVR.G
ATPB_MOUSE	MK_SCX_21.4484.4484.2	2	4.212	0.535	1	1051.9	71.42857	K.VALVYGQMNEPPGAR.A

ATPB_MOUSE	MK_SCX_21.4667.4667.2	2	4.377	0.451	1	1257.2	81.818184	R.IMNVIGEPIDER.G
ATPB_MOUSE	MK_SCX_21.5342.5342.2	2	4.902	0.541	1	2083.6	80.769226	R.FTQAGSEVSALLGR.I
ATPB_MOUSE	MK_SCX_21.5394.5394.3	3	3.243	0.346	1	1031.6	48.076923	R.FTQAGSEVSALLGR.I
ATPB_MOUSE	MK_SCX_21.7274.7274.2	2	4.166	0.529	1	1743.2	79.16667	R.VALTGLTVAEYFR.D
ATPB_MOUSE	MK_SCX_21.7301.7301.3	3	3.318	0.34	1	732.1	45.833336	R.VALTGLTVAEYFR.D
ATPB_MOUSE	MK_SCX_21.8918.8918.3	3	7.671	0.707	1	2855.7	38.235294	R.FTQAGSEVSALLGRIPSAVGYQPTLATDMGTMQER.I
ATPB_MOUSE	MK_SCX_21.9227.9227.2	2	4.577	0.604	1	579.8	39.655174	K.GFQQILAGEYDHLPEQAFYM*VGPIEEAVAK.A
ATPB_MOUSE	MK_SCX_21.9466.9466.3	3	5.966	0.595	1	2162.1	37.931034	K.GFQQILAGEYDHLPEQAFYM*VGPIEEAVAK.A
ATPB_MOUSE	MK_SCX_2201.10662.10662.3	3	4.085	0.559	1	804.4	28.448275	K.GFQQILAGEYDHLPEQAFYMVGPIEEAVAK.A
ATPB_MOUSE	MK_SCX_23.4316.4316.2	2	3.43	0.356	1	1090.4	80	K.IGLFGGAGVGK.T
ATPB_MOUSE	MK_SCX_26.5756.5756.1	1	4.133	0.539	1	480.8	58.333332	K.VLDSGAPIKIPVGPETLGR.I
ATPB_MOUSE	MK_SCX_26.5831.5831.3	3	5.479	0.493	1	1692.8	51.38889	K.VLDSGAPIKIPVGPETLGR.I
ATPB_MOUSE	MK_SCX_27.6232.6232.2	2	4.688	0.515	1	740.7	66.66667	K.VLDSGAPIKIPVGPETLGR.I
ATPB_MOUSE	MK_SCX_27.7263.7263.2	2	5.247	0.522	1	1157.7	58.823532	R.EGNDLYHEMIESGVINLK.D
ATPB_MOUSE	MK_SCX_29.4147.4147.2	2	5.332	0.586	1	1222.2	76.666664	R.IM*DPNIVGNEHYDVAR.G
ATPB_MOUSE	MK_SCX_29.4284.4284.3	3	3.344	0.368	1	849	45	R.IM*DPNIVGNEHYDVAR.G
ATPB_MOUSE	MK_SCX_29.4527.4527.2	2	5.705	0.585	1	1076.4	76.666664	R.IMDPNIVGNEHYDVAR.G
ATPB_MOUSE	MK_SCX_29.4566.4566.3	3	3.11	0.346	1	614.1	41.666664	R.IMDPNIVGNEHYDVAR.G
ATPB_MOUSE	MK_SCX_29.7432.7432.2	2	4.329	0.509	1	793.3	58.823532	R.FLSQPFQVAEVFTGHM*GK.L
ATPB_MOUSE	MK_SCX_29.8497.8497.3	3	4.339	0.567	1	544.2	41.17647	R.FLSQPFQVAEVFTGHMGK.L
ATPB_MOUSE	MK_SCX_29.8558.8558.2	2	4.908	0.633	1	1245.6	70.588234	R.FLSQPFQVAEVFTGHMGK.L
ATPB_MOUSE	MK_SCX_30.5003.5003.3	3	3.85	0.488	1	964.9	48.333332	R.IMNVIGEPIDERGPIK.T
ATPB_MOUSE	MK_SCX_30.5045.5045.2	2	4.85	0.459	1	1355.6	70	R.IMNVIGEPIDERGPIK.T
ATPB_MOUSE	MK_SCX_31.5474.5474.2	2	5.126	0.66	1	2285.1	82.14286	R.LVLEVAQHLGESTVR.T
ATPB_MOUSE	MK_SCX_31.8778.8778.3	3	4.262	0.612	1	707.8	51.785713	R.LVLEVAQHLGESTVR.T
ATPB_MOUSE	MK_SCX_34.7204.7204.3	3	5.37	0.567	1	1221.3	38.636364	R.EGNDLYHEMIESGVINLKDATSK.V
ATPB_MOUSE	MK_SCX_40.6240.6240.3	3	3.274	0.36	1	946.2	36.904762	R.GQKVLDSGAPIKIPVGPETLGR.I
ATPB_MOUSE	MK_SCX_42.3864.3864.3	3	3.536	0.286	1	937.4	44.230766	K.AHGGYSVFAGVGER.T
ATPB_MOUSE	MK_SCX_42.4215.4215.3	3	3.501	0.408	1	811.9	41.17647	R.IM*NVIGEPIDERGPIKTK.Q
ATPB_MOUSE	MK_SCX_42.4228.4228.2	2	4.561	0.489	1	779.2	67.64706	R.IM*NVIGEPIDERGPIKTK.Q
ATPB_MOUSE	MK_SCX_42.4587.4587.2	2	4.903	0.581	1	1084.9	61.764706	R.IMNVIGEPIDERGPIKTK.Q
ATPB_MOUSE	MK_SCX_42.4703.4703.3	3	3.553	0.367	1	673.9	38.235294	R.IMNVIGEPIDERGPIKTK.Q
ATPB_MOUSE	MK_SCX_43.4029.4029.2	2	4.087	0.677	1	1304.2	92.30769	K.AHGGYSVFAGVGER.T
ATPB_MOUSE	MK_SCX_46.5005.5005.3	3	4.614	0.309	1	1052.5	38.157894	R.TREGNDLYHEM*IESGVINLK.D
ATPB_MOUSE	MK_SCX_46.6579.6579.2	2	6.388	0.524	1	1777.7	63.15789	R.TREGNDLYHEMIESGVINLK.D
ATPB_MOUSE	MK_SCX_46.6635.6635.3	3	5.671	0.407	1	2730.9	48.684208	R.TREGNDLYHEMIESGVINLK.D
ATPB_MOUSE	MK_SCX_51.5285.5285.3	3	5.02	0.255	1	1003	36.458336	R.TREGNDLYHEM*IESGVINLKDATSK.V
ATPB_MOUSE	MK_SCX_51.7076.7076.3	3	7.243	0.475	1	3707.8	47.916664	R.TREGNDLYHEMIESGVINLKDATSK.V
ATPD_MOUSE	MK_SCX_21.3291.3291.2	2	5.084	0.612	1	2354.5	88.46153	K.AQSELSGAADEAAR.A
ATPD_MOUSE	MK_SCX_2201.3149.3149.2	2	3.111	0.271	1	899.9	93.75	R.IEANEALVK.A
ATPG_MOUSE	MK_SCX_2201.3334.3334.2	2	3.057	0.483	1	1884.3	90	K.NEVAALTAAGK.E
ATPG_MOUSE	MK_SCX_2201.4880.4880.2	2	4.157	0.578	1	1182	86.36364	R.VYGTGSLALYEK.A
ATPG_MOUSE	MK_SCX_30.5303.5303.2	2	4.818	0.599	1	1104.6	75	K.NASDM*IDKLTLTFNR.T
ATPG_MOUSE	MK_SCX_30.5331.5331.3	3	3.804	0.487	1	792.5	42.857143	K.NASDM*IDKLTLTFNR.T
ATPG_MOUSE	MK_SCX_31.6885.6885.2	2	5.203	0.557	1	2056.7	78.57143	K.NASDMIDKLTLTFNR.T
ATPG_MOUSE	MK_SCX_31.7091.7091.3	3	4.54	0.525	1	1487.7	50	K.NASDMIDKLTLTFNR.T
ATPG_MOUSE	MK_SCX_39.4615.4615.2	2	2.967	0.446	1	394.1	70	R.THSDQFLVSFK.D
ATPG_MOUSE	MK_SCX_43.3524.3524.2	2	2.818	0.413	1	876	83.33333	K.HLIIGVSSDR.G
ATPG_MOUSE	MK_SCX_51.4784.4784.3	3	3.826	0.513	1	700.2	46.42857	R.THSDQFLVSFKDVGR.K
ATPK_MOUSE	MK_SCX_19.5246.5246.2	2	2.507	0.334	1	674.8	72.72727	R.DFTPSGIAGAFR.R
ATPK_MOUSE	MK_SCX_21.7330.7330.2	2	3.967	0.542	1	712.4	85	K.LGELPSWIMMR.D

ATPK_MOUSE	MK_SCX_21.7435.7435.1	1	3.194	0.503	1	371.5	65	K.LGELPSWIMMR.D
ATPK_MOUSE	MK_SCX_2201.5712.5712.2	2	3.25	0.511	1	578.5	80	K.LGELPSWIM*M*R.D
ATPO_MOUSE	MK_SCX_11.7634.7634.2	2	6.413	0.589	1	1933.2	66.66667	R.GEVPCTVTTASPLDDAVLSELK.T
ATPO_MOUSE	MK_SCX_11.7670.7670.3	3	5.744	0.559	1	1884.8	45.238094	R.GEVPCTVTTASPLDDAVLSELK.T
ATPO_MOUSE	MK_SCX_18.7249.7249.2	2	2.649	0.423	1	1105.5	59.375	K.FSPLTANLM*NLLAENGR.L
ATPO_MOUSE	MK_SCX_19.8213.8213.2	2	3.144	0.385	1	1512.5	71.875	K.FSPLTANLMNLLAENGR.L
ATPO_MOUSE	MK_SCX_20_1.3639.3639.2	2	2.682	0.415	1	366.6	77.27273	K.TDPSIM*GGM*IVR.I
ATPO_MOUSE	MK_SCX_20_1.4151.4151.2	2	3.383	0.219	1	549.9	86.36364	K.TDPSIM*GGMIVR.I
ATPO_MOUSE	MK_SCX_20_1.5029.5029.2	2	3.138	0.426	1	559.6	77.27273	K.TDPSIMGGMIVR.I
ATPO_MOUSE	MK_SCX_2201.3382.3382.2	2	4.277	0.553	1	982	90	R.YATALYSAASK.E
ATPO_MOUSE	MK_SCX_2201.6235.6235.3	3	3.331	0.28	1	1322	55	K.VSLAVLNPYIK.R
ATPO_MOUSE	MK_SCX_2201.6279.6279.2	2	4.735	0.464	1	1489.2	90	K.VSLAVLNPYIK.R
ATPO_MOUSE	MK_SCX_23.5135.5135.2	2	2.397	0.271	1	592.3	72.22222	K.SFLSPNQILK.L
ATPO_MOUSE	MK_SCX_28.6066.6066.3	3	3.922	0.386	1	669.9	38.333332	K.LEIKTDPSIMGGMIVR.I
ATPO_MOUSE	MK_SCX_28.8248.8248.3	3	3.206	0.397	1	351.3	30.555555	R.LGNTQGIISAFSTIMSVHR.G
ATPO_MOUSE	MK_SCX_29.7576.7576.2	2	2.489	0.307	1	486.9	44.444447	R.EKFSPLTANLM*NLLAENGR.L
ATPO_MOUSE	MK_SCX_29.7746.7746.3	3	4.053	0.445	1	1197.9	40.27778	R.EKFSPLTANLM*NLLAENGR.L
ATPO_MOUSE	MK_SCX_29.8572.8572.2	2	3.223	0.427	1	1352.2	63.88889	R.EKFSPLTANLMNLLAENGR.L
ATPO_MOUSE	MK_SCX_31.8523.8523.3	3	3.292	0.19	1	1305.4	37.5	R.EKFSPLTANLMNLLAENGR.L
ATPO_MOUSE	MK_SCX_34.7180.7180.2	2	2.626	0.332	1	926.8	69.230774	K.SFLSPNQILKLEIK.T
ATPO_MOUSE	MK_SCX_35.3989.3989.2	2	2.766	0.211	1	445.8	83.33333	K.LDQVEKELLR.V
ATPO_MOUSE	MK_SCX_35.5456.5456.2	2	3.313	0.409	1	489.3	61.538464	K.LVRPPVQVYGIEGR.Y
ATPO_MOUSE	MK_SCX_35.5775.5775.3	3	4.935	0.465	1	1995.4	59.615387	K.LVRPPVQVYGIEGR.Y
ATPO_MOUSE	MK_SCX_36.3459.3459.3	3	3.465	0.458	1	669	47.916664	R.YATALYSAASKEK.K
ATPO_MOUSE	MK_SCX_53.4052.4052.3	3	3.916	0.33	1	1111.9	57.5	K.KLDQVEKELLR.V
ATPO_MOUSE	MK_SCX_53.4056.4056.2	2	3.908	0.299	1	1179.5	85	K.KLDQVEKELLR.V
ATRX_MOUSE	MK_SCX_16.3254.3254.2	2	2.392	0.285	1	358.1	41.17647	K.SVPATVDDDDDNDPENR.I
ATRX_MOUSE	MK_SCX_20_1.9359.9359.3	3	3.072	0.184	1	395.2	23.958332	R.EAIYNDVLTKQQMLINCVQRILMNR.R
ATX2_MOUSE	MK_SCX_19.4468.4468.2	2	3.882	0.54	1	594.5	73.333336	R.LQPSSTSESMDQLLSK.N
ATX2_MOUSE	MK_SCX_19.6637.6637.2	2	4.532	0.605	1	846.5	75	K.GLPQPTISFDGIYANVR.M
ATX2_MOUSE	MK_SCX_21.5102.5102.2	2	4.323	0.449	1	1481.3	75	R.ANQLAEEIESSAQYK.A
ATX2_MOUSE	MK_SCX_21.5191.5191.2	2	5.053	0.506	1	1510.8	71.875	R.TSPAGGTWSSVVSGVPR.L
ATX2L_MOUSE	MK_SCX_51.8432.8432.3	3	3.104	0.244	1	682	32.5	R.REDIVDTMVFKPSDVLLVHFR.N
ATX2L_MOUSE	MK_SCX_53.2864.2864.3	3	3.923	0.448	1	2200	43.421055	R.IAMENDDGRTEEEKHSAVQR.Q
AUHM_MOUSE	MK_SCX_21.4787.4787.2	2	2.635	0.402	1	503	70	R.EFLPQGPVAMR.V
AUHM_MOUSE	MK_SCX_29.5314.5314.2	2	6.546	0.632	1	2791.5	71.05263	K.AVGLISHVLEQNQEGDAAYR.K
AUHM_MOUSE	MK_SCX_29.5399.5399.3	3	5.691	0.38	1	3073.6	50	K.AVGLISHVLEQNQEGDAAYR.K
AUHM_MOUSE	MK_SCX_35.10332.10332.2	2	2.203	0.242	1	621	66.66667	K.DRLEGLLAFK.E
AUHM_MOUSE	MK_SCX_36.4232.4232.2	2	3.128	0.339	1	575.7	60.714287	K.LAIIPGGGGTQRLPR.A
AUHM_MOUSE	MK_SCX_42.4906.4906.3	3	6.236	0.544	1	1876.5	48.75	K.AVGLISHVLEQNQEGDAAYRK.A
AVEN_MOUSE	MK_SCX_32.3402.3402.3	3	6.936	0.521	1	1661.5	43.47826	R.DQEPEKDGQVAQEETGPEKPSVTR.E
AVEN_MOUSE	MK_SCX_42.7603.7603.3	3	3.229	0.127	1	497.4	25.961538	R.ERPGGDREPVGAATALARGGCGDGGGR.R
AXN2_MOUSE	MK_SCX_37.14571.14571.2	2	2.034	0.155	1	310.8	45.454548	K.EEIEAEATQRVR.C
AXN2_MOUSE	MK_SCX_9.7571.7571.2	2	2.93	0.309	1	303.9	44.11765	K.ANGQVSLPHFPRTHRLPK.E
B2L13_MOUSE	MK_SCX_16.7422.7422.2	2	4.085	0.577	1	309.6	31.25	K.QQGPSPPGVQLDVAPQSLNPEVLLK.L
B2L13_MOUSE	MK_SCX_2201.2504.2504.2	2	4.223	0.469	1	2213.7	95	R.AEGAAQLSEER.A
BAD_MOUSE	MK_SCX_23.6698.6698.3	3	5.042	0.567	1	646.4	31.25	R.GLGPSLTEDQPGPYLAPGLLGSNIHQQGR.A
BAF_MOUSE	MK_SCX_21.6965.6965.3	3	4.668	0.51	1	1525.4	40.217392	R.DFVAEPM*GEKPVGSLAGIGDVLSK.R
BAF_MOUSE	MK_SCX_21.7555.7555.3	3	5.654	0.533	1	1313.1	38.04348	R.DFVAEPMGEKPVGSLAGIGDVLSK.R
BAF_MOUSE	MK_SCX_21.7588.7588.2	2	5.313	0.587	1	1765.2	58.69565	R.DFVAEPMGEKPVGSLAGIGDVLSK.R
BAF_MOUSE	MK_SCX_52.6203.6203.3	3	5.952	0.64	1	2080.9	44	K.HRDFVAEPMGEKPVGSLAGIGDVLSK.R

BAG1_MOUSE	MK_SCX_17.6709.6709.2	2	4.225	0.526	1	1384.8	82.14286	K.ILEEIDTMVLPEQFK.D
BAG1_MOUSE	MK_SCX_19.3327.3327.2	2	3.427	0.463	1	419.3	61.538464	K.TEEMVQTEEM*ETPR.L
BAG1_MOUSE	MK_SCX_44.7139.7139.3	3	6.32	0.601	1	3583.3	51.19048	K.IANHLQELNKELSGIQQGFLAK.E
BAIP2_MOUSE	MK_SCX_21.9130.9130.3	3	4.224	0.552	1	1202.1	34.615387	K.SNLVISDPIPGAKPLPVPPELAPFVGR.M
BAIP2_MOUSE	MK_SCX_2201.4768.4768.2	2	3.003	0.224	1	1188.3	75	K.TIMEQFNPSLR.N
BAIP2_MOUSE	MK_SCX_31.6099.6099.3	3	4.32	0.347	1	2595.4	43.75	K.QRPYSVAVPAFSQGLDDYGAR.S
BAIP2_MOUSE	MK_SCX_42.3899.3899.3	3	3.458	0.242	1	755.3	44.11765	R.VLDSDGSDRLHMSLQQGK.S
BAP31_MOUSE	MK_SCX_2201.2622.2622.2	2	2.154	0.224	1	589.8	72.22222	K.AENEALAMQK.Q
BAP31_MOUSE	MK_SCX_28.3777.3777.2	2	5.439	0.556	1	2167.3	75	K.GAAEDGDKLDIGNTEMK.L
BAP31_MOUSE	MK_SCX_28.3789.3789.3	3	4.691	0.34	1	1900.4	50	K.GAAEDGDKLDIGNTEMK.L
BAP31_MOUSE	MK_SCX_36.4563.4563.3	3	3.403	0.345	1	655.6	30.952381	K.GAAEDGDKLDIGNTEMKLEENK.S
BAP31_MOUSE	MK_SCX_45.3555.3555.3	3	4.579	0.525	1	1221.1	44.11765	K.KGAAEDGDKLDIGNTEMK.L
BASI_MOUSE	MK_SCX_13.6529.6529.2	2	3.528	0.497	1	775.1	64.28571	R.SGEYSCIFLPEPVGR.S
BASI_MOUSE	MK_SCX_19.8861.8861.2	2	4.152	0.497	1	323.4	60.714287	K.SDASYPPITDWFWFK.T
BASI_MOUSE	MK_SCX_2201.3262.3262.2	2	3.112	0.409	1	1100.6	83.33333	R.SEINVEGPPR.I
BASI_MOUSE	MK_SCX_29.4091.4091.2	2	4.565	0.549	1	1099	79.16667	K.VLQEDTLPDLHTK.Y
BASP_MOUSE	MK_SCX_15.4048.4048.3	3	5.173	0.665	1	1354.4	36.290325	K.SEGAAEEQPEPAPAPEQEAAAPGPAAGGEAPK.A
BASP_MOUSE	MK_SCX_17.2930.2930.2	2	6.53	0.631	1	2056.3	68.181816	K.AGEASAESTGAADGAAPEEGEAK.K
BASP_MOUSE	MK_SCX_19.3814.3814.3	3	5.304	0.58	1	1054.1	27.777779	K.AEPEKSEGAAEEQPEPAPAPEQEAAAPGPAAGGEAPK.A
BASP_MOUSE	MK_SCX_23.3645.3645.3	3	4.859	0.475	1	1595.3	36.53846	K.AEGAGTEEEGTPKESEPQAAADATEVK.E
BAT3_MOUSE	MK_SCX_25.4228.4228.3	3	5.5	0.633	1	1194.7	37.5	R.APPQTQLPSGASSGTGSASATHGGAPLPGTR.G
BAXA_MOUSE	MK_SCX_17.4378.4378.2	2	5.785	0.534	1	1247.6	63.15789	R.M*AGETPELTLEQPPQDASTK.K
BAXA_MOUSE	MK_SCX_17.4588.4588.2	2	5.906	0.626	1	1336.2	71.05263	R.MAGETPELTLEQPPQDASTK.K
BAXA_MOUSE	MK_SCX_21.7317.7317.2	2	2.169	0.199	1	415.1	58.333332	K.TGAFLLQGFIQDR.A
BAXA_MOUSE	MK_SCX_25.4415.4415.3	3	4.168	0.498	1	342	31.25	R.MAGETPELTLEQPPQDASTKK.L
BAZ1B_MOUSE	MK_SCX_28.5506.5506.3	3	3.051	0.27	1	408.9	33.82353	K.LQNEDKIISNVPADSLIR.T
BAZ1B_MOUSE	MK_SCX_46.4238.4238.3	3	4.586	0.287	1	1724.3	51.5625	R.SRPKDDPEVDDLVLQTK.R
BCAR1_MOUSE	MK_SCX_18.5567.5567.2	2	3.399	0.426	1	565.7	60.000004	K.ALYDNVAESPDELSFR.K
BCKD_MOUSE	MK_SCX_16.10876.10876.2	2	4.513	0.516	1	1507.1	81.25	R.FPFIPM*PLDYILPELLK.N
BCKD_MOUSE	MK_SCX_16.11692.11692.2	2	3.891	0.428	1	723.5	62.5	R.FPFIPMPLDYILPELLK.N
BCKD_MOUSE	MK_SCX_25.8339.8339.3	3	3.105	0.165	1	417.9	27.5	K.DLDRVM*DYHFTTAEASTQDPR.I
BCKD_MOUSE	MK_SCX_29.4155.4155.3	3	3.361	0.346	1	431.7	34.375	R.VMDYHFTTAEASTQDPR.I
BDH_MOUSE	MK_SCX_17.6439.6439.2	2	5.947	0.636	1	1443.1	71.05263	K.VSVVEPGNFIAATSLYSPER.I
BDH_MOUSE	MK_SCX_2201.5830.5830.2	2	3.383	0.469	1	1090.1	88.88889	K.MWDDLPEVVR.K
BDH_MOUSE	MK_SCX_24.4815.4815.2	2	3.021	0.498	1	1444.1	94.44444	R.VVNISSMLGR.M
BDH_MOUSE	MK_SCX_33.4953.4953.2	2	5.433	0.397	1	2751.4	88.46153	R.MQIMTHFPGAISDK.I
BDH_MOUSE	MK_SCX_33.5028.5028.3	3	3.784	0.327	1	820	42.307693	R.MQIMTHFPGAISDK.I
BDH_MOUSE	MK_SCX_39.5334.5334.2	2	3.021	0.311	1	625.5	75	K.KM*WDDLPEVVR.K
BDH_MOUSE	MK_SCX_39.6276.6276.2	2	3.981	0.397	1	1468.6	85	K.KMWDDLPEVVR.K
BDH_MOUSE	MK_SCX_43.3713.3713.2	2	4.802	0.51	1	1480.2	80.769226	K.DKGDAGVKELDSLK.S
BDH_MOUSE	MK_SCX_43.4613.4613.2	2	3.627	0.502	1	925.2	81.818184	K.GRVVNISSMLGR.M
BDH_MOUSE	MK_SCX_43.6932.6932.2	2	2.654	0.303	1	312.7	70	R.YHPMDYYWWLR.M
BDH_MOUSE	MK_SCX_48.3539.3539.3	3	4.024	0.519	1	567.3	42.857143	K.GDAGVKELDSLKSDR.L
BGAL_MOUSE	MK_SCX_2201.8172.8172.2	2	3.351	0.454	1	1843.7	86.36364	K.TLATSLYNLLAR.G
BGLR_MOUSE	MK_SCX_25.4884.4884.2	2	3.245	0.448	1	827.3	83.33333	R.LQGFEQQWYR.Q
BGLR_MOUSE	MK_SCX_43.5640.5640.2	2	2.34	0.289	1	1038.1	87.5	K.ALDGLWHFR.A
BID_MOUSE	MK_SCX_15.7811.7811.2	2	3.708	0.519	1	629	35.416664	R.ELPVQAYWEADLEDELQTDGSQASR.S
BID_MOUSE	MK_SCX_27.4863.4863.2	2	5.629	0.542	1	693.2	65.625	R.IEPDSESQEEIIHNIAR.H
BID_MOUSE	MK_SCX_44.5119.5119.3	3	4.743	0.574	1	714.8	37.5	R.HLAQIGDEMDHNIQPTLVR.Q
BID_MOUSE	MK_SCX_44.5158.5158.2	2	6.069	0.649	1	1435.9	69.44444	R.HLAQIGDEMDHNIQPTLVR.Q
BIEA_MOUSE	MK_SCX_20_1.4149.4149.2	2	4.717	0.51	1	1224	76.92308	K.SGSLEEVPNVGVNK.N

BIEA_MOUSE	MK_SCX_20_1.4692.4692.2	2	3	0.465	1	352.5	65.38461	K.LLGQVSAEDLAAEK.K
BIEA_MOUSE	MK_SCX_23.4440.4440.2	2	2.828	0.338	1	1160.2	100	K.FGVVVVGVGR.A
BIEA_MOUSE	MK_SCX_23.5986.5986.2	2	2.764	0.561	1	853.6	90	K.FGFPAFSGISR.L
BIEA_MOUSE	MK_SCX_24.4903.4903.2	2	3.194	0.409	1	1039.8	87.5	K.SPLSWIEEK.G
BIEA_MOUSE	MK_SCX_27.7828.7828.3	3	3.582	0.447	1	808.8	35	R.FTASPLEEEKFGFPAFSGISR.L
BIEA_MOUSE	MK_SCX_29.6055.6055.3	3	3.268	0.427	1	414.7	31.944445	K.SGSLEEVPNVGVNKNIFLK.D
BIEA_MOUSE	MK_SCX_33.7364.7364.3	3	5.214	0.503	1	532.3	33.653847	K.SGSLEEVPNVGVNKNIFLKDQDIFIQK.L
BIN1_MOUSE	MK_SCX_15.8406.8406.2	2	2.824	0.578	1	328.9	31.25	K.AGDVVLVIPFQNPEEQDEGWLMGVK.E
BIN1_MOUSE	MK_SCX_27.4136.4136.3	3	3.952	0.444	1	1305.6	45.588234	K.VQAQHDYTATDTDELQLK.A
BIN1_MOUSE	MK_SCX_31.3359.3359.3	3	5.13	0.537	1	1474.5	43.055553	R.VNHEPEPASGASPGATIPK.S
BL1S3_MOUSE	MK_SCX_16.4227.4227.2	2	4.411	0.53	1	672.5	70.588234	R.DPAETWGTEETPAM*APAR.S
BL1S3_MOUSE	MK_SCX_16.4753.4753.2	2	5.516	0.561	1	2513.2	76.47059	R.DPAETWGTEETPAMAPAR.S
BLMH_MOUSE	MK_SCX_11.5156.5156.3	3	3.332	0.434	1	326.1	25	K.AERLAFGESLM*THAM*TFTAVSEK.D
BLMH_MOUSE	MK_SCX_21.5997.5997.2	2	2.169	0.317	1	352.4	60.000004	K.IGPITPLQFYK.E
BLVRB_MOUSE	MK_SCX_23.6848.6848.3	3	4.874	0.576	1	1047.6	35.416664	K.YVAVMPPHIGDQPLTGAYTVTLDGR.G
BLVRB_MOUSE	MK_SCX_29.4097.4097.3	3	4.097	0.515	1	930.3	48.4375	R.LPSEGPQPAHVVVGDVR.Q
BLVRB_MOUSE	MK_SCX_36.4142.4142.3	3	4.164	0.562	1	624.3	38.75	R.DSSRLPSEGPQPAHVVVGDVR.Q
BOLA1_MOUSE	MK_SCX_19.7771.7771.2	2	2.06	0.139	1	326.2	46.666668	R.ENPQLDISPPCLGGSK.K
BOLA1_MOUSE	MK_SCX_32.5637.5637.2	2	4.831	0.51	1	1613	78.57143	R.AKLEQALSPEVLELR.N
BOLA1_MOUSE	MK_SCX_32.5904.5904.3	3	3.485	0.263	1	949.1	41.07143	R.AKLEQALSPEVLELR.N
BOLA1_MOUSE	MK_SCX_37.3187.3187.3	3	4.55	0.44	1	970.4	45.3125	R.NESGGHAVPAGSETHFR.V
BOLA1_MOUSE	MK_SCX_38.7686.7686.3	3	3.632	0.299	1	907.4	36.904762	R.LVHEALSEELAGPVHALAIQAK.T
BOLA2_MOUSE	MK_SCX_42.4244.4244.3	3	5.056	0.59	1	1950.7	48.61111	K.LRQDLEAEHVEVEDTTLNR.C
BOLA3_MOUSE	MK_SCX_48.3827.3827.3	3	4.811	0.528	1	939.4	43.75	R.TVQQHQMVNQALKEEIK.G
BPA1_MOUSE	MK_SCX_19.3235.3235.2	2	2.405	0.29	1	349.2	57.692307	R.LEQDQTSAQLQVQK.A
BPA1_MOUSE	MK_SCX_26.5266.5266.2	2	2.799	0.143	1	394.7	65	R.LPHQSVAGWGK.S
BPA1_MOUSE	MK_SCX_26.6275.6275.2	2	2.064	0.229	1	739.5	57.14286	K.DQAEALGQTSCAVPK.M
BPA1_MOUSE	MK_SCX_32.5330.5330.3	3	3.312	0.174	1	524.1	34.375	K.KTAEVLLDAKGSLLPAK.N
BPA1_MOUSE	MK_SCX_34.3963.3963.3	3	3.06	0.321	1	734.9	42.1875	K.GFHSGEDSALITTAAAR.V
BPAEA_MOUSE	MK_SCX_18.3835.3835.2	2	2.866	0.142	1	913.1	50	K.TLSVFQAM*ENRMLDR.K
BPAEA_MOUSE	MK_SCX_18.4359.4359.2	2	2.539	0.123	1	903.1	53.571426	K.TLSVFQAMENRMLDR.K
BPAEA_MOUSE	MK_SCX_34.6139.6139.2	2	2.821	0.193	1	1156.4	70	R.RLEEELLAQRR.E
BPHL_MOUSE	MK_SCX_16.10852.10852.3	3	3.195	0.181	1	353.9	26.190477	K.M*VIWGANAYVTEEDSRIYQGIR.D
BPHL_MOUSE	MK_SCX_20_1.4776.4776.2	2	4.044	0.385	1	879	81.818184	K.TDFAPQLQSLNK.K
BPHL_MOUSE	MK_SCX_23.5570.5570.2	2	3.65	0.424	1	1342.7	93.75	R.FTLVAWDPR.G
BPHL_MOUSE	MK_SCX_27.6041.6041.2	2	2.403	0.131	1	319.4	41.666664	R.VGEGEHAILLLPGM*LGSGK.T
BPHL_MOUSE	MK_SCX_27.6909.6909.3	3	4.006	0.383	1	740	40.27778	R.VGEGEHAILLLPGMLGSGK.T
BPHL_MOUSE	MK_SCX_27.6995.6995.2	2	5.604	0.53	1	1701	69.44444	R.VGEGEHAILLLPGMLGSGK.T
BPHL_MOUSE	MK_SCX_30.7468.7468.3	3	6.256	0.513	1	1297.7	35	R.VGEGEHAILLLPGM*LGSGKTDFAPQLQSLNK.K
BPHL_MOUSE	MK_SCX_30.8103.8103.3	3	7.71	0.597	1	1782.2	34.166668	R.VGEGEHAILLLPGMLGSGKTDFAPQLQSLNK.K
BPHL_MOUSE	MK_SCX_33.4204.4204.2	2	3.665	0.431	1	826.2	79.16667	K.TDFAPQLQSLNKK.R
BPHL_MOUSE	MK_SCX_34.5626.5626.2	2	4.218	0.552	1	1664	80.769226	R.KPLEALYGYDYLAK.T
BPHL_MOUSE	MK_SCX_34.5642.5642.3	3	3.894	0.503	1	1949.5	57.692307	R.KPLEALYGYDYLAK.T
BPHL_MOUSE	MK_SCX_52.5266.5266.2	2	4.756	0.583	1	1557.2	76.666664	K.ARKPLEALYGYDYLAK.T
BPHL_MOUSE	MK_SCX_52.5290.5290.3	3	4.276	0.513	1	1234.2	48.333332	K.ARKPLEALYGYDYLAK.T
BPHL_MOUSE	MK_SCX_53.4888.4888.3	3	4.285	0.417	1	1774	62.5	R.FHADFLLQHVK.G
BPHL_MOUSE	MK_SCX_53.4937.4937.2	2	3.79	0.527	1	1446.7	90	R.FHADFLLQHVK.G
BPNT1_MOUSE	MK_SCX_17.6001.6001.2	2	5.121	0.669	1	1068.5	56.81818	K.AIAGIINQPYYNYQAGPDAALGR.T
BPNT1_MOUSE	MK_SCX_2201.3967.3967.2	2	2.749	0.154	1	832.1	92.85714	K.IIQLIEGK.A
BPNT1_MOUSE	MK_SCX_35.11153.11153.2	2	3.048	0.413	1	349.8	60.714287	R.VGGAGNKIIQLIEGK.A
BPNT1_MOUSE	MK_SCX_35.4198.4198.2	2	5.076	0.48	1	1662.7	84.61539	R.NYEYYASHVPESVK.N

BPNT1_MOUSE	MK_SCX_45.3822.3822.2	2	2.638	0.453	1	701.5	72.72727	K.HM*NSAGVLAALR.N
BPNT1_MOUSE	MK_SCX_45.4358.4358.2	2	3.802	0.507	1	1665.8	95.454544	K.HMNSAGVLAALR.N
BPNT1_MOUSE	MK_SCX_45.4369.4369.3	3	3.713	0.303	1	1454.1	52.272724	K.HMNSAGVLAALR.N
BPNT1_MOUSE	MK_SCX_47.3664.3664.3	3	4.415	0.536	1	778.3	46.666668	K.LTDIHGNALQYNKEVK.H
BR44_MOUSE	MK_SCX_2201.6668.6668.2	2	2.369	0.215	1	414.7	66.66667	R.TVFFWAPIM*K.W
BR44_MOUSE	MK_SCX_2201.7764.7764.2	2	2.808	0.319	1	784.9	72.22222	R.TVFFWAPIMK.W
BR44_MOUSE	MK_SCX_27.4413.4413.2	2	2.008	0.177	1	377.5	71.42857	R.YNQELKSK.G
BR44_MOUSE	MK_SCX_32.4767.4767.3	3	3.794	0.219	1	1719.3	62.5	R.LM*DKVELLLPK.K
BR44_MOUSE	MK_SCX_32.4815.4815.2	2	2.88	0.294	1	804.3	80	R.LM*DKVELLLPK.K
BR44_MOUSE	MK_SCX_32.5576.5576.3	3	3.748	0.263	1	1475.3	62.5	R.LMDKVELLLPK.K
BR44_MOUSE	MK_SCX_32.5616.5616.2	2	4.316	0.352	1	1461.3	90	R.LMDKVELLLPK.K
BRCC3_MOUSE	MK_SCX_10.7570.7570.2	2	3.014	0.132	1	791.8	56.666668	K.EEVMGLCIGELNDDIR.S
BRCC3_MOUSE	MK_SCX_18.4712.4712.2	2	5.131	0.543	1	2052.1	75	R.VEISPEQLSAASTEAER.L
BRD4_MOUSE	MK_SCX_15.5352.5352.2	2	2.443	0.392	1	423.7	40.476192	K.MPDEPEEPVVTVSSPAVPPPTK.V
BRD4_MOUSE	MK_SCX_34.4925.4925.3	3	4.744	0.542	1	781.1	31.52174	R.KADTTTPTTIDPIHEPPSLAPEPK.T
BRE1A_MOUSE	MK_SCX_44.5489.5489.3	3	4.347	0.418	1	1025.2	45.3125	K.LLKEEKEELADQVLTLK.T
BRE1B_MOUSE	MK_SCX_18.5793.5793.2	2	2.117	0.176	1	600.3	65	K.LLLDM*YKSAPK.E
BRE1B_MOUSE	MK_SCX_23.3551.3551.2	2	3.239	0.138	1	1160.6	94.44444	K.LQAELQGAVR.T
BRE1B_MOUSE	MK_SCX_30.5264.5264.2	2	2.055	0.225	1	351.9	50	K.ELTLRSQALELNK.R
BRE1B_MOUSE	MK_SCX_45.5411.5411.3	3	3.251	0.409	1	568	39.0625	K.LLREEKDELGEQVLGLK.S
BSND_MOUSE	MK_SCX_16.6605.6605.2	2	4.024	0.505	1	417.1	45.454548	R.FSDFALIDDTPTSEDTVLDGQAR.E
BSND_MOUSE	MK_SCX_25.7217.7217.3	3	4.258	0.484	1	502.3	30.263159	R.LWEEAAYDQSLPDFTHIQMK.V
BSND_MOUSE	MK_SCX_25.7297.7297.2	2	5.438	0.572	1	1303.2	65.789474	R.LWEEAAYDQSLPDFTHIQMK.V
BSND_MOUSE	MK_SCX_27.5771.5771.2	2	3.406	0.388	1	534.3	59.375	K.VMGYSEDPRPLLAPELK.T
BSND_MOUSE	MK_SCX_27.5911.5911.3	3	3.109	0.299	1	391	34.375	K.VMGYSEDPRPLLAPELK.T
BSND_MOUSE	MK_SCX_32.4679.4679.3	3	4.53	0.526	1	884.8	50	R.TAQAWMEAPVVVHR.G
BSND_MOUSE	MK_SCX_32.4733.4733.2	2	4.4	0.511	1	1933.2	80.769226	R.TAQAWMEAPVVVHR.G
BTD_MOUSE	MK_SCX_19.7807.7807.2	2	3.756	0.57	1	629.5	69.230774	R.TSIYPFLDFMPSPK.L
BTF3_MOUSE	MK_SCX_14.6875.6875.2	2	5.457	0.629	1	825	52.083332	K.APLATGEDDDDEVPDLVENFDEASK.N
BTF3_MOUSE	MK_SCX_17.8599.8599.2	2	4.597	0.487	1	1040.2	52.380955	K.QLTEM*LPSILNQLGADSLTSLR.R
BTF3_MOUSE	MK_SCX_17.9440.9440.2	2	5.196	0.465	1	1251.6	66.66667	K.QLTEMLPSILNQLGADSLTSLR.R
BTF3_MOUSE	MK_SCX_17.9482.9482.3	3	3.66	0.164	1	567.7	30.952381	K.QLTEMLPSILNQLGADSLTSLR.R
BTF3_MOUSE	MK_SCX_23.7950.7950.3	3	5.438	0.292	1	1264.9	38.88889	K.LGVNNISGIEEVNMFTNQGTVIHFNNPK.V
C1QR1_MOUSE	MK_SCX_2201.5473.5473.2	2	3.135	0.166	1	1125.1	77.27273	R.CNENGGNLATVK.S
C1QR1_MOUSE	MK_SCX_41.4365.4365.2	2	3.439	0.595	1	1211.9	90	R.HVQQALTQLLK.T
C1TC_MOUSE	MK_SCX_15.9530.9530.2	2	4.286	0.66	1	634.1	42.857143	R.ASFITPVPGGVGPMTVAMLMQSTVESAQR.F
C1TC_MOUSE	MK_SCX_15.9674.9674.3	3	3.792	0.461	1	961.4	31.25	R.ASFITPVPGGVGPMTVAMLMQSTVESAQR.F
C1TC_MOUSE	MK_SCX_18.3864.3864.2	2	4.597	0.361	1	1071.1	87.5	K.TDPTTLTDDEINR.F
C1TC_MOUSE	MK_SCX_18.5560.5560.2	2	2.761	0.145	1	548.1	56.666668	R.IYGADDIELLPEAQNK.A
C1TC_MOUSE	MK_SCX_19.4248.4248.2	2	2.987	0.322	1	440.7	63.636364	K.AYTEEDLDLVEK.G
C1TC_MOUSE	MK_SCX_19.8216.8216.2	2	2.142	0.196	1	598.2	60.714287	K.TDTDAELDLVSRLSR.E
C1TC_MOUSE	MK_SCX_2201.2574.2574.2	2	2.071	0.3	1	719.8	85.71429	K.AAEEIGIK.A
C43BP_MOUSE	MK_SCX_26.3722.3722.3	3	4.242	0.206	1	1163.5	51.785713	R.DKVVEDDEDDFPTTR.S
C43BP_MOUSE	MK_SCX_35.10829.10829.2	2	2.377	0.168	1	608.4	68.181816	K.FLKRFTSYVQEK.T
CABC1_MOUSE	MK_SCX_26.15172.15172.3	3	3.881	0.476	1	527.4	28.260868	K.LTQAAVETHLQNLGLGGELLLAAR.A
CABC1_MOUSE	MK_SCX_50.6633.6633.3	3	3.213	0.437	1	529	29.545454	R.DKLEYFEERPFAAASIGQVHLAR.M
CACP_MOUSE	MK_SCX_18.7318.7318.2	2	3.865	0.532	1	693.1	67.85714	R.LPVPPLQQSLDYYLK.A
CAD16_MOUSE	MK_SCX_14.7730.7730.2	2	4.615	0.653	1	920.1	41.666664	K.LDQESYETSIPVSTPAGSLLLTIQPSDPM*SR.T
CAD16_MOUSE	MK_SCX_14.8185.8185.2	2	4.381	0.709	1	1029.5	40	K.LDQESYETSIPVSTPAGSLLLTIQPSDPMSR.T
CAD16_MOUSE	MK_SCX_16.5231.5231.2	2	3.691	0.283	1	621.6	71.42857	K.DENDQVPQFSQAIYR.A
CAD16_MOUSE	MK_SCX_17.4938.4938.2	2	6.111	0.609	1	1543.3	66.66667	R.QDYGVVVSGVSEDPDLANR.N

CAD16_MOUSE	MK_SCX_18.12506.12506.3	3	5.803	0.54	1	1332.1	32.258064	R.DEGHIVLSGDSNTADQNTFAVDTDSGFLVATR.T
CAD16_MOUSE	MK_SCX_18.6838.6838.3	3	5.145	0.609	1	1465.9	31.617647	R.QDYGVVVSGVSEDPDLANRNGPYSFALGPNPTVQR.D
CAD16_MOUSE	MK_SCX_18.8199.8199.3	3	5.009	0.616	1	703.2	25	R.VVAPLKLDQESYETSIPVSTPAGSLLLTIQPSDPM*SR.T
CAD16_MOUSE	MK_SCX_18.8498.8498.3	3	5.663	0.551	1	1078.9	29.166666	R.VVAPLKLDQESYETSIPVSTPAGSLLLTIQPSDPMSR.T
CAD16_MOUSE	MK_SCX_19.10241.10241.3	3	6.421	0.618	1	1303.5	33.035713	R.LMDFAIEEGDPEGIFDLSWEPDSDHVQLR.L
CAD16_MOUSE	MK_SCX_19.6514.6514.2	2	2.927	0.309	1	431.2	64.28571	K.AEYQLQVTLESEDGR.I
CAD16_MOUSE	MK_SCX_20_1.5045.5045.2	2	4.309	0.484	1	1187.1	75	K.ASPVPALTLSAGPSR.H
CAD16_MOUSE	MK_SCX_20_1.7631.7631.3	3	6.84	0.577	1	1111	32.258064	R.LSAEDLDAPGSPNSHIVYQLLSPEPEEGAENK.A
CAD16_MOUSE	MK_SCX_21.5093.5093.2	2	4.451	0.533	1	957.2	66.66667	R.NGPYSFALGPNPTVQR.D
CAD16_MOUSE	MK_SCX_2201.3618.3618.2	2	4.496	0.453	1	1720.3	90	R.EAQAEYQLQVR.A
CAD16_MOUSE	MK_SCX_2201.7578.7578.2	2	4.103	0.522	1	301.1	32.258064	R.AQLSQGTRPGVPFLFLEASDGDAPGTANSDLR.F
CAD16_MOUSE	MK_SCX_23.7314.7314.3	3	7.192	0.625	1	1183.7	32.258064	R.AQLSQGTRPGVPFLFLEASDGDAPGTANSDLR.F
CAD16_MOUSE	MK_SCX_33.7967.7967.3	3	6.234	0.638	1	1228.3	36.11111	R.ILWGPQLVTVHVKDENDQVPQFSQAIYR.A
CAD16_MOUSE	MK_SCX_34.6163.6163.2	2	2.64	0.262	1	483.9	70.83333	R.ILWGPQLVTVHVK.D
CADH1_MOUSE	MK_SCX_14.5896.5896.3	3	6.346	0.582	1	1867.4	44	K.DINDNAPVFNPSTYQGQVPENEVNAR.I
CADH1_MOUSE	MK_SCX_14.6942.6942.3	3	6.101	0.509	1	1916.4	40.384613	K.AVYTVVNDPDQQFVVVTDPTTNDGILK.T
CADH1_MOUSE	MK_SCX_14.6958.6958.2	2	5.429	0.583	1	1644.2	50	K.AVYTVVNDPDQQFVVVTDPTTNDGILK.T
CADH1_MOUSE	MK_SCX_15.5802.5802.2	2	5.259	0.638	1	776.4	46	K.DINDNAPVFNPSTYQGQVPENEVNAR.I
CADH1_MOUSE	MK_SCX_16.7341.7341.2	2	6.016	0.486	1	1545.9	67.64706	R.DTANWLEINPETGAIFTR.A
CADH1_MOUSE	MK_SCX_17.6164.6164.2	2	5.066	0.659	1	1437.6	77.77778	R.VEVPEDFGVGQEITSYTAR.E
CADH1_MOUSE	MK_SCX_20_1.3642.3642.2	2	4.316	0.423	1	1127.4	75	K.VTDDDAPNTPAWK.A
CADH1_MOUSE	MK_SCX_2201.6820.6820.3	3	4.02	0.474	1	613.5	25	K.AVITVKDINDNAPVFNPSTYQGQVPENEVNAR.I
CADH1_MOUSE	MK_SCX_25.7025.7025.3	3	4.037	0.545	1	1311.1	46.42857	K.VFYSITGQGADKPPVGVFIIER.E
CADH1_MOUSE	MK_SCX_25.7053.7053.2	2	5.915	0.646	1	1188.1	64.28571	K.VFYSITGQGADKPPVGVFIIER.E
CADH1_MOUSE	MK_SCX_30.6253.6253.3	3	3.032	0.229	1	812.7	34.210526	R.RVEVPEDFGVGQEITSYTAR.E
CADH1_MOUSE	MK_SCX_33.7384.7384.3	3	5.262	0.61	1	793.8	34.82143	R.RVEVPEDFGVGQEITSYTAREPDTFMDQK.I
CAH12_MOUSE	MK_SCX_26.9004.9004.3	3	3.406	0.438	1	529.7	30.000002	K.YKGQQVLIPGFNIEELLPESPGEYYR.Y
CAH12_MOUSE	MK_SCX_50.5429.5429.2	2	5.362	0.58	1	1534.2	67.64706	R.LNLNSDMYIQGLQPHHYR.A
CAH12_MOUSE	MK_SCX_50.5543.5543.3	3	3.483	0.243	1	1044.2	42.647057	R.LNLNSDMYIQGLQPHHYR.A
CAH14_MOUSE	MK_SCX_20_1.9113.9113.2	2	3.278	0.439	1	710.8	72.72727	R.ELFPQQLEQFFR.Y
CAH14_MOUSE	MK_SCX_2201.6206.6206.3	3	5.568	0.613	1	1077.5	33.92857	K.LQETLSSTEEDPSEPLVQNYRVPQPLNQR.T
CAH15_MOUSE	MK_SCX_25.8948.8948.3	3	5.521	0.619	1	941.8	29.83871	R.DYTLKPFIFQGYDSAPQDPWVLENDGHTVLLR.V
CAH2_MOUSE	MK_SCX_2201.6304.6304.3	3	5.394	0.322	1	2213.9	40.909092	K.SIVNNGHSFNVEFDDSQDNAVLK.G
CAH2_MOUSE	MK_SCX_23.6109.6109.2	2	3.82	0.524	1	1133.6	47.727272	K.SIVNNGHSFNVEFDDSQDNAVLK.G
CAH2_MOUSE	MK_SCX_24.15508.15508.3	3	6.402	0.652	1	994.6	34.375	R.TLNFNEEGDAEEAMVDNWRPAQPLK.N
CAH2_MOUSE	MK_SCX_24.2758.2758.2	2	2.89	0.407	1	352.9	77.77778	K.IGPASQGLQK.V
CAH2_MOUSE	MK_SCX_28.6945.6945.3	3	4.572	0.418	1	2178.3	34.677418	K.SIVNNGHSFNVEFDDSQDNAVLKGGPLSDSYR.L
CAH2_MOUSE	MK_SCX_36.6016.6016.3	3	4.153	0.529	1	899.3	28.846153	R.TLNFNEEGDAEEAM*VDNWRPAQPLKNR.K
CAH2_MOUSE	MK_SCX_36.6800.6800.3	3	5.188	0.498	1	953.9	29.807693	R.TLNFNEEGDAEEAMVDNWRPAQPLKNR.K
CAH2_MOUSE	MK_SCX_51.6224.6224.3	3	5.104	0.537	1	719.9	24.074074	R.TLNFNEEGDAEEAMVDNWRPAQPLKNRK.I
CAH3_MOUSE	MK_SCX_25.7488.7488.3	3	5.709	0.601	1	660.7	30.208334	R.SLFSSAENEPPVPLVGNWRPPQPVK.G
CAH3_MOUSE	MK_SCX_31.4514.4514.3	3	3.164	0.338	1	613.3	33.82353	K.HDPSLQPWSASYDPGSAK.T
CAH3_MOUSE	MK_SCX_31.4543.4543.2	2	3.969	0.574	1	642.3	58.823532	K.HDPSLQPWSASYDPGSAK.T
CAH4_MOUSE	MK_SCX_20_1.6933.6933.2	2	3.055	0.298	1	755.7	72.72727	R.LTPFILVGYDQK.Q
CAH4_MOUSE	MK_SCX_29.5248.5248.3	3	3.068	0.125	1	482.8	28.260868	K.NNQHTVEMTLGGGACIIGGDLPAR.Y
CAH4_MOUSE	MK_SCX_8.5766.5766.2	2	2.395	0.171	1	562.5	50	K.LNMKDNVRPLQPLGKR.Q
CALB1_MOUSE	MK_SCX_20_1.6631.6631.2	2	3.023	0.51	1	452.1	80	R.LLPVQENFLLK.F
CALB1_MOUSE	MK_SCX_20_1.9816.9816.2	2	3.728	0.186	1	1132.7	70.83333	K.ELQNLIQELLQAR.K
CALB1_MOUSE	MK_SCX_21.4499.4499.2	2	3.623	0.501	1	1413.1	88.88889	K.LAEYTDLM*LK.L
CALB1_MOUSE	MK_SCX_21.4986.4986.2	2	3.404	0.445	1	1080.7	88.88889	K.LAEYTDLMLK.L
CALB1_MOUSE	MK_SCX_2201.3010.3010.2	2	2.496	0.229	1	755.2	85.71429	K.LELTEM*AR.L

CALB1_MOUSE	MK_SCX_26.6121.6121.3	3	4.634	0.461	1	438.1	45	K.TVDDTKLAEYTDLMLK.L
CALB1_MOUSE	MK_SCX_26.6212.6212.2	2	4.038	0.435	1	2429.9	80	K.TVDDTKLAEYTDLMLK.L
CALB1_MOUSE	MK_SCX_28.16811.16811.3	3	3.909	0.322	1	680	42.1875	K.LFDSNNDGKLELTEM*AR.L
CALB1_MOUSE	MK_SCX_28.4339.4339.2	2	5.477	0.533	1	2873.8	89.28571	K.YDTDHSGFIETEELK.N
CALB1_MOUSE	MK_SCX_28.5282.5282.2	2	5.469	0.601	1	1618.2	71.875	K.LFDSNNDGKLELTEMAR.L
CALB1_MOUSE	MK_SCX_28.6266.6266.3	3	4.646	0.426	1	1070.6	46.875	K.LFDSNNDGKLELTEMAR.L
CALB1_MOUSE	MK_SCX_37.3942.3942.2	2	3.537	0.408	1	518.9	66.66667	K.NIM*ALSDGGKLYR.T
CALB1_MOUSE	MK_SCX_37.4765.4765.2	2	4.228	0.459	1	1612.5	83.33333	K.NIMALSDGGKLYR.T
CALB1_MOUSE	MK_SCX_40.6328.6328.2	2	2.495	0.171	1	889.1	87.5	K.NFLKDLLEK.A
CALB1_MOUSE	MK_SCX_42.5843.5843.3	3	3.062	0.201	1	456.8	36.11111	K.ANKTVDDTKLAEYTDLMLK.L
CALB1_MOUSE	MK_SCX_45.9607.9607.3	3	4.593	0.417	1	793.4	32.608696	K.YDTDHSGFIETEELKNFLKDLLEK.A
CALB1_MOUSE	MK_SCX_46.4091.4091.3	3	5.07	0.392	1	1947.3	55	R.KYDTDHSGFIETEELK.N
CALB1_MOUSE	MK_SCX_46.4093.4093.2	2	5.314	0.462	1	2131.5	80	R.KYDTDHSGFIETEELK.N
CALB1_MOUSE	MK_SCX_49.3928.3928.2	2	3.425	0.161	1	1379.4	71.42857	K.NKQELDINNITTYKK.N
CALB1_MOUSE	MK_SCX_51.3834.3834.3	3	4.693	0.266	1	1316	48.214287	K.NKQELDINNITTYKK.N
CALB1_MOUSE	MK_SCX_52.6128.6128.2	2	5.831	0.565	1	1832.6	68.42105	R.KYDTDHSGFIETEELKNFLK.D
CALB1_MOUSE	MK_SCX_52.6159.6159.3	3	7	0.562	1	3572.2	53.947372	R.KYDTDHSGFIETEELKNFLK.D
CALB1_MOUSE	MK_SCX_54.3834.3834.3	3	3.037	0.405	1	599.6	46.153847	K.KNIMALSDGGKLYR.T
CALU_MOUSE	MK_SCX_23.8056.8056.3	3	3.456	0.352	1	638.4	28.260868	K.EEIVDKYDLFVGSQATDFGEALVR.H
CALU_MOUSE	MK_SCX_25.4757.4757.2	2	5.106	0.404	1	2455.9	85.71429	K.IDDDKDGFVTVDELK.G
CALU_MOUSE	MK_SCX_48.4923.4923.3	3	5.213	0.482	1	1399.9	38.636364	K.MDKEETKDWILPSDYDHAEAEAR.H
CALU_MOUSE	MK_SCX_51.1738.1738.3	3	3.693	0.521	1	670.8	50	R.HLVYESDQNKDGK.L
CALX_MOUSE	MK_SCX_18.5734.5734.2	2	4.637	0.556	1	1177.4	73.333336	K.APVPTGEVYFADSFDR.G
CALX_MOUSE	MK_SCX_19.7813.7813.2	2	3.721	0.402	1	883.7	65.38461	K.IPNPDFFEDLEPFK.M
CALX_MOUSE	MK_SCX_21.5574.5574.2	2	3.369	0.535	1	680.4	66.66667	R.VVDDWANDGWGLK.K
CALX_MOUSE	MK_SCX_24.4316.4316.3	3	4.096	0.577	1	795.1	40.27778	K.IADPDAVKPDDWDEDAPSK.I
CALX_MOUSE	MK_SCX_24.4895.4895.2	2	3.256	0.379	1	1051.6	77.77778	R.GSLSGWILSK.A
CALX_MOUSE	MK_SCX_27.16436.16436.3	3	4.211	0.442	1	1011.7	36.842106	K.VTYKAPVPTGEVYFADSFDR.G
CALX_MOUSE	MK_SCX_27.6589.6589.2	2	3.991	0.582	1	1018.5	60.526318	K.VTYKAPVPTGEVYFADSFDR.G
CALX_MOUSE	MK_SCX_31.4076.4076.3	3	3.418	0.336	1	463.6	45.454548	K.TAELSLDQFHDK.T
CALX_MOUSE	MK_SCX_31.4093.4093.2	2	3.91	0.374	1	1435.2	81.818184	K.TAELSLDQFHDK.T
CALX_MOUSE	MK_SCX_31.5132.5132.2	2	3.835	0.458	1	333.3	40	K.CESAPGCGVWQRPMIDNPNYK.G
CALX_MOUSE	MK_SCX_32.8015.8015.2	2	4.288	0.516	1	1012.1	75	R.KIPNPDFFEDLEPFK.M
CALX_MOUSE	MK_SCX_32.8407.8407.3	3	5.353	0.479	1	2321.6	55.35714	R.KIPNPDFFEDLEPFK.M
CALX_MOUSE	MK_SCX_33.4805.4805.2	2	4.262	0.43	1	1388.5	73.07692	R.VVDDWANDGWGLKK.A
CALX_MOUSE	MK_SCX_35.4314.4314.3	3	3.659	0.383	1	522.7	43.18182	K.LPGDKGLVLMSR.A
CALX_MOUSE	MK_SCX_35.4392.4392.2	2	3.15	0.32	1	694.2	86.36364	K.LPGDKGLVLMSR.A
CALX_MOUSE	MK_SCX_45.4668.4668.3	3	3.656	0.366	1	699.3	32.954548	K.CESAPGCGVWQRPM*IDNPNYKGK.W
CALX_MOUSE	MK_SCX_45.4902.4902.3	3	4.088	0.472	1	945.3	36.363636	K.CESAPGCGVWQRPMIDNPNYKGK.W
CALX_MOUSE	MK_SCX_45.4932.4932.2	2	2.798	0.18	1	321.8	38.636364	K.CESAPGCGVWQRPMIDNPNYKGK.W
CALX_MOUSE	MK_SCX_49.3848.3848.3	3	4.223	0.506	1	1604.9	55.76923	K.YDGKWEVDEMKETK.L
CALX_MOUSE	MK_SCX_49.3884.3884.2	2	4.799	0.48	1	852.4	69.230774	K.YDGKWEVDEMKETK.L
CALX_MOUSE	MK_SCX_51.6254.6254.2	2	3.213	0.528	1	606.3	70.588234	K.WKPPMIDNPNYQGIWKPR.K
CALX_MOUSE	MK_SCX_53.3661.3661.3	3	4.16	0.4	1	840.6	38.235294	R.EIEDPEDRKPEDWDERPK.I
CALX_MOUSE	MK_SCX_53.3693.3693.2	2	2.659	0.146	1	408.3	47.058823	R.EIEDPEDRKPEDWDERPK.I
CAND1_MOUSE	MK_SCX_33.5421.5421.2	2	2.118	0.169	1	325.8	50	K.ISGSILNELIGLVR.S
CAND1_MOUSE	MK_SCX_41.4248.4248.2	2	2.902	0.455	1	882.7	81.25	R.HEMLPEFYK.T
CAP1_MOUSE	MK_SCX_16.7385.7385.2	2	3.995	0.519	1	440.5	47.916664	K.SSEM*NVLIPTEGGDFNEFPVPEQFK.T
CAP1_MOUSE	MK_SCX_16.7847.7847.2	2	4.696	0.538	1	370.5	45.833336	K.SSEMNVLIPTEGGDFNEFPVPEQFK.T
CAP1_MOUSE	MK_SCX_16.9516.9516.2	2	5.191	0.632	1	2311.5	64.28571	K.GAVPYVQAFDSLLANPVAEYLK.M
CAP1_MOUSE	MK_SCX_16.9564.9564.3	3	5.328	0.439	1	2127.5	50	K.GAVPYVQAFDSLLANPVAEYLK.M

CAP1_MOUSE	MK_SCX_16.9720.9720.3	3	4.062	0.385	1	459.8	20.3125	K.GAVPYVQAFDSLLANPVAEYLKM*SKEIGGDVQK.H
CAP1_MOUSE	MK_SCX_53.3103.3103.3	3	3.981	0.516	1	780.6	33.333336	R.SGPKPFSAPKPQTSPSPKPATK.K
CAP1_MOUSE	MK_SCX_7.4678.4678.2	2	2.011	0.442	1	423.4	65	K.NSLDCEIVSAK.S
CAPZB_MOUSE	MK_SCX_31.5259.5259.2	2	4.638	0.581	1	701.9	63.15789	R.SPWSNKYDPPLEDGAMPSAR.L
CAPZB_MOUSE	MK_SCX_31.5296.5296.3	3	5.045	0.468	1	1358.5	44.736843	R.SPWSNKYDPPLEDGAMPSAR.L
CATA_MOUSE	MK_SCX_13.6198.6198.2	2	3.511	0.408	1	765.3	64.28571	R.LGPNYLQIPVNCPYR.A
CATA_MOUSE	MK_SCX_15.5424.5424.3	3	3.418	0.284	1	500	23.148148	R.DGPM*CM*HDNQGGAPNYYPNSFSAPEQQR.S
CATA_MOUSE	MK_SCX_15.5506.5506.3	3	5.907	0.583	1	1773	37.962963	R.DGPMCMHDNQGGAPNYYPNSFSAPEQQR.S
CATA_MOUSE	MK_SCX_15.5517.5517.2	2	5.366	0.648	1	1126.5	48.148148	R.DGPMCMHDNQGGAPNYYPNSFSAPEQQR.S
CATA_MOUSE	MK_SCX_15.8758.8758.2	2	5.224	0.592	1	982.7	42.857143	K.NPVNYFAEVEQMAFDPSNMPPGIEPSPDK.M
CATA_MOUSE	MK_SCX_17.8368.8368.2	2	5.42	0.707	1	1601.6	65	K.FYTEDGNWDLVGNNTPIFFIR.D
CATA_MOUSE	MK_SCX_17.8465.8465.3	3	4.409	0.429	1	586.9	36.25	K.FYTEDGNWDLVGNNTPIFFIR.D
CATA_MOUSE	MK_SCX_20_1.10453.10453.3	3	6.278	0.209	1	1525.5	29.545454	K.LVLNKNPVNYFAEVEQMAFDPSNM*PPGIEPSPDK.M
CATA_MOUSE	MK_SCX_20_1.11036.11036.3	3	5.928	0.583	1	843.2	27.272728	K.LVLNKNPVNYFAEVEQMAFDPSNMPPGIEPSPDK.M
CATA_MOUSE	MK_SCX_20_1.9514.9514.3	3	6.355	0.588	1	1654	31.060606	K.LVLNKNPVNYFAEVEQM*AFDPSNM*PPGIEPSPDK.M
CATA_MOUSE	MK_SCX_20_1.9858.9858.3	3	6.242	0.255	1	1045.8	27.272728	K.LVLNKNPVNYFAEVEQM*AFDPSNMPPGIEPSPDK.M
CATA_MOUSE	MK_SCX_23.3371.3371.2	2	4.537	0.502	1	1548.9	79.16667	R.FNSANEDNVTQVR.T
CATA_MOUSE	MK_SCX_23.4368.4368.2	2	3.506	0.554	1	726.5	73.07692	R.SALEHSVQCAVDVK.R
CATA_MOUSE	MK_SCX_25.15335.15335.3	3	3.438	0.41	1	585.8	34.72222	R.GPLLVQDVVFTDEMAHFDR.E
CATA_MOUSE	MK_SCX_25.7845.7845.2	2	6.466	0.64	1	2394	77.77778	R.GPLLVQDVVFTDEMAHFDR.E
CATA_MOUSE	MK_SCX_28.5977.5977.2	2	3.427	0.244	1	528.8	66.66667	R.DAILFPSFIHSQK.R
CATA_MOUSE	MK_SCX_31.3756.3756.3	3	4.234	0.537	1	1045.9	44.444447	R.ASQRPDVLTTGGGNPIGDK.L
CATA_MOUSE	MK_SCX_31.3758.3758.2	2	5.094	0.581	1	1750.1	66.66667	R.ASQRPDVLTTGGGNPIGDK.L
CATA_MOUSE	MK_SCX_32.15847.15847.3	3	3.808	0.535	1	1053.3	51.666664	K.GAGAFGYFEVTHDITR.Y
CATA_MOUSE	MK_SCX_32.6040.6040.2	2	4.801	0.57	1	2013.3	76.666664	K.GAGAFGYFEVTHDITR.Y
CATA_MOUSE	MK_SCX_33.3616.3616.3	3	3.03	0.316	1	734.6	41.07143	K.TDQGIKNLPVGEAGR.L
CATA_MOUSE	MK_SCX_35.3569.3569.2	2	3.998	0.426	1	1112.5	81.818184	K.NFTDVHPDYGAR.I
CATA_MOUSE	MK_SCX_37.14093.14093.3	3	3.249	0.317	1	371.3	28.75	R.GPLLVQDVVFTDEMAHFDRER.I
CATA_MOUSE	MK_SCX_37.3525.3525.2	2	3.246	0.247	1	1149.3	63.333332	K.NAIHTYTQAGSHMAAK.G
CATA_MOUSE	MK_SCX_37.5511.5511.3	3	6.386	0.618	1	891	35.185184	R.ASQRPDVLTTGGGNPIGDKLNIM*TAGSR.G
CATA_MOUSE	MK_SCX_37.6334.6334.2	2	5.278	0.622	1	1041	55.555557	R.ASQRPDVLTTGGGNPIGDKLNIMTAGSR.G
CATA_MOUSE	MK_SCX_37.6507.6507.3	3	6.699	0.662	1	2324	37.962963	R.ASQRPDVLTTGGGNPIGDKLNIMTAGSR.G
CATA_MOUSE	MK_SCX_45.3147.3147.2	2	6.355	0.541	1	1500.3	84.61539	K.RFNSANEDNVTQVR.T
CATA_MOUSE	MK_SCX_45.3181.3181.3	3	4.807	0.479	1	1884	59.615387	K.RFNSANEDNVTQVR.T
CATA_MOUSE	MK_SCX_46.5621.5621.2	2	3.714	0.33	1	882.6	76.92308	R.DAILFPSFIHSQKR.N
CATA_MOUSE	MK_SCX_49.3783.3783.3	3	3.775	0.446	1	685.6	50	R.IQALLDKYNAEKPK.N
CATA_MOUSE	MK_SCX_49.3796.3796.2	2	4.954	0.55	1	1813.8	84.61539	R.IQALLDKYNAEKPK.N
CATA_MOUSE	MK_SCX_51.3015.3015.3	3	3.667	0.343	1	848.1	45	K.NAIHTYTQAGSHMAAK.G
CATA_MOUSE	MK_SCX_51.3491.3491.3	3	3.287	0.398	1	665.1	39.285713	K.AVKNFTDVHPDYGAR.I
CATA_MOUSE	MK_SCX_51.4656.4656.2	2	2.756	0.24	1	515.7	57.692307	K.VWPHKDYPLIPVGK.L
CATB_MOUSE	MK_SCX_13.10550.10550.2	2	3.072	0.425	1	304.8	41.17647	K.NGPVEGAFTVFSDFLTYK.S
CATB_MOUSE	MK_SCX_14.5117.5117.2	2	3.456	0.39	1	537.4	62.5	R.EQWSNCPTIGQIR.D
CATB_MOUSE	MK_SCX_17.7827.7827.2	2	2.049	0.297	1	304.3	47.368423	R.DQGSCGSCWAFGAVEAISDR.T
CATD_MOUSE	MK_SCX_13.6939.6939.2	2	3.523	0.301	1	633.6	55.88235	K.AIGAVPLIQGEYMIPCEK.V
CATD_MOUSE	MK_SCX_16.4006.4006.2	2	4.775	0.549	1	659.1	58.823532	R.DPEGQPGGELM*LGGTDSK.Y
CATD_MOUSE	MK_SCX_16.4401.4401.2	2	4.892	0.649	1	1543.1	82.35294	R.DPEGQPGGELMLGGTDSK.Y
CATD_MOUSE	MK_SCX_2201.10230.10230.3	3	5.936	0.491	1	1072	37.037037	K.FDGILGM*GYPHISVNNVLPVFDNLMQQK.L
CATD_MOUSE	MK_SCX_2201.10704.10704.3	3	5.17	0.422	1	824.2	32.407406	K.FDGILGMGYPHISVNNVLPVFDNLMQQK.L
CATD_MOUSE	MK_SCX_2201.9215.9215.3	3	6.179	0.517	1	930.6	36.11111	K.FDGILGM*GYPHISVNNVLPVFDNLM*QQK.L
CATD_MOUSE	MK_SCX_2201.9822.9822.3	3	5.868	0.498	1	754.9	32.407406	K.FDGILGMGYPHISVNNVLPVFDNLM*QQK.L
CATD_MOUSE	MK_SCX_24.9192.9192.3	3	3.867	0.404	1	603.4	28.846153	K.NIFSFYLNRDPEGQPGGELMLGGTDSK.Y

CATD_MOUSE	MK_SCX_25.7033.7033.3	3	3.175	0.481	1	753.1	39.473686	R.TMTEVGGSVEDLILKGPITK.Y
CATD_MOUSE	MK_SCX_29.5594.5594.2	2	5.005	0.402	1	1404	70.588234	K.QIFGEATKQPGIVFVAAK.F
CATD_MOUSE	MK_SCX_29.5668.5668.3	3	3.928	0.521	1	1258.5	47.058823	K.QIFGEATKQPGIVFVAAK.F
CATD_MOUSE	MK_SCX_42.6649.6649.3	3	3.111	0.321	1	354.3	30.000002	R.RTMTEVGGSVEDLILKGPITK.Y
CATD_MOUSE	MK_SCX_51.4335.4335.2	2	3.779	0.324	1	1647.9	81.818184	K.NYELHPDKYILK.V
CATH_MOUSE	MK_SCX_14.7478.7478.2	2	2.291	0.126	1	783	56.25	K.NSWGSQWGENGYFLIER.G
CATH_MOUSE	MK_SCX_20_1.4354.4354.2	2	3.336	0.478	1	1143.4	70.83333	K.GIM*EEDSYPYIGK.D
CATH_MOUSE	MK_SCX_20_1.8139.8139.2	2	4.864	0.424	1	939.4	67.85714	K.GGLPSQAFEYILYNK.G
CATH_MOUSE	MK_SCX_30.6450.6450.3	3	3.26	0.471	1	539.2	32.142857	K.MLSLAEQQLVDCAQAFNNHGCK.G
CATL_MOUSE	MK_SCX_17.3811.3811.2	2	3.032	0.309	1	482.8	46.666668	K.ENGGLDSEESYPYEAK.D
CATL_MOUSE	MK_SCX_19.9593.9593.3	3	3.085	0.257	1	351.3	20.454546	K.LISLSEQNLVDCSHAQGNQGCNGGLMDFAFQYIK.E
CATL_MOUSE	MK_SCX_24.15244.15244.2	2	4.227	0.456	1	1509.5	76.92308	K.NSWGSEWGMEGYIK.I
CAZA1_MOUSE	MK_SCX_18.3546.3546.2	2	3.759	0.474	1	770	71.42857	K.EASDPQPEDVDGGLK.S
CAZA1_MOUSE	MK_SCX_19.5994.5994.2	2	3.864	0.357	1	917.3	82.14286	K.FTITPPSAQVVGVLK.I
CAZA1_MOUSE	MK_SCX_23.4866.4866.2	2	3.291	0.249	1	1198.9	88.88889	R.LLLNNDNLLR.E
CAZA1_MOUSE	MK_SCX_30.5349.5349.3	3	3.501	0.303	1	720.1	33.333336	R.EGAAHAFAQYNMDQFTPVK.I
CAZA1_MOUSE	MK_SCX_31.6317.6317.2	2	3.563	0.549	1	446	55.88235	K.FITHAPPGEFNEVFNDVR.L
CAZA1_MOUSE	MK_SCX_31.6394.6394.3	3	5.841	0.375	1	1395.4	45.588234	K.FITHAPPGEFNEVFNDVR.L
CAZA2_MOUSE	MK_SCX_16.6385.6385.2	2	5.351	0.697	1	830.6	44	K.IVEAAENEYQTAISENYQTMSDTTFK.A
CAZA2_MOUSE	MK_SCX_16.6398.6398.3	3	6.262	0.645	1	1033	32	K.IVEAAENEYQTAISENYQTMSDTTFK.A
CAZA2_MOUSE	MK_SCX_19.5574.5574.2	2	2.987	0.399	1	412.6	53.571426	K.FTVTPSTTQVVGILK.I
CAZA2_MOUSE	MK_SCX_28.5199.5199.3	3	3.846	0.506	1	951.3	41.17647	K.EATDPRPYEAENAIESWR.T
CAZA2_MOUSE	MK_SCX_30.5892.5892.2	2	3.76	0.483	1	551.1	50	R.EGAAHAFAQYNLDQFTPVK.I
CAZA2_MOUSE	MK_SCX_30.7050.7050.3	3	4.55	0.406	1	1430.6	44.11765	K.FIIHAPPGEFNEVFNDVR.L
CAZA2_MOUSE	MK_SCX_30.7136.7136.2	2	3.596	0.567	1	486.3	58.823532	K.FIIHAPPGEFNEVFNDVR.L
CB004_MOUSE	MK_SCX_25.8528.8528.3	3	4.713	0.605	1	537.5	22.5	R.EASHAGSWYTASGPQLNAQLEGWLSQVQSTK.R
CBP_MOUSE	MK_SCX_26.3133.3133.3	3	3.257	0.262	1	537.2	39.473686	K.TEVQTDDAEPEPTESKGEPR.S
CBP_MOUSE	MK_SCX_27.2758.2758.3	3	4.499	0.515	1	1087.8	39.583336	K.VEAKEEEENSSNDTASQSTSPSQPR.K
CBPM_MOUSE	MK_SCX_25.6845.6845.2	2	4.819	0.672	1	1249	61.11111	R.SLTPDDDVFQHLAYTYASR.N
CBPM_MOUSE	MK_SCX_50.3922.3922.3	3	3.348	0.378	1	1087.6	50	R.KDPEITHLIDSTR.I
CBPM_MOUSE	MK_SCX_55.3524.3524.3	3	3.913	0.428	1	1097	50	R.YHHQEGMEAFLK.S
CBX1_MOUSE	MK_SCX_18.14487.14487.2	2	4.156	0.3	1	928.8	63.333332	R.IIGATDSSGELMFLMK.W
CBX1_MOUSE	MK_SCX_38.3595.3595.3	3	3.246	0.237	1	785.3	50	K.WKNSDEADLVPAK.E
CBX1_MOUSE	MK_SCX_49.3976.3976.3	3	4.12	0.451	1	1219.7	47.058823	K.WKNSDEADLVPAKEANVK.C
CBX3_MOUSE	MK_SCX_18.4313.4313.2	2	4.226	0.394	1	922.3	83.33333	K.VEEAEPEEFVVEK.V
CBX3_MOUSE	MK_SCX_31.3963.3963.3	3	4.131	0.411	1	663.4	46.153847	K.KVEEAEPEEFVVEK.V
CBX3_MOUSE	MK_SCX_31.3967.3967.2	2	5.614	0.454	1	1681.1	84.61539	K.KVEEAEPEEFVVEK.V
CBX3_MOUSE	MK_SCX_34.4192.4192.3	3	4.874	0.398	1	2715.9	66.66667	K.WKDSDEADLVLAK.E
CBX3_MOUSE	MK_SCX_34.4216.4216.2	2	3.673	0.535	1	1585.3	87.5	K.WKDSDEADLVLAK.E
CBX3_MOUSE	MK_SCX_49.3756.3756.3	3	4.616	0.415	1	764.1	45	K.SKKVEEAEPEEFVVEK.V
CBX5_MOUSE	MK_SCX_18.9193.9193.2	2	3.166	0.245	1	504.7	53.333336	K.IIGATDSCGDLMFLMK.W
CBX5_MOUSE	MK_SCX_33.4431.4431.3	3	3.115	0.128	1	1289.8	52.083332	K.WKDTDEADLVLAK.E
CC47_MOUSE	MK_SCX_15.10022.10022.2	2	4.687	0.555	1	1911.3	56.521736	K.YGLPDSLAILSEMGEVTEGMMDTK.M
CC47_MOUSE	MK_SCX_15.10106.10106.2	2	6.374	0.362	1	1233.5	82.35294	K.DM*ESLLPLMNMVIYSIDK.A
CC47_MOUSE	MK_SCX_15.10826.10826.2	2	5.33	0.593	1	985.4	76.47059	K.DMESLLPLMNMVIYSIDK.A
CC47_MOUSE	MK_SCX_15.9139.9139.2	2	4.624	0.139	1	549.6	58.823532	K.DM*ESLLPLMNM*VIYSIDK.A
CC47_MOUSE	MK_SCX_19.6638.6638.2	2	2.866	0.348	1	446.7	50	R.TLLFTFNVPGSGNTYPK.D
CC47_MOUSE	MK_SCX_29.4292.4292.2	2	3.099	0.389	1	515.1	57.14286	K.IMQEEGQPLKLPDTK.R
CC47_MOUSE	MK_SCX_43.4763.4763.2	2	3.093	0.174	1	1242.1	88.88889	K.RQDLLNVLAR.M
CC47_MOUSE	MK_SCX_45.4115.4115.2	2	4.196	0.371	1	1457	70	K.IMQEEGQPLKLPDTKR.T
CCAR1_MOUSE	MK_SCX_19.4135.4135.2	2	4.192	0.374	1	687.7	62.5	R.IQTLPNQNQSQTQPLLK.T

CCD16_MOUSE	MK_SCX_18.3810.3810.2	2	5.602	0.616	1	1056.4	64.28571	K.ASAGPQVQPSTSASSANLDAAR.A
CCD44_MOUSE	MK_SCX_42.6965.6965.3	3	4.633	0.473	1	1208.8	40	K.KLDSLGLCPVSCSMEFIPHSK.V
CCD44_MOUSE	MK_SCX_54.6530.6530.3	3	3.556	0.419	1	847.3	34.523808	R.KKLDSLGLCPVSCSMEFIPHSK.V
CCD46_MOUSE	MK_SCX_14.6422.6422.2	2	2.094	0.25	1	319.8	43.75	K.RDAQVIADM*EAQVHKLR.E
CCD46_MOUSE	MK_SCX_17.3275.3275.3	3	3.67	0.197	1	339.9	31.25	K.FKGLM*PASLRQELEDTISSLK.S
CCD58_MOUSE	MK_SCX_31.4580.4580.2	2	5.453	0.58	1	1734.1	81.25	R.IVHELNTTVPTASFAGK.I
CCD58_MOUSE	MK_SCX_8.4054.4054.2	2	2.029	0.176	1	714.1	70	K.NCIAQTSAVVK.S
CCHL_MOUSE	MK_SCX_13.4194.4194.2	2	3.767	0.571	1	624.1	83.33333	R.AYDYVECPVTGAR.A
CCS_MOUSE	MK_SCX_38.3234.3234.3	3	4.411	0.495	1	901.5	31.52174	R.DCNSCGDHFNPDGASHGGPQDTDR.H
CD19_MOUSE	MK_SCX_34.6420.6420.3	3	4.236	0.132	1	501.1	32.142857	-.MPSPLPVSFLLFLTLVGGRPQK.S
CD2AP_MOUSE	MK_SCX_27.3323.3323.3	3	3.34	0.547	1	433.7	39.705883	K.DASYSSKPSLSTPSSASK.V
CD2AP_MOUSE	MK_SCX_27.3359.3359.2	2	4.813	0.615	1	831.2	64.70589	K.DASYSSKPSLSTPSSASK.V
CD2AP_MOUSE	MK_SCX_29.4407.4407.3	3	3.51	0.498	1	545.9	38.235294	R.ISTYGLPAGGIQPHPQTK.A
CD2AP_MOUSE	MK_SCX_30.4301.4301.2	2	3.691	0.449	1	388.2	50	R.ISTYGLPAGGIQPHPQTK.A
CD2AP_MOUSE	MK_SCX_34.5899.5899.2	2	3.266	0.274	1	740.8	65.38461	R.GIGFGDIFKEGSVK.L
CD2AP_MOUSE	MK_SCX_49.3022.3022.3	3	4.241	0.526	1	1006.2	43.055553	K.KDASYSSKPSLSTPSSASK.V
CD2L1_MOUSE	MK_SCX_19.6563.6563.2	2	3.285	0.265	1	950.6	71.42857	R.FGALLSDQGFDLMNK.F
CD34_MOUSE	MK_SCX_15.4348.4348.3	3	3.786	0.458	1	1112.5	29.464287	R.LGEDPYYTENGGGQGYSSGPGASPETQGK.A
CD34_MOUSE	MK_SCX_15.4355.4355.2	2	4.02	0.531	1	821.3	39.285713	R.LGEDPYYTENGGGQGYSSGPGASPETQGK.A
CD36_MOUSE	MK_SCX_17.7875.7875.2	2	4.501	0.463	1	1414.3	78.125	R.TYLDVEPITGFTLQFAK.R
CD47_MOUSE	MK_SCX_18.6786.6786.2	2	2.528	0.121	1	1537.7	84.61539	K.ISVSDLINGIASLK.M
CD47_MOUSE	MK_SCX_21.4452.4452.2	2	2.815	0.311	1	530	66.66667	R.NVEAQSTEEMFVK.W
CDC37_MOUSE	MK_SCX_16.7715.7715.2	2	4.554	0.588	1	1090.3	55	R.LGPGGLDPVEVYESLPEELQK.C
CDC37_MOUSE	MK_SCX_17.5142.5142.2	2	5.898	0.615	1	1305.9	71.875	K.EGEEAGPGDPLLEAVPK.A
CDC37_MOUSE	MK_SCX_25.4912.4912.3	3	4.606	0.444	1	2051.1	46.42857	K.SGEAKEGEEAGPGDPLLEAVPK.A
CDC37_MOUSE	MK_SCX_25.4931.4931.2	2	5.798	0.672	1	1757.3	69.047615	K.SGEAKEGEEAGPGDPLLEAVPK.A
CDC37_MOUSE	MK_SCX_45.2865.2865.3	3	4.856	0.598	1	1564.7	44.11765	K.SMVNTKPEKAEEDSEEAR.E
CDC37_MOUSE	MK_SCX_50.4555.4555.3	3	5.054	0.581	1	2297.4	52.941177	R.KLKELEVAESDGQVELER.L
CDC37_MOUSE	MK_SCX_51.3275.3275.3	3	3.847	0.37	1	1726.1	54.166668	R.MEQFQKEKEELDR.G
CDC37_MOUSE	MK_SCX_53.2894.2894.3	3	4.437	0.401	1	887.6	36.25	K.SMVNTKPEKAEEDSEEAREQK.H
CDC5L_MOUSE	MK_SCX_17.6516.6516.2	2	3.78	0.446	1	431.6	41.666664	K.ESDLPSAILQTSGVSEFTK.K
CDC5L_MOUSE	MK_SCX_17.6664.6664.2	2	4.323	0.55	1	571.4	70.588234	K.LVLPAPQISDAELQEVVK.V
CDC5L_MOUSE	MK_SCX_27.7191.7191.3	3	4.362	0.467	1	925.8	36.842106	R.SKLVLPAPQISDAELQEVVK.V
CDC5L_MOUSE	MK_SCX_47.6220.6220.3	3	4.704	0.449	1	772.3	35	R.KKESDLPSAILQTSGVSEFTK.K
CDD_MOUSE	MK_SCX_17.6183.6183.2	2	4.496	0.512	1	551.6	59.375	R.TVQELLPASFGPEDLQK.I
CDD_MOUSE	MK_SCX_18.7782.7782.2	2	3.63	0.327	1	739.5	52.63158	R.AIAISSDLQEEFISPCGACR.Q
CDD_MOUSE	MK_SCX_21.5282.5282.2	2	4.483	0.491	1	2208.6	87.5	R.FPVGAALLTGDGR.I
CDD_MOUSE	MK_SCX_26.6360.6360.3	3	3.261	0.424	1	440.7	28.947369	R.EFGTDWAVYM*TKPDGTFVVR.T
CDD_MOUSE	MK_SCX_26.7391.7391.3	3	3.578	0.457	1	838.3	36.842106	R.EFGTDWAVYMTKPDGTFVVR.T
CDD_MOUSE	MK_SCX_26.7423.7423.2	2	4.673	0.509	1	887	55.263157	R.EFGTDWAVYMTKPDGTFVVR.T
CDK5_MOUSE	MK_SCX_24.7034.7034.3	3	4.041	0.387	1	1529.4	38.095238	K.LPDYKPYPMYPATTSLVNVVPK.L
CDN1B_MOUSE	MK_SCX_18.3417.3417.2	2	5.168	0.625	1	1840.7	73.68421	R.TEENVSDGSPNAGTVEQTPK.K
CDN1B_MOUSE	MK_SCX_21.2772.2772.2	2	3.7	0.644	1	1623.9	79.16667	K.VLAQESQDVSGSR.Q
CDN1B_MOUSE	MK_SCX_30.3203.3203.3	3	6.662	0.585	1	2170.4	46.590908	R.ANRTEENVSDGSPNAGTVEQTPK.K
CELR2_MOUSE	MK_SCX_16.8904.8904.2	2	2.512	0.198	1	336.5	43.18182	K.SLDLTGPLLLGGVPDLPESFPVR.M
CELR2_MOUSE	MK_SCX_44.6833.6833.2	2	2.525	0.27	1	556.9	57.692307	R.TRQADGVLLQAVTR.G
CENA2_MOUSE	MK_SCX_17.8309.8309.2	2	3.991	0.589	1	1444.8	75	K.LAFPDLPESELVPLITR.N
CES3_MOUSE	MK_SCX_17.7976.7976.2	2	3.899	0.484	1	555	62.5	K.ESYPFLPTVIDGVVLPK.A
CES3_MOUSE	MK_SCX_19.4447.4447.2	2	2.183	0.4	1	904.5	65	K.MVMKFWANFAR.N
CES3_MOUSE	MK_SCX_20_1.4096.4096.2	2	2.976	0.304	1	721.3	68.181816	K.ISENM*IPVVAEK.Y
CES3_MOUSE	MK_SCX_20_1.4853.4853.2	2	4.044	0.382	1	1210.6	86.36364	K.ISENMIPVVAEK.Y

CES3_MOUSE	MK_SCX_21.9515.9515.3	3	4.408	0.478	1	984.5	33.333336	K.LDLLGNPKESYPFLPTVIDGVVLPK.A
CES3_MOUSE	MK_SCX_2201.10763.10763.3	3	5.828	0.605	1	724.7	33.333336	K.YVNLEGFTQPVAVFLGVPFAKPPLGSLR.F
CES3_MOUSE	MK_SCX_26.8253.8253.2	2	5.87	0.615	1	3244.9	73.68421	K.AVIGDHGDEIFSVFGSPFLK.D
CES3_MOUSE	MK_SCX_33.7235.7235.3	3	4.125	0.286	1	806.4	31.52174	R.DAGASTYMYEFEYRPSFVSAMRPK.A
CES3_MOUSE	MK_SCX_40.4988.4988.2	2	3.274	0.359	1	531.3	66.66667	R.GNWGHLDQVAALR.W
CES3_MOUSE	MK_SCX_43.12056.12056.3	3	4.461	0.42	1	558.9	29.545454	K.KKDLFQDLMADVVFGVPSVIVSR.S
CES3_MOUSE	MK_SCX_53.5317.5317.3	3	3.254	0.358	1	1487.2	54.166668	R.LKDKEVSFWAELR.A
CETN2_MOUSE	MK_SCX_46.3111.3111.3	3	3.994	0.29	1	1362.5	50	R.M*SPKPELTEDQKQEIR.E
CETN2_MOUSE	MK_SCX_46.3307.3307.3	3	3.549	0.395	1	802	41.666664	R.MSPKPELTEDQKQEIR.E
CF055_MOUSE	MK_SCX_13.6302.6302.2	2	2.045	0.147	1	605.4	53.571426	-.MAALAPLPPLPAQFK.S
CF055_MOUSE	MK_SCX_18.5616.5616.2	2	3.99	0.604	1	566.8	60.526318	K.YAGSALQYEDVGTAVQNLQK.A
CF057_MOUSE	MK_SCX_29.5767.5767.3	3	5.116	0.565	1	734	28	R.FDSLEDSPEEREPLQKFPDDVNPVTK.E
CF064_MOUSE	MK_SCX_19.3417.3417.2	2	4.111	0.539	1	536	52.63158	R.ASLVAQPSTSSQSVQTSGAK.A
CF066_MOUSE	MK_SCX_23.5068.5068.2	2	4.594	0.519	1	1674.4	73.333336	R.DVYVDSKDPVPALPVK.V
CF149_MOUSE	MK_SCX_33.4667.4667.2	2	4.601	0.397	1	1021.9	76.92308	K.NVKDPVEIQALVNK.A
CF149_MOUSE	MK_SCX_46.4433.4433.3	3	4.168	0.489	1	802	45.3125	R.ENKNVKDPVEIQALVNK.A
CF152_MOUSE	MK_SCX_12.9993.9993.2	2	3.095	0.333	1	388.1	61.538464	K.ELDIKNIYANRLPK.S
CF152_MOUSE	MK_SCX_41.4581.4581.2	2	2.199	0.133	1	333.1	66.66667	K.ELQRLHHKLK.E
CF203_MOUSE	MK_SCX_19.3411.3411.2	2	3.07	0.358	1	448.5	53.571426	R.SEQEEELESEPGVAK.D
CF203_MOUSE	MK_SCX_24.5641.5641.3	3	5.363	0.604	1	1351.2	40.476192	K.VGDTLDLITGENKETGTEVVMR.I
CF203_MOUSE	MK_SCX_27.3935.3935.3	3	3.663	0.367	1	746.8	39.705883	R.SEQEEELESEPGVAKDYK.D
CF203_MOUSE	MK_SCX_53.3896.3896.3	3	3.349	0.352	1	521.2	41.666664	R.NKVEDAFYKGELR.L
CFAB_MOUSE	MK_SCX_2201.5131.5131.2	2	2.926	0.363	1	1097.7	85	R.YGLLTYATVPK.V
CFAB_MOUSE	MK_SCX_26.6651.6651.2	2	2.685	0.338	1	619.1	72.72727	R.DLEIEEVLFHPK.Y
CFAH_MOUSE	MK_SCX_29.6387.6387.2	2	3.95	0.554	1	1472.4	67.64706	R.LYYEESLRPNFPVSIGNK.Y
CG010_MOUSE	MK_SCX_17.6730.6730.2	2	5.798	0.533	1	2307.4	68.42105	R.VLAGPFATM*NLGDLGAEVIK.V
CG010_MOUSE	MK_SCX_17.8145.8145.2	2	6.173	0.561	1	1814	71.05263	R.VLAGPFATMNLGDLGAEVIK.V
CG010_MOUSE	MK_SCX_19.7746.7746.2	2	4.311	0.536	1	812.1	58.333332	R.SWGPPFVNTESTYFLSVNR.N
CGL_MOUSE	MK_SCX_17.7369.7369.2	2	3.023	0.414	1	375.1	50	R.LSVGLEDEQDLLEDLDR.A
CGL_MOUSE	MK_SCX_23.8515.8515.3	3	6.622	0.59	1	1882.6	43.269234	R.AVVLPISLATTFKQDFPGQSSGFEYSR.S
CGRE1_MOUSE	MK_SCX_16.6028.6028.2	2	5.805	0.625	1	1466.2	59.523808	R.LDPEVQQQLTPNPFQPGPEQLR.H
CGRE1_MOUSE	MK_SCX_16.6073.6073.3	3	3.443	0.318	1	483.3	29.761904	R.LDPEVQQQLTPNPFQPGPEQLR.H
CGRE1_MOUSE	MK_SCX_24.4294.4294.3	3	5.257	0.599	1	1756.9	47.826088	K.VDTLSPEEEARGQAESEGDVPGPR.E
CGRE1_MOUSE	MK_SCX_27.3148.3148.3	3	3.038	0.257	1	905.7	42.1875	R.EGAEEQVEIKDNEGEAK.E
CGRE1_MOUSE	MK_SCX_50.3861.3861.3	3	6.662	0.575	1	2151.3	35.185184	K.RASLEPEQEAGHQTEGKVDTLSPEEEAR.G
CH10_MOUSE	MK_SCX_21.4090.4090.3	3	4.327	0.431	1	1373.6	51.923077	K.VLQATVVAVGSGGK.G
CH10_MOUSE	MK_SCX_21.4098.4098.2	2	5.646	0.664	1	2601.5	88.46153	K.VLQATVVAVGSGGK.G
CH10_MOUSE	MK_SCX_31.16058.16058.2	2	4.171	0.557	1	1291	81.818184	K.VVLDDKDYFLFR.D
CH10_MOUSE	MK_SCX_31.3932.3932.3	3	3.222	0.213	1	946.4	51.666664	R.SAAETVTKGGIM*LPEK.S
CH10_MOUSE	MK_SCX_31.4059.4059.2	2	4.429	0.43	1	2010.4	76.666664	R.SAAETVTKGGIMLPEK.S
CH10_MOUSE	MK_SCX_31.4074.4074.3	3	4.89	0.506	1	1967.4	61.666668	R.SAAETVTKGGIMLPEK.S
CH10_MOUSE	MK_SCX_31.6485.6485.1	1	2.182	0.371	1	407.2	54.545456	K.VVLDDKDYFLFR.D
CH10_MOUSE	MK_SCX_31.7562.7562.3	3	3.675	0.609	1	581.5	56.81818	K.VVLDDKDYFLFR.D
CH10_MOUSE	MK_SCX_32.3831.3831.2	2	4.388	0.422	1	1416.4	80.769226	K.VGDKVLLPEYGGTK.V
CH10_MOUSE	MK_SCX_33.3568.3568.2	2	4.157	0.538	1	1561.8	73.333336	K.VLQATVVAVGSGGKGK.S
CH10_MOUSE	MK_SCX_34.16362.16362.3	3	3.021	0.344	1	353.9	30.952381	K.VLLPEYGGTKVVLDDKDYFLFR.D
CH10_MOUSE	MK_SCX_34.5919.5919.3	3	4.193	0.508	1	576.5	32.608696	K.SGEIEPVSVKVGDKVLLPEYGGTK.V
CH10_MOUSE	MK_SCX_43.3743.3743.3	3	4.534	0.476	1	1169.6	39.473686	R.SAAETVTKGGIM*LPEKSQGK.V
CH10_MOUSE	MK_SCX_43.3864.3864.3	3	4.107	0.325	1	872.3	38.157894	R.SAAETVTKGGIMLPEKSQGK.V
CH10_MOUSE	MK_SCX_43.3914.3914.2	2	4.713	0.557	1	1090.9	55.263157	R.SAAETVTKGGIMLPEKSQGK.V
CH10_MOUSE	MK_SCX_47.5032.5032.2	2	2.834	0.219	1	529.6	78.57143	R.KFLPLFDR.V

CH10_MOUSE	MK_SCX_49.3426.3426.3	3	3.316	0.473	1	639.5	40	K.GKSGEIEPVSVKVGDK.V
CH60_MOUSE	MK_SCX_13.6917.6917.2	2	5.55	0.441	1	2377.6	81.25	R.AAVEEGIVLGGGCALLR.C
CH60_MOUSE	MK_SCX_13.6955.6955.2	2	4.607	0.514	1	1711	83.33333	K.CEFQDAYVLLSEK.K
CH60_MOUSE	MK_SCX_14.4730.4730.1	1	2.285	0.202	1	482.8	63.636364	K.NAGVEGSLIVEK.I
CH60_MOUSE	MK_SCX_15.15247.15247.3	3	4.557	0.461	1	962.6	33.333336	R.TALLDAAGVASLLTTAEAVVTEIPK.E
CH60_MOUSE	MK_SCX_15.4615.4615.3	3	4.421	0.435	1	441.7	28.125	K.LVQDVANNTNEEAGDGTTTATVLAR.S
CH60_MOUSE	MK_SCX_16.12117.12117.3	3	4.371	0.42	1	2269.6	45	R.ALM*LQGVDLLADAVAVTMGPK.G
CH60_MOUSE	MK_SCX_16.12384.12384.2	2	4.92	0.243	1	1268.1	54.545456	K.ILQSSSEVGYDAM*LGDFVNMVEK.G
CH60_MOUSE	MK_SCX_16.12917.12917.3	3	5.295	0.555	1	2101.7	45	R.ALMLQGVDLLADAVAVTMGPK.G
CH60_MOUSE	MK_SCX_16.13139.13139.2	2	6.53	0.647	1	2405.2	70	R.ALMLQGVDLLADAVAVTMGPK.G
CH60_MOUSE	MK_SCX_16.15181.15181.3	3	3.318	0.187	1	576.8	31.818182	K.ILQSSSEVGYDAM*LGDFVNMVEK.G
CH60_MOUSE	MK_SCX_16.5127.5127.2	2	6.658	0.66	1	2533.1	60.416668	K.LVQDVANNTNEEAGDGTTTATVLAR.S
CH60_MOUSE	MK_SCX_16.6581.6581.2	2	4.537	0.658	1	1008.6	52.272724	K.ILQSSSEVGYDAM*LGDFVNM*VEK.G
CH60_MOUSE	MK_SCX_16.6614.6614.3	3	5.414	0.472	1	2247.7	40.909092	K.ILQSSSEVGYDAM*LGDFVNM*VEK.G
CH60_MOUSE	MK_SCX_16.7635.7635.3	3	5.488	0.417	1	2905.6	46.590908	K.ILQSSSEVGYDAMLGDFVNM*VEK.G
CH60_MOUSE	MK_SCX_16.7789.7789.2	2	5.156	0.369	1	1620.5	56.81818	K.ILQSSSEVGYDAMLGDFVNM*VEK.G
CH60_MOUSE	MK_SCX_16.8251.8251.2	2	5.502	0.633	1	1461	57.5	R.ALM*LQGVDLLADAVAVTM*GPK.G
CH60_MOUSE	MK_SCX_16.8312.8312.3	3	3.968	0.523	1	825.5	35	R.ALM*LQGVDLLADAVAVTM*GPK.G
CH60_MOUSE	MK_SCX_16.8613.8613.3	3	5.555	0.513	1	1333	37.5	K.ILQSSSEVGYDAMLGDFVNMVEK.G
CH60_MOUSE	MK_SCX_16.8686.8686.2	2	6.222	0.707	1	1970.5	61.363636	K.ILQSSSEVGYDAMLGDFVNMVEK.G
CH60_MOUSE	MK_SCX_16.8860.8860.2	2	5.725	0.676	1	1841.9	62.5	R.ALMLQGVDLLADAVAVTM*GPK.G
CH60_MOUSE	MK_SCX_16.8887.8887.3	3	4.824	0.549	1	1201.7	38.75	R.ALMLQGVDLLADAVAVTM*GPK.G
CH60_MOUSE	MK_SCX_16.9245.9245.2	2	5.493	0.335	1	2076.4	62.5	R.ALM*LQGVDLLADAVAVTMGPK.G
CH60_MOUSE	MK_SCX_17.15724.15724.2	2	3.863	0.374	1	432	46.875	R.IQEITEQLDITTSEYEK.E
CH60_MOUSE	MK_SCX_18.12367.12367.3	3	5.963	0.617	1	1569.4	34.482758	K.DMAIATGGAVFGEEGLNLNLEDVQAHDLGK.V
CH60_MOUSE	MK_SCX_18.13456.13456.3	3	3.734	0.466	1	660.3	26.724138	K.DM*AIATGGAVFGEEGLNLNLEDVQAHDLGK.V
CH60_MOUSE	MK_SCX_18.4849.4849.2	2	4.261	0.483	1	901	70	R.CIPALDSLKPANEDQK.I
CH60_MOUSE	MK_SCX_19.5311.5311.2	2	3.37	0.473	1	1777.2	75	K.TLNDELEIIEGM*K.F
CH60_MOUSE	MK_SCX_19.6480.6480.2	2	5.159	0.58	1	2089.9	83.33333	K.TLNDELEIIEGMK.F
CH60_MOUSE	MK_SCX_21.3066.3066.2	2	4.007	0.522	1	688.9	77.27273	K.VGGTSDVEVNEK.K
CH60_MOUSE	MK_SCX_2201.11479.11479.3	3	4.544	0.442	1	877.2	30.555555	R.TALLDAAGVASLLTTAEAVVTEIPKEEK.D
CH60_MOUSE	MK_SCX_2201.2329.2329.2	2	3.633	0.454	1	1037.7	93.75	R.VTDALNATR.A
CH60_MOUSE	MK_SCX_2201.2725.2725.2	2	2.865	0.366	1	896.8	85.71429	K.VGEVIVTK.D
CH60_MOUSE	MK_SCX_2201.3600.3600.2	2	2.536	0.263	1	856.1	93.75	K.LSDGVAVLK.V
CH60_MOUSE	MK_SCX_2201.3942.3942.2	2	3.821	0.447	1	1312.5	86.36364	K.NAGVEGSLIVEK.I
CH60_MOUSE	MK_SCX_2201.3962.3962.2	2	3.049	0.213	1	1583.7	93.75	K.VGLQVVAVK.A
CH60_MOUSE	MK_SCX_2201.4238.4238.2	2	3.99	0.376	1	1374	81.818184	R.TVIIEQSWGSPK.V
CH60_MOUSE	MK_SCX_2201.5006.5006.2	2	4.105	0.606	1	1367.7	86.36364	R.GYISPYFINTSK.G
CH60_MOUSE	MK_SCX_25.5379.5379.3	3	3.238	0.406	1	325.9	36.11111	R.IQEITEQLDITTSEYEKEK.L
CH60_MOUSE	MK_SCX_26.6545.6545.3	3	3.984	0.429	1	1376.9	45	K.DGKTLNDELEIIEGMK.F
CH60_MOUSE	MK_SCX_26.6554.6554.2	2	5.107	0.522	1	1920.8	76.666664	K.DGKTLNDELEIIEGMK.F
CH60_MOUSE	MK_SCX_26.8120.8120.3	3	4.164	0.479	1	482	21.969696	K.QSKPVTTPEEIAQVATISANGDKDIGNIISDAMK.K
CH60_MOUSE	MK_SCX_27.4704.4704.2	2	3.889	0.512	1	802.9	64.28571	K.VGEVIVTKDDAM*LLK.G
CH60_MOUSE	MK_SCX_27.4718.4718.3	3	4.042	0.565	1	986.3	53.571426	K.VGEVIVTKDDAM*LLK.G
CH60_MOUSE	MK_SCX_27.5220.5220.2	2	4.825	0.531	1	1942.8	82.14286	K.VGEVIVTKDDAMLLK.G
CH60_MOUSE	MK_SCX_27.5387.5387.3	3	4.42	0.538	1	724.9	50	K.VGEVIVTKDDAMLLK.G
CH60_MOUSE	MK_SCX_28.12269.12269.3	3	3.307	0.521	1	323.7	41.666664	K.TLNDELEIIEGMKFDR.G
CH60_MOUSE	MK_SCX_28.6139.6139.2	2	5.24	0.568	1	892.1	64.70589	K.ISSVQSIVPALEIANAHR.K
CH60_MOUSE	MK_SCX_28.6157.6157.3	3	4.099	0.597	1	1398.6	44.11765	K.ISSVQSIVPALEIANAHR.K
CH60_MOUSE	MK_SCX_28.6418.6418.2	2	4.159	0.522	1	932.5	66.66667	K.TLNDELEIIEGM*KFDR.G
CH60_MOUSE	MK_SCX_28.7455.7455.2	2	4.029	0.436	1	1623.9	70	K.TLNDELEIIEGMKFDR.G

CH60_MOUSE	MK_SCX_30.5179.5179.3	3	4.297	0.634	1	2293.9	54.6875	K.VGLQVVAVKAPGFGDNR.K
CH60_MOUSE	MK_SCX_30.5215.5215.2	2	5.35	0.59	1	1905.6	71.875	K.VGLQVVAVKAPGFGDNR.K
CH60_MOUSE	MK_SCX_34.2149.2149.3	3	3.432	0.361	1	383.8	45.833336	K.VGGTSDVEVNEKK.D
CH60_MOUSE	MK_SCX_34.2158.2158.2	2	3.279	0.417	1	1627.3	79.16667	K.VGGTSDVEVNEKK.D
CH60_MOUSE	MK_SCX_34.5408.5408.3	3	3.533	0.288	1	829.8	42.857143	K.FDRGYISPYFINTSK.G
CH60_MOUSE	MK_SCX_34.5448.5448.2	2	5.043	0.475	1	1962	78.57143	K.FDRGYISPYFINTSK.G
CH60_MOUSE	MK_SCX_35.4195.4195.2	2	2.848	0.293	1	829	88.88889	K.GIIDPTKVVR.T
CH60_MOUSE	MK_SCX_35.4812.4812.2	2	4.282	0.45	1	1485.2	78.57143	R.GYISPYFINTSKGQK.C
CH60_MOUSE	MK_SCX_36.4319.4319.2	2	2.677	0.301	1	564.7	80	R.ALKIPAMTIAK.N
CH60_MOUSE	MK_SCX_39.3852.3852.2	2	2.981	0.159	1	1136.2	92.85714	K.IGIEIIKR.A
CH60_MOUSE	MK_SCX_43.5583.5583.3	3	5.846	0.493	1	1867	44.736843	K.RIQEITEQLDITTSEYEKEK.L
CH60_MOUSE	MK_SCX_45.5119.5119.3	3	3.811	0.576	1	1584.8	47.058823	K.VGLQVVAVKAPGFGDNRK.N
CH60_MOUSE	MK_SCX_46.5871.5871.2	2	6.827	0.691	1	1533.5	75	K.KISSVQSIVPALEIANAHR.K
CH60_MOUSE	MK_SCX_46.5891.5891.3	3	6.074	0.616	1	2454.3	52.77778	K.KISSVQSIVPALEIANAHR.K
CHCH1_MOUSE	MK_SCX_54.4457.4457.3	3	4.311	0.5	1	696	35.526314	R.FANPGKPILKPNKPLILANR.V
CHCH2_MOUSE	MK_SCX_31.3774.3774.2	2	6.206	0.671	1	1712.7	63.04348	R.RAPAAQPPAAAAPSAVGSPAAAPR.Q
CHCH2_MOUSE	MK_SCX_31.3780.3780.3	3	3.443	0.451	1	535	28.260868	R.RAPAAQPPAAAAPSAVGSPAAAPR.Q
CHCH3_MOUSE	MK_SCX_19.6424.6424.2	2	2.601	0.193	1	317.3	50	R.VTFEADENENITVVK.G
CHCH3_MOUSE	MK_SCX_20_1.4208.4208.2	2	4.949	0.629	1	1461.9	78.57143	R.YSSVYGASVSDEDLK.R
CHCH3_MOUSE	MK_SCX_21.3986.3986.2	2	3.662	0.171	1	1002.5	80	R.VAEELALEQAK.K
CHCH3_MOUSE	MK_SCX_2201.567.567.2	2	2.495	0.475	1	874.6	92.85714	K.AAEEVEAK.F
CHCH3_MOUSE	MK_SCX_24.3223.3223.2	2	2.905	0.181	1	1031	92.85714	R.LSENVIDR.M
CHCH3_MOUSE	MK_SCX_31.3878.3878.2	2	4.868	0.533	1	1350.5	70	R.YSSVYGASVSDEDLKR.R
CHCH3_MOUSE	MK_SCX_33.3454.3454.3	3	3.3	0.356	1	491.8	47.727272	R.VAEELALEQAKK.E
CHCH3_MOUSE	MK_SCX_35.12805.12805.2	2	3.842	0.497	1	773.5	56.666668	R.RVTFEADENENITVVK.G
CHCH3_MOUSE	MK_SCX_35.15152.15152.3	3	3.467	0.434	1	591.3	35	R.RVTFEADENENITVVK.G
CHCH3_MOUSE	MK_SCX_40.3564.3564.3	3	3.986	0.293	1	1078.1	54.545456	R.RVAEELALEQAK.K
CHCH3_MOUSE	MK_SCX_54.3096.3096.3	3	3.154	0.287	1	456.1	43.18182	R.KQDAFYKEQLAR.L
CHCH3_MOUSE	MK_SCX_54.3528.3528.3	3	5.102	0.512	1	1722.2	50	R.VAEELALEQAKKESEHQR.R
CHCH6_MOUSE	MK_SCX_20_1.4128.4128.2	2	6.027	0.502	1	2616.1	88.46153	K.FQQEQLAVQDEM*VR.V
CHCH6_MOUSE	MK_SCX_36.4186.4186.3	3	3.862	0.433	1	2963.2	58.928574	K.KFQQEQLAVQDEMVR.V
CHCH6_MOUSE	MK_SCX_41.3445.3445.3	3	4.242	0.264	1	1625.6	62.5	R.RVSFEMDEEER.V
CHCH6_MOUSE	MK_SCX_41.3447.3447.2	2	3.172	0.276	1	952.2	75	R.RVSFEMDEEER.V
CHD4_MOUSE	MK_SCX_19.6634.6634.2	2	3.327	0.464	1	489.1	63.333332	R.YGMPPQDAFTTQWLVR.D
CHD4_MOUSE	MK_SCX_20_1.3013.3013.3	3	3.062	0.322	1	761.1	35.294117	R.AYHMVCLDPDM*EKAPEGK.W
CHD4_MOUSE	MK_SCX_41.3261.3261.3	3	5.955	0.489	1	1119.3	47.22222	K.IEENSLKEEESTEGEKEVK.S
CHD9_MOUSE	MK_SCX_17.9441.9441.2	2	2.516	0.173	1	382.2	37.5	K.KEVSPGVM*LDIEEFFVK.Y
CHD9_MOUSE	MK_SCX_26.10948.10948.2	2	2.192	0.25	1	400.6	50	R.DLLIGAAKHGVSR.T
CHDH_MOUSE	MK_SCX_15.6427.6427.2	2	5.327	0.58	1	1106.7	52.083332	R.VVDASIM*PSVVSGNLNAPTVMIAEK.A
CHDH_MOUSE	MK_SCX_15.6778.6778.2	2	5.604	0.645	1	955.6	47.916664	R.VVDASIMPSVVSGNLNAPTVM*IAEK.A
CHDH_MOUSE	MK_SCX_15.7132.7132.2	2	4.754	0.667	1	798.1	45.833336	R.VVDASIMPSVVSGNLNAPTVMIAEK.A
CHDH_MOUSE	MK_SCX_15.7186.7186.3	3	5.948	0.535	1	1378.6	38.541664	R.VVDASIMPSVVSGNLNAPTVMIAEK.A
CHDH_MOUSE	MK_SCX_16.6717.6717.2	2	2.862	0.368	1	690.5	53.333336	R.VLLLEAGPKDLLM*GSK.R
CHDH_MOUSE	MK_SCX_32.5564.5564.2	2	4.323	0.34	1	840.3	73.07692	R.EIFAQEALAPFRGK.E
CHDH_MOUSE	MK_SCX_42.4354.4354.2	2	4.457	0.496	1	2016.8	72.22222	K.ELQPGSHVQSDKEIDAFVR.A
CHDH_MOUSE	MK_SCX_53.4196.4196.3	3	4.176	0.439	1	740.2	37.5	R.GKELQPGSHVQSDKEIDAFVR.A
CHDH_MOUSE	MK_SCX_59.10881.10881.3	3	3.325	0.412	1	356.2	33.333336	R.GKTNHPLHQAFLQAAR.Q
CHM2A_MOUSE	MK_SCX_17.15669.15669.2	2	3.311	0.225	1	837	61.764706	K.AEATASALADADADLEER.L
CHM2A_MOUSE	MK_SCX_58.8314.8314.2	2	2.467	0.146	1	357.4	75	R.RKTPEELLR.Q
CHM2B_MOUSE	MK_SCX_16.6654.6654.2	2	3.454	0.134	1	346.4	32.608696	K.VM*NSQMKMAGAM*STTAKTMQAVNK.K
CHM2B_MOUSE	MK_SCX_16.7229.7229.3	3	3.916	0.177	1	750.9	28.260868	K.VM*NSQMKMAGAM*STTAKTMQAVNK.K

CHM4B_MOUSE	MK_SCX_19.5864.5864.2	2	3.33	0.431	1	553.5	60.000004	K.QLAQIDGTLSTIEFQR.E
CHM4B_MOUSE	MK_SCX_20_1.3724.3724.2	2	4	0.449	1	984.3	76.92308	R.EALENANTNTEVLK.N
CHMP3_MOUSE	MK_SCX_49.4664.4664.3	3	3.878	0.454	1	713.4	36.666668	K.TQEKPPKELVNEWSLK.I
CHMP3_MOUSE	MK_SCX_49.4665.4665.2	2	5.044	0.612	1	915.1	76.666664	K.TQEKPPKELVNEWSLK.I
CHMP5_MOUSE	MK_SCX_23.6507.6507.3	3	4.459	0.547	1	1032.5	34.782608	R.DNLAQQSFNMEQANYTIQSLKDTK.T
CHMP6_MOUSE	MK_SCX_19.3887.3887.2	2	4.923	0.539	1	1772.6	83.33333	R.ILDETQEAVEYQR.Q
CHP1_MOUSE	MK_SCX_18.12025.12025.2	2	2.957	0.213	1	404	50	R.MMVGVNISDEQLGSIADR.T
CHP1_MOUSE	MK_SCX_20_1.10553.10553.2	2	3.022	0.396	1	494.1	56.666668	R.IINAFFSEGEDQVNFR.G
CHP1_MOUSE	MK_SCX_20_1.15423.15423.3	3	3.002	0.411	1	593.3	35	R.IINAFFSEGEDQVNFR.G
CHP1_MOUSE	MK_SCX_20_1.6122.6122.2	2	4.002	0.461	1	1904.1	86.36364	R.IPELAINPLGDR.I
CHP1_MOUSE	MK_SCX_27.6939.6939.3	3	4.966	0.415	1	1288.7	46.875	R.EDFQRIPELAINPLGDR.I
CHP1_MOUSE	MK_SCX_27.6967.6967.2	2	3.796	0.479	1	420.1	56.25	R.EDFQRIPELAINPLGDR.I
CHP1_MOUSE	MK_SCX_47.3572.3572.3	3	3.368	0.339	1	1466.3	61.363636	R.LYDLDKDDKISR.D
CHP1_MOUSE	MK_SCX_47.4399.4399.3	3	4.272	0.472	1	1529.5	57.14286	R.ASTLLRDEELEEIKK.E
CHSTA_MOUSE	MK_SCX_23.8282.8282.3	3	4.09	0.278	1	1012.7	32.608696	K.DPDGYSAKQEFVFLTTM*PEAEKLR.G
CI078_MOUSE	MK_SCX_11.6856.6856.2	2	2.574	0.144	1	377.6	40.625	K.RPANEKATDDYHYEKFK.K
CI078_MOUSE	MK_SCX_13.7411.7411.1	1	3.088	0.208	1	349.5	65	R.FYHEELNAPIR.R
CIC_MOUSE	MK_SCX_25.4138.4138.3	3	5.056	0.593	1	999.4	37.962963	K.TQESGQGSTAVPLRPPPPGAGGPATPSK.A
CIRBP_MOUSE	MK_SCX_17.13147.13147.2	2	4.247	0.549	1	575.6	52.499996	K.LFVGGLSFDTNEQALEQVFSK.Y
CISY_MOUSE	MK_SCX_18.5572.5572.2	2	4.428	0.629	1	1376.1	70	K.GLVYETSVLDPDEGIR.F
CISY_MOUSE	MK_SCX_24.10390.10390.3	3	6.184	0.549	1	2347	45.454548	R.LDWSHNFTNMLGYTDPQFTELMR.L
CISY_MOUSE	MK_SCX_38.3873.3873.3	3	3.026	0.332	1	1081.6	48.214287	R.NLYREGSSIGAIDSR.L
CISY_MOUSE	MK_SCX_38.4130.4130.2	2	2.244	0.22	1	554.3	77.77778	R.ALGFPLERPK.S
CJ011_MOUSE	MK_SCX_25.3965.3965.3	3	4.258	0.388	1	1973.7	40.625	K.ASSEEEKAAAPENQPQYTPLPSGSR.D
CJ058_MOUSE	MK_SCX_19.6083.6083.2	2	3.788	0.342	1	1001.4	79.16667	K.LDELGVPLYAVVK.E
CJ058_MOUSE	MK_SCX_34.6379.6379.2	2	4.993	0.521	1	1683.2	83.33333	R.KAALEYLEDIDLK.T
CJ058_MOUSE	MK_SCX_46.6844.6844.3	3	3.198	0.344	1	413.3	29.411766	R.EKEFGDRVNPLSVLEAVK.K
CJ070_MOUSE	MK_SCX_2201.3456.3456.2	2	3.151	0.324	1	1100.1	81.25	K.AM*VNLQIQK.D
CJ070_MOUSE	MK_SCX_32.4377.4377.2	2	4.182	0.58	1	1103.4	83.33333	K.VVHAFDMEDLGDK.A
CJ070_MOUSE	MK_SCX_32.4392.4392.3	3	4.431	0.401	1	1411.7	54.166668	K.VVHAFDMEDLGDK.A
CJ070_MOUSE	MK_SCX_34.3842.3842.2	2	3.618	0.42	1	416.3	60.714287	K.HNEETGDNVGPLIIK.K
CJ070_MOUSE	MK_SCX_35.3678.3678.2	2	3.937	0.187	1	1702	75	K.AMVNLQIQKDNPK.V
CJ070_MOUSE	MK_SCX_38.5013.5013.3	3	3.431	0.376	1	1567.6	45.3125	K.DNPKVVHAFDMEDLGDK.A
CJ070_MOUSE	MK_SCX_49.3704.3704.3	3	5.659	0.617	1	2209.7	56.666668	K.HNEETGDNVGPLIIKK.K
CJ070_MOUSE	MK_SCX_49.3717.3717.2	2	5.858	0.573	1	2951.1	90	K.HNEETGDNVGPLIIKK.K
CJ070_MOUSE	MK_SCX_54.6611.6611.3	3	3.292	0.439	1	531.1	32.5	K.VVHAFDMEDLGDKAVYCRCWR.S
CK054_MOUSE	MK_SCX_17.7989.7989.2	2	2.456	0.222	1	528	44.444447	K.APLVCLPVFVSKDPGLDLR.L
CK054_MOUSE	MK_SCX_18.7175.7175.2	2	4.99	0.428	1	951.3	73.333336	R.IAEVGGVPYLLPLVNK.K
CK054_MOUSE	MK_SCX_28.6781.6781.2	2	4.853	0.398	1	754.5	68.75	R.IAEVGGVPYLLPLVNKK.K
CK054_MOUSE	MK_SCX_38.4427.4427.3	3	4.523	0.392	1	849.3	34.523808	R.QTLEEHYGDKPVGM*GGTFIVQK.G
CK054_MOUSE	MK_SCX_38.5060.5060.3	3	5.011	0.514	1	961.9	35.714287	R.QTLEEHYGDKPVGMGGTFIVQK.G
CK054_MOUSE	MK_SCX_38.5213.5213.2	2	5.078	0.552	1	474.6	54.761906	R.QTLEEHYGDKPVGMGGTFIVQK.G
CK054_MOUSE	MK_SCX_47.5084.5084.2	2	2.246	0.334	1	569	71.42857	K.WLHFYEMK.A
CK067_MOUSE	MK_SCX_28.5618.5618.3	3	4.096	0.473	1	942.2	47.058823	R.GMSEALKVPPSTVEYLEK.Q
CK067_MOUSE	MK_SCX_38.3515.3515.3	3	4.869	0.448	1	779.4	38.75	R.ETGTEHSPGVQPADVKEVAEK.G
CL004_MOUSE	MK_SCX_28.3771.3771.3	3	3.006	0.144	1	432.2	31.25	K.EARDSGNQNGGSDDKSK.N
CL004_MOUSE	MK_SCX_31.5928.5928.2	2	3.823	0.496	1	614.7	71.42857	K.SGEVDLHQLASAWAK.A
CLCA_MOUSE	MK_SCX_38.5106.5106.3	3	4.024	0.39	1	819.2	52.499996	K.AIKELEEWYAR.Q
CLCA_MOUSE	MK_SCX_39.5572.5572.2	2	3.434	0.373	1	1156.3	80	K.AIKELEEWYAR.Q
CLCB_MOUSE	MK_SCX_14.4645.4645.2	2	3.114	0.607	1	1066.3	83.33333	K.VAQLCDFNPK.S
CLCB_MOUSE	MK_SCX_30.4939.4939.3	3	5.225	0.546	1	901.8	55	R.LQELDAASKVTEQEWR.E

CLCB_MOUSE	MK_SCX_30.4960.4960.2	2	5.089	0.524	1	2459.2	80	R.LQELDAASKVTEQEWR.E
CLH_MOUSE	MK_SCX_14.7489.7489.2	2	3.204	0.384	1	1553	50	K.ADDPSSYMEVVQAANASGNWEELVK.Y
CLH_MOUSE	MK_SCX_17.9196.9196.2	2	3.873	0.448	1	576.6	53.125	K.AFM*TADLPNELIELLEK.I
CLH_MOUSE	MK_SCX_17.9730.9730.2	2	2.304	0.159	1	365.7	50	K.AFMTADLPNELIELLEK.I
CLH_MOUSE	MK_SCX_18.5739.5739.2	2	3.848	0.478	1	1104.3	65.625	R.TSIDAYDNFDNISLAQR.L
CLH_MOUSE	MK_SCX_18.7083.7083.2	2	4.933	0.534	1	909.3	57.894737	K.SVNESLNNLFITEEDYQALR.T
CLH_MOUSE	MK_SCX_23.4973.4973.2	2	3.276	0.318	1	911.9	81.818184	R.NNLAGAEELFAR.K
CLH_MOUSE	MK_SCX_24.4436.4436.2	2	3.852	0.569	1	1272.1	85	K.LLYNNVSNFGR.L
CLH_MOUSE	MK_SCX_29.5574.5574.3	3	4.626	0.478	1	1583	43.055553	R.LASTLVHLGEYQAAVDGAR.K
CLH_MOUSE	MK_SCX_31.5539.5539.3	3	3.129	0.522	1	374.9	35.294117	K.LHIIEVGTPPTGNQPFPK.K
CLH_MOUSE	MK_SCX_43.3100.3100.3	3	3.095	0.183	1	572.2	43.18182	R.ICRESNCYDPER.V
CLH_MOUSE	MK_SCX_51.4002.4002.3	3	4.003	0.407	1	617.6	40.625	R.KVSQPIEGHAASFAQFK.M
CLIC1_MOUSE	MK_SCX_14.6535.6535.2	2	5.371	0.635	1	671.3	42.307693	K.VLDNYLTSPLPEEVDETSAEDEGISQR.K
CLIC1_MOUSE	MK_SCX_15.6450.6450.3	3	5.539	0.492	1	922	32.692307	K.VLDNYLTSPLPEEVDETSAEDEGISQR.K
CLIC1_MOUSE	MK_SCX_18.6094.6094.2	2	4.52	0.677	1	868.3	64.70589	K.LAALNPESNTSGLDIFAK.F
CLIC1_MOUSE	MK_SCX_21.6500.6500.3	3	3.472	0.373	1	593.9	26.85185	K.VLDNYLTSPLPEEVDETSAEDEGISQRK.F
CLIC1_MOUSE	MK_SCX_2201.3146.3146.2	2	2.779	0.306	1	535.7	68.181816	K.NSNPALNDNLEK.G
CLIC1_MOUSE	MK_SCX_45.5708.5708.2	2	3.046	0.422	1	1123.9	80	K.YRGFTIPEAFR.G
CLIC4_MOUSE	MK_SCX_13.7253.7253.2	2	3.856	0.516	1	408.6	53.125	R.FLDGDEMTLADCNLLPK.L
CLIC4_MOUSE	MK_SCX_15.6015.6015.2	2	4.248	0.57	1	1040.7	52.380955	R.DEFTNTCPSDKEVEIAYSDVAK.R
CLIC4_MOUSE	MK_SCX_15.6099.6099.2	2	5.255	0.61	1	1286.8	59.523808	K.LDEYLNSPLPDEIDENSM*EDIK.F
CLIC4_MOUSE	MK_SCX_15.6595.6595.2	2	6.155	0.598	1	1746.3	64.28571	K.LDEYLNSPLPDEIDENSMEDIK.F
CLIC4_MOUSE	MK_SCX_15.6619.6619.3	3	5.397	0.434	1	1784.3	45.238094	K.LDEYLNSPLPDEIDENSMEDIK.F
CLIC4_MOUSE	MK_SCX_2201.7112.7112.3	3	6.452	0.405	1	2078.1	38	K.TLQKLDEYLNSPLPDEIDENSM*EDIK.F
CLIC4_MOUSE	MK_SCX_2201.7638.7638.3	3	6.523	0.543	1	1816.6	40	K.TLQKLDEYLNSPLPDEIDENSMEDIK.F
CLIC4_MOUSE	MK_SCX_2201.7706.7706.2	2	4.74	0.624	1	991.8	44	K.TLQKLDEYLNSPLPDEIDENSMEDIK.F
CLIC4_MOUSE	MK_SCX_37.3919.3919.2	2	3.86	0.503	1	692.1	76.92308	K.HPESNTAGMDIFAK.F
CLIC4_MOUSE	MK_SCX_43.4266.4266.3	3	3.865	0.168	1	454	39.705883	K.FSAYIKNSRPEANEALER.G
CLIC4_MOUSE	MK_SCX_48.3487.3487.3	3	3.587	0.371	1	630.2	39.705883	K.LSPKHPESNTAGM*DIFAK.F
CLIC4_MOUSE	MK_SCX_49.4189.4189.3	3	3.605	0.518	1	743.6	42.647057	K.LSPKHPESNTAGMDIFAK.F
CLIC5_MOUSE	MK_SCX_13.7614.7614.2	2	3.757	0.306	1	1197.4	65.625	K.FLDGDELTLADCNLLPK.L
CLIC5_MOUSE	MK_SCX_21.7200.7200.2	2	4.566	0.56	1	984.1	87.5	R.NYDIPAEMTGLWR.Y
CLIC5_MOUSE	MK_SCX_2201.5698.5698.3	3	4.222	0.495	1	769.2	36.904762	K.LDDYLNSPLPEEIDTNTHGDEK.G
CLIC5_MOUSE	MK_SCX_27.5984.5984.2	2	4.369	0.466	1	1559.6	66.66667	K.IEEFLEETLTPEKYPK.L
CLIC5_MOUSE	MK_SCX_35.9185.9185.3	3	6.294	0.566	1	1646.8	44.31818	R.KLDDYLNSPLPEEIDTNTHGDEK.G
CLIC5_MOUSE	MK_SCX_36.11718.11718.3	3	6.729	0.473	1	3022.8	48.75	K.TDVNKIEEFLEETLTPEKYPK.L
CLIC5_MOUSE	MK_SCX_48.5365.5365.3	3	6.599	0.552	1	2603.9	39.423077	R.KLDDYLNSPLPEEIDTNTHGDEKGSQR.K
CLIC5_MOUSE	MK_SCX_55.3642.3642.3	3	3.745	0.425	1	2156.1	59.615387	K.HRESNTAGIDIFSK.F
CLIC5_MOUSE	MK_SCX_55.3656.3656.2	2	4.433	0.424	1	1348.7	80.769226	K.HRESNTAGIDIFSK.F
CLMN_MOUSE	MK_SCX_19.4386.4386.2	2	4.285	0.535	1	520.2	59.375	K.TGSIAEPTPESSILSTR.K
CLMN_MOUSE	MK_SCX_2201.8078.8078.3	3	4.794	0.553	1	660.9	33.695652	R.FPELEPEDFVNPDKEAPIESTFVR.I
CLMN_MOUSE	MK_SCX_47.4374.4374.3	3	3.026	0.315	1	691.9	43.75	R.SHSEEGLDFKPSPPLSK.I
CLPP_MOUSE	MK_SCX_19.5601.5601.2	2	2.582	0.194	1	938.7	60.714287	R.GQATDIAIQAEEIMK.L
CLPP_MOUSE	MK_SCX_19.5624.5624.2	2	4.122	0.504	1	1640	78.57143	R.YMSPMEAQEFGILDK.V
CLPP_MOUSE	MK_SCX_2201.3860.3860.2	2	4.004	0.493	1	1312.3	86.36364	K.QSLQVIESAM*ER.D
CLPP_MOUSE	MK_SCX_27.4324.4324.3	3	5.402	0.354	1	1702.4	51.47059	K.VLVHPPQDGEDEPELVQK.E
CLPP_MOUSE	MK_SCX_28.6171.6171.2	2	4.384	0.422	1	1123.5	65.625	R.DRYMSPMEAQEFGILDK.V
CLPP_MOUSE	MK_SCX_28.6233.6233.3	3	5.381	0.513	1	1387.3	43.75	R.DRYMSPMEAQEFGILDK.V
CLPX_MOUSE	MK_SCX_16.8153.8153.2	2	3.417	0.53	1	840.8	73.07692	R.DLIEFGMIPEFVGR.L
CLPX_MOUSE	MK_SCX_18.4014.4014.2	2	4.448	0.41	1	2037.9	87.5	R.DVGGEGVQQGLLK.L
CLPX_MOUSE	MK_SCX_20_1.4801.4801.2	2	4.202	0.492	1	1989.7	90.909096	K.LLEGTIVNVPEK.N

CLPX_MOUSE	MK_SCX_2201.4948.4948.2	2	2.907	0.368	1	1364	88.88889	K.TLVQILTEPR.N
CLPX_MOUSE	MK_SCX_23.4631.4631.2	2	2.2	0.345	1	358.1	54.545456	K.YLGFGTPSNLGK.G
CLPX_MOUSE	MK_SCX_23.6335.6335.3	3	6.885	0.551	1	1721.8	41.37931	K.LLQIAGISPHGNALGASMQQQVNQQMPQEK.R
CLPX_MOUSE	MK_SCX_25.4200.4200.2	2	3.203	0.261	1	778.8	94.44444	R.IYNNIPANLR.Q
CLPX_MOUSE	MK_SCX_26.10051.10051.3	3	3.649	0.464	1	534.5	28.846153	R.DLIEFGM*IPEFVGRLPVVVPLHSLDEK.T
CLPX_MOUSE	MK_SCX_33.3682.3682.2	2	2.469	0.214	1	406.7	50	K.SIIKEPESAAEAVK.L
CLPX_MOUSE	MK_SCX_35.11402.11402.2	2	3.703	0.463	1	1052.3	60.000004	K.CGDLCTHVETFVSSTR.F
CLPX_MOUSE	MK_SCX_48.3765.3765.3	3	5.323	0.564	1	1421.9	45.3125	K.RGGEVLDSSQDDIKLEK.S
CLPX_MOUSE	MK_SCX_50.5786.5786.2	2	2.292	0.302	1	382.7	50	K.KIYNYLDKYVVGQSFAK.K
CLPX_MOUSE	MK_SCX_51.3309.3309.3	3	3.706	0.441	1	698.3	48.214287	K.KSIIKEPESAAEAVK.L
CLUS_MOUSE	MK_SCX_21.6474.6474.2	2	2.714	0.474	1	1081	68.181816	R.ASGIIDTLFQDR.F
CLUS_MOUSE	MK_SCX_33.6384.6384.3	3	3.89	0.441	1	1163	51.785713	R.LTEQYKELLQSFQSK.M
CMC1_MOUSE	MK_SCX_15.8744.8744.2	2	3.893	0.619	1	597.3	38.88889	R.VGGINLLTAGALAGVPAASLVTPADVIK.T
CMC1_MOUSE	MK_SCX_18.6849.6849.2	2	5.352	0.476	1	1151	70.588234	R.IAPLAEGALPYNLAELQR.Q
CMC1_MOUSE	MK_SCX_25.4946.4946.2	2	2.934	0.359	1	1356.9	100	K.FGLYLPK.F
CMC1_MOUSE	MK_SCX_25.5877.5877.2	2	2.687	0.442	1	733.3	87.5	R.YEGFFGLYR.G
CMC1_MOUSE	MK_SCX_39.3482.3482.3	3	3.161	0.434	1	1030.2	45.454548	K.FKSPSVAVAQPK.A
CMC1_MOUSE	MK_SCX_49.4218.4218.3	3	3.504	0.464	1	857	45	R.KDIEVTKEEFAQSAIR.Y
CMC2_MOUSE	MK_SCX_14.9735.9735.2	2	4.629	0.592	1	661	31.944445	K.ASFANEDGQVSPGSLLLAGAIAGMPAASLVTPADVIK.T
CMC2_MOUSE	MK_SCX_17.8735.8735.2	2	6.881	0.609	1	2035.7	64.28571	R.FGLGSIAGAVGATAVYPIDLVK.T
CMC2_MOUSE	MK_SCX_18.6659.6659.2	2	4.448	0.562	1	922.5	61.764706	R.IAPLEEGM*LPFNLAEAQR.Q
CMC2_MOUSE	MK_SCX_18.7288.7288.2	2	4.42	0.576	1	989.9	67.64706	R.IAPLEEGMLPFNLAEAQR.Q
CMC2_MOUSE	MK_SCX_21.16957.16957.2	2	2.126	0.228	1	511.4	63.636364	K.LAVATFAGIENK.F
CMC2_MOUSE	MK_SCX_2201.4780.4780.2	2	2.763	0.197	1	441.9	58.333332	R.YLNIFGESQPNPK.T
CMC2_MOUSE	MK_SCX_30.7040.7040.2	2	2.522	0.328	1	365.1	50	K.FGLYLPLFKPSASTSK.V
CMC2_MOUSE	MK_SCX_30.7407.7407.3	3	4.183	0.521	1	1160.9	37.5	K.ASGDAARPFLLQLAESAYR.F
CMC2_MOUSE	MK_SCX_31.4423.4423.2	2	3.261	0.5	1	888.1	75	R.ITLPAPNPDHVGGYK.L
CMC2_MOUSE	MK_SCX_47.4256.4256.3	3	3.553	0.469	1	833.6	46.666668	R.KDVEVTKEEFALAAQK.F
CMGA_MOUSE	MK_SCX_21.3834.3834.2	2	2.013	0.165	1	500.5	59.090908	R.AQQPLKQQQPPK.Q
CMGA_MOUSE	MK_SCX_23.8830.8830.3	3	3.646	0.138	1	550.1	25	R.STAVLALLLCAGQVFALPVNSPMTK.G
CN092_MOUSE	MK_SCX_26.4071.4071.3	3	5.163	0.563	1	1857	43.421055	R.DTQAAIKGQNPNATFGEVSK.I
CN166_MOUSE	MK_SCX_18.5806.5806.2	2	3.132	0.459	1	1213.1	60.000004	K.NAEPLINLDVNNPDFK.A
CN166_MOUSE	MK_SCX_21.8035.8035.2	2	3.084	0.324	1	1535	73.07692	K.AGVMALANLLQIQR.H
CN166_MOUSE	MK_SCX_24.6798.6798.2	2	2.86	0.132	1	914.4	77.77778	R.NFIVWLEDQK.I
CN166_MOUSE	MK_SCX_29.12486.12486.3	3	3.807	0.302	1	734.9	36.842106	K.HILGFDTGDAVLNEAAQILR.L
CN166_MOUSE	MK_SCX_37.4600.4600.2	2	2.101	0.256	1	428.1	68.75	R.HDDYLVMLK.A
CNBP_MOUSE	MK_SCX_45.3511.3511.2	2	2.677	0.262	1	1265.6	72.72727	K.CYSCGEFGHIQK.D
CNBP_MOUSE	MK_SCX_45.3515.3515.3	3	3.172	0.182	1	794.4	45.454548	K.CYSCGEFGHIQK.D
CNN1_MOUSE	MK_SCX_31.6959.6959.3	3	3.392	0.395	1	485.5	33.333336	K.VNESTQNWHQLENIGNFIK.A
CNN1_MOUSE	MK_SCX_50.6203.6203.3	3	5.146	0.392	1	1010.9	39.473686	K.KVNESTQNWHQLENIGNFIK.A
CNN2_MOUSE	MK_SCX_19.7897.7897.2	2	4.577	0.479	1	2413.4	81.25	R.SWIEGLTGLSIGPDFQK.G
CNN2_MOUSE	MK_SCX_37.7895.7895.3	3	3.594	0.295	1	317.3	31.666666	R.SMQNWHQLENLSNFIK.A
CNN2_MOUSE	MK_SCX_49.3832.3832.3	3	3.513	0.25	1	527.8	40.384613	R.LLSKYDPQKEAELR.S
CNN3_MOUSE	MK_SCX_17.5668.5668.2	2	5.603	0.576	1	724.2	58.333332	K.LTLQPVDNSTISLQMGTNK.V
CNN3_MOUSE	MK_SCX_19.8110.8110.2	2	4.808	0.683	1	991.5	66.66667	K.VNESSLNWPQLENIGNFIK.A
CNN3_MOUSE	MK_SCX_27.5416.5416.3	3	3.57	0.366	1	841.1	38.157894	K.MQTDKPFDQTTISLQMGTNK.G
CNN3_MOUSE	MK_SCX_33.8297.8297.2	2	5.443	0.592	1	947.8	60.526318	K.KVNESSLNWPQLENIGNFIK.A
CNN3_MOUSE	MK_SCX_33.8395.8395.3	3	5.159	0.566	1	2342.2	47.368423	K.KVNESSLNWPQLENIGNFIK.A
CO024_MOUSE	MK_SCX_31.5608.5608.2	2	3.718	0.596	1	665.5	60.000004	R.AVVPGVKPQDWISAAR.V
CO3_MOUSE	MK_SCX_16.4951.4951.2	2	6.056	0.644	1	1095.4	68.42105	K.VDVPAADLSDQVPDTDSETR.I
CO3_MOUSE	MK_SCX_17.7394.7394.2	2	5.199	0.647	1	1754.9	65.789474	R.IILQGSPVVQMAEDAVDGER.L

CO3_MOUSE	MK_SCX_2201.2688.2688.2	2	2.515	0.364	1	545.8	70	K.VLVVTQGSNAK.A
CO4B_MOUSE	MK_SCX_16.7246.7246.2	2	4.038	0.567	1	1121.9	59.090908	R.TLEIPGSSDPNIVPDGDFSSLVR.V
CO6A1_MOUSE	MK_SCX_18.5034.5034.2	2	5.557	0.556	1	1434.6	63.88889	R.VLLFSDGNSQGATAEAIEK.A
CO6A1_MOUSE	MK_SCX_25.4775.4775.2	2	3.141	0.199	1	679.4	93.75	R.VPNYQALLR.G
CO6A1_MOUSE	MK_SCX_39.5815.5815.2	2	2.076	0.123	1	309.6	54.545456	R.RNFTAADWGHSR.D
CO6A1_MOUSE	MK_SCX_46.5533.5533.3	3	3.869	0.477	1	854.2	45	K.GLEELLIGGSHLKENK.Y
CO6A2_MOUSE	MK_SCX_29.6923.6923.2	2	2.714	0.19	1	497.3	57.14286	K.GAKGNMGEPGEPGQK.G
CO6A2_MOUSE	MK_SCX_42.7986.7986.3	3	3.402	0.505	1	399.4	29.545454	R.ATYLNSFSHVGTGIVHAINNVVR.G
CO9_MOUSE	MK_SCX_23.4390.4390.2	2	2.38	0.142	1	702.3	75	K.TSNFNADFALK.F
CO9_MOUSE	MK_SCX_26.8546.8546.3	3	3.403	0.268	1	812.5	32.608696	K.TFDKTDFANWASSLANAPALISQR.M
COASY_MOUSE	MK_SCX_17.9082.9082.3	3	4.685	0.634	1	402.5	17.934782	R.YATSCYSCSPQLASVLLYPDYGTGELPLEPPNALLPSTIRPASPVAR.S
COASY_MOUSE	MK_SCX_23.7240.7240.2	2	4.58	0.59	1	612.1	50	R.AYAPGGPAYQPVVEAFGTDILHK.D
COASY_MOUSE	MK_SCX_41.2942.2942.3	3	4.257	0.398	1	967.7	39.705883	K.DQSHNENEEDKVSSSSFR.Q
COASY_MOUSE	MK_SCX_55.5145.5145.3	3	3.29	0.477	1	517.2	37.5	R.LKNLGAYIIDSDHLGHR.A
COBA2_MOUSE	MK_SCX_18.6811.6811.2	2	2.217	0.14	1	658.6	55.88235	R.GFPGERGLPGTAGGPGLK.G
COBA2_MOUSE	MK_SCX_25.8739.8739.2	2	2.55	0.144	1	311.1	35	K.GEKGHPGLIGLIGPTGEQGEK.G
COF1_MOUSE	MK_SCX_16.6652.6652.2	2	6.21	0.485	1	2303.5	71.05263	K.EILVGDVGQTVDDPYTTFVK.M
COF1_MOUSE	MK_SCX_20_1.7005.7005.2	2	4.634	0.468	1	1278.9	88.46153	K.LGGSAVISLEGK.P
COF1_MOUSE	MK_SCX_21.4226.4226.2	2	3.133	0.47	1	1668.1	90	R.YALYDATYETK.E
COF1_MOUSE	MK_SCX_21.8722.8722.3	3	6.222	0.48	1	1514.1	32.407406	K.NIILEEGKEILVGDVGQTVDDPYTTFVK.M
COF1_MOUSE	MK_SCX_21.8765.8765.2	2	5.835	0.668	1	1436.3	50	K.NIILEEGKEILVGDVGQTVDDPYTTFVK.M
COF1_MOUSE	MK_SCX_29.13066.13066.2	2	2.77	0.425	1	326.3	46.875	K.KEDLVFIFWAPENAPLK.S
COF2_MOUSE	MK_SCX_16.7139.7139.2	2	5.841	0.553	1	1985	68.42105	K.QILVGDIGDTVEDPYTSFVK.L
COF2_MOUSE	MK_SCX_20_1.8829.8829.2	2	2.643	0.22	1	845	73.07692	K.LGGSVVVSLEGK.P
COF2_MOUSE	MK_SCX_50.4364.4364.3	3	3.679	0.34	1	1896.2	51.923077	K.HEWQVNGLDDIKDR.S
COFA1_MOUSE	MK_SCX_21.11914.11914.3	3	3.024	0.135	1	319.5	22.115383	K.ILDQKAYSCANRLIVLCIENSFMTDTR.K
COFA1_MOUSE	MK_SCX_23.5988.5988.2	2	2.056	0.315	1	376.1	75	R.FGLPIVNLK.G
COIA1_MOUSE	MK_SCX_15.7196.7196.2	2	2.728	0.262	1	620.9	41.304348	K.DEVLSPSWDSLFSGSQGQLQPGAR.I
COIA1_MOUSE	MK_SCX_20_1.4072.4072.2	2	4.905	0.547	1	2633.5	78.125	R.TETTGATGQASSLLSGR.L
COIA1_MOUSE	MK_SCX_2201.8958.8958.3	3	6.126	0.459	1	881	29.6875	R.GSVPIVNLKDEVLSPSWDSLFSGSQGQLQPGAR.I
COIA1_MOUSE	MK_SCX_26.5679.5679.3	3	4.286	0.583	1	819.7	38.04348	R.GTGNEVAALQPPLVQLHEGSPYTR.R
COMD1_MOUSE	MK_SCX_17.8219.8219.2	2	5.271	0.629	1	1012.2	60.000004	K.SIASADM*DFNQLEAFLTAQTK.K
COMD1_MOUSE	MK_SCX_17.8533.8533.2	2	5.256	0.602	1	1625.1	65	K.SIASADMDFNQLEAFLTAQTK.K
COMD1_MOUSE	MK_SCX_38.3268.3268.3	3	3.881	0.241	1	1454.7	48.214287	K.KQGGITSEQAAVISK.F
COMD3_MOUSE	MK_SCX_17.8523.8523.2	2	3.437	0.426	1	897.2	52.499996	R.GIQTLADPGSFDSNAFALLLR.A
COMD3_MOUSE	MK_SCX_2201.7175.7175.2	2	3.445	0.381	1	1505.5	81.818184	K.NSLETLLGSIGR.S
COMD3_MOUSE	MK_SCX_23.4594.4594.2	2	3.529	0.32	1	1121.8	83.33333	R.AAFQSLLDAR.A
COMT_MOUSE	MK_SCX_15.9774.9774.2	2	4.865	0.594	1	1186.7	48	R.LLTMEINPDYAAITQQMLDFAGLQDK.V
COPB_MOUSE	MK_SCX_13.6849.6849.2	2	2.13	0.174	1	408.2	50	R.MLIVEKMLEVFHAIK.S
COPB_MOUSE	MK_SCX_17.5676.5676.2	2	3.028	0.183	1	558.7	52.941177	R.NVTVQPDDPISFM*QLTAK.N
COPB_MOUSE	MK_SCX_17.6498.6498.2	2	3.546	0.518	1	370.3	47.058823	R.NVTVQPDDPISFMQLTAK.N
COPD_MOUSE	MK_SCX_31.5768.5768.2	2	4.171	0.366	1	843.4	67.85714	K.LFTAESLIGLKNPEK.S
COPD_MOUSE	MK_SCX_39.6990.6990.2	2	2.335	0.237	1	393.5	59.090908	R.TRIEGLLAAFPK.L
COPD_MOUSE	MK_SCX_50.5234.5234.3	3	3.772	0.312	1	645.9	41.666664	K.KLFTAESLIGLKNPEK.S
COPD_MOUSE	MK_SCX_51.4063.4063.3	3	3.859	0.278	1	1101.4	50	K.VHAPPINMESVHMK.I
COPE_MOUSE	MK_SCX_17.6251.6251.2	2	4.086	0.479	1	427.4	52.380955	-.APPVPGAVSGGSGEVDELFDVK.N
COPE_MOUSE	MK_SCX_23.4338.4338.2	2	4.838	0.56	1	1946	90.909096	R.MFAEYLASENQR.D
COPE_MOUSE	MK_SCX_25.6096.6096.3	3	4.453	0.471	1	1939.1	43.421055	K.YGVVLDEIKPSSAPELQAVR.M
COPE_MOUSE	MK_SCX_42.5649.5649.3	3	5.996	0.546	1	1360.6	45	R.KYGVVLDEIKPSSAPELQAVR.M
COPZ1_MOUSE	MK_SCX_17.6300.6300.2	2	6.2	0.541	1	1850	77.77778	R.GEDVPLTEQTVSQVLQSAK.E
COPZ1_MOUSE	MK_SCX_26.6768.6768.3	3	4.923	0.472	1	1240.4	36.363636	R.VALRGEDVPLTEQTVSQVLQSAK.E

COQ3_MOUSE	MK_SCX_13.6945.6945.2	2	3.647	0.427	1	819.7	65.625	K.ILDVGCGGGLLTEPLGR.L
COQ3_MOUSE	MK_SCX_18.5233.5233.2	2	4.123	0.494	1	1136.7	70	R.LGASVVGIDPVAENIK.I
COQ3_MOUSE	MK_SCX_36.7330.7330.3	3	3.108	0.423	1	570.7	37.5	K.NFQALAHTWWDEYGK.F
COQ5_MOUSE	MK_SCX_23.4421.4421.2	2	2.759	0.38	1	838.7	77.27273	R.TQQNLSWEEIAK.K
COQ5_MOUSE	MK_SCX_29.16423.16423.3	3	3.874	0.398	1	992.9	33.333336	K.MHPLPGTQLLDMAGGTGDIAFR.F
COQ5_MOUSE	MK_SCX_45.3835.3835.3	3	4.854	0.563	1	1034.4	41.666664	R.AAETHFGFETVSEGEKGSK.V
COQ5_MOUSE	MK_SCX_49.6802.6802.2	2	3.228	0.543	1	600	85.71429	K.KYDLMNDMMSLGIHR.A
COQ5_MOUSE	MK_SCX_49.6937.6937.3	3	3.601	0.532	1	1021.5	44.642857	K.KYDLMNDMMSLGIHR.A
COQ9_MOUSE	MK_SCX_19.7959.7959.2	2	3.187	0.18	1	936.6	55.88235	K.STGEALVQGLMGAAVTLK.N
COQ9_MOUSE	MK_SCX_25.3795.3795.2	2	4.948	0.573	1	1075.6	52.499996	R.YTDQSGEEEEDYESEEQLQHR.I
COQ9_MOUSE	MK_SCX_26.3815.3815.3	3	5.411	0.613	1	1223.1	40	R.YTDQSGEEEEDYESEEQLQHR.I
COQ9_MOUSE	MK_SCX_29.6466.6466.3	3	4.158	0.537	1	1255.1	43.055553	R.LNQVLEEEQKLVQLGQAEK.R
COQ9_MOUSE	MK_SCX_36.6968.6968.2	2	2.295	0.223	1	402	65	R.MLIPYIEHWPR.A
COQ9_MOUSE	MK_SCX_42.5939.5939.3	3	3.642	0.371	1	691.2	36.842106	R.LNQVLEEEQKLVQLGQAEKR.K
COR1B_MOUSE	MK_SCX_17.4291.4291.2	2	6.307	0.674	1	1031.3	56.25	R.SGASTATAVTDVPSGNLAGAGEAGK.L
COR1B_MOUSE	MK_SCX_21.6830.6830.3	3	5.28	0.532	1	543.2	24.242424	R.SGASTATAVTDVPSGNLAGAGEAGKLEEVM*QELR.A
COR1B_MOUSE	MK_SCX_21.8127.8127.3	3	5.005	0.481	1	459.3	21.969696	R.SGASTATAVTDVPSGNLAGAGEAGKLEEVMQELR.A
COR1C_MOUSE	MK_SCX_20_1.15341.15341.2	2	3.146	0.468	1	409	56.666668	R.AIFLADGNVFTTGFSR.M
COR1C_MOUSE	MK_SCX_23.9136.9136.3	3	3.516	0.436	1	920.5	29.464287	R.KSDLFQDDLYPDTAGPEAALEAEEWFEGK.N
COR1C_MOUSE	MK_SCX_51.6183.6183.3	3	4.69	0.379	1	525	41.666664	K.KPTDTASVQNEAKLDEILKEIK.S
COTL1_MOUSE	MK_SCX_36.4988.4988.2	2	3.594	0.398	1	1610.7	86.36364	K.TLVKEVVQNFAK.E
COTL1_MOUSE	MK_SCX_39.3804.3804.2	2	2.68	0.429	1	1162.1	93.75	R.KELEEDFIR.S
COTL1_MOUSE	MK_SCX_45.5715.5715.2	2	5.371	0.61	1	1862.9	71.875	K.TGTDKTLVKEVVQNFAK.E
COTL1_MOUSE	MK_SCX_45.5726.5726.3	3	4.073	0.565	1	891.6	42.1875	K.TGTDKTLVKEVVQNFAK.E
COX17_MOUSE	MK_SCX_19.3511.3511.2	2	3.999	0.571	1	749.5	60.000004	-.PGLAAASPAPPEAQEK.K
COX2_MOUSE	MK_SCX_21.5322.5322.2	2	2.675	0.381	1	767.2	88.88889	R.VVLPM*ELPIR.M
COX2_MOUSE	MK_SCX_21.6674.6674.2	2	2.873	0.49	1	754.3	94.44444	R.VVLPMELPIR.M
COX2_MOUSE	MK_SCX_25.7875.7875.2	2	5.318	0.615	1	1117.8	63.15789	R.M*LISSEDVLHSWAVPSLGLK.T
COX2_MOUSE	MK_SCX_26.7495.7495.2	2	4.06	0.344	1	600.5	46.875	R.LLEVDNRVVLPMELPIR.M
COX2_MOUSE	MK_SCX_26.7507.7507.3	3	4.783	0.343	1	1662.6	51.5625	R.LLEVDNRVVLPMELPIR.M
COX2_MOUSE	MK_SCX_26.8057.8057.3	3	5.182	0.557	1	870	38.157894	R.MLISSEDVLHSWAVPSLGLK.T
COX2_MOUSE	MK_SCX_26.8169.8169.2	2	6.206	0.613	1	1615.9	73.68421	R.MLISSEDVLHSWAVPSLGLK.T
COX41_MOUSE	MK_SCX_20_1.4392.4392.2	2	3.883	0.509	1	897.7	81.818184	K.SEDYAFPTYADR.R
COX41_MOUSE	MK_SCX_23.4258.4258.2	2	4.201	0.406	1	1578.1	86.36364	R.IQFNESFAEM*NR.G
COX41_MOUSE	MK_SCX_23.5026.5026.2	2	4.411	0.592	1	1380.3	86.36364	R.IQFNESFAEMNR.G
COX41_MOUSE	MK_SCX_23.5506.5506.2	2	5.194	0.564	1	1049.7	76.47059	R.DYPLPDVAHVTM*LSASQK.A
COX41_MOUSE	MK_SCX_23.6282.6282.2	2	5.791	0.602	1	1561.3	82.35294	R.DYPLPDVAHVTMLSASQK.A
COX41_MOUSE	MK_SCX_24.3678.3678.2	2	3.062	0.421	1	427.2	77.77778	K.ANPIQGFSAK.W
COX41_MOUSE	MK_SCX_25.3571.3571.2	2	2.119	0.216	1	417.3	78.57143	K.ADWSSLSR.D
COX41_MOUSE	MK_SCX_32.5235.5235.2	2	3.621	0.427	1	886.7	64.28571	R.MLDMKANPIQGFSAK.W
COX41_MOUSE	MK_SCX_34.3919.3919.2	2	2.401	0.306	1	357.5	62.5	K.SEDYAFPTYADRR.D
COX41_MOUSE	MK_SCX_43.3876.3876.2	2	2.57	0.344	1	473.2	68.75	K.WDYDKNEWK.K
COX41_MOUSE	MK_SCX_43.5796.5796.3	3	6.402	0.651	1	2724.1	52.77778	R.RDYPLPDVAHVTMLSASQK.A
COX41_MOUSE	MK_SCX_44.5171.5171.3	3	4.554	0.583	1	1376.3	45.833336	R.RDYPLPDVAHVTM*LSASQK.A
COX41_MOUSE	MK_SCX_44.5214.5214.2	2	4.976	0.616	1	478	63.88889	R.RDYPLPDVAHVTM*LSASQK.A
COX41_MOUSE	MK_SCX_44.5635.5635.2	2	6.232	0.676	1	2419.2	72.22222	R.RDYPLPDVAHVTMLSASQK.A
COX41_MOUSE	MK_SCX_49.4316.4316.3	3	3.803	0.535	1	1207.9	44.444447	R.AHGSVVKSEDYAFPTYADR.R
COX41_MOUSE	MK_SCX_49.5248.5248.3	3	4.031	0.434	1	1839.4	53.333336	K.ADWSSLSRDEKVQLYR.I
COX41_MOUSE	MK_SCX_55.4017.4017.3	3	4.132	0.518	1	740.9	36.842106	R.AHGSVVKSEDYAFPTYADRR.D
COX5A_MOUSE	MK_SCX_19.4914.4914.2	2	4.747	0.614	1	1970	85.71429	K.GM*NTLVGYDLVPEPK.I
COX5A_MOUSE	MK_SCX_19.5477.5477.2	2	4.773	0.564	1	1477.8	82.14286	K.GMNTLVGYDLVPEPK.I

COX5A_MOUSE	MK_SCX_31.7466.7466.2	2	5.863	0.563	1	1405.3	80	R.WVTYFNKPDIDAWELR.K
COX5A_MOUSE	MK_SCX_31.7627.7627.3	3	3.53	0.54	1	677.1	46.666668	R.WVTYFNKPDIDAWELR.K
COX5A_MOUSE	MK_SCX_33.4902.4902.2	2	3.937	0.418	1	1361.3	73.333336	R.KGM*NTLVGYDLVPEPK.I
COX5A_MOUSE	MK_SCX_33.4915.4915.3	3	4.263	0.464	1	1619.8	55	R.KGM*NTLVGYDLVPEPK.I
COX5A_MOUSE	MK_SCX_33.5341.5341.2	2	4.527	0.58	1	1442.9	76.666664	R.KGMNTLVGYDLVPEPK.I
COX5A_MOUSE	MK_SCX_33.5388.5388.3	3	4.054	0.492	1	1055.1	46.666668	R.KGMNTLVGYDLVPEPK.I
COX5A_MOUSE	MK_SCX_46.6643.6643.2	2	5.576	0.608	1	1416.8	71.875	R.WVTYFNKPDIDAWELRK.G
COX5A_MOUSE	MK_SCX_47.6803.6803.3	3	4.912	0.502	1	749.7	45.3125	R.WVTYFNKPDIDAWELRK.G
COX5B_MOUSE	MK_SCX_18.3806.3806.2	2	6.572	0.626	1	939.1	71.05263	R.SM*ASGGGVPTDEEQATGLER.E
COX5B_MOUSE	MK_SCX_18.4073.4073.2	2	5.346	0.622	1	1116.9	71.05263	R.SMASGGGVPTDEEQATGLER.E
COX5B_MOUSE	MK_SCX_20_1.4274.4274.2	2	3.162	0.475	1	865.1	80	K.GLDPYNM*LPPK.A
COX5B_MOUSE	MK_SCX_21.5186.5186.2	2	2.736	0.355	1	660.4	75	K.GLDPYNMLPPK.A
COX5B_MOUSE	MK_SCX_2201.2924.2924.2	2	2.647	0.145	1	377.7	85.71429	R.EIMIAAQK.G
COX5B_MOUSE	MK_SCX_29.3829.3829.3	3	3.377	0.225	1	961.6	42.647057	K.AASGTKEDPNLVPSISNK.R
COX5B_MOUSE	MK_SCX_29.4982.4982.2	2	4.888	0.49	1	712.5	68.75	K.GLDPYNMLPPKAASGTK.E
COX5B_MOUSE	MK_SCX_31.6547.6547.3	3	4.33	0.51	1	680.8	26.785713	K.GLDPYNMLPPKAASGTKEDPNLVPSISNK.R
COX5B_MOUSE	MK_SCX_46.3445.3445.3	3	4.019	0.405	1	791.6	41.666664	K.AASGTKEDPNLVPSISNKR.I
COX5B_MOUSE	MK_SCX_46.3447.3447.2	2	4.897	0.518	1	895.6	63.88889	K.AASGTKEDPNLVPSISNKR.I
COX5B_MOUSE	MK_SCX_50.627.627.2	2	2.07	0.192	1	543.4	81.25	R.CPNCGTHYK.L
COX6C_MOUSE	MK_SCX_35.3572.3572.3	3	3.456	0.305	1	405.7	45.454548	R.NYDSM*KDFEEMR.K
COX6C_MOUSE	MK_SCX_35.4744.4744.3	3	3.027	0.458	1	362.5	45.454548	R.NYDSMKDFEEMR.K
COX6C_MOUSE	MK_SCX_36.3716.3716.2	2	4.264	0.568	1	580.1	81.818184	R.NYDSMKDFEEM*R.K
COX6C_MOUSE	MK_SCX_36.3727.3727.3	3	3.21	0.403	1	326.8	43.18182	R.NYDSMKDFEEM*R.K
COX6C_MOUSE	MK_SCX_36.4249.4249.2	2	4.436	0.545	1	1540.1	81.818184	R.NYDSMKDFEEMR.K
COX6C_MOUSE	MK_SCX_51.3852.3852.2	2	4.088	0.468	1	913.1	70.83333	R.NYDSMKDFEEMRK.A
COX6C_MOUSE	MK_SCX_58.8947.8947.2	2	2.319	0.317	1	633.7	81.25	R.KKAYAEFYR.N
COX7C_MOUSE	MK_SCX_42.5017.5017.2	2	3.008	0.368	1	378.8	75	K.NLPFSVENKWR.L
COX7C_MOUSE	MK_SCX_50.4009.4009.3	3	5.442	0.584	1	1380.2	48.52941	R.SHYEEGPGKNLPFSVENK.W
COX7C_MOUSE	MK_SCX_50.4010.4010.2	2	5.895	0.557	1	2225.3	73.52941	R.SHYEEGPGKNLPFSVENK.W
COX7C_MOUSE	MK_SCX_56.4871.4871.3	3	5.316	0.561	1	1399.6	47.368423	R.SHYEEGPGKNLPFSVENKWR.L
COX7R_MOUSE	MK_SCX_21.7956.7956.3	3	4.382	0.523	1	832.7	29.032257	K.LAGAWASEAYTPQGLKPVSTEAPPIIFATPTK.L
COX7R_MOUSE	MK_SCX_39.4729.4729.2	2	2.279	0.139	1	436.6	54.545456	K.FFQKADGFHLKR.G
CP24A_MOUSE	MK_SCX_18.3051.3051.2	2	2.231	0.155	1	533.8	53.846157	K.ESMRLTPSVPFTTR.T
CP24A_MOUSE	MK_SCX_26.13631.13631.3	3	3.106	0.15	1	313.4	27.631578	K.WSFESICLVLYEKRFGLLQK.D
CP4B1_MOUSE	MK_SCX_27.4142.4142.3	3	3.113	0.389	1	905.7	50	R.DESGIKLSDADLR.A
CP4B1_MOUSE	MK_SCX_35.11504.11504.3	3	3.979	0.457	1	1458	42.105263	R.FEFSPDPSKIPIKVPQLILR.S
CP4B1_MOUSE	MK_SCX_35.14745.14745.2	2	3.158	0.434	1	1474.3	86.36364	R.HLDFLDILLGAR.D
CP4B1_MOUSE	MK_SCX_52.6702.6702.3	3	3.361	0.299	1	972.2	39.285713	R.ALDSFPGPPKHWLFGHALEIQK.T
CP4B1_MOUSE	MK_SCX_54.8589.8589.2	2	3.438	0.398	1	853.1	66.66667	R.RHLDFLDILLGAR.D
CPGL1_MOUSE	MK_SCX_16.8356.8356.2	2	3.654	0.452	1	869.7	68.42105	R.LVPDM*IPEVVSEQVSSYLSK.K
CPGL1_MOUSE	MK_SCX_16.8430.8430.3	3	4.83	0.444	1	1382	43.421055	R.LVPDM*IPEVVSEQVSSYLSK.K
CPGL1_MOUSE	MK_SCX_16.8788.8788.2	2	4.779	0.515	1	1349.6	73.68421	R.LVPDMIPEVVSEQVSSYLSK.K
CPGL1_MOUSE	MK_SCX_16.8827.8827.3	3	4.543	0.549	1	1193	44.736843	R.LVPDMIPEVVSEQVSSYLSK.K
CPGL1_MOUSE	MK_SCX_17.7385.7385.2	2	4.733	0.496	1	960.1	76.666664	K.LPDGSEIPLPPILLGK.L
CPGL1_MOUSE	MK_SCX_18.5426.5426.2	2	3.724	0.563	1	420.7	59.375	R.EGGSIPVTLTFQEATGK.N
CPGL1_MOUSE	MK_SCX_20_1.4885.4885.2	2	2.99	0.331	1	1172.9	85	K.TVFGVEPDLTR.E
CPGL1_MOUSE	MK_SCX_20_1.5105.5105.2	2	4.026	0.531	1	1953.7	86.36364	R.LGGSVELVDIGK.Q
CPGL1_MOUSE	MK_SCX_2201.3696.3696.2	2	4.341	0.57	1	2191.5	95	R.MMEVAAADVQR.L
CPGL1_MOUSE	MK_SCX_24.10334.10334.2	2	3.937	0.457	1	709.2	62.5	R.LVPDMIPEVVSEQVSSYLSKK.F
CPGL1_MOUSE	MK_SCX_27.4401.4401.2	2	5.83	0.565	1	1205.4	71.05263	K.NVMLLPVGSADDGAHSQNEK.L
CPGL1_MOUSE	MK_SCX_27.7500.7500.2	2	5.192	0.586	1	1885	65	R.GSTDDKGPVAGWMNALEAYQK.T

CPGL1_MOUSE	MK_SCX_27.7587.7587.3	3	4.838	0.589	1	963	36.25	R.GSTDDKGPVAGWMNALEAYQK.T
CPGL1_MOUSE	MK_SCX_27.8132.8132.2	2	3.42	0.429	1	828.1	58.823532	K.QKLPDGSEIPLPPILLGK.L
CPGL1_MOUSE	MK_SCX_27.8179.8179.3	3	3.933	0.354	1	624	38.235294	K.QKLPDGSEIPLPPILLGK.L
CPGL1_MOUSE	MK_SCX_29.9230.9230.3	3	3.722	0.376	1	723.8	27.586206	R.LGGSVELVDIGKQKLPDGSEIPLPPILLGK.L
CPGL1_MOUSE	MK_SCX_31.7110.7110.3	3	3.655	0.422	1	445.6	37.5	K.LAEWVAIQSVSAWPEKR.G
CPGL1_MOUSE	MK_SCX_32.6729.6729.3	3	4.714	0.542	1	1800.7	50	R.YPSLSLHGIEGAFSGSGAK.T
CPGL1_MOUSE	MK_SCX_41.3395.3395.3	3	3.983	0.32	1	1670.3	54.545456	R.RMMEVAAADVQR.L
CPGL1_MOUSE	MK_SCX_41.3398.3398.2	2	3.389	0.397	1	1204.5	90.909096	R.RMMEVAAADVQR.L
CPGL1_MOUSE	MK_SCX_50.6351.6351.3	3	5.899	0.59	1	2082.9	52.941177	K.KLAEWVAIQSVSAWPEKR.G
CPGL1_MOUSE	MK_SCX_51.7319.7319.3	3	4.743	0.575	1	1841.9	43.75	R.WRYPSLSLHGIEGAFSGSGAK.T
CPGL1_MOUSE	MK_SCX_51.7395.7395.2	2	5.7	0.631	1	2600.2	72.5	R.WRYPSLSLHGIEGAFSGSGAK.T
CPNS1_MOUSE	MK_SCX_16.7893.7893.3	3	3.906	0.412	1	849.5	32.608696	R.ILGGVISAISEAAAQYNPEPPPPR.S
CPNS1_MOUSE	MK_SCX_38.8587.8587.3	3	3.28	0.265	1	416.6	27.272728	K.LFVQLAGDDMEVSATELM*NILNK.V
CPNS1_MOUSE	MK_SCX_39.3320.3320.2	2	4.931	0.54	1	2624.3	85.71429	R.SHYSNIEANESEEVR.Q
CPNS1_MOUSE	MK_SCX_41.3203.3203.3	3	4.666	0.361	1	778.6	41.07143	R.SHYSNIEANESEEVR.Q
CPSF4_MOUSE	MK_SCX_16.5957.5957.2	2	3.664	0.514	1	419.9	42.857143	R.FELPMGTTEQPPLPQQTQPPTK.R
CPSF5_MOUSE	MK_SCX_15.10388.10388.3	3	3.104	0.185	1	386.3	23.214285	K.LVAAPLFELYDNAPGYGPIISSLPQLLSR.F
CPSF5_MOUSE	MK_SCX_28.4571.4571.3	3	3.6	0.417	1	704.4	39.705883	K.LPGGELNPGEDEVEGLKR.L
CPSF5_MOUSE	MK_SCX_46.4935.4935.3	3	4.427	0.43	1	440.5	30.263159	R.GVNQFGNKYIQQTKPLTLER.T
CPSF6_MOUSE	MK_SCX_15.10363.10363.2	2	5.877	0.727	1	3191.8	64.58333	R.AVSDASAGDYGSAIETLVTAISLIK.Q
CPT2_MOUSE	MK_SCX_14.4234.4234.2	2	5.465	0.628	1	998	54.166668	R.DSTQTPAIAPQSQPAATDSSVSVQK.L
CPT2_MOUSE	MK_SCX_15.8378.8378.2	2	3.546	0.365	1	534.9	47.058823	R.DSVVLNFNPFMAFNPDPK.S
CPT2_MOUSE	MK_SCX_19.5105.5105.2	2	3.422	0.561	1	932.7	67.85714	R.GVTLPELYQDPAYQR.I
CPT2_MOUSE	MK_SCX_21.5411.5411.2	2	2.134	0.346	1	352.1	65	K.TLTIDAIQFQR.G
CPT2_MOUSE	MK_SCX_41.8532.8532.3	3	3.349	0.426	1	564.5	32.894737	R.EFLHCVQKCLEDMFDALEGK.A
CRADD_MOUSE	MK_SCX_10.8768.8768.2	2	2.526	0.177	1	468.8	50	R.QRFGKQATFLSLHK.G
CRADD_MOUSE	MK_SCX_19.9658.9658.2	2	2.199	0.141	1	556.4	56.666668	K.AFDTFLDSLQEFPWVR.E
CREB1_MOUSE	MK_SCX_15.6066.6066.2	2	5.278	0.672	1	932	44.642857	R.TAPTSTIAPGVVMASSPALPTQPAEEAAR.K
CRIP1_MOUSE	MK_SCX_56.3814.3814.3	3	6.248	0.602	1	1760.3	34.25926	K.TLTSGGHAEHEGKPYCNHPCYSAMFGPK.G
CRIP2_MOUSE	MK_SCX_21.7170.7170.3	3	6.773	0.66	1	1366.1	33.064518	K.GVNIGGAGSYIYEKPQTEAPQVTGPIEVPVVR.T
CRIP2_MOUSE	MK_SCX_21.7181.7181.2	2	4.614	0.691	1	541.8	38.709675	K.GVNIGGAGSYIYEKPQTEAPQVTGPIEVPVVR.T
CRIP2_MOUSE	MK_SCX_52.3722.3722.3	3	3.137	0.451	1	309.6	26.31579	K.ASSVTTFTGEPNMCPRCNKR.V
CRIP2_MOUSE	MK_SCX_56.4978.4978.3	3	4.051	0.502	1	455.9	28.703705	K.TLTPGGHAEHDGQPYCHKPCYGILFGPK.G
CRK_MOUSE	MK_SCX_16.5073.5073.2	2	4.457	0.602	1	1963.7	76.47059	R.DSSTSPGDYVLSVSENSR.V
CRK_MOUSE	MK_SCX_16.7263.7263.2	2	3.213	0.418	1	899.4	60.000004	R.ALFDFNGNDEEDLPFK.K
CRK_MOUSE	MK_SCX_17.8314.8314.2	2	5.568	0.545	1	1400	78.125	R.IGDQEFDSLPALLEFYK.I
CRK_MOUSE	MK_SCX_31.5678.5678.2	2	4.553	0.541	1	2013.1	75	K.IHYLDTTTLIEPVAR.S
CRK_MOUSE	MK_SCX_41.4478.4478.2	2	3.033	0.322	1	1014.9	80	K.RGMIPVPYVEK.Y
CRKL_MOUSE	MK_SCX_16.6397.6397.2	2	4.895	0.672	1	765.2	52.083332	R.YPSPPVGSVSAPNLPTAEENLEYVR.T
CRKL_MOUSE	MK_SCX_18.6540.6540.2	2	3.69	0.464	1	360.9	50	R.TLYDFPGNDAEDLPFK.K
CRKL_MOUSE	MK_SCX_20_1.4708.4708.2	2	2.853	0.221	1	435.3	70	R.VGM*IPVPYVEK.L
CRKL_MOUSE	MK_SCX_25.4673.4673.2	2	2.982	0.53	1	969.1	77.77778	R.SAWYMGPVTR.Q
CRKL_MOUSE	MK_SCX_26.7898.7898.3	3	3.509	0.234	1	662.3	39.0625	K.IGDQEFDHLPALLEFYK.I
CRKL_MOUSE	MK_SCX_31.5366.5366.2	2	4.202	0.371	1	2047.7	75	K.IHYLDTTTLIEPAPR.Y
CRKL_MOUSE	MK_SCX_40.11969.11969.3	3	3.992	0.395	1	674	36.11111	R.FKIGDQEFDHLPALLEFYK.I
CRLS1_MOUSE	MK_SCX_20_1.3057.3057.2	2	4.045	0.542	1	1156.1	68.42105	K.AAPEPAAGGGGAAAQAPSAR.W
CRLS1_MOUSE	MK_SCX_20_1.3064.3064.3	3	4.078	0.474	1	1189.6	42.105263	K.AAPEPAAGGGGAAAQAPSAR.W
CROP_MOUSE	MK_SCX_2201.6426.6426.2	2	3.709	0.301	1	1441.9	85	R.YLQSLLAEVER.R
CRTC_MOUSE	MK_SCX_15.11555.11555.3	3	5.852	0.53	1	2270.1	36.607143	K.SGTIFDNFLITNDEAYAEEFGNETWGVTK.A
CRTC_MOUSE	MK_SCX_15.12315.12315.2	2	4.654	0.642	1	802.3	35.714287	K.SGTIFDNFLITNDEAYAEEFGNETWGVTK.A
CRTC_MOUSE	MK_SCX_16.6604.6604.2	2	5.256	0.653	1	792.6	60.000004	K.IDNSQVESGSLEDDWDFLPPK.K

CRTC_MOUSE	MK_SCX_21.4786.4786.2	2	3.914	0.456	1	1406.1	81.818184	K.EQFLDGDAWTNR.W
CRTC_MOUSE	MK_SCX_24.6288.6288.2	2	4.847	0.577	1	1357.6	54.761906	K.IDNSQVESGSLEDDWDFLPPKK.I
CRTC_MOUSE	MK_SCX_24.6386.6386.3	3	5.311	0.596	1	1311.1	39.285713	K.IDNSQVESGSLEDDWDFLPPKK.I
CRTC_MOUSE	MK_SCX_25.6691.6691.3	3	4.762	0.457	1	1754.9	40.217392	K.KPEDWDEEMDGEWEPPVIQNPEYK.G
CRTC_MOUSE	MK_SCX_25.6748.6748.2	2	5.521	0.721	1	1001.6	52.173912	K.KPEDWDEEMDGEWEPPVIQNPEYK.G
CRTC_MOUSE	MK_SCX_30.6286.6286.2	2	3.707	0.517	1	1153.5	62.5	K.EQFLDGDAWTNRWVESK.H
CRTC_MOUSE	MK_SCX_37.4438.4438.3	3	3.26	0.319	1	942.2	50	K.SDFGKFVLSSGK.F
CRTC_MOUSE	MK_SCX_37.4439.4439.2	2	3.868	0.502	1	1128.2	86.36364	K.SDFGKFVLSSGK.F
CRTC_MOUSE	MK_SCX_45.3831.3831.3	3	4.856	0.497	1	930.8	50	K.FYGDLEKDKGLQTSQDAR.F
CRTC_MOUSE	MK_SCX_45.3882.3882.2	2	6.311	0.678	1	1837.5	70.588234	K.FYGDLEKDKGLQTSQDAR.F
CRTC_MOUSE	MK_SCX_46.4974.4974.3	3	3.815	0.516	1	758.4	46.666668	K.FVLSSGKFYGDLEKDK.G
CRTC_MOUSE	MK_SCX_46.4992.4992.2	2	5.183	0.421	1	1442.4	73.333336	K.FVLSSGKFYGDLEKDK.G
CRTC_MOUSE	MK_SCX_47.3502.3502.2	2	4.485	0.511	1	1673.3	75	K.IKDPDAAKPEDWDER.A
CRTC_MOUSE	MK_SCX_47.3548.3548.3	3	3.689	0.454	1	1093.7	55.35714	K.IKDPDAAKPEDWDER.A
CRTC_MOUSE	MK_SCX_52.4350.4350.3	3	6.407	0.572	1	1249	37	R.AKIDDPTDSKPEDWDKPEHIPDPDAK.K
CRTC_MOUSE	MK_SCX_56.3154.3154.3	3	3.596	0.404	1	1118.7	48.333332	K.KIKDPDAAKPEDWDER.A
CRTC_MOUSE	MK_SCX_59.12756.12756.2	2	3.255	0.488	1	662	65.38461	K.HKSDFGKFVLSSGK.F
CRYAB_MOUSE	MK_SCX_21.6226.6226.2	2	3.958	0.562	1	1268	79.16667	R.APSWIDTGLSEMR.L
CRYAB_MOUSE	MK_SCX_24.4453.4453.2	2	2.892	0.248	1	1171	100	R.FSVNLDVK.H
CRYL1_MOUSE	MK_SCX_13.5094.5094.2	2	2.611	0.315	1	414.8	61.538464	R.VILSSSSSCLLPSK.L
CRYL1_MOUSE	MK_SCX_15.7267.7267.2	2	4.251	0.267	1	1159.8	56.81818	R.LVEEEIVSPSDLDLVM*SDGLGMR.Y
CRYL1_MOUSE	MK_SCX_15.7684.7684.2	2	4.413	0.306	1	1120.1	54.545456	R.LVEEEIVSPSDLDLVMSDGLGM*R.Y
CRYL1_MOUSE	MK_SCX_15.8119.8119.3	3	4.537	0.419	1	915.5	32.954548	R.LVEEEIVSPSDLDLVMSDGLGMR.Y
CRYL1_MOUSE	MK_SCX_15.8123.8123.2	2	5.51	0.671	1	1450.4	61.363636	R.LVEEEIVSPSDLDLVMSDGLGMR.Y
CRYL1_MOUSE	MK_SCX_18.7198.7198.2	2	6.232	0.531	1	3037.8	78.125	K.LYDIEQQQITDALENIR.K
CRYL1_MOUSE	MK_SCX_2201.9080.9080.3	3	3.891	0.515	1	465.5	24.193548	R.QLSLISGCGNLAEAVEGAVHIQECVPENLELK.K
CRYL1_MOUSE	MK_SCX_24.15132.15132.2	2	3.287	0.364	1	980.4	81.818184	R.SWAMLFASGGFK.V
CRYL1_MOUSE	MK_SCX_27.7270.7270.2	2	5.654	0.461	1	1449	67.64706	K.LYDIEQQQITDALENIRK.E
CRYL1_MOUSE	MK_SCX_27.7379.7379.3	3	3.546	0.2	1	729.5	38.235294	K.LYDIEQQQITDALENIRK.E
CRYL1_MOUSE	MK_SCX_30.7766.7766.3	3	3.249	0.127	1	1046.3	36.11111	K.VKLYDIEQQQITDALENIR.K
CRYL1_MOUSE	MK_SCX_32.5893.5893.3	3	3.887	0.498	1	1246.4	45.833336	K.HVLSTFGPVPEFSGATVER.V
CRYL1_MOUSE	MK_SCX_32.5939.5939.2	2	5.309	0.677	1	2062.1	75	K.HVLSTFGPVPEFSGATVER.V
CRYM_MOUSE	MK_SCX_18.7104.7104.2	2	5.742	0.61	1	1012	72.22222	R.GFLGVMPAYSAAEDALTTK.L
CRYM_MOUSE	MK_SCX_18.9231.9231.1	1	2.989	0.539	1	418.3	55.88235	R.SSSLLIPPLEAALANFSK.G
CRYM_MOUSE	MK_SCX_18.9286.9286.2	2	4.253	0.504	1	632.7	73.52941	R.SSSLLIPPLEAALANFSK.G
CRYM_MOUSE	MK_SCX_20_1.6135.6135.2	2	4.287	0.391	1	2226.5	87.5	K.SLGM*AVEDLVAAK.L
CRYM_MOUSE	MK_SCX_2201.3338.3338.2	2	2.489	0.266	1	501.5	87.5	R.QAVLYVDSR.E
CRYM_MOUSE	MK_SCX_31.5484.5484.3	3	4.312	0.528	1	1077.1	46.42857	R.APAFLSAEEVQDHLR.S
CRYM_MOUSE	MK_SCX_51.6496.6496.2	2	4.06	0.666	1	628.7	52.499996	K.HRGFLGVMPAYSAAEDALTTK.L
CRYM_MOUSE	MK_SCX_52.4843.4843.3	3	6.424	0.506	1	3495.1	60.000004	K.RAPAFLSAEEVQDHLR.S
CS010_MOUSE	MK_SCX_24.5196.5196.2	2	2.786	0.51	1	1069.3	81.25	K.SYLYFTQFK.A
CS010_MOUSE	MK_SCX_28.4278.4278.2	2	4.032	0.451	1	768.7	67.85714	R.ESDVPLKSEEFEVTK.T
CSAD_MOUSE	MK_SCX_23.4907.4907.3	3	3.154	0.177	1	462.8	30.952381	K.QLLDLELQSQGESREQILERCR.T
CSAD_MOUSE	MK_SCX_29.8135.8135.3	3	4.229	0.529	1	1679	48.4375	R.FFNQLFSGLDPHALAGR.I
CSAD_MOUSE	MK_SCX_43.4034.4034.2	2	2.895	0.354	1	1077.4	83.33333	R.RIDQAFALTR.Y
CSDE1_MOUSE	MK_SCX_2201.3235.3235.2	2	3.393	0.431	1	1064.2	77.27273	R.GPDNSMGFGAER.K
CSK21_MOUSE	MK_SCX_2201.8519.8519.3	3	3.569	0.378	1	982.5	36.25	K.VLGTEDLYDYIDKYNIELDPR.F
CSK21_MOUSE	MK_SCX_27.7483.7483.3	3	3.466	0.444	1	767	44.11765	R.GGPNIITLADIVKDPVSR.T
CSK21_MOUSE	MK_SCX_27.7705.7705.2	2	4.034	0.436	1	1013.5	58.823532	R.GGPNIITLADIVKDPVSR.T
CSK21_MOUSE	MK_SCX_28.7651.7651.3	3	3.272	0.434	1	537.8	34.72222	R.LIDWGLAEFYHPGQEYNVR.V
CSK21_MOUSE	MK_SCX_42.6201.6201.3	3	4.199	0.469	1	1667.2	43.421055	R.FVHSENQHLVSPEALDFLDK.L

CSK22_MOUSE	MK_SCX_34.5762.5762.2	2	3.608	0.55	1	708.5	75	R.HLVSPEALDLLDK.L
CSK22_MOUSE	MK_SCX_46.8633.8633.3	3	3.937	0.333	1	1306.2	51.666664	R.HLVSPEALDLLDKLLR.Y
CSKI1_MOUSE	MK_SCX_15.10925.10925.2	2	2.408	0.401	1	611.6	60.714287	K.HKEAIGPDGEVVNRR.R
CSKI1_MOUSE	MK_SCX_35.7119.7119.2	2	2.007	0.131	1	505.6	75	R.RVGRSHSVR.A
CSN4_MOUSE	MK_SCX_17.9388.9388.2	2	3.051	0.268	1	448.5	47.368423	K.LYNNITFEELGALLEIPAAK.A
CSN4_MOUSE	MK_SCX_18.6745.6745.2	2	2.782	0.259	1	633.4	53.333336	K.QYNVDYKLETYLKIAR.L
CSN4_MOUSE	MK_SCX_2201.3218.3218.2	2	2.834	0.433	1	581.4	83.33333	K.IASQMITEGR.M
CSRP1_MOUSE	MK_SCX_29.4547.4547.2	2	5.303	0.603	1	1340.1	52.380955	K.GYGYGQGAGTLSTDKGESLGIK.H
CSRP1_MOUSE	MK_SCX_29.4630.4630.3	3	4.129	0.541	1	1268.6	41.666664	K.GYGYGQGAGTLSTDKGESLGIK.H
CSRP1_MOUSE	MK_SCX_40.4720.4720.3	3	4.732	0.425	1	1906.5	41.666664	K.GLESTTLADKDGEIYCKGCYAK.N
CSRP1_MOUSE	MK_SCX_40.4738.4738.2	2	3.422	0.229	1	813.9	45.238094	K.GLESTTLADKDGEIYCKGCYAK.N
CSRP1_MOUSE	MK_SCX_41.4755.4755.3	3	4.219	0.497	1	602.7	35.714287	K.NLDSTTVAVHGEEIYCKSCYGK.K
CSRP2_MOUSE	MK_SCX_23.3898.3898.2	2	5.038	0.588	1	1834.9	78.57143	K.GYGYGQGAGTLNMDR.G
CSRP2_MOUSE	MK_SCX_42.4674.4674.3	3	4.704	0.47	1	588.5	32.142857	K.SLESTTLTEKEGEIYCKGCYAK.N
CSRP2_MOUSE	MK_SCX_54.3001.3001.3	3	3.29	0.492	1	525.2	40	R.LGIKPESAQPHRPTTNPNTSK.F
CSTF2_MOUSE	MK_SCX_15.6132.6132.2	2	5.233	0.616	1	1064	44.444447	K.SLGTGAPVIESPYGESISPEDAPESISK.A
CSTF3_MOUSE	MK_SCX_25.5733.5733.2	2	2.068	0.221	1	334.6	55	R.FWKLYIEAEIK.A
CSTF3_MOUSE	MK_SCX_27.6141.6141.2	2	2.258	0.18	1	344.5	50	K.SVAFKIFELGLKK.Y
CSTFT_MOUSE	MK_SCX_14.7070.7070.2	2	4.321	0.598	1	467.7	38.88889	K.SLGPAAPIIDSPYGDPIDPEDAPESITR.A
CSTFT_MOUSE	MK_SCX_18.3662.3662.2	2	4.293	0.56	1	977.5	54.545456	K.QGGGQPSSFSPGQSQVTPQDQEK.A
CT077_MOUSE	MK_SCX_18.6184.6184.2	2	3.729	0.516	1	641.8	60.000004	K.IASLPQEVQDVSLLEK.I
CT077_MOUSE	MK_SCX_19.4254.4254.2	2	5.19	0.594	1	2971.2	80	K.ALQDLENAASGDATVR.Q
CT077_MOUSE	MK_SCX_25.4890.4890.2	2	2.69	0.171	1	747.6	92.85714	R.LLNIWQER.S
CT116_MOUSE	MK_SCX_18.10156.10156.2	2	4.116	0.475	1	1587.1	75	K.FIYITPEELAAVANFIR.Q
CTGE5_MOUSE	MK_SCX_30.3515.3515.3	3	3.898	0.394	1	1188.6	44.11765	R.M*TAHPPPGQPYSDPALQR.Q
CTGE5_MOUSE	MK_SCX_30.3687.3687.3	3	3.614	0.406	1	940	44.11765	R.MTAHPPPGQPYSDPALQR.Q
CTGE5_MOUSE	MK_SCX_49.3974.3974.3	3	5.334	0.53	1	1565	48.684208	R.LKGAIKDALNENSQLQESQK.Q
CTN1_MOUSE	MK_SCX_14.4003.4003.2	2	2.784	0.194	1	794.5	62.5	R.NAGNEQDLGIQYK.A
CTN1_MOUSE	MK_SCX_15.5276.5276.2	2	2.148	0.125	1	306.3	36.11111	R.SRTSVQTEDDQLIAGQSAR.A
CTN1_MOUSE	MK_SCX_19.3767.3767.2	2	3.545	0.374	1	610.8	59.375	R.TSVQTEDDQLIAGQSAR.A
CTN1_MOUSE	MK_SCX_20_1.6296.6296.2	2	2.35	0.132	1	300.9	46.42857	K.WDPKSLEIRTLAVER.L
CTN1_MOUSE	MK_SCX_35.3583.3583.3	3	4.509	0.48	1	1499.3	45.833336	R.SRTSVQTEDDQLIAGQSAR.A
CTN1_MOUSE	MK_SCX_41.5046.5046.2	2	2.613	0.542	1	465.7	63.636364	K.HVNPVQALSEFK.A
CTN1_MOUSE	MK_SCX_41.6264.6264.3	3	3.17	0.252	1	751.9	35	R.TIADHCPDSACKQDLLAYLQR.I
CTN1_MOUSE	MK_SCX_47.4746.4746.3	3	3.7	0.576	1	509.5	46.42857	K.ALKPEVDKLNIMAAK.R
CTND1_MOUSE	MK_SCX_15.7226.7226.2	2	4.995	0.633	1	792.4	42.857143	R.TVQPVPMGPDGLPVDASAVSNNYIQTLGR.D
CTND1_MOUSE	MK_SCX_15.7234.7234.3	3	6.053	0.53	1	1208.1	33.035713	R.TVQPVPMGPDGLPVDASAVSNNYIQTLGR.D
CTND1_MOUSE	MK_SCX_17.4210.4210.2	2	5.359	0.679	1	2521.2	72.22222	R.SM*GYDDLDYGM*M*SDYGTAR.R
CTND1_MOUSE	MK_SCX_17.5733.5733.2	2	3.852	0.583	1	953.5	58.333332	R.SMGYDDLDYGMMSDYGTAR.R
CTND1_MOUSE	MK_SCX_31.4467.4467.3	3	3.697	0.445	1	817.5	45.3125	R.SQSSHSYDDSTLPLIDR.N
CTND1_MOUSE	MK_SCX_34.3672.3672.3	3	4.781	0.474	1	1207.8	47.058823	R.HYEDGYPGGSDNYGSLSR.V
CTND1_MOUSE	MK_SCX_36.3786.3786.2	2	3.438	0.499	1	862.3	83.33333	R.FHPEPYGLEDDQR.S
CTND1_MOUSE	MK_SCX_43.3585.3585.3	3	3.241	0.41	1	1196.2	52.499996	R.NFHYPPDGYGR.H
CUBN_MOUSE	MK_SCX_17.11501.11501.2	2	4.027	0.581	1	956.5	52.499996	R.MTTEEGNLVFLTSSAQNIEFR.T
CUBN_MOUSE	MK_SCX_19.4227.4227.2	2	5.368	0.462	1	1435	66.66667	R.SPSANPM*QISSTDNELAIR.F
CUBN_MOUSE	MK_SCX_19.4734.4734.2	2	5.694	0.573	1	1217.4	63.88889	R.SPSANPMQISSTDNELAIR.F
CUBN_MOUSE	MK_SCX_20_1.4103.4103.2	2	4.025	0.383	1	1401.8	85	R.DFQSLQQNVER.K
CUBN_MOUSE	MK_SCX_21.4740.4740.2	2	3.427	0.599	1	435.7	53.125	R.QQPPNSITSSGNSLFVR.F
CUTA_MOUSE	MK_SCX_20_1.7530.7530.2	2	3.674	0.456	1	497.1	65.38461	K.TQSSLVPALTEFVR.S
CUTL1_MOUSE	MK_SCX_16.3294.3294.2	2	3.253	0.44	1	1420.6	65	K.TAEPVQTSSTSSSGNSDDAIR.S
CUTL1_MOUSE	MK_SCX_25.4079.4079.3	3	3.804	0.315	1	635.8	32.608696	K.SQGGLAEVAAAPADREEATQPAEK.A

CUTL1_MOUSE	MK_SCX_45.2863.2863.3	3	3.074	0.312	1	767.6	39.473686	R.NLTSSEETKADETTASGKER.A
CX033_MOUSE	MK_SCX_20_1.4051.4051.2	2	4.12	0.548	1	1001	67.85714	K.SESTSGTTQFIPDPK.L
CX033_MOUSE	MK_SCX_20_1.7626.7626.2	2	4.853	0.547	1	2516.3	80	K.M*GVITASGLAGLLSAR.K
CX033_MOUSE	MK_SCX_20_1.8354.8354.2	2	5.176	0.336	1	2067.3	73.333336	K.MGVITASGLAGLLSAR.K
CX033_MOUSE	MK_SCX_2201.3053.3053.2	2	4.136	0.467	1	1029.9	90	K.YVEEQPGNLQR.G
CX033_MOUSE	MK_SCX_2201.5034.5034.2	2	4.334	0.46	1	1382.6	87.5	K.AYATSQQIFQAIK.S
CX033_MOUSE	MK_SCX_31.4306.4306.3	3	3.764	0.54	1	687.4	52.272724	R.SDEIHASLPDLK.H
CX033_MOUSE	MK_SCX_31.4331.4331.2	2	2.445	0.349	1	371.6	63.636364	R.SDEIHASLPDLK.H
CX033_MOUSE	MK_SCX_41.5575.5575.3	3	3.485	0.388	1	595.7	34.72222	K.QLVRPDQLPIYTAPPLHSK.Y
CX033_MOUSE	MK_SCX_47.2915.2915.3	3	3.11	0.307	1	590.4	35.9375	K.SENESLPEPKEESKEGR.S
CX033_MOUSE	MK_SCX_53.2938.2938.3	3	4.772	0.461	1	1807.5	47.058823	K.LMDHGQSHPDDKDMYSTR.S
CX033_MOUSE	MK_SCX_53.5198.5198.3	3	5.094	0.576	1	779.9	30.208334	K.EEEPKKQLVRPDQLPIYTAPPLHSK.Y
CX033_MOUSE	MK_SCX_53.5236.5236.2	2	4.992	0.608	1	1361.5	63.15789	K.KQLVRPDQLPIYTAPPLHSK.Y
CX6A1_MOUSE	MK_SCX_16.10811.10811.2	2	4.207	0.442	1	739.4	57.5	K.ALTYFVALPGVGVSM*LNVFLK.S
CX6A1_MOUSE	MK_SCX_16.12493.12493.2	2	3.796	0.422	1	302.3	42.5	K.ALTYFVALPGVGVSMLNVFLK.S
CX6B1_MOUSE	MK_SCX_13.8149.8149.2	2	4.735	0.673	1	1832.1	80.769226	K.SLCPVSWVSAWDDR.I
CX6B1_MOUSE	MK_SCX_2201.2994.2994.2	2	2.464	0.223	1	763.3	81.25	R.IAEGTFPGK.I
CX6B1_MOUSE	MK_SCX_35.3645.3645.2	2	4.032	0.49	1	652	64.28571	K.TAPFDSRFPNQNQTK.N
CX7A2_MOUSE	MK_SCX_30.4644.4644.2	2	2.95	0.198	1	1852.6	79.16667	K.LFQEDNGMPVHLK.G
CX7A2_MOUSE	MK_SCX_33.4619.4619.3	3	3.691	0.31	1	1138.3	56.25	K.LFQEDNGMPVHLK.G
CY1_MOUSE	MK_SCX_14.9299.9299.3	3	5.013	0.484	1	766.2	25.641027	R.EGLYFNPYFPGQAIGM*APPIYTEVLEYDDGTPATMSQVAK.D
CY1_MOUSE	MK_SCX_14.9689.9689.3	3	4.235	0.556	1	413	23.076923	R.EGLYFNPYFPGQAIGMAPPIYTEVLEYDDGTPATMSQVAK.D
CY1_MOUSE	MK_SCX_17.5896.5896.2	2	2.72	0.366	1	549.2	73.333336	R.AANNGALPPDLSYIVR.A
CY1_MOUSE	MK_SCX_17.9205.9205.2	2	2.329	0.2	1	414.6	50	K.M*LLMMGLLLPLTYAM*K.R
CY1_MOUSE	MK_SCX_24.5442.5442.2	2	5.165	0.608	1	750	52.173912	K.ALAEEVEVQDGPNDDGEMFMRPGK.L
CY1_MOUSE	MK_SCX_24.5484.5484.3	3	6.328	0.554	1	1862.1	41.304348	K.ALAEEVEVQDGPNDDGEMFMRPGK.L
CY1_MOUSE	MK_SCX_30.4536.4536.2	2	3.82	0.518	1	428.6	56.666668	K.LSDYFPKPYPNPEAAR.A
CY1_MOUSE	MK_SCX_30.4698.4698.3	3	3.911	0.552	1	1162.9	48.333332	K.LSDYFPKPYPNPEAAR.A
CY1_MOUSE	MK_SCX_36.4391.4391.2	2	3.846	0.479	1	1635.7	77.27273	R.GLLSSLDHTSIR.R
CYB5_MOUSE	MK_SCX_2201.4056.4056.2	2	2.596	0.205	1	696.5	81.25	K.YYTLEEIQK.H
CYB5_MOUSE	MK_SCX_24.3757.3757.3	3	3.737	0.197	1	629.9	31.25	R.EQAGGDATENFEDVGHSTDAR.E
CYB5_MOUSE	MK_SCX_25.3755.3755.2	2	5.706	0.599	1	760.4	50	R.EQAGGDATENFEDVGHSTDAR.E
CYB5_MOUSE	MK_SCX_32.4105.4105.2	2	4.249	0.569	1	1911.8	86.36364	K.TYIIGELHPDDR.S
CYB5_MOUSE	MK_SCX_32.4191.4191.3	3	3.042	0.324	1	488.1	47.727272	K.TYIIGELHPDDR.S
CYB5_MOUSE	MK_SCX_33.3979.3979.2	2	4.347	0.418	1	758	79.16667	K.FLEEHPGGEEVLR.E
CYB5_MOUSE	MK_SCX_41.5445.5445.3	3	6.346	0.65	1	1252.9	32.575756	K.FLEEHPGGEEVLREQAGGDATENFEDVGHSTDAR.E
CYB5_MOUSE	MK_SCX_43.4922.4922.3	3	3.656	0.536	1	1071.9	43.333332	R.ELSKTYIIGELHPDDR.S
CYB5_MOUSE	MK_SCX_56.3299.3299.2	2	4.845	0.525	1	1332.8	76.92308	K.YYTLEEIQKHKDSK.S
CYB5B_MOUSE	MK_SCX_21.3798.3798.2	2	3.293	0.449	1	880.5	77.27273	K.VEGSEPSVTYYR.L
CYB5B_MOUSE	MK_SCX_30.7741.7741.3	3	7.002	0.61	1	2574	37.121212	R.FLSEHPGGEEVLLEQAGADATESFEDVGHSPDAR.E
CYB5B_MOUSE	MK_SCX_38.3961.3961.2	2	3.772	0.406	1	1017.3	75	R.NSAEETWM*VIHGR.V
CYB5B_MOUSE	MK_SCX_38.4375.4375.3	3	3.503	0.446	1	949.6	52.083332	R.NSAEETWMVIHGR.V
CYB5B_MOUSE	MK_SCX_38.4426.4426.2	2	4.429	0.388	1	2116.1	83.33333	R.NSAEETWMVIHGR.V
CYB5B_MOUSE	MK_SCX_45.4481.4481.2	2	3.465	0.513	1	814.4	71.42857	K.QYYIGDVHPSDLKPK.G
CYB5B_MOUSE	MK_SCX_52.4754.4754.3	3	6.043	0.543	1	1532	43.055553	R.EMLKQYYIGDVHPSDLKPK.G
CYB5B_MOUSE	MK_SCX_52.4800.4800.2	2	4.333	0.599	1	1051.6	55.555557	R.EMLKQYYIGDVHPSDLKPK.G
CYB5B_MOUSE	MK_SCX_55.3630.3630.3	3	3.168	0.444	1	766.1	46.153847	K.RNSAEETWM*VIHGR.V
CYB5B_MOUSE	MK_SCX_55.3977.3977.3	3	3.324	0.492	1	1029.4	44.230766	K.RNSAEETWMVIHGR.V
CYBP_MOUSE	MK_SCX_34.4467.4467.2	2	3.998	0.432	1	1147	73.07692	K.ISNYGWDQSDKFVK.I
CYBP_MOUSE	MK_SCX_51.4899.4899.3	3	5.453	0.55	1	1778.7	40	K.SQKKPELDNEKPAAVVAPLTTGYTVK.I
CYC_MOUSE	MK_SCX_18.7086.7086.2	2	5.085	0.522	1	2132	68.75	K.GITWGEDTLM*EYLENPK.K

CYC_MOUSE	MK_SCX_18.8150.8150.2	2	5.572	0.556	1	1439.8	68.75	K.GITWGEDTLMEYLENPK.K
CYC_MOUSE	MK_SCX_21.3443.3443.2	2	4.273	0.482	1	841.1	69.230774	K.TGQAAGFSYTDANK.N
CYC_MOUSE	MK_SCX_28.6914.6914.2	2	4.863	0.547	1	830.1	67.64706	K.GITWGEDTLM*EYLENPKK.Y
CYC_MOUSE	MK_SCX_28.7926.7926.2	2	5.365	0.538	1	2399.1	70.588234	K.GITWGEDTLMEYLENPKK.Y
CYC_MOUSE	MK_SCX_34.3031.3031.2	2	3.475	0.314	1	403.1	46.666668	K.TGQAAGFSYTDANKNK.G
CYC_MOUSE	MK_SCX_41.4378.4378.2	2	3.406	0.521	1	1155.4	90	K.TGPNLHGLFGR.K
CYC_MOUSE	MK_SCX_45.7598.7598.3	3	6.082	0.499	1	1007.2	42.105263	K.NKGITWGEDTLMEYLENPKK.Y
CYC_MOUSE	MK_SCX_51.2587.2587.3	3	4.75	0.498	1	1304.5	54.6875	R.KTGQAAGFSYTDANKNK.G
CYC_MOUSE	MK_SCX_55.3749.3749.2	2	3.515	0.549	1	1221	72.72727	K.TGPNLHGLFGRK.T
CYC_MOUSE	MK_SCX_55.3760.3760.3	3	3.314	0.452	1	443.5	47.727272	K.TGPNLHGLFGRK.T
CYTB_MOUSE	MK_SCX_2201.5321.5321.2	2	2.301	0.244	1	428.7	75	R.QIVAGTNLFIK.V
CYTB_MOUSE	MK_SCX_50.3714.3714.2	2	4.914	0.578	1	1919.8	75	K.SQLESKENQKFDVFK.A
CYTB_MOUSE	MK_SCX_52.4302.4302.3	3	3.757	0.267	1	759.5	39.772728	R.VFQPLPHENKPLTLSSYQTNKER.H
CYTC_MOUSE	MK_SCX_30.3559.3559.2	2	3.797	0.286	1	730.6	56.25	R.MLGAPEEADANEEGVRR.A
D3D2_MOUSE	MK_SCX_19.4400.4400.2	2	3.769	0.617	1	556.6	71.42857	R.VLVETEGPAGVAVMK.L
D3D2_MOUSE	MK_SCX_19.5655.5655.2	2	4.106	0.355	1	1307.5	79.16667	R.EADIQNFTSFISK.D
D3D2_MOUSE	MK_SCX_26.4231.4231.3	3	4.133	0.51	1	1979.4	56.666668	K.VGVVDEVVPEDQVHSK.A
D3D2_MOUSE	MK_SCX_27.4156.4156.2	2	4.831	0.472	1	1096.8	73.333336	K.VGVVDEVVPEDQVHSK.A
D3D2_MOUSE	MK_SCX_27.6661.6661.2	2	4.203	0.5	1	972.3	58.823532	R.EADIQNFTSFISKDSIQK.S
DAAM2_MOUSE	MK_SCX_24.8839.8839.2	2	2.17	0.193	1	300.6	46.42857	K.SDIDLLEEHKHEIER.M
DAAM2_MOUSE	MK_SCX_58.2382.2382.3	3	3.343	0.278	1	501.5	38.46154	R.QAILRM*DEQEDLAK.D
DAB2_MOUSE	MK_SCX_20_1.4531.4531.2	2	4.26	0.491	1	1239	70.83333	K.TGQQAEPLVVDLK.D
DAB2_MOUSE	MK_SCX_20_1.4775.4775.2	2	3.444	0.488	1	1510.4	95	K.LIGIDDVPDAR.G
DAB2_MOUSE	MK_SCX_2201.3535.3535.2	2	3.396	0.413	1	1260.1	92.85714	K.TDEYLLAR.F
DAB2_MOUSE	MK_SCX_2201.6608.6608.2	2	5.665	0.557	1	1159.1	72.22222	K.DIPSDAFTGLDPLGDKEVK.E
DAB2_MOUSE	MK_SCX_23.11283.11283.3	3	3.418	0.37	1	425.4	27.272728	K.TGQQAEPLVVDLKDLFQVIYNVK.K
DAB2_MOUSE	MK_SCX_24.7258.7258.3	3	3.734	0.27	1	1196.3	35.869564	R.NGPLKDIPSDAFTGLDPLGDKEVK.E
DAB2_MOUSE	MK_SCX_29.3483.3483.3	3	4.391	0.636	1	578.2	35.526314	K.GFSSSNPSVVSQPASSDPHR.S
DAB2_MOUSE	MK_SCX_29.4369.4369.2	2	3.721	0.428	1	1366	73.07692	K.LIGIDDVPDARGDK.M
DAB2_MOUSE	MK_SCX_34.4331.4331.2	2	3.827	0.428	1	802.8	75	K.AKLIGIDDVPDAR.G
DAB2_MOUSE	MK_SCX_34.4560.4560.3	3	4.277	0.356	1	1008.7	36.842106	R.KGETPPSGTSSAFSSYFNNK.V
DAB2_MOUSE	MK_SCX_34.4578.4578.2	2	6.33	0.588	1	2427.4	78.94737	R.KGETPPSGTSSAFSSYFNNK.V
DAB2_MOUSE	MK_SCX_34.5426.5426.3	3	4.776	0.48	1	810.1	38.75	K.VGIPQEHVDHDDFDANQLLNK.I
DAB2_MOUSE	MK_SCX_45.6296.6296.3	3	3.855	0.329	1	968.8	45.3125	K.EMFKDFQLRQPPLVPSR.K
DAB2_MOUSE	MK_SCX_51.1875.1875.3	3	3.122	0.413	1	794.2	54.545456	K.TGVIEHEHPVNK.I
DAB2_MOUSE	MK_SCX_51.5029.5029.2	2	3.427	0.437	1	610.1	81.818184	K.EVKEMFKDFQLR.Q
DAG1_MOUSE	MK_SCX_26.4352.4352.3	3	4.63	0.482	1	886.2	47.058823	K.IPSDTFYDNEDTTTDKLK.L
DAG1_MOUSE	MK_SCX_26.4380.4380.2	2	5.631	0.543	1	1334.9	67.64706	K.IPSDTFYDNEDTTTDKLK.L
DAK_MOUSE	MK_SCX_16.7188.7188.2	2	6.145	0.637	1	1427	60.869564	R.ASYISSAQLDQPDPGAVAAAAIFR.A
DAK_MOUSE	MK_SCX_17.3711.3711.2	2	3.384	0.598	1	791.1	57.5	R.AAPTEPPEAPEATAAGGVTSK.Q
DAK_MOUSE	MK_SCX_17.5781.5781.2	2	4.762	0.422	1	1569.5	66.66667	K.TDLPTWSAAM*DAGLESMQK.Y
DAK_MOUSE	MK_SCX_19.10000.10000.2	2	3.794	0.512	1	894.7	56.25	R.TMLDSLWAAAQEFQAWK.S
DAK_MOUSE	MK_SCX_20_1.4994.4994.2	2	2.758	0.323	1	611.5	61.538464	K.EGPSLTSPAQVLSR.L
DAK_MOUSE	MK_SCX_29.5540.5540.2	2	2.747	0.236	1	506.2	50	K.VAGALAEEGMGLEEITKR.V
DAK_MOUSE	MK_SCX_29.5586.5586.3	3	3.232	0.379	1	665.5	39.705883	K.VAGALAEEGMGLEEITKR.V
DAZP1_MOUSE	MK_SCX_19.6523.6523.2	2	3.412	0.457	1	356.5	50	K.LFVGGLDWSTTQETLR.S
DAZP1_MOUSE	MK_SCX_31.3004.3004.3	3	4.294	0.508	1	767	41.17647	R.DSKNQAPGQPGASQWGSR.V
DBLOH_MOUSE	MK_SCX_37.3445.3445.3	3	4.165	0.307	1	1113.4	52.083332	K.SEPHSLSNEALMR.R
DBNL_MOUSE	MK_SCX_13.4285.4285.2	2	3.562	0.223	1	984.2	75	R.AEEDVEPECIMEK.V
DBNL_MOUSE	MK_SCX_15.4047.4047.2	2	2.919	0.182	1	1040.7	85	R.NGPALQEAYVR.V
DBNL_MOUSE	MK_SCX_18.4699.4699.2	2	5.302	0.482	1	1192.2	65.789474	K.ESTSFQDVGPQAPVGSVYQK.T

DBNL_MOUSE	MK_SCX_18.7238.7238.2	2	6.107	0.583	1	1895.2	60.526318	R.VAGTGEGGLEELVEELNSGK.V
DBNL_MOUSE	MK_SCX_35.3355.3355.3	3	3.404	0.526	1	870	46.666668	R.AMSTTSVTSSQPGKLR.S
DBNL_MOUSE	MK_SCX_54.3063.3063.3	3	3.846	0.539	1	1746.5	51.666664	R.TRQEWESAGQQAPHPR.E
DBPA_MOUSE	MK_SCX_13.5367.5367.3	3	5.196	0.601	1	354	19.186047	K.SPAASGAPQAPAPAALLAGSPGGDAAPGPAPASSAPAGGEDAEK.K
DBPA_MOUSE	MK_SCX_17.4940.4940.2	2	5.73	0.544	1	1661.2	75	R.SVGDGETVEFDVVEGEK.G
DBPA_MOUSE	MK_SCX_18.8840.8840.2	2	2.471	0.151	1	301	37.5	K.WFNVRNGYGFINRNDTK.E
DBPA_MOUSE	MK_SCX_46.3507.3507.2	2	3.106	0.479	1	329.8	60.714287	R.NDTKEDVFVHQTAIK.K
DBPA_MOUSE	MK_SCX_47.3628.3628.3	3	4.093	0.489	1	1633.8	48.214287	R.NDTKEDVFVHQTAIK.K
DC1I2_MOUSE	MK_SCX_15.5981.5981.2	2	5.033	0.665	1	382.6	40.384613	R.EAEALLQSM*GLTTDSPIVPPPM*SPSSK.S
DC1I2_MOUSE	MK_SCX_15.6486.6486.2	2	5.018	0.617	1	431.8	42.307693	R.EAEALLQSM*GLTTDSPIVPPPMSPSSK.S
DC1I2_MOUSE	MK_SCX_15.7387.7387.3	3	4.091	0.424	1	488.7	28.846153	R.EAEALLQSMGLTTDSPIVPPPMSPSSK.S
DC1I2_MOUSE	MK_SCX_15.7392.7392.2	2	4.691	0.518	1	442.7	44.230766	R.EAEALLQSMGLTTDSPIVPPPMSPSSK.S
DC1I2_MOUSE	MK_SCX_18.2965.2965.2	2	4.904	0.662	1	1298.2	62.5	K.SVSTPSEAGSQDSGDGAVGSR.R
DC1I2_MOUSE	MK_SCX_19.3689.3689.2	2	4.093	0.533	1	1144.2	76.92308	K.EAAVSVQEESDLEK.K
DC1I2_MOUSE	MK_SCX_27.3292.3292.3	3	3.888	0.361	1	595.1	51.923077	R.DLEDKEGEIQAGAK.L
DC1I2_MOUSE	MK_SCX_27.3311.3311.2	2	4.244	0.357	1	1347	73.07692	R.DLEDKEGEIQAGAK.L
DC1I2_MOUSE	MK_SCX_27.3991.3991.3	3	4.274	0.421	1	603.7	38.235294	R.EIVTYTKETQTPVTAQPK.E
DC1I2_MOUSE	MK_SCX_33.3429.3429.3	3	4.928	0.47	1	1528.5	46.42857	K.KEAAVSVQEESDLEK.K
DC1L1_MOUSE	MK_SCX_19.9462.9462.2	2	4.475	0.527	1	1306.6	61.11111	K.AGATSEGVLANFFNSLLSK.K
DC1L1_MOUSE	MK_SCX_27.3836.3836.3	3	3.218	0.415	1	606.2	29.310345	K.TGSPGGPGVGGSPGGGAAGASPSLPPSAKK.S
DC1L1_MOUSE	MK_SCX_30.3633.3633.3	3	5.822	0.548	1	784.9	30.172413	K.KTGSPGGPGVGGSPGGGAAGASPSLPPSAK.K
DCPS_MOUSE	MK_SCX_25.4260.4260.3	3	3.958	0.527	1	763.1	41.666664	K.VNEDSGDTHGEDAVVILEK.T
DCPS_MOUSE	MK_SCX_28.7262.7262.3	3	3.13	0.186	1	472.1	31.944445	K.TPFQVEHVAQLLTGSPELK.L
DCPS_MOUSE	MK_SCX_28.7346.7346.2	2	5.168	0.624	1	2194.5	75	K.TPFQVEHVAQLLTGSPELK.L
DCPS_MOUSE	MK_SCX_49.6153.6153.3	3	3.303	0.368	1	488.6	38.46154	R.SLRDLTPEHLPLLR.N
DCTN2_MOUSE	MK_SCX_15.8741.8741.3	3	4.194	0.25	1	674	27.777779	R.CDQDAQNPLSAGLQGACLMETVELLQAK.V
DCTN2_MOUSE	MK_SCX_15.8794.8794.2	2	2.724	0.425	1	366.8	27.777779	R.CDQDAQNPLSAGLQGACLMETVELLQAK.V
DCTN2_MOUSE	MK_SCX_17.6231.6231.2	2	6.093	0.615	1	1542.6	61.11111	K.LLGPDAAINLADPDGALAK.R
DCTN2_MOUSE	MK_SCX_18.5463.5463.2	2	3.367	0.48	1	788.6	55.88235	R.TGYESGDYEMLGEGLGVK.E
DCTN2_MOUSE	MK_SCX_20_1.6803.6803.2	2	4.318	0.471	1	631	61.538464	R.WSPVASTLPELVQR.L
DCTN2_MOUSE	MK_SCX_26.5943.5943.3	3	3.785	0.495	1	1059.6	46.05263	K.LLGPDAAINLADPDGALAKR.L
DCTN2_MOUSE	MK_SCX_31.5442.5442.3	3	4.309	0.313	1	1175.9	53.846157	R.LLHEVQELTTEVEK.I
DCTN2_MOUSE	MK_SCX_31.5444.5444.2	2	5.146	0.493	1	1506.4	84.61539	R.LLHEVQELTTEVEK.I
DCTN2_MOUSE	MK_SCX_38.5536.5536.3	3	4.763	0.487	1	1300.2	37.5	K.ASVEDADTQNKVHQLYETIQR.W
DCTN4_MOUSE	MK_SCX_19.7522.7522.3	3	3.934	0.442	1	365.5	22.65625	K.IEPAQAVAEVEPLPEDYYTRPVNLTEVTTLQQR.L
DCXR_MOUSE	MK_SCX_20_1.3844.3844.2	2	4.128	0.49	1	877.7	64.28571	R.VNAVNPTVVM*TPM*GR.T
DCXR_MOUSE	MK_SCX_21.4261.4261.2	2	5.015	0.576	1	1590.9	83.33333	R.GVPGAIVNVSSQASQR.A
DCXR_MOUSE	MK_SCX_2201.2740.2740.2	2	4.039	0.5	1	1932.8	90	K.AAGAQVVAVSR.T
DCXR_MOUSE	MK_SCX_37.4489.4489.2	2	3.171	0.3	1	1198.7	83.33333	K.AMLDRIPLGK.F
DD19A_MOUSE	MK_SCX_23.5844.5844.3	3	4.993	0.567	1	734.7	33.333336	R.SNLVDNTNQVEVLQRDPSSPLYSVK.S
DDAH1_MOUSE	MK_SCX_13.6768.6768.2	2	5.43	0.541	1	1377.8	60.000004	K.SFCSMAGPNLIAIGSSESAQK.A
DDAH1_MOUSE	MK_SCX_16.5380.5380.2	2	5.723	0.592	1	2059.2	76.666664	K.DENATLDGGDVLFTGR.E
DDAH1_MOUSE	MK_SCX_2201.3093.3093.2	2	3.338	0.408	1	966.2	88.88889	R.SQGEEVDFAR.A
DDAH1_MOUSE	MK_SCX_23.6846.6846.3	3	5.927	0.62	1	3282	46.875	K.LQLNIVEM*KDENATLDGGDVLFTGR.E
DDAH1_MOUSE	MK_SCX_23.7428.7428.3	3	5.33	0.585	1	2953.8	43.75	K.LQLNIVEMKDENATLDGGDVLFTGR.E
DDAH1_MOUSE	MK_SCX_25.5618.5618.2	2	2.859	0.352	1	319	46.666668	K.DYAVSTVPVADSLHLK.S
DDAH1_MOUSE	MK_SCX_30.8120.8120.2	2	5.727	0.597	1	1458.7	50	R.GAEILADTFKDYAVSTVPVADSLHLK.S
DDAH1_MOUSE	MK_SCX_30.8282.8282.3	3	6.117	0.3	1	806.1	38	R.GAEILADTFKDYAVSTVPVADSLHLK.S
DDAH1_MOUSE	MK_SCX_31.3354.3354.3	3	3.903	0.476	1	767.7	48.076923	R.TPEEYPESAKVYEK.L
DDAH1_MOUSE	MK_SCX_38.4035.4035.2	2	2.383	0.399	1	454.1	77.27273	R.QHELYVGVLGSK.L
DDAH1_MOUSE	MK_SCX_38.4049.4049.3	3	4.145	0.392	1	1693.2	61.363636	R.QHELYVGVLGSK.L

DDAH1_MOUSE	MK_SCX_47.9141.9141.3	3	3.487	0.407	1	580.2	25	K.LKDHLLIPVSNSEMEKVDGLLTCCSVFINK.K
DDAH1_MOUSE	MK_SCX_48.3958.3958.3	3	3.95	0.388	1	1043.3	46.666668	K.LKDHLLIPVSNSEM*EK.V
DDAH1_MOUSE	MK_SCX_49.4495.4495.3	3	3.561	0.249	1	830	41.666664	K.LKDHLLIPVSNSEMEK.V
DDAH1_MOUSE	MK_SCX_49.4512.4512.2	2	4.712	0.335	1	649.4	63.333332	K.LKDHLLIPVSNSEMEK.V
DDAH1_MOUSE	MK_SCX_58.2402.2402.2	2	2.004	0.32	1	401.6	75	K.GHVLLHR.T
DDAH2_MOUSE	MK_SCX_17.6207.6207.2	2	5.305	0.672	1	1625.3	56.81818	R.GVPESLASGEGAGAGLPALDLAK.A
DDX1_MOUSE	MK_SCX_16.9296.9296.2	2	4.139	0.481	1	844.2	57.5	R.FLVLDEADGLLSQGYSDFINR.M
DDX17_MOUSE	MK_SCX_20_1.4563.4563.2	2	4.01	0.583	1	1557.1	86.36364	K.APILIATDVASR.G
DDX17_MOUSE	MK_SCX_21.5579.5579.2	2	3.845	0.438	1	1709.2	90	R.MLDMGFEPQIR.K
DDX17_MOUSE	MK_SCX_23.5647.5647.2	2	2.893	0.415	1	483.1	70	R.QTLMWSATWPK.E
DDX17_MOUSE	MK_SCX_33.4293.4293.3	3	3.165	0.383	1	1118.4	42.857143	R.SGKAPILIATDVASR.G
DDX21_MOUSE	MK_SCX_26.4225.4225.3	3	4.275	0.447	1	678.3	36.11111	K.LGSDGAEESMETLPKPSEK.K
DDX21_MOUSE	MK_SCX_31.3698.3698.2	2	2.426	0.135	1	382.6	50	R.IKDHLQNGKLDLTK.L
DDX21_MOUSE	MK_SCX_32.3545.3545.3	3	5.356	0.564	1	2484.2	53.125	K.SKTEEATEGMEEAVSSK.A
DDX21_MOUSE	MK_SCX_32.3559.3559.2	2	5.841	0.61	1	3049.2	78.125	K.SKTEEATEGMEEAVSSK.A
DDX39_MOUSE	MK_SCX_23.5370.5370.2	2	3.7	0.274	1	1384.8	94.44444	R.ILVATNLFGR.G
DDX3X_MOUSE	MK_SCX_17.10050.10050.3	3	4.134	0.451	1	794.7	39.285713	K.TAAFLLPILSQIYADGPGEALR.A
DDX3X_MOUSE	MK_SCX_17.9840.9840.2	2	5.233	0.596	1	1413.8	59.523808	K.TAAFLLPILSQIYADGPGEALR.A
DDX3X_MOUSE	MK_SCX_2201.6878.6878.2	2	4.659	0.525	1	1781.9	80.769226	R.VGNLGLATSFFNER.N
DDX3X_MOUSE	MK_SCX_29.3803.3803.3	3	3.244	0.493	1	633	41.07143	K.SDEDDWSKPLPPSER.L
DDX3X_MOUSE	MK_SCX_37.5564.5564.2	2	2.515	0.24	1	353.6	58.333332	R.KQYPISLVLAPTR.E
DDX3X_MOUSE	MK_SCX_42.3723.3723.3	3	4.281	0.556	1	1112.9	38.157894	K.DSSGWSSSKDKDAYSSFGSR.G
DDX3X_MOUSE	MK_SCX_42.4469.4469.2	2	2.987	0.511	1	331.1	80	R.HTMMFSATFPK.E
DDX3X_MOUSE	MK_SCX_45.7526.7526.3	3	4.967	0.531	1	1621.5	51.5625	K.HVINFDLPSDIEEYVHR.I
DDX3X_MOUSE	MK_SCX_54.4571.4571.3	3	3.865	0.378	1	682.7	44.230766	R.RKQYPISLVLAPTR.E
DDX46_MOUSE	MK_SCX_16.3549.3549.2	2	4.877	0.494	1	865	63.15789	K.DM*AAPGTSSVPAPTAGNAEK.L
DDX46_MOUSE	MK_SCX_16.3985.3985.2	2	4.553	0.42	1	1115.1	68.42105	K.DMAAPGTSSVPAPTAGNAEK.L
DDX46_MOUSE	MK_SCX_2201.8690.8690.2	2	3.144	0.47	1	920.7	77.77778	K.TIAFLLPMFR.H
DDX46_MOUSE	MK_SCX_29.3745.3745.3	3	3.171	0.317	1	523.4	32.142857	R.VKDMAAPGTSSVPAPTAGNAEK.L
DDX46_MOUSE	MK_SCX_42.5965.5965.2	2	2.841	0.297	1	383.9	65	K.EKDAGNFDQNK.L
DDX46_MOUSE	MK_SCX_47.5307.5307.3	3	4.577	0.568	1	564.4	33.75	K.HGYEKPTPIQTQAIPAIMSGR.D
DDX48_MOUSE	MK_SCX_33.4605.4605.2	2	5.723	0.618	1	1291.5	80	R.GIYAYGFEKPSAIQQR.A
DDX48_MOUSE	MK_SCX_33.4630.4630.3	3	3.442	0.453	1	1215	46.666668	R.GIYAYGFEKPSAIQQR.A
DDX5_MOUSE	MK_SCX_21.5285.5285.2	2	4.526	0.531	1	1584.9	76.92308	K.TGTAYTFFTPNNIK.Q
DDX5_MOUSE	MK_SCX_24.4871.4871.2	2	2.383	0.31	1	1038.3	100	K.WNLDELPK.F
DDX5_MOUSE	MK_SCX_28.5541.5541.3	3	3.546	0.442	1	563.4	35.9375	K.FVINYDYPNSSEDYIHR.I
DDX5_MOUSE	MK_SCX_28.5562.5562.2	2	4.224	0.434	1	466.7	50	K.FVINYDYPNSSEDYIHR.I
DDX5_MOUSE	MK_SCX_39.3638.3638.2	2	4.078	0.427	1	1445.8	85	K.NFYQEHPDLAR.R
DDX5_MOUSE	MK_SCX_53.3833.3833.3	3	4.335	0.351	1	1714.8	50	K.HGKAPILIATDVASR.G
DDX56_MOUSE	MK_SCX_28.4318.4318.2	2	3.448	0.349	1	424.3	44.444447	K.ATGPVMEQAVRGLVLVPTK.E
DECR_MOUSE	MK_SCX_18.7778.7778.2	2	3.205	0.486	1	605.7	53.125	R.FDGGEEVFLSGEFNSLK.K
DECR_MOUSE	MK_SCX_2201.4214.4214.2	2	3.114	0.252	1	1408.1	79.16667	K.VAFITGGGTGLGK.A
DECR_MOUSE	MK_SCX_26.5055.5055.3	3	3.909	0.285	1	1654.2	41.666664	K.VAGHPDVVINNAAGNFISPSER.L
DECR_MOUSE	MK_SCX_27.6764.6764.2	2	4.351	0.503	1	1100.1	64.70589	K.FFQPVLKPM*LPPDAFQGK.V
DECR_MOUSE	MK_SCX_27.7467.7467.2	2	5.218	0.495	1	1055.5	64.70589	K.FFQPVLKPMLPPDAFQGK.V
DECR_MOUSE	MK_SCX_28.5224.5224.2	2	6.801	0.611	1	2661.7	69.047615	K.VAGHPDVVINNAAGNFISPSER.L
DECR_MOUSE	MK_SCX_34.4313.4313.2	2	3.429	0.269	1	1384.5	86.36364	R.FNIIQPGPIKTK.G
DECR_MOUSE	MK_SCX_35.11257.11257.2	2	3.675	0.402	1	2704.4	84.61539	K.VTKEEWDIIEGLIR.K
DECR_MOUSE	MK_SCX_35.11346.11346.3	3	4.907	0.232	1	2888.9	65.38461	K.VTKEEWDIIEGLIR.K
DECR_MOUSE	MK_SCX_49.7259.7259.3	3	4.116	0.373	1	1185.3	44.642857	K.VTKEEWDIIEGLIRK.T
DECR_MOUSE	MK_SCX_53.7372.7372.3	3	3.264	0.348	1	1139.3	48.214287	K.KVTKEEWDIIEGLIR.K

DEK_MOUSE	MK_SCX_19.4765.4765.2	2	3.821	0.447	1	1392.8	79.16667	K.LLADANLEEVTMK.Q
DEK_MOUSE	MK_SCX_23.5477.5477.2	2	3.805	0.503	1	1117.7	77.27273	K.NVGQFSGFPFEK.G
DEK_MOUSE	MK_SCX_41.4990.4990.2	2	3.091	0.361	1	456.3	62.5	K.KNVGQFSGFPFEK.G
DEMA_MOUSE	MK_SCX_16.4346.4346.2	2	3.29	0.348	1	771.9	41.666664	R.LQSTEFSPSGSEAGSPGLQNGEGQR.G
DEMA_MOUSE	MK_SCX_40.5188.5188.3	3	3.183	0.154	1	496.6	38.235294	K.MDNQVLGYKDLAAIPKDK.A
DENR_MOUSE	MK_SCX_18.3473.3473.2	2	3.251	0.394	1	455.8	44.11765	K.QETGITEGQGPVGEEEEK.K
DENR_MOUSE	MK_SCX_24.4108.4108.3	3	7.035	0.585	1	2087.9	40	K.LTVENSPKQETGITEGQGPVGEEEEK.K
DESM_MOUSE	MK_SCX_18.7417.7417.2	2	3.964	0.543	1	863.1	47.5	R.TFGGAPGFSLGSPLSSPVFPR.A
DESM_MOUSE	MK_SCX_20_1.3683.3683.2	2	2.977	0.356	1	934.6	73.07692	R.FASEANGYQDNIAR.L
DESM_MOUSE	MK_SCX_20_1.8571.8571.2	2	3.97	0.448	1	1532.3	80.769226	R.INLPIQTFSALNFR.E
DESM_MOUSE	MK_SCX_37.3585.3585.3	3	3.86	0.317	1	646.9	45.833336	R.TNEKVELQELNDR.F
DESM_MOUSE	MK_SCX_51.5344.5344.3	3	3.786	0.275	1	1713.6	51.923077	R.RIESLNEEIAFLKK.V
DEST_MOUSE	MK_SCX_21.4571.4571.2	2	3.618	0.463	1	1461.7	90	R.YALYDASFETK.E
DEST_MOUSE	MK_SCX_41.7718.7718.3	3	4.753	0.428	1	1044.2	43.055553	R.KEELMFFLWAPEQAPLKSK.M
DEST_MOUSE	MK_SCX_56.3793.3793.3	3	3.551	0.346	1	754.1	43.055553	K.KFPGIKHEYQANGPEDLNR.T
DHB4_MOUSE	MK_SCX_16.3378.3378.2	2	3.011	0.331	1	973.4	64.28571	K.NNIHCNTIAPNAGSR.M
DHB4_MOUSE	MK_SCX_18.5584.5584.2	2	4.896	0.616	1	846	54.545456	R.ILMTSSASGIYGNFGQANYSAAK.L
DHB4_MOUSE	MK_SCX_21.3286.3286.2	2	3.925	0.55	1	1607.1	79.16667	K.AVANYDSVEAGEK.L
DHB4_MOUSE	MK_SCX_21.4989.4989.2	2	3.687	0.455	1	1460.3	77.27273	R.IDVVVNNAGILR.D
DHB4_MOUSE	MK_SCX_2201.3975.3975.2	2	4.807	0.506	1	2615.3	95.83333	R.VVLVTGAGGGLGR.A
DHB4_MOUSE	MK_SCX_30.4104.4104.2	2	3.057	0.178	1	397.6	60.000004	K.AVANYDSVEAGEKLVK.T
DHB4_MOUSE	MK_SCX_32.4716.4716.2	2	3.603	0.507	1	1851	90	R.ISDEDWDIIHR.V
DHB4_MOUSE	MK_SCX_32.4753.4753.3	3	3.705	0.486	1	808	57.5	R.ISDEDWDIIHR.V
DHB4_MOUSE	MK_SCX_32.4968.4968.3	3	3.792	0.508	1	696.3	39.0625	R.FAKPVYPGQTLQTEM*WK.E
DHB4_MOUSE	MK_SCX_32.5480.5480.2	2	4.177	0.528	1	1324.1	78.125	R.FAKPVYPGQTLQTEMWK.E
DHB4_MOUSE	MK_SCX_32.5493.5493.3	3	5.359	0.48	1	2099	54.6875	R.FAKPVYPGQTLQTEMWK.E
DHB4_MOUSE	MK_SCX_34.4052.4052.2	2	3.968	0.404	1	690.7	64.28571	R.NQPMTPEAVRDNWEK.I
DHB4_MOUSE	MK_SCX_39.3648.3648.3	3	4.195	0.245	1	2547.6	62.5	R.HVLQQFADNDVSR.F
DHB4_MOUSE	MK_SCX_39.3664.3664.2	2	5.044	0.529	1	1828.3	91.66667	R.HVLQQFADNDVSR.F
DHB4_MOUSE	MK_SCX_48.5145.5145.2	2	5.698	0.674	1	2350.4	76.666664	K.ALHGEQYLELYKPLPR.S
DHB4_MOUSE	MK_SCX_51.3891.3891.3	3	3.648	0.24	1	1871.6	46.875	K.GIGKGSSAADKVVAEIR.R
DHB5_MOUSE	MK_SCX_43.4334.4334.3	3	3.807	0.296	1	916.7	50	K.YNRTPALIALR.Y
DHB8_MOUSE	MK_SCX_21.3397.3397.2	2	3.07	0.441	1	692.9	70.83333	R.LLGSPGSEDGAPR.G
DHB8_MOUSE	MK_SCX_39.3348.3348.2	2	4.951	0.665	1	2081.2	82.14286	K.HAAFQADVSQGPAAR.R
DHCA_MOUSE	MK_SCX_19.5947.5947.2	2	3.54	0.306	1	1117.4	71.42857	R.SETITEEELVGLMNK.F
DHCA_MOUSE	MK_SCX_23.3900.3900.2	2	4.257	0.585	1	1443.6	95	R.VVNVSSM*VSLR.A
DHCA_MOUSE	MK_SCX_23.3959.3959.2	2	3.004	0.48	1	771.1	88.88889	K.FSGDVVLAAR.D
DHCA_MOUSE	MK_SCX_23.4708.4708.2	2	4.019	0.495	1	1668.6	90	R.VVNVSSMVSLR.A
DHCA_MOUSE	MK_SCX_24.6186.6186.2	2	2.784	0.252	1	460.9	59.090908	K.ILLNACCPGWVR.T
DHCA_MOUSE	MK_SCX_37.4635.4635.3	3	3.658	0.29	1	661.7	47.916664	R.FHQLDIDNPQSIR.A
DHCA_MOUSE	MK_SCX_38.4436.4436.2	2	4.185	0.385	1	2584.9	91.66667	R.FHQLDIDNPQSIR.A
DHCA_MOUSE	MK_SCX_41.3688.3688.2	2	2.231	0.327	1	359.6	65	R.KFSGDVVLAAR.D
DHCA_MOUSE	MK_SCX_52.3493.3493.3	3	4.842	0.47	1	1062.3	47.058823	K.KGVHAEEGWPNSAYGVTK.I
DHE3_MOUSE	MK_SCX_13.5999.5999.2	2	3.378	0.378	1	632.6	52.63158	K.CVGVGESDGSIWNPDGIDPK.E
DHE3_MOUSE	MK_SCX_17.5260.5260.2	2	4.797	0.561	1	761.9	61.11111	K.GFIGPGIDVPAPDM*STGER.E
DHE3_MOUSE	MK_SCX_17.5905.5905.2	2	5.09	0.521	1	667	58.333332	K.GFIGPGIDVPAPDMSTGER.E
DHE3_MOUSE	MK_SCX_18.3384.3384.2	2	3.411	0.343	1	1828.6	76.666664	K.IIAEGANGPTTPEADK.I
DHE3_MOUSE	MK_SCX_21.3875.3875.2	2	2.679	0.536	1	772.3	80	R.YSTDVSVDEVK.A
DHE3_MOUSE	MK_SCX_2201.2628.2628.2	2	2.309	0.143	1	379.3	68.75	K.NYTDNELEK.I
DHE3_MOUSE	MK_SCX_2201.3375.3375.2	2	2.959	0.378	1	1417	88.88889	R.TAAYVNAIEK.V
DHE3_MOUSE	MK_SCX_24.5460.5460.3	3	3.236	0.256	1	647.9	33.75	K.IIAEGANGPTTPEADKIFLER.N

DHE3_MOUSE	MK_SCX_24.5478.5478.2	2	3.3	0.436	1	513.5	50	K.IIAEGANGPTTPEADKIFLER.N
DHE3_MOUSE	MK_SCX_25.4443.4443.2	2	2.945	0.298	1	641.2	92.85714	K.MVEGFFDR.G
DHE3_MOUSE	MK_SCX_25.4799.4799.2	2	3.083	0.318	1	1123.2	92.85714	K.YNLGLDLR.T
DHE3_MOUSE	MK_SCX_29.4418.4418.3	3	3.499	0.327	1	1314.4	50	K.DIVHSGLAYTM*ER.S
DHE3_MOUSE	MK_SCX_29.4969.4969.2	2	4.117	0.522	1	1659.8	87.5	K.DIVHSGLAYTMER.S
DHE3_MOUSE	MK_SCX_29.4974.4974.3	3	3.68	0.269	1	1013.2	43.75	K.DIVHSGLAYTMER.S
DHE3_MOUSE	MK_SCX_30.4930.4930.3	3	3.836	0.417	1	549.1	34.210526	K.KGFIGPGIDVPAPDM*STGER.E
DHE3_MOUSE	MK_SCX_30.4967.4967.2	2	5.205	0.617	1	706.1	63.15789	K.KGFIGPGIDVPAPDM*STGER.E
DHE3_MOUSE	MK_SCX_30.5018.5018.3	3	3.947	0.408	1	741.6	46.153847	R.GASIVEDKLVEDLK.T
DHE3_MOUSE	MK_SCX_30.5030.5030.2	2	4.759	0.445	1	1388.8	76.92308	R.GASIVEDKLVEDLK.T
DHE3_MOUSE	MK_SCX_30.5634.5634.2	2	5.882	0.631	1	1814.7	71.05263	K.KGFIGPGIDVPAPDMSTGER.E
DHE3_MOUSE	MK_SCX_30.5650.5650.3	3	4.075	0.529	1	1137.9	44.736843	K.KGFIGPGIDVPAPDMSTGER.E
DHE3_MOUSE	MK_SCX_34.11997.11997.2	2	2.245	0.255	1	342.7	56.666668	K.TFVVQGFGNVGLHSMR.Y
DHE3_MOUSE	MK_SCX_34.4633.4633.2	2	4.786	0.603	1	828	73.333336	K.HGGTIPVVPTAEFQDR.I
DHE3_MOUSE	MK_SCX_34.4839.4839.3	3	3.411	0.234	1	1414	53.333336	K.HGGTIPVVPTAEFQDR.I
DHE3_MOUSE	MK_SCX_34.5959.5959.3	3	3.956	0.593	1	1001.6	51.666664	K.TFVVQGFGNVGLHSMR.Y
DHE3_MOUSE	MK_SCX_36.4858.4858.2	2	3.952	0.453	1	1409.5	87.5	R.RDDGSWEVIEGYR.A
DHE3_MOUSE	MK_SCX_41.4205.4205.2	2	2.016	0.188	1	591	70	K.LQHGSILGFPK.A
DHE3_MOUSE	MK_SCX_42.6296.6296.2	2	5.174	0.606	1	2078.7	78.125	K.ELEDFKLQHGSILGFPK.A
DHE3_MOUSE	MK_SCX_43.4782.4782.3	3	5.518	0.622	1	1635.8	43.421055	R.ISGASEKDIVHSGLAYTM*ER.S
DHE3_MOUSE	MK_SCX_43.5107.5107.3	3	5.716	0.571	1	2177.2	48.684208	R.ISGASEKDIVHSGLAYTMER.S
DHE3_MOUSE	MK_SCX_43.5130.5130.3	3	4.464	0.479	1	1278	50	R.GASIVEDKLVEDLKTR.E
DHE3_MOUSE	MK_SCX_43.5230.5230.2	2	6.203	0.649	1	2302.9	71.05263	R.ISGASEKDIVHSGLAYTMER.S
DHE3_MOUSE	MK_SCX_43.6570.6570.3	3	4.539	0.534	1	1212.2	46.875	K.ELEDFKLQHGSILGFPK.A
DHE3_MOUSE	MK_SCX_57.11063.11063.3	3	3.292	0.192	1	344.1	28.947369	R.KFGKHGGTIPVVPTAEFQDR.I
DHI1_MOUSE	MK_SCX_2201.7898.7898.2	2	3.313	0.498	1	1741.6	90	K.FALDGFFSTIR.T
DHI1_MOUSE	MK_SCX_24.5949.5949.2	2	2.441	0.295	1	512.7	78.57143	K.IMEFFSLR.Y
DHI1_MOUSE	MK_SCX_43.6006.6006.3	3	4.214	0.431	1	1615.4	41.25	R.KSEVYYDKSPLTPILLGNPGR.K
DHPR_MOUSE	MK_SCX_16.6478.6478.2	2	5.908	0.66	1	1306	56.521736	K.NSGM*PPGAAAIAVLPVTLDTPM*NR.K
DHPR_MOUSE	MK_SCX_16.6916.6916.2	2	5.628	0.379	1	1256	56.521736	K.NSGMPPGAAAIAVLPVTLDTPM*NR.K
DHPR_MOUSE	MK_SCX_16.7428.7428.2	2	5.805	0.63	1	1246.7	58.69565	K.NSGMPPGAAAIAVLPVTLDTPMNR.K
DHPR_MOUSE	MK_SCX_17.4734.4734.2	2	6.114	0.616	1	3503	79.411766	K.M*TDSFTEQADQVTADVGK.L
DHPR_MOUSE	MK_SCX_17.5011.5011.2	2	6.229	0.687	1	3235.7	76.47059	K.MTDSFTEQADQVTADVGK.L
DHPR_MOUSE	MK_SCX_18.4781.4781.2	2	2.922	0.261	1	698.9	60.000004	K.AALDGTPGMIGYGMAK.G
DHPR_MOUSE	MK_SCX_19.4217.4217.2	2	3.707	0.132	1	712.6	70	K.AALDGTPGMIGYGM*AK.G
DHPR_MOUSE	MK_SCX_19.4234.4234.2	2	3.199	0.143	1	560.8	63.333332	K.AALDGTPGM*IGYGMAK.G
DHPR_MOUSE	MK_SCX_23.8328.8328.3	3	3.448	0.175	1	964.9	33.695652	K.M*TDSFTEQADQVTADVGKLLGDQK.V
DHPR_MOUSE	MK_SCX_23.8556.8556.3	3	5.012	0.564	1	1140.5	36.95652	K.MTDSFTEQADQVTADVGKLLGDQK.V
DHPR_MOUSE	MK_SCX_25.7257.7257.2	2	4.406	0.632	1	890.9	50	K.NSGMPPGAAAIAVLPVTLDTPMNRK.S
DHPR_MOUSE	MK_SCX_50.2444.2444.2	2	2.435	0.36	1	554.3	78.57143	R.RVLVYGGR.G
DHPR_MOUSE	MK_SCX_54.3842.3842.3	3	3.693	0.393	1	981.7	46.153847	K.HLKEGGLLTLAGAK.A
DHPR_MOUSE	MK_SCX_54.3857.3857.2	2	3.593	0.533	1	1408.7	88.46153	K.HLKEGGLLTLAGAK.A
DHRS1_MOUSE	MK_SCX_2201.2343.2343.2	2	3.848	0.443	1	1755.7	81.818184	R.ATAQEAQSLGGR.C
DHRS1_MOUSE	MK_SCX_33.3698.3698.3	3	3.501	0.223	1	1643.7	51.666664	K.MKPDFSSAESPEMSGK.C
DHRS4_MOUSE	MK_SCX_23.4564.4564.2	2	2.728	0.305	1	782.1	75	R.FPSLGPYNVSK.T
DHRS4_MOUSE	MK_SCX_33.3140.3140.2	2	3.461	0.457	1	1351.3	75	R.LAEDGAHVVVSSR.K
DHRS4_MOUSE	MK_SCX_33.3144.3144.3	3	3.41	0.512	1	519.5	52.083332	R.LAEDGAHVVVSSR.K
DHRS4_MOUSE	MK_SCX_34.5721.5721.2	2	3.129	0.495	1	448.9	75	R.EDFIKEAMQIR.R
DHRS4_MOUSE	MK_SCX_52.4908.4908.3	3	4.26	0.425	1	913.2	50	K.AREDFIKEAMQIR.R
DHRS4_MOUSE	MK_SCX_52.4931.4931.2	2	4.114	0.395	1	1729.5	87.5	K.AREDFIKEAMQIR.R
DHRS6_MOUSE	MK_SCX_21.4842.4842.2	2	4.585	0.545	1	2134.2	87.5	K.VIVLTAAAQGIGR.A

DHRS6_MOUSE	MK_SCX_2201.4759.4759.2	2	3.634	0.39	1	1546.3	81.818184	K.SVAADFIQQGIR.C
DHRS6_MOUSE	MK_SCX_28.5706.5706.2	2	5.668	0.621	1	1842.5	73.52941	K.VIATDINESKLQELESYR.G
DHRS6_MOUSE	MK_SCX_28.5733.5733.3	3	3.836	0.547	1	512.6	42.647057	K.VIATDINESKLQELESYR.G
DHRS6_MOUSE	MK_SCX_35.3935.3935.2	2	3.674	0.228	1	705.6	62.5	K.LQELESYRGIQTR.V
DHRS6_MOUSE	MK_SCX_38.6356.6356.3	3	3.443	0.475	1	435.5	29.545454	K.VIATDINESKLQELESYRGIQTR.V
DHRS6_MOUSE	MK_SCX_49.4975.4975.3	3	3.727	0.441	1	984.1	50	R.DNPKEALKTFLNR.Q
DHRS6_MOUSE	MK_SCX_49.5013.5013.2	2	3.648	0.332	1	738.7	70.83333	R.DNPKEALKTFLNR.Q
DHRS8_MOUSE	MK_SCX_18.9075.9075.2	2	2.912	0.334	1	514.7	59.375	K.QM*IFVPSSIALLTVLER.I
DHRS8_MOUSE	MK_SCX_18.9715.9715.2	2	3.284	0.443	1	438.8	50	K.QMIFVPSSIALLTVLER.I
DHRS8_MOUSE	MK_SCX_20_1.5693.5693.2	2	3.569	0.442	1	1551.1	95	R.ALTDELAALGR.T
DHRS8_MOUSE	MK_SCX_26.5665.5665.2	2	2.725	0.161	1	595.9	56.25	K.LVLWDINKNGIEETAAK.C
DHSA_MOUSE	MK_SCX_13.6579.6579.2	2	4.202	0.289	1	1317.6	68.75	R.AAFGLSEAGFNTACLTK.L
DHSA_MOUSE	MK_SCX_19.7498.7498.3	3	5.236	0.527	1	927.3	26.51515	K.GSDWLGDQDAIHYM*TEQAPASVVELENYGM*PFSR.T
DHSA_MOUSE	MK_SCX_19.7951.7951.3	3	5.953	0.567	1	1888.1	31.818182	K.GSDWLGDQDAIHYM*TEQAPASVVELENYGMPFSR.T
DHSA_MOUSE	MK_SCX_19.8661.8661.3	3	6.114	0.644	1	1973.5	32.575756	K.GSDWLGDQDAIHYMTEQAPASVVELENYGM*PFSR.T
DHSA_MOUSE	MK_SCX_19.9342.9342.3	3	6.3	0.541	1	2608.2	34.848484	K.GSDWLGDQDAIHYMTEQAPASVVELENYGMPFSR.T
DHSA_MOUSE	MK_SCX_20_1.10063.10063.2	2	4.288	0.525	1	2197.8	84.61539	R.LGANSLLDLVVFGR.A
DHSA_MOUSE	MK_SCX_21.15386.15386.3	3	5.17	0.591	1	1459.1	33.035713	K.VSDAISTQYPVVDHEFDAVVVGAGGAGLR.A
DHSA_MOUSE	MK_SCX_21.4024.4024.2	2	2.849	0.306	1	774.6	75	K.ANAGEESVMNLDK.L
DHSA_MOUSE	MK_SCX_21.7752.7752.2	2	4.756	0.628	1	848.5	41.07143	K.VSDAISTQYPVVDHEFDAVVVGAGGAGLR.A
DHSA_MOUSE	MK_SCX_2201.3802.3802.2	2	2.574	0.374	1	1063.1	81.25	K.ISQLYGDLK.H
DHSA_MOUSE	MK_SCX_29.3395.3395.3	3	3.375	0.355	1	875.1	44.642857	R.VDEYDYSKPIQGQQK.K
DHSA_MOUSE	MK_SCX_29.3411.3411.2	2	5.087	0.521	1	995.6	67.85714	R.VDEYDYSKPIQGQQK.K
DHSA_MOUSE	MK_SCX_32.4495.4495.2	2	4.555	0.392	1	1979.7	78.57143	K.ANAGEESVMNLDKLR.F
DHSA_MOUSE	MK_SCX_32.4599.4599.3	3	3.401	0.44	1	535.3	39.285713	K.ANAGEESVMNLDKLR.F
DHSA_MOUSE	MK_SCX_33.4151.4151.2	2	3.463	0.312	1	1154.5	80	K.VTLEYRPVIDK.T
DHSA_MOUSE	MK_SCX_37.5622.5622.3	3	5.338	0.489	1	763.8	29.62963	K.HVNGQDQIVPGLYACGEAACASVHGANR.L
DHSA_MOUSE	MK_SCX_45.4970.4970.2	2	3.009	0.144	1	606.3	50	R.ACALSIAESCRPGDKVPSIK.A
DHSA_MOUSE	MK_SCX_47.3762.3762.2	2	5.345	0.594	1	1642.8	71.875	K.VRVDEYDYSKPIQGQQK.K
DHSA_MOUSE	MK_SCX_48.3655.3655.3	3	4.352	0.437	1	932.5	43.75	K.VRVDEYDYSKPIQGQQK.K
DHSA_MOUSE	MK_SCX_50.3735.3735.3	3	3.554	0.367	1	1077.2	46.153847	K.TGKVTLEYRPVIDK.T
DHSA_MOUSE	MK_SCX_60.13530.13530.2	2	2.034	0.172	1	361	85.71429	K.KPFGEHWR.K
DHSB_MOUSE	MK_SCX_13.6873.6873.2	2	3.004	0.248	1	977.4	75	K.CGPMVLDALIK.I
DHSB_MOUSE	MK_SCX_17.6663.6663.2	2	4.204	0.619	1	722.3	76.92308	K.DLVPDLSNFYAQYK.S
DHSB_MOUSE	MK_SCX_17.7917.7917.2	2	4.358	0.642	1	921.2	57.5	R.MQTYEVDLNKCGPMVLDALIK.I
DHSB_MOUSE	MK_SCX_2201.5014.5014.2	2	2.567	0.267	1	566.1	68.181816	K.YLGPAVLM*QAYR.W
DHSB_MOUSE	MK_SCX_2201.6160.6160.2	2	4.015	0.482	1	1613	86.36364	K.YLGPAVLMQAYR.W
DHSB_MOUSE	MK_SCX_23.4916.4916.2	2	3.235	0.296	1	686.1	87.5	K.LQDPFSVYR.C
DHSB_MOUSE	MK_SCX_26.4169.4169.3	3	5.371	0.501	1	1944.3	46.875	K.DESQEGKQQYLQSIEDR.E
DHSB_MOUSE	MK_SCX_26.4178.4178.2	2	5.176	0.467	1	1231.4	62.5	K.DESQEGKQQYLQSIEDR.E
DHSB_MOUSE	MK_SCX_34.5559.5559.2	2	2.317	0.183	1	362.7	75	K.IYPLPHMYVIK.D
DHSB_MOUSE	MK_SCX_36.3693.3693.2	2	2.667	0.159	1	314.9	68.181816	K.QQYLQSIEDREK.L
DHSB_MOUSE	MK_SCX_38.3994.3994.2	2	3.967	0.376	1	1298.2	86.36364	K.IKNEVDSTLTFR.R
DHSB_MOUSE	MK_SCX_38.4184.4184.3	3	4.129	0.359	1	413	50	K.IKNEVDSTLTFR.R
DHSB_MOUSE	MK_SCX_48.3711.3711.3	3	5.365	0.415	1	2172.4	48.52941	K.KDESQEGKQQYLQSIEDR.E
DHSB_MOUSE	MK_SCX_48.3712.3712.2	2	4.467	0.391	1	1828.5	64.70589	K.KDESQEGKQQYLQSIEDR.E
DHSB_MOUSE	MK_SCX_60.4339.4339.2	2	2.004	0.184	1	350.6	71.42857	R.RIDTDLSK.V
DHSO_MOUSE	MK_SCX_13.9271.9271.2	2	2.445	0.231	1	322.2	47.058823	K.VLVCGAGPVGMVTLLVAK.A
DHSO_MOUSE	MK_SCX_17.6599.6599.2	2	4.789	0.506	1	847.2	68.75	R.LENYPIPELGPNDVLLK.M
DHSO_MOUSE	MK_SCX_17.7720.7720.2	2	2.498	0.32	1	331	35	R.YNLTPTIFFCATPPDDGNLCR.F
DHSO_MOUSE	MK_SCX_19.4350.4350.2	2	5.394	0.593	1	2790.8	83.33333	K.AM*GAAQVVVTDLSASR.L

DHSO_MOUSE	MK_SCX_19.4962.4962.2	2	3.164	0.13	1	420.4	46.666668	K.AMGAAQVVVTDLSASR.L
DHSO_MOUSE	MK_SCX_20_1.15664.15664.2	2	2.442	0.43	1	486.3	63.636364	K.EVGADFTIQVGK.E
DHSO_MOUSE	MK_SCX_2201.2723.2723.2	2	3.137	0.517	1	796.9	93.75	K.AVEAFETAK.K
DHSO_MOUSE	MK_SCX_2201.3599.3599.2	2	3.013	0.43	1	807.8	87.5	R.VAIEPGVPR.E
DHSO_MOUSE	MK_SCX_25.5453.5453.2	2	3.979	0.49	1	499.8	42.5	K.EVGADFTIQVGKETPQEIASK.V
DHSO_MOUSE	MK_SCX_32.4651.4651.2	2	4.183	0.517	1	1407.7	71.42857	K.GENLSLVVHGPGDIR.L
DHSO_MOUSE	MK_SCX_32.4711.4711.3	3	3.948	0.525	1	1191.6	51.785713	K.GENLSLVVHGPGDIR.L
DHSO_MOUSE	MK_SCX_34.4835.4835.2	2	3.274	0.265	1	518.4	61.538464	K.AKEVGADFTIQVGK.E
DHSO_MOUSE	MK_SCX_51.4389.4389.3	3	5.717	0.591	1	853	40.476192	R.IGDFVVKKPM*VLGHEAAGTVTK.V
DHSO_MOUSE	MK_SCX_51.4408.4408.2	2	4.758	0.524	1	725.1	57.14286	R.IGDFVVKKPM*VLGHEAAGTVTK.V
DHSO_MOUSE	MK_SCX_51.4771.4771.3	3	6.71	0.632	1	2094.2	45.238094	R.IGDFVVKKPMVLGHEAAGTVTK.V
DHSO_MOUSE	MK_SCX_51.4790.4790.2	2	5.713	0.53	1	1000.9	59.523808	R.IGDFVVKKPMVLGHEAAGTVTK.V
DHSO_MOUSE	MK_SCX_52.2959.2959.3	3	3.958	0.468	1	826.7	46.42857	K.KPMVLGHEAAGTVTK.V
DHSO_MOUSE	MK_SCX_55.4913.4913.2	2	4.258	0.589	1	753	66.66667	K.TLNVKPLVTHRFPLEK.A
DHSO_MOUSE	MK_SCX_55.4972.4972.3	3	4.02	0.484	1	880	45	K.TLNVKPLVTHRFPLEK.A
DHX30_MOUSE	MK_SCX_2201.2808.2808.2	2	2.618	0.345	1	902	83.33333	K.SVEVEGYGSK.K
DHX30_MOUSE	MK_SCX_29.7416.7416.3	3	3.735	0.226	1	599.9	31.25	R.FGSPADSWWRPEPTMPPTSWR.Q
DHX30_MOUSE	MK_SCX_32.7066.7066.2	2	2.038	0.209	1	318.1	39.285713	K.GVLMAGLYPNLIQVR.Q
DIC_MOUSE	MK_SCX_17.8273.8273.3	3	5.372	0.494	1	976	34.782608	K.VLLGGISGLTGGFVGTPADLVNVR.M
DIC_MOUSE	MK_SCX_17.8279.8279.2	2	4.535	0.603	1	1237.4	56.521736	K.VLLGGISGLTGGFVGTPADLVNVR.M
DIC_MOUSE	MK_SCX_19.10848.10848.3	3	3.422	0.371	1	444.2	22.058825	K.DSQGPLPFYNKVLLGGISGLTGGFVGTPADLVNVR.M
DIC_MOUSE	MK_SCX_42.3959.3959.3	3	3.207	0.44	1	529.1	50	R.NYSHALDGLYR.V
DIC_MOUSE	MK_SCX_42.3977.3977.2	2	3.293	0.561	1	1306	85	R.NYSHALDGLYR.V
DJC12_MOUSE	MK_SCX_19.8062.8062.2	2	5.788	0.578	1	984.3	76.47059	R.SQMSMPFEQWEALADSVK.T
DJC12_MOUSE	MK_SCX_25.4967.4967.3	3	3.399	0.468	1	754.2	38.157894	K.DLMLEGSGQTFTSSVPNKER.S
DJC12_MOUSE	MK_SCX_25.4979.4979.2	2	5.042	0.626	1	1008.8	55.263157	K.DLMLEGSGQTFTSSVPNKER.S
DJC12_MOUSE	MK_SCX_34.8584.8584.3	3	3.422	0.292	1	471.4	33.333336	R.RSQMSMPFEQWEALADSVK.T
DJC12_MOUSE	MK_SCX_42.5456.5456.3	3	3.561	0.285	1	1236	54.166668	R.FRWSGDAPSELLR.K
DJC12_MOUSE	MK_SCX_42.5458.5458.2	2	4.188	0.367	1	800.7	79.16667	R.FRWSGDAPSELLR.K
DKC1_MOUSE	MK_SCX_19.6687.6687.2	2	3.421	0.246	1	1380.9	73.07692	K.VAQLDTSQWPLLLK.N
DKC1_MOUSE	MK_SCX_31.8471.8471.2	2	2.111	0.191	1	557.1	54.166668	R.VEKTGHSGTLDPK.V
DLC2A_MOUSE	MK_SCX_26.7176.7176.2	2	4.383	0.594	1	1052.9	52.499996	K.STMDNPTTTQYANLMHNFILK.A
DLDH_MOUSE	MK_SCX_18.7807.7807.2	2	2.801	0.186	1	401.4	42.857143	K.AEVITCDVLLVCIGR.R
DLDH_MOUSE	MK_SCX_20_1.3528.3528.2	2	4.292	0.494	1	1994.6	90.909096	K.IDVSVEAASGGK.A
DLDH_MOUSE	MK_SCX_20_1.5221.5221.2	2	3.82	0.343	1	899.5	59.375	K.NETLGGTCLNVGCIPSK.A
DLDH_MOUSE	MK_SCX_21.2183.2183.2	2	2.908	0.358	1	599.6	75	K.ADGSTQVIDTK.N
DLDH_MOUSE	MK_SCX_25.8939.8939.3	3	5.931	0.578	1	861.1	24.342104	K.AEDEGIICVEGMAGGAVHIDYNCVPSVIYTHPEVAWVGK.S
DLDH_MOUSE	MK_SCX_26.6639.6639.3	3	5.801	0.517	1	3225.8	52.77778	K.IPNIYAIGDVVAGPM*LAHK.A
DLDH_MOUSE	MK_SCX_26.6689.6689.2	2	5.449	0.596	1	1920.2	63.88889	K.IPNIYAIGDVVAGPM*LAHK.A
DLDH_MOUSE	MK_SCX_26.7807.7807.3	3	6.343	0.52	1	4063.1	61.11111	K.IPNIYAIGDVVAGPMLAHK.A
DLDH_MOUSE	MK_SCX_26.7824.7824.2	2	6.241	0.597	1	2782.6	77.77778	K.IPNIYAIGDVVAGPMLAHK.A
DLDH_MOUSE	MK_SCX_29.3268.3268.2	2	4.278	0.446	1	963	58.823532	K.NQVTATKADGSTQVIDTK.N
DLDH_MOUSE	MK_SCX_29.3278.3278.3	3	5.509	0.549	1	1290.7	47.058823	K.NQVTATKADGSTQVIDTK.N
DLDH_MOUSE	MK_SCX_30.3353.3353.3	3	4.696	0.457	1	1969.8	53.333336	K.SDGKIDVSVEAASGGK.A
DLDH_MOUSE	MK_SCX_32.16039.16039.3	3	5.109	0.336	1	1387.2	43.055553	R.RPFTQNLGLEELGIELDPK.G
DLDH_MOUSE	MK_SCX_32.3775.3775.2	2	4.085	0.432	1	1975.7	83.33333	K.SEEQLKEEGIEFK.I
DLDH_MOUSE	MK_SCX_32.7584.7584.2	2	4.315	0.502	1	403.6	58.333332	R.RPFTQNLGLEELGIELDPK.G
DLDH_MOUSE	MK_SCX_33.3762.3762.3	3	4.534	0.483	1	1065.3	60.416668	K.SEEQLKEEGIEFK.I
DLDH_MOUSE	MK_SCX_39.5503.5503.2	2	3.635	0.416	1	692.9	75	K.ALTGGIAHLFK.Q
DLDH_MOUSE	MK_SCX_42.3288.3288.3	3	6.077	0.623	1	1499.1	45.238094	K.ITGKNQVTATKADGSTQVIDTK.N
DLDH_MOUSE	MK_SCX_43.3991.3991.3	3	3.787	0.253	1	2309.1	65	K.IGKFPFAANSR.A

DLDH_MOUSE	MK_SCX_43.4005.4005.2	2	2.431	0.412	1	966.8	85	K.IGKFPFAANSR.A
DLDH_MOUSE	MK_SCX_49.3146.3146.3	3	4.69	0.592	1	1121.2	45.3125	K.KSDGKIDVSVEAASGGK.A
DLDH_MOUSE	MK_SCX_57.2915.2915.3	3	3.648	0.261	1	610	45.454548	K.GRIPVNNRFQTK.I
DLG1_MOUSE	MK_SCX_19.5317.5317.2	2	2.872	0.215	1	758.5	50	K.QIIEEQSGPYIWVPAK.E
DLG1_MOUSE	MK_SCX_2201.2287.2287.2	2	4.061	0.512	1	1829.5	95.454544	K.IITGGAAAQDGR.L
DLG1_MOUSE	MK_SCX_28.3886.3886.3	3	3.467	0.501	1	836.5	41.17647	R.QVTPDGESDEVGVIPSKR.R
DLG1_MOUSE	MK_SCX_48.8066.8066.3	3	3.48	0.295	1	817.2	42.1875	K.RLQIAQLYPISIFIKPK.S
DMXL1_MOUSE	MK_SCX_10.10412.10412.2	2	2.522	0.254	1	357.3	44.11765	K.ENQAFPLWDSTKIVPLSK.F
DMXL1_MOUSE	MK_SCX_20_1.3148.3148.2	2	2.547	0.283	1	542.8	50	R.ESPVSSSSGNQEPPAVK.E
DMXL1_MOUSE	MK_SCX_25.6560.6560.3	3	3.159	0.348	1	881.7	46.42857	R.NNDPLDAAIFYLAMK.K
DNJA1_MOUSE	MK_SCX_17.10551.10551.2	2	5.665	0.693	1	903.8	47.826088	K.EGGAGGGFGSPMDIFDMFFGGGGR.M
DNJA1_MOUSE	MK_SCX_17.8795.8795.2	2	4.609	0.623	1	873.9	47.826088	K.EGGAGGGFGSPM*DIFDM*FFGGGGR.M
DNJA1_MOUSE	MK_SCX_17.9529.9529.2	2	4.114	0.26	1	740	41.304348	K.EGGAGGGFGSPM*DIFDMFFGGGGR.M
DNJA1_MOUSE	MK_SCX_17.9679.9679.2	2	5.552	0.166	1	1009.1	50	K.EGGAGGGFGSPMDIFDM*FFGGGGR.M
DNJA1_MOUSE	MK_SCX_17.9741.9741.3	3	4.393	0.3	1	1393.4	40.217392	K.EGGAGGGFGSPMDIFDM*FFGGGGR.M
DNJA1_MOUSE	MK_SCX_21.4519.4519.2	2	3.767	0.417	1	794.3	87.5	K.QISQAYEVLADSK.K
DNJA1_MOUSE	MK_SCX_24.7886.7886.2	2	3.437	0.331	1	844.9	58.333332	K.VNFPENGFLSPDKLSLLEK.L
DNJA1_MOUSE	MK_SCX_35.5932.5932.3	3	3.088	0.196	1	815.6	46.42857	K.FKQISQAYEVLADSK.K
DNJA1_MOUSE	MK_SCX_44.5799.5799.3	3	5.542	0.393	1	1000.7	35.416664	R.IHQIGPGMVQQIQSVCMECQGHGER.I
DNJA2_MOUSE	MK_SCX_15.7964.7964.2	2	5.429	0.686	1	730.6	50	R.ITFTGEADQAPGVEPGDIVLLLQEK.E
DNJA2_MOUSE	MK_SCX_34.8568.8568.2	2	2.483	0.213	1	429.8	62.5	K.EYHPDKNPNAGDK.F
DNJA3_MOUSE	MK_SCX_17.6248.6248.2	2	4.587	0.507	1	749.7	57.5	K.AQGLYETINVTIPAGIQTDQK.I
DNJA3_MOUSE	MK_SCX_19.7288.7288.2	2	3.891	0.507	1	1895.3	76.666664	K.FSQLAEAYEVLSDEVK.R
DNJA3_MOUSE	MK_SCX_20_1.5706.5706.2	2	2.953	0.418	1	497.3	68.181816	R.GGPSVDPEELFR.K
DNJA3_MOUSE	MK_SCX_29.7844.7844.2	2	4.596	0.227	1	1579.1	62.5	K.FSQLAEAYEVLSDEVKR.K
DNJA3_MOUSE	MK_SCX_44.7320.7320.3	3	4.126	0.537	1	1405.9	48.61111	K.EKFSQLAEAYEVLSDEVKR.K
DNJA3_MOUSE	MK_SCX_51.5327.5327.2	2	4.027	0.501	1	919.6	47.727272	K.VQHCHYCGGSGMETINTGPFVMR.S
DNJB4_MOUSE	MK_SCX_31.5759.5759.2	2	3.579	0.578	1	691.8	67.85714	R.NLPMSVTDIVKPGMR.R
DNJBB_MOUSE	MK_SCX_19.5020.5020.2	2	4.044	0.406	1	1043.1	63.333332	R.FQMTQEVVCDECPNVK.L
DNJBB_MOUSE	MK_SCX_21.4503.4503.2	2	2.285	0.145	1	448.9	55	R.TLEVEIEPGVR.D
DNJBB_MOUSE	MK_SCX_25.7405.7405.3	3	3.479	0.419	1	356.8	21.969696	R.TLEVEIEPGVRDGMEYPFIGEGEPHVDGEPGDLR.F
DNJBB_MOUSE	MK_SCX_28.5838.5838.3	3	3.443	0.502	1	864.9	45.3125	K.FQDLGAAYEVLSDSEKR.K
DNJBB_MOUSE	MK_SCX_40.3872.3872.3	3	5.387	0.564	1	1616.3	50	K.LALQLHPDRNPDDPQAQEK.F
DNJC3_MOUSE	MK_SCX_23.8427.8427.2	2	5.046	0.633	1	2159.5	73.52941	R.SWNSWQGFNPFSSGGPFR.F
DNJC3_MOUSE	MK_SCX_30.5808.5808.2	2	3.579	0.331	1	857.7	44.736843	K.AALPDLTKVIALKMDFTAAR.L
DNJC3_MOUSE	MK_SCX_31.3812.3812.2	2	5.798	0.584	1	1347.7	71.875	K.SNPSEQEEKEAESQLVK.A
DNJC3_MOUSE	MK_SCX_50.4601.4601.3	3	3.918	0.375	1	1083.6	46.875	R.KLALQWHPDNFQNEEEK.K
DNJC3_MOUSE	MK_SCX_53.4510.4510.3	3	6.335	0.605	1	1774	38.541664	R.KKFDDGEDPLDAESQQGGGGNPFHR.S
DNJC7_MOUSE	MK_SCX_23.5568.5568.2	2	2.299	0.215	1	887.9	77.77778	K.AVQFFVQALR.M
DNJC7_MOUSE	MK_SCX_34.3363.3363.3	3	3.176	0.487	1	435.2	35.714287	R.EAESFKEQGNAYYAK.K
DNJC7_MOUSE	MK_SCX_39.3469.3469.2	2	2.319	0.242	1	521.7	68.181816	K.KDYNEAYNYYTK.A
DNJC7_MOUSE	MK_SCX_41.3355.3355.3	3	3.054	0.312	1	1398.7	50	R.FREALGDAQQSVR.L
DNJC7_MOUSE	MK_SCX_44.4944.4944.2	2	2.083	0.172	1	378	60.000004	K.QLLKNAQLELK.K
DNJC8_MOUSE	MK_SCX_2201.9713.9713.3	3	5.735	0.496	1	813.1	26.785713	R.LTRPGSSYFNLNPFEVLQIDPEVTDEEIK.K
DNJC8_MOUSE	MK_SCX_48.3738.3738.3	3	3.647	0.422	1	1183.1	45.3125	K.KEGKPTNVEEDDPELFK.Q
DNM1L_MOUSE	MK_SCX_15.9292.9292.2	2	4.959	0.572	1	699.8	40.74074	K.LQDVFNTVGADIIQLPQIVVVGTQSSGK.S
DNM1L_MOUSE	MK_SCX_15.9308.9308.3	3	5.701	0.579	1	2326.3	37.962963	K.LQDVFNTVGADIIQLPQIVVVGTQSSGK.S
DNM1L_MOUSE	MK_SCX_17.7948.7948.2	2	3.746	0.433	1	508.1	52.77778	K.VFSPNVVNLTLVDLPGMTK.V
DNM1L_MOUSE	MK_SCX_48.6659.6659.3	3	3.537	0.481	1	430.5	24.074074	K.SKPIPIMPASPQKGHAVNLLDVPVPVAR.K
DOC10_MOUSE	MK_SCX_14.8674.8674.2	2	2.232	0.124	1	339.4	41.17647	R.NM*GRLNLFSLDPDIDTLK.L
DOC10_MOUSE	MK_SCX_31.4338.4338.2	2	2.464	0.211	1	373.8	43.75	R.YELIADVNKPIIAVFEK.Q

DOPD_MOUSE	MK_SCX_19.4300.4300.2	2	4.572	0.453	1	1288	71.42857	R.LCAATATILDKPEDR.V
DOPD_MOUSE	MK_SCX_20_1.5623.5623.2	2	3.786	0.479	1	740.8	72.72727	K.FLTEELSLDQDR.I
DOPD_MOUSE	MK_SCX_20_1.5775.5775.2	2	4.571	0.363	1	1445.1	75	-.PFVELETNLPASR.I
DOPD_MOUSE	MK_SCX_2201.7363.7363.3	3	3.627	0.375	1	526.1	52.499996	R.FFPLEAWQIGK.K
DOPD_MOUSE	MK_SCX_2201.7462.7462.2	2	3.684	0.524	1	916	90	R.FFPLEAWQIGK.K
DOPD_MOUSE	MK_SCX_27.7190.7190.3	3	4.659	0.62	1	1669.2	41.25	-.PFVELETNLPASRIPAGLENR.L
DOPD_MOUSE	MK_SCX_32.5170.5170.3	3	3.428	0.342	1	703.4	44.642857	R.VSVTIRPGMTLLM*NK.S
DOPD_MOUSE	MK_SCX_32.5181.5181.2	2	3.95	0.347	1	462.9	64.28571	R.VSVTIRPGMTLLM*NK.S
DOPD_MOUSE	MK_SCX_32.8148.8148.2	2	2.508	0.128	1	812.7	53.571426	R.VSVTIRPGMTLLMNK.S
DOPD_MOUSE	MK_SCX_36.6692.6692.2	2	3.138	0.44	1	442.4	72.72727	R.FFPLEAWQIGKK.G
DPEP1_MOUSE	MK_SCX_18.11815.11815.3	3	6.089	0.668	1	948	26.35135	K.VAGAGAVGLGGDYDGVTM*LPVGLEDVSKYPDLIAELLR.R
DPEP1_MOUSE	MK_SCX_21.8379.8379.2	2	2.837	0.388	1	1018.3	83.33333	K.YPDLIAELLR.R
DPEP1_MOUSE	MK_SCX_26.7515.7515.3	3	5.178	0.663	1	2240.1	47.5	K.VASLIGVEGGHLIDSSLGVLR.T
DPEP1_MOUSE	MK_SCX_33.4228.4228.3	3	3.722	0.393	1	403.9	52.77778	R.ILEQMDVIHR.M
DPEP1_MOUSE	MK_SCX_33.4251.4251.2	2	3.569	0.428	1	1537.8	88.88889	R.ILEQMDVIHR.M
DPEP1_MOUSE	MK_SCX_41.7603.7603.3	3	5.87	0.656	1	2758.7	46.590908	R.GKVASLIGVEGGHLIDSSLGVLR.T
DPP2_MOUSE	MK_SCX_20_1.3689.3689.2	2	4.242	0.454	1	1084.1	73.07692	R.ASNSEDPPSVVEVR.K
DPP2_MOUSE	MK_SCX_41.5282.5282.2	2	2.301	0.34	1	498.2	68.75	R.FLVSDKFWK.M
DPP4_MOUSE	MK_SCX_19.4824.4824.2	2	3.663	0.39	1	416.8	65.38461	R.LGTLEVEDQIEAAR.Q
DPP4_MOUSE	MK_SCX_33.7966.7966.3	3	3.112	0.159	1	880	45.833336	R.FWYQMILPPHFDK.S
DPP4_MOUSE	MK_SCX_51.4500.4500.3	3	3.351	0.33	1	923.2	43.75	R.FRPAEPHFTSDGSSFYK.I
DPP4_MOUSE	MK_SCX_51.4524.4524.2	2	4.201	0.399	1	551.9	56.25	R.FRPAEPHFTSDGSSFYK.I
DPP4_MOUSE	MK_SCX_53.3788.3788.3	3	4.709	0.507	1	1567.8	51.785713	R.HSYTASYNIYDVNKR.Q
DPY30_MOUSE	MK_SCX_16.10947.10947.3	3	6.355	0.548	1	2671.2	50	R.AYLDQTVVPILLQGLAVLAK.E
DPY30_MOUSE	MK_SCX_16.10973.10973.2	2	5.726	0.634	1	1133	73.68421	R.AYLDQTVVPILLQGLAVLAK.E
DPY30_MOUSE	MK_SCX_31.16443.16443.2	2	4.367	0.413	1	1554.5	70	K.ERPPNPIEFLASYLLK.N
DPY30_MOUSE	MK_SCX_32.15999.15999.3	3	4.157	0.32	1	1267.1	43.333332	K.ERPPNPIEFLASYLLK.N
DPYL2_MOUSE	MK_SCX_17.9111.9111.3	3	3.632	0.566	1	374.7	20.930233	K.AAAFVTSPPLSPDPTTPDFLNSLLSCGDLQVTGSAHCTFNTAQK.A
DPYL2_MOUSE	MK_SCX_2201.3109.3109.2	2	3.73	0.378	1	1446.7	88.88889	K.SAAEVIAQAR.K
DPYL2_MOUSE	MK_SCX_32.5427.5427.3	3	4.04	0.412	1	319.5	30.263159	R.NLHQSGFSLSGAQIDDNIPR.R
DPYL2_MOUSE	MK_SCX_32.6723.6723.3	3	4.817	0.578	1	721.1	31.730768	R.ILDLGITGPEGHVLSRPEEVEAEAVNR.S
DPYL2_MOUSE	MK_SCX_55.4126.4126.3	3	3.211	0.395	1	721.4	52.77778	R.KPFPDFVYKR.I
DREB_MOUSE	MK_SCX_33.3181.3181.3	3	4.386	0.416	1	1131.8	50	R.LREDENAEPVGTTYQK.T
DRG1_MOUSE	MK_SCX_2201.3810.3810.2	2	3.28	0.433	1	855.1	88.88889	K.IAEIEAEMAR.T
DRG1_MOUSE	MK_SCX_2201.5604.5604.2	2	3.456	0.35	1	845.9	80	R.IGFVGFPSVGK.S
DSG2_MOUSE	MK_SCX_18.3635.3635.2	2	4.157	0.496	1	1351	71.42857	K.ITATDADDPETLNAK.V
DUS3_MOUSE	MK_SCX_16.5851.5851.2	2	3.691	0.384	1	1463.8	65.625	R.VYVGNASVAQDITQLQK.L
DUS3_MOUSE	MK_SCX_36.4198.4198.3	3	4.505	0.444	1	1812.7	60.416668	K.LGITHVLNAAEGR.S
DUS3_MOUSE	MK_SCX_36.4200.4200.2	2	4.145	0.353	1	1659.1	83.33333	K.LGITHVLNAAEGR.S
DYH5_MOUSE	MK_SCX_13.5043.5043.2	2	2.305	0.146	1	390.5	71.42857	K.FHDKIYDR.I
DYH5_MOUSE	MK_SCX_20_1.5713.5713.2	2	2.003	0.334	1	375.4	37.5	R.ASDVQNELGALQPSFRK.E
DYHC_MOUSE	MK_SCX_16.8229.8229.2	2	3.415	0.598	1	486.7	40.909092	R.FGNPLLVQDVESYDPVLNPVLNR.E
DYHC_MOUSE	MK_SCX_18.7231.7231.2	2	2.525	0.135	2	381.9	42.857143	R.LRSIPLDEGEDEAQR.R
DYHC_MOUSE	MK_SCX_24.6508.6508.3	3	5.819	0.568	1	2959.1	44.31818	K.VTDFGDKVEDPTFLNQLQSGVNR.W
DYHC_MOUSE	MK_SCX_26.8480.8480.3	3	4.804	0.53	1	2097.6	41.304348	R.KLVPLLLEDGGDAPAALEAALEEK.S
DYHC_MOUSE	MK_SCX_26.8553.8553.2	2	6.085	0.663	1	2242	65.21739	R.KLVPLLLEDGGDAPAALEAALEEK.S
DYHC_MOUSE	MK_SCX_27.4077.4077.3	3	5.352	0.528	1	758	35.869564	R.VLRPQVTAVAQQNQGEAPEPQDM*K.V
DYHC_MOUSE	MK_SCX_27.4373.4373.3	3	6.533	0.529	1	1984.4	43.47826	R.VLRPQVTAVAQQNQGEAPEPQDMK.V
DYHC_MOUSE	MK_SCX_30.3752.3752.3	3	3.32	0.476	1	1241.1	48.214287	R.APVIDADKPVSSQLR.V
DYHC_MOUSE	MK_SCX_30.4639.4639.2	2	4.212	0.453	1	1346.7	84.61539	K.FLSDPQVHTVLVER.S
DYHC_MOUSE	MK_SCX_35.9553.9553.2	2	2.143	0.211	1	389.5	61.11111	K.DIQMPDGIRR.E

DYHC_MOUSE	MK_SCX_41.6762.6762.3	3	3.628	0.547	1	408.4	32.954548	K.TKPVTGNLRPEEALQALTIYEGK.F
DYHC_MOUSE	MK_SCX_49.4640.4640.3	3	3.94	0.323	1	869.1	48.214287	R.KFLSDPQVHTVLVER.S
DYHC_MOUSE	MK_SCX_51.3514.3514.3	3	3.391	0.465	1	857.7	46.666668	K.RAPVIDADKPVSSQLR.V
DYL2_MOUSE	MK_SCX_36.6483.6483.2	2	3.929	0.426	1	1714.4	86.36364	K.YNIEKDIAAYIK.K
DYL2_MOUSE	MK_SCX_36.6624.6624.3	3	3.109	0.222	1	1120.1	56.81818	K.YNIEKDIAAYIK.K
DYL2_MOUSE	MK_SCX_51.5544.5544.2	2	4.661	0.43	1	2482.5	87.5	K.YNIEKDIAAYIKK.E
DYLT1_MOUSE	MK_SCX_19.8599.8599.2	2	4.828	0.52	1	1161.7	70.588234	K.VNQWTTNVLEQTLSQLTK.L
DYNA_MOUSE	MK_SCX_16.4848.4848.2	2	5.17	0.541	1	700.7	43.75	R.QSQIQVFEDGADTTSPETPDSSASK.V
DYNA_MOUSE	MK_SCX_21.4640.4640.2	2	3.357	0.407	1	835.1	69.230774	K.SPSAQLM*EQVAQLK.S
DYR_MOUSE	MK_SCX_16.7211.7211.2	2	5.255	0.619	1	1462.8	70.588234	R.IM*QEFESDTFFPEIDLGK.Y
DYR_MOUSE	MK_SCX_21.3816.3816.2	2	2.678	0.397	1	786.6	83.33333	R.LIEQPELASK.V
DYR_MOUSE	MK_SCX_25.5079.5079.2	2	2.233	0.188	1	349.3	71.42857	K.TWFSIPEK.N
DYR_MOUSE	MK_SCX_25.7937.7937.2	2	5.092	0.596	1	1015.6	57.894737	R.IMQEFESDTFFPEIDLGKYK.L
DYR_MOUSE	MK_SCX_25.8007.8007.3	3	4.595	0.416	1	788.2	38.157894	R.IMQEFESDTFFPEIDLGKYK.L
DYR_MOUSE	MK_SCX_30.6466.6466.3	3	4.57	0.411	1	2447	50	K.YKLLPEYPGVLSEVQEEK.G
DYSF_MOUSE	MK_SCX_28.4807.4807.2	2	3.383	0.243	1	339.5	38.235294	R.M*PVQIRIKLWFGLSVDEK.E
E41L1_MOUSE	MK_SCX_19.3233.3233.2	2	4.611	0.624	1	1103.1	71.875	R.VSAADSTQVDGGTPM*VK.D
E41L1_MOUSE	MK_SCX_19.3581.3581.2	2	5.432	0.606	1	2450.9	81.25	R.VSAADSTQVDGGTPMVK.D
E41L1_MOUSE	MK_SCX_19.4728.4728.2	2	3.197	0.43	1	556.6	59.375	K.GAAAM*IPGPQTVATEIR.S
E41L1_MOUSE	MK_SCX_19.5110.5110.2	2	3.551	0.389	1	606.5	62.5	K.GAAAMIPGPQTVATEIR.S
E41L1_MOUSE	MK_SCX_23.3547.3547.3	3	5.327	0.447	1	1212.2	41.25	R.DLKGPSSQEDESGGLEDSPDR.G
E41L1_MOUSE	MK_SCX_32.5207.5207.3	3	4.113	0.506	1	1194.9	46.05263	K.SGKGAAAMIPGPQTVATEIR.S
E41L2_MOUSE	MK_SCX_14.7000.7000.3	3	4.066	0.468	1	532.2	26.470589	R.SLDGAPIGVVDQSPPGEGSVPGPGVISYTTIQDGR.R
E41L2_MOUSE	MK_SCX_14.7030.7030.2	2	5.426	0.645	1	498.5	35.294117	R.SLDGAPIGVVDQSPPGEGSVPGPGVISYTTIQDGR.R
E41L2_MOUSE	MK_SCX_18.7337.7337.2	2	2.543	0.159	1	344.6	50	K.SSVETQPAEEVRKDK.E
E41L2_MOUSE	MK_SCX_20_1.3459.3459.2	2	2.792	0.401	1	372.1	70	R.EAAVPDAVPDR.Q
E41L2_MOUSE	MK_SCX_23.3826.3826.3	3	4.62	0.468	1	766.5	32	K.EVENEQTPVSEPEEEKGSQPGPPVER.Q
E41L2_MOUSE	MK_SCX_29.3114.3114.3	3	4.1	0.39	1	808.5	39.705883	K.AKEVENEQTPVSEPEEEK.G
E41L2_MOUSE	MK_SCX_31.4198.4198.2	2	3.172	0.349	1	463.7	53.571426	R.EASTLIDRPAPQFER.A
E41L2_MOUSE	MK_SCX_45.7097.7097.3	3	3.542	0.399	1	645.8	39.705883	R.HSNLMLEDLDKAQEAILK.H
E41L3_MOUSE	MK_SCX_17.3546.3546.2	2	4.971	0.525	1	1684.5	71.875	K.DSVSAAEVGTGQYATTK.G
E41L3_MOUSE	MK_SCX_23.4133.4133.3	3	5.323	0.535	1	889.9	37.962963	R.GEEVDQSAPEQEQPATVSHEEEQASTIR.T
E41L3_MOUSE	MK_SCX_24.4376.4376.2	2	5.505	0.685	1	1082.9	57.5	K.TEPVEAEVESTPHPQPLSTEK.V
E41L3_MOUSE	MK_SCX_31.5708.5708.2	2	4.432	0.612	1	926.2	63.333332	R.GMTPAEAEMHFLENAK.K
E41L3_MOUSE	MK_SCX_34.4519.4519.3	3	3.819	0.53	1	905.4	46.153847	R.ASALIDRPAPYFER.S
E41L3_MOUSE	MK_SCX_34.4525.4525.2	2	3.403	0.433	1	465.9	57.692307	R.ASALIDRPAPYFER.S
E41L3_MOUSE	MK_SCX_53.4328.4328.3	3	3.45	0.212	1	1369.7	48.214287	R.RASALIDRPAPYFER.S
EBP2_MOUSE	MK_SCX_15.5716.5716.2	2	4.661	0.556	1	518.7	41.304348	R.LDVTLGPVPEVSETQPTPQNQDQK.K
EBP2_MOUSE	MK_SCX_42.5428.5428.3	3	3.398	0.454	1	988.8	39.705883	K.GFSDKLDFLEGDQKPVER.S
ECH1_MOUSE	MK_SCX_15.9047.9047.2	2	4.555	0.541	1	807.7	47.916664	K.MFTSGIDLMDMASELMQPSGDDAAR.I
ECH1_MOUSE	MK_SCX_15.9130.9130.3	3	3.488	0.356	1	476.5	28.125	K.MFTSGIDLMDMASELMQPSGDDAAR.I
ECH1_MOUSE	MK_SCX_18.4613.4613.2	2	4.102	0.368	1	1539.4	71.42857	K.EVDM*GLAADVGTLQR.L
ECH1_MOUSE	MK_SCX_20_1.15471.15471.3	3	3.169	0.444	1	523.5	38.333332	K.VIGNQSLVNELTFSAR.K
ECH1_MOUSE	MK_SCX_20_1.6773.6773.2	2	4.634	0.465	1	1601.5	70	K.VIGNQSLVNELTFSAR.K
ECH1_MOUSE	MK_SCX_25.12621.12621.2	2	3.164	0.375	1	439.9	42.857143	R.VFQDKDAMLNAAFALAADISSK.S
ECH1_MOUSE	MK_SCX_25.9271.9271.3	3	4.743	0.593	1	1829.8	40.476192	R.VFQDKDAMLNAAFALAADISSK.S
ECH1_MOUSE	MK_SCX_26.4089.4089.2	2	3.776	0.505	1	386.8	44.736843	R.APEEVSDHNYESIQVTSAQK.H
ECH1_MOUSE	MK_SCX_34.4395.4395.2	2	5.382	0.516	1	2826	89.28571	R.KMMADEALDSGLVSR.V
ECH1_MOUSE	MK_SCX_34.4487.4487.3	3	4.501	0.355	1	1974.6	51.785713	R.KMMADEALDSGLVSR.V
ECH1_MOUSE	MK_SCX_44.3728.3728.3	3	6.026	0.541	1	2234.2	41.25	K.RAPEEVSDHNYESIQVTSAQK.H
ECHB_MOUSE	MK_SCX_17.4783.4783.2	2	2.684	0.357	1	653.5	66.66667	K.DQLLLGPTYATPK.V

ECHB_MOUSE	MK_SCX_17.4801.4801.1	1	2.531	0.361	1	352	54.166668	K.DQLLLGPTYATPK.V
ECHB_MOUSE	MK_SCX_21.6234.6234.2	2	4.548	0.646	1	1767.4	76.92308	K.AMDSDWFAQNYMGR.K
ECHB_MOUSE	MK_SCX_2201.2841.2841.2	2	2.788	0.132	1	646.6	81.25	R.M*EQDEYALR.S
ECHB_MOUSE	MK_SCX_2201.3214.3214.2	2	2.43	0.145	1	925.1	87.5	R.MEQDEYALR.S
ECHB_MOUSE	MK_SCX_2201.5412.5412.2	2	3.326	0.278	1	1019.2	85	R.IPFLLSGTSYK.D
ECHB_MOUSE	MK_SCX_23.14901.14901.3	3	3.083	0.256	1	414	24	K.AGLTMNDIDAFEFHEAFSGQILANFK.A
ECHB_MOUSE	MK_SCX_23.15224.15224.3	3	3.139	0.13	1	328.2	24	K.AGLTM*NDIDAFEFHEAFSGQILANFK.A
ECHB_MOUSE	MK_SCX_24.3819.3819.2	2	3.722	0.248	1	1322.5	93.75	R.LAAAFAVSR.M
ECHB_MOUSE	MK_SCX_37.13271.13271.3	3	4.959	0.504	1	403.6	26.85185	K.FRLNFLSPELPAVAEFSTNETM*GHSADR.L
ECHB_MOUSE	MK_SCX_39.10598.10598.3	3	5.089	0.551	1	2037.8	48.684208	R.IPFLLSGTSYKDLMPHDLAR.A
ECHB_MOUSE	MK_SCX_39.7496.7496.3	3	4.494	0.268	1	1284.9	38.157894	R.IPFLLSGTSYKDLM*PHDLAR.A
ECHM_MOUSE	MK_SCX_13.4001.4001.2	2	4.202	0.499	1	1446.2	90	K.LVEEAIQCAEK.I
ECHM_MOUSE	MK_SCX_18.6542.6542.2	2	5.809	0.575	1	1612.1	72.5	K.AQFGQPEILLGTIPGAGGTQR.L
ECHM_MOUSE	MK_SCX_19.10608.10608.2	2	2.844	0.215	1	438.5	56.666668	K.ESVNAAFEMTLTEGNK.L
ECHM_MOUSE	MK_SCX_21.16949.16949.2	2	3.414	0.394	1	1144.8	77.27273	K.SLAMEMVLTGDR.I
ECHM_MOUSE	MK_SCX_21.4693.4693.2	2	2.952	0.407	1	898.4	80	R.LFYSTFATDDR.R
ECHM_MOUSE	MK_SCX_21.4764.4764.2	2	2.471	0.2	1	739.2	68.181816	K.SLAMEM*VLTGDR.I
ECHM_MOUSE	MK_SCX_2201.2464.2464.2	2	2.355	0.309	1	318.5	68.75	R.EGM*TAFVEK.R
ECHM_MOUSE	MK_SCX_23.6896.6896.3	3	4.894	0.448	1	3197.2	37.931034	K.KPVIAAVNGYALGGGCELAM*M*CDIIYAGEK.A
ECHM_MOUSE	MK_SCX_24.13005.13005.3	3	4.545	0.485	1	1360.5	31.896553	K.KPVIAAVNGYALGGGCELAMMCDIIYAGEK.A
ECHM_MOUSE	MK_SCX_30.13040.13040.2	2	3.709	0.551	1	544.2	47.22222	K.ESVNAAFEMTLTEGNKLEK.R
ECHM_MOUSE	MK_SCX_34.3708.3708.2	2	4.663	0.448	1	2591.5	88.46153	K.AFAAGADIKEMQNR.T
ECHM_MOUSE	MK_SCX_35.3186.3186.2	2	3.84	0.472	1	679.8	76.92308	K.AFAAGADIKEM*QNR.T
ECHM_MOUSE	MK_SCX_35.4440.4440.2	2	2.721	0.306	1	526.9	54.545456	R.LFYSTFATDDRR.E
ECHM_MOUSE	MK_SCX_37.12249.12249.3	3	3.248	0.121	1	378.2	22.580645	R.VKKPVIAAVNGYALGGGCELAMMCDIIYAGEK.A
ECHM_MOUSE	MK_SCX_37.3929.3929.2	2	3.877	0.441	1	1182.3	70.83333	K.NSSVGLIQLNRPK.A
ECHM_MOUSE	MK_SCX_42.4341.4341.2	2	3.102	0.469	1	602.8	72.72727	K.RLFYSTFATDDR.R
ECHM_MOUSE	MK_SCX_53.3551.3551.3	3	4.419	0.419	1	549.5	44.642857	K.GKNSSVGLIQLNRPK.A
ECHM_MOUSE	MK_SCX_56.3682.3682.2	2	3.583	0.559	1	1049.3	88.88889	K.FLSHWDHITR.V
ECHP_MOUSE	MK_SCX_13.6472.6472.2	2	4.38	0.463	1	974.6	61.764706	R.LCNPPVNAISPTVITEVR.N
ECHP_MOUSE	MK_SCX_18.5403.5403.2	2	5.154	0.606	1	1821.8	73.68421	K.TASAQPVSSVGVLGLGTMGR.G
ECHP_MOUSE	MK_SCX_19.5480.5480.2	2	3.584	0.349	1	849.8	71.42857	R.QNPDIPQLEPSDYLR.R
ECHP_MOUSE	MK_SCX_19.6719.6719.2	2	5.738	0.5	1	1842.9	80	K.SPTGLTLGSLVDEIQR.Y
ECHP_MOUSE	MK_SCX_19.6746.6746.2	2	3.057	0.401	1	1610.2	67.85714	R.VGFPEVM*LGILPGAR.G
ECHP_MOUSE	MK_SCX_19.6759.6759.2	2	4.53	0.604	1	787.6	82.14286	R.VVGVPVALDLITSGR.H
ECHP_MOUSE	MK_SCX_19.8109.8109.2	2	4.744	0.47	1	2758.1	85.71429	R.VGFPEVMLGILPGAR.G
ECHP_MOUSE	MK_SCX_21.5863.5863.1	1	2.11	0.324	1	552.4	68.75	K.LGILDVVVK.S
ECHP_MOUSE	MK_SCX_23.4912.4912.2	2	2.904	0.437	1	1397.4	88.88889	R.ALQYAFFAEK.S
ECHP_MOUSE	MK_SCX_25.7819.7819.2	2	3.802	0.544	1	832.7	54.761906	R.ILNKPVPSLPNMDSVFAEAIAK.V
ECHP_MOUSE	MK_SCX_26.7607.7607.3	3	4.177	0.577	1	1771.1	42.857143	R.ILNKPVPSLPNMDSVFAEAIAK.V
ECHP_MOUSE	MK_SCX_27.4821.4821.2	2	2.03	0.291	1	370.4	90	K.LFMYLR.G
ECHP_MOUSE	MK_SCX_31.7151.7151.2	2	5.457	0.629	1	1971.1	73.68421	R.HVGGPMYYAASVGLPTVLEK.L
ECHP_MOUSE	MK_SCX_34.3752.3752.3	3	3.883	0.548	1	1308.1	56.25	K.FAQTVIGKPIEPR.R
ECHP_MOUSE	MK_SCX_34.3757.3757.2	2	3.872	0.45	1	1560.7	83.33333	K.FAQTVIGKPIEPR.R
ECHP_MOUSE	MK_SCX_37.14321.14321.3	3	3.71	0.352	1	799.1	35.714287	K.SDPVEEAIKFAQTVIGKPIEPR.R
ECHP_MOUSE	MK_SCX_37.3886.3886.2	2	5.041	0.596	1	1171.5	71.875	R.KGQGLTGPSLPPGTPTR.K
ECHP_MOUSE	MK_SCX_41.4096.4096.2	2	3.511	0.342	1	985.4	90	K.GWYQYDKPLGR.I
ECHP_MOUSE	MK_SCX_42.5244.5244.3	3	3.558	0.491	1	601.5	29.761904	R.LVAQGSPPLKEWQSLAGPHSSK.L
ECHP_MOUSE	MK_SCX_44.6300.6300.3	3	5.105	0.588	1	711.4	35.227272	R.RILNKPVPSLPNM*DSVFAEAIAK.V
ECHP_MOUSE	MK_SCX_44.7324.7324.3	3	6.308	0.587	1	2097.3	46.590908	R.RILNKPVPSLPNMDSVFAEAIAK.V
ECHP_MOUSE	MK_SCX_45.7247.7247.3	3	4.446	0.546	1	1484.4	48.52941	R.HISTDEALKLGILDVVVK.S

ECHP_MOUSE	MK_SCX_52.6863.6863.3	3	4.137	0.434	1	1516.4	48.333332	R.IHKPDPWLSEFLSQYR.E
ECHP_MOUSE	MK_SCX_54.3531.3531.3	3	5.8	0.576	1	1256.4	44.444447	R.SVQASVKHPYEVAIKEEAK.L
EDD1_MOUSE	MK_SCX_14.10497.10497.3	3	5.073	0.461	1	992.3	29.6875	R.GSGLLGSQPQPVIPASVIPEELISQAQVVLQGK.S
EDF1_MOUSE	MK_SCX_32.3714.3714.3	3	4.918	0.463	1	2007.2	55.35714	K.INEKPQVIADYESGR.A
EDF1_MOUSE	MK_SCX_32.3735.3735.2	2	4.505	0.573	1	1044.6	82.14286	K.INEKPQVIADYESGR.A
EEA1_MOUSE	MK_SCX_14.6638.6638.3	3	5.72	0.607	1	1450.9	30.000002	R.VGSQGSDLDSSATPINTVDVNNESSSEGFICPQCMK.S
EEA1_MOUSE	MK_SCX_14.6667.6667.2	2	3.181	0.476	1	610.7	31.428572	R.VGSQGSDLDSSATPINTVDVNNESSSEGFICPQCMK.S
EEA1_MOUSE	MK_SCX_15.8826.8826.3	3	5.542	0.604	1	1446	32.75862	K.SDGLVTDSSAELQALEQQLEEAQTENFNIK.Q
EEA1_MOUSE	MK_SCX_15.8835.8835.2	2	5.886	0.635	1	1151.7	36.206894	K.SDGLVTDSSAELQALEQQLEEAQTENFNIK.Q
EEA1_MOUSE	MK_SCX_19.4631.4631.2	2	6.801	0.548	1	3258	84.375	K.IQAGEGETAVLNQLQEK.N
EEA1_MOUSE	MK_SCX_2201.4009.4009.2	2	2.621	0.249	1	762.1	66.66667	K.ESVSLLEKER.E
EEA1_MOUSE	MK_SCX_2201.4471.4471.2	2	3.582	0.26	1	1617.5	77.27273	K.LTLAQEDLISNR.N
EEA1_MOUSE	MK_SCX_30.5074.5074.3	3	3.321	0.294	1	482.2	33.82353	K.IELNSVKGEVSQAQNTLK.Q
EEA1_MOUSE	MK_SCX_31.3299.3299.3	3	3.316	0.493	1	399.3	35.294117	K.LQQQSSQAAQELAAEKGK.L
EEA1_MOUSE	MK_SCX_38.3337.3337.3	3	5.022	0.312	1	1814.5	53.846157	K.ASKEQALQSLQQQR.Q
EEA1_MOUSE	MK_SCX_39.3392.3392.2	2	4.919	0.42	1	2293.6	80.769226	K.ASKEQALQSLQQQR.Q
EEA1_MOUSE	MK_SCX_41.5068.5068.3	3	5.825	0.595	1	2277.3	52.77778	K.VTHLTEDLNKQTTVIQDLK.T
EEA1_MOUSE	MK_SCX_44.5184.5184.3	3	3.189	0.435	1	511.7	34.72222	R.VDSLKAALEQEKESQQLMR.E
EEA1_MOUSE	MK_SCX_46.3592.3592.3	3	3.528	0.443	1	1105.8	45	R.AAAEQKVTHLTEDLNK.Q
EEA1_MOUSE	MK_SCX_55.2632.2632.3	3	4.565	0.476	1	1346.9	40	K.NQSESHKQAEENLHDQVQEQK.A
EF1A1_MOUSE	MK_SCX_14.9003.9003.2	2	4.008	0.199	1	404.9	29.032257	K.IGYNPDTVAFVPISGWNGDNMLEPSANM*PWFK.G
EF1A1_MOUSE	MK_SCX_14.9167.9167.2	2	4.163	0.14	1	487.1	30.645163	K.IGYNPDTVAFVPISGWNGDNM*LEPSANMPWFK.G
EF1A1_MOUSE	MK_SCX_15.7534.7534.2	2	3.574	0.502	1	439.7	40.74074	K.SGDAAIVDMVPGKPMCVESFSDYPPLGR.F
EF1A1_MOUSE	MK_SCX_19.9829.9829.3	3	3.045	0.278	1	315	22.580645	K.IGYNPDTVAFVPISGWNGDNMLEPSANMPWFK.G
EF1A1_MOUSE	MK_SCX_19.9851.9851.2	2	4.506	0.475	1	552.8	32.258064	K.IGYNPDTVAFVPISGWNGDNMLEPSANMPWFK.G
EF1A1_MOUSE	MK_SCX_21.6933.6933.3	3	3.516	0.323	1	919.2	28.703705	K.SGDAAIVDMVPGKPMCVESFSDYPPLGR.F
EF1A1_MOUSE	MK_SCX_23.3914.3914.2	2	3.652	0.489	1	1302.8	95	K.IGGIGTVPVGR.V
EF1A1_MOUSE	MK_SCX_23.6378.6378.3	3	5.616	0.482	1	1109.1	39.130436	R.VETGVLKPGM*VVTFAPVNVTTEVK.S
EF1A1_MOUSE	MK_SCX_23.6694.6694.2	2	4.341	0.494	1	500.6	41.304348	R.VETGVLKPGM*VVTFAPVNVTTEVK.S
EF1A1_MOUSE	MK_SCX_23.8040.8040.2	2	5.614	0.666	1	840.5	56.521736	R.VETGVLKPGMVVTFAPVNVTTEVK.S
EF1A1_MOUSE	MK_SCX_23.8066.8066.3	3	5.687	0.418	1	1377.8	42.391304	R.VETGVLKPGMVVTFAPVNVTTEVK.S
EF1A1_MOUSE	MK_SCX_24.11174.11174.3	3	5.756	0.618	1	1284.6	32.03125	K.KIGYNPDTVAFVPISGWNGDNMLEPSANMPWFK.G
EF1A1_MOUSE	MK_SCX_31.5916.5916.3	3	4.122	0.483	1	1307.7	54.166668	R.YEEIVKEVSTYIK.K
EF1A1_MOUSE	MK_SCX_31.6002.6002.2	2	4.943	0.481	1	2482	91.66667	R.YEEIVKEVSTYIK.K
EF1A1_MOUSE	MK_SCX_34.4244.4244.2	2	3.164	0.522	1	951.2	72.72727	K.YYVTIIDAPGHR.D
EF1A1_MOUSE	MK_SCX_36.15553.15553.3	3	3.542	0.299	1	500.4	45.454548	R.EHALLAYTLGVK.Q
EF1A1_MOUSE	MK_SCX_36.4641.4641.2	2	3.361	0.532	1	763.2	77.27273	R.EHALLAYTLGVK.Q
EF1A1_MOUSE	MK_SCX_46.5462.5462.3	3	4.364	0.582	1	914	51.923077	R.YEEIVKEVSTYIKK.I
EF1A1_MOUSE	MK_SCX_46.5477.5477.2	2	5.014	0.497	1	2600.9	88.46153	R.YEEIVKEVSTYIKK.I
EF1A1_MOUSE	MK_SCX_48.5356.5356.2	2	5.536	0.547	1	1363.9	76.666664	K.YYVTIIDAPGHRDFIK.N
EF1A1_MOUSE	MK_SCX_51.3212.3212.3	3	3.343	0.401	1	578.9	44.230766	R.TIEKFEKEAAEM*GK.G
EF1A1_MOUSE	MK_SCX_51.3224.3224.2	2	3.993	0.646	1	708.1	76.92308	R.TIEKFEKEAAEM*GK.G
EF1A1_MOUSE	MK_SCX_51.3898.3898.2	2	4.235	0.489	1	1920.6	80.769226	R.TIEKFEKEAAEMGK.G
EF1A1_MOUSE	MK_SCX_51.3972.3972.3	3	3.411	0.412	1	471.9	44.230766	R.TIEKFEKEAAEMGK.G
EF1A1_MOUSE	MK_SCX_52.5869.5869.3	3	3.474	0.359	1	728.4	42.307693	K.RYEEIVKEVSTYIK.K
EF1B_MOUSE	MK_SCX_19.3301.3301.2	2	5.484	0.575	1	2551.4	82.35294	K.YGPSSVEDTTGSGAADAK.D
EF1B_MOUSE	MK_SCX_19.6167.6167.2	2	5.092	0.601	1	2444.9	85.71429	K.TPAGLQVLNDYLADK.S
EF1B_MOUSE	MK_SCX_2201.4912.4912.2	2	3.997	0.575	1	1359.1	79.16667	R.SIQADGLVWGSSK.L
EF1B_MOUSE	MK_SCX_25.6344.6344.2	2	5.803	0.583	1	1399.6	73.52941	K.SSILLDVKPWDDETDMTK.L
EF1B_MOUSE	MK_SCX_25.6349.6349.3	3	3.929	0.535	1	1328.7	48.52941	K.SSILLDVKPWDDETDMTK.L
EF1B_MOUSE	MK_SCX_26.7788.7788.2	2	5.086	0.655	1	1579.6	72.5	-.GFGDLKTPAGLQVLNDYLADK.S

EF1B_MOUSE	MK_SCX_26.7791.7791.3	3	4.995	0.494	1	1912.5	43.75	-.GFGDLKTPAGLQVLNDYLADK.S
EF1D_MOUSE	MK_SCX_16.3100.3100.2	2	2.155	0.174	1	367.1	50	K.DIDLFGSDEEEEDK.E
EF1D_MOUSE	MK_SCX_20_1.3332.3332.2	2	3.179	0.439	1	1536.8	80.769226	R.FYEQM*NGPVTSGSR.Q
EF1D_MOUSE	MK_SCX_20_1.3837.3837.2	2	2.238	0.244	1	556	57.692307	R.FYEQMNGPVTSGSR.Q
EF1D_MOUSE	MK_SCX_21.5533.5533.2	2	4.152	0.45	1	1341	81.818184	R.GVVQDLQQAISK.L
EF1D_MOUSE	MK_SCX_21.6400.6400.2	2	3.701	0.493	1	1009.7	79.16667	R.SIQLDGLVWGASK.L
EF1D_MOUSE	MK_SCX_21.6430.6430.1	1	2.501	0.443	1	365.7	50	R.SIQLDGLVWGASK.L
EF1D_MOUSE	MK_SCX_28.4037.4037.3	3	3.522	0.394	1	973.9	33.695652	K.SLAGSSGPGASSGPGGDHSELIVR.I
EF1D_MOUSE	MK_SCX_28.4047.4047.2	2	4.698	0.554	1	1560.9	52.173912	K.SLAGSSGPGASSGPGGDHSELIVR.I
EF1D_MOUSE	MK_SCX_38.3612.3612.2	2	2.238	0.222	1	364.8	60.714287	R.RFYEQMNGPVTSGSR.Q
EF1D_MOUSE	MK_SCX_40.3752.3752.3	3	3.338	0.428	1	2329.2	55.35714	R.RFYEQMNGPVTSGSR.Q
EF1D_MOUSE	MK_SCX_52.4998.4998.2	2	3.862	0.476	1	1295.5	75	K.IWFDKFKYDDAER.R
EF1G_MOUSE	MK_SCX_2201.3980.3980.2	2	4.012	0.537	1	2199.8	83.33333	K.ALIAAQYSGAQVR.V
EF1G_MOUSE	MK_SCX_34.5076.5076.2	2	3.171	0.182	1	1129.6	88.88889	R.ILGLLDTHLK.T
EF2_MOUSE	MK_SCX_17.8255.8255.2	2	4.543	0.586	1	1349.7	70.588234	R.ALLELQLEPEELYQTFQR.I
EF2_MOUSE	MK_SCX_23.5591.5591.2	2	2.327	0.154	1	809.6	93.75	K.VFDAIMNFR.K
EF2_MOUSE	MK_SCX_32.5505.5505.2	2	6.733	0.587	1	2557.9	76.47059	R.GHVFEESQVAGTPMFVVK.A
EF2_MOUSE	MK_SCX_43.5431.5431.3	3	6.856	0.547	1	2803.3	51.315792	K.ARPFPDGLAEDIDKGEVSAR.Q
EF2_MOUSE	MK_SCX_43.5477.5477.2	2	5.017	0.526	1	724	52.63158	K.ARPFPDGLAEDIDKGEVSAR.Q
EFG1_MOUSE	MK_SCX_2201.6020.6020.2	2	2.555	0.21	1	820.7	81.25	K.GIIDLIEER.A
EFG1_MOUSE	MK_SCX_27.5153.5153.2	2	2.273	0.259	1	325.4	54.545456	R.DDSHPFVGLAFK.L
EFG1_MOUSE	MK_SCX_41.6314.6314.3	3	4.93	0.561	1	1428.8	47.368423	R.FVLQDGAHHMVDSNEISFIR.A
EFG1_MOUSE	MK_SCX_48.6805.6805.3	3	3.21	0.281	1	335.5	28.75	R.SKLNHNAAFVQIPIGLEGDFK.G
EFHD1_MOUSE	MK_SCX_18.5406.5406.2	2	3.48	0.191	1	496	56.25	K.AAAGELQEDSGLLALAK.F
EFHD2_MOUSE	MK_SCX_33.3238.3238.3	3	3.026	0.326	1	443.6	47.727272	R.FEEEIKAEQEER.K
EFHD2_MOUSE	MK_SCX_33.3294.3294.2	2	2.735	0.361	1	511.9	72.72727	R.FEEEIKAEQEER.K
EFHD2_MOUSE	MK_SCX_37.3318.3318.3	3	3.879	0.358	1	1098.5	45	R.RADLNQGIGEPQSPSR.R
EFHD2_MOUSE	MK_SCX_37.3347.3347.2	2	3.663	0.38	1	1018.3	66.66667	R.RADLNQGIGEPQSPSR.R
EFTS_MOUSE	MK_SCX_14.9204.9204.2	2	4.12	0.628	1	584	36.206894	R.M*LPQPYLLDPSITLGQYVQPQGVTVVDFVR.F
EFTS_MOUSE	MK_SCX_14.9334.9334.3	3	3.477	0.397	1	840.8	30.172413	R.M*LPQPYLLDPSITLGQYVQPQGVTVVDFVR.F
EFTS_MOUSE	MK_SCX_14.9352.9352.2	2	3.906	0.608	1	642.9	36.206894	R.MLPQPYLLDPSITLGQYVQPQGVTVVDFVR.F
EFTS_MOUSE	MK_SCX_14.9358.9358.3	3	5.068	0.518	1	1811.7	33.62069	R.MLPQPYLLDPSITLGQYVQPQGVTVVDFVR.F
EFTS_MOUSE	MK_SCX_24.15450.15450.3	3	5.744	0.552	1	1397.2	36.53846	K.VPSGFYVGSYVHGVTQSPSLQNLVLGK.Y
EFTS_MOUSE	MK_SCX_24.5738.5738.2	2	6.123	0.684	1	2021.7	57.692307	R.LGQHVVGMAPLSVGSLDDEPGGETETR.M
EFTS_MOUSE	MK_SCX_25.5810.5810.3	3	5.998	0.609	1	2029.3	40.384613	R.LGQHVVGMAPLSVGSLDDEPGGETETR.M
EFTS_MOUSE	MK_SCX_31.14062.14062.3	3	3.864	0.5	1	346.4	23.333334	K.GFLNSSELSELAAGPDREGSLKDQLALAIGK.L
EFTS_MOUSE	MK_SCX_42.5464.5464.3	3	8.012	0.636	1	2308.1	40.74074	R.RLGQHVVGMAPLSVGSLDDEPGGETETR.M
EFTU_MOUSE	MK_SCX_12.7330.7330.3	3	4.425	0.388	1	470.7	30.769232	K.NM*ITGTAPLDGCILVVAANDGPM*PQTR.E
EFTU_MOUSE	MK_SCX_17.4682.4682.2	2	4.816	0.552	1	1089.6	67.64706	K.TIGTGLVTDVPAM*TEEDK.N
EFTU_MOUSE	MK_SCX_17.5283.5283.2	2	3.748	0.624	1	852.2	61.764706	K.TIGTGLVTDVPAMTEEDK.N
EFTU_MOUSE	MK_SCX_18.6150.6150.2	2	3.81	0.507	1	1242.8	76.92308	K.LLDAVDTYIPVPTR.D
EFTU_MOUSE	MK_SCX_21.3230.3230.2	2	3.258	0.242	1	1497	85	K.YEEIDNAPEER.A
EFTU_MOUSE	MK_SCX_2201.3125.3125.2	2	3.414	0.384	1	866.3	77.77778	R.GTVVTGTLER.G
EFTU_MOUSE	MK_SCX_2201.3303.3303.2	2	2.095	0.339	1	437.1	81.25	K.TTLTAAITK.I
EFTU_MOUSE	MK_SCX_2201.4051.4051.2	2	2.905	0.332	1	612.9	72.72727	R.AEAGDNLGALVR.G
EFTU_MOUSE	MK_SCX_2201.7982.7982.2	2	5.093	0.556	1	1443.7	72.22222	R.DLDKPFLLPVESVYSIPGR.G
EFTU_MOUSE	MK_SCX_2201.8142.8142.3	3	3.654	0.429	1	875	38.88889	R.DLDKPFLLPVESVYSIPGR.G
EFTU_MOUSE	MK_SCX_24.6676.6676.3	3	3.777	0.265	1	404.5	29.545454	K.TIGTGLVTDVPAMTEEDKNIK.W
EFTU_MOUSE	MK_SCX_25.4847.4847.3	3	3.15	0.386	1	355.7	31.25	K.TIGTGLVTDVPAM*TEEDKNIK.W
EFTU_MOUSE	MK_SCX_25.5703.5703.2	2	4.257	0.585	1	719.1	52.499996	K.TIGTGLVTDVPAMTEEDKNIK.W
EFTU_MOUSE	MK_SCX_25.9557.9557.3	3	6.644	0.623	1	1059	29.6875	K.LLDAVDTYIPVPTRDLDKPFLLPVESVYSIPGR.G

EFTU_MOUSE	MK_SCX_27.11407.11407.3	3	4.487	0.378	1	331.3	26.785713	R.DLDKPFLLPVESVYSIPGRGTVVTGTLER.G
EFTU_MOUSE	MK_SCX_27.4897.4897.3	3	3.511	0.391	1	428.8	37.5	R.VILPPGKELAM*PGEDLK.L
EFTU_MOUSE	MK_SCX_27.5712.5712.2	2	3.885	0.546	1	485.4	62.5	R.VILPPGKELAMPGEDLK.L
EFTU_MOUSE	MK_SCX_27.5760.5760.3	3	4.064	0.372	1	1474.9	50	R.VILPPGKELAMPGEDLK.L
EFTU_MOUSE	MK_SCX_30.5309.5309.3	3	6.016	0.585	1	1910.3	39.583336	R.DGNKTIGTGLVTDVPAMTEEDKNIK.W
EFTU_MOUSE	MK_SCX_32.5579.5579.2	2	3.614	0.341	1	422.5	75	K.LSLILRQPM*ILEK.G
EFTU_MOUSE	MK_SCX_33.4412.4412.2	2	3.356	0.393	1	1285.3	85	R.TVVTGIEMFHK.S
EFTU_MOUSE	MK_SCX_37.3052.3052.3	3	3.159	0.137	1	720.7	47.727272	K.KYEEIDNAPEER.A
EFTU_MOUSE	MK_SCX_49.3516.3516.3	3	3.243	0.357	1	559.1	39.285713	R.GLVM*VKPGSIQPHQK.V
EFTU_MOUSE	MK_SCX_49.3755.3755.3	3	3.299	0.513	1	713.7	44.642857	R.GLVMVKPGSIQPHQK.V
EFTU_MOUSE	MK_SCX_53.3151.3151.3	3	3.814	0.233	1	1407.5	55.76923	K.FKKYEEIDNAPEER.A
EFTU_MOUSE	MK_SCX_53.3178.3178.2	2	5.721	0.535	1	2276.2	80.769226	K.FKKYEEIDNAPEER.A
EFTU_MOUSE	MK_SCX_58.8638.8638.3	3	4.546	0.552	1	915.8	46.875	R.DKPHVNVGTIGHVDHGK.T
EGF_MOUSE	MK_SCX_27.3680.3680.3	3	3.04	0.372	1	463.8	40.384613	R.TEDAAKDPDPELLK.Q
EGF_MOUSE	MK_SCX_41.3438.3438.3	3	3.157	0.421	1	374.4	35	R.TEDAAKDPDPELLKQR.G
EGFR_MOUSE	MK_SCX_14.7528.7528.2	2	4.174	0.503	1	382.4	33.333336	R.YSSDPTGAVTEDNIDDAFLPVPEYVNQSVPK.R
EHD1_MOUSE	MK_SCX_17.5595.5595.2	2	4.942	0.409	1	2205.7	85.71429	K.LLDTVDDM*LANDIAR.L
EHD1_MOUSE	MK_SCX_17.6393.6393.2	2	5.4	0.438	1	2787	85.71429	K.LLDTVDDMLANDIAR.L
EHD1_MOUSE	MK_SCX_19.6544.6544.3	3	3.942	0.495	1	674.5	26.612906	R.IGPEPTTDSFIAVM*HGPTEGVVPGNALVVDPR.R
EHD1_MOUSE	MK_SCX_19.7415.7415.3	3	5.262	0.582	1	605.8	27.419355	R.IGPEPTTDSFIAVMHGPTEGVVPGNALVVDPR.R
EHD1_MOUSE	MK_SCX_23.6776.6776.2	2	3.801	0.484	1	2151.5	67.64706	K.DKPTYDEIFYTLSPVNGK.I
EHD1_MOUSE	MK_SCX_25.8626.8626.3	3	3.38	0.34	1	931.1	39.705883	K.LFEAEEQDLFKDIQSLPR.N
EHD1_MOUSE	MK_SCX_31.7730.7730.3	3	6.298	0.522	1	4451.1	54.545456	K.LADVDKDGLLDDEEFALANHLIK.V
EHD1_MOUSE	MK_SCX_31.7734.7734.2	2	5.068	0.634	1	1519	54.545456	K.LADVDKDGLLDDEEFALANHLIK.V
EHD1_MOUSE	MK_SCX_37.4337.4337.2	2	3.515	0.418	1	1043.5	75	R.EHQISSGDFPSLR.K
EHD1_MOUSE	MK_SCX_38.5804.5804.3	3	3.133	0.386	1	488.8	33.333336	K.LEGHELPADLPPHLIPPSK.R
EHD1_MOUSE	MK_SCX_40.3721.3721.2	2	2.895	0.254	1	769.2	65	R.HLIEQDFPGM*R.I
EHD1_MOUSE	MK_SCX_42.7843.7843.3	3	4.053	0.474	1	928	40.27778	R.KLFEAEEQDLFKDIQSLPR.N
EHD1_MOUSE	MK_SCX_51.6006.6006.3	3	3.525	0.307	1	634.3	31.25	K.VKLEGHELPADLPPHLIPPSK.R
EHD1_MOUSE	MK_SCX_53.3864.3864.3	3	3.344	0.24	1	480.5	42.307693	K.KKELVNNLGEIYQK.I
EHD1_MOUSE	MK_SCX_56.5597.5597.3	3	3.672	0.496	1	602.9	30.952381	K.VKLEGHELPADLPPHLIPPSKR.R
EHD3_MOUSE	MK_SCX_2201.7787.7787.3	3	7.184	0.636	1	1774.1	35.9375	K.GGAFEGTLQGPFGHGYGEGAGEGIDDAEWVVAR.D
EHD3_MOUSE	MK_SCX_24.7518.7518.2	2	5.229	0.637	1	2510.2	70.588234	R.DKPMYDEIFYTLSPVDGK.I
EHD3_MOUSE	MK_SCX_31.7496.7496.3	3	3.734	0.365	1	977.6	34.090908	K.LADIDKDGMLDDEEFALANHLIK.V
EHD4_MOUSE	MK_SCX_16.6305.6305.2	2	4.496	0.514	1	1818.8	72.22222	K.LADCDCDGMLDEEEFALAK.H
EHD4_MOUSE	MK_SCX_20_1.5492.5492.2	2	2.781	0.326	1	328.3	66.66667	R.EYQISAGDFPEVK.A
EHD4_MOUSE	MK_SCX_21.6166.6166.2	2	2.948	0.29	1	806.1	80	R.LFEAEAQDLFR.D
EHD4_MOUSE	MK_SCX_23.7765.7765.2	2	3.497	0.386	1	2117.1	67.64706	K.DKPVYDELFYTLSPINGK.I
EHD4_MOUSE	MK_SCX_31.4400.4400.3	3	4.059	0.507	1	823.7	46.666668	R.VVLNKADQVDTQQLMR.V
EHD4_MOUSE	MK_SCX_51.3859.3859.3	3	3.884	0.404	1	946	41.07143	K.KEMVTSKLPNSVLGK.I
EIF1B_MOUSE	MK_SCX_19.4325.4325.2	2	3.636	0.564	1	1579	76.92308	K.TLTTVQGIADDYDK.K
EIF1B_MOUSE	MK_SCX_28.6017.6017.2	2	3.985	0.432	1	707.7	73.333336	K.GDDLLPAGTEDYIHIR.I
EIF1B_MOUSE	MK_SCX_29.4344.4344.2	2	4.06	0.461	1	1620.8	75	K.TLTTVQGIADDYDKK.K
EIF1B_MOUSE	MK_SCX_43.4211.4211.3	3	3.472	0.55	1	431	41.666664	K.TLTTVQGIADDYDKKK.L
EIF1B_MOUSE	MK_SCX_44.4084.4084.2	2	4.245	0.338	1	1620.1	73.333336	K.TLTTVQGIADDYDKKK.L
EIF1B_MOUSE	MK_SCX_47.4060.4060.3	3	4.571	0.459	1	871.2	46.666668	R.KTLTTVQGIADDYDKK.K
ELAV1_MOUSE	MK_SCX_21.3811.3811.2	2	3.664	0.492	1	1416.6	90	R.VLVDQTTGLSR.G
ELAV1_MOUSE	MK_SCX_21.5339.5339.2	2	2.262	0.245	1	415.6	58.333332	R.SLFSSIGEVESAK.L
ELAV1_MOUSE	MK_SCX_33.5289.5289.3	3	4.4	0.524	1	1447.2	43.75	K.VAGHSLGYGFVNYVTAK.D
ELAV1_MOUSE	MK_SCX_40.7792.7792.3	3	3.397	0.534	1	508.5	34.090908	K.VSYARPSSEVIKDANLYISGLPR.T
ELOC_MOUSE	MK_SCX_19.5367.5367.2	2	3.971	0.476	1	520.1	60.526318	K.AMLSGPGQFAENETNEVNFR.E

EM55_MOUSE	MK_SCX_14.8449.8449.2	2	5.546	0.512	1	849.9	44.444447	R.TAELSPFIVFIAPTDQGTQTEALQQLQK.D
EM55_MOUSE	MK_SCX_14.8466.8466.3	3	5.69	0.431	1	2453.3	43.51852	R.TAELSPFIVFIAPTDQGTQTEALQQLQK.D
EM55_MOUSE	MK_SCX_19.5460.5460.2	2	4.263	0.437	1	1347	81.818184	K.IAILDIEPQTLK.T
EM55_MOUSE	MK_SCX_19.7624.7624.2	2	4.601	0.609	1	727.1	60.000004	K.NISANEFLEFGSYQGNMFGTK.F
EM55_MOUSE	MK_SCX_19.9112.9112.3	3	4.486	0.6	1	506.3	25.757576	R.TAELSPFIVFIAPTDQGTQTEALQQLQKDSEAIR.S
EM55_MOUSE	MK_SCX_20_1.4000.4000.2	2	3.486	0.387	1	1289.6	85	K.ITEEPM*GITLK.L
EM55_MOUSE	MK_SCX_20_1.4573.4573.2	2	2.153	0.27	1	659.4	70	K.ITEEPMGITLK.L
EM55_MOUSE	MK_SCX_20_1.7013.7013.2	2	3.157	0.29	1	992.8	72.72727	K.FVTGDIIQIINK.D
EM55_MOUSE	MK_SCX_23.5374.5374.2	2	2.828	0.156	1	631.7	81.25	R.LPALQM*FMR.A
EM55_MOUSE	MK_SCX_24.6084.6084.2	2	2.96	0.502	1	955.9	93.75	R.LPALQMFMR.A
EM55_MOUSE	MK_SCX_28.5627.5627.2	2	5.316	0.629	1	2165.2	70	R.VEGSSKESAGLIPSPELQEWR.V
EM55_MOUSE	MK_SCX_28.5629.5629.3	3	5.583	0.446	1	1407.8	50	R.VEGSSKESAGLIPSPELQEWR.V
EM55_MOUSE	MK_SCX_30.6740.6740.2	2	4.723	0.429	1	1929.9	73.07692	R.TALSDLYLEHLLQK.R
EM55_MOUSE	MK_SCX_40.3697.3697.3	3	3.326	0.312	1	443.6	38.235294	K.SEEDGKEYHFISTEEM*TK.N
EM55_MOUSE	MK_SCX_46.6433.6433.2	2	4.934	0.455	1	2046.6	75	R.TALSDLYLEHLLQKR.N
EM55_MOUSE	MK_SCX_53.3368.3368.3	3	3.429	0.341	1	700.2	31.944445	K.KSEEDGKEYHFISTEEM*TK.N
EM55_MOUSE	MK_SCX_53.3830.3830.3	3	4.694	0.501	1	1303.4	43.055553	K.KSEEDGKEYHFISTEEMTK.N
EM55_MOUSE	MK_SCX_57.2729.2729.3	3	3.125	0.33	1	865.1	48.076923	R.SHIKNGLLSHNPEK.F
EMD_MOUSE	MK_SCX_16.5010.5010.2	2	5.036	0.59	1	2107.4	82.14286	K.DYNDDYYEESYLTTK.T
EMIL1_MOUSE	MK_SCX_16.6796.6796.2	2	2.557	0.315	1	733.6	45.454548	R.VLLNDGGYYDPETGVFTAPLAGR.Y
EMIL1_MOUSE	MK_SCX_55.14460.14460.3	3	3.066	0.203	1	334	21.875	R.LEDRFNSTLGPSEEQEKNWPGGPGR.L
ENAH_MOUSE	MK_SCX_38.3249.3249.3	3	5.126	0.461	1	1702.6	41.666664	K.GSTIETEQKEDRNEDAEPITAK.A
ENAH_MOUSE	MK_SCX_38.4399.4399.2	2	2.355	0.184	1	653.5	72.22222	K.LKEELIDAIR.Q
ENOA_MOUSE	MK_SCX_13.5099.5099.2	2	3.634	0.348	1	355.6	60.714287	K.VNQIGSVTESLQACK.L
ENOA_MOUSE	MK_SCX_15.5551.5551.2	2	5.456	0.523	1	2386.6	75	K.DATNVGDEGGFAPNILENK.E
ENOA_MOUSE	MK_SCX_15.8092.8092.2	2	5.638	0.579	1	808.4	45.833336	K.SFVQNYPVVSIEDPFDQDDWGAWQK.F
ENOA_MOUSE	MK_SCX_15.8114.8114.3	3	6.516	0.493	1	2001.9	39.583336	K.SFVQNYPVVSIEDPFDQDDWGAWQK.F
ENOA_MOUSE	MK_SCX_17.12527.12527.2	2	6.936	0.697	1	2360.7	76.31579	K.AGYTDQVVIGMDVAASEFYR.S
ENOA_MOUSE	MK_SCX_17.12922.12922.3	3	4.716	0.534	1	1187.3	38.157894	K.AGYTDQVVIGMDVAASEFYR.S
ENOA_MOUSE	MK_SCX_17.15629.15629.2	2	5.001	0.577	1	1246.6	60.526318	K.AGYTDQVVIGM*DVAASEFYR.S
ENOA_MOUSE	MK_SCX_18.7278.7278.2	2	5.307	0.24	1	1625.5	81.25	K.LAMQEFM*ILPVGASSFR.E
ENOA_MOUSE	MK_SCX_18.7630.7630.2	2	5.652	0.578	1	2114.3	68.42105	K.FTASAGIQVVGDDLTVTNPK.R
ENOA_MOUSE	MK_SCX_18.8422.8422.2	2	5.535	0.48	1	1236.3	75	K.LAMQEFMILPVGASSFR.E
ENOA_MOUSE	MK_SCX_19.6110.6110.2	2	5.474	0.635	1	822.2	70.588234	R.AAVPSGASTGIYEALELR.D
ENOA_MOUSE	MK_SCX_19.6203.6203.2	2	5.244	0.446	1	1594	81.25	K.LAM*QEFM*ILPVGASSFR.E
ENOA_MOUSE	MK_SCX_19.6814.6814.2	2	5.508	0.219	1	1691.1	81.25	K.LAM*QEFMILPVGASSFR.E
ENOA_MOUSE	MK_SCX_20_1.2869.2869.2	2	2.961	0.292	1	985.4	80	K.LM*IEM*DGTENK.S
ENOA_MOUSE	MK_SCX_20_1.3234.3234.2	2	3.915	0.336	1	812.6	75	K.LMIEM*DGTENK.S
ENOA_MOUSE	MK_SCX_20_1.3728.3728.2	2	3.868	0.222	1	1329.3	90	K.LM*IEMDGTENK.S
ENOA_MOUSE	MK_SCX_20_1.4010.4010.2	2	3.587	0.292	1	1125.1	85	K.LMIEMDGTENK.S
ENOA_MOUSE	MK_SCX_20_1.4855.4855.2	2	4.379	0.538	1	762.6	75	R.GNPTVEVDLYTAK.G
ENOA_MOUSE	MK_SCX_20_1.6522.6522.2	2	4.221	0.424	1	965.6	86.36364	R.YITPDQLADLYK.S
ENOA_MOUSE	MK_SCX_20_1.8283.8283.2	2	4.266	0.452	1	588.9	42	K.DATNVGDEGGFAPNILENKEALELLK.T
ENOA_MOUSE	MK_SCX_20_1.8560.8560.3	3	5.046	0.609	1	660.9	36	K.DATNVGDEGGFAPNILENKEALELLK.T
ENOA_MOUSE	MK_SCX_23.12767.12767.3	3	4.151	0.384	1	557.2	25.925926	R.SGETEDTFIADLVVGLCTGQIKTGAPCR.S
ENOA_MOUSE	MK_SCX_26.5539.5539.2	2	3.337	0.412	1	693.3	50	R.EIFDSRGNPTVEVDLYTAK.G
ENOA_MOUSE	MK_SCX_27.14875.14875.3	3	4.826	0.604	1	876.9	40	K.FTASAGIQVVGDDLTVTNPKR.I
ENOA_MOUSE	MK_SCX_28.5070.5070.3	3	5.623	0.405	1	1552.2	42.857143	K.YGKDATNVGDEGGFAPNILENK.E
ENOA_MOUSE	MK_SCX_28.5077.5077.2	2	6.345	0.691	1	2270.9	66.66667	K.YGKDATNVGDEGGFAPNILENK.E
ENOA_MOUSE	MK_SCX_29.4157.4157.3	3	3.532	0.422	1	1170.7	56.25	K.YDLDFKSPDDPSR.Y
ENOA_MOUSE	MK_SCX_29.4200.4200.2	2	3.805	0.436	1	1069.2	75	K.YDLDFKSPDDPSR.Y

ENOA_MOUSE	MK_SCX_31.4134.4134.3	3	3.447	0.283	1	790.9	50	K.LAQSNGWGVMVSHR.S
ENOA_MOUSE	MK_SCX_31.4218.4218.3	3	3.622	0.308	1	1256.9	46.153847	K.IDKLMIEMDGTENK.S
ENOA_MOUSE	MK_SCX_31.4231.4231.2	2	4.618	0.365	1	2114.6	80.769226	K.IDKLMIEMDGTENK.S
ENOA_MOUSE	MK_SCX_32.4084.4084.2	2	3.133	0.179	1	1652.1	80.769226	K.LAQSNGWGVMVSHR.S
ENOA_MOUSE	MK_SCX_32.7987.7987.2	2	3.561	0.51	1	638.6	34.482758	R.HIADLAGNPEVILPVPAFNVINGGSHAGNK.L
ENOA_MOUSE	MK_SCX_32.8256.8256.3	3	5.862	0.46	1	2263.1	36.206894	R.HIADLAGNPEVILPVPAFNVINGGSHAGNK.L
ENOA_MOUSE	MK_SCX_33.7726.7726.2	2	4.746	0.67	1	1031.5	41.07143	K.YGKDATNVGDEGGFAPNILENKEALELLK.T
ENOA_MOUSE	MK_SCX_33.7787.7787.3	3	7.733	0.623	1	2531.2	40.17857	K.YGKDATNVGDEGGFAPNILENKEALELLK.T
ENOA_MOUSE	MK_SCX_39.3391.3391.2	2	3.209	0.272	1	1358.1	88.88889	R.IGAEVYHNLK.N
ENOA_MOUSE	MK_SCX_42.4962.4962.3	3	5.631	0.498	1	1579.8	41.304348	K.EKYGKDATNVGDEGGFAPNILENK.E
ENOA_MOUSE	MK_SCX_42.5273.5273.3	3	4.707	0.523	1	1057.2	36.842106	K.GVSQAVEHINKTIAPALVSK.K
ENOA_MOUSE	MK_SCX_45.4015.4015.2	2	5.233	0.585	1	1544.7	76.666664	R.SGKYDLDFKSPDDPSR.Y
ENOA_MOUSE	MK_SCX_46.3881.3881.3	3	4.192	0.476	1	1940.3	53.333336	R.SGKYDLDFKSPDDPSR.Y
ENOA_MOUSE	MK_SCX_51.4520.4520.2	2	4.505	0.493	1	2003.9	80.769226	R.IGAEVYHNLKNVIK.E
ENOA_MOUSE	MK_SCX_51.4603.4603.3	3	4.573	0.431	1	750.9	44.230766	R.IGAEVYHNLKNVIK.E
ENOA_MOUSE	MK_SCX_52.2542.2542.3	3	4.622	0.432	1	952.5	52.272724	K.KVNVVEQEKIDK.L
ENOA_MOUSE	MK_SCX_52.3486.3486.3	3	4.231	0.423	1	1021.5	44.642857	R.FMGKGVSQAVEHINK.T
ENOA_MOUSE	MK_SCX_55.4581.4581.3	3	4.9	0.502	1	1024.7	50	R.IGAEVYHNLKNVIKEK.Y
ENOA_MOUSE	MK_SCX_55.4703.4703.2	2	4.636	0.401	1	1325.8	66.66667	R.IGAEVYHNLKNVIKEK.Y
ENP5_MOUSE	MK_SCX_16.7606.7606.2	2	4.331	0.527	1	1036.7	47.916664	R.GQETVGTLDLGGASTQITFLPQFEK.T
ENP5_MOUSE	MK_SCX_27.9703.9703.3	3	3.893	0.447	1	993	41.666664	K.QGAETVQELLEVAKDSIPR.S
ENP5_MOUSE	MK_SCX_44.4284.4284.3	3	3.36	0.49	1	1208.3	45	R.AADTHLIDYEKGGVLK.V
ENPL_MOUSE	MK_SCX_17.15760.15760.2	2	5.757	0.601	1	2546.1	72.22222	R.LISLTDENALAGNEELTVK.I
ENPL_MOUSE	MK_SCX_18.5185.5185.2	2	4.568	0.482	1	2320.1	76.666664	R.EEEAIQLDGLNASQIR.E
ENPL_MOUSE	MK_SCX_19.4609.4609.2	2	4.87	0.418	1	1620	84.61539	K.GVVDSDDLPLNVSR.E
ENPL_MOUSE	MK_SCX_2201.4775.4775.2	2	3.124	0.318	1	704.1	75	K.SILFVPTSAPR.G
ENPL_MOUSE	MK_SCX_23.6426.6426.3	3	3.662	0.304	1	657.6	31.52174	R.TDDEVVQREEEAIQLDGLNASQIR.E
ENPL_MOUSE	MK_SCX_23.6443.6443.2	2	3.709	0.457	1	637.3	47.826088	R.TDDEVVQREEEAIQLDGLNASQIR.E
ENPL_MOUSE	MK_SCX_25.4151.4151.2	2	3.151	0.435	1	1340.2	93.75	K.FAFQAEVNR.M
ENPL_MOUSE	MK_SCX_28.6202.6202.2	2	4.2	0.485	1	1278.9	75	R.VFITDDFHDMMPK.Y
ENPL_MOUSE	MK_SCX_30.3621.3621.3	3	4.566	0.577	1	1410.6	45.833336	K.AQAYQTGKDISTNYYASQK.K
ENPL_MOUSE	MK_SCX_30.5051.5051.2	2	4.238	0.494	1	2002.7	80.769226	R.ELISNASDALDKIR.L
ENPL_MOUSE	MK_SCX_30.5928.5928.2	2	5.018	0.516	1	1046.9	67.85714	R.EATEKEFEPLLNWM*K.D
ENPL_MOUSE	MK_SCX_30.7123.7123.2	2	5.203	0.521	1	1163.4	67.85714	R.EATEKEFEPLLNWMK.D
ENPL_MOUSE	MK_SCX_30.7173.7173.3	3	3.288	0.477	1	488.8	41.07143	R.EATEKEFEPLLNWMK.D
ENPL_MOUSE	MK_SCX_31.3660.3660.2	2	5.569	0.531	1	2793.1	75	K.AQAYQTGKDISTNYYASQK.K
ENPL_MOUSE	MK_SCX_31.3997.3997.2	2	4.19	0.481	1	470.8	68.181816	K.EVEEDEYKAFYK.S
ENPL_MOUSE	MK_SCX_33.7112.7112.2	2	3.601	0.286	1	489.8	72.72727	K.EFEPLLNWMKDK.A
ENPL_MOUSE	MK_SCX_34.6434.6434.3	3	5.047	0.461	1	1462.6	34.25926	R.EGSRTDDEVVQREEEAIQLDGLNASQIR.E
ENPL_MOUSE	MK_SCX_35.11496.11496.2	2	4.057	0.496	1	1295.2	73.333336	R.KYSQFINFPIYVWSSK.T
ENPL_MOUSE	MK_SCX_35.11514.11514.3	3	3.221	0.142	1	931.2	40	R.KYSQFINFPIYVWSSK.T
ENPL_MOUSE	MK_SCX_35.3867.3867.3	3	3.391	0.464	1	1730.9	59.615387	K.NLLHVTDTGVGM*TR.E
ENPL_MOUSE	MK_SCX_35.4512.4512.3	3	4.63	0.551	1	1359.3	57.692307	K.NLLHVTDTGVGMTR.E
ENPL_MOUSE	MK_SCX_35.4728.4728.2	2	4.462	0.603	1	1799	88.46153	K.NLLHVTDTGVGMTR.E
ENPL_MOUSE	MK_SCX_36.6353.6353.3	3	3.542	0.313	1	670	42.307693	K.YNDTFWKEFGTNIK.L
ENPL_MOUSE	MK_SCX_36.6492.6492.2	2	4.957	0.52	1	2240.8	88.46153	K.YNDTFWKEFGTNIK.L
ENPL_MOUSE	MK_SCX_42.5651.5651.3	3	3.509	0.14	1	617.6	37.5	R.EATEKEFEPLLNWM*KDK.A
ENPL_MOUSE	MK_SCX_42.6873.6873.2	2	5.076	0.532	1	917.5	65.625	R.EATEKEFEPLLNWMKDK.A
ENPL_MOUSE	MK_SCX_42.6881.6881.3	3	5.233	0.494	1	1097.5	40.625	R.EATEKEFEPLLNWMKDK.A
ENPL_MOUSE	MK_SCX_43.6307.6307.3	3	5.855	0.54	1	1756.3	45.833336	K.IADEKYNDTFWKEFGTNIK.L
ENPL_MOUSE	MK_SCX_44.4763.4763.3	3	4.025	0.489	1	857.7	37.5	K.NLLHVTDTGVGM*TREELVK.N

ENPL_MOUSE	MK_SCX_44.5331.5331.3	3	4.196	0.5	1	558.8	36.11111	K.NLLHVTDTGVGMTREELVK.N
ENPL_MOUSE	MK_SCX_44.5368.5368.2	2	4.725	0.523	1	679.1	52.77778	K.NLLHVTDTGVGMTREELVK.N
ENPL_MOUSE	MK_SCX_45.3554.3554.3	3	5.982	0.586	1	1403.3	47.368423	K.AQAYQTGKDISTNYYASQKK.T
ENPL_MOUSE	MK_SCX_47.4864.4864.2	2	6.009	0.669	1	2563.5	72.22222	R.FQSSHHSTDITSLDQYVER.M
ENPL_MOUSE	MK_SCX_48.4714.4714.3	3	6.287	0.529	1	2267.5	48.61111	R.FQSSHHSTDITSLDQYVER.M
ENPL_MOUSE	MK_SCX_49.6043.6043.2	2	3.861	0.38	1	1383.9	76.92308	R.RVFITDDFHDMMPK.Y
ENPL_MOUSE	MK_SCX_49.6081.6081.3	3	3.951	0.536	1	2183.9	57.692307	R.RVFITDDFHDMMPK.Y
ENPP5_MOUSE	MK_SCX_50.6436.6436.3	3	4.138	0.358	1	890.1	37.5	R.YLDKEHYTLIDHSPVAAILPK.E
ENPP6_MOUSE	MK_SCX_17.8948.8948.2	2	3.85	0.307	1	366.8	50	K.TVPTDINFANAVSDALDSLK.S
EP15_MOUSE	MK_SCX_21.6447.6447.3	3	3.62	0.438	1	525.8	24.166666	K.VNNEDAFNPTISSSTSSVTIAKPMLEETASK.S
EP15_MOUSE	MK_SCX_23.8628.8628.3	3	3.472	0.337	1	390.4	25	K.QQVQELLGELDEQKAQLEEQLQEVR.K
EPIPL_MOUSE	MK_SCX_15.9719.9719.3	3	3.325	0.24	1	383.1	22.222223	R.AAALRQVIGM*LTTLVEAAEKQPSQATFK.G
EPIPL_MOUSE	MK_SCX_16.4628.4628.3	3	3.557	0.27	1	606.9	26.724138	R.DGLLPTGLGQQLLEAQVASGFLVNPLTNQR.L
EPN1_MOUSE	MK_SCX_14.7934.7934.3	3	4.122	0.516	1	723.3	27.205881	K.ASNPFLPSGAPPTGPSVTNPFQPAPPATLTLNQLR.L
EPN1_MOUSE	MK_SCX_42.3725.3725.3	3	3.854	0.486	1	1235.9	46.875	K.DFQYVDRDGKDQGVNVR.E
EPN4_MOUSE	MK_SCX_23.4014.4014.2	2	3.662	0.51	1	1350.7	87.5	K.YVGVSSDSVGGFR.Y
EPN4_MOUSE	MK_SCX_26.5495.5495.3	3	4.422	0.547	1	1294.2	47.058823	K.LGELSDKIGSTIDDTISK.F
EPN4_MOUSE	MK_SCX_30.5888.5888.3	3	5.104	0.489	1	1365.9	38.095238	K.VREATNDDPWGPSGQLMGEIAK.A
EPN4_MOUSE	MK_SCX_31.4020.4020.2	2	3.466	0.483	1	772.8	65.38461	K.TIDLGAAAHYTGDK.A
EPN4_MOUSE	MK_SCX_31.6102.6102.2	2	3.452	0.306	1	326.7	50	K.SAFPFSDKLGELSDK.I
EPN4_MOUSE	MK_SCX_32.13259.13259.3	3	3.825	0.44	1	497.7	26	K.SAFPFSDKLGELSDKIGSTIDDTISK.F
EPN4_MOUSE	MK_SCX_40.2954.2954.3	3	4.058	0.574	1	494.2	31.52174	K.ASPDQNASTHTPQSSAKPSVPSSK.S
EPN4_MOUSE	MK_SCX_50.3086.3086.3	3	4.055	0.446	1	1175.5	50	R.SPKGEFKDEEETVTTK.H
EPN4_MOUSE	MK_SCX_53.3658.3658.3	3	3.677	0.414	1	488.6	37.5	K.NKDKYVGVSSDSVGGFR.Y
EPN4_MOUSE	MK_SCX_53.4649.4649.3	3	4.34	0.55	1	1469.6	39.473686	R.SLENYHFVDEHGKDQGINIR.Q
EPN4_MOUSE	MK_SCX_54.2849.2849.3	3	3.619	0.378	1	707.7	44.230766	K.HIHITQATETTTTR.H
EPS8_MOUSE	MK_SCX_19.7235.7235.2	2	4.1	0.427	1	397.9	50	R.NASGDSGFVPNNILDIMR.T
EPS8_MOUSE	MK_SCX_36.5404.5404.2	2	3.202	0.386	1	431.4	65.38461	R.KSQMEEVQDELFQR.L
ERGI1_MOUSE	MK_SCX_26.6867.6867.3	3	3.932	0.45	1	431.2	26.086956	K.LSFGDTLQVQNVHGAFNALGGADR.L
ERGI1_MOUSE	MK_SCX_31.4854.4854.2	2	2.821	0.346	1	608.9	57.692307	R.LTSNPLASHDYILK.I
ERH_MOUSE	MK_SCX_17.4483.4483.2	2	4.645	0.479	1	780.9	62.5	R.TYADYESVNECMEGVCK.M
ERH_MOUSE	MK_SCX_19.3933.3933.2	2	3.79	0.41	1	822.9	56.25	R.TYADYESVNECM*EGVCK.M
ERO1A_MOUSE	MK_SCX_19.3704.3704.2	2	4.487	0.554	1	1618.7	76.92308	R.LGAVDESLSEETQK.A
ERP29_MOUSE	MK_SCX_13.9240.9240.2	2	3.769	0.552	1	388.5	43.47826	K.GQGVYLGMPGCLPAYDALAGEFIK.A
ERP29_MOUSE	MK_SCX_16.4809.4809.2	2	3.2	0.18	1	1974	82.14286	R.DGDLENPVLYNGAVK.V
ERP29_MOUSE	MK_SCX_16.4883.4883.1	1	2.136	0.141	1	736.4	60.714287	R.DGDLENPVLYNGAVK.V
ERP29_MOUSE	MK_SCX_19.4005.4005.2	2	4.885	0.597	1	1125.5	75	K.ILDQGEDFPASEM*AR.I
ERP29_MOUSE	MK_SCX_19.4477.4477.2	2	4.695	0.542	1	1279.2	71.42857	K.ILDQGEDFPASEMAR.I
ERP29_MOUSE	MK_SCX_20_1.6335.6335.2	2	3.447	0.552	1	477.6	72.72727	K.GALPLDTVTFYK.V
ERP29_MOUSE	MK_SCX_24.5924.5924.2	2	3.606	0.464	1	1379.2	93.75	K.SLNILTAFR.K
ERP29_MOUSE	MK_SCX_32.7364.7364.3	3	3.662	0.486	1	809	45.833336	K.LDKESYPVFYLFR.D
ERP29_MOUSE	MK_SCX_40.5579.5579.2	2	2.765	0.275	1	386.4	61.11111	K.SLNILTAFRK.K
ERP29_MOUSE	MK_SCX_46.4031.4031.2	2	4.553	0.537	1	881.6	70	K.FDTQYPYGEKQDEFKR.L
ERP29_MOUSE	MK_SCX_47.4174.4174.3	3	3.734	0.517	1	635.8	40	K.FDTQYPYGEKQDEFKR.L
ERP29_MOUSE	MK_SCX_50.7128.7128.3	3	4.44	0.459	1	1570.1	51.785713	K.YKLDKESYPVFYLFR.D
ERP29_MOUSE	MK_SCX_50.7145.7145.2	2	5.254	0.578	1	2233.5	85.71429	K.YKLDKESYPVFYLFR.D
ES1_MOUSE	MK_SCX_15.4474.4474.2	2	2.568	0.334	1	1133.7	70.83333	K.NLSTFAVDGKDCK.V
ES1_MOUSE	MK_SCX_24.4353.4353.2	2	2.793	0.332	1	1129.3	80	K.WPYAGTAEAIK.A
ES1_MOUSE	MK_SCX_26.6392.6392.3	3	6.104	0.479	1	1312	39.583336	K.ITSLAQLNAANHDAAIFPGGFGAAK.N
ES1_MOUSE	MK_SCX_26.6417.6417.2	2	5.681	0.623	1	1201.8	54.166668	K.ITSLAQLNAANHDAAIFPGGFGAAK.N
ES1_MOUSE	MK_SCX_35.10293.10293.2	2	4.629	0.525	1	948.1	57.14286	R.GGAEVQIFAPDVPQMHVIDHTK.G

ES1_MOUSE	MK_SCX_35.10447.10447.3	3	4.706	0.497	1	507.6	32.142857	R.GGAEVQIFAPDVPQMHVIDHTK.G
ES1_MOUSE	MK_SCX_35.6679.6679.3	3	3.195	0.313	1	1085.2	46.42857	K.KPIGLCCIAPVLAAK.V
ES1_MOUSE	MK_SCX_35.7029.7029.2	2	4.096	0.581	1	1058.4	71.42857	K.KPIGLCCIAPVLAAK.V
ES1_MOUSE	MK_SCX_36.5070.5070.3	3	5.822	0.491	1	1386.5	37	K.GVEVTVGHEQEEGGKWPYAGTAEAIK.A
ES1_MOUSE	MK_SCX_36.5104.5104.2	2	5.807	0.64	1	1114.1	44	K.GVEVTVGHEQEEGGKWPYAGTAEAIK.A
ES1_MOUSE	MK_SCX_41.6418.6418.3	3	6.448	0.637	1	2179.2	38.46154	R.GKITSLAQLNAANHDAAIFPGGFGAAK.N
ES1_MOUSE	MK_SCX_41.6428.6428.2	2	5.086	0.558	1	1266.6	50	R.GKITSLAQLNAANHDAAIFPGGFGAAK.N
ES1_MOUSE	MK_SCX_45.3499.3499.3	3	3.663	0.445	1	669.2	39.705883	K.VIKGVEVTVGHEQEEGGK.W
ES1_MOUSE	MK_SCX_45.5174.5174.3	3	3.595	0.388	1	460.2	23.148148	R.GGAEVQIFAPDVPQM*HVIDHTKGEPSER.E
ES1_MOUSE	MK_SCX_45.6008.6008.3	3	4.142	0.461	1	568.4	25.925926	R.GGAEVQIFAPDVPQMHVIDHTKGEPSER.E
ES1_MOUSE	MK_SCX_45.6097.6097.2	2	4.872	0.556	1	639.2	38.88889	R.GGAEVQIFAPDVPQMHVIDHTKGEPSER.E
ES1_MOUSE	MK_SCX_48.5281.5281.3	3	6.64	0.509	1	1784.8	34.82143	K.VIKGVEVTVGHEQEEGGKWPYAGTAEAIK.A
ES1_MOUSE	MK_SCX_56.5869.5869.3	3	4.133	0.27	1	1515.3	39.130436	R.VLKEFHGAKKPIGLCCIAPVLAAK.V
ES8L2_MOUSE	MK_SCX_19.6654.6654.2	2	4.127	0.52	1	1504.7	73.333336	R.SVSSPLLSTDAVSFLR.G
ES8L2_MOUSE	MK_SCX_19.6744.6744.2	2	2.408	0.319	1	801.7	57.14286	R.VGLPLPVPFSEPGYR.R
ES8L2_MOUSE	MK_SCX_21.8434.8434.3	3	5.531	0.469	1	1641.9	34.82143	R.LLDVESQEELENFPLPTVQHSQTVLNQLR.Y
ES8L2_MOUSE	MK_SCX_2201.3111.3111.2	2	3.372	0.408	1	757.3	87.5	R.VYSQLTVQK.A
ES8L2_MOUSE	MK_SCX_29.5690.5690.2	2	4.853	0.443	1	1108.8	73.52941	R.SQPVHLPLTFESGPDEVR.A
ES8L2_MOUSE	MK_SCX_29.5832.5832.3	3	3.324	0.194	1	529	41.17647	R.SQPVHLPLTFESGPDEVR.A
ES8L2_MOUSE	MK_SCX_32.5741.5741.2	2	2.028	0.202	1	413.7	53.571426	R.SEWPREPQVPLYVPK.F
ES8L2_MOUSE	MK_SCX_34.6788.6788.2	2	3.522	0.443	1	444.3	50	K.NRVGLPLPVPFSEPGYR.R
ES8L2_MOUSE	MK_SCX_45.3668.3668.3	3	5.679	0.559	1	1974.1	46.739132	K.HSLSSESQAPEDIAPPGSSPHANR.G
ESAM_MOUSE	MK_SCX_16.5340.5340.2	2	4.236	0.573	1	682.1	52.272724	R.VDEPPPQAVSLTPGGVSSSALSR.M
EST1_MOUSE	MK_SCX_20_1.3425.3425.2	2	3.597	0.34	1	1577.2	81.818184	K.EGASEEEINLSK.M
EST22_MOUSE	MK_SCX_2201.11238.11238.3	3	4.925	0.404	1	535.5	26.85185	K.YISLEGFTQPVAVFLGVPFAKPPLGSLR.F
EST22_MOUSE	MK_SCX_31.5094.5094.2	2	2.348	0.219	1	389.5	63.636364	K.LDQMTAMSLLKK.S
ESTD_MOUSE	MK_SCX_16.7904.7904.2	2	4.357	0.59	1	570.5	43.18182	R.M*YSYVTEELPQLINANFPVDPQR.M
ESTD_MOUSE	MK_SCX_16.7925.7925.3	3	5.343	0.566	1	1750.8	44.31818	R.M*YSYVTEELPQLINANFPVDPQR.M
ESTD_MOUSE	MK_SCX_16.8116.8116.2	2	5.051	0.506	1	914.7	52.272724	R.MYSYVTEELPQLINANFPVDPQR.M
ESTD_MOUSE	MK_SCX_16.8123.8123.3	3	5.468	0.578	1	2026.5	44.31818	R.MYSYVTEELPQLINANFPVDPQR.M
ESTD_MOUSE	MK_SCX_27.16764.16764.3	3	4.165	0.324	1	878.7	34.523808	K.SGYQQAASEHGLVVIAPDTSPR.G
ESTD_MOUSE	MK_SCX_27.4777.4777.2	2	6.041	0.487	1	1957.5	59.523808	K.SGYQQAASEHGLVVIAPDTSPR.G
ESTD_MOUSE	MK_SCX_34.4573.4573.2	2	4.102	0.509	1	911.9	69.230774	K.AFSGYLGPDESKWK.A
ESTD_MOUSE	MK_SCX_34.4575.4575.3	3	3.18	0.457	1	912.9	55.76923	K.AFSGYLGPDESKWK.A
ESTD_MOUSE	MK_SCX_52.3997.3997.3	3	3.764	0.369	1	941.6	41.07143	K.KAFSGYLGPDESKWK.A
ESTN_MOUSE	MK_SCX_2201.11279.11279.3	3	4.865	0.598	1	1027.4	34.25926	K.YISLEGFEQPVAVFLGVPFAKPPLGSLR.F
ETFA_MOUSE	MK_SCX_14.10030.10030.2	2	6.44	0.657	1	3248.2	81.57895	K.DPEAPIFQVADYGIVADLFK.V
ETFA_MOUSE	MK_SCX_17.7567.7567.2	2	4.8	0.469	1	1097.3	63.333332	K.GLLPEELTPLILETQK.Q
ETFA_MOUSE	MK_SCX_18.4194.4194.2	2	4.903	0.529	1	896.9	72.22222	R.AAVDAGFVPNDM*QVGQTGK.I
ETFA_MOUSE	MK_SCX_18.4768.4768.2	2	4.644	0.563	1	671.1	63.88889	R.AAVDAGFVPNDMQVGQTGK.I
ETFA_MOUSE	MK_SCX_18.4794.4794.1	1	3.354	0.515	1	554.6	50	R.AAVDAGFVPNDMQVGQTGK.I
ETFA_MOUSE	MK_SCX_19.5757.5757.2	2	4.225	0.562	1	1043.3	91.66667	K.LNVAPVSDIIEIK.S
ETFA_MOUSE	MK_SCX_20_1.6060.6060.2	2	4.316	0.369	1	1596.4	73.333336	K.APSSSSVGISEWLDQK.L
ETFA_MOUSE	MK_SCX_21.10239.10239.2	2	5.207	0.683	1	1163.8	48.076923	K.TIVAINKDPEAPIFQVADYGIVADLFK.V
ETFA_MOUSE	MK_SCX_21.10531.10531.3	3	7.295	0.652	1	2123.4	43.269234	K.TIVAINKDPEAPIFQVADYGIVADLFK.V
ETFA_MOUSE	MK_SCX_21.3266.3266.3	3	4.136	0.52	1	1048.5	51.47059	R.GTSFEAAATSGGSASSEK.A
ETFA_MOUSE	MK_SCX_21.3410.3410.2	2	5.558	0.599	1	2972.5	76.47059	R.GTSFEAAATSGGSASSEK.A
ETFA_MOUSE	MK_SCX_2201.6663.6663.3	3	6.753	0.559	1	2559.7	34.090908	R.GTSFEAAATSGGSASSEKAPSSSSVGISEWLDQK.L
ETFA_MOUSE	MK_SCX_23.6167.6167.2	2	4.271	0.558	1	770.1	36.363636	R.GTSFEAAATSGGSASSEKAPSSSSVGISEWLDQK.L
ETFA_MOUSE	MK_SCX_25.7197.7197.2	2	5.544	0.628	1	1942.5	65.789474	K.LNVAPVSDIIEIKSPDTFVR.T
ETFA_MOUSE	MK_SCX_26.2714.2714.2	2	2.418	0.265	1	698.3	91.66667	K.SPDTFVR.T

ETFA_MOUSE	MK_SCX_27.11771.11771.3	3	4.595	0.533	1	1485.8	53.125	K.LLYDLADQLHAAVGASR.A
ETFA_MOUSE	MK_SCX_27.16189.16189.2	2	5.911	0.563	1	3475.2	84.375	K.LLYDLADQLHAAVGASR.A
ETFA_MOUSE	MK_SCX_28.4032.4032.2	2	3.761	0.275	1	870.7	60.000004	R.TIYAGNALCTVKCDEK.V
ETFA_MOUSE	MK_SCX_29.6431.6431.3	3	3.39	0.504	1	676.1	40.27778	K.APSSSSVGISEWLDQKLTK.S
ETFA_MOUSE	MK_SCX_31.8168.8168.2	2	5.75	0.546	1	972.6	46	K.VLVAQHDAYKGLLPEELTPLILETQK.Q
ETFA_MOUSE	MK_SCX_31.8274.8274.3	3	7.171	0.548	1	2438.8	43	K.VLVAQHDAYKGLLPEELTPLILETQK.Q
ETFA_MOUSE	MK_SCX_33.4332.4332.2	2	2.653	0.244	1	587	46.666668	R.LGGEVSCLVAGTKCDK.V
ETFA_MOUSE	MK_SCX_41.4024.4024.2	2	3.654	0.417	1	612.7	52.941177	R.TIYAGNALCTVKCDEKVK.V
ETFA_MOUSE	MK_SCX_42.8794.8794.3	3	3.182	0.359	1	336.8	27.272728	K.SGENFKLLYDLADQLHAAVGASR.A
ETFB_MOUSE	MK_SCX_19.6672.6672.2	2	3.138	0.213	1	357.1	46.875	K.EIIAVSCGPSQCQETIR.T
ETFB_MOUSE	MK_SCX_20_1.3207.3207.2	2	3.193	0.429	1	629.4	83.33333	K.VETTEDLVAK.L
ETFB_MOUSE	MK_SCX_20_1.3791.3791.2	2	3.481	0.397	1	1145.4	85	K.AGDLGVDLTSK.V
ETFB_MOUSE	MK_SCX_20_1.4039.4039.2	2	3.459	0.405	1	1097.6	81.818184	K.VSVISVEEPPQR.S
ETFB_MOUSE	MK_SCX_20_1.4065.4065.3	3	4.07	0.233	1	1630.5	59.090908	K.VSVISVEEPPQR.S
ETFB_MOUSE	MK_SCX_21.4219.4219.2	2	2.717	0.324	1	612.8	66.66667	R.EIDGGLETLR.L
ETFB_MOUSE	MK_SCX_2201.4326.4326.2	2	2.919	0.475	1	1437.9	94.44444	K.LPAVVTADLR.L
ETFB_MOUSE	MK_SCX_2201.5614.5614.2	2	3.325	0.411	1	867.7	92.85714	K.VDLLFLGK.Q
ETFB_MOUSE	MK_SCX_23.6238.6238.2	2	2.698	0.261	1	429.2	43.333332	K.HSM*NPFCEIAVEEAVR.L
ETFB_MOUSE	MK_SCX_24.3350.3350.2	2	2.37	0.32	1	1304.9	87.5	R.TALAMGADR.G
ETFB_MOUSE	MK_SCX_28.6501.6501.3	3	5.201	0.3	1	1158.5	42.5	R.GIHVEIPGAQAESLGPLQVAR.V
ETFB_MOUSE	MK_SCX_29.6615.6615.2	2	6.218	0.684	1	1206.8	72.5	R.GIHVEIPGAQAESLGPLQVAR.V
ETFB_MOUSE	MK_SCX_31.3750.3750.2	2	3.749	0.423	1	1598.2	67.85714	R.SAGVKVETTEDLVAK.L
ETFB_MOUSE	MK_SCX_31.6427.6427.3	3	4.536	0.388	1	2043.9	60.714287	R.SAGVKVETTEDLVAK.L
ETFB_MOUSE	MK_SCX_35.4073.4073.3	3	3.22	0.209	1	536.2	41.666664	K.VEREIDGGLETLR.L
ETFB_MOUSE	MK_SCX_35.9471.9471.3	3	3.745	0.327	1	1222.9	52.272724	R.LKLPAVVTADLR.L
ETFB_MOUSE	MK_SCX_36.5854.5854.2	2	3.947	0.57	1	1116	90.909096	R.LKLPAVVTADLR.L
ETFB_MOUSE	MK_SCX_48.5088.5088.2	2	4.685	0.509	1	1966.1	73.07692	K.LAEKEKVDLLFLGK.Q
ETFB_MOUSE	MK_SCX_48.5100.5100.3	3	5.598	0.537	1	2639.2	61.538464	K.LAEKEKVDLLFLGK.Q
ETFB_MOUSE	MK_SCX_57.3127.3127.3	3	3.43	0.391	1	1240.9	45	K.IRVKPDKSGVVTDGVK.H
ETFD_MOUSE	MK_SCX_13.8024.8024.2	2	3.425	0.487	1	623.8	55.555557	R.FCPAGVYEFVPLEQGDGFR.L
ETFD_MOUSE	MK_SCX_13.8745.8745.2	2	4.182	0.458	1	637.7	52.63158	K.LTFPGGLLIGCSPGFMNVPK.I
ETFD_MOUSE	MK_SCX_17.8261.8261.2	2	4.237	0.586	1	1145.2	57.14286	R.FAEEADVVIVGAGPAGLSAAIR.L
ETFD_MOUSE	MK_SCX_21.3655.3655.2	2	2.909	0.316	1	309.4	63.636364	K.GAPLNTPVTEDR.F
ETHE1_MOUSE	MK_SCX_13.4915.4915.2	2	2.383	0.153	1	527	72.72727	R.SLLPGCQSVISR.L
ETHE1_MOUSE	MK_SCX_25.6384.6384.3	3	4.019	0.396	1	742.3	33.333336	K.VMDNLNLPKPQQIDIAVPANM*R.C
ETHE1_MOUSE	MK_SCX_25.6947.6947.2	2	3.77	0.49	1	920.6	50	K.VMDNLNLPKPQQIDIAVPANMR.C
ETHE1_MOUSE	MK_SCX_25.6951.6951.3	3	5.902	0.584	1	1527.9	41.666664	K.VMDNLNLPKPQQIDIAVPANMR.C
ETHE1_MOUSE	MK_SCX_27.5142.5142.2	2	4.537	0.371	1	1192.8	80.769226	R.EAVLIDPVLETAHR.D
ETHE1_MOUSE	MK_SCX_30.12450.12450.3	3	4.1	0.515	1	681.5	42.1875	R.LSGAQADLHIGEGDSIR.F
ETHE1_MOUSE	MK_SCX_30.4215.4215.2	2	5.227	0.572	1	1979.9	71.875	R.LSGAQADLHIGEGDSIR.F
ETHE1_MOUSE	MK_SCX_30.5029.5029.3	3	3.333	0.362	1	1229.8	62.5	R.DAQLIKELGLK.L
ETHE1_MOUSE	MK_SCX_30.5037.5037.2	2	3.535	0.422	1	1762.5	85	R.DAQLIKELGLK.L
EWS_MOUSE	MK_SCX_2201.5340.5340.2	2	3.381	0.341	1	819	50	R.AGDWQCPNPGCGNQNFAWR.T
EWS_MOUSE	MK_SCX_32.4801.4801.2	2	4.562	0.589	1	1926.8	75	K.AAVEWFDGKDFQGSK.L
EWS_MOUSE	MK_SCX_39.3185.3185.3	3	3.291	0.426	1	855.7	45.588234	R.RGGPGGPPGPLM*EQM*GGR.R
EWS_MOUSE	MK_SCX_51.4989.4989.3	3	3.919	0.522	1	616.8	30.645163	R.TGQPMIHIYLDKETGKPKGDATVSYEDPPTAK.A
EXOC3_MOUSE	MK_SCX_43.4926.4926.3	3	4.464	0.579	1	1408.2	44.11765	R.VAGMLQRPDQLDKVEQYR.R
EXOC7_MOUSE	MK_SCX_34.6024.6024.3	3	3.933	0.324	1	796.5	48.333332	K.NKLPGLITSMETIGAK.A
EXOC7_MOUSE	MK_SCX_46.3817.3817.3	3	3.319	0.256	1	644.8	37.5	K.LENSIIPVHKQTENLQR.L
EYA3_MOUSE	MK_SCX_23.3773.3773.3	3	6.384	0.61	1	889.8	35.416664	R.LPSDSSASPPLSQTTPNKDADDQAR.K
EZRI_MOUSE	MK_SCX_17.15709.15709.2	2	2.917	0.386	1	507.6	38.88889	R.VTTMDAELEFAIQPNTTGK.Q

EZRI_MOUSE	MK_SCX_17.5158.5158.2	2	5.757	0.554	1	1704.8	66.66667	R.VTTM*DAELEFAIQPNTTGK.Q
EZRI_MOUSE	MK_SCX_17.9783.9783.2	2	6.117	0.551	1	1472.2	78.125	K.FYPEDVAEELIQDITQK.L
EZRI_MOUSE	MK_SCX_18.11350.11350.2	2	3.761	0.391	1	923.7	60.000004	K.IAQDLEMYGINYFEIK.N
EZRI_MOUSE	MK_SCX_20_1.5929.5929.3	3	4.322	0.535	1	1597.6	50	K.SQEQLAAELAEYTAK.I
EZRI_MOUSE	MK_SCX_20_1.6047.6047.2	2	5.818	0.639	1	2839.9	82.14286	K.SQEQLAAELAEYTAK.I
EZRI_MOUSE	MK_SCX_21.5123.5123.2	2	3.937	0.504	1	1292.1	79.16667	R.QLLTLSNELSQAR.D
EZRI_MOUSE	MK_SCX_2201.3903.3903.2	2	2.665	0.319	2	902.5	92.85714	K.IALLEEAR.R
EZRI_MOUSE	MK_SCX_2201.5118.5118.2	2	3.997	0.559	1	1205	88.88889	K.APDFVFYAPR.L
EZRI_MOUSE	MK_SCX_25.6264.6264.2	2	3.196	0.305	1	709.1	93.75	K.IGFPWSEIR.N
EZRI_MOUSE	MK_SCX_26.6130.6130.3	3	4.658	0.347	1	1442.9	34.523808	R.QAQDQIKSQEQLAAELAEYTAK.I
EZRI_MOUSE	MK_SCX_26.6189.6189.2	2	4.484	0.5	1	621.2	47.61905	R.QAQDQIKSQEQLAAELAEYTAK.I
EZRI_MOUSE	MK_SCX_28.14459.14459.2	2	5.894	0.63	1	2151.7	75	R.AKFYPEDVAEELIQDITQK.L
EZRI_MOUSE	MK_SCX_28.14581.14581.3	3	4.971	0.418	1	1039.6	40.27778	R.AKFYPEDVAEELIQDITQK.L
EZRI_MOUSE	MK_SCX_34.4547.4547.2	2	3.466	0.452	1	1307.7	79.16667	R.GMLKDSAM*LEYLK.I
EZRI_MOUSE	MK_SCX_34.4999.4999.3	3	4.102	0.339	1	1356.3	52.083332	R.GM*LKDSAMLEYLK.I
EZRI_MOUSE	MK_SCX_34.5704.5704.2	2	4.15	0.523	1	1328.7	83.33333	R.GMLKDSAMLEYLK.I
EZRI_MOUSE	MK_SCX_34.5743.5743.3	3	4.367	0.366	1	1102.6	50	R.GMLKDSAMLEYLK.I
EZRI_MOUSE	MK_SCX_38.4794.4794.2	2	3.587	0.574	1	672.4	75	K.KAPDFVFYAPR.L
EZRI_MOUSE	MK_SCX_39.5315.5315.3	3	4.002	0.334	1	1204.7	55	K.KAPDFVFYAPR.L
EZRI_MOUSE	MK_SCX_51.2788.2788.3	3	3.006	0.208	1	696.3	45	R.KEDEVEEWQHR.A
EZRI_MOUSE	MK_SCX_52.6026.6026.3	3	3.348	0.375	1	546.7	31.944445	K.FVIKPIDKKAPDFVFYAPR.L
EZRI_MOUSE	MK_SCX_54.2672.2672.3	3	3.244	0.177	1	869	43.18182	K.RAEKELSEQIEK.A
F107B_MOUSE	MK_SCX_20_1.3883.3883.2	2	3.49	0.41	1	1222.7	83.33333	K.LQEEQENAPEFVK.V
F10A1_MOUSE	MK_SCX_19.3798.3798.2	2	4.957	0.544	1	2063.7	90.909096	K.LDYDEDASAM*LR.E
F10A1_MOUSE	MK_SCX_19.4424.4424.2	2	4.696	0.544	1	2488.5	90.909096	K.LDYDEDASAMLR.E
F10A1_MOUSE	MK_SCX_20_1.4136.4136.2	2	3.979	0.248	1	1547.9	83.33333	R.AIEINPDSAQPYK.W
F10A1_MOUSE	MK_SCX_21.5570.5570.2	2	3.452	0.278	1	1001.6	88.88889	K.AIDLFTDAIK.L
F10A1_MOUSE	MK_SCX_30.6587.6587.2	2	4.665	0.541	1	2463.1	84.61539	K.AIDLFTDAIKLNPR.L
F10A1_MOUSE	MK_SCX_30.6599.6599.3	3	3.035	0.519	1	457.6	44.230766	K.AIDLFTDAIKLNPR.L
F10A1_MOUSE	MK_SCX_33.4933.4933.2	2	4.346	0.526	1	1161.3	75	R.AIEINPDSAQPYKWR.G
F10A1_MOUSE	MK_SCX_35.3823.3823.2	2	5.58	0.514	1	3017.6	85.71429	K.KGAAIEALNDGELQK.A
F10A1_MOUSE	MK_SCX_41.5316.5316.2	2	3.905	0.404	1	2232.5	90.909096	R.FLREWVESMGGK.V
F16P1_MOUSE	MK_SCX_13.6566.6566.2	2	3.972	0.159	1	1339.6	69.230774	R.LLYECNPIAYVM*EK.A
F16P1_MOUSE	MK_SCX_13.7917.7917.2	2	4.31	0.55	1	1917.9	80.769226	R.LLYECNPIAYVMEK.A
F16P1_MOUSE	MK_SCX_13.8840.8840.2	2	5.044	0.62	1	778.3	66.66667	K.AQGTGELTQLLNSLCTAIK.A
F16P1_MOUSE	MK_SCX_17.6554.6554.2	2	5.402	0.662	1	1789.2	66.66667	K.APVVM*GSSEDVQEFLEIYR.K
F16P1_MOUSE	MK_SCX_17.7839.7839.2	2	6.019	0.541	1	3320.3	77.77778	K.APVVMGSSEDVQEFLEIYR.K
F16P1_MOUSE	MK_SCX_18.5468.5468.2	2	4.33	0.481	1	1499.4	86.36364	K.DFDPAINEYLQR.K
F16P1_MOUSE	MK_SCX_18.5757.5757.3	3	4.244	0.452	1	431.3	32.142857	R.QAGIAQLYGIAGSTNVTGDQVK.K
F16P1_MOUSE	MK_SCX_18.5861.5861.2	2	6.015	0.468	1	1857.2	69.047615	R.QAGIAQLYGIAGSTNVTGDQVK.K
F16P1_MOUSE	MK_SCX_18.6892.6892.2	2	2.836	0.357	1	598.6	65.38461	K.LDILSNDLVINM*LK.S
F16P1_MOUSE	MK_SCX_18.8443.8443.2	2	5.047	0.414	1	1756.8	76.92308	K.LDILSNDLVINMLK.S
F16P1_MOUSE	MK_SCX_20_1.7364.7364.2	2	4.052	0.407	1	1739.2	80.769226	R.TLVYGGIFLYPANK.K
F16P1_MOUSE	MK_SCX_2201.3953.3953.2	2	3.958	0.507	1	1522.4	86.36364	K.GNIYSLNEGYAK.D
F16P1_MOUSE	MK_SCX_25.7384.7384.2	2	4.965	0.612	1	573.6	47.826088	K.GNIYSLNEGYAKDFDPAINEYLQR.K
F16P1_MOUSE	MK_SCX_25.7434.7434.3	3	5.784	0.635	1	2552.7	45.652176	K.GNIYSLNEGYAKDFDPAINEYLQR.K
F16P1_MOUSE	MK_SCX_26.6529.6529.3	3	5.864	0.586	1	1644	47.368423	K.APVVM*GSSEDVQEFLEIYRK.H
F16P1_MOUSE	MK_SCX_26.7172.7172.2	2	5.508	0.656	1	2255.4	65.789474	K.APVVMGSSEDVQEFLEIYRK.H
F16P1_MOUSE	MK_SCX_26.8351.8351.3	3	5.569	0.599	1	757.9	38.157894	K.APVVMGSSEDVQEFLEIYRK.H
F16P1_MOUSE	MK_SCX_27.5755.5755.3	3	5.684	0.428	1	1056.3	39.772728	R.QAGIAQLYGIAGSTNVTGDQVKK.L
F16P1_MOUSE	MK_SCX_28.5253.5253.2	2	4.244	0.499	1	587.9	87.5	K.DFDPAINEYLQRK.K

F16P1_MOUSE	MK_SCX_31.6902.6902.2	2	4.439	0.489	1	1102.5	75	K.KLDILSNDLVINM*LK.S
F16P1_MOUSE	MK_SCX_31.8240.8240.2	2	5.329	0.57	1	3168.9	89.28571	K.KLDILSNDLVINMLK.S
F16P1_MOUSE	MK_SCX_31.8540.8540.3	3	3.318	0.202	1	766.8	41.07143	K.KLDILSNDLVINMLK.S
F16P1_MOUSE	MK_SCX_32.5673.5673.2	2	3.162	0.257	1	320.6	46.42857	R.TLVYGGIFLYPANKK.S
F16P1_MOUSE	MK_SCX_34.6306.6306.2	2	5.775	0.562	1	1515.9	56.81818	K.AGGLATTGDKDILDIVPTEIHQK.A
F16P1_MOUSE	MK_SCX_35.9762.9762.3	3	7.202	0.561	1	1005.8	40.909092	K.AGGLATTGDKDILDIVPTEIHQK.A
F16P1_MOUSE	MK_SCX_36.3680.3680.2	2	3.496	0.518	1	1722.9	90	R.YVGSMVADIHR.T
F16P1_MOUSE	MK_SCX_41.3546.3546.3	3	3.341	0.387	1	386	41.666664	K.KGNIYSLNEGYAK.D
F16P1_MOUSE	MK_SCX_41.3584.3584.2	2	3.874	0.554	1	1711.7	83.33333	K.KGNIYSLNEGYAK.D
F16P1_MOUSE	MK_SCX_41.6946.6946.3	3	7.095	0.624	1	2019.7	45.833336	K.KGNIYSLNEGYAKDFDPAINEYLQR.K
F16P1_MOUSE	MK_SCX_41.7011.7011.2	2	5.147	0.667	1	715.4	52.083332	K.KGNIYSLNEGYAKDFDPAINEYLQR.K
F16P1_MOUSE	MK_SCX_55.3238.3238.3	3	3.952	0.408	1	655.5	44.230766	R.KKFPPDGSAPYGAR.Y
F16P2_MOUSE	MK_SCX_18.6017.6017.2	2	2.702	0.306	1	309.1	38.095238	R.KAGLANLYGISGSVNVTGDEVK.K
F16P2_MOUSE	MK_SCX_19.9976.9976.2	2	3.631	0.331	1	380.1	50	K.GTGELTQLLNSMLTAIK.A
FA82B_MOUSE	MK_SCX_19.5150.5150.2	2	3.944	0.481	1	797.6	73.333336	K.VLFANPPSSTYEEALR.Y
FA82B_MOUSE	MK_SCX_20_1.5660.5660.2	2	2.875	0.27	1	745.2	75	K.VLVYEALDYAK.R
FA82B_MOUSE	MK_SCX_25.6034.6034.2	2	2.053	0.324	1	679.8	92.85714	K.LAAFWLVK.A
FA82B_MOUSE	MK_SCX_45.5736.5736.2	2	2.023	0.23	1	461.6	75	K.KLAAFWLVK.A
FA82B_MOUSE	MK_SCX_51.3810.3810.3	3	3.717	0.396	1	1081	44.642857	R.YFHKAEEVDPNFYSK.N
FA96B_MOUSE	MK_SCX_26.4168.4168.3	3	3.961	0.201	1	523.3	35.714287	R.SGERPVTAGEEDEEVPDSIDAR.E
FA98B_MOUSE	MK_SCX_17.6346.6346.2	2	5.287	0.637	1	1156.8	76.666664	K.SDTSDIPLLLSQVESK.V
FAAA_MOUSE	MK_SCX_15.10135.10135.2	2	3.219	0.553	1	379.3	39.583336	K.SFGTTISPWVVPMDALMPFVVPNPK.Q
FAAA_MOUSE	MK_SCX_18.8184.8184.2	2	5.762	0.58	1	1586.7	80	R.IGVAIGDQILDLSVIK.H
FAAA_MOUSE	MK_SCX_23.4787.4787.2	2	4.49	0.526	1	1529.5	79.16667	R.ASLQNLLSASQAR.L
FAAA_MOUSE	MK_SCX_24.9044.9044.3	3	5.386	0.56	1	1607.8	37	R.AFTSQASATMHLPATIGDYTDFYSSR.Q
FAAA_MOUSE	MK_SCX_50.6657.6657.3	3	4.195	0.491	1	2465.6	46.42857	K.HQHVFDETTLNNFM*GLGQAAWK.E
FAAA_MOUSE	MK_SCX_50.6668.6668.2	2	5.77	0.657	1	910.7	59.523808	K.HQHVFDETTLNNFM*GLGQAAWK.E
FAAA_MOUSE	MK_SCX_50.7624.7624.2	2	5.888	0.689	1	1919.2	64.28571	K.HQHVFDETTLNNFMGLGQAAWK.E
FAAA_MOUSE	MK_SCX_50.7663.7663.3	3	5.362	0.64	1	1848.7	41.666664	K.HQHVFDETTLNNFMGLGQAAWK.E
FAAA_MOUSE	MK_SCX_55.7546.7546.3	3	3.728	0.413	1	414.4	31.578945	R.GKENALLPNWLHLPVGYHGR.A
FAAH_MOUSE	MK_SCX_15.9780.9780.2	2	4.669	0.559	1	1267.8	57.14286	R.LQNPDLDSEALLALPLLQLVQK.L
FABD_MOUSE	MK_SCX_17.3374.3374.2	2	3.539	0.212	1	1285.6	71.42857	R.DSSVAEEGAQEAVAR.R
FABPA_MOUSE	MK_SCX_15.5373.5373.2	2	2.314	0.383	1	371.6	63.636364	R.DGDKLVVECVMK.G
FABPA_MOUSE	MK_SCX_19.10615.10615.2	2	3.921	0.334	1	991.6	67.85714	K.SIITLDGGALVQVQK.W
FABPA_MOUSE	MK_SCX_19.4853.4853.2	2	4.708	0.581	1	2251.3	87.5	K.LGVEFDEITADDR.K
FABPA_MOUSE	MK_SCX_20_1.3914.3914.2	2	4.039	0.52	1	1150.1	86.36364	K.LVSSENFDDYM*K.E
FABPA_MOUSE	MK_SCX_25.7313.7313.2	2	4.828	0.608	1	1681	60.000004	K.LVSSENFDDYMKEVGVGFATR.K
FABPA_MOUSE	MK_SCX_25.7345.7345.3	3	5.458	0.515	1	726.9	43.75	K.LVSSENFDDYMKEVGVGFATR.K
FABPA_MOUSE	MK_SCX_29.4696.4696.3	3	4.329	0.498	1	757.4	48.076923	K.LGVEFDEITADDRK.V
FABPA_MOUSE	MK_SCX_29.4771.4771.2	2	4.626	0.513	1	2258.2	76.92308	K.LGVEFDEITADDRK.V
FABPA_MOUSE	MK_SCX_42.6388.6388.3	3	4.475	0.509	1	1478.7	42.857143	R.KVAGMAKPNMIISVNGDLVTIR.S
FABPB_MOUSE	MK_SCX_30.3831.3831.3	3	3.921	0.504	1	615.8	39.705883	R.QVGNVTKPTVIISQEGGK.V
FABPB_MOUSE	MK_SCX_51.4133.4133.3	3	3.109	0.348	1	1093.4	48.333332	K.LIHVQKWDGKETNCTR.E
FABPH_MOUSE	MK_SCX_21.3547.3547.2	2	2.344	0.301	1	463.5	61.11111	K.WNGQETTLTR.E
FABPH_MOUSE	MK_SCX_2201.4977.4977.3	3	4.211	0.136	1	823.7	35.714287	R.QVASMTKPTTIIEKNGDTITIK.T
FABPH_MOUSE	MK_SCX_25.3949.3949.2	2	2.57	0.322	1	741.5	81.25	K.SLGVGFATR.Q
FABPH_MOUSE	MK_SCX_33.4265.4265.2	2	3.522	0.553	1	908	77.27273	K.LVDSKNFDDYMK.S
FABPH_MOUSE	MK_SCX_48.4255.4255.2	2	3.018	0.213	1	1030.1	60.000004	K.LIHVQKWNGQETTLTR.E
FABPH_MOUSE	MK_SCX_49.4961.4961.2	2	4.627	0.55	1	2050.7	75	K.SLVTLDGGKLIHVQK.W
FABPH_MOUSE	MK_SCX_51.4142.4142.3	3	3.109	0.369	1	1338.8	51.666664	K.LIHVQKWNGQETTLTR.E
FABPL_MOUSE	MK_SCX_20_1.5610.5610.2	2	2.452	0.423	1	329.3	60.000004	K.AIGLPEDLIQK.G

FABPL_MOUSE	MK_SCX_25.5747.5747.2	2	2.063	0.142	1	331.9	60.000004	K.KIKLTITYGPK.V
FAHD1_MOUSE	MK_SCX_18.6566.6566.2	2	5.251	0.556	1	2826.9	83.33333	K.IITLEEGDLILTGTPK.G
FAHD1_MOUSE	MK_SCX_18.9040.9040.2	2	4.304	0.503	1	470.3	53.125	K.TSSM*IFSIPYIISYVSK.I
FAHD1_MOUSE	MK_SCX_18.9806.9806.2	2	4.224	0.546	1	558.8	62.5	K.TSSMIFSIPYIISYVSK.I
FAHD1_MOUSE	MK_SCX_23.5677.5677.3	3	3.387	0.344	1	1523.8	36.904762	K.GVGPVKENDEIEAGIDGVVSM*R.F
FAHD1_MOUSE	MK_SCX_26.12537.12537.3	3	4.527	0.539	1	1152.8	38.095238	K.GVGPVKENDEIEAGIDGVVSMR.F
FAHD1_MOUSE	MK_SCX_52.16698.16698.3	3	3.232	0.334	1	1215.1	48.076923	R.NLHHEVELGVLLGK.R
FAHD1_MOUSE	MK_SCX_52.4648.4648.2	2	2.858	0.385	1	1074.2	69.230774	R.NLHHEVELGVLLGK.R
FARP2_MOUSE	MK_SCX_19.5358.5358.2	2	2.343	0.148	1	308.8	50	K.GDHQRIGDILLRNMR.Q
FARP2_MOUSE	MK_SCX_31.6987.6987.2	2	2.712	0.241	1	721.1	56.25	K.TIGDSPPVLLGGPVYTR.T
FBRL_MOUSE	MK_SCX_28.4590.4590.3	3	5.053	0.41	1	876.4	37.5	K.MQQENMKPQEQLTLEPYER.D
FBRL_MOUSE	MK_SCX_32.3689.3689.3	3	3.256	0.513	1	875.9	50	R.VSISEGDDKIEYR.A
FBRL_MOUSE	MK_SCX_32.3695.3695.2	2	3.613	0.567	1	1477	75	R.VSISEGDDKIEYR.A
FBRL_MOUSE	MK_SCX_45.3577.3577.3	3	3.871	0.538	1	900.6	48.076923	R.DHAVVVGVYRPPPK.V
FBRL_MOUSE	MK_SCX_46.4488.4488.3	3	4.808	0.476	1	1393.6	38.157894	K.KMQQENMKPQEQLTLEPYER.D
FBX38_MOUSE	MK_SCX_18.3890.3890.2	2	3.76	0.403	1	599	52.77778	R.TVTSSGSSEPSPPEVDVSR.Q
FCL_MOUSE	MK_SCX_16.5299.5299.2	2	5.798	0.494	1	2685.1	83.33333	K.DADLTDAAQTQALFQK.V
FETUA_MOUSE	MK_SCX_47.4856.4856.3	3	5.851	0.639	1	2188.4	53.750004	R.HAFSPVASVESASGETLHSPK.V
FHIT_MOUSE	MK_SCX_36.3685.3685.2	2	3.9	0.493	1	2165.9	87.5	R.SEKEMAAEAEALR.V
FHIT_MOUSE	MK_SCX_46.6603.6603.3	3	3.719	0.378	1	1075.4	47.058823	R.FRDLHPDEVADLFQVTQR.V
FHIT_MOUSE	MK_SCX_51.5422.5422.2	2	2.938	0.179	1	346.7	57.692307	R.FGQHLIKPSVVFLK.T
FHL1_MOUSE	MK_SCX_19.4708.4708.2	2	3.952	0.265	1	993.4	57.14286	R.FTAVEDQYYCVDCYK.N
FHL1_MOUSE	MK_SCX_21.3618.3618.2	2	3.341	0.442	1	1168.7	77.27273	K.AIVAGDQNVEYK.G
FHL1_MOUSE	MK_SCX_27.6485.6485.3	3	3.288	0.274	1	461.1	30.263159	R.FTAVEDQYYCVDCYKNFVAK.K
FHL1_MOUSE	MK_SCX_35.5224.5224.2	2	3.263	0.274	1	1008	60.714287	R.FVFHNEQVYCPDCAK.K
FHL1_MOUSE	MK_SCX_44.4162.4162.3	3	3.076	0.36	1	543.1	38.235294	K.AIVAGDQNVEYKGTVWHK.D
FHL1_MOUSE	MK_SCX_49.5372.5372.2	2	3.522	0.509	1	1086.8	50	K.GSSVVAYEGQSWHDYCFHCK.K
FHL1_MOUSE	MK_SCX_50.9661.9661.3	3	3.498	0.371	1	629.1	30.263159	K.GEDFYCVTCHETKFAKHCVK.C
FHL1_MOUSE	MK_SCX_56.3538.3538.3	3	4.707	0.516	1	1127.5	45.833336	R.CAKCLHPLASETFVSKDGK.I
FHL2_MOUSE	MK_SCX_18.4638.4638.2	2	2.149	0.179	1	416.4	43.75	K.EEQLLCTDCYSNEYSSK.C
FHL2_MOUSE	MK_SCX_28.6674.6674.3	3	3.355	0.291	1	464.9	35.294117	R.GFLTERDDILCPDCGK.D
FIBG_MOUSE	MK_SCX_24.4862.4862.3	3	4.466	0.522	1	832.9	32.954548	K.AIQVYYNPDQPPKPGMIDSATQK.S
FINC_MOUSE	MK_SCX_23.4538.4538.2	2	2.291	0.437	1	312.3	63.636364	R.WLPSTSPVTGYR.V
FINC_MOUSE	MK_SCX_45.4088.4088.3	3	3.57	0.483	1	890.3	50	R.WSRPQAPITGYR.I
FIP1_MOUSE	MK_SCX_19.4748.4748.2	2	4.711	0.473	1	677.2	50	K.TGAPQYGSYGTAPVNLNIK.A
FIS1_MOUSE	MK_SCX_20_1.9090.9090.2	2	2.693	0.406	1	797.7	75	R.GIVLLEELLPK.G
FIS1_MOUSE	MK_SCX_31.3914.3914.3	3	3.527	0.292	1	662.6	37.5	R.GLLQTEPQNNQAKELER.L
FIS1_MOUSE	MK_SCX_31.3936.3936.2	2	4.011	0.353	1	424.7	53.125	R.GLLQTEPQNNQAKELER.L
FKB11_MOUSE	MK_SCX_25.6533.6533.3	3	4.833	0.498	1	2443.9	52.941177	R.IIDTSLTRDPLVIELGQK.Q
FKB1A_MOUSE	MK_SCX_20_1.3571.3571.2	2	3.83	0.518	1	1330	83.33333	-.GVQVETISPGDGR.T
FKB1A_MOUSE	MK_SCX_2201.3808.3808.2	2	5.134	0.618	1	2135.7	88.46153	R.GWEEGVAQM*SVGQR.A
FKB1A_MOUSE	MK_SCX_2201.4834.4834.2	2	4.916	0.376	1	2126.2	80.769226	R.GWEEGVAQMSVGQR.A
FKBP2_MOUSE	MK_SCX_14.7766.7766.3	3	4.822	0.429	1	900.2	33.333336	K.LEDGTEFDSSLPQNQPFVFSLGTGQVIK.G
FKBP2_MOUSE	MK_SCX_14.7776.7776.2	2	4.108	0.622	1	372.6	38.88889	K.LEDGTEFDSSLPQNQPFVFSLGTGQVIK.G
FKBP2_MOUSE	MK_SCX_37.5188.5188.3	3	4.137	0.288	1	1928.4	58.333332	R.KLVIPSELGYGER.G
FKBP2_MOUSE	MK_SCX_38.5014.5014.2	2	3.244	0.391	1	590.2	75	R.KLVIPSELGYGER.G
FKBP3_MOUSE	MK_SCX_21.5229.5229.2	2	3.638	0.394	1	1117.7	90	R.LEIEPEWAYGK.K
FKBP3_MOUSE	MK_SCX_21.5718.5718.2	2	2.846	0.383	1	599.7	70	R.GWDEALLTMSK.G
FKBP3_MOUSE	MK_SCX_2201.4508.4508.2	2	3.891	0.486	1	1711.2	85	R.GWDEALLTM*SK.G
FKBP3_MOUSE	MK_SCX_31.3218.3218.3	3	3.418	0.414	1	377.7	42.307693	K.SEETLDEGPPKYTK.S
FKBP3_MOUSE	MK_SCX_31.3238.3238.2	2	3.988	0.364	1	1117.2	69.230774	K.SEETLDEGPPKYTK.S

FKBP3_MOUSE	MK_SCX_33.4673.4673.2	2	3.377	0.227	1	880.9	86.36364	R.LEIEPEWAYGKK.G
FKBP3_MOUSE	MK_SCX_46.3968.3968.2	2	5.109	0.549	1	1861.6	78.57143	K.FLQDHGSDSFLAEHK.L
FKBP3_MOUSE	MK_SCX_46.4001.4001.3	3	4.608	0.474	1	1070.9	51.785713	K.FLQDHGSDSFLAEHK.L
FKBP3_MOUSE	MK_SCX_51.2926.2926.3	3	3.728	0.227	1	1423.9	36.25	K.LSDDKPKDSKSEETLDEGPPK.Y
FKBP3_MOUSE	MK_SCX_55.4440.4440.3	3	5.28	0.588	1	1181.3	47.058823	K.TANKDHLVNAYNHLFESK.R
FKBP4_MOUSE	MK_SCX_16.5664.5664.2	2	4.173	0.433	1	1014.2	62.5	K.AAENGAQSAPLPLEGVDISPK.Q
FKBP4_MOUSE	MK_SCX_2201.9076.9076.3	3	3.139	0.252	1	454.1	24.107143	R.LFDQRELCFEVGEGESLDLPCGLEEAIQR.M
FKBP4_MOUSE	MK_SCX_34.6400.6400.2	2	2.132	0.189	1	305.4	57.692307	R.VFVHYTGWLLDGTK.F
FKBP4_MOUSE	MK_SCX_42.3869.3869.3	3	5.489	0.604	1	2244.7	45.454548	K.VGEVCHITCKPEYAYGAAGSPPK.I
FKBP4_MOUSE	MK_SCX_43.2857.2857.3	3	4.281	0.579	1	1196	45.588234	K.AEVAAGDHPTDAEMKGER.N
FKBP4_MOUSE	MK_SCX_45.3031.3031.3	3	4.103	0.495	1	425	39.0625	K.VKAEVAAGDHPTDAEMK.G
FKBP4_MOUSE	MK_SCX_45.5304.5304.3	3	3.21	0.386	1	623.2	44.230766	K.DKFSFDLGKGEVIK.A
FKBP4_MOUSE	MK_SCX_46.5185.5185.2	2	5.176	0.611	1	1231.8	63.88889	K.GEHSIVYLKPSYAFGSVGK.E
FKBP4_MOUSE	MK_SCX_53.4432.4432.2	2	4.257	0.408	1	1703.6	82.14286	R.RGEAHLAVNDFDLAR.A
FKBP4_MOUSE	MK_SCX_53.4457.4457.3	3	4.675	0.494	1	3335.5	60.714287	R.RGEAHLAVNDFDLAR.A
FKBP4_MOUSE	MK_SCX_53.5180.5180.3	3	5.451	0.412	1	1892.2	51.19048	R.MEKGEHSIVYLKPSYAFGSVGK.E
FKBP4_MOUSE	MK_SCX_54.4758.4758.3	3	3.987	0.453	1	664.9	32.5	K.GEHSIVYLKPSYAFGSVGKER.F
FKBP4_MOUSE	MK_SCX_56.4895.4895.3	3	5.543	0.609	1	1486.1	42.391304	R.MEKGEHSIVYLKPSYAFGSVGKER.F
FKBP5_MOUSE	MK_SCX_17.7615.7615.2	2	3.46	0.38	1	907.1	66.66667	K.FGIDPNAELMYEVTLK.S
FKBP5_MOUSE	MK_SCX_20_1.3402.3402.2	2	3.868	0.492	1	870.3	75	R.VGTSDEAPM*FGDK.V
FKBP5_MOUSE	MK_SCX_20_1.3771.3771.2	2	3.857	0.499	1	1041.4	79.16667	R.VGTSDEAPMFGDK.V
FKBP8_MOUSE	MK_SCX_13.9767.9767.2	2	2.711	0.449	1	532.9	35.714287	R.EFLAATEPEPAPAPAPEEWLDILGNGLLR.M
FKBP8_MOUSE	MK_SCX_14.10166.10166.3	3	3.143	0.248	1	306.4	20.535715	R.EFLAATEPEPAPAPAPEEWLDILGNGLLR.M
FKBP8_MOUSE	MK_SCX_19.5297.5297.2	2	3.129	0.485	1	669.8	60.000004	K.VLAQQGEYSEAIPILR.A
FLNA_MOUSE	MK_SCX_16.5985.5985.2	2	3.262	0.51	1	499.9	41.304348	K.GLVEPVDVVDNADGTQTVNYVPSR.E
FLNA_MOUSE	MK_SCX_16.6955.6955.2	2	4.356	0.478	1	499.9	45.652176	K.ASGPGLNTTGVPASLPVEFTIDAK.D
FLNA_MOUSE	MK_SCX_21.3732.3732.2	2	3.283	0.565	1	1050.9	80	R.EATTEFSVDAR.A
FLNA_MOUSE	MK_SCX_2201.4604.4604.2	2	3.395	0.49	1	840.1	70.83333	R.AWGPGLEGGIVGK.S
FLNA_MOUSE	MK_SCX_2201.5844.5844.2	2	3.225	0.427	1	792.8	85	K.LPQLPITNFSR.D
FLNA_MOUSE	MK_SCX_2201.7722.7722.3	3	4.973	0.537	1	599.2	30.172413	R.ALGALVDSCAPGLCPDWDSWDASKPVNNAR.E
FLNA_MOUSE	MK_SCX_25.6863.6863.3	3	3.155	0.256	1	448.7	23.148148	R.FGGEHVPNSPFQVTALAGDQPTVQTPLR.S
FLNA_MOUSE	MK_SCX_27.3932.3932.3	3	6.057	0.394	1	1896.9	46.739132	K.SQGDASKVTAQGPGLEPSGNIANK.T
FLNA_MOUSE	MK_SCX_28.3375.3375.3	3	3.787	0.603	1	851.1	43.055553	K.VATVPQHATSGPGPADVSK.V
FLNA_MOUSE	MK_SCX_36.4002.4002.3	3	3.655	0.26	1	1004.8	46.153847	K.YNDQHIPGSPFTAR.V
FLNA_MOUSE	MK_SCX_36.4028.4028.2	2	2.615	0.349	1	407.8	61.538464	K.YNDQHIPGSPFTAR.V
FLNA_MOUSE	MK_SCX_49.3996.3996.3	3	3.576	0.424	1	611	35	R.TGVELGKPTHFTVNAK.T
FLNB_MOUSE	MK_SCX_14.3888.3888.2	2	2.353	0.269	1	397.9	63.636364	R.SSTETCYSAIPK.S
FLNB_MOUSE	MK_SCX_15.15340.15340.3	3	4.428	0.124	1	484.1	26.923079	R.SADFVVESIGSEVGTLGFAIEGPSQAK.I
FLNB_MOUSE	MK_SCX_15.6936.6936.2	2	4.915	0.618	1	796.4	43.103447	K.VGEPGILCVDCSEAGPGTLGLEAVSDSGAK.A
FLNB_MOUSE	MK_SCX_19.4581.4581.2	2	3.223	0.244	1	512.4	53.125	K.SPFGVQIGEACNPNACR.A
FLNB_MOUSE	MK_SCX_19.5645.5645.2	2	4.039	0.373	1	1286.5	67.85714	K.SPFTVGVAAPLDLSK.I
FLNB_MOUSE	MK_SCX_19.5987.5987.2	2	3.164	0.5	1	391	56.666668	K.VMYTPMAPGNYLIGVK.Y
FLNB_MOUSE	MK_SCX_19.6750.6750.2	2	4.972	0.524	1	1218.1	78.125	K.IPYLPITNFNQNWQDGK.A
FLNB_MOUSE	MK_SCX_20_1.6772.6772.2	2	3.358	0.253	1	508.8	61.538464	K.VLFASQEIPASPFR.V
FLNB_MOUSE	MK_SCX_23.3355.3355.2	2	3.696	0.511	1	1286.8	81.818184	K.AAGSGELGVTVK.G
FLNB_MOUSE	MK_SCX_30.5370.5370.3	3	3.896	0.398	1	738.8	32.5	K.GAGKAPLIVQFSSPLPGEAVK.D
FLNB_MOUSE	MK_SCX_32.4572.4572.2	2	2.659	0.256	1	364.2	53.846157	K.FADEHVPGSPFTVK.I
FLNB_MOUSE	MK_SCX_34.6605.6605.2	2	2.104	0.18	1	446.4	57.692307	K.YGGELVPHFPAWVK.V
FLNB_MOUSE	MK_SCX_36.5056.5056.3	3	4.227	0.51	1	915.7	33.333336	R.EATTDFTVDSRPLTQVGGDHIK.A
FLNB_MOUSE	MK_SCX_41.4196.4196.3	3	3.719	0.485	1	774.2	35.526314	K.SPFEVNVDKAQGDASKVTAK.G
FMO2_MOUSE	MK_SCX_31.5120.5120.3	3	4.402	0.299	1	1074.6	48.214287	K.YLMKEPVLNDDLPSR.L

FMR1_MOUSE	MK_SCX_19.8290.8290.2	2	3.666	0.474	1	809.9	65.38461	R.SFLEFAEDVIQVPR.N
FN3K_MOUSE	MK_SCX_31.4735.4735.2	2	4.197	0.459	1	1416.7	65.38461	K.LGEQMADLHLYNQK.L
FOLH1_MOUSE	MK_SCX_20_1.3529.3529.2	2	3.076	0.366	1	523.8	68.181816	K.ELQSPDEGFEGK.S
FOLH1_MOUSE	MK_SCX_21.6611.6611.2	2	5.111	0.57	1	1150	75	K.LGSGNDFEVFFQR.L
FOLH1_MOUSE	MK_SCX_2201.4851.4851.2	2	4.792	0.555	1	769.4	75	K.SYPDGWNLPGGGVQR.G
FOLH1_MOUSE	MK_SCX_2201.5066.5066.2	2	3.647	0.435	1	1251.9	80	R.IMNDQLMYLER.A
FOLH1_MOUSE	MK_SCX_23.4378.4378.2	2	3.333	0.47	1	1374.2	90	K.SPSPEFIGMPR.I
FOLH1_MOUSE	MK_SCX_26.5650.5650.3	3	3.099	0.318	1	984.8	33.333336	R.GNVLNLNGAGDPLTPGYPANEHAYR.H
FOLH1_MOUSE	MK_SCX_31.4629.4629.2	2	4.45	0.342	1	1871.3	83.33333	R.LQELDKSNPILLR.I
FOLH1_MOUSE	MK_SCX_31.4650.4650.3	3	3.803	0.345	1	431.8	43.75	R.LQELDKSNPILLR.I
FOLH1_MOUSE	MK_SCX_36.3990.3990.3	3	3.836	0.436	1	766.7	46.42857	R.TPHLAGTQNNFELAK.Q
FOLH1_MOUSE	MK_SCX_36.4012.4012.2	2	4.027	0.502	1	668.8	75	R.TPHLAGTQNNFELAK.Q
FOLH1_MOUSE	MK_SCX_42.6317.6317.3	3	3.971	0.401	1	593	32.894737	K.SLYDSWKEKSPSPEFIGMPR.I
FOLH1_MOUSE	MK_SCX_49.4589.4589.3	3	3.913	0.368	1	1323.6	50	K.GALEPDRYVILGGHR.D
FOLH1_MOUSE	MK_SCX_49.4612.4612.2	2	2.775	0.32	1	556.5	60.714287	K.GALEPDRYVILGGHR.D
FOLR1_MOUSE	MK_SCX_13.6752.6752.2	2	3.26	0.395	1	417.8	47.22222	R.CIQMWFDPAQGNPNEEVAR.F
FOLR1_MOUSE	MK_SCX_28.11273.11273.3	3	6.188	0.582	1	2693.1	39.423077	R.HFIQDTCLYECSPNLGPWIQQVDQSWR.K
FOLR1_MOUSE	MK_SCX_43.4138.4138.2	2	2.426	0.161	1	587.9	57.692307	R.FNWNHCGTMTSECK.R
FRIH_MOUSE	MK_SCX_06.7597.7597.2	2	2.404	0.155	1	313.8	46.666668	R.KM*GAPEAGM*AEYLFDK.H
FRIH_MOUSE	MK_SCX_19.5818.5818.2	2	4.147	0.418	1	930	71.42857	K.MGAPEAGMAEYLFDK.H
FRIH_MOUSE	MK_SCX_39.3198.3198.3	3	4.241	0.377	1	2266.7	62.5	R.QNYHQDAEAAINR.Q
FRIH_MOUSE	MK_SCX_52.3539.3539.3	3	4.105	0.338	1	838	50	K.SIKELGDHVTNLR.K
FRIL1_MOUSE	MK_SCX_17.5713.5713.2	2	5.413	0.624	1	1372.6	61.363636	R.VAGPQPAQTGAPQGSLGEYLFER.L
FRIL1_MOUSE	MK_SCX_17.5735.5735.3	3	5.648	0.656	1	2100.5	43.18182	R.VAGPQPAQTGAPQGSLGEYLFER.L
FRIL1_MOUSE	MK_SCX_30.4542.4542.2	2	4.237	0.381	1	1765.5	70	R.ALFQDVQKPSQDEWGK.T
FRIL1_MOUSE	MK_SCX_31.5715.5715.3	3	5.939	0.573	1	2543.5	45.652176	R.RVAGPQPAQTGAPQGSLGEYLFER.L
FRIL1_MOUSE	MK_SCX_31.5752.5752.2	2	5.835	0.666	1	841.1	50	R.RVAGPQPAQTGAPQGSLGEYLFER.L
FRIL1_MOUSE	MK_SCX_31.8264.8264.3	3	6.819	0.534	1	2255.8	39.285713	R.ALFQDVQKPSQDEWGKTQEAMEAALAMEK.N
FRIL1_MOUSE	MK_SCX_33.6662.6662.2	2	5.356	0.606	1	2748.1	80	K.NLNQALLDLHALGSAR.T
FRIL1_MOUSE	MK_SCX_33.6664.6664.3	3	3.547	0.332	1	883.3	45	K.NLNQALLDLHALGSAR.T
FSCN1_MOUSE	MK_SCX_31.5709.5709.3	3	4.071	0.41	1	1190.4	46.666668	R.LVARPEPATGFTLEFR.S
FTHFD_MOUSE	MK_SCX_17.6469.6469.2	2	3.648	0.34	1	620.6	76.666664	R.ILPNVPEVEDSTDFFK.S
FTHFD_MOUSE	MK_SCX_18.7169.7169.2	2	6.052	0.568	1	2198.2	62.5	K.GSASSALELTEEELATAEAVR.S
FTHFD_MOUSE	MK_SCX_20_1.4784.4784.2	2	3.834	0.515	1	2066.7	90.909096	R.FADGDVDAVLSR.A
FTHFD_MOUSE	MK_SCX_20_1.5861.5861.2	2	3.894	0.561	1	650.5	66.66667	K.GVVNILPGSGSLVGQR.L
FTHFD_MOUSE	MK_SCX_20_1.6011.6011.2	2	2.492	0.122	1	433.3	59.090908	K.EESFGPIM*IISR.F
FTHFD_MOUSE	MK_SCX_21.5722.5722.2	2	3.599	0.456	1	1013.4	67.85714	R.ANATEFGLASGVFTR.D
FTHFD_MOUSE	MK_SCX_2201.3578.3578.2	2	2.551	0.34	1	404.9	65	K.IGFTGSTEVGK.H
FTHFD_MOUSE	MK_SCX_24.2742.2742.2	2	2.07	0.124	1	318.9	75	R.LIAEGTAPR.R
FTHFD_MOUSE	MK_SCX_24.4956.4956.2	2	2.222	0.191	1	716.4	92.85714	R.FLFPEGIK.G
FTHFD_MOUSE	MK_SCX_26.5541.5541.2	2	2.817	0.393	1	307.1	50	K.ADPLGLEAEKDGVPVFK.F
FTHFD_MOUSE	MK_SCX_38.3201.3201.3	3	4.659	0.505	1	1920.7	57.14286	R.RPQPEEGATYEGIQK.K
FTHFD_MOUSE	MK_SCX_38.3222.3222.2	2	4.352	0.401	1	1682.6	78.57143	R.RPQPEEGATYEGIQK.K
FTHFD_MOUSE	MK_SCX_38.7111.7111.3	3	3.153	0.46	1	478.2	31.578945	K.ADPLGLEAEKDGVPVFKFPR.W
FTHFD_MOUSE	MK_SCX_41.4630.4630.3	3	3.068	0.459	1	373.4	29.6875	K.EGHEVVGVFTIPDKDGK.A
FTHFD_MOUSE	MK_SCX_47.7741.7741.3	3	4.904	0.477	1	1264.5	43.421055	K.KETAMINWDQPAEAIHNWIR.G
FTHFD_MOUSE	MK_SCX_51.2845.2845.3	3	4.434	0.416	1	1208.8	45	R.RPQPEEGATYEGIQKK.E
FTHFD_MOUSE	MK_SCX_53.4217.4217.3	3	3.657	0.436	1	917.8	47.916664	R.HGSIIYHPSLLPR.H
FUBP1_MOUSE	MK_SCX_16.6156.6156.2	2	4.818	0.635	1	836	51.851852	K.MGQAVPAPAGAPPGGQPDYSAAWAEYYR.Q
FUBP1_MOUSE	MK_SCX_21.4955.4955.2	2	4.196	0.514	1	1459.9	83.33333	R.IGGNEGIDVPIPR.F
FUBP1_MOUSE	MK_SCX_26.7560.7560.3	3	3.064	0.407	1	1210	40.789474	R.SVMTEEYKVPDGMVGFIIGR.G

FUBP1_MOUSE	MK_SCX_26.7592.7592.2	2	3.428	0.437	1	564.5	44.736843	R.SVMTEEYKVPDGMVGFIIGR.G
FUBP1_MOUSE	MK_SCX_30.3918.3918.3	3	3.568	0.46	1	690	39.705883	K.MVMIQDGPQNTGADKPLR.I
FUCO_MOUSE	MK_SCX_33.8164.8164.3	3	4.282	0.325	1	1588.8	48.4375	R.FFHPDQWAELFQAAGAK.Y
FUCO_MOUSE	MK_SCX_43.5087.5087.3	3	3.577	0.321	1	1404.7	43.75	K.DVGPHRDLVGELGAAVR.K
FUMH_MOUSE	MK_SCX_17.6401.6401.2	2	4.031	0.457	1	442.1	50	R.SGLGELILPENEPGSSIMPGK.V
FUMH_MOUSE	MK_SCX_20_1.3729.3729.2	2	4.881	0.438	1	1462	76.92308	R.AAAEVNQEYGLDPK.I
FUMH_MOUSE	MK_SCX_20_1.9320.9320.2	2	3.746	0.53	1	880	87.5	R.MPIPVIQAFGILK.R
FUMH_MOUSE	MK_SCX_2201.4168.4168.2	2	2.228	0.242	1	427.4	71.42857	K.VLLPGLQK.L
FUMH_MOUSE	MK_SCX_25.7931.7931.3	3	6.944	0.602	1	2750.7	40.17857	K.LNDHFPLVVWQTGSGTQTNMNVNEVISNR.A
FUMH_MOUSE	MK_SCX_25.9636.9636.3	3	5.833	0.618	1	1054.2	35.185184	R.THTQDAVPLTLGQEFSGYVQQVQYAMVR.I
FUMH_MOUSE	MK_SCX_26.7506.7506.2	2	5.256	0.606	1	1279.6	57.894737	K.LMNESLMLVTALNPHIGYDK.A
FUMH_MOUSE	MK_SCX_26.7511.7511.3	3	6.303	0.572	1	1305.5	51.315792	K.LMNESLMLVTALNPHIGYDK.A
FUMH_MOUSE	MK_SCX_32.7400.7400.3	3	3.671	0.422	1	696	53.846157	R.M*PIPVIQAFGILKR.A
FUMH_MOUSE	MK_SCX_32.8076.8076.2	2	3.376	0.546	1	948.9	76.92308	R.MPIPVIQAFGILKR.A
FUMH_MOUSE	MK_SCX_32.8327.8327.3	3	3.85	0.616	1	564.2	44.230766	R.MPIPVIQAFGILKR.A
FUMH_MOUSE	MK_SCX_35.10929.10929.3	3	4.944	0.532	1	2282.6	42.045452	R.VEFDTFGELKVPTDKYYGAQTVR.S
FUMH_MOUSE	MK_SCX_38.3441.3441.2	2	3.825	0.386	1	495.6	78.57143	K.RAAAEVNQEYGLDPK.I
FUMH_MOUSE	MK_SCX_38.3448.3448.3	3	4.303	0.45	1	1883.4	51.785713	K.RAAAEVNQEYGLDPK.I
FUMH_MOUSE	MK_SCX_56.3675.3675.3	3	5.819	0.46	1	1374.5	38.636364	R.AIEMLGGELGSKKPVHPNDHVNK.S
FUS_MOUSE	MK_SCX_20_1.3760.3760.2	2	4.631	0.537	1	1330.5	80.769226	K.GEATVSFDDPPSAK.A
FUS_MOUSE	MK_SCX_25.5309.5309.2	2	3.067	0.345	1	996	57.692307	K.CPNPTCENMNFSWR.N
FUS_MOUSE	MK_SCX_29.6580.6580.2	2	4.982	0.539	1	1948.9	75	K.AAIDWFDGKEFSGNPIK.V
FUS_MOUSE	MK_SCX_29.6776.6776.3	3	4.412	0.404	1	1847.8	51.5625	K.AAIDWFDGKEFSGNPIK.V
FUS_MOUSE	MK_SCX_33.4027.4027.3	3	3.956	0.4	1	1692.9	46.666668	K.LKGEATVSFDDPPSAK.A
FUS_MOUSE	MK_SCX_34.5211.5211.3	3	3.31	0.342	1	913.2	38.88889	R.AGDWKCPNPTCENM*NFSWR.N
FUS_MOUSE	MK_SCX_34.5961.5961.3	3	4.824	0.307	1	698	34.72222	R.AGDWKCPNPTCENMNFSWR.N
FUS_MOUSE	MK_SCX_34.6005.6005.2	2	3.351	0.339	1	308.1	50	R.AGDWKCPNPTCENMNFSWR.N
FUS_MOUSE	MK_SCX_47.3047.3047.3	3	3.942	0.484	1	1414.8	40.217392	K.APKPDGPGGGPGGSHMGGNYGDDR.R
FUS_MOUSE	MK_SCX_50.3657.3657.3	3	3.811	0.545	1	546.5	37.5	K.KTGQPMINLYTDRETGK.L
FUSIP_MOUSE	MK_SCX_17.7788.7788.2	2	3.345	0.544	1	717.6	53.333336	R.YGPIVDVYVPLDFYTR.R
FUSIP_MOUSE	MK_SCX_2201.4193.4193.2	2	2.382	0.171	1	417.9	65	R.QIEIQFAQGDR.K
FUSIP_MOUSE	MK_SCX_40.5038.5038.2	2	2.68	0.403	1	422.9	59.090908	R.YLRPPNTSLFVR.N
FXR1_MOUSE	MK_SCX_19.10426.10426.2	2	2.414	0.277	1	376.1	50	R.GFLEFVEDFIQVPR.N
FXR1_MOUSE	MK_SCX_20_1.3441.3441.2	2	4.652	0.526	1	1526.1	73.333336	K.GYATDESTVSSVQGSR.S
FXR1_MOUSE	MK_SCX_32.5435.5435.3	3	4.219	0.301	1	1903.5	47.058823	R.KVPGVTAIELDEDTGTFR.I
FXR1_MOUSE	MK_SCX_33.3210.3210.3	3	3.977	0.409	1	768.2	39.705883	R.EKGYATDESTVSSVQGSR.S
FXR1_MOUSE	MK_SCX_34.3571.3571.3	3	3.524	0.42	1	1059	46.153847	K.KEISEGDEVEVYSR.A
G3B2_MOUSE	MK_SCX_27.7760.7760.3	3	4.532	0.447	1	1085	36.904762	K.GVGGKLPNFGFVVFDDSEPVQR.I
G3B2_MOUSE	MK_SCX_32.2706.2706.3	3	3.171	0.369	1	1349	54.166668	R.VDAKPEVQSQPPR.V
G3BP_MOUSE	MK_SCX_18.3977.3977.2	2	4.429	0.553	1	1664.3	75	K.STSPAPADVAPAQEDLR.T
G3BP_MOUSE	MK_SCX_19.5157.5157.2	2	5.152	0.496	1	1010.3	73.333336	R.FMQTFVLAPEGSVANK.F
G3BP_MOUSE	MK_SCX_25.4667.4667.2	2	3.394	0.395	1	985.4	88.88889	R.TFSWASVTSK.N
G3BP_MOUSE	MK_SCX_26.6988.6988.2	2	5.179	0.608	1	1081.8	54.545456	R.INSGGKLPNFGFVVFDDSEPVQK.V
G3BP_MOUSE	MK_SCX_26.6992.6992.3	3	5.031	0.538	1	612	31.818182	R.INSGGKLPNFGFVVFDDSEPVQK.V
G3BP_MOUSE	MK_SCX_29.5979.5979.3	3	3.362	0.375	1	945.7	42.857143	R.QYYTLLNQAPDMLHR.F
G3BP_MOUSE	MK_SCX_30.8670.8670.3	3	4.056	0.425	1	411.1	43.75	K.SELKDFFQNFGNVVELR.I
G3BP_MOUSE	MK_SCX_31.8946.8946.2	2	4.44	0.555	1	410	65.625	K.SELKDFFQNFGNVVELR.I
G3BP_MOUSE	MK_SCX_42.3568.3568.3	3	3.329	0.404	1	844.9	34.090908	K.NSSYAHGGLDSNGKPADAVYGQK.E
G3BP_MOUSE	MK_SCX_54.4492.4492.3	3	4.767	0.613	1	1994.4	48.52941	R.HPDSHQLFIGNLPHEVDK.S
G3P_MOUSE	MK_SCX_17.10940.10940.2	2	4.888	0.517	1	1318	60.000004	K.WGEAGAEYVVESTGVFTTM*EK.A
G3P_MOUSE	MK_SCX_17.13415.13415.2	2	6.585	0.661	1	2894.3	72.5	K.WGEAGAEYVVESTGVFTTMEK.A

G3P_MOUSE	MK_SCX_17.4658.4658.1	1	2.969	0.298	1	303	40.625	K.IVSNASCTTNCLAPLAK.V
G3P_MOUSE	MK_SCX_19.4196.4196.2	2	4.039	0.447	1	727.7	56.25	K.IVSNASCTTNCLAPLAK.V
G3P_MOUSE	MK_SCX_20_1.3809.3809.2	2	4.383	0.574	1	1464.4	90	R.VVDLM*AYM*ASK.E
G3P_MOUSE	MK_SCX_20_1.4844.4844.2	2	3.644	0.311	1	1278.4	90	R.VVDLM*AYMASK.E
G3P_MOUSE	MK_SCX_20_1.6300.6300.2	2	4.302	0.575	1	1728.2	90	R.VVDLMAYMASK.E
G3P_MOUSE	MK_SCX_21.3882.3882.2	2	3.949	0.372	1	1044.3	75	R.GAAQNIIPASTGAAK.A
G3P_MOUSE	MK_SCX_2201.16666.16666.3	3	3.734	0.355	1	711	40.384613	K.LISWYDNEYGYSNR.V
G3P_MOUSE	MK_SCX_2201.16695.16695.2	2	4.784	0.515	1	1522.8	69.230774	K.LISWYDNEYGYSNR.V
G3P_MOUSE	MK_SCX_26.5491.5491.2	2	3.511	0.129	1	511.6	42.5	R.VIISAPSADAPMFVM*GVNHEK.Y
G3P_MOUSE	MK_SCX_26.5555.5555.2	2	3.661	0.382	1	925.3	47.727272	K.YDNSLKIVSNASCTTNCLAPLAK.V
G3P_MOUSE	MK_SCX_26.6168.6168.3	3	3.677	0.585	1	660.3	38.75	R.VIISAPSADAPMFVMGVNHEK.Y
G3P_MOUSE	MK_SCX_26.6208.6208.2	2	5.014	0.616	1	1270.4	65	R.VIISAPSADAPMFVMGVNHEK.Y
G3P_MOUSE	MK_SCX_29.3553.3553.2	2	5.47	0.589	1	834.2	67.64706	R.DGRGAAQNIIPASTGAAK.A
G3P_MOUSE	MK_SCX_29.3566.3566.3	3	3.222	0.241	1	670	39.705883	R.DGRGAAQNIIPASTGAAK.A
G3P_MOUSE	MK_SCX_33.6797.6797.2	2	3.95	0.582	1	503.3	36.53846	R.VIISAPSADAPMFVMGVNHEKYDNSLK.I
G3P_MOUSE	MK_SCX_36.6260.6260.2	2	2.834	0.215	1	365	36.842106	K.LVINGKPITIFQERDPTNIK.W
G3P_MOUSE	MK_SCX_41.7995.7995.2	2	6.481	0.67	1	946.2	54.347824	K.VIHDNFGIVEGLM*TTVHAITATQK.T
G3P_MOUSE	MK_SCX_41.7996.7996.3	3	7.692	0.682	1	1301.8	43.47826	K.VIHDNFGIVEGLM*TTVHAITATQK.T
G3P_MOUSE	MK_SCX_41.9047.9047.3	3	6.774	0.631	1	2181.1	45.652176	K.VIHDNFGIVEGLMTTVHAITATQK.T
G3P_MOUSE	MK_SCX_44.5013.5013.3	3	5.458	0.584	1	1293.6	41.666664	K.RVIISAPSADAPM*FVM*GVNHEK.Y
G3P_MOUSE	MK_SCX_44.5389.5389.3	3	5.963	0.246	1	2027.2	45.238094	K.RVIISAPSADAPMFVM*GVNHEK.Y
G3P_MOUSE	MK_SCX_44.5521.5521.2	2	5.445	0.24	1	497.3	57.14286	K.RVIISAPSADAPM*FVMGVNHEK.Y
G3P_MOUSE	MK_SCX_44.5523.5523.3	3	5.549	0.254	1	1742.8	46.42857	K.RVIISAPSADAPM*FVMGVNHEK.Y
G3P_MOUSE	MK_SCX_44.5916.5916.2	2	6.419	0.624	1	839.5	54.761906	K.RVIISAPSADAPMFVMGVNHEK.Y
G3P_MOUSE	MK_SCX_44.5941.5941.3	3	6.349	0.618	1	2558.2	50	K.RVIISAPSADAPMFVMGVNHEK.Y
G3P_MOUSE	MK_SCX_48.4537.4537.3	3	4.099	0.41	1	740.6	35	K.LWRDGRGAAQNIIPASTGAAK.A
G3P_MOUSE	MK_SCX_50.5829.5829.3	3	5.663	0.131	1	452.1	31.48148	K.RVIISAPSADAPMFVM*GVNHEKYDNSLK.I
G3P_MOUSE	MK_SCX_50.5855.5855.3	3	5.576	0.19	1	512.1	32.407406	K.RVIISAPSADAPM*FVMGVNHEKYDNSLK.I
G3P_MOUSE	MK_SCX_50.6239.6239.3	3	6.789	0.609	1	2150.4	38.88889	K.RVIISAPSADAPMFVMGVNHEKYDNSLK.I
G3P_MOUSE	MK_SCX_51.2743.2743.2	2	2.967	0.172	1	761.4	70	R.LEKPAKYDDIK.K
G3P_MOUSE	MK_SCX_56.2509.2509.3	3	3.455	0.251	1	1248.4	52.272724	R.LEKPAKYDDIKK.V
G3PT_MOUSE	MK_SCX_13.5365.5365.2	2	4.209	0.533	1	1802.7	84.61539	R.VPTPNVSVVDLTCR.L
G3PT_MOUSE	MK_SCX_19.7320.7320.2	2	4.746	0.538	1	558.5	50	K.LTGMAFRVPTPNVSVVDLTCR.L
G45IP_MOUSE	MK_SCX_16.7291.7291.2	2	5.496	0.674	1	830.1	55	R.ELEAEEQEWYPSLATMQESLR.L
G45IP_MOUSE	MK_SCX_32.4797.4797.2	2	4.502	0.537	1	1814.4	87.5	R.FQELLQDLDKQQR.K
G45IP_MOUSE	MK_SCX_32.6543.6543.3	3	3.179	0.343	1	806.8	35.526314	R.HGAISGVPPASLWPTPEQLR.E
G6PD1_MOUSE	MK_SCX_14.4217.4217.2	2	2.492	0.288	1	676.3	64.28571	R.NSYVAGQYDDAASYK.H
G6PD1_MOUSE	MK_SCX_2201.16491.16491.2	2	2.124	0.123	1	511.3	72.22222	K.EMVQNLMVLR.F
G6PE_MOUSE	MK_SCX_17.13455.13455.2	2	2.908	0.197	1	328.2	40.625	R.YRQSPLITAWPEELISK.L
G6PE_MOUSE	MK_SCX_43.3498.3498.3	3	3.432	0.279	1	433.3	47.5	K.WKEVQDQPGLR.L
G6PI_MOUSE	MK_SCX_19.7529.7529.2	2	4.191	0.497	1	1368.3	75	K.ILLANFLAQTEALM*K.G
G6PI_MOUSE	MK_SCX_19.9469.9469.2	2	4.889	0.527	1	2510.9	85.71429	K.ILLANFLAQTEALMK.G
G6PI_MOUSE	MK_SCX_26.4984.4984.3	3	3.633	0.43	1	468.6	34.72222	R.FAAYFQQGDMESNGKYITK.S
G6PI_MOUSE	MK_SCX_26.5010.5010.2	2	3.203	0.442	1	1736.4	61.11111	R.FAAYFQQGDMESNGKYITK.S
G6PI_MOUSE	MK_SCX_33.6615.6615.3	3	4.05	0.374	1	1124.8	33.653847	K.KIEPELEGSSAVTSHDSSTNGLISFIK.Q
G6PI_MOUSE	MK_SCX_33.8582.8582.3	3	4.277	0.226	1	841.6	46.666668	K.NLVNKEVM*QMLVELAK.S
G6PI_MOUSE	MK_SCX_33.9304.9304.3	3	4.622	0.469	1	1596.1	51.666664	K.NLVNKEVMQMLVELAK.S
G6PI_MOUSE	MK_SCX_33.9699.9699.2	2	2.69	0.32	1	405.2	56.666668	K.NLVNKEVMQMLVELAK.S
G6PI_MOUSE	MK_SCX_34.5676.5676.2	2	3.55	0.568	1	1329.6	73.07692	R.VWFVSNIDGTHIAK.T
G6PI_MOUSE	MK_SCX_38.3981.3981.2	2	3.1	0.325	1	752.4	80	K.LRELFEADPER.F
G6PI_MOUSE	MK_SCX_43.4450.4450.3	3	4.894	0.527	1	1284	47.058823	R.SNTPIKVDGKDVMPEVNR.V

G6PI_MOUSE	MK_SCX_43.7389.7389.3	3	4.667	0.324	1	1117	30.172413	R.VDHQTGPIVWGEPGTNGQHAFYQLIHQGTK.M
G6PI_MOUSE	MK_SCX_44.3734.3734.3	3	3.884	0.554	1	1156.8	45.588234	R.SNTPIKVDGKDVM*PEVNR.V
GABT_MOUSE	MK_SCX_17.8751.8751.2	2	4.652	0.547	1	1103.2	55.555557	K.TLLTGLLDLQAQYPQFISR.V
GABT_MOUSE	MK_SCX_18.5625.5625.2	2	3.673	0.485	1	1354.7	75	K.VDIEFDYDGPLM*K.T
GABT_MOUSE	MK_SCX_18.6352.6352.2	2	4.578	0.547	1	1875.5	87.5	K.VDIEFDYDGPLMK.T
GABT_MOUSE	MK_SCX_18.8945.8945.2	2	3.45	0.538	1	756.1	60.714287	K.IDIPSFDWPIAPFPR.L
GABT_MOUSE	MK_SCX_2201.7567.7567.3	3	6.079	0.578	1	880	36.11111	K.LVQQPQNASTFINRPALGILPPENFVDK.L
GABT_MOUSE	MK_SCX_23.6563.6563.3	3	3.395	0.434	1	416	28.125	R.TVAGIIVEPIQSEGGDNHASDDFFR.K
GABT_MOUSE	MK_SCX_25.6777.6777.3	3	4.633	0.463	1	1044.6	36.904762	R.M*LDLYSQISSVPIGYNHPALAK.L
GABT_MOUSE	MK_SCX_25.7072.7072.3	3	5.496	0.533	1	2002.9	44.04762	R.MLDLYSQISSVPIGYNHPALAK.L
GABT_MOUSE	MK_SCX_25.7083.7083.2	2	4.926	0.616	1	598	52.380955	R.MLDLYSQISSVPIGYNHPALAK.L
GABT_MOUSE	MK_SCX_26.4237.4237.3	3	3.112	0.159	1	630.1	30.952381	K.VDIEFDYDGPLMKTEVPGPRSK.E
GABT_MOUSE	MK_SCX_30.4973.4973.3	3	6.393	0.516	1	2088.4	51.47059	R.LKYPLEEFTTDNQQEEAR.C
GABT_MOUSE	MK_SCX_30.5015.5015.2	2	6.26	0.583	1	2813.6	79.411766	R.LKYPLEEFTTDNQQEEAR.C
GAK_MOUSE	MK_SCX_14.9578.9578.3	3	5.281	0.425	1	545.8	22.972973	K.SQNLDPFADLSDLSSSLQGLPAGLPAGGFVGAPAPTQK.S
GALC_MOUSE	MK_SCX_20_1.4125.4125.2	2	5.799	0.474	1	2437.8	86.666664	R.EFDGIGAVSGGGATSR.L
GALM_MOUSE	MK_SCX_16.7923.7923.2	2	4.568	0.649	1	528.3	47.826088	R.ILEVYTTQPGVQFYTGNFLDGTLK.G
GALM_MOUSE	MK_SCX_18.8128.8128.2	2	4.032	0.414	1	1981.1	76.666664	K.VLWTPQVLTNGVQFFR.V
GALM_MOUSE	MK_SCX_18.8491.8491.2	2	4.063	0.517	1	819.9	62.5	K.ASDVVLGFAELEGYLQK.Q
GALM_MOUSE	MK_SCX_20_1.4456.4456.2	2	2.802	0.196	1	912.8	64.28571	R.TVFGELPSGGGTVEK.F
GALT1_MOUSE	MK_SCX_14.4148.4148.2	2	3.461	0.298	1	649.1	70.83333	R.NVETNQCLDNMAR.K
GALT2_MOUSE	MK_SCX_15.6342.6342.2	2	3.881	0.244	1	622.4	47.368423	K.EIILVDDYSNDPEDGALLGK.I
GALT3_MOUSE	MK_SCX_26.9368.9368.3	3	3.553	0.453	1	685.2	41.17647	K.MLDFMLEAVNNIKDAMPK.M
GALT3_MOUSE	MK_SCX_26.9499.9499.2	2	2.217	0.229	1	414.7	44.11765	K.MLDFMLEAVNNIKDAMPK.M
GALT3_MOUSE	MK_SCX_33.4705.4705.2	2	3.653	0.192	1	1123.1	80.769226	K.MQIGAPIKENIDVR.E
GANAB_MOUSE	MK_SCX_21.6046.6046.2	2	2.872	0.206	1	494.8	50	K.VLLVLELQGLQKNMTR.I
GANAB_MOUSE	MK_SCX_28.7817.7817.3	3	3.27	0.31	1	454.9	38.333332	K.IILTAQPFRLDLLEDR.S
GANAB_MOUSE	MK_SCX_40.4088.4088.2	2	2.539	0.232	1	891.5	87.5	R.IRIDELEPR.R
GAS2_MOUSE	MK_SCX_11.8083.8083.2	2	3.048	0.233	1	374.2	46.875	K.FKESM*DANKPAKTLPLK.K
GAS2_MOUSE	MK_SCX_17.3883.3883.2	2	5.553	0.528	1	707	55.263157	K.EIEQEETLSAPSPSPSPSSK.S
GAS2_MOUSE	MK_SCX_17.3914.3914.3	3	3.993	0.462	1	1299.7	44.736843	K.EIEQEETLSAPSPSPSPSSK.S
GAS2_MOUSE	MK_SCX_21.8234.8234.2	2	4.636	0.498	1	1758.8	80.769226	R.VGGGWETFAGYLLK.H
GAS2_MOUSE	MK_SCX_26.4234.4234.2	2	6.313	0.622	1	1555	56.81818	K.LEKEIEQEETLSAPSPSPSPSSK.S
GAS2_MOUSE	MK_SCX_26.4264.4264.3	3	5.453	0.461	1	882.8	42.045452	K.LEKEIEQEETLSAPSPSPSPSSK.S
GAS2_MOUSE	MK_SCX_28.5256.5256.3	3	3.964	0.292	1	612.1	32.894737	K.SPTLKDM*NPDNYLVVSATYK.A
GAS2_MOUSE	MK_SCX_32.4987.4987.3	3	3.711	0.573	1	764.9	39.705883	R.SGPGLSDM*HQYSQWLASR.H
GAS2_MOUSE	MK_SCX_32.5044.5044.2	2	3.683	0.442	1	619.3	75	R.YGVEPPGLIKLEK.E
GAS2_MOUSE	MK_SCX_32.6115.6115.2	2	5.641	0.597	1	1760.3	67.64706	R.SGPGLSDMHQYSQWLASR.H
GAS2_MOUSE	MK_SCX_32.6209.6209.3	3	3.938	0.599	1	881.9	45.588234	R.SGPGLSDMHQYSQWLASR.H
GAS6_MOUSE	MK_SCX_28.4577.4577.3	3	3.947	0.165	1	480.1	29.545454	K.NPDFAKCVQNLPDQCTPNPCDKK.G
GATM_MOUSE	MK_SCX_13.7915.7915.2	2	3.293	0.492	1	875.7	65.625	K.FVTTEFEPCFDAADFIR.A
GATM_MOUSE	MK_SCX_19.4341.4341.2	2	4.113	0.519	1	1518.4	75	K.TPDFESTGLYSAM*PR.D
GATM_MOUSE	MK_SCX_19.5075.5075.2	2	4.257	0.535	1	1383	67.85714	K.TPDFESTGLYSAMPR.D
GATM_MOUSE	MK_SCX_20_1.3937.3937.2	2	3.546	0.421	1	1061.8	81.818184	R.VM*VDANEVPIQK.M
GATM_MOUSE	MK_SCX_20_1.4193.4193.2	2	3.175	0.376	1	696.3	68.181816	R.VMVDANEVPIQK.M
GATM_MOUSE	MK_SCX_2201.7073.7073.2	2	3.535	0.497	1	1092.7	75	R.SQVTNYLGIEWMR.R
GATM_MOUSE	MK_SCX_23.7971.7971.3	3	3.985	0.369	1	361.3	27.173912	K.AGWTIVTPPTPVIPDDHPLWMSSK.W
GATM_MOUSE	MK_SCX_32.5631.5631.3	3	4.217	0.509	1	2072.7	50	K.YKTPDFESTGLYSAMPR.D
GATM_MOUSE	MK_SCX_32.5700.5700.2	2	5.253	0.603	1	1682.4	84.375	K.YKTPDFESTGLYSAMPR.D
GATM_MOUSE	MK_SCX_34.5482.5482.3	3	6.481	0.638	1	1111.5	39	K.WTTAPKPTM*ADELYDQNYPIHSVEDR.H
GATM_MOUSE	MK_SCX_34.5978.5978.3	3	7.103	0.515	1	1847.6	40	K.WTTAPKPTMADELYDQNYPIHSVEDR.H

GATM_MOUSE	MK_SCX_37.6329.6329.3	3	3.048	0.286	1	480.7	41.666664	K.ANTYEKYWPFYQK.N
GATM_MOUSE	MK_SCX_41.4522.4522.2	2	3.112	0.537	1	833.4	77.77778	R.RPDPIDWSLK.Y
GATM_MOUSE	MK_SCX_42.7083.7083.3	3	5.263	0.565	1	662.9	27.205881	R.NSCAAEDKATHPLPKDCPVSSYNEWDPLEEVIVGR.A
GBLP_MOUSE	MK_SCX_23.8223.8223.2	2	4.199	0.507	1	649.6	43.47826	K.GHNGWVTQIATTPQFPDMILSASR.D
GBLP_MOUSE	MK_SCX_28.5490.5490.3	3	4.337	0.361	1	748.5	45	K.IIVDELKQEVISTSSK.A
GBLP_MOUSE	MK_SCX_28.5522.5522.2	2	4.631	0.571	1	2337.8	76.666664	K.IIVDELKQEVISTSSK.A
GBLP_MOUSE	MK_SCX_36.3492.3492.3	3	3.001	0.412	1	630.5	43.75	K.LTRDETNYGIPQR.A
GCDH_MOUSE	MK_SCX_16.7817.7817.2	2	2.259	0.242	1	316	37.5	R.DIVYEMGELGVLGPTIK.G
GCDH_MOUSE	MK_SCX_23.5572.5572.2	2	2.951	0.301	1	1027.3	60.000004	R.DILGGNGISDEYHVIR.H
GCDH_MOUSE	MK_SCX_25.6033.6033.2	2	2.298	0.171	1	324.5	32.5	K.DPLILEEQLTADEKLIRDTFR.N
GCDH_MOUSE	MK_SCX_38.12728.12728.3	3	6.146	0.625	1	1619.9	40.909092	K.SSRPVFDWKDPLILEEQLTADEK.L
GCDH_MOUSE	MK_SCX_52.6312.6312.3	3	6.854	0.592	1	1967.1	45.652176	R.HAMNLEAVNTYEGTHDIHALILGR.A
GCNT1_MOUSE	MK_SCX_20_1.3533.3533.2	2	3.521	0.357	1	1170.7	85	K.ILQGDPEEIQK.V
GCNT1_MOUSE	MK_SCX_24.6518.6518.3	3	3.107	0.324	1	317.6	27.173912	R.IPEVPGSFPSSNKYDLSDMNAIAR.F
GCP60_MOUSE	MK_SCX_15.8182.8182.2	2	4.55	0.676	1	1013.7	50	K.QVLLGPYNPDTSPEVGFFDVLGNDR.R
GCP60_MOUSE	MK_SCX_18.3454.3454.2	2	3.038	0.29	1	1235.1	62.5	K.VNTAGASDTLSVNGQAK.T
GCR_MOUSE	MK_SCX_17.5770.5770.2	2	3.962	0.486	1	695.4	47.5	K.IQDTGDTILSSPSSVALPQVK.T
GCR_MOUSE	MK_SCX_20_1.3120.3120.2	2	4.317	0.494	1	1008.8	68.75	K.VSASSPSVAAASQADSK.Q
GDIA_MOUSE	MK_SCX_18.8119.8119.2	2	6.444	0.663	1	1893.8	80.55556	K.SPYLYPLYGLGELPQGFAR.L
GDIA_MOUSE	MK_SCX_29.8568.8568.3	3	5.078	0.335	1	1562.3	40.476192	R.YGKSPYLYPLYGLGELPQGFAR.L
GDIA_MOUSE	MK_SCX_41.4899.4899.3	3	3.737	0.224	1	1341.5	60.000004	R.GRDWNVDLIPK.F
GDIA_MOUSE	MK_SCX_41.4916.4916.2	2	2.722	0.27	1	454.4	75	R.GRDWNVDLIPK.F
GDIA_MOUSE	MK_SCX_41.9015.9015.3	3	7.291	0.574	1	4129.4	59.210526	R.KFDLGQDVIDFTGHALALYR.T
GDIB_MOUSE	MK_SCX_17.4865.4865.2	2	4.477	0.519	1	2061.1	86.36364	K.DLGTDSQIFISR.A
GDIB_MOUSE	MK_SCX_21.7522.7522.2	2	3.83	0.5	1	1201.3	90	K.FVSISDLFVPK.D
GDIB_MOUSE	MK_SCX_2201.5160.5160.2	2	3.12	0.224	1	1283	93.75	K.MLLFTEVTR.Y
GDIB_MOUSE	MK_SCX_2201.7818.7818.3	3	3.711	0.44	1	1257.1	35.869564	R.LSAIYGGTYMLNKPIEEIIVQNGK.V
GDIB_MOUSE	MK_SCX_25.6937.6937.3	3	5.994	0.57	1	1503.8	42.708336	K.VLHMDQNPYYGGESASITPLEDLYK.R
GDIB_MOUSE	MK_SCX_25.8809.8809.2	2	5.573	0.651	1	2588.9	75	K.FDLGQDVIDFTGHSLALYR.T
GDIB_MOUSE	MK_SCX_25.8855.8855.3	3	5.957	0.559	1	1494.6	50	K.FDLGQDVIDFTGHSLALYR.T
GDIB_MOUSE	MK_SCX_33.4510.4510.2	2	4.397	0.536	1	1311.2	79.16667	R.MTGSEFDFEEMKR.K
GDIB_MOUSE	MK_SCX_35.10378.10378.3	3	7.299	0.616	1	2517.7	43	K.VLHMDQNPYYGGESASITPLEDLYKR.F
GDIB_MOUSE	MK_SCX_39.3777.3777.2	2	2.664	0.382	1	526.4	66.66667	R.FKLPGQPPASM*GR.G
GDIB_MOUSE	MK_SCX_39.4613.4613.2	2	3.106	0.395	1	478	66.66667	R.FKLPGQPPASMGR.G
GDIB_MOUSE	MK_SCX_41.8274.8274.3	3	6.485	0.563	1	3877.2	59.210526	K.KFDLGQDVIDFTGHSLALYR.T
GDIR_MOUSE	MK_SCX_18.4731.4731.2	2	3.137	0.272	1	324.6	42.857143	R.AEEYEFLTPM*EEAPK.G
GDIR_MOUSE	MK_SCX_18.4894.4894.2	2	4.906	0.549	1	1308.1	76.666664	R.VAVSADPNVPNVIVTR.L
GDIR_MOUSE	MK_SCX_18.5620.5620.2	2	5.221	0.57	1	1162.5	71.42857	R.AEEYEFLTPMEEAPK.G
GDIR_MOUSE	MK_SCX_25.6759.6759.3	3	4.559	0.571	1	1083.1	43.421055	R.AEEYEFLTPMEEAPKGMLAR.G
GDIR_MOUSE	MK_SCX_27.4779.4779.3	3	4.077	0.468	1	630	45	K.SIQEIQELDKDDESLR.K
GDIR_MOUSE	MK_SCX_29.4816.4816.2	2	5.944	0.576	1	1899.1	76.666664	K.SIQEIQELDKDDESLR.K
GDIR_MOUSE	MK_SCX_32.3604.3604.2	2	4.959	0.483	1	1510.6	84.61539	K.IDKTDYM*VGSYGPR.A
GDIR_MOUSE	MK_SCX_32.3609.3609.3	3	4.273	0.339	1	2460.1	61.538464	K.IDKTDYM*VGSYGPR.A
GDIR_MOUSE	MK_SCX_32.3817.3817.3	3	3.742	0.39	1	1774.2	59.615387	K.IDKTDYMVGSYGPR.A
GDIR_MOUSE	MK_SCX_32.3819.3819.2	2	5.119	0.497	1	2810.6	88.46153	K.IDKTDYMVGSYGPR.A
GDIR_MOUSE	MK_SCX_32.3927.3927.1	1	3.082	0.437	1	475.4	65.38461	K.IDKTDYMVGSYGPR.A
GDIR_MOUSE	MK_SCX_41.4266.4266.3	3	4.195	0.481	1	803.1	40.625	K.SIQEIQELDKDDESLRK.Y
GDIR_MOUSE	MK_SCX_41.4301.4301.2	2	5.703	0.549	1	1354.6	65.625	K.SIQEIQELDKDDESLRK.Y
GDIR_MOUSE	MK_SCX_49.4443.4443.3	3	3.256	0.528	1	1413.9	43.75	K.GVKIDKTDYMVGSYGPR.A
GDIS_MOUSE	MK_SCX_16.4148.4148.2	2	2.967	0.385	1	769.8	60.000004	K.DAQPQLEEADDDLDSK.L
GDIS_MOUSE	MK_SCX_16.6739.6739.2	2	4.705	0.657	1	971.3	57.5	K.TLLGDVPVVADPTVPNVTVTR.L

GDIS_MOUSE	MK_SCX_2201.7217.7217.3	3	5.515	0.545	1	1375.1	38	K.ATFM*VGSYGPRPEEYEFLTPVEEAPK.G
GDIS_MOUSE	MK_SCX_2201.7543.7543.3	3	5.284	0.618	1	954.5	31	K.ATFMVGSYGPRPEEYEFLTPVEEAPK.G
GDIS_MOUSE	MK_SCX_43.3667.3667.3	3	4.045	0.377	1	780.4	40.625	K.SLKELQEM*DKDDESLTK.Y
GDIS_MOUSE	MK_SCX_43.4390.4390.3	3	5.138	0.614	1	1895.6	53.125	K.SLKELQEMDKDDESLTK.Y
GDIS_MOUSE	MK_SCX_43.4415.4415.2	2	5.922	0.47	1	2251.5	71.875	K.SLKELQEMDKDDESLTK.Y
GELS_MOUSE	MK_SCX_15.7023.7023.2	2	4.562	0.579	1	1094.4	47.916664	K.VSNGAGSMSVSLVADENPFAQGALR.S
GELS_MOUSE	MK_SCX_19.5287.5287.2	2	2.873	0.334	1	341.4	46.875	R.QTQVSVLPEGGETPLFK.Q
GELS_MOUSE	MK_SCX_28.6411.6411.2	2	6.527	0.614	1	2831.5	66.66667	R.SQHVQVEEGSEPDAFWEALGGK.T
GELS_MOUSE	MK_SCX_42.6258.6258.3	3	6.753	0.571	1	3307.6	50	K.NWRDPDQTDGPGLGYLSSHIANVER.V
GGA2_MOUSE	MK_SCX_14.8768.8768.2	2	5.449	0.555	1	1376.3	44.230766	R.LASDTTDDDDALAEILQANDLLTQGVR.L
GGT1_MOUSE	MK_SCX_16.6107.6107.2	2	5.889	0.653	1	2382	76.47059	K.DIQEAGGIMTVEDLNNYR.A
GGT1_MOUSE	MK_SCX_17.4891.4891.2	2	4.808	0.522	1	1251.9	76.666664	K.DSEEGGLSVAVPGEIR.G
GGT1_MOUSE	MK_SCX_17.7775.7775.2	2	5.318	0.602	1	1037	52.272724	R.VSGILFNDEM*DDFSSPNFINQFR.V
GGT1_MOUSE	MK_SCX_17.8383.8383.2	2	5.895	0.608	1	1101	54.545456	R.VSGILFNDEMDDFSSPNFINQFR.V
GGT1_MOUSE	MK_SCX_17.8402.8402.3	3	5.437	0.364	1	2118.1	39.772728	R.VSGILFNDEMDDFSSPNFINQFR.V
GGT1_MOUSE	MK_SCX_18.4798.4798.1	1	3.091	0.463	1	650.7	70	K.DIDQVVTAGLK.I
GGT1_MOUSE	MK_SCX_20_1.15408.15408.2	2	5.215	0.518	1	2430.5	84.61539	K.LADTLQILAQEGAK.A
GGT1_MOUSE	MK_SCX_20_1.15418.15418.3	3	4.71	0.425	1	1598.4	55.76923	K.LADTLQILAQEGAK.A
GGT1_MOUSE	MK_SCX_20_1.3857.3857.2	2	4.418	0.459	1	1934.6	90.909096	K.VLQEGETVTMPK.L
GGT1_MOUSE	MK_SCX_21.3269.3269.2	2	4.062	0.552	1	2095.1	86.36364	K.VLQEGETVTM*PK.L
GGT1_MOUSE	MK_SCX_2201.3855.3855.2	2	2.585	0.271	1	760	92.85714	K.GLAIALDK.K
GGT1_MOUSE	MK_SCX_23.4554.4554.2	2	3.418	0.467	1	942.6	93.75	K.FVDVSQVIR.N
GGT1_MOUSE	MK_SCX_23.4690.4690.2	2	3.448	0.377	1	815.2	94.44444	R.LFQPSIQLAR.H
GGT1_MOUSE	MK_SCX_25.16053.16053.2	2	2.469	0.341	1	345.7	61.11111	K.TPALCEVFCR.Q
GGT1_MOUSE	MK_SCX_27.6627.6627.2	2	3.08	0.281	1	367	50	R.DVIEKTPALCEVFCR.Q
GGT1_MOUSE	MK_SCX_51.5653.5653.2	2	2.447	0.124	1	548	50	K.RDVIEKTPALCEVFCR.Q
GGT5_MOUSE	MK_SCX_17.8585.8585.2	2	4.402	0.467	1	1255.7	66.66667	R.SQDPFPWPALANTLETVAK.E
GIMA3_MOUSE	MK_SCX_13.5381.5381.2	2	3.556	0.258	1	534.1	44.444447	R.ASGEEQQGQLAELMALVRR.L
GIMA4_MOUSE	MK_SCX_15.6810.6810.2	2	6.047	0.661	1	1498.4	64.28571	K.ELVVVDTPGIFDTEVPDADTQR.E
GIMA4_MOUSE	MK_SCX_26.7576.7576.2	2	4.04	0.498	1	1015.3	50	R.YVALTSPGPHALLLVVPLGR.Y
GIMA4_MOUSE	MK_SCX_35.3876.3876.3	3	4.692	0.363	1	1011.6	48.333332	R.SSHELGNQDQGIPQLR.I
GLGB_MOUSE	MK_SCX_26.7651.7651.3	3	3.863	0.371	1	635.5	40.625	R.LLEIDPYLKPFAADFQR.R
GLO2_MOUSE	MK_SCX_16.7971.7971.2	2	4.618	0.456	1	456.2	47.727272	K.YAIGEPTVPSTLAEEFTYNPFM*R.V
GLO2_MOUSE	MK_SCX_16.7983.7983.3	3	3.205	0.435	1	695.4	29.545454	K.YAIGEPTVPSTLAEEFTYNPFM*R.V
GLO2_MOUSE	MK_SCX_16.8347.8347.3	3	4.336	0.504	1	1397	38.636364	K.YAIGEPTVPSTLAEEFTYNPFMR.V
GLO2_MOUSE	MK_SCX_16.8355.8355.2	2	4.688	0.494	1	783	54.545456	K.YAIGEPTVPSTLAEEFTYNPFMR.V
GLO2_MOUSE	MK_SCX_25.8618.8618.2	2	5.371	0.519	1	602.1	52.083332	K.EKYAIGEPTVPSTLAEEFTYNPFMR.V
GLO2_MOUSE	MK_SCX_25.8685.8685.3	3	5.395	0.646	1	1176.8	31.25	K.EKYAIGEPTVPSTLAEEFTYNPFMR.V
GLO2_MOUSE	MK_SCX_32.3249.3249.3	3	4.63	0.458	1	1591	50	K.TVQQHAGETDPVTTMR.A
GLO2_MOUSE	MK_SCX_40.2425.2425.2	2	2.299	0.283	1	563.2	63.636364	R.HVEPGNAAIQEK.L
GLO2_MOUSE	MK_SCX_47.2676.2676.3	3	3.521	0.25	1	748.2	41.17647	K.EKTVQQHAGETDPVTTM*R.A
GLO2_MOUSE	MK_SCX_47.3179.3179.3	3	3.971	0.514	1	1157	45.588234	K.EKTVQQHAGETDPVTTMR.A
GLPK_MOUSE	MK_SCX_19.4868.4868.2	2	3.675	0.548	1	1337.2	64.70589	K.AVLGPLVGAVDQGTSSTR.F
GLPK_MOUSE	MK_SCX_21.4490.4490.2	2	3.69	0.506	1	743.1	87.5	R.FEPQINAEESEIR.Y
GLPK_MOUSE	MK_SCX_21.5629.5629.2	2	4.229	0.311	1	1615.4	86.36364	K.LGQLNIDISNIK.A
GLPK_MOUSE	MK_SCX_33.4744.4744.3	3	4.829	0.544	1	1332.4	51.38889	K.KAVLGPLVGAVDQGTSSTR.F
GLPK_MOUSE	MK_SCX_33.4748.4748.2	2	6.014	0.615	1	2229.8	80.55556	K.KAVLGPLVGAVDQGTSSTR.F
GLRX1_MOUSE	MK_SCX_16.4999.4999.2	2	3.966	0.367	1	1050.9	57.5	K.DCIGGCSDLISM*QQTGELM*TR.L
GLRX1_MOUSE	MK_SCX_25.7017.7017.2	2	4.857	0.521	1	648.7	42	R.VFIGKDCIGGCSDLISMQQTGELMTR.L
GLRX1_MOUSE	MK_SCX_25.7130.7130.3	3	6.245	0.436	1	2061	35	R.VFIGKDCIGGCSDLISMQQTGELMTR.L
GLRX1_MOUSE	MK_SCX_37.5876.5876.2	2	2.903	0.237	1	1326.4	81.818184	R.KTQEILSQLPFK.Q

GLRX5_MOUSE	MK_SCX_17.5625.5625.2	2	5.426	0.609	1	2774.7	88.46153	R.DYAAYNVLDDPELR.Q
GLRX5_MOUSE	MK_SCX_48.5613.5613.2	2	4.592	0.563	1	706.5	63.88889	R.LHGVRDYAAYNVLDDPELR.Q
GLRX5_MOUSE	MK_SCX_48.5648.5648.3	3	4.647	0.52	1	1304.2	44.444447	R.LHGVRDYAAYNVLDDPELR.Q
GLU2B_MOUSE	MK_SCX_14.3669.3669.2	2	3.893	0.571	1	1639.7	87.5	K.YEQGTGCWQGPNR.S
GLU2B_MOUSE	MK_SCX_17.4742.4742.2	2	3.024	0.53	1	427.5	62.5	R.SEVPPTDIPVPEETEPK.E
GLU2B_MOUSE	MK_SCX_17.6391.6391.2	2	7.002	0.484	1	3793.6	80.55556	K.MPPYDEETQAIIDAAQEAR.S
GLU2B_MOUSE	MK_SCX_21.4332.4332.2	2	3.891	0.423	1	1547.6	88.88889	K.SLEDQVETLR.A
GLU2B_MOUSE	MK_SCX_2201.3000.3000.2	2	3.431	0.462	1	899.4	88.88889	K.LWEEQQAAAK.A
GLU2B_MOUSE	MK_SCX_2201.4414.4414.2	2	3.325	0.392	1	1447.2	80	K.ESLQQLAEVTR.E
GLU2B_MOUSE	MK_SCX_36.4063.4063.3	3	3.947	0.481	1	1207.8	52.083332	K.EKESLQQLAEVTR.E
GLU2B_MOUSE	MK_SCX_38.3955.3955.3	3	3.418	0.179	1	865.6	52.499996	K.KSLEDQVETLR.A
GLU2B_MOUSE	MK_SCX_38.3966.3966.2	2	3.032	0.344	1	880.4	85	K.KSLEDQVETLR.A
GLU2B_MOUSE	MK_SCX_41.3653.3653.3	3	3.443	0.153	1	352.4	41.666664	R.SLKEMEESIR.S
GLU2B_MOUSE	MK_SCX_41.3671.3671.2	2	2.929	0.303	1	1221.3	94.44444	R.SLKEMEESIR.S
GLU2B_MOUSE	MK_SCX_53.3706.3706.2	2	5.023	0.411	1	2800.6	92.30769	R.KEKESLQQLAEVTR.E
GLU2B_MOUSE	MK_SCX_53.3725.3725.3	3	3.609	0.419	1	787.5	42.307693	R.KEKESLQQLAEVTR.E
GLU2B_MOUSE	MK_SCX_53.5844.5844.3	3	5.548	0.623	1	2303.3	43	K.HGGSPTSLGTWGSWAGPDHDKFSAMK.Y
GLYC_MOUSE	MK_SCX_19.5818.5818.3	3	3.041	0.243	1	502.7	31.25	-.M*ADRDATLWASHEKMLSQPLK.D
GLYC_MOUSE	MK_SCX_27.6279.6279.3	3	4.481	0.424	1	2471.9	48.52941	K.MLSQPLKDSDAEVYSIIK.K
GLYC_MOUSE	MK_SCX_27.6319.6319.2	2	2.886	0.39	1	622.2	50	K.MLSQPLKDSDAEVYSIIK.K
GLYG_MOUSE	MK_SCX_29.5039.5039.3	3	3.892	0.389	1	508.3	36.11111	R.WEQGQADYMGADSFDNIKR.K
GLYG_MOUSE	MK_SCX_41.3707.3707.3	3	4.033	0.526	1	1410.5	50	R.TKPWNYTYNPQTK.S
GMFB_MOUSE	MK_SCX_21.5320.5320.2	2	2.537	0.246	1	576.2	75	R.NTEDLTEEWLR.E
GMFB_MOUSE	MK_SCX_2201.3083.3083.2	2	2.272	0.345	1	524.2	68.75	K.LVQTAELTK.V
GMPR1_MOUSE	MK_SCX_50.5167.5167.3	3	3.648	0.494	1	813.9	52.499996	K.LDFKDVLLRPK.R
GMPR1_MOUSE	MK_SCX_50.5179.5179.2	2	2.812	0.321	1	864.9	80	K.LDFKDVLLRPK.R
GNA1_MOUSE	MK_SCX_29.5824.5824.3	3	4.238	0.356	1	1221.1	48.214287	-.MKPDETPMFDPSLLK.E
GNA11_MOUSE	MK_SCX_16.5600.5600.2	2	2.038	0.152	1	355.5	30.952381	R.DAQAAREFILKMFVDLNPDSDK.I
GNA11_MOUSE	MK_SCX_35.4788.4788.2	2	2.499	0.151	1	407.8	60.000004	K.ESKRINAEIEK.Q
GNA13_MOUSE	MK_SCX_17.6491.6491.2	2	3.531	0.472	1	461.1	59.375	K.LGVPDYIPSQQDILLAR.R
GNA13_MOUSE	MK_SCX_2201.6936.6936.2	2	2.786	0.218	1	638.6	88.88889	R.VFLQYLPAIR.A
GNAI2_MOUSE	MK_SCX_19.4563.4563.2	2	3.562	0.466	1	421.1	57.14286	R.IAQSDYIPTQQDVLR.T
GNAI3_MOUSE	MK_SCX_20_1.5036.5036.2	2	4.622	0.321	1	718.8	75	R.ISQTNYIPTQQDVLR.T
GNAQ_MOUSE	MK_SCX_18.4923.4923.2	2	4.364	0.401	1	979.1	75	R.VADPSYLPTQQDVLR.V
GNAS_MOUSE	MK_SCX_18.3568.3568.2	2	4.267	0.487	1	980.7	66.66667	R.YTTPEDATPEPGEDPR.V
GNAS_MOUSE	MK_SCX_37.6649.6649.2	2	2.822	0.397	1	618.2	68.181816	K.SKIEDYFPEFAR.Y
GNPI_MOUSE	MK_SCX_21.11983.11983.3	3	3.193	0.377	1	547.7	25	K.IQAAGGIELFVGGIGPDGHIAFNEPGSSLVSR.T
GNPI_MOUSE	MK_SCX_21.5352.5352.2	2	4.054	0.541	1	1968.8	86.36364	K.TLAMDTILANAR.F
GNPI_MOUSE	MK_SCX_2201.3473.3473.2	2	2.024	0.142	1	334.9	55.555557	R.IIQFNPGPDK.Y
GNPI_MOUSE	MK_SCX_36.5288.5288.2	2	2.898	0.452	1	434.5	76.92308	R.VKTLAMDTILANAR.F
GOGA3_MOUSE	MK_SCX_14.5462.5462.2	2	2.109	0.151	1	415.2	58.333332	R.EAKTMVEEDLQRR.L
GOGA3_MOUSE	MK_SCX_18.3737.3737.2	2	5.738	0.585	1	2739.8	81.25	R.LPDQQDTAQDASVEVNR.G
GOGA4_MOUSE	MK_SCX_11.7386.7386.2	2	2.437	0.179	1	330.2	40.625	R.QM*LETLELKEDEIAQLR.S
GOGA4_MOUSE	MK_SCX_17.3510.3510.2	2	2.298	0.337	1	444.4	40.625	K.TLQEKELTCQALEQRVK.E
GOGA4_MOUSE	MK_SCX_23.4704.4704.2	2	2.431	0.176	1	506.6	54.545456	R.SDLESKLTGAER.D
GOGA4_MOUSE	MK_SCX_26.3277.3277.3	3	5.153	0.507	1	1756.3	40.217392	K.SPDGVSKDESSPSQSGDTQTFAQK.L
GOGA5_MOUSE	MK_SCX_18.3848.3848.2	2	5.415	0.586	1	1537.8	65	K.NTDYPELQQQNTDSNYQTGQK.A
GOGA5_MOUSE	MK_SCX_25.4148.4148.3	3	6.592	0.556	1	2456.8	39.655174	R.LEQQVHSASSGPNSGSAINMSGVDSGEGTR.L
GOGA5_MOUSE	MK_SCX_43.4877.4877.3	3	7.011	0.52	1	2614.4	47.61905	R.IMQDHKEGSSLQNQALQTLQER.L
GORS2_MOUSE	MK_SCX_18.4146.4146.2	2	4.724	0.632	1	1766.4	73.333336	K.ADASSLTVDVTSPASK.V
GORS2_MOUSE	MK_SCX_2201.5248.5248.3	3	4.929	0.618	1	740.8	33.695652	K.ADASSLTVDVTSPASKVPTTVEDR.V

GOSR1_MOUSE	MK_SCX_18.3833.3833.2	2	3.084	0.418	1	643.5	63.333332	R.YSSDTTPLLNGSSQDR.M
GOSR1_MOUSE	MK_SCX_40.6882.6882.3	3	3.558	0.415	1	477.1	32.954548	K.MAEYTHSAGVPSLNAALMHTLQR.H
GOSR2_MOUSE	MK_SCX_36.4096.4096.3	3	3.011	0.457	1	707.4	55	K.YDVQHLQTALR.N
GOSR2_MOUSE	MK_SCX_36.4108.4108.2	2	3.499	0.457	1	1101.4	80	K.YDVQHLQTALR.N
GP137_MOUSE	MK_SCX_16.9451.9451.2	2	4.876	0.657	1	812.3	59.523808	K.QGLSGVPILSEEELSLLDEFYK.L
GP137_MOUSE	MK_SCX_21.3539.3539.2	2	3.743	0.481	1	1628.8	90	R.LNQDQLDAVSK.Y
GP137_MOUSE	MK_SCX_2201.10302.10302.3	3	5.949	0.51	1	2320.2	38	R.TDLKQGLSGVPILSEEELSLLDEFYK.L
GP137_MOUSE	MK_SCX_2201.4482.4482.2	2	3.473	0.495	1	1000	75	R.SFMALSQDIQK.T
GP137_MOUSE	MK_SCX_26.8001.8001.3	3	4.222	0.484	1	722.2	42.647057	K.TVLELQYVLDKLGDDDVR.T
GP137_MOUSE	MK_SCX_28.5145.5145.3	3	3.952	0.347	1	1462.3	31.730768	R.GGYDGYRPSFSNTPNSGYSQSQFTAPR.D
GP137_MOUSE	MK_SCX_43.7724.7724.3	3	4.335	0.542	1	830.2	41.666664	R.LNEQYEHASIHLWDLLEGK.E
GPC5C_MOUSE	MK_SCX_21.4779.4779.2	2	4.038	0.473	1	2223.1	79.16667	K.GPSEGAYDVILPR.A
GPC5C_MOUSE	MK_SCX_38.3437.3437.3	3	3.916	0.16	1	628.4	36.842106	R.AEDM*YMVQSHQVATPPKDGK.I
GPC5C_MOUSE	MK_SCX_38.3754.3754.3	3	5.168	0.632	1	1113.9	47.368423	R.AEDMYMVQSHQVATPPKDGK.I
GPDA_MOUSE	MK_SCX_29.4601.4601.3	3	3.085	0.224	1	659.1	38.235294	K.FCETTIGCKDPAQGQLLK.D
GPDA_MOUSE	MK_SCX_31.7639.7639.3	3	4.339	0.541	1	935.9	53.846157	K.GLVDKFPLFTAVYK.V
GPDA_MOUSE	MK_SCX_31.7715.7715.2	2	4.268	0.544	1	1169.1	80.769226	K.GLVDKFPLFTAVYK.V
GPM6A_MOUSE	MK_SCX_57.2599.2599.3	3	4.664	0.513	1	1403.5	55.76923	K.SKEEQELHDIHSTR.S
GPX1_MOUSE	MK_SCX_16.5096.5096.1	1	2.064	0.266	1	446.2	68.75	R.NDIAWNFEK.F
GPX1_MOUSE	MK_SCX_17.4403.4403.2	2	4.104	0.612	1	757.1	64.70589	R.NALPTPSDDPTALM*TDPK.Y
GPX1_MOUSE	MK_SCX_17.5131.5131.2	2	3.836	0.586	1	1071	70.588234	R.NALPTPSDDPTALMTDPK.Y
GPX1_MOUSE	MK_SCX_24.15484.15484.3	3	6.416	0.604	1	2402.6	38.392857	R.LSAAAQSTVYAFSARPLTGGEPVSLGSLR.G
GPX1_MOUSE	MK_SCX_33.6679.6679.2	2	4.866	0.409	1	1080.2	65.625	K.YVRPGGGFEPNFTLFEK.C
GPX1_MOUSE	MK_SCX_34.6543.6543.3	3	4.845	0.399	1	1336.9	51.5625	K.YVRPGGGFEPNFTLFEK.C
GPX1_MOUSE	MK_SCX_38.4322.4322.2	2	2.717	0.176	1	527.3	63.636364	K.FLVGPDGVPVRR.Y
GPX1_MOUSE	MK_SCX_44.5251.5251.2	2	2.733	0.418	1	341.8	81.25	K.AHPLFTFLR.N
GPX3_MOUSE	MK_SCX_18.4643.4643.2	2	2.326	0.236	1	426.9	46.153847	K.QEPGENSEILPSLK.Y
GPX3_MOUSE	MK_SCX_21.5163.5163.2	2	2.819	0.196	1	757.4	68.181816	K.FLVGPDGIPVM*R.W
GPX3_MOUSE	MK_SCX_21.6258.6258.2	2	3.49	0.455	1	1126.1	81.818184	K.FLVGPDGIPVMR.W
GPX3_MOUSE	MK_SCX_23.5480.5480.2	2	3.131	0.343	1	1220.5	92.85714	K.MDILSYMR.R
GPX3_MOUSE	MK_SCX_25.4975.4975.2	2	2.565	0.153	1	400.6	91.66667	R.LFWEPMK.I
GPX3_MOUSE	MK_SCX_32.6132.6132.3	3	3.115	0.305	1	494.8	35.714287	R.TTVSNVKM*DILSYMR.R
GPX3_MOUSE	MK_SCX_34.6887.6887.3	3	4.414	0.384	1	1499.8	53.125	K.YVRPGGGFVPNFQLFEK.G
GPX3_MOUSE	MK_SCX_34.6903.6903.2	2	4.825	0.422	1	908.1	59.375	K.YVRPGGGFVPNFQLFEK.G
GPX3_MOUSE	MK_SCX_49.6530.6530.3	3	4.107	0.406	1	1238.1	33.653847	K.YVRPGGGFVPNFQLFEKGDVNGEKEQK.F
GPX3_MOUSE	MK_SCX_51.830.830.2	2	2.535	0.247	1	456.8	85.71429	R.RQAALSAR.G
GPX3_MOUSE	MK_SCX_52.5525.5525.2	2	3.186	0.392	1	751.8	77.27273	R.LFWEPMKIHDIR.W
GPX3_MOUSE	MK_SCX_7.5364.5364.2	2	3.01	0.42	1	867.7	64.28571	K.NSCPPTAELLGSPGR.L
GPX41_MOUSE	MK_SCX_20_1.3692.3692.2	2	3.431	0.441	1	747	81.818184	R.YGPM*EEPQVIEK.D
GPX41_MOUSE	MK_SCX_20_1.4194.4194.2	2	3.395	0.391	1	694.2	77.27273	R.YGPMEEPQVIEK.D
GPX41_MOUSE	MK_SCX_30.4531.4531.3	3	3.872	0.457	1	724.5	43.055553	R.QEPGSNQEIKEFAAGYNVK.F
GPX41_MOUSE	MK_SCX_30.4534.4534.2	2	4.599	0.493	1	666.8	61.11111	R.QEPGSNQEIKEFAAGYNVK.F
GRAM3_MOUSE	MK_SCX_18.3390.3390.2	2	4.12	0.406	1	722.7	50	K.SPTAQSPTSSVEAESPDQK.R
GRAM3_MOUSE	MK_SCX_29.3235.3235.3	3	4.649	0.458	1	844.9	38.157894	K.SPTAQSPTSSVEAESPDQKR.S
GRAM3_MOUSE	MK_SCX_41.3144.3144.3	3	4.733	0.379	1	622.3	36.363636	R.VSKSPTAQSPTSSVEAESPDQKR.S
GRAP1_MOUSE	MK_SCX_20_1.15607.15607.2	2	3.148	0.209	1	520.5	54.166668	K.EADLKAQLARTQK.L
GRAP1_MOUSE	MK_SCX_20_1.15676.15676.3	3	3.422	0.279	1	732.2	43.75	K.EADLKAQLARTQK.L
GRB2_MOUSE	MK_SCX_52.5659.5659.2	2	3.379	0.31	1	1342.1	69.230774	K.NYIEMKPHPWFFGK.I
GRHPR_MOUSE	MK_SCX_13.6624.6624.3	3	3.725	0.399	1	460.5	34.523808	R.AALAQAADCEVEQWNSDDPIPR.K
GRHPR_MOUSE	MK_SCX_17.8971.8971.3	3	6.176	0.648	1	1809.4	26.875002	R.GDVVNQEDLYQALASGQIAAAGLDVTTPEPLPPSHPLLTLK.N
GRHPR_MOUSE	MK_SCX_21.7339.7339.2	2	3.672	0.485	1	1024.8	66.66667	R.NTM*SLLAANNLLAGLR.G

GRHPR_MOUSE	MK_SCX_21.8154.8154.2	2	2.848	0.305	1	526.2	50	R.NTMSLLAANNLLAGLR.G
GRP75_MOUSE	MK_SCX_16.6840.6840.3	3	3.556	0.161	1	2167	48.61111	K.STNGDTFLGGEDFDQALLR.H
GRP75_MOUSE	MK_SCX_16.6940.6940.2	2	4.445	0.486	1	794	55.555557	K.STNGDTFLGGEDFDQALLR.H
GRP75_MOUSE	MK_SCX_19.4696.4696.2	2	4.188	0.525	1	1516.5	76.92308	K.SDIGEVILVGGM*TR.M
GRP75_MOUSE	MK_SCX_19.5008.5008.2	2	5.115	0.353	1	641	62.5	K.SQVFSTAADGQTQVEIK.V
GRP75_MOUSE	MK_SCX_19.5060.5060.2	2	5.487	0.593	1	1761.5	86.666664	R.VINEPTAAALAYGLDK.S
GRP75_MOUSE	MK_SCX_19.5754.5754.2	2	3.057	0.359	1	1073.5	73.07692	K.SDIGEVILVGGMTR.M
GRP75_MOUSE	MK_SCX_19.6973.6973.2	2	3.716	0.494	1	1264.6	89.28571	K.LLGQFTLIGIPPAPR.G
GRP75_MOUSE	MK_SCX_20_1.3640.3640.2	2	2.608	0.323	1	476.7	57.692307	R.QAVTNPNNTFYATK.R
GRP75_MOUSE	MK_SCX_20_1.4391.4391.2	2	4.311	0.623	1	1779.9	80.769226	R.TTPSVVAFTADGER.L
GRP75_MOUSE	MK_SCX_20_1.5823.5823.2	2	3.699	0.501	1	669.6	75	K.NAVITVPAYFNDSQR.Q
GRP75_MOUSE	MK_SCX_20_1.7148.7148.3	3	3.582	0.473	1	698.8	46.153847	K.LYSPSQIGAFVLM*K.M
GRP75_MOUSE	MK_SCX_20_1.7343.7343.2	2	4.784	0.475	1	1012.5	76.92308	K.LYSPSQIGAFVLM*K.M
GRP75_MOUSE	MK_SCX_20_1.7407.7407.2	2	4.099	0.497	1	1453.9	86.36364	R.AQFEGIVTDLIK.R
GRP75_MOUSE	MK_SCX_20_1.8034.8034.2	2	4.027	0.422	1	1350.2	76.92308	K.LYSPSQIGAFVLMK.M
GRP75_MOUSE	MK_SCX_20_1.8185.8185.3	3	3.415	0.291	1	1131.6	46.153847	K.LYSPSQIGAFVLMK.M
GRP75_MOUSE	MK_SCX_2201.1522.1522.2	2	2.595	0.275	1	831	87.5	K.AMQDAEVSK.S
GRP75_MOUSE	MK_SCX_2201.4294.4294.2	2	3.748	0.545	1	1542.1	85	K.VQQTVQDLFGR.A
GRP75_MOUSE	MK_SCX_24.3047.3047.2	2	2.194	0.143	1	827.2	87.5	K.VLENAEGAR.T
GRP75_MOUSE	MK_SCX_24.6398.6398.2	2	5.511	0.541	1	2307.2	61.363636	K.AMQDAEVSKSDIGEVILVGGMTR.M
GRP75_MOUSE	MK_SCX_24.6484.6484.3	3	3.585	0.352	1	1169.3	36.363636	K.AMQDAEVSKSDIGEVILVGGMTR.M
GRP75_MOUSE	MK_SCX_25.4900.4900.2	2	4.899	0.578	1	537.9	45.238094	R.EQQIVIQSSGGLSKDDIENM*VK.N
GRP75_MOUSE	MK_SCX_25.5401.5401.2	2	4.333	0.549	1	856.9	50	R.VINEPTAAALAYGLDKSEDK.V
GRP75_MOUSE	MK_SCX_25.5903.5903.3	3	3.364	0.532	1	1373.5	42.857143	R.EQQIVIQSSGGLSKDDIENMVK.N
GRP75_MOUSE	MK_SCX_25.5986.5986.2	2	5.382	0.652	1	1449.3	57.14286	R.EQQIVIQSSGGLSKDDIENMVK.N
GRP75_MOUSE	MK_SCX_25.7920.7920.2	2	3.983	0.613	1	1026.2	54.761906	R.EMAGDNKLLGQFTLIGIPPAPR.G
GRP75_MOUSE	MK_SCX_25.8096.8096.3	3	3.75	0.532	1	376	28.57143	R.EMAGDNKLLGQFTLIGIPPAPR.G
GRP75_MOUSE	MK_SCX_27.4109.4109.3	3	3.873	0.5	1	985.6	42.857143	R.ETGVDLTKDNMALQR.V
GRP75_MOUSE	MK_SCX_27.4801.4801.3	3	3.238	0.464	1	448.6	40.625	R.VEAVNM*AEGIIHDTETK.M
GRP75_MOUSE	MK_SCX_27.4901.4901.2	2	4.83	0.548	1	1257.8	71.875	R.VEAVNM*AEGIIHDTETK.M
GRP75_MOUSE	MK_SCX_27.6261.6261.2	2	5.418	0.585	1	2582	78.125	R.VEAVNMAEGIIHDTETK.M
GRP75_MOUSE	MK_SCX_27.6451.6451.3	3	3.982	0.502	1	934.7	48.4375	R.VEAVNMAEGIIHDTETK.M
GRP75_MOUSE	MK_SCX_29.3679.3679.2	2	3.346	0.4	1	477.1	60.714287	R.ETGVDLTKDNM*ALQR.V
GRP75_MOUSE	MK_SCX_29.4064.4064.2	2	3.945	0.485	1	614.8	60.714287	R.ETGVDLTKDNMALQR.V
GRP75_MOUSE	MK_SCX_31.3060.3060.2	2	2.7	0.32	1	702.7	66.66667	R.ASNGDAWVEAHGK.L
GRP75_MOUSE	MK_SCX_32.4265.4265.2	2	4.161	0.248	1	1804.1	75	K.VQQTVQDLFGRAPSK.A
GRP75_MOUSE	MK_SCX_32.6440.6440.2	2	3.604	0.346	1	1502.4	75	R.AQFEGIVTDLIKR.T
GRP75_MOUSE	MK_SCX_34.4104.4104.3	3	3.858	0.361	1	1771.2	51.666664	R.QATKDAGQISGLNVLR.V
GRP75_MOUSE	MK_SCX_34.4109.4109.2	2	4.059	0.55	1	919.2	73.333336	R.QATKDAGQISGLNVLR.V
GRP75_MOUSE	MK_SCX_43.6011.6011.3	3	5.178	0.573	1	2922.2	52.77778	K.ERVEAVNMAEGIIHDTETK.M
GRP75_MOUSE	MK_SCX_44.3630.3630.3	3	5.726	0.49	1	2022.5	46.05263	K.DKGTGREQQIVIQSSGGLSK.D
GRP75_MOUSE	MK_SCX_50.3360.3360.3	3	3.514	0.399	1	575.2	38.333332	K.RETGVDLTKDNM*ALQR.V
GRP75_MOUSE	MK_SCX_50.3680.3680.3	3	4.71	0.437	1	1684.3	51.666664	K.RETGVDLTKDNMALQR.V
GRP75_MOUSE	MK_SCX_50.3688.3688.2	2	5.868	0.546	1	1393.6	80	K.RETGVDLTKDNMALQR.V
GRP75_MOUSE	MK_SCX_51.2728.2728.3	3	3.681	0.36	1	1395.3	48.076923	K.M*KETAENYLGHTAK.N
GRP75_MOUSE	MK_SCX_52.2955.2955.3	3	3.759	0.398	1	585.1	38.46154	K.MKETAENYLGHTAK.N
GRP78_MOUSE	MK_SCX_17.13466.13466.2	2	5.541	0.557	1	2517.6	73.52941	R.IEIESFFEGEDFSETLTR.A
GRP78_MOUSE	MK_SCX_18.3319.3319.2	2	4.45	0.549	1	973.1	61.764706	K.LYGSGGPPPTGEEDTSEK.D
GRP78_MOUSE	MK_SCX_18.5249.5249.2	2	4.482	0.378	1	1542.5	81.818184	K.ELEEIVQPIISK.L
GRP78_MOUSE	MK_SCX_19.5149.5149.2	2	5.841	0.57	1	1639.4	83.33333	R.IINEPTAAAIAYGLDK.R
GRP78_MOUSE	MK_SCX_19.6205.6205.2	2	4.633	0.667	1	1382.8	84.61539	K.TFAPEEISAMVLTK.M

GRP78_MOUSE	MK_SCX_20_1.4320.4320.2	2	4.415	0.488	1	937.4	67.85714	K.NQLTSNPENTVFDAK.R
GRP78_MOUSE	MK_SCX_20_1.4795.4795.2	2	5.105	0.62	1	2104.5	75	K.SQIFSTASDNQPTVTIK.V
GRP78_MOUSE	MK_SCX_20_1.4850.4850.3	3	3.271	0.308	1	775.4	40.384613	R.ITPSYVAFTPEGER.L
GRP78_MOUSE	MK_SCX_20_1.5002.5002.2	2	4.271	0.475	1	806.8	69.230774	R.ITPSYVAFTPEGER.L
GRP78_MOUSE	MK_SCX_21.4144.4144.2	2	2.115	0.239	1	441.4	63.636364	R.TWNDPSVQQDIK.F
GRP78_MOUSE	MK_SCX_21.6868.6868.2	2	3.91	0.301	1	1478.5	88.88889	K.FEELNMDLFR.S
GRP78_MOUSE	MK_SCX_2201.2893.2893.2	2	3.766	0.45	1	1601.8	90	R.VEIIANDQGNR.I
GRP78_MOUSE	MK_SCX_2201.4686.4686.2	2	3.734	0.394	1	1539	90	R.NELESYAYSLK.N
GRP78_MOUSE	MK_SCX_24.7004.7004.3	3	3.541	0.495	1	641.5	44.11765	K.DNHLLGTFDLTGIPPAPR.G
GRP78_MOUSE	MK_SCX_24.7126.7126.2	2	5.666	0.676	1	1355.2	73.52941	K.DNHLLGTFDLTGIPPAPR.G
GRP78_MOUSE	MK_SCX_26.5123.5123.3	3	3.887	0.355	1	518.7	28.57143	R.LIGDAAKNQLTSNPENTVFDAK.R
GRP78_MOUSE	MK_SCX_27.5951.5951.2	2	5.692	0.47	1	2370	83.33333	K.IEWLESHQDADIEDFK.A
GRP78_MOUSE	MK_SCX_28.5825.5825.3	3	4.008	0.49	1	620.8	46.666668	K.IEWLESHQDADIEDFK.A
GRP78_MOUSE	MK_SCX_29.5641.5641.2	2	5.027	0.593	1	1620.8	78.125	R.IINEPTAAAIAYGLDKR.E
GRP78_MOUSE	MK_SCX_31.5117.5117.2	2	5.089	0.573	1	1113.3	75	K.VTHAVVTVPAYFNDAQR.Q
GRP78_MOUSE	MK_SCX_32.4975.4975.3	3	4.692	0.484	1	1138.1	46.875	K.VTHAVVTVPAYFNDAQR.Q
GRP78_MOUSE	MK_SCX_32.5108.5108.2	2	5.158	0.465	1	2617.2	87.5	K.KELEEIVQPIISK.L
GRP78_MOUSE	MK_SCX_33.4019.4019.2	2	4.416	0.532	1	1139.5	73.333336	K.NQLTSNPENTVFDAKR.L
GRP78_MOUSE	MK_SCX_33.4253.4253.3	3	3.313	0.476	1	985.1	46.666668	R.QATKDAGTIAGLNVMR.I
GRP78_MOUSE	MK_SCX_33.4704.4704.2	2	5.32	0.505	1	3607.6	89.28571	K.KSDIDEIVLVGGSTR.I
GRP78_MOUSE	MK_SCX_34.4120.4120.3	3	5.419	0.493	1	1010.7	45.588234	K.KSQIFSTASDNQPTVTIK.V
GRP78_MOUSE	MK_SCX_34.4143.4143.2	2	6.11	0.593	1	3580.6	79.411766	K.KSQIFSTASDNQPTVTIK.V
GRP78_MOUSE	MK_SCX_35.3993.3993.2	2	4.003	0.5	1	1599.8	85.71429	K.TKPYIQVDIGGGQTK.T
GRP78_MOUSE	MK_SCX_35.4116.4116.3	3	4.115	0.304	1	1536.8	53.571426	K.TKPYIQVDIGGGQTK.T
GRP78_MOUSE	MK_SCX_36.5304.5304.3	3	4.318	0.434	1	1969.1	59.090908	R.AKFEELNM*DLFR.S
GRP78_MOUSE	MK_SCX_36.6598.6598.2	2	3.922	0.511	1	1391.5	81.818184	R.AKFEELNMDLFR.S
GRP78_MOUSE	MK_SCX_37.5582.5582.2	2	3.596	0.458	1	791.2	72.72727	R.AKFEELNM*DLFR.S
GRP78_MOUSE	MK_SCX_37.6876.6876.3	3	4.882	0.451	1	1699	59.090908	R.AKFEELNMDLFR.S
GRP78_MOUSE	MK_SCX_39.16694.16694.3	3	4.075	0.456	1	989.2	45.588234	K.IEWLESHQDADIEDFKAK.K
GRP78_MOUSE	MK_SCX_40.3211.3211.3	3	3.422	0.408	1	1385.1	60.000004	K.MKETAEAYLGK.K
GRP78_MOUSE	MK_SCX_43.5805.5805.2	2	5.151	0.552	1	1250.7	66.66667	R.NELESYAYSLKNQIGDKEK.L
GRP78_MOUSE	MK_SCX_44.4967.4967.2	2	3.244	0.392	1	1329.6	70	K.IQQLVKEFFNGKEPSR.G
GRP78_MOUSE	MK_SCX_46.6748.6748.3	3	5.436	0.611	1	750.5	30.555555	K.VYEGERPLTKDNHLLGTFDLTGIPPAPR.G
GRP78_MOUSE	MK_SCX_47.3278.3278.3	3	4.643	0.47	1	594.7	50	R.MVNDAEKFAEEDKK.L
GRP78_MOUSE	MK_SCX_47.3291.3291.2	2	4.038	0.343	1	1438.7	80.769226	R.MVNDAEKFAEEDKK.L
GRP78_MOUSE	MK_SCX_47.5469.5469.3	3	3.629	0.349	1	901.5	46.666668	K.IQQLVKEFFNGKEPSR.G
GRP78_MOUSE	MK_SCX_51.4435.4435.3	3	4.727	0.434	1	1572.4	48.52941	K.KVTHAVVTVPAYFNDAQR.Q
GRP78_MOUSE	MK_SCX_51.4459.4459.2	2	5.506	0.558	1	1173.2	70.588234	K.KVTHAVVTVPAYFNDAQR.Q
GRP78_MOUSE	MK_SCX_51.4570.4570.2	2	5.591	0.432	1	2157.6	84.61539	K.KKELEEIVQPIISK.L
GRP78_MOUSE	MK_SCX_51.4586.4586.3	3	4.445	0.379	1	1069.5	46.153847	K.KKELEEIVQPIISK.L
GRP78_MOUSE	MK_SCX_53.2956.2956.3	3	4.461	0.459	1	1671.2	63.636364	K.MKETAEAYLGKK.V
GRP78_MOUSE	MK_SCX_53.3539.3539.3	3	4.506	0.467	1	840	40	K.KTKPYIQVDIGGGQTK.T
GRPE1_MOUSE	MK_SCX_19.10536.10536.2	2	2.155	0.157	1	370	53.571426	K.SLYEGLVMTEVQIQK.V
GRPE1_MOUSE	MK_SCX_35.3497.3497.2	2	2.837	0.165	1	484.3	60.000004	K.LEEQLRETMEK.Y
GRPE1_MOUSE	MK_SCX_36.4211.4211.2	2	3.005	0.441	1	615.8	70.83333	R.TLRPALVGVVK.D
GRPE1_MOUSE	MK_SCX_36.4254.4254.3	3	3.987	0.51	1	881.3	45.833336	R.TLRPALVGVVK.D
GRPE1_MOUSE	MK_SCX_41.5050.5050.3	3	3.653	0.545	1	903.8	54.6875	K.FDPYEHEALFHTPVEGK.E
GRPE1_MOUSE	MK_SCX_43.6224.6224.3	3	4.972	0.512	1	641.9	31.730768	K.FDPYEHEALFHTPVEGKEPGTVALVSK.V
GRPE1_MOUSE	MK_SCX_47.3329.3329.3	3	5.182	0.54	1	844	42.647057	K.ATQSVPKEEISNNNPHLK.S
GRPE1_MOUSE	MK_SCX_52.3638.3638.2	2	4.613	0.464	1	1478.7	75	K.AKLEEQLRETMEK.Y
GRSF1_MOUSE	MK_SCX_19.4999.4999.2	2	2.498	0.236	1	340.1	68.181816	K.LGDEVDDVYLIR.A

GRSF1_MOUSE	MK_SCX_19.9043.9043.3	3	3.14	0.343	1	302.3	26.666668	K.TTYLEDLPPLPEYELSPSKLGDEVDDVYLIR.A
GRSF1_MOUSE	MK_SCX_2201.2895.2895.2	2	2.32	0.178	1	408.2	61.11111	R.ITMEYSSSGK.A
GRSF1_MOUSE	MK_SCX_31.4559.4559.2	2	3.695	0.443	1	677	70	K.SLQVKPSPVLSDGVVR.L
GRSF1_MOUSE	MK_SCX_34.3560.3560.3	3	4.997	0.404	1	964.6	46.666668	R.RGDALIEM*ESEQDVQK.A
GRSF1_MOUSE	MK_SCX_42.7450.7450.3	3	3.745	0.351	1	1204.1	40.27778	K.LPEAVDFGTLPSLHFVHMR.G
GSHB_MOUSE	MK_SCX_18.4127.4127.2	2	3.854	0.125	1	831.7	63.333332	R.DGYM*PSQYNSQNWEAR.L
GSHB_MOUSE	MK_SCX_18.4611.4611.2	2	5.226	0.599	1	1395.1	80	R.DGYMPSQYNSQNWEAR.L
GSHR_MOUSE	MK_SCX_31.5412.5412.2	2	4.534	0.511	1	1175.3	73.333336	K.IYSTAFTPMYHAVTTR.K
GSHR_MOUSE	MK_SCX_45.4258.4258.3	3	3.348	0.399	1	534.6	28.75	R.AAVVESHKLGGTCVNVGCVPK.K
GSTA1_MOUSE	MK_SCX_15.7389.7389.2	2	5.201	0.473	1	970.8	67.64706	K.DGNLMFDQVPMVEIDGMK.L
GSTA3_MOUSE	MK_SCX_17.6748.6748.2	2	5.144	0.349	1	1164	66.66667	R.SDGSLMFQQVPMVEIDGMK.L
GSTK1_MOUSE	MK_SCX_18.5033.5033.2	2	4.113	0.254	1	1169.3	64.28571	R.FLTTVSMEQPEM*LEK.V
GSTK1_MOUSE	MK_SCX_27.11051.11051.3	3	3.06	0.278	1	466.2	29.166666	K.QFFQVPLNIPKDFFGETVK.K
GSTK1_MOUSE	MK_SCX_29.5210.5210.2	2	4.099	0.529	1	794.7	66.66667	K.YGAFGLPTTVAHVDGK.T
GSTK1_MOUSE	MK_SCX_29.5214.5214.3	3	3.358	0.514	1	1320.9	48.333332	K.YGAFGLPTTVAHVDGK.T
GSTK1_MOUSE	MK_SCX_35.4064.4064.2	2	3.856	0.422	1	1075.2	69.230774	K.AGMSTAQAQHFLEK.I
GSTM1_MOUSE	MK_SCX_19.4225.4225.2	2	5.053	0.494	1	2020.4	91.66667	R.MLLEYTDSSYDEK.R
GSTM1_MOUSE	MK_SCX_20_1.2659.2659.3	3	3.34	0.253	1	1458.3	56.81818	R.KHHLDGETEEER.I
GSTM1_MOUSE	MK_SCX_20_1.3384.3384.2	2	4.287	0.434	1	1322.8	77.27273	R.ADIVENQVM*DTR.M
GSTM1_MOUSE	MK_SCX_21.3556.3556.2	2	2.461	0.372	1	756.4	75	R.YTM*GDAPDFDR.S
GSTM1_MOUSE	MK_SCX_2201.4071.4071.2	2	3.036	0.516	1	823.3	80	R.YTMGDAPDFDR.S
GSTM1_MOUSE	MK_SCX_24.5520.5520.2	2	3.243	0.388	1	1196.4	88.88889	-.PM*ILGYWNVR.G
GSTM1_MOUSE	MK_SCX_24.6364.6364.2	2	3.804	0.52	1	1110	88.88889	-.PMILGYWNVR.G
GSTM1_MOUSE	MK_SCX_26.8047.8047.2	2	5.085	0.525	1	1116.1	68.75	K.LGLDFPNLPYLIDGSHK.I
GSTM1_MOUSE	MK_SCX_26.8058.8058.3	3	4.406	0.501	1	1027.6	43.75	K.LGLDFPNLPYLIDGSHK.I
GSTM1_MOUSE	MK_SCX_29.5769.5769.2	2	4.608	0.624	1	1070.1	67.64706	R.YTMGDAPDFDRSQWLNEK.F
GSTM1_MOUSE	MK_SCX_30.3688.3688.2	2	3.713	0.419	1	536.3	69.230774	R.M*LLEYTDSSYDEKR.Y
GSTM1_MOUSE	MK_SCX_30.3740.3740.3	3	3.374	0.364	1	623.2	40.384613	R.M*LLEYTDSSYDEKR.Y
GSTM1_MOUSE	MK_SCX_30.3930.3930.3	3	3.435	0.392	1	616.1	48.076923	R.MLLEYTDSSYDEKR.Y
GSTM1_MOUSE	MK_SCX_30.3954.3954.2	2	4.572	0.543	1	1967	84.61539	R.MLLEYTDSSYDEKR.Y
GSTM1_MOUSE	MK_SCX_35.14405.14405.2	2	2.452	0.173	1	326.2	57.692307	R.IRADIVENQVMDTR.M
GSTM1_MOUSE	MK_SCX_35.8168.8168.3	3	3.959	0.261	1	1445.4	51.923077	R.IRADIVENQVMDTR.M
GSTM1_MOUSE	MK_SCX_41.4815.4815.3	3	3.444	0.437	1	1069.9	58.333332	K.MKLYSEFLGK.R
GSTM1_MOUSE	MK_SCX_41.8396.8396.2	2	5.509	0.575	1	2106.6	72.22222	K.FKLGLDFPNLPYLIDGSHK.I
GSTM1_MOUSE	MK_SCX_41.8565.8565.3	3	5.789	0.588	1	2744.4	52.77778	K.FKLGLDFPNLPYLIDGSHK.I
GSTM1_MOUSE	MK_SCX_42.3762.3762.2	2	3.66	0.493	1	1280.5	86.36364	K.RYTMGDAPDFDR.S
GSTM1_MOUSE	MK_SCX_43.3358.3358.3	3	3.134	0.403	1	607.9	38.636364	K.RYTM*GDAPDFDR.S
GSTM1_MOUSE	MK_SCX_48.9006.9006.3	3	4.138	0.558	1	719.3	28.703705	K.FKLGLDFPNLPYLIDGSHKITQSNAILR.Y
GSTM1_MOUSE	MK_SCX_50.5250.5250.3	3	3.499	0.449	1	881	40.27778	K.RYTMGDAPDFDRSQWLNEK.F
GSTM2_MOUSE	MK_SCX_23.4996.4996.2	2	2.669	0.32	1	1039.4	83.33333	-.PM*TLGYWDIR.G
GSTM2_MOUSE	MK_SCX_23.5854.5854.2	2	3.488	0.437	1	1271.3	88.88889	-.PMTLGYWDIR.G
GSTO1_MOUSE	MK_SCX_17.9768.9768.2	2	4.701	0.602	1	740.6	52.499996	K.SFLGGDSPSMVDYLTWPWFQR.L
GSTO1_MOUSE	MK_SCX_23.6243.6243.2	2	3.909	0.446	1	803.1	94.44444	K.VPPLIASFVR.S
GSTO1_MOUSE	MK_SCX_33.3651.3651.3	3	3.364	0.434	1	1320.6	45.588234	R.SLGKGSAPPGPVPEGQIR.V
GSTP1_MOUSE	MK_SCX_18.3776.3776.2	2	4.407	0.31	1	959.8	60.000004	R.EAAQM*DM*VNDGVEDLR.G
GSTP1_MOUSE	MK_SCX_18.4935.4935.2	2	5.237	0.476	1	2963.7	86.666664	R.EAAQMDMVNDGVEDLR.G
GSTP1_MOUSE	MK_SCX_18.6078.6078.2	2	6.471	0.59	1	1951	80	K.FEDGDLTLYQSNAILR.H
GSTP1_MOUSE	MK_SCX_21.6178.6178.2	2	3.781	0.451	1	1041.4	75	-.PPYTIVYFPVR.G
GSTP1_MOUSE	MK_SCX_27.3712.3712.3	3	3.274	0.374	1	496.1	35.294117	R.EAAQM*DM*VNDGVEDLRGK.Y
GSTP1_MOUSE	MK_SCX_27.5024.5024.2	2	4.828	0.533	1	777.6	55.88235	R.EAAQMDMVNDGVEDLRGK.Y
GSTP1_MOUSE	MK_SCX_27.5044.5044.3	3	3.536	0.364	1	892.4	47.058823	R.EAAQMDMVNDGVEDLRGK.Y

GSTP1_MOUSE	MK_SCX_35.6202.6202.2	2	3.054	0.259	1	447.8	66.66667	-.PPYTIVYFPVRGR.C
GSTP1_MOUSE	MK_SCX_35.9063.9063.3	3	3.337	0.505	1	456.7	43.75	-.PPYTIVYFPVRGR.C
GSTP1_MOUSE	MK_SCX_47.5864.5864.2	2	5.504	0.475	1	1219.1	65.789474	K.ALPGHLKPFETLLSQNQGGK.A
GSTP1_MOUSE	MK_SCX_47.5872.5872.3	3	5.178	0.454	1	1566.7	44.736843	K.ALPGHLKPFETLLSQNQGGK.A
GSTT2_MOUSE	MK_SCX_19.5109.5109.2	2	4.035	0.476	1	1007.4	69.230774	K.VLGPLIGVQVPQEK.V
GSTT2_MOUSE	MK_SCX_47.5350.5350.3	3	3.353	0.495	1	571.4	39.0625	K.KMLPVPPPEVHASMQLR.I
GTF2I_MOUSE	MK_SCX_15.9040.9040.2	2	4.129	0.604	1	1091.4	47.916664	K.VPYPVFESNPEFLYVEGLPEGIPFR.S
GTF2I_MOUSE	MK_SCX_16.5952.5952.2	2	2.869	0.454	1	501.3	46.875	R.DQSAVVVQGLPEGVAFK.H
GTF2I_MOUSE	MK_SCX_23.4472.4472.2	2	3.318	0.427	1	1274.5	80	K.APSYLEISSMR.R
GTF2I_MOUSE	MK_SCX_26.5194.5194.2	2	2.194	0.268	1	485.3	75	R.SPTWFGIPR.L
GTF2I_MOUSE	MK_SCX_51.3404.3404.3	3	4.887	0.54	1	1616.3	56.666668	K.RPELLTHSTTEVTQPR.T
GTR2_MOUSE	MK_SCX_23.5533.5533.2	2	3.388	0.421	1	1801.6	88.88889	K.SFEEIAAEFR.K
GUC2B_MOUSE	MK_SCX_51.3334.3334.3	3	3.796	0.399	1	949.1	40	K.KLNELEEKEMSNPQPR.R
H10_MOUSE	MK_SCX_20_1.5433.5433.2	2	3.808	0.383	1	1176.7	79.16667	K.YSDM*IVAAIQAEK.N
H10_MOUSE	MK_SCX_20_1.6037.6037.2	2	4.708	0.549	1	2389.4	91.66667	K.YSDMIVAAIQAEK.N
H10_MOUSE	MK_SCX_2201.1901.1901.2	2	3.366	0.352	1	1397	88.88889	K.VGENADSQIK.L
H10_MOUSE	MK_SCX_28.2810.2810.2	2	2.875	0.453	1	706.1	81.25	K.GVGASGSFR.L
H10_MOUSE	MK_SCX_32.4474.4474.3	3	3.187	0.358	1	530.5	35.714287	K.YSDM*IVAAIQAEKNR.A
H10_MOUSE	MK_SCX_32.5231.5231.3	3	4.039	0.591	1	733.9	44.642857	K.YSDMIVAAIQAEKNR.A
H10_MOUSE	MK_SCX_32.5250.5250.2	2	4.319	0.54	1	1296.7	75	K.YSDMIVAAIQAEKNR.A
H10_MOUSE	MK_SCX_36.3414.3414.2	2	2.048	0.147	1	308.9	65	R.LVTTGVLKQTK.G
H10_MOUSE	MK_SCX_44.4889.4889.3	3	3.383	0.35	1	823.4	40.27778	K.STDHPKYSDM*IVAAIQAEK.N
H10_MOUSE	MK_SCX_44.4957.4957.2	2	4.082	0.467	1	634.8	55.555557	K.STDHPKYSDM*IVAAIQAEK.N
H10_MOUSE	MK_SCX_44.5816.5816.2	2	5.781	0.625	1	2558.2	75	K.STDHPKYSDMIVAAIQAEK.N
H10_MOUSE	MK_SCX_44.5911.5911.3	3	4.148	0.495	1	1397.3	41.666664	K.STDHPKYSDMIVAAIQAEK.N
H10_MOUSE	MK_SCX_53.6004.6004.3	3	4.338	0.579	1	716.5	37.5	K.STDHPKYSDMIVAAIQAEKNR.A
H10_MOUSE	MK_SCX_54.2762.2762.3	3	3.416	0.4	1	700.1	48.076923	K.SHYKVGENADSQIK.L
H10_MOUSE	MK_SCX_54.4502.4502.3	3	5.146	0.624	1	1198.4	40.789474	K.KSTDHPKYSDM*IVAAIQAEK.N
H10_MOUSE	MK_SCX_54.5499.5499.2	2	5.577	0.518	1	2281.4	68.42105	K.KSTDHPKYSDMIVAAIQAEK.N
H10_MOUSE	MK_SCX_54.5509.5509.3	3	5.956	0.557	1	1882.4	46.05263	K.KSTDHPKYSDMIVAAIQAEK.N
H11_MOUSE	MK_SCX_40.3377.3377.2	2	2.467	0.251	1	367.8	72.22222	R.SGVSLAALKK.S
H11_MOUSE	MK_SCX_50.5999.5999.3	3	4.402	0.549	1	1407.9	43.421055	R.KKPAGPSVSELIVQAVSSSK.E
H12_MOUSE	MK_SCX_12.3273.3273.2	2	2.449	0.483	1	424.9	46.666668	-.SEAAPAAPAAAPPAEK.A
H12_MOUSE	MK_SCX_21.3610.3610.2	2	3.311	0.569	1	1249.9	85	K.ALAAAGYDVEK.N
H12_MOUSE	MK_SCX_21.4314.4314.2	2	3.034	0.429	1	627.8	72.72727	K.ASGPPVSELITK.A
H12_MOUSE	MK_SCX_34.3250.3250.2	2	4.582	0.662	1	2094.7	82.14286	K.ALAAAGYDVEKNNSR.I
H12_MOUSE	MK_SCX_37.3674.3674.2	2	4.387	0.583	1	1175	79.16667	R.KASGPPVSELITK.A
H12_MOUSE	MK_SCX_37.3678.3678.3	3	4.785	0.474	1	1023.3	52.083332	R.KASGPPVSELITK.A
H12_MOUSE	MK_SCX_39.3200.3200.3	3	3.991	0.259	1	1345.1	54.545456	K.KALAAAGYDVEK.N
H12_MOUSE	MK_SCX_42.2653.2653.2	2	2.705	0.26	1	602.8	68.181816	K.GTGASGSFKLNK.K
H12_MOUSE	MK_SCX_52.2804.2804.3	3	4.537	0.5	1	1459.9	51.666664	K.KALAAAGYDVEKNNSR.I
H12_MOUSE	MK_SCX_55.2111.2111.2	2	3.173	0.395	1	594.5	62.5	K.GTGASGSFKLNKK.A
H13_MOUSE	MK_SCX_18.3557.3557.2	2	4.135	0.556	1	787.8	70	-.SETAPAAPAAPAPVEK.T
H14_MOUSE	MK_SCX_12.3365.3365.2	2	3.561	0.446	1	1068.1	73.333336	-.SETAPAAPAAPAPAEK.T
H14_MOUSE	MK_SCX_21.4304.4304.2	2	2.981	0.455	1	754	72.72727	K.TSGPPVSELITK.A
H14_MOUSE	MK_SCX_37.3818.3818.2	2	3.684	0.466	1	1081.9	75	R.KTSGPPVSELITK.A
H14_MOUSE	MK_SCX_55.3390.3390.3	3	3.563	0.546	1	1161.3	48.076923	K.RKTSGPPVSELITK.A
H15_MOUSE	MK_SCX_18.3307.3307.2	2	3.981	0.445	1	1138.7	70	-.SETAPAETAAPAPVEK.S
H15_MOUSE	MK_SCX_37.3817.3817.2	2	4.212	0.564	1	1438.7	79.16667	R.KATGPPVSELITK.A
H15_MOUSE	MK_SCX_37.3865.3865.3	3	4.872	0.555	1	1432.6	58.333332	R.KATGPPVSELITK.A
H2A1F_MOUSE	MK_SCX_17.7436.7436.3	3	4.577	0.512	1	1300.4	44.444447	R.VTIAQGGVLPNIQAVLLPK.K

H2A1F_MOUSE	MK_SCX_17.8063.8063.2	2	5.629	0.528	1	1227.4	69.44444	R.VTIAQGGVLPNIQAVLLPK.K
H2A1F_MOUSE	MK_SCX_25.4400.4400.2	2	3.423	0.3	1	880.8	87.5	R.AGLQFPVGR.V
H2A1F_MOUSE	MK_SCX_26.7306.7306.2	2	5.017	0.601	1	793.1	55.263157	R.VTIAQGGVLPNIQAVLLPKK.T
H2A1F_MOUSE	MK_SCX_35.4663.4663.3	3	3.436	0.278	1	1196.1	57.5	R.NDEELNKLLGR.V
H2A1F_MOUSE	MK_SCX_35.4687.4687.2	2	3.767	0.367	1	757.6	80	R.NDEELNKLLGR.V
H2A1F_MOUSE	MK_SCX_43.10618.10618.3	3	3.02	0.38	1	325	23.863636	K.LLGRVTIAQGGVLPNIQAVLLPK.K
H2A1F_MOUSE	MK_SCX_52.3551.3551.3	3	3.437	0.434	1	1238.9	51.923077	R.HLQLAIRNDEELNK.L
H2A1F_MOUSE	MK_SCX_52.3555.3555.2	2	4.803	0.446	1	1423.4	76.92308	R.HLQLAIRNDEELNK.L
H2A1F_MOUSE	MK_SCX_55.6548.6548.3	3	5.923	0.517	1	1171.3	48.52941	R.HLQLAIRNDEELNKLLGR.V
H2A1F_MOUSE	MK_SCX_55.6701.6701.2	2	5.466	0.542	1	1057.4	61.764706	R.HLQLAIRNDEELNKLLGR.V
H2A2A_MOUSE	MK_SCX_2201.4598.4598.2	2	2.215	0.135	1	627.6	75	R.NDEELNKLLGK.V
H2A2A_MOUSE	MK_SCX_25.8549.8549.2	2	4.854	0.652	1	1891.3	59.090908	K.LLGKVTIAQGGVLPNIQAVLLPK.K
H2A2A_MOUSE	MK_SCX_25.8599.8599.3	3	6.259	0.469	1	845.6	36.363636	K.LLGKVTIAQGGVLPNIQAVLLPK.K
H2A2A_MOUSE	MK_SCX_44.12675.12675.3	3	5.656	0.573	1	1614.5	35.714287	R.VGAGAPVYM*AAVLEYLTAEILELAGNAAR.D
H2A2A_MOUSE	MK_SCX_51.434.434.2	2	2.215	0.197	1	400.6	91.66667	R.KGNYAER.V
H2A2A_MOUSE	MK_SCX_55.6292.6292.2	2	4.844	0.506	1	809.6	55.88235	R.HLQLAIRNDEELNKLLGK.V
H2A2A_MOUSE	MK_SCX_55.6308.6308.3	3	5.456	0.553	1	1073.9	45.588234	R.HLQLAIRNDEELNKLLGK.V
H2A2B_MOUSE	MK_SCX_16.8488.8488.3	3	4.205	0.505	1	395.4	35.227272	K.LLGGVTIAQGGVLPNIQAVLLPK.K
H2A2B_MOUSE	MK_SCX_16.8659.8659.2	2	5.221	0.639	1	1363.3	61.363636	K.LLGGVTIAQGGVLPNIQAVLLPK.K
H2A2B_MOUSE	MK_SCX_21.10122.10122.2	2	4.788	0.564	1	925.3	37.931034	R.NDEELNKLLGGVTIAQGGVLPNIQAVLLPK.K
H2A2B_MOUSE	MK_SCX_21.10314.10314.3	3	6.446	0.586	1	1883.8	40.517242	R.NDEELNKLLGGVTIAQGGVLPNIQAVLLPK.K
H2AV_MOUSE	MK_SCX_20_1.4609.4609.2	2	3.097	0.378	1	1434.1	88.88889	R.GDEELDSLIK.A
H2AV_MOUSE	MK_SCX_48.10862.10862.2	2	4.094	0.444	1	774.1	59.375	R.HLQLAIRGDEELDSLIK.A
H2AV_MOUSE	MK_SCX_48.11033.11033.3	3	4.108	0.58	1	1313.8	50	R.HLQLAIRGDEELDSLIK.A
H2AV_MOUSE	MK_SCX_51.3611.3611.2	2	4.527	0.479	1	1626	80.769226	K.ATIAGGGVIPHIHK.S
H2AW_MOUSE	MK_SCX_18.6620.6620.2	2	4.471	0.503	1	1005.2	52.499996	K.SQGPLEVAEAAVSQSSGLAAK.F
H2AW_MOUSE	MK_SCX_23.4952.4952.2	2	2.379	0.354	1	438.3	70	K.SVAFPPFPSGR.N
H2AW_MOUSE	MK_SCX_31.6621.6621.3	3	3.988	0.563	1	929.4	36.904762	R.KSQGPLEVAEAAVSQSSGLAAK.F
H2AW_MOUSE	MK_SCX_31.6624.6624.2	2	6.104	0.689	1	3498.6	71.42857	R.KSQGPLEVAEAAVSQSSGLAAK.F
H2AW_MOUSE	MK_SCX_31.7475.7475.2	2	6.051	0.574	1	3371.7	87.5	R.HILLAVANDEELNQLLK.G
H2AW_MOUSE	MK_SCX_32.5105.5105.3	3	3.102	0.296	1	642.1	42.857143	K.LSLTQSDISHIGSMR.V
H2AW_MOUSE	MK_SCX_32.5113.5113.2	2	4.541	0.583	1	1746.9	75	K.LSLTQSDISHIGSMR.V
H2AW_MOUSE	MK_SCX_32.7448.7448.3	3	4.946	0.271	1	835.4	46.875	R.HILLAVANDEELNQLLK.G
H2AW_MOUSE	MK_SCX_52.4437.4437.3	3	3.44	0.371	1	946.6	44.230766	K.AGGKEFLETVKELR.K
H2AY_MOUSE	MK_SCX_17.4644.4644.2	2	5.823	0.694	1	850.5	54.761906	K.AASADSTTEGTPTDGFTVLSTK.S
H2AY_MOUSE	MK_SCX_20_1.6425.6425.2	2	2.858	0.379	1	537	55.263157	K.NGPLEVAGAAISAGHGLPAK.F
H2AY_MOUSE	MK_SCX_24.4566.4566.2	2	2.19	0.14	1	334.9	65	K.SIAFPSIGSGR.N
H2AY_MOUSE	MK_SCX_26.6407.6407.2	2	5.795	0.554	1	1033.3	60.526318	K.GVTIASGGVLPNIHPELLAK.K
H2AY_MOUSE	MK_SCX_26.6423.6423.3	3	3.593	0.294	1	1187.3	38.157894	K.GVTIASGGVLPNIHPELLAK.K
H2AY_MOUSE	MK_SCX_29.5503.5503.3	3	4.29	0.65	1	1645.8	48.684208	K.NGPLEVAGAAISAGHGLPAK.F
H2AY_MOUSE	MK_SCX_35.4039.4039.2	2	3.1	0.526	1	1377.6	75	K.GKLEAIITPPPAK.K
H2AY_MOUSE	MK_SCX_53.6146.6146.2	2	3.859	0.383	1	1105.1	69.230774	K.KGGKEFVEAVLELR.K
H2AY_MOUSE	MK_SCX_53.6297.6297.3	3	4.369	0.408	1	1520.9	50	K.KGGKEFVEAVLELR.K
H2B1A_MOUSE	MK_SCX_20_1.12043.12043.2	2	2.887	0.146	1	470.2	50	K.AM*SIMNSFVTDIFER.I
H2B1A_MOUSE	MK_SCX_2201.11078.11078.2	2	2.892	0.211	1	521.3	81.25	R.LLLPGELAK.H
H2B1A_MOUSE	MK_SCX_47.419.419.2	2	2.96	0.453	1	1308.5	100	K.HAVSEGTK.A
H2B1A_MOUSE	MK_SCX_51.2778.2778.3	3	3.077	0.319	1	1553.7	51.923077	K.VLKQVHPDTGISSK.A
H2B1A_MOUSE	MK_SCX_51.490.490.2	2	2.622	0.286	1	419	91.66667	K.RSTITSR.E
H2B1A_MOUSE	MK_SCX_60.5958.5958.2	2	2.479	0.287	1	381.6	91.66667	R.LAHYNKR.S
H2B1B_MOUSE	MK_SCX_19.8819.8819.1	1	2.246	0.363	1	301.8	50	K.AMGIMNSFVNDIFER.I
H2B1B_MOUSE	MK_SCX_20_1.6980.6980.3	3	4.915	0.387	1	1716.1	48.214287	K.AM*GIM*NSFVNDIFER.I

H2B1B_MOUSE	MK_SCX_20_1.7575.7575.2	2	5.05	0.399	1	1520.7	71.42857	K.AM*GIM*NSFVNDIFER.I
H2B1B_MOUSE	MK_SCX_20_1.8026.8026.2	2	5.094	0.135	1	2148.2	78.57143	K.AMGIM*NSFVNDIFER.I
H2B1B_MOUSE	MK_SCX_20_1.9120.9120.2	2	5.639	0.178	1	2565.4	85.71429	K.AM*GIMNSFVNDIFER.I
H2B1B_MOUSE	MK_SCX_20_1.9453.9453.3	3	4.565	0.405	1	1627.8	55.35714	K.AMGIMNSFVNDIFER.I
H2B1B_MOUSE	MK_SCX_20_1.9456.9456.2	2	5.126	0.531	1	2563.8	82.14286	K.AMGIMNSFVNDIFER.I
H2B1B_MOUSE	MK_SCX_2201.3847.3847.2	2	2.171	0.453	1	322	62.5	K.ESYSVYVYK.V
H2B1B_MOUSE	MK_SCX_42.3545.3545.2	2	2.942	0.452	1	914.6	83.33333	R.KESYSVYVYK.V
H2B1B_MOUSE	MK_SCX_55.3223.3223.3	3	3.603	0.313	1	748.7	47.727272	R.SRKESYSVYVYK.V
H31_MOUSE	MK_SCX_23.11524.11524.3	3	5.823	0.537	1	466.2	27.419355	R.FQSSAVM*ALQEACEAYLVGLFEDTNLCAIHAK.R
H31_MOUSE	MK_SCX_23.11879.11879.3	3	4.456	0.306	1	701.2	26.612906	R.FQSSAVMALQEACEAYLVGLFEDTNLCAIHAK.R
H31_MOUSE	MK_SCX_24.3782.3782.2	2	2.374	0.182	1	618	91.66667	K.STELLIR.K
H31_MOUSE	MK_SCX_33.3755.3755.2	2	3.228	0.388	1	845.9	85	R.EIAQDFKTDLR.F
H31_MOUSE	MK_SCX_47.3057.3057.2	2	2.273	0.212	1	345.4	68.75	R.YRPGTVALR.E
H31_MOUSE	MK_SCX_56.3397.3397.2	2	2.716	0.361	1	379.1	59.090908	R.YRPGTVALREIR.R
H4_MOUSE	MK_SCX_20_1.5375.5375.2	2	3.715	0.49	1	941.5	81.818184	K.TVTAM*DVVYALK.R
H4_MOUSE	MK_SCX_20_1.6703.6703.2	2	4.181	0.582	1	1287.6	90.909096	K.TVTAMDVVYALK.R
H4_MOUSE	MK_SCX_23.3930.3930.2	2	3.985	0.447	1	1210.7	94.44444	R.ISGLIYEETR.G
H4_MOUSE	MK_SCX_24.4700.4700.2	2	3.27	0.157	1	535.6	85.71429	K.VFLENVIR.D
H4_MOUSE	MK_SCX_31.3570.3570.3	3	3.397	0.312	1	1002.2	50	R.DNIQGITKPAIR.R
H4_MOUSE	MK_SCX_31.3602.3602.2	2	3.299	0.374	1	1273.8	81.818184	R.DNIQGITKPAIR.R
H4_MOUSE	MK_SCX_32.15687.15687.3	3	3.069	0.345	1	339.3	37.5	K.TVTAMDVVYALKR.Q
H4_MOUSE	MK_SCX_32.2159.2159.2	2	3.382	0.42	1	772.8	83.33333	R.DAVTYTEHAK.R
H4_MOUSE	MK_SCX_32.5056.5056.2	2	3.452	0.537	1	689.6	75	K.TVTAM*DVVYALKR.Q
H4_MOUSE	MK_SCX_32.5791.5791.2	2	3.857	0.472	1	1725.6	87.5	K.TVTAMDVVYALKR.Q
H4_MOUSE	MK_SCX_35.4979.4979.2	2	3.863	0.471	1	809.3	79.16667	R.KTVTAM*DVVYALK.R
H4_MOUSE	MK_SCX_35.9786.9786.2	2	3.647	0.444	1	1158.4	83.33333	R.KTVTAMDVVYALK.R
H4_MOUSE	MK_SCX_42.8024.8024.3	3	5.42	0.525	1	1420.7	45.588234	K.VFLENVIRDAVTYTEHAK.R
H4_MOUSE	MK_SCX_42.8026.8026.2	2	4.102	0.598	1	1221.2	61.764706	K.VFLENVIRDAVTYTEHAK.R
H4_MOUSE	MK_SCX_44.3761.3761.2	2	3.083	0.352	1	721.7	75	K.RISGLIYEETR.G
H4_MOUSE	MK_SCX_44.3762.3762.3	3	3.306	0.293	1	1057.8	50	K.RISGLIYEETR.G
H4_MOUSE	MK_SCX_51.4386.4386.3	3	3.673	0.371	1	771.5	44.230766	R.KTVTAM*DVVYALKR.Q
H4_MOUSE	MK_SCX_51.5276.5276.2	2	5.738	0.58	1	3068.1	88.46153	R.KTVTAMDVVYALKR.Q
H4_MOUSE	MK_SCX_51.5314.5314.3	3	4.974	0.495	1	1531.4	50	R.KTVTAMDVVYALKR.Q
H4_MOUSE	MK_SCX_54.8202.8202.3	3	3.01	0.186	1	349.9	30.555555	K.VFLENVIRDAVTYTEHAKR.K
HA10_MOUSE	MK_SCX_25.3854.3854.2	2	3.213	0.322	1	1619.4	85	K.WEQAGAAEYYR.A
HA12_MOUSE	MK_SCX_23.4938.4938.2	2	4.225	0.517	1	1302.1	81.818184	R.WIEQEGPEYWER.E
HA12_MOUSE	MK_SCX_35.4960.4960.2	2	3.8	0.48	1	420.2	64.28571	R.YFVTAVSRPGFGEPR.Y
HA12_MOUSE	MK_SCX_35.5036.5036.3	3	3.571	0.578	1	867.8	44.642857	R.YFVTAVSRPGFGEPR.Y
HA1D_MOUSE	MK_SCX_17.5225.5225.2	2	5.561	0.57	1	1122.5	65	K.GVNYALAPGSQTSDLSLPDGK.V
HA1D_MOUSE	MK_SCX_19.5398.5398.2	2	4.679	0.168	1	667.3	61.764706	R.APWMEQEGPEYWEEQTQR.A
HA1D_MOUSE	MK_SCX_21.5511.5511.2	2	2.553	0.186	1	811.7	75	K.TWTAADTAALITR.R
HA1D_MOUSE	MK_SCX_43.3544.3544.3	3	3.104	0.286	1	2467.3	65.909096	R.KWEQAGDAEYYR.A
HA1L_MOUSE	MK_SCX_34.4055.4055.3	3	3.521	0.44	1	1251.7	48.214287	R.YFETAVSRPGLGEPR.Y
HA1L_MOUSE	MK_SCX_34.4074.4074.2	2	4.062	0.445	1	579.3	67.85714	R.YFETAVSRPGLGEPR.Y
HA2D_MOUSE	MK_SCX_16.8333.8333.2	2	4.525	0.595	1	728.6	50	R.LPEFGQLILFEPQGGLQNIAAEK.H
HAOX2_MOUSE	MK_SCX_19.4856.4856.2	2	2.724	0.278	1	639.9	66.66667	R.QLDEVPASIDALR.E
HAOX2_MOUSE	MK_SCX_19.5143.5143.2	2	4.327	0.616	1	1417	76.92308	K.ALVVTVDAPVLGNR.R
HAOX2_MOUSE	MK_SCX_21.5474.5474.2	2	3.969	0.256	1	1161.8	83.33333	R.SLLDLEANIK.L
HAOX2_MOUSE	MK_SCX_2201.3378.3378.2	2	2.44	0.345	1	872.4	88.88889	K.IEVYM*DGGVR.T
HAOX2_MOUSE	MK_SCX_2201.5372.5372.2	2	2.184	0.196	1	626.1	66.66667	K.IEVYMDGGVR.T
HAOX2_MOUSE	MK_SCX_25.16402.16402.3	3	4.872	0.503	1	1130.7	35.416664	K.TSWDFIEGEADDGITYNDNLAAFRR.I

HAOX2_MOUSE	MK_SCX_33.4191.4191.2	2	2.881	0.32	1	848.7	70.83333	K.GILTKEDAELAVK.H
HAP28_MOUSE	MK_SCX_19.4396.4396.2	2	4.277	0.54	1	830.6	63.333332	R.QYTSPEEIDAQLQAEK.Q
HAP28_MOUSE	MK_SCX_33.4613.4613.3	3	4.312	0.446	1	1009.7	41.17647	R.VRQYTSPEEIDAQLQAEK.Q
HAP28_MOUSE	MK_SCX_36.4420.4420.3	3	3.211	0.267	1	870.3	42.307693	R.KGVEGLIDIENPNR.V
HAP28_MOUSE	MK_SCX_50.3866.3866.3	3	3.266	0.348	1	846.6	44.642857	K.KVTQLDLDGPKELSR.R
HAP28_MOUSE	MK_SCX_54.4197.4197.3	3	3.583	0.315	1	1530.7	46.42857	K.RKGVEGLIDIENPNR.V
HAX1_MOUSE	MK_SCX_17.11159.11159.2	2	5.303	0.441	1	1903.1	75	R.SSALDDPFSILDLLLGR.W
HAX1_MOUSE	MK_SCX_17.6616.6616.2	2	5.444	0.564	1	830.4	65	R.ESYAFDGSQPPEEFGFSFSPR.G
HAX1_MOUSE	MK_SCX_18.8956.8956.3	3	4.491	0.626	1	501.8	25.80645	R.DFNSIFSEMGAWTLPSHSPELPGPESETPGER.L
HAX1_MOUSE	MK_SCX_41.5495.5495.2	2	2.325	0.316	1	307.5	68.181816	R.FHGNFGFDDLVR.D
HAX1_MOUSE	MK_SCX_47.3352.3352.3	3	3.952	0.426	1	1485.4	58.333332	R.DSMLKYPDSHQPR.I
HB2A_MOUSE	MK_SCX_14.4772.4772.2	2	2.551	0.259	1	341.6	56.666668	R.NGQEETVGVSSTQLIR.N
HB2A_MOUSE	MK_SCX_24.5880.5880.3	3	3.663	0.411	1	1091.8	36.904762	R.AVTELGRPDAEYWNSQPEILER.T
HB2A_MOUSE	MK_SCX_25.6082.6082.2	2	3.847	0.509	1	706.8	47.61905	R.AVTELGRPDAEYWNSQPEILER.T
HB2D_MOUSE	MK_SCX_40.4428.4428.2	2	3.927	0.141	1	583.5	66.66667	R.RLEQPNVAISLSR.T
HBA_MOUSE	MK_SCX_24.4244.4244.2	2	2.739	0.424	1	726.4	81.25	R.MFASFPTTK.T
HBA_MOUSE	MK_SCX_37.3625.3625.3	3	4.721	0.239	1	1879.8	50	K.IGGHGAEYGAEALER.M
HBA_MOUSE	MK_SCX_38.3558.3558.2	2	4.535	0.48	1	1672.1	78.57143	K.IGGHGAEYGAEALER.M
HBA_MOUSE	MK_SCX_43.4026.4026.2	2	2.679	0.355	1	339.2	62.5	K.LRVDPVNFK.L
HBA_MOUSE	MK_SCX_49.4840.4840.2	2	4.688	0.647	1	1650.6	76.666664	K.TYFPHFDVSHGSAQVK.G
HBA_MOUSE	MK_SCX_49.4856.4856.3	3	3.265	0.564	1	614.9	43.333332	K.TYFPHFDVSHGSAQVK.G
HBB1_MOUSE	MK_SCX_18.5829.5829.2	2	5.215	0.597	1	1569.2	66.66667	R.YFDSFGDLSSASAIM*GNAK.V
HBB1_MOUSE	MK_SCX_18.6822.6822.2	2	7.269	0.685	1	3942.9	86.11111	R.YFDSFGDLSSASAIMGNAK.V
HBB1_MOUSE	MK_SCX_2201.5934.5934.2	2	3.102	0.392	1	1337.6	88.88889	R.LLVVYPWTQR.Y
HBB1_MOUSE	MK_SCX_29.5972.5972.2	2	4.361	0.507	1	2206.8	80	K.VITAFNDGLNHLDSLK.G
HBB1_MOUSE	MK_SCX_47.5648.5648.3	3	4.782	0.494	1	1778.2	53.125	K.KVITAFNDGLNHLDSLK.G
HCC1_MOUSE	MK_SCX_18.3973.3973.2	2	5.68	0.603	1	2343	77.77778	R.FGIVTSSAGTGTTEDTEAK.K
HCC1_MOUSE	MK_SCX_2201.4504.4504.2	2	2.679	0.286	1	676.1	77.77778	R.FNVPVSLESK.K
HCC1_MOUSE	MK_SCX_24.4535.4535.2	2	3.524	0.432	1	1126	88.88889	R.FGLNVSSISR.K
HCD2_MOUSE	MK_SCX_16.8171.8171.2	2	5.622	0.635	1	1352.7	63.15789	R.VVTIAPGLFATPLLTTLPEK.V
HCD2_MOUSE	MK_SCX_16.8206.8206.3	3	3.395	0.451	1	653	36.842106	R.VVTIAPGLFATPLLTTLPEK.V
HCD2_MOUSE	MK_SCX_2201.11182.11182.3	3	5.71	0.516	1	2129.3	38	R.LGDPAEYAHLVQTIIENPFLNGEVIR.L
HCD2_MOUSE	MK_SCX_2201.5567.5567.2	2	4.591	0.545	1	1448.9	86.36364	R.NFLASQVPFPSR.L
HCD2_MOUSE	MK_SCX_24.9812.9812.3	3	3.183	0.436	1	356	29.761904	R.VVTIAPGLFATPLLTTLPEKVR.N
HCDH_MOUSE	MK_SCX_15.10976.10976.3	3	3.826	0.431	1	546.6	25.862068	K.TLSCLSTSTDAASVVHSTDLVVEAIVENLK.L
HCDH_MOUSE	MK_SCX_16.8661.8661.3	3	4.53	0.545	1	985.1	34.523808	K.LGAGYPM*GPFELLDYVGLDTTK.F
HCDH_MOUSE	MK_SCX_16.8755.8755.2	2	5.44	0.641	1	853.3	54.761906	K.LGAGYPM*GPFELLDYVGLDTTK.F
HCDH_MOUSE	MK_SCX_16.9236.9236.2	2	5.719	0.622	1	1294.5	61.904762	K.LGAGYPMGPFELLDYVGLDTTK.F
HCDH_MOUSE	MK_SCX_16.9270.9270.3	3	5.06	0.426	1	1393.8	38.095238	K.LGAGYPMGPFELLDYVGLDTTK.F
HCDH_MOUSE	MK_SCX_20_1.8031.8031.2	2	3.466	0.398	1	803.5	90	R.LLVPYLIEAVR.L
HCDH_MOUSE	MK_SCX_20_1.8208.8208.3	3	3.618	0.272	1	1798.2	60.000004	R.LLVPYLIEAVR.L
HCDH_MOUSE	MK_SCX_21.7724.7724.3	3	5.452	0.403	1	735.7	27.67857	K.FILDGWHEM*EPENPLFQPSPSM*NNLVAQK.K
HCDH_MOUSE	MK_SCX_21.8316.8316.3	3	4.173	0.205	1	369.6	25	K.FILDGWHEMEPENPLFQPSPSM*NNLVAQK.K
HCDH_MOUSE	MK_SCX_21.8675.8675.3	3	5.154	0.548	1	797	31.25	K.FILDGWHEMEPENPLFQPSPSMNNLVAQK.K
HCDH_MOUSE	MK_SCX_21.8691.8691.2	2	4.217	0.482	1	394.7	26.785713	K.FILDGWHEMEPENPLFQPSPSMNNLVAQK.K
HCDH_MOUSE	MK_SCX_30.7556.7556.3	3	5.531	0.177	1	887.4	29.310345	K.FILDGWHEM*EPENPLFQPSPSMNNLVAQKK.L
HCDH_MOUSE	MK_SCX_30.8223.8223.3	3	6.139	0.579	1	984	31.896553	K.FILDGWHEMEPENPLFQPSPSMNNLVAQKK.L
HCDH_MOUSE	MK_SCX_31.4030.4030.2	2	4.138	0.469	1	1235.8	75	K.LVEVIKTPMTSQK.T
HCDH_MOUSE	MK_SCX_33.3586.3586.3	3	3.237	0.461	1	652.2	48.076923	K.FTENPKAGDEFVEK.T
HCDH_MOUSE	MK_SCX_33.3603.3603.2	2	4.797	0.538	1	1847.2	84.61539	K.FTENPKAGDEFVEK.T
HCDH_MOUSE	MK_SCX_34.6063.6063.2	2	3.358	0.532	1	579.5	73.07692	R.FAGLHFFNPVPM*M*K.L

HCDH_MOUSE	MK_SCX_34.6612.6612.2	2	3.417	0.173	1	896.8	73.07692	R.FAGLHFFNPVPMM*K.L
HCDH_MOUSE	MK_SCX_34.7255.7255.2	2	4.327	0.45	1	1111.2	76.92308	R.FAGLHFFNPVPMMK.L
HCDH_MOUSE	MK_SCX_34.7343.7343.3	3	3.704	0.388	1	761.5	48.076923	R.FAGLHFFNPVPMMK.L
HCDH_MOUSE	MK_SCX_48.5723.5723.3	3	3.536	0.366	1	497	34.375	R.QDRFAGLHFFNPVPM*M*K.L
HCDH_MOUSE	MK_SCX_48.7129.7129.3	3	3.486	0.348	1	409.8	31.25	R.QDRFAGLHFFNPVPMMK.L
HCDH_MOUSE	MK_SCX_51.3282.3282.3	3	3.995	0.444	1	533	42.857143	K.KFTENPKAGDEFVEK.T
HCFC1_MOUSE	MK_SCX_15.5837.5837.2	2	4.841	0.599	1	535.8	42.857143	R.AVTTVTQSTPVPGPSVPPPEELQVSPGPR.Q
HCFC1_MOUSE	MK_SCX_17.3132.3132.3	3	3.205	0.17	1	304.4	18.965517	K.TVAVTPGTTTLPATVKVASSPVMVSNPATR.M
HCLS1_MOUSE	MK_SCX_16.5158.5158.2	2	4.96	0.622	1	1524.4	68.42105	R.EVQQPSMPVEEPAAPAQLPK.K
HDAC2_MOUSE	MK_SCX_33.4591.4591.3	3	4.768	0.394	1	563.9	40.27778	K.LHISPSNMTNQNTPEYMEK.I
HDGF_MOUSE	MK_SCX_15.5196.5196.3	3	3.729	0.305	1	457.5	24.264706	K.NSTPSEPDSGQGPPAEEEEGEEEAAKEEAEAQGVR.D
HDGF_MOUSE	MK_SCX_16.3669.3669.2	2	3.08	0.553	1	356.1	34	K.NSTPSEPDSGQGPPAEEEEGEEEAAK.E
HDGF_MOUSE	MK_SCX_35.8845.8845.3	3	3.826	0.302	1	1250.2	43.75	R.KGFSEGLWEIENNPTVK.A
HDGF_MOUSE	MK_SCX_36.5889.5889.2	2	5.878	0.535	1	1893	68.75	R.KGFSEGLWEIENNPTVK.A
HDGF_MOUSE	MK_SCX_41.5888.5888.3	3	4.565	0.536	1	954.7	37.5	K.ESGDHEEEDKEIAALEGERPLPVEVEK.N
HDGF_MOUSE	MK_SCX_43.7055.7055.3	3	3.425	0.496	1	493	30.952381	K.STANKYQVFFFGTHETAFLGPK.D
HDGF_MOUSE	MK_SCX_57.3757.3757.2	2	2.175	0.371	1	481	77.77778	K.MKGYPHWPAR.I
HEBP1_MOUSE	MK_SCX_19.3988.3988.2	2	5.634	0.458	1	658.8	58.823532	R.IPNQFQGSPPAPSDESVK.I
HEBP1_MOUSE	MK_SCX_2201.7850.7850.3	3	3.863	0.543	1	805.2	29.032257	K.YVGGTNDKGVGMGMTVPVSFAVFPNEDGSLQK.K
HEBP1_MOUSE	MK_SCX_23.8930.8930.3	3	4.73	0.392	1	767.3	29.62963	R.NSLFGSVETWPWQVLSTGGKEDVSYEER.A
HEBP1_MOUSE	MK_SCX_26.4473.4473.3	3	6.07	0.462	1	1144.7	41.666664	R.IPNQFQGSPPAPSDESVKIEER.E
HEBP1_MOUSE	MK_SCX_26.4499.4499.2	2	5.071	0.518	1	795.2	54.761906	R.IPNQFQGSPPAPSDESVKIEER.E
HEBP1_MOUSE	MK_SCX_27.4918.4918.2	2	4.581	0.488	1	1530.9	73.333336	K.FATVEVTDKPVDEALR.E
HEBP1_MOUSE	MK_SCX_27.4923.4923.3	3	4.54	0.502	1	1018.9	50	K.FATVEVTDKPVDEALR.E
HEM2_MOUSE	MK_SCX_14.9503.9503.3	3	4.743	0.531	1	340.4	20.27027	R.SWQTAASTVSASNLIYPIFVTDVPDDVQPIASLPGVAR.Y
HEM2_MOUSE	MK_SCX_16.3826.3826.2	2	5.896	0.538	1	2329.3	69.44444	K.DEQGSAADSEDSPTIEAVR.L
HEM2_MOUSE	MK_SCX_18.9020.9020.2	2	2.315	0.14	1	364.3	43.333332	R.AGADIIITYFAPQLLK.W
HEM2_MOUSE	MK_SCX_21.8536.8536.2	2	5.246	0.481	1	724.6	52.380955	R.DIQEGADMLMVKPGLPYLDMVR.E
HEM2_MOUSE	MK_SCX_28.3678.3678.3	3	5.795	0.405	1	1706.6	41.666664	R.VPKDEQGSAADSEDSPTIEAVR.L
HEM6_MOUSE	MK_SCX_19.6656.6656.2	2	3.647	0.358	1	1474.1	79.16667	R.IESILMSLPLTAR.W
HEM6_MOUSE	MK_SCX_20_1.7044.7044.2	2	2.289	0.249	1	397.3	58.333332	R.IESILM*SLPLTAR.W
HEM6_MOUSE	MK_SCX_26.6963.6963.3	3	3.414	0.467	1	459.4	31.578945	R.GIGGIFFDDLDSPSKEEAFR.F
HEM6_MOUSE	MK_SCX_32.5890.5890.3	3	3.796	0.469	1	1517.6	50	K.LPFTAMGVSSVIHPK.N
HEMH_MOUSE	MK_SCX_16.7868.7868.2	2	3.415	0.468	1	600.4	43.18182	K.TGILM*LNM*GGPETLGEVQDFLQR.L
HEMH_MOUSE	MK_SCX_16.8843.8843.2	2	4.944	0.709	1	1383.7	54.545456	K.TGILMLNMGGPETLGEVQDFLQR.L
HEMH_MOUSE	MK_SCX_16.8880.8880.3	3	5.182	0.435	1	892.7	32.954548	K.TGILMLNMGGPETLGEVQDFLQR.L
HEMH_MOUSE	MK_SCX_29.3907.3907.3	3	3.089	0.306	1	417.2	45.833336	K.LLDELSPATAPHK.Y
HEMH_MOUSE	MK_SCX_29.7242.7242.2	2	3.015	0.239	1	572.1	57.14286	R.LFLDRDLMTLPIQNK.L
HEMH_MOUSE	MK_SCX_33.5244.5244.2	2	2.988	0.389	1	449.3	57.14286	R.YVHPLTEEAIEEMER.D
HEMO_MOUSE	MK_SCX_23.4519.4519.2	2	2.176	0.127	1	302.7	55	R.FNPVTGEVPPR.Y
HEMO_MOUSE	MK_SCX_41.5984.5984.2	2	2.06	0.294	1	410.5	54.166668	R.WKNPITSVDAAFR.G
HERC2_MOUSE	MK_SCX_12.7894.7894.2	2	2.32	0.275	1	377.8	55	K.IHGLILLGRIR.A
HERC2_MOUSE	MK_SCX_21.4419.4419.2	2	2.298	0.213	1	516.4	50	K.FISDGSVNGWGWR.F
HEXB_MOUSE	MK_SCX_23.6501.6501.2	2	2.933	0.473	1	508.4	83.33333	R.LQPALWPFPR.S
HEXB_MOUSE	MK_SCX_31.4717.4717.2	2	3.113	0.6	1	381.6	63.333332	R.VIPEFDTPGHTQSWGK.G
HFE_MOUSE	MK_SCX_17.8652.8652.2	2	3.584	0.439	1	681.9	50	R.YLFMGASEPDLGLPLFEAR.G
HINT1_MOUSE	MK_SCX_20_1.4047.4047.2	2	4.614	0.57	1	1272.2	88.46153	K.AQVAQPGGDTIFGK.I
HINT1_MOUSE	MK_SCX_51.7085.7085.3	3	5.968	0.601	1	1652	42.708336	K.KHISQISVADDDDESLLGHLMIVGK.K
HINT1_MOUSE	MK_SCX_53.5188.5188.3	3	3.038	0.411	1	433.9	27.173912	R.M*VVNEGADGGQSVYHIHLHVLGGR.Q
HINT1_MOUSE	MK_SCX_53.5506.5506.3	3	4.576	0.221	1	1363.1	38.04348	R.MVVNEGADGGQSVYHIHLHVLGGR.Q
HINT2_MOUSE	MK_SCX_2201.3841.3841.2	2	2.288	0.418	1	325.4	66.66667	K.AAPGGASPTIFSR.I

HINT2_MOUSE	MK_SCX_25.6103.6103.3	3	3.311	0.498	1	525.6	41.07143	R.DVAPQAPVHFLVIPR.K
HINT2_MOUSE	MK_SCX_25.6105.6105.2	2	4.474	0.566	1	807.4	71.42857	R.DVAPQAPVHFLVIPR.K
HINT2_MOUSE	MK_SCX_25.7312.7312.2	2	6.429	0.461	1	3034.3	68.42105	R.ISQAEEDDQQLLGHLLLVAK.K
HINT2_MOUSE	MK_SCX_25.7407.7407.3	3	5.413	0.511	1	1225.2	44.736843	R.ISQAEEDDQQLLGHLLLVAK.K
HIRP5_MOUSE	MK_SCX_36.3888.3888.3	3	5.362	0.476	1	2691.2	58.928574	R.IRPTVQEDGGDVIYR.G
HIRP5_MOUSE	MK_SCX_41.6186.6186.3	3	5.368	0.533	1	873.1	35.227272	R.IRPTVQEDGGDVIYRGFEDGIVR.L
HMGA2_MOUSE	MK_SCX_18.3274.3274.2	2	4.245	0.62	1	1023.9	54.761906	R.GEGAGQPSTSAQGQPAAPVPQK.R
HMGA2_MOUSE	MK_SCX_29.3122.3122.3	3	3.182	0.464	1	512.1	35.227272	R.GEGAGQPSTSAQGQPAAPVPQKR.G
HMGB1_MOUSE	MK_SCX_28.4144.4144.2	2	5.177	0.566	1	1890.4	67.64706	K.LGEMWNNTAADDKQPYEK.K
HMGB1_MOUSE	MK_SCX_29.3811.3811.3	3	4.373	0.504	1	1359.8	45.588234	K.LGEM*WNNTAADDKQPYEK.K
HMGB1_MOUSE	MK_SCX_29.4089.4089.3	3	4.163	0.52	1	706.6	39.705883	K.LGEMWNNTAADDKQPYEK.K
HMGB1_MOUSE	MK_SCX_42.3761.3761.3	3	4.573	0.543	1	909	43.055553	K.LGEMWNNTAADDKQPYEKK.A
HMGB1_MOUSE	MK_SCX_48.3531.3531.3	3	5.287	0.498	1	2109.9	52.77778	K.KLGEM*WNNTAADDKQPYEK.K
HMGB1_MOUSE	MK_SCX_48.3923.3923.2	2	4.512	0.373	1	1689.5	63.88889	K.KLGEMWNNTAADDKQPYEK.K
HMGB1_MOUSE	MK_SCX_48.3947.3947.3	3	4.96	0.489	1	1438.3	45.833336	K.KLGEMWNNTAADDKQPYEK.K
HMGB1_MOUSE	MK_SCX_51.3501.3501.3	3	3.942	0.419	1	744	46.42857	K.IKGEHPGLSIGDVAK.K
HMGB1_MOUSE	MK_SCX_52.3429.3429.2	2	3.873	0.497	1	798.3	75	K.IKGEHPGLSIGDVAK.K
HMGB1_MOUSE	MK_SCX_52.3600.3600.3	3	3.393	0.315	1	650.2	40.384613	K.HPDASVNFSEFSKK.C
HMGB1_MOUSE	MK_SCX_54.3720.3720.2	2	4.755	0.352	1	978.8	84.61539	K.KHPDASVNFSEFSK.K
HMGB1_MOUSE	MK_SCX_58.13499.13499.2	2	2.108	0.392	1	317	50	K.KHPDASVNFSEFSKK.C
HMGB2_MOUSE	MK_SCX_35.3620.3620.3	3	4.68	0.393	1	1184.6	56.25	K.IEHPGLSIGDTAK.K
HMGB2_MOUSE	MK_SCX_43.3952.3952.3	3	4.774	0.5	1	1528.8	44.444447	K.LGEMWSEQSAKDKQPYEQK.A
HMGB2_MOUSE	MK_SCX_50.3956.3956.3	3	3.747	0.483	1	867	50	K.IKIEHPGLSIGDTAK.K
HMGB2_MOUSE	MK_SCX_55.3728.3728.3	3	3.421	0.214	1	733.2	43.333332	K.IKIEHPGLSIGDTAKK.L
HMGB3_MOUSE	MK_SCX_28.5046.5046.3	3	3.4	0.415	1	415.3	31.944445	K.LGEMWNNLSDNEKQPYVTK.A
HMGB3_MOUSE	MK_SCX_46.4868.4868.3	3	5.188	0.544	1	2139.2	51.315792	K.KLGEMWNNLSDNEKQPYVTK.A
HMGCL_MOUSE	MK_SCX_19.8569.8569.2	2	2.932	0.135	1	762.5	65.38461	K.EVSVFGAVSELFTR.K
HMGCL_MOUSE	MK_SCX_20_1.6261.6261.2	2	4.819	0.47	1	1094.3	76.92308	K.FPGINYPVLTPNM*K.G
HMGCL_MOUSE	MK_SCX_20_1.6915.6915.2	2	4.035	0.488	1	1239.2	76.92308	K.FPGINYPVLTPNMK.G
HMGCL_MOUSE	MK_SCX_25.8291.8291.3	3	4.324	0.444	1	917.5	33.333336	K.FPGINYPVLTPNMKGFEEAVAAGAK.E
HMGCL_MOUSE	MK_SCX_30.6435.6435.3	3	3.31	0.334	1	802.5	36.764706	K.GIQKFPGINYPVLTPNMK.G
HMGCL_MOUSE	MK_SCX_31.6576.6576.2	2	4.432	0.573	1	746	58.823532	K.GIQKFPGINYPVLTPNMK.G
HMGCL_MOUSE	MK_SCX_38.4017.4017.2	2	2.039	0.342	1	382.4	66.66667	K.SIVPTPVKIR.L
HMGCL_MOUSE	MK_SCX_8.4666.4666.2	2	2.497	0.366	1	418.1	59.090908	K.NANCSIEESFQR.F
HMOX2_MOUSE	MK_SCX_47.5462.5462.3	3	5.389	0.574	1	789.3	44.444447	R.IHYVGQNEPELLVAHAYTR.Y
HNF1A_MOUSE	MK_SCX_23.5288.5288.3	3	5.967	0.442	1	1274.9	42	R.EVAQQFTHAGQGGLIEEPTGDELPTK.K
HNF1A_MOUSE	MK_SCX_25.5139.5139.2	2	3.157	0.574	1	924.3	87.5	R.AALYTWYVR.K
HNF1B_MOUSE	MK_SCX_19.9087.9087.2	2	3.442	0.504	1	522.4	52.77778	K.LTSLQQELLSALLSSGVTK.E
HNF1B_MOUSE	MK_SCX_20_1.3803.3803.2	2	2.584	0.27	1	691.4	73.07692	K.ELQALNTEEAAEQR.A
HNF4A_MOUSE	MK_SCX_18.3632.3632.2	2	3.42	0.424	1	864.5	50	R.GQAATPETPQPSPPSGSGSESYK.L
HNRH1_MOUSE	MK_SCX_14.5180.5180.2	2	3.155	0.441	1	303.4	58.333332	R.GLPWSCSADEVQR.F
HNRH1_MOUSE	MK_SCX_16.5465.5465.2	2	2.888	0.419	1	502.7	36	R.GAYGGGYGGYDDYNGYNDGYGFGSDR.F
HNRH1_MOUSE	MK_SCX_18.7543.7543.2	2	5.098	0.565	1	1512.9	68.75	R.ATENDIYNFFSPLNPVR.V
HNRH1_MOUSE	MK_SCX_19.6513.6513.2	2	4.327	0.466	1	1357.1	68.75	R.STGEAFVQFASQEIAEK.A
HNRH1_MOUSE	MK_SCX_38.3235.3235.3	3	5.328	0.454	1	1637.8	55	K.HTGPNSPDTANDGFVR.L
HNRH2_MOUSE	MK_SCX_18.7210.7210.2	2	4.266	0.444	1	1620.5	68.75	R.ATENDIYNFFSPLNPM*R.V
HNRH2_MOUSE	MK_SCX_18.8099.8099.2	2	4.911	0.431	1	2138.6	75	R.ATENDIYNFFSPLNPMR.V
HNRH2_MOUSE	MK_SCX_24.11730.11730.3	3	3.66	0.486	1	327	31.25	R.VTGEADVEFATHEDAVAAMAK.D
HNRH2_MOUSE	MK_SCX_24.13732.13732.2	2	2.685	0.308	1	300.1	35	R.VTGEADVEFATHEDAVAAMAK.D
HNRL1_MOUSE	MK_SCX_14.5169.5169.2	2	2.579	0.268	1	466.8	60.714287	R.NYILDQTNVYGSAQR.R
HNRL1_MOUSE	MK_SCX_2201.3070.3070.2	2	2.312	0.131	1	644	92.85714	K.INEEISVK.H

HNRL1_MOUSE	MK_SCX_46.4458.4458.3	3	5.866	0.546	1	2044	51.5625	R.RPLDMEPQQQVYHPELK.T
HNRPC_MOUSE	MK_SCX_18.6074.6074.3	3	3.217	0.474	1	399.7	22.916668	R.AAVAGEDGRM*IAGQVLDINLAAEPK.V
HNRPC_MOUSE	MK_SCX_18.6402.6402.2	2	5.271	0.33	1	2749	86.666664	R.MIAGQVLDINLAAEPK.V
HNRPC_MOUSE	MK_SCX_21.6154.6154.2	2	3.598	0.431	1	1572.7	86.36364	R.VFIGNLNTLVVK.K
HNRPC_MOUSE	MK_SCX_23.5364.5364.2	2	3.328	0.342	1	948.5	75	K.GFAFVQYVNER.N
HNRPC_MOUSE	MK_SCX_31.5054.5054.2	2	2.855	0.402	1	367.2	43.333332	R.MYSYPARVPPPPPIAR.A
HNRPD_MOUSE	MK_SCX_17.6517.6517.2	2	4.136	0.525	1	588.6	47.22222	R.EYFGGFGEVESIELPM*DNK.T
HNRPD_MOUSE	MK_SCX_19.4289.4289.2	2	4.771	0.432	1	1619.5	80.769226	K.IFVGGLSPDTPEEK.I
HNRPD_MOUSE	MK_SCX_2201.6439.6439.2	2	3.497	0.489	1	619.3	81.818184	K.MFIGGLSWDTTK.K
HNRPD_MOUSE	MK_SCX_25.6591.6591.2	2	3.056	0.439	1	619.1	85.71429	R.GFGFVLFK.E
HNRPD_MOUSE	MK_SCX_34.4075.4075.2	2	4.128	0.385	1	695.2	71.42857	K.KIFVGGLSPDTPEEK.I
HNRPD_MOUSE	MK_SCX_34.5541.5541.2	2	3.568	0.466	1	1267.6	75	K.MFIGGLSWDTTKK.D
HNRPG_MOUSE	MK_SCX_19.6312.6312.2	2	2.562	0.125	1	303.3	43.333332	K.SRGFAFVTLESPADAK.D
HNRPG_MOUSE	MK_SCX_21.4606.4606.2	2	3.686	0.259	1	1206.2	75	K.LFIGGLNTETNEK.A
HNRPK_MOUSE	MK_SCX_16.7265.7265.3	3	6.803	0.65	1	3239.3	43.18182	R.IITITGTQDQIQNAQYLLQNSVK.Q
HNRPK_MOUSE	MK_SCX_17.7024.7024.2	2	5.704	0.61	1	1490.1	54.545456	R.IITITGTQDQIQNAQYLLQNSVK.Q
HNRPK_MOUSE	MK_SCX_18.3893.3893.2	2	4.593	0.549	1	727.6	65.625	R.TDYNASVSVPDSSGPER.I
HNRPK_MOUSE	MK_SCX_18.6183.6183.2	2	4.13	0.579	1	1143.4	61.11111	R.GSYGDLGGPIITTQVTIPK.D
HNRPK_MOUSE	MK_SCX_19.8125.8125.3	3	5.047	0.571	1	1215.1	28.030304	K.IDEPLEGSEDRIITITGTQDQIQNAQYLLQNSVK.Q
HNRPK_MOUSE	MK_SCX_30.6296.6296.2	2	3.466	0.31	1	898.6	61.538464	K.IILDLISESPIKGR.A
HNRPK_MOUSE	MK_SCX_46.3900.3900.3	3	5.136	0.497	1	803.5	41.666664	R.HESGASIKIDEPLEGSEDR.I
HNRPL_MOUSE	MK_SCX_20_1.3527.3527.2	2	2.207	0.179	1	413.5	50	-.MVKMAAAGGGGGGGR.Y
HNRPL_MOUSE	MK_SCX_26.4338.4338.3	3	5.741	0.561	1	1535.1	35	K.NDQDTWDYTNPNLSGQGDPGSNPNKR.Q
HNRPL_MOUSE	MK_SCX_26.6456.6456.3	3	6.654	0.656	1	3335.5	36.764706	R.YGPQYGHPPPPPPPPDYGPHADSPVLMVYGLDQSK.M
HNRPL_MOUSE	MK_SCX_29.6177.6177.3	3	3.064	0.441	1	446.1	31.666666	K.SDALETLGFLNHYQM*K.N
HNRPL_MOUSE	MK_SCX_29.6945.6945.3	3	3.066	0.46	1	654.2	41.666664	K.SDALETLGFLNHYQMK.N
HNRPL_MOUSE	MK_SCX_32.3524.3524.3	3	4.146	0.494	1	810	45.588234	K.SKPGAAM*VEM*ADGYAVDR.A
HNRPL_MOUSE	MK_SCX_33.4206.4206.3	3	3.444	0.155	1	1157.8	48.52941	K.SKPGAAMVEM*ADGYAVDR.A
HNRPL_MOUSE	MK_SCX_33.4773.4773.2	2	5.633	0.684	1	1669.5	76.47059	K.SKPGAAMVEMADGYAVDR.A
HNRPL_MOUSE	MK_SCX_33.4820.4820.3	3	3.931	0.57	1	1342.4	42.647057	K.SKPGAAMVEMADGYAVDR.A
HNRPM_MOUSE	MK_SCX_18.7303.7303.2	2	3.563	0.448	1	527.8	43.18182	K.GIGMGNLGPAGMGMEGIGFGINK.I
HNRPM_MOUSE	MK_SCX_21.4688.4688.2	2	3.076	0.426	1	1355	75	R.MGPAMGPALGAGIER.M
HNRPM_MOUSE	MK_SCX_21.6496.6496.2	2	3.958	0.374	1	1055.5	80	R.AFITNIPFDVK.W
HNRPM_MOUSE	MK_SCX_2201.10052.10052.2	2	2.106	0.123	1	413.2	72.22222	R.M*MNGM*KLSGR.E
HNRPM_MOUSE	MK_SCX_2201.15224.15224.2	2	2.381	0.373	1	313.2	50	R.MGLAMGGAGGASFDR.A
HNRPM_MOUSE	MK_SCX_2201.3842.3842.2	2	3.76	0.344	1	1416.8	75	R.MGLAM*GGAGGASFDR.A
HNRPM_MOUSE	MK_SCX_24.4384.4384.2	2	4.004	0.578	1	1361.4	95	R.MGAGMGFGLER.M
HNRPM_MOUSE	MK_SCX_28.6455.6455.2	2	2.09	0.142	1	516	58.333332	K.FNECGHVLYADIK.M
HNRPM_MOUSE	MK_SCX_32.5258.5258.3	3	4.002	0.634	1	1228.8	55.35714	R.MGPLGLDHMASSIER.M
HNRPM_MOUSE	MK_SCX_32.5260.5260.2	2	5.345	0.557	1	2153.7	82.14286	R.MGPLGLDHMASSIER.M
HNRPM_MOUSE	MK_SCX_34.4896.4896.3	3	4.425	0.545	1	730.8	34.090908	R.GNFGGSFAGSFGGAGGHAPGVAR.K
HNRPM_MOUSE	MK_SCX_43.4615.4615.2	2	2.88	0.293	1	592.4	87.5	K.WQSLKDLVK.E
HNRPM_MOUSE	MK_SCX_50.4091.4091.3	3	3.446	0.165	1	472.5	27.380953	R.MGSSIERMGPLGLDHM*ASSIER.M
HNRPQ_MOUSE	MK_SCX_16.5902.5902.2	2	3.773	0.535	1	569.9	37.5	K.YGGPPPDSVYSGQQPSVGTEIFVGK.I
HNRPQ_MOUSE	MK_SCX_16.8127.8127.2	2	3.806	0.502	1	1022.6	79.16667	R.DLFEDELVPLFEK.A
HNRPQ_MOUSE	MK_SCX_21.4063.4063.2	2	2.866	0.497	1	485.9	72.72727	R.TGYTLDVTTGQR.K
HNRPQ_MOUSE	MK_SCX_21.6344.6344.2	2	3.189	0.282	1	507.5	62.5	R.NLANTVTEEILEK.S
HNRPQ_MOUSE	MK_SCX_24.6486.6486.3	3	5.634	0.598	1	1404.7	36.11111	K.YGGPPPDSVYSGQQPSVGTEIFVGKIPR.D
HNRPQ_MOUSE	MK_SCX_38.4093.4093.2	2	2.701	0.387	1	589	75	K.TKEQILEEFSK.V
HOME3_MOUSE	MK_SCX_25.4252.4252.3	3	4.565	0.413	1	1182	38.75	R.EAPDTAEREETQQQVQDLETR.N
HOME3_MOUSE	MK_SCX_35.3633.3633.3	3	3.564	0.307	1	582.4	39.285713	R.AM*ECNLEEARAERER.A

HOOK2_MOUSE	MK_SCX_43.3484.3484.3	3	3.029	0.282	1	640.2	31.25	K.ALQEQGGKTEDPTLLKR.K
HOOK2_MOUSE	MK_SCX_49.3506.3506.3	3	4.702	0.504	1	3318.4	50	R.AGEEHAPAHAQSFLAQQR.M
HOOK3_MOUSE	MK_SCX_16.3438.3438.2	2	2.543	0.255	1	401.8	43.333332	R.LNQSDSIEDPNSPAGR.R
HOOK3_MOUSE	MK_SCX_23.9238.9238.3	3	4.281	0.491	1	910	33.695652	R.QQNDELTTLADEAQSLKDEIDVLR.H
HOOK3_MOUSE	MK_SCX_44.3862.3862.3	3	3.631	0.4	1	560.4	39.0625	K.SLQDQGSKAEDSVLLKK.K
HRBL_MOUSE	MK_SCX_16.5589.5589.2	2	5.076	0.629	1	1345.5	53.846157	R.TLLGDPVPSLSDPASTSSQPGSQSQAR.S
HRBL_MOUSE	MK_SCX_31.3707.3707.3	3	3.748	0.505	1	638.7	34.72222	K.GSVSATPVQGSVPEGKPIR.T
HRX_MOUSE	MK_SCX_19.7437.7437.2	2	2.094	0.185	1	818.1	72.72727	R.FIEDEDYDPPM*K.I
HRX_MOUSE	MK_SCX_19.7971.7971.2	2	2.896	0.255	1	363.2	59.090908	K.NLLDTYNAELLK.S
HRX_MOUSE	MK_SCX_27.4688.4688.2	2	2.092	0.132	1	385.2	37.5	R.ANREQQDAAGVEQPSQK.E
HS105_MOUSE	MK_SCX_10.4519.4519.2	2	2.393	0.29	1	334.8	36.842106	K.AEDVSAIEIVGGATRIPAVK.E
HS105_MOUSE	MK_SCX_14.10626.10626.2	2	2.039	0.139	1	446.1	59.090908	K.KVDQPPEAKKPK.I
HS105_MOUSE	MK_SCX_16.8907.8907.2	2	2.825	0.25	1	309.5	42.105263	R.AKVKELNNVCEPVVTQPKPK.I
HS105_MOUSE	MK_SCX_17.6010.6010.2	2	3.607	0.441	1	542.5	50	R.LLTETEDWLYEEGEDQAK.Q
HS105_MOUSE	MK_SCX_21.5496.5496.2	2	3.251	0.384	1	683.9	69.230774	R.AGGIETIANEFSDR.C
HS105_MOUSE	MK_SCX_30.5078.5078.3	3	3.036	0.351	1	732.2	39.705883	K.FFGKDVSTTLNADEAVAR.G
HS105_MOUSE	MK_SCX_30.5976.5976.3	3	4.541	0.463	1	1002	53.571426	K.IEVPLHSLMAQTQLK.A
HS105_MOUSE	MK_SCX_34.6681.6681.2	2	3.66	0.366	1	351	72.72727	K.QAYIDKLEELMK.M
HS70A_MOUSE	MK_SCX_17.6261.6261.2	2	5.022	0.624	1	674.5	45.652176	K.QTQTFTTYSDNQPGVLIQVYEGER.A
HS70A_MOUSE	MK_SCX_19.5294.5294.2	2	4.602	0.573	1	1763.8	80	R.IINEPTAAAIAYGLDR.T
HS70A_MOUSE	MK_SCX_19.7328.7328.2	2	2.873	0.168	1	713.3	69.230774	R.SFFPEEISSMVLTK.M
HS70A_MOUSE	MK_SCX_20_1.4279.4279.2	2	3.569	0.539	1	703.5	79.16667	R.TTPSYVAFTDTER.L
HS70A_MOUSE	MK_SCX_52.4024.4024.3	3	4.004	0.57	1	1304.7	50	K.HWPFQVVNDGDKPK.V
HS90A_MOUSE	MK_SCX_13.6957.6957.2	2	4.763	0.68	1	424.1	31.818182	K.LGLGIDEDDPTVDDTSAAVTEEMPPLEGDDDTSR.M
HS90A_MOUSE	MK_SCX_18.4018.4018.2	2	2.917	0.352	1	419.1	72.72727	K.EGLELPEDEEEK.K
HS90A_MOUSE	MK_SCX_18.4767.4767.2	2	5.402	0.562	1	2336.9	85.71429	R.NPDDITNEEYGEFYK.S
HS90A_MOUSE	MK_SCX_18.6604.6604.2	2	3.022	0.506	1	663.6	45	R.LVTSPCCIVTSTYGWTANMER.I
HS90A_MOUSE	MK_SCX_19.4940.4940.2	2	4.521	0.506	1	1351.7	76.92308	R.GVVDSEDIPLNLSR.E
HS90A_MOUSE	MK_SCX_20_1.4549.4549.2	2	4.583	0.559	1	2066.8	87.5	R.TLTIVDTGIGM*TK.A
HS90A_MOUSE	MK_SCX_20_1.5311.5311.2	2	4.272	0.41	1	1273.7	86.36364	K.ADLINNLGTIAK.S
HS90A_MOUSE	MK_SCX_20_1.5503.5503.2	2	4.807	0.639	2	2393.4	87.5	R.TLTIVDTGIGMTK.A
HS90A_MOUSE	MK_SCX_2201.3032.3032.2	2	3.359	0.278	1	634.7	87.5	K.YIDQEELNK.T
HS90A_MOUSE	MK_SCX_23.5120.5120.2	2	2.918	0.376	1	765.2	93.75	R.APFDLFENR.K
HS90A_MOUSE	MK_SCX_23.7757.7757.3	3	3.915	0.465	2	582.4	31.25	R.TLTIVDTGIGM*TKADLINNLGTIAK.S
HS90A_MOUSE	MK_SCX_23.8419.8419.3	3	6.053	0.566	1	3097.2	48.958336	R.TLTIVDTGIGMTKADLINNLGTIAK.S
HS90A_MOUSE	MK_SCX_31.4446.4446.2	2	3.863	0.406	1	1574.2	80.769226	R.ELISNSSDALDKIR.Y
HS90A_MOUSE	MK_SCX_33.4928.4928.2	2	4.891	0.562	1	1668.3	79.16667	K.SLTNDWEEHLAVK.H
HS90A_MOUSE	MK_SCX_34.7225.7225.3	3	4.443	0.536	1	1713.3	55.35714	K.HSQFIGYPITLFVEK.E
HS90A_MOUSE	MK_SCX_34.7250.7250.2	2	5.152	0.595	1	1878.5	82.14286	K.HSQFIGYPITLFVEK.E
HS90A_MOUSE	MK_SCX_36.14112.14112.3	3	3.407	0.404	1	390.8	28.947369	K.HNDDEQYAWESSAGGSFTVR.T
HS90A_MOUSE	MK_SCX_39.3460.3460.3	3	3.2	0.254	1	778.2	52.499996	K.IRYESLTDPSK.L
HS90A_MOUSE	MK_SCX_42.6442.6442.3	3	5.144	0.515	1	963.2	26.666668	K.YIDQEELNKTKPIWTRNPDDITNEEYGEFYK.S
HS90A_MOUSE	MK_SCX_43.3483.3483.2	2	3.383	0.424	1	659.8	69.230774	K.EGLELPEDEEEKKK.Q
HS90A_MOUSE	MK_SCX_43.5135.5135.2	2	3.083	0.356	1	1047.6	80	K.HFSVEGQLEFR.A
HS90A_MOUSE	MK_SCX_44.5140.5140.2	2	3.592	0.427	1	893.3	83.33333	R.RAPFDLFENR.K
HS90A_MOUSE	MK_SCX_44.5148.5148.3	3	3.336	0.187	1	589.6	47.22222	R.RAPFDLFENR.K
HS90A_MOUSE	MK_SCX_44.5828.5828.3	3	5.268	0.533	1	710.7	44.04762	K.TKPIWTRNPDDITNEEYGEFYK.S
HS90A_MOUSE	MK_SCX_45.5042.5042.3	3	3.46	0.207	1	533.7	37.5	K.LDSGKELHINLIPSK.Q
HS90A_MOUSE	MK_SCX_45.6410.6410.3	3	4.931	0.527	1	627.4	34.782608	R.YESLTDPSKLDSGKELHINLIPSK.Q
HS90A_MOUSE	MK_SCX_46.10451.10451.3	3	5.363	0.573	1	1818.1	53.333336	K.VILHLKEDQTEYLEER.R
HS90A_MOUSE	MK_SCX_47.4824.4824.3	3	4.129	0.505	1	740.7	48.333332	K.YIDQEELNKTKPIWTR.N

HS90A_MOUSE	MK_SCX_47.4875.4875.2	2	4.039	0.44	1	457.7	53.333336	K.YIDQEELNKTKPIWTR.N
HS90A_MOUSE	MK_SCX_48.4106.4106.3	3	4.007	0.371	1	1361.3	53.333336	K.IRYESLTDPSKLDSGK.E
HS90A_MOUSE	MK_SCX_48.5160.5160.3	3	4.048	0.558	1	1774.2	50	K.HLEINPDHSIIETLR.Q
HS90A_MOUSE	MK_SCX_49.5530.5530.2	2	4.865	0.407	1	1908.4	85.71429	K.HLEINPDHSIIETLR.Q
HS90A_MOUSE	MK_SCX_49.7148.7148.2	2	5.364	0.615	1	1855	81.25	K.HSQFIGYPITLFVEKER.D
HS90A_MOUSE	MK_SCX_49.7255.7255.3	3	5.511	0.565	1	1612.1	50	K.HSQFIGYPITLFVEKER.D
HS90A_MOUSE	MK_SCX_52.6408.6408.3	3	4.355	0.393	1	915.6	45	K.KHSQFIGYPITLFVEK.E
HS90A_MOUSE	MK_SCX_53.4428.4428.3	3	6.216	0.408	1	1873.3	57.352943	K.LDSGKELHINLIPSKQDR.T
HS90A_MOUSE	MK_SCX_53.4453.4453.2	2	4.777	0.426	1	1361.5	64.70589	K.LDSGKELHINLIPSKQDR.T
HS90A_MOUSE	MK_SCX_55.4455.4455.3	3	3.395	0.123	1	784.1	52.499996	R.RAPFDLFENRK.K
HS90A_MOUSE	MK_SCX_56.11523.11523.3	3	3.133	0.446	1	499.2	38.235294	K.KHSQFIGYPITLFVEKER.D
HS90A_MOUSE	MK_SCX_56.4681.4681.2	2	5.817	0.527	1	1993	80	K.KHLEINPDHSIIETLR.Q
HS90A_MOUSE	MK_SCX_56.4756.4756.3	3	4.116	0.512	1	656.6	46.666668	K.KHLEINPDHSIIETLR.Q
HS90B_MOUSE	MK_SCX_13.7070.7070.2	2	4.934	0.545	1	518.1	34.848484	K.LGLGIDEDEVTAEEPSAAVPDEIPPLEGDEDASR.M
HS90B_MOUSE	MK_SCX_18.4783.4783.2	2	5.568	0.55	1	2253.8	85.71429	R.NPDDITQEEYGEFYK.S
HS90B_MOUSE	MK_SCX_18.5849.5849.2	2	2.84	0.493	1	538	40	R.LVSSPCCIVTSTYGWTANM*ER.I
HS90B_MOUSE	MK_SCX_18.6558.6558.2	2	3.431	0.619	1	693.8	47.5	R.LVSSPCCIVTSTYGWTANMER.I
HS90B_MOUSE	MK_SCX_20_1.4549.4549.2	2	4.583	0.559	1	2066.8	87.5	R.TLTIVDTGIGM*TK.A
HS90B_MOUSE	MK_SCX_20_1.5503.5503.2	2	4.807	0.639	2	2393.4	87.5	R.TLTIVDTGIGMTK.A
HS90B_MOUSE	MK_SCX_2201.3770.3770.2	2	2.834	0.528	1	1046.2	88.88889	K.SIYYITGESK.E
HS90B_MOUSE	MK_SCX_23.7757.7757.3	3	3.915	0.465	2	582.4	31.25	R.TLTIVDTGIGM*TKADLINNLGTIAK.S
HS90B_MOUSE	MK_SCX_23.8419.8419.3	3	6.053	0.566	1	3097.2	48.958336	R.TLTIVDTGIGMTKADLINNLGTIAK.S
HS90B_MOUSE	MK_SCX_31.5272.5272.2	2	2.347	0.192	1	326	58.333332	K.ELKIDILPNPQER.T
HS90B_MOUSE	MK_SCX_32.4875.4875.2	2	6.134	0.65	1	2774.5	75	R.YHTSQSGDEMTSLSEYVSR.M
HS90B_MOUSE	MK_SCX_32.4885.4885.2	2	4.414	0.553	1	1150.8	75	K.SLTNDWEDHLAVK.H
HS90B_MOUSE	MK_SCX_33.3837.3837.2	2	4.267	0.563	1	2005.4	73.333336	K.AQALRDNSTMGYMMAK.K
HS90B_MOUSE	MK_SCX_33.3886.3886.3	3	3.535	0.429	1	1047.7	50	K.AQALRDNSTMGYMMAK.K
HS90B_MOUSE	MK_SCX_33.4061.4061.3	3	4.733	0.57	1	632.9	37.5	R.YHTSQSGDEM*TSLSEYVSR.M
HS90B_MOUSE	MK_SCX_33.5527.5527.3	3	4.045	0.575	1	852.9	43.055553	R.YHTSQSGDEMTSLSEYVSR.M
HS90B_MOUSE	MK_SCX_34.7039.7039.2	2	5.21	0.528	1	2244.1	85.71429	K.HSQFIGYPITLYLEK.E
HS90B_MOUSE	MK_SCX_34.7111.7111.3	3	4.506	0.473	1	1676.8	53.571426	K.HSQFIGYPITLYLEK.E
HS90B_MOUSE	MK_SCX_40.4033.4033.3	3	3.877	0.463	1	525.2	43.75	R.YESLTDPSKLDSGKELK.I
HS90B_MOUSE	MK_SCX_40.4034.4034.2	2	4.744	0.573	1	904.8	68.75	R.YESLTDPSKLDSGKELK.I
HS90B_MOUSE	MK_SCX_42.4847.4847.2	2	2.994	0.269	1	932.2	77.77778	R.RAPFDLFENK.K
HS90B_MOUSE	MK_SCX_42.6476.6476.3	3	5.451	0.613	1	1344.5	31.666666	K.YIDQEELNKTKPIWTRNPDDITQEEYGEFYK.S
HS90B_MOUSE	MK_SCX_43.5991.5991.3	3	5.509	0.477	1	995.3	46.42857	K.TKPIWTRNPDDITQEEYGEFYK.S
HS90B_MOUSE	MK_SCX_48.4728.4728.2	2	5.047	0.572	1	1647.3	78.57143	K.HLEINPDHPIVETLR.Q
HS90B_MOUSE	MK_SCX_48.4729.4729.3	3	4.496	0.47	1	1726.3	53.571426	K.HLEINPDHPIVETLR.Q
HS90B_MOUSE	MK_SCX_49.6853.6853.3	3	5.992	0.66	1	2791.5	53.125	K.HSQFIGYPITLYLEKER.E
HS90B_MOUSE	MK_SCX_49.6882.6882.2	2	5.437	0.644	1	1732	78.125	K.HSQFIGYPITLYLEKER.E
HS90B_MOUSE	MK_SCX_52.6131.6131.2	2	4.861	0.593	1	1463	76.666664	K.KHSQFIGYPITLYLEK.E
HS90B_MOUSE	MK_SCX_52.6172.6172.3	3	3.617	0.495	1	1131.8	43.333332	K.KHSQFIGYPITLYLEK.E
HS90B_MOUSE	MK_SCX_56.4175.4175.2	2	5.792	0.457	1	2061.2	76.666664	K.KHLEINPDHPIVETLR.Q
HS90B_MOUSE	MK_SCX_56.4225.4225.3	3	4.415	0.516	1	884.9	48.333332	K.KHLEINPDHPIVETLR.Q
HS90B_MOUSE	MK_SCX_56.6698.6698.3	3	3.219	0.461	1	449	41.17647	K.KHSQFIGYPITLYLEKER.E
HSC20_MOUSE	MK_SCX_18.6202.6202.2	2	3.971	0.607	1	617.7	64.70589	R.LADAQSEAAMEEIEATVR.A
HSC20_MOUSE	MK_SCX_31.5471.5471.3	3	3.487	0.472	1	462.5	31.944445	K.QKEFTDNINSAFEQGDFEK.A
HSP47_MOUSE	MK_SCX_14.11414.11414.2	2	4.489	0.523	1	826.4	44	K.DQAVENILLSPLVVASSLGLVSLGGK.A
HSP47_MOUSE	MK_SCX_14.11542.11542.3	3	6.585	0.605	1	2964.8	43	K.DQAVENILLSPLVVASSLGLVSLGGK.A
HSP47_MOUSE	MK_SCX_20_1.6755.6755.2	2	3.654	0.474	1	926.3	64.28571	R.LYGPSSVSFADDFVR.S
HSP47_MOUSE	MK_SCX_21.6966.6966.2	2	2.449	0.313	1	475.6	53.846157	R.STGLAFSLYQAMAK.D

HSP47_MOUSE	MK_SCX_32.4386.4386.3	3	3.184	0.254	1	747.6	52.499996	K.LQMVEMPLAHK.L
HSP47_MOUSE	MK_SCX_32.8157.8157.2	2	3.842	0.489	1	1030.8	75	K.LFYADHPFIFLVR.D
HSP47_MOUSE	MK_SCX_34.7106.7106.3	3	4.824	0.376	1	916.6	32.692307	R.SALQSINEWASQTTDGKLPEVTKDVER.T
HSP47_MOUSE	MK_SCX_50.4760.4760.2	2	5.231	0.416	1	2407.1	85.71429	K.LRDEEVHTGLGELLR.S
HSP47_MOUSE	MK_SCX_51.4090.4090.3	3	5.092	0.504	1	1306.9	51.785713	K.HLAGLGLTEAIDKNK.A
HSP47_MOUSE	MK_SCX_52.6165.6165.3	3	4.894	0.571	1	1581.4	39.285713	K.AVLSAEKLRDEEVHTGLGELLR.S
HSP47_MOUSE	MK_SCX_54.4842.4842.3	3	4.038	0.5	1	748.3	39.473686	K.HLAGLGLTEAIDKNKADLSR.M
HSP72_MOUSE	MK_SCX_2201.5734.5734.2	2	3.198	0.387	1	968.9	77.77778	R.FEELNADLFR.G
HSP72_MOUSE	MK_SCX_26.12319.12319.3	3	3.153	0.222	1	439.3	25	-.MSARGPAIGIDLGTTYSCVGVFQHGK.V
HSP72_MOUSE	MK_SCX_28.5327.5327.2	2	5.755	0.53	1	1292.9	75	R.IINEPTAAAIAYGLDKK.G
HSP72_MOUSE	MK_SCX_31.3242.3242.3	3	3.257	0.529	1	374.4	40	K.STAGDTHLGGEDFDNR.M
HSP72_MOUSE	MK_SCX_31.3250.3250.2	2	4.694	0.547	1	1539.3	73.333336	K.STAGDTHLGGEDFDNR.M
HSP72_MOUSE	MK_SCX_33.5358.5358.2	2	3.596	0.304	1	880.4	70.83333	K.LLQDFFNGKELNK.S
HSP72_MOUSE	MK_SCX_39.6419.6419.3	3	4.685	0.369	1	1504.7	56.81818	R.ARFEELNADLFR.G
HSP72_MOUSE	MK_SCX_39.6493.6493.2	2	3.065	0.463	1	903.1	77.27273	R.ARFEELNADLFR.G
HSP72_MOUSE	MK_SCX_51.4672.4672.2	2	2.651	0.183	1	691.3	57.692307	K.QKELERVCNPIISK.L
HSP74_MOUSE	MK_SCX_17.4839.4839.2	2	6.017	0.535	1	1864.8	75	K.LEDTENWLYEDGEDQPK.Q
HSP74_MOUSE	MK_SCX_18.7548.7548.2	2	6.278	0.6	1	2085.2	82.35294	K.SNLAYDIVQLPTGLTGIK.V
HSP74_MOUSE	MK_SCX_18.8131.8131.2	2	4.327	0.592	1	402.4	53.571426	R.EFSITDVVPYPISLR.W
HSP74_MOUSE	MK_SCX_19.4008.4008.2	2	4.168	0.429	1	993.9	73.07692	K.ELSTTLNADEAVTR.G
HSP74_MOUSE	MK_SCX_2201.3771.3771.2	2	2.144	0.139	1	488.9	91.66667	K.LFEELGK.Q
HSP74_MOUSE	MK_SCX_2201.4047.4047.2	2	2.355	0.179	1	586	70	K.NAVEEYVYEM*R.D
HSP74_MOUSE	MK_SCX_2201.4988.4988.2	2	2.947	0.462	1	785	70	K.NAVEEYVYEMR.D
HSP74_MOUSE	MK_SCX_23.8125.8125.3	3	5.089	0.425	1	1157.9	33.62069	R.KEPFTLEAYYSSPQDLPYPDPAIAQFSVQK.V
HSP74_MOUSE	MK_SCX_24.3482.3482.3	3	5.909	0.446	1	1350.7	35.869564	K.MQVDQEEPHTEEQQQQPQTPAENK.A
HSP74_MOUSE	MK_SCX_46.3514.3514.2	2	5.43	0.49	1	2155.1	73.333336	K.NKEDQYEHLDAADVTK.V
HSP74_MOUSE	MK_SCX_46.3553.3553.3	3	4.811	0.483	1	1104.2	45	K.NKEDQYEHLDAADVTK.V
HSP74_MOUSE	MK_SCX_52.4144.4144.3	3	4.211	0.398	1	1280.4	36.904762	K.VISSFKNKEDQYEHLDAADVTK.V
HSP7C_MOUSE	MK_SCX_15.4656.4656.2	2	2.755	0.431	1	1327.9	85	K.NSLESYAFNM*K.A
HSP7C_MOUSE	MK_SCX_17.7801.7801.3	3	4.405	0.524	1	597.6	30.681818	K.SINPDEAVAYGAAVQAAILSGDK.S
HSP7C_MOUSE	MK_SCX_17.7913.7913.2	2	5.714	0.691	1	2268.5	65.909096	K.SINPDEAVAYGAAVQAAILSGDK.S
HSP7C_MOUSE	MK_SCX_19.4862.4862.2	2	2.189	0.334	1	383.3	53.846157	K.SFYPEEVSSM*VLTK.M
HSP7C_MOUSE	MK_SCX_19.5124.5124.2	2	4.051	0.5	1	586.6	61.764706	K.TVTNAVVTVPAYFNDSQR.Q
HSP7C_MOUSE	MK_SCX_19.5829.5829.2	2	5.296	0.652	1	1212.9	88.46153	K.SFYPEEVSSMVLTK.M
HSP7C_MOUSE	MK_SCX_20_1.3872.3872.2	2	4.119	0.602	1	1390.9	90	R.FDDAVVQSDMK.H
HSP7C_MOUSE	MK_SCX_20_1.4074.4074.2	2	3.794	0.398	1	1854	75	K.NQVAM*NPTNTVFDAK.R
HSP7C_MOUSE	MK_SCX_20_1.4617.4617.2	2	4.422	0.552	1	1836.8	82.14286	K.NQVAMNPTNTVFDAK.R
HSP7C_MOUSE	MK_SCX_23.4632.4632.1	1	3.086	0.41	1	333.4	70	K.NSLESYAFNMK.A
HSP7C_MOUSE	MK_SCX_23.4658.4658.2	2	4.313	0.407	1	1463.6	85	K.NSLESYAFNMK.A
HSP7C_MOUSE	MK_SCX_23.6499.6499.3	3	6.244	0.633	1	1544	33.333336	R.NTTIPTKQTQTFTTYSDNQPGVLIQVYEGER.A
HSP7C_MOUSE	MK_SCX_25.7028.7028.2	2	4.503	0.649	1	1263.1	70.588234	K.DNNLLGKFELTGIPPAPR.G
HSP7C_MOUSE	MK_SCX_26.5132.5132.3	3	3.385	0.37	1	730.2	29.761904	R.LIGDAAKNQVAMNPTNTVFDAK.R
HSP7C_MOUSE	MK_SCX_33.4172.4172.2	2	4.941	0.463	1	1223.5	73.333336	K.NQVAMNPTNTVFDAKR.L
HSP7C_MOUSE	MK_SCX_35.4052.4052.2	2	4.997	0.609	1	2458.5	88.46153	K.SQIHDIVLVGGSTR.I
HSP7C_MOUSE	MK_SCX_37.3553.3553.2	2	3.925	0.556	1	1180.5	81.818184	R.RFDDAVVQSDMK.H
HSP7C_MOUSE	MK_SCX_38.2355.2355.2	2	3.08	0.374	1	940.3	83.33333	K.VQVEYKGETK.S
HSP7C_MOUSE	MK_SCX_38.3832.3832.3	3	3.512	0.361	1	1501	57.5	K.MKEIAEAYLGK.T
HSP7C_MOUSE	MK_SCX_38.3841.3841.2	2	4.219	0.442	1	1369.3	90	K.MKEIAEAYLGK.T
HSP7C_MOUSE	MK_SCX_40.4500.4500.3	3	3.332	0.454	1	594.4	52.77778	R.MVNHFIAEFK.R
HSP7C_MOUSE	MK_SCX_40.7228.7228.3	3	3.72	0.521	1	760.3	34.523808	R.AMTKDNNLLGKFELTGIPPAPR.G
HSP7C_MOUSE	MK_SCX_41.4011.4011.3	3	3.038	0.176	1	522.7	52.77778	R.M*VNHFIAEFK.R

HSP7C_MOUSE	MK_SCX_41.4315.4315.2	2	3.504	0.494	1	1265	88.88889	R.MVNHFIAEFK.R
HSP7C_MOUSE	MK_SCX_45.4817.4817.3	3	5.493	0.601	1	1665.8	51.5625	K.LDKSQIHDIVLVGGSTR.I
HSP7C_MOUSE	MK_SCX_45.4839.4839.2	2	5.205	0.372	1	1755.8	75	K.LDKSQIHDIVLVGGSTR.I
HSP7C_MOUSE	MK_SCX_46.4632.4632.3	3	3.298	0.475	1	855.5	40.625	R.DKVSSKNSLESYAFNMK.A
HSP7C_MOUSE	MK_SCX_48.7150.7150.3	3	4.288	0.416	1	1691.8	39.583336	R.FDDAVVQSDMKHWPFMVVNDAGRPK.V
HSP7C_MOUSE	MK_SCX_53.4392.4392.3	3	3.696	0.34	1	1176.2	51.923077	K.HWPFM*VVNDAGRPK.V
HSP7C_MOUSE	MK_SCX_53.4395.4395.2	2	3.424	0.525	1	708	73.07692	K.HWPFM*VVNDAGRPK.V
HSP7C_MOUSE	MK_SCX_53.4832.4832.2	2	3.883	0.596	1	910.8	84.61539	K.HWPFMVVNDAGRPK.V
HSP7C_MOUSE	MK_SCX_53.4844.4844.3	3	4.415	0.511	1	1268.5	51.923077	K.HWPFMVVNDAGRPK.V
HSP7C_MOUSE	MK_SCX_54.4000.4000.3	3	3.229	0.439	1	541.5	45	R.MVNHFIAEFKR.K
HSPB1_MOUSE	MK_SCX_14.7924.7924.2	2	5.107	0.444	1	572.5	44.827587	K.YTLPPGVDPTLVSSSLSPEGTLTVEAPLPK.A
HSPB1_MOUSE	MK_SCX_2201.8110.8110.3	3	5.791	0.55	1	1811.7	34.166668	R.KYTLPPGVDPTLVSSSLSPEGTLTVEAPLPK.A
HSPB1_MOUSE	MK_SCX_2201.8156.8156.2	2	5.182	0.54	1	721.9	41.666664	R.KYTLPPGVDPTLVSSSLSPEGTLTVEAPLPK.A
HSPB1_MOUSE	MK_SCX_23.4931.4931.2	2	2.88	0.362	1	1050.6	83.33333	R.LFDQAFGVPR.L
HSPB1_MOUSE	MK_SCX_27.6101.6101.3	3	3.37	0.228	1	457	36.666668	R.VSLDVNHFAPEELTVK.T
HSPB1_MOUSE	MK_SCX_28.5994.5994.2	2	4.143	0.543	1	1394.2	66.66667	R.VSLDVNHFAPEELTVK.T
HTRA2_MOUSE	MK_SCX_25.8666.8666.3	3	3.918	0.407	1	481.1	27.000002	R.VRLPSGDTYEAMVTAVDPVADIATLR.I
HUWE1_MOUSE	MK_SCX_17.8457.8457.2	2	3.882	0.557	1	843.4	50	R.LLGPSAAADILQLSSSLPLQSR.G
HUWE1_MOUSE	MK_SCX_2201.8760.8760.3	3	3.665	0.438	1	310.4	29.807693	R.ELAQNASSDTPMDPVTFIQTLPSDLRR.S
HUWE1_MOUSE	MK_SCX_37.6753.6753.2	2	2.732	0.559	1	703.9	80	R.HVLDTLIQLAK.V
HXK1_MOUSE	MK_SCX_2201.3713.3713.2	2	2.654	0.308	1	576.8	65	R.TTVGVDGSLYK.M
HXK1_MOUSE	MK_SCX_43.4254.4254.3	3	6.033	0.613	1	1621.3	47.61905	R.GKFTTSDVAAIETDKEGVQNAK.E
HYES_MOUSE	MK_SCX_16.5551.5551.2	2	3.713	0.491	1	442.7	42.105263	R.AVASLNTPFM*PPDPDVSPM*K.V
HYES_MOUSE	MK_SCX_16.5920.5920.2	2	4.575	0.561	1	317.1	42.105263	R.AVASLNTPFMPPDPDVSPM*K.V
HYES_MOUSE	MK_SCX_16.5997.5997.2	2	3.689	0.322	1	418	39.473686	R.AVASLNTPFM*PPDPDVSPMK.V
HYES_MOUSE	MK_SCX_16.6307.6307.2	2	5.287	0.557	1	751.4	60.526318	R.AVASLNTPFMPPDPDVSPMK.V
HYES_MOUSE	MK_SCX_16.8012.8012.2	2	4.872	0.588	1	434	43.18182	R.SIPVFNYQLYFQEPGVAEAELEK.N
HYES_MOUSE	MK_SCX_17.5522.5522.2	2	4.865	0.608	1	1184.3	61.11111	K.ATEIGGILVNTPEDPNLSK.I
HYES_MOUSE	MK_SCX_17.9433.9433.2	2	5.641	0.524	1	1301.7	71.05263	R.VAAFDLDGVLALPSIAGAFR.R
HYES_MOUSE	MK_SCX_19.8442.8442.2	2	2.258	0.201	1	530	57.14286	K.ITTEEEIEFYIQQFK.K
HYES_MOUSE	MK_SCX_27.16299.16299.3	3	3.595	0.472	1	779.7	35	R.VAAFDLDGVLALPSIAGAFRR.S
IAG2_MOUSE	MK_SCX_11.13994.13994.2	2	2.02	0.237	1	434.2	38.88889	K.APPRNYSVVVMFTALQLHR.Q
IAG2_MOUSE	MK_SCX_52.4851.4851.3	3	3.026	0.462	1	437.6	33.92857	K.VSQLMEWANKRPVIR.M
IBP7_MOUSE	MK_SCX_11.7880.7880.2	2	2.127	0.163	1	315.9	32.608696	R.DACGCCPVCARGEGEPCGGGAAGR.G
IBP7_MOUSE	MK_SCX_27.6273.6273.2	2	2.385	0.189	1	590.7	58.333332	R.ENLAIQTRGGPEK.H
IBP7_MOUSE	MK_SCX_30.4438.4438.2	2	3.917	0.357	1	692	63.333332	R.TELLPGDRENLAIQTR.G
IBP7_MOUSE	MK_SCX_45.5764.5764.3	3	4.265	0.4	1	839.6	36.11111	R.GGPEKHEVTGWVLVSPLSK.E
ICAL_MOUSE	MK_SCX_18.6866.6866.2	2	4.976	0.486	1	1199.3	70.588234	K.GVVPEDAVETLAGSLGTR.E
ICAL_MOUSE	MK_SCX_20_1.3015.3015.2	2	3.909	0.507	1	527.8	60.714287	K.SNDTSQTPPGETVPR.A
ICAL_MOUSE	MK_SCX_26.7561.7561.3	3	3.484	0.439	1	874.4	31.818182	K.LASLKGVVPEDAVETLAGSLGTR.E
ICAL_MOUSE	MK_SCX_29.2994.2994.3	3	3.888	0.473	1	1140.8	44.736843	K.VTASSAATSKSPSMSTTETK.A
ICAL_MOUSE	MK_SCX_45.3218.3218.3	3	3.522	0.314	1	402.6	30.952381	K.AASLGSSQPSRPHVGEAATATK.V
ICT1_MOUSE	MK_SCX_21.3716.3716.2	2	3.764	0.49	1	1537.8	81.818184	K.AGELVLTSESSR.Y
ICT1_MOUSE	MK_SCX_36.6272.6272.3	3	3.053	0.142	1	1508.3	51.923077	R.FHLASADWIEEPVR.Q
ICT1_MOUSE	MK_SCX_50.5428.5428.2	2	2.536	0.336	1	489.1	46.666668	R.FHLASADWIEEPVRQK.I
ICT1_MOUSE	MK_SCX_50.5449.5449.3	3	3.963	0.4	1	1127.4	51.666664	R.FHLASADWIEEPVRQK.I
IDH3A_MOUSE	MK_SCX_21.5035.5035.2	2	2.667	0.149	1	589.4	77.27273	K.TPYTDVNIVTIR.E
IDH3A_MOUSE	MK_SCX_23.5146.5146.2	2	4.182	0.558	1	1629	94.44444	R.IAEFAFEYAR.N
IDH3A_MOUSE	MK_SCX_24.8138.8138.3	3	3.591	0.327	1	417.3	26.136362	R.ENTEGEYSGIEHVIVDGVVQSIK.L
IDH3A_MOUSE	MK_SCX_25.4306.4306.2	2	2.113	0.245	1	399	64.28571	K.WMIPPEAK.E
IDH3A_MOUSE	MK_SCX_33.5054.5054.3	3	3.694	0.451	1	644.7	42.857143	K.TPIAAGHPSMNLLLR.K

IDH3A_MOUSE	MK_SCX_33.5065.5065.2	2	3.07	0.581	1	402	50	K.TPIAAGHPSMNLLLR.K
IDH3A_MOUSE	MK_SCX_33.5169.5169.2	2	3.566	0.275	1	1038.4	76.92308	K.IFDAAKAPIQWEER.N
IDH3A_MOUSE	MK_SCX_33.5203.5203.3	3	3.971	0.467	1	693.5	51.923077	K.IFDAAKAPIQWEER.N
IDH3A_MOUSE	MK_SCX_44.4849.4849.2	2	3.328	0.455	1	580.1	75	K.RIAEFAFEYAR.N
IDH3A_MOUSE	MK_SCX_45.5113.5113.3	3	3.352	0.628	1	327.3	30.555555	K.GPLKTPIAAGHPSM*NLLLR.K
IDH3A_MOUSE	MK_SCX_45.5884.5884.3	3	3.883	0.577	1	672.1	34.72222	K.GPLKTPIAAGHPSMNLLLR.K
IDH3A_MOUSE	MK_SCX_45.5931.5931.2	2	4.952	0.646	1	1528.8	75	K.GPLKTPIAAGHPSMNLLLR.K
IDH3A_MOUSE	MK_SCX_52.5947.5947.3	3	5.4	0.58	1	1651	47.727272	K.MGLKGPLKTPIAAGHPSMNLLLR.K
IDH3G_MOUSE	MK_SCX_21.7332.7332.3	3	7.835	0.686	1	2957.8	37.5	K.AVLASMDNENMHTPDIGGQGTTSQAIQDIIR.H
IDH3G_MOUSE	MK_SCX_24.6502.6502.3	3	3.319	0.483	1	962.1	34.090908	R.ENTEGEYSSLEHESVAGVVESLK.I
IDH3G_MOUSE	MK_SCX_24.6546.6546.2	2	4.816	0.571	1	781.9	45.454548	R.ENTEGEYSSLEHESVAGVVESLK.I
IDH3G_MOUSE	MK_SCX_34.6836.6836.3	3	7.648	0.606	1	2220.1	33.870968	R.KAVLASMDNENMHTPDIGGQGTTSQAIQDIIR.H
IDH3G_MOUSE	MK_SCX_42.6625.6625.3	3	5.917	0.549	1	2504.4	50	R.HTVTMIPGDGIGPELMLHVK.S
IDH3G_MOUSE	MK_SCX_43.6836.6836.2	2	5.331	0.612	1	937.7	68.42105	R.HTVTMIPGDGIGPELMLHVK.S
IDHC_MOUSE	MK_SCX_19.4007.4007.2	2	4.802	0.616	1	2220.9	83.33333	K.IQGGSVVEMQGDEMTR.I
IDHC_MOUSE	MK_SCX_20_1.3175.3175.3	3	5.803	0.499	1	2731.5	58.333332	K.IQGGSVVEM*QGDEM*TR.I
IDHC_MOUSE	MK_SCX_20_1.3179.3179.2	2	5.006	0.506	1	1950.8	76.666664	K.IQGGSVVEM*QGDEM*TR.I
IDHC_MOUSE	MK_SCX_20_1.3346.3346.2	2	3.433	0.532	1	1257.8	80	R.LIDDM*VAQAM*K.S
IDHC_MOUSE	MK_SCX_20_1.3660.3660.2	2	5.252	0.34	1	1730.4	73.333336	K.IQGGSVVEMQGDEM*TR.I
IDHC_MOUSE	MK_SCX_20_1.3682.3682.3	3	3.444	0.251	1	800.5	41.666664	K.IQGGSVVEMQGDEM*TR.I
IDHC_MOUSE	MK_SCX_20_1.3733.3733.3	3	4.635	0.453	1	1298.6	50	K.IQGGSVVEM*QGDEMTR.I
IDHC_MOUSE	MK_SCX_20_1.4159.4159.2	2	4.406	0.234	1	1401.7	90	R.LIDDMVAQAM*K.S
IDHC_MOUSE	MK_SCX_20_1.5321.5321.2	2	4.425	0.459	1	1342.3	90	R.LIDDMVAQAMK.S
IDHC_MOUSE	MK_SCX_20_1.5784.5784.2	2	4.933	0.454	1	1473.3	81.818184	R.SDYLNTFEFM*DK.L
IDHC_MOUSE	MK_SCX_20_1.5787.5787.2	2	4.368	0.498	1	1515.2	90.909096	K.LDNNTELSFFAK.A
IDHC_MOUSE	MK_SCX_20_1.6879.6879.2	2	4.779	0.561	1	1429.4	81.818184	R.SDYLNTFEFMDK.L
IDHC_MOUSE	MK_SCX_20_1.8599.8599.2	2	4.926	0.587	1	1426.6	71.875	K.GQETSTNPIASIFAWSR.G
IDHC_MOUSE	MK_SCX_21.4116.4116.2	2	2.555	0.302	1	344.8	60.000004	R.ATDFVVPGPGK.V
IDHC_MOUSE	MK_SCX_2201.3302.3302.2	2	2.334	0.331	1	636.9	85.71429	K.VEITYTPK.D
IDHC_MOUSE	MK_SCX_23.12474.12474.3	3	4.842	0.461	1	707	46.05263	K.LILPYVELDLHSYDLGIENR.D
IDHC_MOUSE	MK_SCX_23.8675.8675.2	2	6.008	0.512	1	2232.8	65.789474	K.LILPYVELDLHSYDLGIENR.D
IDHC_MOUSE	MK_SCX_24.5653.5653.2	2	2.825	0.159	1	376.9	91.66667	R.IIWELIK.E
IDHC_MOUSE	MK_SCX_25.3944.3944.2	2	4.667	0.449	1	1860.8	70	R.DATNDQVTKDAAEAIK.K
IDHC_MOUSE	MK_SCX_25.3983.3983.3	3	3.353	0.51	1	791.9	48.333332	R.DATNDQVTKDAAEAIK.K
IDHC_MOUSE	MK_SCX_25.4696.4696.2	2	2.251	0.135	1	669.5	87.5	R.NILGGTVFR.E
IDHC_MOUSE	MK_SCX_25.6184.6184.3	3	4.092	0.49	1	1284.4	34.090908	K.VTYMVHDFEEGGGVAMGMYNQDK.S
IDHC_MOUSE	MK_SCX_26.5536.5536.3	3	4.253	0.574	1	1136	43.055553	R.ATDFVVPGPGKVEITYTPK.D
IDHC_MOUSE	MK_SCX_26.5682.5682.2	2	3.933	0.53	1	340.5	50	R.ATDFVVPGPGKVEITYTPK.D
IDHC_MOUSE	MK_SCX_27.12350.12350.3	3	3.313	0.493	1	320.9	30.882353	R.SDYLNTFEFMDKLGENLK.A
IDHC_MOUSE	MK_SCX_27.16420.16420.2	2	4.203	0.494	1	789.6	55.88235	R.SDYLNTFEFMDKLGENLK.A
IDHC_MOUSE	MK_SCX_29.16382.16382.3	3	4.61	0.577	1	867.6	58.333332	K.SIEDFAHSSFQMALSK.G
IDHC_MOUSE	MK_SCX_29.5935.5935.2	2	5.169	0.519	1	710.9	70	K.SIEDFAHSSFQM*ALSK.G
IDHC_MOUSE	MK_SCX_29.6079.6079.3	3	4.502	0.546	1	449.7	50	K.SIEDFAHSSFQM*ALSK.G
IDHC_MOUSE	MK_SCX_29.6687.6687.2	2	5.281	0.421	1	2385.6	80	K.SIEDFAHSSFQMALSK.G
IDHC_MOUSE	MK_SCX_31.7543.7543.3	3	3.458	0.296	1	754.6	35	R.M*YQKGQETSTNPIASIFAWSR.G
IDHC_MOUSE	MK_SCX_31.7578.7578.3	3	5.662	0.621	1	1949.7	46.25	R.MYQKGQETSTNPIASIFAWSR.G
IDHC_MOUSE	MK_SCX_31.7580.7580.2	2	5.976	0.645	1	3904.9	77.5	R.MYQKGQETSTNPIASIFAWSR.G
IDHC_MOUSE	MK_SCX_33.5987.5987.3	3	3.971	0.476	1	996.2	60.416668	R.LVTGWVKPIIIGR.H
IDHC_MOUSE	MK_SCX_33.5989.5989.2	2	4.214	0.44	1	1168.1	79.16667	R.LVTGWVKPIIIGR.H
IDHC_MOUSE	MK_SCX_34.3439.3439.3	3	5.426	0.291	1	2208.5	56.25	R.KIQGGSVVEMQGDEM*TR.I
IDHC_MOUSE	MK_SCX_34.3492.3492.2	2	4.702	0.222	1	679.9	65.625	R.KIQGGSVVEM*QGDEMTR.I

IDHC_MOUSE	MK_SCX_34.5383.5383.3	3	5.559	0.491	1	452.4	32.608696	R.ATDFVVPGPGKVEITYTPKDGTQK.V
IDHC_MOUSE	MK_SCX_35.12386.12386.2	2	3.838	0.464	1	980.4	73.07692	R.AKLDNNTELSFFAK.A
IDHC_MOUSE	MK_SCX_35.16383.16383.3	3	3.997	0.329	1	758.8	44.230766	R.AKLDNNTELSFFAK.A
IDHC_MOUSE	MK_SCX_35.2520.2520.2	2	3.996	0.429	1	1122.2	70.83333	K.TVEAEAAHGTVTR.H
IDHC_MOUSE	MK_SCX_35.3369.3369.2	2	4.869	0.136	1	887.6	68.75	R.KIQGGSVVEMQGDEM*TR.I
IDHC_MOUSE	MK_SCX_35.3421.3421.3	3	5.635	0.225	1	2967.6	56.25	R.KIQGGSVVEM*QGDEMTR.I
IDHC_MOUSE	MK_SCX_35.3789.3789.2	2	5.624	0.507	1	2277.6	75	R.KIQGGSVVEMQGDEMTR.I
IDHC_MOUSE	MK_SCX_35.3803.3803.3	3	5.548	0.571	1	1958.5	54.6875	R.KIQGGSVVEMQGDEMTR.I
IDHC_MOUSE	MK_SCX_37.3657.3657.3	3	5.159	0.477	1	1592.9	54.6875	R.DATNDQVTKDAAEAIKK.Y
IDHC_MOUSE	MK_SCX_37.6636.6636.2	2	3.578	0.41	1	1290	85	R.FKDIFQEIYDK.K
IDHC_MOUSE	MK_SCX_37.6675.6675.3	3	4	0.306	1	2065.9	62.5	R.FKDIFQEIYDK.K
IDHC_MOUSE	MK_SCX_38.5447.5447.2	2	2.575	0.238	1	600.9	81.25	R.IIWELIKEK.L
IDHC_MOUSE	MK_SCX_46.3751.3751.3	3	3.045	0.439	1	633.9	46.153847	K.DAAEAIKKYNVGVK.C
IDHC_MOUSE	MK_SCX_49.1673.1673.2	2	2.61	0.372	1	709.8	92.85714	R.HAYGDQYR.A
IDHC_MOUSE	MK_SCX_51.5414.5414.3	3	4.725	0.28	1	1670.9	61.363636	R.FKDIFQEIYDKK.Y
IDHC_MOUSE	MK_SCX_51.5538.5538.2	2	5.02	0.334	1	1495.4	86.36364	R.FKDIFQEIYDKK.Y
IDHP_MOUSE	MK_SCX_19.4565.4565.2	2	4.737	0.542	1	2042.6	87.5	K.DLAGCIHGLSNVK.L
IDHP_MOUSE	MK_SCX_20_1.5224.5224.2	2	4.562	0.381	1	1175.1	90	R.LIDDM*VAQVLK.S
IDHP_MOUSE	MK_SCX_20_1.6778.6778.2	2	4.105	0.383	1	1372.8	90	R.LIDDMVAQVLK.S
IDHP_MOUSE	MK_SCX_2201.3182.3182.2	2	2.266	0.43	1	1004.3	92.85714	K.ATDFVVDR.A
IDHP_MOUSE	MK_SCX_23.4955.4955.2	2	3.15	0.461	1	1285.5	93.75	K.YFDLGLPNR.D
IDHP_MOUSE	MK_SCX_25.4932.4932.2	2	2.286	0.225	1	663.5	92.85714	K.WPLYLSTK.N
IDHP_MOUSE	MK_SCX_25.5807.5807.2	2	2.639	0.169	1	387.9	91.66667	R.IIWQFIK.E
IDHP_MOUSE	MK_SCX_29.3372.3372.3	3	4.428	0.373	1	2154.1	58.928574	K.VEKPVVEM*DGDEMTR.I
IDHP_MOUSE	MK_SCX_30.3800.3800.2	2	4.577	0.608	1	1409.3	85.71429	K.VEKPVVEMDGDEMTR.I
IDHP_MOUSE	MK_SCX_30.3811.3811.3	3	5.178	0.538	1	3077.1	62.5	K.VEKPVVEMDGDEMTR.I
IDHP_MOUSE	MK_SCX_30.6934.6934.2	2	5.131	0.474	1	1945.3	73.333336	K.LNEHFLNTTDFLDTIK.S
IDHP_MOUSE	MK_SCX_30.6941.6941.3	3	5.04	0.472	1	1024.4	48.333332	K.LNEHFLNTTDFLDTIK.S
IDHP_MOUSE	MK_SCX_33.1796.1796.2	2	3.252	0.391	1	700.2	80	K.LILPHVDVQLK.Y
IDHP_MOUSE	MK_SCX_35.2936.2936.2	2	4.004	0.603	1	1111.6	75	K.TIEAEAAHGTVTR.H
IDHP_MOUSE	MK_SCX_37.8333.8333.3	3	3.2	0.136	1	2502.7	67.5	R.FKDIFQEIFDK.H
IDHP_MOUSE	MK_SCX_37.8372.8372.2	2	2.751	0.245	1	1568.3	90	R.FKDIFQEIFDK.H
IDHP_MOUSE	MK_SCX_40.10679.10679.3	3	3.969	0.437	1	426.5	32.5	K.LNEHFLNTTDFLDTIKSNLDR.A
IDHP_MOUSE	MK_SCX_45.3380.3380.3	3	3.957	0.484	1	683	46.875	R.IKVEKPVVEM*DGDEM*TR.I
IDHP_MOUSE	MK_SCX_45.3828.3828.3	3	4.741	0.302	1	697.8	43.75	R.IKVEKPVVEMDGDEM*TR.I
IDHP_MOUSE	MK_SCX_45.3836.3836.2	2	4.402	0.362	1	1168.1	71.875	R.IKVEKPVVEMDGDEM*TR.I
IDHP_MOUSE	MK_SCX_45.4331.4331.2	2	5.278	0.542	1	1357.1	81.25	R.IKVEKPVVEMDGDEMTR.I
IDHP_MOUSE	MK_SCX_46.4310.4310.3	3	4.502	0.367	1	661.2	43.75	R.IKVEKPVVEMDGDEMTR.I
IDHP_MOUSE	MK_SCX_48.5458.5458.2	2	3.726	0.525	1	1336.2	83.33333	K.EKLILPHVDVQLK.Y
IDHP_MOUSE	MK_SCX_56.11487.11487.2	2	3.179	0.316	1	625.8	61.538464	R.FKDIFQEIFDKHYK.T
IDHP_MOUSE	MK_SCX_56.6883.6883.3	3	3.682	0.361	1	556	44.230766	R.FKDIFQEIFDKHYK.T
IDHP_MOUSE	MK_SCX_58.11798.11798.3	3	4.333	0.595	1	1737.3	58.333332	R.HAHGDQYKATDFVVDR.A
IDHP_MOUSE	MK_SCX_58.12311.12311.2	2	4.045	0.567	1	1040.6	66.66667	R.HAHGDQYKATDFVVDR.A
IDI1_MOUSE	MK_SCX_16.8111.8111.2	2	4.939	0.668	1	729.6	57.894737	K.AELGIPLEEVDLNEMDYLTR.I
IDI1_MOUSE	MK_SCX_25.8552.8552.3	3	5.075	0.532	1	1743.5	41.666664	R.LKAELGIPLEEVDLNEMDYLTR.I
IF2A_MOUSE	MK_SCX_17.3308.3308.2	2	5.092	0.609	1	1508.7	75	R.ENAEVDGDDDAEEMEAK.A
IF2A_MOUSE	MK_SCX_19.3238.3238.2	2	3.66	0.525	1	1246.3	90.909096	K.VVTDTDETELAR.Q
IF2A_MOUSE	MK_SCX_19.6962.6962.2	2	4.121	0.351	1	1528.4	75	R.TEGLSVLNQAMAVIK.E
IF2A_MOUSE	MK_SCX_29.3095.3095.3	3	6.535	0.519	1	3382.3	47.368423	R.LERENAEVDGDDDAEEM*EAK.A
IF2A_MOUSE	MK_SCX_36.3558.3558.3	3	4.977	0.517	1	1766.5	39.473686	R.LERENAEVDGDDDAEEMEAK.A
IF2A_MOUSE	MK_SCX_43.6994.6994.3	3	5.283	0.617	1	1641	51.315792	R.HVAEVLEYTKDEQLESLFQR.T

IF2A_MOUSE	MK_SCX_47.6261.6261.3	3	3.359	0.268	1	635.9	33.82353	R.FYQHKFPEVEDVVMVNVR.S
IF2A_MOUSE	MK_SCX_56.3412.3412.3	3	3.599	0.357	1	1006.8	50	K.YKRPGYGAYDAFK.H
IF2B_MOUSE	MK_SCX_15.5383.5383.2	2	4.348	0.629	1	804.6	52.380955	K.IESDAQEPAEPEDDLDIM*LGNK.K
IF2B_MOUSE	MK_SCX_15.6172.6172.2	2	4.89	0.581	1	951.4	59.523808	K.IESDAQEPAEPEDDLDIMLGNK.K
IF2B_MOUSE	MK_SCX_15.6590.6590.2	2	3.952	0.495	1	1367.3	75	K.DASDDLDDLNFFNQK.K
IF2B_MOUSE	MK_SCX_19.4816.4816.2	2	3.012	0.325	1	717.9	83.33333	R.DYTYEELLNR.V
IF2B_MOUSE	MK_SCX_23.3435.3435.3	3	3.316	0.468	1	523.9	35.526314	K.EVEPEPTEEKDVDADEEDSR.K
IF2B_MOUSE	MK_SCX_27.7259.7259.3	3	3.826	0.431	1	1183.5	55.35714	K.IFDIDEAEEAIKDVK.I
IF2B_MOUSE	MK_SCX_27.7272.7272.2	2	3.825	0.425	1	2029.4	82.14286	K.IFDIDEAEEAIKDVK.I
IF2B_MOUSE	MK_SCX_44.3874.3874.3	3	6.61	0.432	1	1888.3	42.045452	K.KKPFMLDEEGDAQTEETQPSETK.E
IF2B_MOUSE	MK_SCX_44.6640.6640.2	2	5.113	0.509	1	2844	80	K.KIFDIDEAEEAIKDVK.I
IF2B_MOUSE	MK_SCX_44.6663.6663.3	3	4.91	0.482	1	833.8	50	K.KIFDIDEAEEAIKDVK.I
IF2B_MOUSE	MK_SCX_49.5809.5809.3	3	4.145	0.45	1	1241.1	46.875	R.KKDASDDLDDLNFFNQK.K
IF2B2_MOUSE	MK_SCX_49.4176.4176.2	2	3.498	0.202	1	899.6	69.230774	R.M*ILEIMQKEADETK.L
IF2M_MOUSE	MK_SCX_15.5947.5947.2	2	3.754	0.474	1	928.6	59.375	R.DLPSAGDEILEVESEPR.A
IF31_MOUSE	MK_SCX_18.5706.5706.2	2	3.397	0.381	1	686.4	55.263157	K.ETFGVNNTVYGIDAMNPSSR.D
IF31_MOUSE	MK_SCX_32.4825.4825.2	2	2.709	0.296	1	632.4	66.66667	K.VLTPEEQLADKLR.L
IF31_MOUSE	MK_SCX_34.5840.5840.3	3	5.506	0.59	1	1076.8	42.5	R.LEEPEESKVLTPEEQLADKLR.L
IF32_MOUSE	MK_SCX_2201.3259.3259.2	2	3.559	0.281	1	1453.2	93.75	K.SGEVLVNVK.E
IF32_MOUSE	MK_SCX_26.4268.4268.2	2	3.261	0.569	1	609.2	60.714287	R.DMTMFVTASKDNTAK.L
IF32_MOUSE	MK_SCX_51.4274.4274.3	3	4.627	0.64	1	1120.4	48.333332	K.GHFGPINSVAFHPDGK.S
IF34_MOUSE	MK_SCX_18.7300.7300.3	3	3.58	0.498	1	410.6	24.21875	K.GIPLPTGDTSPEPELLPGDPLPPPKEVINGNIK.T
IF34_MOUSE	MK_SCX_30.5896.5896.2	2	3.258	0.276	1	392.7	30.357143	K.ELAEQLGLSTGEKEKLPGELEPVQAAQSK.T
IF34_MOUSE	MK_SCX_30.5900.5900.3	3	6.211	0.526	1	1156	32.142857	K.ELAEQLGLSTGEKEKLPGELEPVQAAQSK.T
IF36_MOUSE	MK_SCX_30.4951.4951.3	3	3.792	0.476	1	801.1	36.842106	K.LGHVVMGNNAVSPYQQVIEK.T
IF37_MOUSE	MK_SCX_14.6554.6554.2	2	4.978	0.606	1	407	35.714287	R.DNSDFDLLTVSETANEPPQDEGNSFNSPR.N
IF37_MOUSE	MK_SCX_2201.5079.5079.2	2	4.097	0.293	1	1495.3	86.36364	R.NM*VQFNLQTLPK.S
IF37_MOUSE	MK_SCX_2201.5809.5809.2	2	4.261	0.424	1	1841.7	86.36364	R.NMVQFNLQTLPK.S
IF37_MOUSE	MK_SCX_2201.7577.7577.2	2	3.444	0.295	1	820.5	69.230774	R.WTCCALLAGSEYLK.L
IF37_MOUSE	MK_SCX_23.6704.6704.3	3	3.75	0.514	1	358.6	30.208334	R.YNFPNPNPFVEDDM*DKNEIASVAYR.Y
IF37_MOUSE	MK_SCX_23.7044.7044.2	2	4.692	0.583	1	1045.7	45.833336	R.YNFPNPNPFVEDDMDKNEIASVAYR.Y
IF37_MOUSE	MK_SCX_23.7094.7094.3	3	3.854	0.531	1	341.2	30.208334	R.YNFPNPNPFVEDDMDKNEIASVAYR.Y
IF37_MOUSE	MK_SCX_33.4240.4240.2	2	4.499	0.434	1	1659.4	80.769226	R.IFHTVTTTDDPVIR.K
IF37_MOUSE	MK_SCX_48.3820.3820.3	3	3.35	0.395	1	1403.9	48.214287	R.IFHTVTTTDDPVIRK.L
IF39_MOUSE	MK_SCX_14.4756.4756.3	3	4.012	0.481	1	354.3	19.871796	R.QQPASESPPTDEAAGSGGSEVGQTEDAEEDAEAGPEPEVR.A
IF39_MOUSE	MK_SCX_28.3352.3352.3	3	6.154	0.544	1	1273.3	35	R.AKPAAQSEEETATSPAASPTPQSAER.S
IF39_MOUSE	MK_SCX_30.3119.3119.3	3	5.033	0.605	1	1058.3	44.736843	R.SPSQEPSAPGKAEAVGEQAR.G
IF39_MOUSE	MK_SCX_30.3137.3137.2	2	4.875	0.62	1	1201.8	65.789474	R.SPSQEPSAPGKAEAVGEQAR.G
IF39_MOUSE	MK_SCX_31.2659.2659.3	3	6.103	0.599	1	2257.6	42.045452	R.GHPSAGAEEEGGSDGSAAEAEPR.A
IF3A_MOUSE	MK_SCX_23.8432.8432.3	3	3.607	0.374	1	569.2	30.000002	K.AFKDIDIEDLEELDPDFIM*AK.Q
IF3A_MOUSE	MK_SCX_23.8862.8862.3	3	3.659	0.318	1	1157.7	37.5	K.AFKDIDIEDLEELDPDFIMAK.Q
IF3A_MOUSE	MK_SCX_35.10821.10821.2	2	2.121	0.228	1	545.2	62.5	K.KQPALDVLYDVMK.S
IF3A_MOUSE	MK_SCX_39.5388.5388.2	2	2.414	0.149	1	536.8	54.166668	K.SGNALFHASTLHR.L
IF3C_MOUSE	MK_SCX_23.4291.4291.2	2	3.084	0.331	1	690.4	80	R.YNPENLATLER.Y
IF3C_MOUSE	MK_SCX_34.5109.5109.2	2	4.526	0.421	1	1273.4	78.57143	K.GIDRYNPENLATLER.Y
IF4A1_MOUSE	MK_SCX_19.6904.6904.2	2	5.419	0.505	1	2654.9	91.66667	K.MFVLDEADEMLSR.G
IF4A1_MOUSE	MK_SCX_21.5324.5324.2	2	3.233	0.414	1	1409.7	94.44444	R.VLITTDLLAR.G
IF4A1_MOUSE	MK_SCX_36.6500.6500.2	2	3.666	0.449	1	1416.3	81.818184	R.GFKDQIYDIFQK.L
IF4B_MOUSE	MK_SCX_17.7772.7772.2	2	4.317	0.567	1	680.4	52.499996	K.SPPYTAFLGNLPYDVTEDSIK.D
IF4B_MOUSE	MK_SCX_21.3376.3376.2	2	2.378	0.272	1	326.7	58.333332	K.VAAVQPPEEGPSR.K
IF4B_MOUSE	MK_SCX_23.10180.10180.3	3	5.731	0.635	1	1318.6	39.583336	K.SPPYTAFLGNLPYDVTEDSIKDFFR.G

IF4B_MOUSE	MK_SCX_23.10383.10383.2	2	5.276	0.572	1	1118.2	52.083332	K.SPPYTAFLGNLPYDVTEDSIKDFFR.G
IF4B_MOUSE	MK_SCX_24.3827.3827.3	3	5.806	0.556	1	1215.8	32.75862	R.SQSSDTEQPSPTSGGGKVAAVQPPEEGPSR.K
IF4B_MOUSE	MK_SCX_35.3573.3573.3	3	4.193	0.336	1	867.5	44.230766	R.ARPTTDSFDDYPPR.R
IF4B_MOUSE	MK_SCX_35.3660.3660.2	2	3.241	0.382	1	612.1	61.538464	R.ARPTTDSFDDYPPR.R
IF4E_MOUSE	MK_SCX_32.3395.3395.2	2	3.78	0.521	1	1132.3	76.92308	K.IVIGYQSHADTATK.S
IF4E3_MOUSE	MK_SCX_37.3116.3116.3	3	4.249	0.401	1	1424.9	50	R.TPGRPTSSQSYEQNIK.Q
IF4G1_MOUSE	MK_SCX_18.6379.6379.2	2	2.229	0.335	1	356.7	46.875	R.GLPLVDDGGWNTVPISK.G
IF4G1_MOUSE	MK_SCX_21.7918.7918.2	2	3.045	0.447	2	1178.5	83.33333	R.FMLQDVIDLR.L
IF4G1_MOUSE	MK_SCX_23.3754.3754.2	2	2.732	0.267	1	302	36.666668	R.TAADKDRGEEDADGSK.T
IF4G1_MOUSE	MK_SCX_31.5568.5568.2	2	2.896	0.37	1	1016.8	75	R.LLTTIGKDLDFAK.A
IF4G1_MOUSE	MK_SCX_32.4200.4200.3	3	3.329	0.494	1	851.5	37.5	K.ITKPGSIDSNNQLFAPGGR.L
IF4G1_MOUSE	MK_SCX_34.3597.3597.3	3	4.264	0.508	1	2006.6	55	K.KVEYTLGEESEAPGQR.T
IF4G1_MOUSE	MK_SCX_35.4244.4244.3	3	3.355	0.249	1	1119.6	54.545456	K.SDQWKPLNLEEK.K
IF4G1_MOUSE	MK_SCX_43.3137.3137.3	3	3.23	0.438	1	450.9	30.208334	K.GSSGGSGAKPSDTASEATRPATLNR.F
IF4G1_MOUSE	MK_SCX_47.4244.4244.3	3	4.022	0.446	1	1300.4	43.421055	R.IRDPNQGGKDITEEIM*SGAR.T
IF4G1_MOUSE	MK_SCX_47.4930.4930.3	3	4.358	0.48	1	1249.3	38.157894	R.IRDPNQGGKDITEEIMSGAR.T
IF4G1_MOUSE	MK_SCX_51.3250.3250.3	3	4.261	0.441	1	548.2	42.1875	K.IHNAENIQPGEQKYEYK.S
IF4G2_MOUSE	MK_SCX_16.5846.5846.2	2	3.429	0.372	1	1615.6	63.636364	K.LTEAVVTDYLNSGNANDAVSGVR.E
IF4G2_MOUSE	MK_SCX_18.4090.4090.2	2	3.561	0.5	1	1031.9	52.63158	R.LAEDAPNFDGPAAEGQPGQK.Q
IF4G2_MOUSE	MK_SCX_23.6194.6194.2	2	2.89	0.222	1	391.9	36.95652	K.SQGLSQLYHNQSQGLLSQLQGQSK.D
IF4G2_MOUSE	MK_SCX_23.8685.8685.3	3	4.452	0.544	1	872.3	30.833334	K.MDRDPLGGLADMFGQMPGSGIGTGPGVIQDR.F
IF4G3_MOUSE	MK_SCX_16.6174.6174.2	2	2.844	0.374	1	560.5	43.18182	R.FSPLQPPAPSGSPSATPLEFDSR.R
IF4G3_MOUSE	MK_SCX_17.3404.3404.2	2	4.119	0.522	1	467.1	44.736843	K.EQAGQM*PETAAGEPTPEPPR.T
IF4G3_MOUSE	MK_SCX_21.7918.7918.2	2	3.045	0.447	2	1178.5	83.33333	R.FMLQDVIDLR.L
IF4G3_MOUSE	MK_SCX_2201.5777.5777.2	2	3.118	0.462	1	318.6	70	K.LTPQMFNQLMK.Q
IF4G3_MOUSE	MK_SCX_26.3150.3150.3	3	4.356	0.532	1	873.6	38.095238	K.AESESDGQAEETADPQSLHSGR.S
IF4G3_MOUSE	MK_SCX_30.3610.3610.3	3	4.284	0.4	1	1375	45	K.KEQAGQMPETAAGEPTPEPPR.T
IF4H_MOUSE	MK_SCX_14.10015.10015.2	2	4.49	0.456	1	567.6	39.285713	K.ELPTEPPYTAYVGNLPFNTVQGDIDAIFK.D
IF4H_MOUSE	MK_SCX_14.10062.10062.3	3	4.778	0.548	1	940.8	30.357143	K.ELPTEPPYTAYVGNLPFNTVQGDIDAIFK.D
IF4H_MOUSE	MK_SCX_16.5784.5784.3	3	3.267	0.417	1	345.5	33.333336	R.GSNMDFREPTEEERAQRPR.L
IF4H_MOUSE	MK_SCX_2201.10732.10732.3	3	6.26	0.528	1	2065.1	37.903225	R.SQKELPTEPPYTAYVGNLPFNTVQGDIDAIFK.D
IF5_MOUSE	MK_SCX_18.8658.8658.2	2	4.478	0.502	1	1294.2	75	K.AMGPLVLTEVLFDEK.I
IF5_MOUSE	MK_SCX_2201.7240.7240.2	2	3.157	0.292	1	1021.3	87.5	R.VNILFDFVK.K
IF5_MOUSE	MK_SCX_27.6517.6517.3	3	3.306	0.358	1	418.1	36.11111	K.FVLCPECENPETDLHVNPK.K
IF5_MOUSE	MK_SCX_44.5820.5820.3	3	5.532	0.467	1	820.6	39.473686	K.KFVLCPECENPETDLHVNPK.K
IF5_MOUSE	MK_SCX_49.7931.7931.3	3	3.717	0.372	1	921.6	35.714287	R.YIVNGSHEANKLQDMLDGFIKK.F
IF5A1_MOUSE	MK_SCX_16.10688.10688.2	2	5.438	0.544	1	1823.9	59.090908	R.NDFQLIGIQDGYLSLLQDSGEVR.E
IF5A1_MOUSE	MK_SCX_16.15262.15262.3	3	4.865	0.374	1	727.5	30.681818	R.NDFQLIGIQDGYLSLLQDSGEVR.E
IF5A1_MOUSE	MK_SCX_32.3762.3762.3	3	3.14	0.449	1	739.7	52.083332	R.LPEGDLGKEIEQK.Y
IF5A1_MOUSE	MK_SCX_35.10715.10715.2	2	2.694	0.61	1	1129.5	81.818184	K.VHLVGIDIFTGK.K
IF5A1_MOUSE	MK_SCX_36.6606.6606.3	3	3.9	0.309	1	1168.4	50	K.VHLVGIDIFTGK.K
IF5A1_MOUSE	MK_SCX_39.5548.5548.3	3	4.751	0.45	1	939.1	48.4375	R.EDLRLPEGDLGKEIEQK.Y
IF5A1_MOUSE	MK_SCX_39.5784.5784.2	2	5.078	0.415	1	444.7	56.25	R.EDLRLPEGDLGKEIEQK.Y
IF5A1_MOUSE	MK_SCX_51.5375.5375.2	2	3.136	0.522	1	1872.1	83.33333	K.VHLVGIDIFTGKK.Y
IF5A1_MOUSE	MK_SCX_51.5400.5400.3	3	3.827	0.435	1	868.5	50	K.VHLVGIDIFTGKK.Y
IF6_MOUSE	MK_SCX_15.8027.8027.2	2	3.551	0.36	1	515.1	41.304348	K.TSIEDQDELSSLLQVPLVAGTVNR.G
IF6_MOUSE	MK_SCX_49.4797.4797.3	3	3.519	0.397	1	928.7	36.764706	R.HGLLVPNNTTDQELQHIR.N
IFI4_MOUSE	MK_SCX_18.3163.3163.2	2	6.119	0.661	1	1744.5	54.545456	R.GETSATQEETSTAQAGTSTAQAR.T
IFIH1_MOUSE	MK_SCX_16.3589.3589.2	2	4.957	0.499	1	1747	71.875	R.DSGTMGSDSDESVIQTK.R
IGBP1_MOUSE	MK_SCX_19.4670.4670.2	2	3.882	0.401	1	482.6	79.16667	R.NEDLEEIASTDLK.Y
IGBP1_MOUSE	MK_SCX_21.5743.5743.2	2	2.616	0.154	1	630.2	62.5	K.AAGM*LSQLDLFSR.N

IGBP1_MOUSE	MK_SCX_21.7194.7194.2	2	3.377	0.309	1	846.9	62.5	K.AAGMLSQLDLFSR.N
IGBP1_MOUSE	MK_SCX_26.7248.7248.3	3	3.874	0.43	1	581.8	36.363636	K.VFGTGYPSLATMTVSDWYEQHQK.Y
IGBP1_MOUSE	MK_SCX_32.4307.4307.3	3	3.055	0.385	1	522.3	36.666668	K.KLLEDVEVATEPTGSR.T
IGBP1_MOUSE	MK_SCX_4.6628.6628.2	2	2.238	0.214	1	452.3	53.846157	K.YLMVPALQGALTMK.Q
IGF1R_MOUSE	MK_SCX_20_1.4411.4411.2	2	2.254	0.149	1	313	40	R.ELGQGSFGMVYEGVAK.G
IGF1R_MOUSE	MK_SCX_51.2946.2946.3	3	3.832	0.551	1	884.1	46.42857	K.TEAEKQAEKEEAEYR.K
ILF3_MOUSE	MK_SCX_17.5553.5553.2	2	2.556	0.179	1	321.9	44.11765	K.VLAGETLSVNDPPDVLDR.Q
ILF3_MOUSE	MK_SCX_20_1.3761.3761.2	2	4.457	0.489	1	802.9	73.07692	K.VLQDM*GLPTGAEGR.D
ILF3_MOUSE	MK_SCX_25.3471.3471.3	3	3.315	0.493	1	1431.2	28.676472	R.SGGNSYGSSGSSSYNTGSHGGYGTGSGGSSSYQGK.Q
ILF3_MOUSE	MK_SCX_27.4016.4016.3	3	5.489	0.397	1	1313.2	30.000002	K.ASYSSGYQSHQGQQQPYNQSQYSSYGTPQGK.Q
ILF3_MOUSE	MK_SCX_42.6715.6715.3	3	4.638	0.6	1	676.5	36.363636	K.HSSVYPTQEELEAVQNMVSHTER.A
ILK_MOUSE	MK_SCX_18.6257.6257.3	3	3.012	0.152	1	409.3	21.73913	R.GARINVMNRGDDTPLHLAASHGHR.D
ILK_MOUSE	MK_SCX_43.4464.4464.2	2	3.076	0.41	1	495.3	81.25	R.IPYKDTFWK.G
IMB1_MOUSE	MK_SCX_19.7795.7795.2	2	4.834	0.463	1	956.3	75	R.AAVENLPTFLVELSR.V
IMB1_MOUSE	MK_SCX_46.5679.5679.3	3	3.599	0.359	1	1012.3	41.666664	K.LVEARPMIHELLTEGR.R
IMDH1_MOUSE	MK_SCX_49.6077.6077.3	3	3.958	0.233	1	784.9	32.894737	R.RFGVPVIADGGIQTVGHVVK.A
IMMT_MOUSE	MK_SCX_17.4975.4975.2	2	3.077	0.197	1	954.3	47.368423	R.TSSVTLQTITAQNAAVQAVK.A
IMMT_MOUSE	MK_SCX_18.14385.14385.2	2	3.281	0.36	1	485.6	40	K.IAGAGLLFVGGGIGGTILYAK.W
IMMT_MOUSE	MK_SCX_18.6496.6496.2	2	3.535	0.279	1	699.3	55.555557	K.TSSAEMPTIPLGSAVEAIR.V
IMMT_MOUSE	MK_SCX_18.6872.6872.2	2	2.996	0.522	1	493.5	50	K.LFGM*VLGSAPYTVPLPK.K
IMMT_MOUSE	MK_SCX_18.7567.7567.2	2	4.401	0.637	1	1342.3	65.625	K.LFGMVLGSAPYTVPLPK.K
IMMT_MOUSE	MK_SCX_19.5353.5353.2	2	3.399	0.224	1	879.9	65.38461	K.ELDSITPDITPGWK.G
IMMT_MOUSE	MK_SCX_2201.2565.2565.2	2	3.128	0.537	1	1162.5	79.16667	R.YSTSSSSGLTAGK.I
IMMT_MOUSE	MK_SCX_23.4845.4845.2	2	2.8	0.129	1	607.3	83.33333	K.FVNQLKGESR.R
IMMT_MOUSE	MK_SCX_24.10540.10540.3	3	5.719	0.481	1	1272.8	36.95652	K.TIPYSDKLFGMVLGSAPYTVPLPK.K
IMMT_MOUSE	MK_SCX_24.10543.10543.2	2	5.261	0.647	1	1873.6	56.521736	K.TIPYSDKLFGMVLGSAPYTVPLPK.K
IMMT_MOUSE	MK_SCX_30.5122.5122.3	3	4.346	0.554	1	1243.1	48.4375	K.VQEQELKYEFEQGLSEK.L
IMMT_MOUSE	MK_SCX_30.5144.5144.2	2	6.066	0.521	1	1838.9	75	K.VQEQELKYEFEQGLSEK.L
IMMT_MOUSE	MK_SCX_31.4021.4021.3	3	4.768	0.463	1	1452.4	45.3125	R.GIEQAVQSHAVAEEEAR.K
IMMT_MOUSE	MK_SCX_34.5788.5788.2	2	4.25	0.642	1	2043.3	87.5	R.LHNMIVDLDNVVK.K
IMMT_MOUSE	MK_SCX_35.3717.3717.3	3	3.046	0.249	1	459.4	50	K.VVSQYHELVVQAR.D
IMMT_MOUSE	MK_SCX_36.7681.7681.3	3	3.044	0.352	1	580.7	34.72222	R.DDFRKELDSITPDITPGWK.G
IMMT_MOUSE	MK_SCX_41.3951.3951.3	3	4.119	0.417	1	656.1	32.894737	K.ISSVSEVM*KDSKLPVAQSQK.T
IMMT_MOUSE	MK_SCX_41.4842.4842.3	3	5.306	0.325	1	957.4	39.473686	K.ISSVSEVMKDSKLPVAQSQK.T
IMMT_MOUSE	MK_SCX_41.4860.4860.2	2	5.026	0.594	1	1358.8	60.526318	K.ISSVSEVMKDSKLPVAQSQK.T
IMMT_MOUSE	MK_SCX_45.5690.5690.2	2	3.809	0.434	1	1542.7	71.42857	K.LSTDDLNSLIAHAHR.R
IMMT_MOUSE	MK_SCX_49.4241.4241.3	3	5.974	0.481	1	1626	43.055553	R.LRGIEQAVQSHAVAEEEAR.K
IMMT_MOUSE	MK_SCX_49.5461.5461.2	2	3.794	0.583	1	1287.8	76.92308	R.LHNMIVDLDNVVKK.V
IMMT_MOUSE	MK_SCX_52.3414.3414.3	3	3.264	0.434	1	511.2	44.230766	R.KVEEVRDAMENEMR.T
IMPA1_MOUSE	MK_SCX_20_1.4140.4140.2	2	3.975	0.415	1	1205.9	79.16667	K.SSPADLVTVTDQK.V
IMPA1_MOUSE	MK_SCX_21.3469.3469.2	2	3.878	0.358	1	1090.4	90	K.LQVSQQEDITK.S
IMPA1_MOUSE	MK_SCX_28.4312.4312.2	2	4.595	0.466	1	1868.6	80	K.SSPADLVTVTDQKVEK.M
IN35_MOUSE	MK_SCX_18.7554.7554.2	2	4.098	0.566	1	924.1	78.125	K.IPFSVPEVPLVFQGQTK.Q
INADL_MOUSE	MK_SCX_13.7897.7897.2	2	4.769	0.535	1	669.3	50	R.DPVGEIAVTPPTPVSLPVALPAVATR.T
INADL_MOUSE	MK_SCX_19.5135.5135.2	2	3.625	0.453	1	471.3	47.22222	K.IGGTNVQGMTSEQVAQVLR.N
INMT_MOUSE	MK_SCX_14.3930.3930.2	2	2.562	0.367	1	1016.2	85	K.AIQDAGCQVLK.C
INMT_MOUSE	MK_SCX_17.5712.5712.2	2	5.841	0.555	1	1530.2	78.125	R.EIIVTDYTPQNLQELQK.W
INMT_MOUSE	MK_SCX_23.6347.6347.3	3	4.812	0.585	1	2128.8	46.25	K.DYLTTYYSFHSGPVAEQEIVK.F
INMT_MOUSE	MK_SCX_23.6357.6357.2	2	5.466	0.603	1	1277.3	52.499996	K.DYLTTYYSFHSGPVAEQEIVK.F
INMT_MOUSE	MK_SCX_30.4764.4764.2	2	5.068	0.505	1	2616.7	82.14286	K.VYIGGEDYEKEFTPK.D
INMT_MOUSE	MK_SCX_45.6841.6841.2	2	4.158	0.539	1	614.1	67.64706	R.LAGLLKPGGHLVTLVTLR.F

INMT_MOUSE	MK_SCX_51.4066.4066.3	3	4.263	0.413	1	543.2	44.230766	K.KFSGVYLEKEVVEK.A
INPP_MOUSE	MK_SCX_19.4617.4617.2	2	3.29	0.251	1	483.2	56.666668	K.VFGEESNEFTNDLGEK.I
IPP1_MOUSE	MK_SCX_15.4407.4407.3	3	3.815	0.361	1	328.9	19.078947	K.QGEEPEGATESTGNQESCPPGIPDTGSASRPDTPGTAQK.S
IPP1_MOUSE	MK_SCX_37.8270.8270.3	3	4.025	0.544	1	1007	39.285713	R.KIQFTVPLLEPHLDPEAAEQIR.R
IPYR_MOUSE	MK_SCX_23.5654.5654.3	3	4.041	0.539	1	786.8	35.714287	K.VIAINVDDPDAANYKDISDVER.L
IPYR_MOUSE	MK_SCX_36.7364.7364.3	3	3.889	0.48	1	1048	50	R.LKPGYLEATVDWFR.R
IPYR_MOUSE	MK_SCX_39.4169.4169.2	2	2.803	0.329	1	754.3	77.77778	K.NKDFAVDIIK.S
IPYR_MOUSE	MK_SCX_41.4084.4084.3	3	3.726	0.337	1	335.8	37.5	K.M*EIATKDPLNPIKQDVK.K
IPYR_MOUSE	MK_SCX_41.4565.4565.3	3	3.95	0.448	1	1129.1	42.1875	K.MEIATKDPLNPIKQDVK.K
IPYR_MOUSE	MK_SCX_41.4624.4624.2	2	5.035	0.554	1	906.4	65.625	K.MEIATKDPLNPIKQDVK.K
IPYR_MOUSE	MK_SCX_42.8042.8042.3	3	4.148	0.492	1	750.8	28.846153	K.GQYISPFHDVPIYADKDVFHMVVEVPR.W
IPYR2_MOUSE	MK_SCX_18.7458.7458.2	2	4.13	0.289	1	1477.3	70	R.NDEYENLFNM*VVEIPR.W
IPYR2_MOUSE	MK_SCX_18.8534.8534.2	2	3.562	0.262	1	517.3	56.666668	R.NDEYENLFNMVVEIPR.W
IPYR2_MOUSE	MK_SCX_20_1.3873.3873.2	2	3.342	0.486	1	545.7	75	R.SLVESVPTPSM*NK.E
IPYR2_MOUSE	MK_SCX_20_1.4434.4434.2	2	3.569	0.407	1	582.6	70.83333	R.SLVESVPTPSMNK.E
IPYR2_MOUSE	MK_SCX_27.5486.5486.2	2	5.243	0.501	1	1478	71.875	K.MEIATEEPLNPIKQDIK.N
IPYR2_MOUSE	MK_SCX_27.5518.5518.3	3	4.066	0.481	1	894	43.75	K.MEIATEEPLNPIKQDIK.N
IPYR2_MOUSE	MK_SCX_28.7465.7465.2	2	4.174	0.454	1	934	61.11111	K.GYIWNYGALPQTWEDPHLR.D
IPYR2_MOUSE	MK_SCX_28.7621.7621.3	3	3.733	0.557	1	1318	50	K.GYIWNYGALPQTWEDPHLR.D
IPYR2_MOUSE	MK_SCX_34.4664.4664.2	2	4.441	0.5	1	1635.9	83.33333	K.AFALDVINSAHER.W
IPYR2_MOUSE	MK_SCX_46.5420.5420.3	3	4.248	0.428	1	868.1	35.714287	K.IIAINVNDPEAEKFHDIDDVKK.F
IPYR2_MOUSE	MK_SCX_56.4724.4724.3	3	3.975	0.425	1	1036.6	41.666664	K.HVAGHYISPFHDIPLK.A
IQGA1_MOUSE	MK_SCX_21.4580.4580.2	2	3.676	0.513	1	1207.5	77.27273	K.ATFYGEQVDYYK.S
IQGA1_MOUSE	MK_SCX_24.7005.7005.3	3	3.215	0.38	1	458.7	35.416664	R.FQPGETLTEILETPATNEQEAEHQR.A
IREB1_MOUSE	MK_SCX_15.8117.8117.2	2	4.364	0.565	1	573.7	40.384613	R.SNLVGM*GVIPLEYLPGETADSLGLTGR.E
IREB1_MOUSE	MK_SCX_15.8724.8724.2	2	4.381	0.522	1	434.9	40.384613	R.SNLVGMGVIPLEYLPGETADSLGLTGR.E
IREB1_MOUSE	MK_SCX_17.7693.7693.2	2	5.475	0.583	1	890	66.66667	R.VILQDFTGVPAVVDFAAM*R.D
IREB1_MOUSE	MK_SCX_17.8271.8271.2	2	6.27	0.687	1	1125.3	75	R.VILQDFTGVPAVVDFAAMR.D
IREB1_MOUSE	MK_SCX_18.7926.7926.2	2	3.381	0.325	1	628.5	53.333336	K.SPPFFESLTLDLQPPK.S
IREB1_MOUSE	MK_SCX_2201.13540.13540.3	3	3.523	0.304	1	327.2	23.148148	K.SIVDAYVLLNLGDSVTTDHISPAGNIAR.N
IREB1_MOUSE	MK_SCX_2201.3120.3120.2	2	2.294	0.295	1	524.3	81.25	K.AVLAESYER.I
IREB1_MOUSE	MK_SCX_25.4819.4819.2	2	2.147	0.331	1	1259.7	93.75	K.YLQAVGMFR.D
IREB1_MOUSE	MK_SCX_25.6351.6351.3	3	4.434	0.534	1	729.6	30.952381	K.QAPQTVHLPSGETLDVFDAAER.Y
IREB1_MOUSE	MK_SCX_28.6511.6511.3	3	3.589	0.466	1	730.6	35.714287	R.YQQAGLPLIVLAGKEYGSGSSR.D
IREB1_MOUSE	MK_SCX_31.4413.4413.2	2	3.923	0.46	1	588.6	60.714287	R.IDFEKEPLGVNAQGR.Q
IREB1_MOUSE	MK_SCX_31.4418.4418.3	3	3.587	0.414	1	1021.3	48.214287	R.IDFEKEPLGVNAQGR.Q
IREB1_MOUSE	MK_SCX_33.5124.5124.3	3	3.239	0.546	1	660.8	45.454548	R.YTINIPEDLKPR.M
IREB1_MOUSE	MK_SCX_33.5159.5159.2	2	2.325	0.431	1	306.5	63.636364	R.YTINIPEDLKPR.M
IREB1_MOUSE	MK_SCX_39.4084.4084.2	2	2.476	0.452	1	640.5	77.77778	K.NIEVPFKPAR.V
IREB1_MOUSE	MK_SCX_40.5171.5171.2	2	2.88	0.259	1	858.9	80	R.FFNLNKLEDSR.Y
IREB1_MOUSE	MK_SCX_49.6738.6738.3	3	3.722	0.211	1	875.4	38.333332	K.KNDIENILNWNVMQHK.N
IREB1_MOUSE	MK_SCX_55.5556.5556.3	3	4.587	0.469	1	891	40.789474	-.MKNPFAHLAEPLDAAQPGKR.F
IRF6_MOUSE	MK_SCX_27.4487.4487.3	3	4.834	0.427	1	1955.8	47.368423	K.AWAVETGKYQEGVDDPDPAK.W
IRK10_MOUSE	MK_SCX_29.4414.4414.3	3	4.778	0.531	1	1147.6	39.473686	K.VYYSQTTQTESRPLVAPGIR.R
ITA3_MOUSE	MK_SCX_19.7351.7351.2	2	5.321	0.603	1	702.7	73.333336	K.LGLPGLATFGYSLSGK.M
ITA3_MOUSE	MK_SCX_20_1.5645.5645.2	2	3.683	0.327	1	1033.1	70.83333	R.AAFLSEQLQPLSR.L
ITA3_MOUSE	MK_SCX_21.9613.9613.3	3	4.334	0.438	1	765.8	33.695652	K.M*DVDENLYPDLLVGSLSDHIVLLR.A
ITA3_MOUSE	MK_SCX_21.9797.9797.3	3	3.623	0.327	1	322	23.913044	K.MDVDENLYPDLLVGSLSDHIVLLR.A
ITA3_MOUSE	MK_SCX_29.6492.6492.2	2	4.018	0.56	1	1244.3	65.625	K.EAVNPGSLFGYSVALHR.Q
ITA3_MOUSE	MK_SCX_34.6535.6535.3	3	3.077	0.211	1	526.4	35.9375	R.M*ELLIAFEVIGVTLHTR.D
ITA6_MOUSE	MK_SCX_15.6546.6546.2	2	6.046	0.624	1	1321.8	56.521736	K.NIGDINQDGYPDIAVGAPYDDLGK.V

ITA6_MOUSE	MK_SCX_18.7362.7362.2	2	4.914	0.625	1	1939.6	72.22222	R.NSYPDLAVGSLSDSVTIFR.S
ITA6_MOUSE	MK_SCX_19.5856.5856.2	2	3.832	0.439	1	599.2	67.85714	K.LIATFPDTLTYSAYR.E
ITA6_MOUSE	MK_SCX_32.4312.4312.3	3	3.497	0.327	1	887.1	48.076923	R.EGNQDKFSYLPIQK.G
ITA6_MOUSE	MK_SCX_32.4330.4330.2	2	3.955	0.319	1	931.7	61.538464	R.EGNQDKFSYLPIQK.G
ITA6_MOUSE	MK_SCX_52.5438.5438.3	3	3.409	0.416	1	870.6	36.11111	R.RRVNSLPEVLPILNSNEAK.T
ITAV_MOUSE	MK_SCX_14.6905.6905.2	2	2.478	0.203	1	600.1	60.714287	R.NGYPDLVVGAFGVDR.A
ITAV_MOUSE	MK_SCX_14.7962.7962.2	2	3.888	0.582	1	806.1	39.655174	R.FGSAIAPLGDLDQDGFNDIAIAAPYGGEDK.K
ITAV_MOUSE	MK_SCX_17.8348.8348.2	2	2.029	0.127	1	400.6	37.5	K.IQSSNSFDNVSPVVSYK.V
ITB1_MOUSE	MK_SCX_2201.2912.2912.2	2	3.33	0.386	1	1407.8	83.33333	K.SAVTTVVNPK.Y
ITB1_MOUSE	MK_SCX_2201.4222.4222.2	2	2.919	0.3	1	1089.5	80	K.SLGTDLM*NEMR.R
ITB1_MOUSE	MK_SCX_2201.5266.5266.2	2	3.188	0.427	1	824.9	80	K.SLGTDLMNEMR.R
ITB1_MOUSE	MK_SCX_23.5139.5139.2	2	3.372	0.471	1	749	68.181816	R.GEFFNELVGQQR.I
ITB1_MOUSE	MK_SCX_29.7074.7074.2	2	4.977	0.411	1	1435.2	68.75	K.LRPEDITQIQPQQLLLK.L
ITB1_MOUSE	MK_SCX_29.7115.7115.3	3	5.111	0.361	1	1931.9	53.125	K.LRPEDITQIQPQQLLLK.L
ITB2_MOUSE	MK_SCX_20_1.3645.3645.2	2	3.389	0.394	1	1143.2	69.230774	K.LTDNSNQFQTEVGK.Q
ITB5_MOUSE	MK_SCX_27.7777.7777.3	3	3.12	0.272	1	650.6	35.526314	R.LGFGSFVDKDISPFSYTAPR.Y
ITB5_MOUSE	MK_SCX_33.5942.5942.2	2	2.558	0.17	1	385.9	54.545456	K.CPTCPDACSSKR.D
ITSN2_MOUSE	MK_SCX_06.6393.6393.2	2	2.094	0.148	1	385.7	44.444447	R.QKGWFPASHVKLLGPSSER.T
ITSN2_MOUSE	MK_SCX_25.5058.5058.2	2	3.944	0.375	1	1519.8	79.16667	K.NTSGWWQGELQAR.G
ITSN2_MOUSE	MK_SCX_48.4048.4048.3	3	3.42	0.43	1	809.3	48.214287	R.LKEQLDALEKETASK.L
IVD_MOUSE	MK_SCX_16.6504.6504.2	2	5.409	0.674	1	1256.9	52.083332	K.LISGEFIGALAM*SEPNAGSDVVSM*K.L
IVD_MOUSE	MK_SCX_16.7007.7007.2	2	5.144	0.455	1	1632.6	56.25	K.LISGEFIGALAM*SEPNAGSDVVSMK.L
IVD_MOUSE	MK_SCX_16.7056.7056.3	3	5.247	0.352	1	2365	40.625	K.LISGEFIGALAM*SEPNAGSDVVSMK.L
IVD_MOUSE	MK_SCX_16.7654.7654.3	3	4.16	0.466	1	870.1	34.375	K.LISGEFIGALAMSEPNAGSDVVSMK.L
IVD_MOUSE	MK_SCX_16.7676.7676.2	2	5.162	0.642	1	1497.9	58.333332	K.LISGEFIGALAMSEPNAGSDVVSMK.L
IVD_MOUSE	MK_SCX_20_1.15515.15515.2	2	2.443	0.142	1	403.9	50	R.LM*ASRQYVYNVAK.A
IVD_MOUSE	MK_SCX_21.3319.3319.2	2	2.839	0.381	1	958.8	77.77778	K.TDLTAVPASR.G
IVD_MOUSE	MK_SCX_2201.3784.3784.2	2	3.14	0.288	1	842.1	87.5	K.FLQENLAPK.A
IVD_MOUSE	MK_SCX_2201.3912.3912.2	2	2.934	0.477	1	627.6	72.72727	K.LYEIGAGTSEVR.R
IVD_MOUSE	MK_SCX_25.6408.6408.3	3	4.569	0.375	1	765.9	32.291664	R.GSNTCELVFEDCKVPAANVLSQESK.G
IVD_MOUSE	MK_SCX_25.6417.6417.2	2	3.805	0.377	1	372.8	35.416664	R.GSNTCELVFEDCKVPAANVLSQESK.G
IVD_MOUSE	MK_SCX_33.5303.5303.3	3	3.517	0.315	1	1243.7	41.666664	R.EAFGQKIGQFQLMQGK.M
IVD_MOUSE	MK_SCX_36.3635.3635.2	2	2.512	0.294	1	585.8	62.5	K.LYEIGAGTSEVRR.L
IWS1_MOUSE	MK_SCX_19.4753.4753.2	2	3.326	0.123	1	449.9	61.538464	K.ILQELPSVSQETLK.H
IWS1_MOUSE	MK_SCX_32.6949.6949.3	3	3.574	0.224	1	800.3	39.0625	K.LINEWSRPIFGLTSNYK.G
IYD1_MOUSE	MK_SCX_23.4856.4856.2	2	2.424	0.347	1	503.4	72.22222	R.SQEFYELLNK.R
IYD1_MOUSE	MK_SCX_28.6395.6395.3	3	3.762	0.308	1	766.4	40.625	R.FISSEHVPM*EVIENVIK.A
IYD1_MOUSE	MK_SCX_28.7418.7418.2	2	4.074	0.496	1	819.4	68.75	R.FISSEHVPMEVIENVIK.A
IYD1_MOUSE	MK_SCX_29.7888.7888.3	3	3.904	0.152	1	806.8	42.1875	R.FISSEHVPMEVIENVIK.A
JAG1_MOUSE	MK_SCX_24.3910.3910.3	3	3.883	0.36	1	370.3	25	K.DHCRTTTCEVIDSCTVAMASNDTPEGVR.Y
JIP4_MOUSE	MK_SCX_15.6461.6461.2	2	4.176	0.613	1	916.3	50	R.MGDEGGESELLGEDLPLEPSVTK.A
JIP4_MOUSE	MK_SCX_16.6270.6270.2	2	4.791	0.665	1	1259.2	60.416668	K.FFVAVPGQVISPQSSSGGADLTADK.A
JIP4_MOUSE	MK_SCX_17.6460.6460.2	2	3.481	0.481	1	770.4	60.000004	R.EVENLILENTQLLETK.N
JIP4_MOUSE	MK_SCX_17.9360.9360.2	2	2.999	0.202	1	596.2	41.17647	K.CLHSIKLKDSILSIVHVK.G
JIP4_MOUSE	MK_SCX_23.5326.5326.2	2	2.727	0.232	1	708.2	75	R.VQAFGWSLPQK.Y
JIP4_MOUSE	MK_SCX_25.8171.8171.3	3	3.713	0.396	1	375.7	25	K.ERPISLGIFPLPAGDGLLTPDTQK.G
JIP4_MOUSE	MK_SCX_41.4173.4173.3	3	4.284	0.48	1	889.3	47.058823	K.FIEFEDSQEQEKKDLQTR.V
JIP4_MOUSE	MK_SCX_50.3343.3343.3	3	4.733	0.496	1	1754.3	56.25	K.LHQLSGSDQLEATAHSR.I
JIP4_MOUSE	MK_SCX_56.3220.3220.3	3	5.896	0.515	1	2971.7	50	R.TKLHQLSGSDQLEATAHSR.I
JUN_MOUSE	MK_SCX_19.6810.6810.2	2	2.626	0.309	1	355.4	43.75	K.TLKAQNSELASTANMLR.E
JUN_MOUSE	MK_SCX_20_1.6891.6891.2	2	2.074	0.143	1	301.6	37.5	K.TLKAQNSELASTANM*LR.E

K0152_MOUSE	MK_SCX_20_1.4351.4351.2	2	3.919	0.521	1	750.2	75	R.STPEDQILYQTER.Y
K0152_MOUSE	MK_SCX_2201.4151.4151.2	2	5.021	0.442	1	2264.6	90.909096	K.FAEVYFAQSQQK.V
K0152_MOUSE	MK_SCX_23.6974.6974.3	3	4.759	0.474	1	955.5	34.090908	R.YNEETFGYEVPVKEEGDYVLVLK.F
K0152_MOUSE	MK_SCX_34.5327.5327.3	3	3.763	0.431	1	981.3	54.545456	R.ASDYGMKLPILR.S
K0152_MOUSE	MK_SCX_34.5346.5346.2	2	2.207	0.322	1	438.1	54.545456	R.ASDYGMKLPILR.S
K0152_MOUSE	MK_SCX_48.3044.3044.3	3	6.227	0.48	1	2214.1	55.88235	K.KEEEEEEEEYDEGSNLKR.Q
K0152_MOUSE	MK_SCX_50.3984.3984.3	3	4.188	0.315	1	1453.5	53.333336	R.VGHSTAHDEIIPMSIR.K
K0157_MOUSE	MK_SCX_19.5344.5344.2	2	3.794	0.514	1	525.4	59.375	R.ISLAIPNLGNTSQQEYK.V
K0157_MOUSE	MK_SCX_21.4828.4828.2	2	3.114	0.433	1	614.3	68.181816	R.AIYQVYNALQEK.V
K0174_MOUSE	MK_SCX_14.9097.9097.3	3	3.487	0.467	1	454.8	23.648647	R.GGGGGFTAPVGGPDGIVPMPMPMPMPSPNAPFAYPLPK.G
K0174_MOUSE	MK_SCX_51.3570.3570.3	3	4.56	0.33	1	1333.9	55.76923	R.KEIADYLAAGKDER.A
K0971_MOUSE	MK_SCX_19.6799.6799.2	2	2.16	0.23	1	390.9	54.166668	R.LNVFDEGLQPSVR.Y
K0971_MOUSE	MK_SCX_54.11173.11173.3	3	3.025	0.421	1	312.5	27.380953	R.CLSILSTALVSM*EPCM*NVNALR.A
K1543_MOUSE	MK_SCX_15.6763.6763.2	2	5.023	0.646	1	619.5	43.103447	K.AEAESGLGSPTSTPVAPEALSSEMSELGAR.L
K1543_MOUSE	MK_SCX_15.7037.7037.2	2	4.426	0.556	1	936.9	51.923077	R.LLAPPEAPGPAPPPAAWVIPGPATGPK.A
K1543_MOUSE	MK_SCX_15.7264.7264.2	2	4.79	0.652	1	1261.2	44.642857	R.LAQEAPGLAFTTPVVASAAPVATLAPTTR.A
K1967_MOUSE	MK_SCX_16.3628.3628.2	2	3.602	0.578	1	699.9	50	K.EAAPDTGAEPSPEDSDPTYSSK.V
K1967_MOUSE	MK_SCX_2201.1732.1732.2	2	2.135	0.335	1	695.1	87.5	R.LAEAEETAR.T
K1967_MOUSE	MK_SCX_25.10544.10544.3	3	3.222	0.398	1	565.8	33.695652	K.DEVQNEGTAAESDSPLKEDGLLPK.R
K1C18_MOUSE	MK_SCX_21.5042.5042.2	2	4.002	0.433	1	2055.1	80	K.NQNINLENSLGDVEAR.Y
K1C18_MOUSE	MK_SCX_26.9244.9244.2	2	4.578	0.423	1	457.8	58.823532	R.LQLETEIEALKEELLFM*K.K
K1C18_MOUSE	MK_SCX_38.3273.3273.3	3	4.383	0.523	1	2096.6	43.421055	R.VRPASSAASVYAGAGGSGSR.I
K1C18_MOUSE	MK_SCX_41.3522.3522.3	3	3.514	0.149	1	999.7	58.333332	R.VKYETELAMR.Q
K1C18_MOUSE	MK_SCX_41.3542.3542.2	2	3.178	0.521	1	533.4	83.33333	R.VKYETELAMR.Q
K1C18_MOUSE	MK_SCX_48.4780.4780.3	3	3.97	0.407	1	633.6	38.333332	K.SAEIRDAETTLTELRR.T
K1KB5_MOUSE	MK_SCX_50.5727.5727.3	3	5.36	0.549	1	1181.2	42.857143	R.LKKPADITDVVKPIDLPTEEPK.L
K1KB5_MOUSE	MK_SCX_50.5756.5756.2	2	5.034	0.557	1	1331.5	52.380955	R.LKKPADITDVVKPIDLPTEEPK.L
K2C8_MOUSE	MK_SCX_23.5422.5422.2	2	3.769	0.503	1	1313.2	77.27273	K.SLNNKFASFIDK.V
K2C8_MOUSE	MK_SCX_47.4298.4298.3	3	4.479	0.528	1	1340.6	48.333332	K.KDVDEAYMNKVELESR.L
K6PL_MOUSE	MK_SCX_32.6836.6836.2	2	3.84	0.544	1	936.1	65.625	K.GQVQEVGWHDVAGWLGR.G
KAD1_MOUSE	MK_SCX_18.5017.5017.2	2	2.895	0.431	1	1178.3	69.230774	K.VDSSNGFLIDGYPR.E
KAD1_MOUSE	MK_SCX_18.8055.8055.2	2	3.446	0.426	1	652	73.07692	K.GELVPLDTVLDM*LR.D
KAD1_MOUSE	MK_SCX_18.9464.9464.2	2	4.079	0.474	1	474.8	73.07692	K.GELVPLDTVLDMLR.D
KAD1_MOUSE	MK_SCX_36.4596.4596.2	2	3.979	0.485	1	1789.2	83.33333	K.YGYTHLSTGDLLR.A
KAD1_MOUSE	MK_SCX_36.4623.4623.3	3	4.303	0.567	1	956.8	52.083332	K.YGYTHLSTGDLLR.A
KAD1_MOUSE	MK_SCX_46.6019.6019.3	3	5.938	0.562	1	1683.3	50	K.RLETYYNATEPVISFYDKR.G
KAD1_MOUSE	MK_SCX_51.2263.2263.3	3	3.193	0.237	1	943	36.666668	K.RGETSGRVDDNEETIK.K
KAD2_MOUSE	MK_SCX_15.6570.6570.2	2	2.661	0.303	1	1451.2	88.88889	K.NGFLLDGFPR.T
KAD2_MOUSE	MK_SCX_18.5774.5774.2	2	3.4	0.259	1	1264.9	75	K.LVSDEM*VVELIEK.N
KAD2_MOUSE	MK_SCX_18.7010.7010.2	2	4.297	0.412	1	1714.3	87.5	K.LVSDEMVVELIEK.N
KAD2_MOUSE	MK_SCX_2201.2837.2837.2	2	3.195	0.331	1	1446.3	85	R.AMVASGSELGK.K
KAD2_MOUSE	MK_SCX_24.14639.14639.3	3	3.113	0.232	1	372.3	31.578945	K.ATMDAGKLVSDEMVVELIEK.N
KAD2_MOUSE	MK_SCX_30.4627.4627.2	2	5.77	0.555	1	2321.8	73.333336	R.LEAYHTQTTPLVEYYR.K
KAD2_MOUSE	MK_SCX_30.4775.4775.1	1	4.342	0.544	1	693.4	66.66667	R.LEAYHTQTTPLVEYYR.K
KAD2_MOUSE	MK_SCX_30.4794.4794.3	3	4.552	0.414	1	1611.2	50	R.LEAYHTQTTPLVEYYR.K
KAD2_MOUSE	MK_SCX_31.6810.6810.3	3	4.613	0.434	1	840.3	42.1875	K.LAENFCVCHLATGDMLR.A
KAD2_MOUSE	MK_SCX_31.6858.6858.2	2	4.266	0.389	1	1398.3	65.625	K.LAENFCVCHLATGDMLR.A
KAD2_MOUSE	MK_SCX_32.4712.4712.3	3	3.303	0.333	1	994.6	47.727272	R.QAEMLDDLMEKR.K
KAD2_MOUSE	MK_SCX_32.5074.5074.3	3	4.629	0.558	1	896.3	48.333332	-.APNVLASEPEIPKGIR.A
KAD2_MOUSE	MK_SCX_44.4373.4373.3	3	5.369	0.449	1	1781.5	46.875	R.LEAYHTQTTPLVEYYRK.R
KAD2_MOUSE	MK_SCX_49.4860.4860.3	3	4.654	0.409	1	1770.1	50	K.TRLEAYHTQTTPLVEYYR.K

KAD2_MOUSE	MK_SCX_49.4931.4931.2	2	6.192	0.55	1	1193.5	70.588234	K.TRLEAYHTQTTPLVEYYR.K
KAD2_MOUSE	MK_SCX_50.4880.4880.3	3	5.455	0.596	1	1185.8	38.04348	R.SYHEEFNPPKEPM*KDDITGEPLIR.R
KAD2_MOUSE	MK_SCX_50.5407.5407.3	3	6.382	0.615	1	2287.5	40.217392	R.SYHEEFNPPKEPMKDDITGEPLIR.R
KAD2_MOUSE	MK_SCX_50.5424.5424.2	2	5.615	0.609	1	1226.1	54.347824	R.SYHEEFNPPKEPMKDDITGEPLIR.R
KAD3_MOUSE	MK_SCX_13.7544.7544.2	2	3.686	0.434	1	1020.7	69.230774	K.TLTQCSWLLDGFPR.T
KAD3_MOUSE	MK_SCX_17.8752.8752.2	2	4.138	0.373	1	791.7	55.88235	K.VYQIDTVINLNVPFEVIK.Q
KAD3_MOUSE	MK_SCX_18.5676.5676.2	2	4.882	0.463	1	1014.5	75	K.TVGIDDLTGEPLIQR.E
KAD3_MOUSE	MK_SCX_19.4364.4364.2	2	4.182	0.554	1	1027	67.85714	K.AYEAQTEPVLQYYQK.K
KAD3_MOUSE	MK_SCX_21.11534.11534.3	3	3.578	0.363	1	439.1	24.074074	R.TLPQAEALDKVYQIDTVINLNVPFEVIK.Q
KAD3_MOUSE	MK_SCX_21.5299.5299.2	2	4.587	0.443	1	1243.9	80.769226	R.QNMLQGTEIGVLAK.T
KAD3_MOUSE	MK_SCX_2201.4486.4486.2	2	3.736	0.473	1	1537.1	88.88889	R.VYNIEFNPPK.T
KAD3_MOUSE	MK_SCX_2201.7935.7935.3	3	5.233	0.594	1	1873.9	44.791664	R.VYNIEFNPPKTVGIDDLTGEPLIQR.E
KAD3_MOUSE	MK_SCX_30.5941.5941.3	3	5.15	0.465	1	989.1	31.25	K.TVGIDDLTGEPLIQREDDKPETVIK.R
KAD3_MOUSE	MK_SCX_31.4826.4826.2	2	5.556	0.556	1	2140.2	68.75	R.LKAYEAQTEPVLQYYQK.K
KAD3_MOUSE	MK_SCX_32.4791.4791.3	3	5.927	0.486	1	1788.2	50	R.LKAYEAQTEPVLQYYQK.K
KAD3_MOUSE	MK_SCX_36.10478.10478.3	3	3.923	0.363	1	698.4	34.375	K.GVLETFSGTETNKIWPHVYSFLQTK.V
KAD3_MOUSE	MK_SCX_38.6877.6877.2	2	2.388	0.362	1	348.6	63.636364	K.IWPHVYSFLQTK.V
KAD3_MOUSE	MK_SCX_42.5658.5658.3	3	5.846	0.52	1	849.5	27.000002	K.TVGIDDLTGEPLIQREDDKPETVIKR.L
KAD3_MOUSE	MK_SCX_44.6803.6803.3	3	4.431	0.521	1	769.7	38.636364	K.HLSSGDLLRQNMLQGTEIGVLAK.T
KAD3_MOUSE	MK_SCX_46.4600.4600.3	3	5.135	0.575	1	861.8	50	R.LKAYEAQTEPVLQYYQKK.G
KAD3_MOUSE	MK_SCX_51.8299.8299.3	3	5.392	0.445	1	1553	38	K.KGVLETFSGTETNKIWPHVYSFLQTK.V
KAD4_MOUSE	MK_SCX_18.7327.7327.3	3	6.187	0.68	1	780.1	30.000002	R.VYNLDFNPPQVQGIDDITGEPLVQQEDDKPEAVAAR.L
KAD4_MOUSE	MK_SCX_21.8168.8168.1	1	3.389	0.489	1	595.1	63.636364	R.IWPYVYTLFSNK.I
KAD4_MOUSE	MK_SCX_21.8194.8194.2	2	4.012	0.501	1	773.2	86.36364	R.IWPYVYTLFSNK.I
KAD4_MOUSE	MK_SCX_2201.3254.3254.2	2	3.174	0.171	1	849.6	87.5	R.LMM*SELETR.S
KAD4_MOUSE	MK_SCX_2201.4035.4035.2	2	2.433	0.241	1	648.6	81.25	R.LMMSELETR.S
KAD4_MOUSE	MK_SCX_29.3966.3966.3	3	3.794	0.338	1	1062.3	60.000004	K.DAAKPVIELYK.S
KAD4_MOUSE	MK_SCX_29.3971.3971.2	2	2.732	0.321	1	496	75	K.DAAKPVIELYK.S
KAD4_MOUSE	MK_SCX_33.4399.4399.2	2	2.296	0.317	1	416.2	70	K.GLLVPDHVITR.L
KAD4_MOUSE	MK_SCX_39.3331.3331.3	3	3.626	0.468	1	589.8	45.833336	R.GVLHQFSGTETNR.I
KAD4_MOUSE	MK_SCX_41.5826.5826.2	2	4.105	0.47	1	1589.3	81.818184	R.SAQHWLLDGFPR.T
KAD4_MOUSE	MK_SCX_41.5839.5839.3	3	4.01	0.311	1	818.7	50	R.SAQHWLLDGFPR.T
KAD4_MOUSE	MK_SCX_50.5074.5074.2	2	5.964	0.516	1	2450	78.125	R.IAQNFGLQHLSSGHLLR.E
KAD4_MOUSE	MK_SCX_50.5095.5095.3	3	4.6	0.484	1	1175.4	48.4375	R.IAQNFGLQHLSSGHLLR.E
KAD4_MOUSE	MK_SCX_51.3666.3666.3	3	4.132	0.544	1	1256.1	60.416668	R.YKDAAKPVIELYK.S
KAD4_MOUSE	MK_SCX_51.3668.3668.2	2	4.547	0.457	1	1640.7	83.33333	R.YKDAAKPVIELYK.S
KAD4_MOUSE	MK_SCX_54.5320.5320.3	3	4.843	0.5	1	825.9	36.25	R.IAQNFGLQHLSSGHLLRENLK.T
KAD4_MOUSE	MK_SCX_54.5348.5348.2	2	5.69	0.59	1	1385.9	60.000004	R.IAQNFGLQHLSSGHLLRENLK.T
KAP2_MOUSE	MK_SCX_18.6546.6546.2	2	3.907	0.141	1	950.4	65.625	K.NLDQEQLSQVLDAM*FEK.I
KAP2_MOUSE	MK_SCX_18.8566.8566.2	2	5.736	0.589	1	2719	78.125	K.NLDQEQLSQVLDAMFEK.I
KAP2_MOUSE	MK_SCX_19.8337.8337.2	2	4.343	0.485	1	1484.9	83.33333	K.MFESFIESVPLFK.S
KAP2_MOUSE	MK_SCX_34.8322.8322.3	3	3.418	0.243	1	720.1	40.384613	R.KMFESFIESVPLFK.S
KAP2_MOUSE	MK_SCX_39.3865.3865.2	2	3.036	0.32	1	1026.3	85	R.NISHYEEQLVK.M
KBL_MOUSE	MK_SCX_18.6816.6816.2	2	4.533	0.318	1	887.9	50	K.ALGGASGGYTTGPEPLVSLLR.Q
KCC2B_MOUSE	MK_SCX_17.5843.5843.2	2	4.835	0.549	1	692.3	55.555557	K.AGAYDFPSPEWDTVTPEAK.N
KCD12_MOUSE	MK_SCX_19.6402.6402.2	2	2.65	0.304	1	638.2	68.181816	R.EAEYFELPELVR.R
KCD12_MOUSE	MK_SCX_33.3220.3220.3	3	4.471	0.556	1	2384	60.9375	R.LGAPQQPGPGPPPPHSR.R
KCRS_MOUSE	MK_SCX_13.6544.6544.2	2	3.95	0.503	1	717	66.66667	R.LGYILTCPSNLGTGLR.A
KCRS_MOUSE	MK_SCX_27.6393.6393.2	2	2.871	0.408	1	1059	81.25	R.GWEFMWNER.L
KCRU_MOUSE	MK_SCX_17.5871.5871.2	2	5.759	0.594	1	1975	60.000004	R.GTGGVDTAATGSVFDISNLDR.L
KCRU_MOUSE	MK_SCX_26.8391.8391.3	3	6.901	0.614	1	1376.5	26.51515	R.LSEMTEAEQQQLIDDHFLFDKPVSPLLTAAGMAR.D

KCRU_MOUSE	MK_SCX_29.5618.5618.2	2	3.863	0.505	1	1230	63.333332	R.VVVDALSGLKGDLAGR.Y
KCRU_MOUSE	MK_SCX_29.5623.5623.3	3	3.363	0.464	1	623.6	50	R.VVVDALSGLKGDLAGR.Y
KCRU_MOUSE	MK_SCX_31.5786.5786.3	3	3.866	0.396	1	689.4	30.952381	K.RGTGGVDTAATGSVFDISNLDR.L
KCRU_MOUSE	MK_SCX_31.5872.5872.2	2	6.873	0.626	1	1444.9	69.047615	K.RGTGGVDTAATGSVFDISNLDR.L
KCRU_MOUSE	MK_SCX_33.7035.7035.3	3	4.368	0.422	1	509	25	R.LCDKTTPTGWTLDQCIQTGVDNPGHPFIK.T
KCY_MOUSE	MK_SCX_21.2496.2496.2	2	2.921	0.35	1	406.2	63.636364	R.EMDQTMAANAQK.N
KCY_MOUSE	MK_SCX_21.4938.4938.2	2	4.246	0.494	1	1437.3	85	K.SVDEVFGEVVK.I
KCY_MOUSE	MK_SCX_24.5504.5504.2	2	3.1	0.248	1	754.1	100	K.FLIDGFPR.N
KCY_MOUSE	MK_SCX_25.7909.7909.2	2	5.618	0.618	1	1308.3	65.789474	R.IQTYLESTKPIIDLYEEMGK.V
KCY_MOUSE	MK_SCX_25.7981.7981.3	3	5.414	0.638	1	1051.4	44.736843	R.IQTYLESTKPIIDLYEEMGK.V
KCY_MOUSE	MK_SCX_35.11506.11506.3	3	4.8	0.509	1	621.9	32.142857	R.IQTYLESTKPIIDLYEEMGKVK.K
KCY_MOUSE	MK_SCX_38.5075.5075.3	3	4.854	0.509	1	1096.3	58.333332	K.YGYTHLSAGELLR.D
KCY_MOUSE	MK_SCX_38.5179.5179.2	2	4.096	0.451	1	1534.2	83.33333	K.YGYTHLSAGELLR.D
KCY_MOUSE	MK_SCX_42.7555.7555.3	3	6.124	0.691	1	1172.5	42.5	K.RIQTYLESTKPIIDLYEEMGK.V
KCY_MOUSE	MK_SCX_44.4916.4916.3	3	3.705	0.258	1	1606.8	66.66667	K.NKFLIDGFPR.N
KCY_MOUSE	MK_SCX_44.4921.4921.2	2	3.129	0.174	1	962.8	77.77778	K.NKFLIDGFPR.N
KGUA_MOUSE	MK_SCX_2201.15284.15284.3	3	3.582	0.441	1	464.9	32.142857	R.DIAAGDFIEHAEFSGNLYGTSK.E
KGUA_MOUSE	MK_SCX_49.3930.3930.3	3	4.181	0.501	1	1399.4	45.3125	R.NPRPGEEDGKDYYFVTR.E
KGUA_MOUSE	MK_SCX_49.3943.3943.2	2	4.523	0.555	1	1340.8	71.875	R.NPRPGEEDGKDYYFVTR.E
KHK_MOUSE	MK_SCX_13.6592.6592.2	2	5.303	0.426	1	1243.9	62.5	R.GGNASNSCTVLSLLGAR.C
KHK_MOUSE	MK_SCX_16.13179.13179.2	2	5.493	0.606	1	2092.4	67.5	R.VVDTLGAGDTFNASVIFSLSK.G
KHK_MOUSE	MK_SCX_17.9076.9076.2	2	4.753	0.572	1	998.5	62.5	R.CAFMGSLAPGHVADFLVADFR.Q
KHK_MOUSE	MK_SCX_18.5797.5797.2	2	5.268	0.611	1	1765.5	78.57143	R.TIILYDTNLPDVSAK.D
KHK_MOUSE	MK_SCX_21.15714.15714.3	3	4.677	0.574	1	779.6	35	R.VVDTLGAGDTFNASVIFSLSK.G
KHK_MOUSE	MK_SCX_25.6664.6664.2	2	5.08	0.357	1	1016.4	61.11111	R.TIILYDTNLPDVSAKDFEK.V
KHK_MOUSE	MK_SCX_31.13403.13403.3	3	3.119	0.267	1	480.4	29.347824	R.TIILYDTNLPDVSAKDFEKVDLTR.F
KI21A_MOUSE	MK_SCX_16.5421.5421.2	2	5.203	0.502	1	1421.4	68.75	R.DQVLQNLGSVESYSEEK.A
KI21A_MOUSE	MK_SCX_23.3322.3322.3	3	3.612	0.445	1	503.9	31.034481	K.LSSSESPAPDTGSSAASGEADTSRPGTQQK.M
KI21A_MOUSE	MK_SCX_24.14210.14210.3	3	3.01	0.189	1	317.4	30.000002	R.RVTDIIMQKM*TISNMEADM*NR.L
KI21A_MOUSE	MK_SCX_38.3187.3187.3	3	6.666	0.552	1	2381.4	40.833332	R.KLSSSESPAPDTGSSAASGEADTSRPGTQQK.M
KI21A_MOUSE	MK_SCX_44.6478.6478.3	3	3.757	0.255	1	531.4	32.894737	R.LKQTEITSATQNQLLFHMLK.E
KIF17_MOUSE	MK_SCX_2201.4929.4929.2	2	2.78	0.17	2	823.3	80	K.LNLVDLAGSER.Q
KIF17_MOUSE	MK_SCX_28.11366.11366.3	3	3.177	0.175	1	857.9	33.333336	K.INLSLSALGNVISALVDGR.C
KIF5A_MOUSE	MK_SCX_20_1.7053.7053.2	2	4.325	0.478	1	2071.9	83.33333	K.ISFLENNLEQLTK.V
KIF5A_MOUSE	MK_SCX_21.3448.3448.2	2	3.864	0.484	1	1024.5	72.72727	K.TGAEGAVLDEAK.N
KIF5A_MOUSE	MK_SCX_33.3351.3351.3	3	3.844	0.364	1	1195.2	48.214287	K.VSKTGAEGAVLDEAK.N
KINH_MOUSE	MK_SCX_13.3497.3497.2	2	5.564	0.576	1	1642.3	75	R.VFQSSTSQEQVYNDCAK.K
KINH_MOUSE	MK_SCX_21.4858.4858.2	2	3.397	0.298	1	770.8	66.66667	K.SATLASIDAELQK.L
KINH_MOUSE	MK_SCX_24.7513.7513.2	2	3.52	0.434	1	1259.7	77.27273	R.NTIQWLENELNR.W
KINH_MOUSE	MK_SCX_25.9120.9120.3	3	3.72	0.431	1	459.1	30.208334	K.SLSALGNVISALAEGSTYVPYRDSK.M
KINH_MOUSE	MK_SCX_33.5088.5088.3	3	3.251	0.422	1	447.3	35	K.LKTQMLDQEELLASTR.R
KINH_MOUSE	MK_SCX_41.4140.4140.2	2	2.677	0.338	1	1515.7	94.44444	R.KLFVQDLATR.V
KINH_MOUSE	MK_SCX_45.3384.3384.3	3	3.623	0.329	1	1251.6	57.5	R.FRPLNESEVNR.G
KINH_MOUSE	MK_SCX_46.7678.7678.3	3	3.449	0.142	1	1205.9	42.1875	K.GLEETVAKELQTLHNLR.K
KINH_MOUSE	MK_SCX_51.3382.3382.3	3	4.068	0.48	1	1067.6	51.785713	K.ALESALKEAKENASR.D
KIRR1_MOUSE	MK_SCX_16.4328.4328.2	2	5.042	0.457	1	2053.2	76.666664	R.DGTQQEGAVTSTELLK.D
KLC4_MOUSE	MK_SCX_19.4662.4662.2	2	5.415	0.511	1	1055.3	61.764706	R.ALAIYESQLGPDNPNVAR.T
KLC4_MOUSE	MK_SCX_52.5128.5128.2	2	2.838	0.391	1	383.7	57.14286	K.YKEAAHLLNDALSIR.E
KLC4_MOUSE	MK_SCX_53.5787.5787.3	3	3.595	0.375	1	580.8	33.333336	R.SEQAVAQLEEEKKHLEFLR.Q
KPRA_MOUSE	MK_SCX_12.6994.6994.2	2	2.36	0.211	1	418.2	46.153847	K.EKPPITVVGDVGGR.I
KPRA_MOUSE	MK_SCX_19.8506.8506.2	2	2.178	0.145	1	385.9	46.875	R.ASPFLLQYIQEEIPNYR.N

KPRA_MOUSE	MK_SCX_23.6358.6358.3	3	3.263	0.26	1	353	30.952381	R.LIEESPIDEVVVTNTVPHELQK.L
KPYM_MOUSE	MK_SCX_16.7020.7020.2	2	4.002	0.23	1	961.3	52.380955	R.LAPITSDPTEAAAVGAVEASFK.C
KPYM_MOUSE	MK_SCX_18.9236.9236.2	2	3.313	0.426	1	848.4	66.66667	K.FGVEQDVDM*VFASFIR.K
KPYM_MOUSE	MK_SCX_19.5248.5248.2	2	2.194	0.145	1	379.8	46.875	K.GVNLPGAAVDLPAVSEK.D
KPYM_MOUSE	MK_SCX_19.6138.6138.2	2	3.495	0.36	1	595.8	70.83333	K.IYVDDGLISLQVK.E
KPYM_MOUSE	MK_SCX_20_1.4273.4273.2	2	3.77	0.449	1	1082.3	85	R.LDIDSAPITAR.N
KPYM_MOUSE	MK_SCX_20_1.4635.4635.2	2	3.195	0.386	1	1061.1	80	R.GDLGIEIPAEK.V
KPYM_MOUSE	MK_SCX_2201.7331.7331.2	2	4.714	0.664	1	513.3	47.727272	R.EATESFASDPILYRPVAVALDTK.G
KPYM_MOUSE	MK_SCX_2201.7392.7392.3	3	4.051	0.54	1	1189.5	36.363636	R.EATESFASDPILYRPVAVALDTK.G
KPYM_MOUSE	MK_SCX_27.5341.5341.3	3	3.568	0.208	1	1125.6	38.157894	K.EKGADFLVTEVENGGSLGSK.K
KPYM_MOUSE	MK_SCX_32.5306.5306.2	2	5.304	0.45	1	1436.6	73.52941	K.KGVNLPGAAVDLPAVSEK.D
KPYM_MOUSE	MK_SCX_35.3523.3523.3	3	3.337	0.255	1	795.7	46.153847	R.APIIAVTRNPQTAR.Q
KPYM_MOUSE	MK_SCX_38.8554.8554.3	3	5.512	0.452	1	1539.2	40.217392	K.KGVNLPGAAVDLPAVSEKDIQDLK.F
KPYM_MOUSE	MK_SCX_48.3499.3499.3	3	3.283	0.438	1	731.9	46.666668	R.TGLIKGSGTAEVELKK.G
KTN1_MOUSE	MK_SCX_15.6299.6299.2	2	5.347	0.584	1	751.2	44.230766	K.LSDASPAEDEQFVPAPLNVAETSSSVR.E
KTN1_MOUSE	MK_SCX_28.8007.8007.3	3	3.022	0.126	1	387.1	25	K.AEVQKWQALANEQAATAHEVEKMQK.S
KTN1_MOUSE	MK_SCX_29.13454.13454.3	3	3.472	0.249	1	605.9	35.294117	R.LTQEMMTEKERSSVVIAR.M
L2HDH_MOUSE	MK_SCX_20_1.8397.8397.2	2	2.243	0.232	1	500.2	77.27273	K.FIPEITISDVLR.G
L2HDH_MOUSE	MK_SCX_21.4085.4085.2	2	3.294	0.61	1	587.5	71.42857	R.NAPSPAATSSLAISR.M
L2HDH_MOUSE	MK_SCX_23.7368.7368.3	3	5.183	0.563	1	1667	34	R.AQALDRDGNLVEDFVFDGGTGEIADR.V
L2HDH_MOUSE	MK_SCX_23.7614.7614.2	2	2.503	0.428	1	387.8	38	R.AQALDRDGNLVEDFVFDGGTGEIADR.V
LA_MOUSE	MK_SCX_19.6795.6795.2	2	4.119	0.4	1	684	75	K.LDEGWVPLETMIK.F
LA_MOUSE	MK_SCX_19.7699.7699.2	2	4.702	0.45	1	936.7	76.92308	R.LTTDFNVIVQALSK.S
LA_MOUSE	MK_SCX_41.7896.7896.3	3	3.157	0.151	1	390.6	28.947369	K.FLKEQIKLDEGWVPLETMIK.F
LA_MOUSE	MK_SCX_55.3702.3702.3	3	4.728	0.527	1	925.4	41.666664	R.RSPSRPLPEVTDEYKNDVK.N
LACTB_MOUSE	MK_SCX_21.6334.6334.2	2	3.488	0.346	1	914.5	67.85714	K.FGNAMLYGYQVGQFK.N
LACTB_MOUSE	MK_SCX_21.9075.9075.2	2	4.868	0.476	1	2410.7	85.71429	K.WAGGGFLSTVGDLLK.F
LACTB_MOUSE	MK_SCX_23.4495.4495.2	2	3.228	0.494	1	1373.6	88.88889	K.YAMAWGVVEK.K
LACTB_MOUSE	MK_SCX_31.7372.7372.3	3	4.192	0.473	1	540.3	34.090908	K.LDLDLPVQHYVPEFPEKEYEGEK.V
LACTB_MOUSE	MK_SCX_40.4464.4464.2	2	2.577	0.366	1	1196.8	83.33333	R.LLISHLSGIR.H
LACTB_MOUSE	MK_SCX_51.3894.3894.3	3	3.611	0.359	1	822.2	50	K.KNDFEQGELYLKEK.F
LAD1_MOUSE	MK_SCX_17.4079.4079.2	2	4.128	0.572	1	798.3	73.333336	R.DSLGPEQTSSQPLVPK.K
LAD1_MOUSE	MK_SCX_20_1.2976.2976.2	2	3.747	0.569	1	1258.6	66.66667	K.SPPSSAEQSTPAPPTK.A
LAD1_MOUSE	MK_SCX_20_1.4042.4042.2	2	3.576	0.391	1	1045.3	75	R.TEVLVTPAGVASK.R
LAD1_MOUSE	MK_SCX_25.4073.4073.3	3	3.338	0.527	1	824.5	35.526314	R.QVVEAVQAPVQERPEAEEER.D
LAD1_MOUSE	MK_SCX_27.3583.3583.2	2	4.412	0.533	1	814.9	57.5	R.ALPDKSPPSSAEQSTPAPPTK.A
LAD1_MOUSE	MK_SCX_28.3497.3497.2	2	2.633	0.164	1	1271.4	52.63158	R.NLSSTTDDESPKLTQNGAQR.S
LAD1_MOUSE	MK_SCX_42.3533.3533.3	3	3.791	0.408	1	890.9	38.235294	R.ELAEGEKRLPEETVAQQK.T
LAD1_MOUSE	MK_SCX_43.4578.4578.3	3	3.296	0.357	1	1185.4	67.5	R.KDWSALSSLAR.Q
LAD1_MOUSE	MK_SCX_43.4618.4618.2	2	2.645	0.396	1	1095	85	R.KDWSALSSLAR.Q
LAD1_MOUSE	MK_SCX_44.3724.3724.3	3	4.816	0.556	1	874.2	37.5	R.RQVVEAVQAPVQERPEAEEER.D
LAD1_MOUSE	MK_SCX_45.3431.3431.3	3	5.529	0.564	1	1908.6	45.454548	R.GRALPDKSPPSSAEQSTPAPPTK.A
LAD1_MOUSE	MK_SCX_49.2699.2699.3	3	3.121	0.264	1	623.2	30.952381	K.KAAVSEQPQTTGGSQATTREPR.G
LAMA4_MOUSE	MK_SCX_30.6693.6693.3	3	3.214	0.323	1	395.8	35	K.LSDLQESINQALDHVR.D
LAMA4_MOUSE	MK_SCX_30.7005.7005.3	3	3.017	0.278	1	901.8	39.473686	K.KIPFTDIYIGGAPQEVLQSR.T
LAMA5_MOUSE	MK_SCX_13.4261.4261.2	2	2.516	0.341	1	605.2	67.85714	R.ITQDDDVICTTEYSR.I
LAMA5_MOUSE	MK_SCX_14.3110.3110.2	2	3.631	0.375	1	1731.1	76.92308	R.ITASATCGEEAPTR.S
LAMA5_MOUSE	MK_SCX_16.5454.5454.2	2	5.358	0.54	1	2958.3	76.47059	R.DLGAQGAVAEAELAEAQR.L
LAMA5_MOUSE	MK_SCX_17.6106.6106.2	2	5.65	0.746	1	1967.7	56.25	R.LGSQATGVQGQAGQLLDTTESTLGR.A
LAMA5_MOUSE	MK_SCX_17.6133.6133.3	3	4.294	0.413	1	1087	35.416664	R.LGSQATGVQGQAGQLLDTTESTLGR.A
LAMA5_MOUSE	MK_SCX_18.6520.6520.2	2	4.155	0.52	1	625.1	55.263157	K.ATGDPWLTDGSYLDGSGFAR.I

LAMA5_MOUSE	MK_SCX_2201.9193.9193.2	2	3.727	0.489	1	824.2	65.38461	R.FGFNPLEFENFSWR.G
LAMA5_MOUSE	MK_SCX_27.4751.4751.2	2	2.492	0.427	1	989.1	87.5	R.WWQSPPLSR.G
LAMB1_MOUSE	MK_SCX_16.9454.9454.2	2	3.69	0.53	1	538.3	42.857143	R.NFLTEDSADLDSIEAVANEVLK.S
LAMB1_MOUSE	MK_SCX_25.10281.10281.3	3	3.37	0.407	1	793.4	35.526314	K.AMDFDRDVLSALAEVEQLSK.M
LAMB1_MOUSE	MK_SCX_25.5373.5373.2	2	3.228	0.397	1	917.9	85	R.IPSWTGPGFVR.V
LAMB1_MOUSE	MK_SCX_30.3445.3445.2	2	4.309	0.383	1	641.9	64.28571	K.EALEEAEKAQVAAEK.A
LAMB1_MOUSE	MK_SCX_33.4313.4313.2	2	3.252	0.34	1	547.4	77.77778	R.DRVEDLMLER.E
LAMB1_MOUSE	MK_SCX_53.3867.3867.3	3	3.039	0.255	1	1311.3	44.642857	R.NCEQCKPFYFQHPER.D
LAMB2_MOUSE	MK_SCX_15.6229.6229.2	2	3.527	0.343	1	1061	66.66667	K.DFLSQEGADPDSIEMVATR.V
LAMB2_MOUSE	MK_SCX_16.7821.7821.3	3	3.027	0.138	1	373.7	25	K.LGM*VQAIMSARNASAASTAKLVEATEGLR.H
LAMB2_MOUSE	MK_SCX_20_1.3629.3629.2	2	5.883	0.691	1	2186.6	63.15789	R.AGNSLAASTAEETAGSAQSR.A
LAMB2_MOUSE	MK_SCX_2201.8320.8320.3	3	3.158	0.418	1	329.7	24	R.ELIQNVKDFLSQEGADPDSIEM*VATR.V
LAMB2_MOUSE	MK_SCX_38.3412.3412.3	3	3.932	0.458	1	1059.9	50	K.QLREQVGDQYQTVR.A
LAMC1_MOUSE	MK_SCX_17.5877.5877.2	2	3.429	0.468	1	2553.8	78.125	R.TFGEVTDLDNEVNGMLR.Q
LAMC1_MOUSE	MK_SCX_18.5072.5072.2	2	5.669	0.573	1	1062.8	58.333332	R.VSVPLIAQGNSYPSETTVK.Y
LAMC1_MOUSE	MK_SCX_19.6418.6418.2	2	2.918	0.232	1	865.6	69.230774	R.LSAEDLVLEGAGLR.V
LAMC1_MOUSE	MK_SCX_29.3631.3631.3	3	3.463	0.511	1	845.8	46.42857	R.EAQEVKDVDQNLM*DR.L
LAMC1_MOUSE	MK_SCX_36.5080.5080.3	3	3.2	0.372	1	851.5	42.857143	R.SRVESTEQLIEIASR.E
LAMC1_MOUSE	MK_SCX_37.3430.3430.2	2	4.534	0.552	1	2493.2	84.61539	K.GKAEQQTADQLLAR.A
LAMC1_MOUSE	MK_SCX_43.6834.6834.3	3	3.135	0.558	1	577.9	35.526314	R.LHEATDYPWRPALSPFEFQK.L
LAMC1_MOUSE	MK_SCX_49.8235.8235.3	3	4.074	0.451	1	1299.7	45.3125	R.DIAEIIKDIHNLEDIKK.T
LAMP2_MOUSE	MK_SCX_23.5221.5221.2	2	3.423	0.277	1	1192.6	83.33333	R.AFQINTFNLK.V
LAP2A_MOUSE	MK_SCX_19.4888.4888.2	2	3.669	0.431	1	1533.7	81.818184	-.PEFLEDPSVLTK.D
LAP2A_MOUSE	MK_SCX_21.8203.8203.3	3	5.384	0.387	1	1640.6	39.772728	R.LEDKDDLDVTELSNEELLDQLVR.Y
LAP2B_MOUSE	MK_SCX_18.5235.5235.2	2	3.266	0.415	1	574.7	59.375	R.IDGAVISESTPIAETIK.A
LAP2B_MOUSE	MK_SCX_33.6830.6830.2	2	4.892	0.676	1	1614.4	86.666664	K.HASSILPITEFSDITR.R
LARP4_MOUSE	MK_SCX_30.3650.3650.3	3	3.658	0.327	1	599.5	34.523808	R.SSSGSEHSTEGSVSLGDGPLSR.S
LARP4_MOUSE	MK_SCX_36.3642.3642.2	2	4.066	0.501	1	845.3	70	R.HNPTVTGQQEQTYLPK.E
LASP1_MOUSE	MK_SCX_20_1.4437.4437.2	2	2.532	0.345	1	440	53.846157	K.QSFTM*VADTPENLR.L
LASP1_MOUSE	MK_SCX_21.4845.4845.2	2	3.764	0.421	1	1211.4	70.83333	K.GFSVVADTPELQR.I
LASP1_MOUSE	MK_SCX_21.4909.4909.2	2	3.8	0.488	1	619.3	61.538464	K.QSFTMVADTPENLR.L
LASP1_MOUSE	MK_SCX_36.4207.4207.3	3	3.712	0.254	1	1249.7	46.42857	K.GKGFSVVADTPELQR.I
LASP1_MOUSE	MK_SCX_36.4219.4219.2	2	5.293	0.543	1	2125.6	78.57143	K.GKGFSVVADTPELQR.I
LASP1_MOUSE	MK_SCX_41.2763.2763.3	3	4.399	0.235	1	1128	56.81818	R.LKQQSELQSQVR.Y
LASP1_MOUSE	MK_SCX_41.2784.2784.2	2	3.114	0.334	1	992.7	72.72727	R.LKQQSELQSQVR.Y
LASP1_MOUSE	MK_SCX_47.3995.3995.2	2	5.477	0.567	1	1523.4	70	K.TQDQISNIKYHEEFEK.S
LASP1_MOUSE	MK_SCX_47.4058.4058.3	3	3.151	0.466	1	378.5	41.666664	K.TQDQISNIKYHEEFEK.S
LASP1_MOUSE	MK_SCX_52.3760.3760.3	3	4.845	0.406	1	867.3	40.625	K.NKGKGFSVVADTPELQR.I
LAT2_MOUSE	MK_SCX_31.3367.3367.3	3	6.245	0.488	1	1268.6	38.636364	K.NHPGSDTSPEAEASSGGGGVALK.K
LAT2_MOUSE	MK_SCX_45.3260.3260.3	3	4.701	0.389	1	447	39.130436	K.NHPGSDTSPEAEASSGGGGVALKK.E
LC7L2_MOUSE	MK_SCX_20_1.3231.3231.2	2	5.394	0.555	1	2347.4	82.14286	K.VEQLGAEGNVEESQK.V
LC7L2_MOUSE	MK_SCX_2201.4738.4738.2	2	3.072	0.338	1	675.8	70	R.AMLDQLMGTSR.D
LC7L2_MOUSE	MK_SCX_42.6000.6000.2	2	2.49	0.335	1	839.6	93.75	K.LHLGFIEIR.E
LC7L2_MOUSE	MK_SCX_52.4181.4181.3	3	3.403	0.265	1	990.6	45	K.SHLLNCCPHDVLSGTR.M
LDHA_MOUSE	MK_SCX_25.6017.6017.3	3	3.795	0.455	1	1112.5	42.1875	K.DQLIVNLLKEEQAPQNK.I
LDHA_MOUSE	MK_SCX_28.4594.4594.3	3	4.75	0.49	2	856.5	42.1875	K.GEM*M*DLQHGSLFLQTPK.I
LDHA_MOUSE	MK_SCX_28.5954.5954.2	2	5.087	0.549	1	1904.2	68.75	K.GEMMDLQHGSLFLQTPK.I
LDHA_MOUSE	MK_SCX_30.3968.3968.2	2	4.976	0.52	1	1724.9	76.666664	K.SLNPELGTDADKEQWK.E
LDHA_MOUSE	MK_SCX_44.12436.12436.3	3	3.737	0.415	1	402	36.11111	K.LKGEMMDLQHGSLFLQTPK.I
LDHA_MOUSE	MK_SCX_44.4482.4482.3	3	3.584	0.646	2	549.9	34.72222	K.LKGEM*M*DLQHGSLFLQTPK.I
LDHA_MOUSE	MK_SCX_44.5879.5879.2	2	5.959	0.63	1	2169.4	69.44444	K.LKGEMMDLQHGSLFLKTPK.I

LDHA_MOUSE	MK_SCX_49.4099.4099.3	3	3.463	0.373	1	748.9	41.07143	K.EVHKQVVDSAYEVIK.L
LDHA_MOUSE	MK_SCX_52.3791.3791.3	3	3.053	0.457	1	488	38.157894	K.SLNPELGTDADKEQWKEVHK.Q
LDHB_MOUSE	MK_SCX_17.7905.7905.2	2	4.844	0.524	1	1823.4	75	K.SLADELALVDVLEDK.L
LDHB_MOUSE	MK_SCX_17.8052.8052.1	1	4.107	0.415	1	1249.9	60.714287	K.SLADELALVDVLEDK.L
LDHB_MOUSE	MK_SCX_19.3989.3989.2	2	4.313	0.454	1	864.8	76.666664	K.LIASVADDEAAVPNNK.I
LDHB_MOUSE	MK_SCX_21.4730.4730.2	2	4.349	0.603	1	1091.1	90	K.MVVDSAYEVIK.L
LDHB_MOUSE	MK_SCX_23.3634.3634.2	2	3.456	0.421	1	873.8	93.75	K.IVVVTAGVR.Q
LDHB_MOUSE	MK_SCX_24.3560.3560.2	2	2.886	0.332	1	403.3	81.25	R.GLTSVINQK.L
LDHB_MOUSE	MK_SCX_25.9224.9224.2	2	5.561	0.508	1	3161.4	81.25	K.SLADELALVDVLEDKLK.G
LDHB_MOUSE	MK_SCX_25.9375.9375.3	3	3.327	0.469	1	430.1	39.0625	K.SLADELALVDVLEDKLK.G
LDHB_MOUSE	MK_SCX_28.4594.4594.3	3	4.75	0.49	2	856.5	42.1875	K.GEM*M*DLQHGSLFLQTPK.I
LDHB_MOUSE	MK_SCX_28.5954.5954.2	2	5.087	0.549	1	1904.2	68.75	K.GEMMDLQHGSLFLQTPK.I
LDHB_MOUSE	MK_SCX_29.9131.9131.3	3	3.41	0.359	1	851.7	42.857143	K.SADTLWDIQKDLK.D
LDHB_MOUSE	MK_SCX_32.3026.3026.2	2	4.365	0.511	1	1831.4	84.61539	K.IVADKDYSVTANSK.I
LDHB_MOUSE	MK_SCX_32.3381.3381.3	3	3.609	0.459	1	343.5	40.384613	K.IVADKDYSVTANSK.I
LDHB_MOUSE	MK_SCX_32.5545.5545.2	2	3.912	0.318	1	1542.3	83.33333	K.SADTLWDIQKDLK.D
LDHB_MOUSE	MK_SCX_39.4025.4025.2	2	3.517	0.358	1	1686	90	R.KSADTLWDIQK.D
LDHB_MOUSE	MK_SCX_44.12436.12436.3	3	3.737	0.415	1	402	36.11111	K.LKGEMMDLQHGSLFLQTPK.I
LDHB_MOUSE	MK_SCX_44.4482.4482.3	3	3.584	0.646	2	549.9	34.72222	K.LKGEM*M*DLQHGSLFLQTPK.I
LDHB_MOUSE	MK_SCX_44.5581.5581.3	3	3.025	0.198	1	389.5	31.944445	R.GLTSVINQKLKDDEVAQLR.K
LDHB_MOUSE	MK_SCX_44.5879.5879.2	2	5.959	0.63	1	2169.4	69.44444	K.LKGEMMDLQHGSLFLKTPK.I
LDHB_MOUSE	MK_SCX_50.4985.4985.3	3	4.455	0.354	1	502.9	46.153847	R.KSADTLWDIQKDLK.D
LDHB_MOUSE	MK_SCX_50.4992.4992.2	2	5.133	0.379	1	2300.2	84.61539	R.KSADTLWDIQKDLK.D
LDHB_MOUSE	MK_SCX_51.2880.2880.2	2	2.597	0.262	1	500.6	75	K.LKDDEVAQLRK.S
LDHB_MOUSE	MK_SCX_51.2894.2894.3	3	3.693	0.424	1	770.6	57.5	K.LKDDEVAQLRK.S
LEG3_MOUSE	MK_SCX_33.4437.4437.3	3	3.541	0.341	1	489.4	47.5	K.IQVLVEADHFK.V
LEG3_MOUSE	MK_SCX_50.3654.3654.3	3	3.86	0.576	1	541.6	48.076923	K.VAVNDAHLLQYNHR.M
LETM1_MOUSE	MK_SCX_16.7120.7120.2	2	4.677	0.674	1	840.1	50	R.VQQM*IGQIDGLITQLETTQQDGK.L
LETM1_MOUSE	MK_SCX_16.7124.7124.3	3	5.304	0.492	1	1822	39.772728	R.VQQM*IGQIDGLITQLETTQQDGK.L
LETM1_MOUSE	MK_SCX_16.8102.8102.2	2	5.111	0.586	1	2146.7	61.363636	R.VQQMIGQIDGLITQLETTQQDGK.L
LETM1_MOUSE	MK_SCX_16.8104.8104.3	3	6.277	0.579	1	2143.9	42.045452	R.VQQMIGQIDGLITQLETTQQDGK.L
LETM1_MOUSE	MK_SCX_16.8475.8475.2	2	4.808	0.679	1	939.5	54.347824	K.LGPSQSTPTGESVISITELISAMK.Q
LETM1_MOUSE	MK_SCX_18.4746.4746.2	2	4.98	0.529	1	1463	78.125	K.LEEGGPVYSPPAQVVVR.K
LETM1_MOUSE	MK_SCX_18.5150.5150.2	2	4.497	0.52	1	891.5	75	R.AM*YLPDTLSPADQLK.S
LETM1_MOUSE	MK_SCX_18.5653.5653.2	2	4.387	0.611	1	1065.8	78.57143	R.AMYLPDTLSPADQLK.S
LETM1_MOUSE	MK_SCX_20_1.5020.5020.2	2	2.9	0.337	1	1024.9	72.72727	K.STLQTLPEIVAK.E
LETM1_MOUSE	MK_SCX_20_1.5239.5239.2	2	3.668	0.382	1	2379.6	90.909096	K.FLQDTIEEM*ALK.N
LETM1_MOUSE	MK_SCX_20_1.5653.5653.2	2	2.032	0.317	1	390.5	71.42857	K.DFSAFFQK.I
LETM1_MOUSE	MK_SCX_20_1.6868.6868.2	2	3.477	0.311	1	1436.2	81.818184	K.FLQDTIEEMALK.N
LETM1_MOUSE	MK_SCX_23.7660.7660.3	3	6.194	0.57	1	1277.5	45.454548	K.LISLTSALDDNKDGNINIDDLVK.V
LETM1_MOUSE	MK_SCX_23.7668.7668.2	2	5.375	0.557	1	1865.6	56.81818	K.LISLTSALDDNKDGNINIDDLVK.V
LETM1_MOUSE	MK_SCX_28.4352.4352.3	3	4.122	0.424	1	954	41.17647	K.LEEGGPVYSPPAQVVVRK.S
LETM1_MOUSE	MK_SCX_31.4583.4583.2	2	6.223	0.499	1	2090	82.35294	K.KLEEGGPVYSPPAQVVVR.K
LETM1_MOUSE	MK_SCX_31.4610.4610.3	3	4.11	0.521	1	1469.1	52.941177	K.KLEEGGPVYSPPAQVVVR.K
LETM1_MOUSE	MK_SCX_36.5828.5828.3	3	3.124	0.293	1	514.7	33.75	K.LEATLQEEAAIQQEHLEELKR.A
LETM1_MOUSE	MK_SCX_41.5079.5079.3	3	5.263	0.523	1	2588.9	51.315792	R.SIKADDKLISEEGVDSLTVK.E
LETM1_MOUSE	MK_SCX_45.4260.4260.3	3	5.051	0.512	1	2506.9	50	K.KLEEGGPVYSPPAQVVVRK.S
LETM1_MOUSE	MK_SCX_53.2996.2996.3	3	3.749	0.308	1	1236.2	46.42857	K.IRETGERPSNEEIMR.F
LGMN_MOUSE	MK_SCX_13.3857.3857.2	2	3.056	0.405	1	1090.7	86.36364	K.ESSYACYYDEER.G
LGMN_MOUSE	MK_SCX_16.7803.7803.2	2	4.531	0.325	1	813.5	62.5	K.DYTGEDVTPENFLAVLR.G
LGMN_MOUSE	MK_SCX_26.8020.8020.2	2	6.122	0.652	1	2162.6	70	K.GVLKDYTGEDVTPENFLAVLR.G

LGMN_MOUSE	MK_SCX_26.8087.8087.3	3	4.046	0.406	1	1290.3	40	K.GVLKDYTGEDVTPENFLAVLR.G
LGUL_MOUSE	MK_SCX_17.8703.8703.2	2	6.486	0.472	1	1739.9	71.05263	K.GLAFIQDPDGYWIEILNPNK.I
LGUL_MOUSE	MK_SCX_2201.4171.4171.2	2	2.093	0.193	1	381.4	83.33333	K.LDFPAMK.F
LGUL_MOUSE	MK_SCX_2201.5182.5182.2	2	3.364	0.529	1	812.8	93.75	R.VLGLTLLQK.L
LHR2A_MOUSE	MK_SCX_19.4783.4783.2	2	4.31	0.507	1	1337	88.46153	R.YVQELPLETDGALR.Y
LHR2A_MOUSE	MK_SCX_2201.6435.6435.2	2	3.144	0.381	1	1080	88.88889	R.YLLPAILNPR.Y
LIAS_MOUSE	MK_SCX_19.6691.6691.2	2	3.212	0.57	1	613.6	50	K.VGNELGFLYTASGPLVR.S
LIMA1_MOUSE	MK_SCX_21.5296.5296.2	2	3.639	0.401	1	587.7	70.83333	K.SQDVGFWEGEVVR.E
LIMA1_MOUSE	MK_SCX_29.4290.4290.3	3	6.076	0.583	1	945.7	29.83871	K.SDNEETLGRPAQPPNAGESPHSPGVEDAPIAK.V
LIMA1_MOUSE	MK_SCX_34.5116.5116.3	3	3.784	0.421	1	1143.5	37.5	R.KGWSESEQSEEFGGGIATMER.K
LIMA1_MOUSE	MK_SCX_40.3294.3294.2	2	2.81	0.384	1	597	68.181816	R.FGSRPEAVIQSR.Y
LIMA1_MOUSE	MK_SCX_46.3939.3939.3	3	3.103	0.293	1	724.3	45	R.SRPFTVAASFR.T
LIMA1_MOUSE	MK_SCX_46.3964.3964.2	2	3.641	0.556	1	681.5	80	R.SRPFTVAASFR.T
LIMA1_MOUSE	MK_SCX_47.2786.2786.3	3	3.123	0.452	1	686.6	40.625	K.SEAQQPM*HPKPLSPDAR.T
LIMA1_MOUSE	MK_SCX_47.3094.3094.3	3	3.35	0.445	1	505.5	35.9375	K.SEAQQPMHPKPLSPDAR.T
LIN7C_MOUSE	MK_SCX_2201.2751.2751.2	2	2.456	0.135	1	706.6	92.85714	K.VLEEMESR.F
LIN7C_MOUSE	MK_SCX_26.6256.6256.3	3	5.937	0.424	1	1690.5	45.454548	K.TEEGLGFNIMGGKEQNSPIYISR.I
LIN7C_MOUSE	MK_SCX_26.6272.6272.2	2	4.907	0.413	1	1319.2	52.272724	K.TEEGLGFNIMGGKEQNSPIYISR.I
LIN7C_MOUSE	MK_SCX_27.6600.6600.3	3	4.741	0.436	1	1787.6	45.833336	R.VVELPKTEEGLGFNIMGGK.E
LIPB1_MOUSE	MK_SCX_13.10481.10481.2	2	2.19	0.169	1	324.7	35	K.RDAM*ELPDYVLLTATAKVKPK.K
LIPB1_MOUSE	MK_SCX_23.6024.6024.3	3	3.062	0.329	1	512.5	37.5	R.LYEEDDLDRLEQMEDSEGTVR.Q
LIPL_MOUSE	MK_SCX_17.5214.5214.2	2	4.922	0.547	1	1318.3	69.44444	R.ITGLDPAGPNFEYAEAPSR.L
LMAN1_MOUSE	MK_SCX_2201.4068.4068.2	2	3.213	0.334	1	1142	85	R.YVSSLTEEISR.R
LMAN1_MOUSE	MK_SCX_29.5994.5994.3	3	3.461	0.537	1	967.8	38.157894	K.GHPDLQGQPADDIFESIGDR.E
LMAN2_MOUSE	MK_SCX_14.5299.5299.2	2	2.728	0.291	1	395.8	63.636364	R.WSELAGCTADFR.N
LMAN2_MOUSE	MK_SCX_23.6707.6707.3	3	6.366	0.651	1	1987.4	40	R.LPTGYYFGASAGTGDLSDNHDIISIK.L
LMAN2_MOUSE	MK_SCX_33.4249.4249.2	2	2.492	0.254	1	817.9	68.181816	R.DRLVPGPVFGSK.D
LMCD1_MOUSE	MK_SCX_20_1.4024.4024.2	2	3.49	0.607	1	698.6	76.92308	K.GAAPVDSPVVYADR.A
LMCD1_MOUSE	MK_SCX_21.7072.7072.2	2	2.539	0.188	1	315.5	55	K.LGLQYMELIPK.E
LMCD1_MOUSE	MK_SCX_39.3403.3403.3	3	3.618	0.456	1	821.5	51.923077	K.ERQPVTGTEGALYR.R
LMNA_MOUSE	MK_SCX_19.5986.5986.2	2	5.113	0.451	1	2421.7	85.71429	R.M*QQQLDEYQELLDIK.L
LMNA_MOUSE	MK_SCX_19.6189.6189.2	2	5.395	0.572	1	2432.8	82.14286	R.MQQQLDEYQELLDIK.L
LMNA_MOUSE	MK_SCX_19.7538.7538.2	2	2.393	0.143	1	316.2	45.833336	K.SNEDQSMGNWQIR.R
LMNA_MOUSE	MK_SCX_20_1.3832.3832.2	2	3.42	0.247	1	931.6	61.538464	R.TALINSTGEEVAM*R.K
LMNA_MOUSE	MK_SCX_20_1.5244.5244.2	2	3.694	0.244	1	1214.7	75	R.IDSLSAQLSQLQK.Q
LMNA_MOUSE	MK_SCX_2201.2822.2822.2	2	3.011	0.344	1	1197.9	88.88889	R.ITESEEVVSR.E
LMNA_MOUSE	MK_SCX_2201.3987.3987.2	2	4.898	0.465	1	1299.1	59.375	R.SVGGSGGGSFGDNLVTR.S
LMNA_MOUSE	MK_SCX_25.6855.6855.3	3	4.263	0.364	1	701.8	32.291664	K.AGQVVTIWASGAGATHSPPTDLVWK.A
LMNA_MOUSE	MK_SCX_31.4056.4056.2	2	4.44	0.51	1	1876.3	80.769226	R.VAVEEVDEEGKFVR.L
LMNA_MOUSE	MK_SCX_31.4087.4087.3	3	3.343	0.462	1	726.9	53.846157	R.VAVEEVDEEGKFVR.L
LMNA_MOUSE	MK_SCX_33.3549.3549.2	2	5.112	0.462	1	2478.6	87.5	R.LQEKEDLQELNDR.L
LMNA_MOUSE	MK_SCX_33.3565.3565.3	3	4.233	0.251	1	1416	56.25	R.LQEKEDLQELNDR.L
LMNA_MOUSE	MK_SCX_34.3737.3737.2	2	5.671	0.623	1	1466.4	76.666664	R.NSNLVGAAHEELQQSR.I
LMNA_MOUSE	MK_SCX_34.4409.4409.2	2	3.859	0.424	1	1318.4	77.27273	R.LQTLKEELDFQK.N
LMNA_MOUSE	MK_SCX_34.4447.4447.3	3	3.02	0.433	1	531	52.272724	R.LQTLKEELDFQK.N
LMNA_MOUSE	MK_SCX_35.8480.8480.3	3	3.749	0.242	1	1036	46.42857	R.IRIDSLSAQLSQLQK.Q
LMNA_MOUSE	MK_SCX_35.9500.9500.2	2	3.352	0.361	1	396.7	64.28571	R.IRIDSLSAQLSQLQK.Q
LMNA_MOUSE	MK_SCX_37.5437.5437.3	3	3.73	0.288	1	1051	55	R.LKDLEALLNSK.E
LMNA_MOUSE	MK_SCX_37.5549.5549.2	2	3.243	0.135	1	1049.6	80	R.LKDLEALLNSK.E
LMNA_MOUSE	MK_SCX_39.3598.3598.2	2	2.616	0.249	1	1099.8	88.88889	K.LRDLEDSLAR.E
LMNA_MOUSE	MK_SCX_41.3714.3714.3	3	5.483	0.388	1	1376.4	51.785713	R.NKSNEDQSMGNWQIR.R

LMNA_MOUSE	MK_SCX_41.3734.3734.2	2	4.65	0.486	1	1603.4	78.57143	R.NKSNEDQSMGNWQIR.R
LMNA_MOUSE	MK_SCX_45.5024.5024.2	2	2.064	0.203	1	337.4	53.846157	K.SNEDQSMGNWQIRR.Q
LMNA_MOUSE	MK_SCX_49.4883.4883.2	2	4.272	0.556	1	1050.7	67.85714	R.TLEGELHDLRGQVAK.L
LMNA_MOUSE	MK_SCX_55.3405.3405.3	3	3.732	0.445	1	1405	53.333336	R.AQHEDQVEQYKKELEK.T
LMNB1_MOUSE	MK_SCX_15.4152.4152.2	2	2.539	0.174	1	1150	80	K.NMYEEEINETR.R
LMNB1_MOUSE	MK_SCX_15.5526.5526.2	2	4.105	0.57	1	772.7	45.454548	K.TTIPEEEEEEEEEPIGVAVEEER.F
LMNB1_MOUSE	MK_SCX_16.7273.7273.2	2	6.271	0.566	1	2950.2	81.25	R.DQMQQQLSDYEQLLDVK.L
LMNB1_MOUSE	MK_SCX_16.8549.8549.2	2	2.769	0.257	1	437.5	41.17647	R.ELTGLKALYETELADARR.A
LMNB1_MOUSE	MK_SCX_34.4953.4953.3	3	4.095	0.41	1	1464.7	42.647057	R.LKNTSEQDQPMGGWEMIR.K
LMNB1_MOUSE	MK_SCX_52.4067.4067.3	3	3.742	0.304	1	998.4	46.153847	K.FKAEHDQLLLNYAK.K
LMNB2_MOUSE	MK_SCX_16.6789.6789.2	2	2.788	0.302	1	580.6	56.25	R.DAM*QQQLAEYQELLDIK.L
LMNB2_MOUSE	MK_SCX_16.7744.7744.2	2	3.647	0.5	1	1089.7	65.625	R.DAMQQQLAEYQELLDIK.L
LMNB2_MOUSE	MK_SCX_2201.2638.2638.2	2	3.264	0.415	1	698.6	87.5	K.VQAELEEAR.K
LMNB2_MOUSE	MK_SCX_25.4064.4064.2	2	3.334	0.528	1	844.9	72.72727	K.SQTNWGPGESFR.T
LMNB2_MOUSE	MK_SCX_53.4422.4422.3	3	3.235	0.286	1	1026.4	58.333332	R.VKDLESLFHR.S
LPP_MOUSE	MK_SCX_18.5821.5821.2	2	3.704	0.369	1	645.1	63.333332	K.MLYDMENPPADDYFGR.C
LPP_MOUSE	MK_SCX_20_1.4645.4645.2	2	2.458	0.16	1	343.9	54.166668	R.MVIPQQPPLTATK.K
LPP_MOUSE	MK_SCX_21.4432.4432.2	2	4.469	0.516	1	1648.5	78.57143	R.YYEPYYAAGPSYGGR.S
LPP_MOUSE	MK_SCX_29.4343.4343.3	3	5.157	0.435	1	1030.5	41.25	R.METTHSFGNPSISVSTQQPPK.K
LPP_MOUSE	MK_SCX_30.3730.3730.3	3	4.412	0.588	1	893.4	43.18182	K.SAQPSPHYM*AGPSSGQIYGPGPR.G
LPP_MOUSE	MK_SCX_30.3943.3943.3	3	4.942	0.475	1	1445.2	43.18182	K.SAQPSPHYMAGPSSGQIYGPGPR.G
LPP_MOUSE	MK_SCX_31.4379.4379.3	3	3.893	0.353	1	687.9	34.72222	R.CSVCKEPIMPAPGQEETVR.I
LPP_MOUSE	MK_SCX_32.3653.3653.3	3	4.734	0.504	1	932.4	47.058823	R.SEGDTAYGGQQVQPNTWKR.E
LPP_MOUSE	MK_SCX_32.4260.4260.2	2	3.542	0.349	1	1360.4	69.44444	R.CSVCKEPIMPAPGQEETVR.I
LPP_MOUSE	MK_SCX_42.3883.3883.3	3	6.631	0.609	1	2616.5	45.238094	R.METTHSFGNPSISVSTQQPPKK.Y
LPP_MOUSE	MK_SCX_44.6991.6991.3	3	3.721	0.596	1	582.4	30.555555	K.GGYPGPMGPPSIPPSFRPEDELEHLTKK.M
LRC47_MOUSE	MK_SCX_18.6698.6698.2	2	3.461	0.377	1	695.6	55.88235	R.NALGPGLSPELGPLPALR.V
LRC59_MOUSE	MK_SCX_20_1.7098.7098.2	2	3.273	0.426	1	647.6	81.818184	R.LVTLPVSFAQLK.N
LRC59_MOUSE	MK_SCX_21.9250.9250.3	3	5.926	0.598	1	1246.3	35	K.LDGNELDLSLSDLNEVPVKELAALPK.A
LRC59_MOUSE	MK_SCX_28.7221.7221.3	3	4.234	0.47	1	822	46.42857	K.WLDLKDNPLDPVLAK.V
LRC59_MOUSE	MK_SCX_28.7261.7261.2	2	4.594	0.368	1	1417.5	82.14286	K.WLDLKDNPLDPVLAK.V
LRC59_MOUSE	MK_SCX_34.5845.5845.2	2	4.615	0.594	1	1679.3	83.33333	R.LVNLQHLDLLNNR.L
LRC59_MOUSE	MK_SCX_40.4087.4087.2	2	3.751	0.244	1	1281.5	77.27273	K.NKLQQLPADFGR.L
LRC8D_MOUSE	MK_SCX_33.3293.3293.3	3	4.1	0.436	1	640.5	38.157894	K.HVSTSSDEGSPSASTPMINK.T
LRP16_MOUSE	MK_SCX_27.5836.5836.3	3	5.323	0.492	1	2132.6	43.18182	K.YVIHTVGPIAVGQPTASQAAELR.S
LRP16_MOUSE	MK_SCX_27.5879.5879.2	2	5.916	0.59	1	1686.8	63.636364	K.YVIHTVGPIAVGQPTASQAAELR.S
LRRF2_MOUSE	MK_SCX_19.4652.4652.2	2	3.208	0.492	1	670.8	69.230774	K.AEQDIATLEQSISR.L
LRRF2_MOUSE	MK_SCX_21.4816.4816.2	2	2.391	0.26	1	697.8	66.66667	R.FSAEDEALSNIAR.E
LRRF2_MOUSE	MK_SCX_44.4196.4196.3	3	5.071	0.462	1	1782.4	42.647057	R.TAQDKIEEMEMTNSHLAK.R
LRRK2_MOUSE	MK_SCX_34.5820.5820.2	2	2.184	0.203	1	307.8	42.307693	R.M*LVM*ELASKGSLDR.L
LRRK2_MOUSE	MK_SCX_48.4785.4785.3	3	3.296	0.284	1	356.1	26.136362	K.QSKQSNFLLVGTADGNLMIFEDK.A
LRSM1_MOUSE	MK_SCX_18.4458.4458.2	2	2.427	0.152	1	321	37.5	K.RKSLDTETLQEMVSEQR.W
LRSM1_MOUSE	MK_SCX_39.3678.3678.2	2	2.161	0.124	1	525	81.25	R.AQDLLAVPR.V
LSM4_MOUSE	MK_SCX_21.5819.5819.2	2	3.399	0.518	1	964.9	94.44444	R.IPDEIIDMVR.E
LSM4_MOUSE	MK_SCX_33.7940.7940.2	2	2.433	0.352	1	380	54.166668	K.YLRIPDEIIDMVR.E
LSM8_MOUSE	MK_SCX_29.7585.7585.3	3	4.022	0.426	1	903.2	48.333332	K.GFDQTINLILDESHER.V
LSP1_MOUSE	MK_SCX_27.4101.4101.3	3	3.865	0.426	1	782	32.608696	R.QQFWGNEGTAEGTEPSQSERPEEK.Q
LTB4D_MOUSE	MK_SCX_24.5590.5590.2	2	2.27	0.449	1	511	66.66667	K.MPAAFMGMLK.G
LTB4D_MOUSE	MK_SCX_36.5659.5659.2	2	3.22	0.367	1	906.9	69.230774	K.HFEGFPTDGNFELK.T
LUM_MOUSE	MK_SCX_18.8706.8706.2	2	4.536	0.524	1	913.9	70.588234	K.LPAGLPTSLLTLYLDNNK.I
LUM_MOUSE	MK_SCX_2201.10495.10495.3	3	4.721	0.38	1	465.9	26.85185	K.LPAGLPTSLLTLYLDNNKISNIPDEYFK.R

LX12L_MOUSE	MK_SCX_15.7246.7246.2	2	5.644	0.538	1	2180.7	73.68421	K.DGTILNVAATSISDLPVDQR.F
LX12L_MOUSE	MK_SCX_50.5992.5992.3	3	4.01	0.362	1	1258.5	48.214287	K.FREELAALDKEIEIR.N
LYAG_MOUSE	MK_SCX_17.5796.5796.2	2	4.734	0.573	1	491	58.823532	R.EGYIIPLQGPSLTTTESR.K
LYPL1_MOUSE	MK_SCX_50.5983.5983.3	3	6.502	0.647	1	1390.5	37.5	K.SLGVSTTFHSLPNLNHELNKTELEK.L
LYPL1_MOUSE	MK_SCX_53.4057.4057.3	3	3.157	0.343	1	813.7	45.833336	K.HVLNQDLTFQHIK.I
LYRIC_MOUSE	MK_SCX_16.6453.6453.2	2	4.828	0.697	1	663.8	46.153847	R.SIFSGIGSTAEPVSQSTTSDYQWDVSR.N
LYRIC_MOUSE	MK_SCX_17.4775.4775.2	2	3.489	0.569	1	929.1	78.125	R.EEAAPPTPAPDDLAQLK.N
LYRIC_MOUSE	MK_SCX_20_1.4772.4772.2	2	2.733	0.306	1	343.9	62.5	K.TMSTSDPAEVLIK.N
LYRIC_MOUSE	MK_SCX_24.3983.3983.3	3	4.979	0.432	1	1091.9	42.5	K.GDSDNSSSQVPPMLQDTDKPK.S
LYRIC_MOUSE	MK_SCX_32.3694.3694.2	2	2.869	0.294	1	567.6	65.38461	R.TVEVPEDEVVRNPR.S
LYRIC_MOUSE	MK_SCX_49.4383.4383.3	3	4.785	0.517	1	1409.2	43.055553	R.KREEAAPPTPAPDDLAQLK.N
LYSCM_MOUSE	MK_SCX_13.7945.7945.3	3	3.235	0.337	1	405.8	22.916668	R.AVNACGINCSALLQDDITAAIQCAK.R
LYSCM_MOUSE	MK_SCX_13.7951.7951.2	2	3.505	0.536	1	724.1	43.75	R.AVNACGINCSALLQDDITAAIQCAK.R
LYSCM_MOUSE	MK_SCX_20_1.5394.5394.2	2	4.064	0.396	1	2016.7	71.42857	R.GDQSTDYGIFQINSR.Y
LYSCM_MOUSE	MK_SCX_31.4901.4901.2	2	5.177	0.541	1	765.3	55	R.ATNYNRGDQSTDYGIFQINSR.Y
LYSCM_MOUSE	MK_SCX_31.4955.4955.3	3	6.37	0.589	1	1422.1	43.75	R.ATNYNRGDQSTDYGIFQINSR.Y
LYSCM_MOUSE	MK_SCX_58.4806.4806.2	2	2.625	0.322	1	333.4	72.22222	K.RVVRDPQGIR.A
LYST_MOUSE	MK_SCX_15.4575.4575.2	2	2.288	0.276	1	373.2	46.666668	R.LHNIQIANHICNLLQK.G
LYST_MOUSE	MK_SCX_30.4647.4647.2	2	2.587	0.131	1	388.5	50	K.EAQSILLEPSQLK.G
M2GD_MOUSE	MK_SCX_17.6395.6395.2	2	5.06	0.418	1	1738.3	76.47059	K.NYPATIIQEPLVLTEPAR.A
M2GD_MOUSE	MK_SCX_21.3917.3917.2	2	3.958	0.507	1	603.3	56.666668	K.VVGNTTSGSYSYSIQK.S
M2GD_MOUSE	MK_SCX_2201.8535.8535.3	3	3.299	0.215	1	360.6	23.076923	K.ILAGLYNPGDGHIDPYSLTM*ALAAGAR.K
M2GD_MOUSE	MK_SCX_2201.9041.9041.3	3	5.484	0.591	1	1274.5	34.615387	K.ILAGLYNPGDGHIDPYSLTMALAAGAR.K
M2GD_MOUSE	MK_SCX_29.4388.4388.3	3	3.681	0.385	1	585.6	33.82353	R.LEEETGQVVGFHQPGSIR.L
M2OM_MOUSE	MK_SCX_16.12436.12436.2	2	5.561	0.513	1	1165.6	59.523808	K.FLFGGLAGMGATVFVQPLDLVK.N
M2OM_MOUSE	MK_SCX_16.9431.9431.2	2	4.134	0.547	1	715.3	45.238094	K.FLFGGLAGM*GATVFVQPLDLVK.N
M2OM_MOUSE	MK_SCX_20_1.5282.5282.2	2	4.225	0.321	1	661.3	69.230774	R.LTGADGTPPGFLLK.A
M2OM_MOUSE	MK_SCX_32.6917.6917.3	3	3.885	0.35	1	1233.3	42.1875	K.TEGLKGIYTGLSAGLLR.Q
M3K4_MOUSE	MK_SCX_24.3448.3448.2	2	2.006	0.172	1	322.6	62.5	K.NDALELCNR.I
M3K4_MOUSE	MK_SCX_31.3836.3836.3	3	3.144	0.19	1	345.5	27.941175	R.LSPEGKAFLSHCLESDPK.I
M3K5_MOUSE	MK_SCX_33.7048.7048.2	2	2.256	0.138	1	320.4	54.166668	K.LSSLLGKKGNLEK.L
M3K5_MOUSE	MK_SCX_35.9266.9266.3	3	3.034	0.154	1	596.1	41.666664	K.ARNLYTGKELAAELAR.I
M6PBP_MOUSE	MK_SCX_14.9708.9708.3	3	5.479	0.558	1	1816	34.482758	R.EALDNTVEYVAQNTPAMWLVGPFAPGITEK.T
M6PBP_MOUSE	MK_SCX_18.9120.9120.2	2	6.083	0.578	1	2839.3	75	K.AQETLQQLTSVLGLMESVK.Q
M6PBP_MOUSE	MK_SCX_19.4541.4541.2	2	2.693	0.135	1	610.3	53.333336	K.TLTTAAVSTAQPILSK.L
M6PBP_MOUSE	MK_SCX_23.8462.8462.3	3	5.89	0.546	1	1737.5	34.25926	R.SQVNDLQATFSGIHSFQDLSAGVLAQTR.E
M6PBP_MOUSE	MK_SCX_23.9836.9836.3	3	4.911	0.608	1	1176.6	29.032257	R.AREALDNTVEYVAQNTPAMWLVGPFAPGITEK.T
M6PBP_MOUSE	MK_SCX_26.5155.5155.3	3	3.562	0.335	1	329.7	34.210526	R.VTGAVDVTLGAVQNSVDKTK.S
M6PBP_MOUSE	MK_SCX_31.3972.3972.2	2	4.572	0.626	1	1047.7	88.46153	K.LEPQIATASEYAHR.G
M6PBP_MOUSE	MK_SCX_41.2731.2731.3	3	4.88	0.453	1	1536.2	48.52941	K.TPQDAEKDPAKPEQVEAR.A
MA2B1_MOUSE	MK_SCX_21.5328.5328.2	2	3.983	0.483	1	2387.4	91.66667	R.FLQDTFGSDGLPR.V
MA2B1_MOUSE	MK_SCX_2201.6606.6606.2	2	3.806	0.46	1	1490.8	90	R.FQVTLYNPLGR.K
MA2B2_MOUSE	MK_SCX_32.4553.4553.2	2	3.733	0.403	1	1511.1	76.92308	R.SGVALQHGPVVLLK.E
MAAI_MOUSE	MK_SCX_17.6360.6360.2	2	4.922	0.439	1	1500.7	65.625	K.DGGQQFTEEFQTLNPMK.Q
MAAI_MOUSE	MK_SCX_17.8272.8272.2	2	5.259	0.663	1	1444.3	68.42105	R.M*ISDLIASGIQPLQNLSVLK.Q
MAAI_MOUSE	MK_SCX_17.8593.8593.2	2	5.737	0.498	1	2425.3	73.68421	R.MISDLIASGIQPLQNLSVLK.Q
MAAI_MOUSE	MK_SCX_23.6478.6478.3	3	4.015	0.426	1	460	25	K.ILQSTAGKYCVGDEVSM*ADVCLVPQVANAER.F
MAAI_MOUSE	MK_SCX_23.6834.6834.3	3	4.359	0.467	1	318.5	23.333334	K.ILQSTAGKYCVGDEVSMADVCLVPQVANAER.F
MAAI_MOUSE	MK_SCX_48.5515.5515.3	3	4.071	0.364	1	1086.1	46.666668	R.FKVDLSPYPTISHINK.E
MAAI_MOUSE	MK_SCX_48.5566.5566.2	2	4.85	0.47	1	1524.8	73.333336	R.FKVDLSPYPTISHINK.E
MANA_MOUSE	MK_SCX_14.8244.8244.2	2	3.654	0.497	1	333.2	34.615387	R.LFAPAQSQDDPYLSIYDPPVPDFTVMK.M

MAOX_MOUSE	MK_SCX_13.7595.7595.2	2	2.68	0.309	1	333	47.058823	R.ILGLGDLGCNGMGIPVGK.L
MAOX_MOUSE	MK_SCX_18.6545.6545.2	2	4.902	0.639	1	1446.7	67.64706	R.AIFASGSPFDPVTLPDGR.T
MAOX_MOUSE	MK_SCX_42.9171.9171.3	3	3.106	0.292	1	658.6	34.523808	K.IKPTALIGVAAIGGAFTEQILK.D
MAOX_MOUSE	MK_SCX_43.5108.5108.3	3	3.369	0.411	1	375.6	39.285713	R.DPHLNKDLAFTLEER.Q
MAOX_MOUSE	MK_SCX_52.6060.6060.3	3	3.62	0.544	1	516	36.25	R.GYLLTRDPHLNKDLAFTLEER.Q
MAP4_MOUSE	MK_SCX_14.2964.2964.2	2	2.214	0.316	1	433.3	56.25	R.NTTPTGAAPPAGM*TSTR.V
MAP4_MOUSE	MK_SCX_14.7911.7911.2	2	5.346	0.686	1	1503.4	70	R.DFMAALEAEPYDDIVGETVEK.T
MAP4_MOUSE	MK_SCX_15.4513.4513.2	2	2.361	0.187	1	392.6	47.368423	K.DVAPPM*EEEIVPGNDTTSPK.E
MAP4_MOUSE	MK_SCX_16.5163.5163.2	2	3.855	0.462	1	799.7	65.38461	K.DMSPLPESEVTLGK.D
MAP4_MOUSE	MK_SCX_18.5243.5243.2	2	5.358	0.431	1	605	52.77778	K.VAEFNNVTPLSEEEVTSVK.D
MAP4_MOUSE	MK_SCX_18.5779.5779.2	2	2.723	0.402	1	511.6	70.83333	K.MDLAPPEDVLLTK.E
MAP4_MOUSE	MK_SCX_20_1.3439.3439.2	2	3.92	0.612	1	456.3	59.375	R.NTTPTGAAPPAGMTSTR.V
MAP4_MOUSE	MK_SCX_31.3444.3444.2	2	4.588	0.504	1	1859.7	73.07692	K.AAEVESVKEQLPAK.A
MAP4_MOUSE	MK_SCX_35.3760.3760.3	3	4.511	0.546	1	534.9	30.208334	K.AAVGVTGNDITTPPNKEPPPSPEKK.A
MAP4_MOUSE	MK_SCX_38.3221.3221.3	3	3.494	0.318	1	497.2	38.235294	K.RNTTPTGAAPPAGMTSTR.V
MAP4_MOUSE	MK_SCX_53.2928.2928.3	3	3.071	0.388	1	363.6	32.8125	K.RPAAATATARPSTLPAR.D
MARCS_MOUSE	MK_SCX_14.3790.3790.3	3	5.145	0.614	1	2113.6	36.71875	K.DEAAAAAGGEGAAAPGEQAGGAGAEGAAGGEPR.E
MARCS_MOUSE	MK_SCX_15.3876.3876.2	2	4.733	0.656	1	897.2	51.785713	K.EAAEAEPAEPSSPAAEAEGASASSTSSPK.A
MARCS_MOUSE	MK_SCX_16.3967.3967.3	3	4.326	0.596	1	537.1	23.214285	K.EAAEAEPAEPSSPAAEAEGASASSTSSPK.A
MARCS_MOUSE	MK_SCX_43.2863.2863.3	3	4.4	0.49	1	588.1	35.227272	K.TAAKGEATAERPGEAAVASSPSK.A
MARE1_MOUSE	MK_SCX_24.12020.12020.3	3	4.584	0.448	1	690.8	30.000002	K.IEQLCSGAAYCQFMDMLFPGSIALKK.V
MARE1_MOUSE	MK_SCX_27.4756.4756.3	3	4.246	0.544	1	1194.6	39.473686	R.QGQETAVAPSLVAPALSKPK.K
MARE1_MOUSE	MK_SCX_27.4776.4776.2	2	4.403	0.617	1	725.2	57.894737	R.QGQETAVAPSLVAPALSKPK.K
MARE1_MOUSE	MK_SCX_27.5281.5281.2	2	3.387	0.476	1	914.1	60.526318	R.KNPGVGNGDDEAAELMQQVK.V
MARE1_MOUSE	MK_SCX_28.4954.4954.2	2	4.914	0.59	1	1061.1	68.75	K.FFDANYDGKEYDPVAAR.Q
MARE1_MOUSE	MK_SCX_33.4117.4117.2	2	2.748	0.259	1	720.5	83.33333	K.LTVEDLEKER.D
MARE1_MOUSE	MK_SCX_39.3555.3555.3	3	3.334	0.322	1	890.5	55.555557	K.LEHEYIQNFK.I
MARE1_MOUSE	MK_SCX_46.5548.5548.3	3	3.701	0.437	1	1043.1	50	R.MGVDKIIPVDKLVK.G
MARE2_MOUSE	MK_SCX_28.4368.4368.3	3	4.74	0.595	1	710.5	45.3125	K.FYDANYDGKEYDPVEAR.Q
MARE2_MOUSE	MK_SCX_33.8140.8140.3	3	3.667	0.37	1	626.5	52.083332	R.FQDNLDFIQWFKK.F
MARE2_MOUSE	MK_SCX_38.6734.6734.3	3	5.112	0.595	1	1506.5	44.736843	R.SDKDLETQVIQLNEQVHSLK.L
MARE2_MOUSE	MK_SCX_46.4373.4373.3	3	3.934	0.335	1	1263.6	47.058823	K.KFYDANYDGKEYDPVEAR.Q
MARE3_MOUSE	MK_SCX_28.5882.5882.3	3	4.24	0.521	1	693.9	43.75	K.FFDANYDGKDYNPLLAR.Q
MARE3_MOUSE	MK_SCX_47.5925.5925.3	3	3.715	0.481	1	597.7	38.235294	K.KFFDANYDGKDYNPLLAR.Q
MARK2_MOUSE	MK_SCX_30.3346.3346.3	3	4.766	0.484	1	2696.6	51.315792	R.VPVASPSAHNISSSSGAPDR.T
MARK3_MOUSE	MK_SCX_49.3346.3346.3	3	3.838	0.354	1	1483.5	43.055553	K.RSQTSTADSDLKEDGIPSR.K
MATR3_MOUSE	MK_SCX_14.9152.9152.2	2	4.866	0.613	1	1062	52.083332	R.DLSAAGIGLLAAATQSLSM*PASLGR.M
MATR3_MOUSE	MK_SCX_14.9222.9222.3	3	6.255	0.544	1	1841.1	42.708336	R.DLSAAGIGLLAAATQSLSM*PASLGR.M
MATR3_MOUSE	MK_SCX_14.9639.9639.2	2	4.915	0.621	1	974.9	47.916664	R.DLSAAGIGLLAAATQSLSMPASLGR.M
MATR3_MOUSE	MK_SCX_14.9647.9647.3	3	4.914	0.502	1	952.4	35.416664	R.DLSAAGIGLLAAATQSLSMPASLGR.M
MATR3_MOUSE	MK_SCX_17.6471.6471.2	2	4.626	0.574	1	774.5	64.70589	R.IGPYQPNVPVGIDYVIPK.T
MATR3_MOUSE	MK_SCX_18.7161.7161.2	2	3.067	0.455	1	424.9	47.058823	R.GDTDQASNILASFGLSAR.D
MATR3_MOUSE	MK_SCX_19.7907.7907.2	2	3.238	0.298	1	352.6	65.38461	K.ITPENLPQILLQLK.R
MATR3_MOUSE	MK_SCX_2201.3203.3203.2	2	2.35	0.158	1	1629.3	76.92308	R.GNLGAGNGNLQGPR.H
MATR3_MOUSE	MK_SCX_23.9157.9157.3	3	4.883	0.472	1	807.5	33.75	R.YQLLQLVEPFGVISNHLILNK.I
MATR3_MOUSE	MK_SCX_26.9042.9042.3	3	4.573	0.486	1	897.8	29	R.DLDELSRYPEDKITPENLPQILLQLK.R
MATR3_MOUSE	MK_SCX_27.4087.4087.2	2	3.264	0.297	1	1252.7	77.27273	R.DLDELSRYPEDK.I
MATR3_MOUSE	MK_SCX_33.5225.5225.3	3	3.603	0.396	1	1072.1	36.25	R.DSFDDRGPSLNPVLDYDHGSR.S
MATR3_MOUSE	MK_SCX_41.3871.3871.2	2	2.764	0.398	1	572.6	72.72727	R.MKSQAFIEMETR.E
MATR3_MOUSE	MK_SCX_41.3878.3878.3	3	3.492	0.388	1	1328.6	56.81818	R.MKSQAFIEMETR.E
MATR3_MOUSE	MK_SCX_41.4044.4044.2	2	2.39	0.347	1	563.9	75	R.VVHIMDFQR.G

MATR3_MOUSE	MK_SCX_46.4877.4877.2	2	5.042	0.546	1	979.1	63.88889	R.VIHLSNLPHSGYSDSAVLK.L
MATR3_MOUSE	MK_SCX_46.4911.4911.3	3	4.056	0.601	1	891.3	45.833336	R.VIHLSNLPHSGYSDSAVLK.L
MATR3_MOUSE	MK_SCX_50.5035.5035.3	3	5.241	0.51	1	1422	48.52941	K.RGAPPSSNIEDFHGLLPK.G
MATR3_MOUSE	MK_SCX_57.2834.2834.3	3	4.116	0.458	1	1045.4	47.916664	R.RRTEEGPTLSYGR.D
MAVS_MOUSE	MK_SCX_2201.4529.4529.2	2	3.171	0.307	1	537.6	66.66667	R.VYQSYLPPGTSLR.S
MAVS_MOUSE	MK_SCX_30.8027.8027.3	3	3.107	0.474	1	409.3	26.612906	R.SLEPLQLPDFPAAVSGPSAFAPGHNIPDHGLR.E
MAWB1_MOUSE	MK_SCX_21.4967.4967.2	2	3.22	0.224	1	971.9	68.181816	K.AAIGDTLVQDIR.Y
MAWB1_MOUSE	MK_SCX_27.7109.7109.2	2	3.958	0.47	1	631.2	47.058823	R.SFLESLKVNTEPLPAIEK.T
MAWB1_MOUSE	MK_SCX_32.16071.16071.3	3	4.365	0.553	1	1700.1	46.875	-.MKLPIFIADAFTATAFR.G
MAWB1_MOUSE	MK_SCX_32.16437.16437.3	3	3.834	0.492	1	1211.6	45.3125	-.M*KLPIFIADAFTATAFR.G
MAWB2_MOUSE	MK_SCX_2201.5208.5208.2	2	2.203	0.181	1	495.3	60.000004	R.EMNLSETAFIR.K
MAWB2_MOUSE	MK_SCX_23.9324.9324.3	3	4.781	0.586	1	977	35.344826	R.YFAPWVGIAEDPVTGSAHTVLSSYWSQQLR.K
MBB1A_MOUSE	MK_SCX_23.5884.5884.2	2	3.565	0.427	1	1688.7	88.88889	R.LYDLYWQAMR.M
MBB1A_MOUSE	MK_SCX_24.3833.3833.3	3	5.483	0.539	1	1812.1	37.037037	K.SEGTTPEKNAASQQDAVTEGAMPAATGK.D
MBB1A_MOUSE	MK_SCX_25.3882.3882.3	3	5.398	0.512	1	778.8	33.333336	K.NAASQQDAVTEGAMPAATGKDQPPSTGK.K
MBB1A_MOUSE	MK_SCX_30.3344.3344.3	3	3.928	0.516	1	759.1	37.5	K.SPAPSNPTLSPSTPAKTPK.L
MBD3_MOUSE	MK_SCX_16.6991.6991.2	2	2.194	0.209	1	358.7	36.842106	K.AFMVTDDDIRKQEELVQQVR.K
MBD3_MOUSE	MK_SCX_48.5546.5546.2	2	2.171	0.135	1	345.2	61.11111	K.MLM*NKM*NKSR.Q
MBP_MOUSE	MK_SCX_19.10667.10667.2	2	2.06	0.19	1	312.4	46.42857	R.ELSAEKASKDGEIHR.G
MBP_MOUSE	MK_SCX_23.7748.7748.2	2	2.009	0.122	1	370.7	35.294117	R.HRDTGILDSIGRFFSGDR.G
MCA1_MOUSE	MK_SCX_20_1.6221.6221.2	2	3.876	0.297	1	1364.9	77.27273	K.GAEADQIIEYLK.Q
MCA1_MOUSE	MK_SCX_27.5740.5740.2	2	3.993	0.477	1	567.3	59.375	R.ITFDAFPGEPDKELNPK.K
MCA1_MOUSE	MK_SCX_29.5747.5747.2	2	3.788	0.519	1	429.9	47.058823	R.TVVSGLVNHVPLEQMQNR.M
MCA1_MOUSE	MK_SCX_30.5427.5427.3	3	5.411	0.558	1	1344.1	51.47059	R.TVVSGLVNHVPLEQMQNR.M
MCCA_MOUSE	MK_SCX_17.4788.4788.2	2	6.244	0.607	1	1569.4	58.69565	K.YLSPVSAEGAQGGTIAPM*TGTIEK.V
MCCA_MOUSE	MK_SCX_17.5258.5258.2	2	6.003	0.529	1	1424.8	56.521736	K.YLSPVSAEGAQGGTIAPMTGTIEK.V
MCCA_MOUSE	MK_SCX_19.4233.4233.2	2	4.076	0.519	1	1236.1	78.57143	K.IIEEAPAPGINPEVR.R
MCCA_MOUSE	MK_SCX_21.7178.7178.3	3	3.714	0.494	1	708.7	27.586206	R.IYAEDPDNNFMPGAGPLVHLSTPSADMSTR.I
MCCA_MOUSE	MK_SCX_24.3684.3684.2	2	3.155	0.383	1	1456.2	85	K.VFFSEGAQANR.H
MCCA_MOUSE	MK_SCX_24.6209.6209.3	3	6.778	0.528	1	2628.1	40.74074	R.NSMHVDMADEAYSIGPAPSQQSYLAMEK.I
MCCA_MOUSE	MK_SCX_29.5616.5616.3	3	5.195	0.638	1	1430.3	48.52941	K.IPLSQEEIPLQGHAFEAR.I
MCCA_MOUSE	MK_SCX_35.8020.8020.3	3	3.123	0.378	1	389.1	38.333332	K.LHTQDQFSPFSFSSGR.R
MCCA_MOUSE	MK_SCX_37.6255.6255.3	3	4.757	0.419	1	1341.6	36.95652	R.IAAGEKIPLSQEEIPLQGHAFEAR.I
MCCA_MOUSE	MK_SCX_39.3742.3742.2	2	3.887	0.305	1	523.5	66.66667	R.SEREFQEQLESAR.R
MCCA_MOUSE	MK_SCX_39.3750.3750.3	3	3.165	0.264	1	384.4	39.583336	R.SEREFQEQLESAR.R
MCCA_MOUSE	MK_SCX_46.4952.4952.2	2	2.657	0.488	1	544.1	70	R.HNFYFMEMNTR.L
MCCA_MOUSE	MK_SCX_54.3393.3393.3	3	3.572	0.379	1	865.8	42.307693	R.SEREFQEQLESARR.E
MCE1_MOUSE	MK_SCX_31.7400.7400.3	3	3.931	0.13	1	954.3	38.46154	K.VKM*SLLVDLTNTSR.F
MCEE_MOUSE	MK_SCX_19.11653.11653.3	3	5.922	0.49	1	1161.8	34.82143	R.DVLGAQVSEVVPLPEHGVSVVFVNLGNTK.M
MCEE_MOUSE	MK_SCX_26.7351.7351.3	3	6.682	0.541	1	2684.9	52.77778	K.MELLHPLGSDSPITGFLQK.N
MCEE_MOUSE	MK_SCX_27.7255.7255.3	3	5.291	0.466	1	1544.4	45.833336	K.M*ELLHPLGSDSPITGFLQK.N
MCEE_MOUSE	MK_SCX_27.7987.7987.2	2	5.22	0.591	1	1489.7	80.55556	K.MELLHPLGSDSPITGFLQK.N
MCEE_MOUSE	MK_SCX_33.4457.4457.2	2	2.909	0.358	1	694.3	62.5	R.LNHVAVAVPDLEK.A
MCL1_MOUSE	MK_SCX_15.5069.5069.2	2	3.583	0.568	1	331.8	43.47826	K.SSGADGSLPSTPPPPEEEEDDLYR.Q
MCLN1_MOUSE	MK_SCX_15.5470.5470.2	2	5.201	0.471	1	883	42.592594	R.LLTPNPGYGTQVGTSPAPTTPTEEEDLR.R
MCM3A_MOUSE	MK_SCX_23.16503.16503.2	2	2.053	0.133	1	337.9	78.57143	R.DRIM*RQAR.V
MCM3A_MOUSE	MK_SCX_31.6495.6495.2	2	2.238	0.138	1	314.1	61.11111	R.LLDQRDRIM*R.Q
MCM6_MOUSE	MK_SCX_10.6805.6805.3	3	3.431	0.242	1	321.7	20.689655	R.ISGQVVRTHPVHPELVSGTFLCLDCQTVIK.D
MCM6_MOUSE	MK_SCX_21.7389.7389.2	2	2.789	0.152	1	456.3	62.5	R.FLLDTNKSR.F
MDHC_MOUSE	MK_SCX_13.4621.4621.2	2	5.207	0.588	1	1722.9	81.25	K.VIVVGNPANTNCLTASK.S
MDHC_MOUSE	MK_SCX_13.4662.4662.1	1	3.02	0.543	1	420.9	56.25	K.VIVVGNPANTNCLTASK.S

MDHC_MOUSE	MK_SCX_21.5906.5906.2	2	3.584	0.46	1	772.9	86.36364	K.FVEGLPINDFSR.E
MDHC_MOUSE	MK_SCX_2201.2997.2997.2	2	3.102	0.479	1	962.3	87.5	K.LGVTADDVK.N
MDHC_MOUSE	MK_SCX_23.3726.3726.2	2	3.906	0.452	1	1152.7	88.88889	K.GEFITTVQQR.G
MDHC_MOUSE	MK_SCX_29.6784.6784.2	2	5.132	0.455	1	1708.9	75	K.EVGVYEALKDDSWLK.G
MDHC_MOUSE	MK_SCX_43.6388.6388.2	2	5.273	0.587	1	2371.8	69.44444	K.LQGKEVGVYEALKDDSWLK.G
MDHC_MOUSE	MK_SCX_43.6395.6395.3	3	5.735	0.6	1	2115.4	51.38889	K.LQGKEVGVYEALKDDSWLK.G
MDHC_MOUSE	MK_SCX_45.4536.4536.2	2	5.901	0.578	1	1316.2	60.526318	K.NVIIWGNHSSTQYPDVNHAK.V
MDHC_MOUSE	MK_SCX_45.4547.4547.3	3	5.884	0.542	1	1580.8	47.368423	K.NVIIWGNHSSTQYPDVNHAK.V
MDHM_MOUSE	MK_SCX_15.8045.8045.3	3	4.681	0.572	1	1019.4	38	K.TIIPLISQCTPKVDFPQDQLATLTGR.I
MDHM_MOUSE	MK_SCX_18.8023.8023.2	2	7.081	0.551	1	2031.6	75	K.VAVLGASGGIGQPLSLLLK.N
MDHM_MOUSE	MK_SCX_19.5533.5533.2	2	4.932	0.522	1	1264.6	84.61539	K.VDFPQDQLATLTGR.I
MDHM_MOUSE	MK_SCX_21.4517.4517.2	2	3.335	0.418	1	1596.8	88.88889	K.M*IAEAIPELK.A
MDHM_MOUSE	MK_SCX_21.4794.4794.2	2	3.558	0.372	1	957.7	83.33333	K.MIAEAIPELK.A
MDHM_MOUSE	MK_SCX_21.6678.6678.1	1	2.408	0.302	1	339.6	70	K.IFGVTTLDIVR.A
MDHM_MOUSE	MK_SCX_21.6738.6738.2	2	3.489	0.372	1	1059.9	85	K.IFGVTTLDIVR.A
MDHM_MOUSE	MK_SCX_2201.2804.2804.2	2	2.901	0.249	1	590.3	77.77778	R.IQEAGTEVVK.A
MDHM_MOUSE	MK_SCX_2201.4070.4070.3	3	4.616	0.511	1	1874.1	51.666664	K.AGAGSATLSMAYAGAR.F
MDHM_MOUSE	MK_SCX_2201.4547.4547.2	2	5.231	0.523	1	845	66.66667	K.AGAGSATLSMAYAGAR.F
MDHM_MOUSE	MK_SCX_23.7372.7372.2	2	4.569	0.428	1	529.2	38	K.GYLGPEQLPDCLKGCDVVVIPAGVPR.K
MDHM_MOUSE	MK_SCX_23.7682.7682.3	3	4.657	0.223	1	476.5	25	K.GYLGPEQLPDCLKGCDVVVIPAGVPR.K
MDHM_MOUSE	MK_SCX_32.5698.5698.3	3	3.324	0.342	1	696.1	44.642857	R.ANTFVAELKGLDPAR.V
MDHM_MOUSE	MK_SCX_32.5836.5836.2	2	3.598	0.402	1	759.1	60.714287	R.ANTFVAELKGLDPAR.V
MDHM_MOUSE	MK_SCX_33.7468.7468.3	3	5.813	0.597	1	1103.9	27.586206	R.ANVKGYLGPEQLPDCLKGCDVVVIPAGVPR.K
MDHM_MOUSE	MK_SCX_34.7583.7583.3	3	7.643	0.532	1	2807	51.19048	R.LTLYDIAHTPGVAADLSHIETR.A
MDHM_MOUSE	MK_SCX_34.7704.7704.2	2	5.722	0.629	1	1284.9	61.904762	R.LTLYDIAHTPGVAADLSHIETR.A
MDHM_MOUSE	MK_SCX_37.3352.3352.3	3	4.365	0.44	1	1711.5	50	K.AKAGAGSATLSM*AYAGAR.F
MDHM_MOUSE	MK_SCX_37.3371.3371.2	2	5.685	0.561	1	1284	79.411766	K.AKAGAGSATLSM*AYAGAR.F
MDHM_MOUSE	MK_SCX_37.3570.3570.2	2	3.508	0.599	1	941	90.909096	R.VNVPVIGGHAGK.T
MDHM_MOUSE	MK_SCX_46.4684.4684.3	3	3.811	0.462	1	1619.6	45.588234	K.GLDPARVNVPVIGGHAGK.T
MDHM_MOUSE	MK_SCX_50.6397.6397.2	2	6.074	0.621	1	2690.3	83.33333	K.HGVYNPNKIFGVTTLDIVR.A
MDHM_MOUSE	MK_SCX_50.6559.6559.3	3	5.433	0.574	1	1946	47.22222	K.HGVYNPNKIFGVTTLDIVR.A
MDHM_MOUSE	MK_SCX_50.873.873.2	2	3.205	0.398	1	1031.7	92.85714	K.HGVYNPNK.I
MECP2_MOUSE	MK_SCX_16.6140.6140.2	2	5.463	0.64	1	875.1	64.70589	K.VGDTSLDPNDFDFTVTGR.G
MECP2_MOUSE	MK_SCX_17.3003.3003.2	2	2.153	0.253	1	350.4	46.666668	R.KPGSVVAAAAAEAKKK.A
MECP2_MOUSE	MK_SCX_18.3360.3360.2	2	4.853	0.604	1	1133.8	62.5	K.AETSESSGSAPAVPEASASPK.Q
MECP2_MOUSE	MK_SCX_2201.1675.1675.2	2	3.478	0.411	1	1259.7	88.88889	K.AAASEGVQVK.R
MECP2_MOUSE	MK_SCX_27.5073.5073.3	3	6.191	0.613	1	2131.3	45	K.MPFQASPGGKGEGGGATTSAQVMVIK.R
MECP2_MOUSE	MK_SCX_28.5454.5454.2	2	3.694	0.499	1	577.8	62.5	R.DRGPMYDDPTLPEGWTR.K
MECP2_MOUSE	MK_SCX_30.4683.4683.2	2	4.042	0.526	1	607.6	73.07692	K.EVVKPLLVSTLGEK.S
MECP2_MOUSE	MK_SCX_34.4273.4273.2	2	4.747	0.598	1	1886.3	75	R.SAGKYDVYLINPQGK.A
MECP2_MOUSE	MK_SCX_39.3343.3343.2	2	4.159	0.49	1	873.5	76.92308	R.KPGSVVAAAAAEAK.K
MECP2_MOUSE	MK_SCX_39.3347.3347.3	3	4.141	0.4	1	1823	55.76923	R.KPGSVVAAAAAEAK.K
MECP2_MOUSE	MK_SCX_54.3130.3130.3	3	3.302	0.531	1	1118.8	43.333332	R.GRKPGSVVAAAAAEAK.K
MECR_MOUSE	MK_SCX_14.10088.10088.3	3	5.778	0.642	1	1722	33.088234	R.M*LVDFEQLQPGDSVIQNASNSGVGQAVIQIASALR.L
MECR_MOUSE	MK_SCX_14.10169.10169.3	3	5.34	0.585	1	1503.5	30.147058	R.MLVDFEQLQPGDSVIQNASNSGVGQAVIQIASALR.L
MECR_MOUSE	MK_SCX_16.7008.7008.2	2	3.507	0.568	1	458.9	45.454548	R.M*LAAPINPSDINM*IQGNYGLLPK.L
MECR_MOUSE	MK_SCX_16.7262.7262.2	2	3.721	0.463	1	633.2	45.454548	R.MLAAPINPSDINM*IQGNYGLLPK.L
MECR_MOUSE	MK_SCX_16.7462.7462.2	2	4.981	0.446	1	1068.5	56.81818	R.M*LAAPINPSDINMIQGNYGLLPK.L
MECR_MOUSE	MK_SCX_16.7693.7693.2	2	3.4	0.506	1	585.4	43.18182	R.MLAAPINPSDINMIQGNYGLLPK.L
MECR_MOUSE	MK_SCX_31.5760.5760.3	3	3.897	0.371	1	1431.4	45	R.LKDLGADYVLTEEELR.M
MECR_MOUSE	MK_SCX_37.3531.3531.3	3	3.203	0.263	1	1224.4	48.333332	R.HLAPGGTMVTYGGM*AK.Q

MECR_MOUSE	MK_SCX_38.5738.5738.3	3	3.037	0.403	1	363.4	47.22222	K.TIFKDLPLPR.L
MECR_MOUSE	MK_SCX_38.5836.5836.2	2	2.214	0.461	1	621.5	66.66667	K.TIFKDLPLPR.L
MED4_MOUSE	MK_SCX_19.6574.6574.2	2	3.087	0.177	1	418.9	54.166668	R.ELIEMLAISRNQK.L
MED4_MOUSE	MK_SCX_25.8330.8330.2	2	2.16	0.128	1	328.6	38.235294	K.ERMGGVSGM*AGLGSTRER.L
MED8_MOUSE	MK_SCX_18.6199.6199.2	2	3.335	0.203	1	326.5	43.333332	K.QLTTDAARIGADAAQK.Q
MEP1A_MOUSE	MK_SCX_16.9795.9795.3	3	3.858	0.288	1	823.5	35	K.IPEFNTIIGQLPDFSAIDLIR.L
MEP1A_MOUSE	MK_SCX_16.9923.9923.2	2	6.752	0.543	1	2020.6	72.5	K.IPEFNTIIGQLPDFSAIDLIR.L
MEP1A_MOUSE	MK_SCX_18.5696.5696.2	2	3.276	0.35	1	671.2	52.941177	R.FYNSEGYGVGVTLYPNGR.I
MEP1A_MOUSE	MK_SCX_19.4387.4387.2	2	4.855	0.67	1	1281.6	78.125	R.GLLLQGQESPALGESSR.K
MEP1A_MOUSE	MK_SCX_19.5061.5061.2	2	5.038	0.516	1	1933.7	84.61539	R.QAIMTILDQEADTR.N
MEP1A_MOUSE	MK_SCX_31.11957.11957.3	3	3.962	0.343	1	1197.6	41.17647	R.WKLPIPYILADNLELNAK.G
MEP1A_MOUSE	MK_SCX_33.7995.7995.2	2	4.629	0.398	1	1361.4	70	R.SLDWGWGQAISHQLLK.R
MEP1A_MOUSE	MK_SCX_36.5521.5521.2	2	3.227	0.332	1	790.3	65.38461	K.MTGSPADRFEVWVR.R
MEP1B_MOUSE	MK_SCX_13.10361.10361.2	2	4.304	0.505	1	1359.6	46.153847	K.DIDGGIDQDIFDINQGLGLDLFEGDIK.L
MEP1B_MOUSE	MK_SCX_13.10761.10761.3	3	5.038	0.524	1	485.6	29.807693	K.DIDGGIDQDIFDINQGLGLDLFEGDIK.L
MEP1B_MOUSE	MK_SCX_20_1.4171.4171.3	3	4.039	0.362	1	815	46.42857	R.EYTTGQQGGVLTLQR.Q
MEP1B_MOUSE	MK_SCX_20_1.4183.4183.2	2	5.582	0.5	1	1790.3	75	R.EYTTGQQGGVLTLQR.Q
MEP1B_MOUSE	MK_SCX_21.4643.4643.2	2	4.766	0.53	1	1863.7	90	R.ISEFEDVIGQR.M
MEP1B_MOUSE	MK_SCX_2201.5225.5225.2	2	3.507	0.364	1	1184.7	77.27273	R.GIGYGTTVFITR.E
MESD2_MOUSE	MK_SCX_26.7220.7220.3	3	3.142	0.3	1	775.4	35.9375	R.DGSYAWEIKDFLVSQDR.C
MESD2_MOUSE	MK_SCX_29.3413.3413.3	3	3.792	0.426	1	729.1	50	K.DIRDYNDADMAR.L
MET_MOUSE	MK_SCX_19.3762.3762.2	2	3.92	0.514	1	815.6	62.5	R.SVSPTTEM*VSNESVDYR.A
METK2_MOUSE	MK_SCX_21.4619.4619.2	2	3.951	0.454	1	1604.2	82.14286	R.FVIGGPQGDAGLTGR.K
METK2_MOUSE	MK_SCX_29.4918.4918.3	3	3.11	0.387	1	394.3	42.857143	K.YLDEDTIYHLQPSGR.F
METK2_MOUSE	MK_SCX_29.4936.4936.2	2	5.203	0.475	1	1478.6	75	K.YLDEDTIYHLQPSGR.F
METK2_MOUSE	MK_SCX_29.4936.4936.3	3	3.056	0.378	1	434.9	23.913044	K.VIKAVVPAKYLDEDTIYHLQPSGR.F
METL2_MOUSE	MK_SCX_23.5765.5765.2	2	2.879	0.22	1	592.8	70	R.LLKPGGVMLLR.D
METL2_MOUSE	MK_SCX_25.7636.7636.3	3	3.052	0.213	1	368.7	26.086956	R.HWLFTEFPELAPSHSHLTGVPLEK.Q
MGDP1_MOUSE	MK_SCX_16.8144.8144.2	2	5	0.602	1	2204.5	75	R.DGMSLQTLTQGLETFAK.A
MGDP1_MOUSE	MK_SCX_18.8903.8903.2	2	5.815	0.563	1	2951.2	82.35294	R.TSEIQGANQLLELFDLGK.Y
MGDP1_MOUSE	MK_SCX_19.6798.6798.2	2	3.864	0.515	1	512.7	56.666668	K.TGVPFSQMVFFDDENR.N
MGDP1_MOUSE	MK_SCX_35.9268.9268.3	3	4.202	0.473	1	1109	45.3125	R.RGQNIQLYPEVPEVLGR.L
MIA2_MOUSE	MK_SCX_15.6054.6054.2	2	4.566	0.442	1	711.9	50	K.VFESDTEPTQELALEEESDLEK.L
MIF_MOUSE	MK_SCX_2201.4310.4310.2	2	4.149	0.479	1	1675.3	90	-.PM*FIVNTNVPR.A
MIF_MOUSE	MK_SCX_2201.4830.4830.2	2	4.639	0.502	1	2438.9	90	-.PMFIVNTNVPR.A
MIMIT_MOUSE	MK_SCX_17.7882.7882.2	2	3.12	0.372	1	425	50	K.EVDYEAGDIPTEWEAWIR.R
MIMIT_MOUSE	MK_SCX_18.4460.4460.2	2	4.094	0.335	1	532.4	63.333332	K.ETSEELLPSPTATQVK.G
MIMIT_MOUSE	MK_SCX_47.3980.3980.3	3	3.253	0.373	1	374.6	30.952381	K.GHASAPYFGREEPSVAPTSTGK.T
MIOX_MOUSE	MK_SCX_21.5741.5741.2	2	3.484	0.515	1	442.5	47.5	K.VDVGPDPSLVYRPDVDPEMAK.S
MIOX_MOUSE	MK_SCX_33.5971.5971.3	3	4.018	0.45	1	596.2	34.090908	-.MKVDVGPDPSLVYRPDVDPEMAK.S
MLRN_MOUSE	MK_SCX_18.4049.4049.2	2	3.021	0.223	1	1318.5	90	K.LNGTDPEDVIR.N
MLRN_MOUSE	MK_SCX_18.6300.6300.2	2	4.06	0.559	1	765.8	58.823532	R.ATSNVFAM*FDQSQIQEFK.E
MLRN_MOUSE	MK_SCX_18.7192.7192.2	2	5.089	0.553	1	1747.8	67.64706	R.ATSNVFAMFDQSQIQEFK.E
MLRN_MOUSE	MK_SCX_20_1.3671.3671.2	2	3.942	0.483	1	1329.6	85	R.FTDEEVDEM*YR.E
MLRN_MOUSE	MK_SCX_23.10136.10136.3	3	5.374	0.503	1	1042.4	33.333336	R.ATSNVFAMFDQSQIQEFKEAFNMIDQNR.D
MLRN_MOUSE	MK_SCX_23.8852.8852.3	3	4.417	0.402	1	734.5	29.62963	R.ATSNVFAM*FDQSQIQEFKEAFNM*IDQNR.D
MLRN_MOUSE	MK_SCX_23.9486.9486.3	3	4.626	0.173	1	852.1	28.703705	R.ATSNVFAMFDQSQIQEFKEAFNM*IDQNR.D
MLRN_MOUSE	MK_SCX_23.9644.9644.3	3	3.565	0.171	1	345.7	22.222223	R.ATSNVFAM*FDQSQIQEFKEAFNMIDQNR.D
MLRN_MOUSE	MK_SCX_25.4760.4760.2	2	3.504	0.577	1	1110.8	77.77778	K.GNFNYVEFTR.I
MMAB_MOUSE	MK_SCX_13.6817.6817.2	2	3.856	0.298	1	967.9	70	K.IQCMLQDVGSALATPR.S
MMAB_MOUSE	MK_SCX_36.4472.4472.3	3	3.055	0.358	1	375	40.384613	K.HTAFQEGPVLELER.W

MO4L1_MOUSE	MK_SCX_13.14558.14558.2	2	2.391	0.251	1	317.5	50	R.VLCFHGPLLYEAK.C
MO4L1_MOUSE	MK_SCX_41.8799.8799.3	3	3.5	0.245	1	494.4	36.842106	K.VKIPEELKPWLVDDWDLITR.Q
MOES_MOUSE	MK_SCX_17.8285.8285.2	2	2.988	0.365	1	844.9	68.75	K.FYPEDVSEELIQDITQR.L
MOES_MOUSE	MK_SCX_19.12179.12179.2	2	4.486	0.607	1	1509.5	76.666664	K.IAQDLEMYGVNYFSIK.N
MOES_MOUSE	MK_SCX_20_1.3603.3603.2	2	2.986	0.146	1	580.9	72.72727	K.KKESEAVEWQQK.A
MOES_MOUSE	MK_SCX_20_1.4085.4085.2	2	4.636	0.511	1	1465.6	75	K.TQEQLASEM*AELTAR.I
MOES_MOUSE	MK_SCX_20_1.6053.6053.2	2	2.568	0.274	1	729.5	64.28571	K.TQEQLASEMAELTAR.I
MOES_MOUSE	MK_SCX_2201.2019.2019.2	2	3.269	0.342	1	1040.9	88.88889	K.AQKELEEQTR.K
MOES_MOUSE	MK_SCX_2201.3021.3021.2	2	3.189	0.21	1	753.1	77.77778	K.ESEAVEWQQK.A
MOES_MOUSE	MK_SCX_2201.3022.3022.2	2	2.201	0.167	1	521.6	71.42857	R.ALELEQER.K
MOES_MOUSE	MK_SCX_28.10174.10174.3	3	3.295	0.176	1	544.5	34.72222	R.AKFYPEDVSEELIQDITQR.L
MOES_MOUSE	MK_SCX_29.10620.10620.2	2	4.53	0.585	1	1475.1	66.66667	R.AKFYPEDVSEELIQDITQR.L
MOES_MOUSE	MK_SCX_30.4167.4167.3	3	3.866	0.451	1	1509.4	30.000002	K.TAMSTPHVAEPAENEHDEQDENGAEASAELR.A
MOES_MOUSE	MK_SCX_32.3810.3810.3	3	3.444	0.36	1	1181.7	44.642857	R.QEAEEAKEALLQASR.D
MOES_MOUSE	MK_SCX_32.3829.3829.2	2	4.744	0.435	1	1021.1	78.57143	R.QEAEEAKEALLQASR.D
MOES_MOUSE	MK_SCX_34.5674.5674.2	2	2.903	0.318	1	369.2	62.5	R.GM*LREDAVLEYLK.I
MOES_MOUSE	MK_SCX_34.6245.6245.2	2	3.4	0.354	1	808.2	62.5	R.GMLREDAVLEYLK.I
MOES_MOUSE	MK_SCX_34.6567.6567.3	3	4.014	0.442	1	1029.9	47.916664	R.GMLREDAVLEYLK.I
MOES_MOUSE	MK_SCX_35.4285.4285.2	2	3.396	0.471	1	1727.8	79.16667	K.SGYLAGDKLLPQR.V
MOES_MOUSE	MK_SCX_35.5842.5842.2	2	5.616	0.496	1	2186.2	80	K.KTQEQLASEMAELTAR.I
MOES_MOUSE	MK_SCX_35.5928.5928.3	3	3.593	0.51	1	1223.8	48.333332	K.KTQEQLASEMAELTAR.I
MOES_MOUSE	MK_SCX_41.5315.5315.2	2	6.085	0.545	1	2611.5	77.77778	R.DQKKTQEQLASEMAELTAR.I
MOES_MOUSE	MK_SCX_41.5338.5338.3	3	4.787	0.566	1	1340.4	38.88889	R.DQKKTQEQLASEMAELTAR.I
MOES_MOUSE	MK_SCX_51.4031.4031.3	3	4.873	0.473	1	1743.4	46.875	K.ERQEAEEAKEALLQASR.D
MOES_MOUSE	MK_SCX_51.4061.4061.2	2	5.603	0.52	1	1211.8	78.125	K.ERQEAEEAKEALLQASR.D
MOES_MOUSE	MK_SCX_53.3200.3200.3	3	3.856	0.398	1	1218.4	45.3125	R.DESKKTANDMIHAENMR.L
MOES_MOUSE	MK_SCX_57.3597.3597.3	3	3.82	0.266	1	930.4	44.642857	R.VLEQHKLNKDQWEER.I
MOL2B_MOUSE	MK_SCX_23.3690.3690.2	2	4.053	0.526	1	2054.8	95.454544	R.AQASLNSGVDLR.A
MP2K4_MOUSE	MK_SCX_33.4540.4540.3	3	5.019	0.582	1	932.1	43.75	R.FTLNPNTTGVQNPHIER.L
MPCP_MOUSE	MK_SCX_23.3597.3597.2	2	3.614	0.444	1	773.4	81.818184	R.IQTQPGYANTLR.E
MPCP_MOUSE	MK_SCX_23.9219.9219.3	3	4.24	0.403	1	1089.4	31.730768	K.YYALCGFGGVLSCGLTHTAVVPLDLVK.C
MPCP_MOUSE	MK_SCX_23.9252.9252.2	2	3.021	0.364	1	384	30.769232	K.YYALCGFGGVLSCGLTHTAVVPLDLVK.C
MPCP_MOUSE	MK_SCX_38.4040.4040.3	3	3.855	0.326	1	1324	61.363636	K.M*YKEEGLNAFYK.G
MPCP_MOUSE	MK_SCX_39.4141.4141.2	2	3.664	0.461	1	1574.7	81.818184	K.M*YKEEGLNAFYK.G
MPCP_MOUSE	MK_SCX_39.4587.4587.3	3	4.227	0.399	1	764	54.545456	K.MYKEEGLNAFYK.G
MPCP_MOUSE	MK_SCX_39.4748.4748.2	2	3.887	0.43	1	1533.5	86.36364	K.MYKEEGLNAFYK.G
MPCP_MOUSE	MK_SCX_41.3718.3718.2	2	4.995	0.487	1	1549.1	80.769226	K.VRIQTQPGYANTLR.E
MPCP_MOUSE	MK_SCX_41.3728.3728.3	3	3.544	0.4	1	1177.8	53.846157	K.VRIQTQPGYANTLR.E
MPCP_MOUSE	MK_SCX_47.7482.7482.3	3	3.024	0.241	1	465.7	28.947369	K.MYKEEGLNAFYKGVAPLWMR.Q
MPP10_MOUSE	MK_SCX_19.8248.8248.3	3	4.273	0.53	1	505.7	24.193548	K.VVSNLPAITMEEVAPVSVSDAALLAPEEIKEK.N
MPP6_MOUSE	MK_SCX_16.9389.9389.2	2	4.138	0.63	1	1005.8	50	R.TSEFMPYVVFIAAPELETLR.A
MPP6_MOUSE	MK_SCX_19.5351.5351.2	2	3.523	0.425	1	629.4	63.333332	K.EGGSAGLIPSQFLEEK.R
MPRD_MOUSE	MK_SCX_19.5867.5867.2	2	5.722	0.549	1	1704.8	76.47059	K.SFESTVGQGSDTYSYIFR.V
MPRI_MOUSE	MK_SCX_32.3474.3474.3	3	4.769	0.422	1	1822.9	44.736843	R.HSESEQNWEAVDGSQAESEK.Y
MRCKB_MOUSE	MK_SCX_14.7379.7379.2	2	3.181	0.568	1	637.7	40.384613	R.NLEAPYIPDVSSPSDTSNFDVDDDMLR.N
MRCKB_MOUSE	MK_SCX_31.6138.6138.3	3	4.01	0.537	1	795.9	34.523808	R.SKPYVSWPSSGGSEPGVPVPLR.S
MRIP_MOUSE	MK_SCX_17.6363.6363.2	2	6.07	0.625	1	1824.7	73.52941	R.FGMLDTIDGPGMEDTALR.M
MRIP_MOUSE	MK_SCX_26.3920.3920.3	3	4.851	0.624	1	1112.7	33.653847	K.MAVTSSSGGSSGSSSSIPSAEKVPTTK.S
MRIP_MOUSE	MK_SCX_28.5321.5321.3	3	4.205	0.267	1	1197.3	46.666668	R.MDIDRSPGLLGTPDLK.T
MSAP_MOUSE	MK_SCX_14.4720.4720.2	2	3.01	0.229	1	1503.9	75	R.NGESSELDLQGIR.I
MSAP_MOUSE	MK_SCX_19.4288.4288.2	2	4.476	0.586	1	1099.3	76.666664	R.INPDGSQSVVEVPYAR.S

MSAP_MOUSE	MK_SCX_21.3754.3754.2	2	2.832	0.405	1	787.9	83.33333	R.IDSDISGTLK.F
MSAP_MOUSE	MK_SCX_57.2917.2917.3	3	3.323	0.352	1	719.1	39.285713	R.MKEYGEQIDPSTHRK.N
MSI2H_MOUSE	MK_SCX_44.7334.7334.3	3	5.186	0.624	1	668.8	29.807693	R.GFGFVTFADPASVDKVLGQPHHELDSK.T
MSRA_MOUSE	MK_SCX_21.4071.4071.2	2	2.749	0.305	1	910	85	K.VISAEEALPGR.T
MSRA_MOUSE	MK_SCX_27.5519.5519.2	2	4.917	0.641	1	1574.7	61.764706	R.EGQVFYYAEDYHQQYLSK.N
MSRA_MOUSE	MK_SCX_35.3791.3791.3	3	3.04	0.472	1	425.2	45.833336	K.VFWENHDPTQGM*R.Q
MSRA_MOUSE	MK_SCX_35.4671.4671.3	3	3.578	0.471	1	585.4	47.916664	K.VFWENHDPTQGMR.Q
MSRA_MOUSE	MK_SCX_36.3761.3761.2	2	3.421	0.557	1	472.2	62.5	K.VFWENHDPTQGM*R.Q
MSRA_MOUSE	MK_SCX_36.4263.4263.2	2	4.425	0.569	1	548.9	66.66667	K.VFWENHDPTQGMR.Q
MSRA_MOUSE	MK_SCX_38.3522.3522.3	3	3.145	0.438	1	315.7	39.285713	K.GVYSTQVGFAGGHTR.N
MSRA_MOUSE	MK_SCX_42.4161.4161.2	2	3.499	0.558	1	898.1	80	K.HNFGPITTDIR.E
MSRA_MOUSE	MK_SCX_43.4322.4322.3	3	3.237	0.195	1	438.1	52.499996	K.HNFGPITTDIR.E
MSRA_MOUSE	MK_SCX_48.6184.6184.3	3	4.634	0.391	1	1587.7	53.571426	R.VVYRPEHISFEELLK.V
MSRA_MOUSE	MK_SCX_49.6305.6305.2	2	4.349	0.294	1	1061.8	67.85714	R.VVYRPEHISFEELLK.V
MST4_MOUSE	MK_SCX_21.4094.4094.2	2	3.745	0.468	1	601.5	70.83333	K.AANVLLSEQGDVK.L
MST4_MOUSE	MK_SCX_21.7206.7206.2	2	2.248	0.221	1	697.1	77.77778	K.ELLKHKFIVK.N
MST4_MOUSE	MK_SCX_29.5208.5208.3	3	3.617	0.576	1	1463.9	50	K.LADFGVAGQLTDTQIKR.N
MST4_MOUSE	MK_SCX_40.5103.5103.3	3	3.464	0.353	1	1010	57.5	K.KTSYLTELIDR.F
MST4_MOUSE	MK_SCX_40.5275.5275.2	2	2.51	0.305	1	344.7	60.000004	K.KTSYLTELIDR.F
MTA2_MOUSE	MK_SCX_27.6489.6489.2	2	2.691	0.481	1	1177.4	70.83333	R.DITLFHAMDTLQR.N
MTA2_MOUSE	MK_SCX_29.3667.3667.2	2	2.173	0.431	1	321.3	60.000004	K.DLVAQAPLKPK.T
MTA2_MOUSE	MK_SCX_36.3971.3971.2	2	3.03	0.295	1	622.4	57.14286	K.YGGLKTPTQLEGAAR.G
MTA2_MOUSE	MK_SCX_36.3974.3974.3	3	3.488	0.244	1	1359	50	K.YGGLKTPTQLEGAAR.G
MTA2_MOUSE	MK_SCX_52.3422.3422.3	3	5.726	0.508	1	1344.6	44.736843	R.GHLSRPEAQSLSPYTTSANR.A
MTAP_MOUSE	MK_SCX_17.6400.6400.2	2	5.676	0.603	1	2127.8	76.47059	K.IGIIGGTGLDDPEILEGR.T
MTAP_MOUSE	MK_SCX_28.5926.5926.3	3	3.181	0.457	1	367.6	29.6875	K.YVDTPFGKPSDALILGK.I
MTAP_MOUSE	MK_SCX_28.5967.5967.2	2	4.105	0.545	1	1915.4	75	K.YVDTPFGKPSDALILGK.I
MTCH2_MOUSE	MK_SCX_25.4896.4896.2	2	6.147	0.571	1	1138.9	60.000004	K.VLQYYQESEKPEELGSVTVQK.E
MTHFS_MOUSE	MK_SCX_38.3800.3800.3	3	3.803	0.496	1	662.8	48.076923	K.TSWNIHQPGEGDVR.E
MTM1_MOUSE	MK_SCX_19.3880.3880.2	2	3.842	0.479	1	1146.7	70	K.VSQDGVSQDVSETVPR.L
MTMR3_MOUSE	MK_SCX_42.4101.4101.2	2	2.766	0.371	1	434.6	66.66667	K.SRLESQYLTSSLR.F
MTMR3_MOUSE	MK_SCX_46.3594.3594.3	3	3.442	0.457	1	806.6	43.333332	R.GHTEVPEVKEEAPLAK.E
MTPN_MOUSE	MK_SCX_18.4728.4728.2	2	5.682	0.593	1	1583.2	68.75	K.GPDGLTALEATDNQAIK.A
MTPN_MOUSE	MK_SCX_25.4553.4553.2	2	2.18	0.237	1	420.8	66.66667	K.NGDLDEVKDYVAK.G
MTPN_MOUSE	MK_SCX_28.4754.4754.2	2	5.187	0.643	1	1819.7	68.42105	K.TVKGPDGLTALEATDNQAIK.A
MTPN_MOUSE	MK_SCX_38.4725.4725.3	3	5.795	0.502	1	1975.1	40.217392	K.GADKTVKGPDGLTALEATDNQAIK.A
MTX1_MOUSE	MK_SCX_20_1.3488.3488.2	2	3.173	0.47	1	747.9	65.38461	R.QTPAAPETEEEPYR.R
MTX1_MOUSE	MK_SCX_21.5412.5412.2	2	4.031	0.551	1	1202.7	70	K.TSNPWQSPSGTLPALR.T
MTX2_MOUSE	MK_SCX_26.9082.9082.3	3	4.565	0.488	1	1108.9	36.904762	K.VPFIHVGNQVVSELGPIVQFVK.A
MTX2_MOUSE	MK_SCX_31.7816.7816.2	2	3.043	0.499	1	618.1	50	R.YGSPYPWPLNHILAYQK.Q
MUCDL_MOUSE	MK_SCX_21.6986.6986.2	2	3.917	0.493	1	1592.9	87.5	K.FFSLEGVNYPALK.L
MUP1_MOUSE	MK_SCX_18.6591.6591.2	2	4.525	0.402	1	1913	70.588234	K.AGEYSVTYDGFNTFTIPK.T
MUP1_MOUSE	MK_SCX_21.6800.6800.3	3	4.142	0.385	1	960.9	33.333336	K.DGETFQLM*GLYGREPDLSSDIK.E
MUP1_MOUSE	MK_SCX_29.6880.6880.2	2	4.635	0.414	1	1865.8	75	K.TDYDNFLMAHLINEK.D
MUP1_MOUSE	MK_SCX_30.6247.6247.3	3	5.261	0.499	1	1906.9	42.391304	K.DGETFQLM*GLYGREPDLSSDIKER.F
MUP1_MOUSE	MK_SCX_30.6733.6733.3	3	5.634	0.512	1	2082.5	40.217392	K.DGETFQLMGLYGREPDLSSDIKER.F
MUP1_MOUSE	MK_SCX_53.4929.4929.3	3	3.473	0.416	1	884.7	43.333332	K.HGILRENIIDLSNANR.C
MUTA_MOUSE	MK_SCX_14.9206.9206.2	2	2.89	0.372	1	627.2	27.419355	R.DQALAEQCLSALTQCAASGDGNILALAVDAAR.A
MUTA_MOUSE	MK_SCX_14.9337.9337.3	3	4.577	0.455	1	1055.9	27.419355	R.DQALAEQCLSALTQCAASGDGNILALAVDAAR.A
MUTA_MOUSE	MK_SCX_20_1.4649.4649.2	2	2.902	0.374	1	1271.4	75	R.AAVQVLDDIEK.C
MUTA_MOUSE	MK_SCX_21.5182.5182.2	2	2.072	0.154	1	471.9	65	R.IIIQEESGIPK.V

MUTA_MOUSE	MK_SCX_26.6588.6588.2	2	3.971	0.437	1	848.1	58.823532	R.ADTLDLPEELPGVKPFTR.G
MUTA_MOUSE	MK_SCX_52.6658.6658.3	3	4.991	0.584	1	1587.7	45.454548	K.GKNPEDLIWHTPEGISIKPLYSR.A
MY18A_MOUSE	MK_SCX_23.7167.7167.3	3	4.514	0.541	1	457.1	32.608696	K.VVSLEAELQDISSQESKDEASLAK.V
MY18A_MOUSE	MK_SCX_26.5315.5315.3	3	4.861	0.327	1	1496.8	40.476192	K.VKDQEEELDEQAGSIQMLEQAK.L
MY18A_MOUSE	MK_SCX_52.14902.14902.2	2	2.217	0.283	1	356.9	54.166668	K.EMESRDEEVEEAR.Q
MY18A_MOUSE	MK_SCX_52.3156.3156.3	3	5.097	0.431	1	2417	60.714287	R.RFDSELSQAHEETQR.E
MYCBP_MOUSE	MK_SCX_21.3614.3614.2	2	2.681	0.311	1	583.2	70	K.LVQYEPPQEEK.R
MYCBP_MOUSE	MK_SCX_24.7589.7589.3	3	3.476	0.432	1	559.7	32.894737	K.VLVALYEEPEKPTSALDFLK.H
MYG1_MOUSE	MK_SCX_21.4860.4860.2	2	2.615	0.342	1	438.1	72.72727	R.LLPEYANAEIVR.T
MYG1_MOUSE	MK_SCX_25.5352.5352.2	2	2.338	0.194	1	359.9	78.57143	R.LPLPEPWR.G
MYH10_MOUSE	MK_SCX_15.7115.7115.2	2	4.679	0.619	1	1532	60.000004	K.DAAGLESQLQDTQELLQEETR.Q
MYH10_MOUSE	MK_SCX_15.7151.7151.3	3	5.237	0.575	1	1159.2	43.75	K.DAAGLESQLQDTQELLQEETR.Q
MYH10_MOUSE	MK_SCX_18.3470.3470.2	2	4.722	0.55	1	1966.4	88.46153	R.ELDDATEANEGLSR.E
MYH10_MOUSE	MK_SCX_18.6400.6400.2	2	4.688	0.404	1	1424.8	68.75	R.TQLEELEDELQATEDAK.L
MYH10_MOUSE	MK_SCX_19.6139.6139.2	2	2.742	0.184	1	467.6	56.666668	R.QLLQANPILESFGNAK.T
MYH10_MOUSE	MK_SCX_19.8608.8608.2	2	5.043	0.51	1	2538.8	76.47059	K.SLEAEILQLQEELASSER.A
MYH10_MOUSE	MK_SCX_21.4595.4595.2	2	3.843	0.447	1	999.1	75	K.IGQLEEQLEQEAK.E
MYH10_MOUSE	MK_SCX_31.3247.3247.3	3	4.835	0.387	1	1392.5	53.571426	R.VEEEEERNQILQNEK.K
MYH10_MOUSE	MK_SCX_47.4442.4442.3	3	5.621	0.444	1	2271.6	48.684208	R.HAEQERDELADEIANSASGK.S
MYH10_MOUSE	MK_SCX_48.7061.7061.3	3	3.093	0.356	1	884.5	41.17647	K.NRLQQELDDLTVDLDHQR.Q
MYH10_MOUSE	MK_SCX_49.6056.6056.3	3	4.383	0.521	1	1548.5	46.875	R.HATALEELSEQLEQAKR.F
MYH10_MOUSE	MK_SCX_50.6818.6818.3	3	3.649	0.379	1	1919.2	51.923077	K.KQELEEILHDLESR.V
MYH10_MOUSE	MK_SCX_52.3124.3124.3	3	3.87	0.494	1	435.5	32.8125	K.RQLEEAEEEATRANASR.R
MYH11_MOUSE	MK_SCX_14.12735.12735.2	2	2.075	0.18	1	387.3	40.625	K.LQDFASTIEVMEEGKKR.L
MYH11_MOUSE	MK_SCX_15.7107.7107.2	2	5.427	0.665	1	1992	60.000004	K.DVASLGSQLQDTQELLQEETR.Q
MYH11_MOUSE	MK_SCX_17.6380.6380.2	2	3.856	0.316	1	1224.9	70	R.LQQELDDLVVDLDNQR.Q
MYH11_MOUSE	MK_SCX_18.8912.8912.2	2	4.112	0.498	1	1424.8	67.64706	K.SLEADLM*QLQEDLAAAER.A
MYH11_MOUSE	MK_SCX_19.6821.6821.2	2	4.642	0.418	1	1747.1	76.666664	K.QLLQANPILEAFGNAK.T
MYH11_MOUSE	MK_SCX_2201.3090.3090.2	2	2.299	0.213	1	579.8	72.22222	K.EVLLQVEDER.K
MYH11_MOUSE	MK_SCX_2201.9584.9584.2	2	2.058	0.177	1	581.5	54.545456	R.TVGQLYKEQLGK.L
MYH11_MOUSE	MK_SCX_44.6363.6363.3	3	4.272	0.62	1	884.8	35	R.KQADLEKEELAEELASSLSGR.N
MYH9_MOUSE	MK_SCX_15.8527.8527.2	2	5.264	0.611	1	1806.6	59.090908	K.LEGDSTDLSDQIAELQAQIAELK.M
MYH9_MOUSE	MK_SCX_17.6421.6421.2	2	4.592	0.547	1	1667.2	75	R.ELETQISELQEDLESER.A
MYH9_MOUSE	MK_SCX_17.6426.6426.2	2	4.177	0.525	1	764.5	55.263157	R.IIGLDQVAGM*SETALPGAFK.T
MYH9_MOUSE	MK_SCX_17.6557.6557.2	2	4.491	0.615	1	1320.3	73.52941	K.LQVELDSVTGLLSQSDSK.S
MYH9_MOUSE	MK_SCX_17.7464.7464.2	2	3.428	0.444	1	593.3	55.263157	R.IIGLDQVAGMSETALPGAFK.T
MYH9_MOUSE	MK_SCX_18.3678.3678.2	2	3.852	0.372	1	998.8	69.230774	R.ELEDATETADAMNR.E
MYH9_MOUSE	MK_SCX_19.10749.10749.3	3	3.979	0.492	1	993.6	29.807693	R.DLGEELEALKTELEDTLDSTAAQQELR.S
MYH9_MOUSE	MK_SCX_19.3086.3086.2	2	3.045	0.389	1	616.8	60.714287	R.LTEM*ETM*QSQLM*AEK.L
MYH9_MOUSE	MK_SCX_19.4009.4009.2	2	3.829	0.182	1	1311	71.42857	R.LTEMETM*QSQLMAEK.L
MYH9_MOUSE	MK_SCX_19.4064.4064.2	2	4.459	0.448	1	941.4	65.38461	R.VAEFTTNLM*EEEEK.S
MYH9_MOUSE	MK_SCX_19.4776.4776.2	2	4.55	0.469	1	1531.9	71.42857	R.LTEMETMQSQLMAEK.L
MYH9_MOUSE	MK_SCX_19.5669.5669.2	2	2.945	0.145	1	588.1	53.333336	K.ANLQIDQINTDLNLER.S
MYH9_MOUSE	MK_SCX_19.6063.6063.2	2	5.223	0.613	1	1296.2	61.11111	K.MQQNIQELEEQLEEEESAR.Q
MYH9_MOUSE	MK_SCX_19.7815.7815.2	2	5.454	0.547	1	2072.8	70.588234	K.SMEAEMIQLQEELAAAER.A
MYH9_MOUSE	MK_SCX_20_1.15653.15653.2	2	2.902	0.315	1	391.7	50	K.QIATLHAQVTDM*KK.K
MYH9_MOUSE	MK_SCX_20_1.4163.4163.2	2	4.519	0.319	1	1527.4	79.16667	K.IAQLEEQLDNETK.E
MYH9_MOUSE	MK_SCX_20_1.7860.7860.2	2	2.782	0.412	1	646.1	65.38461	R.VISGVLQLGNIAFK.K
MYH9_MOUSE	MK_SCX_21.3540.3540.2	2	2.593	0.395	1	600.7	66.66667	R.EMEAELEDER.K
MYH9_MOUSE	MK_SCX_21.8028.8028.3	3	3.11	0.318	1	500.6	26.136362	K.DFSALESQLQDTQELLQEENRQK.L
MYH9_MOUSE	MK_SCX_2201.8105.8105.3	3	4.711	0.439	1	664.1	31.730768	R.ALEQQVEEM*KTQLEELEDELQATEDAK.L

MYH9_MOUSE	MK_SCX_2201.9978.9978.3	3	3.805	0.229	1	638.5	28.846153	R.ALEQQVEEMKTQLEELEDELQATEDAK.L
MYH9_MOUSE	MK_SCX_25.7738.7738.3	3	4.695	0.559	1	1353	33.695652	K.LTKDFSALESQLQDTQELLQEENR.Q
MYH9_MOUSE	MK_SCX_26.7031.7031.3	3	3.676	0.387	1	794	48.333332	R.LQQELDDLLVDLDHQR.Q
MYH9_MOUSE	MK_SCX_26.7115.7115.2	2	5.74	0.497	1	2922.8	86.666664	R.LQQELDDLLVDLDHQR.Q
MYH9_MOUSE	MK_SCX_27.7655.7655.3	3	4.19	0.34	1	894	33.75	K.YLYVDKNFINNPLAQADWAAK.K
MYH9_MOUSE	MK_SCX_28.4139.4139.2	2	4.169	0.462	1	2048.6	78.57143	R.TEMEDLMSSKDDVGK.S
MYH9_MOUSE	MK_SCX_30.7045.7045.3	3	5.381	0.537	1	1642.9	40.27778	K.IRELETQISELQEDLESER.A
MYH9_MOUSE	MK_SCX_33.5694.5694.3	3	3.902	0.329	1	1000.5	43.75	K.KANLQIDQINTDLNLER.S
MYH9_MOUSE	MK_SCX_35.3220.3220.3	3	3.354	0.371	1	1348.6	48.214287	R.NAEQFKDQADKASTR.L
MYH9_MOUSE	MK_SCX_38.3886.3886.3	3	4.324	0.325	1	1604.1	56.81818	K.KVEAQLQELQVK.F
MYH9_MOUSE	MK_SCX_43.4380.4380.2	2	2.869	0.421	1	580.8	83.33333	R.RGDLPFVVTR.R
MYH9_MOUSE	MK_SCX_43.4626.4626.3	3	4.315	0.562	1	779.3	45.3125	K.TLEDEAKTHEAQIQEMR.Q
MYH9_MOUSE	MK_SCX_46.8343.8343.3	3	5.241	0.337	1	4358	60.294117	K.TRLQQELDDLLVDLDHQR.Q
MYH9_MOUSE	MK_SCX_49.5036.5036.3	3	4.579	0.457	1	932.5	40.625	K.HSQAVEELADQLEQTKR.V
MYH9_MOUSE	MK_SCX_50.4399.4399.3	3	3.625	0.478	1	1400	41.666664	R.HEMPPHIYAITDTAYR.S
MYH9_MOUSE	MK_SCX_52.3191.3191.3	3	4.245	0.342	1	1763.9	51.5625	K.RQLEEAEEEAQRANASR.R
MYH9_MOUSE	MK_SCX_52.3331.3331.3	3	4.527	0.486	1	1134.5	41.17647	K.DLEAHIDTANKNREEAIK.Q
MYL6_MOUSE	MK_SCX_14.3716.3716.2	2	3.443	0.469	1	1479.7	90	K.ILYSQCGDVM*R.A
MYL6_MOUSE	MK_SCX_14.4496.4496.2	2	3.822	0.542	1	1273	90	K.ILYSQCGDVMR.A
MYL6_MOUSE	MK_SCX_21.3940.3940.2	2	3.848	0.451	1	859.6	70.83333	R.ALGQNPTNAEVLK.V
MYL6_MOUSE	MK_SCX_26.7794.7794.2	2	4.537	0.481	1	814.3	66.66667	K.VLDFEHFLPM*LQTVAK.N
MYL6_MOUSE	MK_SCX_26.7856.7856.3	3	3.733	0.449	1	1404.8	51.666664	K.VLDFEHFLPM*LQTVAK.N
MYL6_MOUSE	MK_SCX_26.8786.8786.2	2	5.226	0.595	1	1184	70	K.VLDFEHFLPMLQTVAK.N
MYL6_MOUSE	MK_SCX_26.9031.9031.3	3	5.223	0.475	1	1658.7	51.666664	K.VLDFEHFLPMLQTVAK.N
MYL6_MOUSE	MK_SCX_27.4278.4278.2	2	3.499	0.201	1	2165.5	83.33333	R.VFDKEGNGTVMGAEIR.H
MYL6_MOUSE	MK_SCX_28.3663.3663.2	2	3.659	0.245	1	1524.1	73.333336	R.VFDKEGNGTVM*GAEIR.H
MYL6_MOUSE	MK_SCX_28.4207.4207.3	3	3.138	0.189	1	1742.5	55	R.VFDKEGNGTVMGAEIR.H
MYL6_MOUSE	MK_SCX_35.4863.4863.3	3	4.611	0.483	1	1906.9	53.571426	K.NKDQGTYEDYVEGLR.V
MYL6_MOUSE	MK_SCX_35.5047.5047.2	2	4.908	0.618	1	2344.7	82.14286	K.NKDQGTYEDYVEGLR.V
MYLK_MOUSE	MK_SCX_18.6865.6865.2	2	3.097	0.375	1	625.4	46.875	K.VSSMPLTEAPAFILPPR.N
MYLK_MOUSE	MK_SCX_19.3390.3390.2	2	4.124	0.582	1	1199	71.875	R.FESQPQSQEVTEGQTVK.F
MYLK_MOUSE	MK_SCX_19.6243.6243.2	2	3.054	0.4	1	511.9	62.5	K.IEGYPDPEVVWFK.D
MYLK_MOUSE	MK_SCX_24.4109.4109.3	3	5.627	0.567	1	626.2	31.25	K.NSAGQAECSCQVTVDDAQTSENTKAPEMK.S
MYLK_MOUSE	MK_SCX_25.6627.6627.2	2	3.404	0.326	1	824.4	58.333332	K.IEGYPDPEVVWFKDDQSIR.E
MYLK_MOUSE	MK_SCX_35.4109.4109.3	3	3.247	0.321	1	566.7	38.46154	K.TIRDLEVVEGSAAR.F
MYLK_MOUSE	MK_SCX_35.4119.4119.2	2	3.623	0.471	1	776.7	65.38461	K.TIRDLEVVEGSAAR.F
MYO1C_MOUSE	MK_SCX_18.6040.6040.2	2	4.112	0.414	1	1172.2	61.764706	R.VLQSLGSEPIQYAVPVVK.Y
MYO1C_MOUSE	MK_SCX_20_1.6569.6569.2	2	3.341	0.52	1	947.5	71.42857	R.LLQSNPVLEAFGNAK.T
MYO1C_MOUSE	MK_SCX_40.3124.3124.3	3	3.371	0.406	1	936.3	42.105263	R.DQAVMISGESGAGKTEATKR.L
MYO5B_MOUSE	MK_SCX_2201.3364.3364.2	2	2.097	0.147	1	547.4	81.25	K.LANEELEVR.K
MYO5B_MOUSE	MK_SCX_50.3793.3793.3	3	3.41	0.319	1	476.2	37.5	K.KVQDLEAAQALAQSDRR.H
MYO6_MOUSE	MK_SCX_13.6338.6338.2	2	2.83	0.276	1	362.7	57.692307	R.CGGIQYLQSAIESR.Q
MYO6_MOUSE	MK_SCX_18.4755.4755.2	2	3.502	0.403	1	848.7	57.894737	R.IAQNESELISDEAQGDM*ALR.R
MYO6_MOUSE	MK_SCX_18.5358.5358.2	2	4.733	0.467	1	743.4	57.894737	R.IAQNESELISDEAQGDMALR.R
MYO6_MOUSE	MK_SCX_19.5938.5938.2	2	4.098	0.242	1	1292.1	75	R.IVEANPLLEAFGNAK.T
MYO6_MOUSE	MK_SCX_25.4695.4695.2	2	2.219	0.187	1	308.3	85.71429	R.QFEEIWER.C
MYO6_MOUSE	MK_SCX_33.4103.4103.2	2	3.535	0.413	1	569.9	77.27273	R.EQIQKEYDALVK.S
MYO6_MOUSE	MK_SCX_38.4552.4552.2	2	2.872	0.234	1	498.5	80	R.NLRDDEGFIIR.Q
MYO6_MOUSE	MK_SCX_42.7442.7442.2	2	3.634	0.505	1	1580.8	76.92308	K.GWWYAHFDGPWIAR.Q
MYO6_MOUSE	MK_SCX_47.4328.4328.3	3	3.623	0.299	1	1009.2	45	R.IPFIRPADQYKDPQNK.K
MYO6_MOUSE	MK_SCX_47.4500.4500.3	3	3.706	0.482	1	846.9	39.0625	K.FNEVVSALKDGKPEVNR.Q

MYO6_MOUSE	MK_SCX_52.3536.3536.3	3	4.14	0.472	1	1166.2	48.214287	R.FFANKETDKQILQNR.K
MYO6_MOUSE	MK_SCX_58.7001.7001.2	2	2.593	0.16	1	397.1	68.75	R.KRGAEILPR.Q
MYO9B_MOUSE	MK_SCX_18.3156.3156.2	2	4.349	0.583	1	1027.9	55	K.SGDPSAGPDAGLSPGSQGDSK.S
MYO9B_MOUSE	MK_SCX_32.8675.8675.2	2	2.046	0.148	1	383.8	53.846157	R.EAIAALLEKLQVDR.Q
MYPT1_MOUSE	MK_SCX_15.9588.9588.2	2	5.503	0.692	1	2956.7	69.047615	K.VGQTAFDVADEDILGYLEELQK.K
MYPT1_MOUSE	MK_SCX_16.9741.9741.2	2	2.569	0.281	1	446.4	38.636364	K.VKFDDGAVFLAACSSGDTDEVLK.L
MYPT1_MOUSE	MK_SCX_2201.2894.2894.2	2	2.119	0.263	1	339.3	68.75	R.QGVDIEAAR.K
MYPT1_MOUSE	MK_SCX_2201.4432.4432.2	2	2.795	0.476	1	548.2	61.11111	R.LAYVTPTIPR.R
MYPT1_MOUSE	MK_SCX_27.3226.3226.3	3	4.899	0.577	1	1566.4	38.88889	R.SLPSSTSTAAKTPPGSSSAGTQSSTSNR.L
MYPT1_MOUSE	MK_SCX_29.4010.4010.3	3	3.038	0.509	1	494.3	35.294117	R.YDSSSTSSSDRYDSLLGR.S
MYPT1_MOUSE	MK_SCX_29.4012.4012.2	2	3.653	0.449	1	303.6	50	R.YDSSSTSSSDRYDSLLGR.S
MYPT1_MOUSE	MK_SCX_32.4059.4059.3	3	3.06	0.161	1	948.1	42.307693	K.AQLHDTNMELTDLK.L
MYPT1_MOUSE	MK_SCX_32.4067.4067.2	2	4.037	0.33	1	1216.7	73.07692	K.AQLHDTNMELTDLK.L
MYPT1_MOUSE	MK_SCX_33.3485.3485.2	2	3.504	0.418	1	933.8	57.14286	R.SYLTPVRDEESESQR.K
MYPT1_MOUSE	MK_SCX_36.4041.4041.3	3	4.141	0.346	1	2657.7	53.333336	R.RSTQGVTLTDLQEAEK.T
MYPT1_MOUSE	MK_SCX_36.4049.4049.2	2	4.576	0.503	1	1667.8	76.666664	R.RSTQGVTLTDLQEAEK.T
MYPT1_MOUSE	MK_SCX_49.3306.3306.3	3	3.682	0.393	1	669	41.666664	R.SYLTPVRDEESESQRK.A
NAC1_MOUSE	MK_SCX_2201.15315.15315.3	3	3.407	0.228	1	483	26.136362	R.VGIIDDDIFEEDENFLVHLSNVR.V
NAC1_MOUSE	MK_SCX_47.2895.2895.3	3	4.491	0.375	1	1429.1	46.05263	R.KAVSM*HEVNM*EM*AENDPVSK.I
NAC1_MOUSE	MK_SCX_47.4590.4590.3	3	5.533	0.571	1	2666.6	50	R.KAVSMHEVNMEMAENDPVSK.I
NACAM_MOUSE	MK_SCX_17.3980.3980.2	2	4.531	0.433	1	2272.7	87.5	K.DIELVM*SQANVSR.A
NACAM_MOUSE	MK_SCX_17.4834.4834.2	2	2.528	0.411	1	579.9	66.66667	K.DIELVMSQANVSR.A
NACAM_MOUSE	MK_SCX_19.4059.4059.2	2	5.457	0.483	1	2505	78.57143	K.IEDLSQQAQLAAAEK.F
NACAM_MOUSE	MK_SCX_20_1.5107.5107.2	2	4.536	0.566	1	885.4	84.61539	K.SPASDTYIVFGEAK.I
NACAM_MOUSE	MK_SCX_31.6043.6043.2	2	3.363	0.326	1	1438	79.16667	K.NILFVITKPDVYK.S
NACAM_MOUSE	MK_SCX_49.5803.5803.3	3	3.815	0.47	1	873	46.42857	K.SKNILFVITKPDVYK.S
NACAM_MOUSE	MK_SCX_49.5812.5812.2	2	2.995	0.569	1	598.1	60.714287	K.SKNILFVITKPDVYK.S
NAGAB_MOUSE	MK_SCX_18.6801.6801.2	2	2.798	0.188	1	427.5	43.75	R.TISPQNMDILQNPLM*IK.I
NAGAB_MOUSE	MK_SCX_18.7088.7088.2	2	4.973	0.433	1	788	62.5	R.TISPQNMDILQNPLMIK.I
NAGAB_MOUSE	MK_SCX_24.6485.6485.2	2	2.332	0.403	1	674.7	75	R.TPPMGWLAWER.F
NALP6_MOUSE	MK_SCX_21.4878.4878.2	2	3.484	0.124	1	797.4	53.571426	K.TLSLTSVELSENSLR.D
NAMPT_MOUSE	MK_SCX_17.8081.8081.2	2	4.803	0.611	1	1207.6	65.789474	R.VIQGDGVDINTLQEIVEGMK.Q
NAMPT_MOUSE	MK_SCX_18.4907.4907.2	2	2.65	0.132	1	522.8	42.105263	K.WSIENVSFGSGGALLQKLTR.D
NAMPT_MOUSE	MK_SCX_19.5300.5300.2	2	4.829	0.582	1	1284.7	82.14286	K.YLLETSGNLDGLEYK.L
NAMPT_MOUSE	MK_SCX_24.6860.6860.2	2	4.249	0.587	1	644.2	47.5	R.STEAPLIIRPDSGNPLDTVLK.V
NAMPT_MOUSE	MK_SCX_42.8964.8964.3	3	5.976	0.442	1	848.9	30.357143	R.TPAGNFVTLEEGKGDLEEYGHDLLHTVFK.N
NAMPT_MOUSE	MK_SCX_50.4235.4235.3	3	3.8	0.431	1	621.8	42.857143	K.EVYREHFQDDVFNER.G
NAMPT_MOUSE	MK_SCX_50.4252.4252.2	2	5.566	0.419	1	1383.2	71.42857	K.EVYREHFQDDVFNER.G
NAPSA_MOUSE	MK_SCX_15.8189.8189.2	2	4.497	0.532	1	1051.4	36.666668	K.VGTGLSLCAQGCSAILDTGTSLITGPSEEIR.A
NAPSA_MOUSE	MK_SCX_15.8371.8371.3	3	3.529	0.276	1	1247.6	32.5	K.VGTGLSLCAQGCSAILDTGTSLITGPSEEIR.A
NAPSA_MOUSE	MK_SCX_18.7756.7756.2	2	3.433	0.436	1	881.4	63.333332	R.ILNPLNGWEQLAELSR.T
NCB5R_MOUSE	MK_SCX_18.7032.7032.2	2	4.865	0.354	1	1216.5	61.11111	K.SVGMIAGGTGITPMLQVIR.A
NCB5R_MOUSE	MK_SCX_2201.3874.3874.2	2	2.745	0.25	1	629.7	92.85714	K.IGDTIEFR.G
NCB5R_MOUSE	MK_SCX_28.5664.5664.3	3	3.366	0.484	1	481.7	43.75	R.STPAITLENPDIKYPLR.L
NCLN_MOUSE	MK_SCX_2201.7107.7107.2	2	2.788	0.21	1	413.9	50	R.LLDFSYEHYQK.A
NCLN_MOUSE	MK_SCX_50.6125.6125.3	3	3.299	0.343	1	1348.5	44.642857	K.KINLADDVLAWEHER.F
NCOA5_MOUSE	MK_SCX_15.5364.5364.3	3	5.668	0.531	1	1290.3	30.303032	K.SQPSSQPLQSGQVLPSATPTPAAPPTSQQELQAK.I
NCOA5_MOUSE	MK_SCX_15.5371.5371.2	2	4.114	0.574	1	468.8	27.272728	K.SQPSSQPLQSGQVLPSATPTPAAPPTSQQELQAK.I
NCOA5_MOUSE	MK_SCX_33.8667.8667.3	3	3.003	0.164	1	610.5	30.882353	R.GGHPPAIQSLINLLADNR.Y
NCPR_MOUSE	MK_SCX_15.7098.7098.2	2	5.126	0.549	1	1588.1	57.14286	R.GMSADPEEYDLADLSSLPEIDK.S
NCPR_MOUSE	MK_SCX_23.7986.7986.3	3	6.759	0.572	1	1546	38.88889	R.LPFKPTTPVIM*VGPGTGVAPFM*GFIQER.A

NCPR_MOUSE	MK_SCX_23.8341.8341.3	3	5.138	0.497	1	1589	37.037037	R.LPFKPTTPVIMVGPGTGVAPFM*GFIQER.A
NCPR_MOUSE	MK_SCX_23.8699.8699.3	3	6.566	0.529	1	2490.4	40.74074	R.LPFKPTTPVIM*VGPGTGVAPFMGFIQER.A
NCPR_MOUSE	MK_SCX_23.9075.9075.3	3	7.214	0.51	1	1910.7	39.814816	R.LPFKPTTPVIMVGPGTGVAPFMGFIQER.A
NCPR_MOUSE	MK_SCX_25.4948.4948.2	2	2.906	0.139	1	703.1	93.75	K.FAVFGLGNK.T
NCPR_MOUSE	MK_SCX_31.4186.4186.3	3	3.065	0.467	1	625	45.833336	R.SDEDYLYREELAR.F
NCPR_MOUSE	MK_SCX_46.4662.4662.2	2	2.567	0.226	1	645.2	87.5	R.RALVPMFVR.K
NCPR_MOUSE	MK_SCX_52.3676.3676.3	3	3.031	0.239	1	1687.3	51.923077	R.RSDEDYLYREELAR.F
NDK3_MOUSE	MK_SCX_18.4312.4312.2	2	4.425	0.508	1	940.2	65.625	R.ALIGATDPGDAM*PGTIR.G
NDK3_MOUSE	MK_SCX_18.4964.4964.2	2	5.122	0.575	1	1357	71.875	R.ALIGATDPGDAMPGTIR.G
NDK3_MOUSE	MK_SCX_26.8612.8612.3	3	4.304	0.435	1	365.6	30.952381	K.YMSSGPVVAMVWQGLDVVHASR.A
NDK3_MOUSE	MK_SCX_30.11696.11696.3	3	3.34	0.298	1	452.9	28.125	R.LVKYM*SSGPVVAM*VWQGLDVVHASR.A
NDKA_MOUSE	MK_SCX_18.8337.8337.2	2	3.538	0.451	1	467.2	46.42857	K.EISLWFQPEELVEYK.S
NDKA_MOUSE	MK_SCX_18.9148.9148.2	2	3.329	0.416	1	466.8	41.666664	K.YMHSGPVVAMVWEGLNVVK.T
NDKA_MOUSE	MK_SCX_2201.4528.4528.2	2	2.434	0.169	1	930.5	83.33333	K.FLQASEDLLK.E
NDKA_MOUSE	MK_SCX_27.10414.10414.2	2	4.599	0.535	1	1389.8	61.11111	K.SAEKEISLWFQPEELVEYK.S
NDKA_MOUSE	MK_SCX_27.10588.10588.3	3	4.246	0.352	1	929.9	37.5	K.SAEKEISLWFQPEELVEYK.S
NDKA_MOUSE	MK_SCX_29.3772.3772.3	3	3.756	0.407	1	638.6	43.75	R.VMLGETNPADSKPGTIR.G
NDKA_MOUSE	MK_SCX_30.3526.3526.3	3	3.695	0.548	1	626.2	46.875	R.VM*LGETNPADSKPGTIR.G
NDKA_MOUSE	MK_SCX_30.3542.3542.2	2	5.036	0.538	1	780.7	62.5	R.VM*LGETNPADSKPGTIR.G
NDKA_MOUSE	MK_SCX_30.3667.3667.2	2	5.026	0.574	1	1211	68.75	R.VMLGETNPADSKPGTIR.G
NDKA_MOUSE	MK_SCX_31.16418.16418.3	3	4.623	0.299	1	1191.3	41.666664	K.YMHSGPVVAMVWEGLNVVK.T
NDKA_MOUSE	MK_SCX_35.4011.4011.2	2	3.419	0.444	1	552.3	63.636364	R.TFIAIKPDGVQR.G
NDKA_MOUSE	MK_SCX_36.4577.4577.2	2	2.76	0.259	1	333.8	72.22222	K.DRPFFTGLVK.Y
NDKA_MOUSE	MK_SCX_39.3988.3988.2	2	2.66	0.293	1	1012.4	93.75	R.GLVGEIIKR.F
NDKA_MOUSE	MK_SCX_52.7530.7530.3	3	5.413	0.58	1	1774.5	34.615387	K.FLQASEDLLKEHYTDLKDRPFFTGLVK.Y
NDKA_MOUSE	MK_SCX_54.5364.5364.2	2	4.57	0.535	1	721.5	62.5	K.EHYTDLKDRPFFTGLVK.Y
NDKB_MOUSE	MK_SCX_18.7092.7092.2	2	5.847	0.644	1	1401.2	66.66667	K.YM*NSGPVVAM*VWEGLNVVK.T
NDKB_MOUSE	MK_SCX_18.7442.7442.2	2	4.952	0.402	1	648.7	52.77778	K.YMNSGPVVAM*VWEGLNVVK.T
NDKB_MOUSE	MK_SCX_18.8631.8631.2	2	5.67	0.498	1	1642.8	69.44444	K.YM*NSGPVVAMVWEGLNVVK.T
NDKB_MOUSE	MK_SCX_18.9186.9186.3	3	4.445	0.503	1	1735	47.22222	K.YMNSGPVVAMVWEGLNVVK.T
NDKB_MOUSE	MK_SCX_18.9238.9238.2	2	6.224	0.655	1	1548.3	69.44444	K.YMNSGPVVAMVWEGLNVVK.T
NDKB_MOUSE	MK_SCX_35.3040.3040.3	3	3.93	0.154	1	545.4	48.076923	R.NIIHGSDSVESAEK.E
NDKB_MOUSE	MK_SCX_35.3043.3043.2	2	3.855	0.365	1	1338.4	80.769226	R.NIIHGSDSVESAEK.E
NDKB_MOUSE	MK_SCX_35.5232.5232.2	2	2.673	0.257	1	484.3	72.22222	K.DRPFFPGLVK.Y
NDKB_MOUSE	MK_SCX_44.7510.7510.2	2	4.931	0.62	1	2393.2	82.14286	K.EIHLWFKPEELIDYK.S
NDKB_MOUSE	MK_SCX_52.8261.8261.3	3	5.689	0.52	1	2137.4	38.392857	R.NIIHGSDSVESAEKEIHLWFKPEELIDYK.S
NDKB_MOUSE	MK_SCX_55.6274.6274.2	2	2.971	0.425	1	320.9	50	K.QHYIDLKDRPFFPGLVK.Y
NDKB_MOUSE	MK_SCX_56.3290.3290.2	2	4.434	0.308	1	1306.3	69.230774	R.ASEEHLKQHYIDLK.D
NDRG1_MOUSE	MK_SCX_13.4051.4051.2	2	4.324	0.578	1	1319.1	82.14286	K.M*ADCGGLPQISQPAK.L
NDRG1_MOUSE	MK_SCX_13.4323.4323.1	1	3.335	0.425	1	786.9	60.714287	K.MADCGGLPQISQPAK.L
NDRG1_MOUSE	MK_SCX_13.4328.4328.2	2	4.5	0.449	1	1329.4	82.14286	K.MADCGGLPQISQPAK.L
NDRG1_MOUSE	MK_SCX_20_1.3541.3541.2	2	2.883	0.461	1	882.4	71.875	R.LNITPNSGATGNNAGPK.S
NDRG1_MOUSE	MK_SCX_20_1.3944.3944.2	2	5.166	0.609	1	1453.4	80	K.YFVQGM*GYM*PSASM*TR.L
NDRG1_MOUSE	MK_SCX_20_1.5388.5388.2	2	3.119	0.368	1	1144.6	66.66667	K.SVIGM*GTGAGAYILTR.F
NDRG1_MOUSE	MK_SCX_21.4442.4442.2	2	5.755	0.354	1	1927.6	83.33333	K.YFVQGM*GYM*PSASMTR.L
NDRG1_MOUSE	MK_SCX_21.4443.4443.3	3	3.166	0.396	1	1307.6	46.666668	K.YFVQGM*GYM*PSASMTR.L
NDRG1_MOUSE	MK_SCX_21.5136.5136.2	2	5.545	0.24	1	1777.8	86.666664	K.YFVQGMGYMPSASM*TR.L
NDRG1_MOUSE	MK_SCX_21.5834.5834.2	2	6.21	0.586	1	2118.9	86.666664	K.YFVQGMGYMPSASMTR.L
NDRG1_MOUSE	MK_SCX_2201.10124.10124.2	2	2.129	0.152	1	310.3	32.5	K.YFVQGMGYM*PSASMTRLMRSR.T
NDRG1_MOUSE	MK_SCX_28.16479.16479.3	3	4.413	0.489	1	769	32.142857	K.LAEAFKYFVQGMGYMPSASMTR.L
NDRG1_MOUSE	MK_SCX_28.7306.7306.3	3	4.265	0.17	1	1400.6	40.476192	K.LAEAFKYFVQGMGYM*PSASMTR.L

NDRG1_MOUSE	MK_SCX_39.5669.5669.2	2	5.85	0.582	1	2355.8	73.333336	R.ELHDVDLAEVKPLVEK.G
NDRG1_MOUSE	MK_SCX_39.5688.5688.3	3	3.471	0.525	1	828.7	41.666664	R.ELHDVDLAEVKPLVEK.G
NDRG1_MOUSE	MK_SCX_41.2978.2978.3	3	4.261	0.413	1	1435.5	43.333332	R.SRTASGSSVTSLEGTR.S
NDRG1_MOUSE	MK_SCX_45.7121.7121.3	3	3.257	0.368	1	576.7	33.333336	R.QHILNDMNPSNLHLFISAYNSR.R
NDRG2_MOUSE	MK_SCX_21.3886.3886.2	2	3.701	0.467	1	1393.4	80.769226	R.TASLTSAASIDGSR.S
NDUA1_MOUSE	MK_SCX_24.4748.4748.2	2	2.643	0.456	1	1080.6	83.33333	R.VQYQWYLM*ER.D
NDUA1_MOUSE	MK_SCX_24.5310.5310.2	2	3.34	0.469	1	649	77.77778	R.VQYQWYLMER.D
NDUA2_MOUSE	MK_SCX_20_1.4768.4768.2	2	3.393	0.482	1	1291.9	73.07692	K.TVSLNNLSADEVTR.A
NDUA2_MOUSE	MK_SCX_38.3986.3986.3	3	3.718	0.453	1	425.7	42.307693	R.SPGSQGVRDFIVQR.Y
NDUA2_MOUSE	MK_SCX_55.3981.3981.3	3	4.203	0.401	1	1196.4	60.000004	K.KAHPNLPILIR.E
NDUA2_MOUSE	MK_SCX_55.4033.4033.2	2	2.738	0.419	1	343.4	65	K.KAHPNLPILIR.E
NDUA4_MOUSE	MK_SCX_21.6722.6722.1	1	2.877	0.485	1	501.3	63.636364	R.LALFNPDVSWDR.K
NDUA4_MOUSE	MK_SCX_21.6907.6907.2	2	3.609	0.408	1	1081.7	81.818184	R.LALFNPDVSWDR.K
NDUA4_MOUSE	MK_SCX_23.4203.4203.2	2	3.612	0.451	1	632.9	77.77778	K.FYSVNVDYSK.L
NDUA4_MOUSE	MK_SCX_24.8025.8025.3	3	3.372	0.16	1	325.4	28.75	R.LALFNPDVSWDRKNNPEPWNK.L
NDUA4_MOUSE	MK_SCX_29.5289.5289.3	3	5.358	0.643	1	1318.4	42.647057	K.LGPNEQYKFYSVNVDYSK.L
NDUA4_MOUSE	MK_SCX_29.5367.5367.2	2	5.024	0.66	1	2286.5	70.588234	K.LGPNEQYKFYSVNVDYSK.L
NDUA4_MOUSE	MK_SCX_33.5827.5827.2	2	4.633	0.456	1	931	75	R.LALFNPDVSWDRK.N
NDUA4_MOUSE	MK_SCX_35.4220.4220.3	3	3.88	0.471	1	716.3	50	K.NNPEPWNKLGPNEQYK.F
NDUA4_MOUSE	MK_SCX_35.4513.4513.2	2	5.188	0.492	1	1368.9	73.333336	K.NNPEPWNKLGPNEQYK.F
NDUA4_MOUSE	MK_SCX_43.3690.3690.2	2	2.567	0.264	1	759.8	72.22222	-.MLRQILGQAK.K
NDUA4_MOUSE	MK_SCX_52.3607.3607.2	2	5.26	0.435	1	1247.7	65.625	R.KNNPEPWNKLGPNEQYK.F
NDUA4_MOUSE	MK_SCX_53.3832.3832.3	3	5.584	0.526	1	1040.2	46.875	R.KNNPEPWNKLGPNEQYK.F
NDUA5_MOUSE	MK_SCX_31.3746.3746.3	3	3.65	0.494	1	421.6	42.307693	K.YTEQITNEKLDM*VK.A
NDUA5_MOUSE	MK_SCX_31.4168.4168.2	2	4.973	0.546	1	1439	76.92308	K.YTEQITNEKLDMVK.A
NDUA5_MOUSE	MK_SCX_31.4196.4196.3	3	3.984	0.506	1	769.9	51.923077	K.YTEQITNEKLDMVK.A
NDUA5_MOUSE	MK_SCX_34.6751.6751.2	2	5.184	0.465	1	1651.9	78.125	K.WKPWEPLVEEPPANQWK.W
NDUA5_MOUSE	MK_SCX_34.6927.6927.3	3	4.264	0.29	1	1818.2	53.125	K.WKPWEPLVEEPPANQWK.W
NDUA5_MOUSE	MK_SCX_50.3503.3503.3	3	4.753	0.47	1	1133.5	51.785713	R.KYTEQITNEKLDM*VK.A
NDUA5_MOUSE	MK_SCX_50.3872.3872.2	2	5.501	0.475	1	2139	82.14286	R.KYTEQITNEKLDMVK.A
NDUA5_MOUSE	MK_SCX_50.3912.3912.3	3	5.476	0.494	1	1775.9	57.14286	R.KYTEQITNEKLDMVK.A
NDUA6_MOUSE	MK_SCX_28.5481.5481.2	2	4.531	0.465	1	964.6	70	R.EVPNTVHLM*QLDITVK.Q
NDUA6_MOUSE	MK_SCX_32.3801.3801.3	3	3.085	0.492	1	1143.1	42.1875	R.QAAAAAASTSVKPIFSR.D
NDUA6_MOUSE	MK_SCX_32.3815.3815.2	2	4.352	0.613	1	658.6	65.625	R.QAAAAAASTSVKPIFSR.D
NDUA7_MOUSE	MK_SCX_16.3754.3754.2	2	2.667	0.156	1	1531.5	85	R.NWASGQDLQAK.L
NDUA7_MOUSE	MK_SCX_2201.2075.2075.2	2	4.175	0.57	1	2142.3	81.818184	K.AAESSAMAATEK.K
NDUA7_MOUSE	MK_SCX_36.4154.4154.2	2	4.57	0.477	1	1660.2	76.92308	R.REVVPPSIIMSSQK.A
NDUA7_MOUSE	MK_SCX_43.3591.3591.3	3	4.314	0.447	1	2076.6	58.333332	K.LRNWASGQDLQAK.L
NDUA7_MOUSE	MK_SCX_57.2857.2857.3	3	3.662	0.216	1	683.6	37.5	K.RTQPPPKLPVGPSHK.L
NDUA8_MOUSE	MK_SCX_13.6826.6826.2	2	2.13	0.152	1	323.9	60.000004	K.LVNGCALNFFR.Q
NDUA8_MOUSE	MK_SCX_19.6366.6366.2	2	4.601	0.405	1	1358.2	87.5	-.PGIVELPTLEELK.V
NDUA8_MOUSE	MK_SCX_25.7057.7057.3	3	5.285	0.581	1	1353.1	44.11765	-.PGIVELPTLEELKVEEVK.V
NDUA8_MOUSE	MK_SCX_25.7064.7064.2	2	5.339	0.587	1	1134.2	70.588234	-.PGIVELPTLEELKVEEVK.V
NDUA8_MOUSE	MK_SCX_29.10287.10287.3	3	4.059	0.348	1	1087.5	31.818182	K.SHCAEPFTEYWTCLDYSNM*QLFR.H
NDUA8_MOUSE	MK_SCX_29.11823.11823.3	3	4.753	0.453	1	928.9	28.40909	K.SHCAEPFTEYWTCLDYSNMQLFR.H
NDUA8_MOUSE	MK_SCX_31.12752.12752.3	3	4.703	0.569	1	470.3	28.125	-.PGIVELPTLEELKVEEVKVSSAVLK.A
NDUA8_MOUSE	MK_SCX_34.5405.5405.2	2	3.552	0.436	1	736.6	66.66667	K.LGWVRPDLGQLSK.V
NDUA8_MOUSE	MK_SCX_47.3710.3710.2	2	4.208	0.496	1	765.3	59.375	R.ARPEPNPVIEGDLKPAK.H
NDUA8_MOUSE	MK_SCX_47.3711.3711.3	3	6.066	0.433	1	2827.4	59.375	R.ARPEPNPVIEGDLKPAK.H
NDUA8_MOUSE	MK_SCX_57.3082.3082.3	3	4.656	0.466	1	1012.6	57.14286	K.VKTDRPLPENPYHSR.A
NDUA8_MOUSE	MK_SCX_57.3131.3131.2	2	3.71	0.443	1	394.8	53.571426	K.VKTDRPLPENPYHSR.A

NDUA8_MOUSE	MK_SCX_58.12439.12439.3	3	4.507	0.409	1	506.3	38.75	R.ARPEPNPVIEGDLKPAKHGTR.F
NDUA9_MOUSE	MK_SCX_19.7866.7866.2	2	3.724	0.562	1	1617.7	83.33333	R.NFDFEDVFVNIPR.A
NDUA9_MOUSE	MK_SCX_2201.8462.8462.3	3	7.091	0.626	1	1951	41.346153	R.IHISDVM*PTDLPGLEDLGVQPTPLELK.S
NDUA9_MOUSE	MK_SCX_2201.9144.9144.2	2	4.861	0.49	1	1262.8	48.076923	R.IHISDVMPTDLPGLEDLGVQPTPLELK.S
NDUA9_MOUSE	MK_SCX_2201.9350.9350.3	3	6.314	0.519	1	1392.2	37.5	R.IHISDVMPTDLPGLEDLGVQPTPLELK.S
NDUA9_MOUSE	MK_SCX_49.3918.3918.2	2	2.683	0.448	1	837.7	87.5	R.FLNHFANYR.W
NDUAA_MOUSE	MK_SCX_21.6158.6158.2	2	3.724	0.512	1	697.5	76.92308	K.VTSAYLQDIENAYK.K
NDUAA_MOUSE	MK_SCX_21.8927.8927.3	3	5.578	0.529	1	1169.5	40.625	R.LTLPEYLPPHAVIYIDVPVPEVQSR.I
NDUAA_MOUSE	MK_SCX_2201.7614.7614.2	2	4.176	0.505	1	1722.8	90	R.YGLLAAILGDK.T
NDUAA_MOUSE	MK_SCX_23.8184.8184.3	3	4.298	0.47	1	466.4	26.041666	K.TEVLNYTTIPVYLPEITIGAHQGSR.I
NDUAA_MOUSE	MK_SCX_25.9495.9495.3	3	4.888	0.488	1	1818.1	40.476192	R.LLQYADALEHLLSTGQGVVLER.S
NDUAA_MOUSE	MK_SCX_32.5329.5329.2	2	4.662	0.514	1	1577.6	75	K.VTSAYLQDIENAYKK.T
NDUAA_MOUSE	MK_SCX_32.6375.6375.3	3	3.639	0.498	1	899.2	48.076923	R.YGLLAAILGDKTTK.K
NDUAA_MOUSE	MK_SCX_32.6385.6385.2	2	3.791	0.507	1	1832.8	80.769226	R.YGLLAAILGDKTTK.K
NDUAA_MOUSE	MK_SCX_41.7424.7424.2	2	3.325	0.421	1	1832.9	83.33333	K.LRYGLLAAILGDK.T
NDUAB_MOUSE	MK_SCX_21.6542.6542.2	2	2.528	0.229	1	682.9	85	K.LEGWELFPTPK.V
NDUAB_MOUSE	MK_SCX_33.6735.6735.2	2	3.366	0.556	1	1053.8	76.92308	K.IGKLEGWELFPTPK.V
NDUAB_MOUSE	MK_SCX_33.6823.6823.3	3	3.419	0.343	1	535.6	40.384613	K.IGKLEGWELFPTPK.V
NDUAC_MOUSE	MK_SCX_19.4970.4970.2	2	5.028	0.65	1	860.9	65.625	K.FNVSATPEQYVPYSTTR.K
NDUAC_MOUSE	MK_SCX_30.6876.6876.2	2	5.709	0.619	1	939.5	75	K.NTFWDVDGSMVPPEWHR.W
NDUAC_MOUSE	MK_SCX_30.6885.6885.3	3	3.775	0.406	1	1121.6	45.3125	K.NTFWDVDGSMVPPEWHR.W
NDUAC_MOUSE	MK_SCX_31.5155.5155.3	3	3.083	0.414	1	525.3	35.294117	K.FNVSATPEQYVPYSTTRK.K
NDUAC_MOUSE	MK_SCX_42.3848.3848.3	3	3.729	0.324	1	933.6	40.27778	R.IGTLVGEDKYGNKYYEDNK.Q
NDUAC_MOUSE	MK_SCX_54.6128.6128.3	3	6.175	0.529	1	1460	39.583336	R.KFIWTNHKFNVSATPEQYVPYSTTR.K
NDUAC_MOUSE	MK_SCX_56.2438.2438.3	3	3.983	0.347	1	1373.8	52.083332	K.RGVQQVTGHGGLR.G
NDUAD_MOUSE	MK_SCX_19.4096.4096.2	2	2.667	0.232	1	444.8	60.000004	K.QDMPPPGGYGPIDYKR.N
NDUAD_MOUSE	MK_SCX_20_1.4143.4143.2	2	3.288	0.265	1	719.7	70	R.ENLEEEAIIM*K.D
NDUAD_MOUSE	MK_SCX_21.4940.4940.2	2	2.29	0.367	1	747.3	87.5	R.LLIEDLEAR.I
NDUAD_MOUSE	MK_SCX_21.5223.5223.2	2	3.271	0.41	1	773.6	85	R.IALM*PLFQAEK.D
NDUAD_MOUSE	MK_SCX_21.6539.6539.2	2	3.205	0.357	1	700.6	85	R.IALMPLFQAEK.D
NDUAD_MOUSE	MK_SCX_21.8620.8620.1	1	2.411	0.248	1	572.5	62.5	R.WVPPLIGEMYGLR.T
NDUAD_MOUSE	MK_SCX_28.6799.6799.2	2	6.172	0.496	1	2174.3	75	R.ENLEEEAIIMKDVPNWK.V
NDUAD_MOUSE	MK_SCX_32.3715.3715.3	3	4.571	0.467	1	995.8	53.125	K.VKQDM*PPPGGYGPIDYK.R
NDUAD_MOUSE	MK_SCX_32.4122.4122.3	3	5.261	0.473	1	1649.7	50	K.VKQDMPPPGGYGPIDYK.R
NDUAD_MOUSE	MK_SCX_32.4177.4177.2	2	4.912	0.476	1	1064.6	75	K.VKQDMPPPGGYGPIDYK.R
NDUAD_MOUSE	MK_SCX_40.5039.5039.2	2	2.853	0.321	1	1034.5	88.88889	R.RLLIEDLEAR.I
NDUAD_MOUSE	MK_SCX_47.3657.3657.3	3	5.764	0.571	1	3108	52.941177	K.VKQDM*PPPGGYGPIDYKR.N
NDUAD_MOUSE	MK_SCX_47.4226.4226.2	2	4.971	0.588	1	868.5	67.64706	K.VKQDMPPPGGYGPIDYKR.N
NDUAD_MOUSE	MK_SCX_47.4254.4254.3	3	6.082	0.61	1	2288.3	52.941177	K.VKQDMPPPGGYGPIDYKR.N
NDUAD_MOUSE	MK_SCX_50.5227.5227.2	2	2.888	0.218	1	309.7	46.153847	R.IALMPLFQAEKDRR.T
NDUB3_MOUSE	MK_SCX_19.7245.7245.2	2	2.732	0.179	1	614.5	53.125	R.YM*GGFAGNITFPSVILK.G
NDUB3_MOUSE	MK_SCX_19.7845.7845.2	2	4.577	0.506	1	1032.2	59.375	R.YMGGFAGNITFPSVILK.G
NDUB3_MOUSE	MK_SCX_24.3992.3992.2	2	2.42	0.426	1	710.3	83.33333	K.MELPDYR.Q
NDUB4_MOUSE	MK_SCX_25.6736.6736.3	3	6.11	0.617	1	1599.1	35.416664	K.YKPAPLATLPSTLDPAEYDVSPETR.R
NDUB4_MOUSE	MK_SCX_25.6751.6751.2	2	4.234	0.47	1	698.1	50	K.YKPAPLATLPSTLDPAEYDVSPETR.R
NDUB4_MOUSE	MK_SCX_36.6793.6793.3	3	3.431	0.462	1	472	31	K.YKPAPLATLPSTLDPAEYDVSPETRR.A
NDUB4_MOUSE	MK_SCX_37.3943.3943.2	2	3.497	0.48	1	446.2	62.5	R.SANIYPNFRPTPK.N
NDUB5_MOUSE	MK_SCX_21.5360.5360.2	2	3.721	0.408	1	1249.6	70.83333	R.GDGPWYQFPTPEK.E
NDUB5_MOUSE	MK_SCX_31.5973.5973.2	2	3.411	0.314	1	698.5	60.714287	K.TLAILQIESEKAELR.L
NDUB5_MOUSE	MK_SCX_31.6014.6014.3	3	3.655	0.539	1	705.4	46.42857	K.TLAILQIESEKAELR.L
NDUB5_MOUSE	MK_SCX_37.5256.5256.2	2	4.263	0.545	1	1495.6	75	R.ARGDGPWYQFPTPEK.E

NDUB5_MOUSE	MK_SCX_37.5469.5469.3	3	3.545	0.405	1	756.8	44.642857	R.ARGDGPWYQFPTPEK.E
NDUB5_MOUSE	MK_SCX_38.3426.3426.2	2	2.958	0.252	1	698.5	63.636364	R.NFYDGPEKNYEK.T
NDUB5_MOUSE	MK_SCX_38.7741.7741.3	3	3.661	0.556	1	815.5	36.25	R.GDGPWYQFPTPEKEFIDHSPK.A
NDUB5_MOUSE	MK_SCX_53.6340.6340.3	3	5.266	0.537	1	616	31.818182	R.ARGDGPWYQFPTPEKEFIDHSPK.A
NDUB6_MOUSE	MK_SCX_17.6375.6375.2	2	4.251	0.394	1	943.9	67.64706	R.IFPGDTILETGEVIPPM*R.D
NDUB6_MOUSE	MK_SCX_17.6769.6769.2	2	3.969	0.448	1	863.5	70.588234	R.IFPGDTILETGEVIPPMR.D
NDUB6_MOUSE	MK_SCX_28.5674.5674.2	2	2.386	0.171	1	592.3	100	R.FWDNFLR.D
NDUB7_MOUSE	MK_SCX_18.14463.14463.2	2	3.971	0.463	1	734.4	58.333332	R.VMVATQQEMMDAQLTLQQR.D
NDUB7_MOUSE	MK_SCX_21.9002.9002.3	3	5.118	0.571	1	2487.4	43	R.YLWDASVEPDPEKIPSFPPDLGFPER.K
NDUB7_MOUSE	MK_SCX_29.9977.9977.3	3	4.275	0.485	1	1038.3	34.615387	R.YLWDASVEPDPEKIPSFPPDLGFPERK.E
NDUB7_MOUSE	MK_SCX_32.6135.6135.3	3	3.116	0.554	1	426	42.307693	K.IPSFPPDLGFPERK.E
NDUB7_MOUSE	MK_SCX_34.9560.9560.3	3	4.385	0.332	1	513.3	26.923079	R.RYLWDASVEPDPEKIPSFPPDLGFPER.K
NDUB7_MOUSE	MK_SCX_54.6627.6627.3	3	3.237	0.426	1	371.6	23	R.DSFPNFLACKHEQHDWDYCEHLDYVK.R
NDUB8_MOUSE	MK_SCX_15.5571.5571.3	3	5.27	0.658	1	1122.7	37.5	R.VEDYEPYPDDGM*GYGDYPM*LPNR.S
NDUB8_MOUSE	MK_SCX_15.5594.5594.2	2	5.012	0.624	1	1131.4	56.81818	R.VEDYEPYPDDGM*GYGDYPM*LPNR.S
NDUB8_MOUSE	MK_SCX_15.5843.5843.2	2	5.152	0.297	1	1390	56.81818	R.VEDYEPYPDDGM*GYGDYPMLPNR.S
NDUB8_MOUSE	MK_SCX_15.5848.5848.3	3	3.874	0.333	1	993.6	35.227272	R.VEDYEPYPDDGM*GYGDYPMLPNR.S
NDUB8_MOUSE	MK_SCX_15.5958.5958.2	2	4.871	0.471	1	1211.7	56.81818	R.VEDYEPYPDDGMGYGDYPM*LPNR.S
NDUB8_MOUSE	MK_SCX_15.6204.6204.2	2	5.225	0.648	1	1418.1	56.81818	R.VEDYEPYPDDGMGYGDYPMLPNR.S
NDUB8_MOUSE	MK_SCX_15.6206.6206.3	3	4.364	0.581	1	1106.1	34.090908	R.VEDYEPYPDDGMGYGDYPMLPNR.S
NDUB8_MOUSE	MK_SCX_23.4499.4499.2	2	2.594	0.578	1	372.5	65	K.QYPYNNLYLER.G
NDUB8_MOUSE	MK_SCX_32.5455.5455.2	2	3.932	0.408	1	1063.5	81.818184	R.DPWYQWDHSELR.M
NDUB8_MOUSE	MK_SCX_57.13647.13647.3	3	3.56	0.461	1	854.2	40.625	R.SQHERDPWYQWDHSELR.M
NDUB9_MOUSE	MK_SCX_17.4496.4496.2	2	5.53	0.571	1	1075.7	60.526318	K.QLQEETSPDGIM*TEALPPAR.R
NDUB9_MOUSE	MK_SCX_17.5259.5259.2	2	5.2	0.624	1	956.8	60.526318	K.QLQEETSPDGIMTEALPPAR.R
NDUB9_MOUSE	MK_SCX_2201.7014.7014.3	3	7.123	0.597	1	1183.5	33.333336	R.EAEEEFWQNQHPQPYIFPDSPGGTSFER.Y
NDUB9_MOUSE	MK_SCX_2201.7046.7046.2	2	3.755	0.552	1	420	35.185184	R.EAEEEFWQNQHPQPYIFPDSPGGTSFER.Y
NDUB9_MOUSE	MK_SCX_27.4357.4357.3	3	3.385	0.412	1	481	28.75	K.QLQEETSPDGIM*TEALPPARR.E
NDUB9_MOUSE	MK_SCX_27.5459.5459.3	3	3.606	0.393	1	819.8	35	K.QLQEETSPDGIMTEALPPARR.E
NDUB9_MOUSE	MK_SCX_27.5478.5478.2	2	3.435	0.439	1	304.9	40	K.QLQEETSPDGIMTEALPPARR.E
NDUB9_MOUSE	MK_SCX_39.4145.4145.2	2	2.153	0.346	1	571	72.22222	K.AMYPDYFSKR.E
NDUB9_MOUSE	MK_SCX_45.6792.6792.3	3	4.031	0.392	1	870	37.5	R.YECYKVPEWCLDYWHPSEK.A
NDUB9_MOUSE	MK_SCX_46.6529.6529.2	2	3.188	0.441	1	491.9	44.444447	R.YECYKVPEWCLDYWHPSEK.A
NDUB9_MOUSE	MK_SCX_47.7041.7041.2	2	3.364	0.491	1	305.4	60.000004	R.EGDLPPLWWHIVTRPR.E
NDUBA_MOUSE	MK_SCX_18.6671.6671.2	2	5.369	0.684	1	1070.6	63.88889	R.TPAPSPQTSLPNPITYLTK.A
NDUBA_MOUSE	MK_SCX_19.7957.7957.2	2	4.98	0.595	1	1898.5	84.61539	K.AYDLVVDWPVTLVR.E
NDUBA_MOUSE	MK_SCX_20_1.4872.4872.2	2	3.351	0.232	1	1553.8	77.27273	K.VDQEIM*NIIQER.L
NDUBA_MOUSE	MK_SCX_2201.5391.5391.2	2	4.467	0.508	1	1616.2	90	R.YLDLGAYYSAR.K
NDUBA_MOUSE	MK_SCX_2201.8576.8576.3	3	4.175	0.336	1	1116.1	32.954548	R.VPDITECKEGDVLCIYEAEMQWR.R
NDUBA_MOUSE	MK_SCX_26.6925.6925.2	2	4.727	0.429	1	1691.6	82.14286	R.DFKVDQEIM*NIIQER.L
NDUBA_MOUSE	MK_SCX_26.7332.7332.3	3	5.898	0.641	1	758.1	28.030304	-.PDSWDKDVYPEPPSRTPAPSPQTSLPNPITYLTK.A
NDUBA_MOUSE	MK_SCX_26.7743.7743.3	3	4.692	0.362	1	1400.5	51.785713	R.DFKVDQEIMNIIQER.L
NDUBA_MOUSE	MK_SCX_26.7744.7744.2	2	5.246	0.443	1	1891.7	78.57143	R.DFKVDQEIMNIIQER.L
NDUBA_MOUSE	MK_SCX_29.4105.4105.3	3	3.632	0.361	1	1557.4	53.571426	-.PDSWDKDVYPEPPSR.T
NDUBA_MOUSE	MK_SCX_29.4113.4113.2	2	4.754	0.594	1	2155.1	78.57143	-.PDSWDKDVYPEPPSR.T
NDUBA_MOUSE	MK_SCX_35.11060.11060.3	3	3.544	0.441	1	309.3	28.260868	R.RVPDITECKEGDVLCIYEAEM*QWR.R
NDUBA_MOUSE	MK_SCX_35.14280.14280.3	3	4.9	0.536	1	654.1	29.347824	R.RVPDITECKEGDVLCIYEAEMQWR.R
NDUBA_MOUSE	MK_SCX_51.6330.6330.3	3	4.778	0.426	1	1619.8	53.333336	R.RDFKVDQEIM*NIIQER.L
NDUBA_MOUSE	MK_SCX_51.7080.7080.2	2	4.456	0.507	1	1134.7	76.666664	R.RDFKVDQEIMNIIQER.L
NDUBA_MOUSE	MK_SCX_51.7162.7162.3	3	4.835	0.393	1	2329.4	53.333336	R.RDFKVDQEIMNIIQER.L
NDUBB_MOUSE	MK_SCX_16.4588.4588.2	2	3.535	0.358	1	490.8	44.736843	R.EPTM*QWQEDPEPEDENVYAK.N

NDUBB_MOUSE	MK_SCX_16.5062.5062.2	2	4.496	0.587	1	742.1	52.63158	R.EPTMQWQEDPEPEDENVYAK.N
NDUBB_MOUSE	MK_SCX_16.5830.5830.2	2	3.02	0.42	1	797.1	62.5	R.EVNGLPIM*ESNYFDPSK.I
NDUBB_MOUSE	MK_SCX_16.6619.6619.2	2	3.55	0.466	1	741.8	68.75	R.EVNGLPIMESNYFDPSK.I
NDUBB_MOUSE	MK_SCX_27.5899.5899.3	3	3.432	0.478	1	1097.2	43.055553	K.YREVNGLPIM*ESNYFDPSK.I
NDUBB_MOUSE	MK_SCX_27.6748.6748.2	2	3.805	0.432	1	1301.7	69.44444	K.YREVNGLPIMESNYFDPSK.I
NDUBB_MOUSE	MK_SCX_27.6820.6820.3	3	3.59	0.267	1	1122.6	41.666664	K.YREVNGLPIMESNYFDPSK.I
NDUBB_MOUSE	MK_SCX_28.4201.4201.3	3	5.021	0.441	1	1539.2	41.666664	R.QREPTM*QWQEDPEPEDENVYAK.N
NDUBB_MOUSE	MK_SCX_28.4581.4581.3	3	5.416	0.48	1	1674.8	41.666664	R.QREPTMQWQEDPEPEDENVYAK.N
NEB2_MOUSE	MK_SCX_30.7341.7341.3	3	4.184	0.4	1	809.3	31.25	R.ERPGEQSEVAQLIQQTLEQER.W
NEB2_MOUSE	MK_SCX_41.3343.3343.3	3	3.817	0.542	1	831.5	40.789474	R.FDSKPAPSAQPAPPPHPPSR.L
NEB2_MOUSE	MK_SCX_53.3351.3351.3	3	4.764	0.415	1	1804.9	60.416668	K.HAVTEAEIQQLKR.K
NECP1_MOUSE	MK_SCX_15.7203.7203.2	2	4.954	0.688	1	1108.4	50	K.VSGELFAQAPVEQYPGIAVETVTDSSR.Y
NECP1_MOUSE	MK_SCX_15.7239.7239.3	3	5.425	0.639	1	2449.4	37.5	K.VSGELFAQAPVEQYPGIAVETVTDSSR.Y
NECP1_MOUSE	MK_SCX_45.3159.3159.3	3	3.534	0.407	1	505.1	35.9375	K.QETEISKESQEMDNRPK.L
NECP2_MOUSE	MK_SCX_15.6852.6852.2	2	5.294	0.652	1	803.5	44.230766	R.TSGELFAQAPVDQFPGTAVESVTDSSR.Y
NECP2_MOUSE	MK_SCX_15.6859.6859.3	3	5.362	0.565	1	2346.2	37.5	R.TSGELFAQAPVDQFPGTAVESVTDSSR.Y
NECP2_MOUSE	MK_SCX_2201.5027.5027.2	2	3.671	0.366	1	746	65.38461	R.ASEWQLDQPSWSGR.L
NECP2_MOUSE	MK_SCX_33.4661.4661.2	2	3.996	0.352	1	688.2	63.88889	R.ARPTSAGGLSLLPPPPGGK.S
NEDD1_MOUSE	MK_SCX_16.6640.6640.2	2	3.72	0.505	1	411.1	42.5	R.EAPSPSIATVLPQPVTTALGK.G
NEDD1_MOUSE	MK_SCX_31.15619.15619.2	2	2.571	0.2	1	338.1	54.545456	K.QPIRHIKYSLFR.K
NEDD4_MOUSE	MK_SCX_15.7484.7484.2	2	4.61	0.496	1	754.9	55.263157	R.DDFLGQVDVPLYPLPTENPR.M
NEDD4_MOUSE	MK_SCX_17.6700.6700.2	2	6.061	0.618	1	2036.3	81.25	R.VTLYDPMSGILTSVQTK.T
NEDD4_MOUSE	MK_SCX_56.5291.5291.3	3	3.043	0.408	1	585.8	37.5	R.MERPYTFKDFVLHPR.S
NEDD8_MOUSE	MK_SCX_27.4733.4733.2	2	3.751	0.344	1	807.8	69.230774	K.EIEIDIEPTDKVER.I
NEDD8_MOUSE	MK_SCX_28.4859.4859.3	3	4.25	0.352	1	786.8	45	K.TLTGKEIEIDIEPTDK.V
NEDD8_MOUSE	MK_SCX_38.5322.5322.3	3	4.268	0.453	1	1167.3	40.27778	K.TLTGKEIEIDIEPTDKVER.I
NEK4_MOUSE	MK_SCX_25.4343.4343.3	3	6.386	0.519	1	2278.1	43.75	R.TPSAVEPLKPQEEDQPIPAQR.F
NENF_MOUSE	MK_SCX_25.5722.5722.3	3	3.226	0.307	1	445.5	33.82353	K.MSLDPADLTHDTTGLTAK.E
NENF_MOUSE	MK_SCX_25.5769.5769.2	2	4.471	0.508	1	1340.5	70.588234	K.MSLDPADLTHDTTGLTAK.E
NEO1_MOUSE	MK_SCX_17.9322.9322.2	2	3.611	0.427	1	759.3	52.941177	R.TFTPFYFLVEPVDTLSVR.G
NEP_MOUSE	MK_SCX_15.8018.8018.2	2	5.914	0.631	1	693.8	44.230766	K.LLPDIYGWPVASDNWDQTYGTSWTAEK.S
NEP_MOUSE	MK_SCX_17.4925.4925.2	2	5.106	0.542	1	584.1	58.823532	R.QEQSLPIDENQLSLEM*NK.V
NEP_MOUSE	MK_SCX_17.5556.5556.2	2	5.742	0.609	1	683.5	64.70589	R.QEQSLPIDENQLSLEMNK.V
NEP_MOUSE	MK_SCX_19.6971.6971.2	2	2.572	0.163	1	458.1	62.5	R.EDEYFENIIQNLK.F
NEP_MOUSE	MK_SCX_21.7482.7482.2	2	2.656	0.239	1	1236.7	85	R.FIMDLVSSLSR.N
NEP_MOUSE	MK_SCX_23.4011.4011.2	2	4.066	0.547	1	2492.6	83.33333	K.ALYGTTSETATWR.R
NEP_MOUSE	MK_SCX_24.4514.4514.3	3	3.796	0.399	1	785.4	43.333332	K.DVLQEPKTEDIVAVQK.A
NEP_MOUSE	MK_SCX_38.6074.6074.2	2	2.484	0.474	1	1210.2	90	K.HVVEDLIAQIR.E
NEP_MOUSE	MK_SCX_48.3234.3234.3	3	4.575	0.396	1	928.8	44.11765	-.GRSESQM*DITDINAPKPK.K
NEP_MOUSE	MK_SCX_48.3792.3792.3	3	5.346	0.557	1	1689.8	45.588234	-.GRSESQMDITDINAPKPK.K
NET4_MOUSE	MK_SCX_19.4718.4718.2	2	4.021	0.541	1	705.2	78.57143	R.ALSPPYDIENPYSAK.V
NFIA_MOUSE	MK_SCX_47.3849.3849.3	3	3.882	0.485	1	882.2	44.230766	R.AVKDELLSEKPEVK.Q
NFIB_MOUSE	MK_SCX_23.5520.5520.2	2	3.311	0.254	1	979.8	80	K.SGVFNVSELVR.V
NFIB_MOUSE	MK_SCX_46.4172.4172.3	3	3.331	0.443	1	660.2	42.307693	R.AVKDELLSEKPEIK.Q
NFIC_MOUSE	MK_SCX_2201.8284.8284.3	3	4.683	0.637	1	603	26.612906	R.TPVVTGTGPNFSLGELQGHLAYDLNPASAGMR.R
NFS1_MOUSE	MK_SCX_18.8878.8878.2	2	4.168	0.399	1	1171.8	70	R.EMSPLWEMVQDGIDLK.S
NFS1_MOUSE	MK_SCX_19.3725.3725.2	2	4.431	0.558	1	1228.5	83.33333	R.FTTEEEVDYTAEK.C
NFS1_MOUSE	MK_SCX_41.3788.3788.3	3	4.673	0.38	1	1415.3	50	R.THAYGWESEAAMER.A
NFS1_MOUSE	MK_SCX_41.3808.3808.2	2	5.2	0.648	1	2074.7	88.46153	R.THAYGWESEAAMER.A
NFYA_MOUSE	MK_SCX_17.6747.6747.2	2	3.219	0.288	1	421.5	55.555557	R.IPLPGAEMLEEEPLYVNAK.Q
NHERF_MOUSE	MK_SCX_13.4040.4040.2	2	2.75	0.345	1	1389.4	90.909096	R.IVEVNGVCMEGK.Q

NHERF_MOUSE	MK_SCX_15.12903.12903.3	3	3.451	0.414	1	351.9	25.925926	R.QVSTEPSSTSSSSSDPILDLNISLAVAK.E
NHERF_MOUSE	MK_SCX_15.9149.9149.2	2	3.948	0.572	1	488	35.185184	R.QVSTEPSSTSSSSSDPILDLNISLAVAK.E
NHERF_MOUSE	MK_SCX_19.2861.2861.2	2	4.763	0.523	1	1366	65.625	R.SASSDTSEELNSQDSPK.R
NHERF_MOUSE	MK_SCX_20_1.3561.3561.3	3	4.551	0.518	1	2294.4	61.538464	R.AVDPDSPAEASGLR.A
NHERF_MOUSE	MK_SCX_20_1.3575.3575.2	2	4.357	0.668	1	1438.5	84.61539	R.AVDPDSPAEASGLR.A
NHERF_MOUSE	MK_SCX_2201.2832.2832.2	2	2.861	0.35	1	569.8	77.77778	R.LVEPGSPAEK.S
NHERF_MOUSE	MK_SCX_2201.5990.5990.2	2	3.298	0.273	1	786.2	47.727272	K.VIPSQEHLDGPLPEPFSNGEIQK.E
NHERF_MOUSE	MK_SCX_25.3018.3018.2	2	3.831	0.589	1	809	50	K.SEQAEPPAAADTHEAGDQNEAEK.S
NHERF_MOUSE	MK_SCX_26.4937.4937.2	2	3.867	0.375	1	1147.4	61.11111	K.SGLLAGDRLVEVNGENVEK.E
NHERF_MOUSE	MK_SCX_29.3952.3952.2	2	3.524	0.399	1	373.6	56.25	R.EALVEPASESPRPALAR.S
NHERF_MOUSE	MK_SCX_30.5439.5439.2	2	4.584	0.492	1	1931.2	83.33333	K.LLVVDKETDEFFK.K
NHERF_MOUSE	MK_SCX_30.5659.5659.3	3	3.152	0.389	1	684.5	29.807693	K.VIPSQEHLDGPLPEPFSNGEIQKESSR.E
NHERF_MOUSE	MK_SCX_31.2643.2643.3	3	3.501	0.447	1	888.3	47.058823	R.SASSDTSEELNSQDSPKR.Q
NHERF_MOUSE	MK_SCX_37.4387.4387.2	2	3.81	0.404	1	1016.6	57.894737	R.LVEVNGENVEKETHQQVVSR.I
NHERF_MOUSE	MK_SCX_40.4211.4211.3	3	4.175	0.405	1	1317.9	48.684208	R.LVEVNGENVEKETHQQVVSR.I
NHERF_MOUSE	MK_SCX_43.4075.4075.3	3	4.388	0.547	1	525.3	30.000002	K.ESSREALVEPASESPRPALAR.S
NHERF_MOUSE	MK_SCX_43.5168.5168.2	2	5.58	0.459	1	2522.9	84.61539	K.LLVVDKETDEFFKK.C
NHERF_MOUSE	MK_SCX_44.5131.5131.3	3	3.849	0.458	1	689.1	48.076923	K.LLVVDKETDEFFKK.C
NHERF_MOUSE	MK_SCX_44.5494.5494.3	3	6.412	0.454	1	1249.3	33.333336	K.SGLLAGDRLVEVNGENVEKETHQQVVSR.I
NHERF_MOUSE	MK_SCX_48.3104.3104.3	3	3.498	0.508	1	431	35.576923	K.SEQAEPPAAADTHEAGDQNEAEKSHLR.E
NHERF_MOUSE	MK_SCX_50.4648.4648.3	3	4.651	0.409	1	1188.1	50	K.LGVSIREELLRPQEK.S
NHERF_MOUSE	MK_SCX_54.4436.4436.3	3	3.056	0.384	1	1334.4	41.25	K.GPNGYGFNLHSDKSKPGQFIR.A
NHERF_MOUSE	MK_SCX_55.4828.4828.2	2	3.645	0.412	1	889.3	58.333332	R.LCCLEKGPNGYGFHLHGEK.G
NHERF_MOUSE	MK_SCX_55.4852.4852.3	3	4.356	0.483	1	1296	43.055553	R.LCCLEKGPNGYGFHLHGEK.G
NHERF_MOUSE	MK_SCX_60.5091.5091.2	2	2.294	0.301	1	434	60.000004	R.LLVVDPETDER.L
NHRF2_MOUSE	MK_SCX_25.4947.4947.3	3	5.46	0.459	1	2427.4	52.941177	R.DPFQESGLHLSPTAAEAK.E
NHRF2_MOUSE	MK_SCX_25.4984.4984.2	2	4.966	0.513	1	1036.9	70.588234	R.DPFQESGLHLSPTAAEAK.E
NHRF2_MOUSE	MK_SCX_39.3461.3461.2	2	3.971	0.533	1	1343	80.769226	R.RVEPGSPAEAAALR.A
NHRF2_MOUSE	MK_SCX_40.3332.3332.3	3	4.132	0.276	1	1317.9	46.153847	R.RVEPGSPAEAAALR.A
NHRF2_MOUSE	MK_SCX_45.4433.4433.2	2	3.687	0.235	1	689.5	69.230774	R.LLVVDPETDEHFKR.L
NHRF2_MOUSE	MK_SCX_46.4659.4659.3	3	5.337	0.528	1	1300.4	41.666664	K.RDPFQESGLHLSPTAAEAK.E
NID1_MOUSE	MK_SCX_15.3583.3583.2	2	2.783	0.31	1	489.1	58.333332	R.ILSPGYEATERPR.G
NID1_MOUSE	MK_SCX_18.6359.6359.2	2	4.01	0.616	1	1645.4	76.666664	K.VLEGLQYPFAVTSYGK.N
NID1_MOUSE	MK_SCX_2201.15736.15736.2	2	2.817	0.381	1	373.1	60.000004	R.VLFDTGLVNPR.G
NID1_MOUSE	MK_SCX_2201.5063.5063.2	2	2.768	0.407	1	1183.4	80	R.TIFWTDSQLDR.I
NID1_MOUSE	MK_SCX_25.6516.6516.2	2	3.973	0.419	1	744.4	57.894737	K.SNGAYNIFANDRESIENLAK.S
NID1_MOUSE	MK_SCX_26.5154.5154.2	2	2.069	0.242	1	526.3	72.22222	R.GNLYWTDWNR.D
NID1_MOUSE	MK_SCX_28.5233.5233.2	2	3.38	0.413	1	1226.2	80	R.QDLGSPEGIALDHLGR.T
NID1_MOUSE	MK_SCX_32.6176.6176.2	2	5.907	0.491	1	2634.1	81.25	R.KVLEGLQYPFAVTSYGK.N
NID1_MOUSE	MK_SCX_33.3879.3879.2	2	5.289	0.571	1	1925.6	92.30769	K.KDESQVPAVVGFSK.G
NID1_MOUSE	MK_SCX_41.4847.4847.2	2	4.316	0.496	1	916.2	81.818184	R.RVLFDTGLVNPR.G
NID2_MOUSE	MK_SCX_15.7404.7404.2	2	2.083	0.297	1	895.3	82.14286	K.DIGLPNGLTFDPFSK.L
NID2_MOUSE	MK_SCX_18.3913.3913.2	2	4.945	0.643	1	1325.9	75	K.ADPGLVDVGTSSPGSDR.V
NID2_MOUSE	MK_SCX_20_1.6789.6789.3	3	5.589	0.621	1	977	30.303032	R.FAVTNQIGPVEVDSAPVGVNPCYDGSHTCDTTAR.C
NID2_MOUSE	MK_SCX_21.8898.8898.3	3	3.989	0.536	1	515.2	29.464287	R.ASLEAGAEPETIITSGLISPEGLAIDHFR.R
NID2_MOUSE	MK_SCX_2201.6481.6481.3	3	3.713	0.353	1	457.7	34.523808	R.DGVISVNKDSGQFTDEFLPEQR.S
NID2_MOUSE	MK_SCX_28.4520.4520.3	3	3.187	0.25	1	698.2	34.72222	R.FALDNVRPATVGGDPSTAR.S
NID2_MOUSE	MK_SCX_30.6974.6974.3	3	3.457	0.389	1	896.8	39.705883	R.AITVDPIRGNLYWTDWNR.E
NID2_MOUSE	MK_SCX_36.4289.4289.2	2	3.38	0.256	1	933.8	84.61539	R.HLAIPATQQLTVDR.A
NID2_MOUSE	MK_SCX_42.5621.5621.3	3	5.159	0.579	1	1745.3	42.045452	R.RDGVISVNKDSGQFTDEFLPEQR.S
NID2_MOUSE	MK_SCX_52.4557.4557.3	3	3.834	0.443	1	1612.3	50	R.RTMYWTDSGLDKIER.A

NID2_MOUSE	MK_SCX_53.4016.4016.3	3	3.113	0.435	1	334.8	45.454548	R.KVLFHTDLVNPR.A
NIFUN_MOUSE	MK_SCX_13.4917.4917.2	2	3.189	0.526	1	474.7	50	K.NVGTGLVGAPACGDVMK.L
NIFUN_MOUSE	MK_SCX_17.6035.6035.2	2	2.464	0.316	1	404	37.5	K.LHCSMLAEDAIKAALADYKLK.Q
NIFUN_MOUSE	MK_SCX_50.7191.7191.3	3	4.303	0.312	1	1482.8	36	K.NTDIAKELCLPPVKLHCSMLAEDAIK.A
NIPM_MOUSE	MK_SCX_13.7659.7659.2	2	3.111	0.344	1	1025.8	77.27273	K.IEFDDFEECLLR.Y
NIPM_MOUSE	MK_SCX_40.4752.4752.2	2	3.957	0.548	1	1582.3	90.909096	R.HFM*FLSAEQPYK.N
NIPM_MOUSE	MK_SCX_40.5327.5327.2	2	4.527	0.55	1	1732.5	90.909096	R.HFMFLSAEQPYK.N
NIPM_MOUSE	MK_SCX_49.5113.5113.2	2	3.256	0.224	1	2078.8	76.666664	R.HFMFLSAEQPYKNAAR.C
NIPM_MOUSE	MK_SCX_52.4402.4402.3	3	4.587	0.526	1	1327.7	51.666664	R.HFMFLSAEQPYKNAAR.C
NIPS1_MOUSE	MK_SCX_37.6846.6846.2	2	4.432	0.538	1	1334.7	78.57143	K.RGWDENVYYTVPLVR.H
NIPS1_MOUSE	MK_SCX_39.5590.5590.3	3	3.372	0.338	1	537.4	42.857143	K.LKPGTM*IEWGNNWAR.A
NIPS1_MOUSE	MK_SCX_40.6363.6363.3	3	4.175	0.507	1	649.4	42.857143	K.LKPGTMIEWGNNWAR.A
NIPS1_MOUSE	MK_SCX_52.5616.5616.3	3	3.795	0.395	1	1906.1	48.52941	R.TYKLKPGTMIEWGNNWAR.A
NIPS1_MOUSE	MK_SCX_54.5497.5497.2	2	4.634	0.391	1	1160.2	70	R.KRGWDENVYYTVPLVR.H
NIPS1_MOUSE	MK_SCX_54.5512.5512.3	3	4.784	0.47	1	1235.7	45	R.KRGWDENVYYTVPLVR.H
NIPS2_MOUSE	MK_SCX_42.3903.3903.3	3	3.838	0.197	1	1411.2	60.000004	K.LKENQEFVNFR.K
NIPS2_MOUSE	MK_SCX_42.3908.3908.2	2	4.002	0.515	1	1213.3	85	K.LKENQEFVNFR.K
NIPS2_MOUSE	MK_SCX_52.6309.6309.3	3	3.388	0.26	1	364.5	27.173912	K.LQFHNVKPECLDAYNKICQEVLPK.I
NIT1_MOUSE	MK_SCX_31.5695.5695.2	2	5.874	0.506	1	1564.2	76.47059	R.ASYGHSMVVDPWGTVVAR.C
NIT1_MOUSE	MK_SCX_32.5021.5021.3	3	3.33	0.227	1	999.4	36.764706	R.ASYGHSM*VVDPWGTVVAR.C
NIT1_MOUSE	MK_SCX_32.5537.5537.3	3	4.264	0.267	1	1849.4	45.588234	R.ASYGHSMVVDPWGTVVAR.C
NIT1_MOUSE	MK_SCX_36.6198.6198.2	2	2.726	0.432	1	748.7	77.77778	R.IDLHFLQQMR.Q
NIT1_MOUSE	MK_SCX_36.6270.6270.3	3	3.617	0.363	1	562.9	55.555557	R.IDLHFLQQMR.Q
NIT1_MOUSE	MK_SCX_41.3778.3778.3	3	4.019	0.493	1	580.2	33.75	R.ESNYTKPGGTLEPPVKTPAGK.V
NK1R_MOUSE	MK_SCX_17.6384.6384.2	2	2.354	0.169	1	473.2	50	R.CCPFISAGDYEGLEM*K.S
NK1R_MOUSE	MK_SCX_19.6414.6414.2	2	2.723	0.209	1	305.3	46.666668	R.CCPFISAGDYEGLEMK.S
NLTP_MOUSE	MK_SCX_11.6821.6821.2	2	5.38	0.58	1	2193.2	66.66667	K.ADCTITM*ADSDLLALM*TGK.M
NLTP_MOUSE	MK_SCX_11.8899.8899.2	2	5.037	0.55	1	1424.1	63.88889	K.ADCTITM*ADSDLLALMTGK.M
NLTP_MOUSE	MK_SCX_11.9155.9155.2	2	4.854	0.668	1	1135.7	58.333332	K.ADCTITMADSDLLALMTGK.M
NLTP_MOUSE	MK_SCX_18.8808.8808.2	2	5.114	0.607	1	1975.2	71.05263	K.KADCTITMADSDLLALMTGK.M
NLTP_MOUSE	MK_SCX_21.3787.3787.2	2	3.563	0.337	1	1020.4	83.33333	K.LEEEGEQFVK.K
NLTP_MOUSE	MK_SCX_2201.3566.3566.2	2	3.045	0.449	1	1036.9	83.33333	K.LQNLQLQPGK.A
NLTP_MOUSE	MK_SCX_2201.4154.4154.2	2	2.625	0.326	1	595.6	81.25	K.EATWVVDVK.N
NLTP_MOUSE	MK_SCX_24.3859.3859.2	2	3.509	0.536	1	754	88.88889	K.IAGNMGLAMK.L
NLTP_MOUSE	MK_SCX_25.4319.4319.2	2	3.155	0.563	1	368.9	75	K.MNPQSAFFQGK.L
NLTP_MOUSE	MK_SCX_25.4943.4943.2	2	2.998	0.431	1	804	92.85714	K.IGGIFAFK.V
NLTP_MOUSE	MK_SCX_28.4313.4313.2	2	4.477	0.518	1	1063.5	78.57143	K.DGPGGKEATWVVDVK.N
NLTP_MOUSE	MK_SCX_28.4317.4317.3	3	3.921	0.383	1	1127.1	44.642857	K.DGPGGKEATWVVDVK.N
NLTP_MOUSE	MK_SCX_33.4764.4764.3	3	3.016	0.214	1	1662.5	61.363636	K.LKIAGNMGLAMK.L
NLTP_MOUSE	MK_SCX_37.3926.3926.2	2	3.464	0.376	1	1137.8	88.88889	K.ANLVFKEIEK.K
NLTP_MOUSE	MK_SCX_38.3474.3474.3	3	4.67	0.375	1	1392.1	62.5	K.KLEEEGEQFVK.K
NLTP_MOUSE	MK_SCX_39.3478.3478.2	2	4.684	0.325	1	1953.2	90	K.KLEEEGEQFVK.K
NLTP_MOUSE	MK_SCX_40.3717.3717.3	3	3.269	0.378	1	671	43.18182	K.LKIAGNM*GLAMK.L
NLTP_MOUSE	MK_SCX_50.3734.3734.2	2	5.01	0.496	1	1243.6	68.75	K.VKDGPGGKEATWVVDVK.N
NLTP_MOUSE	MK_SCX_50.3773.3773.3	3	5.197	0.571	1	590.4	45.3125	K.VKDGPGGKEATWVVDVK.N
NLTP_MOUSE	MK_SCX_52.3059.3059.3	3	4.686	0.318	1	988.3	59.090908	K.KLEEEGEQFVKK.I
NLTP_MOUSE	MK_SCX_52.3077.3077.2	2	2.289	0.186	1	345.6	54.545456	K.KLEEEGEQFVKK.I
NMI_MOUSE	MK_SCX_14.3405.3405.2	2	3.245	0.404	1	1642.2	83.33333	R.NGGGEVESVDYDR.K
NMI_MOUSE	MK_SCX_37.3426.3426.3	3	3.815	0.283	1	1931.9	58.333332	K.VSAHPVPLNTGVR.F
NMNA3_MOUSE	MK_SCX_24.4685.4685.3	3	6.007	0.53	1	2059.9	40.217392	R.SSAQMDGPDPSKTPSASAALPELK.L
NMNA3_MOUSE	MK_SCX_24.4729.4729.2	2	3.929	0.496	1	583.5	45.652176	R.SSAQMDGPDPSKTPSASAALPELK.L

NMNA3_MOUSE	MK_SCX_25.5012.5012.2	2	2.276	0.386	1	792.5	81.25	K.TFQTPNLWK.D
NMT1_MOUSE	MK_SCX_26.4328.4328.3	3	3.281	0.344	1	547.6	28.75	K.GSDMESTQDQPVKMTSLPAER.I
NMT1_MOUSE	MK_SCX_52.4325.4325.3	3	3.532	0.415	1	855.1	47.727272	K.KDIPVVHQLLSR.Y
NNTM_MOUSE	MK_SCX_18.7255.7255.2	2	5.716	0.444	1	2807.3	68.42105	R.VTIAQGYDALSSMANISGYK.A
NNTM_MOUSE	MK_SCX_21.5343.5343.2	2	3.452	0.4	1	975.2	83.33333	R.VALSPAGVQALVK.Q
NNTM_MOUSE	MK_SCX_23.4487.4487.2	2	2.378	0.214	1	354	60.000004	K.KTCDALQAKVR.E
NNTM_MOUSE	MK_SCX_23.7302.7302.3	3	3.459	0.33	1	647.4	27.884615	R.SLGVGYAAVDNPIFYKPNTAM*LLGDAK.K
NNTM_MOUSE	MK_SCX_23.7732.7732.3	3	3.522	0.341	1	443.2	27.884615	R.SLGVGYAAVDNPIFYKPNTAMLLGDAK.K
NNTM_MOUSE	MK_SCX_23.7815.7815.2	2	3.456	0.585	1	566.9	34.615387	R.SLGVGYAAVDNPIFYKPNTAMLLGDAK.K
NNTM_MOUSE	MK_SCX_28.5779.5779.2	2	3.469	0.576	1	1890.7	87.5	K.DDFDFGTMSHVIR.G
NNTM_MOUSE	MK_SCX_30.6462.6462.3	3	4.026	0.31	1	1100.1	38.157894	R.AAGAQIQGMKEVLASDLVVK.V
NNTM_MOUSE	MK_SCX_35.4200.4200.2	2	4.482	0.548	1	1414.2	83.33333	K.GITHIGYTDLPSR.M
NNTM_MOUSE	MK_SCX_38.3837.3837.3	3	3.289	0.197	1	1887.2	59.090908	R.KTTVLAMDQVPR.V
NNTM_MOUSE	MK_SCX_41.4541.4541.2	2	2.57	0.183	1	644.8	68.181816	K.EMSKEFIEAEMK.L
NOL3_MOUSE	MK_SCX_17.6226.6226.2	2	6.01	0.591	1	1418.4	66.66667	R.GVLTGPEYEALDALPDAER.R
NOLA2_MOUSE	MK_SCX_13.2682.2682.2	2	4.022	0.448	1	1043.2	71.875	K.AAPEESEAQAEGCSEER.T
NOLA2_MOUSE	MK_SCX_29.7081.7081.3	3	4.064	0.308	1	922.7	40.27778	R.TYKELLVNLNPIAQPLASR.R
NONO_MOUSE	MK_SCX_21.4738.4738.2	2	3.43	0.63	1	853.4	76.92308	R.FAQPGSFEYEYAM*R.W
NONO_MOUSE	MK_SCX_21.5284.5284.2	2	3.454	0.528	1	667.7	69.230774	R.FAQPGSFEYEYAMR.W
NONO_MOUSE	MK_SCX_2201.3619.3619.2	2	2.136	0.219	1	641.1	87.5	K.LEMEMEAAR.H
NONO_MOUSE	MK_SCX_23.4200.4200.2	2	2.003	0.233	1	423.2	64.28571	R.MGQMAMGGAMGINNR.G
NOP14_MOUSE	MK_SCX_32.6107.6107.2	2	2.685	0.278	1	793.1	57.14286	K.GRAAFPGLDVLIYLK.I
NOP14_MOUSE	MK_SCX_4.6866.6866.2	2	2.656	0.15	1	407.5	45.833336	R.QAQREDALELTEK.L
NOP56_MOUSE	MK_SCX_17.3324.3324.2	2	4.93	0.559	1	857.2	65.625	K.EEVASEPEEAASPTTPK.K
NOP56_MOUSE	MK_SCX_19.7871.7871.2	2	3.116	0.279	1	399.5	58.333332	K.MSQVAPSLSALIGEAVGAR.L
NOP56_MOUSE	MK_SCX_28.3480.3480.3	3	4.332	0.426	1	1117.9	35	K.SSPKEEVASEPEEAASPTTPK.K
NOP56_MOUSE	MK_SCX_44.8516.8516.3	3	3.427	0.345	1	672.9	33.333336	R.KNLDVMKEAVVQAEEAAAEITR.K
NOP56_MOUSE	MK_SCX_48.4337.4337.3	3	3.738	0.28	1	804.3	39.705883	K.KLKPQENGMEDPPVSLPK.S
NP1L1_MOUSE	MK_SCX_15.7741.7741.2	2	3.519	0.413	1	530	36.53846	R.SEPDDSDPFSFDGPEIMGCTGCQIDWK.K
NP1L1_MOUSE	MK_SCX_17.6311.6311.2	2	4.412	0.543	1	998	65.625	R.LDGLVDTPTGYIESLPK.V
NP1L1_MOUSE	MK_SCX_18.7148.7148.2	2	6.168	0.636	1	1616.1	66.66667	R.QLTVQMMQNPQILAALQER.L
NP1L1_MOUSE	MK_SCX_2201.9111.9111.2	2	3.472	0.464	1	343.7	65	K.GIPEFWLTVFK.N
NP1L1_MOUSE	MK_SCX_24.6766.6766.2	2	5.806	0.584	1	2249.2	73.52941	K.NVDLLSDMVQEHDEPILK.H
NP1L1_MOUSE	MK_SCX_34.5397.5397.2	2	3.951	0.491	1	1281.2	81.818184	K.YAVLYQPLFDKR.F
NP1L1_MOUSE	MK_SCX_35.3825.3825.2	2	3.253	0.449	1	1081.4	77.77778	K.FYEEVHDLER.K
NP1L4_MOUSE	MK_SCX_17.8329.8329.2	2	6.166	0.516	1	1928.9	73.52941	R.NVDMLSELVQEYDEPILK.H
NP1L4_MOUSE	MK_SCX_18.5163.5163.2	2	6.005	0.552	1	1925.8	70.588234	K.LTDQVM*QNPQVLAALQER.L
NP1L4_MOUSE	MK_SCX_18.5913.5913.2	2	5.901	0.517	1	1947.1	67.64706	K.LTDQVMQNPQVLAALQER.L
NP1L4_MOUSE	MK_SCX_2201.9779.9779.2	2	2.472	0.456	1	414.4	75	K.GIPEFWFTIFR.N
NP1L4_MOUSE	MK_SCX_28.4753.4753.2	2	3.677	0.479	1	451.6	43.75	R.LDNVSHTPSSYIETLPK.A
NP1L4_MOUSE	MK_SCX_28.4805.4805.3	3	4.347	0.426	1	1140.9	48.4375	R.LDNVSHTPSSYIETLPK.A
NP1L4_MOUSE	MK_SCX_38.5254.5254.2	2	2.781	0.492	1	613.3	72.72727	R.KYAALYQPLFDK.R
NPC2_MOUSE	MK_SCX_27.5790.5790.3	3	3.445	0.435	1	314.8	30.000002	R.VPFPIPEPDGCKSGINCPIQK.D
NPC2_MOUSE	MK_SCX_44.5391.5391.3	3	4.843	0.553	1	1215.7	45.588234	K.VYSYLNKLPVKNEYPSIK.L
NPHN_MOUSE	MK_SCX_19.3049.3049.2	2	3.534	0.592	1	497	60.000004	R.TVEDVSSSVNEGSEEK.L
NPHN_MOUSE	MK_SCX_19.8071.8071.2	2	2.057	0.133	1	417.6	39.473686	R.LDDVAAKPQSAPFKGSAASR.S
NPHP1_MOUSE	MK_SCX_50.4139.4139.2	2	3.213	0.121	1	414.6	50	-.MLARRPRDPLQALR.R
NPM_MOUSE	MK_SCX_17.7671.7671.2	2	5.573	0.668	1	1787.6	70	K.M*SVQPTVSLGGFEITPPVVLR.L
NPM_MOUSE	MK_SCX_17.8047.8047.2	2	3.262	0.339	1	995.7	52.499996	K.MSVQPTVSLGGFEITPPVVLR.L
NPM_MOUSE	MK_SCX_21.7955.7955.2	2	3.517	0.293	1	1466.4	65.38461	R.MTDQEAIQDLWQWR.K
NPM_MOUSE	MK_SCX_2201.2439.2439.2	2	2.691	0.296	1	520	81.25	K.GPSSVEDIK.A

NQO1_MOUSE	MK_SCX_2201.4443.4443.2	2	3.295	0.366	1	751.3	75	K.NFQYPSESSLAYK.E
NRP1_MOUSE	MK_SCX_25.4419.4419.3	3	4.7	0.511	1	1832.4	38.636364	K.TGPIQDHTGDGNFIYSQADENQK.G
NSBP1_MOUSE	MK_SCX_16.9940.9940.2	2	2.3	0.228	1	334.4	35	K.EDRKEEGEQEVAVDEGSDENK.V
NSBP1_MOUSE	MK_SCX_28.4879.4879.3	3	3.816	0.489	1	1276.2	46.42857	R.LSAM*PVPFTPELKPK.R
NSBP1_MOUSE	MK_SCX_28.5663.5663.2	2	3.995	0.412	1	724.5	67.85714	R.LSAMPVPFTPELKPK.R
NSF_MOUSE	MK_SCX_23.5371.5371.2	2	2.533	0.232	1	849.8	77.27273	K.NFSGAELEGLVR.A
NSF_MOUSE	MK_SCX_38.7380.7380.2	2	2.261	0.171	1	349	54.545456	K.KNIDSNPYDTDK.M
NSF1C_MOUSE	MK_SCX_17.4360.4360.2	2	5.128	0.616	1	978.4	52.272724	K.LGSTAPQVLNTSSPAQQAENEAK.A
NSF1C_MOUSE	MK_SCX_17.4392.4392.3	3	5.189	0.651	1	2160.9	44.31818	K.LGSTAPQVLNTSSPAQQAENEAK.A
NSF1C_MOUSE	MK_SCX_18.5336.5336.2	2	5.611	0.511	1	1374.1	69.44444	K.ASSSILINEAEPTTNIQIR.L
NSF1C_MOUSE	MK_SCX_19.3829.3829.2	2	4.537	0.519	1	1563.1	83.33333	R.LGAAPEEESAYVAGER.R
NSF1C_MOUSE	MK_SCX_20_1.5553.5553.2	2	5.657	0.466	1	1724.4	82.14286	R.SYQDPSNAQFLESIR.R
NSF1C_MOUSE	MK_SCX_21.6567.6567.2	2	3.266	0.332	1	724	75	K.SPNELVDDLFK.G
NSF1C_MOUSE	MK_SCX_23.3546.3546.3	3	5.318	0.475	1	1314.3	42.1875	R.DLIHDQDEEEEEEEGQR.F
NSF1C_MOUSE	MK_SCX_24.3633.3633.2	2	5.43	0.591	1	2216.6	75	R.DLIHDQDEEEEEEEGQR.F
NSF1C_MOUSE	MK_SCX_24.4828.4828.3	3	6.578	0.533	1	2313.9	36.666668	K.AFTGEGQKLGSTAPQVLNTSSPAQQAENEAK.A
NSF1C_MOUSE	MK_SCX_53.5140.5140.2	2	3.572	0.437	1	1495.4	79.16667	R.KKSPNELVDDLFK.G
NT5C_MOUSE	MK_SCX_15.9227.9227.2	2	5.518	0.484	1	1378	52.083332	K.VASVYESPGFFLNLEPIPGALDALR.E
NT5C_MOUSE	MK_SCX_16.10582.10582.2	2	4.962	0.592	1	2212.1	70	R.VLVDMDGVLADFESGLLQGFR.R
NT5C_MOUSE	MK_SCX_16.9149.9149.2	2	2.738	0.177	1	486.9	42.5	R.VLVDM*DGVLADFESGLLQGFR.R
NT5C_MOUSE	MK_SCX_29.5522.5522.3	3	3.852	0.468	1	512.5	35.294117	R.GFLANEQYGALRPDLAEK.V
NT5C_MOUSE	MK_SCX_29.5544.5544.2	2	4.778	0.438	1	1392.4	58.823532	R.GFLANEQYGALRPDLAEK.V
NT5C_MOUSE	MK_SCX_53.4176.4176.3	3	3.572	0.398	1	843.4	47.916664	R.RFPEEPHVPLEQR.R
NUAM_MOUSE	MK_SCX_16.7646.7646.1	1	2.569	0.372	1	352.1	50	R.DDGAAILAAVSNMVQK.I
NUAM_MOUSE	MK_SCX_17.5987.5987.2	2	4.519	0.558	1	1508	64.70589	R.YDDIEETNYFQQASELAK.L
NUAM_MOUSE	MK_SCX_17.6665.6665.2	2	4.317	0.395	1	1157.3	66.66667	K.LVNQEVLADPLVPPQLTIK.D
NUAM_MOUSE	MK_SCX_17.7367.7367.2	2	4.964	0.627	1	1200.4	69.44444	R.ALSEIAGITLPYDTLDQVR.N
NUAM_MOUSE	MK_SCX_19.4580.4580.2	2	4.402	0.59	1	1793	80	R.FASEIAGVDDLGTTGR.G
NUAM_MOUSE	MK_SCX_20_1.9235.9235.3	3	4.392	0.504	1	494.4	27.67857	K.LVNQEVLADPLVPPQLTIKDFYM*TDSISR.A
NUAM_MOUSE	MK_SCX_2201.3336.3336.2	2	3.173	0.144	1	1209.7	87.5	K.ILQDIASGR.H
NUAM_MOUSE	MK_SCX_23.5663.5663.3	3	3.476	0.327	1	561.3	25.925926	R.FASEIAGVDDLGTTGRGNDM*QVGTYIEK.M
NUAM_MOUSE	MK_SCX_23.6260.6260.3	3	4.075	0.299	1	684.9	30.555555	R.FASEIAGVDDLGTTGRGNDMQVGTYIEK.M
NUAM_MOUSE	MK_SCX_34.4212.4212.2	2	4.305	0.609	1	1152	79.16667	R.MHEDINEEWISDK.T
NUAM_MOUSE	MK_SCX_41.3908.3908.2	2	4.292	0.386	1	1556.7	81.818184	R.NRLEEVSPNLVR.Y
NUAM_MOUSE	MK_SCX_42.3872.3872.3	3	3.217	0.157	1	733.2	43.18182	R.NRLEEVSPNLVR.Y
NUAM_MOUSE	MK_SCX_48.5483.5483.3	3	4.02	0.228	1	1194.8	36.11111	R.LSVAGNCRMCLVEIEKAPK.V
NUBM_MOUSE	MK_SCX_18.5274.5274.2	2	2.758	0.272	1	627.7	53.571426	-.M*LAARHFLGGLVPVR.V
NUBM_MOUSE	MK_SCX_23.5574.5574.2	2	2.628	0.408	1	974.6	72.22222	K.GPDWILGEMK.T
NUBM_MOUSE	MK_SCX_27.5408.5408.2	2	3.127	0.503	1	731.7	83.33333	R.GGTWFAGFGR.E
NUBM_MOUSE	MK_SCX_30.5606.5606.3	3	4.913	0.466	1	1130.7	46.666668	K.GDARPAEIDSLWEISK.Q
NUBM_MOUSE	MK_SCX_44.5740.5740.3	3	3.865	0.511	1	1391.2	50	R.FVKGDARPAEIDSLWEISK.Q
NUBM_MOUSE	MK_SCX_58.6942.6942.2	2	2.172	0.154	1	455.3	62.5	R.HFRPELEDR.M
NUBM_MOUSE	MK_SCX_58.7216.7216.3	3	3.122	0.264	1	762.8	59.375	R.HFRPELEDR.M
NUCB1_MOUSE	MK_SCX_17.3049.3049.2	2	2.891	0.395	1	676.8	63.333332	R.ADTDDAPVPAPAGDQK.D
NUCB1_MOUSE	MK_SCX_21.3893.3893.2	2	3.398	0.36	1	825.4	68.181816	R.ELQQAVLQM*EQR.K
NUCB1_MOUSE	MK_SCX_21.4539.4539.2	2	4.567	0.322	1	1682.6	86.36364	R.ELQQAVLQMEQR.K
NUCB1_MOUSE	MK_SCX_21.4557.4557.3	3	3.627	0.247	1	939.5	47.727272	R.ELQQAVLQMEQR.K
NUCB1_MOUSE	MK_SCX_2201.2847.2847.2	2	3.186	0.364	1	955.1	88.88889	R.LSQETEALGR.S
NUCB1_MOUSE	MK_SCX_2201.3150.3150.2	2	3.058	0.141	1	791.2	92.85714	R.FEEELAAR.E
NUCB1_MOUSE	MK_SCX_2201.3371.3371.2	2	2.687	0.445	1	338.7	60.000004	K.VNVPGSQAQLK.E
NUCB1_MOUSE	MK_SCX_2201.3491.3491.2	2	3.436	0.404	1	917.7	83.33333	R.YLESLGEEQR.K

NUCB1_MOUSE	MK_SCX_24.3685.3685.3	3	3.476	0.507	1	466	34.090908	R.ADTDDAPVPAPAGDQKDVPASEK.K
NUCB1_MOUSE	MK_SCX_30.3988.3988.2	2	3.781	0.494	1	1017	75	K.TVEMSPAYTEEELKR.F
NUCB1_MOUSE	MK_SCX_30.7575.7575.3	3	3.167	0.278	1	776.1	48.333332	R.YLQEVINVLETDGHFR.E
NUCB1_MOUSE	MK_SCX_31.5142.5142.2	2	3.315	0.451	1	1005.8	76.92308	K.QEPNLQVDHMNLLK.Q
NUCB1_MOUSE	MK_SCX_34.4423.4423.3	3	3.566	0.458	1	512.5	45.833336	K.LSQELDFVSHNVR.T
NUCB1_MOUSE	MK_SCX_34.4479.4479.2	2	4.02	0.514	1	1534.8	79.16667	K.LSQELDFVSHNVR.T
NUCB1_MOUSE	MK_SCX_39.6456.6456.2	2	2.587	0.16	1	598.1	70	R.QEVSRLRM*LLK.A
NUCB1_MOUSE	MK_SCX_43.7355.7355.3	3	3.181	0.375	1	1284.7	41.17647	R.YLQEVINVLETDGHFREK.L
NUCB1_MOUSE	MK_SCX_46.3728.3728.3	3	4.855	0.511	1	940.3	38.75	R.KQQLQEQSAPPSKPDGQLQFR.A
NUCB1_MOUSE	MK_SCX_51.4858.4858.3	3	4.238	0.494	1	2083.6	53.333336	K.SGKLSQELDFVSHNVR.T
NUCB2_MOUSE	MK_SCX_26.8236.8236.3	3	4.519	0.469	1	1787.8	40	K.LHDVNNDGFLDEQELEALFTR.E
NUCG_MOUSE	MK_SCX_18.5737.5737.2	2	2.662	0.122	1	547.2	46.875	R.SYVMPNAPVDETIPLER.F
NUCG_MOUSE	MK_SCX_20_1.8213.8213.2	2	2.766	0.244	1	496.3	54.166668	R.ASGLLFVPNILAR.A
NUCG_MOUSE	MK_SCX_23.7910.7910.3	3	5.144	0.506	1	731.5	31.48148	R.AMDDTFYLSNVAPQVPHLNQNAWNNLER.Y
NUCG_MOUSE	MK_SCX_24.4692.4692.2	2	2.896	0.412	1	971.4	94.44444	K.YGLPGVAQLR.S
NUCL_MOUSE	MK_SCX_17.7153.7153.2	2	4.36	0.507	1	1259.3	60.000004	K.VEGSEPTTPFNLFIGNLNPNK.S
NUCL_MOUSE	MK_SCX_18.5851.5851.2	2	5.053	0.601	1	1958	88.46153	K.FGYVDFESAEDLEK.A
NUCL_MOUSE	MK_SCX_20_1.6003.6003.2	2	4.42	0.555	1	1501.1	80.769226	K.GFGFVDFNSEEDAK.A
NUCL_MOUSE	MK_SCX_21.4875.4875.2	2	4.271	0.479	1	1789.9	83.33333	K.TLVLSNLSYSATK.E
NUCL_MOUSE	MK_SCX_23.6114.6114.2	2	3.218	0.262	1	1164.4	93.75	K.FAISELFAK.N
NUCL_MOUSE	MK_SCX_23.8050.8050.3	3	3.047	0.547	1	520.7	33.653847	K.VEGSEPTTPFNLFIGNLNPNKSVNELK.F
NUCL_MOUSE	MK_SCX_31.6839.6839.2	2	3.468	0.291	1	897.5	60.714287	K.ALELTGLKVFGNEIK.L
NUCL_MOUSE	MK_SCX_33.5768.5768.2	2	4.162	0.452	1	1585.7	82.14286	R.KFGYVDFESAEDLEK.A
NUCL_MOUSE	MK_SCX_33.5839.5839.3	3	3.537	0.266	1	950.7	41.07143	R.KFGYVDFESAEDLEK.A
NUCL_MOUSE	MK_SCX_36.5344.5344.3	3	4.495	0.502	1	1324.7	40	K.EAMEDGEIDGNKVTLDWAKPK.G
NUCL_MOUSE	MK_SCX_40.3511.3511.3	3	4.27	0.402	1	1139.8	43.421055	K.SEADAEKNLEEKQGAEIDGR.S
NUCL_MOUSE	MK_SCX_41.3493.3493.2	2	2.431	0.273	1	754	75	K.NLEEKQGAEIDGR.S
NUCL_MOUSE	MK_SCX_46.7078.7078.3	3	4.854	0.585	1	1635.9	41.304348	K.KQKVEGSEPTTPFNLFIGNLNPNK.S
NUCL_MOUSE	MK_SCX_50.4928.4928.3	3	5.367	0.496	1	2192.8	41.304348	K.AAKEAMEDGEIDGNKVTLDWAKPK.G
NUCL_MOUSE	MK_SCX_57.3427.3427.3	3	4.039	0.471	1	432.6	42.857143	K.ATFIKVPQNPHGKPK.G
NUCM_MOUSE	MK_SCX_17.5375.5375.2	2	3.752	0.361	1	332.5	45	K.LYTEGYQVPPGATYTAIEAPK.G
NUCM_MOUSE	MK_SCX_17.6425.6425.2	2	4.358	0.594	1	1083.3	61.11111	K.TQPYDVYDQVEFDVPIGSR.G
NUCM_MOUSE	MK_SCX_20_1.3853.3853.2	2	3.143	0.394	1	529.5	68.181816	R.IDEVEEM*LTNNR.I
NUCM_MOUSE	MK_SCX_45.5313.5313.3	3	3.05	0.242	1	528.6	32.5	K.AVTNMTLNFGPQHPAAHGVLR.L
NUD12_MOUSE	MK_SCX_23.8551.8551.3	3	4.718	0.508	1	1771.1	35.185184	K.LAGILSHSPSLLNETSENGWTALMYAAR.N
NUD12_MOUSE	MK_SCX_49.3148.3148.3	3	3.089	0.473	1	350.8	32.352943	K.KEM*ISELHSSAAEGNVAK.L
NUD19_MOUSE	MK_SCX_15.5970.5970.2	2	6.597	0.66	1	1595.7	68.181816	R.DAAPASQEPSQALSPPAGLAEWR.S
NUD19_MOUSE	MK_SCX_15.6010.6010.3	3	4.302	0.537	1	3083.2	47.727272	R.DAAPASQEPSQALSPPAGLAEWR.S
NUD19_MOUSE	MK_SCX_26.7313.7313.2	2	5.271	0.637	1	1373.3	56.521736	R.FLPGAHVFPGGVLDAADSSPDWVR.L
NUD19_MOUSE	MK_SCX_26.7455.7455.3	3	5.856	0.471	1	1581.9	40.217392	R.FLPGAHVFPGGVLDAADSSPDWVR.L
NUD19_MOUSE	MK_SCX_28.7400.7400.3	3	5.623	0.564	1	1534.4	29.6875	R.FGLGPEPPRQPPFPGLSHGDADPAALPDDVALR.I
NUD19_MOUSE	MK_SCX_42.5728.5728.2	2	5.147	0.459	1	668.3	79.16667	R.RLENFASLSALYR.F
NUD19_MOUSE	MK_SCX_50.6895.6895.3	3	5.715	0.553	1	988.2	36.458336	R.VVIHSPYVYEIYMTLPSENKHVYPR.N
NUDC_MOUSE	MK_SCX_32.4101.4101.2	2	3.676	0.412	1	982.1	81.818184	K.FMDQHPEMDFSK.A
NUDC_MOUSE	MK_SCX_51.3893.3893.3	3	3.304	0.371	1	604.7	41.666664	K.KFMDQHPEMDFSK.A
NUDC2_MOUSE	MK_SCX_30.5235.5235.3	3	6.219	0.56	1	2501.6	45	R.FQKENPGFDFSGAEISGNYTK.G
NUDT5_MOUSE	MK_SCX_20_1.5525.5525.2	2	3.333	0.283	1	891.8	66.66667	K.SADAVSVIPVLQR.T
NUDT8_MOUSE	MK_SCX_20_1.3835.3835.2	2	2.885	0.278	1	825.2	70.83333	-.M*LPDGLSAEDEQR.C
NUDT8_MOUSE	MK_SCX_20_1.4036.4036.2	2	2.419	0.14	1	483.4	62.5	-.MLPDGLSAEDEQR.C
NUDT9_MOUSE	MK_SCX_25.6448.6448.2	2	2.081	0.155	1	388.7	46.153847	K.LHALFSQEHLVIYK.G
NUDT9_MOUSE	MK_SCX_39.4032.4032.3	3	3.077	0.316	1	1066.1	45.833336	R.WGPNHAADPIITR.W

NUDT9_MOUSE	MK_SCX_39.4053.4053.2	2	3.17	0.423	1	754.4	66.66667	R.WGPNHAADPIITR.W
NUGM_MOUSE	MK_SCX_18.6394.6394.2	2	3.767	0.577	1	789.4	90	K.DFPLTGYVELR.Y
NUGM_MOUSE	MK_SCX_19.4835.4835.2	2	4.178	0.517	1	1608.9	83.33333	K.SLADLTAVDVPTR.Q
NUGM_MOUSE	MK_SCX_19.8030.8030.2	2	5.336	0.529	1	2827.8	92.85714	K.QLSAFGEYVAEILPK.Y
NUGM_MOUSE	MK_SCX_20_1.11577.11577.3	3	4.286	0.23	1	559.6	24.13793	K.YVQQVQVSCLDELEICIHPDGVIPTLTFLR.D
NUGM_MOUSE	MK_SCX_20_1.6175.6175.3	3	3.914	0.392	1	1444	54.166668	R.VVAEPVELAQEFR.K
NUGM_MOUSE	MK_SCX_20_1.6232.6232.2	2	4.753	0.479	1	1072.2	83.33333	R.VVAEPVELAQEFR.K
NUGM_MOUSE	MK_SCX_20_1.8141.8141.2	2	3.07	0.469	1	975.5	69.230774	K.FDLNSPWEAFPAYR.Q
NUGM_MOUSE	MK_SCX_26.7193.7193.3	3	4.918	0.54	1	875	38.75	K.FDLNSPWEAFPAYRQPPESLK.L
NUGM_MOUSE	MK_SCX_26.7229.7229.2	2	4.057	0.589	1	997.4	52.499996	K.FDLNSPWEAFPAYRQPPESLK.L
NUGM_MOUSE	MK_SCX_31.5286.5286.2	2	3.303	0.559	1	796.1	69.230774	R.VVAEPVELAQEFRK.F
NUGM_MOUSE	MK_SCX_34.5159.5159.2	2	4.175	0.601	1	2101.2	87.5	R.ILTDYGFEGHPFR.K
NUGM_MOUSE	MK_SCX_35.10504.10504.3	3	3.098	0.316	1	1004.7	48.214287	R.KFDLNSPWEAFPAYR.Q
NUGM_MOUSE	MK_SCX_35.10520.10520.2	2	5.357	0.425	1	2163.6	85.71429	R.KFDLNSPWEAFPAYR.Q
NUGM_MOUSE	MK_SCX_36.5452.5452.2	2	4.34	0.61	1	1896.9	84.61539	K.RVVAEPVELAQEFR.K
NUGM_MOUSE	MK_SCX_36.5774.5774.3	3	4.093	0.332	1	1484.5	54.545456	R.KDFPLTGYVELR.Y
NUGM_MOUSE	MK_SCX_36.5785.5785.2	2	4.274	0.48	1	1427.3	86.36364	R.KDFPLTGYVELR.Y
NUGM_MOUSE	MK_SCX_37.6080.6080.3	3	3.98	0.32	1	1603.2	53.846157	K.RVVAEPVELAQEFR.K
NUGM_MOUSE	MK_SCX_40.11814.11814.3	3	4.569	0.504	1	1948.3	45	R.SDVTHKQLSAFGEYVAEILPK.Y
NUGM_MOUSE	MK_SCX_42.6956.6956.3	3	6.139	0.521	1	2393.6	47.61905	R.KFDLNSPWEAFPAYRQPPESLK.L
NUGM_MOUSE	MK_SCX_51.4491.4491.2	2	3.55	0.421	1	708.3	65.38461	R.ILTDYGFEGHPFRK.D
NUGM_MOUSE	MK_SCX_51.4788.4788.3	3	4.321	0.517	1	1670	51.785713	K.RVVAEPVELAQEFRK.F
NUGM_MOUSE	MK_SCX_53.5072.5072.3	3	3.112	0.335	1	871.3	44.230766	R.RILTDYGFEGHPFR.K
NUGM_MOUSE	MK_SCX_58.4278.4278.3	3	3.038	0.349	1	402.5	40.384613	R.ESAAADKRPTVRPR.S
NUHM_MOUSE	MK_SCX_15.5719.5719.2	2	5.429	0.658	1	1244.8	63.88889	R.DTPENNPDTPFDFTPENYK.R
NUHM_MOUSE	MK_SCX_18.4950.4950.2	2	2.64	0.281	1	1434.4	85	R.DSDSILETLQR.K
NUHM_MOUSE	MK_SCX_20_1.5853.5853.2	2	2.369	0.157	1	312.5	59.090908	R.QNGWLPISAMNK.V
NUHM_MOUSE	MK_SCX_21.4715.4715.2	2	2.631	0.247	1	711.5	75	K.VAEVLQVPPMR.V
NUHM_MOUSE	MK_SCX_21.6776.6776.2	2	3.656	0.469	1	1425.3	83.33333	R.VYEVATFYTMYNR.K
NUHM_MOUSE	MK_SCX_24.4910.4910.2	2	4.899	0.636	1	868.9	52.63158	R.DTPENNPDTPFDFTPENYKR.I
NUHM_MOUSE	MK_SCX_28.6209.6209.2	2	6.816	0.585	1	3145.3	81.57895	K.NYPEGHQAAAVLPVLDLAQR.Q
NUHM_MOUSE	MK_SCX_28.6333.6333.3	3	4.179	0.161	1	1350.2	47.368423	K.NYPEGHQAAAVLPVLDLAQR.Q
NUHM_MOUSE	MK_SCX_29.4625.4625.2	2	3.447	0.368	1	932.8	77.27273	R.DSDSILETLQRK.L
NUHM_MOUSE	MK_SCX_49.7671.7671.3	3	5.175	0.564	1	1479.2	44.736843	K.DIEEIIDELKAGKVPKPGPR.S
NUIM_MOUSE	MK_SCX_18.5743.5743.2	2	4.878	0.304	1	1670.7	64.70589	K.LCEAICPAQAITIEAEPR.A
NUIM_MOUSE	MK_SCX_23.6578.6578.2	2	3.425	0.212	1	1206.4	93.75	R.ILM*WTELIR.G
NUIM_MOUSE	MK_SCX_23.6786.6786.2	2	3.532	0.477	1	1039.2	80	R.GLGM*TLSYLFR.E
NUIM_MOUSE	MK_SCX_23.7147.7147.2	2	3.308	0.308	1	979.9	93.75	R.ILMWTELIR.G
NUIM_MOUSE	MK_SCX_23.7379.7379.2	2	3.617	0.485	1	1345.3	80	R.GLGMTLSYLFR.E
NUIM_MOUSE	MK_SCX_26.8512.8512.3	3	5.455	0.516	1	2193.5	41.666664	R.GLGM*TLSYLFREPATINYPFEK.G
NUIM_MOUSE	MK_SCX_26.8545.8545.2	2	4.049	0.483	1	339.1	45.238094	R.GLGM*TLSYLFREPATINYPFEK.G
NUIM_MOUSE	MK_SCX_26.8936.8936.3	3	6	0.578	1	3129.3	46.42857	R.GLGMTLSYLFREPATINYPFEK.G
NUIM_MOUSE	MK_SCX_30.5218.5218.3	3	3.036	0.439	1	340.6	32.8125	R.EPATINYPFEKGPLSPR.F
NUIM_MOUSE	MK_SCX_30.5238.5238.2	2	4.375	0.559	1	944.9	62.5	R.EPATINYPFEKGPLSPR.F
NUIM_MOUSE	MK_SCX_50.970.970.2	2	2.28	0.173	1	550.3	78.57143	R.RYPSGEER.C
NUIM_MOUSE	MK_SCX_60.15496.15496.2	2	2.078	0.253	1	439.3	78.57143	R.FRGEHALR.R
NUKM_MOUSE	MK_SCX_20_1.4457.4457.2	2	2.348	0.181	1	606.4	61.538464	R.QADVM*IVAGTLTNK.M
NUKM_MOUSE	MK_SCX_2201.3290.3290.2	2	2.87	0.322	1	554.5	87.5	K.VYDQMPEPR.Y
NUKM_MOUSE	MK_SCX_2201.5241.5241.2	2	3.972	0.271	1	1171.7	93.75	K.LDDLINWAR.R
NUKM_MOUSE	MK_SCX_25.9863.9863.3	3	3.958	0.482	1	384.4	34.782608	R.SSLWPMTFGLACCAVEMMHMAAPR.Y
NUKM_MOUSE	MK_SCX_28.4005.4005.3	3	7.134	0.605	1	5606.7	51.923077	R.VHQSVATEGPSPSPSPSLSSTQSAVSK.A

NUKM_MOUSE	MK_SCX_43.9199.9199.3	3	4.231	0.516	1	1701.4	45.833336	R.RSSLWPMTFGLACCAVEMMHMAAPR.Y
NUMM_MOUSE	MK_SCX_23.6939.6939.3	3	4.857	0.481	1	771.4	30.952381	K.EVNENFAIDLIAQQPVNEVEHR.I
NUMM_MOUSE	MK_SCX_38.12662.12662.3	3	4.408	0.341	1	641.7	28.260868	R.QKEVNENFAIDLIAQQPVNEVEHR.I
NUMM_MOUSE	MK_SCX_57.2724.2724.3	3	3.177	0.43	1	707.2	46.42857	K.ITHTGQVYDEKDYRR.V
NUP62_MOUSE	MK_SCX_41.4724.4724.2	2	4.936	0.552	1	2835.6	92.30769	R.HFLQQATQVNAWDR.T
NUPL_MOUSE	MK_SCX_28.3901.3901.3	3	4.681	0.516	1	2082.6	47.727272	K.SSSADFGTFSTSQSHQTASTVSK.V
NUYM_MOUSE	MK_SCX_15.4093.4093.2	2	2.192	0.28	1	710.4	77.77778	K.NGWSYDVEEK.K
NUYM_MOUSE	MK_SCX_16.10397.10397.2	2	5.727	0.664	1	1593.7	56.25	R.WENPLMGWASTADPLSNMVLTFSAK.E
NUYM_MOUSE	MK_SCX_16.10515.10515.3	3	5.581	0.61	1	1648.3	39.583336	R.WENPLMGWASTADPLSNMVLTFSAK.E
NUYM_MOUSE	MK_SCX_16.9141.9141.3	3	3.724	0.366	1	728.7	28.125	R.WENPLMGWASTADPLSNM*VLTFSAK.E
NUYM_MOUSE	MK_SCX_16.9173.9173.2	2	5.217	0.682	1	1067.1	47.916664	R.WENPLMGWASTADPLSNM*VLTFSAK.E
NUYM_MOUSE	MK_SCX_16.9885.9885.3	3	4.696	0.531	1	371.5	33.333336	R.WENPLM*GWASTADPLSNMVLTFSAK.E
NUYM_MOUSE	MK_SCX_16.9958.9958.2	2	5.229	0.715	1	1679.3	56.25	R.WENPLM*GWASTADPLSNMVLTFSAK.E
NUYM_MOUSE	MK_SCX_23.5797.5797.2	2	2.893	0.285	1	1156	58.333332	K.EDAIAFAEKNGWSYDVEEK.K
NUYM_MOUSE	MK_SCX_26.10727.10727.3	3	4.899	0.509	1	606.2	30.769232	R.ERWENPLM*GWASTADPLSNMVLTFSAK.E
NUYM_MOUSE	MK_SCX_26.11471.11471.3	3	5.769	0.639	1	2250.6	38.46154	R.ERWENPLMGWASTADPLSNMVLTFSAK.E
NUYM_MOUSE	MK_SCX_26.9220.9220.3	3	4.867	0.629	1	1961.7	41.346153	R.ERWENPLMGWASTADPLSNM*VLTFSAK.E
NUYM_MOUSE	MK_SCX_27.4220.4220.2	2	2.728	0.315	1	715.8	77.77778	K.SYGANFSWNK.R
NUYM_MOUSE	MK_SCX_28.5646.5646.2	2	4.637	0.53	1	1178.7	75	K.LDITTLTGVPEEHIK.T
NUYM_MOUSE	MK_SCX_28.5665.5665.3	3	4.237	0.428	1	1181.6	51.785713	K.LDITTLTGVPEEHIK.T
NUYM_MOUSE	MK_SCX_47.3883.3883.2	2	2.795	0.423	1	783.8	75	K.SYGANFSWNKR.T
NUYM_MOUSE	MK_SCX_47.4282.4282.2	2	2.373	0.405	1	624.6	78.57143	K.WKMEFDTR.E
NXF1_MOUSE	MK_SCX_17.3377.3377.2	2	5.55	0.601	1	1606	67.64706	R.FEEDDGDVAM*NDPQDGPR.V
OAT_MOUSE	MK_SCX_13.9305.9305.3	3	3.348	0.291	1	328.1	21.527777	R.TLSAISSSTDPTSYDGFGPFMPGFETIPYNDLPALER.A
OAT_MOUSE	MK_SCX_14.8818.8818.2	2	4.66	0.606	1	549.7	35.9375	R.ALQDPNVAAFMVEPIQGEAGVIVPDPGYLTGVR.E
OAT_MOUSE	MK_SCX_19.7322.7322.2	2	4.936	0.557	1	1567	76.666664	R.AFYNNVLGEYEEYITK.L
OAT_MOUSE	MK_SCX_20_1.5250.5250.2	2	2.726	0.223	1	939.3	80	K.IIDAM*KSQVDK.L
OAT_MOUSE	MK_SCX_24.5293.5293.2	2	2.802	0.171	1	839.7	87.5	K.GLLNAIVIR.E
OAT_MOUSE	MK_SCX_31.5107.5107.2	2	3.06	0.449	1	343.8	75	R.LAPPLVIKEDEIR.E
OAT_MOUSE	MK_SCX_31.5504.5504.2	2	4.068	0.461	1	736.9	76.92308	K.ELMKLPSDVVTSVR.G
OAT_MOUSE	MK_SCX_36.3987.3987.2	2	5.244	0.514	1	1427.1	78.57143	K.KTEQGPPSSEYIFER.E
OAT_MOUSE	MK_SCX_36.4022.4022.3	3	4.703	0.451	1	1350.1	50	K.KTEQGPPSSEYIFER.E
OAT_MOUSE	MK_SCX_42.6304.6304.3	3	3.087	0.431	1	445.1	35.294117	R.WLAVDHENVRPDMVLLGK.A
OAT_MOUSE	MK_SCX_50.4787.4787.3	3	4.148	0.563	1	1242.1	51.785713	R.KELMKLPSDVVTSVR.G
OAT_MOUSE	MK_SCX_52.3778.3778.3	3	3.242	0.307	1	420.5	39.285713	K.YGAHNYHPLPVALER.G
OAT_MOUSE	MK_SCX_54.3958.3958.3	3	3.132	0.385	1	1093.9	45.3125	R.LRDNGLLAKPTHGDIIR.L
OCLN_MOUSE	MK_SCX_15.8342.8342.2	2	3.259	0.638	1	625.2	47.61905	K.STPLVPEVAQEIPLTLSVDDFR.Q
OCTC_MOUSE	MK_SCX_24.7892.7892.3	3	4.896	0.512	1	757.2	33.333336	R.TFQYQDSLPSLPVPALEESLKK.Y
OCTC_MOUSE	MK_SCX_48.5261.5261.3	3	6.573	0.575	1	2417.9	47.727272	K.YLESVKPFANEDEYKKTEEIVQK.F
ODB2_MOUSE	MK_SCX_16.12488.12488.3	3	3.929	0.465	1	822.5	18.61702	K.LGSSGQLGTTDLTGGTFTLSNIGSIGGTYAKPVILPPEVAIGALGAIK.A
ODB2_MOUSE	MK_SCX_2201.7320.7320.2	2	3.037	0.275	1	801.4	81.25	K.LSFM*PFFLK.A
ODB2_MOUSE	MK_SCX_2201.8435.8435.2	2	2.757	0.272	1	733	81.25	K.LSFMPFFLK.A
ODB2_MOUSE	MK_SCX_33.7420.7420.2	2	4.366	0.371	1	1410.8	90.909096	R.ILKEDILSFLEK.Q
ODB2_MOUSE	MK_SCX_33.7475.7475.3	3	4.356	0.186	1	1196.6	56.81818	R.ILKEDILSFLEK.Q
ODB2_MOUSE	MK_SCX_44.4928.4928.2	2	3.498	0.442	1	502.4	56.25	R.TFPTPIAKPPVFTGKDR.T
ODB2_MOUSE	MK_SCX_53.3622.3622.2	2	2.465	0.332	1	567	63.636364	K.LREELKPVALAR.G
ODBA_MOUSE	MK_SCX_18.7511.7511.2	2	3.379	0.497	1	583.5	56.666668	K.LEFIQPNVISGIPIYR.V
ODBA_MOUSE	MK_SCX_19.4954.4954.2	2	5.086	0.523	1	1029.3	70	R.AVAENQPFLIEAM*TYR.I
ODBA_MOUSE	MK_SCX_19.6154.6154.2	2	5.404	0.515	1	1269.8	76.666664	R.AVAENQPFLIEAMTYR.I
ODBA_MOUSE	MK_SCX_27.16535.16535.3	3	3.134	0.39	1	357.7	27.272728	R.ISFYMTNYGEEGTHVGSAAALER.T
ODBA_MOUSE	MK_SCX_28.7310.7310.3	3	3.928	0.515	1	868.3	34.782608	R.HFVTISSPLATQIPQAVGAAYAAK.R

ODBA_MOUSE	MK_SCX_44.4123.4123.3	3	3.487	0.437	1	504.1	37.5	R.SVDEVNYWDKQDHPISR.L
ODBA_MOUSE	MK_SCX_46.7901.7901.3	3	4.903	0.497	1	1083.7	36.904762	R.KLKPNPSLLFSDVYQEMPAQLR.R
ODBA_MOUSE	MK_SCX_47.6467.6467.3	3	5.693	0.512	1	1490.9	41.666664	R.KLKPNPSLLFSDVYQEM*PAQLR.R
ODO1_MOUSE	MK_SCX_18.9246.9246.2	2	4.45	0.372	1	1180.6	84.61539	R.IEQLSPFPFDLLLK.E
ODO1_MOUSE	MK_SCX_19.4344.4344.2	2	5.303	0.638	1	702.5	60.526318	R.NTNAGAPPGTAYQSPLSLSR.S
ODO1_MOUSE	MK_SCX_2201.3718.3718.2	2	3.88	0.443	1	1609.8	94.44444	R.NMEEEVAITR.I
ODO1_MOUSE	MK_SCX_27.6787.6787.2	2	2.492	0.358	1	580.6	91.66667	K.SWDIFFR.N
ODO1_MOUSE	MK_SCX_28.4962.4962.3	3	3.499	0.193	1	811.6	37.5	R.SSLATMAHAQSLVEAQPNVDK.L
ODO1_MOUSE	MK_SCX_29.6652.6652.2	2	4.278	0.505	1	1946.7	87.5	R.FLDTAFDLDAFKK.F
ODO1_MOUSE	MK_SCX_30.6570.6570.3	3	3.318	0.43	1	967.2	58.333332	R.FLDTAFDLDAFKK.F
ODO1_MOUSE	MK_SCX_31.5294.5294.2	2	2.855	0.371	1	1207.9	79.16667	K.LVEDHLAVQSLIR.A
ODO1_MOUSE	MK_SCX_33.5217.5217.2	2	3.801	0.498	1	1546	76.92308	R.TSFDEMLPGTHFQR.V
ODO1_MOUSE	MK_SCX_33.5231.5231.3	3	3.115	0.453	1	964.2	51.923077	R.TSFDEMLPGTHFQR.V
ODO1_MOUSE	MK_SCX_33.6977.6977.3	3	4.688	0.57	1	688.5	30.769232	R.VTDRNITLSLVANPSHLEAADPVVMGK.T
ODO1_MOUSE	MK_SCX_33.8944.8944.2	2	4.147	0.545	1	1635.4	67.64706	K.HWLDSPWPGFFTLDGQPR.S
ODO1_MOUSE	MK_SCX_44.4321.4321.2	2	2.594	0.336	1	1028.2	77.77778	R.STRFEEFLQR.K
ODO1_MOUSE	MK_SCX_44.4336.4336.3	3	3.382	0.232	1	1125	55.555557	R.STRFEEFLQR.K
ODO1_MOUSE	MK_SCX_45.5054.5054.2	2	2.038	0.252	1	310.5	60.000004	R.GRLNVLANVIR.K
ODO1_MOUSE	MK_SCX_49.3708.3708.3	3	3.823	0.441	1	520.3	39.473686	R.AKPVWYAGRDPAAAPATGNK.K
ODO2_MOUSE	MK_SCX_14.4801.4801.2	2	2.808	0.275	1	687.8	77.27273	R.NVETMNYADIER.T
ODO2_MOUSE	MK_SCX_16.6171.6171.2	2	5.799	0.633	1	1766.6	72.5	K.NDVITVQTPAFAESVTEGDVR.W
ODO2_MOUSE	MK_SCX_16.7203.7203.2	2	6.465	0.658	1	2034.9	70	K.ASAFALQEQPVVNAVIDDATK.E
ODO2_MOUSE	MK_SCX_21.3898.3898.2	2	3.888	0.437	1	2034.8	86.36364	R.NVETM*NYADIER.T
ODO2_MOUSE	MK_SCX_2201.4584.4584.2	2	3.094	0.297	1	793.5	85	K.VEGGTPLFTLR.K
ODO2_MOUSE	MK_SCX_2201.9062.9062.3	3	5.837	0.516	1	865	32	K.ASAFALQEQPVVNAVIDDATKEVVYR.D
ODO2_MOUSE	MK_SCX_24.5465.5465.2	2	2.676	0.474	1	1317.9	93.75	K.LGFMSAFVK.A
ODO2_MOUSE	MK_SCX_43.5983.5983.3	3	4.017	0.413	1	1738.5	48.333332	K.VEVRPMMYVALTYDHR.L
ODPA_MOUSE	MK_SCX_18.8767.8767.3	3	5.078	0.531	1	1852.1	32.575756	K.EIEDAAQFATADPEPPLEELGYHIYSSDPPFEVR.G
ODPA_MOUSE	MK_SCX_19.4366.4366.2	2	3.495	0.4	1	1490.6	79.16667	R.LEEGPPVTTVLTR.E
ODPA_MOUSE	MK_SCX_20_1.8093.8093.2	2	2.531	0.226	1	349.1	55	K.LPCIFICENNR.Y
ODPA_MOUSE	MK_SCX_21.4590.4590.2	2	3.737	0.247	1	898.1	70.83333	R.MVNSNLASVEELK.E
ODPA_MOUSE	MK_SCX_2201.4924.4924.2	2	2.823	0.255	1	828.8	75	K.GPILM*ELQTYR.Y
ODPA_MOUSE	MK_SCX_2201.5551.5551.2	2	3.856	0.423	1	1548.2	80	K.GPILMELQTYR.Y
ODPA_MOUSE	MK_SCX_26.5147.5147.2	2	4.056	0.409	1	949.1	58.823532	R.LEEGPPVTTVLTREDGLK.Y
ODPA_MOUSE	MK_SCX_27.11855.11855.3	3	4.557	0.565	1	963.2	27.941175	R.KEIEDAAQFATADPEPPLEELGYHIYSSDPPFEVR.G
ODPA_MOUSE	MK_SCX_27.6582.6582.3	3	3.027	0.361	1	653.8	38.157894	R.M*VNSNLASVEELKEIDVEVR.K
ODPA_MOUSE	MK_SCX_27.7150.7150.3	3	4.556	0.459	1	645	39.473686	R.MVNSNLASVEELKEIDVEVR.K
ODPA_MOUSE	MK_SCX_35.11146.11146.3	3	3.95	0.361	1	749.5	25	R.GFCHLCDGQEACCVGLEAGINPTDHLITAYR.A
ODPA_MOUSE	MK_SCX_36.4310.4310.3	3	3.843	0.373	1	933.2	44.230766	R.SGKGPILM*ELQTYR.Y
ODPA_MOUSE	MK_SCX_36.5120.5120.3	3	4.41	0.396	1	1355.9	48.076923	R.SGKGPILMELQTYR.Y
ODPA_MOUSE	MK_SCX_37.5600.5600.2	2	3.992	0.46	1	1593.2	80.769226	R.SGKGPILMELQTYR.Y
ODPA_MOUSE	MK_SCX_55.2949.2949.3	3	4.192	0.472	1	1371.8	53.846157	R.YHGHSMSDPGVSYR.T
ODPB_MOUSE	MK_SCX_19.6014.6014.2	2	3.993	0.438	1	1013.3	53.333336	R.IM*EGPAFNFLDAPAVR.V
ODPB_MOUSE	MK_SCX_19.6353.6353.2	2	5.033	0.522	1	2217.6	76.666664	K.VFLLGEEVAQYDGAYK.V
ODPB_MOUSE	MK_SCX_19.6730.6730.2	2	3.552	0.29	1	798.7	60.000004	K.TYYMSAGLQPVPIVFR.G
ODPB_MOUSE	MK_SCX_19.6733.6733.2	2	4.653	0.624	1	2354.2	73.333336	R.IMEGPAFNFLDAPAVR.V
ODPB_MOUSE	MK_SCX_20_1.3352.3352.2	2	3.58	0.145	1	1050.4	66.66667	R.EAINQGM*DEELER.D
ODPB_MOUSE	MK_SCX_20_1.3641.3641.2	2	3.554	0.383	1	809.1	77.27273	R.VTGADVPM*PYAK.V
ODPB_MOUSE	MK_SCX_20_1.4016.4016.2	2	3.068	0.472	1	1080.5	75	R.EAINQGMDEELER.D
ODPB_MOUSE	MK_SCX_21.3789.3789.2	2	3.677	0.42	1	1054.6	90	K.VLEDNSVPQVK.D
ODPB_MOUSE	MK_SCX_2201.3500.3500.2	2	2.782	0.374	1	410.7	65	K.VVSPWNSEDAK.G

ODPB_MOUSE	MK_SCX_27.15159.15159.3	3	4.046	0.416	1	1064.6	43.055553	K.VFLLGEEVAQYDGAYKVSR.G
ODPB_MOUSE	MK_SCX_28.3958.3958.2	2	3.925	0.537	1	708	56.666668	R.EAINQGMDEELERDEK.V
ODPB_MOUSE	MK_SCX_30.5356.5356.3	3	3.793	0.332	1	1034	43.333332	R.TIRPM*DIEAIEASVM*K.T
ODPB_MOUSE	MK_SCX_30.6939.6939.2	2	4.608	0.475	1	1908.7	73.333336	R.TIRPMDIEAIEASVMK.T
ODPB_MOUSE	MK_SCX_30.6964.6964.3	3	4.678	0.488	1	1910.6	48.333332	R.TIRPMDIEAIEASVMK.T
ODPX_MOUSE	MK_SCX_18.5710.5710.2	2	4.422	0.556	1	818	68.75	K.VLMPSLSPTMEQGNIVK.W
ODPX_MOUSE	MK_SCX_2201.1982.1982.2	2	2.077	0.171	1	313.3	71.42857	K.IVVEEGAK.N
ODPX_MOUSE	MK_SCX_23.5184.5184.2	2	3.273	0.439	1	1760.2	93.75	K.VSVNDFIIR.A
OGFR_MOUSE	MK_SCX_16.4025.4025.2	2	4.699	0.492	1	1915.1	56.81818	K.SLGEDPDSDTTGTSM*SESEELAR.I
OGFR_MOUSE	MK_SCX_16.4523.4523.2	2	5.862	0.551	1	2347.4	65.909096	K.SLGEDPDSDTTGTSMSESEELAR.I
OGFR_MOUSE	MK_SCX_18.3549.3549.2	2	5.906	0.628	1	824.4	55.263157	K.SQVGPEDPQSQVGPEQAASK.S
OGFR_MOUSE	MK_SCX_27.3371.3371.3	3	3.133	0.339	1	684.7	41.17647	K.SQVGPEDPKSQVEPEDPK.S
OGFR_MOUSE	MK_SCX_28.3245.3245.3	3	3.057	0.301	1	561.3	38.235294	K.GQVGPEDPKGQVGPEDPK.G
OLFL3_MOUSE	MK_SCX_12.5986.5986.2	2	2.427	0.253	1	301.5	50	K.NKM*LPLLEVAEKER.E
OLFL3_MOUSE	MK_SCX_31.6220.6220.3	3	3.363	0.248	1	943.1	34.375	K.DPLGPAEKIYVLDGTQNDTAFVFPR.L
OM34_MOUSE	MK_SCX_15.4192.4192.2	2	3.348	0.384	1	2501.2	87.5	R.NGQYGEASALYER.A
OM34_MOUSE	MK_SCX_30.4222.4222.3	3	4.016	0.514	1	1849.2	41.25	R.AAGNQNFRNGQYGEASALYER.A
OM34_MOUSE	MK_SCX_31.6341.6341.3	3	3.516	0.358	1	840	42.857143	R.LKLPPIPVVPVSAQK.R
OPA1_MOUSE	MK_SCX_21.2437.2437.2	2	2.163	0.123	1	746.8	75	R.VVVVGDQSAGK.T
OPA1_MOUSE	MK_SCX_2201.4618.4618.2	2	3.112	0.232	1	463.3	65	K.TSVLEM*IAQAR.I
OPA1_MOUSE	MK_SCX_28.5915.5915.2	2	4.411	0.433	1	1069.3	69.230774	R.EFDLTKEEDLAALR.H
OPA1_MOUSE	MK_SCX_29.5395.5395.3	3	3.035	0.167	1	764.1	42.307693	K.IDQLQEELLHTQLK.Y
OPA1_MOUSE	MK_SCX_29.5443.5443.2	2	3.649	0.283	1	1181.6	69.230774	K.IDQLQEELLHTQLK.Y
OPA1_MOUSE	MK_SCX_30.6652.6652.3	3	3.738	0.382	1	649.9	48.214287	R.SIVTDLVSQMDPHGR.R
OPA1_MOUSE	MK_SCX_30.7648.7648.3	3	3.123	0.16	1	464.9	30.882353	K.AKNEILDEVISLSQVTPK.H
OPA1_MOUSE	MK_SCX_35.5516.5516.2	2	2.201	0.225	1	458.3	85.71429	K.KVKLLTGK.R
OPA1_MOUSE	MK_SCX_41.4159.4159.3	3	5.355	0.589	1	1491.1	48.61111	K.VNDEHPAYLASDEITTVRK.N
OPA1_MOUSE	MK_SCX_50.7021.7021.3	3	4.021	0.446	1	915.1	35.526314	R.EFDLTKEEDLAALRHEIELR.M
OPLA_MOUSE	MK_SCX_17.6005.6005.2	2	4.721	0.427	1	977.1	50	K.GSILDPSPEAAVVGGNVLTSQR.V
OPLA_MOUSE	MK_SCX_18.5019.5019.2	2	4.296	0.441	1	686.1	50	K.LLSEDPANYADAPTEGIR.R
OPLA_MOUSE	MK_SCX_19.8018.8018.2	2	2.89	0.131	1	379.4	45.454548	R.YMREFGFIIPER.S
OPLA_MOUSE	MK_SCX_20_1.8856.8856.2	2	2.005	0.204	1	318.8	39.285713	R.GFQGQLKNVQVLFMR.S
ORN_MOUSE	MK_SCX_18.9553.9553.2	2	2.653	0.479	1	429.2	52.941177	K.ESTVTLQQAEYEFLSFVR.Q
ORN_MOUSE	MK_SCX_21.4182.4182.3	3	3.106	0.248	1	782.2	26.041666	R.RGQFGARGVSEGSAAMAAGESMAQR.M
ORN_MOUSE	MK_SCX_28.7374.7374.3	3	3.047	0.426	1	610.6	43.333332	R.ALDDISESIKELQFYR.N
ORN_MOUSE	MK_SCX_28.7378.7378.2	2	4.556	0.51	1	1890.6	73.333336	R.ALDDISESIKELQFYR.N
ORN_MOUSE	MK_SCX_41.4908.4908.2	2	3.921	0.484	1	1069.1	86.36364	R.RWYPEDYEFAPK.K
ORN_MOUSE	MK_SCX_41.4925.4925.3	3	3.767	0.383	1	1466.3	56.81818	R.RWYPEDYEFAPK.K
OSBL2_MOUSE	MK_SCX_15.6278.6278.2	2	3.407	0.353	1	310.3	30.769232	K.ANSDVPGDVADDVPVAQETVQVIPGSK.L
OSBL3_MOUSE	MK_SCX_17.5602.5602.2	2	4.509	0.462	1	761	52.77778	K.QLMELDTSPSPSAQVVGLK.H
OSBL3_MOUSE	MK_SCX_19.4060.4060.2	2	4.139	0.241	1	1867.5	76.92308	R.LLEEGDIEEAEVQK.Q
OSBL3_MOUSE	MK_SCX_20_1.3127.3127.2	2	4.409	0.441	1	1141	75	R.GEM*TYTQEPPVQK.G
OSBL3_MOUSE	MK_SCX_20_1.3532.3532.2	2	4.124	0.497	1	1259.6	79.16667	R.GEMTYTQEPPVQK.G
OSBL3_MOUSE	MK_SCX_2201.7003.7003.3	3	3.104	0.251	1	423.6	27.083334	R.DVNHFFSGSSVTDSAPGVFESVSSR.K
OSBL3_MOUSE	MK_SCX_28.5879.5879.3	3	3.431	0.284	1	1050.9	38.75	K.LKQLMELDTSPSPSAQVVGLK.H
OSBL3_MOUSE	MK_SCX_51.3437.3437.3	3	3.676	0.466	1	793.2	41.666664	K.HALSSALAQNTDLKER.L
OSBL6_MOUSE	MK_SCX_2201.5324.5324.2	2	3.366	0.294	1	1470	94.44444	K.LLQNLEILQR.T
OSBL6_MOUSE	MK_SCX_2201.6956.6956.2	2	3.678	0.278	1	529.1	75	K.SQDWFDAWVSK.L
OSTF1_MOUSE	MK_SCX_15.7181.7181.2	2	4.223	0.534	1	732.6	55.555557	K.DIVEVLFTQPNVELNQQNK.L
OSTF1_MOUSE	MK_SCX_20_1.9160.9160.2	2	4.141	0.53	1	1646.3	86.36364	K.GYADIVQLLLAK.G
OSTF1_MOUSE	MK_SCX_23.6751.6751.2	2	4.586	0.597	1	724.7	43.75	R.TGLIPSNYVAEQAESIDNPLHEAAK.R

OSTP_MOUSE	MK_SCX_17.3055.3055.2	2	4.599	0.619	1	555.3	55.555557	K.ESQESADQSDVIDSQASSK.A
OSTP_MOUSE	MK_SCX_41.2971.2971.3	3	5.213	0.554	1	1944.1	39.130436	R.LEHSKESQESADQSDVIDSQASSK.A
OTU6B_MOUSE	MK_SCX_21.4404.4404.2	2	4.569	0.419	1	2385.1	87.5	R.IAEAEIENLSGAR.H
OTUB1_MOUSE	MK_SCX_19.4608.4608.2	2	5.528	0.574	1	2475.3	82.14286	R.IQQEIAVQNPLVSER.L
OTUB1_MOUSE	MK_SCX_20_1.3410.3410.2	2	3.257	0.416	1	855.1	68.181816	K.EYAEDDNIYQQK.I
OTUB1_MOUSE	MK_SCX_44.7485.7485.3	3	4.489	0.522	1	1272	43.055553	R.AFGFSHLEALLDDSKELQR.F
OXR1_MOUSE	MK_SCX_18.3122.3122.2	2	3.833	0.434	1	909.4	62.5	K.MDESEANEAPAGEAAAR.E
OXSR1_MOUSE	MK_SCX_24.15502.15502.3	3	3.451	0.255	1	311.9	25	R.FEFTPGRDTAEGVSQELISAGLVDGR.D
OXSR1_MOUSE	MK_SCX_34.5625.5625.3	3	3.718	0.411	1	657.7	47.916664	R.SQETKIPISLVLR.L
P25A_MOUSE	MK_SCX_18.9395.9395.2	2	4.008	0.19	1	1010.8	70	R.TITFEQFQEALEELAK.K
P3H1_MOUSE	MK_SCX_43.3261.3261.3	3	3.883	0.393	1	659.8	28.260868	R.LTNAAATSGDGYRGQTSPHTPNEK.F
P4HA1_MOUSE	MK_SCX_41.4704.4704.3	3	5.616	0.431	1	2191.7	45	R.LTSTATKDPEGFVGHPVNAFK.L
P66B_MOUSE	MK_SCX_18.4292.4292.2	2	5.274	0.667	1	1465.7	68.42105	R.LQQQAALSPTTAPAVSSVSK.Q
P85A_MOUSE	MK_SCX_21.3181.3181.2	2	2.485	0.128	1	382.7	55	K.QAAEYREIDKR.M
P85A_MOUSE	MK_SCX_29.3860.3860.3	3	3.637	0.497	1	784.7	45.3125	K.YQQDQVVKEDNIEAVGK.K
PA1B2_MOUSE	MK_SCX_2201.6750.6750.2	2	3.553	0.387	1	985.7	81.25	K.IIVLGLLPR.G
PA1B2_MOUSE	MK_SCX_28.7691.7691.2	2	5.317	0.588	1	1529.7	69.44444	R.ELFSPLHALNFGIGGDTTR.H
PA1B2_MOUSE	MK_SCX_28.7809.7809.3	3	3.715	0.272	1	938.9	45.833336	R.ELFSPLHALNFGIGGDTTR.H
PA2G4_MOUSE	MK_SCX_20_1.4989.4989.2	2	3.531	0.483	1	576.5	68.181816	R.ITSGPFEPDLYK.S
PA2G4_MOUSE	MK_SCX_23.6454.6454.3	3	3.659	0.439	1	530.4	30.681818	R.ITSGPFEPDLYKSEM*EVQDAELK.A
PA2G4_MOUSE	MK_SCX_23.6523.6523.3	3	4.068	0.376	1	611.9	29.545454	R.ITSGPFEPDLYKSEMEVQDAELK.A
PA2G4_MOUSE	MK_SCX_23.6524.6524.2	2	3.233	0.445	1	441.9	43.18182	R.ITSGPFEPDLYKSEMEVQDAELK.A
PA2G4_MOUSE	MK_SCX_35.3723.3723.3	3	4.655	0.485	1	747.9	39.0625	R.LVKPGNQNTQVTEAWNK.V
PA2G4_MOUSE	MK_SCX_35.3749.3749.2	2	4.487	0.353	1	963.4	65.625	R.LVKPGNQNTQVTEAWNK.V
PA2G4_MOUSE	MK_SCX_35.9981.9981.2	2	3.524	0.282	1	1022.7	75	K.HELLQPFNVLYEK.E
PA2G4_MOUSE	MK_SCX_43.5547.5547.2	2	3.092	0.366	1	535.6	66.66667	R.RFDAMPFTLR.A
PABP1_MOUSE	MK_SCX_20_1.4908.4908.2	2	3.074	0.41	1	1181.3	80	R.ALDTM*NFDVIK.G
PABP1_MOUSE	MK_SCX_20_1.5850.5850.2	2	3.444	0.487	1	1303	85	R.ALDTMNFDVIK.G
PABP1_MOUSE	MK_SCX_2201.5150.5150.2	2	2.537	0.278	1	424.3	75	K.FSPAGPILSIR.V
PABP1_MOUSE	MK_SCX_2201.8952.8952.3	3	3.672	0.259	1	570.8	26.041666	K.ALYDTFSAFGNILSCKVVCDENGSK.G
PABP1_MOUSE	MK_SCX_25.5439.5439.2	2	3.246	0.447	1	1080.3	87.5	K.GFGFVSFER.H
PABP1_MOUSE	MK_SCX_33.4747.4747.3	3	4.032	0.361	1	969.9	53.846157	R.IVATKPLYVALAQR.K
PABP1_MOUSE	MK_SCX_33.4757.4757.2	2	4.387	0.634	1	784.9	84.61539	R.IVATKPLYVALAQR.K
PABP1_MOUSE	MK_SCX_49.4423.4423.3	3	4.075	0.448	1	1266.8	48.333332	R.SKVDEAVAVLQAHQAK.E
PABP2_MOUSE	MK_SCX_16.6169.6169.3	3	4.733	0.521	1	362.4	18.47826	R.APPGAPGPGPGSGAPGSQEEEEEPGLVEADPGDGAIEDPELEAIKAR
PABP2_MOUSE	MK_SCX_17.9306.9306.2	2	2.879	0.129	1	379.9	38.235294	R.EMEEEAEKLKELQNEVEK.Q
PACN2_MOUSE	MK_SCX_23.4782.4782.2	2	3.874	0.28	1	1779.5	90	K.AWIAVMSEAER.V
PACN2_MOUSE	MK_SCX_24.4588.4588.2	2	4.363	0.444	1	1741.2	80	K.AYAQQLTEWAR.R
PACN2_MOUSE	MK_SCX_31.8014.8014.2	2	5.45	0.653	1	1160.8	54.545456	R.ANHGPGMAMNWPQFEEWSADLNR.T
PACN2_MOUSE	MK_SCX_31.8027.8027.3	3	6.043	0.529	1	2115	40.909092	R.ANHGPGMAMNWPQFEEWSADLNR.T
PACN2_MOUSE	MK_SCX_39.4281.4281.2	2	3.313	0.492	1	1019.8	85	K.HLDLSNVASYK.T
PACN2_MOUSE	MK_SCX_39.5216.5216.2	2	2.906	0.281	1	672.7	80	K.TIYRELEQSIK.A
PACN2_MOUSE	MK_SCX_45.3344.3344.3	3	3.66	0.385	1	461.2	39.0625	R.EANSKADPSLNPEQLKK.L
PACN3_MOUSE	MK_SCX_16.5533.5533.2	2	5.594	0.63	1	2531.2	83.33333	R.DLQQSIEAASDEEDLR.W
PACN3_MOUSE	MK_SCX_2201.4241.4241.2	2	2.75	0.343	1	413.3	72.72727	K.TQYEQTLAELNR.Y
PACN3_MOUSE	MK_SCX_24.4622.4622.2	2	3.832	0.557	1	1973.5	85	K.AYAQQLADWAR.K
PACN3_MOUSE	MK_SCX_30.7563.7563.3	3	4.795	0.577	1	986.3	34.090908	R.STHGPGMAMNWPQFEEWSLDTQR.A
PACN3_MOUSE	MK_SCX_32.5010.5010.3	3	3.765	0.316	1	622.5	39.0625	K.GGRSPDEVTLTSIVPTR.D
PAD5_MOUSE	MK_SCX_14.4586.4586.2	2	2.301	0.204	1	900.6	90.909096	K.YPVLPYLVLVLK.Q
PAD5_MOUSE	MK_SCX_16.3095.3095.2	2	2.725	0.165	1	317.9	43.333332	R.VGSQDVSLEVSQAVGK.M
PAD5_MOUSE	MK_SCX_35.4057.4057.2	2	2.413	0.169	1	306.9	46.666668	R.SGERPLGGLAVPAEQR.D

PAHX_MOUSE	MK_SCX_23.5432.5432.2	2	3.443	0.423	1	1269.9	83.33333	K.YGFQGVMDFK.D
PAHX_MOUSE	MK_SCX_36.3629.3629.3	3	3.108	0.23	1	336.5	38.46154	K.M*YHGIQDYDPNSPR.V
PAHX_MOUSE	MK_SCX_36.3644.3644.2	2	4.055	0.506	1	869	73.07692	K.MYHGIQDYDPNSPR.V
PAHX_MOUSE	MK_SCX_47.5621.5621.2	2	4.551	0.648	1	1079.2	73.333336	K.GDTVFFHPLLIHGSGR.N
PAHX_MOUSE	MK_SCX_48.5286.5286.3	3	3.262	0.527	1	927.6	41.666664	K.GDTVFFHPLLIHGSGR.N
PAIP2_MOUSE	MK_SCX_14.10319.10319.3	3	4.573	0.377	1	1074	30.172413	R.DLPQTMDQIQDQFNDLVISDGSSLEDLVVK.S
PAIRB_MOUSE	MK_SCX_16.7419.7419.2	2	6.037	0.495	1	2513.3	80	R.FDQLFDDESDPFEVLK.A
PAIRB_MOUSE	MK_SCX_50.3843.3843.3	3	4.572	0.519	1	1287.1	43.421055	R.GGSGSHNWGTVKDELTESPK.Y
PAIRB_MOUSE	MK_SCX_52.3272.3272.3	3	4.294	0.48	1	1341.7	48.214287	R.RPDQQLQGDGKLIDR.R
PAIRB_MOUSE	MK_SCX_55.2984.2984.3	3	4.414	0.578	1	1220.2	48.333332	K.SEEAHAEDSVM*DHHFR.K
PAIRB_MOUSE	MK_SCX_59.9845.9845.3	3	3.287	0.361	1	440.8	39.705883	K.SKSEEAHAEDSVMDHHFR.K
PAK1_MOUSE	MK_SCX_15.4866.4866.2	2	4.631	0.568	1	1410.2	64.28571	R.DVATSPISPTENNTTPPDALTR.N
PAK1_MOUSE	MK_SCX_19.5982.5982.2	2	4.781	0.476	1	1229.9	75	R.STMVGTPYWMAPEVVTR.K
PAK1_MOUSE	MK_SCX_38.3321.3321.3	3	3.95	0.345	1	1837.3	45.833336	K.DTGTLNHGSKPLPPNPEEK.K
PAK2_MOUSE	MK_SCX_17.4899.4899.2	2	4.81	0.597	1	681.8	63.15789	R.SVIDPIPAPVGDSNVDSGAK.S
PAK2_MOUSE	MK_SCX_26.5764.5764.2	2	3.135	0.164	1	320.8	38.095238	K.YLSFTPPEKDGFPSGTPALNTK.G
PAK2_MOUSE	MK_SCX_30.6720.6720.2	2	4.676	0.578	1	685.8	66.66667	K.LAKPLSSLTPLILAAK.E
PAK2_MOUSE	MK_SCX_40.4372.4372.2	2	2.953	0.424	1	910.9	85	K.KNPQAVLDVLK.F
PAK2_MOUSE	MK_SCX_40.4378.4378.3	3	3.094	0.328	1	656.5	50	K.KNPQAVLDVLK.F
PALM_MOUSE	MK_SCX_16.3908.3908.2	2	4.262	0.593	1	307.8	36.842106	K.EENQTGPTTTPSDTQDLDMK.K
PALM_MOUSE	MK_SCX_18.4031.4031.2	2	5.337	0.549	1	2129.9	69.44444	K.ADEVTLSEAGSTAGPAEPR.G
PALM_MOUSE	MK_SCX_18.4985.4985.2	2	5.472	0.643	1	1509.4	82.35294	R.WLLEGTPSSASEGDEDMR.K
PALM_MOUSE	MK_SCX_28.4534.4534.2	2	4.833	0.652	1	785.7	58.333332	R.WLLEGTPSSASEGDEDMRK.Q
PALM_MOUSE	MK_SCX_30.3852.3852.3	3	4.577	0.584	1	614.5	38.88889	K.SETLVNAQQTPLGTPKENR.T
PAPS1_MOUSE	MK_SCX_17.7987.7987.2	2	4.106	0.578	1	1518.3	71.875	K.VLTM*APGLITLEIVPFR.V
PAPS2_MOUSE	MK_SCX_14.7848.7848.2	2	5.136	0.534	1	861.1	66.66667	R.DGVINMSIPIVLPVSADDK.A
PAPS2_MOUSE	MK_SCX_18.7727.7727.2	2	5.023	0.537	1	1239.5	71.875	K.VLSMAPGLTSVEIIPFR.V
PAPS2_MOUSE	MK_SCX_39.4506.4506.2	2	3.181	0.447	1	1161.5	85	R.HEEFDFISGTR.M
PAPS2_MOUSE	MK_SCX_48.4916.4916.2	2	4.711	0.557	1	974.1	73.333336	K.GIHELFVPENKVDQIR.A
PAPS2_MOUSE	MK_SCX_53.3487.3487.3	3	3.012	0.465	1	585.2	37.5	K.NLGFSAGDREENIRR.I
PARD3_MOUSE	MK_SCX_31.8379.8379.2	2	3.063	0.141	1	779	44.11765	R.ISHSLYSGIEGLDESPTR.N
PARD3_MOUSE	MK_SCX_34.3545.3545.3	3	3.693	0.315	1	811.8	37.5	R.SGRESVSTSSDQPSYSLER.Q
PARG_MOUSE	MK_SCX_14.3530.3530.2	2	3.403	0.491	1	757	67.85714	K.SSQYLNQQQTASVCK.W
PARK7_MOUSE	MK_SCX_13.4735.4735.2	2	2.344	0.254	1	474.8	53.846157	R.DVM*ICPDTSLEDAK.T
PARK7_MOUSE	MK_SCX_13.5047.5047.2	2	3.213	0.403	1	1180.4	76.92308	R.DVMICPDTSLEDAK.T
PARK7_MOUSE	MK_SCX_16.6323.6323.2	2	4.715	0.549	1	526.7	46.153847	K.TQGPYDVVVLPGGNLGAQNLSESPM*VK.E
PARK7_MOUSE	MK_SCX_16.6372.6372.3	3	4.242	0.52	1	1270.7	36.53846	K.TQGPYDVVVLPGGNLGAQNLSESPM*VK.E
PARK7_MOUSE	MK_SCX_16.6707.6707.3	3	4.437	0.515	1	897	31.730768	K.TQGPYDVVVLPGGNLGAQNLSESPMVK.E
PARK7_MOUSE	MK_SCX_16.6867.6867.2	2	4.694	0.473	1	451.2	46.153847	K.TQGPYDVVVLPGGNLGAQNLSESPMVK.E
PARK7_MOUSE	MK_SCX_18.4499.4499.2	2	3.815	0.354	1	885.3	75	K.GAEEM*ETVIPVDVM*R.R
PARK7_MOUSE	MK_SCX_18.5092.5092.2	2	4.419	0.474	1	1012.3	75	K.GAEEM*ETVIPVDVMR.R
PARK7_MOUSE	MK_SCX_18.6046.6046.2	2	4.447	0.447	1	1284.6	78.57143	K.GAEEMETVIPVDVMR.R
PARK7_MOUSE	MK_SCX_29.5841.5841.3	3	3.181	0.251	1	600.2	33.333336	K.GAEEMETVIPVDVMRR.A
PARK7_MOUSE	MK_SCX_36.3482.3482.3	3	3.111	0.207	1	603.7	45	R.VEKDGLILTSR.G
PARK7_MOUSE	MK_SCX_43.3196.3196.2	2	2.902	0.328	1	683	60.714287	K.DKMMNGSHYSYSESR.V
PARK7_MOUSE	MK_SCX_45.9926.9926.3	3	3.172	0.352	1	301.1	23.913044	R.KGLIAAICAGPTALLAHEVGFGCK.V
PARP1_MOUSE	MK_SCX_23.11279.11279.3	3	3.569	0.395	1	721.4	29.166666	R.LGTVIGSNKLEQM*PSKEEAVEQFMK.L
PARP1_MOUSE	MK_SCX_23.5998.5998.2	2	2.942	0.261	1	524.5	58.333332	R.TTNFAGILSQGLR.I
PARP1_MOUSE	MK_SCX_26.7623.7623.3	3	3.983	0.469	1	1053.3	38.636364	R.VADGMAFGALLPCKECSGQLVFK.S
PARP1_MOUSE	MK_SCX_27.3606.3606.3	3	4.863	0.512	1	1301.2	48.4375	R.GGSDDSSKDPIDVNYEK.L
PARP1_MOUSE	MK_SCX_47.5244.5244.3	3	3.328	0.455	1	416.3	37.5	K.VGQSIRHPDVEVDGFSELR.W

PAWR_MOUSE	MK_SCX_36.6298.6298.3	3	6.335	0.527	1	1654.9	37.5	R.KREDAITQQNTIQNEAATLPDPGTSYLPQDPSR.T
PAXI_MOUSE	MK_SCX_06.3449.3449.2	2	2.386	0.437	1	332.3	46.875	K.NSSASNTQDGVGSLCSR.A
PAXI_MOUSE	MK_SCX_17.5021.5021.2	2	5.006	0.491	1	1206.9	66.66667	K.SAEPSPTVM*SSSLGSNLSELDR.L
PAXI_MOUSE	MK_SCX_17.5784.5784.2	2	6.058	0.661	1	1587.9	69.047615	K.SAEPSPTVMSSSLGSNLSELDR.L
PAXI_MOUSE	MK_SCX_18.10200.10200.3	3	5.422	0.585	1	981.9	28.947369	R.GLEDVRPSVESLLDELESSVPSPVPAITVNQGEM*SSPQR.V
PAXI_MOUSE	MK_SCX_18.7368.7368.2	2	4.186	0.465	1	1794.1	87.5	R.ELDELMASLSDFK.M
PAXI_MOUSE	MK_SCX_33.3774.3774.3	3	4.279	0.393	1	904.8	42.647057	R.YAHQQPPSPLPVYSSSAK.N
PAXI_MOUSE	MK_SCX_33.3816.3816.2	2	4.043	0.418	1	761.8	67.64706	R.YAHQQPPSPLPVYSSSAK.N
PAXI_MOUSE	MK_SCX_49.3216.3216.3	3	4.122	0.373	1	1548.3	48.214287	R.AGEEEHVYSFPNKQK.S
PCBP1_MOUSE	MK_SCX_14.3378.3378.2	2	3.787	0.378	1	911.3	80	R.INISEGNCPER.I
PCBP1_MOUSE	MK_SCX_18.4012.4012.2	2	5.81	0.563	1	1235	57.894737	R.ESTGAQVQVAGDM*LPNSTER.A
PCBP1_MOUSE	MK_SCX_18.4744.4744.2	2	5.854	0.57	1	1055.3	60.526318	R.ESTGAQVQVAGDMLPNSTER.A
PCBP1_MOUSE	MK_SCX_20_1.6514.6514.2	2	3.798	0.531	1	792.4	66.66667	R.IITLTGPTNAIFK.A
PCBP1_MOUSE	MK_SCX_23.9509.9509.3	3	3.625	0.311	1	511	23.4375	R.AITIAGVPQSVTECVKQICLVMLETLSQSPQGR.V
PCBP1_MOUSE	MK_SCX_25.4904.4904.3	3	4.333	0.42	1	981.7	39.772728	K.LEEDINSSMTNSTAASRPPVTLR.L
PCBP1_MOUSE	MK_SCX_27.5097.5097.3	3	6.199	0.599	1	1159.1	35.416664	R.QQSHFAMMHGGTGFAGIDSSSPEVK.G
PCBP1_MOUSE	MK_SCX_51.3842.3842.3	3	3.8	0.512	1	679.2	48.076923	R.LLMHGKEVGSIIGK.K
PCBP1_MOUSE	MK_SCX_51.3856.3856.2	2	3.463	0.458	1	1732.6	76.92308	R.LLMHGKEVGSIIGK.K
PCBP2_MOUSE	MK_SCX_20_1.6359.6359.2	2	2.921	0.297	1	575.7	58.333332	R.IITLAGPTNAIFK.A
PCBP2_MOUSE	MK_SCX_41.5157.5157.3	3	4.622	0.58	1	703.5	36.363636	K.GVTIPYRPKPSSSPVIFAGGQDR.Y
PCCA_MOUSE	MK_SCX_16.5916.5916.2	2	4.144	0.515	1	760.4	50	R.VDSGIQPGSDISIYYDPM*ISK.L
PCCA_MOUSE	MK_SCX_16.6443.6443.2	2	5.097	0.601	1	907.1	57.5	R.VDSGIQPGSDISIYYDPMISK.L
PCCA_MOUSE	MK_SCX_21.3915.3915.2	2	4.358	0.651	1	1726.7	80.769226	R.FSSQEAASSFGDDR.L
PCCA_MOUSE	MK_SCX_23.6135.6135.3	3	3.911	0.374	1	857.5	33.333336	R.LAAEDVTFIGPDTHAIQAMGDK.I
PCCA_MOUSE	MK_SCX_24.5898.5898.2	2	2.734	0.18	1	927.3	77.77778	K.NFYFLEMNTR.L
PCCA_MOUSE	MK_SCX_26.6161.6161.2	2	4.171	0.531	1	1079.2	69.44444	K.VNTIPGFDGVVKDADEAVR.I
PCCA_MOUSE	MK_SCX_30.6784.6784.2	2	3.973	0.379	1	416.3	40	R.LAAEDVTFIGPDTHAIQAMGDKIESK.L
PCCA_MOUSE	MK_SCX_31.7630.7630.2	2	3.299	0.296	1	616.8	57.692307	K.SYLNMDAIMEAIKK.T
PCCA_MOUSE	MK_SCX_32.5000.5000.3	3	3.269	0.316	1	395.4	34.615387	R.LSQYQEPIHLPGVR.V
PCCA_MOUSE	MK_SCX_33.4934.4934.2	2	4.094	0.535	1	1519.3	80.769226	R.LSQYQEPIHLPGVR.V
PCCA_MOUSE	MK_SCX_40.4575.4575.2	2	2.064	0.141	1	428.4	61.11111	K.TFDKILIANR.G
PCCA_MOUSE	MK_SCX_41.3980.3980.2	2	2.553	0.405	1	428	66.66667	R.GVTHNIPLLR.E
PCCA_MOUSE	MK_SCX_41.6035.6035.3	3	4.297	0.515	1	1103.8	37.5	R.AKVNTIPGFDGVVKDADEAVR.I
PCCA_MOUSE	MK_SCX_45.3648.3648.3	3	4.843	0.45	1	1879.9	58.333332	K.TVAIHSDVDASSVHVK.M
PCCA_MOUSE	MK_SCX_46.4635.4635.3	3	4.236	0.434	1	1012.9	41.666664	R.AQAVHPGYGFLSENKEFAK.R
PCCA_MOUSE	MK_SCX_48.6765.6765.3	3	5.131	0.602	1	678.6	28.846153	K.RLAAEDVTFIGPDTHAIQAMGDKIESK.L
PCCB_MOUSE	MK_SCX_15.6312.6312.2	2	3.342	0.372	1	1092.9	54.761906	K.IMDQAITVGAPVIGLNDSGGAR.I
PCCB_MOUSE	MK_SCX_18.11500.11500.2	2	3.102	0.434	1	355.5	53.125	R.IQEGVESLAGYADIFLR.N
PCCB_MOUSE	MK_SCX_18.5946.5946.2	2	2.3	0.237	1	516.9	57.14286	R.LVPELDTVVPLESSK.A
PCCB_MOUSE	MK_SCX_20_1.3556.3556.2	2	5.413	0.484	1	1613	73.333336	K.SVTNEDVTQEQLGGAK.T
PCCB_MOUSE	MK_SCX_20_1.5241.5241.2	2	3.518	0.312	1	912.2	70.83333	R.GFVDDIIQPSSTR.A
PCCB_MOUSE	MK_SCX_24.4620.4620.2	2	2.419	0.39	1	465.5	77.77778	K.FANPFPAAVR.G
PCCB_MOUSE	MK_SCX_29.7720.7720.3	3	3.269	0.141	1	727	32.5	K.HLLGDTNYAWPTAEIAVMGAK.G
PCLO_MOUSE	MK_SCX_23.5853.5853.2	2	2.334	0.214	1	410.4	53.846157	R.QIAAVMSRAQGLPK.G
PCLO_MOUSE	MK_SCX_57.11061.11061.2	2	2.085	0.331	1	414.7	63.636364	K.DHTVSGNGLGIR.I
PCNP_MOUSE	MK_SCX_24.4407.4407.2	2	3.119	0.527	1	1010.4	90	K.FGFAIGSQTAR.K
PCNP_MOUSE	MK_SCX_49.3214.3214.3	3	3.338	0.34	1	1443.3	48.333332	K.RSAEDEAADLPTKPTK.M
PCSK4_MOUSE	MK_SCX_16.9593.9593.2	2	3.463	0.307	1	416.2	38.636364	R.CAGEVSATANNGFCGAGVAFNAR.I
PCY1A_MOUSE	MK_SCX_18.9400.9400.2	2	4.719	0.45	1	1682.4	71.875	R.EFIGSFLEMFGPEGALK.H
PCY1A_MOUSE	MK_SCX_24.5397.5397.3	3	5.124	0.528	1	1317.4	40	R.IDFVAHDDIPYSSAGSDDVYK.H
PCY1A_MOUSE	MK_SCX_31.6728.6728.2	2	4.186	0.538	1	964.5	71.42857	R.NAPWTLTPEFLAEHR.I

PCY1A_MOUSE	MK_SCX_49.6932.6932.2	2	2.114	0.129	1	395.1	56.666668	R.VYADGIFDLFHSGHAR.A
PCYOX_MOUSE	MK_SCX_16.6024.6024.2	2	2.497	0.342	1	455.5	52.63158	K.ELGLSSVPASGGLVGVYNGK.S
PCYOX_MOUSE	MK_SCX_16.8942.8942.2	2	4.74	0.523	1	412.6	50	R.NFDPPIEEFNDPYQQLVTTFIK.G
PCYOX_MOUSE	MK_SCX_53.4666.4666.3	3	5.954	0.602	1	2094.9	50	K.VQGHDYEAGGSVIHPLNLHMK.R
PDC10_MOUSE	MK_SCX_31.4117.4117.2	2	4.146	0.413	1	1180.5	75	K.AEKENPGLTQDIIMK.I
PDC6I_MOUSE	MK_SCX_13.9837.9837.3	3	3.282	0.472	1	495.6	24.242424	R.EATTLANGVLASLNLPAAIEDVSGDTVPQSILTK.S
PDC6I_MOUSE	MK_SCX_18.5928.5928.2	2	3.093	0.285	1	308.5	50	K.STSVVEQGGIQTVDQLIK.E
PDCD5_MOUSE	MK_SCX_2201.5337.5337.2	2	3.194	0.315	1	1480	80	K.AVENYLIQMAR.Y
PDCD5_MOUSE	MK_SCX_2201.6084.6084.2	2	4.628	0.573	1	1706.8	83.33333	R.NSILAQVLDQSAR.A
PDCD6_MOUSE	MK_SCX_15.7787.7787.2	2	3.5	0.341	1	989.2	44.230766	R.SGVISDNELQQALSNGTWTPFNPVTVR.S
PDCD6_MOUSE	MK_SCX_2201.7111.7111.2	2	3.692	0.328	1	1459.7	75	K.AGVNFSEFTGVWK.Y
PDCD6_MOUSE	MK_SCX_48.3232.3232.3	3	3.942	0.444	1	714.2	41.666664	R.TYDRDNSGMIDKNELK.Q
PDCD8_MOUSE	MK_SCX_14.9034.9034.2	2	2.874	0.426	1	318.5	31.034481	R.SIYFQPPSFYVSAQDLPNIENGGVAVLTGK.K
PDCD8_MOUSE	MK_SCX_20_1.4475.4475.2	2	4.151	0.581	1	1457	76.666664	K.VM*PNAIVQSVGVSGGR.L
PDCD8_MOUSE	MK_SCX_20_1.6233.6233.2	2	3.529	0.42	1	1660	80.769226	K.TGGLEIDSDFGGFR.V
PDCD8_MOUSE	MK_SCX_21.3919.3919.2	2	3.233	0.386	1	411.2	70	K.LNDGSQITFEK.C
PDCD8_MOUSE	MK_SCX_21.4132.4132.2	2	4.264	0.484	1	1454.9	82.14286	R.AIASATEGGSVPQIR.A
PDCD8_MOUSE	MK_SCX_21.4855.4855.2	2	4.163	0.467	1	868.4	70	K.VMPNAIVQSVGVSGGR.L
PDCD8_MOUSE	MK_SCX_21.5973.5973.2	2	3.553	0.551	1	488.7	70.83333	K.ILPQYLSNWTM*EK.V
PDCD8_MOUSE	MK_SCX_21.6482.6482.2	2	3.786	0.43	1	486	70.83333	K.ILPQYLSNWTMEK.V
PDCD8_MOUSE	MK_SCX_24.5743.5743.3	3	3.627	0.345	1	905	36.842106	K.VETDHIVTAVGLEPNVELAK.T
PDCD8_MOUSE	MK_SCX_24.5774.5774.3	3	3.312	0.479	1	418.2	31.944445	R.VLIVSEDPELPYM*RPPLSK.E
PDCD8_MOUSE	MK_SCX_24.5781.5781.2	2	6.026	0.595	1	1969.6	63.15789	K.VETDHIVTAVGLEPNVELAK.T
PDCD8_MOUSE	MK_SCX_24.6677.6677.2	2	3.562	0.438	1	396.7	50	R.VLIVSEDPELPYMRPPLSK.E
PDCD8_MOUSE	MK_SCX_25.6635.6635.3	3	4.262	0.567	1	665.9	36.11111	R.VLIVSEDPELPYMRPPLSK.E
PDCD8_MOUSE	MK_SCX_30.7019.7019.3	3	4.593	0.281	1	1523.7	37.5	R.APSHVPFLLIGGGTAAFAAAR.S
PDCD8_MOUSE	MK_SCX_39.3652.3652.3	3	4.097	0.454	1	624.6	43.333332	R.RAIASATEGGSVPQIR.A
PDCD8_MOUSE	MK_SCX_39.6380.6380.3	3	3.794	0.416	1	583.3	35	R.KVETDHIVTAVGLEPNVELAK.T
PDCD8_MOUSE	MK_SCX_40.3676.3676.2	2	4.864	0.482	1	1382.8	80	R.RAIASATEGGSVPQIR.A
PDCD8_MOUSE	MK_SCX_45.3237.3237.3	3	3.863	0.384	1	1034.4	50	K.IIKDGEQHEDLNEVAK.L
PDCD8_MOUSE	MK_SCX_45.5570.5570.3	3	4.074	0.467	1	1179.8	41.25	R.KSQASGIEVIQLFPEKGNM*GK.I
PDCD8_MOUSE	MK_SCX_45.5874.5874.3	3	4.295	0.38	1	1175	40	R.KSQASGIEVIQLFPEKGNMGK.I
PDCD8_MOUSE	MK_SCX_55.3044.3044.3	3	5.398	0.49	1	1865.7	53.125	R.KIIKDGEQHEDLNEVAK.L
PDCD8_MOUSE	MK_SCX_55.3054.3054.2	2	5.676	0.548	1	1877.8	75	R.KIIKDGEQHEDLNEVAK.L
PDE4C_MOUSE	MK_SCX_23.8854.8854.3	3	4.226	0.213	1	903.5	32	R.TSSAASDLHGEDM*IVTPFAQVLASLR.T
PDE4C_MOUSE	MK_SCX_23.9439.9439.3	3	4.228	0.273	1	1048.5	31	R.TSSAASDLHGEDMIVTPFAQVLASLR.T
PDIA1_MOUSE	MK_SCX_15.14770.14770.3	3	4.877	0.486	1	871.2	33.62069	R.TGPAATTLSDTAAAESLVDSSEVTVIGFFK.D
PDIA1_MOUSE	MK_SCX_16.9261.9261.3	3	5.721	0.617	1	1596.3	39.583336	K.QFLLAAEAIDDIPFGITSNSGVFSK.Y
PDIA1_MOUSE	MK_SCX_16.9435.9435.2	2	5.999	0.56	1	1185.9	54.166668	K.QFLLAAEAIDDIPFGITSNSGVFSK.Y
PDIA1_MOUSE	MK_SCX_18.10806.10806.3	3	4.578	0.596	1	925.7	29.6875	K.DVESDSAKQFLLAAEAIDDIPFGITSNSGVFSK.Y
PDIA1_MOUSE	MK_SCX_18.4516.4516.2	2	5.627	0.62	1	2334.3	76.666664	K.VDATEESDLAQQYGVR.G
PDIA1_MOUSE	MK_SCX_19.6227.6227.2	2	4.34	0.481	1	1208.1	67.85714	K.VLVGANFEEVAFDEK.K
PDIA1_MOUSE	MK_SCX_20_1.3291.3291.2	2	4.021	0.437	1	2073.1	90.909096	K.MDSTANEVEAVK.V
PDIA1_MOUSE	MK_SCX_21.4523.4523.2	2	2.928	0.466	1	844.9	88.88889	R.LITLEEEMTK.Y
PDIA1_MOUSE	MK_SCX_21.6111.6111.1	1	2.523	0.359	1	320.5	72.22222	R.EADDIVNWLK.K
PDIA1_MOUSE	MK_SCX_21.6114.6114.2	2	2.688	0.509	1	691.7	77.77778	R.EADDIVNWLK.K
PDIA1_MOUSE	MK_SCX_23.6258.6258.2	2	2.859	0.225	1	496.5	92.85714	R.ILEFFGLK.K
PDIA1_MOUSE	MK_SCX_32.6569.6569.2	2	5.723	0.562	1	1243.3	75	K.HNQLPLVIEFTEQTAPK.I
PDIA1_MOUSE	MK_SCX_32.6591.6591.3	3	5.721	0.528	1	1512.8	56.25	K.HNQLPLVIEFTEQTAPK.I
PDIA1_MOUSE	MK_SCX_33.4995.4995.3	3	3.489	0.334	1	983	61.363636	K.YQLDKDGVVLFK.K
PDIA1_MOUSE	MK_SCX_33.5003.5003.2	2	4.221	0.324	1	1596.4	86.36364	K.YQLDKDGVVLFK.K

PDIA1_MOUSE	MK_SCX_33.6367.6367.3	3	3.894	0.436	1	1082	36.904762	R.LITLEEEMTKYKPESDELTAEK.I
PDIA1_MOUSE	MK_SCX_34.3719.3719.2	2	3.271	0.324	1	426	61.538464	K.FDEGRNNFEGEITK.E
PDIA1_MOUSE	MK_SCX_36.11434.11434.3	3	4.037	0.488	1	679.2	32.954548	K.LLDFIKHNQLPLVIEFTEQTAPK.I
PDIA1_MOUSE	MK_SCX_37.5844.5844.2	2	2.369	0.328	1	666.3	81.25	R.ILEFFGLKK.E
PDIA1_MOUSE	MK_SCX_39.5972.5972.3	3	3.779	0.56	1	796.8	36.842106	K.MDSTANEVEAVKVHSFPTLK.F
PDIA1_MOUSE	MK_SCX_40.5777.5777.2	2	2.835	0.527	1	1033.7	81.25	K.THILLFLPK.S
PDIA1_MOUSE	MK_SCX_46.3805.3805.2	2	4.225	0.458	1	1908.7	71.42857	K.LGETYKDHENIIIAK.M
PDIA1_MOUSE	MK_SCX_46.3813.3813.3	3	4.928	0.547	1	1856	57.14286	K.LGETYKDHENIIIAK.M
PDIA1_MOUSE	MK_SCX_49.3907.3907.2	2	3.431	0.247	1	833.5	60.000004	K.FDEGRNNFEGEITKEK.L
PDIA1_MOUSE	MK_SCX_50.3949.3949.3	3	3.904	0.457	1	955.1	48.333332	K.IKPHLM*SQEVPEDWDK.Q
PDIA1_MOUSE	MK_SCX_50.4375.4375.3	3	4.108	0.451	1	856.8	54.166668	K.YQLDKDGVVLFKK.F
PDIA1_MOUSE	MK_SCX_50.4387.4387.2	2	4.703	0.435	1	2578.1	87.5	K.YQLDKDGVVLFKK.F
PDIA1_MOUSE	MK_SCX_50.4451.4451.3	3	4.913	0.405	1	981.7	50	K.IKPHLMSQEVPEDWDK.Q
PDIA1_MOUSE	MK_SCX_50.4477.4477.2	2	4.758	0.607	1	1243.6	76.666664	K.IKPHLMSQEVPEDWDK.Q
PDIA1_MOUSE	MK_SCX_50.7033.7033.3	3	4.15	0.448	1	599.6	30.681818	K.QLAPIWDKLGETYKDHENIIIAK.M
PDIA1_MOUSE	MK_SCX_53.4236.4236.3	3	4.513	0.464	1	857.9	43.421055	K.IKPHLM*SQEVPEDWDKQPVK.V
PDIA1_MOUSE	MK_SCX_53.4646.4646.3	3	5.131	0.564	1	655.5	40.789474	K.IKPHLMSQEVPEDWDKQPVK.V
PDIA1_MOUSE	MK_SCX_53.4675.4675.2	2	4.891	0.621	1	1427.7	60.526318	K.IKPHLMSQEVPEDWDKQPVK.V
PDIA3_MOUSE	MK_SCX_16.6196.6196.2	2	5.382	0.586	1	2005.5	87.5	K.DLLTAYYDVDYEK.N
PDIA3_MOUSE	MK_SCX_18.3972.3972.2	2	5.132	0.536	1	1774.7	85.71429	K.M*DATANDVPSPYEVK.G
PDIA3_MOUSE	MK_SCX_18.4221.4221.2	2	4.456	0.428	1	842.4	75	K.MDATANDVPSPYEVK.G
PDIA3_MOUSE	MK_SCX_19.8159.8159.2	2	5.629	0.425	1	2513.9	75	K.ALEQFLQEYFDGNLK.R
PDIA3_MOUSE	MK_SCX_20_1.3432.3432.2	2	2.872	0.276	1	352.5	66.66667	K.SEPIPESNEGPVK.V
PDIA3_MOUSE	MK_SCX_20_1.5365.5365.2	2	2.601	0.281	1	1229.1	93.75	K.DASVVGFFR.D
PDIA3_MOUSE	MK_SCX_21.6877.6877.3	3	3.469	0.273	1	820.6	50	R.ELNDFISYLQR.E
PDIA3_MOUSE	MK_SCX_21.6898.6898.2	2	4.222	0.466	1	1042.2	85	R.ELNDFISYLQR.E
PDIA3_MOUSE	MK_SCX_2201.3234.3234.2	2	2.759	0.223	1	380.2	80	R.LAPEYEAAATR.L
PDIA3_MOUSE	MK_SCX_2201.5550.5550.2	2	3.179	0.451	1	923.8	72.72727	K.GFPTIYFSPANK.K
PDIA3_MOUSE	MK_SCX_23.3461.3461.2	2	3.34	0.296	1	1126.9	93.75	K.FVM*QEEFSR.D
PDIA3_MOUSE	MK_SCX_23.4042.4042.2	2	3.491	0.407	1	978.4	93.75	K.FVMQEEFSR.D
PDIA3_MOUSE	MK_SCX_24.4114.4114.2	2	2.285	0.356	1	657	77.77778	K.YGVSGYPTLK.I
PDIA3_MOUSE	MK_SCX_27.4332.4332.3	3	4.862	0.532	1	968	38.095238	K.VDCTANTNTCNKYGVSGYPTLK.I
PDIA3_MOUSE	MK_SCX_28.5109.5109.2	2	4.03	0.563	1	1140.2	77.27273	R.DLFSDGHSEFLK.A
PDIA3_MOUSE	MK_SCX_29.3389.3389.2	2	3.593	0.38	1	676.9	69.230774	R.EATNPPIIQEEKPK.K
PDIA3_MOUSE	MK_SCX_29.4217.4217.2	2	3.164	0.46	1	898.1	50	K.VDCTANTNTCNKYGVSGYPTLK.I
PDIA3_MOUSE	MK_SCX_30.7668.7668.3	3	4.784	0.453	1	1206.8	53.333336	K.ALEQFLQEYFDGNLKR.Y
PDIA3_MOUSE	MK_SCX_30.7810.7810.2	2	5.825	0.464	1	2217	73.333336	K.ALEQFLQEYFDGNLKR.Y
PDIA3_MOUSE	MK_SCX_33.6165.6165.2	2	5.097	0.553	1	2444	92.30769	K.FISDKDASVVGFFR.D
PDIA3_MOUSE	MK_SCX_33.6195.6195.3	3	3.657	0.42	1	963.4	48.076923	K.FISDKDASVVGFFR.D
PDIA3_MOUSE	MK_SCX_35.3399.3399.3	3	3.921	0.155	1	1573.1	60.000004	K.LSKDPNIVIAK.M
PDIA3_MOUSE	MK_SCX_35.5329.5329.2	2	3.301	0.375	1	491.3	66.66667	K.GFPTIYFSPANKK.L
PDIA3_MOUSE	MK_SCX_38.11656.11656.3	3	3.29	0.48	1	416.2	31.944445	R.DGKALEQFLQEYFDGNLKR.Y
PDIA3_MOUSE	MK_SCX_40.4214.4214.3	3	3.811	0.143	1	918.4	50	R.FAHTNIESLVK.E
PDIA3_MOUSE	MK_SCX_40.4262.4262.2	2	3.603	0.495	1	897.5	85	R.FAHTNIESLVK.E
PDIA3_MOUSE	MK_SCX_41.7110.7110.3	3	4.107	0.464	1	1213.7	37.5	R.KTFSHELSDFSLESTTGEVPVVAIR.T
PDIA3_MOUSE	MK_SCX_42.3189.3189.3	3	3.502	0.337	1	1068.1	56.81818	K.RLAPEYEAAATR.L
PDIA3_MOUSE	MK_SCX_42.3206.3206.2	2	3.644	0.427	1	758.9	81.818184	K.RLAPEYEAAATR.L
PDIA3_MOUSE	MK_SCX_50.3677.3677.3	3	3.324	0.435	1	838.2	43.75	K.KQAGPASVPLRTEEEFK.K
PDIA3_MOUSE	MK_SCX_50.6783.6783.3	3	3.141	0.362	1	751.3	29.347824	K.EYDDNGEGITIFRPLHLANKFEDK.T
PDIA3_MOUSE	MK_SCX_51.4674.4674.2	2	4.679	0.49	1	2310.6	85.71429	K.FLDAGHKLNFAVASR.K
PDIA3_MOUSE	MK_SCX_51.5589.5589.3	3	3.821	0.559	1	836.3	50	K.KFISDKDASVVGFFR.D

PDIA3_MOUSE	MK_SCX_52.5331.5331.2	2	5.47	0.519	1	2965.2	89.28571	K.KFISDKDASVVGFFR.D
PDIA3_MOUSE	MK_SCX_52.6296.6296.2	2	4.865	0.375	1	492.8	53.125	K.KYEGGRELNDFISYLQR.E
PDIA3_MOUSE	MK_SCX_52.6379.6379.3	3	4.502	0.505	1	584.3	40.625	K.KYEGGRELNDFISYLQR.E
PDIA3_MOUSE	MK_SCX_57.10985.10985.3	3	3.7	0.278	1	1130.6	46.666668	K.KFLDAGHKLNFAVASR.K
PDIA4_MOUSE	MK_SCX_16.6339.6339.2	2	4.069	0.394	1	566	58.333332	R.EVSQPDWTPPPEVTLSLTK.D
PDIA4_MOUSE	MK_SCX_19.3536.3536.2	2	4.729	0.56	1	1473.4	76.92308	K.MDATANDITNDQYK.V
PDIA4_MOUSE	MK_SCX_19.4346.4346.2	2	3.65	0.45	1	755.7	73.07692	K.FAM*EPEEFDSDTLR.E
PDIA4_MOUSE	MK_SCX_19.4498.4498.2	2	3.128	0.282	1	526.2	56.666668	K.YGIVDYM*IEQSGPPSK.E
PDIA4_MOUSE	MK_SCX_19.5117.5117.2	2	3.9	0.403	1	501.7	73.07692	K.FAMEPEEFDSDTLR.E
PDIA4_MOUSE	MK_SCX_20_1.2735.2735.2	2	3.735	0.431	1	1078	90	K.VDATEQTDLAK.R
PDIA4_MOUSE	MK_SCX_20_1.3455.3455.2	2	4.375	0.584	1	1894.3	90.909096	K.IDATSASM*LASK.F
PDIA4_MOUSE	MK_SCX_20_1.3823.3823.2	2	4.02	0.565	1	2152	90.909096	K.IDATSASMLASK.F
PDIA4_MOUSE	MK_SCX_2201.1756.1756.2	2	2.426	0.33	1	494.3	85.71429	R.TQEEIVAK.V
PDIA4_MOUSE	MK_SCX_2201.4398.4398.2	2	3.281	0.436	1	1613.8	88.88889	K.FDVSGYPTIK.I
PDIA4_MOUSE	MK_SCX_2201.4398.4398.2	2	3.281	0.436	1	1613.8	88.88889	K.FDVSGYPTIK.I
PDIA4_MOUSE	MK_SCX_2201.5804.5804.2	2	5.255	0.655	1	2738.6	64.28571	K.DLGLSESGEDVNAAILDESGKK.F
PDIA4_MOUSE	MK_SCX_23.9197.9197.2	2	3.894	0.627	1	538.3	45	K.FAMEPEEFDSDTLREFVTAFK.K
PDIA4_MOUSE	MK_SCX_24.8988.8988.3	3	4.643	0.42	1	462.1	29.761904	K.YGIVDYMIEQSGPPSKEILTLK.Q
PDIA4_MOUSE	MK_SCX_24.9186.9186.2	2	4.256	0.551	1	1592.6	57.14286	K.YGIVDYMIEQSGPPSKEILTLK.Q
PDIA4_MOUSE	MK_SCX_28.6326.6326.3	3	4.488	0.471	1	647.4	40	K.VREVSQPDWTPPPEVTLSLTK.D
PDIA4_MOUSE	MK_SCX_28.6361.6361.2	2	5.555	0.683	1	775	55	K.VREVSQPDWTPPPEVTLSLTK.D
PDIA4_MOUSE	MK_SCX_29.3899.3899.3	3	3.977	0.48	1	996.5	48.333332	K.IASTLKDNDPPIAVAK.I
PDIA4_MOUSE	MK_SCX_30.3756.3756.2	2	4.201	0.521	1	675.6	63.333332	K.IASTLKDNDPPIAVAK.I
PDIA4_MOUSE	MK_SCX_40.4384.4384.3	3	3.835	0.32	1	1148.8	50	K.RFDVSGYPTLK.I
PDIA4_MOUSE	MK_SCX_41.4267.4267.2	2	3.495	0.363	1	1285.6	90	K.RFDVSGYPTLK.I
PDIA4_MOUSE	MK_SCX_41.6100.6100.3	3	5.631	0.643	1	998.3	43.75	R.FHVMDVQGSTEASAIKDYVVK.H
PDIA4_MOUSE	MK_SCX_47.3456.3456.3	3	4.11	0.456	1	655.9	34.72222	K.KGQAVDYDGSRTQEEIVAK.V
PDIA6_MOUSE	MK_SCX_15.9538.9538.2	2	5.37	0.571	1	531.7	50	R.ALDLFSDNAPPPELLEIINEDIAK.K
PDIA6_MOUSE	MK_SCX_19.5220.5220.2	2	5.162	0.566	1	2725.8	85.71429	K.LAAVDATVNQVLASR.Y
PDIA6_MOUSE	MK_SCX_19.6888.6888.2	2	4.208	0.472	1	1970.9	80.769226	R.TGEAIVDAALSALR.Q
PDIA6_MOUSE	MK_SCX_20_1.4816.4816.2	2	4.875	0.441	1	1456.9	68.75	R.GSTAPVGGGSFPTITPR.E
PDIA6_MOUSE	MK_SCX_23.5722.5722.2	2	4.383	0.409	1	1379.3	75	K.GSFSEQGINEFLR.E
PDIA6_MOUSE	MK_SCX_28.5336.5336.2	2	5.127	0.51	1	1249.7	73.52941	K.NLEPEWAAAATEVKEQTK.G
PDIA6_MOUSE	MK_SCX_41.5067.5067.3	3	6.762	0.616	1	3343.9	47.916664	K.VGAVNADKHQSLGGQYGVQGFPTIK.I
PDIA6_MOUSE	MK_SCX_53.2970.2970.3	3	3.103	0.372	1	437.7	36.666668	K.IFGANKNKPEDYQGGR.T
PDIP2_MOUSE	MK_SCX_23.6063.6063.2	2	2.859	0.313	1	653.8	72.22222	R.GVVLFPWQAR.L
PDIP2_MOUSE	MK_SCX_25.9083.9083.3	3	5.499	0.557	1	820.8	31.818182	R.ALYAIPGLDYVSHEDILPYTSTDQVPIQHELFER.F
PDIP3_MOUSE	MK_SCX_33.4331.4331.3	3	3.942	0.519	1	910.6	38.157894	R.RGNPASSNPPAEVDPDTVLR.A
PDIP3_MOUSE	MK_SCX_37.3370.3370.3	3	3.821	0.445	1	1194.5	52.083332	R.VGIQHSLVNQPAR.T
PDK11_MOUSE	MK_SCX_2201.6648.6648.3	3	6.161	0.634	1	2199.3	44.56522	R.AGLQEGDQVLAVNDVDFQDIEHSK.A
PDK11_MOUSE	MK_SCX_23.8516.8516.3	3	5.766	0.618	1	1065.7	33	R.IPYDDYPVVFLPAYENPPAWIPPHER.V
PDK11_MOUSE	MK_SCX_44.4163.4163.3	3	3.551	0.389	1	1454.9	59.090908	K.KPPGAQLGFNIR.G
PDK3_MOUSE	MK_SCX_29.5682.5682.3	3	3.216	0.464	1	607.7	33.82353	R.LFNYMYSTAPRPSLEPTR.A
PDK3_MOUSE	MK_SCX_52.3301.3301.3	3	5.301	0.489	1	1630.8	45.833336	R.HYKTTPEADDWSNPSSEPR.D
PDLI1_MOUSE	MK_SCX_32.4699.4699.3	3	3.752	0.427	1	487.1	42.857143	R.LVGGKDFEQPLAISR.V
PDLI2_MOUSE	MK_SCX_17.4538.4538.2	2	4.193	0.425	1	777.2	50	R.SQTASPGQTNGEGSLEVLATR.F
PDLI2_MOUSE	MK_SCX_18.5387.5387.2	2	4.907	0.55	1	749.4	60.000004	R.GGTPAFVPSSLSSQASLPTSR.A
PDLI2_MOUSE	MK_SCX_19.5851.5851.2	2	3.769	0.342	1	1791.6	76.92308	R.FSSLDLEEDSEVFK.M
PDLI2_MOUSE	MK_SCX_21.4286.4286.2	2	3.736	0.488	1	1963.3	90	R.LLQEALEAEER.G
PDLI2_MOUSE	MK_SCX_41.7086.7086.3	3	5.669	0.523	1	1664.4	31.25	R.GKAEAADLRPGDIIVAINGQSAENMLHAEAQSK.I
PDLI5_MOUSE	MK_SCX_17.3999.3999.2	2	5.185	0.621	1	981.7	57.14286	R.SSGTGASVGPPQPSDQDTLVQR.A

PDLI5_MOUSE	MK_SCX_24.13853.13853.3	3	3.398	0.285	1	431.3	27.173912	R.IGDVVLSIDGISAQGMTHLEAQNK.I
PDLI5_MOUSE	MK_SCX_31.6029.6029.3	3	3.238	0.288	1	992.8	43.75	R.LQGGKDFNMPLTISSLK.D
PDLI5_MOUSE	MK_SCX_31.6100.6100.2	2	4.227	0.547	1	1324.2	68.75	R.LQGGKDFNMPLTISSLK.D
PDLI5_MOUSE	MK_SCX_34.3851.3851.2	2	2.826	0.202	1	726.8	85	R.LIEDTEDWRPR.T
PDLI5_MOUSE	MK_SCX_42.5551.5551.3	3	5.301	0.508	1	1549.8	45	R.LQGGKDFNMPLTISSLKDGGK.A
PDLI5_MOUSE	MK_SCX_42.5589.5589.2	2	5.121	0.533	1	2046.8	67.5	R.LQGGKDFNMPLTISSLKDGGK.A
PDLI5_MOUSE	MK_SCX_49.3612.3612.3	3	3.476	0.43	1	535.5	39.285713	R.NTEFYHIPTHSDASK.K
PDLI5_MOUSE	MK_SCX_49.3620.3620.2	2	5.113	0.619	1	1130.3	78.57143	R.NTEFYHIPTHSDASK.K
PDPK1_MOUSE	MK_SCX_20_1.3653.3653.2	2	4.277	0.558	1	2451.1	84.375	R.SQTEPGSSPGIPSGVSR.Q
PDXK_MOUSE	MK_SCX_17.8297.8297.2	2	4.892	0.335	1	1261.4	65	K.VVPVADIITPNQFEAELLSGR.K
PDXK_MOUSE	MK_SCX_23.8534.8534.2	2	5.481	0.537	1	1569.6	61.363636	R.DKVVPVADIITPNQFEAELLSGR.K
PDXK_MOUSE	MK_SCX_23.8555.8555.3	3	7.381	0.624	1	3550.2	50	R.DKVVPVADIITPNQFEAELLSGR.K
PDXK_MOUSE	MK_SCX_31.3579.3579.3	3	4.807	0.554	1	1729.3	53.125	K.AEAGEGQKPSPAQLELR.M
PDXK_MOUSE	MK_SCX_31.3600.3600.2	2	4.502	0.509	1	649.7	65.625	K.AEAGEGQKPSPAQLELR.M
PDXK_MOUSE	MK_SCX_8.6422.6422.2	2	2.115	0.175	1	302.5	37.5	K.QQNSRLVYVCDPVM*GDK.W
PDZ1I_MOUSE	MK_SCX_24.4142.4142.2	2	3.552	0.495	1	1614.2	81.818184	K.NAYENVLEEEGR.V
PDZ1I_MOUSE	MK_SCX_36.4274.4274.2	2	3.268	0.41	1	413.1	57.692307	K.NAYENVLEEEGRVR.S
PDZ1I_MOUSE	MK_SCX_52.3370.3370.2	2	5.89	0.551	1	1997.4	78.125	R.SSEHKNAYENVLEEEGR.V
PDZ1I_MOUSE	MK_SCX_52.3373.3373.3	3	5.002	0.485	1	935	40.625	R.SSEHKNAYENVLEEEGR.V
PDZ1I_MOUSE	MK_SCX_56.3817.3817.3	3	3.396	0.558	1	509.3	40.27778	R.SSEHKNAYENVLEEEGRVR.S
PDZK1_MOUSE	MK_SCX_13.6191.6191.2	2	2.373	0.297	1	469.1	50	R.FSPLLYCQSQELPNGSVK.E
PDZK1_MOUSE	MK_SCX_15.6164.6164.2	2	4.361	0.475	1	720.4	50	K.AGLENEDVIIEVNGENVQEEPYDR.V
PDZK1_MOUSE	MK_SCX_17.3997.3997.2	2	3.005	0.478	1	487.5	69.230774	K.DIEPGSPAEAAGLK.N
PDZK1_MOUSE	MK_SCX_19.4466.4466.2	2	3.998	0.564	1	1996.4	82.14286	K.GVYLTDIM*PQGVAM*K.A
PDZK1_MOUSE	MK_SCX_19.4930.4930.2	2	4.747	0.496	1	2000	78.57143	K.GVYLTDIM*PQGVAMK.A
PDZK1_MOUSE	MK_SCX_19.5257.5257.2	2	5.195	0.606	1	1899.5	68.75	K.SGNSVTLLVLDGDSYEK.A
PDZK1_MOUSE	MK_SCX_19.5974.5974.2	2	4.998	0.606	1	1950.7	82.14286	K.GVYLTDIMPQGVAMK.A
PDZK1_MOUSE	MK_SCX_2201.2330.2330.2	2	2.973	0.35	1	554.6	72.22222	R.VIEEGSPAEK.A
PDZK1_MOUSE	MK_SCX_2201.4481.4481.2	2	2.281	0.355	1	827.4	83.33333	K.EGNSFGFSLK.T
PDZK1_MOUSE	MK_SCX_24.4805.4805.2	2	3.039	0.42	1	573.5	90	K.QEGQNYGFFLR.I
PDZK1_MOUSE	MK_SCX_25.4083.4083.2	2	2.583	0.58	1	902.1	87.5	K.MAYSYFQAK.K
PDZK1_MOUSE	MK_SCX_25.4519.4519.2	2	2.098	0.164	1	793.1	77.77778	K.GSNGYGFYLR.A
PDZK1_MOUSE	MK_SCX_26.8475.8475.3	3	3.601	0.297	1	1060.4	38.095238	K.GGDQTTLLVLDKEAESIYSLAR.F
PDZK1_MOUSE	MK_SCX_27.5853.5853.3	3	5.626	0.476	1	1269.1	30.555555	K.AGVLADDHLIEVNGENVENASHEEVVEK.V
PDZK1_MOUSE	MK_SCX_28.4726.4726.3	3	3.695	0.333	1	857	38.88889	K.GQIIKDIEPGSPAEAAGLK.N
PDZK1_MOUSE	MK_SCX_28.4729.4729.2	2	5.762	0.548	1	1323.7	72.22222	K.GQIIKDIEPGSPAEAAGLK.N
PDZK1_MOUSE	MK_SCX_29.4268.4268.2	2	5.21	0.629	1	945.7	75	K.SVEALDHDGVVEM*IR.K
PDZK1_MOUSE	MK_SCX_29.4279.4279.3	3	3.958	0.488	1	1142.8	51.785713	K.SVEALDHDGVVEM*IR.K
PDZK1_MOUSE	MK_SCX_29.4910.4910.3	3	4.339	0.459	1	1867.1	48.61111	K.KIPIVSSM*AEPLVAGPDEK.G
PDZK1_MOUSE	MK_SCX_29.4934.4934.2	2	5.116	0.606	1	1627.2	63.88889	K.KIPIVSSM*AEPLVAGPDEK.G
PDZK1_MOUSE	MK_SCX_29.5032.5032.3	3	4.255	0.508	1	1496.8	53.571426	K.SVEALDHDGVVEMIR.K
PDZK1_MOUSE	MK_SCX_29.6054.6054.2	2	5.689	0.686	1	2021.2	69.44444	K.KIPIVSSMAEPLVAGPDEK.G
PDZK1_MOUSE	MK_SCX_29.6078.6078.3	3	4.591	0.54	1	1938.6	48.61111	K.KIPIVSSMAEPLVAGPDEK.G
PDZK1_MOUSE	MK_SCX_30.4965.4965.2	2	5.407	0.523	1	2209.1	82.14286	K.SVEALDHDGVVEMIR.K
PDZK1_MOUSE	MK_SCX_31.4883.4883.2	2	4.07	0.375	1	1017.9	70.83333	K.EDDSYGFHLNAIR.G
PDZK1_MOUSE	MK_SCX_31.6421.6421.3	3	5.377	0.598	1	1433.5	35.344826	K.GQIIKDIEPGSPAEAAGLKNNDLVVAVNGK.S
PDZK1_MOUSE	MK_SCX_32.15591.15591.3	3	3.168	0.392	1	537.6	50	R.IMFLLVDKETAR.C
PDZK1_MOUSE	MK_SCX_32.4404.4404.2	2	3.902	0.606	1	366	34.615387	K.EGPAPIPAPLEATGSEPTEDAEGHKPK.L
PDZK1_MOUSE	MK_SCX_32.5285.5285.2	2	3.465	0.341	1	709.7	77.27273	R.IMFLLVDKETAR.C
PDZK1_MOUSE	MK_SCX_33.4379.4379.3	3	3.776	0.43	1	1674.7	51.666664	K.KGVYLTDIM*PQGVAM*K.A
PDZK1_MOUSE	MK_SCX_33.4392.4392.2	2	5.372	0.562	1	693.2	70	K.KGVYLTDIM*PQGVAM*K.A

PDZK1_MOUSE	MK_SCX_33.4896.4896.2	2	5.371	0.343	1	1794.6	86.666664	K.KGVYLTDIM*PQGVAMK.A
PDZK1_MOUSE	MK_SCX_33.4951.4951.3	3	3.884	0.479	1	1338.9	53.333336	K.KGVYLTDIM*PQGVAMK.A
PDZK1_MOUSE	MK_SCX_33.5140.5140.3	3	3.749	0.447	1	887.6	36.764706	R.KSGNSVTLLVLDGDSYEK.A
PDZK1_MOUSE	MK_SCX_33.5141.5141.2	2	6.254	0.582	1	3451.4	82.35294	R.KSGNSVTLLVLDGDSYEK.A
PDZK1_MOUSE	MK_SCX_33.5196.5196.3	3	3.389	0.354	1	1053.3	50	K.KGVYLTDIMPQGVAM*K.A
PDZK1_MOUSE	MK_SCX_33.5209.5209.2	2	5.092	0.418	1	1330.1	80	K.KGVYLTDIMPQGVAM*K.A
PDZK1_MOUSE	MK_SCX_33.6211.6211.3	3	3.831	0.432	1	1210.8	51.666664	K.KGVYLTDIMPQGVAMK.A
PDZK1_MOUSE	MK_SCX_33.6307.6307.2	2	6.006	0.489	1	3117.3	86.666664	K.KGVYLTDIMPQGVAMK.A
PDZK1_MOUSE	MK_SCX_35.12578.12578.3	3	3.591	0.428	1	975.3	37.5	R.INGVFVDKEEHAQVVELVR.K
PDZK1_MOUSE	MK_SCX_35.4023.4023.2	2	3.002	0.396	1	325.7	75	R.KGGDQTTLLVLDK.E
PDZK1_MOUSE	MK_SCX_38.4706.4706.3	3	4.379	0.553	1	1180.8	53.846157	K.LSKQEGQNYGFFLR.I
PDZK1_MOUSE	MK_SCX_38.4867.4867.2	2	4.311	0.484	1	2164.9	84.61539	K.LSKQEGQNYGFFLR.I
PDZK1_MOUSE	MK_SCX_39.14459.14459.3	3	6.085	0.616	1	2143.8	42.045452	R.KGGDQTTLLVLDKEAESIYSLAR.F
PDZK1_MOUSE	MK_SCX_41.7971.7971.2	2	6.06	0.637	1	2067.2	59.090908	R.KGGDQTTLLVLDKEAESIYSLAR.F
PDZK1_MOUSE	MK_SCX_44.4067.4067.3	3	3.191	0.479	1	409	38.333332	K.SVEALDHDGVVEM*IRK.G
PDZK1_MOUSE	MK_SCX_44.4670.4670.2	2	5.22	0.654	1	1642.3	73.333336	K.SVEALDHDGVVEMIRK.G
PDZK1_MOUSE	MK_SCX_44.4677.4677.3	3	4.89	0.404	1	645.3	45	K.SVEALDHDGVVEMIRK.G
PDZK1_MOUSE	MK_SCX_46.10580.10580.3	3	5.132	0.559	1	1597.2	53.333336	R.LLKEDDSYGFHLNAIR.G
PDZK1_MOUSE	MK_SCX_46.5080.5080.2	2	5.743	0.537	1	2024.8	76.666664	R.LLKEDDSYGFHLNAIR.G
PDZK1_MOUSE	MK_SCX_47.5550.5550.3	3	4.149	0.306	1	523.4	24.13793	R.EAALNDKKPGPGMNGAVEPCAQPRLCYLVK.E
PDZK1_MOUSE	MK_SCX_49.3698.3698.3	3	3.817	0.381	1	1158.9	45	K.AVKNQVDLKELDQSQR.E
PDZK1_MOUSE	MK_SCX_49.3715.3715.2	2	4.797	0.598	1	1952.7	76.666664	K.AVKNQVDLKELDQSQR.E
PDZK1_MOUSE	MK_SCX_51.2335.2335.2	2	2.962	0.333	1	1111.2	85	R.IEKDTDGHLIR.V
PDZK1_MOUSE	MK_SCX_51.5189.5189.3	3	5.189	0.56	1	1494	31.818182	K.KIPIVSSMAEPLVAGPDEKGETSAESEHDAHPAK.D
PEA15_MOUSE	MK_SCX_21.3098.3098.2	2	3.521	0.471	1	1397.1	88.88889	K.ISEEEELDTK.L
PEA15_MOUSE	MK_SCX_42.7669.7669.3	3	3.171	0.342	1	1098.4	39.0625	K.LDKDNLSYIEHIFEISR.R
PEBP1_MOUSE	MK_SCX_13.7272.7272.2	2	3.576	0.553	1	715.1	50	K.YNLGAPVAGTCYQAEWDDYVPK.L
PEBP1_MOUSE	MK_SCX_18.5160.5160.2	2	5.353	0.501	1	2428.2	88.46153	K.LYTLVLTDPDAPSR.K
PEBP1_MOUSE	MK_SCX_19.4458.4458.2	2	5.053	0.561	1	1944.1	91.66667	R.VDYAGVTVDELGK.V
PEBP1_MOUSE	MK_SCX_19.4486.4486.1	1	2.755	0.349	1	674	70.83333	R.VDYAGVTVDELGK.V
PEBP1_MOUSE	MK_SCX_25.5581.5581.2	2	5.301	0.675	1	916.9	46	K.GNDISSGTVLSDYVGSGPPSGTGLHR.Y
PEBP1_MOUSE	MK_SCX_25.5607.5607.3	3	5.621	0.553	1	1273.7	42	K.GNDISSGTVLSDYVGSGPPSGTGLHR.Y
PEBP1_MOUSE	MK_SCX_25.5767.5767.3	3	3.854	0.449	1	709	34.090908	K.VLTPTQVM*NRPSSISWDGLDPGK.L
PEBP1_MOUSE	MK_SCX_25.6487.6487.3	3	5.172	0.578	1	850.5	35.227272	K.VLTPTQVMNRPSSISWDGLDPGK.L
PEBP1_MOUSE	MK_SCX_25.6536.6536.2	2	4.365	0.436	1	525.8	45.454548	K.VLTPTQVMNRPSSISWDGLDPGK.L
PEBP1_MOUSE	MK_SCX_25.9314.9314.3	3	6.163	0.538	1	1234.4	29.86111	K.VLTPTQVMNRPSSISWDGLDPGKLYTLVLTDPDAPSR.K
PEBP1_MOUSE	MK_SCX_40.4558.4558.3	3	3.691	0.464	1	875.9	41.17647	K.LYTLVLTDPDAPSRKDPK.F
PECA1_MOUSE	MK_SCX_25.5272.5272.3	3	5	0.447	1	1027	38.095238	K.SDFQTLEVTSNDPATFTDKPTR.D
PECI_MOUSE	MK_SCX_16.9271.9271.2	2	4.898	0.473	1	840.4	50	R.EAWAQGLVTEVFPESTFETEVWTR.L
PECI_MOUSE	MK_SCX_17.4190.4190.2	2	4.474	0.568	1	1459.5	86.36364	K.DILVTSEDGITK.I
PECI_MOUSE	MK_SCX_21.4799.4799.2	2	5.103	0.431	1	1343	69.230774	R.ASQQDFENALNQVK.L
PECI_MOUSE	MK_SCX_23.6967.6967.2	2	4.276	0.342	1	1539.4	81.818184	K.WDAWNALGSLPK.E
PECI_MOUSE	MK_SCX_30.3754.3754.3	3	3.834	0.41	1	574.2	42.857143	R.ESKDILVTSEDGITK.I
PECI_MOUSE	MK_SCX_30.3755.3755.2	2	4.168	0.492	1	1499.9	78.57143	R.ESKDILVTSEDGITK.I
PECI_MOUSE	MK_SCX_38.7270.7270.2	2	2.71	0.388	1	333.9	50	K.AKWDAWNALGSLPK.E
PECI_MOUSE	MK_SCX_48.4970.4970.3	3	6.426	0.597	1	1398.8	47.61905	R.QNYVDLVSSLSSSSEAPSQGKR.G
PECI_MOUSE	MK_SCX_50.3563.3563.3	3	4.831	0.538	1	1230.5	45.3125	K.ARESKDILVTSEDGITK.I
PECR_MOUSE	MK_SCX_13.5378.5378.2	2	5.278	0.53	1	810.6	66.66667	R.ASLPPSSSAEVSAIQCNIR.K
PECR_MOUSE	MK_SCX_16.6705.6705.2	2	4.054	0.409	1	1373.8	52.380955	K.INFLVNNGGGQFM*APVEDITAK.G
PECR_MOUSE	MK_SCX_2201.3584.3584.2	2	5.02	0.619	1	2743.5	89.28571	K.NQVAVVTGGGTGIGK.A
PECR_MOUSE	MK_SCX_2201.3729.3729.2	2	3.144	0.315	1	1139.9	93.75	R.LTAAVDELR.A

PECR_MOUSE	MK_SCX_2201.4513.4513.2	2	3.617	0.464	1	2000	86.36364	K.TGQSYLAAGLLK.N
PECR_MOUSE	MK_SCX_24.4652.4652.2	2	4.678	0.521	1	2215	90.909096	K.SMALAWASSGVR.I
PECR_MOUSE	MK_SCX_25.7693.7693.3	3	3.848	0.371	1	445.2	32.291664	K.YGKINFLVNNGGGQFMAPVEDITAK.G
PECR_MOUSE	MK_SCX_53.4747.4747.2	2	2.966	0.289	1	1035.3	79.16667	R.KLDRLTAAVDELR.A
PECR_MOUSE	MK_SCX_53.4872.4872.3	3	4.398	0.45	1	1279.7	54.166668	R.KLDRLTAAVDELR.A
PEDF_MOUSE	MK_SCX_2201.10750.10750.3	3	4.706	0.548	1	542.2	27.586206	R.SSASPTGNVLLSPLSVATALSALSLGAEHR.T
PEDF_MOUSE	MK_SCX_34.5829.5829.2	2	2.078	0.177	1	385.9	54.545456	K.LQSLFESPDFSK.I
PEPL1_MOUSE	MK_SCX_14.9927.9927.3	3	3.613	0.418	1	950.1	24.285715	R.VTEELWQAALATLNPNPTDSCPLYLNCATVAALPSR.V
PEPL1_MOUSE	MK_SCX_2201.5764.5764.2	2	2.637	0.137	1	895.5	83.33333	K.ELGITPTIIR.D
PEX14_MOUSE	MK_SCX_24.4861.4861.2	2	2.989	0.39	1	673.3	87.5	K.IPSWQIPVK.S
PEX14_MOUSE	MK_SCX_25.8479.8479.2	2	2.006	0.25	1	309.7	46.42857	R.RGGDGQINEQVEKLR.R
PEX19_MOUSE	MK_SCX_17.11844.11844.2	2	3.849	0.527	1	1395.9	65.789474	K.FFQELFDSELASQATAEFEK.A
PEX19_MOUSE	MK_SCX_19.3409.3409.2	2	4.43	0.559	1	593.1	59.375	K.NATELQNSGM*SEEELM*K.A
PEX19_MOUSE	MK_SCX_42.5200.5200.3	3	4.994	0.517	1	2638.6	51.47059	K.AMKELAEEEPHLVEQFQK.L
PFD2_MOUSE	MK_SCX_21.4451.4451.2	2	4.99	0.445	1	1964.1	86.36364	K.IIETLSQQLQAK.G
PFD2_MOUSE	MK_SCX_2201.5329.5329.2	2	3.677	0.308	1	1550.1	76.92308	K.GAVSAEQVIAGFNR.L
PFD2_MOUSE	MK_SCX_44.4186.4186.3	3	4.641	0.53	1	838.2	44.11765	R.TVKEVLPALEGNKEQIQK.I
PFD3_MOUSE	MK_SCX_19.7731.7731.2	2	4.429	0.402	1	1848.3	83.33333	K.NLDSLEEDLDFLR.D
PFD3_MOUSE	MK_SCX_2201.9897.9897.3	3	5.188	0.434	1	1362.4	35.416664	K.NLDSLEEDLDFLRDQFTTTEVNM*AR.V
PFD5_MOUSE	MK_SCX_18.8950.8950.2	2	5.205	0.562	1	1857.4	70.588234	K.NQLDQEVEFLSTSIAQLK.V
PGAM1_MOUSE	MK_SCX_17.5984.5984.2	2	6.003	0.584	1	745.5	57.5	R.YADLTEDQLPSCESLKDTIAR.A
PGAM1_MOUSE	MK_SCX_17.5993.5993.3	3	5.314	0.571	1	1766.5	47.5	R.YADLTEDQLPSCESLKDTIAR.A
PGAM1_MOUSE	MK_SCX_19.7149.7149.2	2	4.348	0.472	1	981.6	80.769226	R.ALPFWNEEIVPQIK.E
PGAM1_MOUSE	MK_SCX_2201.1986.1986.2	2	2.386	0.131	1	1303.1	83.33333	K.AMEAVAAQGK.V
PGAM1_MOUSE	MK_SCX_2201.873.873.2	2	3.423	0.428	1	1332.8	94.44444	K.AM*EAVAAQGK.V
PGAM1_MOUSE	MK_SCX_23.13094.13094.3	3	4.674	0.554	1	441.7	25.961538	K.HLEGLSEEAIMELNLPTGIPIVYELDK.N
PGAM1_MOUSE	MK_SCX_24.4882.4882.2	2	4.209	0.618	1	428.4	52.499996	R.SYDVPPPPM*EPDHPFYSNISK.D
PGAM1_MOUSE	MK_SCX_24.5702.5702.2	2	3.401	0.613	1	395.7	50	R.SYDVPPPPMEPDHPFYSNISK.D
PGAM1_MOUSE	MK_SCX_29.4963.4963.2	2	5.87	0.409	1	1789.3	70.588234	R.FSGWYDADLSPAGHEEAK.R
PGAM1_MOUSE	MK_SCX_29.5023.5023.3	3	4.383	0.5	1	943.8	44.11765	R.FSGWYDADLSPAGHEEAK.R
PGAM1_MOUSE	MK_SCX_40.5019.5019.3	3	5.952	0.616	1	1129.8	39.285713	R.RSYDVPPPPM*EPDHPFYSNISK.D
PGAM1_MOUSE	MK_SCX_40.5742.5742.3	3	6.364	0.603	1	2456.5	45.238094	R.RSYDVPPPPMEPDHPFYSNISK.D
PGAM1_MOUSE	MK_SCX_45.4912.4912.2	2	5.415	0.462	1	1925.3	72.22222	R.FSGWYDADLSPAGHEEAKR.G
PGAM1_MOUSE	MK_SCX_46.4716.4716.3	3	3.949	0.429	1	861.7	41.666664	R.FSGWYDADLSPAGHEEAKR.G
PGAM1_MOUSE	MK_SCX_47.4828.4828.3	3	3.341	0.401	1	431.1	38.235294	K.NLKPIKPM*QFLGDEETVR.K
PGAM1_MOUSE	MK_SCX_47.5668.5668.2	2	4.792	0.548	1	905.6	64.70589	K.NLKPIKPMQFLGDEETVR.K
PGAM1_MOUSE	MK_SCX_47.5672.5672.3	3	4.023	0.545	1	1086.3	48.52941	K.NLKPIKPMQFLGDEETVR.K
PGAM1_MOUSE	MK_SCX_52.4973.4973.3	3	5.94	0.601	1	1759.6	39.130436	R.RSYDVPPPPMEPDHPFYSNISKDR.R
PGAM1_MOUSE	MK_SCX_53.3275.3275.2	2	4.977	0.592	1	2021.5	83.33333	R.HYGGLTGLNKAETAAK.H
PGAM1_MOUSE	MK_SCX_53.3277.3277.3	3	4.859	0.58	1	973.6	46.666668	R.HYGGLTGLNKAETAAK.H
PGAM1_MOUSE	MK_SCX_54.4737.4737.3	3	5.246	0.545	1	601.2	44.444447	K.NLKPIKPMQFLGDEETVRK.A
PGAM2_MOUSE	MK_SCX_20_1.3367.3367.2	2	3.526	0.432	1	439.2	68.181816	R.SFDTPPPPM*DEK.H
PGAM2_MOUSE	MK_SCX_2201.3431.3431.2	2	2.588	0.315	1	508.7	87.5	R.FLGDEETVR.K
PGAM2_MOUSE	MK_SCX_36.3587.3587.3	3	3.046	0.455	1	1121.6	54.166668	R.RSFDTPPPPMDEK.H
PGBM_MOUSE	MK_SCX_13.3313.3313.2	2	3.146	0.447	1	634.3	60.714287	K.EADQGAYTCEAMNSR.G
PGBM_MOUSE	MK_SCX_13.4976.4976.2	2	3.163	0.591	1	764.6	67.85714	R.LPAIEPSDQGQYLCR.A
PGBM_MOUSE	MK_SCX_16.4619.4619.3	3	3.574	0.318	1	615.4	23.275862	R.CTACEPGYTGQYCEQCAPGYEGDPNVQGGR.C
PGBM_MOUSE	MK_SCX_16.7720.7720.2	2	3.601	0.581	1	396.9	45.454548	K.FQGLDLNEELYLGGYPDYGAIPK.A
PGBM_MOUSE	MK_SCX_17.5235.5235.2	2	6.206	0.647	1	1006.1	69.047615	R.SPVISIEPPSSTVQQGQDASFK.C
PGBM_MOUSE	MK_SCX_19.8074.8074.3	3	3.616	0.525	1	505.3	19.375	R.YTLSYTAGPQGSPLLDPDIQITGNNIM*LVASQPALQGPERR.S
PGBM_MOUSE	MK_SCX_20_1.4049.4049.2	2	2.564	0.295	1	615.8	61.538464	R.SIEYSPQLEDASAK.E

PGBM_MOUSE	MK_SCX_2201.3105.3105.2	2	3.081	0.389	1	942.6	85	R.FDAGSGM*ATIR.H
PGBM_MOUSE	MK_SCX_2201.5368.5368.2	2	3.489	0.482	1	1160.8	80	R.YELGSGLAVLR.S
PGBM_MOUSE	MK_SCX_23.4307.4307.2	2	2.851	0.534	1	530.2	81.818184	R.AAGVPSASITWR.K
PGBM_MOUSE	MK_SCX_23.4698.4698.2	2	2.515	0.178	1	760.9	72.22222	K.AFAYLQVPER.V
PGBM_MOUSE	MK_SCX_23.8253.8253.2	2	2.801	0.288	1	472.2	44.736843	R.LDVEFKPLEPNGILLFSGGK.S
PGBM_MOUSE	MK_SCX_24.4809.4809.2	2	3.804	0.482	1	1312.5	94.44444	K.SPAYTLVWTR.L
PGBM_MOUSE	MK_SCX_25.5247.5247.3	3	3.079	0.3	1	440.4	25	R.ALEVEECRCPPGYVGLSCQDCAPGYTR.T
PGBM_MOUSE	MK_SCX_27.5655.5655.2	2	3.976	0.501	1	674.8	61.11111	R.GM*LEPVQKPDVILVGAGYR.L
PGBM_MOUSE	MK_SCX_27.7923.7923.3	3	3.753	0.366	1	981.7	33.333336	R.QLISTHFAPGDFQGFALVNPQR.N
PGBM_MOUSE	MK_SCX_28.5537.5537.3	3	6.768	0.553	1	1693	45.652176	R.LRSPVISIEPPSSTVQQGQDASFK.C
PGBM_MOUSE	MK_SCX_31.4248.4248.2	2	5.733	0.484	1	1568.9	70.588234	R.NSQLTGGFTVEPVHDGAR.L
PGBM_MOUSE	MK_SCX_31.4260.4260.3	3	3.554	0.541	1	887.5	39.705883	R.NSQLTGGFTVEPVHDGAR.L
PGBM_MOUSE	MK_SCX_35.3585.3585.2	2	2.833	0.337	1	840.5	85	K.ESLEVQIHPSR.S
PGBM_MOUSE	MK_SCX_36.3824.3824.3	3	3.762	0.342	1	996.3	50	R.IAHVELADAGQYR.C
PGBM_MOUSE	MK_SCX_45.6490.6490.3	3	5.234	0.533	1	2138.2	43.47826	R.HQGSELHFPSVQPSDAGVYICTCR.N
PGBM_MOUSE	MK_SCX_50.6228.6228.3	3	3.921	0.605	1	639	37.5	R.HPTPLALGQFHTVTLLR.S
PGES2_MOUSE	MK_SCX_18.7035.7035.2	2	3.28	0.526	1	603.2	55.555557	R.SAAQLPLSNSLQLTLYQYK.T
PGES2_MOUSE	MK_SCX_19.6975.6975.2	2	4.463	0.462	1	2278.5	87.5	R.VMEGLEAFDDLMR.H
PGES2_MOUSE	MK_SCX_2201.2863.2863.2	2	2.827	0.327	1	1192.1	94.44444	K.FGAVEAAM*AK.Y
PGES2_MOUSE	MK_SCX_25.8219.8219.3	3	5.497	0.591	1	2222.9	40.384613	R.KVPILVAQEGDSLQQLNDSSVIISALK.T
PGES2_MOUSE	MK_SCX_26.7863.7863.3	3	4.158	0.366	1	1230	37.5	R.AFLDFHSLPYQVVEVNPVR.R
PGES2_MOUSE	MK_SCX_39.9333.9333.3	3	3.776	0.292	1	803.5	36.842106	R.AFLDFHSLPYQVVEVNPVRR.T
PGK1_MOUSE	MK_SCX_14.8211.8211.2	2	4.92	0.529	1	891.7	42	K.DCVGPEVENACANPAAGTVILLENLR.F
PGK1_MOUSE	MK_SCX_14.8510.8510.3	3	4.744	0.371	1	1179.8	31	K.DCVGPEVENACANPAAGTVILLENLR.F
PGK1_MOUSE	MK_SCX_18.4547.4547.2	2	5.613	0.569	1	1094.2	67.64706	K.VLNNM*EIGTSLYDEEGAK.I
PGK1_MOUSE	MK_SCX_18.5144.5144.2	2	6.243	0.526	1	1625.9	70.588234	K.VLNNMEIGTSLYDEEGAK.I
PGK1_MOUSE	MK_SCX_19.10159.10159.2	2	4.013	0.46	1	300.8	52.941177	K.QIVWNGPVGVFEWEAFAR.G
PGK1_MOUSE	MK_SCX_19.10462.10462.3	3	4.981	0.554	1	681.8	24.193548	K.DVLFLKDCVGPEVENACANPAAGTVILLENLR.F
PGK1_MOUSE	MK_SCX_19.12000.12000.2	2	3.827	0.3	1	1050	66.66667	K.VNEMIIGGGMAFTFLK.V
PGK1_MOUSE	MK_SCX_21.4901.4901.2	2	3.546	0.373	1	975.1	90	K.YSLEPVAAELK.S
PGK1_MOUSE	MK_SCX_2201.4956.4956.2	2	2.774	0.325	1	571.6	72.22222	K.IQLINNMLDK.V
PGK1_MOUSE	MK_SCX_25.6650.6650.2	2	5.519	0.625	1	1015.6	64.70589	K.ITLPVDFVTADKFDENAK.T
PGK1_MOUSE	MK_SCX_26.5755.5755.3	3	3.436	0.377	1	471.4	31.25	K.VLNNMEIGTSLYDEEGAKIVK.D
PGK1_MOUSE	MK_SCX_29.6818.6818.2	2	4.646	0.376	1	914.7	59.375	K.ALESPERPFLAILGGAK.V
PGK1_MOUSE	MK_SCX_29.6982.6982.3	3	4.734	0.542	1	1417.2	54.6875	K.ALESPERPFLAILGGAK.V
PGK1_MOUSE	MK_SCX_30.4416.4416.2	2	4.878	0.577	1	2155.4	78.57143	K.LGDVYVNDAFGTAHR.A
PGK1_MOUSE	MK_SCX_30.4442.4442.3	3	4.263	0.402	1	1127.2	50	K.LGDVYVNDAFGTAHR.A
PGK1_MOUSE	MK_SCX_32.3739.3739.3	3	5.124	0.544	1	774.3	45.3125	R.VDFNVPM*KNNQITNNQR.I
PGK1_MOUSE	MK_SCX_32.3749.3749.2	2	4.153	0.406	1	442.7	65.625	R.VDFNVPM*KNNQITNNQR.I
PGK1_MOUSE	MK_SCX_32.4235.4235.3	3	3.521	0.317	1	789.4	44.230766	K.VADKIQLINNM*LDK.V
PGK1_MOUSE	MK_SCX_32.4297.4297.2	2	4.62	0.481	1	795.4	68.75	R.VDFNVPMKNNQITNNQR.I
PGK1_MOUSE	MK_SCX_32.4319.4319.3	3	4.644	0.463	1	560	42.1875	R.VDFNVPMKNNQITNNQR.I
PGK1_MOUSE	MK_SCX_32.5416.5416.3	3	4.453	0.432	1	1175	53.846157	K.VADKIQLINNMLDK.V
PGK1_MOUSE	MK_SCX_32.5432.5432.2	2	4.567	0.454	1	2349.2	84.61539	K.VADKIQLINNMLDK.V
PGK1_MOUSE	MK_SCX_33.4625.4625.3	3	3.997	0.487	1	1198.1	41.17647	K.VSHVSTGGGASLELLEGK.V
PGK1_MOUSE	MK_SCX_36.6262.6262.2	2	3.244	0.343	1	1056.7	80	K.SLLGKDVLFLK.D
PGK1_MOUSE	MK_SCX_39.3548.3548.3	3	4.869	0.237	1	1377.1	50	R.AHSSMVGVNLPQK.A
PGK1_MOUSE	MK_SCX_39.3549.3549.2	2	4.211	0.521	1	1613.3	83.33333	R.AHSSMVGVNLPQK.A
PGK1_MOUSE	MK_SCX_41.5288.5288.2	2	5.851	0.563	1	2739.4	65.21739	K.WNTEDKVSHVSTGGGASLELLEGK.V
PGK1_MOUSE	MK_SCX_41.5410.5410.3	3	6.202	0.582	1	2633.2	43.47826	K.WNTEDKVSHVSTGGGASLELLEGK.V
PGK1_MOUSE	MK_SCX_48.4623.4623.3	3	4.603	0.481	1	572	35.526314	R.AHSSM*VGVNLPQKAGGFLM*K.K

PGK1_MOUSE	MK_SCX_48.4978.4978.3	3	4.474	0.503	1	1104.8	44.736843	R.AHSSM*VGVNLPQKAGGFLMK.K
PGK1_MOUSE	MK_SCX_48.5012.5012.2	2	5.476	0.611	1	1346.3	73.68421	R.AHSSMVGVNLPQKAGGFLM*K.K
PGK1_MOUSE	MK_SCX_48.5509.5509.2	2	6.053	0.596	1	2702	76.31579	R.AHSSMVGVNLPQKAGGFLMK.K
PGK1_MOUSE	MK_SCX_48.5528.5528.3	3	4.325	0.427	1	821.5	43.421055	R.AHSSMVGVNLPQKAGGFLMK.K
PGM1_MOUSE	MK_SCX_17.7661.7661.2	2	4.128	0.45	1	947.9	55.88235	K.INQDPQVM*LAPLISIALK.V
PGM1_MOUSE	MK_SCX_17.8811.8811.2	2	4.69	0.47	1	2003.4	70.588234	K.INQDPQVMLAPLISIALK.V
PGM1_MOUSE	MK_SCX_18.4483.4483.2	2	4.424	0.299	1	840.9	60.000004	K.ADNFEYSDPVDGSISK.N
PGM1_MOUSE	MK_SCX_20_1.3786.3786.2	2	4.481	0.483	1	1213.5	83.33333	R.YDYEEVEAEGANK.M
PGM1_MOUSE	MK_SCX_35.10561.10561.3	3	3.058	0.243	1	625.7	52.272724	K.MMKDLEALMLDR.S
PGPI_MOUSE	MK_SCX_2201.7776.7776.3	3	4.743	0.32	1	1510.1	40.909092	K.LGLGDSVDLHVYEIPVEYQTVQR.L
PGRC1_MOUSE	MK_SCX_2201.5622.5622.2	2	4.042	0.496	1	1373.1	84.61539	K.FYGPEGPYGVFAGR.D
PGRC1_MOUSE	MK_SCX_23.3483.3483.3	3	4.111	0.534	1	545.8	39.473686	K.EGEEPTVYSDDEEPKDETAR.K
PGRC1_MOUSE	MK_SCX_27.6125.6125.2	2	2.217	0.137	1	1168.5	73.07692	R.ILMAINGKVFDVTK.G
PGRC1_MOUSE	MK_SCX_39.5837.5837.2	2	5.466	0.625	1	1927.3	82.14286	R.KFYGPEGPYGVFAGR.D
PGRC1_MOUSE	MK_SCX_39.5891.5891.3	3	4.751	0.488	1	2218.1	58.928574	R.KFYGPEGPYGVFAGR.D
PGRC2_MOUSE	MK_SCX_18.3416.3416.2	2	7.112	0.662	1	2254.6	68.75	K.LSTLGSGGESGGDGSPGGAGATAAR.S
PGRC2_MOUSE	MK_SCX_20_1.5061.5061.2	2	2.51	0.296	1	515.6	78.57143	R.DFSLEQLR.Q
PGRC2_MOUSE	MK_SCX_2201.6422.6422.2	2	4.036	0.475	1	1652	84.61539	K.FYGPAGPYGIFAGR.D
PGS1_MOUSE	MK_SCX_24.6622.6622.3	3	3.475	0.36	1	715.4	32.5	K.EISPDTTLLDLQNNDISELRK.D
PGS1_MOUSE	MK_SCX_31.7445.7445.3	3	4.121	0.279	1	1180.1	42.647057	K.NHLVEIPPNLPSSLVELR.I
PH4H_MOUSE	MK_SCX_20_1.3778.3778.2	2	3.578	0.375	1	1719.6	90	R.VEVLDNTQQLK.N
PH4H_MOUSE	MK_SCX_26.4543.4543.2	2	2.164	0.398	1	416.6	85.71429	K.NTVPWFPR.T
PH4H_MOUSE	MK_SCX_28.4989.4989.3	3	4.61	0.489	1	1111.9	45.588234	R.YDPYTQRVEVLDNTQQLK.N
PH4H_MOUSE	MK_SCX_28.4992.4992.2	2	2.504	0.429	1	384.6	58.823532	R.YDPYTQRVEVLDNTQQLK.N
PH4H_MOUSE	MK_SCX_35.10653.10653.3	3	4.766	0.586	1	734.8	34.375	R.FANQILSYGAELDADHPGFKDPVYR.A
PH4H_MOUSE	MK_SCX_35.3765.3765.2	2	3.173	0.376	1	755	77.27273	R.NDIGATVHELSR.D
PH4H_MOUSE	MK_SCX_37.5952.5952.2	2	2.41	0.412	1	655.8	66.66667	R.TFAATIPRPFSVR.Y
PH4H_MOUSE	MK_SCX_44.4065.4065.2	2	3.367	0.413	1	1089.6	80	R.KQFADIAYNYR.H
PH4H_MOUSE	MK_SCX_47.4688.4688.3	3	4.089	0.358	1	1355.4	50	K.SLRNDIGATVHELSR.D
PH4H_MOUSE	MK_SCX_48.6157.6157.2	2	5.09	0.543	1	1983.9	85.71429	R.LNKDEYEFFTYLDKR.S
PH4H_MOUSE	MK_SCX_48.6189.6189.3	3	5.018	0.464	1	1196.3	48.214287	R.LNKDEYEFFTYLDKR.S
PH4H_MOUSE	MK_SCX_57.8088.8088.3	3	3.182	0.26	1	1053.6	47.727272	R.RKQFADIAYNYR.H
PH4H_MOUSE	MK_SCX_57.8140.8140.2	2	2.236	0.148	1	337	59.090908	R.RKQFADIAYNYR.H
PHB_MOUSE	MK_SCX_17.12567.12567.2	2	4.354	0.276	1	511.5	55.263157	K.AAELIANSLATAGDGLIELR.K
PHB_MOUSE	MK_SCX_20_1.4716.4716.2	2	3.399	0.32	1	1417.4	88.88889	K.DLQNVNITLR.I
PHB_MOUSE	MK_SCX_20_1.6247.6247.2	2	2.539	0.468	1	429.4	80	R.VLPSITTEILK.S
PHB_MOUSE	MK_SCX_21.2516.2516.2	2	2.828	0.502	1	659	70	K.AAIISAEGDSK.A
PHB_MOUSE	MK_SCX_2201.4431.4431.2	2	3.956	0.336	1	1838.4	94.44444	R.FDAGELITQR.E
PHB_MOUSE	MK_SCX_29.6007.6007.3	3	3.27	0.472	1	704.1	41.666664	R.NVPVITGSKDLQNVNITLR.I
PHB_MOUSE	MK_SCX_34.5316.5316.2	2	5.275	0.389	1	2800.8	92.30769	R.KLEAAEDIAYQLSR.S
PHB_MOUSE	MK_SCX_37.5171.5171.3	3	3.39	0.451	1	573.9	56.81818	R.ILFRPVASQLPR.I
PHB_MOUSE	MK_SCX_37.5198.5198.2	2	3.409	0.36	1	725.2	77.27273	R.ILFRPVASQLPR.I
PHB2_MOUSE	MK_SCX_17.7898.7898.2	2	4.432	0.58	1	748.6	58.333332	R.IYLTADNLVLNLQDESFTR.G
PHB2_MOUSE	MK_SCX_21.2554.2554.3	3	3.107	0.265	1	436.3	45.454548	K.IVQAEGEAEAAK.M
PHB2_MOUSE	MK_SCX_21.2682.2682.2	2	3.927	0.482	1	1899.8	86.36364	K.IVQAEGEAEAAK.M
PHB2_MOUSE	MK_SCX_2201.2319.2319.2	2	2.283	0.227	1	455.9	68.75	R.EYTAAVEAK.Q
PHB2_MOUSE	MK_SCX_2201.3608.3608.2	2	2.905	0.426	1	1136.1	92.85714	R.LGLDYEER.V
PHB2_MOUSE	MK_SCX_2201.5482.5482.2	2	4.343	0.534	1	2380.8	87.5	K.LLLGAGAVAYGVR.E
PHB2_MOUSE	MK_SCX_24.4442.4442.2	2	3.712	0.32	1	1210.7	88.88889	K.FNASQLITQR.A
PHB2_MOUSE	MK_SCX_30.6322.6322.3	3	5.353	0.5	1	2251.5	56.25	R.IGGVQQDTILAEGLHFR.I
PHB2_MOUSE	MK_SCX_31.6448.6448.2	2	4.357	0.518	1	1921.5	71.875	R.IGGVQQDTILAEGLHFR.I

PHB2_MOUSE	MK_SCX_34.4247.4247.3	3	4.345	0.358	1	1115.5	51.666664	R.VLSRPNAQELPSMYQR.L
PHB2_MOUSE	MK_SCX_34.4267.4267.2	2	5.207	0.501	1	1693	73.333336	R.VLSRPNAQELPSMYQR.L
PHB2_MOUSE	MK_SCX_35.4292.4292.2	2	4.885	0.473	1	1760.2	80.769226	K.MLGEALSKNPGYIK.L
PHB2_MOUSE	MK_SCX_49.5355.5355.2	2	5.742	0.55	1	2426.9	72.22222	R.KISSPTGSKDLQMVNISLR.V
PHB2_MOUSE	MK_SCX_49.5395.5395.3	3	3.383	0.308	1	911	41.666664	R.KISSPTGSKDLQMVNISLR.V
PHKG1_MOUSE	MK_SCX_35.7629.7629.2	2	2.053	0.142	1	455.9	75	R.DPYALRPLR.R
PHKG1_MOUSE	MK_SCX_54.6755.6755.2	2	2.018	0.201	1	300.9	34.210526	R.VKPVTREIVIRDPYALRPLR.R
PHKG2_MOUSE	MK_SCX_18.5814.5814.1	1	2.492	0.34	1	344.5	65	R.LRPLTKNALLR.D
PHLB2_MOUSE	MK_SCX_16.5148.5148.2	2	3.981	0.514	1	893.5	52.77778	K.ANGDYSGSYLTLSQPVSAK.R
PHLB2_MOUSE	MK_SCX_30.4437.4437.3	3	4.551	0.392	1	1997.7	57.692307	R.LDEEKENLTQQLLR.E
PHLB2_MOUSE	MK_SCX_41.6829.6829.3	3	3.67	0.439	1	518.8	33.333336	R.KGSLQDVDVAGFGNLGHSASFLAPR.G
PHLB2_MOUSE	MK_SCX_49.5208.5208.3	3	4.289	0.348	1	1663.1	51.785713	K.HFEDLEFQQLEHESR.L
PHLP_MOUSE	MK_SCX_16.5967.5967.2	2	4.522	0.704	1	1149	54.000004	R.GAPAISSTPAEAELAGEGISINTGPK.G
PHP14_MOUSE	MK_SCX_16.3719.3719.2	2	2.337	0.447	1	648	68.181816	R.NGYDCECLGGGR.I
PHP14_MOUSE	MK_SCX_28.6890.6890.3	3	3.131	0.177	1	764.3	34.210526	-.MAADLGQIPDVDIDSDGVFK.Y
PHP14_MOUSE	MK_SCX_29.4021.4021.3	3	4.043	0.474	1	922.4	38.88889	K.VSGELQRNGYDCECLGGGR.I
PHP14_MOUSE	MK_SCX_37.4080.4080.2	2	3.1	0.149	1	858	75	K.WAEYHADIYDK.V
PHP14_MOUSE	MK_SCX_43.4462.4462.2	2	3.051	0.54	1	1259.6	81.818184	K.IHVYGYSMGYGR.A
PHS_MOUSE	MK_SCX_21.4543.4543.2	2	2.083	0.215	1	475.2	71.42857	R.DQLLPNLR.A
PHS_MOUSE	MK_SCX_24.3805.3805.2	2	2.77	0.497	1	966.5	88.88889	R.AVGWNEVEGR.D
PHS_MOUSE	MK_SCX_34.4593.4593.2	2	4.05	0.396	1	1090.3	69.230774	R.LSAEERDQLLPNLR.A
PHS_MOUSE	MK_SCX_34.4807.4807.3	3	4.389	0.372	1	1259.4	55.76923	R.LSAEERDQLLPNLR.A
PHS_MOUSE	MK_SCX_52.4159.4159.2	2	3.879	0.343	1	593	70.83333	K.LDHHPEWFNVYNK.V
PHS_MOUSE	MK_SCX_53.4504.4504.3	3	4.713	0.364	1	2327.9	62.5	K.LDHHPEWFNVYNK.V
PHS_MOUSE	MK_SCX_53.5557.5557.3	3	4.89	0.429	1	1504.6	48.684208	R.VALQAEKLDHHPEWFNVYNK.V
PHS_MOUSE	MK_SCX_56.5805.5805.3	3	3.757	0.496	1	420.6	38.46154	R.DAIFKQFHFKDFNR.A
PHS_MOUSE	MK_SCX_56.5893.5893.2	2	4.202	0.498	1	744.8	73.07692	R.DAIFKQFHFKDFNR.A
PHS2_MOUSE	MK_SCX_25.5465.5465.2	2	2.61	0.381	1	872.1	80	K.NFNQAFGFMSR.V
PHS2_MOUSE	MK_SCX_56.3970.3970.3	3	3.297	0.288	1	708	47.916664	K.MNHHPEWFNVYNK.V
PHS2_MOUSE	MK_SCX_56.3995.3995.2	2	3.599	0.253	1	871.4	70.83333	K.MNHHPEWFNVYNK.V
PI52B_MOUSE	MK_SCX_17.6613.6613.2	2	3.379	0.383	1	609.9	50	R.FFGPGEFDPSVDVYAMK.S
PICA_MOUSE	MK_SCX_18.7443.7443.2	2	5.747	0.679	1	2107.7	68.42105	R.ATTLSNAVSSLASTGLSLTK.V
PICA_MOUSE	MK_SCX_20_1.11903.11903.3	3	3.072	0.38	1	334.7	28.846153	R.GDIPDLSQAPSSLLDALEQHLASLEGK.K
PICA_MOUSE	MK_SCX_2201.2715.2715.2	2	3.034	0.29	1	1353.4	93.75	K.VAEQVGIDR.G
PICA_MOUSE	MK_SCX_2201.4648.4648.2	2	3.047	0.222	1	813.2	58.333332	R.GADGVM*RTM*NTEK.L
PICA_MOUSE	MK_SCX_2201.9384.9384.3	3	4.575	0.476	1	1285.8	31.896553	K.LPPNKLVSDDLDSSLANLVGNLGIGNGTTK.N
PICA_MOUSE	MK_SCX_27.5988.5988.3	3	3.712	0.342	1	1080.6	38.88889	K.SSGDVHLPIASDVSTFTTR.T
PICA_MOUSE	MK_SCX_27.6072.6072.2	2	4.211	0.499	1	733.7	66.66667	K.SSGDVHLPIASDVSTFTTR.T
PIMT_MOUSE	MK_SCX_17.6253.6253.2	2	3.251	0.353	1	338.7	50	R.LILPVGPAGGNQMLEQYDK.L
PIMT_MOUSE	MK_SCX_27.9713.9713.3	3	4.607	0.569	1	849.2	28.030304	R.MGYAEEAPYDAIHVGAAAPVVPQALIDQLKPGGR.L
PIMT_MOUSE	MK_SCX_43.6197.6197.3	3	3.538	0.351	1	477.7	42.1875	K.MKPLMGVIYVPLTDKEK.Q
PIN1_MOUSE	MK_SCX_27.6616.6616.3	3	4.148	0.502	1	2186.6	47.22222	R.TGEM*SGPVFTDSGIHIILR.T
PIN1_MOUSE	MK_SCX_27.7081.7081.3	3	4.307	0.448	1	1194	43.055553	R.TGEMSGPVFTDSGIHIILR.T
PIN1_MOUSE	MK_SCX_33.4979.4979.2	2	4.698	0.491	1	771.6	78.57143	R.GQMQKPFEDASFALR.T
PIN1_MOUSE	MK_SCX_33.4984.4984.3	3	4.277	0.501	1	1419.9	53.571426	R.GQMQKPFEDASFALR.T
PIN4_MOUSE	MK_SCX_20_1.8149.8149.3	3	3.878	0.45	1	498.4	24.13793	R.GSM*VGPFQEAAFALPVSGM*DKPVFTDPPVK.T
PININ_MOUSE	MK_SCX_2201.2717.2717.2	2	2.668	0.401	1	1103.5	87.5	K.LEVQAEEER.K
PININ_MOUSE	MK_SCX_37.5689.5689.2	2	2.065	0.136	1	679.3	72.22222	K.ENINSQEVEK.E
PININ_MOUSE	MK_SCX_51.2847.2847.3	3	3.738	0.328	1	677.2	36.666668	R.NEEQKAEQEEGKVAQR.E
PIPNB_MOUSE	MK_SCX_50.6772.6772.3	3	5.574	0.409	1	1073.7	42.391304	K.IETWHKPDLGTLENVHGLDPNTWK.T
PITM1_MOUSE	MK_SCX_19.8255.8255.3	3	3.129	0.179	1	420.8	18.939394	K.KSREESSGEGSGVEILANRPYTDGPGGNGQYTHK.V

PITM1_MOUSE	MK_SCX_33.5869.5869.2	2	2.188	0.167	1	320.9	50	R.DPLADGVEVLGR.A
PIWL1_MOUSE	MK_SCX_17.6804.6804.2	2	2.664	0.235	1	389.1	53.846157	K.ELIGLIVLTKYNNK.T
PIWL1_MOUSE	MK_SCX_17.7168.7168.1	1	2.021	0.165	1	324.3	38.46154	K.ELIGLIVLTKYNNK.T
PK1IP_MOUSE	MK_SCX_27.3203.3203.3	3	4.804	0.365	1	993.3	33.75	K.TIEKESGDTVQEETSEPNSEK.S
PK3CD_MOUSE	MK_SCX_28.5776.5776.3	3	3.308	0.21	1	448.2	22	R.LEFDISVCDLPRM*ARLCFALYAVVEK.A
PK3CD_MOUSE	MK_SCX_38.4209.4209.2	2	2.402	0.193	1	417.6	59.090908	K.LINSQISLLIAK.G
PKHA6_MOUSE	MK_SCX_2201.3096.3096.2	2	3.009	0.268	1	963.3	87.5	K.MSVEEQMDR.M
PKHA6_MOUSE	MK_SCX_24.6271.6271.2	2	2.746	0.153	1	938.8	66.66667	K.SSMNQLQQWVNLR.R
PKHA6_MOUSE	MK_SCX_25.5793.5793.3	3	3.058	0.397	1	302.3	29.411766	R.YIDLEPDTPLSPEELKEK.Q
PKHA6_MOUSE	MK_SCX_44.3221.3221.3	3	4.76	0.521	1	1577.6	42.105263	K.TGYETSKKDPSQTSPLGTPR.D
PKHA6_MOUSE	MK_SCX_46.4398.4398.3	3	4.151	0.434	1	691.7	41.07143	R.KMELEPQHYDVDISK.E
PKHC1_MOUSE	MK_SCX_16.8311.8311.3	3	5.74	0.647	1	1567	24.468084	K.TMTPTYDAHDGSPLSPTSAWFGDSALSEGNPGILAVSQPVTSPEILAK
PKP4_MOUSE	MK_SCX_23.5768.5768.3	3	4.652	0.632	1	1042.2	30.645163	R.TSLGSGFGSPSVTDSRPLNPSAYSSSTLPAQR.A
PLAP_MOUSE	MK_SCX_18.4838.4838.2	2	3.505	0.578	1	570.2	59.090908	R.YVPGTSGPSNTVQTADPFTGAGR.Y
PLAP_MOUSE	MK_SCX_31.5599.5599.2	2	5.801	0.586	1	2941.5	78.125	K.KEALTFDQANPTQILGK.L
PLAP_MOUSE	MK_SCX_32.5499.5499.3	3	3.783	0.369	1	1142.2	45.3125	K.KEALTFDQANPTQILGK.L
PLDN_MOUSE	MK_SCX_42.7591.7591.3	3	5.112	0.628	1	1923.2	45.833336	R.AVEHLVGGLLSHYLPDLQR.S
PLK2_MOUSE	MK_SCX_15.4621.4621.2	2	3.26	0.177	1	425.1	35.714287	K.CYEMTDLTNNKVYAAKIIPHSR.V
PLK2_MOUSE	MK_SCX_18.5800.5800.2	2	2.039	0.195	1	452.8	55	K.ARKVLTEPEVR.Y
PLSL_MOUSE	MK_SCX_15.10290.10290.2	2	3.879	0.553	1	362.1	35.416664	K.ISTSLPVLDLIDAIQPGSINYDLLK.T
PLSL_MOUSE	MK_SCX_27.5160.5160.2	2	2.744	0.396	1	329.5	75	R.NWMNSLGVNPR.V
PLST_MOUSE	MK_SCX_28.5359.5359.3	3	3.58	0.31	1	1045.5	48.333332	R.IDINMSGFNETDDLKR.A
PLST_MOUSE	MK_SCX_28.5475.5475.2	2	3.549	0.458	1	903.6	80.769226	R.VYALPEDLVEVKPK.M
PLST_MOUSE	MK_SCX_37.5926.5926.2	2	2.027	0.237	1	642.1	68.181816	K.AYFHLLNQIAPK.G
PLST_MOUSE	MK_SCX_37.6035.6035.3	3	3.857	0.379	1	323.7	40.909092	K.AYFHLLNQIAPK.G
PLVAP_MOUSE	MK_SCX_16.6070.6070.2	2	4.481	0.483	1	507	55.263157	R.VSGPPPNPPPIDPASLEEFK.K
PLVAP_MOUSE	MK_SCX_2201.5776.5776.2	2	2.785	0.221	1	359.6	60.000004	K.ETVMQQLLTTR.R
PLVAP_MOUSE	MK_SCX_24.5420.5420.2	2	4.121	0.428	1	1229.8	94.44444	K.FQADVLSAWR.D
PLVAP_MOUSE	MK_SCX_24.5510.5510.3	3	4.011	0.49	1	627.7	36.25	R.VSGPPPNPPPIDPASLEEFKK.R
PLVAP_MOUSE	MK_SCX_27.7104.7104.2	2	4.657	0.606	1	929.6	52.77778	R.TLETLPYHYQLMPEYASLR.R
PLVAP_MOUSE	MK_SCX_27.7171.7171.3	3	3.506	0.365	1	720.7	36.11111	R.TLETLPYHYQLMPEYASLR.R
PMM2_MOUSE	MK_SCX_20_1.3687.3687.2	2	4.483	0.448	1	1947.6	83.33333	R.TVGYTVTAPEDTR.R
PMM2_MOUSE	MK_SCX_24.7000.7000.3	3	3.829	0.439	1	670.3	32.954548	K.IGVVGGSDFEKLQEQLGNDVVEK.Y
PMM2_MOUSE	MK_SCX_31.3952.3952.2	2	2.579	0.369	1	399.7	60.714287	K.TMPGGNDHEIFTDPR.T
PNPH_MOUSE	MK_SCX_17.5704.5704.2	2	6.172	0.347	1	1358.1	69.44444	K.MLGADAVGM*STVPEVIVAR.H
PNPH_MOUSE	MK_SCX_17.6343.6343.2	2	5.661	0.562	1	1778.9	75	K.MLGADAVGMSTVPEVIVAR.H
PNPH_MOUSE	MK_SCX_46.4624.4624.3	3	3.418	0.458	1	385.4	33.333336	K.ANHM*EVLDAGKAAAQTLER.F
PNPO_MOUSE	MK_SCX_28.5290.5290.2	2	4.589	0.574	1	1289.3	71.42857	R.EAFEETHLTSLDPMK.Q
PNPO_MOUSE	MK_SCX_38.5955.5955.3	3	4.314	0.589	1	971.5	34.782608	R.GLATGDSPLGPMTHHGEEDWVYER.L
PO121_MOUSE	MK_SCX_27.3760.3760.3	3	4.429	0.402	1	1282.9	37.5	K.VTDTTTGKQQSSWTSPPTPGSSGQR.K
PP1R7_MOUSE	MK_SCX_17.9144.9144.2	2	6.047	0.678	1	3084.5	79.411766	R.AIENIDTLTNLESLFLGK.N
PP1R7_MOUSE	MK_SCX_36.4353.4353.3	3	3.449	0.204	1	555.1	52.499996	R.IGKIEGLEVLK.K
PP1R8_MOUSE	MK_SCX_15.6179.6179.2	2	5.176	0.59	1	1874.9	67.5	R.VTFSEDDEIINPEDVDPSVGR.F
PP1R8_MOUSE	MK_SCX_21.3765.3765.2	2	4.121	0.535	1	2403.9	85.71429	R.MEGSGSLGLEESGSR.R
PP2AA_MOUSE	MK_SCX_2201.6168.6168.2	2	3.609	0.417	1	1342.9	85	K.YSFLQFDPAPR.R
PP2CA_MOUSE	MK_SCX_2201.2533.2533.2	2	2.379	0.21	1	970.5	93.75	R.VNGSLAVSR.A
PP2CA_MOUSE	MK_SCX_23.3456.3456.2	2	3.347	0.225	1	1382.1	77.27273	R.IQNAGGSVMIQR.V
PP2CA_MOUSE	MK_SCX_26.4482.4482.3	3	3.612	0.331	1	367	32.352943	K.GPTEQLVSPEPEVHDIER.S
PP2CA_MOUSE	MK_SCX_27.4773.4773.2	2	2.705	0.483	1	767.6	83.33333	R.YGLSSMQGWR.V
PP2CA_MOUSE	MK_SCX_28.5059.5059.3	3	4.014	0.303	1	645.1	35.526314	R.TLASENIPSLPPGGELASKR.N
PP2CB_MOUSE	MK_SCX_18.5495.5495.2	2	3.899	0.41	1	865.4	61.11111	R.ILSAENIPNLPPGGGLAGK.R

PP2CB_MOUSE	MK_SCX_30.5259.5259.3	3	3.736	0.471	1	405.2	34.210526	R.ILSAENIPNLPPGGGLAGKR.H
PP2CB_MOUSE	MK_SCX_31.4763.4763.3	3	3.118	0.431	1	898.7	46.153847	K.SGEEGMPDLAHVMR.I
PP2CB_MOUSE	MK_SCX_31.4799.4799.2	2	3.513	0.284	1	568.8	65.38461	K.SGEEGMPDLAHVMR.I
PP2CG_MOUSE	MK_SCX_2201.7732.7732.2	2	3.352	0.477	1	1186.9	84.61539	R.LPLPYGFSAMQGWR.V
PP2CG_MOUSE	MK_SCX_43.3265.3265.3	3	4.968	0.536	1	1646.8	44.736843	R.KLEEALSTEGAEDTGNSDKK.K
PPA6_MOUSE	MK_SCX_16.8547.8547.2	2	4.979	0.483	1	589	47.5	K.NYVEDIPFLSPVYNPQEVFIR.S
PPA6_MOUSE	MK_SCX_2201.5217.5217.2	2	3.561	0.305	1	1284.3	77.27273	K.VGMQQMFALGEK.L
PPA6_MOUSE	MK_SCX_28.11795.11795.3	3	3.422	0.44	1	303.8	28.57143	R.KNYVEDIPFLSPVYNPQEVFIR.S
PPA6_MOUSE	MK_SCX_30.5778.5778.3	3	4.6	0.417	1	1057.5	40.625	R.SPLKPLPLEEQVEWNPK.L
PPA6_MOUSE	MK_SCX_30.5780.5780.2	2	5.169	0.505	1	958.2	75	R.SPLKPLPLEEQVEWNPK.L
PPA6_MOUSE	MK_SCX_52.4517.4517.3	3	3.816	0.377	1	761.8	31.818182	R.FDYTVTNLAGGPKPHSHYDTEYR.K
PPAC_MOUSE	MK_SCX_18.4259.4259.2	2	4.944	0.678	1	1327.6	67.64706	R.IDSAATSTYEVGNPPDYR.G
PPAC_MOUSE	MK_SCX_20_1.4714.4714.2	2	3.614	0.29	1	1398.6	85	K.IELLGSYDPQK.Q
PPBT_MOUSE	MK_SCX_18.4034.4034.2	2	3.073	0.27	1	558	57.14286	K.QALHEAVEMDQAIGK.A
PPBT_MOUSE	MK_SCX_2201.2926.2926.2	2	4.581	0.596	1	1401.1	80.769226	K.ANEGTVGVSAATER.T
PPBT_MOUSE	MK_SCX_30.5415.5415.2	2	3.792	0.475	1	815.6	79.16667	R.LEM*DKFPFVALSK.T
PPBT_MOUSE	MK_SCX_30.5426.5426.3	3	4.234	0.392	1	1050.1	52.083332	R.LEM*DKFPFVALSK.T
PPBT_MOUSE	MK_SCX_30.6606.6606.2	2	3.514	0.513	1	1148.2	75	R.LEMDKFPFVALSK.T
PPBT_MOUSE	MK_SCX_30.6616.6616.3	3	4.13	0.474	1	1313.8	56.25	R.LEMDKFPFVALSK.T
PPBT_MOUSE	MK_SCX_32.3373.3373.3	3	3.302	0.375	1	1226	46.42857	K.QALHEAVEM*DQAIGK.A
PPBT_MOUSE	MK_SCX_34.7698.7698.3	3	3.327	0.26	1	543.4	40.384613	R.GTRLDGLDLISIWK.S
PPBT_MOUSE	MK_SCX_34.7700.7700.2	2	2.744	0.182	1	376.5	53.846157	R.GTRLDGLDLISIWK.S
PPBT_MOUSE	MK_SCX_49.3919.3919.3	3	4.236	0.547	1	1155.3	51.5625	K.AKQALHEAVEMDQAIGK.A
PPBT_MOUSE	MK_SCX_56.5363.5363.3	3	4.218	0.385	1	389.3	29.166666	K.GQLHHNTGEETRLEM*DKFPFVALSK.T
PPCKC_MOUSE	MK_SCX_15.9208.9208.3	3	4.503	0.488	1	1625.3	35.833332	R.FCTPASQCPIIDPAWESPEGVPIEGIIFGGR.R
PPCKC_MOUSE	MK_SCX_15.9229.9229.2	2	3.728	0.485	1	481.8	31.666666	R.FCTPASQCPIIDPAWESPEGVPIEGIIFGGR.R
PPCKC_MOUSE	MK_SCX_18.5663.5663.2	2	5.285	0.391	1	1229.4	70	R.YLEDQVNTDLPYEIER.E
PPCKC_MOUSE	MK_SCX_19.5341.5341.2	2	2.615	0.243	1	1013.6	55.88235	R.AINPENGFFGVAPGTSVK.T
PPCKC_MOUSE	MK_SCX_20_1.4945.4945.2	2	3.738	0.544	1	1372.1	80.769226	K.VIQGSLDSLPQAVR.K
PPCKC_MOUSE	MK_SCX_20_1.8165.8165.2	2	3.115	0.431	1	1156.2	64.28571	K.GLGGVNVEELFGISK.E
PPCKC_MOUSE	MK_SCX_25.5924.5924.2	2	2.705	0.475	1	871.5	92.85714	R.VLEWMFGR.I
PPIA_MOUSE	MK_SCX_13.6029.6029.2	2	2.603	0.558	1	451.5	76.92308	R.IIPGFMCQGGDFTR.H
PPIA_MOUSE	MK_SCX_17.7264.7264.2	2	6.216	0.642	1	1998.1	70.588234	-.VNPTVFFDITADDEPLGR.V
PPIA_MOUSE	MK_SCX_19.6842.6842.2	2	2.853	0.388	1	495.4	34.615387	K.HTGPGILSMANAGPNTNGSQFFICTAK.T
PPIA_MOUSE	MK_SCX_19.6917.6917.3	3	3.087	0.137	1	400.1	22.115383	K.HTGPGILSMANAGPNTNGSQFFICTAK.T
PPIA_MOUSE	MK_SCX_21.4758.4758.2	2	3.241	0.26	1	725.4	87.5	K.FEDENFILK.H
PPIA_MOUSE	MK_SCX_2201.4703.4703.2	2	3.425	0.268	1	582.4	75	K.EGMNIVEAMER.F
PPIA_MOUSE	MK_SCX_2201.5294.5294.2	2	3.34	0.226	1	989.7	87.5	R.VSFELFADK.V
PPIA_MOUSE	MK_SCX_31.6058.6058.2	2	5.581	0.45	1	1776.5	75	R.SIYGEKFEDENFILK.H
PPIA_MOUSE	MK_SCX_33.10829.10829.3	3	3.389	0.543	1	391.2	47.727272	R.VSFELFADKVPK.T
PPIA_MOUSE	MK_SCX_33.5739.5739.2	2	4.21	0.514	1	978.9	77.27273	R.VSFELFADKVPK.T
PPIA_MOUSE	MK_SCX_37.3154.3154.3	3	3.391	0.347	1	987	52.083332	K.VKEGM*NIVEAM*ER.F
PPIA_MOUSE	MK_SCX_37.3857.3857.2	2	4.236	0.461	1	949.9	83.33333	K.VKEGM*NIVEAMER.F
PPIA_MOUSE	MK_SCX_37.3874.3874.3	3	3.429	0.341	1	411.3	37.5	K.VKEGM*NIVEAMER.F
PPIA_MOUSE	MK_SCX_37.4819.4819.2	2	4.749	0.543	1	1648.2	87.5	K.VKEGMNIVEAMER.F
PPIA_MOUSE	MK_SCX_37.5075.5075.3	3	4.582	0.374	1	1033.9	52.083332	K.VKEGMNIVEAMER.F
PPIA_MOUSE	MK_SCX_38.3467.3467.2	2	3.816	0.598	1	1430.9	87.5	K.VKEGMNIVEAM*ER.F
PPIA_MOUSE	MK_SCX_38.3504.3504.3	3	4.168	0.323	1	686.1	47.916664	K.VKEGMNIVEAM*ER.F
PPIA_MOUSE	MK_SCX_44.6496.6496.3	3	3.06	0.3	1	611.7	33.82353	R.VSFELFADKVPKTAENFR.A
PPIA_MOUSE	MK_SCX_51.4383.4383.3	3	3.314	0.46	1	860.8	54.166668	K.TEWLDGKHVVFGK.V
PPIA_MOUSE	MK_SCX_51.4498.4498.2	2	4.481	0.465	1	1372.7	79.16667	K.TEWLDGKHVVFGK.V

PPIA_MOUSE	MK_SCX_57.4092.4092.3	3	3.2	0.394	1	893.9	39.705883	R.ALSTGEKGFGYKGSSFHR.I
PPIB_MOUSE	MK_SCX_16.5318.5318.2	2	3.328	0.393	1	1553.7	83.33333	K.DTNGSQFFITTVK.T
PPIB_MOUSE	MK_SCX_19.6557.6557.2	2	4.461	0.619	1	1610	88.46153	K.VYFDLQIGDESVGR.V
PPIB_MOUSE	MK_SCX_20_1.5559.5559.2	2	4.294	0.5	1	1668.4	83.33333	K.TVDNFVALATGEK.G
PPIB_MOUSE	MK_SCX_2201.3063.3063.2	2	2.626	0.251	1	562.1	81.25	K.VLEGM*DVVR.K
PPIB_MOUSE	MK_SCX_24.4985.4985.2	2	2.581	0.432	1	573.8	85.71429	R.VVFGLFGK.T
PPIB_MOUSE	MK_SCX_34.4652.4652.2	2	3.25	0.515	1	915.2	69.230774	R.VIKDFM*IQGGDFTR.G
PPIB_MOUSE	MK_SCX_34.4673.4673.3	3	4.262	0.381	1	1299.6	53.846157	R.VIKDFM*IQGGDFTR.G
PPIB_MOUSE	MK_SCX_34.5228.5228.2	2	4.312	0.561	1	2060.1	84.61539	R.VIKDFMIQGGDFTR.G
PPIB_MOUSE	MK_SCX_34.5367.5367.3	3	3.776	0.246	1	1068.1	51.923077	R.VIKDFMIQGGDFTR.G
PPIB_MOUSE	MK_SCX_36.4303.4303.2	2	3.867	0.462	1	796.3	66.66667	K.SIYGERFPDENFK.L
PPIB_MOUSE	MK_SCX_38.4140.4140.2	2	2.506	0.255	1	432.4	75	R.FPDENFKLK.H
PPIB_MOUSE	MK_SCX_43.4061.4061.3	3	4.477	0.405	1	922.7	46.153847	R.DKPLKDVIIVDSGK.I
PPIB_MOUSE	MK_SCX_43.4082.4082.2	2	4.104	0.536	1	1089	80.769226	R.DKPLKDVIIVDSGK.I
PPIB_MOUSE	MK_SCX_43.4581.4581.3	3	3.561	0.461	1	510.9	32.5	R.VIKDFM*IQGGDFTRGDGTGGK.S
PPIB_MOUSE	MK_SCX_43.5030.5030.2	2	5.346	0.543	1	1580.3	60.000004	R.VIKDFMIQGGDFTRGDGTGGK.S
PPIB_MOUSE	MK_SCX_44.4051.4051.2	2	4.686	0.639	1	1312.8	84.61539	K.HYGPGWVSMANAGK.D
PPIB_MOUSE	MK_SCX_44.4096.4096.3	3	3.548	0.389	1	1400.6	50	K.HYGPGWVSMANAGK.D
PPIB_MOUSE	MK_SCX_44.4894.4894.3	3	3.475	0.448	1	707.9	36.25	R.VIKDFMIQGGDFTRGDGTGGK.S
PPIB_MOUSE	MK_SCX_50.5009.5009.2	2	4.428	0.256	1	821.3	60.714287	K.SIYGERFPDENFKLK.H
PPIC_MOUSE	MK_SCX_33.5249.5249.3	3	3.859	0.33	1	1358.9	46.42857	R.VIKDFMIQGGDFTAR.D
PPID_MOUSE	MK_SCX_16.5559.5559.2	2	3.066	0.261	1	976.9	52.272724	K.FMIQGGDFSNQNGTGGESIYGEK.F
PPID_MOUSE	MK_SCX_19.7862.7862.3	3	3.764	0.327	1	640.7	25.833332	R.EGLLSMANAGPNTNGSQFFITTVPTPHLDGK.H
PPID_MOUSE	MK_SCX_20_1.8938.8938.2	2	2.675	0.131	1	347	46.666668	R.LQPIALSCVLNIGACK.L
PPID_MOUSE	MK_SCX_21.6054.6054.2	2	2.75	0.349	1	401.3	60.000004	R.VFFDVDIGGER.V
PPID_MOUSE	MK_SCX_26.7535.7535.3	3	3.703	0.487	1	1185	36.25	K.LCVIAECGELKEGDDWGIFPK.D
PPID_MOUSE	MK_SCX_45.6420.6420.3	3	3.013	0.24	1	419	33.333336	K.AQGWQGLKEYDQALADLKK.A
PPID_MOUSE	MK_SCX_50.6047.6047.3	3	4.295	0.351	1	1477.8	48.61111	R.KAQGWQGLKEYDQALADLK.K
PPIE_MOUSE	MK_SCX_14.10768.10768.1	1	1.907	0.123	1	336.6	45.833336	K.KARSNPQVYMDIK.I
PPIE_MOUSE	MK_SCX_33.3438.3438.3	3	5.691	0.567	1	1097.4	32	K.TLEENKEEEGPEPPKAEAQEGEPTAK.K
PPIF_MOUSE	MK_SCX_17.6295.6295.2	2	4.434	0.545	1	880.9	45.652176	R.GANSSSGNPLVYLDVGADGQPLGR.V
PPIF_MOUSE	MK_SCX_17.6321.6321.3	3	4.477	0.493	1	1174.7	33.695652	R.GANSSSGNPLVYLDVGADGQPLGR.V
PPIF_MOUSE	MK_SCX_2201.4457.4457.2	2	2.394	0.308	1	576.7	81.25	R.FPDENFTLK.H
PPIF_MOUSE	MK_SCX_34.5495.5495.2	2	4.469	0.406	1	1068.3	67.85714	R.SIYGSRFPDENFTLK.H
PPIL1_MOUSE	MK_SCX_28.3605.3605.3	3	3.159	0.313	1	599.2	37.5	R.VGMVETNSQDRPVDDVK.I
PPIL1_MOUSE	MK_SCX_31.4595.4595.3	3	3.981	0.34	1	922.9	40.625	R.IIKDFMIQGGDPTGTGR.G
PPP5_MOUSE	MK_SCX_19.5872.5872.2	2	3.657	0.547	1	625.7	52.77778	K.FYSQAIELNPGNAIYYGNR.S
PPT1_MOUSE	MK_SCX_18.4863.4863.2	2	3.557	0.588	1	955.5	78.57143	K.ETIPLQESTLYTEDR.L
PPT1_MOUSE	MK_SCX_28.4558.4558.3	3	4.5	0.434	1	1116.4	39.473686	R.SGQAKETIPLQESTLYTEDR.L
PPT1_MOUSE	MK_SCX_28.4579.4579.2	2	5.657	0.628	1	945.1	65.789474	R.SGQAKETIPLQESTLYTEDR.L
PPT1_MOUSE	MK_SCX_46.5022.5022.3	3	3.702	0.491	1	1034.8	40.625	R.LVQAQYWHDPIKESVYR.N
PQBP1_MOUSE	MK_SCX_26.4020.4020.3	3	4.896	0.529	1	1300	44.11765	R.KDEELDPM*DPSSYSDAPR.G
PRDX1_MOUSE	MK_SCX_19.5083.5083.2	2	3.095	0.197	1	1156.7	63.333332	K.QGGLGPM*NIPLISDPK.R
PRDX1_MOUSE	MK_SCX_19.6000.6000.2	2	2.312	0.288	1	453.7	53.333336	K.QGGLGPMNIPLISDPK.R
PRDX1_MOUSE	MK_SCX_2201.3968.3968.2	2	3.199	0.562	1	716.9	88.88889	R.TIAQDYGVLK.A
PRDX1_MOUSE	MK_SCX_2201.4296.4296.2	2	3.986	0.444	1	1247.2	88.88889	R.LVQAFQFTDK.H
PRDX1_MOUSE	MK_SCX_2201.4782.4782.2	2	3.291	0.521	1	490.9	80	R.QITINDLPVGR.S
PRDX1_MOUSE	MK_SCX_24.3224.3224.2	2	2.496	0.265	1	463.6	91.66667	R.SVDEIIR.L
PRDX1_MOUSE	MK_SCX_24.3330.3330.2	2	2.953	0.339	1	637.5	85.71429	K.ADEGISFR.G
PRDX1_MOUSE	MK_SCX_26.5498.5498.2	2	4.808	0.555	1	791.4	55.555557	K.ATAVMPDGQFKDISLSEYK.G
PRDX1_MOUSE	MK_SCX_29.12870.12870.3	3	4.039	0.589	1	830.1	47.058823	R.TIAQDYGVLKADEGISFR.G

PRDX1_MOUSE	MK_SCX_29.14959.14959.2	2	4.571	0.606	1	1888.4	67.64706	R.TIAQDYGVLKADEGISFR.G
PRDX1_MOUSE	MK_SCX_30.5907.5907.3	3	4.075	0.579	1	1073.9	46.875	K.QGGLGPMNIPLISDPKR.T
PRDX1_MOUSE	MK_SCX_30.5932.5932.2	2	3.395	0.449	1	829.6	59.375	K.QGGLGPMNIPLISDPKR.T
PRDX1_MOUSE	MK_SCX_32.6335.6335.2	2	3.866	0.453	1	1373.3	81.818184	R.GLFIIDDKGILR.Q
PRDX1_MOUSE	MK_SCX_33.5699.5699.3	3	3.309	0.231	1	819.3	39.0625	K.KQGGLGPMNIPLISDPK.R
PRDX1_MOUSE	MK_SCX_34.6007.6007.2	2	4.541	0.487	1	611	62.5	K.KQGGLGPMNIPLISDPK.R
PRDX1_MOUSE	MK_SCX_44.6997.6997.3	3	3.366	0.275	1	477.5	26.086956	K.LNCQVIGASVDSHFCHLAWINTPK.K
PRDX1_MOUSE	MK_SCX_49.13424.13424.3	3	5.067	0.588	1	1263.6	43.055553	K.RTIAQDYGVLKADEGISFR.G
PRDX1_MOUSE	MK_SCX_49.4691.4691.3	3	4.902	0.54	1	835.5	45.588234	K.KQGGLGPM*NIPLISDPKR.T
PRDX1_MOUSE	MK_SCX_49.4713.4713.2	2	3.782	0.543	1	768.4	61.764706	K.KQGGLGPM*NIPLISDPKR.T
PRDX1_MOUSE	MK_SCX_49.5695.5695.2	2	5.062	0.55	1	1060.5	67.64706	K.KQGGLGPMNIPLISDPKR.T
PRDX1_MOUSE	MK_SCX_49.5720.5720.3	3	5.512	0.46	1	1250.7	48.52941	K.KQGGLGPMNIPLISDPKR.T
PRDX1_MOUSE	MK_SCX_54.6682.6682.3	3	3.771	0.427	1	606.3	29.166666	K.KLNCQVIGASVDSHFCHLAWINTPK.K
PRDX2_MOUSE	MK_SCX_17.7971.7971.2	2	4.433	0.537	1	608.4	59.375	K.EGGLGPLNIPLLADVTK.S
PRDX2_MOUSE	MK_SCX_2201.4263.4263.2	2	3.22	0.501	1	712.9	90	R.QITVNDLPVGR.S
PRDX2_MOUSE	MK_SCX_26.6861.6861.2	2	4.92	0.507	1	1079.2	58.333332	K.SAPDFTATAVVDGAFKEIK.L
PRDX2_MOUSE	MK_SCX_30.7677.7677.3	3	4.156	0.5	1	1392.1	52.941177	R.KEGGLGPLNIPLLADVTK.S
PRDX2_MOUSE	MK_SCX_30.7827.7827.2	2	6.083	0.648	1	2320.7	76.47059	R.KEGGLGPLNIPLLADVTK.S
PRDX2_MOUSE	MK_SCX_32.4690.4690.2	2	5.443	0.603	1	1661.5	67.64706	K.SLSQNYGVLKNDEGIAYR.G
PRDX2_MOUSE	MK_SCX_32.4743.4743.3	3	4.043	0.583	1	882.3	48.52941	K.SLSQNYGVLKNDEGIAYR.G
PRDX3_MOUSE	MK_SCX_17.14321.14321.2	2	3.612	0.503	1	697.1	80.769226	R.DYGVLLESAGIALR.G
PRDX3_MOUSE	MK_SCX_20_1.6601.6601.2	2	3.669	0.289	1	1176.9	77.27273	R.GLFIIDPNGVVK.H
PRDX3_MOUSE	MK_SCX_41.4019.4019.3	3	3.059	0.293	1	505.2	50	K.HLSVNDLPVGR.S
PRDX3_MOUSE	MK_SCX_42.3911.3911.2	2	3.132	0.61	1	1053.1	90	K.HLSVNDLPVGR.S
PRDX3_MOUSE	MK_SCX_42.6544.6544.2	2	3.875	0.135	1	587.7	55.88235	R.KNGGLGHMNITLLSDITK.Q
PRDX3_MOUSE	MK_SCX_48.6565.6565.3	3	3.365	0.478	1	1640.2	45.588234	R.KNGGLGHMNITLLSDITK.Q
PRDX5_MOUSE	MK_SCX_13.6677.6677.2	2	4.345	0.568	1	907.1	73.333336	K.GVLFGVPGAFTPGCSK.T
PRDX5_MOUSE	MK_SCX_17.5439.5439.2	2	3.998	0.581	1	646.6	71.875	K.VGDAIPSVEVFEGEPGK.K
PRDX5_MOUSE	MK_SCX_17.8495.8495.2	2	6.344	0.605	1	2530	87.5	K.ATDLLLDDSLVSLFGNR.R
PRDX5_MOUSE	MK_SCX_20_1.4455.4455.2	2	2.917	0.32	1	1751.6	90	R.FSM*VIDNGIVK.A
PRDX5_MOUSE	MK_SCX_21.11652.11652.3	3	3.608	0.435	1	564.3	25	R.LLADPTGAFGKATDLLLDDSLVSLFGNR.R
PRDX5_MOUSE	MK_SCX_21.5276.5276.2	2	3.98	0.358	1	1119.5	85	R.FSMVIDNGIVK.A
PRDX5_MOUSE	MK_SCX_24.5660.5660.2	2	3.132	0.354	1	853.8	92.85714	K.VNLAELFK.G
PRDX5_MOUSE	MK_SCX_24.6350.6350.2	2	4.627	0.574	1	1629.2	78.125	K.KGVLFGVPGAFTPGCSK.T
PRDX5_MOUSE	MK_SCX_24.6375.6375.3	3	4.654	0.522	1	1229	48.4375	K.KGVLFGVPGAFTPGCSK.T
PRDX5_MOUSE	MK_SCX_25.8607.8607.2	2	2.512	0.147	1	661.3	50	K.ATDLLLDDSLVSLFGNRR.L
PRDX5_MOUSE	MK_SCX_27.5355.5355.2	2	4.642	0.581	1	685.9	67.64706	K.VGDAIPSVEVFEGEPGKK.V
PRDX5_MOUSE	MK_SCX_32.9027.9027.3	3	3.008	0.277	1	300.3	27.941175	K.ATDLLLDDSLVSLFGNRR.L
PRDX5_MOUSE	MK_SCX_33.8287.8287.2	2	4.815	0.515	1	677.7	42	K.VGDAIPSVEVFEGEPGKKVNLAELFK.G
PRDX5_MOUSE	MK_SCX_36.4734.4734.3	3	4.945	0.375	1	1967.2	53.846157	K.THLPGFVEQAGALK.A
PRDX5_MOUSE	MK_SCX_36.5006.5006.2	2	3.903	0.59	1	831.5	84.61539	K.THLPGFVEQAGALK.A
PRDX5_MOUSE	MK_SCX_38.5487.5487.2	2	2.51	0.362	1	835.9	83.33333	K.VNLAELFKGK.K
PRDX5_MOUSE	MK_SCX_41.4933.4933.2	2	3.1	0.394	1	1057.8	81.818184	K.RFSMVIDNGIVK.A
PRDX5_MOUSE	MK_SCX_43.5026.5026.2	2	3.084	0.234	1	647.8	75	K.KVNLAELFK.G
PRDX5_MOUSE	MK_SCX_50.4344.4344.3	3	4.656	0.507	1	1281.6	50	K.THLPGFVEQAGALKAK.G
PRDX5_MOUSE	MK_SCX_50.4352.4352.2	2	3.896	0.514	1	773.6	70	K.THLPGFVEQAGALKAK.G
PRDX6_MOUSE	MK_SCX_13.4943.4943.2	2	2.324	0.401	1	423.8	63.636364	R.DFTPVCTTELGR.A
PRDX6_MOUSE	MK_SCX_17.6041.6041.2	2	6.448	0.644	1	2239.4	77.5	-.PGGLLLGDEAPNFEANTTIGR.I
PRDX6_MOUSE	MK_SCX_17.6137.6137.3	3	3.739	0.458	1	431.2	33.75	-.PGGLLLGDEAPNFEANTTIGR.I
PRDX6_MOUSE	MK_SCX_19.6518.6518.2	2	3.139	0.524	1	370.3	40.476192	K.DINAYNGETPTEKLPFPIIDDK.G
PRDX6_MOUSE	MK_SCX_21.5435.5435.2	2	2.364	0.209	1	470	62.5	K.LPFPIIDDK.G

PRDX6_MOUSE	MK_SCX_25.5808.5808.3	3	5.059	0.483	1	1203.2	42.105263	R.VVDSLQLTGTKPVATPVDWK.K
PRDX6_MOUSE	MK_SCX_25.5839.5839.2	2	5.451	0.605	1	1499.3	65.789474	R.VVDSLQLTGTKPVATPVDWK.K
PRDX6_MOUSE	MK_SCX_30.5770.5770.3	3	3.682	0.139	1	634.5	34.523808	K.DINAYNGETPTEKLPFPIIDDK.G
PRDX6_MOUSE	MK_SCX_31.4674.4674.2	2	5.417	0.469	1	1320.8	71.875	K.KGESVMVVPTLSEEEAK.Q
PRDX6_MOUSE	MK_SCX_31.4706.4706.3	3	3.636	0.382	1	994.3	45.3125	K.KGESVMVVPTLSEEEAK.Q
PRDX6_MOUSE	MK_SCX_35.4428.4428.2	2	2.603	0.339	1	575.4	72.22222	R.VVFIFGPDKK.L
PRDX6_MOUSE	MK_SCX_37.5686.5686.3	3	4.179	0.397	1	606.5	35	R.VVDSLQLTGTKPVATPVDWKK.G
PRDX6_MOUSE	MK_SCX_49.8355.8355.3	3	3.245	0.509	1	514.5	39.0625	R.FHDFLGDSWGILFSHPR.D
PRDX6_MOUSE	MK_SCX_49.8474.8474.2	2	6.123	0.614	1	3307.6	84.375	R.FHDFLGDSWGILFSHPR.D
PREB_MOUSE	MK_SCX_19.8907.8907.2	2	2.146	0.183	1	318.1	46.666668	K.NLEAVQTDFSNEPLQK.V
PREB_MOUSE	MK_SCX_24.5247.5247.2	2	2.455	0.367	1	403.3	75	R.APFPLYALR.I
PRELP_MOUSE	MK_SCX_21.4567.4567.2	2	3.548	0.352	1	933.8	72.72727	K.NQLEEVPSALPR.N
PROD_MOUSE	MK_SCX_18.5880.5880.2	2	5.112	0.537	1	2368.4	83.33333	R.LMIDAEQSYFQPAISR.L
PROD_MOUSE	MK_SCX_24.5745.5745.3	3	4.711	0.52	1	1200.4	36.363636	R.AAEIGYEDPINPTYEATNAMYHR.C
PROD_MOUSE	MK_SCX_28.15929.15929.3	3	4.022	0.394	1	1089.8	40.27778	K.LTALGRPQFLLQFSDVLTR.W
PROD_MOUSE	MK_SCX_32.6496.6496.3	3	5.33	0.534	1	1934.5	55.88235	R.HLVVPNVQTGQLEPLLSR.F
PROD_MOUSE	MK_SCX_32.6613.6613.2	2	5.479	0.684	1	1084.4	73.52941	R.HLVVPNVQTGQLEPLLSR.F
PROD_MOUSE	MK_SCX_41.3251.3251.3	3	4.649	0.299	1	2036	59.615387	R.FFHQMAAEQGQAGR.A
PROF1_MOUSE	MK_SCX_17.15635.15635.2	2	4.095	0.339	1	1741.6	80.769226	R.DSLLQDGEFTMDLR.T
PROF1_MOUSE	MK_SCX_17.5640.5640.2	2	4.116	0.344	1	1653.5	80.769226	R.DSLLQDGEFTM*DLR.T
PROF1_MOUSE	MK_SCX_18.6918.6918.2	2	4.843	0.503	1	1070.6	73.333336	K.TFVSITPAEVGVLVGK.D
PROF1_MOUSE	MK_SCX_20_1.6392.6392.2	2	3.048	0.298	1	905.5	73.07692	R.SSFFVNGLTLGGQK.C
PROF1_MOUSE	MK_SCX_28.7053.7053.2	2	4.312	0.531	1	503.3	55.88235	K.TFVSITPAEVGVLVGKDR.S
PROSC_MOUSE	MK_SCX_18.5947.5947.2	2	3.653	0.403	1	542.8	62.5	K.LM*AVPNLSM*LETVDSVK.L
PROSC_MOUSE	MK_SCX_18.6636.6636.2	2	3.148	0.378	1	449.4	59.375	K.LMAVPNLSM*LETVDSVK.L
PROSC_MOUSE	MK_SCX_18.7423.7423.2	2	5.022	0.635	1	915.9	81.25	K.LMAVPNLSMLETVDSVK.L
PROSC_MOUSE	MK_SCX_21.3438.3438.2	2	2.279	0.363	1	340.3	45.833336	K.VM*VQINTSGEDSK.H
PROSC_MOUSE	MK_SCX_21.3786.3786.2	2	4.783	0.509	1	2145.4	87.5	K.VMVQINTSGEDSK.H
PROSC_MOUSE	MK_SCX_21.7490.7490.2	2	4.718	0.53	1	1879.1	87.5	R.TFGENYVQELLEK.A
PROSC_MOUSE	MK_SCX_48.5803.5803.2	2	3.617	0.623	1	1095	73.333336	K.HGLLPSETIAVVEHIK.A
PROSC_MOUSE	MK_SCX_48.6517.6517.3	3	5.217	0.573	1	2302.2	56.666668	K.HGLLPSETIAVVEHIK.A
PROSC_MOUSE	MK_SCX_51.3999.3999.2	2	5.241	0.586	1	1521.1	83.33333	K.TKPADMVIEAYGHGQR.T
PROSC_MOUSE	MK_SCX_54.4027.4027.3	3	3.61	0.459	1	699.4	31.944445	R.IGSTIFGERDYSKKPALDK.T
PROSC_MOUSE	MK_SCX_9.5627.5627.2	2	2.054	0.174	1	306.4	38.235294	-.M*LRGGSMTAELGVGFALR.A
PRP19_MOUSE	MK_SCX_15.6563.6563.2	2	3.96	0.422	1	1247.8	50	K.YIAENGTDPINNQPLSEEQLIDIK.V
PRP19_MOUSE	MK_SCX_16.8825.8825.3	3	5.173	0.62	1	452.8	19.02174	R.EALATLKPQAGLIVPQAVPSSQPSVVGAGEPMDLGELVGMTPEIIQK.L
PRP40_MOUSE	MK_SCX_19.7010.7010.2	2	4.637	0.523	1	1073.2	75	R.FTNMLGQPGSTALDLFK.F
PRP4B_MOUSE	MK_SCX_17.6509.6509.2	2	3.601	0.262	1	1280.2	69.230774	K.DLLDQILM*LDPAKR.I
PRP4B_MOUSE	MK_SCX_19.6641.6641.2	2	2.609	0.133	1	939.1	61.538464	R.ARDNARANQEVAVK.I
PRP4B_MOUSE	MK_SCX_21.6405.6405.2	2	4.79	0.498	1	1501.2	68.75	R.YNVYGYTGQGVFSNVVR.A
PRP6_MOUSE	MK_SCX_11.11997.11997.2	2	2.402	0.362	1	599.6	53.846157	K.KCPHSTPLWLLLSR.L
PRP6_MOUSE	MK_SCX_19.3896.3896.2	2	3.259	0.531	1	782	65.625	R.LSQVSDSVSGQTVVDPK.G
PRPF3_MOUSE	MK_SCX_12.7702.7702.2	2	2.472	0.26	1	458.6	50	K.EVELTHRM*PTLKANIR.A
PRPF3_MOUSE	MK_SCX_15.6058.6058.2	2	5.06	0.536	1	779.7	58.69565	R.FEEVEEEPEVIPGPPSESPGMLTK.L
PRPF3_MOUSE	MK_SCX_2201.2983.2983.2	2	3.717	0.391	1	1262.6	88.88889	K.LQAEISQAAR.K
PRPF3_MOUSE	MK_SCX_41.4155.4155.3	3	3.579	0.561	1	545.2	42.647057	R.VLGTEAVQDPTKVEAHVR.A
PRR6_MOUSE	MK_SCX_15.5884.5884.2	2	5.823	0.537	1	749.8	48.148148	R.AQEAAAEEPPPAVTPAASVSALDLGEQR.E
PRR6_MOUSE	MK_SCX_15.5906.5906.3	3	5.698	0.551	1	1596.7	37.962963	R.AQEAAAEEPPPAVTPAASVSALDLGEQR.E
PRR6_MOUSE	MK_SCX_19.6570.6570.2	2	4.034	0.416	1	1620.5	83.33333	K.LLLDTFEYQGLVK.H
PRR6_MOUSE	MK_SCX_2201.5641.5641.3	3	3.268	0.417	1	565.9	28.448275	R.AQEAAAEEPPPAVTPAASVSALDLGEQRER.W
PRR6_MOUSE	MK_SCX_55.2683.2683.3	3	3.986	0.52	1	668.9	35.416664	R.AGAGGGGGAVGPQPSAKPRPKPPPR.A

PRS10_MOUSE	MK_SCX_29.5200.5200.3	3	3.866	0.215	1	1462.5	43.75	R.TLM*ELLNQM*DGFDTLHR.V
PRS10_MOUSE	MK_SCX_31.4968.4968.2	2	3.283	0.385	1	729	66.66667	R.ADHDFVVQEDFMK.A
PRS10_MOUSE	MK_SCX_31.5056.5056.3	3	3.154	0.43	1	575.8	40	K.VVSSSIVDKYIGESAR.L
PRS10_MOUSE	MK_SCX_37.4247.4247.2	2	3.638	0.6	1	1590.6	85	K.HGEIDYEAIVK.L
PRS4_MOUSE	MK_SCX_17.5744.5744.2	2	5.863	0.544	1	2034.3	68.42105	K.APQETYADIGGLDNQIQEIK.E
PRS4_MOUSE	MK_SCX_19.8185.8185.2	2	3.881	0.48	1	1279.7	71.42857	R.TMLELLNQLDGFDSR.G
PRS4_MOUSE	MK_SCX_47.2906.2906.3	3	3.716	0.264	1	722	41.666664	R.NQEQMKPLEEKQEEER.S
PRS6A_MOUSE	MK_SCX_18.6891.6891.2	2	4.478	0.427	1	691.8	52.77778	R.TM*LELLNQLDGFQPNTQVK.V
PRS6A_MOUSE	MK_SCX_18.7672.7672.2	2	3.575	0.51	1	652.7	67.85714	R.QTYFLPVIGLVDAEK.L
PRS6A_MOUSE	MK_SCX_19.6256.6256.2	2	2.576	0.347	1	457.9	53.333336	K.LAGPQLVQM*FIGDGAK.L
PRS6A_MOUSE	MK_SCX_19.7215.7215.2	2	4.733	0.597	1	1859.4	73.333336	K.LAGPQLVQMFIGDGAK.L
PRS6A_MOUSE	MK_SCX_20_1.6484.6484.2	2	2.208	0.245	1	688.7	77.77778	R.VDILDPALLR.S
PRS6A_MOUSE	MK_SCX_2201.3247.3247.2	2	3.292	0.4	1	883.4	87.5	K.MSTEEIVQR.T
PRS6A_MOUSE	MK_SCX_25.4771.4771.3	3	6.218	0.577	1	1715.3	50	K.AMEVDERPTEQYSDIGGLDK.Q
PRS6A_MOUSE	MK_SCX_26.6349.6349.2	2	4.777	0.555	1	710.8	54.761906	K.FENLGIQPPKGVLMYGPPGTGK.T
PRS6A_MOUSE	MK_SCX_28.6433.6433.3	3	5.319	0.452	1	1869.7	60.9375	K.VIAATNRVDILDPALLR.S
PRS6A_MOUSE	MK_SCX_28.6472.6472.2	2	2.708	0.253	1	934.9	71.875	K.VIAATNRVDILDPALLR.S
PRS6A_MOUSE	MK_SCX_37.4583.4583.2	2	3.389	0.322	1	790	72.72727	R.KIEFPMPNEEAR.A
PRS6B_MOUSE	MK_SCX_18.8479.8479.3	3	7.873	0.646	1	1041.3	36.71875	K.IQDEIPALSVSRPQTGLSFLGPEPEDLEDLYSR.Y
PRS7_MOUSE	MK_SCX_16.6021.6021.2	2	5.869	0.559	1	960.5	63.88889	K.FVVDLSDQVAPTDIEEGM*R.V
PRS7_MOUSE	MK_SCX_16.6520.6520.2	2	3.575	0.343	1	419.6	41.666664	K.FVVDLSDQVAPTDIEEGMR.V
PRS7_MOUSE	MK_SCX_19.8127.8127.2	2	2.784	0.203	1	478.9	50	R.TMLELINQLDGFDPR.G
PRS7_MOUSE	MK_SCX_20_1.5312.5312.2	2	3.189	0.41	1	1303.1	85	R.ALDEGDIALLK.T
PRS7_MOUSE	MK_SCX_26.7717.7717.2	2	4.928	0.648	1	707.4	52.380955	R.FVNLGIEPPKGVLLFGPPGTGK.T
PRS7_MOUSE	MK_SCX_28.4681.4681.2	2	5.025	0.408	1	1043.7	73.333336	K.IINADSEDPKYIINVK.Q
PRS7_MOUSE	MK_SCX_36.6212.6212.2	2	2.516	0.251	1	546.9	68.181816	R.KIEFSLPDLEGR.T
PRS7_MOUSE	MK_SCX_44.6943.6943.3	3	3.97	0.352	1	605.1	40	K.IATEKDFLEAVNKVIK.S
PRS7_MOUSE	MK_SCX_50.4162.4162.3	3	4.44	0.274	1	1934.3	53.846157	K.LREVVETPLLHPER.F
PRS7_MOUSE	MK_SCX_50.4175.4175.2	2	4.828	0.484	1	1603.9	73.07692	K.LREVVETPLLHPER.F
PRS7_MOUSE	MK_SCX_51.4222.4222.3	3	3.339	0.383	1	359.2	32.692307	R.KIATEKDFLEAVNK.V
PRS8_MOUSE	MK_SCX_19.4649.4649.2	2	4.141	0.55	1	1481.5	84.61539	K.VPDSTYEMIGGLDK.Q
PRS8_MOUSE	MK_SCX_19.8197.8197.2	2	3.8	0.516	1	1055	70	R.TMLELLNQLDGFEATK.N
PRS8_MOUSE	MK_SCX_30.7941.7941.3	3	4.629	0.386	1	1881.2	40	R.LLREELQLLQEQGSYVGEVVR.A
PRS8_MOUSE	MK_SCX_31.6223.6223.2	2	2.468	0.316	1	327.6	62.5	R.IDILDSALLRPGR.I
PRS8_MOUSE	MK_SCX_34.8211.8211.3	3	3.126	0.43	1	532.9	29.761904	K.EVIELPVKHPELFEALGIAQPK.G
PRS8_MOUSE	MK_SCX_53.4179.4179.3	3	3.743	0.543	1	1089.6	44.642857	R.RVHVTQEDFEMAVAK.V
PRVA_MOUSE	MK_SCX_19.6367.6367.2	2	4.05	0.143	1	1659.5	80.769226	K.SGFIEEDELGSILK.G
PSA1_MOUSE	MK_SCX_21.4574.4574.2	2	4.209	0.531	1	1486	75	R.NQYDNDVTVWSPQGR.I
PSA1_MOUSE	MK_SCX_31.4091.4091.3	3	3.89	0.439	1	660.6	44.642857	R.ALRETLPAEQDLTTK.N
PSA1_MOUSE	MK_SCX_32.3993.3993.2	2	4.132	0.406	1	1528.1	75	R.ALRETLPAEQDLTTK.N
PSA1_MOUSE	MK_SCX_37.3487.3487.2	2	3.639	0.275	1	1622.4	86.36364	R.IHQIEYAM*EAVK.Q
PSA1_MOUSE	MK_SCX_37.3507.3507.3	3	3.312	0.312	1	714.7	45.454548	R.IHQIEYAM*EAVK.Q
PSA1_MOUSE	MK_SCX_37.4359.4359.2	2	3.705	0.451	1	1563	90.909096	R.IHQIEYAMEAVK.Q
PSA1_MOUSE	MK_SCX_44.6439.6439.3	3	4.253	0.473	1	626.4	36.25	R.IHQIEYAMEAVKQGSATVGLK.S
PSA2_MOUSE	MK_SCX_17.6465.6465.2	2	4.652	0.458	1	929.5	50	K.LAQQYYLVYQEPIPTAQLVQR.V
PSA2_MOUSE	MK_SCX_24.13794.13794.2	2	3.307	0.403	1	458.5	47.22222	R.YNEDLELEDAIHTAILTLK.E
PSA2_MOUSE	MK_SCX_24.15424.15424.3	3	3.998	0.425	1	762.1	36.11111	R.YNEDLELEDAIHTAILTLK.E
PSA2_MOUSE	MK_SCX_38.4110.4110.2	2	2.684	0.209	1	1034.8	65.38461	K.HIGLVYSGM*GPDYR.V
PSA2_MOUSE	MK_SCX_38.4680.4680.2	2	3.272	0.655	1	851.3	69.230774	K.HIGLVYSGMGPDYR.V
PSA2_MOUSE	MK_SCX_41.8503.8503.3	3	3.214	0.349	1	728	34.210526	K.RYNEDLELEDAIHTAILTLK.E
PSA3_MOUSE	MK_SCX_2201.3313.3313.2	2	3.763	0.389	1	1890.3	86.36364	K.AVENSSTAIGIR.C

PSA3_MOUSE	MK_SCX_34.6026.6026.2	2	2.287	0.133	1	414.3	46.153847	R.SLADIAREEASNFR.S
PSA3_MOUSE	MK_SCX_38.5819.5819.2	2	3.395	0.474	1	838.3	80.769226	R.HVGMAVAGLLADAR.S
PSA3_MOUSE	MK_SCX_48.3709.3709.3	3	3.089	0.235	1	411.1	50	K.DIREEAEKYAK.E
PSA4_MOUSE	MK_SCX_20_1.2214.2214.3	3	3.099	0.386	1	387.1	50	K.KHEEEEAKAER.E
PSA4_MOUSE	MK_SCX_21.5755.5755.2	2	3.255	0.362	1	861.9	77.77778	K.LLDEVFFSEK.I
PSA5_MOUSE	MK_SCX_20_1.4253.4253.2	2	3.17	0.449	1	633	75	R.ITSPLM*EPSSIEK.I
PSA5_MOUSE	MK_SCX_20_1.6869.6869.2	2	2.4	0.297	1	626.7	77.77778	K.EELEEVIK.D
PSA5_MOUSE	MK_SCX_27.6937.6937.3	3	3.136	0.328	1	453.4	25	K.LNATNIELATVQPGQNFHMFTK.E
PSA5_MOUSE	MK_SCX_29.4076.4076.2	2	5.355	0.612	1	2130.5	69.44444	R.AIGSASEGAQSSLQEVYHK.S
PSA6_MOUSE	MK_SCX_23.4437.4437.2	2	3.161	0.327	1	957.2	87.5	R.LYQVEYAFK.A
PSA6_MOUSE	MK_SCX_33.5433.5433.2	2	3.969	0.458	1	1325.2	81.818184	K.LLDSSTVTHLFK.I
PSA6_MOUSE	MK_SCX_54.5690.5690.2	2	4.991	0.499	1	1945.5	75	K.KVPDKLLDSSTVTHLFK.I
PSA6_MOUSE	MK_SCX_54.5756.5756.3	3	3.545	0.306	1	456.7	39.0625	K.KVPDKLLDSSTVTHLFK.I
PSA7_MOUSE	MK_SCX_18.4890.4890.2	2	6.104	0.579	1	2188.2	89.28571	K.NYTDDAIETDDLTIK.L
PSA7_MOUSE	MK_SCX_26.5196.5196.2	2	4.806	0.477	1	901.1	73.07692	R.DQPLKILNPEEIEK.Y
PSA7_MOUSE	MK_SCX_28.11553.11553.3	3	3.466	0.222	1	1150.1	33.75	R.KICALDDNVCMAFAGLTADAR.I
PSA7_MOUSE	MK_SCX_28.9907.9907.2	2	2.55	0.449	1	366.1	37.5	R.KICALDDNVCMAFAGLTADAR.I
PSA7_MOUSE	MK_SCX_50.4708.4708.3	3	3.216	0.385	1	861.8	41.07143	R.RDQPLKILNPEEIEK.Y
PSA7L_MOUSE	MK_SCX_18.5630.5630.2	2	4.781	0.607	1	1331.1	80.769226	K.LTVEDPVTVEYITR.F
PSA7L_MOUSE	MK_SCX_24.7321.7321.2	2	5.104	0.645	1	1489.6	54.761906	R.AITVFSPDGHLFQVEYAQEAVK.K
PSA7L_MOUSE	MK_SCX_24.7396.7396.3	3	4.277	0.482	1	1430.7	40.476192	R.AITVFSPDGHLFQVEYAQEAVK.K
PSA7L_MOUSE	MK_SCX_34.3747.3747.2	2	4.233	0.487	1	1095.6	84.61539	R.LYQTDPSGTYHAWK.A
PSA7L_MOUSE	MK_SCX_46.4599.4599.3	3	3.114	0.441	1	334.6	25	R.LYQTDPSGTYHAWKANAIGR.S
PSB1_MOUSE	MK_SCX_18.5673.5673.2	2	5.853	0.621	1	934	63.15789	K.AGGSASAM*LQPLLDNQVGFK.N
PSB1_MOUSE	MK_SCX_18.6881.6881.2	2	4.712	0.488	1	1023.2	63.15789	K.AGGSASAMLQPLLDNQVGFK.N
PSB1_MOUSE	MK_SCX_21.4925.4925.2	2	4.194	0.567	1	809.7	73.07692	K.GAVYSFDPVGSYQR.D
PSB1_MOUSE	MK_SCX_24.15638.15638.3	3	3.442	0.363	1	383.8	27.173912	R.DSFKAGGSASAMLQPLLDNQVGFK.N
PSB1_MOUSE	MK_SCX_33.4516.4516.2	2	4.934	0.49	1	1352.7	76.92308	K.NMQNVEHVPLTLDR.A
PSB1_MOUSE	MK_SCX_33.4531.4531.3	3	3.793	0.467	1	1653.1	57.692307	K.NMQNVEHVPLTLDR.A
PSB1_MOUSE	MK_SCX_36.4172.4172.2	2	3.48	0.532	1	1630.3	90.909096	R.LVKDVFISAAER.D
PSB1_MOUSE	MK_SCX_42.5216.5216.3	3	3.892	0.222	1	946.1	36.25	K.LTDKTVIGCSGFHGDCLTLTK.I
PSB2_MOUSE	MK_SCX_29.8232.8232.2	2	2.855	0.188	1	380.1	46.42857	R.FILNLPTFSVRVIDK.D
PSB2_MOUSE	MK_SCX_43.5898.5898.3	3	4.662	0.458	1	1835.3	46.875	R.VIDKDGIHNLENIAFPK.R
PSB2_MOUSE	MK_SCX_43.5936.5936.2	2	4.652	0.622	1	1667.7	71.875	R.VIDKDGIHNLENIAFPK.R
PSB3_MOUSE	MK_SCX_18.6104.6104.2	2	3.65	0.471	1	894.3	56.666668	R.FGPYYTEPVIAGLDPK.T
PSB4_MOUSE	MK_SCX_17.5588.5588.2	2	5.527	0.57	1	1859.4	68.42105	R.VNDSTM*LGASGDYADFQYLK.Q
PSB4_MOUSE	MK_SCX_17.6129.6129.2	2	4.406	0.608	1	657.6	55.263157	R.VNDSTMLGASGDYADFQYLK.Q
PSB4_MOUSE	MK_SCX_17.9796.9796.2	2	3.483	0.368	1	590.8	55.263157	K.FDGGVVIAADMLGSYGSLAR.F
PSB4_MOUSE	MK_SCX_21.4840.4840.2	2	4.342	0.464	1	1121.9	75	R.TQNPMVTGTSVLGVK.F
PSB4_MOUSE	MK_SCX_25.6829.6829.3	3	4.426	0.527	1	1361.4	36.904762	K.QVLGQMVIDEELLGDGHSYSPR.A
PSB5_MOUSE	MK_SCX_23.4540.4540.2	2	2.707	0.221	1	696.1	66.66667	K.LLANMVYQYK.G
PSB5_MOUSE	MK_SCX_31.4092.4092.2	2	4.299	0.574	1	1736.4	76.92308	R.DAYSGGAVNLYHVR.E
PSB6_MOUSE	MK_SCX_2201.3337.3337.2	2	3.707	0.4	1	1385.1	95	R.LAAIQESGVER.Q
PSD11_MOUSE	MK_SCX_17.3358.3358.2	2	4.499	0.418	1	1604.8	83.33333	R.DIQENDEEAVQVK.E
PSD12_MOUSE	MK_SCX_23.5255.5255.2	2	2.85	0.298	1	396.2	87.5	K.LFTTMELMR.W
PSD12_MOUSE	MK_SCX_45.3698.3698.3	3	5.856	0.529	1	1595.9	43.75	R.KGSSETPATDVFSSTEEGEKR.W
PSD2_MOUSE	MK_SCX_25.3056.3056.3	3	4.26	0.446	1	956.5	37.5	R.DKTPVQSQQPSATTPSGADEK.S
PSD2_MOUSE	MK_SCX_44.4592.4592.3	3	3.669	0.49	1	656.4	37.5	K.TITGFQTHTTPVLLAHGER.A
PSD2_MOUSE	MK_SCX_49.5882.5882.3	3	3.124	0.398	1	633.9	38.235294	R.HLAGEVAKEWQELDDAEK.A
PSD3_MOUSE	MK_SCX_27.3990.3990.3	3	4.144	0.418	1	886.9	39.285713	K.AKPPPGGEQEPPPPAPQDVEMK.E
PSD4_MOUSE	MK_SCX_16.5118.5118.2	2	6.068	0.611	1	597.3	43.75	R.AAAASAAEAGIATPGTEDSDDALLK.M

PSD4_MOUSE	MK_SCX_25.5506.5506.3	3	3.09	0.38	1	467.3	38.235294	R.IIAFVGSPVEDNEKDLVK.L
PSD4_MOUSE	MK_SCX_26.5403.5403.2	2	5.265	0.472	1	1219.5	67.64706	R.IIAFVGSPVEDNEKDLVK.L
PSD7_MOUSE	MK_SCX_23.5095.5095.2	2	3.408	0.534	1	786.1	85	R.VVGVLLGSWQK.K
PSD7_MOUSE	MK_SCX_29.3764.3764.2	2	4.268	0.518	1	1292	87.5	R.DIKDTTVGTLSQR.I
PSD7_MOUSE	MK_SCX_42.6938.6938.3	3	5.561	0.514	1	1484.6	43.47826	K.TFEHVTSEIGAEEAEEVGVEHLLR.D
PSD7_MOUSE	MK_SCX_43.8027.8027.3	3	4.829	0.496	1	1375.4	46.875	K.VVVHPLVLLSVVDHFNR.I
PSD7_MOUSE	MK_SCX_43.8133.8133.2	2	2.476	0.519	1	645.1	53.125	K.VVVHPLVLLSVVDHFNR.I
PSD9_MOUSE	MK_SCX_20_1.4053.4053.2	2	3.516	0.238	1	1039.5	77.27273	R.AAAVSDIQDLM*R.R
PSD9_MOUSE	MK_SCX_2201.3448.3448.2	2	3.044	0.463	1	1101	83.33333	K.ANYDVLESQK.G
PSDE_MOUSE	MK_SCX_14.9744.9744.2	2	4.113	0.581	1	659.5	37.878788	R.LGGGMPGLGQGPPTDAPAVDTAEQVYISSLALLK.M
PSDE_MOUSE	MK_SCX_14.9862.9862.3	3	5.276	0.562	1	806.8	25.757576	R.LGGGMPGLGQGPPTDAPAVDTAEQVYISSLALLK.M
PSDE_MOUSE	MK_SCX_15.7124.7124.2	2	3.695	0.509	1	535.4	36	R.VIDVFAM*PQSGTGVSVEAVDPVFQAK.M
PSDE_MOUSE	MK_SCX_15.7591.7591.3	3	4.119	0.516	1	901.8	34	R.VIDVFAMPQSGTGVSVEAVDPVFQAK.M
PSDE_MOUSE	MK_SCX_27.4331.4331.3	3	4.152	0.432	1	1372.1	46.666668	K.AVEEEDKMTPEQLAIK.N
PSDE_MOUSE	MK_SCX_27.4336.4336.2	2	5.219	0.465	1	1830.3	73.333336	K.AVEEEDKMTPEQLAIK.N
PSIP1_MOUSE	MK_SCX_29.5659.5659.3	3	3.087	0.362	1	892.4	55	R.DFKPGDLIFAK.M
PSIP1_MOUSE	MK_SCX_29.5722.5722.2	2	2.437	0.342	1	422.5	75	R.DFKPGDLIFAK.M
PSME1_MOUSE	MK_SCX_15.10028.10028.2	2	2.547	0.297	1	456	44.444447	K.DVTEQLNLVTTWLQLQIPR.I
PSME1_MOUSE	MK_SCX_20_1.4003.4003.2	2	3.218	0.284	1	978.9	65.38461	R.IEDGNNFGVAVQEK.V
PSME1_MOUSE	MK_SCX_2201.5564.5564.2	2	3.514	0.368	1	1294.2	80	K.TENLLGSYFPK.K
PSME1_MOUSE	MK_SCX_31.4796.4796.3	3	4.721	0.375	1	1737.7	46.42857	R.QLVHELDEAEYQEIR.L
PSME1_MOUSE	MK_SCX_40.8842.8842.3	3	3.995	0.468	1	866.8	36.363636	K.KISELDAFLKEPALNEANLSNLK.A
PSME3_MOUSE	MK_SCX_19.7630.7630.2	2	3.359	0.408	1	1118.7	64.28571	R.ITSEAEDLVANFFPK.K
PSMF1_MOUSE	MK_SCX_25.5991.5991.3	3	3.787	0.388	1	1127.1	37.5	R.VLIDPSSGLPNRLPPGAVPPGAR.F
PTBP1_MOUSE	MK_SCX_17.7736.7736.3	3	4.532	0.428	1	1046.2	37.5	R.IAIPGLAGAGNSVLLVSNLNPER.V
PTBP1_MOUSE	MK_SCX_17.7783.7783.2	2	6.397	0.633	1	1675.5	72.72727	R.IAIPGLAGAGNSVLLVSNLNPER.V
PTBP1_MOUSE	MK_SCX_18.11802.11802.2	2	2.633	0.149	1	467.6	55.88235	R.VTPQSLFILFGVYGDVQR.V
PTBP1_MOUSE	MK_SCX_2201.7975.7975.2	2	5.425	0.51	1	627.8	46.153847	K.NFQNIFPPSATLHLSNIPPSVSEDDLK.S
PTBP1_MOUSE	MK_SCX_2201.8046.8046.3	3	4.66	0.302	1	738.6	30.769232	K.NFQNIFPPSATLHLSNIPPSVSEDDLK.S
PTBP1_MOUSE	MK_SCX_26.12627.12627.3	3	3.687	0.407	1	347.5	22.413794	K.GKNQAFIEMNTEEAANTMVNYYTSVAPVLR.G
PTBP1_MOUSE	MK_SCX_28.7438.7438.2	2	5.422	0.676	1	2005.6	68.42105	R.KLPSDVTEGEVISLGLPFGK.V
PTD4_MOUSE	MK_SCX_19.9118.9118.2	2	4.234	0.61	1	2451.9	85.71429	K.IPAFLNVVDIAGLVK.G
PTER_MOUSE	MK_SCX_19.5428.5428.2	2	4.968	0.465	1	1917.8	85.71429	K.VQTVLGLVEPSQLGR.T
PTER_MOUSE	MK_SCX_21.3498.3498.2	2	3.023	0.342	1	1153.8	85	R.ILQEAGADISK.T
PTER_MOUSE	MK_SCX_23.10612.10612.3	3	4.124	0.582	1	375.2	28.125	R.AM*SVEQLTDVLINEILHGADGTSIK.C
PTER_MOUSE	MK_SCX_36.4150.4150.3	3	3.781	0.339	1	1122.4	52.272724	R.VHFLVDEGYEDR.I
PTER_MOUSE	MK_SCX_36.4158.4158.2	2	3.956	0.55	1	1512.4	86.36364	R.VHFLVDEGYEDR.I
PTER_MOUSE	MK_SCX_52.4484.4484.3	3	3.192	0.294	1	842.5	38.333332	K.YGGHGYSHILTNIVPK.M
PTER_MOUSE	MK_SCX_52.4495.4495.2	2	4.543	0.634	1	860.8	70	K.YGGHGYSHILTNIVPK.M
PTER_MOUSE	MK_SCX_55.4023.4023.3	3	4.314	0.551	1	2052.2	54.166668	R.RVHFLVDEGYEDR.I
PTH2_MOUSE	MK_SCX_25.9652.9652.2	2	4.328	0.657	1	652	47.5	R.TVLGIGPGPVELIDEVTGHLK.L
PTH2_MOUSE	MK_SCX_28.7379.7379.3	3	4.283	0.348	1	1001.2	51.785713	K.APDEDTLIQLLTHAK.T
PTH2_MOUSE	MK_SCX_37.8318.8318.3	3	3.485	0.221	1	678.7	37.5	K.VVVKAPDEDTLIQLLTHAK.T
PTMS_MOUSE	MK_SCX_23.3171.3171.2	2	3.861	0.501	1	1500.1	85	K.SVEAAAELSAK.D
PTN13_MOUSE	MK_SCX_23.5630.5630.2	2	2.046	0.154	1	482.1	65	K.TVTGRTSITPR.K
PTN13_MOUSE	MK_SCX_27.4968.4968.2	2	2.091	0.198	1	717.1	57.692307	K.TWASSMDLLCAANR.D
PTN13_MOUSE	MK_SCX_31.2567.2567.3	3	3.635	0.375	1	985.3	45.588234	R.ERDPAGPQSPPPDQDAQR.Q
PTN13_MOUSE	MK_SCX_9.9027.9027.2	2	2.906	0.159	1	501.4	32.5	K.RFESSSGLPGVDETGQTRPSR.Q
PTPA_MOUSE	MK_SCX_19.9655.9655.3	3	3.159	0.346	1	572.3	26.785713	K.LDQEAENLVATVVPTHLAAAVPEVAVYLK.E
PTPA_MOUSE	MK_SCX_20_1.6165.6165.2	2	2.599	0.411	1	830.4	77.77778	K.LVALLDTLDR.W
PTPRK_MOUSE	MK_SCX_18.4086.4086.2	2	3.125	0.364	1	1049.6	64.28571	K.AAATEEPEVIPDPAK.Q

PTPRK_MOUSE	MK_SCX_2201.5794.5794.2	2	2.341	0.214	1	725.7	68.181816	K.GLNPGTLNILVR.V
PTPRK_MOUSE	MK_SCX_25.5362.5362.2	2	2.983	0.382	1	434.4	72.22222	K.GFWNPPLAPR.K
PTPRK_MOUSE	MK_SCX_33.5727.5727.3	3	3.248	0.27	1	459.3	47.916664	K.LWHLDPDTEYEIR.V
PTPS_MOUSE	MK_SCX_37.3842.3842.3	3	3.996	0.408	1	1649.1	56.25	R.LHSPSLSDEENLR.V
PTRF_MOUSE	MK_SCX_17.4244.4244.2	2	5.071	0.411	1	1543.4	76.47059	K.ATEMVEVGPEDDEVGAER.G
PTRF_MOUSE	MK_SCX_18.5767.5767.2	2	5.296	0.602	1	1801	73.52941	K.IIGAVDQIQLTQAQLEER.Q
PTRF_MOUSE	MK_SCX_19.4246.4246.2	2	3.796	0.463	1	830.9	62.5	R.QAEM*EGAVQSIQGELSK.L
PTRF_MOUSE	MK_SCX_19.5056.5056.2	2	5.868	0.51	1	2155.8	78.125	R.QAEMEGAVQSIQGELSK.L
PTRF_MOUSE	MK_SCX_31.4727.4727.2	2	4.238	0.405	1	1607.3	79.16667	K.VMIYQDEVKLPAK.L
PTRF_MOUSE	MK_SCX_38.4483.4483.2	2	3.425	0.324	1	1610.3	90	K.KLEVNEAELLR.R
PUM1_MOUSE	MK_SCX_25.4617.4617.3	3	4.712	0.492	1	1598.7	47.22222	R.DSAWGTSDHSVSQPIMVQR.R
PUR2_MOUSE	MK_SCX_15.9199.9199.2	2	4.583	0.629	1	1296.3	54.000004	K.IELVVVGPEAPLAAGIVGDLTSAGVR.C
PUR9_MOUSE	MK_SCX_17.4824.4824.2	2	4.211	0.551	1	709.9	62.5	R.EVSDGIVAPGYEEEALK.I
PURA_MOUSE	MK_SCX_14.6176.6176.2	2	5.133	0.536	1	1264.2	52.083332	K.LIDDYGVEEEPAELPEGTSLTVDNK.R
PURA_MOUSE	MK_SCX_17.6703.6703.2	2	6.516	0.66	1	1056	61.363636	R.GPGLGSTQGQTIALPAQGLIEFR.D
PURA_MOUSE	MK_SCX_18.8214.8214.2	2	4.66	0.474	1	751.2	37.5	R.DYLGDFIEHYAQLGPSQPPDLAQAQDEPR.R
PURA_MOUSE	MK_SCX_21.6077.6077.2	2	5.311	0.64	1	1051	50	K.LIDDYGVEEEPAELPEGTSLTVDNKR.F
PURA_MOUSE	MK_SCX_2201.6032.6032.3	3	5.371	0.554	1	987.8	33	K.LIDDYGVEEEPAELPEGTSLTVDNKR.F
PURA_MOUSE	MK_SCX_24.4836.4836.2	2	3.118	0.373	1	684.3	87.5	R.FFFDVGSNK.Y
PURA_MOUSE	MK_SCX_26.6801.6801.3	3	6.754	0.616	1	1159.4	37.962963	R.QTVNRGPGLGSTQGQTIALPAQGLIEFR.D
PURA_MOUSE	MK_SCX_26.8000.8000.3	3	4.991	0.629	1	1739.3	31.034481	R.DYLGDFIEHYAQLGPSQPPDLAQAQDEPRR.A
PURA_MOUSE	MK_SCX_34.6797.6797.2	2	3.721	0.51	1	563.8	75	R.FFFDVGSNKYGVFM*R.V
PURA_MOUSE	MK_SCX_38.4786.4786.2	2	3.169	0.323	1	928.9	81.818184	R.NSITVPYKVWAK.F
PURA_MOUSE	MK_SCX_42.6698.6698.3	3	7.721	0.717	1	830.1	32.75862	R.IRQTVNRGPGLGSTQGQTIALPAQGLIEFR.D
PURA_MOUSE	MK_SCX_47.4836.4836.2	2	3.266	0.296	1	761	77.77778	K.RFFFDVGSNK.Y
PURA2_MOUSE	MK_SCX_17.6971.6971.2	2	4.088	0.521	1	558.2	47.5	R.VGIGAFPTEQDNEIGELLQTR.G
PURA2_MOUSE	MK_SCX_20_1.6055.6055.2	2	3.236	0.236	1	1175.2	80	R.FIEDELQIPVK.W
PURA2_MOUSE	MK_SCX_37.5012.5012.3	3	3.001	0.285	1	823.2	46.153847	R.TFKELPVNAQNYVR.F
PURB_MOUSE	MK_SCX_17.7343.7343.2	2	5.15	0.545	1	539.2	36.666668	R.GGGGFGGGPGPGGLQSGQTIALPAQGLIEFR.D
PURB_MOUSE	MK_SCX_17.7410.7410.3	3	5.167	0.397	1	1033.5	28.333334	R.GGGGFGGGPGPGGLQSGQTIALPAQGLIEFR.D
PURB_MOUSE	MK_SCX_18.6601.6601.3	3	8.057	0.638	1	1862	30.357143	K.LIDDYGGDEDELAGGPGGGAGGPGGGLYGELPEGTSITVDSKR.F
PURB_MOUSE	MK_SCX_19.8141.8141.3	3	5.612	0.614	1	1199.3	32.258064	R.DSLGDFIEHYAQLGPSSPEQLAAGAEEGGGPR.R
PURB_MOUSE	MK_SCX_19.8151.8151.2	2	4.971	0.66	1	724.7	35.48387	R.DSLGDFIEHYAQLGPSSPEQLAAGAEEGGGPR.R
PURB_MOUSE	MK_SCX_21.3224.3224.2	2	3.629	0.609	1	612	42.5	R.GGGGGGGGPGGEQETQELASK.R
PVRL1_MOUSE	MK_SCX_42.4106.4106.2	2	2.246	0.377	1	635	63.636364	K.RPYFTVDEAEAR.Q
PVRL1_MOUSE	MK_SCX_52.5612.5612.3	3	3.09	0.267	1	389.3	37.5	K.RVEFLRPSFIDGTIR.L
PYC_MOUSE	MK_SCX_14.3811.3811.2	2	3.61	0.43	1	1709.7	79.16667	R.ADFAQACQDAGVR.F
PYC_MOUSE	MK_SCX_17.6359.6359.2	2	5.416	0.582	1	1404.3	82.14286	K.DFTATFGPLDSLNTR.L
PYC_MOUSE	MK_SCX_18.6608.6608.2	2	3.344	0.336	1	2272.3	73.333336	K.IVGDLAQFMVQNGLSR.A
PYC_MOUSE	MK_SCX_20_1.4466.4466.2	2	4.927	0.507	1	2353.1	84.61539	R.AEAEAQAEELSFPR.S
PYC_MOUSE	MK_SCX_20_1.4561.4561.2	2	3.343	0.453	1	480.8	59.090908	K.GTPLDTEVPLER.V
PYC_MOUSE	MK_SCX_20_1.5540.5540.2	2	4.635	0.465	1	2337.2	88.46153	K.AYVEANQM*LGDLIK.V
PYC_MOUSE	MK_SCX_21.5710.5710.2	2	2.889	0.288	1	1076.4	81.818184	R.VFDYSEYWEGAR.G
PYC_MOUSE	MK_SCX_2201.3504.3504.2	2	2.1	0.148	1	621.1	75	K.ALAVSDLNR.A
PYC_MOUSE	MK_SCX_2201.4044.4044.2	2	2.118	0.387	1	390.2	72.22222	R.FIGPSPEVVR.K
PYC_MOUSE	MK_SCX_24.6505.6505.2	2	6.084	0.595	1	1912.7	64.28571	R.LDNASAFQGAVISPHYDSLLVK.V
PYC_MOUSE	MK_SCX_24.6506.6506.3	3	5.628	0.433	1	1653.6	44.04762	R.LDNASAFQGAVISPHYDSLLVK.V
PYC_MOUSE	MK_SCX_25.8621.8621.3	3	4.351	0.454	1	1114.3	34.782608	R.HGEEVTPEDVLSAAM*YPDVFAQFK.D
PYC_MOUSE	MK_SCX_25.9381.9381.2	2	5.851	0.649	1	1766.2	58.69565	R.HGEEVTPEDVLSAAMYPDVFAQFK.D
PYC_MOUSE	MK_SCX_25.9415.9415.3	3	5.607	0.528	1	1109.6	39.130436	R.HGEEVTPEDVLSAAMYPDVFAQFK.D
PYC_MOUSE	MK_SCX_26.11027.11027.3	3	3.359	0.516	1	691.5	30.952381	R.SVVEFLQGYIGIPHGGFPEPFR.S

PYC_MOUSE	MK_SCX_26.9178.9178.2	2	4.966	0.647	1	857.5	57.14286	R.SVVEFLQGYIGIPHGGFPEPFR.S
PYC_MOUSE	MK_SCX_28.8207.8207.2	2	4.483	0.491	1	1580.9	73.333336	R.GLAPVQAYLHIPDIIK.V
PYC_MOUSE	MK_SCX_29.4924.4924.2	2	4.841	0.579	1	1041	75	K.VVEIAPATHLDPQLR.S
PYC_MOUSE	MK_SCX_31.3248.3248.2	2	4.757	0.598	1	1891.7	78.57143	R.TVAVYSEQDTGQMHR.Q
PYC_MOUSE	MK_SCX_31.4783.4783.2	2	2.671	0.298	1	431.1	57.14286	K.GQIGAPMPGKVIDIK.V
PYC_MOUSE	MK_SCX_34.6041.6041.2	2	4.723	0.499	1	516.3	75	K.KAYVEANQMLGDLIK.V
PYC_MOUSE	MK_SCX_35.4230.4230.3	3	3.797	0.208	1	1544.6	50	R.VVHSYEELEENYTR.A
PYC_MOUSE	MK_SCX_39.4756.4756.3	3	3.666	0.298	1	1123.1	50	R.NHQGLLLM*DTTFR.D
PYC_MOUSE	MK_SCX_39.6525.6525.2	2	2.505	0.418	1	557	70.83333	R.NHQGLLLMDTTFR.D
PYC_MOUSE	MK_SCX_43.5789.5789.3	3	4.413	0.515	1	1201.2	47.22222	R.IEGRPGASLPPLNLKELEK.D
PYC_MOUSE	MK_SCX_43.7928.7928.3	3	5.409	0.596	1	1661.8	50	R.HIEVQILGDQYGNILHLYER.D
PYC_MOUSE	MK_SCX_50.8128.8128.3	3	4.766	0.458	1	809.9	31.52174	R.IEGRPGASLPPLNLKELEKDLIDR.H
QKI_MOUSE	MK_SCX_18.5786.5786.2	2	4.192	0.475	1	1321.7	68.75	R.SAELPDAVGPIVQLQEK.L
QKI_MOUSE	MK_SCX_29.6731.6731.3	3	4.069	0.463	1	362.5	38.333332	K.LYVPVKEYPDFNFVGR.I
QKI_MOUSE	MK_SCX_34.5701.5701.2	2	2.134	0.161	1	343.4	78.57143	R.LLDEEISR.V
QKI_MOUSE	MK_SCX_38.4840.4840.2	2	2.13	0.182	1	439.5	53.846157	R.TPTPAGPTIM*PLIR.Q
QOR_MOUSE	MK_SCX_17.8060.8060.2	2	3.812	0.145	1	488.1	50	K.GVDVIIEM*LANENLSNDLK.L
QOR_MOUSE	MK_SCX_19.7325.7325.2	2	5.83	0.388	1	1257	73.333336	K.EEFQQFAGLLQAGIEK.G
QOR_MOUSE	MK_SCX_21.5378.5378.2	2	3.589	0.455	1	1270.4	85	R.VFEFGGPEVLK.L
QOR_MOUSE	MK_SCX_2201.10057.10057.3	3	3.503	0.379	1	581.4	25	K.ETSIIGVSLSSSTKEEFQQFAGLLQAGIEK.G
QOR_MOUSE	MK_SCX_2201.1757.1757.2	2	2.209	0.389	1	516.1	80	K.VLGTAGSEEGK.K
QOR_MOUSE	MK_SCX_26.5103.5103.3	3	3.683	0.399	1	773.1	36.764706	K.LQSDVVVPVPQSHQVLIK.V
QOR_MOUSE	MK_SCX_26.5173.5173.2	2	4.667	0.483	1	652.8	58.823532	K.LQSDVVVPVPQSHQVLIK.V
QOR_MOUSE	MK_SCX_28.7002.7002.2	2	5.259	0.64	1	1720	64.28571	R.KPALPYTPGSDVAGIIESVGDK.V
QOR_MOUSE	MK_SCX_28.7245.7245.3	3	4.705	0.492	1	1546.9	36.904762	R.KPALPYTPGSDVAGIIESVGDK.V
QOR_MOUSE	MK_SCX_33.5147.5147.2	2	4.744	0.587	1	1370.6	89.28571	K.GWVKPVIGSEYPLEK.A
QOR_MOUSE	MK_SCX_33.5148.5148.3	3	4.503	0.388	1	1906.1	53.571426	K.GWVKPVIGSEYPLEK.A
QOR_MOUSE	MK_SCX_36.10742.10742.3	3	7.889	0.599	1	1898.1	43.269234	R.KPALPYTPGSDVAGIIESVGDKVSAFK.K
QOR_MOUSE	MK_SCX_41.8996.8996.3	3	4.721	0.424	1	1824.2	36.53846	K.MSVGDKDKGVDVIIEMLANENLSNDLK.L
QOR_MOUSE	MK_SCX_44.3994.3994.2	2	2.716	0.198	1	1204.2	73.07692	K.LVLQNGAHEVFNHK.E
QOR_MOUSE	MK_SCX_49.8720.8720.3	3	6.257	0.625	1	1248	37.037037	R.KPALPYTPGSDVAGIIESVGDKVSAFKK.G
QOR_MOUSE	MK_SCX_55.3730.3730.2	2	2.835	0.146	1	550	60.714287	K.KLVLQNGAHEVFNHK.E
QOR_MOUSE	MK_SCX_56.4557.4557.3	3	4.485	0.179	1	886.6	30.952381	K.KLVLQNGAHEVFNHKEANYIDK.I
RAB10_MOUSE	MK_SCX_21.8187.8187.2	2	4.443	0.52	1	1382.6	85	K.AFLTLAEDILR.K
RAB14_MOUSE	MK_SCX_19.4901.4901.2	2	4.463	0.597	1	1145.9	76.92308	K.TGENVEDAFLEAAK.K
RAB14_MOUSE	MK_SCX_29.5064.5064.3	3	4.907	0.437	1	694.8	44.642857	K.TGENVEDAFLEAAKK.I
RAB14_MOUSE	MK_SCX_30.4983.4983.2	2	4.818	0.533	1	1049.9	67.85714	K.TGENVEDAFLEAAKK.I
RAB14_MOUSE	MK_SCX_34.4970.4970.3	3	5.798	0.588	1	1583.9	32.75862	K.IYQNIQDGSLDLNAAESGVQHKPSAPQGGR.L
RAB14_MOUSE	MK_SCX_38.5568.5568.3	3	3.026	0.3	1	430.8	40.384613	R.STYNHLSSWLTDAR.N
RAB14_MOUSE	MK_SCX_38.5648.5648.2	2	2.954	0.426	1	393.6	57.692307	R.STYNHLSSWLTDAR.N
RAB14_MOUSE	MK_SCX_51.4800.4800.3	3	7.041	0.57	1	1996.8	35.833332	K.KIYQNIQDGSLDLNAAESGVQHKPSAPQGGR.L
RAB18_MOUSE	MK_SCX_11.8115.8115.2	2	2.477	0.156	1	579.3	60.714287	K.FARKHSM*LFIEASAK.T
RAB18_MOUSE	MK_SCX_16.8047.8047.2	2	5.136	0.679	1	2171.4	75	R.FTDDTFDPELAATIGVDFK.V
RAB18_MOUSE	MK_SCX_19.6918.6918.2	2	3.384	0.196	1	1423.8	64.28571	K.TCDGVQCAFEELVEK.I
RAB1A_MOUSE	MK_SCX_18.7414.7414.2	2	5.104	0.559	1	996.4	80	K.EFADSLGIPFLETSAK.N
RAB1A_MOUSE	MK_SCX_19.4540.4540.2	2	5.336	0.498	1	2339.7	75	K.NATNVEQSFM*TM*AAEIK.K
RAB1A_MOUSE	MK_SCX_19.5224.5224.2	2	5.12	0.124	1	2077.1	71.875	K.NATNVEQSFMTM*AAEIK.K
RAB1A_MOUSE	MK_SCX_19.7478.7478.2	2	3.281	0.124	1	377.7	50	K.NATNVEQSFMTMAAEIK.K
RAB1A_MOUSE	MK_SCX_23.8924.8924.2	2	5.701	0.641	1	1514.1	56.521736	K.VVDYTTAKEFADSLGIPFLETSAK.N
RAB1A_MOUSE	MK_SCX_23.9050.9050.3	3	4.089	0.406	1	496.8	31.52174	K.VVDYTTAKEFADSLGIPFLETSAK.N
RAB1A_MOUSE	MK_SCX_36.11144.11144.3	3	6.46	0.633	1	1271.6	44.791664	K.KVVDYTTAKEFADSLGIPFLETSAK.N

RAB1B_MOUSE	MK_SCX_18.6839.6839.2	2	4.074	0.472	1	824.7	70	K.EFADSLGVPFLETSAK.N
RAB1B_MOUSE	MK_SCX_19.4872.4872.2	2	3.01	0.158	1	712.3	65.625	K.NATNVEQAFM*TM*AAEIK.K
RAB1B_MOUSE	MK_SCX_19.8142.8142.2	2	5.095	0.519	1	1480.8	71.875	K.NATNVEQAFMTMAAEIK.K
RAB1B_MOUSE	MK_SCX_23.7671.7671.2	2	3.74	0.466	1	722	45.652176	K.VVDNTTAKEFADSLGVPFLETSAK.N
RAB1B_MOUSE	MK_SCX_37.8616.8616.3	3	5.005	0.646	1	955.5	36.458336	K.KVVDNTTAKEFADSLGVPFLETSAK.N
RAB21_MOUSE	MK_SCX_36.5490.5490.2	2	5.855	0.667	1	1798.1	84.375	R.HVSIQEAESYAESVGAK.H
RAB21_MOUSE	MK_SCX_42.4723.4723.2	2	4.71	0.629	1	971.1	80.769226	K.RVNLAIWDTAGQER.F
RAB21_MOUSE	MK_SCX_44.4420.4420.2	2	2.568	0.294	1	545.5	72.22222	R.FHALGPIYYR.D
RAB24_MOUSE	MK_SCX_50.5020.5020.3	3	4.099	0.288	1	2218.4	53.571426	R.RVDFHDVQDYADNIK.A
RAB2A_MOUSE	MK_SCX_18.4998.4998.2	2	3.401	0.234	1	2003.2	80	K.IQEGVFDINNEANGIK.I
RAB2A_MOUSE	MK_SCX_21.4599.4599.2	2	3.981	0.425	1	820.7	69.230774	K.TASNVEEAFINTAK.E
RAB2A_MOUSE	MK_SCX_21.4629.4629.3	3	3.491	0.299	1	744.3	42.307693	K.TASNVEEAFINTAK.E
RAB2A_MOUSE	MK_SCX_2201.5378.5378.2	2	4.078	0.555	1	1371.3	79.16667	K.LQIWDTAGQESFR.S
RAB2A_MOUSE	MK_SCX_28.7659.7659.2	2	4.52	0.459	1	1291.3	58.333332	K.TASNVEEAFINTAKEIYEK.I
RAB3I_MOUSE	MK_SCX_17.4813.4813.2	2	5.981	0.589	1	1116.9	60.526318	K.TLVLSSSPTSPTQEPLAAAK.T
RAB3I_MOUSE	MK_SCX_20_1.6429.6429.2	2	4.13	0.366	1	1614.6	81.818184	K.IDVLQAEVAALK.T
RAB4B_MOUSE	MK_SCX_2201.4842.4842.2	2	3.284	0.468	1	688.2	70.83333	R.MGSGIQYGDISLR.Q
RAB5A_MOUSE	MK_SCX_24.15316.15316.2	2	2.072	0.132	1	529.5	63.636364	R.GVDLTEPAQPAR.S
RAB5A_MOUSE	MK_SCX_45.3873.3873.3	3	3.118	0.167	1	1092	61.11111	R.YHSLAPMYYR.G
RAB5A_MOUSE	MK_SCX_45.3886.3886.2	2	2.588	0.343	1	793	77.77778	R.YHSLAPMYYR.G
RAB5B_MOUSE	MK_SCX_20_1.8516.8516.2	2	3.756	0.399	1	1412.8	69.230774	K.TAMNVNDLFLAIAK.K
RAB5C_MOUSE	MK_SCX_20_1.8211.8211.2	2	4.083	0.439	1	1350.2	73.07692	K.TAMNVNEIFM*AIAK.K
RAB5C_MOUSE	MK_SCX_20_1.8602.8602.2	2	3.139	0.3	1	1400.9	76.92308	K.TAMNVNEIFMAIAK.K
RAB6A_MOUSE	MK_SCX_20_1.3383.3383.2	2	3.442	0.446	1	559.6	73.07692	R.VAAALPGM*ESTQDR.S
RAB6A_MOUSE	MK_SCX_20_1.3954.3954.2	2	2.34	0.233	1	406.6	57.692307	R.VAAALPGMESTQDR.S
RAB7_MOUSE	MK_SCX_15.4716.4716.2	2	2.186	0.135	1	369.4	75	K.NNIPYFETSAK.E
RAB7_MOUSE	MK_SCX_17.6314.6314.2	2	4.803	0.446	1	1259.2	83.33333	R.DPENFPFVVLGNK.I
RAB7_MOUSE	MK_SCX_18.7326.7326.2	2	2.055	0.307	1	505.1	38.235294	R.GADCCVLVFDVTAPNTFK.T
RAB7_MOUSE	MK_SCX_20_1.7048.7048.2	2	4.855	0.49	1	1591.6	76.92308	K.EAINVEQAFQTIAR.N
RAB7_MOUSE	MK_SCX_21.4254.4254.2	2	2.377	0.355	1	645.5	70	K.VIILGDSGVGK.T
RAB7_MOUSE	MK_SCX_2201.4284.4284.2	2	2.331	0.525	1	765.9	77.77778	K.ATIGADFLTK.E
RAB7_MOUSE	MK_SCX_23.7790.7790.3	3	4.762	0.541	1	662.9	36.11111	R.DPENFPFVVLGNKIDLENR.Q
RAB7_MOUSE	MK_SCX_25.5279.5279.2	2	2.978	0.235	1	921.2	88.88889	R.FQSLGVAFYR.G
RAB7_MOUSE	MK_SCX_31.6744.6744.3	3	3.357	0.227	1	927.8	38.333332	K.TLDSWRDEFLIQASPR.D
RAB8A_MOUSE	MK_SCX_21.7523.7523.2	2	4.82	0.503	1	2061.4	84.61539	K.ANINVENAFFTLAR.D
RAB8A_MOUSE	MK_SCX_21.7603.7603.3	3	3.361	0.399	1	1125.7	50	K.ANINVENAFFTLAR.D
RAB8A_MOUSE	MK_SCX_34.2788.2788.2	2	3.11	0.445	1	1329.1	77.27273	R.NIEEHASADVEK.M
RAC1_MOUSE	MK_SCX_14.4277.4277.2	2	3.384	0.27	1	981.6	83.33333	K.YLECSALTQR.G
RAC1_MOUSE	MK_SCX_19.4898.4898.2	2	3.399	0.425	1	392.4	65.38461	K.LTPITYPQGLAMAK.E
RAC1_MOUSE	MK_SCX_34.4445.4445.2	2	2.526	0.367	1	370.7	57.14286	K.KLTPITYPQGLAM*AK.E
RAC1_MOUSE	MK_SCX_34.5025.5025.2	2	3.804	0.526	1	833.4	71.42857	K.KLTPITYPQGLAMAK.E
RAC1_MOUSE	MK_SCX_34.5095.5095.3	3	4.912	0.481	1	1159.1	51.785713	K.KLTPITYPQGLAMAK.E
RAC1_MOUSE	MK_SCX_45.3424.3424.3	3	3.159	0.309	1	420	40.909092	K.LDLRDDKDTIEK.L
RAD17_MOUSE	MK_SCX_21.6195.6195.2	2	3.11	0.219	1	330.6	54.545456	K.DVSLFLFRALGK.I
RAD17_MOUSE	MK_SCX_60.4203.4203.2	2	2.047	0.167	1	518.2	75	R.GVMHSNKAR.G
RADI_MOUSE	MK_SCX_17.9133.9133.2	2	4.259	0.531	1	1270	71.875	K.FFPEDVSEELIQEITQR.L
RADI_MOUSE	MK_SCX_2201.2019.2019.2	2	3.269	0.342	1	1040.9	88.88889	K.AQKELEEQTR.K
RADI_MOUSE	MK_SCX_2201.3849.3849.2	2	2.567	0.134	1	678	92.85714	K.IALLEEAK.K
RADI_MOUSE	MK_SCX_2201.4367.4367.2	2	3.724	0.473	1	1295	79.16667	K.QLQALSSELAQAR.D
RADI_MOUSE	MK_SCX_29.11851.11851.2	2	3.165	0.516	1	323.2	47.22222	R.AKFFPEDVSEELIQEITQR.L
RADI_MOUSE	MK_SCX_39.4564.4564.2	2	4.258	0.537	1	1843.1	84.61539	K.KQLQALSSELAQAR.D

RADI_MOUSE	MK_SCX_39.4700.4700.3	3	3.077	0.241	1	737.7	44.230766	K.KQLQALSSELAQAR.D
RADI_MOUSE	MK_SCX_43.4543.4543.2	2	5.484	0.491	1	2566.8	76.666664	K.AFAAQEDLEKTKEELK.T
RADI_MOUSE	MK_SCX_44.4377.4377.3	3	4.458	0.47	1	562	46.666668	K.AFAAQEDLEKTKEELK.T
RADI_MOUSE	MK_SCX_53.2962.2962.3	3	4.22	0.414	1	1491.2	45.3125	R.DETKKTQNDVLHAENVK.A
RADI_MOUSE	MK_SCX_53.4275.4275.3	3	3.577	0.481	1	1081.1	40	R.VKKQLQALSSELAQAR.D
RADI_MOUSE	MK_SCX_56.3508.3508.3	3	3.99	0.37	1	1239.3	47.058823	K.YGDYNKEIHKPGYLANDR.L
RAE1L_MOUSE	MK_SCX_28.6402.6402.3	3	3.811	0.452	1	579.2	36.842106	K.MWDLNSNQAIQIAQHDAPVK.T
RAI14_MOUSE	MK_SCX_27.3973.3973.3	3	3.6	0.383	1	304.6	30.263159	K.SQMPQEAPDDSGDMKEAMNR.M
RAI14_MOUSE	MK_SCX_27.4054.4054.3	3	4.125	0.464	1	412.2	37.5	K.SSPPVEHPAGTSTTDNDVIIR.Q
RAI14_MOUSE	MK_SCX_31.4616.4616.3	3	3.742	0.37	1	986.5	41.666664	R.KAPPPPISPTQLSDVSSPR.S
RAI14_MOUSE	MK_SCX_32.4802.4802.3	3	4.678	0.584	1	942.2	46.666668	K.LGLLSQESADGYSHLR.E
RAI14_MOUSE	MK_SCX_48.3550.3550.3	3	3.191	0.461	1	557.9	37.5	K.ISALTGHLANKEAEVAK.L
RAI14_MOUSE	MK_SCX_54.5606.5606.3	3	4.318	0.475	1	1081.9	40.789474	K.KGADLSLVDSLGHNALHYSK.L
RALA_MOUSE	MK_SCX_21.4495.4495.2	2	3.265	0.232	1	921.4	65.38461	R.ADQWNVNYVETSAK.T
RALA_MOUSE	MK_SCX_2201.3366.3366.2	2	3.165	0.475	1	798.1	80	K.VIMVGSGGVGK.S
RALA_MOUSE	MK_SCX_31.5388.5388.3	3	3.265	0.335	1	845.6	41.07143	R.VKEDENVPFLLVGNK.S
RALA_MOUSE	MK_SCX_31.5415.5415.2	2	4.526	0.52	1	1395.7	75	R.VKEDENVPFLLVGNK.S
RALA_MOUSE	MK_SCX_35.11191.11191.2	2	3.375	0.569	1	1305.8	81.818184	R.ANVDKVFFDLMR.E
RALA_MOUSE	MK_SCX_35.11263.11263.3	3	4.62	0.589	1	1190.6	59.090908	R.ANVDKVFFDLMR.E
RALA_MOUSE	MK_SCX_53.6356.6356.3	3	3.658	0.318	1	936.7	44.230766	K.TRANVDKVFFDLMR.E
RALB_MOUSE	MK_SCX_35.4764.4764.3	3	3.861	0.436	1	1030.3	43.333332	R.GKAEEWGVQYVETSAK.T
RALB_MOUSE	MK_SCX_45.4820.4820.3	3	3.824	0.413	1	950.9	40	R.VKSEEDKIPLLVVGNK.S
RALB_MOUSE	MK_SCX_46.4687.4687.2	2	5.156	0.506	1	1730.3	73.333336	R.VKSEEDKIPLLVVGNK.S
RALY_MOUSE	MK_SCX_17.3809.3809.2	2	5.387	0.593	1	1050	57.14286	R.LPAPQEDTASEAGTPQGEVQTR.D
RALY_MOUSE	MK_SCX_24.4305.4305.2	2	3.349	0.56	1	1415.8	80	K.GYAFVQYANER.H
RAN_MOUSE	MK_SCX_36.8006.8006.2	2	4.136	0.523	1	1461.3	76.92308	K.SNYNFEKPFLWLAR.K
RAN_MOUSE	MK_SCX_36.8134.8134.3	3	4.797	0.551	1	1233.6	55.76923	K.SNYNFEKPFLWLAR.K
RANB3_MOUSE	MK_SCX_16.3094.3094.2	2	3.205	0.254	1	732.6	60.714287	R.LNDM*ASTDDGTLQSR.L
RANB3_MOUSE	MK_SCX_16.3603.3603.2	2	3.916	0.515	1	1544.3	78.57143	R.LNDMASTDDGTLQSR.L
RANB3_MOUSE	MK_SCX_41.3583.3583.3	3	4.664	0.5	1	689.1	36.11111	R.SPSESAEETHTLEEKVPQK.T
RANB3_MOUSE	MK_SCX_49.2623.2623.3	3	3.27	0.28	1	1154.5	40.789474	R.SRAEQEQEAKAPPPEPGATR.A
RANG_MOUSE	MK_SCX_19.4697.4697.2	2	2.27	0.409	1	577.7	75	K.TLEEDEEELFK.M
RANG_MOUSE	MK_SCX_23.4493.4493.2	2	3.554	0.308	1	702.4	75	R.FASENDLPEWK.E
RANG_MOUSE	MK_SCX_36.4191.4191.3	3	3.129	0.362	1	406.4	45.833336	R.FASENDLPEWKER.G
RANG_MOUSE	MK_SCX_37.3827.3827.2	2	2.798	0.408	1	1082	85	K.VAEKLEALSVR.E
RANT_MOUSE	MK_SCX_35.5745.5745.3	3	3.595	0.29	1	1070.5	42.857143	R.GPIKFNVWDTAGQEK.F
RANT_MOUSE	MK_SCX_36.7345.7345.2	2	2.296	0.214	1	322.7	53.846157	R.SNYNFEKPFFWLAR.K
RANT_MOUSE	MK_SCX_36.7356.7356.3	3	3.444	0.406	1	1031.9	51.923077	R.SNYNFEKPFFWLAR.K
RAP1A_MOUSE	MK_SCX_21.8971.8971.3	3	3.13	0.156	1	714.4	45.454548	K.INVNEIFYDLVR.Q
RAP1A_MOUSE	MK_SCX_21.9030.9030.1	1	3.605	0.345	1	401.5	68.181816	K.INVNEIFYDLVR.Q
RAP1A_MOUSE	MK_SCX_21.9042.9042.2	2	4.962	0.512	1	1742.8	90.909096	K.INVNEIFYDLVR.Q
RAP1A_MOUSE	MK_SCX_2201.3787.3787.2	2	2.884	0.204	1	771.3	70	K.LVVLGSGGVGK.S
RAP1A_MOUSE	MK_SCX_31.4903.4903.3	3	3.122	0.204	1	1058	46.42857	R.VKDTEDVPMILVGNK.C
RAP1A_MOUSE	MK_SCX_36.8124.8124.3	3	3.728	0.418	1	1395.3	50	K.SKINVNEIFYDLVR.Q
RAP1A_MOUSE	MK_SCX_36.9490.9490.2	2	4.22	0.561	1	1493.9	73.07692	K.SKINVNEIFYDLVR.Q
RASF8_MOUSE	MK_SCX_16.3405.3405.2	2	2.027	0.224	1	327.3	46.666668	R.TGPSLSERPTSDSVAR.I
RASF8_MOUSE	MK_SCX_23.13814.13814.3	3	3.108	0.201	1	311.7	22.826088	R.TGPSLSERPTSDSVARIPERTLYR.Q
RASH_MOUSE	MK_SCX_2201.3556.3556.2	2	3.23	0.363	1	1213.2	90	K.LVVVGAGGVGK.S
RB11A_MOUSE	MK_SCX_20_1.15674.15674.2	2	3.293	0.493	1	1073.3	66.66667	R.GAVGALLVYDIAK.H
RB11A_MOUSE	MK_SCX_2201.3649.3649.2	2	2.756	0.403	1	1307.3	85	K.AQIWDTAGQER.Y
RB11A_MOUSE	MK_SCX_23.3938.3938.2	2	3.57	0.435	1	1319.5	94.44444	K.STIGVEFATR.S

RB11A_MOUSE	MK_SCX_43.3778.3778.2	2	2.943	0.251	1	1050.9	90	R.FTRNEFNLESK.S
RB11B_MOUSE	MK_SCX_18.6209.6209.2	2	2.702	0.149	1	311.7	37.5	K.NILTEIYRIVSQK.Q
RB11B_MOUSE	MK_SCX_18.7484.7484.2	2	3.927	0.516	1	580.6	57.5	K.NNLSFIETSALDSTNVEEAFK.N
RBBP9_MOUSE	MK_SCX_51.5548.5548.3	3	3.95	0.516	1	965.2	45	R.GHFQNTEFHELISVVK.S
RBBP9_MOUSE	MK_SCX_51.5564.5564.2	2	5.269	0.559	1	1576.8	76.666664	R.GHFQNTEFHELISVVK.S
RBM14_MOUSE	MK_SCX_24.3705.3705.2	2	2.237	0.467	1	361.3	59.090908	R.AQPSASLGVGYR.T
RBM14_MOUSE	MK_SCX_25.3959.3959.2	2	3.072	0.506	1	971.1	88.88889	R.YSGSYNDYLR.A
RBM14_MOUSE	MK_SCX_25.4112.4112.2	2	2.778	0.408	1	1099.2	93.75	R.LSESQLSFR.R
RBM14_MOUSE	MK_SCX_29.4171.4171.3	3	4.918	0.507	1	989.9	40.217392	R.TQSSASLAASYAAQQHPQAAASYR.G
RBM3_MOUSE	MK_SCX_30.6387.6387.3	3	4.35	0.466	1	1318.3	50	R.GFGFITFTNPEHASDAMR.A
RBM3_MOUSE	MK_SCX_30.6391.6391.2	2	5.186	0.623	1	2732.7	76.47059	R.GFGFITFTNPEHASDAMR.A
RBM3_MOUSE	MK_SCX_41.3204.3204.3	3	3.624	0.424	1	887.1	48.076923	R.YDSRPGGYGYGYGR.S
RBM3_MOUSE	MK_SCX_50.5938.5938.3	3	4.648	0.451	1	3410.6	52.63158	R.SRGFGFITFTNPEHASDAMR.A
RBM4_MOUSE	MK_SCX_19.4134.4134.2	2	4.67	0.558	1	1506	70	R.VADLTEQYNEQYGAVR.T
RBM4B_MOUSE	MK_SCX_19.4258.4258.2	2	4.26	0.463	1	880.5	60.000004	R.VADFTEQYNEQYGAVR.T
RBM8A_MOUSE	MK_SCX_31.3253.3253.3	3	5.065	0.396	1	1162.5	45.833336	R.M*REDYDSVEQDGDEPGPQR.S
RBM8A_MOUSE	MK_SCX_31.3413.3413.3	3	6.199	0.529	1	1666.7	50	R.MREDYDSVEQDGDEPGPQR.S
RBM8A_MOUSE	MK_SCX_31.3446.3446.2	2	5.289	0.607	1	1836.5	75	R.MREDYDSVEQDGDEPGPQR.S
RBP2_MOUSE	MK_SCX_14.4826.4826.2	2	6.416	0.568	1	1393.2	48.148148	R.TDLTQGDEVIDTTSEAGETSSTSETTPK.A
RBP2_MOUSE	MK_SCX_17.2834.2834.2	2	6.365	0.62	1	1206.2	59.523808	K.VTDEENASSGADAPSASDTTAK.Q
RBP2_MOUSE	MK_SCX_27.9155.9155.3	3	3.018	0.164	1	352.5	22.916668	K.KFEDSQQNIIKLQNGHTSLAAELSK.D
RBP2_MOUSE	MK_SCX_31.5468.5468.3	3	3.243	0.33	1	473.4	38.333332	K.AVVSPPKFVFGSESVK.S
RBP2_MOUSE	MK_SCX_38.4548.4548.3	3	3.5	0.356	1	816.5	55	R.FKTPEEAALFK.C
RBP2_MOUSE	MK_SCX_42.7004.7004.2	2	2.13	0.148	1	377.9	66.66667	R.LLMRREQVLK.I
RBP2_MOUSE	MK_SCX_44.4205.4205.3	3	3.076	0.238	1	406.4	33.92857	K.DSLITPHVSHLSTPR.E
RBPMS_MOUSE	MK_SCX_31.6894.6894.2	2	3.266	0.464	1	777.7	65.38461	R.TLFVSGLPLDIKPR.E
RBPMS_MOUSE	MK_SCX_33.4485.4485.3	3	3.002	0.184	1	544.4	42.857143	K.LTSKQPVGFVSFDSR.S
RCC1_MOUSE	MK_SCX_19.5348.5348.2	2	2.767	0.412	1	1047.3	71.42857	K.SMVPVQVQLDAPVVK.V
RCC1_MOUSE	MK_SCX_23.4334.4334.2	2	4.318	0.414	1	860.9	52.77778	R.DTSVEGSEMVPGKVELQEK.V
RCC1_MOUSE	MK_SCX_25.13988.13988.3	3	6.453	0.666	1	654.5	31.25	R.SHNTEPGLVLTLGQGDVGQLGLGESVLER.K
RCL_MOUSE	MK_SCX_30.3866.3866.3	3	4.408	0.518	1	1389	55.76923	K.VLTEHVADAELEPR.G
RCL_MOUSE	MK_SCX_30.3871.3871.2	2	4.931	0.591	1	1663.8	76.92308	K.VLTEHVADAELEPR.G
RCN1_MOUSE	MK_SCX_17.4361.4361.2	2	5.424	0.569	1	2310.9	89.28571	R.IDSDGDGLVTTEELK.L
RCN2_MOUSE	MK_SCX_25.6875.6875.3	3	4.577	0.433	1	2155.5	43.421055	R.ADYDREALLGVQEDVDEYVK.L
RCN2_MOUSE	MK_SCX_30.5128.5128.2	2	4.872	0.336	1	1274.8	76.666664	R.RDPTANEDPEWILVEK.D
RCN2_MOUSE	MK_SCX_43.4982.4982.3	3	5.512	0.528	1	2411.1	57.352943	R.RDPTANEDPEWILVEKDR.F
RCOR1_MOUSE	MK_SCX_18.4991.4991.2	2	4.241	0.458	1	808.8	60.000004	R.VGPQYQAAVPDFDPAK.L
RD23A_MOUSE	MK_SCX_15.9221.9221.3	3	5.208	0.586	1	910.9	32.75862	R.QVIQQNPALLPALLQQLGQENPQLLQQISR.H
RD23A_MOUSE	MK_SCX_15.9250.9250.2	2	4.356	0.573	1	974.4	37.931034	R.QVIQQNPALLPALLQQLGQENPQLLQQISR.H
RD23A_MOUSE	MK_SCX_49.3307.3307.3	3	3.306	0.446	1	387.1	36.666668	K.IEAEKGRDAFPVAGQK.L
RD23B_MOUSE	MK_SCX_17.6545.6545.3	3	4.32	0.601	1	579.9	22.826088	R.ESQAVVDPPPQAVSTGTPQSPAVAAAAATTTATTTTTSGGHPLEFLR.
RD23B_MOUSE	MK_SCX_18.8106.8106.2	2	6.348	0.672	1	1537.1	75	R.QIIQQNPSLLPALLQQIGR.E
RD23B_MOUSE	MK_SCX_20_1.4120.4120.2	2	2.385	0.304	1	767.9	77.77778	K.IDIDPEETVK.A
RD23B_MOUSE	MK_SCX_31.3741.3741.2	2	3.544	0.504	1	1636.2	86.36364	K.ILSDDTALKEYK.I
RDH14_MOUSE	MK_SCX_26.5512.5512.2	2	2.12	0.187	1	425.6	71.42857	R.VIMGCRDR.A
RDH14_MOUSE	MK_SCX_50.4951.4951.3	3	5.204	0.307	1	2014.2	50	R.RLEGTNVTVNVLHPGIVR.T
RED_MOUSE	MK_SCX_23.5500.5500.2	2	3.473	0.24	1	1312.9	93.75	K.LTQILSYLR.Q
REEP4_MOUSE	MK_SCX_15.6755.6755.2	2	4.397	0.524	1	364.5	45.238094	R.SIPDTPVPTYQDPLYLEDQVPR.R
REFP2_MOUSE	MK_SCX_37.3645.3645.2	2	3.434	0.445	1	1277.2	90	R.SLGTADVHFER.R
RENI1_MOUSE	MK_SCX_19.6034.6034.2	2	4.17	0.502	1	899.5	73.333336	K.VIFDTGSANLWVPSTK.C
REQU_MOUSE	MK_SCX_18.6940.6940.2	2	3.61	0.394	1	1119.1	70	K.EGLISQDGSSLEALLR.T

RETBP_MOUSE	MK_SCX_24.5876.5876.2	2	3.336	0.446	1	1433.6	88.88889	R.FSGLWYAIAK.K
RFIP5_MOUSE	MK_SCX_15.7421.7421.2	2	4.316	0.452	1	787.1	47.916664	K.YDLESASAILPSSALEDPELGSLGK.M
RFIP5_MOUSE	MK_SCX_17.5473.5473.2	2	5.326	0.642	1	1151.2	61.363636	K.TVTSGGIQSVLPASQLGSSVDTK.R
RFIP5_MOUSE	MK_SCX_2201.3011.3011.2	2	3.386	0.56	1	840.2	83.33333	R.TYSDEASQLR.A
RFIP5_MOUSE	MK_SCX_2201.4444.4444.2	2	2.588	0.204	1	1146.3	75	R.TSLSTALSSGLER.L
RFIP5_MOUSE	MK_SCX_2201.4946.4946.2	2	3.627	0.259	1	1230.4	94.44444	R.ATVALDEVFR.A
RFIP5_MOUSE	MK_SCX_24.7617.7617.3	3	3.981	0.387	1	514.6	31	K.KYDLESASAILPSSALEDPELGSLGK.M
RFIP5_MOUSE	MK_SCX_37.5783.5783.2	2	2.35	0.342	1	442.5	60.000004	R.SLIGVDKFLGR.A
RFIP5_MOUSE	MK_SCX_39.9121.9121.3	3	3.794	0.342	1	850.8	27.884615	K.KKYDLESASAILPSSALEDPELGSLGK.M
RFIP5_MOUSE	MK_SCX_50.7217.7217.3	3	3.29	0.241	1	884.9	41.666664	K.YYHLTHDELIGLLLQR.E
RFX1_MOUSE	MK_SCX_15.5166.5166.3	3	4.651	0.537	1	1094	28.90625	R.ASETVSEASPSSTASQTGVPTQVVQQVQGTQQR.L
RGN_MOUSE	MK_SCX_19.5120.5120.2	2	4.841	0.455	1	1466	65.625	R.YFAGTMAEETAPAVLER.H
RHG12_MOUSE	MK_SCX_14.3080.3080.2	2	4.512	0.613	1	643.9	55.88235	K.TSFSQEQSCDSAGEGSER.I
RHG12_MOUSE	MK_SCX_18.3582.3582.2	2	5.859	0.658	1	2116	68.42105	R.IQQDSESGDELSSSSTEQMR.A
RHG12_MOUSE	MK_SCX_31.7517.7517.3	3	5.418	0.561	1	1507.5	51.785713	R.LFGHFPGPEFLDIEK.T
RHG18_MOUSE	MK_SCX_15.9917.9917.2	2	4.879	0.566	1	934.7	44.230766	K.EAGLSNLFGESIDDPQESILFLSTLTR.T
RHG18_MOUSE	MK_SCX_16.9343.9343.2	2	4.34	0.467	1	956.8	67.64706	R.DSGLFGIPLTILLEQDQR.K
RHG18_MOUSE	MK_SCX_2201.4143.4143.2	2	4.303	0.355	1	1289.4	83.33333	R.FLSQESGVAQTLK.K
RHOA_MOUSE	MK_SCX_14.4465.4465.2	2	2.593	0.293	1	570.4	72.72727	R.IGAFGYM*ECSAK.T
RHOA_MOUSE	MK_SCX_14.5213.5213.2	2	3.374	0.489	1	1309.5	86.36364	R.IGAFGYMECSAK.T
RHOA_MOUSE	MK_SCX_36.5907.5907.3	3	3.054	0.279	1	822.6	42.857143	K.HFCPNVPIILVGNKK.D
RHOA_MOUSE	MK_SCX_52.4251.4251.3	3	3.347	0.431	1	458.8	42.307693	K.TKDGVREVFEMATR.A
RHOG_MOUSE	MK_SCX_32.3630.3630.3	3	5.369	0.513	1	965.7	41.666664	R.LKEQGQAPITPQQGQALAK.Q
RIB1_MOUSE	MK_SCX_19.6201.6201.2	2	4.515	0.428	1	1260.3	71.42857	R.ASSFVLALEPELESR.L
RIB1_MOUSE	MK_SCX_21.4455.4455.2	2	3.023	0.45	1	822.1	72.72727	K.NIQVDSPYDISR.A
RIB1_MOUSE	MK_SCX_23.7323.7323.2	2	2.991	0.504	1	384.8	35.714287	R.SEDVLDYGPFKDIPAYSQDTFK.V
RIB1_MOUSE	MK_SCX_26.8376.8376.3	3	5.263	0.625	1	1612	41.666664	K.THYIVGYNLPSYEYLYNLGDQYALK.M
RIB1_MOUSE	MK_SCX_27.6518.6518.2	2	2.598	0.238	1	758.8	92.85714	R.FPLFGGWK.T
RIB1_MOUSE	MK_SCX_31.3794.3794.3	3	3.319	0.384	1	683.8	39.0625	K.VTAEVVLVHPGGGSTSR.A
RIB1_MOUSE	MK_SCX_34.3695.3695.3	3	4.579	0.577	1	2198.8	53.571426	R.YDYQRQPDSGISSIR.S
RIB1_MOUSE	MK_SCX_34.3723.3723.2	2	3.232	0.347	1	645.1	64.28571	R.YDYQRQPDSGISSIR.S
RILP_MOUSE	MK_SCX_18.3548.3548.2	2	5.138	0.496	1	862.8	57.894737	R.GETEAPEAETSNPASSSLQK.G
RIN3_MOUSE	MK_SCX_21.4386.4386.2	2	2.662	0.268	1	398.7	46.153847	R.VSLESQNVGTSTDR.D
RIN3_MOUSE	MK_SCX_2201.8599.8599.3	3	3.459	0.258	1	453.2	24.074074	R.DHSGISRTASLNLPPQSTVSSLGDRPPR.T
RINI_MOUSE	MK_SCX_18.7968.7968.2	2	2.266	0.121	1	319.8	40	K.LQLEYCNLTATSCEPLASVLR.V
RINI_MOUSE	MK_SCX_19.7374.7374.2	2	3.068	0.121	1	616.2	47.5	K.LSLQNCGLTEAGCGILPGMLR.S
RINI_MOUSE	MK_SCX_19.8160.8160.2	2	5.527	0.463	1	923.5	82.14286	R.WTELLPLIQQYEVVR.L
RIOK3_MOUSE	MK_SCX_15.3810.3810.2	2	2.295	0.133	1	438.6	68.75	K.YIKDDFRFK.D
RIOK3_MOUSE	MK_SCX_39.6942.6942.2	2	2.571	0.278	1	385.6	54.545456	K.AGIPCPTVVLLK.K
RIPK1_MOUSE	MK_SCX_15.7348.7348.2	2	3.846	0.627	1	619	37.037037	K.TPVPETNIPGSTPTMPYFSGPVADDLIK.Y
RIPK1_MOUSE	MK_SCX_2201.4099.4099.2	2	2.548	0.155	1	581.2	59.090908	K.KEYPDQSPVLQR.M
RIT2_MOUSE	MK_SCX_13.8715.8715.1	1	2.641	0.357	1	399.2	65	K.VVM*LGAGGVGK.S
RL10_MOUSE	MK_SCX_20_1.4978.4978.2	2	4.41	0.357	1	1819.2	87.5	K.FNADEFEDM*VAEK.R
RL10_MOUSE	MK_SCX_20_1.6385.6385.2	2	4.378	0.482	1	2946	91.66667	K.FNADEFEDMVAEK.R
RL10_MOUSE	MK_SCX_31.5618.5618.3	3	3.759	0.294	1	502.5	44.230766	K.FNADEFEDMVAEKR.L
RL10_MOUSE	MK_SCX_31.5636.5636.2	2	4.065	0.487	1	1619.9	76.92308	K.FNADEFEDMVAEKR.L
RL10_MOUSE	MK_SCX_41.5051.5051.2	2	3.127	0.491	1	1044.9	80	R.VHIGQVIMSIR.T
RL10_MOUSE	MK_SCX_41.5060.5060.3	3	3.222	0.17	1	739.1	47.5	R.VHIGQVIMSIR.T
RL10A_MOUSE	MK_SCX_20_1.16082.16082.2	2	2.763	0.254	1	485.4	59.090908	K.FLETVELQISLK.N
RL10A_MOUSE	MK_SCX_20_1.6333.6333.2	2	3.751	0.448	1	1498.6	81.818184	K.YDAFLASESLIK.Q
RL10A_MOUSE	MK_SCX_21.3671.3671.2	2	2.883	0.432	1	736.4	85.71429	R.DTLYEAVR.E

RL10A_MOUSE	MK_SCX_29.4490.4490.2	2	2.435	0.296	1	500.5	66.66667	K.AVDIPHM*DIEALK.K
RL10A_MOUSE	MK_SCX_29.5846.5846.2	2	3.641	0.482	1	616.6	75	K.AVDIPHMDIEALK.K
RL10A_MOUSE	MK_SCX_36.5590.5590.2	2	4.531	0.488	1	2017.9	87.5	K.KYDAFLASESLIK.Q
RL10A_MOUSE	MK_SCX_36.5614.5614.3	3	4.271	0.427	1	1901.1	58.333332	K.KYDAFLASESLIK.Q
RL10A_MOUSE	MK_SCX_40.3641.3641.2	2	3.135	0.344	1	1025.7	75	K.VSRDTLYEAVR.E
RL10A_MOUSE	MK_SCX_40.3647.3647.3	3	3.47	0.291	1	1306.9	57.5	K.VSRDTLYEAVR.E
RL10A_MOUSE	MK_SCX_43.4320.4320.2	2	3.553	0.444	1	952.5	76.92308	K.AVDIPHM*DIEALKK.L
RL10A_MOUSE	MK_SCX_43.5308.5308.2	2	3.739	0.526	1	825.8	76.92308	K.AVDIPHMDIEALKK.L
RL10A_MOUSE	MK_SCX_49.4505.4505.2	2	3.992	0.434	1	752.7	62.5	K.AGKFPSLLTHNENMVAK.V
RL10A_MOUSE	MK_SCX_49.4607.4607.3	3	3.438	0.38	1	892.9	42.1875	K.AGKFPSLLTHNENMVAK.V
RL11_MOUSE	MK_SCX_20_1.5455.5455.2	2	4.787	0.535	1	1932.3	76.92308	K.VLEQLTGQTPVFSK.A
RL11_MOUSE	MK_SCX_40.4440.4440.2	2	2.978	0.175	1	731.3	57.692307	R.KLCLNICVGESGDR.L
RL11_MOUSE	MK_SCX_43.11431.11431.3	3	4.679	0.527	1	1211.1	36.363636	R.KNNFSDTGNFGFGIQEHIDLGIK.Y
RL12_MOUSE	MK_SCX_13.3753.3753.2	2	3.228	0.486	1	625.9	57.14286	R.CTGGEVGATSALAPK.I
RL12_MOUSE	MK_SCX_13.4258.4258.2	2	4.537	0.683	1	1324.3	80	K.EILGTAQSVGCNVDGR.H
RL12_MOUSE	MK_SCX_18.5934.5934.2	2	4.688	0.474	1	956.5	73.333336	R.QAQIEVVPSASALIIK.A
RL12_MOUSE	MK_SCX_2201.3977.3977.2	2	2.448	0.428	1	410.9	81.25	K.IGPLGLSPK.K
RL12_MOUSE	MK_SCX_36.14399.14399.3	3	4.353	0.387	1	853.7	39.285713	K.HSGNITFDEIVNIAR.Q
RL12_MOUSE	MK_SCX_36.6689.6689.2	2	4.891	0.661	1	1232.8	82.14286	K.HSGNITFDEIVNIAR.Q
RL13_MOUSE	MK_SCX_20_1.4513.4513.2	2	3.097	0.417	1	655.7	58.333332	K.LATQLTGPVM*PIR.N
RL13_MOUSE	MK_SCX_20_1.5479.5479.2	2	3.672	0.483	1	1249.4	75	K.LATQLTGPVMPIR.N
RL13_MOUSE	MK_SCX_2201.2797.2797.3	3	3.396	0.219	1	1502.2	60.000004	K.STESLQANVQR.L
RL13_MOUSE	MK_SCX_23.2891.2891.2	2	4.252	0.332	1	1842.2	90	K.STESLQANVQR.L
RL13_MOUSE	MK_SCX_23.3868.3868.2	2	2.23	0.262	1	1486.5	93.75	R.TIGISVDPR.R
RL13_MOUSE	MK_SCX_25.4784.4784.2	2	2.026	0.165	1	591	85.71429	R.GFSLEELR.V
RL13_MOUSE	MK_SCX_41.6136.6136.3	3	5.567	0.548	1	1600.3	45.454548	K.KGDSSAEELKLATQLTGPVM*PIR.N
RL13_MOUSE	MK_SCX_41.6693.6693.3	3	6.18	0.51	1	2246.1	42.045452	K.KGDSSAEELKLATQLTGPVMPIR.N
RL13_MOUSE	MK_SCX_41.6720.6720.2	2	6.312	0.628	1	1871.2	59.090908	K.KGDSSAEELKLATQLTGPVMPIR.N
RL13_MOUSE	MK_SCX_46.4549.4549.3	3	3.239	0.421	1	343.7	30.882353	K.LATQLTGPVM*PIRNVYKK.E
RL13_MOUSE	MK_SCX_46.4999.4999.2	2	3.787	0.405	1	762.1	50	K.LATQLTGPVMPIRNVYKK.E
RL13_MOUSE	MK_SCX_46.5020.5020.3	3	3.419	0.437	1	567.4	35.294117	K.LATQLTGPVMPIRNVYKK.E
RL13A_MOUSE	MK_SCX_20_1.4266.4266.2	2	2.357	0.203	1	450.7	70	K.VLDGIPPPYDK.K
RL13A_MOUSE	MK_SCX_2201.3646.3646.2	2	3.424	0.525	1	1006.1	85	K.YQAVTATLEEK.R
RL13A_MOUSE	MK_SCX_34.4890.4890.2	2	4.057	0.445	1	1023.8	83.33333	R.LKVLDGIPPPYDK.K
RL13A_MOUSE	MK_SCX_49.4506.4506.2	2	4.45	0.533	1	1075.9	80.769226	R.LKVLDGIPPPYDKK.K
RL13A_MOUSE	MK_SCX_49.4539.4539.3	3	3.205	0.46	1	450.3	42.307693	R.LKVLDGIPPPYDKK.K
RL13A_MOUSE	MK_SCX_51.778.778.2	2	2.399	0.226	1	710.9	92.85714	K.RGQAALER.L
RL14_MOUSE	MK_SCX_19.5303.5303.2	2	5.123	0.483	1	2219.7	86.36364	K.LVAIVDVIDQNR.A
RL14_MOUSE	MK_SCX_37.3934.3934.2	2	3.659	0.495	1	940.9	77.27273	R.VAYISFGPHAGK.L
RL15_MOUSE	MK_SCX_21.4774.4774.2	2	5.2	0.583	1	1630.3	84.61539	R.VLNSYWVGEDSTYK.F
RL15_MOUSE	MK_SCX_43.5944.5944.2	2	3.609	0.507	1	980	80	-.GAYKYIQELWR.K
RL15_MOUSE	MK_SCX_58.6946.6946.2	2	4.627	0.517	1	911.8	70	K.GATYGKPVHHGVNQLK.F
RL15_MOUSE	MK_SCX_58.7174.7174.3	3	3.396	0.445	1	824.2	41.666664	K.GATYGKPVHHGVNQLK.F
RL17_MOUSE	MK_SCX_29.3552.3552.2	2	3.76	0.357	1	746	76.92308	K.EQIVPKPEEEVAQK.K
RL17_MOUSE	MK_SCX_38.3584.3584.3	3	3.15	0.255	1	710.6	50	-.VRYSLDPENPTK.S
RL17_MOUSE	MK_SCX_39.3609.3609.2	2	3.379	0.469	1	687.9	81.818184	-.VRYSLDPENPTK.S
RL17_MOUSE	MK_SCX_50.4794.4794.2	2	3.054	0.408	1	306.9	80	K.QWGWTQGRWPK.K
RL17_MOUSE	MK_SCX_54.3881.3881.3	3	3.322	0.236	1	480.2	45	K.KSAEFLLHM*LK.N
RL17_MOUSE	MK_SCX_54.4780.4780.2	2	4.072	0.387	1	1858.8	90	K.KSAEFLLHMLK.N
RL17_MOUSE	MK_SCX_54.4786.4786.3	3	4.281	0.382	1	969.8	57.5	K.KSAEFLLHMLK.N
RL18_MOUSE	MK_SCX_19.5813.5813.2	2	4.908	0.513	1	2428.2	87.5	K.ILTFDQLALESPK.G

RL18_MOUSE	MK_SCX_20_1.4168.4168.2	2	4.196	0.582	1	1922.7	83.33333	K.TAVVVGTVTDDVR.I
RL18_MOUSE	MK_SCX_24.3463.3463.2	2	2.625	0.214	1	892.6	87.5	R.GTVLLSGPR.K
RL18_MOUSE	MK_SCX_30.5639.5639.2	2	4.21	0.317	1	1378.1	67.85714	K.ILTFDQLALESPKGR.G
RL19_MOUSE	MK_SCX_19.4647.4647.2	2	5.694	0.555	1	1934.9	81.25	K.VWLDPNETNEIANANSR.Q
RL19_MOUSE	MK_SCX_2201.2505.2505.2	2	2.958	0.307	1	639.2	87.5	K.LLADQAEAR.R
RL19_MOUSE	MK_SCX_34.4672.4672.3	3	5.609	0.475	1	1729	47.058823	K.KVWLDPNETNEIANANSR.Q
RL19_MOUSE	MK_SCX_34.4699.4699.2	2	6.279	0.708	1	1039.4	76.47059	K.KVWLDPNETNEIANANSR.Q
RL19_MOUSE	MK_SCX_39.3936.3936.2	2	2.401	0.196	1	404.8	62.5	K.LIKDGLIIR.K
RL19_MOUSE	MK_SCX_52.4027.4027.3	3	6.635	0.624	1	1787.9	48.61111	K.KKVWLDPNETNEIANANSR.Q
RL19_MOUSE	MK_SCX_56.2524.2524.3	3	3.073	0.25	1	1356	55	R.KKLLADQAEAR.R
RL21_MOUSE	MK_SCX_32.4611.4611.2	2	4.947	0.558	1	2700.4	89.28571	R.VYNVTQHAVGIIVNK.Q
RL21_MOUSE	MK_SCX_33.4671.4671.3	3	4.243	0.604	1	1724.7	53.571426	R.VYNVTQHAVGIIVNK.Q
RL21_MOUSE	MK_SCX_41.4226.4226.2	2	3.109	0.434	1	930.6	85	K.HGVVPLATYMR.I
RL21_MOUSE	MK_SCX_42.4192.4192.3	3	3.338	0.459	1	748.7	52.499996	K.HGVVPLATYMR.I
RL22_MOUSE	MK_SCX_15.9027.9027.3	3	5.219	0.554	1	1829.4	35	K.FTLDCTHPVEDGIMDAANFEQFLQER.I
RL22_MOUSE	MK_SCX_2201.3951.3951.2	2	3.63	0.52	1	924.4	75	K.AGNLGGGVVTIER.S
RL22_MOUSE	MK_SCX_36.4054.4054.2	2	3.61	0.554	1	765.4	72.72727	K.ITVTSEVPFSKR.Y
RL22_MOUSE	MK_SCX_38.3010.3010.2	2	3.399	0.482	1	664.3	63.636364	R.VVANSKESYELR.Y
RL23_MOUSE	MK_SCX_13.7006.7006.2	2	5.604	0.594	1	1158.9	71.05263	R.ISLGLPVGAVINCADNTGAK.N
RL23_MOUSE	MK_SCX_19.4046.4046.2	2	3.723	0.544	1	1017.9	78.57143	R.LPAAGVGDM*VM*ATVK.K
RL23_MOUSE	MK_SCX_19.5284.5284.2	2	3.942	0.565	1	1689.3	82.14286	R.LPAAGVGDMVMATVK.K
RL23_MOUSE	MK_SCX_31.6362.6362.3	3	6.131	0.539	1	3239.6	55.88235	R.LNRLPAAGVGDMVMATVK.K
RL23_MOUSE	MK_SCX_31.6380.6380.2	2	5.16	0.595	1	2730	79.411766	R.LNRLPAAGVGDMVMATVK.K
RL23_MOUSE	MK_SCX_45.5134.5134.3	3	5.284	0.183	1	900.3	45.833336	R.LNRLPAAGVGDM*VMATVKK.G
RL23_MOUSE	MK_SCX_45.5920.5920.3	3	5.718	0.576	1	3334.8	55.555557	R.LNRLPAAGVGDMVMATVKK.G
RL23_MOUSE	MK_SCX_51.5907.5907.3	3	4.063	0.589	1	1007.5	40.789474	K.GRLNRLPAAGVGDMVMATVK.K
RL23A_MOUSE	MK_SCX_19.4269.4269.1	1	3.031	0.358	1	989.3	70.83333	R.LAPDYDALDVANK.I
RL23A_MOUSE	MK_SCX_19.4291.4291.2	2	4.108	0.503	1	455.6	83.33333	R.LAPDYDALDVANK.I
RL23A_MOUSE	MK_SCX_41.6296.6296.2	2	5.827	0.575	1	1610.8	70.588234	K.LDHYAIIKFPLTTESAMK.K
RL23A_MOUSE	MK_SCX_41.6298.6298.3	3	4.698	0.477	1	1962.3	50	K.LDHYAIIKFPLTTESAMK.K
RL23A_MOUSE	MK_SCX_52.5445.5445.3	3	3.909	0.521	1	388.8	31.944445	K.LDHYAIIKFPLTTESAMKK.I
RL23A_MOUSE	MK_SCX_53.5758.5758.3	3	5.132	0.499	1	972.9	36.842106	R.NKLDHYAIIKFPLTTESAM*K.K
RL23A_MOUSE	MK_SCX_53.6147.6147.3	3	4.14	0.506	1	800.2	35.526314	R.NKLDHYAIIKFPLTTESAMK.K
RL24_MOUSE	MK_SCX_20_1.4472.4472.2	2	4.912	0.601	1	1895.8	87.5	R.AITGASLADIM*AK.R
RL24_MOUSE	MK_SCX_21.5346.5346.2	2	4.499	0.641	1	2305.4	87.5	R.AITGASLADIMAK.R
RL24_MOUSE	MK_SCX_25.5572.5572.2	2	2.48	0.264	1	379.7	81.25	R.QINWTVLYR.R
RL24_MOUSE	MK_SCX_36.4418.4418.2	2	3.792	0.451	1	1374.7	90.909096	R.TDGKVFQFLNAK.C
RL24_MOUSE	MK_SCX_60.5306.5306.2	2	2.292	0.206	1	395.8	64.28571	K.RNQKPEVR.K
RL26_MOUSE	MK_SCX_43.4533.4533.3	3	3.088	0.2	1	1013.2	55	-.MKFNPFVTSDR.S
RL26_MOUSE	MK_SCX_43.4563.4563.2	2	3.948	0.355	1	1055.7	85	-.MKFNPFVTSDR.S
RL26_MOUSE	MK_SCX_56.3707.3707.3	3	4.506	0.562	1	859.7	42.5	R.EKANGTTVHVGIHPSKVVITR.L
RL26_MOUSE	MK_SCX_58.5519.5519.2	2	2.411	0.361	1	540.5	68.75	R.HFNAPSHIR.R
RL27_MOUSE	MK_SCX_2201.3931.3931.2	2	2.247	0.258	1	517.1	78.57143	K.VVLVLAGR.Y
RL27_MOUSE	MK_SCX_2201.4382.4382.2	2	2.532	0.488	1	732.6	81.25	R.YSVDIPLDK.T
RL27_MOUSE	MK_SCX_40.3959.3959.2	2	3.901	0.532	1	1728	85	K.VYNYNHLMPTR.Y
RL27_MOUSE	MK_SCX_49.4119.4119.2	2	3.874	0.45	1	511	69.230774	K.TVVNKDVFRDPALK.R
RL27_MOUSE	MK_SCX_51.5035.5035.3	3	3.118	0.312	1	388.7	29.347824	K.NIDDGTSDRPYSHALVAGIDRYPR.K
RL27A_MOUSE	MK_SCX_37.4510.4510.2	2	3.441	0.424	1	1081.5	75	K.NKTGVAPIIDVVR.S
RL27A_MOUSE	MK_SCX_53.4493.4493.2	2	4.86	0.374	1	1563.7	83.33333	R.INFDKYHPGYFGK.V
RL28_MOUSE	MK_SCX_27.4241.4241.2	2	3.445	0.342	1	383.5	50	K.TVGVEPAADGKGVVVVM*K.R
RL28_MOUSE	MK_SCX_27.4844.4844.2	2	5.114	0.589	1	1362.5	76.47059	K.TVGVEPAADGKGVVVVMK.R

RL28_MOUSE	MK_SCX_44.3770.3770.3	3	4.253	0.469	1	1418.1	47.22222	R.KTVGVEPAADGKGVVVVM*K.R
RL28_MOUSE	MK_SCX_44.4347.4347.2	2	5.573	0.5	1	1347.8	69.44444	R.KTVGVEPAADGKGVVVVMK.R
RL28_MOUSE	MK_SCX_44.4360.4360.3	3	5.186	0.592	1	882.2	44.444447	R.KTVGVEPAADGKGVVVVMK.R
RL3_MOUSE	MK_SCX_15.6802.6802.3	3	3.57	0.457	1	459.8	31.52174	R.LEQQVPVNQVFGQDEM*IDVIGVTK.G
RL3_MOUSE	MK_SCX_15.7306.7306.3	3	5.284	0.556	1	936.9	42.391304	R.LEQQVPVNQVFGQDEMIDVIGVTK.G
RL3_MOUSE	MK_SCX_15.7466.7466.2	2	5.076	0.663	1	1646.7	54.347824	R.LEQQVPVNQVFGQDEMIDVIGVTK.G
RL3_MOUSE	MK_SCX_25.15192.15192.3	3	3.952	0.42	1	345.5	29.761904	K.SINPLGGFVHYGEVTNDFIMLK.G
RL3_MOUSE	MK_SCX_26.7458.7458.3	3	6.497	0.63	1	1611.2	41	R.ERLEQQVPVNQVFGQDEMIDVIGVTK.G
RL3_MOUSE	MK_SCX_31.4420.4420.2	2	3.673	0.44	1	2819.4	86.666664	K.AHLMEIQVNGGTVAEK.L
RL3_MOUSE	MK_SCX_31.4454.4454.3	3	3.102	0.386	1	1149.4	43.333332	K.AHLMEIQVNGGTVAEK.L
RL3_MOUSE	MK_SCX_33.3445.3445.3	3	3.62	0.344	1	941.3	51.785713	K.LIKNNASTDYDLSDK.S
RL3_MOUSE	MK_SCX_39.6151.6151.3	3	3.63	0.412	1	998.1	41.666664	K.DDASKPVHLTAFLGYK.A
RL3_MOUSE	MK_SCX_50.3919.3919.3	3	3.484	0.31	1	862.5	42.1875	K.KAHLMEIQVNGGTVAEK.L
RL3_MOUSE	MK_SCX_52.5327.5327.2	2	4.849	0.684	1	1701.4	68.42105	K.SFPKDDASKPVHLTAFLGYK.A
RL3_MOUSE	MK_SCX_53.5808.5808.3	3	3.451	0.463	1	833.3	44.736843	K.SFPKDDASKPVHLTAFLGYK.A
RL3_MOUSE	MK_SCX_56.5318.5318.3	3	3.761	0.407	1	411.1	30.952381	K.VKSFPKDDASKPVHLTAFLGYK.A
RL30_MOUSE	MK_SCX_13.5166.5166.2	2	3.903	0.363	1	1513.2	81.818184	K.LVILANNCPALR.K
RL30_MOUSE	MK_SCX_37.5323.5323.3	3	3.66	0.264	1	1703.3	54.545456	R.KSEIEYYAMLAK.T
RL30_MOUSE	MK_SCX_37.5419.5419.2	2	3.541	0.329	1	1280.4	86.36364	R.KSEIEYYAMLAK.T
RL31_MOUSE	MK_SCX_18.6779.6779.2	2	3.986	0.434	1	1106	65.38461	K.LYTLVTYVPVTTFK.N
RL31_MOUSE	MK_SCX_23.7836.7836.2	2	4.877	0.636	1	1390.5	54.545456	R.NEDEDSPNKLYTLVTYVPVTTFK.N
RL31_MOUSE	MK_SCX_23.7890.7890.3	3	4.193	0.409	1	1308	36.363636	R.NEDEDSPNKLYTLVTYVPVTTFK.N
RL31_MOUSE	MK_SCX_24.3411.3411.2	2	2.996	0.279	1	1427.5	87.5	R.SAINEVVTR.E
RL32_MOUSE	MK_SCX_23.4000.4000.2	2	4.237	0.628	1	1657.5	79.16667	K.SYCAEIAHNVSSK.N
RL32_MOUSE	MK_SCX_36.5377.5377.2	2	4.133	0.327	1	1080.5	73.333336	R.FKGQILMPNIGYGSNK.K
RL32_MOUSE	MK_SCX_37.4607.4607.3	3	3.319	0.238	1	929.9	41.666664	R.FKGQILM*PNIGYGSNK.K
RL32_MOUSE	MK_SCX_37.5572.5572.3	3	3.926	0.36	1	975.1	45	R.FKGQILMPNIGYGSNK.K
RL32_MOUSE	MK_SCX_51.4021.4021.3	3	3.86	0.399	1	837.3	43.75	R.FKGQILM*PNIGYGSNKK.T
RL32_MOUSE	MK_SCX_51.4662.4662.2	2	4.963	0.364	1	1189	68.75	R.FKGQILMPNIGYGSNKK.T
RL32_MOUSE	MK_SCX_51.4690.4690.3	3	5.532	0.549	1	1752.3	56.25	R.FKGQILMPNIGYGSNKK.T
RL36_MOUSE	MK_SCX_39.3889.3889.2	2	3.317	0.352	1	446.6	68.181816	-.ALRYPM*AVGLNK.G
RL36_MOUSE	MK_SCX_39.3969.3969.3	3	3.534	0.317	1	2058.5	63.636364	-.ALRYPM*AVGLNK.G
RL36_MOUSE	MK_SCX_39.4513.4513.3	3	3.297	0.16	1	1204	52.272724	-.ALRYPMAVGLNK.G
RL36_MOUSE	MK_SCX_39.4710.4710.2	2	3.174	0.304	1	1578	86.36364	-.ALRYPMAVGLNK.G
RL38_MOUSE	MK_SCX_32.6012.6012.2	2	3.416	0.246	1	980.6	72.72727	K.IEEIKDFLLTAR.R
RL38_MOUSE	MK_SCX_50.5708.5708.3	3	4.459	0.401	1	1758.6	58.333332	R.KIEEIKDFLLTAR.R
RL38_MOUSE	MK_SCX_51.5731.5731.2	2	5.058	0.477	1	2085.2	87.5	R.KIEEIKDFLLTAR.R
RL4_MOUSE	MK_SCX_13.9142.9142.2	2	2.919	0.522	1	377.3	47.22222	R.YAICSALAASALPALVMSK.G
RL4_MOUSE	MK_SCX_17.5968.5968.2	2	3.767	0.443	1	594.2	73.07692	R.IEEVPELPLVVEDK.V
RL4_MOUSE	MK_SCX_21.3722.3722.2	2	3.642	0.524	1	1197	90	K.LEAAATALATK.S
RL4_MOUSE	MK_SCX_21.6026.6026.2	2	3.272	0.469	1	581	81.818184	R.NIPGITLLNVSK.L
RL4_MOUSE	MK_SCX_29.4937.4937.3	3	4.177	0.469	1	1216.9	36.904762	R.QPYAVSELAGHQTSAESWGTGR.A
RL4_MOUSE	MK_SCX_34.6651.6651.2	2	5.255	0.58	1	1204.6	63.15789	R.IEEVPELPLVVEDKVEGYKK.T
RL4_MOUSE	MK_SCX_38.3429.3429.2	2	4.176	0.46	1	1724.9	90.909096	K.KLEAAATALATK.S
RL4_MOUSE	MK_SCX_42.4241.4241.2	2	3.709	0.366	1	1507.4	88.88889	R.KLDELYGTWR.K
RL4_MOUSE	MK_SCX_48.4558.4558.3	3	7.909	0.646	1	2605.9	41.666664	K.NNRQPYAVSELAGHQTSAESWGTGR.A
RL4_MOUSE	MK_SCX_48.5541.5541.2	2	4.126	0.492	1	497.6	59.375	K.GHRIEEVPELPLVVEDK.V
RL4_MOUSE	MK_SCX_49.6180.6180.2	2	3.923	0.486	1	844.4	63.333332	K.APIRPDIVNFVHTNLR.K
RL4_MOUSE	MK_SCX_49.6231.6231.3	3	5.167	0.596	1	1928.2	55	K.APIRPDIVNFVHTNLR.K
RL4_MOUSE	MK_SCX_54.7970.7970.3	3	4.274	0.492	1	358.7	31	K.NVTLPAVFKAPIRPDIVNFVHTNLRK.N
RL4_MOUSE	MK_SCX_55.5636.5636.3	3	3.901	0.397	1	1404.5	48.4375	K.APIRPDIVNFVHTNLRK.N

RL4_MOUSE	MK_SCX_55.5663.5663.2	2	3.359	0.435	1	345.4	53.125	K.APIRPDIVNFVHTNLRK.N
RL5_MOUSE	MK_SCX_14.4593.4593.2	2	2.72	0.308	1	533.1	70.83333	K.NNVTPDMMEEM*YK.K
RL5_MOUSE	MK_SCX_14.5478.5478.2	2	2.349	0.361	1	627.9	75	K.NNVTPDMMEEMYK.K
RL5_MOUSE	MK_SCX_20_1.3401.3401.2	2	3.803	0.5	1	1384.1	90	R.YLM*EEDEDAYK.K
RL5_MOUSE	MK_SCX_31.3384.3384.2	2	4.13	0.425	1	1921.1	90.909096	R.YLMEEDEDAYKK.Q
RL5_MOUSE	MK_SCX_31.4493.4493.2	2	3.398	0.434	1	706.8	73.07692	K.NNVTPDMMEEMYKK.A
RL5_MOUSE	MK_SCX_42.4167.4167.2	2	4.455	0.578	1	1122.4	90.909096	K.HIMGQNVADYMR.Y
RL5_MOUSE	MK_SCX_42.4183.4183.3	3	3.111	0.407	1	602.9	47.727272	K.HIMGQNVADYMR.Y
RL5_MOUSE	MK_SCX_44.12444.12444.3	3	3.457	0.472	1	537.9	32.894737	K.VFGALKGAVDGGLSIPHSTK.R
RL5_MOUSE	MK_SCX_46.4504.4504.3	3	3.76	0.347	1	644	37.5	R.FPGYDSESKEFNAEVHR.K
RL5_MOUSE	MK_SCX_52.5983.5983.3	3	4.304	0.506	1	1549.9	38	R.TTTGNKVFGALKGAVDGGLSIPHSTK.R
RL5_MOUSE	MK_SCX_56.3593.3593.3	3	3.716	0.386	1	773.9	52.083332	R.KHIMGQNVADYMR.Y
RL5_MOUSE	MK_SCX_56.3628.3628.2	2	4.135	0.545	1	1501.1	91.66667	R.KHIMGQNVADYMR.Y
RL5_MOUSE	MK_SCX_56.4129.4129.3	3	4.499	0.458	1	1547.7	45.588234	K.RFPGYDSESKEFNAEVHR.K
RL6_MOUSE	MK_SCX_18.7531.7531.2	2	3.053	0.226	1	654.6	56.25	K.QLDSGLLLVTGPLVINR.V
RL6_MOUSE	MK_SCX_19.7888.7888.2	2	4.705	0.584	1	1194.2	82.14286	R.SSITPGTVLIILTGR.H
RL6_MOUSE	MK_SCX_24.4557.4557.2	2	2.23	0.532	1	351.5	88.88889	K.AVPQLQGYLR.S
RL6_MOUSE	MK_SCX_25.8436.8436.3	3	3.177	0.279	1	604.8	31.25	K.QLDSGLLLVTGPLVINRVPLR.R
RL6_MOUSE	MK_SCX_31.4323.4323.2	2	2.427	0.168	1	1259.6	75	R.SQFSLTNGMYPHK.L
RL6_MOUSE	MK_SCX_33.4276.4276.2	2	3.303	0.487	1	1084.8	88.88889	K.VDISDVKIPK.H
RL6_MOUSE	MK_SCX_39.5379.5379.3	3	3.631	0.251	1	1000.9	59.090908	K.IKAVPQLQGYLR.S
RL6_MOUSE	MK_SCX_39.5409.5409.2	2	3.251	0.421	1	697	81.818184	K.IKAVPQLQGYLR.S
RL6_MOUSE	MK_SCX_51.2764.2764.3	3	3.676	0.493	1	765	45.833336	R.HQEGEIFDTEKEK.Y
RL6_MOUSE	MK_SCX_53.4346.4346.2	2	5.776	0.587	1	1455.1	63.15789	R.HQEGEIFDTEKEKYEITEQR.K
RL6_MOUSE	MK_SCX_54.4232.4232.3	3	5.911	0.587	1	2456.6	51.315792	R.HQEGEIFDTEKEKYEITEQR.K
RL7_MOUSE	MK_SCX_20_1.7276.7276.2	2	3.852	0.435	1	1274.9	69.230774	R.IVEPYIAWGYPNLK.S
RL7_MOUSE	MK_SCX_21.4634.4634.2	2	3.496	0.53	1	1615.9	95	R.IALTDNSLIAR.S
RL7_MOUSE	MK_SCX_40.4364.4364.2	2	3.428	0.413	1	591.8	77.27273	K.RIALTDNSLIAR.S
RL7_MOUSE	MK_SCX_40.7032.7032.2	2	3.588	0.307	1	1275.3	81.818184	R.FKEANNFLWPFK.L
RL7_MOUSE	MK_SCX_40.7374.7374.3	3	3.553	0.159	1	1791.9	65.909096	R.FKEANNFLWPFK.L
RL7_MOUSE	MK_SCX_48.3060.3060.2	2	5.005	0.444	1	770.2	52.77778	K.TTHFVEGGDAGNREDQINR.L
RL7A_MOUSE	MK_SCX_20_1.6491.6491.2	2	4.528	0.555	1	1528.1	80.769226	K.VPPAINQFTQALDR.Q
RL7A_MOUSE	MK_SCX_21.4438.4438.2	2	4.179	0.322	1	1455.3	79.16667	R.AGVNTVTTLVENK.K
RL7A_MOUSE	MK_SCX_21.4469.4469.3	3	3.46	0.391	1	596.5	47.916664	R.AGVNTVTTLVENK.K
RL7A_MOUSE	MK_SCX_2201.4104.4104.2	2	2.82	0.196	1	1090.9	85	K.NFGIGQDIQPK.R
RL7A_MOUSE	MK_SCX_33.7110.7110.2	2	3.778	0.465	1	396	70	R.LKVPPAINQFTQALDR.Q
RL7A_MOUSE	MK_SCX_33.7339.7339.3	3	5.803	0.381	1	2179.2	55	R.LKVPPAINQFTQALDR.Q
RL7A_MOUSE	MK_SCX_53.6776.6776.3	3	4.822	0.459	1	2624.8	56.25	K.RLKVPPAINQFTQALDR.Q
RL7A_MOUSE	MK_SCX_60.16944.16944.3	3	3.295	0.289	1	1186.5	52.499996	R.RHWGGNVLGPK.S
RL8_MOUSE	MK_SCX_21.8483.8483.2	2	3.538	0.429	1	351.9	31.034481	K.AQLNIGNVLPVGTMPEGTIVCCLEEKPGDR.G
RL8_MOUSE	MK_SCX_2201.2853.2853.2	2	3.945	0.5	1	2003.1	95	R.AVVGVVAGGGR.I
RL8_MOUSE	MK_SCX_2201.7879.7879.3	3	5.129	0.514	1	566.9	27.586206	K.AQLNIGNVLPVGTM*PEGTIVCCLEEKPGDR.G
RL8_MOUSE	MK_SCX_2201.8416.8416.3	3	4.32	0.476	1	756.9	27.586206	K.AQLNIGNVLPVGTMPEGTIVCCLEEKPGDR.G
RL8_MOUSE	MK_SCX_31.8077.8077.3	3	4.334	0.553	1	431.6	23.387096	K.AQLNIGNVLPVGTMPEGTIVCCLEEKPGDRGK.L
RL8_MOUSE	MK_SCX_33.3486.3486.2	2	4.785	0.567	1	1684.1	70	R.ASGNYATVISHNPETK.K
RL8_MOUSE	MK_SCX_34.7211.7211.3	3	6.729	0.648	1	1338	36.666668	K.KAQLNIGNVLPVGTM*PEGTIVCCLEEKPGDR.G
RL8_MOUSE	MK_SCX_34.7997.7997.3	3	5.786	0.569	1	2201.6	36.666668	K.KAQLNIGNVLPVGTMPEGTIVCCLEEKPGDR.G
RL8_MOUSE	MK_SCX_46.7529.7529.3	3	5.684	0.554	1	974.7	27.34375	K.KAQLNIGNVLPVGTMPEGTIVCCLEEKPGDRGK.L
RL8_MOUSE	MK_SCX_51.1322.1322.2	2	2.22	0.221	1	493.7	85.71429	R.KGAGSVFR.A
RL8_MOUSE	MK_SCX_51.3526.3526.2	2	3.66	0.498	1	821.8	81.818184	K.GIVKDIIHDPGR.G
RL8_MOUSE	MK_SCX_54.4250.4250.3	3	3.123	0.217	1	601.9	39.705883	K.GIVKDIIHDPGRGAPLAK.V

RL8_MOUSE	MK_SCX_54.4261.4261.2	2	4.053	0.444	1	650.9	61.764706	K.GIVKDIIHDPGRGAPLAK.V
RL9_MOUSE	MK_SCX_17.6564.6564.2	2	4.66	0.469	1	883	66.66667	K.TILSNQTVDIPENVEITLK.G
RL9_MOUSE	MK_SCX_2201.4542.4542.2	2	3.672	0.434	1	915.9	88.88889	K.FLDGIYVSEK.G
RL9_MOUSE	MK_SCX_26.6580.6580.3	3	4.121	0.409	1	650.7	32.5	K.TILSNQTVDIPENVEITLKGR.T
RL9_MOUSE	MK_SCX_27.13683.13683.2	2	3.308	0.475	1	569.8	65.38461	R.DFNHINVELSLLGK.K
RL9_MOUSE	MK_SCX_28.8019.8019.3	3	4.019	0.306	1	697.2	35	-.MKTILSNQTVDIPENVEITLK.G
RL9_MOUSE	MK_SCX_39.5008.5008.2	2	3.39	0.369	1	1303.8	85	R.KFLDGIYVSEK.G
RL9_MOUSE	MK_SCX_42.6021.6021.2	2	3.683	0.531	1	867.7	64.28571	R.DFNHINVELSLLGKK.K
RLA0_MOUSE	MK_SCX_14.6743.6743.2	2	6.266	0.737	1	1372	53.333336	K.AFLADPSAFAAAAPAAATTAAPAAAAAPAK.A
RLA0_MOUSE	MK_SCX_15.6620.6620.3	3	4.931	0.651	1	735.4	30.000002	K.AFLADPSAFAAAAPAAATTAAPAAAAAPAK.A
RLA0_MOUSE	MK_SCX_20_1.6923.6923.3	3	6.401	0.647	1	1319.5	32.352943	K.AFLADPSAFAAAAPAAATTAAPAAAAAPAKAEAK.E
RLA0_MOUSE	MK_SCX_21.5267.5267.2	2	3.903	0.322	1	1591.4	88.88889	K.IIQLLDDYPK.C
RLA0_MOUSE	MK_SCX_2201.7067.7067.2	2	2.694	0.312	1	736.6	68.181816	K.TSFFQALGITTK.I
RLA0_MOUSE	MK_SCX_30.3966.3966.2	2	4.292	0.448	1	1821.7	87.5	R.DMLLANKVPAAAR.A
RLA0_MOUSE	MK_SCX_30.5973.5973.2	2	4.103	0.597	1	685.7	63.333332	R.GNVGFVFTKEDLTEIR.D
RLA1_MOUSE	MK_SCX_19.7509.7509.2	2	4.594	0.571	1	1062.8	70	K.AAGVSVEPFWPGLFAK.A
RLA2_MOUSE	MK_SCX_15.4852.4852.3	3	6.942	0.629	1	1839	32.8125	K.LASVPAGGAVAVSAAPGSAAPAAGSAPAAAEEK.K
RLA2_MOUSE	MK_SCX_15.5229.5229.2	2	5.281	0.648	1	603.4	35.9375	K.LASVPAGGAVAVSAAPGSAAPAAGSAPAAAEEK.K
RLA2_MOUSE	MK_SCX_19.4043.4043.2	2	4.763	0.59	1	1431.6	91.66667	K.ILDSVGIEADDDR.L
RLA2_MOUSE	MK_SCX_20_1.7671.7671.2	2	5.602	0.643	1	2347.6	72.22222	R.YVASYLLAALGGNSSPSAK.D
RLA2_MOUSE	MK_SCX_20_1.7743.7743.3	3	4.126	0.438	1	1142	38.88889	R.YVASYLLAALGGNSSPSAK.D
RLA2_MOUSE	MK_SCX_21.5171.5171.2	2	4.33	0.387	1	1807.4	86.36364	K.NIEDVIAQGVGK.L
RLA2_MOUSE	MK_SCX_2201.4436.4436.3	3	3.604	0.512	1	546.3	28.787878	K.LASVPAGGAVAVSAAPGSAAPAAGSAPAAAEEKK.D
RLA2_MOUSE	MK_SCX_24.6741.6741.2	2	4.472	0.356	1	1583.1	65.789474	K.VISELNGKNIEDVIAQGVGK.L
RLA2_MOUSE	MK_SCX_24.6970.6970.3	3	5.612	0.494	1	2057.5	48.684208	K.VISELNGKNIEDVIAQGVGK.L
RLA2_MOUSE	MK_SCX_27.4428.4428.3	3	3.089	0.37	1	578.6	46.666668	K.ILDSVGIEADDDRLNK.V
RLA2_MOUSE	MK_SCX_27.4573.4573.2	2	4.741	0.502	1	1672.8	73.333336	K.ILDSVGIEADDDRLNK.V
RLA2_MOUSE	MK_SCX_29.4431.4431.3	3	3.438	0.542	1	388.7	21.527777	K.LASVPAGGAVAVSAAPGSAAPAAGSAPAAAEEKKDEK.K
RLA2_MOUSE	MK_SCX_30.7067.7067.3	3	3.263	0.437	1	1122.3	33.695652	K.ILDSVGIEADDDRLNKVISELNGK.N
RLA2_MOUSE	MK_SCX_41.6502.6502.3	3	3.702	0.465	1	744.6	39.772728	R.YVASYLLAALGGNSSPSAKDIKK.I
RLA2_MOUSE	MK_SCX_44.4314.4314.2	2	5.398	0.532	1	1510.9	65.625	K.KILDSVGIEADDDRLNK.V
RLA2_MOUSE	MK_SCX_44.4322.4322.3	3	5.999	0.59	1	1681.8	54.6875	K.KILDSVGIEADDDRLNK.V
RM11_MOUSE	MK_SCX_19.5383.5383.2	2	3.34	0.52	1	430.9	62.5	R.AGQAIPGPPLGPILGQR.G
RM11_MOUSE	MK_SCX_29.6128.6128.2	2	6.167	0.576	1	2036.5	79.411766	K.AKDDAFAMQDVPLSSVVR.S
RM11_MOUSE	MK_SCX_44.4855.4855.3	3	3.255	0.535	1	674.2	43.75	R.VVKDLSAEELEAFQKER.A
RM12_MOUSE	MK_SCX_14.7176.7176.2	2	4.589	0.526	1	497	31.818182	K.IQDVGLMPMGGMVPGPVSAAAPASEAAEEEDVPK.Q
RM12_MOUSE	MK_SCX_19.6473.6473.3	3	6.189	0.623	1	621.2	28.57143	K.IQDVGLMPMGGMVPGPVSAAAPASEAAEEEDVPKQK.E
RM12_MOUSE	MK_SCX_2201.4366.4366.2	2	3.731	0.476	1	1281.9	77.27273	K.NYVQGINLVQAK.K
RM12_MOUSE	MK_SCX_27.4393.4393.2	2	4.571	0.491	1	428.3	47.22222	R.SEALAGAPLDNAPKEYPPK.I
RM12_MOUSE	MK_SCX_27.6929.6929.3	3	5.297	0.569	1	782	29.72973	K.IQDVGLMPMGGMVPGPVSAAAPASEAAEEEDVPKQKER.T
RM12_MOUSE	MK_SCX_37.4139.4139.3	3	3.108	0.266	1	387.7	47.5	K.KLVESLPQEIK.A
RM12_MOUSE	MK_SCX_37.4141.4141.2	2	3.895	0.399	1	1296.9	80	K.KLVESLPQEIK.A
RM13_MOUSE	MK_SCX_25.4296.4296.2	2	3.729	0.608	1	1094.6	94.44444	R.APQQWATFAR.M
RM13_MOUSE	MK_SCX_29.7864.7864.2	2	3.523	0.445	1	1003.2	64.28571	R.LHLFPDEDIPEDILK.N
RM13_MOUSE	MK_SCX_33.5271.5271.3	3	3.338	0.364	1	868.9	41.07143	K.RLDEYTQEEIEAFPR.V
RM13_MOUSE	MK_SCX_33.5680.5680.2	2	3.975	0.419	1	852.7	67.85714	K.RLDEYTQEEIEAFPR.V
RM13_MOUSE	MK_SCX_41.3341.3341.3	3	3.601	0.362	1	406.8	37.5	K.VYSSHTGYPGGFR.Q
RM16_MOUSE	MK_SCX_18.7018.7018.2	2	3.722	0.457	1	820.7	66.66667	K.TLLPVPTFENVSIPER.S
RM19_MOUSE	MK_SCX_2201.5180.5180.2	2	2.348	0.264	1	765	83.33333	R.FLSPEFIPPR.G
RM19_MOUSE	MK_SCX_40.5323.5323.2	2	3.152	0.303	1	560.9	72.22222	K.RLDDNLLYLR.D
RM19_MOUSE	MK_SCX_41.6838.6838.3	3	3.039	0.297	1	924.4	47.727272	K.WNKPWIEFDMMR.E

RM23_MOUSE	MK_SCX_20_1.4218.4218.2	2	3.427	0.455	1	812.8	70.83333	R.SPEPLEEELPQQR.Q
RM23_MOUSE	MK_SCX_26.7161.7161.3	3	5.831	0.577	1	1677	44.04762	R.TNFFIQLVRPGTAQPEDTVQFR.I
RM27_MOUSE	MK_SCX_20_1.5587.5587.2	2	4.525	0.477	1	2337	87.5	K.NTEAVDLVTSLPK.G
RM27_MOUSE	MK_SCX_49.4180.4180.3	3	3.31	0.461	1	864.2	41.07143	K.TFVHVVPAKPEGTFK.L
RM28_MOUSE	MK_SCX_16.10439.10439.2	2	4.778	0.652	1	868.7	54.761906	R.SFVIPEAEAEWVGLTLEEALEK.Q
RM28_MOUSE	MK_SCX_30.5011.5011.3	3	3.056	0.3	1	769.4	50	R.LLEEKDPVPLFK.V
RM28_MOUSE	MK_SCX_30.5038.5038.2	2	3.791	0.48	1	1490.5	86.36364	R.LLEEKDPVPLFK.V
RM33_MOUSE	MK_SCX_49.4795.4795.2	2	3.909	0.29	1	929.8	66.66667	K.LSLLHYDPIVNKK.V
RM40_MOUSE	MK_SCX_18.7571.7571.2	2	2.037	0.246	1	322.1	46.666668	K.ASQELIPIEDFITPVR.F
RM40_MOUSE	MK_SCX_18.7606.7606.1	1	3.046	0.485	1	349.8	46.666668	K.ASQELIPIEDFITPVR.F
RM40_MOUSE	MK_SCX_31.4104.4104.3	3	5.116	0.558	1	874.4	39.705883	K.EGPHYTPPISNYQAPEGR.Y
RM45_MOUSE	MK_SCX_32.6948.6948.3	3	3.277	0.252	1	942.6	43.333332	R.WGFVESLEPAQVVHVR.C
RM45_MOUSE	MK_SCX_49.4120.4120.2	2	2.436	0.495	1	662.3	77.77778	R.HLMNPYGSWR.M
RM46_MOUSE	MK_SCX_17.9300.9300.2	2	4.837	0.543	1	1847.7	63.15789	K.ALTPLQEEMAGLLQQIEVER.S
RM46_MOUSE	MK_SCX_52.4611.4611.3	3	4.641	0.552	1	739.4	46.666668	R.HVWASKEELGDYLQPK.Y
RM47_MOUSE	MK_SCX_23.5347.5347.2	2	2.529	0.401	1	641.3	68.75	R.FFAMPYVDR.F
RM47_MOUSE	MK_SCX_23.5421.5421.2	2	3.004	0.165	1	851.1	80	R.NMLLTLEQEAK.R
RM47_MOUSE	MK_SCX_29.3632.3632.3	3	3.466	0.572	1	492.3	48.214287	K.VVDSM*DNVDKVVQER.E
RM47_MOUSE	MK_SCX_29.4308.4308.2	2	3.604	0.437	1	1043.8	67.85714	K.VVDSMDNVDKVVQER.E
RM49_MOUSE	MK_SCX_17.7234.7234.1	1	2.677	0.372	1	458.5	68.181816	K.DVEEFLSPLLGK.T
RM49_MOUSE	MK_SCX_21.4864.4864.2	2	4.553	0.492	1	2018.5	84.61539	K.TPITQVNEVTGTLR.I
RM49_MOUSE	MK_SCX_40.3874.3874.2	2	3.135	0.314	1	695.6	83.33333	R.IKGYFDEQLK.A
RNF25_MOUSE	MK_SCX_17.5085.5085.2	2	4.049	0.571	1	624.9	52.63158	K.AAPEPQQPMELYQPSAESLR.Q
RNPC2_MOUSE	MK_SCX_17.4924.4924.2	2	6.098	0.618	1	2503.1	78.125	R.TDASSASSFLDSDELER.T
RNPC2_MOUSE	MK_SCX_19.5170.5170.2	2	3.609	0.51	1	402.6	57.14286	R.VLGVPIIVQASQAEK.N
RNPS1_MOUSE	MK_SCX_19.4422.4422.2	2	3.712	0.496	1	443.7	64.28571	K.GYAYVEFENPDEAEK.A
RNT2_MOUSE	MK_SCX_32.15553.15553.3	3	3.548	0.434	1	1432.1	45	K.FGIKPSINYYQLADFK.D
RNT2_MOUSE	MK_SCX_40.9130.9130.3	3	4.215	0.447	1	494.4	31.25	K.FGIKPSINYYQLADFKDALTR.I
ROA1_MOUSE	MK_SCX_20_1.5872.5872.2	2	2.543	0.19	1	317.9	66.66667	K.IEVIEIMTDR.G
ROA1_MOUSE	MK_SCX_38.3542.3542.2	2	4.207	0.543	1	1340.4	66.66667	R.SSGPYGGGGQYFAKPR.N
ROA1_MOUSE	MK_SCX_49.5356.5356.3	3	3.691	0.369	1	580.4	35	K.RGFAFVTFDDHDSVDK.I
ROA1_MOUSE	MK_SCX_55.3940.3940.3	3	4.077	0.551	2	660.6	41.666664	K.IFVGGIKEDTEEHHLR.D
ROA1_MOUSE	MK_SCX_59.13577.13577.3	3	4.52	0.739	1	2065.4	56.25	K.KLFVGGIKEDTEEHHLR.D
ROA1_MOUSE	MK_SCX_59.13588.13588.2	2	4.381	0.346	1	1124.4	65.625	K.KLFVGGIKEDTEEHHLR.D
ROA2_MOUSE	MK_SCX_17.4826.4826.3	3	3.433	0.334	1	432.8	25.925926	R.GFGDGYNGYGGGPGGGNFGGSPGYGGGR.G
ROA2_MOUSE	MK_SCX_19.4698.4698.2	2	4.575	0.656	1	920	38.88889	R.GFGDGYNGYGGGPGGGNFGGSPGYGGGR.G
ROA2_MOUSE	MK_SCX_19.7287.7287.2	2	5.13	0.48	1	2835.3	86.666664	K.LFIGGLSFETTEESLR.N
ROA2_MOUSE	MK_SCX_20_1.5103.5103.2	2	3.581	0.372	1	1352.9	94.44444	K.IDTIEIITDR.Q
ROA2_MOUSE	MK_SCX_2201.1983.1983.2	2	3.299	0.397	1	604	88.88889	R.QEMQEVQSSR.S
ROA2_MOUSE	MK_SCX_2201.3523.3523.2	2	5.882	0.585	1	1916.7	60.416668	R.NM*GGPYGGGNYGPGGSGGSGGYGGR.S
ROA2_MOUSE	MK_SCX_2201.3528.3528.3	3	6.757	0.638	1	3600.7	47.916664	R.NM*GGPYGGGNYGPGGSGGSGGYGGR.S
ROA2_MOUSE	MK_SCX_2201.3769.3769.2	2	6.528	0.717	1	1370	54.166668	R.NMGGPYGGGNYGPGGSGGSGGYGGR.S
ROA2_MOUSE	MK_SCX_2201.3776.3776.3	3	6.016	0.546	1	2794.8	41.666664	R.NMGGPYGGGNYGPGGSGGSGGYGGR.S
ROA2_MOUSE	MK_SCX_27.3720.3720.2	2	3.222	0.433	1	788.2	64.28571	R.GGGGNFGPGPGSNFR.G
ROA2_MOUSE	MK_SCX_28.5590.5590.2	2	4.291	0.514	1	1110.8	67.85714	R.GFGFVTFDDHDPVDK.I
ROA2_MOUSE	MK_SCX_33.9705.9705.3	3	3.758	0.398	1	577	40.625	R.KLFIGGLSFETTEESLR.N
ROA2_MOUSE	MK_SCX_34.7202.7202.3	3	4.19	0.542	1	876.1	39.473686	R.GFGFVTFDDHDPVDKIVLQK.Y
ROA2_MOUSE	MK_SCX_35.10961.10961.2	2	5.404	0.621	1	1361.6	57.894737	R.GFGFVTFDDHDPVDKIVLQK.Y
ROA2_MOUSE	MK_SCX_35.3782.3782.3	3	3.158	0.41	1	898.9	38.541664	R.GGGGNFGPGPGSNFRGGSDGYGSGR.G
ROA2_MOUSE	MK_SCX_41.8738.8738.3	3	4.532	0.472	1	755.4	31.25	R.KLFIGGLSFETTEESLRNYYEQWGK.L
ROA2_MOUSE	MK_SCX_51.1580.1580.2	2	2.243	0.196	1	313	62.5	R.EESGKPGAHVTVK.K

ROA2_MOUSE	MK_SCX_52.6676.6676.3	3	4.951	0.426	1	1300.2	37.5	K.RGFGFVTFDDHDPVDKIVLQK.Y
ROA2_MOUSE	MK_SCX_54.6325.6325.3	3	4.853	0.543	1	2339.5	41.304348	K.LFVGGIKEDTEEHHLRDYFEEYGK.I
ROA2_MOUSE	MK_SCX_55.3940.3940.3	3	4.077	0.551	2	660.6	41.666664	K.IFVGGIKEDTEEHHLR.D
ROA2_MOUSE	MK_SCX_58.4955.4955.2	2	2.348	0.131	1	664.2	62.5	K.YHTINGHNAEVRK.A
ROA2_MOUSE	MK_SCX_59.13577.13577.3	3	4.52	0.739	1	2065.4	56.25	K.KLFVGGIKEDTEEHHLR.D
ROA2_MOUSE	MK_SCX_59.13588.13588.2	2	4.381	0.346	1	1124.4	65.625	K.KLFVGGIKEDTEEHHLR.D
ROA3_MOUSE	MK_SCX_20_1.4252.4252.2	2	3.705	0.547	1	1245.3	88.88889	K.IETIEVMEDR.Q
ROA3_MOUSE	MK_SCX_23.3510.3510.3	3	3.27	0.165	1	441.6	32.142857	R.SSGSPYGGGYGSGGGSGGYGSR.R
ROA3_MOUSE	MK_SCX_23.3748.3748.2	2	5.99	0.656	1	1411	66.66667	R.SSGSPYGGGYGSGGGSGGYGSR.R
ROA3_MOUSE	MK_SCX_27.5717.5717.2	2	2.551	0.306	1	563.6	57.14286	R.GFAFVTFDDHDTVDK.I
ROA3_MOUSE	MK_SCX_30.5023.5023.2	2	5.656	0.428	1	2007.2	80	K.IFVGGIKEDTEEYNLR.D
ROA3_MOUSE	MK_SCX_31.5130.5130.3	3	4.902	0.528	1	1213.3	48.333332	K.IFVGGIKEDTEEYNLR.D
ROA3_MOUSE	MK_SCX_32.15931.15931.3	3	3.514	0.245	1	572.1	35.9375	R.KLFIGGLSFETTDDSLR.E
ROA3_MOUSE	MK_SCX_34.6778.6778.3	3	3.22	0.239	1	513.6	35.526314	R.GFAFVTFDDHDTVDKIVVQK.Y
ROA3_MOUSE	MK_SCX_35.3772.3772.3	3	3.995	0.3	1	2059.5	58.333332	K.YGKIETIEVM*EDR.Q
ROA3_MOUSE	MK_SCX_35.4680.4680.3	3	3.873	0.203	1	1295.5	50	K.YGKIETIEVMEDR.Q
ROA3_MOUSE	MK_SCX_38.6954.6954.3	3	4.756	0.465	1	1318.1	41.25	K.IFVGGIKEDTEEYNLRDYFEK.Y
ROA3_MOUSE	MK_SCX_41.6532.6532.3	3	3.52	0.316	1	438.5	26.136362	R.GFGFVTYSCVEEVDAAMCARPHK.V
ROA3_MOUSE	MK_SCX_48.5041.5041.3	3	4.526	0.393	1	1610.8	46.666668	K.RGFAFVTFDDHDTVDK.I
ROA3_MOUSE	MK_SCX_50.4649.4649.2	2	5.933	0.483	1	2673.2	81.25	K.KIFVGGIKEDTEEYNLR.D
ROA3_MOUSE	MK_SCX_50.4658.4658.3	3	5.221	0.551	1	985.3	45.3125	K.KIFVGGIKEDTEEYNLR.D
ROA3_MOUSE	MK_SCX_52.5757.5757.3	3	3.946	0.499	1	477.6	25.961538	R.GFGFVTYSCVEEVDAAMCARPHKVDGR.V
ROA3_MOUSE	MK_SCX_52.6678.6678.3	3	4.727	0.51	1	1546.5	42.857143	R.KLFIGGLSFETTDDSLREHFEK.W
ROAA_MOUSE	MK_SCX_16.7996.7996.2	2	5.241	0.575	1	1719.1	66.66667	R.EYFGQFGEIEAIELPIDPK.L
ROAA_MOUSE	MK_SCX_20_1.4727.4727.2	2	4.561	0.497	1	1726.9	84.61539	K.IFVGGLNPEATEEK.I
ROAA_MOUSE	MK_SCX_2201.5628.5628.2	2	2.839	0.477	1	745.3	72.72727	K.MFVGGLSWDTSK.K
ROAA_MOUSE	MK_SCX_25.6789.6789.2	2	2.802	0.246	1	634.9	85.71429	R.GFGFILFK.D
ROAA_MOUSE	MK_SCX_30.14668.14668.3	3	4.198	0.381	1	923.1	37.5	K.IREYFGQFGEIEAIELPIDPK.L
ROCK2_MOUSE	MK_SCX_20_1.3357.3357.2	2	3.519	0.44	1	938.4	62.5	K.MPGAPEAAPGDGAGAGR.Q
ROR1_MOUSE	MK_SCX_11.4462.4462.2	2	2.182	0.386	1	391.4	42.857143	R.GQNVEMSMLNAYKPK.S
ROR1_MOUSE	MK_SCX_60.14470.14470.2	2	2.647	0.32	1	486.6	60.000004	R.NILIGEQLHVK.I
RPA1_MOUSE	MK_SCX_21.4340.4340.2	2	2.352	0.141	1	392	50	K.TPM*MSVPVFDTKK.A
RPA1_MOUSE	MK_SCX_55.4918.4918.2	2	2.007	0.19	1	535.1	72.22222	K.RNKFQVYELR.F
RPB1_MOUSE	MK_SCX_18.4293.4293.2	2	4.003	0.613	1	424.6	45	K.YTPTSPSYSPSSPEYTPASPK.Y
RPB1_MOUSE	MK_SCX_19.4579.4579.2	2	2.091	0.159	1	338.2	50	K.KLVIVNGDDPLSR.Q
RPE_MOUSE	MK_SCX_16.6447.6447.2	2	5.041	0.561	1	1932.8	65	R.TQFPTLDIEVDGGVGPDTVQK.C
RPF1_MOUSE	MK_SCX_16.3781.3781.3	3	3.769	0.414	1	479.9	30.208334	K.AAAEEPQEAAAASDGTAESGVQPAK.A
RPF1_MOUSE	MK_SCX_16.3788.3788.2	2	4.484	0.543	1	1528.6	50	K.AAAEEPQEAAAASDGTAESGVQPAK.A
RPF53_MOUSE	MK_SCX_17.6704.6704.2	2	3.562	0.247	1	388.8	53.333336	K.MIEENSHCSYVIEMLK.S
RPN2_MOUSE	MK_SCX_23.4479.4479.2	2	2.129	0.313	1	808.4	80	K.LSSGYYDFSVR.V
RPN2_MOUSE	MK_SCX_27.6198.6198.3	3	3.99	0.475	1	883.9	34.090908	R.YHVPVVVVPEGSTSDTQEQAILR.L
RRAS_MOUSE	MK_SCX_21.7542.7542.2	2	2.242	0.395	1	444.3	72.72727	R.LNVDEAFEQLVR.A
RRAS_MOUSE	MK_SCX_34.3615.3615.3	3	3.308	0.297	1	478	40	R.KYQEQELPPSPPSAPR.K
RRAS_MOUSE	MK_SCX_50.4615.4615.3	3	5.064	0.451	1	1726.1	50	R.VKDRDDFPIVLVGNK.A
RRAS2_MOUSE	MK_SCX_2201.3308.3308.2	2	2.21	0.257	1	439.9	60.000004	R.LVVVGGGGVGK.S
RRAS2_MOUSE	MK_SCX_25.7016.7016.3	3	3.164	0.285	1	662.1	31.25	R.LDILDTAGQEEFGAMREQYMR.T
RRAS2_MOUSE	MK_SCX_36.6372.6372.2	2	3.325	0.568	1	1244	86.36364	R.MNVDQAFHELVR.V
RRAS2_MOUSE	MK_SCX_50.5274.5274.3	3	4.291	0.309	1	1627.6	50	R.VKDRDEFPMILIGNK.A
RRBP1_MOUSE	MK_SCX_13.3511.3511.2	2	3.891	0.471	1	480.3	50	R.EAEETQNSLQAECDQYR.T
RRBP1_MOUSE	MK_SCX_18.3656.3656.2	2	4.714	0.642	1	1476.5	79.16667	R.DALNQATSQVESK.Q
RRBP1_MOUSE	MK_SCX_18.8007.8007.2	2	4.963	0.638	1	1454.8	70.588234	K.VEPAVSSIVNSIQVLASK.S

RRBP1_MOUSE	MK_SCX_19.4237.4237.2	2	3.012	0.207	1	406.4	50	R.QLLLESQSQLDEAK.S
RRBP1_MOUSE	MK_SCX_20_1.3521.3521.2	2	4.679	0.595	1	2040.4	91.66667	K.LLATEQEDAAVAK.S
RRBP1_MOUSE	MK_SCX_2201.2499.2499.2	2	2.714	0.259	1	644.9	87.5	K.LQSSEVEVK.S
RRBP1_MOUSE	MK_SCX_2201.2549.2549.2	2	3.595	0.354	1	1334	85	R.TEATLEAEQTR.R
RRBP1_MOUSE	MK_SCX_2201.3480.3480.2	2	3.64	0.33	1	1219.4	94.44444	K.LTAEFEEAQR.T
RRBP1_MOUSE	MK_SCX_25.5023.5023.3	3	6.006	0.55	1	1568.4	41.346153	R.SIEALLEAGQAQDTQASHAEANQQQTR.L
RRBP1_MOUSE	MK_SCX_26.3657.3657.3	3	3.347	0.325	1	524.7	29.545454	R.SHVEDGDVAGSPAVPPAEQDPM*K.L
RRBP1_MOUSE	MK_SCX_26.4165.4165.2	2	5.822	0.604	1	1468.1	70.454544	R.SHVEDGDVAGSPAVPPAEQDPMK.L
RRBP1_MOUSE	MK_SCX_29.2804.2804.3	3	3.133	0.432	1	435	31.25	K.ASMVQSQEAPKQDAPAK.K
RRBP1_MOUSE	MK_SCX_30.3892.3892.3	3	3.31	0.56	1	589.9	42.1875	R.AAGPLESSGKEEITQLK.E
RRBP1_MOUSE	MK_SCX_30.3910.3910.2	2	4.567	0.509	1	1311	65.625	R.AAGPLESSGKEEITQLK.E
RRBP1_MOUSE	MK_SCX_30.7118.7118.2	2	3.691	0.469	1	1258.1	64.28571	R.TILAETEGMLKDLQK.S
RRBP1_MOUSE	MK_SCX_31.5527.5527.3	3	4.924	0.561	1	492.8	27.586206	R.VSAVAVAPTSVHSSVGHTPIATVPAMPQEK.L
RRBP1_MOUSE	MK_SCX_32.5340.5340.2	2	3.667	0.371	1	755.6	69.230774	K.KPPTLEPSMDIVLK.L
RRBP1_MOUSE	MK_SCX_34.4555.4555.2	2	2.547	0.213	1	434.5	46.666668	K.KLQEQLGKAEDGSSSK.E
RRBP1_MOUSE	MK_SCX_40.3575.3575.3	3	3.614	0.218	1	1156	59.090908	R.QKLTAEFEEAQR.T
RRBP1_MOUSE	MK_SCX_44.4049.4049.3	3	5.241	0.631	1	781.9	43.055553	R.AAGPLESSGKEEITQLKER.L
RRBP1_MOUSE	MK_SCX_44.4094.4094.2	2	4.977	0.481	1	1136.6	55.555557	R.AAGPLESSGKEEITQLKER.L
RRBP1_MOUSE	MK_SCX_45.3714.3714.3	3	3.801	0.362	1	486.1	33.82353	K.QVLQLQASHKESEEALQK.R
RRFM_MOUSE	MK_SCX_16.6390.6390.2	2	4.47	0.566	1	736	52.63158	K.QISQMADDTVAELDQHLAAK.T
RRFM_MOUSE	MK_SCX_26.6971.6971.2	2	4.717	0.604	1	637.7	47.5	R.ESGMNLNPEVEGTLIRVPIPK.V
RRFM_MOUSE	MK_SCX_28.4215.4215.2	2	4.79	0.564	1	590.8	68.75	R.TAPGSLDHITVVTADGK.V
RS10_MOUSE	MK_SCX_2201.8062.8062.2	2	3.261	0.47	1	997.4	87.5	R.IAIYELLFK.E
RS10_MOUSE	MK_SCX_25.7050.7050.2	2	2.948	0.292	1	440.5	64.28571	R.DYLHLPPEIVPATLR.R
RS10_MOUSE	MK_SCX_25.7203.7203.3	3	3.707	0.387	1	787.6	53.571426	R.DYLHLPPEIVPATLR.R
RS10_MOUSE	MK_SCX_39.14580.14580.3	3	3.626	0.228	1	609.7	42.857143	K.KAEAGAGSATEFQFR.G
RS10_MOUSE	MK_SCX_39.3740.3740.2	2	5.328	0.554	1	2731.1	89.28571	K.KAEAGAGSATEFQFR.G
RS10_MOUSE	MK_SCX_39.7427.7427.3	3	3.418	0.469	1	929.5	43.333332	R.DYLHLPPEIVPATLRR.S
RS10_MOUSE	MK_SCX_40.15575.15575.3	3	3.933	0.392	1	1174.5	50	R.HFYWYLTNEGIQYLR.D
RS10_MOUSE	MK_SCX_45.4161.4161.2	2	2.972	0.547	1	1033.8	83.33333	R.GYVKEQFAWR.H
RS10_MOUSE	MK_SCX_45.4192.4192.3	3	3.019	0.494	1	465.9	55.555557	R.GYVKEQFAWR.H
RS10_MOUSE	MK_SCX_56.3905.3905.3	3	3.371	0.55	1	540.6	38.333332	K.HPELADKNVPNLHVMK.A
RS11_MOUSE	MK_SCX_16.6120.6120.2	2	4.359	0.59	1	1426	64.70589	R.DVQIGDIVTVGECRPLSK.T
RS11_MOUSE	MK_SCX_21.3686.3686.2	2	2.434	0.378	1	551.3	72.22222	K.EAIEGTYIDK.K
RS11_MOUSE	MK_SCX_39.3355.3355.3	3	3.05	0.483	1	591.2	45.454548	R.AYQKQPTIFQNK.K
RS11_MOUSE	MK_SCX_42.5411.5411.3	3	3.354	0.193	1	1214.7	38.75	K.EAIEGTYIDKKCPFTGNVSIR.G
RS11_MOUSE	MK_SCX_44.4097.4097.3	3	3.51	0.385	1	839.3	55.555557	R.YYKNIGLGFK.T
RS11_MOUSE	MK_SCX_58.9601.9601.2	2	2.384	0.271	1	557.7	92.85714	R.RDYLHYIR.K
RS12_MOUSE	MK_SCX_32.5271.5271.3	3	3.06	0.263	1	491.8	33.82353	R.QAHLCVLASNCDEPMYVK.L
RS12_MOUSE	MK_SCX_32.5349.5349.2	2	4.407	0.505	1	790.6	75	K.ESQAKDVIEEYFK.C
RS12_MOUSE	MK_SCX_33.5264.5264.2	2	2.596	0.352	1	719	52.941177	R.QAHLCVLASNCDEPMYVK.L
RS12_MOUSE	MK_SCX_39.7076.7076.3	3	3.688	0.432	1	1135.6	46.875	K.DYGKESQAKDVIEEYFK.C
RS13_MOUSE	MK_SCX_21.5747.5747.2	2	3.183	0.364	1	770.3	68.181816	K.GLTPSQIGVILR.D
RS13_MOUSE	MK_SCX_2201.3851.3851.2	2	2.185	0.222	1	477.2	91.66667	R.LILIESR.I
RS13_MOUSE	MK_SCX_27.8382.8382.2	2	3.831	0.506	1	906.2	75	K.GLAPDLPEDLYHLIK.K
RS13_MOUSE	MK_SCX_31.3674.3674.2	2	4.394	0.428	1	1573.7	86.36364	K.LTSDDVKEQIYK.L
RS13_MOUSE	MK_SCX_38.5378.5378.3	3	4.125	0.192	1	1983.9	60.416668	K.KGLTPSQIGVILR.D
RS13_MOUSE	MK_SCX_38.5450.5450.2	2	3.404	0.402	1	1079.9	79.16667	K.KGLTPSQIGVILR.D
RS13_MOUSE	MK_SCX_40.8606.8606.2	2	3.268	0.393	1	579.1	60.000004	K.GLAPDLPEDLYHLIKK.A
RS13_MOUSE	MK_SCX_43.7396.7396.3	3	4.737	0.568	1	1268	51.5625	K.SKGLAPDLPEDLYHLIK.K
RS13_MOUSE	MK_SCX_43.7431.7431.2	2	3.896	0.536	1	785.2	62.5	K.SKGLAPDLPEDLYHLIK.K

RS13_MOUSE	MK_SCX_53.6998.6998.3	3	3.153	0.341	1	405.2	30.882353	K.SKGLAPDLPEDLYHLIKK.A
RS14_MOUSE	MK_SCX_16.4801.4801.2	2	5.046	0.554	1	1699.1	64.70589	R.DESSPYAAM*LAAQDVAQR.C
RS14_MOUSE	MK_SCX_16.5860.5860.2	2	5.828	0.56	1	1948.9	67.64706	R.DESSPYAAMLAAQDVAQR.C
RS14_MOUSE	MK_SCX_17.3723.3723.2	2	3.957	0.533	1	1110.1	87.5	R.IEDVTPIPSDSTR.R
RS14_MOUSE	MK_SCX_27.3692.3692.2	2	2.561	0.261	1	370.2	50	R.IEDVTPIPSDSTRR.K
RS14_MOUSE	MK_SCX_27.4415.4415.3	3	5.075	0.581	1	1744.7	42.5	K.ADRDESSPYAAM*LAAQDVAQR.C
RS14_MOUSE	MK_SCX_27.5715.5715.2	2	6.425	0.513	1	2424.2	80	K.ADRDESSPYAAMLAAQDVAQR.C
RS14_MOUSE	MK_SCX_28.4309.4309.3	3	3.46	0.268	1	732.4	41.666664	K.IGRIEDVTPIPSDSTR.R
RS14_MOUSE	MK_SCX_28.4329.4329.2	2	3.204	0.461	1	479.8	53.333336	K.IGRIEDVTPIPSDSTR.R
RS14_MOUSE	MK_SCX_28.5408.5408.3	3	3.572	0.349	1	560.5	27.5	K.ADRDESSPYAAMLAAQDVAQR.C
RS14_MOUSE	MK_SCX_42.2580.2580.3	3	3.229	0.395	1	502.1	47.916664	R.TKTPGPGAQSALR.A
RS14_MOUSE	MK_SCX_43.4347.4347.3	3	6.19	0.576	1	3069.8	47.727272	K.VKADRDESSPYAAM*LAAQDVAQR.C
RS14_MOUSE	MK_SCX_43.5269.5269.2	2	5.168	0.477	1	1245.1	61.363636	K.VKADRDESSPYAAMLAAQDVAQR.C
RS14_MOUSE	MK_SCX_43.5370.5370.3	3	5.795	0.495	1	1957.4	43.18182	K.VKADRDESSPYAAMLAAQDVAQR.C
RS15_MOUSE	MK_SCX_14.6596.6596.2	2	3.332	0.311	1	833.6	55.555557	R.DMIILPEM*VGSM*VGVYNGK.T
RS15_MOUSE	MK_SCX_14.7368.7368.2	2	3.746	0.156	1	1355.4	66.66667	R.DMIILPEMVGSM*VGVYNGK.T
RS15_MOUSE	MK_SCX_14.7526.7526.2	2	3.703	0.215	1	1419.8	69.44444	R.DMIILPEM*VGSMVGVYNGK.T
RS15_MOUSE	MK_SCX_14.7914.7914.2	2	4.136	0.342	1	1118	63.88889	R.DM*IILPEMVGSMVGVYNGK.T
RS15_MOUSE	MK_SCX_15.5959.5959.2	2	3.179	0.32	1	467.7	47.22222	R.DM*IILPEM*VGSM*VGVYNGK.T
RS15_MOUSE	MK_SCX_15.6725.6725.2	2	3.89	0.156	1	1303	72.22222	R.DM*IILPEMVGSM*VGVYNGK.T
RS15_MOUSE	MK_SCX_15.6887.6887.2	2	3.611	0.233	1	672	55.555557	R.DM*IILPEM*VGSMVGVYNGK.T
RS15_MOUSE	MK_SCX_15.8557.8557.2	2	5.176	0.618	1	1748.2	72.22222	R.DMIILPEMVGSMVGVYNGK.T
RS15_MOUSE	MK_SCX_16.10011.10011.2	2	5.159	0.598	1	1856.9	54.761906	R.GVDLDQLLDM*SYEQLM*QLYSAR.Q
RS15_MOUSE	MK_SCX_16.10063.10063.3	3	6.078	0.614	1	2724.9	46.42857	R.GVDLDQLLDM*SYEQLM*QLYSAR.Q
RS15_MOUSE	MK_SCX_16.10303.10303.2	2	5.115	0.444	1	1865.7	57.14286	R.GVDLDQLLDMSYEQLM*QLYSAR.Q
RS15_MOUSE	MK_SCX_17.10508.10508.2	2	5.313	0.323	1	2120.5	57.14286	R.GVDLDQLLDM*SYEQLMQLYSAR.Q
RS15_MOUSE	MK_SCX_17.10809.10809.2	2	5.962	0.657	1	2209.6	57.14286	R.GVDLDQLLDMSYEQLMQLYSAR.Q
RS15_MOUSE	MK_SCX_46.7768.7768.3	3	5.619	0.544	1	1005.8	36.53846	K.TFNQVEIKPEM*IGHYLGEFSITYKPVK.H
RS15_MOUSE	MK_SCX_46.8188.8188.3	3	5.428	0.498	1	2133.3	37.5	K.TFNQVEIKPEMIGHYLGEFSITYKPVK.H
RS15A_MOUSE	MK_SCX_34.4592.4592.2	2	3.499	0.335	1	925.9	83.33333	R.FDVQLKDLEK.W
RS15A_MOUSE	MK_SCX_41.4770.4770.3	3	3.074	0.165	1	708.4	52.499996	-.VRMNVLADALK.S
RS15A_MOUSE	MK_SCX_42.4474.4474.2	2	3.25	0.165	1	995.6	80	-.VRMNVLADALK.S
RS15A_MOUSE	MK_SCX_43.7831.7831.3	3	5.199	0.392	1	1705.5	45.833336	R.FDVQLKDLEKWQNNLLPSR.Q
RS15A_MOUSE	MK_SCX_50.4939.4939.3	3	4.611	0.394	1	1535.1	51.923077	K.HGYIGEFEIIDDHR.A
RS15A_MOUSE	MK_SCX_50.4965.4965.2	2	5.165	0.59	1	2151.7	88.46153	K.HGYIGEFEIIDDHR.A
RS16_MOUSE	MK_SCX_20_1.6855.6855.2	2	2.777	0.21	1	527.9	72.22222	K.LLEPVLLLGK.E
RS16_MOUSE	MK_SCX_2201.3495.3495.2	2	2.56	0.246	1	1060.3	92.85714	R.TLLVADPR.R
RS16_MOUSE	MK_SCX_23.4559.4559.2	2	3.649	0.55	1	1246.9	85	K.GPLQSVQVFGR.K
RS16_MOUSE	MK_SCX_32.5704.5704.2	2	3.231	0.461	1	1064.2	81.818184	K.LLEPVLLLGKER.F
RS16_MOUSE	MK_SCX_33.4952.4952.2	2	3.299	0.478	1	491.2	75	K.EIKDILIQYDR.T
RS16_MOUSE	MK_SCX_37.4731.4731.2	2	4.8	0.475	1	1523.9	84.61539	-.PSKGPLQSVQVFGR.K
RS16_MOUSE	MK_SCX_37.4771.4771.3	3	4.751	0.509	1	1361.6	53.846157	-.PSKGPLQSVQVFGR.K
RS17_MOUSE	MK_SCX_14.7494.7494.2	2	6.134	0.491	1	1671.5	64.28571	R.DNYVPEVSALDQEIIEVDPDTK.E
RS17_MOUSE	MK_SCX_17.7040.7040.2	2	5.387	0.619	1	1222	57.14286	K.LLDFGSLSNLQVTQPTVGM*NFK.T
RS17_MOUSE	MK_SCX_17.7799.7799.3	3	5.499	0.394	1	1195.7	39.285713	K.LLDFGSLSNLQVTQPTVGMNFK.T
RS17_MOUSE	MK_SCX_17.7935.7935.2	2	5.518	0.613	1	1217.4	57.14286	K.LLDFGSLSNLQVTQPTVGMNFK.T
RS17_MOUSE	MK_SCX_18.8147.8147.2	2	4.267	0.444	1	484.1	36	R.DNYVPEVSALDQEIIEVDPDTKEMLK.L
RS17_MOUSE	MK_SCX_25.7295.7295.3	3	3.996	0.477	1	978.9	39.772728	R.RDNYVPEVSALDQEIIEVDPDTK.E
RS17_MOUSE	MK_SCX_25.7298.7298.2	2	4.285	0.615	1	1319.2	59.090908	R.RDNYVPEVSALDQEIIEVDPDTK.E
RS17_MOUSE	MK_SCX_25.7445.7445.3	3	4.707	0.513	1	772.3	28.125	K.LLDFGSLSNLQVTQPTVGM*NFKTPR.G
RS17_MOUSE	MK_SCX_25.7944.7944.3	3	6.981	0.565	1	1697	41.666664	K.LLDFGSLSNLQVTQPTVGMNFKTPR.G

RS17_MOUSE	MK_SCX_25.8018.8018.2	2	4.285	0.481	1	371.6	37.5	K.LLDFGSLSNLQVTQPTVGMNFKTPR.G
RS17_MOUSE	MK_SCX_32.13618.13618.3	3	5.042	0.452	1	709.2	31.730768	R.RDNYVPEVSALDQEIIEVDPDTKEMLK.L
RS17_MOUSE	MK_SCX_39.4064.4064.2	2	3.499	0.519	1	971.5	83.33333	K.IAGYVTHLMK.R
RS17_MOUSE	MK_SCX_54.4146.4146.3	3	3.396	0.287	1	1137	52.272724	R.NKIAGYVTHLMK.R
RS18_MOUSE	MK_SCX_20_1.3754.3754.2	2	2.637	0.177	1	1065.7	77.27273	K.YSQVLANGLDNK.L
RS18_MOUSE	MK_SCX_21.3197.3197.2	2	3.057	0.364	1	998.7	80	R.AGELTEDEVER.V
RS18_MOUSE	MK_SCX_23.5131.5131.2	2	3.176	0.324	1	890.1	93.75	K.IAFAITAIK.G
RS18_MOUSE	MK_SCX_24.3043.3043.2	2	2.873	0.334	1	762.6	81.25	R.VLNTNIDGR.R
RS18_MOUSE	MK_SCX_24.3869.3869.2	2	2.321	0.421	1	546.3	87.5	R.VITIMQNPR.Q
RS18_MOUSE	MK_SCX_26.5466.5466.2	2	3.061	0.41	1	574.9	92.85714	K.IPDWFLNR.Q
RS18_MOUSE	MK_SCX_27.3978.3978.2	2	3.006	0.191	1	1994.4	75	K.DGKYSQVLANGLDNK.L
RS18_MOUSE	MK_SCX_27.3994.3994.3	3	3.412	0.264	1	1102.6	48.214287	K.DGKYSQVLANGLDNK.L
RS18_MOUSE	MK_SCX_38.5884.5884.3	3	3.461	0.396	1	926.1	44.444447	K.YSQVLANGLDNKLREDLER.L
RS18_MOUSE	MK_SCX_41.6430.6430.3	3	3.506	0.459	1	1143.9	55	R.QYKIPDWFLNR.Q
RS18_MOUSE	MK_SCX_41.6455.6455.2	2	2.104	0.294	1	516.3	70	R.QYKIPDWFLNR.Q
RS18_MOUSE	MK_SCX_59.6772.6772.2	2	2.276	0.152	1	353.4	71.42857	R.RYAHVVLR.K
RS19_MOUSE	MK_SCX_21.3359.3359.2	2	3.441	0.217	1	1130.5	87.5	K.DVNQQEFVR.A
RS19_MOUSE	MK_SCX_2201.4232.4232.2	2	2.781	0.349	1	696	87.5	R.VLQALEGLK.M
RS19_MOUSE	MK_SCX_33.4132.4132.3	3	4.804	0.443	1	1425.2	50	-.PGVTVKDVNQQEFVR.A
RS19_MOUSE	MK_SCX_33.4169.4169.2	2	4.3	0.478	1	1276.3	71.42857	-.PGVTVKDVNQQEFVR.A
RS19_MOUSE	MK_SCX_38.5319.5319.2	2	2.429	0.228	1	628.4	75	K.LKVPEWVDTVK.L
RS19_MOUSE	MK_SCX_38.5466.5466.3	3	3.877	0.43	1	1591.6	57.5	K.LKVPEWVDTVK.L
RS19_MOUSE	MK_SCX_44.4184.4184.2	2	2.852	0.313	1	1034.9	83.33333	R.RVLQALEGLK.M
RS19_MOUSE	MK_SCX_52.4426.4426.3	3	4.007	0.403	1	1245.1	44.230766	K.SGKLKVPEWVDTVK.L
RS19_MOUSE	MK_SCX_53.5354.5354.3	3	5.516	0.47	1	1951.9	53.571426	K.HKELAPYDENWFYTR.A
RS19_MOUSE	MK_SCX_53.5496.5496.2	2	5.052	0.597	1	1341.6	85.71429	K.HKELAPYDENWFYTR.A
RS2_MOUSE	MK_SCX_14.10716.10716.2	2	2.143	0.162	1	733.8	57.692307	R.GAIILAKLSIVPVR.R
RS2_MOUSE	MK_SCX_21.6052.6052.2	2	2.348	0.387	1	351.9	55	K.TYSYLTPDLWK.E
RS2_MOUSE	MK_SCX_2201.2946.2946.2	2	2.212	0.494	1	404.2	71.42857	K.ATFDAISK.T
RS2_MOUSE	MK_SCX_34.4847.4847.3	3	3.899	0.429	1	1518	65.909096	K.SPYQEFTDHLVK.T
RS2_MOUSE	MK_SCX_34.4919.4919.2	2	3.549	0.429	1	1756.6	81.818184	K.SPYQEFTDHLVK.T
RS2_MOUSE	MK_SCX_41.7114.7114.3	3	5.432	0.483	1	1346.8	33.653847	K.KLLMMAGIDDCYTSARGCTATLGNFAK.A
RS20_MOUSE	MK_SCX_31.4587.4587.3	3	3.118	0.347	1	451.7	47.727272	R.LIDLHSPSEIVK.Q
RS20_MOUSE	MK_SCX_31.4714.4714.2	2	3.893	0.411	1	815.5	68.181816	R.LIDLHSPSEIVK.Q
RS20_MOUSE	MK_SCX_39.3525.3525.3	3	4.839	0.548	1	804.5	44.642857	K.DTGKTPVEPEVAIHR.I
RS21_MOUSE	MK_SCX_12.6997.6997.2	2	3.138	0.446	1	958.5	71.42857	-.M*QNDAGEFVDLYVPR.K
RS21_MOUSE	MK_SCX_21.3734.3734.2	2	3.086	0.469	1	707.3	83.33333	R.MGESDDSILR.L
RS21_MOUSE	MK_SCX_30.3370.3370.3	3	4.21	0.416	1	789.8	50	K.DHASIQM*NVAEVDR.T
RS21_MOUSE	MK_SCX_30.3877.3877.3	3	4.024	0.324	1	1097.2	53.846157	K.DHASIQMNVAEVDR.T
RS21_MOUSE	MK_SCX_30.3878.3878.2	2	5.068	0.635	1	2410.8	88.46153	K.DHASIQMNVAEVDR.T
RS23_MOUSE	MK_SCX_2201.5670.5670.2	2	4.419	0.568	1	1529.6	90	K.VANVSLLALYK.G
RS24_MOUSE	MK_SCX_19.5439.5439.2	2	2.946	0.548	1	689.2	60.714287	K.TTGFGM*IYDSLDYAK.K
RS24_MOUSE	MK_SCX_19.6382.6382.2	2	4.626	0.517	1	1421.3	75	K.TTGFGMIYDSLDYAK.K
RS24_MOUSE	MK_SCX_20_1.9395.9395.2	2	3.311	0.303	1	558.7	72.72727	K.TTPDVIFVFGFR.T
RS24_MOUSE	MK_SCX_29.6332.6332.2	2	4.033	0.422	1	666.2	53.333336	K.TTGFGMIYDSLDYAKK.N
RS24_MOUSE	MK_SCX_33.4468.4468.2	2	2.181	0.318	1	389.6	75	K.QMVIDVLHPGK.A
RS24_MOUSE	MK_SCX_55.4341.4341.3	3	3.875	0.45	1	848.2	39.0625	R.KQMVIDVLHPGKATVPK.T
RS25_MOUSE	MK_SCX_2201.4255.4255.2	2	3.069	0.29	1	1384	93.75	R.AALQELLSK.G
RS25_MOUSE	MK_SCX_32.5055.5055.2	2	3.646	0.35	1	1417.3	80	R.DKLNNLVLFDK.A
RS25_MOUSE	MK_SCX_32.5087.5087.3	3	3.191	0.353	1	1038	52.499996	R.DKLNNLVLFDK.A
RS25_MOUSE	MK_SCX_53.4617.4617.3	3	3.228	0.261	1	718.1	43.75	K.VRDKLNNLVLFDK.A

RS25_MOUSE	MK_SCX_53.4627.4627.2	2	4.103	0.454	1	932	75	K.VRDKLNNLVLFDK.A
RS26_MOUSE	MK_SCX_16.6747.6747.2	2	5.042	0.541	1	1723.3	82.14286	R.DISEASVFDAYVLPK.L
RS26_MOUSE	MK_SCX_16.7086.7086.1	1	3.088	0.468	1	901.5	67.85714	R.DISEASVFDAYVLPK.L
RS26_MOUSE	MK_SCX_24.3357.3357.2	2	2.906	0.299	1	996.7	93.75	R.NIVEAAAVR.D
RS26_MOUSE	MK_SCX_53.3382.3382.2	2	3.433	0.476	1	775	71.42857	R.FRPAGAAPRPPPK.P
RS26_MOUSE	MK_SCX_55.3476.3476.3	3	3.064	0.24	1	788.2	47.727272	K.LHYCVSCAIHSK.V
RS27A_MOUSE	MK_SCX_30.4886.4886.2	2	3.347	0.437	1	850.2	55.555557	R.ECPSDECGAGVFMGSHFDR.H
RS27A_MOUSE	MK_SCX_51.3944.3944.3	3	3.937	0.282	1	695.3	39.473686	R.RECPSDECGAGVFM*GSHFDR.H
RS27A_MOUSE	MK_SCX_51.4466.4466.3	3	4.178	0.291	1	1795.2	46.05263	R.RECPSDECGAGVFMGSHFDR.H
RS27L_MOUSE	MK_SCX_21.4664.4664.2	2	2.444	0.428	1	341.9	62.5	R.LVQSPNSYFM*DVK.C
RS27L_MOUSE	MK_SCX_21.5020.5020.2	2	4.245	0.463	1	1569.1	83.33333	R.LVQSPNSYFMDVK.C
RS27L_MOUSE	MK_SCX_27.4222.4222.2	2	2.91	0.38	1	538	70	R.DLLHPSLEEEK.K
RS28_MOUSE	MK_SCX_19.5613.5613.2	2	4.135	0.273	1	1557.5	81.818184	R.EGDVLTLLESER.E
RS28_MOUSE	MK_SCX_21.3554.3554.2	2	3.1	0.506	1	692.2	93.75	R.VEFMDDTSR.S
RS28_MOUSE	MK_SCX_29.5891.5891.2	2	2.523	0.222	1	403.4	53.571426	R.EGDVLTLLESEREAR.R
RS28_MOUSE	MK_SCX_32.6266.6266.2	2	4.826	0.595	1	1360.3	76.666664	K.GPVREGDVLTLLESER.E
RS28_MOUSE	MK_SCX_32.6271.6271.3	3	5.434	0.38	1	1660.6	50	K.GPVREGDVLTLLESER.E
RS28_MOUSE	MK_SCX_47.6112.6112.3	3	5.146	0.571	1	791.2	40.27778	R.NVKGPVREGDVLTLLESER.E
RS28_MOUSE	MK_SCX_47.6120.6120.2	2	4.675	0.432	1	401.9	50	R.NVKGPVREGDVLTLLESER.E
RS3_MOUSE	MK_SCX_17.4466.4466.2	2	3.804	0.445	1	746.5	83.33333	K.DEILPTTPISEQK.G
RS3_MOUSE	MK_SCX_20_1.3973.3973.2	2	4.362	0.591	1	2341.8	87.5	R.ELAEDGYSGVEVR.V
RS3_MOUSE	MK_SCX_20_1.6640.6640.2	2	4.045	0.561	1	1143.3	80.769226	R.FGFPEGSVELYAEK.V
RS3_MOUSE	MK_SCX_24.8389.8389.3	3	4.672	0.497	1	1575.6	40.476192	K.FVDGLMIHSGDPVNYYVDTAVR.H
RS3_MOUSE	MK_SCX_51.3650.3650.3	3	5.005	0.403	1	1379.8	54.166668	K.KPLPDHVSIVEPK.D
RS3_MOUSE	MK_SCX_54.3872.3872.3	3	4.083	0.452	1	728.1	43.75	K.IGPKKPLPDHVSIVEPK.D
RS3_MOUSE	MK_SCX_54.3893.3893.2	2	3.632	0.547	1	726.9	62.5	K.IGPKKPLPDHVSIVEPK.D
RS30_MOUSE	MK_SCX_2201.4784.4784.2	2	2.569	0.21	1	418.7	61.11111	R.FVNVVPTFGK.K
RS30_MOUSE	MK_SCX_37.4590.4590.2	2	2.942	0.172	1	933.3	80	R.FVNVVPTFGKK.K
RS30_MOUSE	MK_SCX_43.4824.4824.2	2	3.456	0.362	1	874	85	R.RFVNVVPTFGK.K
RS30_MOUSE	MK_SCX_55.4176.4176.2	2	3.115	0.287	1	898.7	77.27273	R.RFVNVVPTFGKK.K
RS3A_MOUSE	MK_SCX_18.11862.11862.2	2	4.393	0.514	1	1456.9	75	R.VFEVSLADLQNDEVAFR.K
RS3A_MOUSE	MK_SCX_27.16705.16705.3	3	3.228	0.285	1	598.8	36.764706	R.VFEVSLADLQNDEVAFRK.F
RS3A_MOUSE	MK_SCX_29.3783.3783.2	2	3.663	0.253	1	1119.3	75	R.EVQTNDLKEVVNK.L
RS3A_MOUSE	MK_SCX_32.4082.4082.2	2	2.792	0.401	1	314.1	77.27273	K.LIPDSIGKDIEK.A
RS3A_MOUSE	MK_SCX_36.2765.2765.2	2	3.354	0.414	1	1067.2	70.83333	K.LMELHGEGGSSGK.A
RS3A_MOUSE	MK_SCX_37.3996.3996.3	3	3.224	0.301	1	752.2	52.499996	K.FKLITEDVQGK.N
RS3A_MOUSE	MK_SCX_46.6824.6824.3	3	4.565	0.392	1	1310.5	42.105263	K.GRVFEVSLADLQNDEVAFRK.F
RS4X_MOUSE	MK_SCX_13.5989.5989.2	2	4.368	0.627	1	1712.3	75	K.FDTGNLCMVTGGANLGR.I
RS4X_MOUSE	MK_SCX_16.4933.4933.2	2	3.305	0.4	1	1249.3	75	K.VNDTIQIDLETGK.I
RS4X_MOUSE	MK_SCX_16.6469.6469.2	2	5.511	0.582	1	816.3	75	R.TDITYPAGFM*DVISIDK.T
RS4X_MOUSE	MK_SCX_16.7363.7363.2	2	5.021	0.502	1	886.1	78.125	R.TDITYPAGFMDVISIDK.T
RS4X_MOUSE	MK_SCX_23.7820.7820.2	2	4.695	0.584	1	980.9	47.727272	R.TDITYPAGFMDVISIDKTGENFR.L
RS4X_MOUSE	MK_SCX_23.9112.9112.2	2	2.122	0.191	1	463.7	63.636364	K.LRECLPLIIFLR.N
RS4X_MOUSE	MK_SCX_25.7641.7641.3	3	4.011	0.486	1	493.6	36.11111	K.VNDTIQIDLETGKITDFIK.F
RS4X_MOUSE	MK_SCX_28.7523.7523.2	2	2.858	0.25	1	367.8	44.444447	K.VRTDITYPAGFMDVISIDK.T
RS4X_MOUSE	MK_SCX_28.7541.7541.3	3	3.426	0.367	1	477.5	37.5	K.VRTDITYPAGFMDVISIDK.T
RS4X_MOUSE	MK_SCX_38.4256.4256.3	3	3.742	0.345	1	1681.1	72.22222	R.TIRYPDPLIK.V
RS4X_MOUSE	MK_SCX_59.8995.8995.2	2	3.257	0.47	1	346	62.5	R.ERHPGSFDVVHVK.D
RS4X_MOUSE	MK_SCX_59.9735.9735.3	3	3.634	0.412	1	705.3	45.833336	R.ERHPGSFDVVHVK.D
RS5_MOUSE	MK_SCX_17.12599.12599.2	2	4.22	0.524	1	1101.7	55.263157	K.WSTDDVQINDISLQDYIAVK.E
RS5_MOUSE	MK_SCX_24.14401.14401.3	3	3.731	0.527	1	553.7	35.714287	K.WSTDDVQINDISLQDYIAVKEK.Y

RS6_MOUSE	MK_SCX_17.4673.4673.2	2	2.841	0.532	1	463.4	81.818184	K.DIPGLTDTTVPR.R
RS6_MOUSE	MK_SCX_19.4994.4994.2	2	5.002	0.542	1	2531.6	82.14286	R.MATEVAADALGEEWK.G
RS6_MOUSE	MK_SCX_27.7020.7020.2	2	5.441	0.629	1	1871.7	68.42105	R.MATEVAADALGEEWKGYVVR.I
RS6_MOUSE	MK_SCX_27.7155.7155.3	3	4.909	0.616	1	1457.5	46.05263	R.MATEVAADALGEEWKGYVVR.I
RS6_MOUSE	MK_SCX_36.10383.10383.2	2	2.152	0.249	1	300.8	60.000004	K.KNKEEAAEYAK.L
RS6_MOUSE	MK_SCX_46.6181.6181.3	3	4.383	0.369	1	627.6	40	K.RM*ATEVAADALGEEWKGYVVR.I
RS6_MOUSE	MK_SCX_46.6517.6517.3	3	5.939	0.631	1	3555.2	51.25	K.RMATEVAADALGEEWKGYVVR.I
RS6_MOUSE	MK_SCX_50.3797.3797.3	3	5.463	0.5	1	1955.9	53.333336	K.KGEKDIPGLTDTTVPR.R
RS6_MOUSE	MK_SCX_50.3823.3823.2	2	5.621	0.396	1	1242.4	80	K.KGEKDIPGLTDTTVPR.R
RS6_MOUSE	MK_SCX_55.3546.3546.3	3	3.875	0.429	1	706	32.5	R.ISGGNDKQGFPMKQGVLTHGR.V
RS7_MOUSE	MK_SCX_24.10404.10404.3	3	4.971	0.368	1	1362.1	40.476192	R.TLTAVHDAILEDLVFPSEIVGK.R
RS7_MOUSE	MK_SCX_24.10654.10654.2	2	5.389	0.623	1	2219.3	66.66667	R.TLTAVHDAILEDLVFPSEIVGK.R
RS7_MOUSE	MK_SCX_30.9036.9036.3	3	4.583	0.419	1	1714.5	32.75862	K.IVKPNGEKPDEFESGISQALLELEMNSDLK.A
RS7_MOUSE	MK_SCX_33.7219.7219.2	2	4.386	0.345	1	1550.8	75	R.KAIIIFVPVPQLK.S
RS7_MOUSE	MK_SCX_35.13428.13428.2	2	3.515	0.507	1	334.1	36.363636	R.TLTAVHDAILEDLVFPSEIVGKR.I
RS7_MOUSE	MK_SCX_35.13840.13840.3	3	5.919	0.493	1	2295.7	43.18182	R.TLTAVHDAILEDLVFPSEIVGKR.I
RS7_MOUSE	MK_SCX_48.4191.4191.2	2	4.917	0.498	1	1879.9	70.588234	K.AQQNNVEHKVETFSGVYK.K
RS7_MOUSE	MK_SCX_48.4329.4329.2	2	4.528	0.522	1	1032.9	59.375	R.ELNITAAKEIEVGGGRK.A
RS7_MOUSE	MK_SCX_49.4229.4229.3	3	4.723	0.519	1	847	45.588234	K.AQQNNVEHKVETFSGVYK.K
RS7_MOUSE	MK_SCX_58.5302.5302.2	2	2.453	0.297	1	436.1	85.71429	R.RILPKPTR.K
RS8_MOUSE	MK_SCX_18.5126.5126.2	2	3.831	0.503	1	2344.2	85.71429	R.IIDVVYNASNNELVR.T
RS8_MOUSE	MK_SCX_20_1.5608.5608.3	3	4.701	0.434	1	2288.9	53.571426	R.IIDVVYNASNNELVR.T
RS8_MOUSE	MK_SCX_21.4672.4672.3	3	4.878	0.352	1	2084.7	60.416668	K.ISSLLEEQFQQGK.L
RS8_MOUSE	MK_SCX_21.4725.4725.1	1	3.701	0.368	1	662.6	66.66667	K.ISSLLEEQFQQGK.L
RS8_MOUSE	MK_SCX_21.6404.6404.2	2	4.171	0.389	1	1217.3	71.42857	R.LDVGNFSWGSECCTR.K
RS8_MOUSE	MK_SCX_2201.4480.4480.2	2	4.714	0.41	1	1732.3	75	K.ISSLLEEQFQQGK.L
RS8_MOUSE	MK_SCX_29.7276.7276.3	3	3.52	0.35	1	1035	41.666664	R.ADGYVLEGKELEFYLR.K
RS9_MOUSE	MK_SCX_2201.3970.3970.2	2	2.739	0.316	1	488.3	91.66667	K.IEDFLER.R
RS9_MOUSE	MK_SCX_2201.6588.6588.2	2	2.838	0.201	1	488.3	75	K.QVVNIPSFIVR.L
RS9_MOUSE	MK_SCX_39.6339.6339.2	2	3.804	0.289	1	1437.3	90.909096	R.KQVVNIPSFIVR.L
RS9_MOUSE	MK_SCX_39.7070.7070.2	2	2.627	0.186	1	830	83.33333	K.MKLDYILGLK.I
RS9_MOUSE	MK_SCX_47.4532.4532.2	2	3.785	0.344	1	905.3	88.88889	R.RLFEGNALLR.R
RS9_MOUSE	MK_SCX_51.3800.3800.3	3	3.015	0.387	1	477.1	38.636364	R.LDSQKHIDFSLR.S
RSMB_MOUSE	MK_SCX_23.5628.5628.2	2	3.11	0.242	1	792.5	92.85714	R.VLGLVLLR.G
RSMB_MOUSE	MK_SCX_27.5051.5051.3	3	4.114	0.535	1	791.3	36.25	R.GENLVSMTVEGPPPKDTGIAR.V
RSMB_MOUSE	MK_SCX_27.5079.5079.2	2	4.749	0.675	1	1558.1	65	R.GENLVSMTVEGPPPKDTGIAR.V
RSSA_MOUSE	MK_SCX_18.5515.5515.2	2	4.458	0.613	1	1839.8	68.75	R.AIVAIENPADVSVISSR.N
RSSA_MOUSE	MK_SCX_21.7277.7277.2	2	3.434	0.616	1	508.3	67.85714	R.FTPGTFTNQIQAAFR.E
RSU1_MOUSE	MK_SCX_16.9297.9297.2	2	3.492	0.484	1	510.1	34.782608	K.NLEVLNFFNNQIEELPTQISSLQK.L
RSU1_MOUSE	MK_SCX_16.9339.9339.3	3	5.612	0.467	1	1235.7	36.95652	K.NLEVLNFFNNQIEELPTQISSLQK.L
RSU1_MOUSE	MK_SCX_17.6680.6680.2	2	3.473	0.526	1	713.7	71.875	R.LTVLPPELGNLDLTGQK.Q
RT02_MOUSE	MK_SCX_16.4635.4635.2	2	5.314	0.576	1	1228.3	65	K.VTGAPVPAVSEPQDGDDFQSR.I
RT09_MOUSE	MK_SCX_20_1.8368.8368.2	2	2.772	0.273	1	602.7	58.333332	R.AIAYLFPSGLFEK.R
RT09_MOUSE	MK_SCX_2201.3510.3510.2	2	2.186	0.313	1	302.7	72.22222	R.QAGLLTPDPR.I
RT11_MOUSE	MK_SCX_18.7190.7190.2	2	3.623	0.638	1	349.2	52.63158	R.FSLYPPVPGQESSLQWAGMK.F
RT11_MOUSE	MK_SCX_35.4212.4212.2	2	2.056	0.291	1	365.8	55.555557	K.FEDVPIAHIK.A
RT11_MOUSE	MK_SCX_37.3708.3708.2	2	2.919	0.194	1	1352.2	71.875	K.KGTGIAAQTAGIAAAAK.A
RT11_MOUSE	MK_SCX_37.3729.3729.3	3	3.243	0.296	1	869.7	48.4375	K.KGTGIAAQTAGIAAAAK.A
RT14_MOUSE	MK_SCX_16.5573.5573.2	2	2.807	0.356	1	873.3	60.714287	K.DLQEM*AGDEIAALPR.D
RT14_MOUSE	MK_SCX_16.6321.6321.2	2	2.845	0.348	1	760.7	53.571426	K.DLQEMAGDEIAALPR.D
RT15_MOUSE	MK_SCX_14.3836.3836.2	2	2.129	0.252	1	351.8	53.571426	K.NEGVPENPSNAVPEK.T

RT15_MOUSE	MK_SCX_21.7238.7238.2	2	2.975	0.524	1	1201.4	81.818184	R.TLEAQIIALTVR.I
RT16_MOUSE	MK_SCX_27.11199.11199.3	3	3.729	0.484	1	459.1	32.894737	K.LLGLSGFFPLHPMMITNAER.L
RT16_MOUSE	MK_SCX_29.5128.5128.3	3	3.43	0.522	1	577.5	38.235294	R.FVEQLGSYDPLPNSHGEK.L
RT18B_MOUSE	MK_SCX_16.5854.5854.2	2	5.721	0.62	1	1061.3	61.904762	R.GPPEEDAPSSLPVSPYESEPWK.Y
RT21_MOUSE	MK_SCX_19.5084.5084.2	2	3.552	0.429	1	670	70.83333	R.ILTTDGLTEVISR.R
RT21_MOUSE	MK_SCX_21.4124.4124.2	2	4.436	0.599	1	2644.6	84.61539	R.TVMVQEGNVEGAYR.T
RT22_MOUSE	MK_SCX_17.9088.9088.2	2	2.202	0.141	1	300.4	35	K.LM*TQAQLEEATRLAVEAAKVR.L
RT22_MOUSE	MK_SCX_2201.3978.3978.2	2	4.273	0.439	1	2314.2	90.909096	K.LMTQAQLEEATR.L
RT22_MOUSE	MK_SCX_26.8595.8595.3	3	6.468	0.654	1	2101	40.384613	R.DLVDDATSLVQLYHMLHPDGQSAQEAK.E
RT22_MOUSE	MK_SCX_50.4548.4548.2	2	3.275	0.353	1	505	64.28571	K.TFRPAIQPLKPPTYK.L
RT22_MOUSE	MK_SCX_50.4551.4551.3	3	3.664	0.391	1	836.5	42.857143	K.TFRPAIQPLKPPTYK.L
RT23_MOUSE	MK_SCX_18.6049.6049.2	2	4.868	0.528	1	1605.8	76.92308	K.ADIQDIFYQEDQIR.A
RT23_MOUSE	MK_SCX_2201.6396.6396.2	2	3.325	0.205	1	1201.4	83.33333	K.AFDLFNPNFK.S
RT23_MOUSE	MK_SCX_24.6072.6072.3	3	4.411	0.416	1	871.7	41.25	K.YTELQNLGETDEEKLFVETGK.A
RT23_MOUSE	MK_SCX_27.3299.3299.3	3	4.154	0.411	1	685.2	43.055553	R.LQASSEGHEPQEDDDLAQR.G
RT23_MOUSE	MK_SCX_27.3344.3344.2	2	4.946	0.355	1	1360	66.66667	R.LQASSEGHEPQEDDDLAQR.G
RT23_MOUSE	MK_SCX_38.3368.3368.3	3	5.079	0.473	1	1151.5	39.772728	R.LQASSEGHEPQEDDDLAQRGQVK.Q
RT23_MOUSE	MK_SCX_50.5533.5533.3	3	3.062	0.424	1	323.1	35.714287	R.AGVLKEKPLWYDIYK.A
RT28_MOUSE	MK_SCX_31.5256.5256.3	3	4.096	0.546	1	1225.2	51.785713	R.LESPKPVESFASM*LR.H
RT28_MOUSE	MK_SCX_31.6086.6086.3	3	3.63	0.362	1	1010	46.42857	R.LESPKPVESFASMLR.H
RT28_MOUSE	MK_SCX_31.6118.6118.2	2	2.959	0.598	1	542.3	67.85714	R.LESPKPVESFASMLR.H
RT29_MOUSE	MK_SCX_14.4782.4782.2	2	3.175	0.365	1	539.2	68.181816	R.FLSNCNPEQLER.L
RT29_MOUSE	MK_SCX_50.6048.6048.3	3	3.124	0.257	1	781.9	38.333332	R.VRNATDAVGVVLKELK.A
RT31_MOUSE	MK_SCX_16.7173.7173.2	2	3.005	0.571	1	474	52.380955	K.AFADEPPEPEASPSLWEIEFAK.Q
RT31_MOUSE	MK_SCX_16.9963.9963.2	2	6.373	0.647	1	2928.1	68.181816	R.NEFLSPELVAAASAVADSLPFDK.Q
RT31_MOUSE	MK_SCX_28.7642.7642.2	2	3.476	0.376	1	857.7	83.33333	K.DLLDIIKDMK.V
RT31_MOUSE	MK_SCX_40.3056.3056.3	3	3.726	0.268	1	1227.4	50	R.KPSASLEATVDR.L
RT31_MOUSE	MK_SCX_40.3085.3085.2	2	3.438	0.517	1	567.1	77.27273	R.KPSASLEATVDR.L
RT31_MOUSE	MK_SCX_55.2914.2914.3	3	4.336	0.407	1	1331.2	48.076923	R.GRKPSASLEATVDR.L
RT32_MOUSE	MK_SCX_20_1.4101.4101.2	2	3.853	0.536	1	644.2	70.83333	K.QLEQGPM*IEQLSK.V
RT33_MOUSE	MK_SCX_20_1.5291.5291.2	2	3.882	0.437	1	1816.3	86.36364	K.VVSLFSEQPLAK.K
RT34_MOUSE	MK_SCX_13.5394.5394.2	2	3.611	0.311	1	729.5	57.14286	K.NGDTSVQEPLLNLER.T
RT34_MOUSE	MK_SCX_21.5422.5422.2	2	2.224	0.29	1	370.6	70	R.LNSVPYPPLLR.A
RT34_MOUSE	MK_SCX_47.4038.4038.3	3	5.027	0.515	1	2754.8	60.000004	K.HEEEAFTAFTAKPEDR.L
RT35_MOUSE	MK_SCX_21.7812.7812.3	3	4.089	0.502	1	382.4	26.923079	K.M*DTDQDWPSVYPTAAPFKPSAVPLPVR.M
RT35_MOUSE	MK_SCX_21.7924.7924.2	2	4.144	0.528	1	1937	71.42857	K.SSSENSVLQTLLQMR.A
RT35_MOUSE	MK_SCX_37.3377.3377.2	2	3.062	0.539	1	645	71.42857	R.LKNEGENEASLAQYK.E
RT36_MOUSE	MK_SCX_25.5120.5120.2	2	5.234	0.623	1	523.7	52.499996	K.GSTSPDLLM*HQGPPDTAEIIK.S
RT36_MOUSE	MK_SCX_25.5498.5498.2	2	5.734	0.643	1	1069.3	50	K.GSTSPDLLMHQGPPDTAEIIK.S
RT36_MOUSE	MK_SCX_38.3603.3603.2	2	3.59	0.357	1	582.4	79.16667	R.KPMSQEEM*EFIQR.G
RT36_MOUSE	MK_SCX_38.3616.3616.3	3	4.684	0.344	1	2103	60.416668	R.KPMSQEEM*EFIQR.G
RT36_MOUSE	MK_SCX_38.3756.3756.2	2	3.672	0.447	1	603.9	83.33333	R.KPM*SQEEMEFIQR.G
RT36_MOUSE	MK_SCX_38.3777.3777.3	3	3.83	0.295	1	1567.3	54.166668	R.KPM*SQEEMEFIQR.G
RT36_MOUSE	MK_SCX_38.4115.4115.2	2	4.095	0.479	1	1037.4	79.16667	R.KPMSQEEMEFIQR.G
RT36_MOUSE	MK_SCX_38.4128.4128.3	3	4.557	0.354	1	2494.2	62.5	R.KPMSQEEMEFIQR.G
RT36_MOUSE	MK_SCX_40.4510.4510.2	2	5.474	0.641	1	648	47.826088	K.LSASEALGSAALPSHSSAISQHSK.G
RT36_MOUSE	MK_SCX_40.4638.4638.3	3	4.501	0.477	1	1004.7	34.782608	K.LSASEALGSAALPSHSSAISQHSK.G
RT36_MOUSE	MK_SCX_55.3348.3348.3	3	4.126	0.187	1	1261.3	51.923077	R.RKPMSQEEM*EFIQR.G
RT36_MOUSE	MK_SCX_55.3420.3420.3	3	3.761	0.352	1	802	46.153847	R.RKPM*SQEEMEFIQR.G
RT36_MOUSE	MK_SCX_55.3784.3784.2	2	5.084	0.518	1	1634.1	76.92308	R.RKPMSQEEMEFIQR.G
RT36_MOUSE	MK_SCX_55.3812.3812.3	3	4.573	0.41	1	2032.5	51.923077	R.RKPMSQEEMEFIQR.G

RT4I1_MOUSE	MK_SCX_26.7445.7445.2	2	4.351	0.535	1	724.1	50	R.FTQNMMLPIIHYPNEVIIK.V
RU17_MOUSE	MK_SCX_31.3648.3648.3	3	3.235	0.502	1	878.5	50	K.M*WDPHNDPNAQGDAFK.T
RU17_MOUSE	MK_SCX_31.3957.3957.3	3	4.234	0.415	1	1342.1	53.333336	K.MWDPHNDPNAQGDAFK.T
RU17_MOUSE	MK_SCX_31.3958.3958.2	2	4.213	0.54	1	745.3	66.66667	K.MWDPHNDPNAQGDAFK.T
RU17_MOUSE	MK_SCX_55.3574.3574.3	3	3.21	0.373	1	929.3	47.5	R.REFEVYGPIKR.I
RU1C_MOUSE	MK_SCX_27.8126.8126.3	3	3.9	0.464	1	699.8	36.25	K.WMEEQAQSLIDKTTAAFQQGK.I
RU1C_MOUSE	MK_SCX_29.5974.5974.3	3	4.007	0.495	1	502.1	41.17647	K.FYCDYCDTYLTHDSPSVR.K
RU2A_MOUSE	MK_SCX_23.13077.13077.3	3	3.822	0.468	1	616.2	30.555555	R.GYKIPVIENLGATLDQFDAIDFSDNEIR.K
RU2A_MOUSE	MK_SCX_25.8109.8109.3	3	3.709	0.449	1	675.1	27.000002	K.GGPSAGDVEAIKNAIANASTLAEVER.L
RU2A_MOUSE	MK_SCX_41.5053.5053.2	2	2.309	0.328	1	652.7	75	R.KLDGFPLLR.R
S100G_MOUSE	MK_SCX_20_1.7712.7712.2	2	4.776	0.464	1	2052.9	90.909096	K.LLIQSEFPSLLK.A
S100G_MOUSE	MK_SCX_43.3578.3578.3	3	4.646	0.49	1	1656.1	53.125	K.YAAKEGDPDQLSKEELK.L
S10A1_MOUSE	MK_SCX_16.8033.8033.2	2	4.339	0.381	1	1782.5	75	K.DLLQTELSGFLDVQK.D
S10A1_MOUSE	MK_SCX_26.9072.9072.3	3	3.732	0.339	1	736.8	35.294117	K.ELKDLLQTELSGFLDVQK.D
S10AA_MOUSE	MK_SCX_27.6219.6219.2	2	4.827	0.562	1	1204.1	71.875	R.EFPGFLENQKDPLAVDK.I
S10AA_MOUSE	MK_SCX_44.7023.7023.3	3	4.162	0.515	1	631.5	43.75	-.PSQMEHAMETMMLTFHR.F
S10AB_MOUSE	MK_SCX_19.9158.9158.2	2	5.063	0.563	1	2565.3	80	K.TEFLSFMNTELAAFTK.N
S12A1_MOUSE	MK_SCX_21.4998.4998.2	2	2.778	0.324	1	1266.7	77.77778	K.VFEEMIEPYR.L
S12A1_MOUSE	MK_SCX_33.8963.8963.3	3	3.133	0.143	1	1109.6	43.333332	K.SYRQVRLNELLQEHSR.A
S12A1_MOUSE	MK_SCX_49.4481.4481.3	3	5.053	0.522	1	1098.2	40.27778	K.KDGNISSIQSMHVGEFNQK.L
S12A1_MOUSE	MK_SCX_51.4438.4438.3	3	4.305	0.372	1	925.6	42.857143	K.VNRPSLLEIHEQLAK.N
S12A1_MOUSE	MK_SCX_51.4488.4488.2	2	3.805	0.396	1	806.2	64.28571	K.VNRPSLLEIHEQLAK.N
S12A2_MOUSE	MK_SCX_27.5572.5572.3	3	5.237	0.582	1	2467.1	42.307693	R.AAAAAAAAAAAAAAAGAAGKETPAAGK.A
S12A3_MOUSE	MK_SCX_42.5338.5338.2	2	3.477	0.257	1	795.6	77.77778	K.RFEDMIAPFR.L
S12A6_MOUSE	MK_SCX_17.3671.3671.2	2	4.923	0.503	1	1627.8	67.64706	R.LTSIGSDEDEETETYQEK.V
S12A7_MOUSE	MK_SCX_16.3771.3771.2	2	4.778	0.521	1	1433.2	52.272724	R.TEEPESPESVDQTSPTPGDGNPR.E
S12A7_MOUSE	MK_SCX_17.9621.9621.2	2	4.258	0.465	1	1042.6	55.555557	R.QGDENYMEFLEVLTEGLNR.V
S12A7_MOUSE	MK_SCX_19.5396.5396.2	2	4.43	0.512	1	1581.1	70	K.SQDAQLVLLNMPGPPK.S
S17A5_MOUSE	MK_SCX_17.4055.4055.2	2	5.502	0.558	1	1957.6	65	R.GPAGNDDEESSDSTPLLPGAR.Q
S23A1_MOUSE	MK_SCX_40.3012.3012.3	3	4.799	0.583	1	815	38.095238	K.TPEDPGSPKQHEVVDSAGTSTR.D
S27A2_MOUSE	MK_SCX_18.6575.6575.2	2	3.974	0.424	1	388.1	66.66667	K.TFVPM*TENIYNAIIDK.T
S27A2_MOUSE	MK_SCX_18.7304.7304.2	2	4.407	0.462	1	500.1	66.66667	K.TFVPMTENIYNAIIDK.T
S27A2_MOUSE	MK_SCX_20_1.15884.15884.2	2	3.367	0.397	1	1071.9	77.27273	R.IQDTIEITGTFK.H
S27A2_MOUSE	MK_SCX_20_1.5922.5922.2	2	4.1	0.452	1	1095.2	76.92308	K.ITQLTPFIGYAGGK.T
S27A2_MOUSE	MK_SCX_21.7509.7509.2	2	3.382	0.494	1	690.8	40.384613	R.TSNTNGVDTILDKVDGVSAEPTPESWR.S
S27A2_MOUSE	MK_SCX_2201.7359.7359.3	3	4.918	0.424	1	1360.6	37.5	R.TSNTNGVDTILDKVDGVSAEPTPESWR.S
S27A2_MOUSE	MK_SCX_2201.7864.7864.3	3	4.297	0.513	1	1203.4	36.458336	K.VTLM*EEGFNPTVIKDTLYFM*DDAEK.T
S27A2_MOUSE	MK_SCX_2201.9870.9870.3	3	4.993	0.531	1	1587.4	35.416664	K.VTLMEEGFNPTVIKDTLYFMDDAEK.T
S27A2_MOUSE	MK_SCX_34.5579.5579.3	3	4.548	0.389	1	1438.7	51.785713	R.KVTLMEEGFNPTVIK.D
S27A2_MOUSE	MK_SCX_50.6109.6109.2	2	4.202	0.52	1	1077.6	56.666668	K.LFQHIAEYLPSYARPR.F
S4A4_MOUSE	MK_SCX_14.10849.10849.2	2	5.188	0.627	1	1112.1	45.652176	R.DAEASNVLVGEVDFLDTPFIAFVR.L
S4A4_MOUSE	MK_SCX_14.11000.11000.3	3	3.684	0.369	1	395.5	29.347824	R.DAEASNVLVGEVDFLDTPFIAFVR.L
S4A4_MOUSE	MK_SCX_18.6846.6846.2	2	4.58	0.528	1	2058	73.52941	R.LQQAVMLGALTEVPVPTR.F
S4A4_MOUSE	MK_SCX_2201.7846.7846.2	2	2.522	0.445	1	503.6	75	R.FLFILLGPK.G
S4A4_MOUSE	MK_SCX_25.6608.6608.3	3	5.174	0.53	1	1192.8	44.736843	K.IPMDIMEQQPFLSDNKPLDR.E
S4A4_MOUSE	MK_SCX_27.7318.7318.2	2	5.255	0.618	1	2739.8	81.25	R.AIATLMSDEVFHDIAYK.A
S4A4_MOUSE	MK_SCX_29.14577.14577.3	3	3.859	0.477	1	347.1	25.961538	R.EASSLPQLVEM*IADHQIETGLLKPDLK.D
S4A4_MOUSE	MK_SCX_37.6957.6957.3	3	3.268	0.217	1	320.3	27.380953	K.IPMDIMEQQPFLSDNKPLDRER.S
S4A4_MOUSE	MK_SCX_40.4184.4184.3	3	3.773	0.522	1	757.1	40.789474	R.NLTSSSLNDISDKPEKDQLK.N
S4A4_MOUSE	MK_SCX_52.3644.3644.3	3	4.93	0.46	1	711.5	41.666664	R.NLTSSSLNDISDKPEKDQLKNK.F
S6A19_MOUSE	MK_SCX_19.5978.5978.2	2	4.491	0.441	1	1146.9	90.909096	R.IPSLDELEVIEK.E

S6A19_MOUSE	MK_SCX_38.7055.7055.3	3	4.018	0.618	1	526.3	38.157894	R.IPSLDELEVIEKEEAGSRPK.W
SACS_MOUSE	MK_SCX_17.5145.5145.2	2	2.479	0.214	1	314.3	42.857143	R.LINPENMGFEQSGQR.E
SACS_MOUSE	MK_SCX_28.7019.7019.2	2	2.822	0.219	1	550.5	38.88889	R.VM*ECTACIIIKLENFIQQK.V
SAFB2_MOUSE	MK_SCX_18.3733.3733.2	2	4.701	0.505	1	995.8	70	K.VTLADEEAPM*EPENEK.I
SAFB2_MOUSE	MK_SCX_18.4141.4141.2	2	4.674	0.39	1	687	60.000004	K.VTLADEEAPMEPENEK.I
SAFB2_MOUSE	MK_SCX_39.3208.3208.3	3	4.284	0.469	1	1066.9	38.88889	R.APTAALSPEPQDSKEDVKK.F
SAHH_MOUSE	MK_SCX_19.5329.5329.2	2	3.995	0.556	1	1318.3	84.61539	K.ALDIAENEMPGLMR.M
SAHH_MOUSE	MK_SCX_23.4883.4883.2	2	4.09	0.478	1	1660.2	90	K.VADIGLAAWGR.K
SAHH_MOUSE	MK_SCX_24.6255.6255.2	2	3.327	0.421	1	499.6	47.368423	K.MMSNGILKVPAINVNDSVTK.S
SAHH_MOUSE	MK_SCX_24.6294.6294.3	3	5.141	0.553	1	823.8	43.421055	K.MMSNGILKVPAINVNDSVTK.S
SAHH_MOUSE	MK_SCX_25.4914.4914.2	2	2.547	0.28	1	1331	93.75	K.YPQLLSGIR.G
SAHH_MOUSE	MK_SCX_33.3766.3766.2	2	4.098	0.56	1	1697.7	71.42857	R.GISEETTTGVHNLYK.M
SAHH_MOUSE	MK_SCX_34.4877.4877.2	2	4.752	0.303	1	814.2	71.42857	R.KALDIAENEMPGLM*R.M
SAHH_MOUSE	MK_SCX_34.5489.5489.2	2	5.524	0.547	1	2198.2	82.14286	R.KALDIAENEMPGLMR.M
SAHH_MOUSE	MK_SCX_34.5561.5561.3	3	4.361	0.397	1	1128.7	51.785713	R.KALDIAENEMPGLMR.M
SAHH_MOUSE	MK_SCX_55.4406.4406.3	3	4.948	0.556	1	2395.8	54.6875	K.KLDEAVAEAHLGKLNVK.L
SAHH3_MOUSE	MK_SCX_16.4356.4356.2	2	4.637	0.583	1	1321.7	76.47059	R.DGGEALVSPDGTVTEAPR.T
SAHH3_MOUSE	MK_SCX_17.5849.5849.2	2	5.242	0.347	1	2503.3	76.666664	R.EIEIAEQEM*PALMALR.K
SAHH3_MOUSE	MK_SCX_17.6324.6324.2	2	4.423	0.414	1	1552.1	76.666664	R.EIEIAEQEMPALM*ALR.K
SAHH3_MOUSE	MK_SCX_17.6711.6711.2	2	6.004	0.533	1	2280.4	83.33333	R.EIEIAEQEMPALMALR.K
SAHH3_MOUSE	MK_SCX_44.7533.7533.3	3	6.446	0.595	1	2930.7	45.192307	K.KMDEYVASLHLPTFDAHLTELTDEQAK.Y
SAHH3_MOUSE	MK_SCX_50.3585.3585.2	2	4.656	0.642	1	1068.2	68.42105	R.HRDGGEALVSPDGTVTEAPR.T
SAHH3_MOUSE	MK_SCX_51.3477.3477.3	3	3.011	0.302	1	511.3	38.46154	K.IKGIVEESVTGVHR.L
SAHH3_MOUSE	MK_SCX_51.3534.3534.3	3	5.219	0.551	1	2271.5	47.368423	R.HRDGGEALVSPDGTVTEAPR.T
SAM68_MOUSE	MK_SCX_43.5440.5440.3	3	4.419	0.321	1	1499.3	53.846157	K.KDDEENYLDLFSHK.N
SAP_MOUSE	MK_SCX_17.9219.9219.2	2	4.225	0.414	1	1045.5	71.42857	K.EVVDSYLPVILDMIK.G
SAP_MOUSE	MK_SCX_25.8655.8655.3	3	3.366	0.525	1	662.8	39.473686	R.VVAPFMSNIPLLLYPQDHPR.S
SAP_MOUSE	MK_SCX_32.4042.4042.3	3	3.443	0.16	1	1047.2	48.076923	K.QLESNKIPEVDMAR.V
SAP3_MOUSE	MK_SCX_15.7602.7602.2	2	4.783	0.587	1	1070.6	61.904762	K.SLTIQPDPIVVPGDVVVSLEGK.T
SAP3_MOUSE	MK_SCX_15.7636.7636.3	3	3.892	0.485	1	1016.8	36.904762	K.SLTIQPDPIVVPGDVVVSLEGK.T
SAP3_MOUSE	MK_SCX_28.4886.4886.2	2	4.553	0.604	1	1099.3	65.625	K.TSVPLTAPQKVELTVEK.E
SAP3_MOUSE	MK_SCX_30.6903.6903.3	3	3.573	0.357	1	715.1	44.642857	K.VELTVEKEVAGFWVK.I
SAPS1_MOUSE	MK_SCX_25.5053.5053.3	3	4.185	0.346	1	323	28.57143	K.EADMSSIQIPSSPPAHGSPQLR.S
SAR1B_MOUSE	MK_SCX_15.8943.8943.2	2	4.222	0.587	1	918.4	50	K.EELDSLMTDETIANVPILILGNK.I
SAR1B_MOUSE	MK_SCX_24.8353.8353.3	3	3.201	0.122	1	537.1	30.952381	K.GSVSLKELNARPLEVFM*CSVLK.R
SARDH_MOUSE	MK_SCX_23.4076.4076.2	2	3.468	0.364	1	1168.1	85	R.IEGIQNMPNVR.D
SARDH_MOUSE	MK_SCX_25.5637.5637.3	3	5.312	0.474	1	1278.5	31	R.VAAVETEHGSIQTPCVVNCAGVWASK.V
SARDH_MOUSE	MK_SCX_31.4044.4044.3	3	3.507	0.437	1	406.6	44.642857	K.AYGIESHVLSPAETK.S
SARDH_MOUSE	MK_SCX_31.5028.5028.2	2	3.257	0.332	1	513.5	57.14286	K.NYSVVFPHDEPLAGR.N
SARDH_MOUSE	MK_SCX_32.3963.3963.2	2	4.372	0.318	1	814.3	64.28571	K.AYGIESHVLSPAETK.S
SAS10_MOUSE	MK_SCX_29.3908.3908.2	2	3.286	0.327	1	434.7	57.14286	K.ESPELLELIEDLQAK.L
SASH1_MOUSE	MK_SCX_13.6632.6632.1	1	2.272	0.202	1	715.2	59.090908	K.M*ITIEEALARLK.E
SBP1_MOUSE	MK_SCX_17.4972.4972.2	2	5.89	0.539	1	1288.9	70.588234	R.NTGTEAPDYLATVDVDPK.S
SBP1_MOUSE	MK_SCX_17.5067.5067.1	1	3.167	0.473	1	648	58.823532	R.NTGTEAPDYLATVDVDPK.S
SBP1_MOUSE	MK_SCX_19.5338.5338.2	2	4.52	0.54	1	934.5	71.42857	R.IPGGPQMIQLSLDGK.R
SBP1_MOUSE	MK_SCX_2201.5711.5711.2	2	2.23	0.261	1	750.9	83.33333	K.LILPGLISSR.I
SBP1_MOUSE	MK_SCX_2201.6688.6688.2	2	3.26	0.408	1	1134	75	R.LAGQIFLGGSIVR.G
SBP1_MOUSE	MK_SCX_26.7945.7945.3	3	4.232	0.502	1	1138.9	38.75	R.LYATTSLYSAWDKQFYPDLIR.E
SBP1_MOUSE	MK_SCX_28.6736.6736.2	2	4.597	0.57	1	1035.1	57.5	K.GTWEKPGDAAPMGYDFWYQPR.H
SBP1_MOUSE	MK_SCX_28.6751.6751.3	3	5.007	0.571	1	1297.4	38.75	K.GTWEKPGDAAPMGYDFWYQPR.H
SBP1_MOUSE	MK_SCX_32.5440.5440.3	3	3.495	0.3	1	651.9	48.333332	R.IPGGPQMIQLSLDGKR.L

SBP1_MOUSE	MK_SCX_36.5802.5802.2	2	4.603	0.547	1	1398.5	73.333336	K.RIPGGPQMIQLSLDGK.R
SBP1_MOUSE	MK_SCX_36.5847.5847.3	3	4.229	0.379	1	954.5	38.333332	K.RIPGGPQMIQLSLDGK.R
SBP1_MOUSE	MK_SCX_38.5538.5538.2	2	2.695	0.129	1	711.8	63.636364	R.NKLILPGLISSR.I
SBP1_MOUSE	MK_SCX_42.3989.3989.3	3	3.807	0.33	1	971.5	38.157894	K.DELHHSGWNTCSSCFGDSTK.S
SBP1_MOUSE	MK_SCX_51.5076.5076.3	3	5.145	0.357	1	2310.3	51.5625	K.RIPGGPQMIQLSLDGKR.L
SBP1_MOUSE	MK_SCX_51.6279.6279.3	3	5.239	0.491	1	883.9	30.769232	R.LPMPYLKDELHHSGWNTCSSCFGDSTK.S
SBP1_MOUSE	MK_SCX_52.3675.3675.3	3	3.604	0.362	1	1028	40.27778	K.CTKCGPGYSTPLEAMKGPR.E
SC22B_MOUSE	MK_SCX_18.4773.4773.2	2	5.009	0.653	1	1423.7	69.44444	R.VADGLPLAASMQEDEQSGR.D
SC22B_MOUSE	MK_SCX_21.4798.4798.2	2	4.897	0.508	1	2177.1	84.61539	R.NLGSINTELQDVQR.I
SC22B_MOUSE	MK_SCX_21.6604.6604.2	2	2.83	0.228	1	1103.9	77.27273	R.IMVANIEEVLQR.G
SC22B_MOUSE	MK_SCX_2201.4939.4939.3	3	3.904	0.536	1	350.8	27.67857	R.VADGLPLAASM*QEDEQSGRDLQQYQSQAK.Q
SC22B_MOUSE	MK_SCX_25.8562.8562.3	3	3.869	0.412	1	423.7	32.142857	R.IMVANIEEVLQRGEALSALDSK.A
SC23A_MOUSE	MK_SCX_30.3341.3341.3	3	3.836	0.454	1	469.4	40.789474	K.VPVTQATRGPQVQQPPPSNR.F
SC23B_MOUSE	MK_SCX_23.7357.7357.3	3	4.067	0.528	1	376.8	25	R.IMLFTGGPPTQGPGMVVGDELKTPIR.S
SC5A2_MOUSE	MK_SCX_20_1.3380.3380.2	2	3.108	0.418	1	317.8	55.88235	K.SGSGSPPPTTEEVAATTR.R
SC5A2_MOUSE	MK_SCX_32.3042.3042.3	3	5.296	0.448	1	2161.9	57.14286	-.MEQHVEAGSELGEQK.V
SCFD1_MOUSE	MK_SCX_16.5849.5849.2	2	2.618	0.297	1	488.8	53.125	K.SLLDVISDPDAGTPEDK.M
SCFD1_MOUSE	MK_SCX_50.7044.7044.3	3	4.665	0.424	1	1421.1	37.5	K.HKGSPFPEVAESVQQELESYR.A
SCOT_MOUSE	MK_SCX_15.11226.11226.2	2	6.102	0.52	1	3281	75	K.DLTAVSNNAGVDNFGLGLLLR.S
SCOT_MOUSE	MK_SCX_15.4927.4927.1	1	2.249	0.225	1	300.2	71.42857	R.NFNLPMCK.A
SCOT_MOUSE	MK_SCX_16.8792.8792.2	2	2.404	0.26	1	381.4	42.857143	K.ETVTVLPGASFFSSDESFAMIR.G
SCOT_MOUSE	MK_SCX_17.6209.6209.2	2	5.26	0.664	1	1629.4	50	R.AGGAGVPAFYTSTGYGTLVQEGGSPIK.Y
SCOT_MOUSE	MK_SCX_17.9739.9739.2	2	5.316	0.55	1	1128.2	63.15789	R.QFLSGELEVELTPQGTLAER.I
SCOT_MOUSE	MK_SCX_21.17110.17110.3	3	3.611	0.452	1	1082.2	53.846157	R.MISSYVGENAEFER.Q
SCOT_MOUSE	MK_SCX_21.4400.4400.2	2	5	0.426	1	1676.4	84.61539	R.M*ISSYVGENAEFER.Q
SCOT_MOUSE	MK_SCX_21.4602.4602.2	2	5.078	0.402	1	1909.4	88.46153	R.MISSYVGENAEFER.Q
SCOT_MOUSE	MK_SCX_21.5237.5237.2	2	4.306	0.374	1	1290.1	86.36364	K.YGDLANWM*IPGK.M
SCOT_MOUSE	MK_SCX_21.6042.6042.2	2	4.983	0.548	1	1091.6	90.909096	K.YGDLANWMIPGK.M
SCOT_MOUSE	MK_SCX_21.6069.6069.1	1	3.216	0.308	1	388.5	63.636364	K.YGDLANWMIPGK.M
SCOT_MOUSE	MK_SCX_2201.4017.4017.2	2	2.778	0.515	1	1054.1	75	K.GMGGAMDLVSSSK.T
SCOT_MOUSE	MK_SCX_23.10148.10148.2	2	2.565	0.341	1	348.6	40	R.EFNGQHFILEEAITGDFALVK.A
SCOT_MOUSE	MK_SCX_23.13315.13315.3	3	3.342	0.212	1	808.2	33.75	R.EFNGQHFILEEAITGDFALVK.A
SCOT_MOUSE	MK_SCX_25.6380.6380.2	2	4.973	0.67	1	468	36.206894	R.AGGAGVPAFYTSTGYGTLVQEGGSPIKYNK.D
SCOT_MOUSE	MK_SCX_25.8272.8272.2	2	6.224	0.629	1	2066.4	62.5	K.TGVKDLTAVSNNAGVDNFGLGLLLR.S
SCOT_MOUSE	MK_SCX_25.8479.8479.3	3	7.051	0.566	1	3370.4	45.833336	K.TGVKDLTAVSNNAGVDNFGLGLLLR.S
SCOT_MOUSE	MK_SCX_41.9982.9982.3	3	3.06	0.337	1	335.5	26.31579	K.KNGLTLIELWEGLTVDDIKK.S
SDF2_MOUSE	MK_SCX_32.5571.5571.3	3	3.944	0.355	1	798.6	51.923077	K.AM*EGIFMKPSELLR.A
SDF2_MOUSE	MK_SCX_32.6572.6572.2	2	4.004	0.398	1	889.3	65.38461	K.AMEGIFMKPSELLR.A
SDF2_MOUSE	MK_SCX_32.6593.6593.3	3	3.05	0.312	1	626.2	44.230766	K.AMEGIFMKPSELLR.A
SDF2_MOUSE	MK_SCX_37.3311.3311.3	3	3.08	0.191	1	309.1	33.92857	K.EVHGM*AQPSQNNYWK.A
SDF2_MOUSE	MK_SCX_37.3732.3732.3	3	3.031	0.413	1	694.1	42.857143	K.EVHGMAQPSQNNYWK.A
SDF2_MOUSE	MK_SCX_54.4997.4997.3	3	5.561	0.579	1	927.6	39.583336	R.FKHSSTDVLLSVTGEQYGRPISGQK.E
SDPR_MOUSE	MK_SCX_18.3763.3763.2	2	4.951	0.574	1	1506	61.764706	R.YESGYM*LNSEEM*EEPSEK.Q
SDPR_MOUSE	MK_SCX_18.4507.4507.2	2	5.577	0.592	1	2361.9	70.588234	R.YESGYMLNSEEMEEPSEK.Q
SEC63_MOUSE	MK_SCX_50.4853.4853.3	3	4.435	0.508	1	1342.1	45	K.SKITHPVYSLYFPEEK.Q
SEL1L_MOUSE	MK_SCX_18.6060.6060.2	2	4.869	0.653	1	1951.6	62.5	K.AADMGNPVGQSGLGMAYLYGR.G
SELB_MOUSE	MK_SCX_14.9866.9866.2	2	3.25	0.541	1	479.3	39.130436	R.TLFFSPAPDSFDLEPVLDSFDLSR.E
SELB_MOUSE	MK_SCX_16.3228.3228.2	2	3.715	0.418	1	1009.1	66.66667	K.DSMPTATEGDDEADPK.A
SELO_MOUSE	MK_SCX_16.7843.7843.2	2	3.589	0.434	1	376.9	37.5	R.SSIREFLCSEAM*FHLGIPTTR.A
SELO_MOUSE	MK_SCX_26.4287.4287.3	3	3.743	0.29	1	1058.2	38.157894	K.LLESPYHSEEEATGPEAVAR.S
SELS_MOUSE	MK_SCX_21.3430.3430.2	2	3.702	0.297	1	990.6	85	R.MQEDLNAQVEK.H

SELS_MOUSE	MK_SCX_31.3547.3547.3	3	4.001	0.41	1	1012.2	36.25	R.NSGRPQEEDGPGPSTSSVIPK.G
SEM3C_MOUSE	MK_SCX_17.6119.6119.2	2	3.061	0.303	1	348.2	41.17647	R.QDVRHGNPLTQCRGFNLK.A
SEM3C_MOUSE	MK_SCX_46.9156.9156.2	2	2.15	0.233	1	314.2	37.5	R.LTDNNRSTKQIHSMIAR.I
SEP11_MOUSE	MK_SCX_19.7833.7833.2	2	3.344	0.442	1	791.1	44.736843	K.STSQGFCFNILCVGETGIGK.S
SEP11_MOUSE	MK_SCX_20_1.3580.3580.3	3	4.099	0.411	1	1037.8	38.88889	K.AAAQLLQSQAQQSGAQQTK.K
SEP11_MOUSE	MK_SCX_20_1.3585.3585.2	2	3.218	0.415	1	340.7	66.66667	K.AAAQLLQSQAQQSGAQQTK.K
SEP11_MOUSE	MK_SCX_27.2940.2940.3	3	3.18	0.333	1	606.5	36.764706	R.EMLIRVNM*EDLREQTHTR.H
SEP11_MOUSE	MK_SCX_31.3251.3251.3	3	4.615	0.496	1	937.5	39.473686	K.AAAQLLQSQAQQSGAQQTKK.D
SEP11_MOUSE	MK_SCX_35.3295.3295.3	3	6.502	0.491	1	2395.7	50	K.KAAAQLLQSQAQQSGAQQTK.K
SEP11_MOUSE	MK_SCX_53.2978.2978.3	3	6.967	0.429	1	2247.3	47.5	K.KKAAAQLLQSQAQQSGAQQTK.K
SEP15_MOUSE	MK_SCX_16.7161.7161.2	2	3.853	0.348	1	2106.2	80	K.LLDDNGNIAEELSILK.W
SEP15_MOUSE	MK_SCX_21.6948.6948.2	2	4.136	0.5	1	1083.6	83.33333	K.WNTDSVEEFLSEK.L
SEP15_MOUSE	MK_SCX_41.5261.5261.2	2	3.217	0.449	1	915.5	72.72727	K.LGRFPQVQAFVR.S
SEPT2_MOUSE	MK_SCX_19.5925.5925.2	2	4.87	0.475	1	797.1	59.375	K.ASIPFSVVGSNQLIEAK.G
SEPT2_MOUSE	MK_SCX_21.7373.7373.2	2	3.786	0.427	1	1666.6	81.818184	K.TIISYIDEQFER.Y
SEPT2_MOUSE	MK_SCX_26.7028.7028.2	2	4.752	0.652	1	897.2	61.11111	R.LYPWGVVEVENPEHNDFLK.L
SEPT2_MOUSE	MK_SCX_32.4027.4027.2	2	3.912	0.344	1	1434.8	90	R.ILDEIEEHSIK.I
SEPT2_MOUSE	MK_SCX_43.6972.6972.3	3	5.594	0.463	1	1841.3	45	R.GRLYPWGVVEVENPEHNDFLK.L
SEPT2_MOUSE	MK_SCX_46.3240.3240.3	3	5.454	0.43	1	1028.3	48.333332	R.KVENEDM*NKDQILLEK.E
SEPT2_MOUSE	MK_SCX_46.3680.3680.3	3	5.514	0.408	1	1958.8	53.333336	R.KVENEDMNKDQILLEK.E
SEPT2_MOUSE	MK_SCX_46.3689.3689.2	2	5.818	0.398	1	1925	70	R.KVENEDMNKDQILLEK.E
SEPT6_MOUSE	MK_SCX_19.8025.8025.2	2	3.282	0.164	1	852.3	47.368423	K.SVSQGFCFNILCVGETGLGK.S
SEPT6_MOUSE	MK_SCX_50.3403.3403.3	3	6.705	0.52	1	2966.3	51.19048	R.KAAAELLQSQGSQAGGSQTLKR.D
SEPT7_MOUSE	MK_SCX_25.4915.4915.2	2	4.631	0.513	1	984.6	68.75	K.IYEFPETDDEEENKLVK.K
SEPT7_MOUSE	MK_SCX_36.3507.3507.3	3	3.038	0.232	1	742.9	40.384613	R.QFEEEKANWEAQQR.I
SEPT9_MOUSE	MK_SCX_23.3806.3806.2	2	3.834	0.355	1	1851	88.88889	K.YLQEEVNINR.K
SEPT9_MOUSE	MK_SCX_25.4327.4327.3	3	3.811	0.159	1	430.5	30.952381	R.VETPASKIPEGSAVPATDAAPK.R
SEPT9_MOUSE	MK_SCX_43.4088.4088.3	3	4.156	0.336	1	710.9	34.090908	R.RVETPASKIPEGSAVPATDAAPK.R
SEPT9_MOUSE	MK_SCX_51.4211.4211.3	3	3.708	0.422	1	1015.6	51.785713	R.RTEITIVKPQESVLR.R
SERB_MOUSE	MK_SCX_13.7621.7621.1	1	2.482	0.207	1	325.2	62.5	R.EEGIDELAK.F
SERB_MOUSE	MK_SCX_16.7283.7283.2	2	2.456	0.139	1	350.6	38.88889	R.LLAEHPPHLTPGIRELVSR.L
SET_MOUSE	MK_SCX_18.6682.6682.2	2	4.808	0.423	1	1364.2	76.92308	R.IDFYFDENPYFENK.V
SET_MOUSE	MK_SCX_20_1.4697.4697.2	2	4.184	0.505	1	1228.9	88.88889	R.VEVTEFEDIK.S
SET_MOUSE	MK_SCX_30.4251.4251.3	3	3.162	0.272	1	467	41.07143	R.LNEQASEEILKVEQK.Y
SET_MOUSE	MK_SCX_48.3566.3566.3	3	3.51	0.39	1	715.4	40.625	K.VLSKEFHLNESGDPSSK.S
SET_MOUSE	MK_SCX_58.8377.8377.2	2	2.321	0.375	1	332.4	68.75	K.LRQPFFQKR.S
SF01_MOUSE	MK_SCX_16.8945.8945.2	2	3.392	0.473	1	302.9	40	K.VM*IPQDEYPEINFVGLLIGPR.G
SF01_MOUSE	MK_SCX_18.5388.5388.2	2	2.743	0.334	1	436.8	56.666668	K.TVIPGM*PTVIPPGLTR.E
SF01_MOUSE	MK_SCX_18.6635.6635.2	2	3.051	0.494	1	502.8	56.666668	K.TVIPGMPTVIPPGLTR.E
SF01_MOUSE	MK_SCX_23.9058.9058.3	3	5.156	0.532	1	2927.9	41.666664	R.VSDKVM*IPQDEYPEINFVGLLIGPR.G
SF01_MOUSE	MK_SCX_31.3181.3181.3	3	3.416	0.35	1	756.5	46.153847	K.QGIETPEDQNDLRK.M
SF01_MOUSE	MK_SCX_45.3574.3574.2	2	2.209	0.287	1	710.6	75	R.ILRPWQSSETR.S
SF01_MOUSE	MK_SCX_48.6890.6890.3	3	5.212	0.489	1	1867	35	R.HTLITEMVALNPDFKPPADYKPPATR.V
SF3A1_MOUSE	MK_SCX_20_1.2861.2861.2	2	3.735	0.214	1	786.4	53.333336	K.VM*QQQQQATQQQLPQK.V
SF3A1_MOUSE	MK_SCX_20_1.2863.2863.3	3	4.564	0.425	1	1674	53.333336	K.VM*QQQQQATQQQLPQK.V
SF3A1_MOUSE	MK_SCX_25.5837.5837.3	3	4.602	0.391	1	480.5	31.818182	K.ASKPLPPAPAPDEYLVSPITGEK.I
SF3A1_MOUSE	MK_SCX_33.6086.6086.3	3	3.179	0.387	1	468.2	35.714287	K.FNFLNPNDPYHAYYR.H
SF3A2_MOUSE	MK_SCX_26.6533.6533.3	3	3.125	0.321	1	406.6	32.8125	R.QLALETIDINKDPYFMK.N
SF3A2_MOUSE	MK_SCX_29.4810.4810.3	3	4.36	0.478	1	1157.2	44.11765	K.MEKPPAPPSLPAGPPGVK.R
SF3A3_MOUSE	MK_SCX_17.6045.6045.2	2	5.06	0.521	1	1003.9	57.5	R.ENPSEEAQNLVEFTDEEGYGR.Y
SF3A3_MOUSE	MK_SCX_20_1.6113.6113.2	2	3.651	0.424	1	1885.1	86.36364	K.SLESLDTSLFAK.N

SF3A3_MOUSE	MK_SCX_28.4073.4073.2	2	4.946	0.567	1	749.7	65.789474	R.WQPDTEEEYEDSSGNVVNKK.T
SF3A3_MOUSE	MK_SCX_30.7918.7918.2	2	3.468	0.627	1	516.4	68.75	K.NLPLGWDGKPIPYWLYK.L
SF3B1_MOUSE	MK_SCX_20_1.3216.3216.2	2	3.736	0.183	1	584.8	69.230774	R.WDQTADQTPGATPK.K
SF3B1_MOUSE	MK_SCX_51.4029.4029.3	3	4.931	0.501	1	1337.3	45.588234	K.KLSSWDQAETPGHTPSLR.W
SFPQ_MOUSE	MK_SCX_17.9156.9156.2	2	4.95	0.599	1	1719.3	54.545456	R.NLSPYVSNELLEEAFSQFGPIER.A
SFPQ_MOUSE	MK_SCX_24.3209.3209.2	2	2.646	0.244	1	653.9	57.14286	R.FGQGGAGPVGGQGPR.G
SFPQ_MOUSE	MK_SCX_50.5634.5634.3	3	3.217	0.169	1	547.2	31.578945	K.DKLESEMEDAYHEHQANLLR.Q
SFRS1_MOUSE	MK_SCX_19.5183.5183.2	2	2.891	0.385	1	342.5	53.333336	R.VVVSGLPPSGSWQDLK.D
SFRS1_MOUSE	MK_SCX_2201.4650.4650.2	2	3.757	0.49	1	854.3	85	R.IYVGNLPPDIR.T
SFRS1_MOUSE	MK_SCX_2201.7770.7770.2	2	4.3	0.533	1	382.6	45.454548	R.GGPPFAFVEFEDPRDAEDAVYGR.D
SFRS1_MOUSE	MK_SCX_23.7554.7554.3	3	3.191	0.397	1	762.9	30.681818	R.GGPPFAFVEFEDPRDAEDAVYGR.D
SFRS1_MOUSE	MK_SCX_25.4965.4965.2	2	2.386	0.211	1	661	68.181816	R.GGGGGGGGGAPR.G
SFRS1_MOUSE	MK_SCX_37.4150.4150.2	2	3.42	0.365	1	1112.6	88.88889	R.TKDIEDVFYK.Y
SFRS1_MOUSE	MK_SCX_37.4160.4160.3	3	3.942	0.178	1	1875.3	69.44444	R.TKDIEDVFYK.Y
SFRS1_MOUSE	MK_SCX_37.6902.6902.3	3	3.593	0.382	1	1672.7	50	R.RGGPPFAFVEFEDPR.D
SFRS2_MOUSE	MK_SCX_15.3658.3658.2	2	5.24	0.561	1	2260.8	75	R.DAEDAM*DAM*DGAVLDGR.E
SFRS2_MOUSE	MK_SCX_15.4472.4472.2	2	5.299	0.252	1	2995.3	81.25	R.DAEDAMDAM*DGAVLDGR.E
SFRS2_MOUSE	MK_SCX_15.4740.4740.2	2	5.623	0.153	1	2489.6	75	R.DAEDAM*DAMDGAVLDGR.E
SFRS2_MOUSE	MK_SCX_15.5747.5747.2	2	5.721	0.544	1	3274.8	81.25	R.DAEDAMDAMDGAVLDGR.E
SFRS2_MOUSE	MK_SCX_2201.3694.3694.2	2	2.346	0.418	1	809.7	92.85714	R.VGDVYIPR.D
SFRS3_MOUSE	MK_SCX_20_1.6921.6921.2	2	3.379	0.461	1	1036.9	76.92308	R.NPPGFAFVEFEDPR.D
SFRS3_MOUSE	MK_SCX_24.7037.7037.2	2	3.797	0.432	1	506.4	52.499996	R.NPPGFAFVEFEDPRDAADAVR.E
SFRS3_MOUSE	MK_SCX_24.7070.7070.3	3	3.03	0.481	1	511.5	35	R.NPPGFAFVEFEDPRDAADAVR.E
SFRS3_MOUSE	MK_SCX_26.4466.4466.2	2	2.305	0.383	1	667	75	R.AFGYYGPLR.S
SFRS7_MOUSE	MK_SCX_25.4567.4567.2	2	2.318	0.297	1	668.6	87.5	R.AFSYYGPLR.T
SFRS7_MOUSE	MK_SCX_34.3773.3773.2	2	4.45	0.493	1	1490.5	71.875	K.VYVGNLGTGAGKGELER.A
SFXN1_MOUSE	MK_SCX_14.10502.10502.3	3	6.54	0.622	1	1633	32.575756	R.SGDAPLTVNELGTAYVSATTGAVATALGLNALTK.R
SFXN1_MOUSE	MK_SCX_14.10940.10940.2	2	5.348	0.644	1	1103.6	37.878788	R.SGDAPLTVNELGTAYVSATTGAVATALGLNALTK.R
SFXN1_MOUSE	MK_SCX_17.9002.9002.2	2	3.415	0.496	1	473.7	52.63158	R.ILMAAPGMAIPPFIMNTLEK.K
SFXN1_MOUSE	MK_SCX_2201.4383.4383.2	2	5.136	0.544	1	2306	87.5	R.NILLTNEQLENAR.K
SFXN1_MOUSE	MK_SCX_2201.4409.4409.3	3	3.795	0.386	1	1327.1	56.25	R.NILLTNEQLENAR.K
SFXN1_MOUSE	MK_SCX_31.3710.3710.2	2	4.633	0.583	1	1683.5	80.769226	K.YAYDSAFHPDTGEK.M
SFXN1_MOUSE	MK_SCX_49.3647.3647.2	2	5.121	0.599	1	2449.8	83.33333	R.AKYAYDSAFHPDTGEK.M
SFXN1_MOUSE	MK_SCX_49.3650.3650.3	3	3.846	0.455	1	707.3	46.666668	R.AKYAYDSAFHPDTGEK.M
SGTA_MOUSE	MK_SCX_13.3791.3791.2	2	3.34	0.498	1	890.4	75	K.LGNYVGAVQDCER.A
SGTA_MOUSE	MK_SCX_31.2587.2587.3	3	5.808	0.513	1	2850.6	56.25	R.APDRTPPSEEDSAEAER.L
SGTA_MOUSE	MK_SCX_31.2611.2611.2	2	3.658	0.493	1	735.4	56.25	R.APDRTPPSEEDSAEAER.L
SH3G2_MOUSE	MK_SCX_42.4309.4309.3	3	3.91	0.542	1	2028.4	47.058823	K.VGGAEGTKLDDDFKEMER.K
SH3L1_MOUSE	MK_SCX_48.3600.3600.3	3	4.575	0.397	1	1671.3	50	K.IGFEEKDIAANEENRK.W
SH3L3_MOUSE	MK_SCX_26.5378.5378.3	3	3.121	0.24	1	336.8	34.72222	R.IQYQLVDISQDNALRDEM*R.T
SH3L3_MOUSE	MK_SCX_26.5905.5905.3	3	3.255	0.308	1	433	36.11111	R.IQYQLVDISQDNALRDEMR.T
SH3L3_MOUSE	MK_SCX_26.6063.6063.2	2	3.62	0.552	1	670.2	50	R.IQYQLVDISQDNALRDEMR.T
SH3L3_MOUSE	MK_SCX_46.6024.6024.3	3	3.795	0.455	1	569.1	32.894737	K.RIQYQLVDISQDNALRDEMR.T
SHLB1_MOUSE	MK_SCX_37.3741.3741.2	2	3.293	0.2	1	830.7	66.66667	K.AECTKIWTEKIM*K.Q
SI1L1_MOUSE	MK_SCX_23.8894.8894.3	3	6.297	0.589	1	2420.2	39.285713	R.RQPIPDPGLMPLPDAASDLDWSNLVDAAK.A
SIAT6_MOUSE	MK_SCX_19.7053.7053.2	2	3.22	0.184	1	667.8	50	R.EFVPPFGIKGQDNLIK.A
SIGIR_MOUSE	MK_SCX_17.4633.4633.2	2	5.382	0.593	1	1191.1	75	R.GM*ESELDPDPEGDLGVR.G
SIGIR_MOUSE	MK_SCX_17.5055.5055.2	2	4.96	0.459	1	905.9	65.625	R.GMESELDPDPEGDLGVR.G
SIGIR_MOUSE	MK_SCX_19.4567.4567.2	2	4.642	0.448	1	748.7	75	R.GPVFGEPPTPLQETR.I
SIGIR_MOUSE	MK_SCX_31.4103.4103.3	3	3.641	0.198	1	987.3	54.545456	R.LQDDKDPMLIVR.G
SKP1_MOUSE	MK_SCX_14.8136.8136.2	2	4.998	0.679	1	909.4	48.148148	K.TMLEDLGMDDEGDDDPVPLPNVNAAILK.K

SKP1_MOUSE	MK_SCX_20_1.7900.7900.3	3	5.32	0.139	1	1728.7	35.714287	K.TM*LEDLGMDDEGDDDPVPLPNVNAAILKK.V
SKP1_MOUSE	MK_SCX_21.3829.3829.2	2	4.299	0.494	1	1820.9	77.27273	K.NDFTEEEEAQVR.K
SKP1_MOUSE	MK_SCX_31.16693.16693.3	3	3.91	0.453	1	484.2	37.5	K.TFNIKNDFTEEEEAQVR.K
SKP1_MOUSE	MK_SCX_33.6444.6444.2	2	2.849	0.284	1	1005.8	73.07692	K.RTDDIPVWDQEFLK.V
SLK_MOUSE	MK_SCX_15.6023.6023.2	2	5.175	0.539	1	1128.1	47.916664	R.ATLEQPETDEVEQVSESNSIEELER.L
SLK_MOUSE	MK_SCX_19.4377.4377.3	3	6.519	0.592	1	1001.5	26.973686	K.AAQSGEGDEALAPTQTLAEKPTEGPEAGGAEEEPPGGER.V
SLK_MOUSE	MK_SCX_2201.3520.3520.2	2	2.09	0.322	1	515.6	81.25	K.LADFGVSAK.N
SLK_MOUSE	MK_SCX_47.3306.3306.3	3	4.979	0.367	1	1387.6	47.058823	R.KEELAQSQHAQEQEFVQK.Q
SMC1A_MOUSE	MK_SCX_20_1.15895.15895.2	2	2.877	0.161	1	608.4	53.333336	K.SGVISGGASDLKAKAR.R
SMC1A_MOUSE	MK_SCX_30.3789.3789.3	3	3.669	0.406	1	1001.5	53.846157	R.DKFQETSDEFEAAR.K
SMC1A_MOUSE	MK_SCX_46.7704.7704.3	3	3.198	0.379	1	457.2	30.263159	R.RIDEINKELNQVMEQLGDAR.I
SMCE1_MOUSE	MK_SCX_21.4080.4080.2	2	3.305	0.298	1	546.6	66.66667	K.IAAEIAQAEEQAR.K
SMD1_MOUSE	MK_SCX_15.10152.10152.2	2	4.6	0.61	1	852.2	60.526318	R.YFILPDSLPLDTLLVDVEPK.V
SMD1_MOUSE	MK_SCX_15.9906.9906.3	3	5.533	0.632	1	1442.6	42.105263	R.YFILPDSLPLDTLLVDVEPK.V
SMD1_MOUSE	MK_SCX_39.5171.5171.3	3	4.443	0.49	1	1724.2	54.166668	K.NREPVQLETLSIR.G
SMD1_MOUSE	MK_SCX_39.5214.5214.2	2	3.995	0.531	1	646.1	75	K.NREPVQLETLSIR.G
SMD2_MOUSE	MK_SCX_31.6874.6874.3	3	4.559	0.393	1	1186.4	37.5	K.REEEEFNTGPLSVLTQSVK.N
SMD2_MOUSE	MK_SCX_31.6914.6914.2	2	5.587	0.643	1	2353.4	72.22222	K.REEEEFNTGPLSVLTQSVK.N
SMD2_MOUSE	MK_SCX_33.8237.8237.3	3	5.317	0.387	1	1184.3	33.035713	K.SEMTPEELQKREEEEFNTGPLSVLTQSVK.N
SMD2_MOUSE	MK_SCX_8.3901.3901.2	2	2.783	0.32	1	1171.5	88.88889	K.NNTQVLINCR.N
SMD3_MOUSE	MK_SCX_21.5843.5843.2	2	2.488	0.195	1	1099.3	87.5	R.FLILPDM*LK.N
SMD3_MOUSE	MK_SCX_21.7347.7347.1	1	2.535	0.205	1	843.7	75	R.FLILPDMLK.N
SMG7_MOUSE	MK_SCX_12.11629.11629.2	2	2.082	0.123	1	398.7	46.875	R.SIAVKFPFPAASTNLQK.A
SMG7_MOUSE	MK_SCX_2201.4449.4449.2	2	2.171	0.132	1	488.4	59.090908	K.FPFPAASTNLQK.A
SMRC2_MOUSE	MK_SCX_27.2764.2764.3	3	3.871	0.337	1	694.5	41.666664	R.IEESGTEEARPEGQAADEK.K
SMRC2_MOUSE	MK_SCX_49.4571.4571.3	3	4.961	0.464	1	1762.5	57.14286	K.MKEEVPTALVEAHVR.K
SNAA_MOUSE	MK_SCX_21.8019.8019.2	2	3.299	0.489	1	1512.1	90	R.LDQWLTTMLLR.I
SNAA_MOUSE	MK_SCX_2201.4136.4136.2	2	4.685	0.467	1	2223.5	79.16667	K.VAGYAAQLEQYQK.A
SNAA_MOUSE	MK_SCX_23.6558.6558.2	2	4.53	0.566	1	1987.2	78.57143	K.NSQSFFSGLFGGSSK.I
SNAG_MOUSE	MK_SCX_16.6104.6104.2	2	2.757	0.275	1	615	52.63158	K.ASMMYLENGTPDTAAMALER.A
SNAG_MOUSE	MK_SCX_21.6066.6066.2	2	3.267	0.251	1	857.9	62.5	K.LGLSLVVPGGGIK.K
SNAG_MOUSE	MK_SCX_2201.8511.8511.3	3	5.462	0.516	1	2006.4	39	K.LIENVDPEKAVQLYQQTANVFENEER.L
SNAG_MOUSE	MK_SCX_30.4732.4732.3	3	3.401	0.395	1	931.3	44.230766	K.EM*QKLPEAVQLIEK.A
SNAG_MOUSE	MK_SCX_30.5213.5213.2	2	3.599	0.292	1	892.3	73.07692	K.EMQKLPEAVQLIEK.A
SND1_MOUSE	MK_SCX_17.7052.7052.2	2	4.885	0.484	1	486.9	47.5	R.NLPGLVQEGEPFSEEATLFTK.E
SND1_MOUSE	MK_SCX_18.3813.3813.2	2	4.909	0.524	1	982.9	73.333336	R.ETDGSETPEPFAAEAK.F
SND1_MOUSE	MK_SCX_21.4246.4246.2	2	4.361	0.406	1	1758.8	79.16667	K.VITEYLNAQESAK.S
SND1_MOUSE	MK_SCX_31.3478.3478.3	3	3.428	0.406	1	1151.4	48.333332	R.NDISSHPPVEGSYAPR.R
SND1_MOUSE	MK_SCX_33.4364.4364.2	2	4.197	0.534	1	629.8	71.42857	R.SAYYKPLLSAEEAAK.Q
SND1_MOUSE	MK_SCX_45.4615.4615.3	3	5.187	0.468	1	1061.7	34.523808	R.RAAATQPDGKDTPDEPWAFPAR.E
SNIP_MOUSE	MK_SCX_16.4106.4106.2	2	3.098	0.273	1	430.8	29.62963	K.SSGATPVSGPPPPSASSTPAGQPTAVSR.L
SNIP_MOUSE	MK_SCX_49.3272.3272.3	3	3.408	0.449	1	537.5	39.285713	K.KAESEELEVQKPQVK.L
SNP23_MOUSE	MK_SCX_17.6620.6620.2	2	3.765	0.537	1	760.8	52.77778	R.EDEM*EENLTQVGSILGNLK.N
SNP23_MOUSE	MK_SCX_17.7825.7825.2	2	4.892	0.536	1	1261.1	66.66667	R.EDEMEENLTQVGSILGNLK.N
SNP23_MOUSE	MK_SCX_18.3578.3578.2	2	3.756	0.432	1	722.7	58.333332	R.ITNGQPQQTTGAASGGYIK.R
SNP23_MOUSE	MK_SCX_18.3779.3779.2	2	2.277	0.281	1	379.5	50	K.ATWGDGGDNSPSNVVSK.Q
SNP23_MOUSE	MK_SCX_18.3808.3808.2	2	6.691	0.651	1	2731.8	80.55556	K.NM*ALDM*GNEIDAQNQQIQK.I
SNP23_MOUSE	MK_SCX_18.4166.4166.2	2	4.888	0.143	1	1213.5	63.88889	K.NMALDM*GNEIDAQNQQIQK.I
SNP23_MOUSE	MK_SCX_18.4444.4444.2	2	6.657	0.274	1	2422.1	80.55556	K.NM*ALDMGNEIDAQNQQIQK.I
SNP23_MOUSE	MK_SCX_18.4938.4938.2	2	6.223	0.625	1	2338.9	75	K.NMALDMGNEIDAQNQQIQK.I
SNP23_MOUSE	MK_SCX_20_1.3931.3931.2	2	4.696	0.495	1	1266.7	80.769226	K.TITM*LDEQGEQLNR.I

SNP23_MOUSE	MK_SCX_20_1.5913.5913.2	2	4.81	0.584	1	1759.3	80.769226	R.ILGLAIESQDAGIK.T
SNP23_MOUSE	MK_SCX_24.9284.9284.3	3	4.193	0.459	1	1749.9	38.541664	R.ITNDAREDEMEENLTQVGSILGNLK.N
SNP29_MOUSE	MK_SCX_2201.3921.3921.2	2	3.118	0.489	1	1975.4	95	K.IGVASSEELVR.Q
SNP29_MOUSE	MK_SCX_23.4892.4892.3	3	5.778	0.486	1	1301.6	39.130436	R.LQDAELDSVPKEPSSTVNTEVYPK.N
SNP29_MOUSE	MK_SCX_24.7690.7690.2	2	2.678	0.407	1	489	65	K.SVFGGFINYFK.S
SNP29_MOUSE	MK_SCX_50.3479.3479.2	2	2.784	0.236	1	333.6	66.66667	K.MDQDLKM*SQK.H
SNRPA_MOUSE	MK_SCX_26.5897.5897.3	3	6.07	0.563	1	2721.1	40.17857	K.KAVQGGAAAPVVGAVQPVPGMPPMPQAPR.I
SNRPA_MOUSE	MK_SCX_36.5176.5176.2	2	3.215	0.422	1	645	66.66667	R.SMQGFPFYDKPMR.I
SNW1_MOUSE	MK_SCX_21.7919.7919.3	3	6.339	0.63	1	1393.6	36.458336	R.LLEDFGDGGAFPEIHVAQYPLDM*GR.K
SNW1_MOUSE	MK_SCX_21.8347.8347.3	3	5.944	0.578	1	1835.1	41.666664	R.LLEDFGDGGAFPEIHVAQYPLDMGR.K
SNW1_MOUSE	MK_SCX_31.3899.3899.3	3	4.03	0.455	1	1399.1	57.692307	K.NLDKDMYGDDLEAR.I
SNX1_MOUSE	MK_SCX_20_1.4744.4744.2	2	3.412	0.364	1	549	70	K.SLAMLGSSEDNTALSR.A
SNX1_MOUSE	MK_SCX_21.4699.4699.2	2	2.146	0.221	1	331.2	57.692307	R.AVGTQALSGAGLLK.M
SNX1_MOUSE	MK_SCX_2201.4169.4169.2	2	3.287	0.24	1	702.8	72.72727	K.IGDGMNAYVAYK.V
SNX1_MOUSE	MK_SCX_2201.4931.4931.2	2	4.004	0.423	1	691.5	85	K.MNESDIWFEEK.L
SNX1_MOUSE	MK_SCX_2201.5905.5905.2	2	2.728	0.286	1	661.9	72.22222	R.FSDFLGLYEK.L
SNX1_MOUSE	MK_SCX_23.5700.5700.2	2	3.769	0.497	1	1254.4	83.33333	K.YWEAFLPEAK.A
SNX1_MOUSE	MK_SCX_31.3094.3094.2	2	2.085	0.276	1	355	54.545456	K.SLIGMTKVKVGK.E
SNX1_MOUSE	MK_SCX_32.3741.3741.2	2	3.27	0.284	1	731	61.538464	R.IVNHPTMLQDPDVR.E
SNX1_MOUSE	MK_SCX_33.3478.3478.3	3	3.407	0.264	1	507.5	44.230766	R.IVNHPTM*LQDPDVR.E
SNX1_MOUSE	MK_SCX_33.3738.3738.3	3	3.055	0.367	1	456.3	48.076923	R.IVNHPTMLQDPDVR.E
SNX1_MOUSE	MK_SCX_33.4733.4733.2	2	4.898	0.522	1	1787.9	84.61539	K.YLETLLHSQQQLAK.Y
SNX1_MOUSE	MK_SCX_41.5806.5806.2	2	2.14	0.275	1	472.8	65	R.RFSDFLGLYEK.L
SNX1_MOUSE	MK_SCX_52.6397.6397.3	3	4.377	0.528	1	1504.7	38.636364	R.LLWANKPDKLQQAKDEITEWESR.V
SNX12_MOUSE	MK_SCX_21.9120.9120.3	3	4.346	0.547	1	932	25.78125	R.LNSKPQDLTDAYGPPSNFLEIDIFNPQTVGVGR.A
SNX12_MOUSE	MK_SCX_44.4459.4459.2	2	2.086	0.179	1	523.9	72.22222	R.RQGLEQFINK.I
SNX18_MOUSE	MK_SCX_20_1.3218.3218.2	2	3.589	0.428	1	703.9	68.75	R.APEPGPPADGGPGAPAR.Y
SNX18_MOUSE	MK_SCX_20_1.3243.3243.3	3	4.358	0.441	1	1297.6	46.875	R.APEPGPPADGGPGAPAR.Y
SNX18_MOUSE	MK_SCX_47.4726.4726.3	3	4.596	0.459	1	725.3	38.235294	K.KMDDSALQLNHTANEFAR.K
SNX2_MOUSE	MK_SCX_15.6930.6930.2	2	6.034	0.609	1	1721.4	63.04348	R.ELILSSEPSPAVTPVTPTTLIAPR.I
SNX2_MOUSE	MK_SCX_15.6990.6990.3	3	5.058	0.616	1	2366.5	44.56522	R.ELILSSEPSPAVTPVTPTTLIAPR.I
SNX2_MOUSE	MK_SCX_2201.4167.4167.2	2	3.551	0.469	1	657.5	72.72727	K.QQQFENLDQQLR.K
SNX2_MOUSE	MK_SCX_2201.4478.4478.2	2	3.87	0.354	1	2380.4	80.769226	R.AVNTQALSGAGILR.M
SNX2_MOUSE	MK_SCX_23.4219.4219.2	2	3.863	0.608	1	1465.4	81.818184	K.VGDGMNAYMAYR.V
SNX2_MOUSE	MK_SCX_33.4812.4812.2	2	4.691	0.573	1	1944.8	88.46153	K.YLHVGYIVPPAPEK.S
SNX2_MOUSE	MK_SCX_38.3941.3941.2	2	2.687	0.333	1	538.7	70	K.HPTLLQDPDLR.Q
SNX2_MOUSE	MK_SCX_50.3099.3099.3	3	3.183	0.447	1	342.7	38.333332	K.VKVGKEDSSSTEFVEK.R
SNX2_MOUSE	MK_SCX_51.3704.3704.2	2	2.588	0.397	1	850.7	65.38461	R.TVKHPTLLQDPDLR.Q
SNX2_MOUSE	MK_SCX_51.3754.3754.3	3	4.913	0.454	1	997.2	51.923077	R.TVKHPTLLQDPDLR.Q
SNX3_MOUSE	MK_SCX_2201.7555.7555.3	3	5.95	0.515	1	1969.2	32.8125	R.LITKPQNLNDAYGPPSNFLEIDVSNPQTVGVGR.G
SNX3_MOUSE	MK_SCX_25.5440.5440.2	2	3.399	0.281	1	956.8	92.85714	R.YSDFEWLR.S
SNX3_MOUSE	MK_SCX_48.4968.4968.2	2	3.028	0.408	1	763.2	81.25	R.RYSDFEWLR.S
SNX4_MOUSE	MK_SCX_19.4138.4138.2	2	3.809	0.329	1	1297.5	73.07692	K.VLEEQINEGEQQLK.S
SNX4_MOUSE	MK_SCX_23.6004.6004.2	2	3.277	0.358	1	1647.9	93.75	R.VGLENFLLR.V
SNX4_MOUSE	MK_SCX_31.5334.5334.3	3	3.005	0.309	1	595.3	31.25	R.SVEHADGQSVLTDSLWR.R
SNX4_MOUSE	MK_SCX_32.4947.4947.2	2	4.576	0.587	1	1793.1	73.333336	R.IKVLEEQINEGEQQLK.S
SNX4_MOUSE	MK_SCX_45.5915.5915.2	2	3.179	0.214	1	650.3	88.88889	R.RVGLENFLLR.V
SNX4_MOUSE	MK_SCX_51.8298.8298.3	3	3.973	0.475	1	961.4	46.42857	R.HYSDELQSVISHLLR.V
SNX4_MOUSE	MK_SCX_51.8531.8531.2	2	4.158	0.598	1	1364.4	82.14286	R.HYSDELQSVISHLLR.V
SNX5_MOUSE	MK_SCX_20_1.5696.5696.2	2	4.12	0.488	1	817.1	67.85714	K.TTLSTFQSPEFSVTR.Q
SNX5_MOUSE	MK_SCX_23.4651.4651.2	2	3.262	0.505	1	1028.8	88.88889	R.YYMLNIEAAK.D

SNX5_MOUSE	MK_SCX_24.10831.10831.3	3	3.434	0.418	1	1035	36.25	K.SADEVLFSGVKEVDDFFEQEK.N
SNX5_MOUSE	MK_SCX_26.4579.4579.2	2	2.084	0.157	1	866.9	75	K.NFLINYYNR.I
SNX5_MOUSE	MK_SCX_26.8197.8197.2	2	3.654	0.45	1	829.9	52.77778	K.EVDDFFEQEKNFLINYYNR.I
SNX5_MOUSE	MK_SCX_46.5417.5417.3	3	3.68	0.398	1	685.7	35.9375	K.FEQLSESAKEELINFKR.K
SNX5_MOUSE	MK_SCX_49.5953.5953.3	3	3.501	0.17	1	1023.1	46.42857	K.MKQELEAEYLAVFKK.T
SNX6_MOUSE	MK_SCX_13.5323.5323.2	2	3.616	0.475	1	563.4	55.88235	R.IGSSLYALGTQDSTDICK.F
SNX6_MOUSE	MK_SCX_21.7554.7554.2	2	2.906	0.356	1	1073.1	75	K.NLVELAELELK.H
SNX6_MOUSE	MK_SCX_2201.1925.1925.2	2	2.433	0.243	1	698.5	85.71429	R.VSADEDLK.L
SNX6_MOUSE	MK_SCX_31.3424.3424.2	2	4.758	0.5	1	1515	71.42857	K.LGEGEGSMTKEEFTK.M
SNX6_MOUSE	MK_SCX_31.3460.3460.3	3	3.518	0.383	1	480.6	42.857143	K.LGEGEGSMTKEEFTK.M
SNX6_MOUSE	MK_SCX_35.4081.4081.3	3	3.775	0.457	1	575.6	40.625	K.NKDVLQAETSQQLCCQK.F
SNX6_MOUSE	MK_SCX_35.6601.6601.3	3	3.718	0.346	1	468.7	39.285713	K.SSLPNFKQNEFSVVR.Q
SNX6_MOUSE	MK_SCX_36.10409.10409.3	3	3.04	0.296	1	526.4	32.5	K.SADGVIVSGVKDVDDFFEHER.T
SNX6_MOUSE	MK_SCX_37.7182.7182.2	2	2.091	0.179	1	304.5	59.090908	R.KNLVELAELELK.H
SNX6_MOUSE	MK_SCX_52.3447.3447.3	3	4.634	0.461	1	1934.2	44.444447	R.AKNKDVLQAETSQQLCCQK.F
SNX7_MOUSE	MK_SCX_26.5653.5653.3	3	3.253	0.151	1	999.1	45	K.LEEAHPTLIIPPLPEK.F
SNX7_MOUSE	MK_SCX_31.5067.5067.3	3	3.474	0.428	1	348	38.46154	R.IADHPTLTFNEDFK.V
SNX7_MOUSE	MK_SCX_41.5488.5488.3	3	4.601	0.489	1	1293.3	47.058823	K.GKLEEAHPTLIIPPLPEK.F
SNX9_MOUSE	MK_SCX_30.3832.3832.3	3	4.677	0.534	1	1151.2	37.5	K.SSSPYFKDSEPAEAGGIQR.G
SNX9_MOUSE	MK_SCX_41.6846.6846.3	3	3.412	0.31	1	1064.5	39.705883	K.AMDDGVKELLTVGQEHWK.R
SO1A1_MOUSE	MK_SCX_16.8687.8687.2	2	3.06	0.507	1	478.3	47.368423	R.GIGETPIMPLGISYIEDFAK.S
SO1A1_MOUSE	MK_SCX_50.3515.3515.3	3	3.976	0.349	1	1291.5	51.923077	K.KELQDNVDVTKYEK.V
SO1A6_MOUSE	MK_SCX_23.6046.6046.2	2	2.117	0.169	1	556.9	72.22222	R.LYLGLPAALR.G
SO1A6_MOUSE	MK_SCX_23.8403.8403.3	3	3.447	0.212	1	1179.9	35.714287	K.LQIPGDTDSSEIELAETKPTEK.E
SODC_MOUSE	MK_SCX_19.3475.3475.2	2	4.059	0.488	1	1473.4	95	K.DGVANVSIEDR.V
SODC_MOUSE	MK_SCX_28.5448.5448.3	3	5.482	0.576	1	1633.6	46.05263	K.AVCVLKGDGPVQGTIHFEQK.A
SODC_MOUSE	MK_SCX_34.4111.4111.2	2	3.846	0.475	1	850.1	70.83333	R.VISLSGEHSIIGR.T
SODC_MOUSE	MK_SCX_42.4631.4631.3	3	6.564	0.675	1	2405	48.863636	R.HVGDLGNVTAGKDGVANVSIEDR.V
SODC_MOUSE	MK_SCX_43.4791.4791.2	2	6.751	0.675	1	2060.9	65.909096	R.HVGDLGNVTAGKDGVANVSIEDR.V
SODE_MOUSE	MK_SCX_17.6746.6746.2	2	3.608	0.618	1	322.1	47.5	R.VQPSATLPPDQPQITGLVLFR.Q
SODE_MOUSE	MK_SCX_31.5483.5483.3	3	3.16	0.462	1	794.8	43.333332	R.VGLTASLAGPHAILGR.S
SODM_MOUSE	MK_SCX_19.4179.4179.2	2	4.454	0.348	1	1318.8	76.92308	K.GDVTTQVALQPALK.F
SODM_MOUSE	MK_SCX_2201.7854.7854.2	2	4.556	0.472	1	1717.9	76.92308	K.AIWNVINWENVTER.Y
SODM_MOUSE	MK_SCX_2201.7871.7871.3	3	3.234	0.21	1	557.4	40.384613	K.AIWNVINWENVTER.Y
SODM_MOUSE	MK_SCX_43.4893.4893.3	3	4.274	0.525	1	1084.3	42.5	K.YHEALAKGDVTTQVALQPALK.F
SODM_MOUSE	MK_SCX_45.5999.5999.2	2	3.437	0.3	1	572.2	52.77778	K.FNGGGHINHTIFWTNLSPK.G
SODM_MOUSE	MK_SCX_45.6039.6039.3	3	3.235	0.263	1	1091	40.27778	K.FNGGGHINHTIFWTNLSPK.G
SODM_MOUSE	MK_SCX_51.3719.3719.3	3	4.634	0.339	1	1439.3	51.785713	K.GGGEPKGELLEAIKR.D
SODM_MOUSE	MK_SCX_51.3727.3727.2	2	4.442	0.47	1	1143.9	75	K.GGGEPKGELLEAIKR.D
SODM_MOUSE	MK_SCX_55.2842.2842.3	3	3.707	0.243	1	866.7	44.642857	K.HHAAYVNNLNATEEK.Y
SODM_MOUSE	MK_SCX_57.9156.9156.3	3	5.077	0.478	1	841.3	39.285713	K.HHAAYVNNLNATEEKYHEALAK.G
SOX_MOUSE	MK_SCX_2201.5231.5231.2	2	2.264	0.221	1	601.3	75	K.LPPSYDLAPFR.M
SOX_MOUSE	MK_SCX_31.8711.8711.2	2	2.541	0.352	1	545.3	53.846157	K.SVLLLEQFFLPHSR.G
SOX_MOUSE	MK_SCX_31.8763.8763.3	3	3.926	0.45	1	781.8	44.230766	K.SVLLLEQFFLPHSR.G
SOX_MOUSE	MK_SCX_40.3738.3738.3	3	3.696	0.282	1	1328.6	62.5	R.FTRGEVGLLDK.T
SOX5_MOUSE	MK_SCX_16.10993.10993.2	2	2.242	0.311	1	407.6	53.846157	K.DEVAQPLNLSAKPK.T
SOX5_MOUSE	MK_SCX_19.5976.5976.2	2	2.101	0.275	1	359.7	42.857143	K.NEPEDTPSIEKLLSK.D
SP1_MOUSE	MK_SCX_17.4346.4346.3	3	4.214	0.511	1	354	25	R.VGGLQGSDSLNIQQNQTSGGSLQGSQQK.E
SP17_MOUSE	MK_SCX_17.9827.9827.2	2	2.759	0.371	1	371.8	42.105263	R.EQPDNIPAFAAAYFENLLEK.R
SP17_MOUSE	MK_SCX_21.7884.7884.2	2	3.874	0.609	1	946.7	76.92308	R.IPQGFGNLLEGLTR.E
SPA3C_MOUSE	MK_SCX_20_1.4148.4148.2	2	4.629	0.478	1	1449.7	80.769226	R.M*QQVEASLQPETLR.K

SPA3C_MOUSE	MK_SCX_32.3809.3809.3	3	3.918	0.401	1	1001.8	46.42857	R.MQQVEASLQPETLRK.W
SPA3M_MOUSE	MK_SCX_17.10665.10665.2	2	4.763	0.567	1	1507.8	62.5	K.NIVFSPLSISAALALVSLGAK.G
SPAG1_MOUSE	MK_SCX_20_1.8792.8792.2	2	2.005	0.181	1	327.5	36.666668	K.ASHRLALAQKGLENCR.E
SPAG1_MOUSE	MK_SCX_21.5358.5358.2	2	2.147	0.183	1	744.8	72.22222	R.FKTM*LTLINK.G
SPCS1_MOUSE	MK_SCX_06.8265.8265.2	2	2.493	0.155	1	426.1	46.42857	-.M*LEHLSSLPTQM*DYK.G
SPCS1_MOUSE	MK_SCX_44.4816.4816.3	3	4.745	0.369	1	1566.9	47.058823	-.MLEHLSSLPTQMDYKGQK.L
SPCS2_MOUSE	MK_SCX_27.6535.6535.2	2	4.545	0.534	1	1080.7	58.333332	K.FFDHSGTLVMDAYEPEISR.L
SPF45_MOUSE	MK_SCX_28.3177.3177.2	2	3.208	0.497	1	434.2	50	K.IIVGDATEKGEAQDASK.K
SPFH1_MOUSE	MK_SCX_17.5585.5585.2	2	2.944	0.125	1	763.6	64.28571	K.DLNTMAPGLTIQAVR.V
SPFH1_MOUSE	MK_SCX_20_1.5484.5484.2	2	4.659	0.52	1	2266.6	90.909096	R.ISEIEDAAFLAR.E
SPFH2_MOUSE	MK_SCX_17.5526.5526.2	2	4.389	0.536	1	1517.8	65.625	K.LGFGLEDEPLEAPTK.E
SPFH2_MOUSE	MK_SCX_17.6017.6017.2	2	4.028	0.668	1	655.6	82.14286	K.DIPNMFMDSAGGLGK.Q
SPFH2_MOUSE	MK_SCX_17.7794.7794.2	2	5.901	0.577	1	1469.7	68.42105	K.LALQQDLTSMAPGLVIQAVR.V
SPFH2_MOUSE	MK_SCX_21.3932.3932.2	2	3.215	0.429	1	1079	81.818184	K.VAQVAEITYGQK.V
SPFH2_MOUSE	MK_SCX_2201.7142.7142.3	3	4.841	0.467	1	894.4	29	K.QFEGLSDDKLGFGLEDEPLEAPTK.E
SPFH2_MOUSE	MK_SCX_28.6904.6904.3	3	3.328	0.247	1	1111.4	38.157894	K.IYFGKDIPNMFM*DSAGGLGK.Q
SPFH2_MOUSE	MK_SCX_28.7515.7515.2	2	5.422	0.51	1	1374	68.42105	K.IYFGKDIPNMFMDSAGGLGK.Q
SPFH2_MOUSE	MK_SCX_28.7598.7598.3	3	3.785	0.331	1	1552.3	42.105263	K.IYFGKDIPNMFMDSAGGLGK.Q
SPFH2_MOUSE	MK_SCX_36.5075.5075.2	2	4.336	0.515	1	2146.4	91.66667	K.KISEIEDAAFLAR.E
SPFH2_MOUSE	MK_SCX_38.3673.3673.3	3	3.066	0.366	1	704.6	50	R.VTKPNIPEAIR.R
SPP24_MOUSE	MK_SCX_23.5140.5140.2	2	3.319	0.508	1	825.4	72.72727	K.VNSQSLSPYLFR.A
SPRE_MOUSE	MK_SCX_12.7338.7338.2	2	2.721	0.373	1	700.2	58.823532	-.MEADGLGCAVCVLTGASR.G
SPRE_MOUSE	MK_SCX_17.5760.5760.2	2	4.412	0.586	1	2252.6	82.14286	R.DM*LYQVLAAEEPSVR.V
SPRE_MOUSE	MK_SCX_17.6459.6459.2	2	4.194	0.545	1	1639.3	78.57143	R.DMLYQVLAAEEPSVR.V
SPRE_MOUSE	MK_SCX_18.4785.4785.2	2	3.837	0.529	1	425.4	52.941177	R.VLSYAPGPLDNDM*QQLAR.E
SPRE_MOUSE	MK_SCX_18.5830.5830.2	2	3.915	0.501	1	485.6	55.88235	R.VLSYAPGPLDNDMQQLAR.E
SPRE_MOUSE	MK_SCX_19.4456.4456.2	2	5.181	0.427	1	3137	80	K.VVLAAADLGTEAGVQR.L
SPRE_MOUSE	MK_SCX_19.5270.5270.2	2	4.911	0.593	1	2223.5	82.14286	R.LLLINNAATLGDVSK.G
SPRE_MOUSE	MK_SCX_21.4893.4893.2	2	3.075	0.405	1	506.9	62.5	R.LLSPGSVM*LVSAR.S
SPRE_MOUSE	MK_SCX_33.3656.3656.2	2	3.838	0.406	1	771.8	76.92308	R.QLKEELGAQQPDLK.V
SPTA2_MOUSE	MK_SCX_15.5136.5136.2	2	5.713	0.647	1	1593.5	65	K.DLNSQADSLM*TSSAFDTSQVK.E
SPTA2_MOUSE	MK_SCX_15.6071.6071.2	2	6.783	0.695	1	1745.4	62.5	K.DLNSQADSLMTSSAFDTSQVK.E
SPTA2_MOUSE	MK_SCX_16.6281.6281.2	2	5.88	0.536	1	2619.7	72.5	K.EAALTNEEVGADLEQVEVLQK.K
SPTA2_MOUSE	MK_SCX_16.8800.8800.2	2	3.809	0.571	1	677.4	47.5	K.MTLVASEDYGDTLAAIQGLLK.K
SPTA2_MOUSE	MK_SCX_19.3677.3677.2	2	2.273	0.145	1	320.4	57.14286	K.LSDDNTIGQEEIQQR.L
SPTA2_MOUSE	MK_SCX_19.6026.6026.2	2	5.864	0.591	1	2870.4	79.411766	R.SSLSSAQADFNQLAELDR.Q
SPTA2_MOUSE	MK_SCX_19.6379.6379.2	2	3.261	0.334	1	576.9	66.66667	R.ELPTAFDYVEFTR.S
SPTA2_MOUSE	MK_SCX_19.9294.9294.2	2	4.87	0.66	1	1457.8	63.88889	R.VASNPYTWFTMEALEETWR.N
SPTA2_MOUSE	MK_SCX_21.10666.10666.3	3	6.1	0.603	1	1024.3	30.172413	K.QETFDAGLQAFQQEGIANITALKDQLLAAK.H
SPTA2_MOUSE	MK_SCX_21.5250.5250.2	2	3.287	0.286	1	793.1	81.818184	R.GVIDMGNSLIER.G
SPTA2_MOUSE	MK_SCX_21.5288.5288.2	2	2.709	0.255	1	624.4	63.636364	R.EANELQQWITEK.E
SPTA2_MOUSE	MK_SCX_23.6902.6902.2	2	5.818	0.506	1	1192.7	55	R.EAFLNTEDKGDSLDSVEALIK.K
SPTA2_MOUSE	MK_SCX_24.6993.6993.3	3	4.045	0.303	1	926.9	35	R.EAFLNTEDKGDSLDSVEALIK.K
SPTA2_MOUSE	MK_SCX_25.4226.4226.2	2	2.595	0.316	1	469.1	85	R.WTQLLANSATR.K
SPTA2_MOUSE	MK_SCX_25.5202.5202.3	3	4.735	0.589	1	909.9	36.764706	R.DLAALGDKVNSLGETAQR.L
SPTA2_MOUSE	MK_SCX_25.5218.5218.2	2	5.245	0.582	1	2581	76.47059	R.DLAALGDKVNSLGETAQR.L
SPTA2_MOUSE	MK_SCX_25.8087.8087.3	3	3.148	0.361	1	873.4	36.25	R.GLVSSDELAKDVTGAEALLER.H
SPTA2_MOUSE	MK_SCX_26.8496.8496.3	3	4.331	0.557	1	741.3	38.88889	K.IAALQAFADQLIAVDHYAK.G
SPTA2_MOUSE	MK_SCX_26.8530.8530.2	2	5.181	0.625	1	2185.1	63.88889	K.IAALQAFADQLIAVDHYAK.G
SPTA2_MOUSE	MK_SCX_27.3536.3536.2	2	5.665	0.588	1	1434.3	70.588234	K.LQTASDESYKDPTNIQSK.H
SPTA2_MOUSE	MK_SCX_27.4247.4247.2	2	2.855	0.362	1	604.5	68.181816	R.DMDDEESWIKEK.K

SPTA2_MOUSE	MK_SCX_27.5900.5900.3	3	3.871	0.476	1	997	35.227272	R.LKDLNSQADSLMTSSAFDTSQVK.E
SPTA2_MOUSE	MK_SCX_28.3518.3518.3	3	3.509	0.483	1	449	39.705883	K.LQTASDESYKDPTNIQSK.H
SPTA2_MOUSE	MK_SCX_28.5187.5187.3	3	3.012	0.331	1	535.1	31.25	K.NQALNTDNYGHDLASVQALQR.K
SPTA2_MOUSE	MK_SCX_28.5202.5202.2	2	6.914	0.674	1	2200.8	65	K.NQALNTDNYGHDLASVQALQR.K
SPTA2_MOUSE	MK_SCX_33.3548.3548.3	3	5.265	0.305	1	2757.1	56.666668	K.KLSDDNTIGQEEIQQR.L
SPTA2_MOUSE	MK_SCX_36.6442.6442.2	2	2.407	0.184	1	628.5	70	R.KVEDLFLTFAK.K
SPTA2_MOUSE	MK_SCX_39.3869.3869.2	2	3.996	0.282	1	1474.8	73.07692	R.SKLGESQTLQQFSR.D
SPTA2_MOUSE	MK_SCX_39.3875.3875.3	3	4.032	0.254	1	368.8	36.53846	R.SKLGESQTLQQFSR.D
SPTA2_MOUSE	MK_SCX_43.5008.5008.3	3	5.579	0.55	1	2680.9	48.75	R.ALSSEGKPYVTKEELYQNLTR.E
SPTA2_MOUSE	MK_SCX_43.5856.5856.3	3	3.863	0.559	1	726.4	36.25	K.RLEAELAAHEPAIQGVLDTGK.K
SPTA2_MOUSE	MK_SCX_45.3071.3071.3	3	3.528	0.47	1	569.7	50	R.LIQSHPESAEDLKEK.C
SPTA2_MOUSE	MK_SCX_52.5176.5176.3	3	4.482	0.53	1	1370.8	39.285713	K.RLEAELAAHEPAIQGVLDTGKK.L
SPTA2_MOUSE	MK_SCX_53.3224.3224.3	3	3.479	0.286	1	1182.8	50	K.HQAFEAELHANADR.I
SPTB2_MOUSE	MK_SCX_16.7096.7096.2	2	2.515	0.308	1	373.7	44.444447	R.DMGEMVTQGQTDAQYMFLR.Q
SPTB2_MOUSE	MK_SCX_16.7356.7356.2	2	5.058	0.609	1	2090	69.44444	R.DASVAEAWLLGQEPYLSSR.E
SPTB2_MOUSE	MK_SCX_17.6480.6480.2	2	5.719	0.673	1	1536.6	72.22222	R.LVSQDNFGFDLPAVEAATK.K
SPTB2_MOUSE	MK_SCX_18.3321.3321.2	2	2.26	0.287	1	412.7	43.75	K.GDQVSQNGLPAEQGSPR.M
SPTB2_MOUSE	MK_SCX_18.3889.3889.2	2	3.979	0.375	1	1483.8	70.588234	R.MAGTMETSEMVNGAAEQR.T
SPTB2_MOUSE	MK_SCX_18.3996.3996.2	2	5.309	0.618	1	2142.4	75	R.TQTAIASEDM*PNTLTEAEK.L
SPTB2_MOUSE	MK_SCX_19.7735.7735.2	2	4.185	0.356	1	740.2	63.333332	R.FQIQDISVETEDNKEK.K
SPTB2_MOUSE	MK_SCX_20_1.3504.3504.2	2	4.695	0.535	1	2245.3	87.5	K.MLTAQDM*SYDEAR.N
SPTB2_MOUSE	MK_SCX_20_1.3509.3509.2	2	4.53	0.481	1	633	76.92308	R.FESLEPEM*NNQASR.V
SPTB2_MOUSE	MK_SCX_20_1.4586.4586.2	2	4.21	0.555	1	1361.1	78.57143	R.TLETPAAQM*EGFLNR.K
SPTB2_MOUSE	MK_SCX_20_1.6833.6833.3	3	3.537	0.397	1	980	42.857143	R.TLETPAAQMEGFLNR.K
SPTB2_MOUSE	MK_SCX_21.4110.4110.2	2	2.118	0.26	1	378.6	65.38461	K.SALPAQSAATLPAR.T
SPTB2_MOUSE	MK_SCX_21.6562.6562.2	2	4.092	0.335	1	1336.1	90	K.FMELLEPLSER.K
SPTB2_MOUSE	MK_SCX_2201.3041.3041.2	2	2.285	0.295	1	904	93.75	R.VQAVVAVAR.E
SPTB2_MOUSE	MK_SCX_2201.6577.6577.2	2	3.956	0.499	1	1665.4	79.16667	R.EQWANLEQLSAIR.K
SPTB2_MOUSE	MK_SCX_2201.8087.8087.3	3	3.207	0.226	1	655.2	28	R.VDTVNNMADELINSGHSDAATIAEWK.D
SPTB2_MOUSE	MK_SCX_27.4176.4176.2	2	5.186	0.334	1	1050.9	55.555557	K.IVSSNDVGHDEYSTQSLVK.K
SPTB2_MOUSE	MK_SCX_27.4685.4685.2	2	4.407	0.347	1	1268.8	65.625	K.EGMQLISEKPETEAVVK.E
SPTB2_MOUSE	MK_SCX_28.5369.5369.2	2	4.462	0.543	1	1296.4	70	K.ILSSDDYGKDLTSVMR.L
SPTB2_MOUSE	MK_SCX_28.7219.7219.3	3	3.835	0.378	1	593.8	33.333336	K.TKVIESTQDLGNDLAGVMALQR.K
SPTB2_MOUSE	MK_SCX_29.4398.4398.3	3	4.457	0.529	1	786.8	40.625	K.LPEELGRDQNTVETLQR.M
SPTB2_MOUSE	MK_SCX_29.4808.4808.2	2	5.266	0.647	1	1136.1	65.789474	R.AQTLPTSVVTITSESSPGKR.E
SPTB2_MOUSE	MK_SCX_30.4558.4558.2	2	4.657	0.517	1	685.3	62.5	K.SAASGIPYHSEVPVSLK.E
SPTB2_MOUSE	MK_SCX_35.3604.3604.3	3	3.844	0.34	1	999.3	41.666664	K.AKSALPAQSAATLPAR.T
SPTB2_MOUSE	MK_SCX_36.5161.5161.3	3	4.545	0.578	1	1057.7	36.904762	R.EVVAGSHELGQDYEHVTMLQER.F
SPTB2_MOUSE	MK_SCX_38.3811.3811.3	3	3.665	0.365	1	1087.4	43.055553	K.EGM*QLISEKPETEAVVKEK.L
SPTB2_MOUSE	MK_SCX_38.4391.4391.2	2	2.443	0.394	1	344.5	59.090908	R.KQALQDTLALYK.M
SPTB2_MOUSE	MK_SCX_40.3967.3967.3	3	3.654	0.502	1	893.6	34.72222	K.EAEKLESEHPDQAQAILSR.L
SPTB2_MOUSE	MK_SCX_43.7746.7746.3	3	6.148	0.585	1	2079.5	51.315792	K.HQILEQAVEDYAETVHQLSK.T
SPTB2_MOUSE	MK_SCX_44.6700.6700.3	3	3.562	0.389	1	591.3	33.333336	K.EIHQFNRDVEDEILWVGER.M
SPTB2_MOUSE	MK_SCX_45.7498.7498.3	3	4.154	0.279	1	323.2	30.952381	K.SNAHYNLQNAFNLAEQHLGLTK.L
SPTB2_MOUSE	MK_SCX_47.3163.3163.3	3	3.346	0.483	1	603.3	48.333332	R.LQAAYAGDKADDIQKR.E
SPTB2_MOUSE	MK_SCX_48.5018.5018.3	3	4.504	0.504	1	1751.7	51.785713	K.VDKLYAGLKDLAEER.R
SPTB2_MOUSE	MK_SCX_49.6389.6389.3	3	6.094	0.614	1	2154	44.791664	K.LSDLQKEAEKLESEHPDQAQAILSR.L
SPTB2_MOUSE	MK_SCX_50.5058.5058.3	3	3.388	0.3	1	810.3	40.625	R.MHTTFEHDIQALGTQVR.Q
SPTB2_MOUSE	MK_SCX_50.5084.5084.2	2	5.142	0.618	1	2233.8	75	R.MHTTFEHDIQALGTQVR.Q
SPTB2_MOUSE	MK_SCX_51.4546.4546.3	3	5.752	0.605	1	2882.6	48.61111	R.KKEIEELQSQAQALSQEGK.S
SPTB2_MOUSE	MK_SCX_51.4560.4560.2	2	6.752	0.691	1	2717.3	72.22222	R.KKEIEELQSQAQALSQEGK.S

SQRD_MOUSE	MK_SCX_19.6307.6307.2	2	3.125	0.362	1	493.9	60.000004	R.STLSVIPSGVQWIQDR.V
SQRD_MOUSE	MK_SCX_20_1.4040.4040.2	2	3.563	0.557	1	1349.6	69.230774	R.VGAENVAIVEPSER.H
SQRD_MOUSE	MK_SCX_21.4176.4176.2	2	4.726	0.493	1	2397	84.61539	K.TAAAVAAQSGILDR.T
SQRD_MOUSE	MK_SCX_2201.5135.5135.2	2	3.242	0.389	1	547.6	88.88889	K.YADALQEIIR.E
SQRD_MOUSE	MK_SCX_23.5207.5207.2	2	3.582	0.392	1	993.6	88.88889	K.IM*YLSEAYFR.K
SQRD_MOUSE	MK_SCX_23.5655.5655.2	2	3.667	0.412	1	1153.5	88.88889	K.IMYLSEAYFR.K
SQRD_MOUSE	MK_SCX_37.3798.3798.2	2	4.487	0.555	1	1354.9	85.71429	R.RVGAENVAIVEPSER.H
SQRD_MOUSE	MK_SCX_38.7422.7422.3	3	3.674	0.267	1	938.2	46.42857	R.HFYQPIWTLVGAGAK.E
SQRD_MOUSE	MK_SCX_39.5742.5742.2	2	2.851	0.393	1	597.6	70	K.KYADALQEIIR.E
SQRD_MOUSE	MK_SCX_39.8103.8103.2	2	2.873	0.507	1	355.9	60.714287	R.HFYQPIWTLVGAGAK.E
SQRD_MOUSE	MK_SCX_55.3442.3442.3	3	3.457	0.429	1	998.2	38.333332	K.RRVGAENVAIVEPSER.H
SRA1_MOUSE	MK_SCX_19.5174.5174.2	2	4.189	0.634	1	669.2	68.42105	R.GWNDPPQFSYGLQTQTGGPK.R
SRA1_MOUSE	MK_SCX_27.3158.3158.3	3	4.175	0.429	1	1050.7	41.17647	R.APETSGPPPVDHPPPSSK.A
SRBS1_MOUSE	MK_SCX_18.3498.3498.2	2	3.537	0.474	1	907.5	58.333332	K.LSSSADTNGNAQPSPLAAK.G
SRBS1_MOUSE	MK_SCX_18.4585.4585.2	2	4.337	0.506	1	1087.2	64.70589	R.AGEQDPVPTPAELTSPGR.A
SRBS1_MOUSE	MK_SCX_19.4062.4062.2	2	3.144	0.377	1	672	66.66667	R.ESDGTPGGLASLENER.Q
SRBS1_MOUSE	MK_SCX_29.8641.8641.3	3	3.167	0.155	1	613.1	34.72222	R.NNNPQSELAAAHGDSESPR.H
SRBS1_MOUSE	MK_SCX_31.3164.3164.3	3	4.341	0.371	1	1098.4	40	R.ASSSYRGTPSSSPVSPQESPK.H
SRBS1_MOUSE	MK_SCX_31.4808.4808.2	2	4.416	0.567	1	1438.2	71.42857	R.YSFSDDTKSPLSVPR.S
SRBS1_MOUSE	MK_SCX_32.4194.4194.3	3	3.857	0.437	1	610.7	34.72222	R.RAGEQDPVPTPAELTSPGR.A
SRBS1_MOUSE	MK_SCX_32.4285.4285.2	2	4.817	0.563	1	420.9	58.333332	R.RAGEQDPVPTPAELTSPGR.A
SRBS1_MOUSE	MK_SCX_33.6737.6737.2	2	2.728	0.321	1	382	56.666668	R.KLAPVQVLEYGEAIAK.F
SRBS1_MOUSE	MK_SCX_33.6867.6867.3	3	4.419	0.518	1	1567.7	48.333332	R.KLAPVQVLEYGEAIAK.F
SRBS1_MOUSE	MK_SCX_34.7818.7818.2	2	4.995	0.633	1	1217.6	83.33333	R.HFIPADYLESTEEFIR.R
SRC8_MOUSE	MK_SCX_19.5044.5044.2	2	5.108	0.617	1	1326.1	71.875	K.NASTFEEVVQVPSAYQK.T
SRC8_MOUSE	MK_SCX_20_1.3849.3849.2	2	4.531	0.567	1	1400.9	83.33333	R.VDQSAVGFEYQGK.T
SRC8_MOUSE	MK_SCX_20_1.3849.3849.2	2	4.531	0.567	1	1400.9	83.33333	R.VDQSAVGFEYQGK.T
SRC8_MOUSE	MK_SCX_21.6863.6863.2	2	3.402	0.573	1	835	90.909096	R.YGLFPANYVELR.Q
SRC8_MOUSE	MK_SCX_28.5415.5415.3	3	3.396	0.214	1	795.6	36.842106	R.MDKNASTFEEVVQVPSAYQK.T
SRC8_MOUSE	MK_SCX_28.5470.5470.2	2	6.293	0.645	1	2086	76.31579	R.MDKNASTFEEVVQVPSAYQK.T
SRC8_MOUSE	MK_SCX_41.4039.4039.3	3	3.498	0.561	1	517.5	33.333336	K.DYSSGFGGKYGVQADRVDK.S
SRC8_MOUSE	MK_SCX_53.3429.3429.3	3	3.424	0.198	1	1095.9	45.833336	K.LRENVFQEHQTLK.E
SRP09_MOUSE	MK_SCX_23.5054.5054.2	2	4.063	0.581	1	1272.8	90	-.PQFQTWEEFSR.A
SRP54_MOUSE	MK_SCX_16.3987.3987.2	2	3.586	0.317	1	769	55.263157	K.LM*TIM*DSM*NDQELDSTDGAK.V
SRP54_MOUSE	MK_SCX_16.5744.5744.2	2	3.238	0.483	1	805.7	52.63158	K.LMTIMDSMNDQELDSTDGAK.V
SRP54_MOUSE	MK_SCX_17.10499.10499.2	2	4.056	0.392	1	829.8	57.5	K.MGPFSQILGMIPGFGTDFMSK.G
SRPRB_MOUSE	MK_SCX_18.3877.3877.2	2	4.932	0.553	1	1844.4	75	R.SAAPSTLDSSSTAPAQLGK.K
SRRM1_MOUSE	MK_SCX_14.5766.5766.3	3	5.61	0.722	1	1639	31.818182	K.AVTIATPATAAPAAVSAATTTSAQEEPAAAPEPR.K
SRRM1_MOUSE	MK_SCX_14.5777.5777.2	2	4.764	0.683	1	1072.7	37.878788	K.AVTIATPATAAPAAVSAATTTSAQEEPAAAPEPR.K
SSA27_MOUSE	MK_SCX_15.5972.5972.2	2	4.648	0.498	1	581.1	48.076923	K.AAQAPPLPAAPPNTDAVASTQTALLQK.L
SSA27_MOUSE	MK_SCX_15.5994.5994.3	3	4.808	0.52	1	1172.8	31.730768	K.AAQAPPLPAAPPNTDAVASTQTALLQK.L
SSB_MOUSE	MK_SCX_19.2756.2756.2	2	4.478	0.481	1	1728.9	75	R.SGDSEVYQM*GDVSQK.T
SSB_MOUSE	MK_SCX_19.3626.3626.2	2	4.683	0.485	1	1899.9	78.57143	R.SGDSEVYQMGDVSQK.T
SSB_MOUSE	MK_SCX_35.4609.4609.2	2	2.499	0.213	1	677.4	77.77778	R.SLNRVQLLGR.V
SSB_MOUSE	MK_SCX_45.3955.3955.2	2	2.125	0.186	1	303.5	68.75	R.ISVFRPGLR.D
SSDH_MOUSE	MK_SCX_14.10019.10019.3	3	3.635	0.411	1	309.7	22.413794	R.VSMELGGLAPFIVFDSANVDQAVAGAMASK.F
SSDH_MOUSE	MK_SCX_17.4404.4404.2	2	3.434	0.195	1	1293.2	58.823532	R.VGNGFEEGTTQGPLINEK.A
SSDH_MOUSE	MK_SCX_18.6470.6470.2	2	3.119	0.496	1	318	50	R.WLPAPATFPVYDPASGAK.L
SSDH_MOUSE	MK_SCX_21.5454.5454.2	2	2.699	0.296	1	567	72.22222	K.YGIDEYLEVK.Y
SSDH_MOUSE	MK_SCX_2201.3122.3122.2	2	2.74	0.46	1	860.1	80	K.ISFTGSTATGK.I
SSDH_MOUSE	MK_SCX_36.6554.6554.2	2	4.675	0.58	1	1117.6	70.588234	R.HQSGGNFFEPTLLSNVTR.D

SSDH_MOUSE	MK_SCX_50.5535.5535.3	3	3.611	0.359	1	475.2	41.07143	R.KWYDLMIQNKDDLAK.I
SSH3_MOUSE	MK_SCX_12.6198.6198.2	2	2.471	0.178	1	467.1	46.42857	R.QALELRLGCPLQQYR.D
SSH3_MOUSE	MK_SCX_34.3419.3419.3	3	3.574	0.327	1	500.9	32.954548	R.TRAFQEQGQGQEQSEPGMSSTPR.L
SSRA_MOUSE	MK_SCX_15.7052.7052.2	2	4.309	0.564	1	356.6	38	K.VEMGTSSQNDVDMSWIPQETLNQINK.A
SSRA_MOUSE	MK_SCX_18.7762.7762.2	2	3.484	0.397	1	684.8	57.14286	K.GTEDFIVESLDASFR.Y
SSRD_MOUSE	MK_SCX_21.5871.5871.2	2	3.205	0.476	1	1366.6	85	R.FFDEESYSLLR.K
SSRD_MOUSE	MK_SCX_40.3535.3535.3	3	3.417	0.403	1	800	42.857143	K.NRVQNM*ALYADVSGK.Q
ST14_MOUSE	MK_SCX_24.6590.6590.3	3	3.767	0.336	1	636	25	R.MMCVGFLSGGVDSCQGDSGGPLSSAEKDGR.M
ST1C2_MOUSE	MK_SCX_21.5676.5676.2	2	2.669	0.385	1	702.7	68.181816	K.SILDQSISPFM*R.K
ST1C2_MOUSE	MK_SCX_21.6786.6786.2	2	4.232	0.427	1	1235.9	86.36364	K.SILDQSISPFMR.K
ST1C2_MOUSE	MK_SCX_2201.4147.4147.2	2	2.614	0.454	1	451.1	81.25	K.IVLETSFEK.M
ST1C2_MOUSE	MK_SCX_25.7715.7715.3	3	4.64	0.34	1	925	39.705883	K.NLDEDVVDKIVLETSFEK.M
ST1C2_MOUSE	MK_SCX_25.8351.8351.2	2	3.265	0.267	1	311.6	41.17647	K.NLDEDVVDKIVLETSFEK.M
ST1C2_MOUSE	MK_SCX_31.6506.6506.2	2	5.709	0.548	1	1017.6	73.52941	K.LKEVAGIPLQAPTVDNWR.Q
ST1C2_MOUSE	MK_SCX_31.6515.6515.3	3	4.215	0.509	1	2014.3	51.47059	K.LKEVAGIPLQAPTVDNWR.Q
ST1C2_MOUSE	MK_SCX_52.5152.5152.3	3	3.578	0.537	1	363.5	28.125	R.HPFIEWARPPQPSGVDKANEMPAPR.I
STAB1_MOUSE	MK_SCX_10.4748.4748.2	2	2.23	0.25	1	380.5	43.75	R.CQEGFHGTACEM*CELGR.Y
STAB1_MOUSE	MK_SCX_27.4675.4675.2	2	2.21	0.286	1	459.3	59.090908	R.EGYSGDGIQTCK.L
STAM1_MOUSE	MK_SCX_19.5239.5239.2	2	4.724	0.516	1	1291.5	80.769226	-.PLFATNPFDQDVEK.A
STAT1_MOUSE	MK_SCX_34.5764.5764.3	3	3.918	0.175	1	1105.9	48.076923	R.FHDLLSQLDDQYSR.F
STAT1_MOUSE	MK_SCX_48.4401.4401.2	2	4.188	0.564	1	1135.9	73.07692	K.YLYPNIDKDHAFGK.Y
STAT3_MOUSE	MK_SCX_25.4193.4193.3	3	5.801	0.572	1	1348	37	R.LLQTAATAAQQGGQANHPTAAVVTEK.Q
STAT3_MOUSE	MK_SCX_44.4981.4981.3	3	3.168	0.122	1	601.5	31.25	R.QKMQQLEQMLTALDQMR.R
STAU1_MOUSE	MK_SCX_19.4326.4326.2	2	3.331	0.549	1	1223.9	73.07692	K.LLSELDQQSTEMPR.T
STAU1_MOUSE	MK_SCX_19.5675.5675.2	2	2.498	0.239	1	440.1	46.666668	R.VSVGEFVGEGEGKSKK.I
STCH_MOUSE	MK_SCX_34.6603.6603.2	2	4.016	0.416	1	1333.2	75	R.KLFDALNEDLFQK.I
STIP1_MOUSE	MK_SCX_18.3796.3796.2	2	4.455	0.502	1	1680	68.75	R.AM*ADPEVQQIM*SDPAM*R.L
STIP1_MOUSE	MK_SCX_18.5300.5300.2	2	4.829	0.501	1	1807.9	71.875	R.AMADPEVQQIMSDPAMR.L
STIP1_MOUSE	MK_SCX_19.3865.3865.2	2	5.496	0.601	1	1960.3	75	K.ALDLDSSCKEAADGYQR.C
STIP1_MOUSE	MK_SCX_19.5066.5066.2	2	4.388	0.445	1	1318.8	87.5	R.LAYINPDLALEEK.N
STIP1_MOUSE	MK_SCX_21.7224.7224.2	2	3.943	0.464	1	1150.3	75	K.FM*NPFNLPNLYQK.L
STIP1_MOUSE	MK_SCX_21.7786.7786.2	2	4.536	0.58	1	1446.7	83.33333	K.FMNPFNLPNLYQK.L
STIP1_MOUSE	MK_SCX_21.7827.7827.3	3	3.701	0.351	1	1065.2	54.166668	K.FMNPFNLPNLYQK.L
STIP1_MOUSE	MK_SCX_28.4327.4327.2	2	4.886	0.444	1	1573.4	73.333336	R.ELIEQLQNKPSDLGTK.L
STIP1_MOUSE	MK_SCX_28.4362.4362.3	3	3.576	0.445	1	914.5	45	R.ELIEQLQNKPSDLGTK.L
STIP1_MOUSE	MK_SCX_32.5176.5176.3	3	4.693	0.484	1	898.8	41.17647	R.RAMADPEVQQIMSDPAMR.L
STIP1_MOUSE	MK_SCX_34.7939.7939.3	3	4.737	0.532	1	882.8	32.291664	R.SLLSDPTYRELIEQLQNKPSDLGTK.L
STIP1_MOUSE	MK_SCX_38.7479.7479.3	3	3.79	0.329	1	1798.3	55.76923	R.KFMNPFNLPNLYQK.L
STIP1_MOUSE	MK_SCX_38.7843.7843.2	2	3.778	0.382	1	891.9	80.769226	R.KFMNPFNLPNLYQK.L
STIP1_MOUSE	MK_SCX_39.4549.4549.3	3	3.417	0.492	1	609.9	37.5	K.DPQALSEHLKNPVIAQK.I
STIP1_MOUSE	MK_SCX_46.5280.5280.3	3	6.922	0.59	1	1028.5	41.666664	R.LILEQM*QKDPQALSEHLKNPVIAQK.I
STIP1_MOUSE	MK_SCX_46.6125.6125.3	3	6.391	0.581	1	1483.8	39.583336	R.LILEQMQKDPQALSEHLKNPVIAQK.I
STIP1_MOUSE	MK_SCX_48.3315.3315.3	3	4.397	0.421	1	1786.5	55	K.KEPKPEPMEEDLPENK.K
STIP1_MOUSE	MK_SCX_49.4295.4295.3	3	4.321	0.589	1	807.3	45	R.TYEEGLKHEANNLQLK.E
STIP1_MOUSE	MK_SCX_49.4308.4308.2	2	4.952	0.462	1	800.4	60.000004	R.TYEEGLKHEANNLQLK.E
STIP1_MOUSE	MK_SCX_52.5141.5141.3	3	8.182	0.562	1	1434	39.583336	R.TYEEGLKHEANNLQLKEGLQNMEAR.L
STK10_MOUSE	MK_SCX_24.3968.3968.3	3	4.048	0.427	1	720.7	35.714287	R.IQMDEEKQIPDQDENPSPAASK.S
STML2_MOUSE	MK_SCX_13.10405.10405.2	2	4.244	0.604	1	1091.5	39.655174	K.DSNTVLLPSNPSDVTSMVAQAMGVYGALTK.A
STML2_MOUSE	MK_SCX_13.10445.10445.3	3	4.192	0.365	1	567.1	25	K.DSNTVLLPSNPSDVTSMVAQAMGVYGALTK.A
STML2_MOUSE	MK_SCX_17.4306.4306.2	2	4.699	0.542	1	2685.4	84.61539	R.DVQATDTSIEELGR.V
STML2_MOUSE	MK_SCX_18.8074.8074.2	2	4.239	0.501	1	732.8	75	R.ILEPGLNVLIPVLDR.I

STML2_MOUSE	MK_SCX_19.4401.4401.2	2	5.495	0.465	1	3004.9	81.25	K.AEQINQAAGEASAVLAK.A
STML2_MOUSE	MK_SCX_2201.3492.3492.2	2	2.758	0.399	1	841	70	K.ESMQMQVEAER.R
STML2_MOUSE	MK_SCX_2201.7475.7475.3	3	4.288	0.474	1	692.9	29.807693	R.IMDPYKASYGVEDPEYAVTQLAQTTM*R.S
STML2_MOUSE	MK_SCX_2201.8111.8111.3	3	5.211	0.592	1	1556.1	34.615387	R.IMDPYKASYGVEDPEYAVTQLAQTTMR.S
STML2_MOUSE	MK_SCX_34.3729.3729.3	3	3.019	0.332	1	438.9	39.285713	R.RDVQATDTSIEELGR.V
STRAP_MOUSE	MK_SCX_17.5450.5450.2	2	4.552	0.571	1	1033.2	61.764706	K.TVDFTQDSNYLLTGGQDK.L
STRAP_MOUSE	MK_SCX_2201.2807.2807.2	2	2.801	0.531	1	425.3	75	K.AATAAADFTAK.V
STRN_MOUSE	MK_SCX_19.4798.4798.2	2	5.518	0.627	1	1329.8	66.66667	K.GLGPLAEAAAAGDGAAAAGAAR.A
STRN_MOUSE	MK_SCX_35.11308.11308.3	3	3.449	0.318	1	668.8	31.25	R.ALAFHPIEPVLITASEDHTLK.M
STRN3_MOUSE	MK_SCX_23.5718.5718.3	3	3.944	0.395	1	562.5	28	K.MPTFESEETKDVEAPPAPQNSQLTWK.Q
STUB1_MOUSE	MK_SCX_14.3829.3829.2	2	2.315	0.261	1	484.6	63.636364	K.MQQPEQALADCR.R
STUB1_MOUSE	MK_SCX_19.5646.5646.2	2	3.633	0.538	1	1309.9	75	R.SPLTQEQLIPNLAMK.E
STUB1_MOUSE	MK_SCX_23.4352.4352.2	2	3.936	0.504	1	973.5	80	R.NPLVAVYYTNR.A
STUB1_MOUSE	MK_SCX_31.3083.3083.3	3	3.392	0.453	1	1002.8	40.27778	R.LGTGGGGSPDKSPSAQELK.E
STUB1_MOUSE	MK_SCX_43.3321.3321.3	3	6.691	0.567	1	1833.4	42.391304	R.LGTGGGGSPDKSPSAQELKEQGNR.L
STX12_MOUSE	MK_SCX_19.5286.5286.2	2	3.234	0.479	1	483.6	64.28571	K.ELGSLPLPLSASEQR.Q
STX12_MOUSE	MK_SCX_19.6608.6608.2	2	5.652	0.552	1	2306	71.875	R.LMNDFSSALNNFQVVQR.K
STX12_MOUSE	MK_SCX_25.8571.8571.3	3	4.163	0.435	1	1180.2	42.857143	K.ETNELLKELGSLPLPLSASEQR.Q
STX12_MOUSE	MK_SCX_31.4431.4431.2	2	5.207	0.515	1	1969.8	71.875	K.LQENLQQLQHSTNQLAK.E
STX17_MOUSE	MK_SCX_16.10952.10952.2	2	4.987	0.574	1	716.8	48	K.LAALPVAGALIGGVVGGPIGLLAGFK.V
STX3_MOUSE	MK_SCX_14.9168.9168.3	3	5.3	0.593	1	1539.5	33.92857	K.TTDEELEEMLESGNPAIFTSGIIDSQISK.Q
STX3_MOUSE	MK_SCX_15.8980.8980.2	2	4.68	0.613	1	828.9	39.285713	K.TTDEELEEMLESGNPAIFTSGIIDSQISK.Q
STX3_MOUSE	MK_SCX_23.8296.8296.3	3	3.333	0.383	1	389.7	20.689655	K.KTTDEELEEM*LESGNPAIFTSGIIDSQISK.Q
STX3_MOUSE	MK_SCX_23.9326.9326.3	3	6.115	0.631	1	1625.5	36.206894	K.KTTDEELEEMLESGNPAIFTSGIIDSQISK.Q
STX3_MOUSE	MK_SCX_24.3627.3627.2	2	2.256	0.323	1	703.6	93.75	K.YNEAQVDFR.E
STX3_MOUSE	MK_SCX_48.4131.4131.3	3	4.055	0.3	1	1633.7	55.76923	R.LNIDKISEHVEEAK.K
STX4_MOUSE	MK_SCX_29.3455.3455.3	3	4.747	0.519	1	1197.2	37.5	K.AIEPQKEEADENYNSVNTR.M
STX7_MOUSE	MK_SCX_12.8861.8861.2	2	2.305	0.158	1	377.7	46.153847	R.QLEADIMDINEIFK.D
STX7_MOUSE	MK_SCX_14.3595.3595.2	2	4.134	0.42	1	1860.1	94.44444	K.ITQCSVEIQR.T
STX7_MOUSE	MK_SCX_15.6390.6390.2	2	5.992	0.528	1	558.2	44	K.NLVSWESQTQPQVQVQDEEITEDDLR.L
STX7_MOUSE	MK_SCX_20_1.15803.15803.2	2	3.131	0.341	1	508.4	65.38461	R.LVAEFTTSLTNFQK.A
STX7_MOUSE	MK_SCX_20_1.4091.4091.2	2	2.958	0.369	1	491.1	66.66667	K.EFGSLPTTPSEQR.Q
STX7_MOUSE	MK_SCX_20_1.4264.4264.2	2	5.084	0.441	1	1312.1	78.57143	R.TLNQLGTPQDSPELR.Q
STX7_MOUSE	MK_SCX_20_1.4275.4275.3	3	3.013	0.349	1	584.9	44.642857	R.TLNQLGTPQDSPELR.Q
STX7_MOUSE	MK_SCX_42.5068.5068.2	2	5.064	0.626	1	923.3	55.263157	K.ETDKYIKEFGSLPTTPSEQR.Q
STX7_MOUSE	MK_SCX_43.5080.5080.3	3	4.28	0.473	1	527.2	35.526314	K.ETDKYIKEFGSLPTTPSEQR.Q
STX8_MOUSE	MK_SCX_17.6687.6687.2	2	4.711	0.477	1	1676.3	73.52941	K.IIQEQDAGLDALSSIISR.Q
STXB2_MOUSE	MK_SCX_20_1.6217.6217.2	2	2.115	0.124	1	372.1	53.571426	K.WEVLIGSSHILTPTR.F
STXB2_MOUSE	MK_SCX_27.3765.3765.3	3	3.417	0.264	1	657.8	32.894737	K.AAHIFFTDTCPEPLFSELGR.S
SUCA_MOUSE	MK_SCX_17.6369.6369.2	2	5.208	0.641	1	1595.5	56.521736	K.ISALQSAGVVVSM*SPAQLGTTIYK.E
SUCA_MOUSE	MK_SCX_23.13602.13602.3	3	6.651	0.506	1	1062.8	31.48148	K.ISALQSAGVVVSMSPAQLGTTIYKEFEK.R
SUCA_MOUSE	MK_SCX_23.8530.8530.3	3	6.198	0.526	1	1065.8	32.407406	K.ISALQSAGVVVSM*SPAQLGTTIYKEFEK.R
SUCA_MOUSE	MK_SCX_23.9429.9429.2	2	4.061	0.451	1	670.4	38.88889	K.ISALQSAGVVVSMSPAQLGTTIYKEFEK.R
SUCA_MOUSE	MK_SCX_32.5682.5682.2	2	4.504	0.651	1	1510.8	71.875	K.AKPVVSFIAGITAPPGR.R
SUCA_MOUSE	MK_SCX_37.3373.3373.3	3	3.313	0.42	1	548.6	42.857143	K.QGTFHSQQALEYGTK.L
SUCA_MOUSE	MK_SCX_40.5287.5287.2	2	2.775	0.485	1	1003	85	K.HLGLPVFNTVK.E
SUCA_MOUSE	MK_SCX_40.5487.5487.3	3	3.551	0.301	1	616	47.5	K.HLGLPVFNTVK.E
SUCA_MOUSE	MK_SCX_50.5416.5416.2	2	3.432	0.417	1	577.2	47.058823	K.AKPVVSFIAGITAPPGRR.M
SUCA_MOUSE	MK_SCX_51.4528.4528.3	3	4.818	0.568	1	1053.3	50	K.HLGLPVFNTVKEAK.E
SUCA_MOUSE	MK_SCX_51.4549.4549.2	2	4.461	0.671	1	1182.1	84.61539	K.HLGLPVFNTVKEAK.E
SUCA_MOUSE	MK_SCX_56.2516.2516.3	3	3.053	0.375	1	1643.8	50	R.RMGHAGAIIAGGK.G

SUCA_MOUSE	MK_SCX_59.8031.8031.2	2	2.501	0.402	1	506.9	65	K.IGIMPGHIHKK.G
SUCB1_MOUSE	MK_SCX_16.4479.4479.2	2	5.865	0.585	1	1346.7	70.588234	K.YDATM*VEINPM*VEDSDGK.V
SUCB1_MOUSE	MK_SCX_16.5547.5547.2	2	4.877	0.484	1	1203	64.70589	K.YDATMVEINPMVEDSDGK.V
SUCB1_MOUSE	MK_SCX_18.12082.12082.3	3	6.658	0.572	1	1075.6	28.846153	R.SFQGPVLIGSAQGGVNIEDVAAENPEAIVKEPIDIVEGIK.K
SUCB1_MOUSE	MK_SCX_19.5349.5349.2	2	4.727	0.561	1	1777.9	75	K.MGFPSNIVDSAAENM*IK.L
SUCB1_MOUSE	MK_SCX_19.6766.6766.2	2	5.033	0.458	1	1716.6	75	K.MGFPSNIVDSAAENMIK.L
SUCB1_MOUSE	MK_SCX_2201.2719.2719.2	2	2.943	0.292	1	627.5	77.77778	K.SSDEAYAIAK.K
SUCB1_MOUSE	MK_SCX_2201.4034.4034.2	2	2.789	0.248	1	725.1	87.5	K.LSEIVTLAK.E
SUCB1_MOUSE	MK_SCX_25.5129.5129.2	2	2.499	0.171	1	506.3	91.66667	K.LYNLFLK.Y
SUCB1_MOUSE	MK_SCX_30.7552.7552.3	3	4.189	0.373	1	632.9	28	K.LHGGTPANFLDVGGGATVQQVTEAFK.L
SUCB2_MOUSE	MK_SCX_18.5538.5538.2	2	4.937	0.626	1	1162.8	78.125	K.SSGLPITSAVDLEDAAK.K
SUCB2_MOUSE	MK_SCX_19.4630.4630.2	2	5.645	0.662	1	2617.6	71.875	K.VVGELAQQM*IGYNLATK.Q
SUCB2_MOUSE	MK_SCX_19.5853.5853.2	2	5.716	0.563	1	2460.4	78.125	K.VVGELAQQMIGYNLATK.Q
SUCB2_MOUSE	MK_SCX_20_1.4759.4759.2	2	5.303	0.35	1	1782.9	84.61539	R.LEGTNVQEAQNILK.S
SUCB2_MOUSE	MK_SCX_21.17019.17019.2	2	3.614	0.436	1	1439.4	90	K.VMVAEALDISR.E
SUCB2_MOUSE	MK_SCX_21.4669.4669.2	2	3.643	0.139	1	1280.8	80	K.VM*VAEALDISR.E
SUCB2_MOUSE	MK_SCX_2201.4199.4199.2	2	3.502	0.413	1	1077.2	88.88889	K.INFDDNAEFR.Q
SUCB2_MOUSE	MK_SCX_2201.4217.4217.3	3	4.691	0.366	1	1831.8	44.736843	K.DIFAM*DDKSENEPIENEAAR.Y
SUCB2_MOUSE	MK_SCX_2201.5449.5449.2	2	4.138	0.439	1	2104.7	90.909096	R.M*AENLGFLGSLK.N
SUCB2_MOUSE	MK_SCX_2201.5538.5538.2	2	2.446	0.277	1	809.8	72.22222	R.ETYLAILM*DR.S
SUCB2_MOUSE	MK_SCX_2201.6238.6238.2	2	4.53	0.547	1	1473.4	90.909096	R.MAENLGFLGSLK.N
SUCB2_MOUSE	MK_SCX_2201.6782.6782.2	2	2.521	0.362	1	791.4	72.22222	R.ETYLAILMDR.S
SUCB2_MOUSE	MK_SCX_23.4826.4826.3	3	4.548	0.548	1	2146.4	44.736843	K.DIFAMDDKSENEPIENEAAR.Y
SUCB2_MOUSE	MK_SCX_23.4840.4840.2	2	4.431	0.543	1	949.6	63.15789	K.DIFAMDDKSENEPIENEAAR.Y
SUCB2_MOUSE	MK_SCX_23.8070.8070.3	3	3.752	0.459	1	1027.6	32.75862	R.SHNGPVIVGSPQGGVDIEEVAASSPELIFK.E
SUCB2_MOUSE	MK_SCX_27.5413.5413.2	2	5.137	0.606	1	1499.6	73.52941	K.SSGLPITSAVDLEDAAKK.A
SUCB2_MOUSE	MK_SCX_28.6522.6522.3	3	4.122	0.418	1	783	37.5	R.MAENLGFLGSLKNQAADQITK.L
SUCB2_MOUSE	MK_SCX_29.5241.5241.2	2	4.522	0.441	1	1705.8	73.333336	K.EQIDIFEGIKDSQAQR.M
SUCB2_MOUSE	MK_SCX_32.5690.5690.2	2	3.069	0.347	1	863.6	85	R.ELELKVPLVVR.L
SUCB2_MOUSE	MK_SCX_39.4864.4864.3	3	5.295	0.424	1	988.6	36.904762	R.QKDIFAMDDKSENEPIENEAAR.Y
SUCB2_MOUSE	MK_SCX_40.3965.3965.3	3	3.503	0.367	1	377.9	23.809525	R.QKDIFAM*DDKSENEPIENEAAR.Y
SUCB2_MOUSE	MK_SCX_45.4426.4426.2	2	2.137	0.289	1	638.5	91.66667	K.LYHLFLK.I
SUCB2_MOUSE	MK_SCX_50.5075.5075.3	3	3.438	0.528	1	484.5	38.333332	R.FFVANTAKEALEAAKR.L
SUCB2_MOUSE	MK_SCX_56.3662.3662.2	2	4.012	0.537	1	659.1	58.823532	K.GVFNSGLKGGVHLTKDPK.V
SUGT1_MOUSE	MK_SCX_13.3679.3679.2	2	4.245	0.597	1	1343.4	78.57143	K.ALEQNPDDAQYYCQR.A
SUGT1_MOUSE	MK_SCX_16.9070.9070.2	2	5.846	0.596	1	1976.5	61.363636	R.LFQSFSDALIDGDPQAALEELTK.A
SUHW4_MOUSE	MK_SCX_17.3653.3653.2	2	4.026	0.537	1	1046.3	57.894737	K.VDQSSESTNPTEAELSSETR.Q
SUOX_MOUSE	MK_SCX_15.6094.6094.2	2	3.76	0.59	1	854.1	42	K.IGELNPEDSMSPSVEASDPYADDPIR.H
SUOX_MOUSE	MK_SCX_17.6418.6418.2	2	4.729	0.53	1	844.7	55.263157	K.AVDDSYNVQPDTVAPIWNLR.G
SUOX_MOUSE	MK_SCX_21.7543.7543.3	3	3.795	0.413	1	1233.3	27.586206	R.LCDSEAHVCFEGLDSDPTGTAYGASIPLAR.A
SUOX_MOUSE	MK_SCX_23.8562.8562.3	3	6.002	0.638	1	1282.4	31.896553	R.INSQRPFNAEPPPELLTEGYITPNPIFFTR.N
SUOX_MOUSE	MK_SCX_51.4448.4448.2	2	3.25	0.387	1	469.9	57.692307	R.NHLPVPNLDPHTYR.L
SURF1_MOUSE	MK_SCX_17.6487.6487.2	2	4.006	0.356	1	525.6	53.333336	R.VMAEPIPLPADPMELK.N
SURF1_MOUSE	MK_SCX_2201.6752.6752.3	3	5.283	0.54	1	1124.6	39.423077	K.ITGADPIFIDADFHSTAPGGPIGGQTR.V
SURF1_MOUSE	MK_SCX_51.3215.3215.3	3	4.705	0.352	1	768.6	45	R.LTENRKPFVPENSPER.N
SVIL_MOUSE	MK_SCX_15.5536.5536.2	2	2.61	0.152	1	387.5	41.666664	R.RGSLELGNPSAAHLGDELK.E
SVIL_MOUSE	MK_SCX_17.4095.4095.2	2	3.405	0.538	1	597.5	55.88235	R.ESAEPGEPDSSTLSLAEK.L
SVIL_MOUSE	MK_SCX_19.3447.3447.2	2	4.684	0.541	1	1271.5	78.57143	K.EFGETTSEQTEVAAR.K
SVIL_MOUSE	MK_SCX_21.5388.5388.2	2	2.29	0.175	1	910.9	65	K.AISTRNRIDVR.Q
SVIL_MOUSE	MK_SCX_24.4034.4034.3	3	3.942	0.456	1	997.4	31.25	K.LSVDNNTSATDYKSPPAENSDSPVR.S
SYAP1_MOUSE	MK_SCX_21.7431.7431.2	2	3.786	0.582	1	1684.2	78.57143	K.GLGNYLYNFASAATK.K

SYAP1_MOUSE	MK_SCX_30.3554.3554.3	3	3.802	0.513	1	498.4	28.947369	K.QSAQLTALAAQQQASGKEEK.S
SYAP1_MOUSE	MK_SCX_30.4149.4149.2	2	2.814	0.41	1	700.9	70.83333	K.SVEEGKIDDILDK.T
SYD_MOUSE	MK_SCX_20_1.3955.3955.2	2	3.023	0.418	1	1199.6	80	R.EIVDAAEDYAK.E
SYD_MOUSE	MK_SCX_2201.5090.5090.2	2	2.062	0.138	1	601.3	72.22222	K.IYVISLAEPR.L
SYD_MOUSE	MK_SCX_24.5839.5839.3	3	3.793	0.353	1	1532.5	43.421055	R.LPLQLDDAIRPEVEGEEDGR.A
SYD_MOUSE	MK_SCX_33.4263.4263.2	2	3.987	0.487	1	1315	67.85714	R.YGISSMIQSQEKPDR.V
SYEP_MOUSE	MK_SCX_29.3757.3757.3	3	5.037	0.436	1	2551.2	45	K.TGQEYKPGNPSAAAVQTVSTK.S
SYEP_MOUSE	MK_SCX_29.3775.3775.2	2	6.173	0.623	1	1358.5	70	K.TGQEYKPGNPSAAAVQTVSTK.S
SYEP_MOUSE	MK_SCX_29.7486.7486.3	3	3.44	0.129	1	1241.3	43.75	K.ASKDQVDSAVQELLQLK.A
SYEP_MOUSE	MK_SCX_29.7587.7587.2	2	4.389	0.582	1	1666.4	71.875	K.ASKDQVDSAVQELLQLK.A
SYEP_MOUSE	MK_SCX_51.3496.3496.3	3	5.63	0.441	1	873.2	40	K.SLTGIEYKPVSATGAEDKDKK.K
SYH_MOUSE	MK_SCX_41.6798.6798.3	3	3.869	0.424	1	1597.2	43.421055	R.HGAEVIDTPVFELKETLTGK.Y
SYLM_MOUSE	MK_SCX_16.6360.6360.2	2	3.356	0.554	1	540.9	42.857143	R.LAQALGLPYSEVIEASPDGTER.L
SYLM_MOUSE	MK_SCX_23.3238.3238.2	2	4.117	0.575	1	1464	81.818184	R.LSGSAEFTGM*TR.Q
SYLM_MOUSE	MK_SCX_23.3839.3839.2	2	3.826	0.612	1	2036.9	90.909096	R.LSGSAEFTGMTR.Q
SYNC_MOUSE	MK_SCX_21.7763.7763.3	3	3.129	0.13	1	424.4	23	-.MVLELYVSDREGNDATGDGTKEKPFK.T
SYNC_MOUSE	MK_SCX_36.6026.6026.3	3	3.756	0.485	1	643.2	30.681818	K.ALMTVGKEPFPTIYVDSQKENER.W
SYNG_MOUSE	MK_SCX_26.5114.5114.3	3	3.051	0.174	1	562.1	34.72222	R.ELEQTTDSKPLGESFAEFR.S
SYNG_MOUSE	MK_SCX_31.3739.3739.3	3	5.139	0.627	1	1666.1	38.095238	K.SVSTRPQPAGSAAASAALASTK.T
SYNJ1_MOUSE	MK_SCX_23.3883.3883.3	3	5.627	0.619	1	846	27.67857	R.TPGPPSSQGSPVDTQPAAQKDSSQTLEPK.R
SYNP2_MOUSE	MK_SCX_14.7480.7480.3	3	4.29	0.461	1	441.4	22.857143	R.GVSSPVAGPAQPPPWPQPAPWSQPAFYDSSEQIASR.D
SYNPO_MOUSE	MK_SCX_14.6530.6530.3	3	4.862	0.58	1	835.3	28.787878	R.SSPGLYTAPVQDSLQPTAVSPTYSSDISPVSPSR.A
SYNPO_MOUSE	MK_SCX_14.6543.6543.2	2	4.01	0.605	1	425.5	31.818182	R.SSPGLYTAPVQDSLQPTAVSPTYSSDISPVSPSR.A
SYNPO_MOUSE	MK_SCX_17.6320.6320.2	2	4.43	0.507	1	577.4	71.875	K.VTPNPDLLDLVQTADEK.R
SYNPO_MOUSE	MK_SCX_18.6123.6123.2	2	3.793	0.318	1	610.8	57.14286	K.VASEEEEVPLVVYLK.E
SYR_MOUSE	MK_SCX_17.5730.5730.2	2	4.124	0.573	1	779.9	55.263157	R.AAYPDLENPPLIVTPSQQPK.F
SYS_MOUSE	MK_SCX_14.9793.9793.2	2	4.45	0.565	1	1359.6	46.296295	K.EAVGDDESVPENVLNFDDLTADALAALK.V
SYS_MOUSE	MK_SCX_14.9816.9816.3	3	3.564	0.452	1	362	23.148148	K.EAVGDDESVPENVLNFDDLTADALAALK.V
SYS_MOUSE	MK_SCX_21.9948.9948.3	3	4.876	0.557	1	941.7	31.25	K.KEAVGDDESVPENVLNFDDLTADALAALK.V
SYS_MOUSE	MK_SCX_2201.6625.6625.2	2	3.205	0.363	1	689.2	92.85714	-.VLDLDLFR.V
SYS_MOUSE	MK_SCX_29.7531.7531.3	3	3.009	0.126	1	642.3	34.72222	R.KEVMQEVAQLSQFDEELYK.V
SYS_MOUSE	MK_SCX_30.3591.3591.2	2	2.045	0.168	1	307.8	55.555557	R.CRFRADNLNK.L
SYS_MOUSE	MK_SCX_35.7219.7219.3	3	3.42	0.157	1	905.5	52.272724	R.FKDPGLVDQLVK.A
SYS_MOUSE	MK_SCX_54.4510.4510.3	3	4.068	0.221	1	1899	56.25	K.RFKDPGLVDQLVK.A
SYSM_MOUSE	MK_SCX_26.4306.4306.3	3	4.504	0.432	1	1277.1	40	R.ALLANQDSDQVQKDPQYQGLR.A
SYSM_MOUSE	MK_SCX_29.3257.3257.3	3	4.763	0.473	1	1791.2	50	R.LPNQTHPDTPVGDESQAR.V
SYSM_MOUSE	MK_SCX_46.8071.8071.3	3	3.827	0.482	1	1128.8	39.473686	R.KGELRPADLPAIISTWQELR.Q
SYUG_MOUSE	MK_SCX_38.3333.3333.3	3	5.512	0.525	1	1055.6	40.909092	R.KEDLEPPAQDQEAKEQEENEEAK.S
T10_MOUSE	MK_SCX_45.7503.7503.3	3	5.226	0.467	1	2133.3	42.5	R.GRGELVSHFLTSDMDSLSYLK.K
T10_MOUSE	MK_SCX_49.4227.4227.3	3	3.307	0.395	1	452.5	36.666668	R.LILAANRDEFYNRPSK.L
T103_MOUSE	MK_SCX_2201.4363.4363.2	2	3.798	0.437	1	1657.8	83.33333	K.YTQGGLENLELSR.K
T103_MOUSE	MK_SCX_23.4396.4396.2	2	3.258	0.443	1	1392.1	93.75	R.YDVTWEEMR.D
T103_MOUSE	MK_SCX_26.3903.3903.2	2	3.356	0.419	1	987.5	75	K.YASWAANQINR.A
T22D4_MOUSE	MK_SCX_19.3705.3705.2	2	3.796	0.458	1	608.7	56.25	R.VEVESGGSAAATPPLSR.R
TACC1_MOUSE	MK_SCX_2201.4393.4393.2	2	3.267	0.156	1	1692.3	85	K.TIAQMIEDEQR.T
TACC2_MOUSE	MK_SCX_21.7756.7756.3	3	6.374	0.564	1	1593.1	39.423077	R.IGSTEVEKPPGLLFQQPDLDSALQVAR.A
TADBP_MOUSE	MK_SCX_2201.3161.3161.2	2	2.289	0.156	1	913.3	87.5	R.FTEYETQVK.V
TADBP_MOUSE	MK_SCX_24.4161.4161.2	2	4.957	0.465	1	2072	82.35294	R.FGGNPGGFGNQGGFGNSR.G
TADBP_MOUSE	MK_SCX_24.4180.4180.3	3	4.589	0.473	1	1646.4	48.52941	R.FGGNPGGFGNQGGFGNSR.G
TADBP_MOUSE	MK_SCX_24.9940.9940.3	3	4.231	0.532	1	362.4	28.260868	R.LVEGILHAPDAGWGNLVYVVNYPK.D
TAF10_MOUSE	MK_SCX_18.4328.4328.2	2	6.611	0.651	1	1783.9	58.333332	K.ASPAGTAGGPVAGVATAGTGPVAAR.A

TAGL_MOUSE	MK_SCX_19.5181.5181.2	2	3.947	0.491	1	2181.8	79.16667	K.TDMFQTVDLYEGK.D
TAGL_MOUSE	MK_SCX_21.4676.4676.2	2	4.336	0.154	1	1765.6	90.909096	R.TLM*ALGSLAVTK.N
TAGL_MOUSE	MK_SCX_25.6247.6247.3	3	5.202	0.586	1	1613.1	47.368423	K.TDMFQTVDLYEGKDMAAVQR.T
TAGL_MOUSE	MK_SCX_25.6250.6250.2	2	5.674	0.631	1	2408.1	73.68421	K.TDMFQTVDLYEGKDMAAVQR.T
TAGL_MOUSE	MK_SCX_35.9171.9171.3	3	4.626	0.533	1	461.1	28.125	K.LVNSLYPEGSKPVKVPENPPSMVFK.Q
TAGL2_MOUSE	MK_SCX_17.6552.6552.2	2	4.976	0.46	1	1623.6	76.92308	R.DDGLFSGDPNWFPK.K
TAGL2_MOUSE	MK_SCX_18.8638.8638.2	2	6.178	0.625	1	2406.4	76.47059	R.YGINTTDIFQTVDLWEGK.N
TAGL2_MOUSE	MK_SCX_20_1.4587.4587.2	2	3.671	0.494	1	1116.2	73.07692	K.LINSLYPEGQAPVK.K
TAGL2_MOUSE	MK_SCX_21.5608.5608.2	2	4.278	0.454	1	1277	76.92308	K.QM*EQISQFLQAAER.Y
TAGL2_MOUSE	MK_SCX_21.6874.6874.2	2	5.351	0.496	1	1925.3	84.61539	K.QMEQISQFLQAAER.Y
TAGL2_MOUSE	MK_SCX_21.6883.6883.3	3	3.474	0.377	1	953.4	48.076923	K.QMEQISQFLQAAER.Y
TAGL2_MOUSE	MK_SCX_2201.4408.4408.2	2	4.212	0.214	1	1861.1	95.454544	R.TLM*NLGGLAVAR.D
TAGL2_MOUSE	MK_SCX_2201.5426.5426.3	3	3.733	0.293	1	1910.3	61.363636	R.TLMNLGGLAVAR.D
TAGL2_MOUSE	MK_SCX_2201.5470.5470.2	2	4.253	0.368	1	1896.9	95.454544	R.TLMNLGGLAVAR.D
TAGL2_MOUSE	MK_SCX_27.6318.6318.2	2	4.426	0.435	1	619	60.714287	R.DDGLFSGDPNWFPKK.S
TAGL2_MOUSE	MK_SCX_32.3871.3871.2	2	4.122	0.462	1	860	67.85714	K.LINSLYPEGQAPVKK.I
TALDO_MOUSE	MK_SCX_2201.2870.2870.2	2	2.042	0.448	1	311.8	55	K.SYEPQEDPGVK.S
TALDO_MOUSE	MK_SCX_32.4119.4119.2	2	4.03	0.412	1	1268.2	64.28571	K.LGGPQEEQIKNAIDK.L
TALDO_MOUSE	MK_SCX_35.3923.3923.3	3	3.463	0.326	1	667.9	57.5	R.LSFDKDAMVAR.A
TALDO_MOUSE	MK_SCX_35.3934.3934.2	2	4.07	0.324	1	1291.7	85	R.LSFDKDAMVAR.A
TALDO_MOUSE	MK_SCX_35.4127.4127.2	2	2.146	0.284	1	300.8	70	K.LLGELLKDNSK.L
TALDO_MOUSE	MK_SCX_39.3137.3137.3	3	3.9	0.321	1	536.9	47.727272	R.WLHNEDQM*AVEK.L
TALDO_MOUSE	MK_SCX_39.3527.3527.2	2	4.583	0.44	1	1375.5	86.36364	R.WLHNEDQMAVEK.L
TALDO_MOUSE	MK_SCX_39.3547.3547.3	3	3.84	0.229	1	917.6	50	R.WLHNEDQMAVEK.L
TALDO_MOUSE	MK_SCX_41.3700.3700.2	2	3.553	0.238	1	590.3	75	R.QRMESALDQLK.Q
TALDO_MOUSE	MK_SCX_50.3784.3784.3	3	3.921	0.519	1	825.5	53.846157	R.LIELYKEAGVGKDR.I
TALDO_MOUSE	MK_SCX_51.3770.3770.3	3	5.373	0.516	1	1175.6	55	K.KLGGPQEEQIKNAIDK.L
TAOK3_MOUSE	MK_SCX_46.3353.3353.3	3	4.785	0.552	1	918.1	44.11765	K.LQKEVETHANNSSIELEK.L
TARA_MOUSE	MK_SCX_56.4065.4065.3	3	4.038	0.474	1	1423.4	41.666664	R.RGPTSDSHEALEKEVQSLR.A
TAU_MOUSE	MK_SCX_16.3533.3533.2	2	3.251	0.561	1	331.7	47.727272	K.ESPPQPPADDGAEEPGSETSDAK.S
TAU_MOUSE	MK_SCX_17.4390.4390.2	2	4.391	0.529	1	728.5	61.764706	K.EQDLEGATVVGVPGEEQK.A
TAU_MOUSE	MK_SCX_18.4668.4668.2	2	4.239	0.479	1	962.7	60.000004	K.STPTAEDVTAPLVDER.A
TAU_MOUSE	MK_SCX_20_1.4233.4233.2	2	3.359	0.453	1	469.5	63.636364	R.LQTAPVPM*PDLK.N
TAU_MOUSE	MK_SCX_20_1.5248.5248.2	2	3.533	0.37	1	525.1	63.636364	R.LQTAPVPMPDLK.N
TAU_MOUSE	MK_SCX_24.11839.11839.3	3	5.216	0.514	1	836.4	27.419355	R.HLSNVSSTGSIDMVDSPQLATLADEVSASLAK.Q
TAU_MOUSE	MK_SCX_26.4392.4392.3	3	3.673	0.554	1	542	32.954548	K.ASTPKEQDLEGATVVGVPGEEQK.A
TAU_MOUSE	MK_SCX_35.3662.3662.3	3	3.182	0.39	1	376.9	33.333336	R.TPSLPTPPTREPK.K
TAU_MOUSE	MK_SCX_35.3696.3696.2	2	2.553	0.259	1	508.8	62.5	R.TPSLPTPPTREPK.K
TB182_MOUSE	MK_SCX_14.3195.3195.2	2	2.716	0.36	1	499.9	61.538464	R.NMAPGAGCSPGEPR.E
TB182_MOUSE	MK_SCX_18.3098.3098.2	2	5.316	0.568	1	1504.5	66.66667	R.ASVSTNQDTDENDQELGM*K.N
TB182_MOUSE	MK_SCX_18.3528.3528.2	2	5.987	0.635	1	2483.9	75	R.ASVSTNQDTDENDQELGMK.N
TB182_MOUSE	MK_SCX_28.4321.4321.3	3	5.038	0.607	1	2211	45	R.APAIRPGGTLGLSETADSDTR.L
TB182_MOUSE	MK_SCX_32.3340.3340.3	3	3.394	0.196	1	521.9	32.894737	K.RASVSTNQDTDENDQELGMK.N
TBA1_MOUSE	MK_SCX_16.8019.8019.2	2	5.874	0.639	1	1426.6	62.5	R.FDGALNVDLTEFQTNLVPYPR.I
TBA1_MOUSE	MK_SCX_16.8039.8039.3	3	4.177	0.506	1	782.4	37.5	R.FDGALNVDLTEFQTNLVPYPR.I
TBA1_MOUSE	MK_SCX_18.5256.5256.2	2	4.344	0.417	1	937.3	70.588234	K.VGINYQPPTVVPGGDLAK.V
TBA1_MOUSE	MK_SCX_18.6062.6062.2	2	5.89	0.56	1	1208.7	63.15789	K.TIGGGDDSFNTFFSETGAGK.H
TBA1_MOUSE	MK_SCX_18.6878.6878.2	2	5.361	0.407	1	1491.4	85.71429	R.AVFVDLEPTVIDEVR.T
TBA1_MOUSE	MK_SCX_32.6567.6567.2	2	4.592	0.604	1	1004	73.333336	R.IHFPLATYAPVISAEK.A
TBA1_MOUSE	MK_SCX_32.6815.6815.3	3	4.511	0.48	1	1285.7	51.666664	R.IHFPLATYAPVISAEK.A
TBA1_MOUSE	MK_SCX_34.4277.4277.2	2	3.508	0.27	1	457.1	61.538464	R.NLDIERPTYTNLNR.L

TBA1_MOUSE	MK_SCX_43.5388.5388.2	2	3.255	0.575	1	455.1	47.5	R.QLFHPEQLITGKEDAANNYAR.G
TBA1_MOUSE	MK_SCX_43.5413.5413.3	3	4.278	0.561	1	743	33.75	R.QLFHPEQLITGKEDAANNYAR.G
TBA1_MOUSE	MK_SCX_43.5957.5957.2	2	4.223	0.526	1	910.1	43.47826	K.TIGGGDDSFNTFFSETGAGKHVPR.A
TBA1_MOUSE	MK_SCX_52.3766.3766.3	3	4.232	0.379	1	1590.4	62.5	R.LDHKFDLMYAK.R
TBA2_MOUSE	MK_SCX_13.7057.7057.2	2	2.867	0.341	1	625	66.66667	R.SIQFVDWCPTGFK.V
TBA2_MOUSE	MK_SCX_20_1.10924.10924.2	2	3.255	0.437	1	648.3	61.538464	R.LISQIVSSITASLR.F
TBA4_MOUSE	MK_SCX_13.6953.6953.2	2	3.633	0.364	1	369.2	52.63158	K.TIGGGDDSFTTFFCETGAGK.H
TBA4_MOUSE	MK_SCX_17.6761.6761.2	2	4.354	0.455	1	1849.6	85.71429	R.AVFVDLEPTVIDEIR.N
TBA4_MOUSE	MK_SCX_49.5439.5439.3	3	4.518	0.383	1	1223.9	53.333336	R.GHYTIGKEIIDPVLDR.I
TBA4_MOUSE	MK_SCX_49.5602.5602.2	2	5.006	0.393	1	1334.7	76.666664	R.GHYTIGKEIIDPVLDR.I
TBB2C_MOUSE	MK_SCX_18.4722.4722.2	2	4.523	0.426	1	908.2	78.57143	R.AVLVDLEPGTM*DSVR.S
TBB2C_MOUSE	MK_SCX_19.5082.5082.2	2	4.588	0.517	1	823.3	75	R.ALTVPELTQQM*FDAK.N
TBB2C_MOUSE	MK_SCX_19.5125.5125.2	2	4.508	0.402	1	931.1	75	R.AVLVDLEPGTMDSVR.S
TBB2C_MOUSE	MK_SCX_19.6493.6493.2	2	4.61	0.529	1	1038.8	82.14286	R.ALTVPELTQQMFDAK.N
TBB2C_MOUSE	MK_SCX_20_1.3842.3842.2	2	4.24	0.216	1	1629.8	81.818184	K.EVDEQMLNVQNK.N
TBB2C_MOUSE	MK_SCX_21.6797.6797.2	2	4.581	0.392	1	1069.2	76.92308	K.NSSYFVEWIPNNVK.T
TBB2C_MOUSE	MK_SCX_2201.3655.3655.2	2	3.977	0.369	1	1348.8	90.909096	R.INVYYNEATGGK.Y
TBB2C_MOUSE	MK_SCX_2201.4515.4515.2	2	3.907	0.4	1	1147.2	88.88889	K.LAVNM*VPFPR.L
TBB2C_MOUSE	MK_SCX_2201.5576.5576.2	2	3.634	0.485	1	963.5	94.44444	K.LAVNMVPFPR.L
TBB2C_MOUSE	MK_SCX_2201.7152.7152.3	3	6.166	0.657	1	1015.8	28.225807	K.EAESCDCLQGFQLTHSLGGGTGSGM*GTLLISK.I
TBB2C_MOUSE	MK_SCX_2201.7806.7806.3	3	4.952	0.541	1	948.4	28.225807	K.EAESCDCLQGFQLTHSLGGGTGSGMGTLLISK.I
TBB2C_MOUSE	MK_SCX_2201.7840.7840.2	2	4.181	0.606	1	441.3	32.258064	K.EAESCDCLQGFQLTHSLGGGTGSGMGTLLISK.I
TBB2C_MOUSE	MK_SCX_23.4418.4418.2	2	3.379	0.505	1	1226.2	88.88889	R.ISEQFTAM*FR.R
TBB2C_MOUSE	MK_SCX_23.5483.5483.2	2	3.948	0.471	1	2026.2	94.44444	R.ISEQFTAMFR.R
TBB2C_MOUSE	MK_SCX_24.4298.4298.2	2	2.651	0.357	1	801.9	88.88889	R.FPGQLNADLR.K
TBB2C_MOUSE	MK_SCX_24.5076.5076.2	2	2.031	0.207	1	580.3	87.5	R.YLTVAAVFR.G
TBB2C_MOUSE	MK_SCX_28.7967.7967.3	3	8.004	0.629	1	4187.1	49	R.SGPFGQIFRPDNFVFGQSGAGNNWAK.G
TBB2C_MOUSE	MK_SCX_28.8129.8129.2	2	5.785	0.582	1	1060.8	46	R.SGPFGQIFRPDNFVFGQSGAGNNWAK.G
TBB2C_MOUSE	MK_SCX_30.6636.6636.2	2	5.009	0.577	1	544	42.307693	K.FWEVISDEHGIDPTGTYHGDSDLQLER.I
TBB2C_MOUSE	MK_SCX_30.6739.6739.3	3	6.535	0.579	1	1174.8	37.5	K.FWEVISDEHGIDPTGTYHGDSDLQLER.I
TBB2C_MOUSE	MK_SCX_31.8070.8070.2	2	7.275	0.592	1	3788.4	85.29411	K.GHYTEGAELVDSVLDVVR.K
TBB2C_MOUSE	MK_SCX_31.8330.8330.3	3	5.175	0.463	1	1569.6	44.11765	K.GHYTEGAELVDSVLDVVR.K
TBB2C_MOUSE	MK_SCX_33.4969.4969.3	3	3.966	0.228	1	1087.4	41.666664	R.MSMKEVDEQMLNVQNK.N
TBB2C_MOUSE	MK_SCX_34.4344.4344.2	2	4.926	0.627	1	2916.6	86.666664	R.INVYYNEATGGKYVPR.A
TBB2C_MOUSE	MK_SCX_34.6713.6713.3	3	7.064	0.613	1	1526.6	37.5	R.KEAESCDCLQGFQLTHSLGGGTGSGM*GTLLISK.I
TBB2C_MOUSE	MK_SCX_34.7588.7588.3	3	7.161	0.613	1	2486.2	36.71875	R.KEAESCDCLQGFQLTHSLGGGTGSGMGTLLISK.I
TBB2C_MOUSE	MK_SCX_36.6654.6654.3	3	3.219	0.22	1	913.4	50	R.LHFFM*PGFAPLTSR.G
TBB2C_MOUSE	MK_SCX_36.6831.6831.2	2	3.66	0.574	1	1707.9	84.61539	R.LHFFM*PGFAPLTSR.G
TBB2C_MOUSE	MK_SCX_36.7918.7918.2	2	3.911	0.458	1	1320.1	84.61539	R.LHFFMPGFAPLTSR.G
TBB2C_MOUSE	MK_SCX_36.7950.7950.3	3	4.049	0.468	1	1429.1	57.692307	R.LHFFMPGFAPLTSR.G
TBB2C_MOUSE	MK_SCX_40.4451.4451.2	2	3.738	0.475	1	812.8	80	R.KLAVNM*VPFPR.L
TBB2C_MOUSE	MK_SCX_41.5106.5106.2	2	3.866	0.441	1	1112	80	R.KLAVNMVPFPR.L
TBB2C_MOUSE	MK_SCX_44.7333.7333.2	2	6.937	0.606	1	3026	77.77778	K.GHYTEGAELVDSVLDVVRK.E
TBB2C_MOUSE	MK_SCX_44.7387.7387.3	3	5.473	0.452	1	1361.7	52.77778	K.GHYTEGAELVDSVLDVVRK.E
TBB3_MOUSE	MK_SCX_18.5015.5015.2	2	4.893	0.553	1	986.5	78.57143	R.AILVDLEPGTM*DSVR.S
TBB3_MOUSE	MK_SCX_18.5624.5624.2	2	5.117	0.523	1	1000.2	78.57143	R.AILVDLEPGTMDSVR.S
TBB5_MOUSE	MK_SCX_18.6599.6599.2	2	3.809	0.569	1	665.7	71.42857	R.ALTVPELTQQVFDAK.N
TBB5_MOUSE	MK_SCX_2201.3692.3692.2	2	3.637	0.564	1	1302.2	86.36364	R.ISVYYNEATGGK.Y
TBB5_MOUSE	MK_SCX_30.6773.6773.3	3	5.759	0.498	1	730.7	30.769232	K.FWEVISDEHGIDPTGTYHGDSDLQLDR.I
TBB6_MOUSE	MK_SCX_21.4876.4876.2	2	4.663	0.447	1	2211.1	90.909096	R.IMNTFSVMPSPK.V
TBB6_MOUSE	MK_SCX_23.5792.5792.2	2	2.389	0.242	1	525.5	72.22222	R.ISEQFSAM*FR.R

TBC13_MOUSE	MK_SCX_25.4329.4329.3	3	3.372	0.461	1	1106.5	40.625	R.EDVTFEDHPLNPNPDSR.W
TBC13_MOUSE	MK_SCX_25.7121.7121.3	3	3.427	0.363	1	415.4	37.5	R.IADFQDVLKEPSIVLEK.L
TBCD1_MOUSE	MK_SCX_25.4244.4244.3	3	3.859	0.441	1	688.1	33.695652	R.GLQDHSASVDLDSSTSSTLSNTSK.E
TBCD1_MOUSE	MK_SCX_31.4528.4528.3	3	3.003	0.468	1	758.8	45	K.APAQLCEGCPLQGLHK.L
TBL1R_MOUSE	MK_SCX_32.3446.3446.3	3	5.119	0.499	1	1725.3	39.772728	K.LAQQHAAAAAAAAAATNQQGSAK.N
TCEA1_MOUSE	MK_SCX_19.4700.4700.2	2	2.583	0.187	1	677.8	52.941177	R.SADEPMTTFVVCNECGNR.W
TCEA1_MOUSE	MK_SCX_21.4405.4405.2	2	2.086	0.133	1	896.8	77.27273	R.MTAEEMASDELK.E
TCO2_MOUSE	MK_SCX_2201.7399.7399.3	3	5.357	0.626	1	1246.9	38.04348	R.APDTPLLQGIADYKPQDGETIELR.L
TCOF_MOUSE	MK_SCX_45.7814.7814.3	3	4.086	0.438	1	855.4	43.75	R.ELLPLIYHHLLQAGYVR.A
TCP4_MOUSE	MK_SCX_33.4209.4209.2	2	3.218	0.136	1	1116.9	65.38461	R.EYWMDSEGEMKPGR.K
TCPA1_MOUSE	MK_SCX_16.7460.7460.2	2	4.111	0.668	1	955.9	54.545456	R.SLLVIPNTLAVNAAQDSTDLVAK.L
TCPA1_MOUSE	MK_SCX_23.3863.3863.2	2	2.876	0.244	1	1093.4	85	K.YFVEAGAM*AVR.R
TCPB_MOUSE	MK_SCX_26.6762.6762.3	3	4.106	0.502	1	975.2	41.666664	K.LGGSLADSYLDEGFLLDKK.I
TCPB_MOUSE	MK_SCX_28.3786.3786.2	2	4.318	0.503	1	2308.9	80	R.EALLSSAVDHGSDEAR.F
TCPB_MOUSE	MK_SCX_30.4272.4272.2	2	3.799	0.507	1	1662.8	76.92308	K.ILIANTGMDTDKIK.I
TCPB_MOUSE	MK_SCX_38.5999.5999.2	2	2.002	0.226	1	306.1	68.75	K.HGINCFINR.Q
TCPB_MOUSE	MK_SCX_42.6533.6533.3	3	5.235	0.6	1	1593.2	44.736843	K.KLGGSLADSYLDEGFLLDKK.I
TCPD_MOUSE	MK_SCX_15.9138.9138.2	2	5.361	0.635	1	1999	48.214287	R.AFADAMEVIPSTLAENAGLNPISTVTELR.N
TCPD_MOUSE	MK_SCX_15.9178.9178.3	3	5.599	0.595	1	1828.1	37.5	R.AFADAMEVIPSTLAENAGLNPISTVTELR.N
TCPD_MOUSE	MK_SCX_18.6047.6047.2	2	2.911	0.283	1	392.2	47.22222	K.VVSQYSSLLSPM*SVNAVMK.V
TCPD_MOUSE	MK_SCX_18.6633.6633.2	2	5.185	0.603	1	1138.3	61.11111	K.VVSQYSSLLSPMSVNAVMK.V
TCPD_MOUSE	MK_SCX_19.6059.6059.2	2	4.409	0.425	1	1039	73.333336	R.ALIAGGGAPEIELALR.L
TCPD_MOUSE	MK_SCX_36.4175.4175.3	3	4.139	0.347	1	1083.6	47.916664	K.GIHPTIISESFQK.A
TCPD_MOUSE	MK_SCX_36.4187.4187.2	2	3.2	0.455	1	556.5	70.83333	K.GIHPTIISESFQK.A
TCPE_MOUSE	MK_SCX_15.9596.9596.3	3	3.638	0.23	1	302.5	23.214285	R.AFADALEVIPMALSENSGMNPIQTMTEVR.A
TCPE_MOUSE	MK_SCX_15.9695.9695.2	2	3.845	0.667	1	641.8	35.714287	R.AFADALEVIPMALSENSGMNPIQTMTEVR.A
TCPE_MOUSE	MK_SCX_18.5727.5727.2	2	4.195	0.506	1	1669.6	70	K.VLVDINNPEPLIQTAK.T
TCPE_MOUSE	MK_SCX_19.8370.8370.2	2	3.621	0.549	1	578.2	56.25	R.WVGGPEIELIAIATGGR.I
TCPE_MOUSE	MK_SCX_45.3641.3641.3	3	4.266	0.481	1	1105.9	46.42857	K.GVIVDKDFSHPQM*PK.K
TCPE_MOUSE	MK_SCX_45.4374.4374.3	3	4.085	0.335	1	977.2	46.42857	K.GVIVDKDFSHPQMPK.K
TCPE_MOUSE	MK_SCX_45.4380.4380.2	2	3.732	0.472	1	1253.5	67.85714	K.GVIVDKDFSHPQMPK.K
TCPG_MOUSE	MK_SCX_16.9793.9793.2	2	2.647	0.235	1	580.5	50	K.M*LLDPM*GGIVM*TNDGNAILR.E
TCPG_MOUSE	MK_SCX_23.4356.4356.2	2	3.041	0.412	1	750.8	75	K.AMTGVEQWPYR.A
TCPG_MOUSE	MK_SCX_38.3788.3788.2	2	3.66	0.49	1	1380.3	81.818184	R.GASKEILSEVER.N
TCPG_MOUSE	MK_SCX_47.4323.4323.3	3	3.7	0.496	1	767.9	39.705883	K.KGESQTDIEITREEDFTR.I
TCPQ_MOUSE	MK_SCX_20_1.3649.3649.2	2	3.143	0.205	1	839.6	72.72727	R.GSTDNLM*DDIER.A
TCPQ_MOUSE	MK_SCX_21.4072.4072.2	2	3.762	0.453	1	516.4	75	K.AIAGTGANVIVTGGK.V
TCPQ_MOUSE	MK_SCX_2201.4050.4050.2	2	3.19	0.335	1	800.4	85	K.LATNAAVTVLR.V
TCPQ_MOUSE	MK_SCX_2201.4670.4670.2	2	4.241	0.422	1	1566.6	94.44444	K.FAEAFEAIPR.A
TCPQ_MOUSE	MK_SCX_26.6101.6101.3	3	3.753	0.353	1	1165.9	37.5	K.TAEELMNFSKGEENLMDAQVK.A
TCPQ_MOUSE	MK_SCX_43.4185.4185.3	3	3.004	0.127	1	1137.1	50	K.HFSGLEEAVYR.N
TCPQ_MOUSE	MK_SCX_43.4186.4186.2	2	2.652	0.558	1	952.3	80	K.HFSGLEEAVYR.N
TCPW_MOUSE	MK_SCX_2201.3527.3527.2	2	2.83	0.143	1	1104.8	88.88889	K.MLVSGAGDIK.L
TCPW_MOUSE	MK_SCX_34.6149.6149.3	3	5.604	0.484	1	1248.3	40	K.DGNVLLHEMQIQHPTASIIAK.V
TCPZ_MOUSE	MK_SCX_15.7739.7739.2	2	3.91	0.529	1	369.5	35.416664	K.VATAQDDITGDGTTSNVLIIGELLK.Q
TCPZ_MOUSE	MK_SCX_18.14292.14292.2	2	2.755	0.25	1	320.1	43.75	R.AQLGVQAFADALLIIPK.V
TCPZ_MOUSE	MK_SCX_19.5182.5182.2	2	4.286	0.533	1	1004.7	70	K.VLAQNSGFDLQETLVK.V
TCPZ_MOUSE	MK_SCX_19.6080.6080.2	2	2.875	0.356	1	590.4	68.181816	K.GIDPFSLDALAK.E
TCPZ_MOUSE	MK_SCX_32.4115.4115.2	2	2.64	0.324	1	418.5	63.636364	K.EMDRETLIDVAR.T
TCPZ_MOUSE	MK_SCX_34.6149.6149.3	3	5.604	0.484	1	1248.3	40	K.DGNVLLHEMQIQHPTASIIAK.V
TCTP_MOUSE	MK_SCX_25.6402.6402.2	2	5.121	0.534	1	2136.9	80.769226	R.DLISHDELFSDIYK.I

TCTP_MOUSE	MK_SCX_25.6562.6562.3	3	3.086	0.151	1	914	48.076923	R.DLISHDELFSDIYK.I
TCTP_MOUSE	MK_SCX_37.3909.3909.2	2	2.847	0.354	1	510.6	75	R.VKPFMTGAAEQIK.H
TCTP_MOUSE	MK_SCX_37.7759.7759.2	2	2.507	0.357	1	555.3	56.666668	R.DLISHDELFSDIYKIR.E
TCTP_MOUSE	MK_SCX_37.7950.7950.3	3	4.261	0.536	1	1733	51.666664	R.DLISHDELFSDIYKIR.E
TCTP_MOUSE	MK_SCX_38.3864.3864.3	3	4.121	0.231	1	1100.2	56.25	R.VKPFMTGAAEQIK.H
TCTP_MOUSE	MK_SCX_58.6977.6977.3	3	3.554	0.24	1	706.2	45.833336	K.SLKGKLEEQKPER.V
TE2IP_MOUSE	MK_SCX_19.8616.8616.2	2	2.843	0.185	1	616.7	61.538464	K.FNLDLSTVTQALLK.N
TE2IP_MOUSE	MK_SCX_21.4582.4582.3	3	5.5	0.609	1	878.2	32.75862	R.LGLTEQASDPKPGASTEGSTEPEPQPLTGR.I
TERA_MOUSE	MK_SCX_17.4707.4707.2	2	3.578	0.49	1	601.2	50	R.QTNPSAM*EVEEDDPVPEIR.R
TERA_MOUSE	MK_SCX_17.5083.5083.2	2	4.448	0.552	1	1193.2	55.555557	R.QTNPSAMEVEEDDPVPEIR.R
TERA_MOUSE	MK_SCX_18.9356.9356.2	2	4.547	0.624	1	1257.4	75	K.NAPAIIFIDELDAIAPK.R
TERA_MOUSE	MK_SCX_19.7768.7768.2	2	2.057	0.277	1	311.8	46.875	K.GPELLTM*WFGESEANVR.E
TERA_MOUSE	MK_SCX_20_1.6506.6506.2	2	4.548	0.521	1	1823.7	87.5	R.IVSQLLTLM*DGLK.Q
TERA_MOUSE	MK_SCX_24.4373.4373.2	2	3.903	0.469	1	1677.1	86.36364	R.WALSQSNPSALR.E
TERA_MOUSE	MK_SCX_31.4904.4904.2	2	3.093	0.239	1	782.7	57.14286	R.FDREVDIGIPDATGR.L
TERA_MOUSE	MK_SCX_31.7909.7909.3	3	3.233	0.367	1	444.5	44.642857	R.IVSQLLTLMDGLKQR.A
TERA_MOUSE	MK_SCX_48.5681.5681.2	2	4.867	0.581	1	1207.8	76.92308	K.EMVELPLRHPALFK.A
TF3C1_MOUSE	MK_SCX_13.10518.10518.2	2	2.44	0.14	1	527.8	57.692307	K.RPHCPETDAEEATR.L
TF3C1_MOUSE	MK_SCX_19.9121.9121.2	2	2.327	0.152	1	341.6	50	K.RRNLIIEAVTNLR.L
TFAM_MOUSE	MK_SCX_23.3414.3414.2	2	2.552	0.232	1	438.4	70	K.SWEEQM*AEVGR.S
TFAM_MOUSE	MK_SCX_23.4354.4354.2	2	3.934	0.425	1	1660.5	80	K.SWEEQMAEVGR.S
TFAM_MOUSE	MK_SCX_35.3621.3621.3	3	4.87	0.564	1	1539.3	55.35714	K.YKEQLTPSQLM*GM*EK.E
TFAM_MOUSE	MK_SCX_35.4412.4412.3	3	4.056	0.135	1	1065.5	46.42857	K.YKEQLTPSQLMGM*EK.E
TFAM_MOUSE	MK_SCX_35.5457.5457.3	3	4.508	0.453	1	673.2	42.857143	K.YKEQLTPSQLMGMEK.E
TFAM_MOUSE	MK_SCX_35.5460.5460.2	2	4.87	0.363	1	1619.2	85.71429	K.YKEQLTPSQLMGMEK.E
TFR1_MOUSE	MK_SCX_21.7269.7269.2	2	3.81	0.522	1	968.8	63.333332	R.SAFSNLFGGEPLSYTR.F
TFR1_MOUSE	MK_SCX_23.3484.3484.3	3	3.866	0.508	1	625.9	31.818182	K.LAETEETDKSETM*ETEDVPTSSR.L
TGFB1_MOUSE	MK_SCX_17.3903.3903.2	2	4.272	0.568	1	722.2	58.333332	R.VAGESADPEPEPEADYYAK.E
TGFR2_MOUSE	MK_SCX_16.5612.5612.2	2	3.326	0.433	1	538.2	50	R.LDPTLSVDDLANSGQVGTAR.Y
TGM2_MOUSE	MK_SCX_18.4747.4747.2	2	3.983	0.42	1	957.4	67.85714	K.SVEVSDPVPAGDLVK.A
TGM2_MOUSE	MK_SCX_19.6066.6066.2	2	4.467	0.606	1	1144.8	76.666664	R.GLLIEPAANSYLLAER.D
TGM2_MOUSE	MK_SCX_24.4558.4558.2	2	2.85	0.437	1	787	85.71429	R.LTLYFEGR.G
TGM2_MOUSE	MK_SCX_35.2904.2904.3	3	4.095	0.436	1	866.5	56.81818	K.LAEKEETGVAMR.I
TGM2_MOUSE	MK_SCX_36.2983.2983.2	2	2.32	0.253	1	415.9	54.545456	K.LAEKEETGVAMR.I
TGM2_MOUSE	MK_SCX_48.3696.3696.3	3	5.22	0.446	1	1226.1	43.421055	R.DDREDITHTYKYPEGSPEER.E
TGM2_MOUSE	MK_SCX_48.5253.5253.2	2	3.83	0.557	1	1340.1	80.769226	K.ARVDLFPTDIGLHK.L
THEM2_MOUSE	MK_SCX_18.4358.4358.2	2	5.285	0.616	1	652.3	67.64706	R.GAPGVSVDM*NITYM*SPAK.I
THEM2_MOUSE	MK_SCX_18.4734.4734.2	2	5.313	0.253	1	817.8	73.52941	R.GAPGVSVDM*NITYMSPAK.I
THEM2_MOUSE	MK_SCX_18.6482.6482.2	2	3.813	0.414	1	585.9	58.823532	R.GAPGVSVDMNITYMSPAK.I
THEM2_MOUSE	MK_SCX_21.5375.5375.2	2	3.212	0.438	1	1218.7	86.36364	K.TLAFASVDLTNK.T
THEM2_MOUSE	MK_SCX_30.4560.4560.2	2	4.309	0.552	1	2339.6	80	K.TLAFASVDLTNKTTGK.L
THEM2_MOUSE	MK_SCX_40.5054.5054.2	2	2.909	0.36	1	575.2	83.33333	K.VM*FKVPGFDR.V
THEM2_MOUSE	MK_SCX_40.5662.5662.2	2	2.719	0.419	1	917.7	94.44444	K.VMFKVPGFDR.V
THIC_MOUSE	MK_SCX_17.6424.6424.2	2	4.187	0.563	1	1090.3	54.545456	R.IVSWSQAGVEPSVM*GVGPIPAIK.Q
THIC_MOUSE	MK_SCX_17.6743.6743.2	2	4.985	0.6	1	1462.5	59.090908	R.IVSWSQAGVEPSVMGVGPIPAIK.Q
THIC_MOUSE	MK_SCX_23.6584.6584.3	3	4.329	0.536	1	1041.3	38.04348	R.TAIGSFNGALSTVPVHEMGTTVIK.E
THIC_MOUSE	MK_SCX_23.6587.6587.2	2	2.606	0.207	1	528	39.130436	R.TAIGSFNGALSTVPVHEMGTTVIK.E
THIC_MOUSE	MK_SCX_23.6741.6741.3	3	6.249	0.238	1	1785.7	36.290325	K.LKPYFLTDGTGTVTPANASGMNDGAAAVVLM*K.K
THIC_MOUSE	MK_SCX_23.7227.7227.3	3	6.036	0.563	1	1893	34.677418	K.LKPYFLTDGTGTVTPANASGMNDGAAAVVLMK.K
THIC_MOUSE	MK_SCX_48.4763.4763.2	2	5.423	0.563	1	2584.6	80	K.AGHFDKEIVPVLVSSR.K
THIC_MOUSE	MK_SCX_49.4957.4957.3	3	3.431	0.4	1	1251.1	46.666668	K.AGHFDKEIVPVLVSSR.K

THIKA_MOUSE	MK_SCX_13.5138.5138.2	2	3.373	0.362	1	765.7	66.66667	R.DCLTPMGMTSENVAER.F
THIKA_MOUSE	MK_SCX_15.5258.5258.2	2	7.143	0.562	1	2453.7	63.04348	K.DGGSTTAGNSSQVSDGAAAVLLAR.R
THIKA_MOUSE	MK_SCX_15.5436.5436.3	3	5.049	0.532	1	1457.1	40.217392	K.DGGSTTAGNSSQVSDGAAAVLLAR.R
THIKA_MOUSE	MK_SCX_15.7307.7307.2	2	4.364	0.711	1	525.5	48	R.SYAVVGVPPDVM*GIGPAYAIPAALQK.A
THIKA_MOUSE	MK_SCX_15.7853.7853.2	2	5.427	0.689	1	724.6	54.000004	R.SYAVVGVPPDVMGIGPAYAIPAALQK.A
THIKA_MOUSE	MK_SCX_15.7866.7866.3	3	3.244	0.443	1	521.9	29	R.SYAVVGVPPDVMGIGPAYAIPAALQK.A
THIKA_MOUSE	MK_SCX_17.11887.11887.2	2	5.489	0.549	1	1733.4	60.526318	K.NTTPDELLSAVLTAVLQDVR.L
THIKA_MOUSE	MK_SCX_18.7063.7063.2	2	5.731	0.488	1	1191.3	75	R.IAQFLSGIPETVPLSTVNR.Q
THIKA_MOUSE	MK_SCX_19.7448.7448.2	2	3.908	0.332	1	1202.2	83.33333	K.AEELGLPILGVLR.S
THIKA_MOUSE	MK_SCX_23.5510.5510.3	3	3.55	0.187	1	598.3	29.347824	R.GGFKNTTPDELLSAVLTAVLQDVR.L
THIKA_MOUSE	MK_SCX_26.4459.4459.2	2	4.803	0.577	1	1138.1	52.499996	K.TITVSQDEGVRPSTTMQGLAK.L
THIKA_MOUSE	MK_SCX_26.6351.6351.3	3	5.239	0.504	1	724.5	35.416664	R.LKPEQLGDISVGNVLEPGAGAVM*AR.I
THIKA_MOUSE	MK_SCX_26.6767.6767.3	3	5.418	0.42	1	1122.6	39.583336	R.LKPEQLGDISVGNVLEPGAGAVMAR.I
THIKA_MOUSE	MK_SCX_26.6776.6776.2	2	6.031	0.624	1	1408.5	56.25	R.LKPEQLGDISVGNVLEPGAGAVMAR.I
THIKA_MOUSE	MK_SCX_26.6978.6978.2	2	4.515	0.41	1	874.4	45.833336	R.LKPEQLGDISVGNVLEPGAGAVM*AR.I
THIKA_MOUSE	MK_SCX_33.4156.4156.3	3	4.413	0.518	1	2662.1	57.14286	R.FPQASASDVVVVHGR.R
THIKA_MOUSE	MK_SCX_33.7619.7619.3	3	4.454	0.343	1	1251.4	50	R.SKAEELGLPILGVLR.S
THIKA_MOUSE	MK_SCX_33.7700.7700.2	2	4.387	0.508	1	1395	78.57143	R.SKAEELGLPILGVLR.S
THIKA_MOUSE	MK_SCX_37.4463.4463.2	2	5.483	0.648	1	1297.8	73.52941	R.AEIVPVTTTVLDDKGDKK.T
THIKA_MOUSE	MK_SCX_43.4328.4328.3	3	4.686	0.499	1	1231.9	41.666664	K.KTITVSQDEGVRPSTTMQGLAK.L
THIKA_MOUSE	MK_SCX_52.6631.6631.2	2	4.313	0.499	1	1073.2	70	R.RSKAEELGLPILGVLR.S
THIKB_MOUSE	MK_SCX_12.5971.5971.2	2	3.877	0.462	1	567.3	66.66667	R.DCLIPM*GITSENVAER.F
THIKB_MOUSE	MK_SCX_12.6662.6662.2	2	3.262	0.372	1	605.3	63.333332	R.DCLIPMGITSENVAER.F
THIKB_MOUSE	MK_SCX_14.12088.12088.2	2	4.458	0.522	1	784.9	55.263157	K.DTTPDELLSAVLTAVLQDVK.L
THIKB_MOUSE	MK_SCX_26.6481.6481.3	3	3.551	0.333	1	668.4	32.291664	K.LKPEQLGDISVGNVLQPGAGAIM*AR.I
THIKB_MOUSE	MK_SCX_26.6948.6948.3	3	5.521	0.305	1	2519	42.708336	K.LKPEQLGDISVGNVLQPGAGAIMAR.I
THIKB_MOUSE	MK_SCX_26.6984.6984.2	2	6.502	0.67	1	1541.3	56.25	K.LKPEQLGDISVGNVLQPGAGAIMAR.I
THIKB_MOUSE	MK_SCX_5.6109.6109.2	2	3.491	0.174	1	668	55.263157	R.NGSYDIGMACGVESM*TLSQR.G
THIL_MOUSE	MK_SCX_14.12590.12590.3	3	3.892	0.33	1	615.2	25	K.ENGTITAANASTLNDGAAALVLMTAEAAQR.L
THIL_MOUSE	MK_SCX_14.7722.7722.2	2	3.813	0.605	1	513.5	27.586206	K.ENGTITAANASTLNDGAAALVLM*TAEAAQR.L
THIL_MOUSE	MK_SCX_14.8398.8398.2	2	4.058	0.616	1	617.8	31.034481	K.ENGTITAANASTLNDGAAALVLMTAEAAQR.L
THIL_MOUSE	MK_SCX_15.7962.7962.2	2	5.982	0.556	1	1495.4	56.521736	R.IAAFADAAVDPIDFPLAPAYAVPK.V
THIL_MOUSE	MK_SCX_15.7986.7986.3	3	5.893	0.516	1	2111.9	42.391304	R.IAAFADAAVDPIDFPLAPAYAVPK.V
THIL_MOUSE	MK_SCX_19.3802.3802.2	2	3.862	0.403	1	1542.1	64.70589	K.EVYM*GNVIQGGEGQAPTR.Q
THIL_MOUSE	MK_SCX_19.4156.4156.2	2	5.563	0.521	1	2378.6	73.52941	K.EVYMGNVIQGGEGQAPTR.Q
THIL_MOUSE	MK_SCX_19.7681.7681.3	3	4.355	0.557	1	942.9	27.941175	K.TVFQKENGTITAANASTLNDGAAALVLM*TAEAAQR.L
THIL_MOUSE	MK_SCX_19.8461.8461.3	3	5.717	0.544	1	1357.2	29.411766	K.TVFQKENGTITAANASTLNDGAAALVLMTAEAAQR.L
THIL_MOUSE	MK_SCX_20_1.6303.6303.2	2	4.347	0.386	1	1139.3	79.16667	K.FASEITPITISVK.G
THIL_MOUSE	MK_SCX_20_1.7223.7223.3	3	4.287	0.246	1	1125.4	42.1875	R.TPIGSFLGSLASQPATK.L
THIL_MOUSE	MK_SCX_20_1.7289.7289.2	2	5.524	0.65	1	1965.1	75	R.TPIGSFLGSLASQPATK.L
THIL_MOUSE	MK_SCX_21.4117.4117.2	2	3.3	0.406	1	523.5	66.66667	R.QEQDTYALSSYTR.S
THIL_MOUSE	MK_SCX_21.4227.4227.2	2	4.135	0.358	1	1118.4	86.36364	K.LGTAAIQGAIEK.A
THIL_MOUSE	MK_SCX_24.5906.5906.3	3	3.558	0.318	1	329.9	21	R.QATLGAGLPISTPCTTVNKVCASGMK.A
THIL_MOUSE	MK_SCX_25.5692.5692.3	3	3.463	0.25	1	511.3	27.5	K.DGLTDVYNKIHMGNCAENTAK.K
THIL_MOUSE	MK_SCX_26.4480.4480.2	2	5.824	0.662	1	2084.5	61.904762	K.EEVKEVYMGNVIQGGEGQAPTR.Q
THIL_MOUSE	MK_SCX_26.5456.5456.2	2	5.135	0.51	1	1989.4	76.666664	K.LEDLIVKDGLTDVYNK.I
THIL_MOUSE	MK_SCX_26.5480.5480.3	3	5.865	0.6	1	2366.3	55	K.LEDLIVKDGLTDVYNK.I
THIL_MOUSE	MK_SCX_26.7259.7259.2	2	5.176	0.604	1	1833.1	68.42105	K.EAWDAGKFASEITPITISVK.G
THIL_MOUSE	MK_SCX_27.4128.4128.3	3	5.519	0.459	1	1792.2	40.476192	K.EEVKEVYM*GNVIQGGEGQAPTR.Q
THIL_MOUSE	MK_SCX_27.4723.4723.3	3	5.895	0.282	1	1955.8	41.666664	K.EEVKEVYMGNVIQGGEGQAPTR.Q
THIL_MOUSE	MK_SCX_30.5770.5770.2	2	4.96	0.379	1	927.6	63.333332	R.GATPYGGVKLEDLIVK.D

THIL_MOUSE	MK_SCX_34.7784.7784.3	3	6.391	0.592	1	1346.5	39.583336	R.GATPYGGVKLEDLIVKDGLTDVYNK.I
THIL_MOUSE	MK_SCX_37.4884.4884.3	3	6.597	0.383	1	1365.9	33.653847	K.AGIPKEEVKEVYM*GNVIQGGEGQAPTR.Q
THIL_MOUSE	MK_SCX_37.5619.5619.3	3	7.043	0.465	1	1525.5	35.576923	K.AGIPKEEVKEVYMGNVIQGGEGQAPTR.Q
THIL_MOUSE	MK_SCX_42.6831.6831.3	3	6.633	0.523	1	2369.9	45.238094	R.SKEAWDAGKFASEITPITISVK.G
THIL_MOUSE	MK_SCX_43.7109.7109.2	2	5.482	0.683	1	2409	64.28571	R.SKEAWDAGKFASEITPITISVK.G
THIL_MOUSE	MK_SCX_48.5481.5481.3	3	4.186	0.516	1	455.2	32.407406	K.M*LEIDPQKVNIHGGAVSLGHPIGM*SGAR.I
THIL_MOUSE	MK_SCX_48.5709.5709.2	2	3.698	0.556	1	415	40.74074	K.MLEIDPQKVNIHGGAVSLGHPIGM*SGAR.I
THIL_MOUSE	MK_SCX_48.5749.5749.3	3	6.553	0.628	1	1252.5	36.11111	K.MLEIDPQKVNIHGGAVSLGHPIGM*SGAR.I
THIL_MOUSE	MK_SCX_48.6008.6008.3	3	5.259	0.473	1	837.9	35.185184	K.M*LEIDPQKVNIHGGAVSLGHPIGMSGAR.I
THIL_MOUSE	MK_SCX_48.6233.6233.3	3	6.868	0.622	1	1354.5	36.11111	K.MLEIDPQKVNIHGGAVSLGHPIGMSGAR.I
THIL_MOUSE	MK_SCX_48.6246.6246.2	2	3.96	0.521	1	480	42.592594	K.MLEIDPQKVNIHGGAVSLGHPIGMSGAR.I
THIL_MOUSE	MK_SCX_49.3116.3116.3	3	3.587	0.562	1	451	50	K.GKPDVVVKEDEEYK.R
THIL_MOUSE	MK_SCX_49.4432.4432.3	3	3.559	0.402	1	654.3	35.526314	K.VNIHGGAVSLGHPIGMSGAR.I
THIL_MOUSE	MK_SCX_50.4180.4180.2	2	6.217	0.542	1	2260.8	68.42105	K.VNIHGGAVSLGHPIGMSGAR.I
THIL_MOUSE	MK_SCX_55.2947.2947.3	3	3.556	0.462	1	543.1	50	K.GKPDVVVKEDEEYKR.V
THIM_MOUSE	MK_SCX_14.15383.15383.3	3	3.668	0.39	1	856.9	30.555555	K.DGTVTAGNASGVSDGAGAVIIASEDAVK.K
THIM_MOUSE	MK_SCX_14.6278.6278.2	2	6.92	0.71	1	2293.2	55.555557	K.DGTVTAGNASGVSDGAGAVIIASEDAVK.K
THIM_MOUSE	MK_SCX_14.7695.7695.2	2	5.88	0.596	1	1961.7	71.05263	K.DM*DLIDVNEAFAPQFLSVQK.A
THIM_MOUSE	MK_SCX_14.8278.8278.2	2	5.853	0.574	1	1665.5	68.42105	K.DMDLIDVNEAFAPQFLSVQK.A
THIM_MOUSE	MK_SCX_14.8371.8371.3	3	5.366	0.553	1	1862.3	46.05263	K.DMDLIDVNEAFAPQFLSVQK.A
THIM_MOUSE	MK_SCX_15.7558.7558.2	2	4.612	0.645	1	1441	52	K.VPPETIDSVIVGNVMQSSSDAAYLAR.H
THIM_MOUSE	MK_SCX_15.7752.7752.3	3	5.115	0.584	1	1525.3	36	K.VPPETIDSVIVGNVMQSSSDAAYLAR.H
THIM_MOUSE	MK_SCX_15.7806.7806.3	3	3.267	0.42	1	519.9	24.074074	R.VVGYFVSGCDPTIMGIGPVPAINGALKK.A
THIM_MOUSE	MK_SCX_17.5847.5847.2	2	5.117	0.639	1	2228.7	87.5	K.DFSATDLTEFAAR.A
THIM_MOUSE	MK_SCX_18.4800.4800.2	2	5.114	0.569	1	1547.4	61.764706	K.AANEAGYFNEEM*APIEVK.T
THIM_MOUSE	MK_SCX_18.5445.5445.1	1	3.439	0.596	1	574.4	58.823532	K.AANEAGYFNEEMAPIEVK.T
THIM_MOUSE	MK_SCX_18.5475.5475.2	2	5.604	0.575	1	1252.5	61.764706	K.AANEAGYFNEEMAPIEVK.T
THIM_MOUSE	MK_SCX_20_1.4672.4672.2	2	3.844	0.512	1	1211.3	80.769226	R.VGVPTETGALTLNR.L
THIM_MOUSE	MK_SCX_20_1.6348.6348.3	3	3.31	0.286	1	698.3	25.892857	K.DGTVTAGNASGVSDGAGAVIIASEDAVKK.H
THIM_MOUSE	MK_SCX_21.12037.12037.3	3	4.192	0.489	1	721.3	25.78125	R.AALSAGKVPPETIDSVIVGNVMQSSSDAAYLAR.H
THIM_MOUSE	MK_SCX_21.3603.3603.2	2	3.013	0.451	1	427.5	66.66667	K.LPM*GM*TAENLAAK.Y
THIM_MOUSE	MK_SCX_21.4611.4611.2	2	4.087	0.541	1	2156.5	91.66667	K.LPMGMTAENLAAK.Y
THIM_MOUSE	MK_SCX_21.7563.7563.3	3	5.271	0.432	1	1218.6	27.34375	R.AALSAGKVPPETIDSVIVGNVM*QSSSDAAYLAR.H
THIM_MOUSE	MK_SCX_21.8304.8304.2	2	4.457	0.627	1	552.9	37.5	R.AALSAGKVPPETIDSVIVGNVMQSSSDAAYLAR.H
THIM_MOUSE	MK_SCX_23.11826.11826.3	3	6.013	0.56	1	1644.5	36	K.AGLSLKDMDLIDVNEAFAPQFLSVQK.A
THIM_MOUSE	MK_SCX_23.12629.12629.3	3	5.18	0.484	1	738.7	29	K.AGLSLKDM*DLIDVNEAFAPQFLSVQK.A
THIM_MOUSE	MK_SCX_23.8247.8247.2	2	5.789	0.579	1	1598.1	52	K.AGLSLKDM*DLIDVNEAFAPQFLSVQK.A
THIM_MOUSE	MK_SCX_23.9069.9069.2	2	5.924	0.483	1	1638.7	50	K.AGLSLKDMDLIDVNEAFAPQFLSVQK.A
THIM_MOUSE	MK_SCX_24.4989.4989.2	2	3.329	0.481	1	1050.6	80	R.TPFGAYGGLLK.D
THIM_MOUSE	MK_SCX_25.9184.9184.3	3	6.287	0.554	1	3953	44.56522	R.TPFGAYGGLLKDFSATDLTEFAAR.A
THIM_MOUSE	MK_SCX_29.5745.5745.2	2	5.369	0.533	1	1869.1	82.14286	K.LEDTLWAGLTDQHVK.L
THIM_MOUSE	MK_SCX_29.5822.5822.3	3	4.526	0.489	1	620.7	48.214287	K.LEDTLWAGLTDQHVK.L
THIM_MOUSE	MK_SCX_30.7464.7464.3	3	3.463	0.166	1	547.7	23.148148	K.LEDTLWAGLTDQHVKLPM*GMTAENLAAK.Y
THIM_MOUSE	MK_SCX_30.7990.7990.3	3	4.773	0.545	1	395.3	28.703705	K.LEDTLWAGLTDQHVKLPMGMTAENLAAK.Y
THIM_MOUSE	MK_SCX_32.3857.3857.2	2	5.959	0.674	1	2059.3	71.05263	K.TNVSGGAIALGHPLGGSGSR.I
THIM_MOUSE	MK_SCX_32.3875.3875.3	3	5.061	0.628	1	967	43.421055	K.TNVSGGAIALGHPLGGSGSR.I
THIM_MOUSE	MK_SCX_32.5287.5287.3	3	5.61	0.491	1	2661.3	46.05263	R.WKAANEAGYFNEEM*APIEVK.T
THIM_MOUSE	MK_SCX_32.6071.6071.3	3	6.09	0.493	1	2309.3	47.368423	R.WKAANEAGYFNEEMAPIEVK.T
THIM_MOUSE	MK_SCX_32.6080.6080.2	2	5.661	0.547	1	1874.1	68.42105	R.WKAANEAGYFNEEMAPIEVK.T
THIM_MOUSE	MK_SCX_34.5767.5767.3	3	7.435	0.663	1	2195	43.51852	K.ALDLDPSKTNVSGGAIALGHPLGGSGSR.I
THIM_MOUSE	MK_SCX_34.5808.5808.2	2	5.031	0.667	1	641.5	48.148148	K.ALDLDPSKTNVSGGAIALGHPLGGSGSR.I

THIM_MOUSE	MK_SCX_40.3799.3799.3	3	3.308	0.311	1	310.5	30.263159	K.QTM*QVDEHARPQTTLEQLQK.L
THIM_MOUSE	MK_SCX_41.4352.4352.3	3	4.938	0.46	1	1008	40.789474	K.QTMQVDEHARPQTTLEQLQK.L
THIM_MOUSE	MK_SCX_42.8475.8475.3	3	5.889	0.558	1	2118.3	40.625	K.RTPFGAYGGLLKDFSATDLTEFAAR.A
THIM_MOUSE	MK_SCX_44.4756.4756.2	2	3.282	0.372	1	994	77.27273	K.RTPFGAYGGLLK.D
THIM_MOUSE	MK_SCX_44.4820.4820.3	3	3.816	0.173	1	1671.6	61.363636	K.RTPFGAYGGLLK.D
THIM_MOUSE	MK_SCX_47.6670.6670.3	3	5.233	0.527	1	776.5	30.000002	K.QTMQVDEHARPQTTLEQLQKLPSVFK.K
THIM_MOUSE	MK_SCX_53.3952.3952.3	3	5.467	0.487	1	1047.5	36.904762	K.GKQTMQVDEHARPQTTLEQLQK.L
THIM_MOUSE	MK_SCX_58.6863.6863.2	2	2.219	0.248	1	324.1	68.75	K.KHNFTPLAR.V
THIO_MOUSE	MK_SCX_20_1.4679.4679.2	2	4.744	0.562	1	2455.8	91.66667	K.EAFQEALAAAGDK.L
THIOM_MOUSE	MK_SCX_25.8194.8194.3	3	3.723	0.362	1	701.6	30.952381	K.NGDVVDKFVGIKDEDQLEAFLK.K
THIOM_MOUSE	MK_SCX_43.6532.6532.3	3	4.115	0.429	1	1033.1	50	K.FVGIKDEDQLEAFLKK.L
THOC4_MOUSE	MK_SCX_23.6806.6806.3	3	3.84	0.314	1	384.3	26.041666	K.QYNGVPLDGRPMNIQLVTSQIDTQR.R
THTM_MOUSE	MK_SCX_17.8469.8469.2	2	2.595	0.48	1	509.3	38.46154	R.DGIEPGHIPGSVNIPFTEFLTNEGLEK.S
THTM_MOUSE	MK_SCX_18.7489.7489.2	2	4.266	0.42	1	852.3	63.333332	K.SDVPVYDGSWVEWYMR.A
THTM_MOUSE	MK_SCX_20_1.9695.9695.3	3	7.541	0.54	1	1950.8	39.423077	R.DGIEPGHIPGSVNIPFTEFLTNEGLEK.S
THTM_MOUSE	MK_SCX_21.5948.5948.2	2	3.058	0.356	1	870.2	83.33333	K.LLDASWYLPK.L
THTM_MOUSE	MK_SCX_21.7219.7219.2	2	2.992	0.396	1	498.6	75	R.ALVSAQWVAEALK.A
THTM_MOUSE	MK_SCX_31.5651.5651.3	3	4.817	0.417	1	1347.3	48.52941	K.SHSEPAEFSAQLDPSFIK.T
THTM_MOUSE	MK_SCX_31.5660.5660.2	2	5.663	0.604	1	1611.6	76.47059	K.SHSEPAEFSAQLDPSFIK.T
THTM_MOUSE	MK_SCX_31.7186.7186.3	3	4.193	0.512	1	754.2	50	R.ALVSAQWVAEALKAPR.S
THTM_MOUSE	MK_SCX_31.7200.7200.2	2	3.996	0.407	1	1409.2	63.333332	R.ALVSAQWVAEALKAPR.S
THTM_MOUSE	MK_SCX_31.7550.7550.2	2	4.165	0.55	1	1779.6	76.666664	R.SSQPLKLLDASWYLPK.L
THTM_MOUSE	MK_SCX_35.4283.4283.2	2	4.508	0.571	1	1277.5	77.27273	K.THEDILENLDAR.R
THTR_MOUSE	MK_SCX_20_1.5800.5800.2	2	4.062	0.452	1	1049.2	79.16667	R.GSVNM*PFM*DFLTK.D
THTR_MOUSE	MK_SCX_20_1.6945.6945.2	2	3.593	0.307	1	709.9	66.66667	R.GSVNM*PFMDFLTK.D
THTR_MOUSE	MK_SCX_20_1.7095.7095.2	2	3.619	0.14	1	420.2	62.5	R.GSVNMPFM*DFLTK.D
THTR_MOUSE	MK_SCX_20_1.8624.8624.2	2	3.367	0.427	1	688.2	70.83333	R.GSVNMPFMDFLTK.D
THTR_MOUSE	MK_SCX_2201.4275.4275.2	2	2.638	0.354	1	998.4	72.72727	R.VLDASWYSPGTR.Q
THTR_MOUSE	MK_SCX_2201.5024.5024.2	2	4.325	0.498	1	1797.6	86.36364	K.TYEQVLENLQSK.R
THTR_MOUSE	MK_SCX_23.4971.4971.2	2	2.665	0.395	1	1034.3	93.75	K.ATLNLSLLK.T
THTR_MOUSE	MK_SCX_27.5573.5573.2	2	5.11	0.598	1	999.2	63.88889	R.YLGTQPEPDIVGLDSGHIR.G
THTR_MOUSE	MK_SCX_27.5576.5576.3	3	4.656	0.631	1	1209.5	47.22222	R.YLGTQPEPDIVGLDSGHIR.G
THUM1_MOUSE	MK_SCX_21.5199.5199.2	2	4.369	0.487	1	775.6	71.42857	R.FQSVESGANNVVFIR.T
TI8AB_MOUSE	MK_SCX_21.5858.5858.2	2	3.817	0.368	1	1567.4	90	R.FIDTSQFILNR.L
TIF1B_MOUSE	MK_SCX_19.5051.5051.2	2	3.375	0.586	1	568.4	78.125	K.LSPPYSSPQEFAQDVGR.M
TIF1B_MOUSE	MK_SCX_31.3352.3352.3	3	5.046	0.579	1	1965.1	58.333332	R.VLVNDAQKVTEGQQER.L
TIF1B_MOUSE	MK_SCX_43.3693.3693.2	2	3.379	0.408	1	792.5	75	R.SRSGEGEVSGLLR.K
TIF1B_MOUSE	MK_SCX_51.3253.3253.3	3	4.375	0.212	1	1360.8	44.11765	R.GRVLVNDAQKVTEGQQER.L
TIM10_MOUSE	MK_SCX_18.8448.8448.2	2	2.494	0.195	1	635.4	53.846157	R.KCVPPHYKEAELSK.G
TIM10_MOUSE	MK_SCX_49.4075.4075.3	3	4.036	0.139	1	766.1	44.642857	K.KLTELSM*QDEELMKR.V
TIM10_MOUSE	MK_SCX_49.4567.4567.2	2	4.597	0.376	1	1473.6	71.42857	K.KLTELSMQDEELMKR.V
TIM10_MOUSE	MK_SCX_49.4599.4599.3	3	4.435	0.387	1	1493.7	53.571426	K.KLTELSMQDEELMKR.V
TIM13_MOUSE	MK_SCX_20_1.6031.6031.2	2	5.246	0.262	1	1955.5	84.61539	K.VQIAVANAQELLQR.M
TIM14_MOUSE	MK_SCX_44.4171.4171.3	3	4.005	0.457	1	660.8	42.647057	R.IM*LLNHPDKGGSPYIAAK.I
TIM16_MOUSE	MK_SCX_29.7680.7680.3	3	4.041	0.519	1	749.6	28.846153	R.AGHQSAAASNLSGLSLQEAQQILNVSK.L
TIM16_MOUSE	MK_SCX_37.3737.3737.3	3	4.175	0.498	1	947.3	41.666664	R.ALRQEFAASQAAADAR.G
TIM16_MOUSE	MK_SCX_48.7075.7075.3	3	5.462	0.563	1	983.7	30.357143	R.GRAGHQSAAASNLSGLSLQEAQQILNVSK.L
TIM22_MOUSE	MK_SCX_15.8942.8942.2	2	4.189	0.633	1	486.3	37.5	K.AGGSAPEAAGSAEAPLQYSLLLQYLVGDK.R
TIM22_MOUSE	MK_SCX_2201.9222.9222.3	3	3.378	0.329	1	343.7	22.413794	K.AGGSAPEAAGSAEAPLQYSLLLQYLVGDKR.Q
TIM44_MOUSE	MK_SCX_28.9847.9847.2	2	3.177	0.458	1	775	63.333332	R.ALTDKVTDLLGGLFSK.T
TIM44_MOUSE	MK_SCX_40.7831.7831.2	2	3.342	0.341	1	677.8	68.181816	R.KGFLSGLLDNIK.Q

TIM44_MOUSE	MK_SCX_47.4508.4508.3	3	3.186	0.34	1	695.5	39.705883	K.ELDESVLGQTGPYRRPER.L
TIM44_MOUSE	MK_SCX_49.10617.10617.3	3	4.715	0.483	1	991	45.3125	R.KGFLSGLLDNIKQELAK.N
TIM50_MOUSE	MK_SCX_2201.4240.4240.2	2	3.152	0.437	1	1207	80	K.TIALNQVEDVR.T
TIM50_MOUSE	MK_SCX_26.6705.6705.3	3	3.971	0.398	1	1156.8	43.75	R.TVLEHYALEDDPLEAFK.Q
TIM8A_MOUSE	MK_SCX_52.5974.5974.3	3	3.903	0.431	1	1229.8	35.227272	R.FQQLVHQMTELCWEKCMDKPGPK.L
TIM9_MOUSE	MK_SCX_34.3791.3791.3	3	3.982	0.432	1	988.5	53.571426	R.FQEYHIQQNEALAAK.A
TIM9_MOUSE	MK_SCX_34.3797.3797.2	2	5.448	0.495	1	3356	89.28571	R.FQEYHIQQNEALAAK.A
TIM9B_MOUSE	MK_SCX_18.4393.4393.2	2	4.115	0.549	1	820	58.333332	R.IADYEAASAAPGIPAEQTR.D
TINAL_MOUSE	MK_SCX_12.5447.5447.2	2	3.669	0.352	1	751.9	52.77778	R.CPNGQVDSNDIYQVTPAYR.L
TINAL_MOUSE	MK_SCX_21.4551.4551.2	2	4.141	0.508	1	1230.7	79.16667	K.ITGWGEETLPDGR.T
TINAL_MOUSE	MK_SCX_21.9677.9677.3	3	5.151	0.597	1	1631.1	32.352943	R.LGTIRPSSTVMNMNEIYTVLGQGEVLPTAFEASEK.W
TINAL_MOUSE	MK_SCX_24.7535.7535.2	2	3.285	0.494	1	508.6	50	K.YWTAANSWGPWWGER.G
TINAL_MOUSE	MK_SCX_25.7637.7637.2	2	3.308	0.291	1	1178.4	93.75	R.LDGAWWFLR.R
TKT_MOUSE	MK_SCX_17.5489.5489.2	2	4.801	0.66	1	845.3	72.22222	K.ILATPPQEDAPSVDIANIR.M
TKT_MOUSE	MK_SCX_18.4603.4603.2	2	3.743	0.555	1	707	67.64706	R.SVPM*STVFYPSDGVATEK.A
TKT_MOUSE	MK_SCX_18.5133.5133.2	2	4.912	0.454	1	847.5	64.70589	R.SVPMSTVFYPSDGVATEK.A
TKT_MOUSE	MK_SCX_20_1.9079.9079.2	2	3.962	0.439	1	907.6	59.375	K.NM*AEQIIQEIYSQVQSK.K
TKT_MOUSE	MK_SCX_20_1.9269.9269.3	3	4.53	0.294	1	2485.6	51.5625	K.NM*AEQIIQEIYSQVQSK.K
TKT_MOUSE	MK_SCX_2201.2125.2125.2	2	2.631	0.468	1	972.7	87.5	K.AVELAANTK.G
TKT_MOUSE	MK_SCX_29.5636.5636.3	3	3.439	0.398	1	1142.3	38.157894	K.KILATPPQEDAPSVDIANIR.M
TKT_MOUSE	MK_SCX_30.4535.4535.3	3	5.575	0.515	1	2476.2	44.04762	R.TSRPENAIIYSNNEDFQVGQAK.V
TKT_MOUSE	MK_SCX_31.5414.5414.2	2	5.219	0.571	1	1894.6	88.46153	K.M*FGIDKDAIVQAVK.G
TKT_MOUSE	MK_SCX_31.5804.5804.2	2	2.793	0.377	1	416.6	68.181816	R.VLDPFTIKPLDR.K
TKT_MOUSE	MK_SCX_31.5962.5962.2	2	5.192	0.595	1	2701.6	88.46153	K.MFGIDKDAIVQAVK.G
TKT_MOUSE	MK_SCX_31.6018.6018.3	3	4.337	0.473	1	1735.3	57.692307	K.MFGIDKDAIVQAVK.G
TKT_MOUSE	MK_SCX_41.4164.4164.3	3	3.004	0.409	1	650.1	38.88889	R.LGQSDPAPLQHQVDIYQKR.C
TKT_MOUSE	MK_SCX_48.5017.5017.3	3	5.174	0.548	1	2377.4	51.25	K.KKILATPPQEDAPSVDIANIR.M
TKT_MOUSE	MK_SCX_48.5107.5107.2	2	2.334	0.267	1	329	58.333332	R.VLDPFTIKPLDRK.L
TKT_MOUSE	MK_SCX_48.7851.7851.3	3	3.636	0.439	1	709.1	31.730768	K.SKDDQVTVIGAGVTLHEALAAAESLKK.D
TKT_MOUSE	MK_SCX_54.3948.3948.3	3	4.256	0.526	1	1101.6	42.647057	R.GITGIEDKEAWHGKPLPK.N
TLN1_MOUSE	MK_SCX_14.10462.10462.3	3	4.37	0.492	1	1127.3	30.172413	K.GTEWVDPEDPTVIAENELLGAAAAIEAAAK.K
TLN1_MOUSE	MK_SCX_14.10555.10555.2	2	4.936	0.547	1	987.9	43.103447	K.GTEWVDPEDPTVIAENELLGAAAAIEAAAK.K
TLN1_MOUSE	MK_SCX_14.5780.5780.1	1	2.722	0.318	1	332.7	50	K.NGNLPEFGDAIATASK.A
TLN1_MOUSE	MK_SCX_14.9734.9734.2	2	5.041	0.651	1	625.8	34.848484	R.AATAPLLEAVDNLSAFASNPEFSSVPAQISPEGR.A
TLN1_MOUSE	MK_SCX_14.9790.9790.3	3	4.215	0.461	1	895.2	27.272728	R.AATAPLLEAVDNLSAFASNPEFSSVPAQISPEGR.A
TLN1_MOUSE	MK_SCX_15.8663.8663.2	2	6.13	0.68	1	1462	52.083332	R.GVAALTSDPAVQAIVLDTASDVLDK.A
TLN1_MOUSE	MK_SCX_15.8720.8720.3	3	4.676	0.475	1	1061.6	35.416664	R.GVAALTSDPAVQAIVLDTASDVLDK.A
TLN1_MOUSE	MK_SCX_16.6369.6369.2	2	4.982	0.571	1	1954.5	66.66667	K.LGAASLGAEDPETQVVLINAVK.D
TLN1_MOUSE	MK_SCX_16.7420.7420.2	2	5.293	0.379	1	1079.3	57.14286	R.IPEALAGPPNDFGLFLSDDDPK.K
TLN1_MOUSE	MK_SCX_16.7457.7457.3	3	3.508	0.352	1	539.8	32.142857	R.IPEALAGPPNDFGLFLSDDDPK.K
TLN1_MOUSE	MK_SCX_16.7534.7534.2	2	5.769	0.665	1	1458.8	48.148148	R.GSQAQPDSPSAQLALIAASQSFLQPGGK.M
TLN1_MOUSE	MK_SCX_17.5948.5948.2	2	3.557	0.35	1	995.5	79.16667	R.DPVQLNLLYVQAR.D
TLN1_MOUSE	MK_SCX_17.6537.6537.2	2	4.65	0.654	1	887.4	54.545456	K.VGAIPANALDDGQWSQGLISAAR.M
TLN1_MOUSE	MK_SCX_18.5953.5953.2	2	4.469	0.597	1	721.5	50	K.SNTSPEELGPLANQLTSDYGR.L
TLN1_MOUSE	MK_SCX_19.4773.4773.2	2	5.112	0.561	1	2258	73.52941	K.LLGEIAQGNENYAGIAAR.D
TLN1_MOUSE	MK_SCX_19.5998.5998.2	2	4.988	0.586	1	1367.1	63.15789	R.LNEAAAGLNQAATELVQASR.G
TLN1_MOUSE	MK_SCX_20_1.8295.8295.2	2	3.001	0.412	1	935.3	61.538464	K.TLAESALQLLYTAK.E
TLN1_MOUSE	MK_SCX_21.3928.3928.2	2	3.937	0.498	1	639.5	76.92308	K.LAQAAQSSVATITR.L
TLN1_MOUSE	MK_SCX_21.4086.4086.2	2	3.142	0.308	1	537.8	77.27273	R.QEDVIATANLSR.R
TLN1_MOUSE	MK_SCX_21.5106.5106.2	2	4.25	0.424	1	2482.5	80.769226	K.TMLESAGGLIQTAR.A
TLN1_MOUSE	MK_SCX_2201.7894.7894.3	3	3.922	0.544	1	420.8	31	K.LGAASLGAEDPETQVVLINAVKDVAK.A

TLN1_MOUSE	MK_SCX_23.5872.5872.2	2	2.818	0.463	1	334.2	50	K.DHFGLEGDEESTMLEDSVSPK.K
TLN1_MOUSE	MK_SCX_23.6119.6119.2	2	2.904	0.426	1	790.4	88.88889	K.TYGVSFFLVK.E
TLN1_MOUSE	MK_SCX_23.7198.7198.3	3	5.809	0.517	1	1368.6	40.909092	R.IPEALAGPPNDFGLFLSDDDPKK.G
TLN1_MOUSE	MK_SCX_24.4702.4702.2	2	2.429	0.157	1	523.1	83.33333	K.NLGTALAELR.T
TLN1_MOUSE	MK_SCX_25.5043.5043.3	3	5.834	0.56	1	851.2	33.333336	R.SGASGPENFQVGSMPPAQQQITSGQMHR.G
TLN1_MOUSE	MK_SCX_25.7560.7560.3	3	4.588	0.43	1	1031.9	38.04348	R.ERIPEALAGPPNDFGLFLSDDDPK.K
TLN1_MOUSE	MK_SCX_31.7899.7899.3	3	3.036	0.244	1	945.4	54.545456	K.AGFLDLKDFLPK.E
TLN1_MOUSE	MK_SCX_31.7907.7907.2	2	3.337	0.294	1	1597.9	81.818184	K.AGFLDLKDFLPK.E
TLN1_MOUSE	MK_SCX_33.4158.4158.3	3	4.74	0.32	1	1597.6	51.785713	K.TLSHPQQMALLDQTK.T
TLN1_MOUSE	MK_SCX_34.4097.4097.3	3	3.901	0.404	1	755.2	52.083332	R.IGITNHDEYSLVR.E
TLN1_MOUSE	MK_SCX_41.4075.4075.3	3	6.084	0.453	1	3318.4	48.80952	R.LASQAKPAAVAAENEEIGAHIK.H
TLN1_MOUSE	MK_SCX_41.5453.5453.3	3	3.922	0.3	1	701.1	29.545454	K.SKDHFGLEGDEESTMLEDSVSPK.K
TLN1_MOUSE	MK_SCX_49.4341.4341.3	3	4.09	0.479	1	1351.4	41.666664	R.TLREQGVEEHETLLLR.R
TLN1_MOUSE	MK_SCX_49.4895.4895.3	3	4.191	0.427	1	1006.3	51.923077	K.LHTDDELNWLDHGR.T
TLN1_MOUSE	MK_SCX_49.5035.5035.2	2	4.62	0.446	1	1694.5	84.61539	K.LHTDDELNWLDHGR.T
TLN1_MOUSE	MK_SCX_51.4797.4797.3	3	4.772	0.504	1	1089.8	55.35714	K.TKEVIQEWSLTNIKR.W
TM16F_MOUSE	MK_SCX_24.3574.3574.2	2	2.46	0.264	1	353.9	55.555557	R.LNITCESSKK.L
TM16F_MOUSE	MK_SCX_24.6776.6776.2	2	2.867	0.326	1	396.2	70	R.SPFGNLNWFTK.V
TMED4_MOUSE	MK_SCX_28.5262.5262.2	2	4.16	0.35	1	808.7	59.375	R.QLLDQVEQIQKEQDYQR.Y
TMED4_MOUSE	MK_SCX_28.5277.5277.3	3	3.806	0.302	1	862.3	39.0625	R.QLLDQVEQIQKEQDYQR.Y
TMED4_MOUSE	MK_SCX_48.4979.4979.3	3	3.642	0.484	1	762.1	36.11111	R.ARQLLDQVEQIQKEQDYQR.Y
TMED9_MOUSE	MK_SCX_24.6490.6490.2	2	3.145	0.372	1	1308.1	94.44444	K.FSLFAGGMLR.V
TMED9_MOUSE	MK_SCX_50.4659.4659.3	3	7.154	0.602	1	3091.4	52.77778	R.VRQLVEQVEQIQKEQNYQR.W
TMEDA_MOUSE	MK_SCX_16.6301.6301.2	2	2.656	0.242	1	506.8	53.333336	R.LEDLSESIVNDFAYMK.K
TMEDA_MOUSE	MK_SCX_20_1.5599.5599.2	2	3.935	0.411	1	1934	90	R.IPDQLVILDM*K.H
TMEDA_MOUSE	MK_SCX_31.8138.8138.2	2	4.96	0.6	1	2479.8	78.125	R.RLEDLSESIVNDFAYMK.K
TMEDA_MOUSE	MK_SCX_32.5171.5171.3	3	4.007	0.305	1	905.8	42.857143	K.GTGRIPDQLVILDM*K.H
TMEDA_MOUSE	MK_SCX_32.5864.5864.3	3	3.905	0.466	1	1864.3	50	K.GTGRIPDQLVILDMK.H
TMEDA_MOUSE	MK_SCX_32.6082.6082.2	2	3.456	0.424	1	387.1	57.14286	K.GTGRIPDQLVILDMK.H
TMEDA_MOUSE	MK_SCX_39.4124.4124.2	2	2.436	0.352	1	407.8	87.5	K.LKPLEVELR.R
TMEDA_MOUSE	MK_SCX_42.3555.3555.3	3	4.486	0.585	1	913	45.3125	K.ITDSAGHILYAKEDATK.G
TMEDA_MOUSE	MK_SCX_44.7235.7235.3	3	4.641	0.387	1	1180.6	41.17647	R.RLEDLSESIVNDFAYMKK.R
TMEM9_MOUSE	MK_SCX_2201.2903.2903.2	2	4.059	0.588	1	1577.7	83.33333	R.TM*ATAAASIGGPR.A
TMEM9_MOUSE	MK_SCX_23.3646.3646.2	2	3.394	0.54	1	1569	87.5	R.TMATAAASIGGPR.A
TMM24_MOUSE	MK_SCX_27.3909.3909.3	3	4.233	0.46	1	458.9	32.5	K.EAGLSQSHDDLSNTTATPSVR.K
TMM24_MOUSE	MK_SCX_28.6459.6459.3	3	3.078	0.317	1	412.2	32.8125	R.ERDEEQPELSTVEELIK.D
TMM27_MOUSE	MK_SCX_28.15897.15897.3	3	3.343	0.334	1	308.6	26.38889	R.AALGDKAYVWDTDQEYLFR.A
TMM27_MOUSE	MK_SCX_36.3729.3729.3	3	3.105	0.311	1	1055.4	44.230766	K.GGHINDGFLTEDER.L
TMM27_MOUSE	MK_SCX_36.3762.3762.2	2	4.59	0.606	1	2190.3	80.769226	K.GGHINDGFLTEDER.L
TMM27_MOUSE	MK_SCX_48.7458.7458.3	3	4.648	0.561	1	1581.3	45.588234	R.NRINSAFFLDDHTLEFLK.I
TMM51_MOUSE	MK_SCX_19.5737.5737.2	2	3.553	0.433	1	597.9	52.941177	R.YYVPSYEEVMNTGYPETR.G
TMM51_MOUSE	MK_SCX_24.3533.3533.3	3	5.743	0.653	1	900.7	30.000002	R.IQQQAGTVPHSQEEDSQEEEEDVSSR.Y
TMM9B_MOUSE	MK_SCX_14.10312.10312.3	3	3.275	0.122	1	326.3	19.285715	-.M*ASLWCGNLLRLGSGLSMSCLALSVLLLAQLTGAAK.N
TMM9B_MOUSE	MK_SCX_55.3309.3309.3	3	4.038	0.438	1	694.7	41.07143	R.SRANVLNKVEYAQQR.W
TMOD3_MOUSE	MK_SCX_16.8391.8391.2	2	4.827	0.486	1	827.6	52.499996	K.QLETVLDDLDPENALLPAGFR.Q
TMOD3_MOUSE	MK_SCX_17.10107.10107.2	2	3.538	0.334	1	640.3	45.238094	K.SLNM*ESNFITGAGVLALIDALR.D
TMOD3_MOUSE	MK_SCX_19.6959.6959.2	2	4.133	0.537	1	1527.8	76.92308	R.SNDPVAVAFADMLK.V
TMOD3_MOUSE	MK_SCX_2201.5931.5931.3	3	3.002	0.378	1	306.4	30.000002	K.DLGDYKDLDEDELLGK.L
TMOD3_MOUSE	MK_SCX_24.4206.4206.2	2	3.81	0.563	2	1501.1	85	K.FGYQFTQQGPR.T
TMOD3_MOUSE	MK_SCX_41.5143.5143.3	3	3.567	0.261	1	495.6	37.5	R.KDLGDYKDLDEDELLGK.L
TOIP1_MOUSE	MK_SCX_19.4968.4968.2	2	3.256	0.345	1	858	64.28571	R.FSEEPAEVYGDFEPR.A

TOIP1_MOUSE	MK_SCX_33.3044.3044.3	3	4.497	0.46	1	1345.8	43.75	R.LEQHSQQPQLSPATSGR.G
TOIP2_MOUSE	MK_SCX_46.4278.4278.3	3	4.789	0.52	1	1434.8	47.058823	R.RLPVPEAGSHEEETELVK.E
TOLIP_MOUSE	MK_SCX_34.6147.6147.3	3	4.307	0.373	1	313.3	41.666664	R.IAWTHITIPESLK.Q
TOM1_MOUSE	MK_SCX_14.9423.9423.3	3	4.167	0.52	1	616.8	27.884615	R.VMSEMLTELVPTQVEPADLELLQELNR.T
TOM1_MOUSE	MK_SCX_19.4547.4547.2	2	5.386	0.541	1	1454.9	75	K.YEAPQTTDGLAGALDAR.Q
TOM1_MOUSE	MK_SCX_21.9192.9192.2	2	3.323	0.536	1	959.4	75	K.VLNLIQSWADAFR.S
TOM1_MOUSE	MK_SCX_33.3283.3283.3	3	3.47	0.407	1	1154	46.25	R.TVFNSETPSRQNSVSSNTSQR.G
TOM22_MOUSE	MK_SCX_16.6455.6455.2	2	3.811	0.553	1	487.8	39.583336	R.QILLGPNTGLSGGM*PGALPPLPGK.M
TOM22_MOUSE	MK_SCX_16.7047.7047.2	2	4.068	0.667	1	536.8	41.304348	R.QILLGPNTGLSGGMPGALPPLPGK.M
TOM22_MOUSE	MK_SCX_2201.6042.6042.2	2	2.409	0.167	1	637.4	70	R.LWGLTEM*FPER.V
TOM22_MOUSE	MK_SCX_2201.7528.7528.2	2	3.441	0.457	1	1341.7	85	R.LWGLTEMFPER.V
TOM70_MOUSE	MK_SCX_13.6658.6658.2	2	2.395	0.127	1	375.9	43.75	K.WKEVAQDCTKAVELNPK.Y
TOM70_MOUSE	MK_SCX_18.7906.7906.2	2	3.208	0.139	1	903.5	53.125	K.SYFSSFTDDIISQPMLK.G
TOM70_MOUSE	MK_SCX_2201.4739.4739.2	2	4.034	0.515	1	1504.1	85	K.NVDLSTFYQNR.A
TOM70_MOUSE	MK_SCX_25.5530.5530.2	2	2.888	0.176	1	1225.7	85.71429	K.GLLQLQWK.Q
TOP1_MOUSE	MK_SCX_18.5597.5597.2	2	2.845	0.446	1	460.8	63.333332	K.GPVFAPPYEPLPESVK.F
TOP1_MOUSE	MK_SCX_20_1.3497.3497.2	2	3.123	0.423	1	982.6	81.818184	K.ELTAPDENVPAK.I
TOP1_MOUSE	MK_SCX_41.3730.3730.3	3	5.161	0.443	1	1554	43.421055	K.LEVQATDREENKQIALGTSK.L
TOP1_MOUSE	MK_SCX_41.6110.6110.3	3	3.158	0.146	1	637.6	33.75	K.FLEHKGPVFAPPYEPLPESVK.F
TPD52_MOUSE	MK_SCX_14.7764.7764.2	2	4.749	0.564	1	652	39.655174	K.TEPVAEEGEDAVTMLSAPEALTEEEQEELR.R
TPD52_MOUSE	MK_SCX_18.6086.6086.2	2	3.696	0.499	1	665.3	64.28571	K.VEEEIQTLSQVLAAK.E
TPD52_MOUSE	MK_SCX_19.7505.7505.3	3	3.262	0.215	1	420.7	20.833332	K.TEPVAEEGEDAVTMLSAPEALTEEEQEELRR.E
TPD52_MOUSE	MK_SCX_21.4572.4572.2	2	3.238	0.414	1	894.9	65.38461	K.ASAAFSSVGSVITK.K
TPD52_MOUSE	MK_SCX_23.4954.4954.2	2	3.825	0.436	1	1129.9	88.88889	K.LGISSLQEFK.Q
TPD52_MOUSE	MK_SCX_28.8817.8817.2	2	4.256	0.535	1	1582.6	66.66667	R.ELTKVEEEIQTLSQVLAAK.E
TPD52_MOUSE	MK_SCX_38.3609.3609.2	2	3.545	0.34	1	1178.7	88.88889	K.SFEEKVENLK.S
TPD52_MOUSE	MK_SCX_41.4559.4559.2	2	3.251	0.396	1	1140	80	R.KLGISSLQEFK.Q
TPD52_MOUSE	MK_SCX_41.4698.4698.3	3	3.737	0.321	1	558.3	52.499996	R.KLGISSLQEFK.Q
TPD52_MOUSE	MK_SCX_48.8371.8371.3	3	5.385	0.416	1	2887.8	46.05263	R.RELTKVEEEIQTLSQVLAAK.E
TPD52_MOUSE	MK_SCX_51.5202.5202.3	3	3.699	0.427	1	525	36.666668	R.KLGISSLQEFKQNIAK.G
TPD52_MOUSE	MK_SCX_52.4999.4999.2	2	5.457	0.511	1	2095	76.666664	R.KLGISSLQEFKQNIAK.G
TPD54_MOUSE	MK_SCX_18.4960.4960.2	2	5.288	0.424	1	2863.1	86.666664	R.TPVVEGLTEGEEEELR.A
TPD54_MOUSE	MK_SCX_2201.4059.4059.2	2	2.123	0.14	1	372.8	57.692307	K.TSAALSTMGSAISR.K
TPD54_MOUSE	MK_SCX_2201.4899.4899.2	2	2.796	0.383	1	800.8	83.33333	R.LGLSTLGELK.Q
TPD54_MOUSE	MK_SCX_25.6567.6567.3	3	4.222	0.381	1	728.7	36.25	R.TPVVEGLTEGEEEELRAELAK.V
TPD54_MOUSE	MK_SCX_25.7261.7261.3	3	4.19	0.493	1	1160	41.666664	K.GVLSDFMTDVPVDPGVVHR.T
TPD54_MOUSE	MK_SCX_33.6372.6372.3	3	3.789	0.55	1	523.1	37.5	R.LGLSTLGELKQNLSR.S
TPD54_MOUSE	MK_SCX_33.6428.6428.2	2	3.666	0.435	1	1103.4	71.42857	R.LGLSTLGELKQNLSR.S
TPD54_MOUSE	MK_SCX_38.4335.4335.2	2	3.145	0.386	1	752.5	66.66667	R.SWHDVQVSTAYVK.T
TPD54_MOUSE	MK_SCX_47.4498.4498.3	3	4.066	0.457	1	1071	48.333332	K.LGEWNEKVTQSDLYKK.T
TPIS_MOUSE	MK_SCX_14.3866.3866.2	2	4.248	0.586	1	875.9	83.33333	R.IIYGGSVTGATCK.E
TPIS_MOUSE	MK_SCX_18.4565.4565.2	2	3.544	0.445	1	1365	66.66667	K.VTNGAFTGEISPGM*IK.D
TPIS_MOUSE	MK_SCX_18.5075.5075.2	2	3.868	0.304	1	1017.4	56.666668	K.VTNGAFTGEISPGMIK.D
TPIS_MOUSE	MK_SCX_19.6293.6293.2	2	4.52	0.547	1	2066.4	82.14286	K.VVLAYEPVWAIGTGK.T
TPIS_MOUSE	MK_SCX_19.8272.8272.2	2	2.999	0.397	1	658.4	35.714287	K.ELASQPDVDGFLVGGASLKPEFVDIINAK.Q
TPIS_MOUSE	MK_SCX_20_1.10104.10104.3	3	3.738	0.529	1	340.9	33.92857	K.ELASQPDVDGFLVGGASLKPEFVDIINAK.Q
TPIS_MOUSE	MK_SCX_24.3377.3377.2	2	3.885	0.497	1	1623.3	81.818184	K.SNVNDGVAQSTR.I
TPIS_MOUSE	MK_SCX_28.4438.4438.2	2	2.276	0.358	1	707.5	100	K.FFVGGNWK.M
TPIS_MOUSE	MK_SCX_29.5494.5494.2	2	5.148	0.61	1	2024	88.46153	K.DLGATWVVLGHSER.R
TPIS_MOUSE	MK_SCX_37.3981.3981.2	2	4.742	0.526	1	983.9	87.5	R.HVFGESDELIGQK.V
TPIS_MOUSE	MK_SCX_54.3483.3483.2	2	4.725	0.453	1	1473.2	76.92308	R.RHVFGESDELIGQK.V

TPIS_MOUSE	MK_SCX_55.3665.3665.3	3	5.413	0.508	1	2740.6	57.692307	R.RHVFGESDELIGQK.V
TPM1_MOUSE	MK_SCX_21.4507.4507.2	2	3.645	0.329	1	994.8	88.88889	R.IQLVEEELDR.A
TPM1_MOUSE	MK_SCX_31.4850.4850.2	2	4.003	0.285	1	872.1	61.538464	R.IQLVEEELDRAQER.L
TPM1_MOUSE	MK_SCX_35.3372.3372.3	3	3.507	0.225	1	1110.5	46.153847	K.KATDAEADVASLNR.R
TPM1_MOUSE	MK_SCX_35.4427.4427.2	2	3.628	0.416	1	864	75	R.LATALQKLEEAEK.A
TPM1_MOUSE	MK_SCX_36.4199.4199.3	3	3.808	0.369	1	1922.7	62.5	R.KLVIIESDLER.A
TPM1_MOUSE	MK_SCX_40.4247.4247.2	2	3.214	0.259	1	1356.3	90	R.RIQLVEEELDR.A
TPM1_MOUSE	MK_SCX_43.5618.5618.3	3	5.067	0.595	1	1720.3	42.105263	R.LATALQKLEEAEKAADESER.G
TPM1_MOUSE	MK_SCX_43.5645.5645.2	2	5.307	0.56	1	2030	65.789474	R.LATALQKLEEAEKAADESER.G
TPM1_MOUSE	MK_SCX_45.3775.3775.3	3	4.547	0.276	1	1176.4	48.333332	R.AQKDEEKM*EIQEIQLK.E
TPM1_MOUSE	MK_SCX_45.4324.4324.2	2	5.813	0.453	1	2571.5	76.666664	R.AQKDEEKMEIQEIQLK.E
TPM1_MOUSE	MK_SCX_45.4368.4368.3	3	4.476	0.411	1	1420.6	50	R.AQKDEEKMEIQEIQLK.E
TPM1_MOUSE	MK_SCX_50.4089.4089.3	3	3.093	0.297	1	691.5	44.230766	R.SKQLEDELVSLQKK.L
TPM1_MOUSE	MK_SCX_52.4242.4242.3	3	4.093	0.402	1	1210.7	46.42857	R.RIQLVEEELDRAQER.L
TPM1_MOUSE	MK_SCX_60.2966.2966.2	2	2.16	0.136	1	318.9	71.42857	K.HIAEDADR.K
TPM2_MOUSE	MK_SCX_38.4708.4708.3	3	3.618	0.38	1	1711.1	62.5	R.KLVILEGELER.S
TPM2_MOUSE	MK_SCX_38.4718.4718.2	2	3.84	0.288	1	1902.6	90	R.KLVILEGELER.S
TPM2_MOUSE	MK_SCX_47.4358.4358.3	3	5.47	0.598	1	1140.9	45.3125	K.YSESVKDAQEKLEQAEK.K
TPM3_MOUSE	MK_SCX_21.3691.3691.2	2	3.784	0.455	1	802	66.66667	K.AADAEAEVASLNR.R
TPM3_MOUSE	MK_SCX_21.3692.3692.3	3	3.247	0.396	1	798.5	50	K.AADAEAEVASLNR.R
TPM3_MOUSE	MK_SCX_37.4588.4588.3	3	4.418	0.36	1	2257.9	67.5	R.KLVIIEGDLER.T
TPM3_MOUSE	MK_SCX_37.4625.4625.2	2	3.643	0.545	1	1914.9	90	R.KLVIIEGDLER.T
TPM3_MOUSE	MK_SCX_44.4518.4518.3	3	3.407	0.378	1	723.3	36.666668	R.ALKDEEKM*ELQEIQLK.E
TPM3_MOUSE	MK_SCX_44.4613.4613.2	2	5.177	0.524	1	815.4	73.333336	R.ALKDEEKM*ELQEIQLK.E
TPM3_MOUSE	MK_SCX_44.4934.4934.2	2	5.557	0.433	1	2301	73.333336	R.ALKDEEKMELQEIQLK.E
TPM3_MOUSE	MK_SCX_44.4944.4944.3	3	5.274	0.39	1	1709.8	50	R.ALKDEEKMELQEIQLK.E
TPM3_MOUSE	MK_SCX_49.4447.4447.3	3	3.206	0.434	1	607.4	42.857143	R.KLVIIEGDLERTEER.A
TPM3_MOUSE	MK_SCX_51.3373.3373.3	3	3.131	0.394	1	347.8	45.454548	K.ILTDKLKEAETR.A
TPM4_MOUSE	MK_SCX_19.5855.5855.2	2	6.135	0.502	1	2826.4	78.125	R.AQEQLATALQNLEEAEK.A
TPM4_MOUSE	MK_SCX_20_1.3418.3418.2	2	5.267	0.511	1	2389.3	84.61539	K.IQALQQQADDAEDR.A
TPM4_MOUSE	MK_SCX_21.4983.4983.2	2	3.613	0.344	1	1072.7	88.88889	R.IQLLEEELDR.A
TPM4_MOUSE	MK_SCX_23.11599.11599.3	3	3.381	0.363	1	798.4	28.846153	R.IQLLEEELDRAQEQLATALQNLEEAEK.A
TPM4_MOUSE	MK_SCX_24.7140.7140.3	3	6.248	0.549	1	1316.7	40.217392	R.AQEQLATALQNLEEAEKAADESER.G
TPM4_MOUSE	MK_SCX_35.3207.3207.3	3	6.156	0.449	1	2652.7	58.928574	R.KIQALQQQADDAEDR.A
TPM4_MOUSE	MK_SCX_35.3223.3223.2	2	5.949	0.587	1	3696.2	89.28571	R.KIQALQQQADDAEDR.A
TPM4_MOUSE	MK_SCX_39.5023.5023.2	2	2.384	0.167	1	695.2	72.22222	R.KLVILEGELK.R
TPM4_MOUSE	MK_SCX_40.5143.5143.2	2	3.074	0.299	1	1317.5	85	R.RIQLLEEELDR.A
TPM4_MOUSE	MK_SCX_45.4770.4770.3	3	3.658	0.353	1	867.2	45	R.AMKDEEKM*EILEMQLK.E
TPM4_MOUSE	MK_SCX_46.4363.4363.3	3	4.413	0.432	1	1723.2	41.25	R.KIQALQQQADDAEDRAQGLQR.E
TPM4_MOUSE	MK_SCX_46.4470.4470.3	3	4.586	0.141	1	1288.7	48.333332	R.AMKDEEKMEILEM*QLK.E
TPMT_MOUSE	MK_SCX_16.7343.7343.2	2	5.174	0.637	1	1262.6	57.14286	R.EFFAEQNLSYTEEPLAEIAGAK.V
TPMT_MOUSE	MK_SCX_19.7954.7954.2	2	3.509	0.39	1	1009	58.823532	K.SSSGSISLYCCSIFDLPR.A
TPMT_MOUSE	MK_SCX_23.7168.7168.2	2	3.157	0.363	1	921.7	88.88889	K.AWGLDYLFEK.L
TPMT_MOUSE	MK_SCX_34.4442.4442.2	2	3.566	0.422	1	1742.2	86.36364	K.NQVLTLEDWKEK.W
TPMT_MOUSE	MK_SCX_54.3779.3779.3	3	3.493	0.456	1	1120.1	47.916664	K.HLDTFLKGQSGLR.V
TPMT_MOUSE	MK_SCX_55.6375.6375.3	3	3.473	0.326	1	984.5	47.727272	R.HKAWGLDYLFEK.L
TPMT_MOUSE	MK_SCX_56.6390.6390.2	2	3.216	0.438	1	811.3	77.27273	R.HKAWGLDYLFEK.L
TPP1_MOUSE	MK_SCX_15.9919.9919.2	2	5.8	0.637	1	896.2	56.81818	K.YLTLEDVAELVQPSPLTLLTVQK.W
TPP1_MOUSE	MK_SCX_37.4124.4124.2	2	4.689	0.432	1	1245.8	80.769226	R.LFGGSFTHQASVAK.V
TPSN_MOUSE	MK_SCX_16.7934.7934.2	2	2.798	0.479	1	531.8	36	R.FAYAPSALEGSPSLDAGPPPFGLEWR.R
TPSN_MOUSE	MK_SCX_27.7551.7551.2	2	2.771	0.193	1	631.3	52.941177	K.LYFKVDDPAGMLLAAFRR.Y

TR150_MOUSE	MK_SCX_26.5666.5666.2	2	3.255	0.5	1	357.3	50	R.MDSFDEDLARPSGLLAQER.K
TR150_MOUSE	MK_SCX_27.3471.3471.3	3	6.226	0.624	1	1275.1	41.346153	K.SPPATGSAYGSSQKEESAASGGAAYSK.R
TR150_MOUSE	MK_SCX_30.3743.3743.3	3	5.309	0.426	1	1361.8	40	K.GSESSKPWPDATTYGAGSASR.A
TR150_MOUSE	MK_SCX_41.3691.3691.2	2	4.338	0.565	1	1371.4	75	R.SIFQHIQSAQSQR.S
TR150_MOUSE	MK_SCX_42.3302.3302.3	3	5.02	0.577	1	828.2	32.407406	K.SPPATGSAYGSSQKEESAASGGAAYSKR.Y
TRA2A_MOUSE	MK_SCX_21.5152.5152.2	2	3.508	0.509	1	1776.2	76.92308	R.YGPLSGVNVVYDQR.T
TRA2B_MOUSE	MK_SCX_18.5586.5586.2	2	4.196	0.47	1	1460.6	66.66667	K.YGPIADVSIVYDQQSR.R
TRAP1_MOUSE	MK_SCX_17.5656.5656.2	2	6.286	0.655	1	1036.8	65.789474	R.YESSALPAGQLTSLPDYASR.M
TRAP1_MOUSE	MK_SCX_19.4940.4940.2	2	4.521	0.506	1	1351.7	76.92308	R.GVVDSEDIPLNLSR.E
TRAP1_MOUSE	MK_SCX_21.3628.3628.2	2	2.284	0.133	1	313.7	60.000004	R.LNDLLVKVLEK.H
TRAP1_MOUSE	MK_SCX_21.4710.4710.2	2	2.692	0.334	1	798.2	83.33333	R.ELLQESALIR.K
TRAP1_MOUSE	MK_SCX_2201.7054.7054.2	2	2.408	0.285	1	851	83.33333	K.FFEDYGLFMR.E
TRAP1_MOUSE	MK_SCX_26.7674.7674.3	3	3.62	0.476	1	873.1	26.923079	K.KGTITIQDTGIGMTQEELVSNLGTIAR.S
TREA_MOUSE	MK_SCX_10.6555.6555.2	2	3.093	0.287	1	503.3	56.25	K.QFVDMSLATSPDEVLQK.F
TREA_MOUSE	MK_SCX_18.9281.9281.2	2	4.887	0.557	1	1062.9	55	R.NTGQQWDFPNAWAPLQDLVIR.G
TREA_MOUSE	MK_SCX_19.6997.6997.2	2	4.668	0.584	1	1498.5	78.57143	R.WLVGGPDPDLLSSIR.T
TREA_MOUSE	MK_SCX_21.4472.4472.2	2	4.683	0.455	1	2085.2	82.14286	R.TVSVVSGGQSYVLNR.Y
TREA_MOUSE	MK_SCX_21.5764.5764.2	2	4.263	0.485	1	924.8	75	K.ILTYQYGIPTSLR.N
TREA_MOUSE	MK_SCX_21.7430.7430.2	2	5.094	0.552	1	2871.1	84.61539	R.TQEVAFQLAQNWIK.T
TREA_MOUSE	MK_SCX_23.5427.5427.2	2	2.793	0.209	1	783.6	77.77778	K.GAWFDYDLEK.G
TRFE_MOUSE	MK_SCX_2201.3756.3756.2	2	4.767	0.594	1	2477.7	88.46153	K.YLGAEYM*QSVGNM*R.K
TRFE_MOUSE	MK_SCX_2201.4192.4192.2	2	4.958	0.421	1	2700.4	88.46153	K.YLGAEYM*QSVGNMR.K
TRFE_MOUSE	MK_SCX_2201.5362.5362.2	2	4.348	0.611	1	2034.9	84.61539	K.YLGAEYMQSVGNMR.K
TRFE_MOUSE	MK_SCX_2201.7231.7231.2	2	4.578	0.386	1	842.8	80.769226	R.TAGWNIPMGMLYNR.I
TRFE_MOUSE	MK_SCX_37.5656.5656.2	2	2.713	0.274	1	650.7	70.83333	K.SKDFQLFSSPLGK.D
TRFE_MOUSE	MK_SCX_38.4096.4096.3	3	3.642	0.315	1	1286.9	50	K.KGTDFQLNQLEGK.K
TRFE_MOUSE	MK_SCX_38.4101.4101.2	2	4.062	0.507	1	1444.4	75	K.KGTDFQLNQLEGK.K
TRFE_MOUSE	MK_SCX_38.4631.4631.2	2	3.221	0.352	1	1116.1	81.818184	R.LYLGHNYVTAIR.N
TRFE_MOUSE	MK_SCX_41.6276.6276.3	3	3.301	0.247	1	518.4	26.923079	R.NQQEGVCPEGSIDNSPVKWCALSHLER.T
TRFE_MOUSE	MK_SCX_50.3785.3785.3	3	5.625	0.573	1	1991.9	55.88235	K.HQTVLDNTEGKNPAEWAK.N
TRFE_MOUSE	MK_SCX_50.3828.3828.2	2	5.216	0.591	1	1901.8	76.47059	K.HQTVLDNTEGKNPAEWAK.N
TRIA1_MOUSE	MK_SCX_30.5803.5803.2	2	3.661	0.333	1	763.5	80.769226	K.EIPIEGLEFMGHGK.E
TRPS1_MOUSE	MK_SCX_18.3530.3530.2	2	5.932	0.592	1	1045.8	63.88889	R.NVASEGEGQTLEPTATESK.V
TRPV4_MOUSE	MK_SCX_16.9796.9796.2	2	2.583	0.217	1	359.5	38.636364	K.GVPNPIDLLESTLYESSVVPGPK.K
TRPV4_MOUSE	MK_SCX_36.5721.5721.3	3	3.184	0.305	1	682.8	42.307693	K.KAPMDSLFDYGTYR.H
TRPV6_MOUSE	MK_SCX_41.4613.4613.2	2	2.073	0.181	1	370.1	59.090908	K.ENDVQALSKLLK.F
TRPV6_MOUSE	MK_SCX_43.7322.7322.3	3	3.377	0.24	1	355.3	20.535715	R.FFGQTILGGPFHVIIITYAFM*VLVTMVMR.L
TRXR1_MOUSE	MK_SCX_18.7172.7172.2	2	5.248	0.614	1	1577	82.14286	K.VLVLDFVTPTPLGTR.W
TRXR1_MOUSE	MK_SCX_23.8731.8731.3	3	5.588	0.599	1	481.2	26.923079	K.TGKIPVTDEEQTNVPYIYAIGDILEGK.L
TRXR1_MOUSE	MK_SCX_32.5035.5035.3	3	3.286	0.392	1	397.1	32.8125	R.QFVPTKIEQIEAGTPGR.L
TRY2_MOUSE	MK_SCX_26.5431.5431.3	3	3.82	0.207	1	2014.5	44.736843	R.LGEHNINVLEGNEQFVDSAK.I
TS101_MOUSE	MK_SCX_15.6480.6480.2	2	2.167	0.189	1	404.1	38.095238	K.MENQSENNDIDEVIIPTAPLYK.Q
TS101_MOUSE	MK_SCX_48.3843.3843.3	3	4.044	0.224	1	1859.3	53.125	R.MKEEM*DGAQAELNALKR.T
TSYL4_MOUSE	MK_SCX_10.8381.8381.2	2	2.765	0.158	1	463.6	40	K.CAISVATGKEGEAGAAMQEKK.G
TSYL4_MOUSE	MK_SCX_32.15831.15831.3	3	3.707	0.252	1	612.2	33.333336	K.ALEACGAVGLGSQQMPGPKKTK.E
TTBK2_MOUSE	MK_SCX_23.5939.5939.2	2	2.578	0.138	1	388.2	50	K.LLQKKAYQPEIVK.L
TTBK2_MOUSE	MK_SCX_35.5850.5850.2	2	2.334	0.135	1	373	61.11111	K.ERYDHRLMLK.H
TTC1_MOUSE	MK_SCX_18.3287.3287.2	2	4.425	0.503	1	977.1	58.823532	K.VADPQEGESASPM*VSDPK.G
TTHY_MOUSE	MK_SCX_36.13217.13217.3	3	4.552	0.457	1	1180.8	36.363636	K.TLGISPFHEFADVVFTANDSGHR.H
TTHY_MOUSE	MK_SCX_39.3792.3792.2	2	2.839	0.231	1	649.7	65.38461	K.KTSEGSWEPFASGK.T
TULP3_MOUSE	MK_SCX_14.6938.6938.3	3	4.316	0.578	1	483.8	22.058825	K.AASETGASGVTAQQGDAQLGEVENLEDFAYSPAPR.G

TULP3_MOUSE	MK_SCX_14.6959.6959.2	2	5.239	0.622	1	1240.6	35.294117	K.AASETGASGVTAQQGDAQLGEVENLEDFAYSPAPR.G
TULP4_MOUSE	MK_SCX_18.11151.11151.2	2	2.48	0.268	1	328.7	41.666664	R.LDSRAEEGSVQAITEGKVK.K
TULP4_MOUSE	MK_SCX_58.7898.7898.2	2	2.191	0.143	1	439.6	81.25	R.SSKSPKLPR.I
TWF1_MOUSE	MK_SCX_18.6336.6336.2	2	5.962	0.49	1	1222.6	69.44444	K.YLLSQSSPAPLTAAEEELR.Q
TWF1_MOUSE	MK_SCX_19.3549.3549.2	2	5.334	0.365	1	2109.5	87.5	K.INEVQTDVSVDTK.H
TWF1_MOUSE	MK_SCX_2201.4679.4679.2	2	2.84	0.359	1	1297.8	93.75	K.SPLLEIVER.Q
TWF1_MOUSE	MK_SCX_40.4574.4574.3	3	3.307	0.418	1	1464.8	58.333332	K.HQTLQGVAFPISR.D
TWF1_MOUSE	MK_SCX_40.4582.4582.2	2	4.373	0.594	1	1944.4	87.5	K.HQTLQGVAFPISR.D
TWF1_MOUSE	MK_SCX_50.4650.4650.2	2	4.534	0.466	1	653.7	63.333332	K.EFGGGHIKDEVFGTVK.E
TX1B3_MOUSE	MK_SCX_19.4625.4625.2	2	4.297	0.556	1	1506.7	68.75	R.VSEGGPAEIAGLQIGDK.I
TXD12_MOUSE	MK_SCX_38.3602.3602.3	3	5.227	0.452	1	1625.2	51.785713	K.VRPEIINESGNPSYK.Y
TXLNA_MOUSE	MK_SCX_29.2786.2786.3	3	3.457	0.46	1	564.5	32.954548	R.QTAPGAEAEGSTSQAPGKTEGAR.A
TXLNA_MOUSE	MK_SCX_46.2932.2932.3	3	6.101	0.589	1	2317.5	45.833336	R.RPEATTASKEQGVESPGAQPASSPR.A
TXND1_MOUSE	MK_SCX_20_1.3452.3452.2	2	4.515	0.462	1	1977.5	90.909096	K.VDVTEQTGLSGR.F
TXND4_MOUSE	MK_SCX_16.5926.5926.2	2	3.951	0.565	1	931.1	64.28571	K.DDTESLEIFQNEVAR.Q
TXND4_MOUSE	MK_SCX_29.5871.5871.3	3	4.107	0.461	1	1156.2	44.11765	K.SNPVHEIQSLDEVTNLDR.S
TXND4_MOUSE	MK_SCX_29.5890.5890.2	2	6.215	0.561	1	2790.2	76.47059	K.SNPVHEIQSLDEVTNLDR.S
TXND4_MOUSE	MK_SCX_44.4502.4502.2	2	2.28	0.365	1	992.5	81.25	R.HMYVFGDFK.D
TXND4_MOUSE	MK_SCX_47.6542.6542.3	3	3.404	0.459	1	949.4	40	R.HM*YVFGDFKDVLIPGK.L
TXND4_MOUSE	MK_SCX_47.7171.7171.3	3	3.059	0.295	1	514.4	40	R.HMYVFGDFKDVLIPGK.L
TXNL1_MOUSE	MK_SCX_19.3937.3937.2	2	5.507	0.458	1	2398.5	82.14286	R.IDQYQGADAVGLEEK.I
TXNL1_MOUSE	MK_SCX_2201.3351.3351.2	2	3.231	0.265	1	920.5	93.75	R.SMDFEEAER.S
TXNL1_MOUSE	MK_SCX_2201.7588.7588.2	2	5.123	0.593	1	1808.9	56.81818	R.SEPTQALELTEDDIKEDGIVPLR.Y
TXNL1_MOUSE	MK_SCX_23.7172.7172.3	3	4.087	0.516	1	378.4	32.954548	R.SEPTQALELTEDDIKEDGIVPLR.Y
TXNL1_MOUSE	MK_SCX_25.4921.4921.2	2	2.741	0.13	1	575.5	91.66667	K.IFINLPR.S
TXNL1_MOUSE	MK_SCX_27.4724.4724.3	3	4.328	0.398	1	897.8	39.285713	-.VGVKPVGSDPDFQPELSGAGSR.L
TXNL1_MOUSE	MK_SCX_27.4745.4745.2	2	5.312	0.654	1	946.4	61.904762	-.VGVKPVGSDPDFQPELSGAGSR.L
TXNL1_MOUSE	MK_SCX_31.3277.3277.3	3	3.09	0.289	1	477	37.5	K.QHLENDPGSNEDADIPK.G
TXNL1_MOUSE	MK_SCX_48.3313.3313.3	3	5.327	0.386	1	2148.1	52.77778	K.IKQHLENDPGSNEDADIPK.G
TXNL2_MOUSE	MK_SCX_14.6551.6551.2	2	2.26	0.147	1	334.1	45	K.ASVM*LFM*KGNK.Q
TXNL2_MOUSE	MK_SCX_20_1.9147.9147.2	2	3.962	0.425	1	1102.7	79.16667	K.YEISSVPTFLFFK.N
TXNL2_MOUSE	MK_SCX_21.7002.7002.2	2	4.431	0.565	1	675	73.07692	K.TFSNWPTYPQLYVR.G
TXNL5_MOUSE	MK_SCX_24.4943.4943.2	2	3.572	0.535	1	634	88.88889	K.TIFAYFSGSK.D
TXNL5_MOUSE	MK_SCX_32.4747.4747.2	2	3.45	0.344	1	898.9	60.714287	K.TIFAYFSGSKDTEGK.S
TXNL5_MOUSE	MK_SCX_36.5335.5335.2	2	3.306	0.238	1	761.3	75	K.LKITAVPTLLK.Y
TXNL5_MOUSE	MK_SCX_51.7428.7428.3	3	4.674	0.523	1	962	30.769232	K.HVTEDCVFIYCQVGDKPYWKDPNNDFR.Q
TXNL5_MOUSE	MK_SCX_53.7605.7605.3	3	4.863	0.601	1	1166.4	30.833334	R.EGLKHVTEDCVFIYCQVGDKPYWKDPNNDFR.Q
TYB4_MOUSE	MK_SCX_41.3372.3372.3	3	5.31	0.553	1	721.6	41.666664	K.TETQEKNPLPSKETIEQEK.Q
TYB4_MOUSE	MK_SCX_42.3468.3468.2	2	4.998	0.53	1	1343.2	61.11111	K.TETQEKNPLPSKETIEQEK.Q
U235_MOUSE	MK_SCX_18.3514.3514.2	2	2.704	0.433	1	438.8	59.375	K.EPETAPPAAGPVATDPK.G
U235_MOUSE	MK_SCX_28.3451.3451.3	3	3.415	0.363	1	601.1	37.5	K.NQTKEPETAPPAAGPVATDPK.G
U315_MOUSE	MK_SCX_20_1.6169.6169.2	2	3.696	0.259	1	773.6	83.33333	R.INPVEFNPEFVAR.M
U315_MOUSE	MK_SCX_53.3637.3637.2	2	2.867	0.41	1	1090	77.27273	K.LLTHNLLSSHVR.G
U33K_MOUSE	MK_SCX_17.3652.3652.2	2	3.2	0.577	1	349.3	40.476192	R.SSPPATDPGPVPSSPSQEPPTK.R
U33K_MOUSE	MK_SCX_17.6276.6276.2	2	5.483	0.635	1	1125.2	72.22222	R.GEEPGQDQDPVQLLSGFPR.R
U33K_MOUSE	MK_SCX_23.4227.4227.3	3	3.182	0.484	1	724.2	31.25	R.EPTPSEQVGPEGSGSAAGESRPILTEEER.Q
UAP56_MOUSE	MK_SCX_25.6173.6173.3	3	3.423	0.505	1	478.2	33.333336	R.VNIAFNYDMPEDSDTYLHR.V
UAP56_MOUSE	MK_SCX_25.6210.6210.2	2	5.154	0.652	1	1502	69.44444	R.VNIAFNYDMPEDSDTYLHR.V
UB2L3_MOUSE	MK_SCX_2201.11335.11335.3	3	4.103	0.34	1	579.1	26.785713	R.NIQVDEANLLTWQGLIVPDNPPYDKGAFR.I
UB2L3_MOUSE	MK_SCX_24.10436.10436.3	3	3.809	0.386	1	555.4	30.952381	K.TDQVIQSLIALVNDPQPEHPLR.A
UB2L3_MOUSE	MK_SCX_29.6618.6618.3	3	3.568	0.407	1	653.2	44.642857	R.IEINFPAEYPFKPPK.I

UB2L3_MOUSE	MK_SCX_30.6565.6565.2	2	4.179	0.444	1	1492.4	78.57143	R.IEINFPAEYPFKPPK.I
UB2V1_MOUSE	MK_SCX_19.11047.11047.3	3	4.699	0.502	1	659.5	27.5	R.LLEELEEGQKGVGDGTVSWGLEDDEDMTLTR.W
UB2V1_MOUSE	MK_SCX_19.3793.3793.2	2	2.958	0.322	1	1718.4	76.666664	R.VNMSGVSSSNGVVDPR.A
UB2V1_MOUSE	MK_SCX_20_1.3336.3336.2	2	3.257	0.256	1	1099	70	R.VNM*SGVSSSNGVVDPR.A
UB2V1_MOUSE	MK_SCX_24.4789.4789.2	2	3.719	0.499	1	1306.2	94.44444	R.WTGMIIGPPR.T
UB2V2_MOUSE	MK_SCX_20_1.3256.3256.2	2	3.416	0.357	1	1121.2	60.000004	K.INM*NGINNSSGM*VDAR.S
UBC1_MOUSE	MK_SCX_41.7088.7088.3	3	4.338	0.529	1	1613.2	44.444447	R.YQLEIKIPETYPFNPPKVR.F
UBC12_MOUSE	MK_SCX_24.4381.4381.2	2	3.251	0.498	1	1029.1	85	R.GGYIGSTYFER.C
UBE1_MOUSE	MK_SCX_14.4420.4420.2	2	3.585	0.237	1	1368	73.07692	K.NGSEADIDESLYSR.Q
UBE1_MOUSE	MK_SCX_15.8892.8892.2	2	5.464	0.569	1	1447.8	56.81818	R.IYDDDFFQNLDGVANALDNIDAR.M
UBE1_MOUSE	MK_SCX_15.8909.8909.3	3	3.825	0.358	1	1070.1	35.227272	R.IYDDDFFQNLDGVANALDNIDAR.M
UBE1_MOUSE	MK_SCX_17.6565.6565.2	2	3.089	0.365	1	325	35.294117	K.SLPASLVEPDFVM*TDFAK.Y
UBE1_MOUSE	MK_SCX_32.4228.4228.3	3	3.382	0.426	1	354.5	36.666668	R.SPPSVKQNSLDEDLIR.K
UBE1X_MOUSE	MK_SCX_19.5920.5920.2	2	2.593	0.184	1	477.2	50	R.LAGTQPLEVLEAVQR.S
UBE1X_MOUSE	MK_SCX_20_1.3560.3560.2	2	2.317	0.331	1	490.4	65	R.LDQPM*TEIVSR.V
UBE1X_MOUSE	MK_SCX_32.4881.4881.2	2	2.579	0.42	1	378	54.166668	K.ATLPSPDKLPGFK.M
UBE2N_MOUSE	MK_SCX_17.6087.6087.2	2	4.481	0.539	1	634.1	67.85714	K.LELFLPEEYPM*AAPK.V
UBE2N_MOUSE	MK_SCX_17.6551.6551.2	2	4.684	0.453	1	758.5	67.85714	K.LELFLPEEYPMAAPK.V
UBE2N_MOUSE	MK_SCX_2201.2943.2943.2	2	3.045	0.291	1	1217.5	85	K.TNEAQAIETAR.A
UBE2N_MOUSE	MK_SCX_26.4383.4383.3	3	3.253	0.406	1	709.6	40.27778	R.LLAEPVPGIKAEPDESNAR.Y
UBE2N_MOUSE	MK_SCX_26.4400.4400.2	2	5.429	0.665	1	1105.8	63.88889	R.LLAEPVPGIKAEPDESNAR.Y
UBE2N_MOUSE	MK_SCX_30.6540.6540.3	3	3.791	0.497	1	1344.3	43.421055	R.YFHVVIAGPQDSPFEGGTFK.L
UBE2N_MOUSE	MK_SCX_30.6566.6566.2	2	5.616	0.648	1	1622.1	68.42105	R.YFHVVIAGPQDSPFEGGTFK.L
UBE3A_MOUSE	MK_SCX_13.7347.7347.2	2	2.148	0.179	1	522	59.090908	R.M*M*ETFQQLITYK.V
UBE3A_MOUSE	MK_SCX_2201.3294.3294.2	2	2.487	0.218	1	621.1	68.181816	K.DFKDVIYLTEEK.V
UBIQ_MOUSE	MK_SCX_18.5203.5203.2	2	3.491	0.245	1	745.7	60.000004	K.TITLEVEPSDTIENVK.A
UBIQ_MOUSE	MK_SCX_26.4944.4944.3	3	3.176	0.407	1	761.3	44.11765	K.TITLEVEPSDTIENVKAK.I
UBIQ_MOUSE	MK_SCX_26.4955.4955.2	2	6.045	0.485	1	777.3	61.764706	K.TITLEVEPSDTIENVKAK.I
UBIQ_MOUSE	MK_SCX_42.5668.5668.3	3	3.983	0.514	1	463.7	35.294117	R.TLSDYNIQKESTLHLVLR.L
UBL4A_MOUSE	MK_SCX_35.4179.4179.3	3	3.782	0.404	1	1513.7	56.81818	R.FLHPEVTEAMEK.G
UBP10_MOUSE	MK_SCX_28.5431.5431.3	3	3.762	0.194	1	1398.6	50	R.DIRPGAAFEPTYIYR.L
UBP10_MOUSE	MK_SCX_28.5450.5450.2	2	2.687	0.408	1	412.7	67.85714	R.DIRPGAAFEPTYIYR.L
UBP14_MOUSE	MK_SCX_16.9835.9835.2	2	2.573	0.237	1	343.8	42.5	K.WGKEKFEGVELNTDEPPMVFK.A
UBP14_MOUSE	MK_SCX_18.6288.6288.2	2	4.724	0.672	1	1534.3	73.333336	K.FEGVELNTDEPPMVFK.A
UBP14_MOUSE	MK_SCX_21.5236.5236.2	2	3.075	0.447	1	572.4	75	K.AQLFALTGVQPAR.Q
UBP14_MOUSE	MK_SCX_2201.3764.3764.3	3	6.219	0.56	1	2376	43.47826	K.LEAIEDDSGRETDSSSAPAVTPSK.K
UBP2L_MOUSE	MK_SCX_15.6246.6246.3	3	4.309	0.533	1	849	30.172413	K.TPSSMENDSSNLDPSQAPSLAQPLVFSNSK.Q
UBP2L_MOUSE	MK_SCX_15.6247.6247.2	2	4.669	0.607	1	710	43.103447	K.TPSSMENDSSNLDPSQAPSLAQPLVFSNSK.Q
UBP2L_MOUSE	MK_SCX_16.8051.8051.2	2	4.892	0.556	1	497.2	55.263157	R.FPLDYYSIPFPTPTTPLTGR.D
UBP2L_MOUSE	MK_SCX_17.4078.4078.2	2	4.667	0.49	1	1479.8	78.57143	R.DGSLASNPYSGDLTK.F
UBP2L_MOUSE	MK_SCX_17.6122.6122.2	2	4.683	0.567	1	828.8	58.69565	K.IFTASNVSSVPLPAENVTITAGQR.I
UBP2L_MOUSE	MK_SCX_18.3130.3130.3	3	5.37	0.555	1	1767.9	42.708336	K.STSAPQMSPGSSDNQSSSPQPAQQK.L
UBP2L_MOUSE	MK_SCX_2201.4868.4868.2	2	3.263	0.304	1	467.4	57.692307	K.GGSTTGSQFLEQFK.T
UBP2L_MOUSE	MK_SCX_2201.6784.6784.2	2	2.388	0.352	1	736	81.25	R.IDLAVLLGK.T
UBP2L_MOUSE	MK_SCX_26.3465.3465.3	3	3.288	0.269	1	367.9	27.884615	K.STSAPQMSPGSSDNQSSSPQPAQQKLK.Q
UBP2L_MOUSE	MK_SCX_26.6237.6237.2	2	4.722	0.432	1	1017.6	55.263157	R.AINVLLEGNPDTHSWEMVGK.K
UBP2L_MOUSE	MK_SCX_51.3386.3386.3	3	4.376	0.532	1	1400.9	44.11765	R.RYPSSISSSPQKDLTQAK.N
UBP47_MOUSE	MK_SCX_27.4161.4161.3	3	4.08	0.399	1	697.5	33.333336	R.ELEQHIQTSDPENFQSEER.S
UBP5_MOUSE	MK_SCX_17.4900.4900.2	2	5.4	0.583	1	1003.4	69.44444	K.IFQNAPTDPTQDFSTQVAK.L
UBP8_MOUSE	MK_SCX_30.3064.3064.3	3	4.835	0.445	1	1252.5	44.444447	K.TEDRELSADGAQEATGTQR.Q
UBP8_MOUSE	MK_SCX_32.7225.7225.2	2	2.302	0.196	1	505	57.14286	K.SEGAAAAERGAITAK.E

UBQL1_MOUSE	MK_SCX_15.9210.9210.3	3	6.149	0.539	1	1390.9	34.848484	R.SMLQSLSQNPDLAAQMMLNNPLFAGNPQLQEQMR.Q
UBQL1_MOUSE	MK_SCX_33.4223.4223.3	3	3.477	0.255	1	836.6	48.214287	R.NPEISHM*LNNPDIMR.Q
UBQL1_MOUSE	MK_SCX_33.5083.5083.3	3	5.024	0.413	1	1225.7	53.571426	R.NPEISHMLNNPDIMR.Q
UBQL1_MOUSE	MK_SCX_33.5110.5110.2	2	5.404	0.593	1	2135.5	78.57143	R.NPEISHMLNNPDIMR.Q
UBQL2_MOUSE	MK_SCX_32.5477.5477.3	3	5.292	0.409	1	1104.8	53.571426	R.NPEISHLLNNPDIMR.Q
UBR1_MOUSE	MK_SCX_17.5356.5356.2	2	3.572	0.218	1	737.9	50	R.NLPENENNETGLENVINK.V
UBXD2_MOUSE	MK_SCX_17.5793.5793.2	2	5.934	0.659	1	1001.7	60.000004	R.LPDGSSFTNQFPSDAPLEEAR.Q
UBXD2_MOUSE	MK_SCX_25.7628.7628.3	3	4.909	0.485	1	1619.6	41.666664	R.IQFRLPDGSSFTNQFPSDAPLEEAR.Q
UCHL1_MOUSE	MK_SCX_27.5664.5664.2	2	5.129	0.621	1	943.3	57.5	R.MPFPVNHGASSEDSLLQDAAK.V
UCHL1_MOUSE	MK_SCX_27.5750.5750.3	3	4.367	0.487	1	1376.3	40	R.MPFPVNHGASSEDSLLQDAAK.V
UCHL1_MOUSE	MK_SCX_31.5714.5714.3	3	4.467	0.286	1	2195.4	57.14286	-.MQLKPMEINPEMLNK.V
UCHL1_MOUSE	MK_SCX_31.5726.5726.2	2	5.316	0.342	1	1482.6	85.71429	-.MQLKPMEINPEMLNK.V
UCHL3_MOUSE	MK_SCX_20_1.3820.3820.2	2	4.226	0.511	1	1738.4	91.66667	K.FLEESVSM*SPEER.A
UCHL3_MOUSE	MK_SCX_40.4311.4311.3	3	3.199	0.279	1	1018.1	50	R.AKFLENYDAIR.V
UCHL3_MOUSE	MK_SCX_43.2867.2867.3	3	4.723	0.465	1	1604	44.736843	R.VTHETSAHEGQTEAPSIDEK.V
UCHL5_MOUSE	MK_SCX_31.4775.4775.2	2	4.026	0.415	1	816.6	69.230774	K.TLAEHQQLIPLVEK.A
UCR10_MOUSE	MK_SCX_25.5304.5304.2	2	2.404	0.303	1	542	91.66667	R.LYSLLFR.R
UCR10_MOUSE	MK_SCX_28.4987.4987.3	3	3.45	0.447	1	555.4	45.3125	R.AFDQGADAIYEHINEGK.L
UCR10_MOUSE	MK_SCX_28.4997.4997.2	2	5.488	0.537	1	2130.4	78.125	R.AFDQGADAIYEHINEGK.L
UCR6_MOUSE	MK_SCX_23.5198.5198.2	2	2.048	0.162	1	612.3	78.57143	K.FYLEPYLK.E
UCR6_MOUSE	MK_SCX_26.4439.4439.2	2	3.533	0.49	1	955.3	88.88889	K.WYYNAAGFNK.L
UCR6_MOUSE	MK_SCX_30.5458.5458.3	3	4.915	0.489	1	1329.1	58.333332	K.YEEDKFYLEPYLK.E
UCR6_MOUSE	MK_SCX_30.5571.5571.2	2	5.485	0.308	1	2578.4	91.66667	K.YEEDKFYLEPYLK.E
UCR6_MOUSE	MK_SCX_35.3991.3991.3	3	4.607	0.524	1	1984.5	57.14286	R.DDTLHETEDVKEAIR.R
UCR6_MOUSE	MK_SCX_35.4077.4077.2	2	5.491	0.53	1	1764.8	71.42857	R.DDTLHETEDVKEAIR.R
UCR6_MOUSE	MK_SCX_37.7440.7440.3	3	5.226	0.528	1	2338	50	K.DQWTKYEEDKFYLEPYLK.E
UCR6_MOUSE	MK_SCX_37.7656.7656.2	2	4.859	0.509	1	992.8	58.823532	K.DQWTKYEEDKFYLEPYLK.E
UCR6_MOUSE	MK_SCX_41.4087.4087.3	3	3.9	0.429	1	619.7	43.333332	K.LGLMRDDTLHETEDVK.E
UCR6_MOUSE	MK_SCX_41.4107.4107.2	2	4.355	0.453	1	1219	63.333332	K.LGLMRDDTLHETEDVK.E
UCR6_MOUSE	MK_SCX_41.7260.7260.2	2	5.61	0.585	1	1569.7	75	K.YEEDKFYLEPYLKEVIR.E
UCR6_MOUSE	MK_SCX_41.7274.7274.3	3	5.603	0.551	1	2006.3	51.5625	K.YEEDKFYLEPYLKEVIR.E
UCR6_MOUSE	MK_SCX_43.3909.3909.3	3	3.5	0.339	1	784.8	52.77778	R.RLPEDLYNDR.M
UCR6_MOUSE	MK_SCX_43.3943.3943.2	2	3.322	0.415	1	784.9	77.77778	R.RLPEDLYNDR.M
UCR6_MOUSE	MK_SCX_48.4012.4012.2	2	3.076	0.428	1	983	80	R.KWYYNAAGFNK.L
UCR6_MOUSE	MK_SCX_49.4090.4090.3	3	3.479	0.354	1	1100.5	62.5	R.KWYYNAAGFNK.L
UCR6_MOUSE	MK_SCX_50.4546.4546.3	3	4.758	0.487	1	1318.9	47.368423	K.LGLM*RDDTLHETEDVKEAIR.R
UCR6_MOUSE	MK_SCX_50.4999.4999.2	2	5.024	0.562	1	1556.4	63.15789	K.LGLMRDDTLHETEDVKEAIR.R
UCR6_MOUSE	MK_SCX_50.5026.5026.3	3	5.834	0.579	1	2465.1	46.05263	K.LGLMRDDTLHETEDVKEAIR.R
UCR6_MOUSE	MK_SCX_52.3589.3589.3	3	3.328	0.461	1	916	40	R.DDTLHETEDVKEAIRR.L
UCRH_MOUSE	MK_SCX_17.5281.5281.2	2	4.984	0.518	1	1115.1	78.57143	K.EEEEEELVDPLTTVR.E
UCRH_MOUSE	MK_SCX_18.7539.7539.2	2	5.455	0.647	1	2446.7	67.64706	R.SQTEEDCTEELFDFLHAR.D
UCRH_MOUSE	MK_SCX_2201.5862.5862.3	3	4.562	0.49	1	809.5	31.52174	K.M*LTGSGDPKEEEEEELVDPLTTVR.E
UCRH_MOUSE	MK_SCX_2201.6271.6271.2	2	5.257	0.497	1	821	47.826088	K.MLTGSGDPKEEEEEELVDPLTTVR.E
UCRH_MOUSE	MK_SCX_35.9783.9783.3	3	6.051	0.548	1	1185.9	34.375	R.KMLTGSGDPKEEEEEELVDPLTTVR.E
UCRH_MOUSE	MK_SCX_43.6627.6627.3	3	3.567	0.412	1	365	26	K.EEEEEELVDPLTTVREHCEQLEKCVK.A
UCRI_MOUSE	MK_SCX_18.13039.13039.2	2	5.75	0.66	1	1390.9	65	K.NVVSQFVSSMSASADVLAMSK.I
UCRI_MOUSE	MK_SCX_18.15003.15003.3	3	3.675	0.473	1	710.3	40	K.NVVSQFVSSMSASADVLAMSK.I
UCRI_MOUSE	MK_SCX_2201.2456.2456.2	2	2.092	0.185	1	533.9	78.57143	K.LSDIPEGK.N
UCRI_MOUSE	MK_SCX_2201.4479.4479.2	2	3.476	0.367	1	886.9	83.33333	R.SGPFAPVLSATSR.G
UCRI_MOUSE	MK_SCX_24.8572.8572.3	3	3.032	0.171	1	810.2	30.000002	R.GVAGALRPLLQGAVPAASEPPVLDVK.R
UCRI_MOUSE	MK_SCX_25.6258.6258.2	2	4.028	0.448	1	570.5	43.75	R.ESLSGQAAARPLVATVGLNVPASVR.F

UCRI_MOUSE	MK_SCX_32.5408.5408.3	3	3.609	0.489	1	537.2	28.57143	K.EIDQEAAVEVSQLRDPQHDLDR.V
UCRI_MOUSE	MK_SCX_50.4024.4024.3	3	3.381	0.319	1	888.9	35.9375	R.TKKEIDQEAAVEVSQLR.D
UCRI_MOUSE	MK_SCX_50.5103.5103.3	3	4.381	0.452	1	689.8	29.545454	K.KEIDQEAAVEVSQLRDPQHDLDR.V
UCRQ_MOUSE	MK_SCX_39.5467.5467.2	2	4.995	0.551	1	1856.7	91.66667	R.HVISYSLSPFEQR.A
UCRQ_MOUSE	MK_SCX_39.5499.5499.3	3	3.976	0.332	1	836	47.916664	R.HVISYSLSPFEQR.A
UCRQ_MOUSE	MK_SCX_50.2645.2645.2	2	2.662	0.207	1	531.3	75	-.GREFGNLAR.I
UCRQ_MOUSE	MK_SCX_54.4800.4800.3	3	5.214	0.303	1	1702.4	57.14286	R.IRHVISYSLSPFEQR.A
UCRQ_MOUSE	MK_SCX_54.4819.4819.2	2	5.126	0.524	1	1328.5	75	R.IRHVISYSLSPFEQR.A
UD11_MOUSE	MK_SCX_17.9640.9640.2	2	4.897	0.5	1	1032.2	55	R.GAGVTLNVLEM*TADDLENALK.T
UD11_MOUSE	MK_SCX_17.9833.9833.2	2	3.285	0.133	1	424.7	40	R.GAGVTLNVLEMTADDLENALK.T
UD11_MOUSE	MK_SCX_25.4616.4616.2	2	2.677	0.381	1	488.4	85.71429	R.IPQTVLWR.Y
UD11_MOUSE	MK_SCX_29.8168.8168.3	3	3.982	0.43	1	1120.1	39.705883	K.AM*EIAEALGRIPQTVLWR.Y
UD11_MOUSE	MK_SCX_29.9030.9030.3	3	3.643	0.365	1	1018.6	39.705883	K.AMEIAEALGRIPQTVLWR.Y
UD11_MOUSE	MK_SCX_36.4323.4323.2	2	2.822	0.407	2	329	72.72727	K.WLPQNDLIGHPK.T
UDB5_MOUSE	MK_SCX_17.9460.9460.2	2	3.965	0.495	1	1164	62.5	K.SDVLNALEEVIENPFYK.K
UDB5_MOUSE	MK_SCX_2201.5129.5129.2	2	3.316	0.331	1	919.1	93.75	K.IILDELVQR.G
UDB5_MOUSE	MK_SCX_25.3596.3596.3	3	3.186	0.394	1	387.1	27.5	R.TMSKSDVLNALEEVIENPFYK.K
UDB5_MOUSE	MK_SCX_26.9316.9316.2	2	4.503	0.64	1	1308.3	70.588234	K.SDVLNALEEVIENPFYKK.N
UDB5_MOUSE	MK_SCX_45.5514.5514.3	3	3.376	0.432	1	607.8	39.705883	R.GHEVTVLRPSAYYVLDPK.K
UE1D1_MOUSE	MK_SCX_18.4144.4144.2	2	4.085	0.392	1	1211.8	63.333332	R.IQEM*SDEVLDSNPYSR.L
UE1D1_MOUSE	MK_SCX_18.4748.4748.2	2	5.164	0.613	1	2029	73.333336	R.IQEMSDEVLDSNPYSR.L
UE1D1_MOUSE	MK_SCX_18.5437.5437.3	3	3.201	0.195	1	433.2	23.863636	R.TRIQEM*SDEVLDSNPYSRLMALK.R
UFC1_MOUSE	MK_SCX_41.3804.3804.3	3	3.261	0.188	1	650.6	50	R.LKEEYQSLIR.Y
UFC1_MOUSE	MK_SCX_41.3828.3828.2	2	3.412	0.464	1	838.2	88.88889	R.LKEEYQSLIR.Y
UFM1_MOUSE	MK_SCX_19.6166.6166.2	2	2.988	0.588	1	356.5	60.714287	K.VLSVPESTPFTAVLK.F
UFM1_MOUSE	MK_SCX_20_1.9482.9482.3	3	7.07	0.658	1	2602.2	36.764706	K.FAAEEFKVPAATSAIITNDGIGINPAQTAGNVFLK.H
UGDH_MOUSE	MK_SCX_21.4776.4776.2	2	2.799	0.394	1	395.6	62.5	K.ILTTNTWSSELSK.L
UGDH_MOUSE	MK_SCX_2201.2731.2731.2	2	2.044	0.264	1	457.8	75	R.ESSSIYISK.Y
UGDH_MOUSE	MK_SCX_23.8269.8269.3	3	5.201	0.531	1	557.7	26.85185	R.IFDANTKPNLNLQVLSNPEFLAEGTAIK.D
UGDH_MOUSE	MK_SCX_24.4785.4785.2	2	5.74	0.585	1	977.4	63.15789	R.EQIVVDLSHPGVSADDQVSR.L
UGPA2_MOUSE	MK_SCX_17.12642.12642.2	2	2.325	0.21	1	310	42.105263	K.TLDGGLNVIQLETAVGAAIK.S
UGPA2_MOUSE	MK_SCX_23.5082.5082.2	2	3.818	0.562	1	1626.2	86.36364	K.SFENSLGINVPR.S
UGPA2_MOUSE	MK_SCX_41.4397.4397.2	2	2.451	0.216	1	589.1	72.22222	K.REFPTVPLVK.L
UK114_MOUSE	MK_SCX_15.7346.7346.3	3	4.698	0.459	1	939.4	35.576923	R.TIYISGQVGLDPSSGQLVPGGVVEEAK.Q
UK114_MOUSE	MK_SCX_15.7554.7554.2	2	5.47	0.552	1	689.4	46.153847	R.TIYISGQVGLDPSSGQLVPGGVVEEAK.Q
UK114_MOUSE	MK_SCX_17.6088.6088.2	2	4.858	0.597	1	1197.4	63.88889	K.TTVLLADM*NDFGTVNEIYK.T
UK114_MOUSE	MK_SCX_17.7664.7664.2	2	5.355	0.478	1	1949.7	66.66667	K.TTVLLADMNDFGTVNEIYK.T
UK114_MOUSE	MK_SCX_19.6142.6142.2	2	3.753	0.49	1	1403	73.07692	R.VEIEAIAVQGPFIK.A
UK114_MOUSE	MK_SCX_20_1.4431.4431.2	2	5.343	0.659	1	1113.1	76.666664	K.APAAIGPYSQAVQVDR.T
UK114_MOUSE	MK_SCX_20_1.4600.4600.3	3	4.649	0.475	1	1228.2	48.333332	K.APAAIGPYSQAVQVDR.T
UK114_MOUSE	MK_SCX_21.9995.9995.3	3	4.388	0.583	1	1026.1	32.142857	K.TTVLLADMNDFGTVNEIYKTYFQGSLPAR.A
UK114_MOUSE	MK_SCX_2201.3918.3918.2	2	3.095	0.382	1	844	77.77778	R.AAYQVAALPR.G
UK114_MOUSE	MK_SCX_25.4088.4088.2	2	3.694	0.446	1	1535.9	94.44444	K.TYFQGSLPAR.A
UK114_MOUSE	MK_SCX_27.4700.4700.3	3	6.761	0.512	1	3188	50	K.VISTTKAPAAIGPYSQAVQVDR.T
UK114_MOUSE	MK_SCX_27.4832.4832.2	2	6.173	0.595	1	1204.1	69.047615	K.VISTTKAPAAIGPYSQAVQVDR.T
UK114_MOUSE	MK_SCX_38.4563.4563.3	3	3.079	0.341	1	408.5	42.5	K.QALKNLGEILK.A
UK114_MOUSE	MK_SCX_43.4570.4570.3	3	7.839	0.63	1	4232.1	53.409092	R.KVISTTKAPAAIGPYSQAVQVDR.T
UK114_MOUSE	MK_SCX_43.4579.4579.2	2	5.613	0.557	1	1320.2	65.909096	R.KVISTTKAPAAIGPYSQAVQVDR.T
ULE1A_MOUSE	MK_SCX_2201.6846.6846.3	3	5.963	0.498	1	2559.3	38.88889	K.GLTM*LDHEQVSPEDPGAQFLIQTGSVGR.N
ULE1A_MOUSE	MK_SCX_2201.7306.7306.3	3	6.191	0.672	1	1726.8	34.25926	K.GLTMLDHEQVSPEDPGAQFLIQTGSVGR.N
UN84A_MOUSE	MK_SCX_48.4931.4931.3	3	4.801	0.459	1	1901.4	51.666664	R.SATVLRHPVLDESLIR.E

UQCR1_MOUSE	MK_SCX_13.4706.4706.2	2	3.506	0.421	1	800	73.07692	R.VYEEDAVPGLTPCR.F
UQCR1_MOUSE	MK_SCX_13.7067.7067.2	2	3.976	0.669	1	836	50	K.YFYDQCPAVAGYGPIEQLPDYNR.I
UQCR1_MOUSE	MK_SCX_15.7095.7095.2	2	2.799	0.467	1	560.7	57.5	K.VVELLADIVQNSSLEDSQIEK.E
UQCR1_MOUSE	MK_SCX_18.8560.8560.3	3	5.86	0.629	1	1886.1	27.631578	R.EM*QENDASM*QNVVFDYLHATAFQGTPLAQAVEGPSENVR.R
UQCR1_MOUSE	MK_SCX_18.9280.9280.3	3	6.626	0.144	1	1814.4	27.631578	R.EM*QENDASMQNVVFDYLHATAFQGTPLAQAVEGPSENVR.R
UQCR1_MOUSE	MK_SCX_18.9535.9535.3	3	7.003	0.693	1	2852.9	31.578945	R.EMQENDASMQNVVFDYLHATAFQGTPLAQAVEGPSENVR.R
UQCR1_MOUSE	MK_SCX_19.5007.5007.2	2	4.514	0.579	1	862.8	72.22222	R.NALVSHLDGTTPVCEDIGR.S
UQCR1_MOUSE	MK_SCX_20_1.8485.8485.3	3	4.042	0.45	1	339	27.777779	R.DICSKYFYDQCPAVAGYGPIEQLPDYNR.I
UQCR1_MOUSE	MK_SCX_23.7403.7403.3	3	5.166	0.576	1	1401.2	42.045452	K.VVELLADIVQNSSLEDSQIEKER.D
UQCR1_MOUSE	MK_SCX_25.4485.4485.2	2	3.126	0.528	1	965.6	93.75	R.IPLAEWESR.I
UQCR1_MOUSE	MK_SCX_27.13144.13144.2	2	3.55	0.357	1	747.2	52.63158	R.MVLAAAGGVEHQQLLDLAQK.H
UQCR1_MOUSE	MK_SCX_27.6133.6133.3	3	4.702	0.56	1	1301.5	43.421055	R.MVLAAAGGVEHQQLLDLAQK.H
UQCR1_MOUSE	MK_SCX_29.5113.5113.2	2	2.667	0.178	1	701.1	91.66667	R.SGMFWLR.F
UQCR1_MOUSE	MK_SCX_32.3951.3951.2	2	3.933	0.507	1	741.3	64.28571	K.EVESIGAHLNAYSTR.E
UQCR1_MOUSE	MK_SCX_40.7003.7003.3	3	3.309	0.366	1	628.6	40.27778	R.YETEKNNGAGYFLEHLAFK.G
UQCR1_MOUSE	MK_SCX_47.4486.4486.2	2	2.309	0.243	1	717.8	77.77778	R.RIPLAEWESR.I
UQCR1_MOUSE	MK_SCX_48.4204.4204.3	3	3.805	0.352	1	1708.9	63.88889	R.RIPLAEWESR.I
UQCR1_MOUSE	MK_SCX_54.7145.7145.3	3	5.953	0.6	1	2505.8	44.56522	K.NRPGNALEKEVESIGAHLNAYSTR.E
UQCR2_MOUSE	MK_SCX_14.5492.5492.2	2	3.111	0.517	1	691.9	66.66667	K.NALANPLYCPDYR.M
UQCR2_MOUSE	MK_SCX_17.5524.5524.2	2	3.879	0.428	1	658.9	58.333332	K.TSAAPGGVPLQPQDLEFTK.L
UQCR2_MOUSE	MK_SCX_17.7777.7777.2	2	3.864	0.499	1	1578.1	58.823532	K.LPNGLVIASLENYAPLSR.I
UQCR2_MOUSE	MK_SCX_18.9742.9742.3	3	4.751	0.684	1	558.9	26.38889	K.TSAAPGGVPLQPQDLEFTKLPNGLVIASLENYAPLSR.I
UQCR2_MOUSE	MK_SCX_20_1.3440.3440.2	2	5.135	0.627	1	1824.7	80	K.AVAQGNLSSADVQAAK.N
UQCR2_MOUSE	MK_SCX_20_1.3456.3456.3	3	4.896	0.563	1	896.5	55	K.AVAQGNLSSADVQAAK.N
UQCR2_MOUSE	MK_SCX_26.9394.9394.3	3	5.965	0.594	1	1440.9	29.605263	K.VKTSAAPGGVPLQPQDLEFTKLPNGLVIASLENYAPLSR.I
UQCR2_MOUSE	MK_SCX_29.5550.5550.3	3	3.799	0.37	1	522	28.75	K.VKTSAAPGGVPLQPQDLEFTK.L
UQCR2_MOUSE	MK_SCX_30.3885.3885.2	2	3.189	0.326	1	1757.4	84.61539	R.YEDSNNLGTSHLLR.L
UQCR2_MOUSE	MK_SCX_33.5144.5144.2	2	4.287	0.481	1	2199.1	92.30769	R.MALVGLGVSHSVLK.Q
UQCR2_MOUSE	MK_SCX_35.3806.3806.3	3	3.144	0.411	1	979.9	53.846157	R.YEDSNNLGTSHLLR.L
UQCR2_MOUSE	MK_SCX_35.3871.3871.2	2	3.706	0.565	1	1609.4	90	R.IIENLHDVAYK.N
UQCR2_MOUSE	MK_SCX_41.3483.3483.3	3	3.205	0.346	1	366.3	34.210526	K.AVAQGNLSSADVQAAKNKLK.A
UQCR2_MOUSE	MK_SCX_43.3599.3599.3	3	3.394	0.3	1	1197.3	44.642857	K.RGNNTTSLLSQSVAK.G
UQCR2_MOUSE	MK_SCX_43.3651.3651.2	2	3.596	0.385	1	1730.4	75	K.RGNNTTSLLSQSVAK.G
UQCR2_MOUSE	MK_SCX_46.5919.5919.3	3	4.551	0.605	1	1365.3	48.52941	K.ITSEELHYFVQNHFTSAR.M
UQCR2_MOUSE	MK_SCX_46.5953.5953.2	2	5.991	0.678	1	1725.6	64.70589	K.ITSEELHYFVQNHFTSAR.M
UQCR2_MOUSE	MK_SCX_47.4004.4004.3	3	3.081	0.192	1	1255.2	45.588234	K.AGSRYEDSNNLGTSHLLR.L
UROM_MOUSE	MK_SCX_17.3299.3299.2	2	3.009	0.261	1	763.8	50	R.TEDTTIQVTENGESSQAR.F
UROM_MOUSE	MK_SCX_19.6755.6755.2	2	2.58	0.352	1	325.7	41.17647	K.LVCQDPCNTYETLTEYWR.S
USP9X_MOUSE	MK_SCX_19.3286.3286.2	2	3.474	0.311	1	532.3	53.125	R.AQENYEGGEEVSPPQTK.G
USP9X_MOUSE	MK_SCX_34.3539.3539.3	3	3.094	0.371	1	693.6	41.07143	R.MYGRDNEDYDPQTVR.L
UTP15_MOUSE	MK_SCX_19.7466.7466.1	1	2.338	0.123	1	393.9	72.22222	K.FIVLQELVEK.E
VA0D_MOUSE	MK_SCX_2201.5017.5017.2	2	2.169	0.229	1	415.4	80	R.LYPEGLAQLAR.A
VA0D_MOUSE	MK_SCX_28.4408.4408.3	3	3.489	0.491	1	919.4	48.52941	K.LLFEGAGSNPGDKTLEDR.F
VAMP2_MOUSE	MK_SCX_19.4117.4117.2	2	5.426	0.634	1	1924.8	75	R.ADALQAGASQFETSAAK.L
VAMP2_MOUSE	MK_SCX_24.5473.5473.3	3	5.74	0.412	1	2274.1	40.217392	K.LSELDDRADALQAGASQFETSAAK.L
VAMP2_MOUSE	MK_SCX_34.5698.5698.3	3	3.079	0.244	1	505	37.5	R.RLQQTQAQVDEVVDIMR.V
VAMP3_MOUSE	MK_SCX_19.5232.5232.2	2	5.512	0.596	1	2943.9	86.666664	R.LQQTQNQVDEVVDIMR.V
VAMP8_MOUSE	MK_SCX_30.6647.6647.2	2	4.753	0.604	1	1427.3	63.88889	R.NLQSEVEGVKNIMTQNVER.I
VAPA_MOUSE	MK_SCX_21.4973.4973.2	2	4.475	0.425	1	1745.3	81.818184	K.GPFTDVVTTNLK.L
VAPA_MOUSE	MK_SCX_32.5172.5172.2	2	4.594	0.591	1	1033.5	84.61539	K.HEQILVLDPPSDLK.F
VAPA_MOUSE	MK_SCX_36.5475.5475.3	3	4.097	0.277	1	1779.5	55.76923	K.FKGPFTDVVTTNLK.L

VAPA_MOUSE	MK_SCX_36.5502.5502.2	2	5.038	0.43	1	1435.4	88.46153	K.FKGPFTDVVTTNLK.L
VAPA_MOUSE	MK_SCX_43.3958.3958.3	3	4.084	0.552	1	877.7	44.444447	K.QDGPLPKPHSVSLNDTETR.K
VAPB_MOUSE	MK_SCX_29.4443.4443.2	2	2.992	0.336	1	381.1	57.692307	K.VEQVLSLEPQHELK.F
VAPB_MOUSE	MK_SCX_29.4487.4487.3	3	3.825	0.281	1	1051.3	53.846157	K.VEQVLSLEPQHELK.F
VAPB_MOUSE	MK_SCX_38.5810.5810.3	3	4.201	0.338	1	1208	50	K.FRGPFTDVVTTNLK.L
VAPB_MOUSE	MK_SCX_38.5939.5939.2	2	4.463	0.458	1	2106.6	84.61539	K.FRGPFTDVVTTNLK.L
VASP_MOUSE	MK_SCX_21.7564.7564.2	2	3.069	0.403	1	1580.2	73.333336	R.STGGGLMEEMNAMLAR.R
VASP_MOUSE	MK_SCX_23.4541.4541.2	2	2.649	0.258	1	417.6	70.83333	R.WLPAGTGPQAFSR.V
VASP_MOUSE	MK_SCX_39.3787.3787.3	3	3.007	0.335	1	468.2	45.833336	R.VQIYHNPTANSFR.V
VASP_MOUSE	MK_SCX_39.3861.3861.2	2	3.468	0.487	1	894	79.16667	R.VQIYHNPTANSFR.V
VATA1_MOUSE	MK_SCX_18.5039.5039.2	2	5.215	0.573	1	1659	81.25	R.LAEMPADSGYPAYLGAR.L
VATA1_MOUSE	MK_SCX_19.4279.4279.2	2	4.636	0.562	1	1428	71.875	R.LAEM*PADSGYPAYLGAR.L
VATA1_MOUSE	MK_SCX_19.8462.8462.2	2	3.895	0.305	1	789.1	64.28571	K.ADYAQLLEDMQNAFR.S
VATA1_MOUSE	MK_SCX_20_1.4170.4170.2	2	4.853	0.565	1	1707.1	78.57143	R.TALVANTSNMPVAAR.E
VATA1_MOUSE	MK_SCX_23.6116.6116.2	2	3.39	0.35	1	1417	88.88889	K.FSMVQVWPVR.Q
VATA1_MOUSE	MK_SCX_28.3393.3393.3	3	3.459	0.222	1	1000.5	42.857143	R.DM*GYHVSMM*ADSTSR.W
VATA1_MOUSE	MK_SCX_29.12188.12188.2	2	2.804	0.386	1	428.3	57.14286	K.ASLAETDKITLEVAK.L
VATA1_MOUSE	MK_SCX_29.6452.6452.3	3	4.66	0.392	1	690.1	36.25	R.VGSHITGGDIYGIVNENSLIK.H
VATA1_MOUSE	MK_SCX_31.5540.5540.3	3	3.515	0.189	1	670.3	50	R.DIKWEFIPSK.N
VATA1_MOUSE	MK_SCX_31.5562.5562.2	2	2.729	0.47	1	862.3	77.77778	R.DIKWEFIPSK.N
VATA1_MOUSE	MK_SCX_33.6389.6389.3	3	4.893	0.453	1	1601.6	45.3125	K.IKADYAQLLEDM*QNAFR.S
VATA1_MOUSE	MK_SCX_33.8955.8955.3	3	5.191	0.416	1	1460.5	48.4375	K.IKADYAQLLEDMQNAFR.S
VATA1_MOUSE	MK_SCX_33.9043.9043.2	2	5.454	0.55	1	1786.5	78.125	K.IKADYAQLLEDMQNAFR.S
VATA1_MOUSE	MK_SCX_37.4355.4355.3	3	3.345	0.304	1	1040.6	52.272724	R.VGHSELVGEIIR.L
VATA1_MOUSE	MK_SCX_37.4369.4369.2	2	3.359	0.52	1	1447.1	86.36364	R.VGHSELVGEIIR.L
VATA1_MOUSE	MK_SCX_41.6596.6596.2	2	5.324	0.566	1	1187.6	65.625	R.ALDEYYDKHFTEFVPLR.T
VATA1_MOUSE	MK_SCX_41.6608.6608.3	3	4.183	0.535	1	1584.2	43.75	R.ALDEYYDKHFTEFVPLR.T
VATA1_MOUSE	MK_SCX_42.4356.4356.2	2	2.965	0.269	1	635.9	80	R.WAEALREISGR.L
VATA1_MOUSE	MK_SCX_44.4427.4427.2	2	2.028	0.292	1	346.6	75	K.HFTEFVPLR.T
VATB2_MOUSE	MK_SCX_14.9563.9563.3	3	4.278	0.243	1	367	24.305555	R.NGSITQIPILTMPNDDITHPIPDLTGYITEGQIYVDR.Q
VATB2_MOUSE	MK_SCX_16.12377.12377.2	2	3.724	0.36	1	432.3	45	K.AVVGEEALTSDDLLYLEFLQK.F
VATB2_MOUSE	MK_SCX_16.6321.6321.3	3	3.17	0.212	1	613.2	30.952381	R.IYPEEM*IQTGISAIDGM*NSIAR.G
VATB2_MOUSE	MK_SCX_16.7860.7860.2	2	5.45	0.643	1	1673.6	59.523808	R.IYPEEMIQTGISAIDGM*NSIAR.G
VATB2_MOUSE	MK_SCX_16.8583.8583.3	3	5.603	0.517	1	1337.6	36.904762	R.IYPEEMIQTGISAIDGMNSIAR.G
VATB2_MOUSE	MK_SCX_16.8603.8603.2	2	5.155	0.345	1	1806.6	59.523808	R.IYPEEMIQTGISAIDGMNSIAR.G
VATB2_MOUSE	MK_SCX_19.6259.6259.2	2	2.846	0.421	1	651	61.538464	R.QIYPPINVLPSLSR.L
VATB2_MOUSE	MK_SCX_19.6393.6393.2	2	4.749	0.563	1	853.6	80	R.GFPGYM*YTDLATIYER.A
VATB2_MOUSE	MK_SCX_19.7175.7175.2	2	5.31	0.529	1	1193.3	80	R.GFPGYMYTDLATIYER.A
VATB2_MOUSE	MK_SCX_2201.2553.2553.2	2	3.753	0.409	1	1315.9	80	R.SGQVLEVSGSK.A
VATB2_MOUSE	MK_SCX_2201.5121.5121.2	2	3.845	0.463	1	1186.6	86.36364	R.IPQSTLSEFYPR.D
VATB2_MOUSE	MK_SCX_25.4105.4105.2	2	2.5	0.204	1	500.3	75	K.NFITQGPYENR.T
VATB2_MOUSE	MK_SCX_27.6473.6473.2	2	2.139	0.16	1	483.2	53.333336	K.TVSGVNGPLVILDHVK.F
VATB2_MOUSE	MK_SCX_30.5228.5228.2	2	3.081	0.398	1	1310	73.07692	R.YAEIVHLTLPDGTK.R
VATB2_MOUSE	MK_SCX_36.7803.7803.3	3	3.18	0.496	1	333.3	34.375	R.RGFPGYMYTDLATIYER.A
VATB2_MOUSE	MK_SCX_41.5018.5018.3	3	4.026	0.379	1	2035.7	58.333332	K.RIPQSTLSEFYPR.D
VATB2_MOUSE	MK_SCX_42.4964.4964.2	2	3.467	0.542	1	1222.4	79.16667	K.RIPQSTLSEFYPR.D
VATB2_MOUSE	MK_SCX_45.5238.5238.3	3	4.802	0.492	1	777.8	44.642857	R.YAEIVHLTLPDGTKR.S
VATB2_MOUSE	MK_SCX_45.5239.5239.2	2	4.279	0.454	1	2369.4	82.14286	R.YAEIVHLTLPDGTKR.S
VATC_MOUSE	MK_SCX_31.4376.4376.2	2	2.815	0.366	1	575.6	50	R.ASAYNNLKGNLQNLER.K
VATC_MOUSE	MK_SCX_36.4332.4332.3	3	3.667	0.339	1	922.7	45	R.ASAYNNLKGNLQNLER.K
VATD_MOUSE	MK_SCX_13.5688.5688.2	2	2.374	0.141	1	378	46.666668	R.AAGEVMEPANLLAEEK.D

VATD_MOUSE	MK_SCX_19.5309.5309.2	2	4.923	0.576	1	2389.9	80	K.FTAGDFSTTVIQNVNK.A
VATD_MOUSE	MK_SCX_21.7288.7288.2	2	2.526	0.167	1	311.2	59.090908	R.TLAYIITELDER.E
VATD_MOUSE	MK_SCX_28.6182.6182.3	3	3.25	0.393	1	536.4	38.157894	K.FTAGDFSTTVIQNVNKAQVK.I
VATD_MOUSE	MK_SCX_36.4174.4174.3	3	3.53	0.558	1	597.2	47.5	R.VNAIEHVIIPR.I
VATD_MOUSE	MK_SCX_36.4188.4188.2	2	3.74	0.556	1	1974.9	90	R.VNAIEHVIIPR.I
VATD_MOUSE	MK_SCX_46.6969.6969.3	3	7.518	0.564	1	2347.8	38.392857	K.KDNVAGVTLPVFEHYHEGTDSYELTGLAR.G
VATD_MOUSE	MK_SCX_53.6748.6748.3	3	7.509	0.694	1	2171.7	36.666668	R.AKKDNVAGVTLPVFEHYHEGTDSYELTGLAR.G
VATD_MOUSE	MK_SCX_54.3808.3808.3	3	4.243	0.46	1	893.5	52.272724	R.RVNAIEHVIIPR.I
VATD_MOUSE	MK_SCX_54.3841.3841.2	2	3.774	0.521	1	1157.2	86.36364	R.RVNAIEHVIIPR.I
VATE_MOUSE	MK_SCX_16.7793.7793.1	1	3.2	0.395	1	825.1	72.72727	R.DDLITDLLNEAK.Q
VATE_MOUSE	MK_SCX_17.11151.11151.2	2	4.162	0.568	1	649.6	57.894737	R.YQVLLDGLVLQGLYQLLEPR.M
VATE_MOUSE	MK_SCX_19.4159.4159.2	2	4.176	0.518	1	823	66.66667	R.LDLIAQQM*M*PEVR.G
VATE_MOUSE	MK_SCX_19.4727.4727.2	2	3.441	0.122	1	796.2	66.66667	R.LDLIAQQMM*PEVR.G
VATE_MOUSE	MK_SCX_19.5629.5629.2	2	4.038	0.505	1	1225.3	83.33333	R.LDLIAQQMMPEVR.G
VATE_MOUSE	MK_SCX_24.3390.3390.2	2	3.681	0.347	1	1210.2	80	K.IQM*SNLMNQAR.L
VATE_MOUSE	MK_SCX_24.3603.3603.2	2	3.309	0.285	1	1371.4	85	K.IQMSNLM*NQAR.L
VATE_MOUSE	MK_SCX_24.4268.4268.2	2	4.116	0.431	1	1397.2	85	K.IQMSNLMNQAR.L
VATE_MOUSE	MK_SCX_24.4301.4301.3	3	3.422	0.149	1	761.6	52.499996	K.IQMSNLMNQAR.L
VATE_MOUSE	MK_SCX_31.7378.7378.3	3	5.728	0.412	1	3471.1	63.461536	R.ARDDLITDLLNEAK.Q
VATE_MOUSE	MK_SCX_32.7394.7394.2	2	4.896	0.351	1	2099.4	80.769226	R.ARDDLITDLLNEAK.Q
VATE_MOUSE	MK_SCX_39.3192.3192.3	3	3.217	0.264	1	1031	50	K.HM*M*AFIEQEANEK.A
VATE_MOUSE	MK_SCX_40.4020.4020.3	3	3.249	0.26	1	1168.6	59.375	R.LKIMEYYEK.K
VATE_MOUSE	MK_SCX_41.3991.3991.2	2	3.056	0.364	1	566.1	81.25	R.LKIMEYYEK.K
VATE_MOUSE	MK_SCX_41.4422.4422.2	2	3.997	0.586	1	978.9	83.33333	K.HMMAFIEQEANEK.A
VATE_MOUSE	MK_SCX_43.3006.3006.2	2	2.635	0.467	1	616.7	72.22222	K.IKVSNTLESR.L
VATE_MOUSE	MK_SCX_43.4033.4033.2	2	3.872	0.447	1	2609.3	90.909096	K.KIQMSNLMNQAR.L
VATE_MOUSE	MK_SCX_44.3904.3904.3	3	4.144	0.153	1	1234.6	56.81818	K.KIQMSNLMNQAR.L
VATE_MOUSE	MK_SCX_46.7021.7021.3	3	4.769	0.447	1	1967.4	55	R.ARDDLITDLLNEAKQR.L
VATF_MOUSE	MK_SCX_15.10556.10556.2	2	3.345	0.45	1	614.9	59.375	R.DDIGIILINQYIAEMVR.H
VATF_MOUSE	MK_SCX_15.15114.15114.3	3	4.224	0.288	1	443	25	K.LIAVIGDEDTVTGFLLGGIGELNK.N
VATF_MOUSE	MK_SCX_17.5874.5874.2	2	4.21	0.462	1	1227.7	86.36364	K.DTTINEIEDTFR.Q
VATF_MOUSE	MK_SCX_39.5497.5497.2	2	4.379	0.549	1	690.1	52.77778	R.SIPAVLEIPSKEHPYDAAK.D
VATF_MOUSE	MK_SCX_47.6292.6292.3	3	3.783	0.562	1	633.4	33.695652	R.SIPAVLEIPSKEHPYDAAKDSILR.R
VATG1_MOUSE	MK_SCX_21.3794.3794.2	2	4.178	0.366	1	1598.2	90	K.EEAQAEIEQYR.L
VATG1_MOUSE	MK_SCX_23.5263.5263.2	2	4.046	0.45	1	1442.2	77.27273	K.MTVLQNYFEQNR.D
VATG1_MOUSE	MK_SCX_31.3303.3303.3	3	3.797	0.522	1	549.3	32.894737	K.EAAALGSHGSCSSEVEKETR.E
VATG1_MOUSE	MK_SCX_36.3563.3563.2	2	4.06	0.514	1	730.3	69.230774	K.QAKEEAQAEIEQYR.L
VATG1_MOUSE	MK_SCX_36.3577.3577.3	3	4.435	0.514	1	1350.5	50	K.QAKEEAQAEIEQYR.L
VATH_MOUSE	MK_SCX_23.8187.8187.3	3	5.243	0.63	1	688.5	34.090908	K.LLEVSDDPQVLAVAAHDVGEYVR.H
VATH_MOUSE	MK_SCX_23.8238.8238.2	2	5.096	0.702	1	1272.7	56.81818	K.LLEVSDDPQVLAVAAHDVGEYVR.H
VATH_MOUSE	MK_SCX_24.6185.6185.3	3	4.572	0.514	1	600.2	30.952381	K.LGESVQDLSSFDEYSSELKSGR.L
VATH_MOUSE	MK_SCX_38.5107.5107.2	2	2.092	0.175	1	566.7	55	R.QEYALAM*IQCK.V
VATH_MOUSE	MK_SCX_39.3620.3620.3	3	3.922	0.248	1	967.9	58.333332	R.LNEKNYELLK.I
VDAC1_MOUSE	MK_SCX_13.4168.4168.2	2	3.106	0.449	1	808.5	81.818184	K.YQVDPDACFSAK.V
VDAC1_MOUSE	MK_SCX_18.14639.14639.3	3	3.732	0.311	1	426.1	37.5	K.WNTDNTLGTEITVEDQLAR.G
VDAC1_MOUSE	MK_SCX_18.6283.6283.2	2	2.995	0.325	1	419.4	53.125	K.LETAVNLAWTAGNSNTR.F
VDAC1_MOUSE	MK_SCX_18.6590.6590.2	2	5.758	0.586	1	1797.3	69.44444	K.WNTDNTLGTEITVEDQLAR.G
VDAC1_MOUSE	MK_SCX_19.3600.3600.2	2	5.647	0.616	1	2009.8	72.22222	K.SENGLEFTSSGSANTETTK.V
VDAC1_MOUSE	MK_SCX_21.4516.4516.2	2	4.382	0.605	1	1582.9	79.16667	K.LTFDSSFSPNTGK.K
VDAC1_MOUSE	MK_SCX_2201.3598.3598.2	2	3.549	0.442	1	1684.8	90	R.VTQSNFAVGYK.T
VDAC1_MOUSE	MK_SCX_2201.6038.6038.2	2	3.629	0.435	1	1097.9	88.88889	K.LTLSALLDGK.N

VDAC1_MOUSE	MK_SCX_23.5298.5298.2	2	3.829	0.391	1	1260.8	85	R.WTEYGLTFTEK.W
VDAC1_MOUSE	MK_SCX_25.15295.15295.3	3	6.297	0.56	1	2307.6	45.454548	K.TDEFQLHTNVNDGTEFGGSIYQK.V
VDAC1_MOUSE	MK_SCX_25.5709.5709.2	2	5.629	0.616	1	1440	59.090908	K.TDEFQLHTNVNDGTEFGGSIYQK.V
VDAC1_MOUSE	MK_SCX_29.3473.3473.3	3	4.587	0.477	1	887.6	35	K.TKSENGLEFTSSGSANTETTK.V
VDAC1_MOUSE	MK_SCX_29.5802.5802.3	3	4.214	0.143	1	459.1	31.578945	K.VNNSSLIGLGYTQTLKPGIK.L
VDAC1_MOUSE	MK_SCX_30.10623.10623.3	3	4.137	0.388	1	521.7	20.454546	R.VTQSNFAVGYKTDEFQLHTNVNDGTEFGGSIYQK.V
VDAC1_MOUSE	MK_SCX_30.5732.5732.2	2	5.59	0.439	1	890.2	55.263157	K.VNNSSLIGLGYTQTLKPGIK.L
VDAC1_MOUSE	MK_SCX_34.3831.3831.2	2	3.79	0.449	1	1408.3	73.07692	K.LTFDSSFSPNTGKK.N
VDAC1_MOUSE	MK_SCX_36.13280.13280.2	2	4.278	0.492	1	844	55.88235	K.KLETAVNLAWTAGNSNTR.F
VDAC1_MOUSE	MK_SCX_36.5121.5121.3	3	4.308	0.438	1	743.8	41.17647	K.KLETAVNLAWTAGNSNTR.F
VDAC1_MOUSE	MK_SCX_41.5827.5827.2	2	4.918	0.556	1	2824.4	91.66667	K.YRWTEYGLTFTEK.W
VDAC1_MOUSE	MK_SCX_41.5930.5930.3	3	4.75	0.361	1	2515.9	60.416668	K.YRWTEYGLTFTEK.W
VDAC1_MOUSE	MK_SCX_43.3594.3594.3	3	3.431	0.338	1	1481.3	60.416668	K.SRVTQSNFAVGYK.T
VDAC1_MOUSE	MK_SCX_46.5052.5052.2	2	6.06	0.526	1	2109.5	73.52941	K.LTLSALLDGKNVNAGGHK.L
VDAC1_MOUSE	MK_SCX_46.5056.5056.3	3	4.849	0.465	1	756.6	45.588234	K.LTLSALLDGKNVNAGGHK.L
VDAC1_MOUSE	MK_SCX_50.4775.4775.2	2	5.603	0.616	1	1973.2	71.875	R.GLKLTFDSSFSPNTGKK.N
VDAC1_MOUSE	MK_SCX_50.4783.4783.3	3	5.39	0.568	1	1596.3	48.4375	R.GLKLTFDSSFSPNTGKK.N
VDAC2_MOUSE	MK_SCX_21.4283.4283.2	2	3.155	0.459	1	1147.4	90.909096	K.YQLDPTASISAK.V
VDAC2_MOUSE	MK_SCX_2201.4911.4911.2	2	3.377	0.574	1	1083.3	88.88889	K.LTLSALVDGK.S
VDAC2_MOUSE	MK_SCX_31.5170.5170.2	2	4.941	0.437	1	521.3	44.736843	K.VNNSSLIGVGYTQTLRPGVK.L
VDAC2_MOUSE	MK_SCX_31.5178.5178.3	3	4.248	0.23	1	618.7	34.210526	K.VNNSSLIGVGYTQTLRPGVK.L
VDAC2_MOUSE	MK_SCX_31.7035.7035.2	2	3.796	0.405	1	1155.3	79.16667	R.DIFNKGFGFGLVK.L
VDAC2_MOUSE	MK_SCX_33.4311.4311.2	2	3.948	0.425	1	1112.9	69.230774	K.LTFDTTFSPNTGKK.S
VDAC3_MOUSE	MK_SCX_2201.6038.6038.2	2	3.629	0.435	1	1097.9	88.88889	K.LTLSALLDGK.N
VDAC3_MOUSE	MK_SCX_30.5405.5405.3	3	4.502	0.157	1	883.7	40.789474	K.VNNASLIGLGYTQTLRPGVK.L
VDAC3_MOUSE	MK_SCX_30.5431.5431.2	2	5.096	0.278	1	1110.1	57.894737	K.VNNASLIGLGYTQTLRPGVK.L
VDAC3_MOUSE	MK_SCX_31.5604.5604.2	2	3.772	0.217	1	956.1	69.230774	K.LTLDTIFVPNTGKK.S
VDAC3_MOUSE	MK_SCX_32.5131.5131.2	2	3.849	0.46	1	1161.4	83.33333	K.DVFNKGYGFGMVK.I
VIGLN_MOUSE	MK_SCX_17.3603.3603.2	2	2.303	0.319	1	334.8	46.666668	K.VATLNSEEENDPPTYK.D
VIGLN_MOUSE	MK_SCX_21.3652.3652.2	2	2.611	0.486	1	584.2	68.181816	R.LVGEIM*QETGTR.I
VIGLN_MOUSE	MK_SCX_21.4243.4243.2	2	3.036	0.422	1	1296.9	86.36364	R.LVGEIMQETGTR.I
VIGLN_MOUSE	MK_SCX_2201.3255.3255.2	2	2.403	0.275	1	1059.3	92.85714	K.LSVTVDPK.Y
VIGLN_MOUSE	MK_SCX_2201.5616.5616.3	3	4.753	0.499	1	776.1	37.5	K.VATLNSEEENDPPTYKDAFPPLPEK.A
VIGLN_MOUSE	MK_SCX_26.6629.6629.2	2	4.908	0.459	1	997	66.66667	R.DKFPEVIINFPDPAQK.S
VIGLN_MOUSE	MK_SCX_26.6632.6632.3	3	4.362	0.318	1	789.3	43.333332	R.DKFPEVIINFPDPAQK.S
VIGLN_MOUSE	MK_SCX_28.5131.5131.3	3	5.82	0.564	1	2098.3	50	R.TEIVFTGEKEQLAQAVAR.I
VIGLN_MOUSE	MK_SCX_28.5195.5195.2	2	5.459	0.562	1	2241.3	73.52941	R.TEIVFTGEKEQLAQAVAR.I
VIGLN_MOUSE	MK_SCX_30.6446.6446.2	2	4.055	0.483	1	1137	67.85714	K.ASVITQVFHVPLEER.K
VIGLN_MOUSE	MK_SCX_31.4253.4253.2	2	3.609	0.271	1	349	57.692307	K.IQIPRPDDPSNQIK.I
VIGLN_MOUSE	MK_SCX_32.5935.5935.3	3	3.794	0.565	1	434.9	34.375	K.ANSFTVSSVSAPSWLHR.F
VIGLN_MOUSE	MK_SCX_41.4493.4493.2	2	3.29	0.451	1	710.5	72.72727	K.AFHPFIAGPYNR.L
VIGLN_MOUSE	MK_SCX_44.10267.10267.2	2	2.324	0.401	1	341.4	50	R.DSTGARIIFPAAEDK.D
VILI_MOUSE	MK_SCX_15.8554.8554.2	2	5.165	0.645	1	950	46	K.VDVFTANTSLSSGPLPTFPLEQLVNK.S
VILI_MOUSE	MK_SCX_15.8762.8762.3	3	6.136	0.636	1	1311.9	39	K.VDVFTANTSLSSGPLPTFPLEQLVNK.S
VILI_MOUSE	MK_SCX_16.7045.7045.2	2	3.765	0.542	1	400.4	42	K.QYPPSTQVEVQNDGAESPIFQQLFQK.W
VILI_MOUSE	MK_SCX_16.7100.7100.3	3	4.575	0.437	1	1022.4	30.000002	K.QYPPSTQVEVQNDGAESPIFQQLFQK.W
VILI_MOUSE	MK_SCX_17.6647.6647.2	2	5.619	0.652	1	2104.3	65.789474	K.AELGNSGDWSQIADEVMSPK.V
VILI_MOUSE	MK_SCX_18.4952.4952.2	2	3.309	0.438	1	1211.9	86.36364	R.IEDLELVPVESK.W
VILI_MOUSE	MK_SCX_18.6894.6894.2	2	5.677	0.642	1	1394.7	72.22222	K.QVVVEGQEPANFWMALGGK.A
VILI_MOUSE	MK_SCX_20_1.3576.3576.2	2	4.003	0.508	1	1740.5	83.33333	K.AAISDSVVEPAAK.A
VILI_MOUSE	MK_SCX_20_1.5264.5264.2	2	3.768	0.404	1	1344.5	76.92308	R.ALGM*TPAAFSALPR.W

VILI_MOUSE	MK_SCX_21.4394.4394.2	2	3.039	0.445	1	381.8	62.5	K.MVDDGSGEVQVWR.I
VILI_MOUSE	MK_SCX_21.5043.5043.2	2	2.508	0.336	1	1017.1	72.72727	K.LIIQWNGPESNR.M
VILI_MOUSE	MK_SCX_21.5292.5292.2	2	3.32	0.249	1	1652.1	79.16667	R.ATSLNSNDVFILK.T
VILI_MOUSE	MK_SCX_21.5979.5979.2	2	4.41	0.635	1	1020.8	76.92308	R.ALGMTPAAFSALPR.W
VILI_MOUSE	MK_SCX_2201.2537.2537.2	2	3.256	0.436	1	1177.9	85	R.EVQGNESETFR.S
VILI_MOUSE	MK_SCX_2201.3376.3376.2	2	2.901	0.276	1	810.1	87.5	K.YNDEPVQIR.V
VILI_MOUSE	MK_SCX_2201.6903.6903.2	2	3.818	0.424	1	626.3	61.538464	K.GSLNITTPGIQIWR.I
VILI_MOUSE	MK_SCX_23.5474.5474.2	2	3.633	0.347	1	1207.2	72.72727	R.SGAMSQALNFIK.A
VILI_MOUSE	MK_SCX_26.6778.6778.2	2	4.781	0.511	1	828.9	42.592594	K.AKQYPPSTQVEVQNDGAESPIFQQLFQK.W
VILI_MOUSE	MK_SCX_26.6794.6794.3	3	7.388	0.567	1	2244	38.88889	K.AKQYPPSTQVEVQNDGAESPIFQQLFQK.W
VILI_MOUSE	MK_SCX_28.4110.4110.3	3	3.964	0.447	1	1775	50	K.FDALTM*HVQPQVAAQQK.M
VILI_MOUSE	MK_SCX_28.4593.4593.2	2	5.448	0.597	1	1549.4	65.625	K.FDALTMHVQPQVAAQQK.M
VILI_MOUSE	MK_SCX_28.4621.4621.3	3	4.576	0.438	1	1831.6	51.5625	K.FDALTMHVQPQVAAQQK.M
VILI_MOUSE	MK_SCX_32.10901.10901.3	3	5.673	0.45	1	2190.8	39.814816	R.TYVGVVDGEKEGDSPQLMAIMNHVLGPR.K
VILI_MOUSE	MK_SCX_32.4208.4208.2	2	3.731	0.351	1	630.1	66.66667	R.EVATRPLTQDLLK.H
VILI_MOUSE	MK_SCX_35.10527.10527.3	3	3.607	0.343	1	1133.8	48.076923	K.SFNRGDVFLLDLGK.L
VILI_MOUSE	MK_SCX_35.10693.10693.2	2	3.935	0.458	1	1167.5	73.07692	K.SFNRGDVFLLDLGK.L
VILI_MOUSE	MK_SCX_37.5726.5726.3	3	5.528	0.55	1	894.3	36.904762	K.VEQVKFDALTMHVQPQVAAQQK.M
VILI_MOUSE	MK_SCX_45.5247.5247.3	3	4.842	0.589	1	819.9	36.458336	K.SVEDLPEGVDPSRKEEHLSTEDFTR.A
VILI_MOUSE	MK_SCX_48.5090.5090.2	2	4.481	0.498	1	853	71.42857	R.VTMGKEPPHLMSIFK.G
VILI_MOUSE	MK_SCX_48.5141.5141.3	3	4.016	0.425	1	605.5	41.07143	R.VTMGKEPPHLMSIFK.G
VILI_MOUSE	MK_SCX_51.3764.3764.3	3	3.948	0.513	1	697.7	48.076923	K.LYHVSDSEGKLVVR.E
VILL_MOUSE	MK_SCX_31.7455.7455.3	3	4.349	0.552	1	1569.1	53.333336	R.KEFYLSDSDFQDIFGK.S
VILL_MOUSE	MK_SCX_31.7532.7532.2	2	4.031	0.607	1	391	63.333332	R.KEFYLSDSDFQDIFGK.S
VIME_MOUSE	MK_SCX_16.9756.9756.2	2	5.125	0.607	1	2197.2	72.22222	R.LLQDSVDFSLADAINTEFK.N
VIME_MOUSE	MK_SCX_17.5535.5535.2	2	4.105	0.666	1	468.6	55.555557	R.EM*EENFALEAANYQDTIGR.L
VIME_MOUSE	MK_SCX_17.5965.5965.2	2	3.793	0.544	1	515.3	50	R.EMEENFALEAANYQDTIGR.L
VIME_MOUSE	MK_SCX_20_1.8751.8751.2	2	2.245	0.269	1	605.8	61.538464	R.ISLPLPTFSSLNLR.E
VIME_MOUSE	MK_SCX_21.6459.6459.2	2	2.642	0.367	1	1220.4	88.88889	K.ILLAELEQLK.G
VIME_MOUSE	MK_SCX_2201.3419.3419.2	2	2.564	0.352	1	1080.5	94.44444	K.FADLSEAANR.N
VIME_MOUSE	MK_SCX_23.3858.3858.2	2	3.828	0.356	1	1039.8	76.92308	R.SLYSSSPGGAYVTR.S
VIME_MOUSE	MK_SCX_24.14516.14516.3	3	3.094	0.294	1	694.6	29.761904	R.LLQDSVDFSLADAINTEFKNTR.T
VIME_MOUSE	MK_SCX_28.4638.4638.2	2	4.5	0.467	1	1000.2	75	R.ETNLESLPLVDTHSK.R
VIME_MOUSE	MK_SCX_30.4573.4573.3	3	3.178	0.453	1	779.5	46.153847	R.VEVERDNLAEDIMR.L
VIME_MOUSE	MK_SCX_32.4316.4316.2	2	4.587	0.48	1	2078.4	76.92308	R.LQDEIQNMKEEMAR.H
VIME_MOUSE	MK_SCX_32.5396.5396.2	2	4.408	0.412	1	1676.7	76.92308	K.ILLAELEQLKGQGK.S
VIME_MOUSE	MK_SCX_35.9629.9629.2	2	4.285	0.199	1	1110.8	79.16667	R.KVESLQEEIAFLK.K
VIME_MOUSE	MK_SCX_43.3793.3793.2	2	2.993	0.416	1	1473.8	93.75	R.FANYIDKVR.F
VIME_MOUSE	MK_SCX_45.4560.4560.2	2	4.633	0.509	1	487.2	56.666668	R.ETNLESLPLVDTHSKR.T
VIME_MOUSE	MK_SCX_50.5137.5137.3	3	4.852	0.28	1	852.8	50	R.KVESLQEEIAFLKK.L
VIME_MOUSE	MK_SCX_50.5155.5155.2	2	4.649	0.442	1	2376.6	88.46153	R.KVESLQEEIAFLKK.L
VINC_MOUSE	MK_SCX_15.6663.6663.2	2	2.966	0.427	1	553.7	45	R.EAFQPQEPDFPPPPPDLEQLR.L
VINC_MOUSE	MK_SCX_17.9912.9912.3	3	3.044	0.383	1	528.4	33.75	K.AIPDLTAPVAAVQAAVSNLVR.V
VINC_MOUSE	MK_SCX_17.9967.9967.2	2	5.899	0.562	1	1477.3	67.5	K.AIPDLTAPVAAVQAAVSNLVR.V
VINC_MOUSE	MK_SCX_18.5216.5216.2	2	4.839	0.617	1	1120.5	70.588234	K.LVQAAQMLQSDPYSVPAR.D
VINC_MOUSE	MK_SCX_20_1.4394.4394.2	2	3.527	0.533	1	1382.6	76.92308	K.AQQVSQGLDVLTAK.V
VINC_MOUSE	MK_SCX_23.4445.4445.2	2	2.54	0.139	1	744.2	83.33333	K.MSAEINEIIR.V
VINC_MOUSE	MK_SCX_23.6818.6818.3	3	4.13	0.532	1	795	28	K.LVQAAQMLQSDPYSVPARDYLIDGSR.G
VINC_MOUSE	MK_SCX_23.8188.8188.3	3	3.58	0.325	1	358.6	20.192307	R.TNISDEESEQATEM*LVHNAQNLMQSVK.E
VINC_MOUSE	MK_SCX_23.8634.8634.3	3	4.459	0.52	1	760.3	34.615387	R.TNISDEESEQATEMLVHNAQNLMQSVK.E
VINC_MOUSE	MK_SCX_26.6491.6491.3	3	4.211	0.406	1	781.4	31.818182	K.VREAFQPQEPDFPPPPPDLEQLR.L

VINC_MOUSE	MK_SCX_33.4371.4371.3	3	4.811	0.48	1	1173.2	47.22222	K.GWLRDPNASPGDAGEQAIR.Q
VINC_MOUSE	MK_SCX_47.4262.4262.3	3	4.266	0.505	1	1346.8	48.333332	R.VGKETVQTTEDQILKR.D
VINEX_MOUSE	MK_SCX_34.3562.3562.3	3	3.125	0.38	1	789	39.0625	R.SQTQSLNTPGPTLSHPR.A
VINEX_MOUSE	MK_SCX_37.7181.7181.3	3	3.973	0.44	1	1495.3	50	R.RSAFPFPITLQEPR.S
VINEX_MOUSE	MK_SCX_43.5620.5620.3	3	4.321	0.592	1	1263.7	37.5	R.ATSRPINLGPSSPNTEIHWTPYR.A
VINEX_MOUSE	MK_SCX_52.3274.3274.3	3	3.346	0.495	1	594.5	37.5	R.RDFVYPSSAREPSASER.G
VNN1_MOUSE	MK_SCX_19.11701.11701.3	3	6.201	0.575	1	2290.4	40	K.DTLLPVSHSEALALMNQNLDLLEGAIVSAAK.Q
VNN1_MOUSE	MK_SCX_32.5034.5034.3	3	3.55	0.36	1	704.9	37.5	K.QGAHIIVTPEDGIYGVR.F
VPS29_MOUSE	MK_SCX_41.8299.8299.3	3	4.87	0.308	1	1767.7	40.909092	K.IGLIHGHQVIPWGDMASLALLQR.Q
VPS35_MOUSE	MK_SCX_13.3434.3434.2	2	2.596	0.175	1	332.8	63.636364	R.M*QHQGHSRDREK.R
VPS35_MOUSE	MK_SCX_18.4837.4837.2	2	3.673	0.337	1	1126.4	60.714287	R.SDDPDQQYLILNTAR.K
VPS4A_MOUSE	MK_SCX_21.5938.5938.2	2	2.56	0.145	1	621.6	53.846157	K.TEGYSGADISIIVR.D
VPS4A_MOUSE	MK_SCX_51.7821.7821.3	3	3.694	0.34	1	660.4	34.210526	K.EALKEAVILPIKFPHLFTGK.R
VPS4B_MOUSE	MK_SCX_31.4166.4166.2	2	4.604	0.453	1	799.9	73.333336	R.SLSSTKPTVNEQDLLK.L
VPS4B_MOUSE	MK_SCX_50.4609.4609.3	3	3.192	0.446	1	766	48.52941	K.KLQNQLQGAIVIERPNVK.W
VRK3_MOUSE	MK_SCX_15.5131.5131.2	2	5.671	0.53	1	1879.6	76.31579	R.DDQGILYEAEPTSAVPSESR.T
VRK3_MOUSE	MK_SCX_37.3424.3424.3	3	3.221	0.121	1	806.8	44.230766	K.VKCSHTVTSLPLSR.H
VTDB_MOUSE	MK_SCX_23.10451.10451.3	3	5.954	0.56	1	1112.9	35	K.LAQKVPTANLENVLPLAEDFTEILSR.C
VTDB_MOUSE	MK_SCX_38.5605.5605.3	3	3.696	0.28	1	580	39.285713	R.KFSSSTFEQVNQLVK.E
VTI1B_MOUSE	MK_SCX_16.7390.7390.2	2	4.633	0.506	1	1220.1	63.15789	R.IATETDQIGTEIIEELGEQR.D
WASF1_MOUSE	MK_SCX_21.9163.9163.2	2	3.56	0.193	1	1320.5	83.33333	K.FYTNPSYFFDLWK.E
WASF2_MOUSE	MK_SCX_13.3818.3818.2	2	2.44	0.154	1	570.1	70.83333	R.QTLPSDTSELECR.T
WASF2_MOUSE	MK_SCX_26.4893.4893.2	2	4.659	0.617	1	1032.1	66.66667	K.VTQLDPKEEEVSLQGINTR.K
WASL_MOUSE	MK_SCX_15.8607.8607.3	3	3.735	0.318	1	741.9	26.785713	K.SVSDGQESTPPTPAPTSGIVGALMEVMQK.R
WASL_MOUSE	MK_SCX_15.8638.8638.2	2	2.616	0.494	1	312.6	33.92857	K.SVSDGQESTPPTPAPTSGIVGALMEVMQK.R
WBP11_MOUSE	MK_SCX_17.5213.5213.2	2	5.057	0.34	1	1287.4	62.5	K.LDEMEFNPVQQPQLNEK.V
WBP11_MOUSE	MK_SCX_2201.4985.4985.2	2	2.127	0.134	1	413.2	65	R.AQLSQYFDAVK.N
WBP11_MOUSE	MK_SCX_48.4119.4119.3	3	3.148	0.32	1	681.7	40.384613	R.LYEKENPDIYKELR.K
WBP2_MOUSE	MK_SCX_23.3348.3348.2	2	4.059	0.501	1	1510	84.61539	K.AEAGGGWEGSASYK.L
WDR1_MOUSE	MK_SCX_19.4691.4691.2	2	5.025	0.581	1	855.9	76.666664	K.YAPSGFYIASGDISGK.L
WDR1_MOUSE	MK_SCX_23.4347.4347.2	2	3.348	0.37	1	843.4	88.88889	K.VFASLPQVER.G
WDR1_MOUSE	MK_SCX_41.4088.4088.3	3	3.794	0.311	1	678	55	K.KVFASLPQVER.G
WDR1_MOUSE	MK_SCX_49.6234.6234.3	3	4.473	0.559	1	941.2	33.333336	K.AHDGGIYAISWSPDSTHLLSASGDKTSK.I
WIRE_MOUSE	MK_SCX_25.3790.3790.3	3	3.889	0.424	1	645.2	41.17647	K.DASEAPAGKPALQVPSSR.A
WWC1_MOUSE	MK_SCX_12.9945.9945.2	2	2.646	0.231	1	974.2	57.692307	K.SELQADKM*M*RAAAK.D
WWC1_MOUSE	MK_SCX_19.3557.3557.2	2	2.759	0.188	1	769.1	50	R.LILINEKEELLKEMR.F
XIP_MOUSE	MK_SCX_28.5557.5557.3	3	4.571	0.269	1	926.7	36.842106	R.GTLSDEHAGVISVLAQQAAR.L
XIP_MOUSE	MK_SCX_28.5568.5568.2	2	6.684	0.543	1	2394.1	68.42105	R.GTLSDEHAGVISVLAQQAAR.L
XPP1_MOUSE	MK_SCX_18.7153.7153.2	2	3.134	0.504	1	548.4	57.692307	R.VGVDPLIIPTDYWK.K
XPP1_MOUSE	MK_SCX_2201.6940.6940.3	3	4.44	0.519	1	763.2	34.375	R.TLSLDEVYLIDSGAQYKDGTTDVTR.T
XPP1_MOUSE	MK_SCX_26.7103.7103.3	3	3.524	0.296	1	1011.7	32.142857	K.MGLKDTPTQEDWLVSVLPEGSR.V
XYLB_MOUSE	MK_SCX_16.6540.6540.2	2	4.925	0.64	1	945.6	54.166668	K.SAPQPSLAATPNPGASQVYAALLPR.Y
XYLB_MOUSE	MK_SCX_16.6615.6615.3	3	4.494	0.506	1	780	33.333336	K.SAPQPSLAATPNPGASQVYAALLPR.Y
XYLB_MOUSE	MK_SCX_2201.3830.3830.2	2	2.271	0.384	1	690	77.77778	R.ALIEGQFM*AK.R
XYLB_MOUSE	MK_SCX_24.5764.5764.2	2	2.32	0.248	1	618.5	58.333332	R.CCLGWDFSTQQVK.V
XYLB_MOUSE	MK_SCX_52.5531.5531.3	3	3.454	0.478	1	1201.7	40.789474	R.HRFNAENMEVSAFPGDVEIR.A
YAP1_MOUSE	MK_SCX_14.6779.6779.3	3	3.539	0.432	1	347.2	26.785713	R.TPDDFLNSVDEMDTGDTISQSTLPSQQSR.F
YAP1_MOUSE	MK_SCX_14.6780.6780.2	2	4.918	0.532	1	655.5	35.714287	R.TPDDFLNSVDEMDTGDTISQSTLPSQQSR.F
YAP1_MOUSE	MK_SCX_16.4390.4390.2	2	3.232	0.324	1	1822.5	71.875	R.DESTDSGLSM*SSYSIPR.T
YAP1_MOUSE	MK_SCX_16.5066.5066.2	2	5.196	0.618	1	1240.8	48	R.SQLPTLEQDGGTPNAVSSPGM*SQELR.T
YAP1_MOUSE	MK_SCX_16.5128.5128.2	2	5.41	0.624	1	2144.8	75	R.DESTDSGLSMSSYSIPR.T

YAP1_MOUSE	MK_SCX_16.5504.5504.2	2	5.749	0.618	1	1213.3	48	R.SQLPTLEQDGGTPNAVSSPGMSQELR.T
YBOX1_MOUSE	MK_SCX_19.5703.5703.3	3	5.591	0.575	1	1218.8	25.714287	R.SVGDGETVEFDVVEGEKGAEAANVTGPGGVPVQGSK.Y
YBOX1_MOUSE	MK_SCX_20_1.3577.3577.2	2	5.446	0.587	1	1770.3	69.44444	K.GAEAANVTGPGGVPVQGSK.Y
YBOX1_MOUSE	MK_SCX_27.2459.2459.3	3	5.286	0.43	1	1264.5	39.772728	R.EDGNEEDKENQGDETQGQQPPQR.R
YBOX1_MOUSE	MK_SCX_28.3172.3172.3	3	4.82	0.509	1	1388.2	34.82143	R.NYQQNYQNSESGEKNEGSESAPEGQAQQR.R
YBOX1_MOUSE	MK_SCX_39.2403.2403.3	3	3.507	0.423	1	680.8	29.347824	R.EDGNEEDKENQGDETQGQQPPQRR.Y
YMEL1_MOUSE	MK_SCX_18.7498.7498.2	2	5.676	0.434	1	2470	65.789474	R.GTVGFSGAELENLVNQAALK.A
YMEL1_MOUSE	MK_SCX_20_1.5131.5131.2	2	2.332	0.167	1	569	54.166668	R.LVEAQNIAPSFVK.G
YMEL1_MOUSE	MK_SCX_24.6233.6233.3	3	4.266	0.587	1	1571.3	36.458336	K.LGVMTYSDTGKLSPETQSAIEQEIR.I
ZADH1_MOUSE	MK_SCX_19.5192.5192.2	2	4.353	0.43	1	1727.2	65.789474	K.GLENM*GVAFQSMMTGGNVGK.Q
ZADH1_MOUSE	MK_SCX_19.6769.6769.2	2	6.325	0.596	1	2109.9	73.68421	K.GLENMGVAFQSMMTGGNVGK.Q
ZADH1_MOUSE	MK_SCX_21.5774.5774.2	2	2.49	0.27	1	486.1	60.000004	R.TLYLSVDPYMR.C
ZC11A_MOUSE	MK_SCX_18.3386.3386.2	2	5.164	0.493	1	1097.3	73.333336	K.MEANETSDETISDPTK.L
ZC11A_MOUSE	MK_SCX_46.2896.2896.3	3	3.388	0.151	1	565.2	28.57143	K.ENVRTVVRMVTLSSKPEEPLVR.L
ZC11A_MOUSE	MK_SCX_47.4557.4557.3	3	3.975	0.275	1	892.7	40.789474	R.RLSSASTGKPPLSVEDDFEK.L
ZDHC5_MOUSE	MK_SCX_21.5598.5598.2	2	3.018	0.305	1	795.7	63.333332	K.SAQGTGFELGQLQSIR.S
ZDHC5_MOUSE	MK_SCX_51.3036.3036.3	3	4.685	0.444	1	1320.9	38.75	K.YRPGYSSSSTSAAMPHSSSAK.L
ZN185_MOUSE	MK_SCX_13.8064.8064.3	3	4.968	0.539	1	836.1	25.641027	K.EIPGTLQDGQSDPTVASQQLADLSILEPLGSPSGAEQQIK.A
ZN326_MOUSE	MK_SCX_19.6273.6273.2	2	3.603	0.339	1	739.4	53.333336	R.ESVLTATSILNNPIVK.A
ZN592_MOUSE	MK_SCX_15.9696.9696.2	2	4.204	0.521	1	651.6	45.238094	K.TPDFDDLLAAFDIPDPTSLDAK.E
ZN638_MOUSE	MK_SCX_44.4158.4158.3	3	6.342	0.559	1	2014.2	45.238094	R.NKETLSNETVSSNVIDYGHASK.Y
ZNFX1_MOUSE	MK_SCX_25.7769.7769.2	2	2.101	0.215	1	307.3	65	K.FDDIRIYFDAR.I
ZNFX1_MOUSE	MK_SCX_30.3043.3043.2	2	2.067	0.275	1	310.2	50	K.QRLEEIEIVKEK.I
ZO1_MOUSE	MK_SCX_17.4275.4275.2	2	2.827	0.593	1	716.8	60.714287	K.INGTVTENMSLTDAK.T
ZO1_MOUSE	MK_SCX_18.9361.9361.2	2	4.162	0.568	1	746	50	R.LNYAQWYPIVVFLNPDSK.Q
ZO1_MOUSE	MK_SCX_21.4434.4434.2	2	2.989	0.369	1	889	68.181816	R.YEVSSYTDQFSR.N
ZO1_MOUSE	MK_SCX_27.10127.10127.3	3	3.863	0.235	1	689.2	36.11111	R.EAGFLRPVTIFGPIADVAR.E
ZO1_MOUSE	MK_SCX_28.4488.4488.3	3	3.073	0.269	1	495.7	28.125	R.EDLSAQPVQTKFPAYER.V
ZO1_MOUSE	MK_SCX_49.3421.3421.3	3	3.382	0.396	1	493.5	42.857143	R.SNHYDPEEDEEYYRK.Q
ZO1_MOUSE	MK_SCX_51.2478.2478.3	3	3.565	0.363	1	1742.2	55.76923	R.HEEQPAPAYEVHNR.Y
ZO1_MOUSE	MK_SCX_54.5571.5571.3	3	4.622	0.598	1	1055.5	40.476192	R.THFEYEKESPYGLSFNKGEVFR.V
ZO2_MOUSE	MK_SCX_17.3641.3641.2	2	3.004	0.319	1	613.5	53.571426	K.INGTVTENM*SLTDAR.K
ZO2_MOUSE	MK_SCX_17.4364.4364.2	2	3.089	0.419	1	751.6	57.14286	K.INGTVTENMSLTDAR.K
ZO2_MOUSE	MK_SCX_20_1.3877.3877.2	2	2.188	0.173	1	337	40.625	K.SNLPATAGSEIPGGSTK.G
ZO2_MOUSE	MK_SCX_42.7528.7528.3	3	4.681	0.334	1	896.1	31.25	R.EAGFKRPVVLFGPIADIAMER.L
ZYX_MOUSE	MK_SCX_31.4029.4029.3	3	3.977	0.547	1	1145.7	51.38889	K.VNPFRPGDSEPPVAAGAQR.A
ZYX_MOUSE	MK_SCX_31.4063.4063.2	2	5.032	0.576	1	1151.4	69.44444	K.VNPFRPGDSEPPVAAGAQR.A

R.V

[illegible].M

.N
