# Supplementary material for: Comprehensive analysis of the mouse renal cortex using two-dimensional HPLC – tandem mass spectrometry
Source: Proteome Sci. 2008 May 23;6:15. doi: 10.1186/1477-5956-6-15 (PMC2412861; doi:10.1186/1477-5956-6-15)
Supplement: Additional file 3 — List of proteins identified from mouse renal cortex. Annotation of all identified proteins from the list of peptides identified in Additional File 2. [file 1477-5956-6-15-S3.pdf]

Supplemental Table S3. Annotation of identified proteins.

Accession Number	Locus ID	Gene Name	Protein Name	Number of Identified Peptides	Sequence Coverage (%)	Number of Amino Acid	Theoretical MW (Da)	Theoretical pI	Subcellular Location	Protein Family	Number of TMD	NSAF
O08539	BIN1_MOUSE	BIN1	(O08539) Myc box-dependent-interacting protein 1 (Bridging integrator 1) (Amphiphysin-like protein) (Amphiphysin II) (SH3-domain-containing protein 9)	3	10.5	588	64470	5	Nucleus	other	NONE	5.73E-05
O08547	SC22B_MOUSE	SEC22B	(O08547) Vesicle-trafficking protein SEC22b (SEC22 vesicle-trafficking protein-like 1)	5	30.4	214	24609	8.5	Cytoplasm	other	2	0.000262
O08553	DPYL2_MOUSE	DPYSL2	(O08553) Dihydropyrimidinase-related protein 2 (DRP-2) (ULIP 2 protein)	5	19.4	572	62278	6.4	Cytoplasm	enzyme	NONE	7.85E-05
O08579	EMD_MOUSE	EMD	(O08579) Emerin	1	5.8	259	29436	5	Nucleus	other	1	8.67E-05
O08583	THOC4_MOUSE	THOC4	(O08583) THO complex subunit 4 (Tho4) (RNA and export factor-binding protein 1) (REF1-I) (Ally of AML-1 and LEF-1) (Aly/REF)	1	9.8	254	26809	11.2	Nucleus	transcription regulator	NONE	2.21E-05
O08585	CLCA_MOUSE	CLTA	(O08585) Clathrin light chain A (Lca)	2	4.7	235	25557	4.5	Plasma Membrane	other	NONE	0.000287
O08600	NUCG_MOUSE	ENDOG	(O08600) Endonuclease G, mitochondrial precursor (EC 3.1.30.-) (Endo G)	4	23.1	294	32191	9.5	Cytoplasm	enzyme	NONE	0.000134
O08638	MYH11_MOUSE	MYH11	(O08638) Myosin-11 (Myosin heavy chain, smooth muscle isoform) (SMMHC)	8	6.6	1972	227026	5.5	Cytoplasm	other	NONE	3.13E-05
O08648	M3K4_MOUSE	MAP3K4	(O08648) Mitogen-activated protein kinase kinase kinase 4 (EC 2.7.11.25) (MAPK/ERK kinase kinase 4) (MEK kinase 4) (MEKK 4)	2	1.7	1597	179948	6.5	Cytoplasm	kinase	NONE	7.03E-06
O08663	AMPM2_MOUSE	METAP2	(O08663) Methionine aminopeptidase 2 (EC 3.4.11.18) (MetAP 2) (Peptidase M 2) (Initiation factor 2-associated 67 kDa glycoprotein) (p67) (p67eIF2)	2	7.7	478	52922	5.8	Cytoplasm	peptidase	NONE	3.52E-05
O08709	PRDX6_MOUSE	PRDX6	(O08709) Peroxiredoxin-6 (EC 1.11.1.15) (Antioxidant protein 2) (1-Cys peroxiredoxin) (1-Cys PRX) (Acidic calcium-independent phospholipase A2) (EC 3.1.1.-) (aiPLA2) (Non-selenium glutathione peroxidase) (EC 1.11.1.7) (NSGPx)	14	53.4	223	24739	6	Cytoplasm	enzyme	NONE	0.00146
O08749	DLDH_MOUSE	DLD	(O08749) Dihydrolipoyl dehydrogenase, mitochondrial precursor (EC 1.8.1.4) (Dihydrolipoamide dehydrogenase)	22	38.3	509	54212	7.9	Cytoplasm	enzyme	1	0.002183
O08756	HCD2_MOUSE	HSD17B10	(O08756) 3-hydroxyacyl-CoA dehydrogenase type-2 (EC 1.1.1.35) (3-hydroxyacyl-CoA dehydrogenase type II) (Type II HADH) (3-hydroxy-2-methylbutyryl-CoA dehydrogenase) (EC 1.1.1.178) (Endoplasmic reticulum-associated amyloid beta-peptide-binding	5	23.1	260	27287	8.4	Cytoplasm	enzyme	NONE	0.00082
O08759	UBE3A_MOUSE	UBE3A	(O08759) Ubiquitin-protein ligase E3A (EC 6.3.2.-) (Oncogenic protein-associated protein E6-AP)	2	2.7	885	101176	5.1	Nucleus	enzyme	NONE	1.27E-05
O08784	TCOF_MOUSE	TCOF1 (includes EG:6949)	(O08784) Treacle protein (Treacher Collins syndrome protein homolog)	1	1.3	1320	135001	9.3	Nucleus	transporter	NONE	8.5E-06
O08788	DYNA_MOUSE	DCTN1	(O08788) Dynactin-1 (150 kDa dynein-associated polypeptide) (DP-150) (DAP-150) (p150-glued)	2	3	1281	141727	6	Cytoplasm	other	NONE	1.31E-05
O08795	GLU2B_MOUSE	PRKCSH	(O08795) Glucosidase 2 subunit beta precursor (Glucosidase II subunit beta) (Protein kinase C substrate, 60.1 kDa protein, heavy chain) (PKCSH) (80K-H protein)	14	23	521	58793	4.5	Cytoplasm	enzyme	NONE	0.000334
O08912	GALT1_MOUSE	GALNT1	(O08912) Polypeptide N-acetylgalactosaminyltransferase 1 (EC 2.4.1.41) (Protein-UDP acetylgalactosaminyltransferase 1) (UDP-GalNAc:polypeptide N-acetylgalactosaminyltransferase 1) (Polypeptide GalNAc transferase 1) (GalNAc-T1) (pp-GaNTase 1)	1	2.3	559	64255	7.7	Cytoplasm	enzyme	1	1E-05
O08914	FAAH_MOUSE	FAAH	(O08914) Fatty-acid amide hydrolase (EC 3.1.-.-) (Oleamide hydrolase) (Anandamide amidohydrolase)	1	3.8	579	63221	7.9	Plasma Membrane	enzyme	1	9.69E-06
O08915	AIP_MOUSE	AIP	(O08915) AH receptor-interacting protein (AIP) (Aryl-hydrocarbon receptor-interacting protein)	2	10	330	37605	6.4	Nucleus	transcription regulator	NONE	3.4E-05
O08997	ATOX1_MOUSE	ATOX1	(O08997) Copper transport protein ATOX1 (Metal transport protein ATX1)	2	20.6	68	7338	6.5	Cytoplasm	transporter	NONE	0.000413

O09043	NAPSA_MOUSE	NAPSA	(O09043) Napsin-A precursor (EC 3.4.23.-) (Kidney-derived aspartic protease-like protein) (KDAP-1) (KAP)	3	11.2	419	45544	7.5	Extracellular Space	peptidase	NONE	0.000134
O09044	SNP23_MOUSE	SNAP23	(O09044) Synaptosomal-associated protein 23 (SNAP-23) (Vesicle-membrane fusion protein SNAP-23) (Syndet)	11	51.4	210	23261	5	Plasma Membrane	transporter	NONE	0.000802
O09051	GUC2B_MOUSE	GUCA2B	(O09051) Guanylate cyclase activator 2B precursor [Contains: Uroguanylin (UGN)]	1	15.1	106	11628	6.5	Extracellular Space	other	1	5.3E-05
O09061	PSB1_MOUSE	PSMB1	(O09061) Proteasome subunit beta type 1 (EC 3.4.25.1) (Proteasome component C5) (Macropain subunit C5) (Multicatalytic endopeptidase complex subunit C5) (Proteasome gamma chain)	8	35.4	240	26372	7.8	Cytoplasm	peptidase	NONE	0.000491
O09111	NDUBB_MOUSE	NDUFB11	(O09111) NADH dehydrogenase [ubiquinone] 1 beta subcomplex subunit 11, mitochondrial precursor (EC 1.6.5.3) (EC 1.6.99.3) (NADH-ubiquinone oxidoreductase ESSS subunit) (Complex I-ESSS) (CI-ESSS) (Neuronal protein 15.6) (p15.6) (Np15.6)	9	27.2	151	17444	5.2	Cytoplasm	enzyme	2	0.000966
O09131	GSTO1_MOUSE	GSTO1	(O09131) Glutathione transferase omega-1 (EC 2.5.1.18) (GSTO 1-1) (p28)	3	20.4	240	27498	7.4	Cytoplasm	enzyme	NONE	0.000304
O09159	MA2B1_MOUSE	MAN2B1	(O09159) Lysosomal alpha-mannosidase precursor (EC 3.2.1.24) (Mannosidase, alpha B) (Lysosomal acid alpha-mannosidase) (Laman) (Mannosidase alpha class 2B member 1)	2	2.4	1013	114604	8.1	Cytoplasm	enzyme	1	4.43E-05
O09164	SODE_MOUSE	SOD3	(O09164) Extracellular superoxide dismutase [Cu-Zn] precursor (EC 1.15.1.1) (EC-SOD)	2	14.7	251	27392	6.8	Extracellular Space	enzyme	1	8.95E-05
O09167	RL21_MOUSE	RPL21	(O09167) 60S ribosomal protein L21	4	16.4	159	18431	10.5	Cytoplasm	other	NONE	0.000494
O09174	AMACR_MOUSE	AMACR	(O09174) Alpha-methylacyl-CoA racemase (EC 5.1.99.4) (2-methylacyl-CoA racemase)	13	38.4	380	41587	7.4	Cytoplasm	enzyme	NONE	0.001359
O35099	M3K5_MOUSE	MAP3K5 (includes EG:4217)	(O35099) Mitogen-activated protein kinase kinase kinase 5 (EC 2.7.11.25) (MAPK/ERK kinase kinase 5) (MEK kinase 5) (MEKK 5) (Apoptosis signal-regulating kinase 1) (ASK-1)	2	2.1	1380	154459	5.7	Cytoplasm	kinase	NONE	8.13E-06
O35129	PHB2_MOUSE	PHB2	(O35129) Prohibitin-2 (B-cell receptor-associated protein BAP37) (Repressor of estrogen receptor activity)	14	45.8	299	33296	9.8	Cytoplasm	transcription regulator	NONE	0.002027
O35134	RPA1_MOUSE	POLR1A	(O35134) DNA-directed RNA polymerase I largest subunit (EC 2.7.7.6) (RNA polymerase I 194 kDa subunit) (RPA194)	2	1.3	1717	194109	6.9	Nucleus	enzyme	NONE	6.54E-06
O35166	GOSR2_MOUSE	GOSR2	(O35166) Golgi SNAP receptor complex member 2 (27 kDa Golgi SNARE protein) (Membrin)	2	5.2	212	24725	8.2	Cytoplasm	transporter	1	5.3E-05
O35206	COFA1_MOUSE	COL15A1	(O35206) Collagen alpha-1(XV) chain precursor [Contains: Endostatin (Endostatin-XV)]	2	2.6	1367	140525	4.9	Extracellular Space	other	2	8.21E-06
O35215	DOPD_MOUSE	DDT	(O35215) D-dopachrome decarboxylase (EC 4.1.1.84) (D-dopachrome tautomerase)	10	64.1	117	12946	6.5	Cytoplasm	enzyme	NONE	0.002879
O35226	PSD4_MOUSE	PSMD4	(O35226) 26S proteasome non-ATPase regulatory subunit 4 (26S proteasome regulatory subunit S5A) (Rpn10) (Multiubiquitin chain-binding protein)	3	11.4	376	40704	4.8	Cytoplasm	other	NONE	8.96E-05
O35250	EXOC7_MOUSE	EXOC7	(O35250) Exocyst complex component 7 (Exocyst complex component Exo70)	2	4.7	697	79960	7	Plasma Membrane	transporter	NONE	1.61E-05
O35295	PURB_MOUSE	PURB	(O35295) Transcriptional activator protein Pur-beta (Purine-rich element-binding protein B) (Vascular actin single-stranded DNA-binding factor 2 p44 component)	6	39.3	323	33770	5.4	Nucleus	transcription regulator	NONE	0.000278
O35309	NMI_MOUSE	NMI	(O35309) N-myc-interactor (Nmi) (N-myc and STAT interactor)	2	8.3	314	35236	5	Cytoplasm	transcription regulator	NONE	5.36E-05
O35381	AN32A_MOUSE	ANP32A	(O35381) Acidic leucine-rich nuclear phosphoprotein 32 family member A (Potent heat-stable protein phosphatase 2A inhibitor I1PP2A) (Acidic nuclear phosphoprotein pp32) (Leucine-rich acidic nuclear protein)	2	7.7	247	28538	4.1	Nucleus	other	NONE	4.55E-05
O35386	PAHX_MOUSE	PHYH	(O35386) Phytanoyl-CoA dioxygenase, peroxisomal precursor (EC 1.14.11.18) (Phytanoyl-CoA alpha-hydroxylase) (PhyH) (Phytanic acid oxidase) (Lupus nephritis-associated peptide 1)	5	11.8	338	38607	7.5	Cytoplasm	enzyme	NONE	0.000116
O35387	HAX1_MOUSE	HAX1	(O35387) HS1-associating protein X-1 (HAX-1) (HS1-binding protein)	5	33.9	280	31654	4.9	Nucleus	other	NONE	0.000241

O35409	FOLH1_MOUSE	FOLH1	(O35409) Glutamate carboxypeptidase 2 (EC 3.4.17.21) (Glutamate carboxypeptidase II) (Membrane glutamate carboxypeptidase) (mGCP) (N-acetylated-alpha-linked acidic dipeptidase I) (NAALADase I) (Pteroylpoly-gamma-glutamate carboxypeptidase) (F	13	18.5	752	84635	7.5	Plasma Membrane	peptidase	1	0.000299
O35459	ECH1_MOUSE	ECH1	(O35459) Delta3,5-delta2,4-dienoyl-CoA isomerase, mitochondrial precursor (EC 5.3.3.-)	11	34.9	327	36118	7.7	Cytoplasm	enzyme	NONE	0.001562
O35465	FKBP8_MOUSE	FKBP8	(O35465) 38 kDa FK506-binding protein homolog (FKBPR38) (FK506-binding protein 8) (muFKBP38)	3	12.7	355	38615	7	Cytoplasm	other	1	7.91E-05
O35479	HNRPG_MOUSE	RBMXL1	(O35479) Heterogeneous nuclear ribonucleoprotein G (hnRNP G) (RNA-binding motif protein, X chromosome)	2	7.5	388	42234	10	Nucleus	other	NONE	2.89E-05
O35488	S27A2_MOUSE	SLC27A2	(O35488) Very-long-chain acyl-CoA synthetase (EC 6.2.1.-) (VLCS) (Very-long-chain-fatty-acid-CoA ligase) (VLACS) (THCA-CoA ligase) (Fatty-acid-coenzyme A ligase, very long-chain 1) (Long-chain-fatty-acid--CoA ligase) (EC 6.2.1.3) (Fatty acid	10	17.9	620	70367	8.8	Cytoplasm	transporter	1	0.000389
O35593	PSDE_MOUSE	PSMD14 (includes EG:10213)	(O35593) 26S proteasome non-ATPase regulatory subunit 14 (26S proteasome regulatory subunit rpn11) (MAD1)	6	24.5	310	34577	6.5	Cytoplasm	peptidase	NONE	0.000181
O35598	ADA10_MOUSE	ADAM10	(O35598) ADAM 10 precursor (EC 3.4.24.81) (A disintegrin and metalloproteinase domain 10) (Mammalian disintegrin-metalloprotease) (Kuzbanian protein homolog)	5	7.9	749	83967	8	Plasma Membrane	peptidase	2	6E-05
O35639	ANXA3_MOUSE	ANXA3	(O35639) Annexin A3 (Annexin III) (Lipocortin III) (Placental anticoagulant protein III) (PAP-III) (35-alpha calcimedin)	4	16.1	322	36240	5.5	Cytoplasm	enzyme	NONE	8.72E-05
O35643	AP1B1_MOUSE	AP1B1	(O35643) AP-1 complex subunit beta-1 (Adapter-related protein complex 1 beta-1 subunit) (Beta-adaptin 1) (Adaptor protein complex AP-1 beta-1 subunit) (Golgi adaptor HA1/AP1 adaptin beta subunit) (Clathrin assembly protein complex 1 beta larg	3	7.1	943	103979	5.1	Cytoplasm	transporter	NONE	8.33E-05
O35683	NDUA1_MOUSE	NDUFA1	(O35683) NADH dehydrogenase [ubiquinone] 1 alpha subcomplex subunit 1 (EC 1.6.5.3) (EC 1.6.99.3) (NADH-ubiquinone oxidoreductase MWFE subunit) (Complex I-MWFE) (CI-MWFE)	2	14.3	70	8139	9.5	Cytoplasm	enzyme	1	0.000802
O35685	NUDC_MOUSE	NUDC	(O35685) Nuclear migration protein nudC (Nuclear distribution protein C homolog) (Silica-induced gene 92 protein) (SIG-92)	2	3.9	332	38358	5.3	Cytoplasm	other	NONE	5.07E-05
O35691	PININ_MOUSE	PNN (includes EG:5411)	(O35691) Pinin	3	4.8	724	82305	7	Plasma Membrane	other	NONE	2.33E-05
O35737	HNRH1_MOUSE	HNRPH1	(O35737) Heterogeneous nuclear ribonucleoprotein H (hnRNP H)	5	19.9	448	49068	6.3	Nucleus	other	NONE	0.000276
O35841	API5_MOUSE	API5	(O35841) Apoptosis inhibitor 5 (API-5) (AAC-11)	2	6	504	56771	5.8	Cytoplasm	other	NONE	0.0001
O35857	TIM44_MOUSE	TIMM44	(O35857) Import inner membrane translocase subunit TIM44, mitochondrial precursor	4	11.3	452	51176	8.3	Cytoplasm	transporter	NONE	0.000286
O35887	CALU_MOUSE	CALU (includes EG:813)	(O35887) Calumenin precursor (Crocalbin)	4	23.8	315	37064	4.7	Cytoplasm	other	NONE	0.000178
O35904	PK3CD_MOUSE	PIK3CD	(O35904) Phosphatidylinositol-4,5-bisphosphate 3-kinase catalytic subunit delta isoform (EC 2.7.1.153) (PI3-kinase p110 subunit delta) (PtdIns-3-kinase p110) (PI3K) (p110delta)	2	3.6	1043	119647	7.3	Cytoplasm	kinase	NONE	3.23E-05
O35954	PITM1_MOUSE	PITPNM1	(O35954) Membrane-associated phosphatidylinositol transfer protein 1 (Phosphatidylinositol transfer protein, membrane-associated 1) (PITPnm 1) (Mpt-1) (Pyk2 N-terminal domain-interacting receptor 2) (NIR-2) (Drosophila retinal degeneration B	2	3.7	1243	134940	6.1	Cytoplasm	transporter	NONE	1.35E-05
O35972	RM23_MOUSE	MRPL23	(O35972) Mitochondrial 39S ribosomal protein L23 (L23mt) (MRP-L23) (L23 mitochondrial-related protein)	2	24	146	17122	9.7	Cytoplasm	other	NONE	0.000154
O54724	PTRF_MOUSE	PTRF	(O54724) Polymerase I and transcript release factor	6	19.6	392	43954	5.5	Nucleus	transcription regulator	NONE	0.000358

O54774	AP3D1_MOUSE	AP3D1	(O54774) AP-3 complex subunit delta-1 (Adapter-related protein complex 3 subunit delta-1) (Delta-adaptin 3) (AP-3 complex subunit delta) (Delta-adaptin) (mBLVR1)	3	5.3	1199	135081	7.4	Cytoplasm	transporter	1	3.75E-05
O54782	MA2B2_MOUSE	MAN2B2 (includes EG:23324)	(O54782) Epididymis-specific alpha-mannosidase precursor (EC 3.2.1.24) (Mannosidase alpha class 2B member 2)	1	1.4	1018	115609	7.4	Cytoplasm	enzyme	NONE	5.51E-06
O54833	CSK22_MOUSE	CSNK2A2	(O54833) Casein kinase II subunit alpha' (EC 2.7.11.1) (CK II)	2	4.6	350	41215	8.5	Cytoplasm	kinase	NONE	6.42E-05
O54879	HMGB3_MOUSE	HMGB3	(O54879) High mobility group protein B3 (High mobility group protein 4) (HMG-4) (High mobility group protein 2a) (HMG-2a)	2	10.1	199	22879	8.4	Nucleus	other	NONE	5.64E-05
O54931	AKAP2_MOUSE	AKAP2	(O54931) A-kinase anchor protein 2 (Protein kinase A-anchoring protein 2) (PRKA2) (AKAP-2) (AKAP expressed in kidney and lung) (AKAP-KL)	10	16	885	97501	5.4	Cytoplasm	other	NONE	0.000146
O54941	SMCE1_MOUSE	SMARCE1 (includes EG:6605)	(O54941) SWI/SNF-related matrix-associated actin-dependent regulator chromatin subfamily E member 1 (BRG1-associated factor 57)	1	3.2	411	46638	4.9	Nucleus	transcription regulator	NONE	1.37E-05
O54962	BAF_MOUSE	BANF1	(O54962) Barrier-to-autointegration factor (Breakpoint cluster region protein 1) (LAP2-binding protein 1)	4	29.2	89	10103	6.1	Nucleus	other	NONE	0.000694
O54983	CRYM_MOUSE	CRYM	(O54983) Mu-crystallin homolog	8	24.6	313	33523	5.7	Cytoplasm	enzyme	NONE	0.000484
O54988	SLK_MOUSE	SLK	(O54988) STE20-like serine/threonine-protein kinase (EC 2.7.11.1) (STE20-like kinase) (STE20-related serine/threonine-protein kinase) (STE20-related kinase) (mSLK) (Serine/threonine-protein kinase 2) (STE20-related kinase SMAK) (Etk4)	4	7.4	1233	141457	5.1	Nucleus	kinase	NONE	2.73E-05
O55022	PGRC1_MOUSE	PGRMC1	(O55022) Membrane-associated progesterone receptor component 1	5	25.3	194	21563	4.7	Plasma Membrane	other	1	0.000955
O55023	IMPA1_MOUSE	IMPA1	(O55023) Inositol monophosphatase (EC 3.1.3.25) (IMPase) (IMP) (Inositol-1(or 4)-monophosphatase) (Lithium-sensitive myo-inositol monophosphatase A1)	3	9.7	277	30436	5.2	Cytoplasm	phosphatase	NONE	0.000162
O55028	BCKD_MOUSE	BCKDK	(O55028) [3-methyl-2-oxobutanoate dehydrogenase [lipoamide]] kinase, mitochondrial precursor (EC 2.7.11.4) (Branched-chain alpha-ketoacid dehydrogenase kinase) (BCKDHKIN) (BCKD-kinase)	4	9.2	412	46588	8.9	Cytoplasm	kinase	NONE	9.54E-05
O55060	TPMT_MOUSE	TPMT	(O55060) Thiopurine S-methyltransferase (EC 2.1.1.67) (Thiopurine methyltransferase)	7	32.1	240	27586	6.4	Cytoplasm	enzyme	NONE	0.000351
O55098	STK10_MOUSE	STK10	(O55098) Serine/threonine-protein kinase 10 (EC 2.7.11.1) (Lymphocyte-oriented kinase)	1	2.3	966	111993	7	Cytoplasm	kinase	NONE	5.81E-06
O55106	STRN_MOUSE	STRN	(O55106) Striatin	2	5.5	780	86014	5.3	Cytoplasm	other	NONE	2.16E-05
O55111	DSG2_MOUSE	DSG2	(O55111) Desmoglein-2 precursor	1	1.3	1122	122397	5.3	Plasma Membrane	other	2	1.5E-05
O55125	NIPS1_MOUSE	NIPSNAP1	(O55125) Protein NipSnap1	6	12	284	33363	9.4	Unknown	enzyme	NONE	0.000435
O55126	NIPS2_MOUSE	GBAS	(O55126) Protein NipSnap2 (Glioblastoma amplified sequence)	3	12.5	281	32933	9.3	Plasma Membrane	other	NONE	9.99E-05
O55131	SEPT7_MOUSE	SPET7	(O55131) Septin-7 (CDC10 protein homolog)	2	7.1	436	50550	8.6	Cytoplasm	other	NONE	3.86E-05
O55135	IF6_MOUSE	ITGB4BP	(O55135) Eukaryotic translation initiation factor 6 (eIF-6) (B4 integrin interactor) (CAB) (p27(BBP))	2	17.1	245	26511	4.7	Cytoplasm	translation regulator	NONE	9.16E-05
O55137	ACOT1_MOUSE	ACOT1 (includes EG:26897)	(O55137) Acyl-coenzyme A thioesterase 1 (EC 3.1.2.2) (Acyl-CoA thioesterase 1) (Inducible cytosolic acyl-coenzyme A thioester hydrolase) (Long chain acyl-CoA thioester hydrolase) (Long chain acyl-CoA hydrolase) (CTE-I)	2	11	419	46136	6.6	Cytoplasm	enzyme	NONE	4.02E-05
O55143	AT2A2_MOUSE	ATP2A2	(O55143) Sarcoplasmic/endoplasmic reticulum calcium ATPase 2 (EC 3.6.3.8) (Calcium pump 2) (SERCA2) (SR Ca(2+)-ATPase 2) (Calcium-transporting ATPase sarcoplasmic reticulum type, slow twitch skeletal muscle isoform) (Endoplasmic reticulum cla	1	2	1044	114858	5.3	Cytoplasm	transporter	10	1.08E-05
O55222	ILK_MOUSE	ILK	(O55222) Integrin-linked protein kinase (EC 2.7.11.1)	2	7.3	452	51347	8.1	Plasma Membrane	kinase	NONE	2.48E-05
O55234	PSB5_MOUSE	PSMB5	(O55234) Proteasome subunit beta type 5 precursor (EC 3.4.25.1) (Proteasome epsilon chain) (Macropain epsilon chain) (Multicatalytic endopeptidase complex epsilon chain) (Proteasome subunit X) (Proteasome chain 6)	2	11.5	209	22967	8.5	Cytoplasm	peptidase	NONE	8.06E-05

O55236	MCE1_MOUSE	RNGTT	(O55236) mRNA capping enzyme (HCE) (MCE1) [Includes: Polynucleotide 5'-triphosphatase (EC 3.1.3.33) (mRNA 5'-triphosphatase) (TPase); mRNA guanylyltransferase (EC 2.7.7.50) (GTP--RNA guanylyltransferase) (GTase)]	1	2.3	597	68684	8.2	Nucleus	phosphatase	NONE	9.4E-06
O70194	IF37_MOUSE	EIF3S7	(O70194) Eukaryotic translation initiation factor 3 subunit 7 (eIF-3 zeta) (eIF3 p66) (eIF3d)	9	17.4	547	63558	6	Cytoplasm	translation regulator	NONE	0.000215
O70250	PGAM2_MOUSE	PGAM2	(O70250) Phosphoglycerate mutase 2 (EC 5.4.2.1) (EC 5.4.2.4) (EC 3.1.3.13) (Phosphoglycerate mutase isozyme M) (PGAM-M) (BPG-dependent PGAM 2) (Muscle-specific phosphoglycerate mutase)	3	8.7	252	28696	8.5	Cytoplasm	phosphatase	NONE	0.000134
O70251	EF1B_MOUSE	EEF1B2	(O70251) Elongation factor 1-beta (EF-1-beta)	7	31.2	224	24562	4.7	Cytoplasm	translation regulator	NONE	0.000827
O70252	HMOX2_MOUSE	HMOX2	(O70252) Heme oxygenase 2 (EC 1.14.99.3) (HO-2)	1	6	315	35739	5.9	Cytoplasm	enzyme	1	8.91E-05
O70305	ATX2_MOUSE	ATXN2	(O70305) Ataxin-2 (Spinocerebellar ataxia type 2 protein homolog)	4	5.1	1285	136485	9.6	Nucleus	other	NONE	4.37E-05
O70309	ITB5_MOUSE	ITGB5	(O70309) Integrin beta-5 precursor	2	4	798	87909	6.2	Plasma Membrane	other	2	1.41E-05
O70310	NMT1_MOUSE	NMT1	(O70310) Glycylpeptide N-tetradecanoyltransferase 1 (EC 2.3.1.97) (Peptide N-myristoyltransferase 1) (Myristoyl-CoA:protein N-myristoyltransferase 1) (NMT 1) (Type I N-myristoyltransferase)	2	6.7	496	56888	8	Cytoplasm	enzyme	NONE	3.4E-05
O70318	E41L2_MOUSE	EPB41L2	(O70318) Band 4.1-like protein 2 (Generally expressed protein 4.1) (4.1G)	8	12.3	988	109833	5.5	Plasma Membrane	other	NONE	7.39E-05
O70325	GPX41_MOUSE	GPX4	(O70325) Phospholipid hydroperoxide glutathione peroxidase, mitochondrial precursor (EC 1.11.1.12) (PHGPx) (GPX-4)	4	15.7	197	22182	8.4	Cytoplasm	enzyme	NONE	0.000199
O70400	PDLI1_MOUSE	PDLIM1	(O70400) PDZ and LIM domain protein 1 (Elfin) (LIM domain protein CLP-36) (C-terminal LIM domain protein 1)	1	4.6	326	35643	6.8	Cytoplasm	transcription regulator	NONE	5.17E-05
O70404	VAMP8_MOUSE	VAMP8	(O70404) Vesicle-associated membrane protein 8 (VAMP-8) (Endobrevin) (Edb)	1	18.8	101	11451	8.2	Plasma Membrane	other	1	0.000111
O70423	AOC3_MOUSE	AOC3	(O70423) Membrane copper amine oxidase (EC 1.4.3.6) (Semicarbazide-sensitive amine oxidase) (SSAO) (Vascular adhesion protein 1) (VAP-1)	1	3.4	764	84402	6.4	Plasma Membrane	enzyme	1	2.94E-05
O70433	FHL2_MOUSE	FHL2	(O70433) Four and a half LIM domains protein 2 (FHL-2) (Skeletal muscle LIM-protein 3) (SLIM 3)	2	11.8	279	32073	7.3	Nucleus	other	NONE	6.04E-05
O70435	PSA3_MOUSE	PSMA3	(O70435) Proteasome subunit alpha type 3 (EC 3.4.25.1) (Proteasome component C8) (Macropain subunit C8) (Multicatalytic endopeptidase complex subunit C8) (Proteasome subunit K)	4	20.1	254	28274	5.4	Cytoplasm	peptidase	NONE	0.000155
O70439	STX7_MOUSE	STX7	(O70439) Syntaxin-7	9	38.1	260	29690	5.8	Plasma Membrane	transporter	1	0.000453
O70475	UGDH_MOUSE	UGDH	(O70475) UDP-glucose 6-dehydrogenase (EC 1.1.1.22) (UDP-Glc dehydrogenase) (UDP-GlcDH) (UDPGDH)	4	14.2	493	54832	7.6	Nucleus	enzyme	NONE	5.69E-05
O70481	UBR1_MOUSE	UBR1	(O70481) Ubiquitin-protein ligase E3 component N-recognin-1 (EC 6.-.-.-) (Ubiquitin-protein ligase E3-alpha-1) (Ubiquitin-protein ligase E3-alpha-I)	1	1	1757	200215	6	Cytoplasm	enzyme	1	3.19E-06
O70492	SNX3_MOUSE	SNX3	(O70492) Sorting nexin-3 (SDP3 protein)	3	26.1	161	18626	8.7	Cytoplasm	transporter	NONE	0.000314
O70493	SNX12_MOUSE	SNX12	(O70493) Sorting nexin-12 (SDP8 protein)	2	26.1	165	19116	7.3	Unknown	transporter	NONE	6.8E-05
O70591	PFD2_MOUSE	PFDN2	(O70591) Prefoldin subunit 2	3	28.6	154	16534	6.6	Cytoplasm	other	NONE	0.000328
O88291	ZN326_MOUSE	ZNF326	(O88291) Zinc finger protein 326 (Zinc finger protein-associated with nuclear matrix of 75 kDa)	1	2.8	580	65225	5.2	Nucleus	transcription regulator	NONE	3.87E-05
O88322	NID2_MOUSE	NID2	(O88322) Nidogen-2 precursor (NID-2) (Entactin-2)	11	14	1403	154249	5.4	Extracellular Space	other	1	6.8E-05
O88338	CAD16_MOUSE	CDH16	(O88338) Cadherin-16 precursor (Kidney-specific cadherin) (Ksp-cadherin)	18	32	830	89860	4.7	Plasma Membrane	enzyme	2	0.000987
O88342	WDR1_MOUSE	WDR1 (includes EG:9948)	(O88342) WD repeat protein 1 (Actin-interacting protein 1) (AIP1)	4	9.1	605	66276	6.6	Unknown	other	NONE	8.35E-05
O88343	S4A4_MOUSE	SLC4A4	(O88343) Electrogenic sodium bicarbonate cotransporter 1 (Sodium bicarbonate cotransporter) (Na(+)/HCO3(-) cotransporter) (Solute carrier family 4 member 4)	10	12.9	1079	121483	6.8	Plasma Membrane	transporter	9	0.000265
O88384	VTI1B_MOUSE	VTI1B	(O88384) Vesicle transport through interaction with t-SNAREs homolog 1B (Vesicle transport v-SNARE protein Vti1-like 1) (Vti1-rp1)	1	8.6	232	26713	8.8	Plasma Membrane	transporter	1	4.84E-05

O88413	TULP3_MOUSE	TULP3	(O88413) Tubby-related protein 3 (Tubby-like protein 3)	2	7.6	460	51231	6.2	Unknown	other	NONE	7.32E-05
O88428	PAPS2_MOUSE	PAPSS2	(O88428) Bifunctional 3'-phosphoadenosine 5'-phosphosulfate synthetase 2 (PAPS synthetase 2) (PAPSS 2) (Sulfurylase kinase 2) (SK2) (SK 2) [Includes: Sulfate adenylyltransferase (EC 2.7.7.4) (Sulfate adenylate transferase) (SAT) (ATP-sulfuryl	5	12.6	621	70291	7.6	Cytoplasm	enzyme	NONE	8.13E-05
O88441	MTX2_MOUSE	MTX2	(O88441) Metaxin-2	2	14.8	263	29758	5.6	Cytoplasm	transporter	NONE	6.4E-05
O88456	CPNS1_MOUSE	CAPNS1	(O88456) Calpain small subunit 1 (CSS1) (Calcium-dependent protease small subunit 1) (Calcium-dependent protease small subunit) (CDPS) (Calpain regulatory subunit) (Calcium-activated neutral proteinase small subunit) (CANP small subunit)	4	23	269	28463	5.6	Cytoplasm	peptidase	NONE	0.000167
O88487	DC1I2_MOUSE	DYNC1I2	(O88487) Cytoplasmic dynein 1 intermediate chain 2 (Dynein intermediate chain 2, cytosolic) (DH IC-2) (Cytoplasmic dynein intermediate chain 2)	10	15.5	612	68394	5.3	Cytoplasm	other	NONE	0.000275
O88531	PPT1_MOUSE	PPT1	(O88531) Palmitoyl-protein thioesterase 1 precursor (EC 3.1.2.22) (Palmitoyl-protein hydrolase 1)	4	12.1	306	34490	8	Cytoplasm	enzyme	1	0.000128
O88544	CSN4_MOUSE	COPS4	(O88544) COP9 signalosome complex subunit 4 (Signalosome subunit 4) (SGN4) (JAB1-containing signalosome subunit 4)	3	11.3	406	46285	5.8	Unknown	other	NONE	5.53E-05
O88566	AXN2_MOUSE	AXIN2	(O88566) Axin-2 (Axis inhibition protein 2) (Conductin) (Axin-like protein) (Axil)	2	3.6	840	92935	7.9	Cytoplasm	other	NONE	1.34E-05
O88569	ROA2_MOUSE	HNRPA2B1	(O88569) Heterogeneous nuclear ribonucleoproteins A2/B1 (hnRNP A2 / hnRNP B1)	23	57.2	341	35993	8.6	Nucleus	other	NONE	0.003309
O88587	COMT_MOUSE	COMT	(O88587) Catechol O-methyltransferase (EC 2.1.1.6)	1	9.8	265	29496	5.8	Cytoplasm	enzyme	1	4.24E-05
O88622	PARG_MOUSE	PARG	(O88622) Poly(ADP-ribose) glycohydrolase (EC 3.2.1.143)	1	1.5	969	109324	6.9	Cytoplasm	enzyme	NONE	5.79E-06
O88630	GOSR1_MOUSE	GOSR1	(O88630) Golgi SNAP receptor complex member 1 (28 kDa Golgi SNARE protein) (28 kDa cis-Golgi SNARE p28) (GOS-28)	2	15.6	250	28429	9.3	Cytoplasm	transporter	1	6.74E-05
O88643	PAK1_MOUSE	PAK1	(O88643) Serine/threonine-protein kinase PAK 1 (EC 2.7.11.1) (p21-activated kinase 1) (PAK-1) (P65-PAK) (Alpha-PAK) (CDC42/RAC effector kinase PAK-A)	3	10.6	545	60737	5.7	Cytoplasm	kinase	NONE	6.18E-05
O88685	PRS6A_MOUSE	PSMC3	(O88685) 26S protease regulatory subunit 6A (TAT-binding protein 1) (TBP-1)	11	29.4	442	49493	5.2	Nucleus	transcription regulator	NONE	0.000267
O88696	CLPP_MOUSE	CLPP	(O88696) Putative ATP-dependent Clp protease proteolytic subunit, mitochondrial precursor (EC 3.4.21.92) (Endopeptidase Clp)	6	22.8	272	29800	7.5	Cytoplasm	peptidase	NONE	0.000206
O88746	TOM1_MOUSE	TOM1	(O88746) Target of Myb protein 1	4	15.9	492	54325	4.9	Cytoplasm	transporter	NONE	6.85E-05
O88843	CRADD_MOUSE	CRADD	(O88843) Death domain-containing protein CRADD (Caspase and RIP adapter with death domain) (RIP-associated protein with a death domain)	2	15.1	199	22656	6.2	Cytoplasm	other	NONE	0.000113
O88844	IDHC_MOUSE	IDH1	(O88844) Isocitrate dehydrogenase [NADP] cytoplasmic (EC 1.1.1.42) (Cytosolic NADP-isocitrate dehydrogenase) (Oxalosuccinate decarboxylase) (IDH) (NADP(+)-specific ICDH) (IDP)	53	60.6	414	46660	6.9	Cytoplasm	enzyme	NONE	0.007525
O88845	AKA10_MOUSE	AKAP10 (includes EG:11216)	(O88845) A kinase anchor protein 10, mitochondrial precursor (Protein kinase A-anchoring protein 10) (PRKA10) (Dual specificity A kinase-anchoring protein 2) (D-AKAP-2)	1	3.3	662	73632	6.8	Cytoplasm	other	NONE	8.48E-06
O88848	ARL6_MOUSE	ARL6	(O88848) ADP-ribosylation factor-like protein 6	2	16.7	186	20959	8.3	Cytoplasm	transporter	NONE	0.000151
O88851	RBBP9_MOUSE	RBBP9	(O88851) Retinoblastoma-binding protein 9 (RBBP-9) (B5T overexpressed gene protein) (Bog protein)	2	8.6	186	20912	6	Nucleus	other	NONE	0.000121
O88888	APBA3_MOUSE	APBA3	(O88888) Amyloid beta A4 precursor protein-binding family A member 3 (Neuron-specific X11L2 protein) (Neuronal Munc18-1-interacting protein 3) (Mint-3) (Adapter protein X11gamma)	1	3.5	571	60718	5.1	Cytoplasm	transporter	NONE	2.95E-05
O88952	LIN7C_MOUSE	LIN7C	(O88952) LIN-7 homolog C (LIN-7C) (mLin7C) (Mammalian LIN-seven protein 3) (MALS-3) (Vertebrate LIN 7 homolog 3) (Veli-3 protein)	4	18.8	197	21834	8.4	Cytoplasm	other	NONE	0.000171
O88958	GNPI_MOUSE	GNPDA1	(O88958) Glucosamine-6-phosphate isomerase (EC 3.5.99.6) (Glucosamine-6-phosphate deaminase) (GNPDA) (GlcN6P deaminase) (Oscillin)	4	19.4	289	32550	6.5	Cytoplasm	enzyme	NONE	0.000117

O88967	YMEL1_MOUSE	YME1L1	(O88967) ATP-dependent metalloprotease YME1L1 (EC 3.4.24.-) (YME1-like protein 1) (ATP-dependent metalloprotease FtsH1)	3	8.1	715	80028	9	Cytoplasm	peptidase	1	3.93E-05
O88968	TCO2_MOUSE	TCN2	(O88968) Transcobalamin-2 precursor (Transcobalamin II) (TCII) (TC II)	1	5.6	430	47586	6.3	Extracellular Space	transporter	1	6.53E-05
O88983	STX8_MOUSE	STX8	(O88983) Syntaxin-8 (Syntaxin-like protein 3I35)	1	7.6	236	26925	5	Plasma Membrane	other	1	4.76E-05
O88986	KBL_MOUSE	GCAT	(O88986) 2-amino-3-ketobutyrate coenzyme A ligase, mitochondrial precursor (EC 2.3.1.29) (AKB ligase) (Glycine acetyltransferase)	1	5	416	44931	7.3	Cytoplasm	enzyme	NONE	2.7E-05
O89017	LGMN_MOUSE	LGMN	(O89017) Legumain precursor (EC 3.4.22.34) (Asparaginyl endopeptidase) (Protease, cysteine 1)	4	7.6	435	49373	6.4	Cytoplasm	peptidase	1	0.000155
O89023	TPP1_MOUSE	TPP1	(O89023) Tripeptidyl-peptidase 1 precursor (EC 3.4.14.9) (Tripeptidyl-peptidase I) (TPP-I) (Tripeptidyl aminopeptidase) (Lysosomal pepstatin insensitive protease) (LPIC)	2	6.6	562	61342	6.6	Cytoplasm	peptidase	NONE	5.99E-05
O89079	COPE_MOUSE	COPE	(O89079) Coatomer subunit epsilon (Epsilon-coat protein) (Epsilon-COP)	4	17.9	307	34436	5.1	Cytoplasm	transporter	NONE	0.000128
O89086	RBM3_MOUSE	RBM3	(O89086) Putative RNA-binding protein 3 (RNA-binding motif protein 3)	4	22.2	153	16605	7.5	Nucleus	other	NONE	0.000587
O89090	SP1_MOUSE	SP1	(O89090) Transcription factor Sp1	1	3.6	784	80732	7.3	Nucleus	transcription regulator	NONE	7.16E-06
O89103	C1QR1_MOUSE	CD93	(O89103) Complement component C1q receptor precursor (Complement component 1 q subcomponent receptor 1) (C1qRp) (C1qR(p)) (C1q/MBL/SPA receptor) (CD93 antigen) (Cell surface antigen AA4) (Lymphocyte antigen 68)	2	3.6	644	69355	5.1	Plasma Membrane	other	2	4.36E-05
O89106	FHIT_MOUSE	FHIT	(O89106) Bis(5'-adenosyl)-triphosphatase (EC 3.6.1.29) (Diadenosine 5',5'''-P1,P3-triphosphate hydrolase) (Dinucleosidetriphosphatase) (AP3A hydrolase) (AP3AASE) (Fragile histidine triad protein)	3	30.2	149	17103	6.7	Cytoplasm	enzyme	NONE	0.000188
P00329	ADH1_MOUSE	ADH1C (includes EG:126)	(P00329) Alcohol dehydrogenase 1 (EC 1.1.1.1) (Alcohol dehydrogenase A subunit) (ADH-A2)	9	21.7	374	39640	8.1	Cytoplasm	enzyme	NONE	0.000375
P00375	DYR_MOUSE	DHFR	(P00375) Dihydrofolate reductase (EC 1.5.1.3)	6	29	186	21475	8.6	Unknown	enzyme	NONE	0.000332
P00405	COX2_MOUSE	COX2	(P00405) Cytochrome c oxidase subunit 2 (EC 1.9.3.1) (Cytochrome c oxidase polypeptide II)	7	16.3	227	25976	4.7	Cytoplasm	enzyme	2	0.002596
P00920	CAH2_MOUSE	CA2	(P00920) Carbonic anhydrase 2 (EC 4.2.1.1) (Carbonic anhydrase II) (Carbonate dehydratase II) (CA-II)	8	27	259	28960	7	Cytoplasm	enzyme	NONE	0.003208
P01027	CO3_MOUSE	C3	(P01027) Complement C3 precursor (HSE-MSF) [Contains: Complement C3 beta chain; Complement C3 alpha chain; C3a anaphylatoxin; Complement C3b alpha' chain; Complement C3c fragment; Complement C3dg fragment; Complement C3g fragment; Complement C3	3	3.1	1663	186482	6.8	Extracellular Space	peptidase	3	2.7E-05
P01029	CO4B_MOUSE	C4B	(P01029) Complement C4-B precursor [Contains: Complement C4 beta chain; Complement C4 alpha chain; C4a anaphylatoxin; Complement C4 gamma chain]	1	1.3	1738	192870	7.6	Extracellular Space	other	NONE	6.46E-06
P01132	EGF_MOUSE	EGF	(P01132) Pro-epidermal growth factor precursor (EGF) [Contains: Epidermal growth factor]	2	1.3	1217	133144	6.5	Extracellular Space	growth factor	2	9.22E-06
P01897	HA1L_MOUSE	H2-LD	(P01897) H-2 class I histocompatibility antigen, L-D alpha chain precursor	2	4.1	362	40711	6.2	Plasma Membrane	other	2	9.3E-05
P01898	HA10_MOUSE	H2-Q10	(P01898) H-2 class I histocompatibility antigen, Q10 alpha chain precursor	1	3.4	322	36966	5.3	Plasma Membrane	transmembrane receptor	NONE	5.23E-05
P01900	HA12_MOUSE	HLA-C	(P01900) H-2 class I histocompatibility antigen, D-D alpha chain precursor (H-2D(D))	3	7.4	365	41110	6.7	Plasma Membrane	transmembrane receptor	2	0.000108
P01902	HA1D_MOUSE	HLA-A	(P01902) H-2 class I histocompatibility antigen, K-D alpha chain precursor (H-2K(D))	4	17.4	368	41490	6.6	Plasma Membrane	transmembrane receptor	2	9.15E-05
P01921	HB2D_MOUSE	HLA-DQB2	(P01921) H-2 class II histocompatibility antigen, A-D beta chain precursor	1	4.9	265	29954	7.8	Plasma Membrane	transmembrane receptor	2	2.12E-05
P01942	HBA_MOUSE	HBA2	(P01942) Hemoglobin subunit alpha (Hemoglobin alpha chain) (Alpha-globin)	6	34.8	141	14954	8.2	Cytoplasm	transporter	NONE	0.004737

P02088	HBB1_MOUSE	HBD	(P02088) Hemoglobin beta-1 subunit (Hemoglobin beta-1 chain) (Beta-1-globin) (Hemoglobin beta-major chain)	5	31.5	146	15709	7.6	Cytoplasm	transporter	NONE	0.007459
P02468	LAMC1_MOUSE	LAMC1	(P02468) Laminin gamma-1 chain precursor (Laminin B2 chain)	8	8.2	1607	177298	5.2	Extracellular Space	other	NONE	5.24E-05
P02469	LAMB1_MOUSE	LAMB1	(P02469) Laminin beta-1 chain precursor (Laminin B1 chain)	6	5.2	1786	196904	4.9	Extracellular Space	other	1	3.77E-05
P04117	FABPA_MOUSE	FABP4	(P04117) Fatty acid-binding protein, adipocyte (AFABP) (Adipocyte lipid-binding protein) (ALBP) (A-FABP) (P2 adipocyte protein) (Myelin P2 protein homolog) (3T3-L1 lipid-binding protein) (422 protein) (P15)	9	64.1	131	14519	8.4	Cytoplasm	transporter	NONE	0.001842
P04186	CFAB_MOUSE	CFB	(P04186) Complement factor B precursor (EC 3.4.21.47) (C3/C5 convertase) [Contains: Complement factor B Ba fragment; Complement factor B Bb fragment]	2	3	761	85005	7.4	Extracellular Space	peptidase	NONE	2.21E-05
P04202	TGFB1_MOUSE	TGFB1	(P04202) Transforming growth factor beta-1 precursor (TGF-beta-1) [Contains: Latency-associated peptide (LAP)]	1	4.9	390	44310	8.6	Extracellular Space	growth factor	1	1.44E-05
P04228	HA2D_MOUSE	HLA-DQA1	(P04228) H-2 class II histocompatibility antigen, A-D alpha chain precursor	1	9	256	28243	5	Plasma Membrane	transmembrane receptor	2	6.58E-05
P04370	MBP_MOUSE	MBP	(P04370) Myelin basic protein (MBP) (Myelin A1 protein)	2	13.2	250	27168	9.6	Extracellular Space	other	NONE	4.49E-05
P05064	ALDOA_MOUSE	ALDOA	(P05064) Fructose-bisphosphate aldolase A (EC 4.1.2.13) (Muscle-type aldolase) (Aldolase 1)	12	22	363	39225	8.1	Cytoplasm	enzyme	NONE	0.001995
P05201	AATC_MOUSE	GOT1	(P05201) Aspartate aminotransferase, cytoplasmic (EC 2.6.1.1) (Transaminase A) (Glutamate oxaloacetate transaminase 1)	2	8.5	412	46100	7.2	Cytoplasm	enzyme	NONE	5.45E-05
P05202	AATM_MOUSE	GOT2	(P05202) Aspartate aminotransferase, mitochondrial precursor (EC 2.6.1.1) (Transaminase A) (Glutamate oxaloacetate transaminase 2)	14	25.3	430	47411	9	Cytoplasm	enzyme	NONE	0.000522
P05213	TBA2_MOUSE	K-ALPHA-1	(P05213) Tubulin alpha-2 chain (Alpha-tubulin 2) (Alpha-tubulin isotype M-alpha-2)	2	6	451	50152	5.1	Cytoplasm	other	NONE	0.000336
P05627	JUN_MOUSE	JUN	(P05627) Transcription factor AP-1 (Activator protein 1) (AP1) (Proto-oncogene c-jun) (V-jun avian sarcoma virus 17 oncogene homolog) (Jun A) (AH119)	2	5.1	334	35944	8.7	Nucleus	transcription regulator	NONE	5.04E-05
P05784	K1C18_MOUSE	KRT18	(P05784) Keratin, type I cytoskeletal 18 (Cytokeratin-18) (CK-18) (Keratin-18) (K18) (Cytokeratin endo B) (Keratin D)	6	19	422	47373	5.3	Cytoplasm	other	NONE	0.000239
P06151	LDHA_MOUSE	LDHA	(P06151) L-lactate dehydrogenase A chain (EC 1.1.1.27) (LDH-A) (LDH muscle subunit) (LDH-M)	9	20.2	331	36367	7.7	Cytoplasm	enzyme	NONE	0.000203
P06281	RENI1_MOUSE	REN	(P06281) Renin-1 precursor (EC 3.4.23.15) (Angiotensinogenase) (Kidney renin)	1	4	402	44343	7.2	Extracellular Space	peptidase	NONE	1.4E-05
P06537	GCR_MOUSE	NR3C1	(P06537) Glucocorticoid receptor (GR)	2	4.9	783	86053	6.4	Nucleus	ligand-dependent nuclear receptor	NONE	2.87E-05
P06683	CO9_MOUSE	C9	(P06683) Complement component C9 precursor	2	6.4	548	62002	5.8	Extracellular Space	other	1	2.05E-05
P06745	G6PI_MOUSE	GPI	(P06745) Glucose-6-phosphate isomerase (EC 5.3.1.9) (GPI) (Phosphoglucose isomerase) (PGI) (Phosphohexose isomerase) (PHI) (Neuroleukin) (NLK)	13	26.9	557	62637	7.9	Extracellular Space	enzyme	NONE	0.000403
P06797	CATL_MOUSE	CTSL2	(P06797) Cathepsin L precursor (EC 3.4.22.15) (Major excreted protein) (MEP) (p39 cysteine proteinase) [Contains: Cathepsin L heavy chain; Cathepsin L light chain]	3	19.2	334	37547	6.8	Cytoplasm	peptidase	1	0.000538
P06801	MAOX_MOUSE	ME1	(P06801) NADP-dependent malic enzyme (EC 1.1.1.40) (NADP-ME) (Malic enzyme 1)	5	13.8	572	63999	7.4	Cytoplasm	enzyme	NONE	7.85E-05
P06909	CFAH_MOUSE	CFH	(P06909) Complement factor H precursor (Protein beta-1-H)	1	1.5	1234	139082	6.9	Extracellular Space	other	1	4.55E-06
P07146	TRY2_MOUSE	PRSS2 (includes EG:22072)	(P07146) Anionic trypsin-2 precursor (EC 3.4.21.4) (Anionic trypsin II) (Pretrypsinogen II)	1	8.1	246	26204	4.6	Extracellular Space	peptidase	1	2.28E-05
P07309	TTHY_MOUSE	TTR	(P07309) Transthyretin precursor (Prealbumin)	2	25.2	147	15776	6.2	Extracellular Space	transporter	1	0.000802

P07356	ANXA2_MOUSE	ANXA2	(P07356) Annexin A2 (Annexin II) (Lipocortin II) (Calpactin I heavy chain) (Chromobindin-8) (p36) (Protein I) (Placental anticoagulant protein IV) (PAP-IV)	10	18.3	338	38545	7.7	Plasma Membrane	other	NONE	0.000332
P07724	ALBU_MOUSE	ALB	(P07724) Serum albumin precursor	14	27	608	68693	6.1	Extracellular Space	transporter	NONE	0.001025
P07758	A1AT1_MOUSE	SERPINA1	(P07758) Alpha-1-antitrypsin 1-1 precursor (Serine protease inhibitor 1-1) (Alpha-1 protease inhibitor 1) (Alpha-1-antiproteinase) (AAT)	4	14.5	413	46003	5.7	Extracellular Space	other	2	0.000163
P07901	HS90A_MOUSE	HSP90AA1	(P07901) Heat shock protein HSP 90-alpha (HSP 86) (Tumor-specific transplantation 86 kDa antigen) (TSTA)	41	38.9	732	84657	5	Cytoplasm	other	NONE	0.0025
P07934	PHKG1_MOUSE	PHKG1	(P07934) Phosphorylase b kinase gamma catalytic chain, skeletal muscle isoform (EC 2.7.11.19) (Phosphorylase kinase subunit gamma 1)	2	5.2	387	44829	6.5	Cytoplasm	kinase	NONE	2.9E-05
P08003	PDIA4_MOUSE	PDIA4	(P08003) Protein disulfide-isomerase A4 precursor (EC 5.3.4.1) (Protein ERp-72) (ERp72)	23	31.3	638	71973	5.3	Cytoplasm	enzyme	1	0.000607
P08113	ENPL_MOUSE	HSP90B1	(P08113) Endoplasmin precursor (Heat shock protein 90 kDa beta member 1) (94 kDa glucose-regulated protein) (GRP94) (ERP99) (Polymorphic tumor rejection antigen 1) (Tumor rejection antigen gp96)	36	28.8	802	92476	4.8	Cytoplasm	other	1	0.001694
P08207	S10AA_MOUSE	S100A10	(P08207) Protein S100-A10 (S100 calcium-binding protein A10) (Calpactin-1 light chain) (Calpactin I light chain) (p10 protein) (p11) (Cellular ligand of annexin II)	2	35.4	96	11055	6.8	Cytoplasm	other	NONE	0.000468
P08226	APOE_MOUSE	APOE	(P08226) Apolipoprotein E precursor (Apo-E)	2	10.6	311	35867	5.7	Extracellular Space	transporter	1	5.41E-05
P08228	SODC_MOUSE	SOD1	(P08228) Superoxide dismutase [Cu-Zn] (EC 1.15.1.1)	5	36.6	153	15811	6.5	Cytoplasm	enzyme	NONE	0.005797
P08249	MDHM_MOUSE	MDH2	(P08249) Malate dehydrogenase, mitochondrial precursor (EC 1.1.1.37)	24	53.6	338	35596	8.6	Cytoplasm	enzyme	NONE	0.005746
P08752	GNAI2_MOUSE	GNAI2	(P08752) Guanine nucleotide-binding protein G(i), alpha-2 subunit (Adenylate cyclase-inhibiting G alpha protein)	1	4.2	354	40340	5.5	Plasma Membrane	enzyme	NONE	3.17E-05
P08775	RPB1_MOUSE	POLR2A	(P08775) DNA-directed RNA polymerase II largest subunit (EC 2.7.7.6) (RPB1)	2	1.7	1970	217174	7.4	Nucleus	enzyme	NONE	8.55E-06
P08905	LYSCM_MOUSE	LYZ	(P08905) Lysozyme C type M precursor (EC 3.2.1.17) (1,4-beta-N-acetylmuramidase C)	6	37.8	148	16689	8.8	Extracellular Space	enzyme	1	0.001176
P09055	ITB1_MOUSE	ITGB1	(P09055) Integrin beta-1 precursor (Fibronectin receptor subunit beta) (Integrin VLA-4 subunit beta) (CD29 antigen)	6	6.3	798	88231	5.9	Plasma Membrane	transmembrane receptor	2	0.000162
P09103	PDIA1_MOUSE	P4HB	(P09103) Protein disulfide-isomerase precursor (EC 5.3.4.1) (PDI) (Prolyl 4-hydroxylase subunit beta) (Cellular thyroid hormone-binding protein) (p55) (Erp59)	33	50.9	509	57144	4.9	Cytoplasm	enzyme	1	0.00236
P09242	PPBT_MOUSE	ALPL	(P09242) Alkaline phosphatase, tissue-nonspecific isozyme precursor (EC 3.1.3.1) (AP-TNAP) (TNSALP)	11	13.4	524	57455	6.9	Plasma Membrane	phosphatase	2	0.000236
P09405	NUCL_MOUSE	NCL	(P09405) Nucleolin (Protein C23)	15	22	706	76592	4.8	Nucleus	other	NONE	0.000445
P09411	PGK1_MOUSE	PGK1	(P09411) Phosphoglycerate kinase 1 (EC 2.7.2.3)	33	54.8	416	44405	7.6	Cytoplasm	kinase	NONE	0.003751
P09470	ACE_MOUSE	ACE	(P09470) Angiotensin-converting enzyme, somatic isoform precursor (EC 3.4.15.1) (Dipeptidyl carboxypeptidase I) (Kininase II) [Contains: Angiotensin-converting enzyme, somatic isoform, soluble form]	1	1.4	1312	150918	6.6	Plasma Membrane	peptidase	2	1.28E-05
P09528	FRIH_MOUSE	FTH1	(P09528) Ferritin heavy chain (EC 1.16.3.1) (Ferritin H subunit)	4	23.2	181	20935	5.9	Cytoplasm	enzyme	NONE	0.000279
P09671	SODM_MOUSE	SOD2	(P09671) Superoxide dismutase [Mn], mitochondrial precursor (EC 1.15.1.1)	10	37.8	222	24603	8.6	Cytoplasm	enzyme	NONE	0.001239
P09803	CADH1_MOUSE	CDH1	(P09803) Epithelial-cadherin precursor (E-cadherin) (Uvomorulin) (Cadherin-1) (ARC-1) (CD324 antigen) [Contains: E-Cad/CTF1; E-Cad/CTF2; E-Cad/CTF3]	12	16	884	98256	4.8	Plasma Membrane	other	3	0.000222
P09925	SURF1_MOUSE	SURF1	(P09925) Surfeit locus protein 1	3	19.3	306	34798	9.7	Cytoplasm	enzyme	NONE	0.000165
P0C0A3	CHMP6_MOUSE	CHMP6	(P0C0A3) Charged multivesicular body protein 6 (Chromatin-modifying protein 6)	1	6.5	199	23284	5.4	Cytoplasm	other	NONE	5.64E-05
P10107	ANXA1_MOUSE	ANXA1	(P10107) Annexin A1 (Annexin I) (Lipocortin I) (Calpactin II) (Chromobindin-9) (p35) (Phospholipase A2 inhibitory protein)	4	17.1	345	38603	7.4	Plasma Membrane	other	NONE	0.00013

P10126	EF1A1_MOUSE	EEF1A1	(P10126) Elongation factor 1-alpha 1 (EF-1-alpha-1) (Elongation factor 1 A-1) (eEF1A-1) (Elongation factor Tu) (EF-Tu)	25	32.9	462	50114	9	Cytoplasm	translation regulator	NONE	0.003365
P10493	NID1_MOUSE	NID1	(P10493) Nidogen-1 precursor (Entactin)	10	9.1	1245	136623	5.5	Extracellular Space	other	1	0.000122
P10518	HEM2_MOUSE	ALAD	(P10518) Delta-aminolevulinic acid dehydratase (EC 4.2.1.24) (Porphobilinogen synthase) (ALADH)	5	29.7	330	36024	6.8	Cytoplasm	enzyme	NONE	0.000204
P10605	CATB_MOUSE	CTSB	(P10605) Cathepsin B precursor (EC 3.4.22.1) (Cathepsin B1) [Contains: Cathepsin B light chain; Cathepsin B heavy chain]	3	15	339	37280	5.9	Cytoplasm	peptidase	NONE	9.93E-05
P10637	TAU_MOUSE	MAPT	(P10637) Microtubule-associated protein tau (Neurofibrillary tangle protein) (Paired helical filament-tau) (PHF-tau)	9	16.3	732	76112	6.8	Cytoplasm	other	NONE	0.000245
P10639	THIO_MOUSE	TXN	(P10639) Thioredoxin (ATL-derived factor) (ADF)	1	12.5	104	11544	4.9	Cytoplasm	enzyme	NONE	0.000486
P10649	GSTM1_MOUSE	GSTM5	(P10649) Glutathione S-transferase Mu 1 (EC 2.5.1.18) (GST class-mu 1) (Glutathione S-transferase GT8.7) (pmGT10) (GST 1-1)	23	48.8	217	25839	8	Cytoplasm	enzyme	NONE	0.003337
P10711	TCEA1_MOUSE	TCEA1	(P10711) Transcription elongation factor A protein 1 (Transcription elongation factor S-II protein 1) (Transcription elongation factor TFIIS.o)	2	10	301	33880	8.4	Nucleus	transcription regulator	NONE	5.59E-05
P10833	RRAS_MOUSE	RRAS	(P10833) Ras-related protein R-Ras (P23)	3	19.7	218	23764	6.8	Cytoplasm	enzyme	NONE	0.000129
P10852	4F2_MOUSE	SLC3A2	(P10852) 4F2 cell-surface antigen heavy chain (4F2hc)	8	17.1	526	58337	5.9	Plasma Membrane	transporter	1	0.000224
P10922	H10_MOUSE	H1F0	(P10922) Histone H1' (H1.0) (H1(0))	17	29	193	20730	10.9	Nucleus	other	NONE	0.003374
P10923	OSTP_MOUSE	SPP1	(P10923) Osteopontin precursor (Bone sialoprotein-1) (Secreted phosphoprotein 1) (SPP-1) (Minopontin) (Early T-lymphocyte activation 1 protein) (2AR) (Calcium oxalate crystal growth inhibitor protein)	2	8.2	294	32459	4.5	Extracellular Space	cytokine	1	7.64E-05
P11031	TCP4_MOUSE	SUB1	(P11031) Activated RNA polymerase II transcriptional coactivator p15 precursor (SUB1 homolog) (Positive cofactor 4) (PC4) (p14) (Single-stranded DNA-binding protein p9)	1	11.1	126	14296	9.6	Nucleus	transcription regulator	NONE	4.45E-05
P11103	PARP1_MOUSE	PARP1	(P11103) Poly [ADP-ribose] polymerase 1 (EC 2.4.2.30) (PARP-1) (ADPRT) (NAD(+) ADP-ribosyltransferase 1) (Poly[ADP-ribose] synthetase 1) (msPARP)	5	9.6	1012	112969	9	Nucleus	enzyme	NONE	3.33E-05
P11152	LIPL_MOUSE	LPL	(P11152) Lipoprotein lipase precursor (EC 3.1.1.34) (LPL)	1	4	474	53127	8	Cytoplasm	enzyme	1	4.74E-05
P11276	FINC_MOUSE	FN1	(P11276) Fibronectin precursor (FN)	2	1	2477	272487	5.6	Plasma Membrane	enzyme	NONE	4.53E-06
P11352	GPX1_MOUSE	GPX1	(P11352) Glutathione peroxidase 1 (EC 1.11.1.9) (GSHPx-1) (GPx-1) (Cellular glutathione peroxidase)	8	46.8	201	22282	7.2	Cytoplasm	enzyme	NONE	0.00377
P11404	FABPH_MOUSE	FABP3	(P11404) Fatty acid-binding protein, heart (H-FABP) (Heart-type fatty acid-binding protein) (Mammary-derived growth inhibitor) (MDGI)	7	51.5	132	14688	6.6	Cytoplasm	transporter	NONE	0.000595
P11499	HS90B_MOUSE	HSP90AB1	(P11499) Heat shock protein HSP 90-beta (HSP 84) (Tumor-specific transplantation 84 kDa antigen) (TSTA)	32	33.2	723	83194	5	Cytoplasm	other	NONE	0.001133
P11588	MUP1_MOUSE	MUP1	(P11588) Major urinary protein 1 precursor (MUP 1)	6	40.6	180	20648	5.1	Extracellular Space	transporter	1	0.000967
P11679	K2C8_MOUSE	KRT8	(P11679) Keratin, type II cytoskeletal 8 (Cytokeratin-8) (CK-8) (Keratin-8) (K8) (Cytokeratin endo A)	2	5.7	489	54434	5.8	Cytoplasm	kinase	NONE	2.3E-05
P11835	ITB2_MOUSE	ITGB2	(P11835) Integrin beta-2 precursor (Cell surface adhesion glycoproteins LFA-1/CR3/P150,95 subunit beta) (Complement receptor C3 subunit beta) (CD18 antigen)	1	1.8	771	85026	7.1	Plasma Membrane	other	2	1.46E-05
P11862	GAS2_MOUSE	GAS2	(P11862) Growth-arrest-specific protein 2 (GAS-2)	11	32.5	314	34901	8.6	Cytoplasm	other	NONE	0.000644
P11930	NUD19_MOUSE	NUDT19	(P11930) Nucleoside diphosphate-linked moiety X motif 19 (EC 3.-.-.-) (Nudix motif 19) (Testosterone-regulated RP2 protein) (Androgen-regulated protein RP2)	7	33.1	357	40368	6.7	Unknown	other	NONE	0.000252
P11984	TCPA1_MOUSE	TCP1	(P11984) T-complex protein 1 subunit alpha A (TCP-1-alpha) (CCT-alpha) (Tailless complex polypeptide 1A) (TCP-1-A)	2	6.1	556	60341	6.1	Cytoplasm	other	NONE	2.02E-05
P12265	BGLR_MOUSE	GUSB	(P12265) Beta-glucuronidase precursor (EC 3.2.1.31)	2	2.9	648	74239	6.6	Cytoplasm	enzyme	1	2.6E-05
P12367	KAP2_MOUSE	PRKAR2A	(P12367) cAMP-dependent protein kinase type II-alpha regulatory subunit	5	10.5	400	45258	4.9	Cytoplasm	kinase	NONE	0.000154

P12382	K6PL_MOUSE	PFKL	(P12382) 6-phosphofructokinase, liver type (EC 2.7.1.11) (Phosphofructokinase 1) (Phosphohexokinase) (Phosphofructo-1-kinase isozyme B) (PFK-B)	1	2.2	779	85170	7.1	Cytoplasm	kinase	NONE	7.21E-06
P12658	CALB1_MOUSE	CALB1	(P12658) Calbindin (Vitamin D-dependent calcium-binding protein, avian-type) (Calbindin D28) (D-28K) (Spot 35 protein) (PCD-29)	23	43.5	260	29863	4.8	Cytoplasm	other	NONE	0.003109
P12710	FABPL_MOUSE	FABP1	(P12710) Fatty acid-binding protein, liver (L-FABP) (14 kDa selenium-binding protein)	2	17.3	127	14246	8.5	Cytoplasm	transporter	NONE	8.84E-05
P12787	COX5A_MOUSE	COX5A	(P12787) Cytochrome c oxidase polypeptide Va, mitochondrial precursor (EC 1.9.3.1)	10	22.1	145	16030	6.5	Cytoplasm	enzyme	NONE	0.003407
P12815	PDCD6_MOUSE	PDCD6	(P12815) Programmed cell death protein 6 (Probable calcium-binding protein ALG-2) (PMP41) (ALG-257)	3	29.3	191	21867	5.4	Cytoplasm	other	NONE	0.000147
P12970	RL7A_MOUSE	RPL7A (includes EG:27176)	(P12970) 60S ribosomal protein L7a (Surfeit locus protein 3)	8	19.6	265	29845	10.6	Nucleus	other	NONE	0.000699
P13020	GELS_MOUSE	GSN	(P13020) Gelsolin precursor (Actin-depolymerizing factor) (ADF) (Brevin)	4	11.4	780	85942	6.2	Extracellular Space	other	1	5.76E-05
P13707	GPDA_MOUSE	GPD1	(P13707) Glycerol-3-phosphate dehydrogenase [NAD+], cytoplasmic (EC 1.1.1.8) (GPD-C) (GPDH-C)	3	9.2	348	37442	7.2	Cytoplasm	enzyme	NONE	0.000161
P13745	GSTA1_MOUSE	GSTA5	(P13745) Glutathione S-transferase Ya chain (EC 2.5.1.18) (GST class-alpha) (Ya1)	1	8.1	222	25477	9	Cytoplasm	enzyme	NONE	7.59E-05
P14094	AT1B1_MOUSE	ATP1B1	(P14094) Sodium/potassium-transporting ATPase subunit beta-1 (Sodium/potassium-dependent ATPase beta-1 subunit)	14	31.9	304	35195	8.6	Plasma Membrane	transporter	1	0.001237
P14115	RL27A_MOUSE	RPL27A	(P14115) 60S ribosomal protein L27a (L29)	2	17.7	147	16458	11.1	Cytoplasm	other	NONE	0.00042
P14131	RS16_MOUSE	RPS16	(P14131) 40S ribosomal protein S16	7	31	145	16314	10.2	Cytoplasm	other	NONE	0.001045
P14148	RL7_MOUSE	RPL7	(P14148) 60S ribosomal protein L7	6	21.1	270	31420	10.9	Cytoplasm	transcription regulator	NONE	0.000499
P14152	MDHC_MOUSE	MDH1	(P14152) Malate dehydrogenase, cytoplasmic (EC 1.1.1.37) (Cytosolic malate dehydrogenase)	10	26.1	333	36380	6.6	Cytoplasm	enzyme	NONE	0.004197
P14206	RSSA_MOUSE	RPSA	(P14206) 40S ribosomal protein SA (p40) (34/67 kDa laminin receptor)	2	10.9	294	32588	4.8	Plasma Membrane	transmembrane receptor	NONE	9.55E-05
P14211	CRTC_MOUSE	CALR	(P14211) Calreticulin precursor (CRP55) (Calregulin) (HACBP) (ERp60)	20	39.7	416	47995	4.5	Cytoplasm	transcription regulator	1	0.00251
P14246	GTR2_MOUSE	SLC2A2	(P14246) Solute carrier family 2, facilitated glucose transporter member 2 (Glucose transporter type 2, liver)	1	1.9	523	57107	6.7	Plasma Membrane	transporter	9	2.15E-05
P14483	HB2A_MOUSE	HLA-DQB2	(P14483) H-2 class II histocompatibility antigen, A beta chain precursor	3	14.3	265	30128	7.2	Plasma Membrane	transmembrane receptor	2	8.47E-05
P14576	SRP54_MOUSE	SRP54	(P14576) Signal recognition particle 54 kDa protein (SRP54)	3	8.1	504	55721	8.8	Cytoplasm	other	NONE	5.57E-05
P14602	HSPB1_MOUSE	HSPB1	(P14602) Heat-shock protein beta-1 (HspB1) (Heat shock 27 kDa protein) (HSP 27) (Growth-related 25 kDa protein) (P25) (HSP25)	6	27.3	209	23014	6.5	Cytoplasm	other	NONE	0.000349
P14685	PSD3_MOUSE	PSMD3	(P14685) 26S proteasome non-ATPase regulatory subunit 3 (26S proteasome regulatory subunit S3) (Proteasome subunit p58) (Transplantation antigen P91A) (Tum-P91A antigen)	1	4.2	530	60699	8.2	Cytoplasm	other	NONE	1.06E-05
P14733	LMNB1_MOUSE	LMNB1	(P14733) Lamin-B1	6	17.2	587	66654	5.2	Nucleus	other	NONE	0.000105
P14824	ANXA6_MOUSE	ANXA6	(P14824) Annexin A6 (Annexin VI) (Lipocortin VI) (P68) (P70) (Protein III) (Chromobindin-20) (67 kDa calelectrin) (Calphobindin-II) (CPB-II)	5	13.1	672	75755	5.5	Plasma Membrane	other	NONE	7.52E-05
P14869	RLA0_MOUSE	RPLP0 (includes EG:6175)	(P14869) 60S acidic ribosomal protein P0 (L10E)	7	27.1	317	34216	6.2	Cytoplasm	other	NONE	0.000336
P15092	IFI4_MOUSE	IFI16	(P15092) Interferon-activable protein 204 (Ifi-204) (Interferon-inducible protein p204)	1	3.6	640	71648	8.8	Nucleus	transcription regulator	NONE	3.51E-05
P15532	NDKA_MOUSE	NME1	(P15532) Nucleoside diphosphate kinase A (EC 2.7.4.6) (NDK A) (NDP kinase A) (Tumor metastatic process-associated protein) (Metastasis inhibition factor NM23) (NDPK-A) (nm23-M1)	15	67.8	152	17208	7.4	Nucleus	kinase	NONE	0.002511
P15626	GSTM2_MOUSE	GSTM1	(P15626) Glutathione S-transferase Mu 2 (EC 2.5.1.18) (GST class-mu 2) (Glutathione S-transferase pmGT2) (GST 5-5)	2	4.6	217	25585	7.4	Cytoplasm	enzyme	NONE	0.000129

P15864	H12_MOUSE	HIST1H1C	(P15864) Histone H1.2 (H1 VAR.1) (H1c)	10	27.5	211	21135	11	Nucleus	other	NONE	0.001064
P15945	K1KB5_MOUSE	KLK1B5	(P15945) Kallikrein 1-related peptidase b5 precursor (EC 3.4.21.35) (Glandular kallikrein K5) (Tissue kallikrein-5) (mGK-5)	2	8.4	261	28748	5.6	Unknown	peptidase	1	4.3E-05
P16015	CAH3_MOUSE	CA3	(P16015) Carbonic anhydrase 3 (EC 4.2.1.1) (Carbonic anhydrase III) (Carbonate dehydratase III) (CA-III)	3	16.6	259	29235	7.4	Cytoplasm	enzyme	NONE	8.67E-05
P16056	MET_MOUSE	MET	(P16056) Hepatocyte growth factor receptor precursor (EC 2.7.10.1) (HGF receptor) (Scatter factor receptor) (SF receptor) (HGF/SF receptor) (Met proto-oncogene tyrosine kinase) (c-Met)	1	1.2	1379	153548	7.1	Plasma Membrane	kinase	2	4.07E-06
P16110	LEG3_MOUSE	LGALS3	(P16110) Galectin-3 (Galactose-specific lectin 3) (Mac-2 antigen) (IgE-binding protein) (35 kDa lectin) (Carbohydrate-binding protein 35) (CBP 35) (Laminin-binding protein) (Lectin L-29) (L-34 galactoside-binding lectin)	2	9.5	263	27384	8.4	Extracellular Space	other	NONE	8.54E-05
P16125	LDHB_MOUSE	LDHB	(P16125) L-lactate dehydrogenase B chain (EC 1.1.1.27) (LDH-B) (LDH heart subunit) (LDH-H)	23	35.1	333	36441	6.1	Cytoplasm	enzyme	NONE	0.001079
P16331	PH4H_MOUSE	PAH	(P16331) Phenylalanine-4-hydroxylase (EC 1.14.16.1) (PAH) (Phe-4-monooxygenase)	13	23.5	452	51798	6.4	Cytoplasm	enzyme	NONE	0.000472
P16332	MUTA_MOUSE	MUT	(P16332) Methylmalonyl-CoA mutase, mitochondrial precursor (EC 5.4.99.2) (MCM) (Methylmalonyl-CoA isomerase)	6	12.7	748	82965	7.1	Cytoplasm	enzyme	NONE	8.25E-05
P16406	AMPE_MOUSE	ENPEP	(P16406) Glutamyl aminopeptidase (EC 3.4.11.7) (EAP) (Aminopeptidase A) (APA) (BP-1/6C3 antigen)	14	17.8	945	107956	5.4	Plasma Membrane	peptidase	1	0.000137
P16460	ASSY_MOUSE	ASS1	(P16460) Argininosuccinate synthase (EC 6.3.4.5) (Citrulline--aspartate ligase)	28	44.9	412	46585	8.2	Cytoplasm	enzyme	NONE	0.004305
P16546	SPTA2_MOUSE	SPTAN1	(P16546) Spectrin alpha chain, brain (Spectrin, non-erythroid alpha chain) (Alpha-II spectrin) (Fodrin alpha chain) (Fragment)	34	28.9	1458	167552	5.4	Plasma Membrane	other	NONE	0.000262
P16858	G3P_MOUSE	LOC14433	(P16858) Glyceraldehyde-3-phosphate dehydrogenase (EC 1.2.1.12) (GAPDH)	33	50.6	332	35679	8.2	Cytoplasm	enzyme	NONE	0.011801
P17047	LAMP2_MOUSE	LAMP2	(P17047) Lysosome-associated membrane glycoprotein 2 precursor (LAMP-2) (Lysosomal membrane glycoprotein type B) (LGP-B) (CD107b antigen)	1	2.4	415	45647	7.4	Plasma Membrane	enzyme	2	8.12E-05
P17156	HSP72_MOUSE	HSPA2	(P17156) Heat shock-related 70 kDa protein 2 (Heat shock protein 70.2)	9	15.5	633	69741	5.8	Cytoplasm	other	NONE	0.000514
P17182	ENOA_MOUSE	ENO1	(P17182) Alpha-enolase (EC 4.2.1.11) (2-phospho-D-glycerate hydro-lyase) (Non-neural enolase) (NNE) (Enolase 1)	47	75.5	433	47010	6.8	Cytoplasm	transcription regulator	NONE	0.010915
P17225	PTBP1_MOUSE	PTBP1	(P17225) Polypyrimidine tract-binding protein 1 (PTB) (Heterogeneous nuclear ribonucleoprotein I) (hnRNP I)	7	22.4	527	56478	8.3	Nucleus	enzyme	NONE	0.000543
P17563	SBP1_MOUSE	SELENBP1	(P17563) Selenium-binding protein 1 (56 kDa selenium-binding protein) (SP56)	16	31.4	472	52352	6.4	Cytoplasm	other	NONE	0.000714
P17665	COX7C_MOUSE	COX7C	(P17665) Cytochrome c oxidase polypeptide VIIc, mitochondrial precursor (EC 1.9.3.1)	4	31.7	63	7333	11	Cytoplasm	enzyme	1	0.000713
P17710	HXK1_MOUSE	HK1	(P17710) Hexokinase-1 (EC 2.7.1.1) (Hexokinase type I) (HK I) (Hexokinase, tumor isozyme)	2	3.4	974	108302	6.9	Cytoplasm	kinase	NONE	2.31E-05
P17717	UDB5_MOUSE	UGT2B5	(P17717) UDP-glucuronosyltransferase 2B5 precursor (EC 2.4.1.17) (UDPGT) (M-1)	5	9.2	530	60856	7.9	Cytoplasm	enzyme	2	0.000127
P17742	PPIA_MOUSE	PPIA	(P17742) Peptidyl-prolyl cis-trans isomerase A (EC 5.2.1.8) (PPIase A) (Rotamase A) (Cyclophilin A) (Cyclosporin A-binding protein) (SP18)	21	83.4	163	17840	7.9	Cytoplasm	enzyme	NONE	0.012328
P17751	TPIS_MOUSE	TPI1	(P17751) Triosephosphate isomerase (EC 5.3.1.1) (TIM) (Triose-phosphate isomerase)	12	48.8	248	26581	7.3	Cytoplasm	enzyme	NONE	0.003305
P18242	CATD_MOUSE	CTSD	(P18242) Cathepsin D precursor (EC 3.4.23.5)	13	30.2	410	44954	7.1	Cytoplasm	peptidase	1	0.000493
P18572	BASI_MOUSE	BSG	(P18572) Basigin precursor (Basic immunoglobulin superfamily) (Membrane glycoprotein gp42) (HT7 antigen) (CD147 antigen)	4	13.6	389	42445	5.8	Plasma Membrane	other	2	0.000188
P18760	COF1_MOUSE	CFL1	(P18760) Cofilin-1 (Cofilin, non-muscle isoform)	6	41.2	165	18428	8.1	Nucleus	other	NONE	0.005035
P19157	GSTP1_MOUSE	GSTP1	(P19157) Glutathione S-transferase P 1 (EC 2.5.1.18) (GST YF-YF) (GST-piB) (GST class-pi) (Gst P1) (Preadipocyte growth factor)	11	32.1	209	23478	7.8	Cytoplasm	enzyme	NONE	0.001585

P19253	RL13A_MOUSE	RPL13A	(P19253) 60S ribosomal protein L13a (Transplantation antigen P198) (Tum-P198 antigen)	6	16.3	202	23333	11	Cytoplasm	other	NONE	0.0005
P19324	HSP47_MOUSE	SERPINH1	(P19324) 47 kDa heat shock protein precursor (Collagen-binding protein 1) (Serine protease inhibitor J6)	11	35.5	417	46590	8.8	Extracellular Space	other	2	0.00074
P19536	COX5B_MOUSE	COX5B	(P19536) Cytochrome c oxidase polypeptide Vb, mitochondrial precursor (EC 1.9.3.1)	11	52.3	128	13813	8.4	Cytoplasm	enzyme	NONE	0.00171
P19783	COX41_MOUSE	COX4I1	(P19783) Cytochrome c oxidase subunit 4 isoform 1, mitochondrial precursor (EC 1.9.3.1) (Cytochrome c oxidase subunit IV isoform 1) (COX IV-1) (Cytochrome c oxidase polypeptide IV)	17	53.3	169	19530	9.2	Cytoplasm	enzyme	2	0.002192
P19973	LSP1_MOUSE	LSP1	(P19973) Lymphocyte-specific protein 1 (Protein pp52) (52 kDa phosphoprotein) (Lymphocyte-specific antigen WP34) (S37 protein)	1	7.3	330	36714	4.8	Cytoplasm	other	NONE	1.7E-05
P20029	GRP78_MOUSE	HSPA5	(P20029) 78 kDa glucose-regulated protein precursor (GRP 78) (Immunoglobulin heavy chain-binding protein) (BiP)	47	52.2	655	72422	5.2	Cytoplasm	other	1	0.002742
P20060	HEXB_MOUSE	HEXB	(P20060) Beta-hexosaminidase beta chain precursor (EC 3.2.1.52) (N-acetyl-beta-glucosaminidase) (Beta-N-acetylhexosaminidase) (Hexosaminidase B)	2	4.9	536	61116	8.1	Cytoplasm	enzyme	1	3.14E-05
P20065	TYB4_MOUSE	TMSB4X	(P20065) Thymosin beta-4 (T beta 4) [Contains: Hematopoietic system regulatory peptide (Seraspenide)]	2	38	50	5679	5	Cytoplasm	other	NONE	0.001684
P20108	PRDX3_MOUSE	PRDX3	(P20108) Thioredoxin-dependent peroxide reductase, mitochondrial precursor (EC 1.11.1.15) (Perioredoxin-3) (PRX III) (Antioxidant protein 1) (AOP-1) (Protein MER5)	6	21.4	257	28127	7.6	Cytoplasm	enzyme	NONE	0.00107
P20152	VIME_MOUSE	VIM	(P20152) Vimentin	17	34.4	465	53557	5.1	Cytoplasm	other	NONE	0.001014
P21107	TPM3_MOUSE	TPM3	(P21107) Tropomyosin alpha-3 chain (Tropomyosin-3) (Tropomyosin gamma)	10	19.7	284	32863	4.7	Cytoplasm	other	NONE	0.000593
P21126	UBL4A_MOUSE	UBL4A	(P21126) Ubiquitin-like protein 4A (Ubiquitin-like protein GDX)	1	7.6	157	17801	8.4	Unknown	enzyme	NONE	3.58E-05
P21271	MYO5B_MOUSE	MYO5B	(P21271) Myosin-5B (Myosin Vb) (Fragment)	2	3.6	723	83200	5.5	Cytoplasm	enzyme	NONE	1.55E-05
P21278	GNA11_MOUSE	GNA11	(P21278) Guanine nucleotide-binding protein alpha-11 subunit	2	9.2	359	42024	6	Plasma Membrane	enzyme	NONE	3.13E-05
P21279	GNAQ_MOUSE	GNAQ	(P21279) Guanine nucleotide-binding protein G(q) subunit alpha (Guanine nucleotide-binding protein alpha-q)	1	4.2	353	41483	5.8	Plasma Membrane	enzyme	NONE	1.59E-05
P21460	CYTC_MOUSE	CST3	(P21460) Cystatin C precursor (Cystatin 3)	1	12.1	140	15531	9	Extracellular Space	other	1	4.01E-05
P21614	VTDB_MOUSE	GC	(P21614) Vitamin D-binding protein precursor (DBP) (Group-specific component) (Gc-globulin) (VDB)	2	8.6	476	53600	5.5	Extracellular Space	transporter	NONE	5.9E-05
P21619	LMNB2_MOUSE	LMNB2	(P21619) Lamin-B2	5	8.1	596	67318	5.5	Nucleus	other	NONE	8.48E-05
P21981	TGM2_MOUSE	TGM2	(P21981) Protein-glutamine gamma-glutamyltransferase 2 (EC 2.3.2.13) (Tissue transglutaminase) (TGase C) (TGC) (TG(C)) (Transglutaminase-2)	7	12.4	686	77046	5.1	Cytoplasm	enzyme	NONE	0.000131
P22315	HEMH_MOUSE	FECH	(P22315) Ferrochelatase, mitochondrial precursor (EC 4.99.1.1) (Protoheme ferro-lyase) (Heme synthetase)	6	15.7	420	47130	8.9	Cytoplasm	enzyme	NONE	0.000134
P22361	HNF1A_MOUSE	TCF1	(P22361) Hepatocyte nuclear factor 1-alpha (HNF-1A) (Liver-specific transcription factor LF-B1) (LFB1)	2	5.6	628	67237	6.2	Nucleus	transcription regulator	NONE	2.68E-05
P22599	A1AT2_MOUSE	SERPINA1	(P22599) Alpha-1-antitrypsin 1-2 precursor (Serine protease inhibitor 1-2) (Alpha-1 protease inhibitor 2) (Alpha-1-antiproteinase) (AAT)	3	10.2	413	45975	5.5	Extracellular Space	other	2	4.08E-05
P22892	AP1G1_MOUSE	AP1G1	(P22892) AP-1 complex subunit gamma-1 (Adapter-related protein complex 1 gamma-1 subunit) (Gamma-adaptin) (Adaptor protein complex AP-1 gamma-1 subunit) (Golgi adaptor HA1/AP1 adaptin subunit gamma-1) (Clathrin assembly protein complex 1 gamm	2	3.3	821	91219	6.8	Cytoplasm	transporter	NONE	4.79E-05
P23116	IF3A_MOUSE	EIF3S10	(P23116) Eukaryotic translation initiation factor 3 subunit 10 (eIF-3 theta) (eIF3 p167) (eIF3 p180) (eIF3 p185) (eIF3a) (p162 protein) (Centrosomin)	4	3.5	1344	161950	6.8	Cytoplasm	translation regulator	NONE	2.09E-05
P23198	CBX3_MOUSE	CBX3	(P23198) Chromobox protein homolog 3 (Heterochromatin protein 1 homolog gamma) (HP1 gamma) (Modifier 2 protein) (M32)	6	15.8	183	20855	5.2	Nucleus	other	NONE	0.000368

P23492	PNPH_MOUSE	NP	(P23492) Purine nucleoside phosphorylase (EC 2.4.2.1) (Inosine phosphorylase) (PNP)	3	13.1	289	32277	6.2	Nucleus	enzyme	NONE	0.000117
P23506	PIMT_MOUSE	PCMT1	(P23506) Protein-L-isoaspartate(D-aspartate) O-methyltransferase (EC 2.1.1.77) (Protein-beta-aspartate methyltransferase) (PIMT) (Protein L-isoaspartyl/D-aspartyl methyltransferase) (L-isoaspartyl protein carboxyl methyltransferase)	3	31	226	24503	7.6	Cytoplasm	enzyme	NONE	7.45E-05
P23591	FCL_MOUSE	TSTA3	(P23591) GDP-L-fucose synthetase (EC 1.1.1.271) (Protein FX) (Red cell NADP(H)-binding protein) (GDP-4-keto-6-deoxy-D-mannose-3,5-epimerase-4-reductase) (Transplantation antigen P35B) (Tum-P35B antigen)	1	5	321	35878	6.7	Plasma Membrane	enzyme	NONE	3.5E-05
P23708	NFYA_MOUSE	NFYA	(P23708) Nuclear transcription factor Y subunit alpha (Nuclear transcription factor Y subunit A) (NF-YA) (CAAT box DNA-binding protein subunit A)	1	5.5	346	36779	8.9	Nucleus	transcription regulator	NONE	1.62E-05
P23780	BGAL_MOUSE	GLB1	(P23780) Beta-galactosidase precursor (EC 3.2.1.23) (Lactase) (Acid beta-galactosidase)	1	1.9	647	73121	7.5	Cytoplasm	enzyme	NONE	1.74E-05
P23927	CRYAB_MOUSE	CRYAB	(P23927) Alpha crystallin B chain (Alpha(B)-crystallin) (P23)	2	12	175	20069	7.3	Nucleus	other	NONE	0.00016
P23953	ESTN_MOUSE	ES1 (includes EG:13884)	(P23953) Liver carboxylesterase N precursor (EC 3.1.1.1) (PES-N) (Lung surfactant convertase)	1	5.1	554	61140	5.2	Cytoplasm	enzyme	1	5.07E-05
P24270	CATA_MOUSE	CAT	(P24270) Catalase (EC 1.11.1.6)	35	53	526	59634	7.9	Cytoplasm	enzyme	NONE	0.001633
P24369	PPIB_MOUSE	PPIB	(P24369) Peptidyl-prolyl cis-trans isomerase B precursor (EC 5.2.1.8) (PPIase) (Rotamase) (Cyclophilin B) (S-cyclophilin) (SCYLP) (CYP-S1)	19	58.2	208	22713	9.5	Cytoplasm	enzyme	1	0.001943
P24549	AL1A1_MOUSE	ALDH1A1	(P24549) Retinal dehydrogenase 1 (EC 1.2.1.36) (RalDH1) (RALDH 1) (Aldehyde dehydrogenase family 1 member A1) (Aldehyde dehydrogenase, cytosolic) (ALHDII) (ALDH-E1)	2	6.4	500	54337	7.8	Cytoplasm	enzyme	NONE	7.86E-05
P24668	MPRD_MOUSE	M6PR	(P24668) Cation-dependent mannose-6-phosphate receptor precursor (CD Man-6-P receptor) (CD-MPR) (46 kDa mannose 6-phosphate receptor) (MPR 46)	1	6.5	278	31172	5.4	Cytoplasm	transporter	2	6.06E-05
P24788	CD2L1_MOUSE	CDC2L1 (includes EG:12537)	(P24788) PITSLRE serine/threonine-protein kinase CDC2L1 (EC 2.7.11.22) (Galactosyltransferase-associated protein kinase p58/GTA) (Cell division cycle 2-like protein kinase 1)	1	1.9	784	91514	5.4	Nucleus	other	NONE	2.15E-05
P25444	RS2_MOUSE	RPS2 (includes EG:6187)	(P25444) 40S ribosomal protein S2 (S4) (LLRep3 protein)	6	24.6	293	31231	10.2	Cytoplasm	other	NONE	0.000441
P25918	CD19_MOUSE	CD19	(P25918) B-lymphocyte antigen CD19 precursor (Differentiation antigen CD19)	1	4	547	60163	5	Plasma Membrane	other	3	1.03E-05
P26039	TLN1_MOUSE	TLN1	(P26039) Talin-1	37	20.1	2541	269831	6.1	Plasma Membrane	other	NONE	0.000152
P26040	EZRI_MOUSE	VIL2	(P26040) Ezrin (p81) (Cytovillin) (Villin-2)	23	27.5	585	69276	6.1	Plasma Membrane	other	NONE	0.001996
P26041	MOES_MOUSE	MSN	(P26041) Moesin (Membrane-organizing extension spike protein)	25	33	576	67636	6.6	Plasma Membrane	other	NONE	0.001091
P26043	RADI_MOUSE	RDX	(P26043) Radixin (ESP10)	12	17.8	583	68601	6.1	Cytoplasm	other	NONE	0.00026
P26149	3BHS2_MOUSE	HSD3B2 (includes EG:15493)	(P26149) 3 beta-hydroxysteroid dehydrogenase/delta 5--4-isomerase type II (3Beta-HSD II) [Includes: 3-beta-hydroxy-delta(5)-steroid dehydrogenase (EC 1.1.1.145) (3-beta-hydroxy-5-ene steroid dehydrogenase) (Progesterone reductase); Steroid d	1	3.8	372	41864	6.5	Unknown	enzyme	NONE	4.53E-05
P26150	3BHS3_MOUSE	HSD3B3	(P26150) 3 beta-hydroxysteroid dehydrogenase/delta 5--4-isomerase type III (3Beta-HSD III) [Includes: 3-beta-hydroxy-delta(5)-steroid dehydrogenase (EC 1.1.1.145) (3-beta-hydroxy-5-ene steroid dehydrogenase) (Progesterone reductase); Steroid	1	4.6	372	41900	8	Cytoplasm	enzyme	NONE	3.02E-05
P26231	CTN1_MOUSE	CTNNA1	(P26231) Alpha-1 catenin (102 kDa cadherin-associated protein) (CAP102) (Alpha E-catenin)	8	10.5	906	100106	6.2	Plasma Membrane	other	NONE	8.67E-05
P26339	CMGA_MOUSE	CHGA	(P26339) Chromogranin A precursor (CgA) [Contains: Pancreastatin; Beta-granin; WE-14]	2	8	463	51789	4.7	Extracellular Space	other	NONE	2.42E-05
P26443	DHE3_MOUSE	GLUD1	(P26443) Glutamate dehydrogenase 1, mitochondrial precursor (EC 1.4.1.3) (GDH)	33	37.5	558	61337	8	Cytoplasm	enzyme	NONE	0.001117
P26450	P85A_MOUSE	PIK3R1	(P26450) Phosphatidylinositol 3-kinase regulatory subunit alpha (PI3-kinase p85-subunit alpha) (PtdIns-3-kinase p85-alpha) (PI3K)	2	3.9	724	83414	6.4	Cytoplasm	kinase	NONE	1.55E-05

P26516	PSD7_MOUSE	PSMD7	(P26516) 26S proteasome non-ATPase regulatory subunit 7 (26S proteasome regulatory subunit rpn8) (26S proteasome regulatory subunit S12) (Proteasome subunit p40) (Mov34 protein)	5	20.2	321	36540	6.8	Cytoplasm	other	NONE	0.000385
P26638	SYS_MOUSE	SARS	(P26638) Seryl-tRNA synthetase (EC 6.1.1.11) (Serine--tRNA ligase) (SerRS)	8	15.5	511	58258	6.3	Cytoplasm	enzyme	NONE	0.000286
P26645	MARCS_MOUSE	MARCKS	(P26645) Myristoylated alanine-rich C-kinase substrate (MARCKS)	4	27.6	308	29530	4.3	Plasma Membrane	other	NONE	0.0002
P26883	FKB1A_MOUSE	FKBP1A	(P26883) FK506-binding protein 1A (EC 5.2.1.8) (Peptidyl-prolyl cis-trans isomerase) (PPIase) (Rotamase) (12 kDa FKBP) (FKBP-12) (Immunophilin FKBP12)	3	25.2	107	11791	8.2	Cytoplasm	enzyme	NONE	0.00063
P27048	RSMB_MOUSE	SNRPB	(P27048) Small nuclear ribonucleoprotein-associated protein B (snRNP-B) (Sm protein B) (Sm-B) (SmB)	3	12.6	231	23656	10.9	Nucleus	other	NONE	0.00017
P27546	MAP4_MOUSE	MAP4	(P27546) Microtubule-associated protein 4 (MAP 4)	11	14.3	1125	117675	5	Cytoplasm	other	NONE	9.98E-05
P27601	GNA13_MOUSE	GNA13	(P27601) Guanine nucleotide-binding protein alpha-13 subunit (G alpha-13)	2	7.2	377	44055	8.2	Plasma Membrane	enzyme	NONE	4.47E-05
P27612	PLAP_MOUSE	PLAA	(P27612) Phospholipase A-2-activating protein (PLAP)	3	5	794	87235	6.1	Cytoplasm	other	NONE	3.53E-05
P27659	RL3_MOUSE	RPL3	(P27659) 60S ribosomal protein L3 (J1 protein)	13	25.4	402	45993	10.2	Cytoplasm	other	NONE	0.001313
P27773	PDIA3_MOUSE	PDIA3	(P27773) Protein disulfide-isomerase A3 precursor (EC 5.3.4.1) (Disulfide isomerase ER-60) (ERp60) (58 kDa microsomal protein) (p58) (ERp57)	37	55.2	504	56621	6.4	Cytoplasm	peptidase	NONE	0.002339
P27889	HNF1B_MOUSE	TCF2	(P27889) Hepatocyte nuclear factor 1-beta (HNF-1beta) (HNF-1B) (Variant hepatic nuclear factor 1) (VHNF1) (Homeoprotein LFB3)	2	5.9	558	61588	7.8	Nucleus	transcription regulator	NONE	3.02E-05
P28271	IREB1_MOUSE	ACO1	(P28271) Iron-responsive element-binding protein 1 (IRE-BP 1) (Iron regulatory protein 1) (IRP1) (Ferritin repressor protein) (Aconitate hydratase) (EC 4.2.1.3) (Citrate hydro-lyase) (Aconitase)	18	26.5	889	98179	7.5	Cytoplasm	enzyme	NONE	0.000227
P28352	APEX1_MOUSE	APEX1	(P28352) DNA-(apurinic or apyrimidinic site) lyase (EC 4.2.99.18) (AP endonuclease 1) (APEX nuclease) (APEN)	5	13.9	316	35359	7.9	Nucleus	enzyme	NONE	0.000178
P28474	ADHX_MOUSE	ADH5	(P28474) Alcohol dehydrogenase class 3 (EC 1.1.1.1) (Alcohol dehydrogenase class III) (Alcohol dehydrogenase 2) (S-(hydroxymethyl)glutathione dehydrogenase) (EC 1.1.1.284) (Glutathione-dependent formaldehyde dehydrogenase) (FDH) (FALDH) (Alcoh	8	34.9	373	39502	7.5	Cytoplasm	enzyme	NONE	0.000166
P28652	KCC2B_MOUSE	CAMK2B	(P28652) Calcium/calmodulin-dependent protein kinase type II beta chain (EC 2.7.11.17) (CaM-kinase II beta chain) (CaM kinase II subunit beta) (CaMK-II subunit beta)	1	3.5	542	60461	7.3	Cytoplasm	kinase	1	2.07E-05
P28653	PGS1_MOUSE	BGN	(P28653) Biglycan precursor (Bone/cartilage proteoglycan I) (PG-S1)	2	10.6	369	41639	7.3	Extracellular Space	other	1	4.56E-05
P28656	NP1L1_MOUSE	NAP1L1	(P28656) Nucleosome assembly protein 1-like 1 (NAP-1-related protein) (Brain protein DN38)	7	29.2	391	45345	4.5	Nucleus	other	NONE	0.00033
P28825	MEP1A_MOUSE	MEP1A	(P28825) Meprin A subunit alpha precursor (EC 3.4.24.18) (Endopeptidase-2) (MEP-1)	8	15.8	747	84197	6.2	Plasma Membrane	peptidase	2	0.000308
P28843	DPP4_MOUSE	DPP4	(P28843) Dipeptidyl peptidase 4 (EC 3.4.14.5) (Dipeptidyl peptidase IV) (DPP IV) (T-cell activation antigen CD26) (Thymocyte-activating molecule) (THAM) [Contains: Dipeptidyl peptidase 4 membrane form (Dipeptidyl peptidase IV membrane form); D	5	7.8	760	87437	6.4	Plasma Membrane	peptidase	1	6.65E-05
P29121	PCSK4_MOUSE	PCSK4	(P29121) Proprotein convertase subtilisin/kexin type 4 precursor (EC 3.4.21.-) (PC4) (Neuroendocrine convertase 3) (NEC 3) (Prohormone convertase 3) (KEX2-like endoprotease 3)	1	3.5	655	73214	8.2	Extracellular Space	peptidase	NONE	0.00048
P29341	PABP1_MOUSE	PABPC1	(P29341) Polyadenylate-binding protein 1 (Poly(A)-binding protein 1) (PABP 1)	8	13.5	636	70643	9.5	Cytoplasm	translation regulator	NONE	0.000132
P29391	FRIL1_MOUSE	FTL1	(P29391) Ferritin light chain 1 (Ferritin L subunit 1)	8	37.9	182	20671	6	Cytoplasm	other	NONE	0.000925
P29595	NEDD8_MOUSE	NEDD8	(P29595) NEDD8 precursor (Ubiquitin-like protein Nedd8) (Neddylin) (Neural precursor cell expressed developmentally down-regulated protein 8)	3	23.5	81	8972	7.2	Nucleus	enzyme	NONE	0.000485

P29621	SPA3C_MOUSE	SERPINA3C (includes EG:16625)	(P29621) Serine protease inhibitor A3C precursor (Kallikrein-binding protein) (KBP) (Serpin A3C)	2	3.6	417	46766	7.9	Cytoplasm	other	1	5.38E-05
P29699	FETUA_MOUSE	AHSG	(P29699) Alpha-2-HS-glycoprotein precursor (Fetuin-A) (Countertrypin)	1	6.1	345	37326	6.5	Extracellular Space	other	NONE	3.25E-05
P29758	OAT_MOUSE	OAT	(P29758) Ornithine aminotransferase, mitochondrial precursor (EC 2.6.1.13) (Ornithine--oxo-acid aminotransferase)	13	45.3	439	48355	6.6	Cytoplasm	enzyme	NONE	0.000281
P30115	GSTA3_MOUSE	GSTA3	(P30115) Glutathione S-transferase Yc (EC 2.5.1.18) (GST class-alpha) (Ya3)	1	8.6	220	25229	8.7	Cytoplasm	enzyme	NONE	5.1E-05
P30275	KCRU_MOUSE	CKMT1B	(P30275) Creatine kinase, ubiquitous mitochondrial precursor (EC 2.7.3.2) (U-MtCK) (Mia-CK) (Acidic-type mitochondrial creatine kinase)	7	24.2	418	47004	8.2	Cytoplasm	kinase	NONE	0.000255
P30412	PPIC_MOUSE	PPIC	(P30412) Peptidyl-prolyl cis-trans isomerase C (EC 5.2.1.8) (PPIase) (Rotamase) (Cyclophilin C)	1	7.1	212	22794	7.5	Cytoplasm	enzyme	NONE	2.65E-05
P30416	FKBP4_MOUSE	FKBP4	(P30416) FK506-binding protein 4 (EC 5.2.1.8) (Peptidyl-prolyl cis-trans isomerase) (PPIase) (Rotamase) (p59 protein) (HSP-binding immunophilin) (HBI) (FKBP52 protein) (52 kDa FK506-binding protein) (FKBP59)	13	35	457	51441	5.7	Nucleus	enzyme	NONE	0.000295
P30548	NK1R_MOUSE	TACR1	(P30548) Substance-P receptor (SPR) (NK-1 receptor) (NK-1R) (Tachykinin receptor 1)	2	3.9	407	46304	7.6	Plasma Membrane	G-protein coupled receptor	7	0.000386
P30681	HMGB2_MOUSE	HMGB2	(P30681) High mobility group protein B2 (High mobility group protein 2) (HMG-2)	4	16.7	209	24031	7.3	Nucleus	transcription regulator	NONE	0.000188
P30999	CTND1_MOUSE	CTNND1	(P30999) Catenin delta-1 (p120 catenin) (p120(ctn)) (Cadherin-associated Src substrate) (CAS) (p120(cas))	8	11.7	911	101731	6.9	Nucleus	other	NONE	0.000123
P31001	DESM_MOUSE	DES	(P31001) Desmin	5	16.2	468	53367	5.3	Cytoplasm	other	NONE	0.00012
P31230	MCA1_MOUSE	SCYE1	(P31230) Multisynthetase complex auxiliary component p43 [Contains: Endothelial monocyte-activating polypeptide 2 (EMAP-II) (Small inducible cytokine subfamily E member 1)]	4	15.2	310	33997	8.4	Extracellular Space	cytokine	NONE	0.000181
P31253	UBE1X_MOUSE	UBE1	(P31253) Ubiquitin-activating enzyme E1 X (Fragment)	3	8.7	450	50986	6.8	Cytoplasm	enzyme	2	7.48E-05
P31428	DPEP1_MOUSE	DPEP1	(P31428) Dipeptidase 1 precursor (EC 3.4.13.19) (Microsomal dipeptidase) (Renal dipeptidase) (Membrane-bound dipeptidase 1) (MBD-1)	6	17.3	410	45682	6.4	Cytoplasm	peptidase	1	0.000301
P31786	ACBP_MOUSE	DBI	(P31786) Acyl-CoA-binding protein (ACBP) (Diazepam-binding inhibitor) (DBI) (Endozepine) (EP)	11	44.2	86	9869	8.8	Cytoplasm	other	NONE	0.005548
P32020	NLTP_MOUSE	SCP2	(P32020) Nonspecific lipid-transfer protein (EC 2.3.1.176) (Propanoyl-CoA C-acyltransferase) (NSL-TP) (Sterol carrier protein 2) (SCP-2) (Sterol carrier protein X) (SCP-X) (SCP-chi) (SCPX)	21	18.3	547	59126	7.4	Cytoplasm	transporter	NONE	0.000883
P32067	LA_MOUSE	SSB	(P32067) Lupus La protein homolog (La ribonucleoprotein) (La autoantigen homolog)	4	12.8	415	47756	9.8	Nucleus	enzyme	NONE	9.47E-05
P32233	DRG1_MOUSE	DRG1	(P32233) Developmentally-regulated GTP-binding protein 1 (DRG 1) (Protein NEDD3) (Neural precursor cell expressed developmentally down-regulated protein 3)	2	5.7	367	40512	8.9	Cytoplasm	other	NONE	3.06E-05
P32261	ANT3_MOUSE	SERPINC1	(P32261) Antithrombin-III precursor (ATIII)	1	3	465	52004	6.5	Extracellular Space	other	NONE	1.21E-05
P32848	PRVA_MOUSE	PVALB	(P32848) Parvalbumin alpha	1	12.8	109	11799	5.2	Cytoplasm	other	NONE	5.15E-05
P33175	KIF5A_MOUSE	KIF5A	(P33175) Kinesin heavy chain isoform 5A (Neuronal kinesin heavy chain) (NKHC) (Kinesin heavy chain neuron-specific 1)	3	2.7	1027	117019	5.9	Cytoplasm	transporter	NONE	2.73E-05
P33215	NEDD1_MOUSE	NEDD1	(P33215) Protein NEDD1 (Neural precursor cell expressed developmentally down-regulated protein 1)	2	5	660	71292	7.8	Cytoplasm	other	NONE	0.00017
P33622	APOC3_MOUSE	APOC3	(P33622) Apolipoprotein C-III precursor (Apo-CIII) (ApoC-III)	1	19.2	99	10982	4.7	Extracellular Space	transporter	1	0.00017
P34022	RANG_MOUSE	RANBP1	(P34022) Ran-specific GTPase-activating protein (Ran-binding protein 1) (RANBP1) (HpaII tiny fragments locus 9a protein)	4	17.2	203	23596	5.2	Nucleus	other	NONE	0.000138
P34884	MIF_MOUSE	MIF	(P34884) Macrophage migration inhibitory factor (MIF) (Phenylpyruvate tautomerase) (EC 5.3.2.1) (Glycosylation-inhibiting factor) (GIF) (Delayed early response protein 6) (DER6)	2	9.6	114	12373	7.3	Extracellular Space	cytokine	NONE	0.003594

P34914	HYES_MOUSE	EPHX2	(P34914) Epoxide hydrolase 2 (EC 3.3.2.3) (Soluble epoxide hydrolase) (SEH) (Epoxide hydratase) (Cytosolic epoxide hydrolase) (CEH)	9	17.7	554	62515	6.2	Cytoplasm	enzyme	NONE	0.000344
P35278	RAB5C_MOUSE	RAB5C	(P35278) Ras-related protein Rab-5C	2	6.5	216	23412	8.4	Cytoplasm	enzyme	NONE	0.00013
P35279	RAB6A_MOUSE	RAB6A	(P35279) Ras-related protein Rab-6A (Rab-6)	2	6.8	207	23459	5.5	Cytoplasm	enzyme	NONE	0.00019
P35282	RAB21_MOUSE	RAB21	(P35282) Ras-related protein Rab-21 (Rab-12)	3	18.6	221	23975	7.9	Cytoplasm	enzyme	NONE	0.000152
P35290	RAB24_MOUSE	RAB24	(P35290) Ras-related protein Rab-24 (Rab-16)	1	7.4	203	23144	6.2	Cytoplasm	enzyme	NONE	5.53E-05
P35293	RAB18_MOUSE	RAB18	(P35293) Ras-related protein Rab-18	3	23.8	206	23035	5.4	Cytoplasm	enzyme	NONE	0.0003
P35486	ODPA_MOUSE	PDHA1 (includes EG:5160)	(P35486) Pyruvate dehydrogenase E1 component alpha subunit, somatic form, mitochondrial precursor (EC 1.2.4.1) (PDHE1-A type I)	15	36.7	390	43232	8.2	Cytoplasm	enzyme	NONE	0.001108
P35505	FAAA_MOUSE	FAH	(P35505) Fumarylacetoacetase (EC 3.7.1.2) (Fumarylacetoacetate hydrolase) (Beta-diketonase) (FAA)	9	29.1	419	46104	7.4	Cytoplasm	enzyme	NONE	0.001125
P35550	FBRL_MOUSE	FBL	(P35550) Fibrillarin (Nucleolar protein 1)	5	14.4	327	34307	10.2	Nucleus	other	NONE	0.000103
P35564	CALX_MOUSE	CANX	(P35564) Calnexin precursor	23	29.6	591	67278	4.6	Cytoplasm	other	2	0.001159
P35585	AP1M1_MOUSE	AP1M1	(P35585) AP-1 complex subunit mu-1 (Adaptor-related protein complex 1 mu-1 subunit) (Mu-adaptin 1) (Adaptor protein complex AP-1 mu-1 subunit) (Golgi adaptor HA1/AP1 adaptin mu-1 subunit) (Clathrin assembly protein assembly protein complex 1	3	8.1	422	48412	7.3	Cytoplasm	transporter	NONE	3.99E-05
P35700	PRDX1_MOUSE	PRDX1	(P35700) Peroxiredoxin-1 (EC 1.11.1.15) (Thioredoxin peroxidase 2) (Thioredoxin-dependent peroxide reductase 2) (Osteoblast-specific factor 3) (OSF-3) (Macrophage 23 kDa stress protein)	22	60.3	199	22176	8.1	Cytoplasm	enzyme	NONE	0.009421
P35710	SOX5_MOUSE	SOX5	(P35710) Transcription factor SOX-5	2	3.8	763	84090	6.6	Nucleus	transcription regulator	NONE	1.47E-05
P35802	GPM6A_MOUSE	GPM6A	(P35802) Neuronal membrane glycoprotein M6-a (M6a)	1	5	278	31149	5.3	Plasma Membrane	ion channel	4	2.02E-05
P35822	PTPRK_MOUSE	PTPRK	(P35822) Receptor-type tyrosine-protein phosphatase kappa precursor (EC 3.1.3.48) (Protein-tyrosine phosphatase kappa) (R-PTP-kappa)	4	3.4	1457	164185	5.9	Plasma Membrane	phosphatase	2	2.31E-05
P35846	FOLR1_MOUSE	FOLR1	(P35846) Folate receptor alpha precursor (FR-alpha) (Folate receptor 1) (Folate-binding protein 1)	3	23.5	255	29415	6.6	Plasma Membrane	transporter	1	0.000154
P35922	FMR1_MOUSE	FMR1	(P35922) Fragile X mental retardation protein 1 homolog (Protein FMR-1) (FMRP) (mFmr1p)	1	2.3	614	68989	7.6	Nucleus	other	NONE	3.66E-05
P35979	RL12_MOUSE	RPL12	(P35979) 60S ribosomal protein L12	6	43	165	17805	9.4	Cytoplasm	other	NONE	0.00313
P35980	RL18_MOUSE	RPL18	(P35980) 60S ribosomal protein L18	4	19.8	187	21513	11.8	Cytoplasm	other	NONE	0.00039
P36552	HEM6_MOUSE	CPOX	(P36552) Coproporphyrinogen III oxidase, mitochondrial precursor (EC 1.3.3.3) (Coproporphyrinogenase) (Coprogen oxidase) (COX)	4	10.8	443	49715	8.5	Cytoplasm	enzyme	NONE	7.6E-05
P36993	PP2CB_MOUSE	PPM1B	(P36993) Protein phosphatase 2C isoform beta (EC 3.1.3.16) (PP2C-beta) (IA) (Protein phosphatase 1B)	4	8.7	390	42795	5.2	Unknown	phosphatase	NONE	0.000101
P37040	NCPR_MOUSE	POR	(P37040) NADPH--cytochrome P450 reductase (EC 1.6.2.4) (CPR) (P450R)	9	12.1	677	76913	5.5	Cytoplasm	enzyme	1	0.000133
P37804	TAGL_MOUSE	TAGLN	(P37804) Transgelin (Smooth muscle protein 22-alpha) (SM22-alpha) (Actin-associated protein p27)	5	28.5	200	22445	8.8	Cytoplasm	other	NONE	0.000281
P38060	HMGCL_MOUSE	HMGCL	(P38060) Hydroxymethylglutaryl-CoA lyase, mitochondrial precursor (EC 4.1.3.4) (HMG-CoA lyase) (HL) (3-hydroxy-3-methylglutarate-CoA lyase)	8	20	325	34161	8.3	Cytoplasm	enzyme	NONE	0.000259
P38647	GRP75_MOUSE	HSPA9	(P38647) Stress-70 protein, mitochondrial precursor (75 kDa glucose-regulated protein) (GRP 75) (Peptide-binding protein 74) (PBP74) (P66 MOT) (Mortalin)	45	44.3	679	73528	6.2	Cytoplasm	other	NONE	0.002034
P39061	COIA1_MOUSE	COL18A1	(P39061) Collagen alpha-1(XVIII) chain precursor [Contains: Endostatin]	4	4.2	1774	182230	5.6	Extracellular Space	other	3	3.48E-05
P39447	ZO1_MOUSE	TJP1	(P39447) Tight junction protein ZO-1 (Zonula occludens 1 protein) (Zona occludens 1 protein) (Tight junction protein 1)	8	7.6	1745	194710	6.7	Plasma Membrane	other	NONE	4.83E-05
P39654	LX12L_MOUSE	ALOX15	(P39654) Arachidonate 12-lipoxygenase, leukocyte-type (EC 1.13.11.31) (12-LOX)	2	5.3	662	75286	6	Cytoplasm	enzyme	NONE	3.39E-05
P40124	CAP1_MOUSE	CAP1	(P40124) Adenylyl cyclase-associated protein 1 (CAP 1)	7	19.2	473	51444	7.5	Plasma Membrane	other	NONE	0.000214
P40142	TKT_MOUSE	TKT	(P40142) Transketolase (EC 2.2.1.1) (TK) (P68)	17	28.6	623	67631	7.5	Cytoplasm	enzyme	NONE	0.000432

P40630	TFAM_MOUSE	TFAM	(P40630) Transcription factor A, mitochondrial precursor (mtTFA) (Testis-specific high mobility group protein) (TS-HMG)	6	10.7	243	27988	9.7	Cytoplasm	transcription regulator	NONE	0.000277
P40936	INMT_MOUSE	INMT	(P40936) Indolethylamine N-methyltransferase (EC 2.1.1.49) (Aromatic alkylamine N-methyltransferase) (Indolamine N-methyltransferase) (Arylamine N-methyltransferase) (Amine N-methyltransferase)	7	36.4	264	29460	6.4	Cytoplasm	enzyme	NONE	0.000425
P41105	RL28_MOUSE	RPL28	(P41105) 60S ribosomal protein L28	5	14	136	15602	12	Cytoplasm	other	NONE	0.000371
P41216	ACSL1_MOUSE	ACSL1	(P41216) Long-chain-fatty-acid--CoA ligase 1 (EC 6.2.1.3) (Long-chain acyl-CoA synthetase 1) (LACS 1)	2	4.7	699	77923	7.1	Cytoplasm	enzyme	NONE	1.61E-05
P42125	D3D2_MOUSE	DCI	(P42125) 3,2-trans-enoyl-CoA isomerase, mitochondrial precursor (EC 5.3.3.8) (Dodecenoyl-CoA isomerase) (Delta(3),delta(2)-enoyl-CoA isomerase) (D3,D2-enoyl-CoA isomerase)	5	17	289	32078	8.7	Cytoplasm	enzyme	NONE	0.000524
P42208	SEPT2_MOUSE	SPET2	(P42208) Septin-2 (Protein NEDD5) (Neural precursor cell expressed developmentally down-regulated protein 5)	8	21.3	361	41526	6.5	Cytoplasm	enzyme	NONE	0.000342
P42225	STAT1_MOUSE	STAT1	(P42225) Signal transducer and activator of transcription 1	2	3.7	749	87197	5.6	Nucleus	transcription regulator	NONE	3E-05
P42227	STAT3_MOUSE	STAT3	(P42227) Signal transducer and activator of transcription 3 (Acute-phase response factor)	2	5.6	770	88054	6.3	Nucleus	transcription regulator	NONE	2.92E-05
P42567	EP15_MOUSE	EPS15	(P42567) Epidermal growth factor receptor substrate 15 (Protein Eps15) (AF-1p protein)	2	6.2	897	98471	4.6	Plasma Membrane	other	NONE	1.25E-05
P42669	PURA_MOUSE	PURA	(P42669) Transcriptional activator protein Pur-alpha (Purine-rich single-stranded DNA-binding protein alpha)	12	35.2	321	34884	6.4	Nucleus	transcription regulator	NONE	0.000699
P42932	TCPQ_MOUSE	CCT8	(P42932) T-complex protein 1 subunit theta (TCP-1-theta) (CCT-theta)	7	14.6	547	59424	5.6	Cytoplasm	enzyme	NONE	0.000113
P43024	CX6A1_MOUSE	COX6A1	(P43024) Cytochrome c oxidase polypeptide VIa-liver, mitochondrial precursor (EC 1.9.3.1)	2	18.9	111	12352	10	Cytoplasm	enzyme	1	0.003793
P43274	H14_MOUSE	HIST1H1E	(P43274) Histone H1.4 (H1 VAR.2) (H1e)	4	13.8	218	21846	11.1	Nucleus	other	NONE	0.000257
P43275	H11_MOUSE	HIST1H1A	(P43275) Histone H1.1 (H1 VAR.3) (H1a)	2	14.2	212	21654	10.9	Nucleus	other	NONE	7.94E-05
P43276	H15_MOUSE	HIST1H1B	(P43276) Histone H1.5 (H1 VAR.5) (H1b)	3	13.1	222	22445	10.9	Nucleus	other	NONE	0.000278
P43277	H13_MOUSE	HIST1H1D	(P43277) Histone H1.3 (H1 VAR.4) (H1d)	1	7.3	220	21968	11	Nucleus	other	NONE	0.000102
P43406	ITAV_MOUSE	ITGAV	(P43406) Integrin alpha-V precursor (Vitronectin receptor subunit alpha) (CD51 antigen) [Contains: Integrin alpha-V heavy chain; Integrin alpha-V light chain]	3	5.9	1044	115278	5.7	Plasma Membrane	other	2	1.61E-05
P45376	ALDR_MOUSE	AKR1B1	(P45376) Aldose reductase (EC 1.1.1.21) (AR) (Aldehyde reductase)	6	17.5	315	35601	7.2	Cytoplasm	enzyme	NONE	0.000606
P45481	CBP_MOUSE	CREBBP	(P45481) CREB-binding protein (EC 2.3.1.48)	2	1.8	2441	265471	8.4	Nucleus	transcription regulator	NONE	4.6E-06
P45591	COF2_MOUSE	CFL2	(P45591) Cofilin-2 (Cofilin, muscle isoform)	3	27.7	166	18710	7.9	Nucleus	other	NONE	0.000304
P45878	FKBP2_MOUSE	FKBP2	(P45878) FK506-binding protein 2 precursor (EC 5.2.1.8) (Peptidyl-prolyl cis-trans isomerase) (PPIase) (Rotamase) (13 kDa FKBP) (FKBP-13)	4	29.3	140	15344	8.9	Cytoplasm	enzyme	1	0.000882
P45952	ACADM_MOUSE	ACADM	(P45952) Medium-chain specific acyl-CoA dehydrogenase, mitochondrial precursor (EC 1.3.99.3) (MCAD)	27	47.3	421	46481	8.4	Cytoplasm	enzyme	NONE	0.002053
P46412	GPX3_MOUSE	GPX3	(P46412) Glutathione peroxidase 3 precursor (EC 1.11.1.9) (GSHPx-3) (GPx-3) (Plasma glutathione peroxidase) (GSHPx-P)	12	45.6	226	25377	8.1	Extracellular Space	enzyme	NONE	0.00077
P46414	CDN1B_MOUSE	CDKN1B	(P46414) Cyclin-dependent kinase inhibitor 1B (Cyclin-dependent kinase inhibitor p27) (p27Kip1)	3	18.3	197	22210	7	Nucleus	other	NONE	0.000256
P46460	NSF_MOUSE	NSF	(P46460) Vesicle-fusing ATPase (EC 3.6.4.6) (Vesicular-fusion protein NSF) (N-ethylmaleimide sensitive fusion protein) (NEM-sensitive fusion protein) (SKD2 protein)	2	3.2	744	82565	6.9	Cytoplasm	transporter	NONE	1.51E-05
P46467	VPS4B_MOUSE	VPS4B	(P46467) Vacuolar sorting protein 4b (SKD1 protein)	2	7.7	444	49419	7.1	Cytoplasm	transporter	NONE	6.32E-05
P46471	PRS7_MOUSE	PSMC2	(P46471) 26S protease regulatory subunit 7 (Protein MSS1)	11	29.2	432	48517	5.9	Nucleus	peptidase	NONE	0.000247
P46638	RB11B_MOUSE	RAB11B	(P46638) Ras-related protein Rab-11B	2	15.7	217	24358	5.9	Cytoplasm	enzyme	NONE	0.000155
P46656	ADX_MOUSE	FDX1	(P46656) Adrenodoxin, mitochondrial precursor (Adrenal ferredoxin) (Ferredoxin-1)	4	20.2	188	20123	5.6	Cytoplasm	transporter	NONE	0.000328

P46660	AINX_MOUSE	INA	(P46660) Alpha-internexin (Alpha-Inx) (66 kDa neurofilament protein) (Neurofilament-66) (NF-66)	1	2.4	504	55742	5.3	Cytoplasm	other	NONE	2.23E-05
P46664	PURA2_MOUSE	ADSS	(P46664) Adenylosuccinate synthetase isozyme 2 (EC 6.3.4.4) (Adenylosuccinate synthetase, non-muscle isozyme) (Adenylosuccinate synthetase, acidic isozyme) (IMP--aspartate ligase 2) (AdSS 2) (AMPSase 2)	3	10.1	456	50021	6.4	Cytoplasm	enzyme	NONE	7.39E-05
P46737	BRCC3_MOUSE	BRCC3	(P46737) BRCA1/BRCA2-containing complex subunit 3	2	11.3	291	33340	5.8	Unknown	other	NONE	5.79E-05
P46935	NEDD4_MOUSE	NEDD4	(P46935) E3 ubiquitin-protein ligase NEDD4 (EC 6.3.2.-) (Neural precursor cell expressed developmentally down-regulated protein 4)	3	5.9	887	102706	5.3	Cytoplasm	enzyme	NONE	5.06E-05
P46938	YAP1_MOUSE	YAP1	(P46938) 65 kDa Yes-associated protein (YAP65)	6	15.3	472	50703	5	Nucleus	transcription regulator	NONE	0.000119
P47199	QOR_MOUSE	CRYZ	(P47199) Quinone oxidoreductase (EC 1.6.5.5) (NADPH:quinone reductase) (Zeta-crystallin)	17	48.9	331	35269	8.1	Cytoplasm	enzyme	NONE	0.002272
P47738	ALDH2_MOUSE	ALDH2	(P47738) Aldehyde dehydrogenase, mitochondrial precursor (EC 1.2.1.3) (ALDH class 2) (AHD-M1) (ALDHI) (ALDH-E2)	14	20.6	519	56538	7.6	Cytoplasm	enzyme	NONE	0.000476
P47740	AL3A2_MOUSE	ALDH3A2	(P47740) Fatty aldehyde dehydrogenase (EC 1.2.1.3) (Aldehyde dehydrogenase, microsomal) (Aldehyde dehydrogenase family 3 member A2) (Aldehyde dehydrogenase 10)	5	11.6	484	53943	8.4	Cytoplasm	enzyme	NONE	0.000116
P47753	CAZA1_MOUSE	CAPZA1 (includes EG:829)	(P47753) F-actin capping protein alpha-1 subunit (CapZ alpha-1)	6	27	285	32809	5.6	Cytoplasm	other	NONE	0.000335
P47754	CAZA2_MOUSE	CAPZA2	(P47754) F-actin capping protein alpha-2 subunit (CapZ alpha-2)	7	33.7	285	32836	5.8	Cytoplasm	other	NONE	0.000276
P47757	CAPZB_MOUSE	CAPZB	(P47757) F-actin capping protein subunit beta (CapZ beta)	2	7.2	276	31214	5.7	Cytoplasm	other	NONE	0.000122
P47758	SRPRB_MOUSE	SRPRB	(P47758) Signal recognition particle receptor subunit beta (SR-beta)	1	7.1	269	29579	9.3	Cytoplasm	other	1	8.35E-05
P47791	GSHR_MOUSE	GSR	(P47791) Glutathione reductase, mitochondrial precursor (EC 1.8.1.7) (GR) (GRase)	2	7.4	500	53663	8	Cytoplasm	enzyme	NONE	5.61E-05
P47802	MTX1_MOUSE	MTX1	(P47802) Metaxin-1	2	9.5	317	35624	6.2	Cytoplasm	transporter	NONE	0.000106
P47809	MP2K4_MOUSE	MAP2K4	(P47809) Dual specificity mitogen-activated protein kinase kinase 4 (EC 2.7.12.2) (MAP kinase kinase 4) (MAPKK 4) (MAPK/ERK kinase 4) (JNK-activating kinase 1) (C-JUN N-terminal kinase kinase 1) (JNK kinase 1) (JNKK 1) (SAPK/ERK kinase 1) (SE	1	4.3	397	44114	8.1	Cytoplasm	kinase	NONE	2.83E-05
P47911	RL6_MOUSE	RPL6	(P47911) 60S ribosomal protein L6 (TAX-responsive enhancer element-binding protein 107) (TAXREB107)	11	30.8	295	33378	10.7	Cytoplasm	other	NONE	0.000552
P47934	CACP_MOUSE	CRAT	(P47934) Carnitine O-acetyltransferase (EC 2.3.1.7) (Carnitine acetylase) (CAT) (Carnitine acetyltransferase) (CrAT)	1	2.4	626	70925	8.3	Cytoplasm	enzyme	NONE	8.97E-06
P47941	CRKL_MOUSE	CRKL	(P47941) Crk-like protein	7	31.7	303	33830	6.7	Cytoplasm	kinase	NONE	0.000408
P47955	RLA1_MOUSE	RPLP1	(P47955) 60S acidic ribosomal protein P1	1	14	114	11475	4.3	Cytoplasm	other	NONE	0.000739
P47962	RL5_MOUSE	RPL5	(P47962) 60S ribosomal protein L5	13	28	296	34269	9.8	Cytoplasm	other	NONE	0.000626
P47963	RL13_MOUSE	RPL13	(P47963) 60S ribosomal protein L13 (A52)	12	26.7	210	24174	11.5	Cytoplasm	other	NONE	0.000989
P47964	RL36_MOUSE	RPL36	(P47964) 60S ribosomal protein L36	4	11.5	104	12084	11.4	Cytoplasm	other	NONE	0.000918
P48036	ANXA5_MOUSE	ANXA5	(P48036) Annexin A5 (Annexin V) (Lipocortin V) (Endonexin II) (Calphobindin I) (CBP-I) (Placental anticoagulant protein I) (PAP-I) (PP4) (Thromboplastin inhibitor) (Vascular anticoagulant-alpha) (VAC-alpha) (Anchorin CII)	15	43.3	319	35752	5	Plasma Membrane	other	NONE	0.000933
P48193	41_MOUSE	EPB41	(P48193) Protein 4.1 (Band 4.1) (P4.1) (4.1R)	8	11.4	858	95990	5.6	Plasma Membrane	other	NONE	9.16E-05
P48377	RFX1_MOUSE	RFX1	(P48377) DNA-binding protein RFX1	1	3.4	963	103693	6.3	Nucleus	transcription regulator	NONE	5.83E-06
P48678	LMNA_MOUSE	LMNA	(P48678) Lamin-A/C	25	32.9	665	74238	7	Nucleus	other	NONE	0.000582
P48758	DHCA_MOUSE	CBR1	(P48758) Carbonyl reductase [NADPH] 1 (EC 1.1.1.184) (NADPH-dependent carbonyl reductase 1)	9	29	276	30597	7.8	Cytoplasm	enzyme	NONE	0.000529
P48771	CX7A2_MOUSE	COX7A2	(P48771) Cytochrome c oxidase polypeptide VIIa-liver/heart, mitochondrial precursor (EC 1.9.3.1) (Cytochrome c oxidase subunit VIIa-L)	2	15.7	83	9291	10.3	Cytoplasm	enzyme	1	0.000473

P48962	ADT1_MOUSE	SLC25A4	(P48962) ADP/ATP translocase 1 (Adenine nucleotide translocator 1) (ANT 1) (ADP,ATP carrier protein 1) (Solute carrier family 25 member 4) (ADP,ATP carrier protein, heart/skeletal muscle isoform T1) (mANC1)	7	13.8	297	32773	9.7	Cytoplasm	transporter	NONE	0.000529
P49312	ROA1_MOUSE	HNRPA1	(P49312) Heterogeneous nuclear ribonucleoprotein A1 (Helix-destabilizing protein) (Single-strand-binding protein) (hnRNP core protein A1) (HDP-1) (Topoisomerase-inhibitor suppressed)	6	18.5	319	34065	9.2	Nucleus	other	NONE	0.000862
P49442	INPP_MOUSE	INPP1	(P49442) Inositol polyphosphate 1-phosphatase (EC 3.1.3.57) (IPPase) (IPP)	1	4	396	43346	5	Cytoplasm	phosphatase	NONE	1.42E-05
P49443	PP2CA_MOUSE	PPM1A	(P49443) Protein phosphatase 2C isoform alpha (EC 3.1.3.16) (PP2C-alpha) (IA) (Protein phosphatase 1A)	5	18.1	382	42433	5.4	Cytoplasm	phosphatase	NONE	0.000103
P49586	PCY1A_MOUSE	PCYT1A	(P49586) Choline-phosphate cytidylyltransferase A (EC 2.7.7.15) (Phosphorylcholine transferase A) (CTP:phosphocholine cytidylyltransferase A) (CT A) (CCT A) (CCT-alpha)	4	18.8	367	41667	7	Cytoplasm	enzyme	NONE	0.000107
P49615	CDK5_MOUSE	CDK5	(P49615) Cell division protein kinase 5 (EC 2.7.11.22) (Tau protein kinase II catalytic subunit) (TPKII catalytic subunit) (Serine/threonine-protein kinase PSSALRE) (CRK6)	1	7.5	292	33288	7.7	Nucleus	kinase	NONE	3.84E-05
P49698	HNF4A_MOUSE	HNF4A	(P49698) Hepatocyte nuclear factor 4-alpha (HNF-4-alpha) (Transcription factor HNF-4) (Transcription factor 14)	1	4.9	465	51755	7.1	Nucleus	transcription regulator	NONE	2.41E-05
P49710	HCLS1_MOUSE	HCLS1	(P49710) Hematopoietic lineage cell-specific protein (Hematopoietic cell-specific LYN substrate 1) (LckBP1)	1	4.1	486	54212	4.8	Nucleus	transcription regulator	NONE	2.31E-05
P49722	PSA2_MOUSE	PSMA2	(P49722) Proteasome subunit alpha type 2 (EC 3.4.25.1) (Proteasome component C3) (Macropain subunit C3) (Multicatalytic endopeptidase complex subunit C3)	6	23.6	233	25794	8.3	Cytoplasm	peptidase	NONE	0.000506
P49935	CATH_MOUSE	CTSH	(P49935) Cathepsin H precursor (EC 3.4.22.16) (Cathepsin B3) (Cathepsin BA) [Contains: Cathepsin H mini chain; Cathepsin H heavy chain; Cathepsin H light chain]	4	20.1	333	37184	8.4	Cytoplasm	peptidase	NONE	0.000219
P49962	SRP09_MOUSE	SRP9	(P49962) Signal recognition particle 9 kDa protein (SRP9)	1	12.9	85	10063	8	Cytoplasm	other	NONE	0.000132
P50096	IMDH1_MOUSE	IMPDH1	(P50096) Inosine-5'-monophosphate dehydrogenase 1 (EC 1.1.1.205) (IMP dehydrogenase 1) (IMPDH-I) (IMPD 1)	1	3.9	514	55294	6.8	Cytoplasm	enzyme	NONE	1.09E-05
P50136	ODBA_MOUSE	BCKDHA	(P50136) 2-oxoisovalerate dehydrogenase subunit alpha, mitochondrial precursor (EC 1.2.4.4) (Branched-chain alpha-keto acid dehydrogenase E1 component alpha chain) (BCKDH E1-alpha)	8	26.7	442	50371	8.1	Cytoplasm	enzyme	NONE	0.000229
P50171	DHB8_MOUSE	HSD17B8	(P50171) Estradiol 17-beta-dehydrogenase 8 (EC 1.1.1.62) (17-beta-HSD 8) (17-beta-hydroxysteroid dehydrogenase 8) (Protein Ke6) (Ke-6)	2	10.8	260	26645	6.5	Cytoplasm	enzyme	NONE	0.00013
P50172	DHI1_MOUSE	HSD11B1	(P50172) Corticosteroid 11-beta-dehydrogenase isozyme 1 (EC 1.1.1.146) (11-DH) (11-beta-hydroxysteroid dehydrogenase 1) (11-beta-HSD1) (11beta-HSD1A)	3	13.7	291	32233	8.5	Cytoplasm	enzyme	1	5.79E-05
P50247	SAHH_MOUSE	AHCY	(P50247) Adenosylhomocysteinase (EC 3.3.1.1) (S-adenosyl-L-homocysteine hydrolase) (AdoHcyase) (Liver copper-binding protein) (CUBP)	10	20.2	431	47557	6.5	Cytoplasm	enzyme	NONE	0.000352
P50295	ARY2_MOUSE	NAT1	(P50295) Arylamine N-acetyltransferase 2 (EC 2.3.1.5) (Arylamide acetylase 2) (N-acetyltransferase type 2) (NAT-2)	1	8.3	290	33701	5.9	Cytoplasm	enzyme	NONE	3.87E-05
P50396	GDIA_MOUSE	GDI1	(P50396) Rab GDP dissociation inhibitor alpha (Rab GDI alpha) (Guanosine diphosphate dissociation inhibitor 1) (GDI-1)	5	11.9	447	50522	5.1	Cytoplasm	other	NONE	0.000289
P50431	GLYC_MOUSE	SHMT1	(P50431) Serine hydroxymethyltransferase, cytosolic (EC 2.1.2.1) (Serine methylase) (Glycine hydroxymethyltransferase) (SHMT)	3	6.7	478	52585	6.9	Cytoplasm	enzyme	NONE	4.7E-05

P50516	VATA1_MOUSE	ATP6V1A	(P50516) Vacuolar ATP synthase catalytic subunit A, ubiquitous isoform (EC 3.6.3.14) (V-ATPase subunit A 1) (Vacuolar proton pump alpha subunit 1) (V-ATPase 69 kDa subunit 1)	19	25.9	617	68268	5.9	Cytoplasm	transporter	NONE	0.000455
P50518	VATE_MOUSE	ATP6V1E1	(P50518) Vacuolar ATP synthase subunit E (EC 3.6.3.14) (V-ATPase E subunit) (Vacuolar proton pump E subunit) (V-ATPase 31 kDa subunit) (P31)	19	40.8	228	26588	9.2	Cytoplasm	transporter	NONE	0.002437
P50543	S10AB_MOUSE	S100A11 (includes EG:20195)	(P50543) Protein S100-A11 (S100 calcium-binding protein A11) (Protein S100C) (Calgizzarin) (Endothelial monocyte-activating polypeptide) (EMAP)	1	16.3	98	11083	5.5	Unknown	other	NONE	0.002005
P50544	ACADV_MOUSE	ACADVL	(P50544) Very-long-chain specific acyl-CoA dehydrogenase, mitochondrial precursor (EC 1.3.99.-) (VLCAD) (MVLCAD)	18	31.1	656	70876	8.7	Cytoplasm	enzyme	NONE	0.000342
P50580	PA2G4_MOUSE	PA2G4	(P50580) Proliferation-associated protein 2G4 (Proliferation-associated protein 1) (Protein p38-2G4) (Mpp1) (IRES-specific cellular trans-acting factor 45 kDa) (ITAF45)	8	16	393	43568	6.9	Nucleus	transcription regulator	NONE	0.000414
P51125	ICAL_MOUSE	CAST	(P51125) Calpastatin (Calpain inhibitor)	5	10.2	788	84922	5.5	Cytoplasm	peptidase	NONE	0.000128
P51150	RAB7_MOUSE	RAB7	(P51150) Ras-related protein Rab-7	9	52.7	207	23490	6.7	Cytoplasm	enzyme	NONE	0.001708
P51174	ACADL_MOUSE	ACADL	(P51174) Long-chain specific acyl-CoA dehydrogenase, mitochondrial precursor (EC 1.3.99.13) (LCAD)	16	28.1	430	47908	8.3	Cytoplasm	enzyme	NONE	0.000718
P51410	RL9_MOUSE	RPL9	(P51410) 60S ribosomal protein L9	7	25.5	192	21881	10	Cytoplasm	other	NONE	0.001257
P51660	DHB4_MOUSE	HSD17B4	(P51660) Peroxisomal multifunctional enzyme type 2 (MFE-2) (D-bifunctional protein) (DBP) (17-beta-hydroxysteroid dehydrogenase 4) (17-beta-HSD 4) [Includes: D-3-hydroxyacyl-CoA dehydratase (EC 4.2.1.107) (3-alpha,7-alpha,12-alpha-trihydroxy-5	16	22.9	734	79351	8.6	Cytoplasm	enzyme	NONE	0.000336
P51807	DYLT1_MOUSE	DYNLT1	(P51807) Dynein light chain Tctex-type 1 (T-complex testis-specific protein 1) (TCTEX-1)	1	15.9	113	12483	5.1	Cytoplasm	other	NONE	4.97E-05
P51855	GSHB_MOUSE	GSS	(P51855) Glutathione synthetase (EC 6.3.2.3) (Glutathione synthase) (GSH synthetase) (GSH-S)	2	3.4	474	52247	5.8	Cytoplasm	enzyme	NONE	5.92E-05
P51859	HDGF_MOUSE	HDGF	(P51859) Hepatoma-derived growth factor (HDGF)	7	46.8	237	26269	4.8	Extracellular Space	growth factor	NONE	0.00045
P51863	VA0D_MOUSE	ATP6V0D1	(P51863) Vacuolar ATP synthase subunit d (EC 3.6.3.14) (V-ATPase d subunit) (Vacuolar proton pump d subunit) (V-ATPase AC39 subunit) (V-ATPase 40 kDa accessory protein) (P39) (Physophilin)	2	8.3	351	40301	5	Cytoplasm	transporter	NONE	4.8E-05
P51880	FABPB_MOUSE	FABP7	(P51880) Fatty acid-binding protein, brain (B-FABP) (Brain lipid-binding protein) (BLBP)	2	26	131	14762	5.6	Cytoplasm	transporter	NONE	0.000129
P51881	ADT2_MOUSE	SLC25A5	(P51881) ADP/ATP translocase 2 (Adenine nucleotide translocator 2) (ANT 2) (ADP,ATP carrier protein 2) (Solute carrier family 25 member 5)	6	12.1	297	32800	9.7	Cytoplasm	transporter	NONE	0.000416
P51885	LUM_MOUSE	LUM	(P51885) Lumican precursor (Keratan sulfate proteoglycan lumican) (KSPG lumican)	2	8.3	338	38265	6.4	Extracellular Space	other	1	0.000133
P52196	THTR_MOUSE	TST	(P52196) Thiosulfate sulfurtransferase (EC 2.8.1.1) (Rhodanese)	9	22	296	33335	7.9	Cytoplasm	enzyme	NONE	0.000683
P52479	UBP10_MOUSE	USP10	(P52479) Ubiquitin carboxyl-terminal hydrolase 10 (EC 3.1.2.15) (Ubiquitin thioesterase 10) (Ubiquitin-specific-processing protease 10) (Deubiquitinating enzyme 10)	2	1.9	792	87056	5.2	Cytoplasm	peptidase	NONE	2.13E-05
P52480	KPYM_MOUSE	PKM2	(P52480) Pyruvate kinase isozyme M2 (EC 2.7.1.40)	13	32.1	530	57756	7.5	Cytoplasm	kinase	NONE	0.000445
P52503	NUMM_MOUSE	NDUFS6	(P52503) NADH-ubiquinone oxidoreductase 13 kDa-A subunit, mitochondrial precursor (EC 1.6.5.3) (EC 1.6.99.3) (Complex I-13KD-A) (CI-13KD-A)	3	33.6	116	13020	8.7	Cytoplasm	enzyme	NONE	0.001597
P52760	UK114_MOUSE	HRSP12	(P52760) Ribonuclease UK114 (EC 3.1.-.-) (Heat-responsive protein 12)	15	85.1	134	14124	8.7	Cytoplasm	other	NONE	0.013865
P52825	CPT2_MOUSE	CPT2	(P52825) Carnitine O-palmitoyltransferase 2, mitochondrial precursor (EC 2.3.1.21) (Carnitine palmitoyltransferase II) (CPT II)	5	13.5	658	73927	8.2	Cytoplasm	enzyme	NONE	5.97E-05
P52927	HMGA2_MOUSE	HMGA2	(P52927) High mobility group protein HMGI-C (High mobility group AT-hook protein 2)	2	21.3	108	11819	10.6	Nucleus	other	NONE	0.000156
P53026	RL10A_MOUSE	RPL10A	(P53026) 60S ribosomal protein L10a (CSA-19) (Protein NEDD6) (Neural precursor cell expressed developmentally down-regulated protein 6)	13	31	216	24785	10	Cytoplasm	other	NONE	0.001143

P53351	PLK2_MOUSE	PLK2	(P53351) Serine/threonine-protein kinase PLK2 (EC 2.7.11.21) (Polo-like kinase 1) (PLK-2) (Serine/threonine-protein kinase SNK) (Serum-inducible kinase)	2	4.8	682	77812	8.2	Nucleus	kinase	NONE	1.65E-05
P53395	ODB2_MOUSE	DBT	(P53395) Lipoamide acyltransferase component of branched-chain alpha-keto acid dehydrogenase complex, mitochondrial precursor (EC 2.3.1.168) (Dihydrolipoyllysine-residue (2-methylpropanoyl)transferase) (E2) (Dihydrolipoamide branched chain tra	7	20.3	482	53160	8.7	Cytoplasm	enzyme	NONE	0.000303
P53564	CUTL1_MOUSE	CUTL1	(P53564) Homeobox protein cut-like 1 (CCAAT displacement protein) (CDP) (Homeobox protein Cux)	3	4.3	1515	165595	6.3	Nucleus	transcription regulator	NONE	2.22E-05
P53702	CCHL_MOUSE	HCCS	(P53702) Cytochrome c-type heme lyase (EC 4.4.1.17) (CCHL) (Holocytochrome c-type synthase)	1	4.8	272	31008	6.9	Cytoplasm	enzyme	NONE	4.13E-05
P53811	PIPNB_MOUSE	PITPNB	(P53811) Phosphatidylinositol transfer protein beta isoform (PtdIns transfer protein beta) (PtdInsTP) (PI-TP-beta)	1	8.9	270	31356	6.9	Cytoplasm	transporter	NONE	2.08E-05
P53994	RAB2A_MOUSE	RAB2	(P53994) Ras-related protein Rab-2A	5	22.6	212	23548	6.5	Cytoplasm	enzyme	NONE	0.000238
P53996	CNBP_MOUSE	CNBP	(P53996) Cellular nucleic acid-binding protein (CNBP) (Zinc finger protein 9)	2	6.7	178	19592	7.6	Nucleus	transcription regulator	NONE	9.46E-05
P54071	IDHP_MOUSE	IDH2	(P54071) Isocitrate dehydrogenase [NADP], mitochondrial precursor (EC 1.1.1.42) (Oxalosuccinate decarboxylase) (IDH) (NADP(+)-specific ICDH) (IDP) (ICD-M)	27	27.2	523	58749	8.7	Cytoplasm	enzyme	NONE	0.001953
P54726	RD23A_MOUSE	RAD23A	(P54726) UV excision repair protein RAD23 homolog A (mHR23A)	3	12.7	363	39770	4.6	Nucleus	other	NONE	9.28E-05
P54728	RD23B_MOUSE	RAD23B	(P54728) UV excision repair protein RAD23 homolog B (mHR23B) (XP-C repair-complementing complex 58 kDa protein) (p58)	4	21.2	416	43517	4.8	Nucleus	other	NONE	0.000135
P54775	PRS6B_MOUSE	PSMC4	(P54775) 26S protease regulatory subunit 6B (MIP224) (MB67-interacting protein) (TAT-binding protein 7) (TBP-7) (CIP21)	1	7.9	418	47281	5.3	Nucleus	peptidase	NONE	2.69E-05
P54797	T10_MOUSE	C22ORF25	(P54797) Ser/Thr-rich protein T10 in DGCR region	2	13.4	276	30947	5.1	Unknown	other	NONE	8.13E-05
P54818	GALC_MOUSE	GALC	(P54818) Galactocerebrosidase precursor (EC 3.2.1.46) (GALCERase) (Galactosylceramidase) (Galactosylceramide beta-galactosidase) (Galactocerebroside beta-galactosidase)	1	2.4	668	75503	6.5	Cytoplasm	enzyme	1	2.52E-05
P55012	S12A2_MOUSE	SLC12A2	(P55012) Solute carrier family 12 member 2 (Bumetanide-sensitive sodium-(potassium)-chloride cotransporter 1) (Basolateral Na-K-Cl symporter)	1	2.2	1205	130950	7.2	Plasma Membrane	transporter	11	4.66E-06
P55014	S12A1_MOUSE	SLC12A1	(P55014) Solute carrier family 12 member 1 (Bumetanide-sensitive sodium-(potassium)-chloride cotransporter 2) (BSC1) (Kidney-specific Na-K-Cl symporter)	5	5.5	1095	120355	7.8	Plasma Membrane	transporter	9	4.61E-05
P55096	ABCD3_MOUSE	ABCD3	(P55096) ATP-binding cassette sub-family D member 3 (70 kDa peroxisomal membrane protein) (PMP70) (PMP68)	4	10	659	75483	9.3	Cytoplasm	transporter	3	6.81E-05
P55200	HRX_MOUSE	MLL	(P55200) Zinc finger protein HRX (ALL-1) (Fragment)	3	1.1	3866	420987	9.1	Nucleus	transcription regulator	NONE	5.81E-06
P55258	RAB8A_MOUSE	RAB8A	(P55258) Ras-related protein Rab-8A (Oncogene c-mel)	3	12.6	207	23668	9.1	Cytoplasm	enzyme	NONE	0.000136
P55264	ADK_MOUSE	ADK	(P55264) Adenosine kinase (EC 2.7.1.20) (AK) (Adenosine 5'-phosphotransferase)	2	11.6	361	40149	6.2	Unknown	kinase	NONE	6.22E-05
P55302	AMRP_MOUSE	LRPAP1	(P55302) Alpha-2-macroglobulin receptor-associated protein precursor (Alpha-2-MRAP) (Low density lipoprotein receptor-related protein-associated protein 1) (RAP) (Heparin-binding protein 44) (HBP-44)	23	36.1	360	42215	7.9	Plasma Membrane	transmembrane receptor	NONE	0.001216
P55937	GOGA3_MOUSE	GOLGA3	(P55937) Golgin subfamily A member 3 (Golgin-160) (Male-enhanced antigen 2) (MEA-2)	2	2	1487	167219	5.4	Cytoplasm	transporter	NONE	1.51E-05
P56135	ATPK_MOUSE	ATP5J2	(P56135) ATP synthase f chain, mitochondrial (EC 3.6.3.14)	4	26.4	87	10213	9.9	Cytoplasm	transporter	1	0.001548
P56375	ACYP2_MOUSE	ACYP2	(P56375) Acylphosphatase, muscle type isozyme (EC 3.6.1.7) (Acylphosphate phosphohydrolase)	2	18.6	97	10895	8.7	Unknown	enzyme	NONE	0.000174

P56376	ACYP1_MOUSE	ACYP1	(P56376) Acylphosphatase, organ-common type isozyme (EC 3.6.1.7) (Acylphosphate phosphohydrolase)	2	21.4	98	11110	9.1	Unknown	enzyme	NONE	0.000229
P56382	ATP5E_MOUSE	ATP5E	(P56382) ATP synthase epsilon chain, mitochondrial (EC 3.6.3.14)	3	39.2	51	5707	10	Cytoplasm	transporter	NONE	0.00044
P56389	CDD_MOUSE	CDA	(P56389) Cytidine deaminase (EC 3.5.4.5) (Cytidine aminohydrolase)	6	47.9	146	16131	5.6	Nucleus	enzyme	NONE	0.001538
P56391	CX6B1_MOUSE	COX6B1	(P56391) Cytochrome c oxidase subunit VIb isoform 1 (EC 1.9.3.1) (COX VIb-1)	3	44.7	85	9940	8.7	Cytoplasm	enzyme	NONE	0.00066
P56394	COX17_MOUSE	COX17	(P56394) Cytochrome c oxidase copper chaperone	1	25.8	62	6653	7.7	Cytoplasm	enzyme	NONE	0.000272
P56395	CYB5_MOUSE	CYB5A	(P56395) Cytochrome b5	9	48.1	133	15110	5.1	Cytoplasm	enzyme	1	0.003503
P56399	UBP5_MOUSE	USP5	(P56399) Ubiquitin carboxyl-terminal hydrolase 5 (EC 3.1.2.15) (Ubiquitin thioesterase 5) (Ubiquitin-specific-processing protease 5) (Deubiquitinating enzyme 5) (Isopeptidase T)	1	2.2	858	95833	5	Cytoplasm	peptidase	NONE	1.31E-05
P56480	ATPB_MOUSE	ATP5B	(P56480) ATP synthase beta chain, mitochondrial precursor (EC 3.6.3.14)	57	59.7	529	56301	5.3	Cytoplasm	transporter	NONE	0.007088
P56565	S10A1_MOUSE	S100A1	(P56565) Protein S100-A1 (S100 calcium-binding protein A1) (S-100 protein alpha subunit) (S-100 protein alpha chain)	2	19.4	93	10374	4.5	Cytoplasm	other	1	0.000181
P56677	ST14_MOUSE	ST14	(P56677) Suppressor of tumorigenicity protein 14 (EC 3.4.21.-) (Serine protease 14) (Epithin)	1	3.5	855	94655	6.8	Plasma Membrane	peptidase	1	6.57E-06
P56812	PDCD5_MOUSE	PDCD5	(P56812) Programmed cell death protein 5 (Protein TFAR19) (TF-1 cell apoptosis-related gene 19 protein)	2	19.2	125	14144	5.7	Nucleus	other	NONE	0.000314
P56873	SSA27_MOUSE	SSSCA1 (includes EG:10534)	(P56873) Sjoegren syndrome/scleroderma autoantigen 1 homolog (Autoantigen p27 homolog) (Protein C184L)	2	13.6	199	21336	5.1	Unknown	other	NONE	0.000169
P56959	FUS_MOUSE	FUS	(P56959) RNA-binding protein FUS (Pigpen protein)	10	18	518	52673	9.4	Nucleus	transcription regulator	NONE	0.000271
P57016	LAD1_MOUSE	LAD1	(P57016) Ladinin 1 (Lad-1) (Linear IgA disease autoantigen)	12	27.3	528	58864	9.7	Extracellular Space	other	NONE	0.000202
P57746	VATD_MOUSE	ATP6V1D	(P57746) Vacuolar ATP synthase subunit D (EC 3.6.3.14) (V-ATPase D subunit) (Vacuolar proton pump D subunit) (V-ATPase 28 kDa accessory protein)	10	36.8	247	28369	9.4	Cytoplasm	transporter	NONE	0.000568
P57759	ERP29_MOUSE	ERP29	(P57759) Endoplasmic reticulum protein ERp29 precursor	13	40.8	262	28823	6.1	Cytoplasm	transporter	NONE	0.000707
P57776	EF1D_MOUSE	EEF1D	(P57776) Elongation factor 1-delta (EF-1-delta)	11	32.5	280	31162	5	Cytoplasm	translation regulator	NONE	0.000401
P57780	ACTN4_MOUSE	ACTN4	(P57780) Alpha-actinin-4 (Non-muscle alpha-actinin 4) (F-actin cross linking protein)	17	21.4	912	104977	5.4	Cytoplasm	other	NONE	0.000209
P57784	RU2A_MOUSE	SNRPA1	(P57784) U2 small nuclear ribonucleoprotein A' (U2 snRNP-A')	3	24.7	255	28357	8.6	Nucleus	other	NONE	0.000154
P58044	IDI1_MOUSE	IDI1	(P58044) Isopentenyl-diphosphate delta-isomerase 1 (EC 5.3.3.2) (IPP isomerase 1) (Isopentenyl pyrophosphate isomerase 1) (IPPI1)	2	9.7	227	26289	6.2	Cytoplasm	enzyme	NONE	0.000124
P58059	RT21_MOUSE	MRPS21	(P58059) Mitochondrial 28S ribosomal protein S21 (S21mt) (MRP-S21)	2	31	87	10561	10.3	Cytoplasm	other	NONE	0.000194
P58137	ACOT8_MOUSE	ACOT8	(P58137) Acyl-coenzyme A thioesterase 8 (EC 3.1.2.2) (Acyl-CoA thioesterase 8) (Peroxisomal acyl-coenzyme A thioester hydrolase 1) (PTE-1) (Peroxisomal long-chain acyl-coA thioesterase 1) (Peroxisomal acyl-CoA thioesterase 2) (PTE-2)	1	5	320	35827	7.6	Cytoplasm	enzyme	NONE	5.26E-05
P58252	EF2_MOUSE	EEF2	(P58252) Elongation factor 2 (EF-2)	5	7.6	857	95183	6.8	Cytoplasm	translation regulator	NONE	7.86E-05
P58281	OPA1_MOUSE	OPA1	(P58281) Dynamin-like 120 kDa protein, mitochondrial precursor (Large GTP-binding protein) (LargeG)	10	12.1	960	111339	7.5	Cytoplasm	enzyme	NONE	9.36E-05
P58389	PTPA_MOUSE	PPP2R4	(P58389) Serine/threonine-protein phosphatase 2A regulatory subunit B' (PP2A, subunit B', PR53 isoform) (Phosphotyrosyl phosphatase activator) (PTPA)	2	12.1	323	36710	6.4	Cytoplasm	phosphatase	1	3.48E-05
P58771	TPM1_MOUSE	TPM1	(P58771) Tropomyosin 1 alpha chain (Alpha-tropomyosin)	14	34.5	284	32681	4.7	Cytoplasm	other	NONE	0.000712

P58774	TPM2_MOUSE	TPM2	(P58774) Tropomyosin beta chain (Tropomyosin 2) (Beta-tropomyosin)	3	9.9	284	32837	4.7	Cytoplasm	other	NONE	0.000158
P58871	TB182_MOUSE	TNKS1BP1	(P58871) 182 kDa tankyrase 1-binding protein (Fragment)	5	6.1	909	97079	4.8	Nucleus	other	NONE	9.26E-05
P59017	B2L13_MOUSE	BCL2L13	(P59017) Bcl-2-like 13 protein (Protein Mil1) (Bcl-rambo)	2	8.3	434	46669	4.6	Cytoplasm	other	1	7.76E-05
P59158	S12A3_MOUSE	SLC12A3	(P59158) Solute carrier family 12 member 3 (Thiazide-sensitive sodium-chloride cotransporter) (Na-Cl symporter)	1	1	1002	110694	7.6	Plasma Membrane	transporter	10	1.12E-05
P59325	IF5_MOUSE	EIF5	(P59325) Eukaryotic translation initiation factor 5 (eIF-5)	5	15.2	429	48968	5.5	Cytoplasm	translation regulator	NONE	0.000157
P59729	RIN3_MOUSE	RIN3	(P59729) Ras and Rab interactor 3 (Ras interaction/interference protein 3)	2	4.3	980	107275	7	Unknown	other	NONE	1.72E-05
P59808	SASH1_MOUSE	SASH1	(P59808) SAM and SH3 domain-containing protein 1	1	1	1230	135591	6.2	Unknown	other	NONE	4.56E-06
P59999	ARPC4_MOUSE	ARPC4	(P59999) Actin-related protein 2/3 complex subunit 4 (ARP2/3 complex 20 kDa subunit) (p20-ARC)	2	13.2	167	19536	8.4	Unknown	other	NONE	0.000168
P60229	IF36_MOUSE	EIF3S6	(P60229) Eukaryotic translation initiation factor 3 subunit 6 (eIF-3 p48) (eIF3e) (Mammary tumor-associated protein INT-6) (Viral integration site protein INT-6) (MMTV integration site 6)	1	4.5	445	52221	6	Cytoplasm	translation regulator	NONE	1.26E-05
P60335	PCBP1_MOUSE	PCBP1 (includes EG:5093)	(P60335) Poly(rC)-binding protein 1 (Alpha-CP1) (hnRNP-E1)	9	39	356	37498	7.1	Nucleus	translation regulator	NONE	0.000457
P60710	ACTB_MOUSE	ACTB	(P60710) Actin, cytoplasmic 1 (Beta-actin)	16	33.9	375	41737	5.5	Cytoplasm	other	NONE	0.004236
P60762	MO4L1_MOUSE	MORF4L1	(P60762) Mortality factor 4-like protein 1 (MORF-related gene 15 protein) (Transcription factor-like protein MRG15) (Testis-expressed gene 189 protein)	2	9.1	362	41493	9.3	Nucleus	other	NONE	3.1E-05
P60824	CIRBP_MOUSE	CIRBP	(P60824) Cold-inducible RNA-binding protein (Glycine-rich RNA-binding protein CIRP) (A18 hnRNP)	1	12.2	172	18607	9.6	Nucleus	other	NONE	0.000653
P60843	IF4A1_MOUSE	EIF4A1	(P60843) Eukaryotic initiation factor 4A-I (EC 3.6.1.-) (ATP-dependent RNA helicase eIF4A-1) (eIF4A-I) (eIF-4A-I)	3	8.6	406	46154	5.5	Cytoplasm	translation regulator	NONE	0.000124
P60867	RS20_MOUSE	RPS20	(P60867) 40S ribosomal protein S20	3	22.7	119	13373	9.9	Cytoplasm	other	NONE	0.000472
P61021	RAB5B_MOUSE	RAB5B	(P61021) Ras-related protein Rab-5B	1	6.5	215	23707	8.1	Cytoplasm	enzyme	NONE	7.83E-05
P61022	CHP1_MOUSE	CHP	(P61022) Calcium-binding protein p22 (Calcium-binding protein CHP) (Calcineurin homologous protein) (Sid 470)	8	40.2	194	22301	5.1	Cytoplasm	transporter	NONE	0.000665
P61027	RAB10_MOUSE	RAB10	(P61027) Ras-related protein Rab-10	1	5.5	200	22541	8.4	Cytoplasm	enzyme	NONE	0.00014
P61082	UBC12_MOUSE	UBE2M	(P61082) NEDD8-conjugating enzyme Ubc12 (EC 6.3.2.-) (Ubiquitin-conjugating enzyme E2 M) (NEDD8 protein ligase) (NEDD8 carrier protein)	1	6	183	20900	7.7	Cytoplasm	enzyme	NONE	6.13E-05
P61087	UBC1_MOUSE	HIP2	(P61087) Ubiquitin-conjugating enzyme E2-25 kDa (EC 6.3.2.19) (Ubiquitin-protein ligase) (Ubiquitin carrier protein) (E2(25K)) (Huntingtin-interacting protein 2) (HIP-2)	1	9.5	199	22275	5.4	Cytoplasm	transcription regulator	NONE	5.64E-05
P61089	UBE2N_MOUSE	UBE2N	(P61089) Ubiquitin-conjugating enzyme E2 N (EC 6.3.2.19) (Ubiquitin-protein ligase N) (Ubiquitin carrier protein N) (Ubc13) (Bendless-like ubiquitin-conjugating enzyme)	7	42.8	152	17138	6.6	Cytoplasm	enzyme	NONE	0.000665
P61110	ANRE_MOUSE	KAP	(P61110) Kidney androgen-regulated protein precursor (KAP) (ARP)	2	22.3	121	13263	4.1	Extracellular Space	other	1	0.000139
P61161	ARP2_MOUSE	ACTR2	(P61161) Actin-like protein 2 (Actin-related protein 2)	2	7.6	394	44761	6.7	Plasma Membrane	other	NONE	7.12E-05
P61164	ACTZ_MOUSE	ACTR1A	(P61164) Alpha-centractin (Centractin) (Centrosome-associated actin homolog) (Actin-RPV) (ARP1)	3	9.8	376	42614	6.6	Cytoplasm	other	NONE	7.46E-05
P61211	ARL1_MOUSE	ARL1	(P61211) ADP-ribosylation factor-like protein 1	1	8.8	181	20412	5.7	Cytoplasm	enzyme	NONE	9.3E-05
P61255	RL26_MOUSE	RPL26	(P61255) 60S ribosomal protein L26 (Silica-induced gene 20 protein) (SIG-20)	4	28.3	145	17258	10.6	Cytoplasm	other	NONE	0.00089
P61290	PSME3_MOUSE	PSME3	(P61290) Proteasome activator complex subunit 3 (Proteasome activator 28-gamma subunit) (PA28gamma) (PA28g) (Activator of multicatalytic protease subunit 3) (11S regulator complex subunit gamma) (REG-gamma) (Ki nuclear autoantigen)	1	5.9	254	29506	6	Cytoplasm	peptidase	NONE	4.42E-05
P61358	RL27_MOUSE	RPL27	(P61358) 60S ribosomal protein L27	5	48.9	135	15667	10.6	Cytoplasm	other	NONE	0.000416

P61458	PHS_MOUSE	PCBD1	(P61458) Pterin-4-alpha-carbinolamine dehydratase (EC 4.2.1.96) (PHS) (4-alpha-hydroxy-tetrahydropterin dehydratase) (Phenylalanine hydroxylase-stimulating protein) (Pterin carbinolamine dehydratase) (PCD) (Dimerization cofactor of hepatocyte n	9	56.3	103	11854	6.8	Nucleus	transcription regulator	NONE	0.001798
P61759	PFD3_MOUSE	VBP1	(P61759) Prefoldin subunit 3 (Von Hippel-Lindau-binding protein 1) (VHL-binding protein 1) (VBP-1)	2	12.8	196	22436	6.3	Cytoplasm	other	NONE	0.000172
P61922	GABT_MOUSE	ABAT	(P61922) 4-aminobutyrate aminotransferase, mitochondrial precursor (EC 2.6.1.19) ((S)-3-amino-2-methylpropionate transaminase) (EC 2.6.1.22) (Gamma-amino-N-butyrate transaminase) (GABA transaminase) (GABA aminotransferase) (GABA-AT) (GABA-T) (	12	29.8	500	56452	8.1	Cytoplasm	enzyme	NONE	0.000292
P61924	COPZ1_MOUSE	COPZ1	(P61924) Coatomer subunit zeta-1 (Zeta-1 coat protein) (Zeta-1 COP)	2	13	177	20198	4.8	Cytoplasm	transporter	NONE	0.000159
P61961	UFM1_MOUSE	UFM1	(P61961) Ubiquitin-fold modifier 1 precursor	2	58.8	85	9118	9.3	Unknown	other	NONE	0.00066
P61967	AP1S1_MOUSE	AP1S1	(P61967) AP-1 complex subunit sigma-1A (Adapter-related protein complex 1 sigma-1A subunit) (Sigma-adaptin 1A) (Adaptor protein complex AP-1 sigma-1A subunit) (Golgi adaptor HA1/AP1 adaptin sigma-1A subunit) (Clathrin assembly protein complex	2	14.6	158	18733	5.7	Cytoplasm	transporter	NONE	0.000178
P61979	HNRPK_MOUSE	HNRPK	(P61979) Heterogeneous nuclear ribonucleoprotein K	7	19.9	463	50976	5.5	Nucleus	other	NONE	0.000618
P61982	1433G_MOUSE	YWHAG	(P61982) 14-3-3 protein gamma	2	9.8	246	28171	4.9	Cytoplasm	other	NONE	0.00016
P62071	RRAS2_MOUSE	RRAS2	(P62071) Ras-related protein R-Ras2	4	28.9	204	23400	6	Plasma Membrane	enzyme	NONE	0.000193
P62073	TIM10_MOUSE	TIMM10	(P62073) Mitochondrial import inner membrane translocase subunit Tim10	4	32.2	90	10333	6.3	Cytoplasm	transporter	NONE	0.000312
P62075	TIM13_MOUSE	TIMM13	(P62075) Mitochondrial import inner membrane translocase subunit Tim13	1	14.7	95	10458	8.2	Cytoplasm	transporter	NONE	0.001241
P62082	RS7_MOUSE	RPS7	(P62082) 40S ribosomal protein S7	10	55.7	194	22127	10.1	Cytoplasm	other	NONE	0.002488
P62192	PRS4_MOUSE	PSMC1	(P62192) 26S protease regulatory subunit 4 (P26s4) (Proteasome 26S subunit ATPase 1)	3	11.6	440	49185	6.2	Nucleus	peptidase	NONE	8.93E-05
P62196	PRS8_MOUSE	PSMC5	(P62196) 26S protease regulatory subunit 8 (Proteasome subunit p45) (p45/SUG) (Proteasome 26S subunit ATPase 5) (mSUG1)	6	24.9	406	45626	7.5	Nucleus	transcription regulator	NONE	8.3E-05
P62242	RS8_MOUSE	RPS8	(P62242) 40S ribosomal protein S8	7	28.5	207	24074	10.3	Cytoplasm	other	NONE	0.000841
P62245	RS15A_MOUSE	RPS15A	(P62245) 40S ribosomal protein S15a	6	34.1	129	14708	10.1	Cytoplasm	other	NONE	0.000609
P62259	1433E_MOUSE	YWHAE	(P62259) 14-3-3 protein epsilon (14-3-3E)	11	57.3	255	29174	4.7	Cytoplasm	other	NONE	0.001299
P62264	RS14_MOUSE	RPS14	(P62264) 40S ribosomal protein S14	13	35.3	150	16141	10.1	Cytoplasm	other	NONE	0.00116
P62267	RS23_MOUSE	RPS23	(P62267) 40S ribosomal protein S23	1	7.7	142	15676	10.5	Cytoplasm	translation regulator	NONE	0.000632
P62270	RS18_MOUSE	RPS18	(P62270) 40S ribosomal protein S18 (Ke-3) (Ke3)	12	52	152	17719	11	Cytoplasm	other	NONE	0.001182
P62281	RS11_MOUSE	RPS11	(P62281) 40S ribosomal protein S11	6	43.9	157	18300	10.3	Cytoplasm	other	NONE	0.000429
P62301	RS13_MOUSE	RPS13	(P62301) 40S ribosomal protein S13	10	33.3	150	17091	10.5	Cytoplasm	other	NONE	0.001908
P62315	SMD1_MOUSE	SNRPD1	(P62315) Small nuclear ribonucleoprotein Sm D1 (snRNP core protein D1) (Sm-D1) (Sm-D autoantigen)	4	27.7	119	13282	11.6	Nucleus	other	NONE	0.001462
P62317	SMD2_MOUSE	SNRPD2	(P62317) Small nuclear ribonucleoprotein Sm D2 (snRNP core protein D2) (Sm-D2)	4	33.1	118	13527	9.9	Nucleus	other	NONE	0.000618
P62320	SMD3_MOUSE	SNRPD3	(P62320) Small nuclear ribonucleoprotein Sm D3 (snRNP core protein D3) (Sm-D3)	2	7.1	126	13916	10.3	Nucleus	other	NONE	8.91E-05
P62334	PRS10_MOUSE	PSMC6	(P62334) 26S protease regulatory subunit S10B (Proteasome subunit p42) (Proteasome 26S subunit ATPase 6)	4	14.7	389	44173	7.5	Nucleus	peptidase	NONE	8.66E-05
P62492	RB11A_MOUSE	RAB11A	(P62492) Ras-related protein Rab-11A (Rab-11)	4	20.9	215	24262	6.5	Cytoplasm	enzyme	NONE	0.000392
P62627	DLC2A_MOUSE	DYNLRB1	(P62627) Dynein light chain 2A, cytoplasmic	1	22.1	95	10858	7.2	Cytoplasm	other	NONE	0.000177
P62702	RS4X_MOUSE	RPS4X	(P62702) 40S ribosomal protein S4, X isoform	12	36.6	262	29467	10.2	Cytoplasm	other	NONE	0.001007
P62737	ACTA_MOUSE	ACTA2	(P62737) Actin, aortic smooth muscle (Alpha-actin-2)	41	44	377	42009	5.4	Cytoplasm	other	NONE	0.002948
P62751	RL23A_MOUSE	RPL23A	(P62751) 60S ribosomal protein L23a	7	21.8	156	17695	10.4	Cytoplasm	other	NONE	0.000468
P62754	RS6_MOUSE	RPS6	(P62754) 40S ribosomal protein S6 (Phosphoprotein NP33)	10	27.7	249	28681	10.8	Cytoplasm	other	NONE	0.001623
P62774	MTPN_MOUSE	MTPN	(P62774) Myotrophin (Protein V-1) (Granule cell differentiation protein)	4	31.6	117	12730	5.5	Nucleus	transcription regulator	NONE	0.000624
P62806	H4_MOUSE	HIST1H4C	(P62806) Histone H4	20	54.9	102	11236	11.4	Nucleus	other	NONE	0.005723

P62814	VATB2_MOUSE	ATP6V1B2	(P62814) Vacuolar ATP synthase subunit B, brain isoform (EC 3.6.3.14) (V-ATPase B2 subunit) (Vacuolar proton pump B isoform 2) (Endomembrane proton pump 58 kDa subunit)	19	34.6	511	56551	5.8	Cytoplasm	transporter	NONE	0.000626
P62821	RAB1A_MOUSE	RAB1A	(P62821) Ras-related protein Rab-1A (YPT1-related protein)	7	20.6	204	22547	6.2	Cytoplasm	enzyme	NONE	0.001376
P62827	RAN_MOUSE	RAN	(P62827) GTP-binding nuclear protein Ran (GTPase Ran) (Ras-like protein TC4)	2	6.5	215	24292	7.5	Nucleus	enzyme	NONE	0.000653
P62830	RL23_MOUSE	RPL23	(P62830) 60S ribosomal protein L23	8	29.3	140	14865	10.5	Cytoplasm	other	NONE	0.001965
P62835	RAP1A_MOUSE	RAP1A	(P62835) Ras-related protein Rap-1A precursor (Ras-related protein Krev-1)	7	21.7	184	20987	6.6	Cytoplasm	enzyme	NONE	0.001464
P62843	RS15_MOUSE	RPS15 (includes EG:20054)	(P62843) 40S ribosomal protein S15 (RIG protein)	15	47.2	144	16909	10.4	Unknown	other	NONE	0.002612
P62849	RS24_MOUSE	RPS24 (includes EG:20088)	(P62849) 40S ribosomal protein S24	6	33.8	133	15423	10.8	Unknown	other	NONE	0.001984
P62852	RS25_MOUSE	RPS25	(P62852) 40S ribosomal protein S25	5	17.6	125	13742	10.1	Cytoplasm	other	NONE	0.000584
P62855	RS26_MOUSE	RPS26	(P62855) 40S ribosomal protein S26	5	43	114	12884	11	Cytoplasm	other	NONE	0.001083
P62858	RS28_MOUSE	RPS28 (includes EG:54127)	(P62858) 40S ribosomal protein S28	7	44.9	69	7841	10.7	Nucleus	other	NONE	0.001708
P62862	RS30_MOUSE	FAU	(P62862) 40S ribosomal protein S30	4	20.3	59	6648	12.1	Cytoplasm	other	NONE	0.001237
P62889	RL30_MOUSE	RPL30	(P62889) 60S ribosomal protein L30	3	21.1	114	12653	9.6	Cytoplasm	other	NONE	0.000443
P62897	CYC_MOUSE	CYCS	(P62897) Cytochrome c, somatic	11	44.2	104	11474	9.6	Cytoplasm	enzyme	NONE	0.003562
P62900	RL31_MOUSE	RPL31	(P62900) 60S ribosomal protein L31	4	25.6	125	14463	10.5	Cytoplasm	other	NONE	0.000629
P62908	RS3_MOUSE	RPS3	(P62908) 40S ribosomal protein S3	7	32.5	243	26674	9.7	Cytoplasm	other	NONE	0.000785
P62911	RL32_MOUSE	RPL32	(P62911) 60S ribosomal protein L32	7	22.4	134	15729	11.3	Cytoplasm	other	NONE	0.00067
P62918	RL8_MOUSE	RPL8	(P62918) 60S ribosomal protein L8	13	33.6	256	27893	11	Cytoplasm	other	NONE	0.000833
P62960	YBOX1_MOUSE	YBX1	(P62960) Nuclease sensitive element-binding protein 1 (Y-box-binding protein 1) (Y-box transcription factor) (YB-1) (CCAAT-binding transcription factor I subunit A) (CBF-A) (Enhancer factor I subunit A) (EFI-A) (DNA-binding protein B) (DBPB)	5	27.7	321	35599	9.9	Nucleus	transcription regulator	NONE	0.001067
P62962	PROF1_MOUSE	PFN1	(P62962) Profilin-1 (Profilin I)	5	33.1	139	14826	8.3	Cytoplasm	other	NONE	0.001656
P62983	RS27A_MOUSE	RPS27A	(P62983) 40S ribosomal protein S27a	3	25	80	9404	9.8	Cytoplasm	other	NONE	0.000702
P62991	UBIQ_MOUSE	RPS27A	(P62991) Ubiquitin	4	47.4	76	8565	7.2	Cytoplasm	other	NONE	0.000591
P62996	TRA2B_MOUSE	SFRS10	(P62996) Arginine/serine-rich-splicing factor 10 (Transformer-2-beta) (HTRA2-beta) (Transformer 2 protein homolog) (Silica-induced gene 41 protein) (SIG-41)	1	5.6	288	33666	11.2	Nucleus	other	NONE	3.9E-05
P63001	RAC1_MOUSE	RAC1	(P63001) Ras-related C3 botulinum toxin substrate 1 precursor (p21-Rac1)	6	19.3	192	21450	8.5	Cytoplasm	enzyme	NONE	0.00038
P63017	HSP7C_MOUSE	HSPA8	(P63017) Heat shock cognate 71 kDa protein (Heat shock 70 kDa protein 8)	33	34.5	646	70871	5.5	Cytoplasm	enzyme	NONE	0.001234
P63024	VAMP3_MOUSE	VAMP3	(P63024) Vesicle-associated membrane protein 3 (VAMP-3) (Synaptobrevin-3) (Cellubrevin) (CEB)	1	15.5	103	11480	8.5	Plasma Membrane	other	1	0.000163
P63028	TCTP_MOUSE	TPT1	(P63028) Translationally-controlled tumor protein (TCTP) (p23) (21 kDa polypeptide) (p21)	7	24.4	172	19462	4.9	Cytoplasm	other	NONE	0.000816
P63037	DNJA1_MOUSE	DNAJA1	(P63037) DnaJ homolog subfamily A member 1 (Heat shock 40 kDa protein 4) (DnaJ protein homolog 2) (HSJ-2)	9	20.9	397	44868	7.1	Nucleus	other	NONE	0.000424
P63038	CH60_MOUSE	HSPD1	(P63038) 60 kDa heat shock protein, mitochondrial precursor (Hsp60) (60 kDa chaperonin) (CPN60) (Heat shock protein 60) (HSP-60) (Mitochondrial matrix protein P1) (HSP-65)	64	69.3	573	60956	6.2	Cytoplasm	other	NONE	0.010188
P63044	VAMP2_MOUSE	VAMP2	(P63044) Vesicle-associated membrane protein 2 (VAMP-2) (Synaptobrevin-2)	3	35.7	115	12560	8.1	Plasma Membrane	other	1	0.000293
P63073	IF4E_MOUSE	EIF4E	(P63073) Eukaryotic translation initiation factor 4E (eIF4E) (eIF-4E) (mRNA cap-binding protein) (eIF-4F 25 kDa subunit)	1	6.5	217	25053	6.1	Cytoplasm	translation regulator	NONE	2.59E-05
P63094	GNAS_MOUSE	GNAS	(P63094) Guanine nucleotide-binding protein G(s) subunit alpha (Adenylate cyclase-stimulating G alpha protein)	2	7.1	394	45664	6	Plasma Membrane	enzyme	NONE	7.12E-05
P63101	1433Z_MOUSE	YWHAZ	(P63101) 14-3-3 protein zeta/delta (Protein kinase C inhibitor protein 1) (KCIP-1) (SEZ-2)	16	38.4	245	27771	4.8	Cytoplasm	enzyme	NONE	0.00181

P63158	HMGB1_MOUSE	HMGB1	(P63158) High mobility group protein B1 (High mobility group protein 1) (HMG-1)	12	23.4	214	24763	5.7	Nucleus	other	NONE	0.001207
P63242	IF5A1_MOUSE	EIF5A	(P63242) Eukaryotic translation initiation factor 5A-1 (eIF-5A-1) (eIF-5A1) (Eukaryotic initiation factor 5A isoform 1) (eIF-5A) (eIF-4D)	9	34.6	153	16701	5.2	Cytoplasm	translation regulator	NONE	0.004879
P63254	CRIP1_MOUSE	CRIP1	(P63254) Cysteine-rich protein 1 (Cysteine-rich intestinal protein) (CRIP)	1	36.8	76	8419	8.6	Cytoplasm	other	NONE	0.000148
P63276	RS17_MOUSE	RPS17 (includes EG:6218)	(P63276) 40S ribosomal protein S17	13	47.8	134	15393	9.8	Cytoplasm	other	NONE	0.004147
P63321	RALA_MOUSE	RALA	(P63321) Ras-related protein Ral-A	7	26.2	206	23553	7.1	Cytoplasm	enzyme	NONE	0.000545
P63323	RS12_MOUSE	RPS12	(P63323) 40S ribosomal protein S12	4	26.7	131	14394	7.2	Cytoplasm	other	NONE	0.000514
P63325	RS10_MOUSE	RPS10	(P63325) 40S ribosomal protein S10	10	49.1	165	18916	10.2	Cytoplasm	other	NONE	0.003402
P63330	PP2AA_MOUSE	PPP2CA	(P63330) Serine/threonine-protein phosphatase 2A catalytic subunit alpha isoform (EC 3.1.3.16) (PP2A-alpha)	1	3.6	309	35608	5.5	Cytoplasm	phosphatase	NONE	7.27E-05
P67778	PHB_MOUSE	PHB	(P67778) Prohibitin (B-cell receptor-associated protein 32) (BAP 32)	9	35.7	272	29820	5.8	Nucleus	transcription regulator	NONE	0.001156
P67984	RL22_MOUSE	RPL22	(P67984) 60S ribosomal protein L22 (Heparin-binding protein HBp15)	4	49.6	127	14628	9.2	Nucleus	other	NONE	0.000575
P68037	UB2L3_MOUSE	UBE2L3	(P68037) Ubiquitin-conjugating enzyme E2 L3 (EC 6.3.2.19) (Ubiquitin-protein ligase L3) (Ubiquitin carrier protein L3) (UbcM4)	4	42.9	154	17862	8.5	Cytoplasm	enzyme	NONE	0.000656
P68040	GBLP_MOUSE	GNB2L1	(P68040) Guanine nucleotide-binding protein subunit beta 2-like 1 (Receptor of activated protein kinase C 1) (RACK1) (Receptor for activated C kinase) (p205) (12-3)	4	16.8	316	34946	7.7	Cytoplasm	enzyme	NONE	0.000107
P68254	1433T_MOUSE	YWHAQ	(P68254) 14-3-3 protein theta (14-3-3 protein tau)	5	24.1	245	27778	4.8	Cytoplasm	other	NONE	0.000321
P68368	TBA4_MOUSE	TUBA1	(P68368) Tubulin alpha-4 chain (Alpha-tubulin 4) (Alpha-tubulin isotype M-alpha-4)	4	11.4	448	49924	5.1	Cytoplasm	other	NONE	0.0002
P68369	TBA1_MOUSE	TUBA3	(P68369) Tubulin alpha-1 chain (Alpha-tubulin 1) (Alpha-tubulin isotype M-alpha-1)	12	31	451	50136	5.1	Cytoplasm	other	NONE	0.001419
P68372	TBB2C_MOUSE	TUBB2C	(P68372) Tubulin beta-2C chain	34	52.6	445	49831	4.9	Cytoplasm	other	NONE	0.004427
P68433	H31_MOUSE	HIST1H3B (includes EG:8358)	(P68433) Histone H3.1	6	45.9	135	15273	11.1	Nucleus	other	NONE	0.004532
P68510	1433F_MOUSE	YWHAH	(P68510) 14-3-3 protein eta	2	9	245	28081	4.9	Cytoplasm	transcription regulator	NONE	0.000115
P70158	ASM3A_MOUSE	SMPDL3A	(P70158) Acid sphingomyelinase-like phosphodiesterase 3a precursor (EC 3.1.4.-) (ASM-like phosphodiesterase 3a)	5	15.5	445	49843	6.3	Extracellular Space	enzyme	1	0.000189
P70168	IMB1_MOUSE	KPNB1	(P70168) Importin beta-1 subunit (Karyopherin beta-1 subunit) (Nuclear factor P97) (Pore targeting complex 97 kDa subunit) (PTAC97) (SCG)	2	3.5	876	97152	4.8	Nucleus	transporter	NONE	2.56E-05
P70255	NFIC_MOUSE	NFIC	(P70255) Nuclear factor 1 C-type (Nuclear factor 1/C) (NF1-C) (NFI-C) (NF-I/C) (CCAAT-box-binding transcription factor) (CTF) (TGGCA-binding protein)	1	7.3	439	48768	8.3	Nucleus	transcription regulator	NONE	1.28E-05
P70288	HDAC2_MOUSE	HDAC2 (includes EG:3066)	(P70288) Histone deacetylase 2 (HD2) (YY1 transcription factor-binding protein)	1	3.9	488	55302	5.9	Nucleus	transcription regulator	NONE	1.15E-05
P70290	EM55_MOUSE	MPP1	(P70290) 55 kDa erythrocyte membrane protein (p55) (Membrane protein, palmitoylated 1) (Palmitoylated protein p55)	18	36.1	466	52227	7.2	Plasma Membrane	kinase	NONE	0.000542
P70296	PEBP1_MOUSE	PEBP1	(P70296) Phosphatidylethanolamine-binding protein 1 (PEBP-1) (HCNPpp) [Contains: Hippocampal cholinergic neurostimulating peptide (HCNP)]	11	54.8	186	20699	5.4	Cytoplasm	other	NONE	0.001539
P70297	STAM1_MOUSE	STAM (includes EG:8027)	(P70297) Signal transducing adapter molecule 1 (STAM-1)	1	2.6	547	59640	4.8	Cytoplasm	other	NONE	3.08E-05
P70333	HNRH2_MOUSE	HNRPH2	(P70333) Heterogeneous nuclear ribonucleoprotein H' (hnRNP H')	4	8.5	449	49280	6.3	Nucleus	other	NONE	0.000213
P70336	ROCK2_MOUSE	ROCK2	(P70336) Rho-associated protein kinase 2 (EC 2.7.11.1) (Rho-associated, coiled-coil-containing protein kinase 2) (p164 ROCK-2)	1	1.2	1388	160585	6	Cytoplasm	kinase	NONE	4.04E-06

P70349	HINT1_MOUSE	HINT1 (includes EG:3094)	(P70349) Histidine triad nucleotide-binding protein 1 (Adenosine 5'-monophosphoramidase) (Protein kinase C inhibitor 1) (Protein kinase C-interacting protein 1) (PKCI-1)	4	50.4	125	13646	6.9	Nucleus	enzyme	NONE	0.000629
P70372	ELAV1_MOUSE	ELAVL1	(P70372) ELAV-like protein 1 (Hu-antigen R) (HuR) (Elav-like generic protein) (MelG)	4	19.6	326	36069	9.2	Cytoplasm	other	NONE	0.000103
P70387	HFE_MOUSE	HFE	(P70387) Hereditary hemochromatosis protein homolog precursor	1	5.3	359	40548	6.2	Plasma Membrane	transmembrane receptor	2	3.13E-05
P70398	USP9X_MOUSE	USP9X	(P70398) Probable ubiquitin carboxyl-terminal hydrolase FAF-X (EC 3.1.2.15) (Ubiquitin thioesterase FAF-X) (Ubiquitin-specific-processing protease FAF-X) (Deubiquitinating enzyme FAF-X) (Fat facets protein-related, X-linked) (Ubiquitin-specif	2	1.3	2559	290543	6	Plasma Membrane	peptidase	NONE	6.58E-06
P70404	IDH3G_MOUSE	IDH3G	(P70404) Isocitrate dehydrogenase [NAD] subunit gamma, mitochondrial precursor (EC 1.1.1.41) (Isocitric dehydrogenase) (NAD(+)-specific ICDH)	6	19.1	393	42785	9	Cytoplasm	enzyme	NONE	0.000229
P70414	NAC1_MOUSE	SLC8A1	(P70414) Sodium/calcium exchanger 1 precursor (Na(+)/Ca(2+)-exchange protein 1)	3	4.4	970	108035	5	Plasma Membrane	transporter	11	4.05E-05
P70419	GALT3_MOUSE	GALNT3	(P70419) Polypeptide N-acetylgalactosaminyltransferase 3 (EC 2.4.1.41) (Protein-UDP acetylgalactosaminyltransferase 3) (UDP-GalNAc:polypeptide N-acetylgalactosaminyltransferase 3) (Polypeptide GalNAc transferase 3) (GalNAc-T3) (pp-GaNTase 3)	3	5.1	633	72959	8.3	Cytoplasm	enzyme	1	3.55E-05
P70425	RIT2_MOUSE	RIT2	(P70425) GTP-binding protein Rit2 (Ras-like protein expressed in neurons) (Ras-like without CAAX protein 2)	1	5.1	217	24802	6.9	Plasma Membrane	enzyme	NONE	2.59E-05
P70441	NHERF_MOUSE	SLC9A3R1	(P70441) Ezrin-radixin-moesin-binding phosphoprotein 50 (EBP50) (Na(+)/H(+) exchange regulatory cofactor NHE-RF) (NHERF-1) (Regulatory cofactor of Na(+)/H(+) exchanger) (Sodium-hydrogen exchanger regulatory factor 1) (Solute carrier family 9	26	73.7	354	38469	5.9	Plasma Membrane	other	NONE	0.00195
P70444	BID_MOUSE	BID	(P70444) BH3-interacting domain death agonist (BID) (p22 BID) [Contains: BH3-interacting domain death agonist p15 (p15 BID); BH3-interacting domain death agonist p13 (p13 BID); BH3-interacting domain death agonist p11 (p11 BID)]	4	31.3	195	21952	4.8	Cytoplasm	other	NONE	0.000288
P70452	STX4_MOUSE	STX4	(P70452) Syntaxin-4	1	6.4	298	34165	6.1	Plasma Membrane	transporter	1	0.000132
P70460	VASP_MOUSE	VASP	(P70460) Vasodilator-stimulated phosphoprotein (VASP)	4	11.2	374	39535	8.5	Plasma Membrane	other	NONE	0.000105
P70670	NACAM_MOUSE	NACA	(P70670) Nascent polypeptide-associated complex subunit alpha, muscle-specific form (Alpha-NAC, muscle-specific form)	7	2.6	2187	220599	9.4	Nucleus	transcription regulator	NONE	3.59E-05
P70694	DHB5_MOUSE	AKR1C2	(P70694) Estradiol 17 beta-dehydrogenase 5 (EC 1.1.1.-) (17-beta-HSD 5)	1	3.4	323	37048	8.3	Cytoplasm	enzyme	NONE	3.48E-05
P70695	F16P2_MOUSE	FBP2	(P70695) Fructose-1,6-bisphosphatase isozyme 2 (EC 3.1.3.11) (D-fructose-1,6-bisphosphate 1-phosphohydrolase 2) (FBPase 2) (RAE-30)	2	11.5	339	36947	6.2	Cytoplasm	phosphatase	NONE	3.31E-05
P70696	H2B1A_MOUSE	HIST1H2BA	(P70696) Histone H2B type 1-A (Histone H2B, testis) (Testis-specific histone H2B)	6	46.8	126	14105	10.3	Nucleus	other	NONE	0.003208
P70699	LYAG_MOUSE	GAA	(P70699) Lysosomal alpha-glucosidase precursor (EC 3.2.1.20) (Acid maltase)	1	1.9	953	106248	5.8	Cytoplasm	enzyme	NONE	2.36E-05
P80314	TCPB_MOUSE	CCT2	(P80314) T-complex protein 1 subunit beta (TCP-1-beta) (CCT-beta)	5	11	534	57346	6.4	Cytoplasm	kinase	NONE	8.41E-05
P80315	TCPD_MOUSE	CCT4	(P80315) T-complex protein 1 subunit delta (TCP-1-delta) (CCT-delta) (A45)	7	14.3	538	57935	8	Cytoplasm	other	NONE	0.000136
P80316	TCPE_MOUSE	CCT5	(P80316) T-complex protein 1 subunit epsilon (TCP-1-epsilon) (CCT-epsilon)	7	14.2	541	59624	6	Cytoplasm	other	NONE	0.000135
P80317	TCPZ_MOUSE	CCT6A	(P80317) T-complex protein 1 subunit zeta (TCP-1-zeta) (CCT-zeta) (CCT-zeta-1)	6	19.4	530	57873	7.1	Cytoplasm	other	NONE	0.00018
P80318	TCPG_MOUSE	CCT3	(P80318) T-complex protein 1 subunit gamma (TCP-1-gamma) (CCT-gamma) (Matricin) (mTRiC-P5)	4	11.2	545	60630	6.7	Cytoplasm	other	NONE	7.21E-05
P81117	NUCB2_MOUSE	NUCB2	(P81117) Nucleobindin-2 precursor (DNA-binding protein NEFA)	1	5	420	50305	5.1	Nucleus	other	NONE	2.67E-05
P81269	ATF1_MOUSE	ATF1	(P81269) Cyclic AMP-dependent transcription factor ATF-1 (Activating transcription factor 1) (TCR-ATF1)	1	9.7	269	29238	8.4	Nucleus	transcription regulator	NONE	2.09E-05

P83917	CBX1_MOUSE	CBX1	(P83917) Chromobox protein homolog 1 (Heterochromatin protein 1 homolog beta) (HP1 beta) (Modifier 1 protein) (M31) (Heterochromatin protein p25)	3	18.4	185	21418	4.9	Nucleus	other	NONE	0.001578
P83940	ELOC_MOUSE	TCEB1	(P83940) Transcription elongation factor B polypeptide 1 (RNA polymerase II transcription factor SIII subunit C) (SIII p15) (Elongin-C) (EloC) (Elongin 15 kDa subunit) (Stromal membrane-associated protein SMAP1B homolog)	1	17.9	112	12473	4.8	Nucleus	transcription regulator	NONE	0.0002
P84078	ARF1_MOUSE	ARF1	(P84078) ADP-ribosylation factor 1	2	10	180	20566	6.8	Cytoplasm	transporter	NONE	9.36E-05
P84089	ERH_MOUSE	ERH (includes EG:2079)	(P84089) Enhancer of rudimentary homolog	2	16.3	104	12259	5.9	Nucleus	other	NONE	0.000378
P84091	AP2M1_MOUSE	AP2M1	(P84091) AP-2 complex subunit mu-1 (Adaptin mu-1) (AP-2 mu-2 chain) (Clathrin coat assembly protein AP50) (Clathrin coat-associated protein AP50) (Plasma membrane adaptor AP-2 50 kDa protein) (Clathrin assembly protein complex 2 medium chain)	5	7.4	435	49655	9.5	Cytoplasm	transporter	NONE	0.000206
P84096	RHOG_MOUSE	RHOG	(P84096) Rho-related GTP-binding protein RhoG precursor (Sid 10750)	1	9.9	191	21308	8.1	Cytoplasm	enzyme	NONE	8.82E-05
P84099	RL19_MOUSE	RPL19 (includes EG:19921)	(P84099) 60S ribosomal protein L19	7	19.9	196	23466	11.5	Nucleus	other	NONE	0.000659
P84104	SFRS3_MOUSE	SFRS3	(P84104) Splicing factor, arginine/serine-rich 3 (Pre-mRNA-splicing factor SRP20) (X16 protein)	4	18.3	164	19330	11.6	Nucleus	other	NONE	0.000411
P84309	ADCY5_MOUSE	ADCY5	(P84309) Adenylate cyclase type 5 (EC 4.6.1.1) (Adenylate cyclase type V) (ATP pyrophosphate-lyase 5) (Adenylyl cyclase 5) (Fragment)	1	2.9	795	89334	6.4	Plasma Membrane	enzyme	6	2.82E-05
P97287	MCL1_MOUSE	MCL1	(P97287) Induced myeloid leukemia cell differentiation protein Mcl-1 homolog (Bcl-2-related protein EAT/mcl1)	1	7.3	331	35217	6.2	Cytoplasm	transporter	3	1.7E-05
P97298	PEDF_MOUSE	SERPINF1	(P97298) Pigment epithelium-derived factor precursor (PEDF) (Stromal cell-derived factor 3) (SDF-3) (Caspin)	2	10.1	417	46234	7	Extracellular Space	other	NONE	6.73E-05
P97311	MCM6_MOUSE	MCM6	(P97311) DNA replication licensing factor MCM6 (Mis5 homolog)	2	4.8	821	92867	5.5	Nucleus	enzyme	NONE	1.37E-05
P97314	CSRP2_MOUSE	CSRP2	(P97314) Cysteine and glycine-rich protein 2 (Cysteine-rich protein 2) (CRP2) (Double LIM protein 1) (DLP-1)	3	30.2	192	20795	8.6	Nucleus	other	NONE	0.000322
P97315	CSRP1_MOUSE	CSRP1	(P97315) Cysteine and glycine-rich protein 1 (Cysteine-rich protein 1) (CRP1) (CRP)	5	34.4	192	20452	8.6	Nucleus	other	NONE	0.000614
P97325	SIAT6_MOUSE	ST3GAL3	(P97325) CMP-N-acetylneuraminate-beta-1,4-galactoside alpha-2,3-sialyltransferase (EC 2.4.99.6) (N-acetyllactosaminide alpha-2,3-sialyltransferase) (Gal beta-1,3(4) GlcNAc alpha-2,3 sialyltransferase) (ST3N) (ST3GalIII) (Sialyltransferase 6)	1	4.3	374	42131	9.1	Cytoplasm	enzyme	1	1.5E-05
P97328	KHK_MOUSE	KHK	(P97328) Ketohexokinase (EC 2.7.1.3) (Hepatic fructokinase)	7	27.9	298	32750	6.2	Cytoplasm	kinase	NONE	0.004596
P97333	NRP1_MOUSE	NRP1	(P97333) Neuropilin-1 precursor (A5 protein)	1	2.5	923	103020	6	Plasma Membrane	transmembrane receptor	2	1.22E-05
P97351	RS3A_MOUSE	RPS3A	(P97351) 40S ribosomal protein S3a	7	26.2	263	29754	9.7	Cytoplasm	other	NONE	0.002134
P97371	PSME1_MOUSE	PSME1	(P97371) Proteasome activator complex subunit 1 (Proteasome activator 28-alpha subunit) (PA28alpha) (PA28a) (Activator of multicatalytic protease subunit 1) (11S regulator complex subunit alpha) (REG-alpha)	5	32.9	249	28673	6	Cytoplasm	other	NONE	0.000203
P97379	G3B2_MOUSE	G3BP2	(P97379) Ras-GTPase-activating protein-binding protein 2 (GAP SH3-domain-binding protein 2) (G3BP-2)	2	7.3	482	54088	5.6	Nucleus	enzyme	NONE	6.99E-05
P97384	ANX11_MOUSE	ANXA11	(P97384) Annexin A11 (Annexin XI) (Calcyclin-associated annexin 50) (CAP-50)	1	3.2	503	54111	7.7	Nucleus	other	NONE	1.12E-05
P97412	LYST_MOUSE	LYST	(P97412) Lysosomal-trafficking regulator (Beige protein) (CHS1 homolog)	2	0.8	3788	425289	6.6	Cytoplasm	transporter	NONE	4.45E-06
P97429	ANXA4_MOUSE	ANXA4	(P97429) Annexin A4 (Annexin IV)	8	27.4	318	35859	5.6	Plasma Membrane	other	NONE	0.000406
P97431	IRF6_MOUSE	IRF6	(P97431) Interferon regulatory factor 6 (IRF-6)	1	4.3	467	53107	5.3	Nucleus	transcription regulator	NONE	2.4E-05

P97434	MRIP_MOUSE	M-RIP	(P97434) Myosin phosphatase Rho-interacting protein (Rho-interacting protein 3) (p116Rip) (RIP3)	3	6	1024	116408	6.2	Cytoplasm	other	NONE	2.74E-05
P97447	FHL1_MOUSE	FHL1	(P97447) Four and a half LIM domains protein 1 (FHL-1) (Skeletal muscle LIM-protein 1) (SLIM 1) (SLIM) (KyoT) (RBP-associated molecule 14-1) (RAM14-1)	8	40	280	31889	8.4	Cytoplasm	other	NONE	0.000401
P97449	AMPN_MOUSE	ANPEP	(P97449) Aminopeptidase N (EC 3.4.11.2) (mAPN) (Alanyl aminopeptidase) (Microsomal aminopeptidase) (Aminopeptidase M) (Membrane protein p161) (CD13 antigen)	27	30.8	965	109520	5.9	Plasma Membrane	peptidase	1	0.000861
P97450	ATP5J_MOUSE	ATP5J	(P97450) ATP synthase coupling factor 6, mitochondrial precursor (EC 3.6.3.14) (ATPase subunit F6)	18	42.6	108	12496	9.4	Cytoplasm	transporter	NONE	0.003846
P97461	RS5_MOUSE	RPS5	(P97461) 40S ribosomal protein S5	2	10.8	203	22758	9.7	Cytoplasm	other	NONE	0.00177
P97480	EYA3_MOUSE	EYA3	(P97480) Eyes absent homolog 3 (EC 3.1.3.48)	1	4.9	510	55945	5.2	Nucleus	phosphatase	NONE	2.2E-05
P97493	THIOM_MOUSE	TXN2	(P97493) Thioredoxin, mitochondrial precursor (Mt-Trx) (MTRX) (Thioredoxin-2)	2	13.9	166	18255	7.9	Cytoplasm	enzyme	NONE	0.000135
P97765	WBP2_MOUSE	WBP2	(P97765) WW domain-binding protein 2 (WBP-2)	1	5.4	261	28032	6.3	Cytoplasm	other	NONE	4.3E-05
P97798	NEO1_MOUSE	NEO1	(P97798) Neogenin precursor	1	1.2	1493	163159	6.5	Plasma Membrane	transcription regulator	2	7.52E-06
P97807	FUMH_MOUSE	FH	(P97807) Fumarate hydratase, mitochondrial precursor (EC 4.2.1.2) (Fumarase) (EF-3)	15	35.5	507	54371	9	Cytoplasm	enzyme	NONE	0.001196
P97816	S100G_MOUSE	S100G	(P97816) Protein S100-G (S100 calcium-binding protein G) (Vitamin D-dependent calcium-binding protein, intestinal) (CABP) (Calbindin D9K)	2	37.2	78	8839	4.8	Cytoplasm	other	NONE	0.001079
P97822	AN32E_MOUSE	ANP32E	(P97822) Acidic leucine-rich nuclear phosphoprotein 32 family member E (LANP-like protein) (LANP-L) (Cerebellar postnatal development protein 1)	2	5.4	260	29622	3.9	Unknown	other	NONE	6.48E-05
P97855	G3BP_MOUSE	G3BP1	(P97855) Ras-GTPase-activating protein-binding protein 1 (EC 3.6.1.-) (ATP-dependent DNA helicase VIII) (GAP SH3-domain-binding protein 1) (G3BP-1) (HDH-VIII)	10	29.9	465	51829	5.6	Nucleus	enzyme	NONE	0.000338
P97863	NFIB_MOUSE	NFIB	(P97863) Nuclear factor 1 B-type (Nuclear factor 1/B) (NF1-B) (NFI-B) (NF-I/B) (CCAAT-box-binding transcription factor) (CTF) (TGGCA-binding protein)	2	4.4	570	63507	8.7	Nucleus	transcription regulator	NONE	2.95E-05
P97927	LAMA4_MOUSE	LAMA4 (includes EG:3910)	(P97927) Laminin alpha-4 chain precursor	2	2	1816	201818	6.2	Extracellular Space	enzyme	1	6.18E-06
P98078	DAB2_MOUSE	DAB2	(P98078) Disabled homolog 2 (DOC-2) (Mitogen-responsive phosphoprotein)	15	21.4	766	82312	6.1	Plasma Membrane	other	NONE	0.000337
P99024	TBB5_MOUSE	TUBB	(P99024) Tubulin beta-5 chain	3	12.2	444	49671	4.9	Cytoplasm	other	NONE	7.59E-05
P99026	PSB4_MOUSE	PSMB4	(P99026) Proteasome subunit beta type 4 precursor (EC 3.4.25.1) (Proteasome beta chain) (Macropain beta chain) (Multicatalytic endopeptidase complex beta chain) (Proteasome chain 3)	5	29.2	264	29116	5.7	Cytoplasm	peptidase	NONE	0.000213
P99027	RLA2_MOUSE	RPLP2	(P99027) 60S acidic ribosomal protein P2	16	83.5	115	11651	4.5	Cytoplasm	other	NONE	0.004832
P99028	UCRH_MOUSE	UQCRH	(P99028) Ubiquinol-cytochrome c reductase complex 11 kDa protein, mitochondrial precursor (EC 1.10.2.2) (Mitochondrial hinge protein) (Cytochrome C1, nonheme 11 kDa protein) (Complex III subunit VIII)	6	60.7	89	10435	4.9	Cytoplasm	enzyme	NONE	0.001703
P99029	PRDX5_MOUSE	PRDX5	(P99029) Peroxiredoxin-5, mitochondrial precursor (EC 1.11.1.15) (Prx-V) (Peroxisomal antioxidant enzyme) (PLP) (Thioredoxin reductase) (Thioredoxin peroxidase PMP20) (Antioxidant enzyme B166) (AOEB166) (Liver tissue 2D-page spot 2D-0014IV)	20	48.6	210	21897	8.9	Cytoplasm	enzyme	NONE	0.009034
Q00612	G6PD1_MOUSE	G6PD	(Q00612) Glucose-6-phosphate 1-dehydrogenase X (EC 1.1.1.49) (G6PD)	2	4.9	514	59131	6.5	Cytoplasm	enzyme	NONE	2.18E-05
Q00623	APOA1_MOUSE	APOA1	(Q00623) Apolipoprotein A-I precursor (Apo-AI) (ApoA-I)	9	16.7	264	30587	5.9	Extracellular Space	transporter	1	0.000383
Q00724	RETBP_MOUSE	RBP4	(Q00724) Plasma retinol-binding protein precursor (PRBP) (RBP)	1	5	201	23206	6	Extracellular Space	transporter	NONE	5.59E-05
Q00898	A1AT5_MOUSE	SERPINA1	(Q00898) Alpha-1-antitrypsin 1-5 precursor (Serine protease inhibitor 1-5) (Alpha-1 protease inhibitor 5)	2	6.3	413	45891	5.7	Extracellular Space	other	2	5.44E-05
Q01147	CREB1_MOUSE	CREB1	(Q01147) cAMP response element-binding protein (CREB)	1	8.5	341	36674	5.6	Nucleus	transcription regulator	NONE	3.29E-05

Q01279	EGFR_MOUSE	EGFR	(Q01279) Epidermal growth factor receptor precursor (EC 2.7.10.1)	1	2.6	1210	134853	6.9	Plasma Membrane	kinase	2	4.64E-06
Q01405	SC23A_MOUSE	SEC23A	(Q01405) Protein transport protein Sec23A (SEC23-related protein A)	1	2.6	765	86162	7.1	Cytoplasm	transporter	NONE	7.34E-06
Q01730	RSU1_MOUSE	RSU1 (includes EG:6251)	(Q01730) Ras suppressor protein 1 (Rsu-1) (RSP-1)	3	14.9	276	31419	8.9	Cytoplasm	other	NONE	0.000183
Q01768	NDKB_MOUSE	NME2	(Q01768) Nucleoside diphosphate kinase B (EC 2.7.4.6) (NDK B) (NDP kinase B) (nm23-M2) (P18)	12	47.4	152	17363	7.5	Nucleus	kinase	NONE	0.004025
Q01853	TERA_MOUSE	VCP	(Q01853) Transitional endoplasmic reticulum ATPase (TER ATPase) (15S Mg(2+)-ATPase p97 subunit) (Valosin-containing protein) (VCP)	9	13.5	805	89177	5.3	Cytoplasm	enzyme	NONE	0.000119
Q02013	AQP1_MOUSE	AQP1	(Q02013) Aquaporin-1 (AQP-1) (Aquaporin-CHIP) (Water channel protein for red blood cells and kidney proximal tubule) (Early response protein DER2)	1	7.5	268	28662	7.4	Plasma Membrane	transporter	5	0.000105
Q02053	UBE1_MOUSE	UBE1	(Q02053) Ubiquitin-activating enzyme E1 1	5	6.7	1058	117809	5.7	Cytoplasm	enzyme	2	3.71E-05
Q02780	NFIA_MOUSE	NFIA	(Q02780) Nuclear factor 1 A-type (Nuclear factor 1/A) (NF1-A) (NFI-A) (NF-I/A) (CCAAT-box-binding transcription factor) (CTF) (TGGCA-binding protein)	1	2.6	532	58553	8.6	Nucleus	transcription regulator	NONE	2.11E-05
Q02788	CO6A2_MOUSE	COL6A2	(Q02788) Collagen alpha-2(VI) chain precursor	2	3.7	1029	109812	6.3	Extracellular Space	other	1	1.64E-05
Q02819	NUCB1_MOUSE	NUCB1	(Q02819) Nucleobindin-1 precursor (CALNUC)	18	36.8	459	53409	5.1	Cytoplasm	other	NONE	0.000416
Q03141	MARK3_MOUSE	MARK3	(Q03141) MAP/microtubule affinity-regulating kinase 3 (EC 2.7.11.1) (MPK-10) (ELKL motif kinase 2)	1	2.5	753	84390	9.5	Cytoplasm	kinase	NONE	1.49E-05
Q03173	ENAH_MOUSE	ENAH	(Q03173) Protein enabled homolog (NPC-derived proline-rich protein 1) (NDPP-1)	2	4	802	85844	7.7	Cytoplasm	other	NONE	1.4E-05
Q03265	ATPA_MOUSE	ATP5A1	(Q03265) ATP synthase alpha chain, mitochondrial precursor (EC 3.6.3.14)	44	49.7	553	59753	9.2	Cytoplasm	transporter	NONE	0.006445
Q03734	SPA3M_MOUSE	SERPINA3M	(Q03734) Serine protease inhibitor A3M precursor (Serpin A3M)	1	5	418	47004	5.5	Extracellular Space	other	1	4.03E-05
Q04646	ATNG_MOUSE	FXYD2	(Q04646) Sodium/potassium-transporting ATPase gamma chain (Sodium pump gamma chain) (Na+/K+ ATPase subunit gamma) (FXYD domain-containing ion transport regulator 2)	3	45.7	70	7520	7.2	Plasma Membrane	ion channel	1	0.001203
Q04750	TOP1_MOUSE	TOP1	(Q04750) DNA topoisomerase 1 (EC 5.99.1.2) (DNA topoisomerase I)	4	6.9	767	90790	9.3	Nucleus	enzyme	NONE	5.85E-05
Q04857	CO6A1_MOUSE	COL6A1	(Q04857) Collagen alpha-1(VI) chain precursor	4	5.5	1025	108489	5.4	Extracellular Space	other	NONE	4.38E-05
Q05186	RCN1_MOUSE	RCN1	(Q05186) Reticulocalbin-1 precursor	1	4.6	325	38113	4.8	Cytoplasm	other	1	1.73E-05
Q05512	MARK2_MOUSE	MARK2	(Q05512) Serine/threonine-protein kinase MARK2 (EC 2.7.11.1) (MAP/microtubule affinity-regulating kinase 2) (ELKL Motif Kinase) (EMK1)	1	2.6	776	86382	9.7	Cytoplasm	kinase	NONE	1.45E-05
Q05793	PGBM_MOUSE	HSPG2 (includes EG:3339)	(Q05793) Basement membrane-specific heparan sulfate proteoglycan core protein precursor (HSPG) (Perlecan) (PLC)	23	10.4	3707	398295	6.3	Plasma Membrane	other	NONE	5.3E-05
Q05920	PYC_MOUSE	PC	(Q05920) Pyruvate carboxylase, mitochondrial precursor (EC 6.4.1.1) (Pyruvic carboxylase) (PCB)	27	26.8	1178	129685	6.7	Cytoplasm	enzyme	NONE	0.000381
Q06185	ATP5I_MOUSE	ATP5I	(Q06185) ATP synthase e chain, mitochondrial (EC 3.6.3.14)	2	34.3	70	8104	9.4	Cytoplasm	transporter	NONE	0.000481
Q06890	CLUS_MOUSE	CLU	(Q06890) Clusterin precursor (Sulfated glycoprotein 2) (SGP-2) (Clustrin) (Apolipoprotein J) (Apo-J) [Contains: Clusterin beta chain; Clusterin alpha chain]	2	6	448	51656	5.7	Extracellular Space	other	1	5.01E-05
Q07076	ANXA7_MOUSE	ANXA7	(Q07076) Annexin A7 (Annexin VII) (Synexin)	1	3.5	463	49939	6.2	Plasma Membrane	ion channel	NONE	2.42E-05
Q07113	MPRI_MOUSE	IGF2R	(Q07113) Cation-independent mannose-6-phosphate receptor precursor (CI Man-6-P receptor) (CI-MPR) (M6PR) (Insulin-like growth factor 2 receptor) (Insulin-like growth factor II receptor) (IGF-II receptor) (M6P/IGF2 receptor) (M6P/IGF2R) (300 kD	1	0.8	2483	273814	5.7	Plasma Membrane	transmembrane receptor	4	4.52E-06
Q07417	ACADS_MOUSE	ACADS	(Q07417) Short-chain specific acyl-CoA dehydrogenase, mitochondrial precursor (EC 1.3.99.2) (SCAD) (Butyryl-CoA dehydrogenase)	7	17	412	44947	8.8	Cytoplasm	enzyme	NONE	0.0003
Q07813	BAXA_MOUSE	BAX	(Q07813) Apoptosis regulator BAX, membrane isoform alpha	4	17.7	192	21395	5	Cytoplasm	other	NONE	0.000292
Q08091	CNN1_MOUSE	CNN1	(Q08091) Calponin-1 (Calponin H1, smooth muscle) (Basic calponin)	2	6.7	297	33356	9	Cytoplasm	other	NONE	7.56E-05
Q08093	CNN2_MOUSE	CNN2	(Q08093) Calponin-2 (Calponin H2, smooth muscle) (Neutral calponin)	3	15.4	305	33156	7.6	Cytoplasm	other	NONE	0.000147

Q08481	PECA1_MOUSE	PECAM1	(Q08481) Platelet endothelial cell adhesion molecule precursor (PECAM-1) (CD31 antigen)	1	3	727	81263	6.7	Plasma Membrane	other	2	7.72E-06
Q08509	EPS8_MOUSE	EPS8	(Q08509) Epidermal growth factor receptor kinase substrate 8	2	3.9	821	91738	7.5	Plasma Membrane	peptidase	NONE	1.37E-05
Q08857	CD36_MOUSE	CD36	(Q08857) Platelet glycoprotein 4 (Platelet glycoprotein IV) (GPIV) (GPIIIB) (CD36 antigen) (PAS IV) (PAS-4 protein)	1	3.6	471	52567	8.3	Plasma Membrane	other	3	3.58E-05
Q09324	GCNT1_MOUSE	GCNT1	(Q09324) Beta-1,3-galactosyl-O-glycosyl-glycoprotein beta-1,6-N-acetylglucosaminyltransferase (EC 2.4.1.102) (Core 2 branching enzyme) (Core2-GlcNAc-transferase) (C2GNT)	2	8.2	428	49850	8.2	Cytoplasm	enzyme	1	3.93E-05
Q3TCJ1	K0157_MOUSE	KIAA0157	(Q3TCJ1) Protein KIAA0157	2	7	415	46943	6.2	Unknown	other	NONE	4.06E-05
Q3TGF2	F107B_MOUSE	FAM107B	(Q3TGF2) Protein FAM107B	1	9.9	131	15572	8.3	Unknown	other	NONE	4.28E-05
Q3THS6	METK2_MOUSE	MAT2A	(Q3THS6) S-adenosylmethionine synthetase isoform type-2 (EC 2.5.1.6) (Methionine adenosyltransferase 2) (AdoMet synthetase 2)	4	9.9	395	43689	6.5	Cytoplasm	enzyme	NONE	0.000114
Q3THW5	H2AV_MOUSE	H2AFV	(Q3THW5) Histone H2AV (H2A.F/Z)	4	24.4	127	13377	10.6	Nucleus	other	NONE	0.007514
Q3TNA1	XYLB_MOUSE	XYLB	(Q3TNA1) Xylulose kinase (EC 2.7.1.17) (Xylulokinase)	5	12.3	551	59544	6.9	Unknown	kinase	NONE	0.000143
Q3TQI7	CI078_MOUSE	C9ORF78	(Q3TQI7) Protein C9orf78 homolog	2	9.7	289	33445	6.1	Unknown	other	NONE	3.88E-05
Q3U319	BRE1B_MOUSE	RNF40	(Q3U319) Ubiquitin-protein ligase BRE1B (EC 6.3.2.-) (BRE1-B) (RING finger protein 40)	4	5.1	1001	113967	6.5	Cytoplasm	enzyme	NONE	2.8E-05
Q3U5C8	ARHGG_MOUSE	ARHGEF16	(Q3U5C8) Rho guanine nucleotide exchange factor 16	1	5.5	713	80395	7.5	Cytoplasm	other	NONE	1.57E-05
Q3UEI1	PDE4C_MOUSE	PDE4C	(Q3UEI1) cAMP-specific 3',5'-cyclic phosphodiesterase 4C (EC 3.1.4.17)	2	3.8	686	76090	5	Cytoplasm	enzyme	NONE	1.64E-05
Q3UFF7	LYPL1_MOUSE	LYPLAL1	(Q3UFF7) Lysophospholipase-like protein 1 (EC 3.1.2.-)	2	16	238	26223	7.9	Cytoplasm	enzyme	NONE	7.08E-05
Q3UHX2	HAP28_MOUSE	PDAP1	(Q3UHX2) 28 kDa heat- and acid-stable phosphoprotein (PDGF-associated protein) (PAP) (PDGFA-associated protein 1) (PAP1)	5	26.5	181	20605	7.4	Cytoplasm	other	NONE	0.000155
Q3UIU2	NDUB6_MOUSE	NDUFB6	(Q3UIU2) NADH dehydrogenase [ubiquinone] 1 beta subcomplex subunit 6 (EC 1.6.5.3) (EC 1.6.99.3) (NADH-ubiquinone oxidoreductase B17 subunit) (Complex I-B17) (CI-B17)	3	19.7	127	15384	9.8	Cytoplasm	enzyme	NONE	0.000309
Q3UM45	PP1R7_MOUSE	PPP1R7	(Q3UM45) Protein phosphatase 1 regulatory subunit 7 (Protein phosphatase 1 regulatory subunit 22)	2	8	361	41292	4.9	Nucleus	phosphatase	NONE	7.77E-05
Q3UVR3	TTBK2_MOUSE	TTBK2	(Q3UVR3) Tau-tubulin kinase 2	2	1.9	1243	136770	7.2	Unknown	kinase	NONE	9.03E-06
Q3V1T4	P3H1_MOUSE	LEPRE1	(Q3V1T4) Prolyl 3-hydroxylase 1 precursor (EC 1.14.11.7) (Leucine proline-enriched proteoglycan 1) (Leprecan-1) (Growth suppressor 1)	1	3.2	739	83651	5.1	Nucleus	enzyme	1	7.6E-06
Q4FZG7	TI8AB_MOUSE	TIMM8A2	(Q4FZG7) Putative mitochondrial import inner membrane translocase subunit Tim8 A-B	1	11.3	97	11283	6.5	Unknown	other	NONE	0.000347
Q4KMM3	OXR1_MOUSE	OXR1	(Q4KMM3) Oxidation resistance protein 1 (Protein C7)	1	2.3	751	83016	5	Cytoplasm	other	NONE	2.24E-05
Q4U2R1	HERC2_MOUSE	HERC2	(Q4U2R1) HECT domain and RCC1-like domain-containing protein 2	2	0.5	4836	527536	6.2	Cytoplasm	enzyme	NONE	9.29E-06
Q501J6	DDX17_MOUSE	DDX17	(Q501J6) Probable ATP-dependent RNA helicase DDX17 (EC 3.6.1.-) (DEAD box protein 17)	4	5.7	650	72400	8.6	Nucleus	enzyme	NONE	6.04E-05
Q505F5	LRC47_MOUSE	LRRC47	(Q505F5) Leucine-rich repeat-containing protein 47	1	3.1	581	63590	8.1	Unknown	other	NONE	9.66E-06
Q52KI8	SRRM1_MOUSE	SRRM1	(Q52KI8) Serine/arginine repetitive matrix protein 1 (Plenty-of-prolines 101)	2	3.6	946	106892	11.9	Nucleus	other	NONE	2.37E-05
Q569Z5	DDX46_MOUSE	DDX46	(Q569Z5) Probable ATP-dependent RNA helicase DDX46 (EC 3.6.1.-) (DEAD box protein 46)	6	6.2	1032	117448	9.3	Nucleus	enzyme	NONE	5.44E-05
Q569Z6	TR150_MOUSE	THRAP3	(Q569Z6) Thyroid hormone receptor-associated protein 3 (Thyroid hormone receptor-associated protein complex 150 kDa component) (Trap150)	5	8.5	951	108178	10.2	Nucleus	transcription regulator	NONE	5.9E-05
Q58A65	JIP4_MOUSE	SPAG9	(Q58A65) C-jun-amino-terminal kinase-interacting protein 4 (JNK-interacting protein 4) (JIP-4) (JNK-associated leucine-zipper protein) (JLP) (Sperm-associated antigen 9) (Mitogen-activated protein kinase 8-interacting protein 4) (JNK/SAPK-asso	9	11.7	1321	146219	5.1	Plasma Membrane	other	NONE	8.07E-05
Q59J78	MIMIT_MOUSE	NDUFA12L	(Q59J78) Mimitin, mitochondrial precursor (Myc-induced mitochondrial protein) (MMTN)	3	33.3	168	19628	8.2	Cytoplasm	other	NONE	0.000134

Q5DTM8	BRE1A_MOUSE	RNF20	(Q5DTM8) Ubiquitin-protein ligase BRE1A (EC 6.3.2.-) (BRE1-A) (RING finger protein 20)	1	1.7	973	113520	6	Nucleus	other	NONE	1.15E-05
Q5ND29	RILP_MOUSE	RILP	(Q5ND29) Rab-interacting lysosomal protein	1	5.4	369	41139	5.3	Cytoplasm	other	NONE	3.04E-05
Q5PR68	CCD46_MOUSE	CCDC46	(Q5PR68) Coiled coil domain-containing protein 46	2	4	954	112676	6.8	Unknown	other	NONE	1.18E-05
Q5RJH6	SMG7_MOUSE	SMG7	(Q5RJH6) Protein SMG7 (SMG-7 homolog) (EST1-like protein C)	2	1.5	1138	126841	8	Nucleus	other	NONE	9.86E-06
Q5S006	LRRK2_MOUSE	LRRK2	(Q5S006) Leucine-rich repeat serine/threonine-protein kinase 2 (EC 2.7.11.1)	2	1.5	2527	284790	6.8	Cytoplasm	kinase	NONE	4.44E-06
Q5SF07	IF2B2_MOUSE	IGF2BP2	(Q5SF07) Insulin-like growth factor 2 mRNA-binding protein 2 (IGF-II mRNA-binding protein 2) (IMP-2)	1	2.4	592	65584	8	Cytoplasm	other	NONE	9.48E-06
Q5SUF2	CROP_MOUSE	CROP	(Q5SUF2) Cisplatin resistance-associated overexpressed protein	1	2.5	432	51450	9.8	Nucleus	other	NONE	1.3E-05
Q5SV85	SYNG_MOUSE	AP1GBP1	(Q5SV85) AP1 subunit gamma-binding protein 1 (Gamma-synergin)	2	3.1	1306	139616	5	Cytoplasm	other	NONE	1.29E-05
Q5SXA9	WWC1_MOUSE	WWC1	(Q5SXA9) WW domain-containing protein 1 (Kidney and brain protein) (KIBRA)	2	2.6	1104	124110	6	Unknown	other	NONE	3.05E-05
Q5U5M8	BL1S3_MOUSE	BLOC1S3 (includes EG:388552)	(Q5U5M8) Biogenesis of lysosome-related organelles complex-1 subunit 3 (Reduced pigmentation protein) (BLOC subunit 3)	2	9.2	195	20398	4.9	Cytoplasm	other	NONE	5.76E-05
Q5XJY5	COPD_MOUSE	ARCN1	(Q5XJY5) Coatomer subunit delta (Delta-coat protein) (Delta-COP) (Archain)	4	8.2	511	57217	6.2	Cytoplasm	other	NONE	6.59E-05
Q5YD48	ACF_MOUSE	ACF	(Q5YD48) APOBEC1 complementation factor (APOBEC1-stimulating protein)	2	5.4	595	65725	8.7	Nucleus	enzyme	NONE	1.89E-05
Q60597	ODO1_MOUSE	OGDH	(Q60597) 2-oxoglutarate dehydrogenase E1 component, mitochondrial precursor (EC 1.2.4.2) (Alpha-ketoglutarate dehydrogenase)	16	19.4	1019	116118	7	Cytoplasm	enzyme	NONE	0.000215
Q60598	SRC8_MOUSE	CTTN	(Q60598) Src substrate cortactin	8	16.5	546	61260	5.4	Plasma Membrane	other	NONE	0.000216
Q60605	MYL6_MOUSE	MYL6	(Q60605) Myosin light polypeptide 6 (Smooth muscle and nonmuscle myosin light chain alkali 6) (Myosin light chain alkali 3) (Myosin light chain 3) (MLC-3) (LC17)	12	47.3	150	16799	4.7	Cytoplasm	other	NONE	0.004453
Q60631	GRB2_MOUSE	GRB2	(Q60631) Growth factor receptor-bound protein 2 (Adapter protein GRB2) (SH2/SH3 adapter GRB2)	1	6.5	217	25238	6.3	Unknown	cytokine	NONE	5.17E-05
Q60648	SAP3_MOUSE	GM2A	(Q60648) Ganglioside GM2 activator precursor (GM2-AP) (Cerebroside sulfate activator protein) (Shingolipid activator protein 3) (SAP-3)	4	24.4	193	20824	5.9	Cytoplasm	enzyme	1	0.002298
Q60668	HNRPD_MOUSE	HNRPD	(Q60668) Heterogeneous nuclear ribonucleoprotein D0 (hnRNP D0) (AU-rich element RNA-binding protein 1)	6	15.5	355	38354	7.8	Nucleus	transcription regulator	NONE	0.000206
Q60676	PPP5_MOUSE	PPP5C	(Q60676) Serine/threonine-protein phosphatase 5 (EC 3.1.3.16) (PP5) (Protein phosphatase T) (PPT)	1	3.8	499	56847	6.2	Nucleus	phosphatase	NONE	1.12E-05
Q60692	PSB6_MOUSE	PSMB6	(Q60692) Proteasome subunit beta type 6 precursor (EC 3.4.25.1) (Proteasome delta chain) (Macropain delta chain) (Multicatalytic endopeptidase complex delta chain) (Proteasome subunit Y)	1	4.6	238	25379	5.1	Cytoplasm	peptidase	NONE	4.72E-05
Q60715	P4HA1_MOUSE	P4HA1	(Q60715) Prolyl 4-hydroxylase alpha-1 subunit precursor (EC 1.14.11.2) (4-PH alpha-1) (Procollagen-proline,2-oxoglutarate-4-dioxygenase alpha-1 subunit)	1	3.9	534	60910	5.9	Cytoplasm	enzyme	NONE	2.1E-05
Q60737	CSK21_MOUSE	CSNK2A1	(Q60737) Casein kinase II subunit alpha (EC 2.7.11.1) (CK II)	5	19.9	391	45162	8	Cytoplasm	kinase	NONE	0.000115
Q60739	BAG1_MOUSE	BAG1	(Q60739) BAG family molecular chaperone regulator 1 (BCL-2-binding athanogene-1) (BAG-1)	3	14.4	355	39740	8.5	Cytoplasm	other	NONE	7.91E-05
Q60749	SAM68_MOUSE	KHDRBS1	(Q60749) KH domain-containing, RNA-binding, signal transduction-associated protein 1 (p21 Ras GTPase-activating protein-associated p62) (GAP-associated tyrosine phosphoprotein p62) (Src-associated in mitosis 68 kDa protein) (Sam68) (p68)	1	3.2	443	48371	8.7	Nucleus	transcription regulator	NONE	6.34E-05
Q60751	IGF1R_MOUSE	IGF1R	(Q60751) Insulin-like growth factor 1 receptor precursor (EC 2.7.10.1) (Insulin-like growth factor I receptor) (IGF-I receptor) [Contains: Insulin-like growth factor 1 receptor alpha chain; Insulin-like growth factor 1 receptor beta chain]	2	2.3	1373	155788	5.7	Plasma Membrane	transmembrane receptor	2	1.23E-05
Q60759	GCDH_MOUSE	GCDH	(Q60759) Glutaryl-CoA dehydrogenase, mitochondrial precursor (EC 1.3.99.7) (GCD)	5	19.9	438	48647	8.6	Cytoplasm	enzyme	NONE	0.000308

Q60823	AKT2_MOUSE	AKT2	(Q60823) RAC-beta serine/threonine-protein kinase (EC 2.7.11.1) (RAC-PK-beta) (Protein kinase Akt-2) (Protein kinase B, beta) (PKB beta)	2	5	481	55742	6.4	Cytoplasm	kinase	NONE	2.33E-05
Q60855	RIPK1_MOUSE	RIPK1	(Q60855) Receptor-interacting serine/threonine-protein kinase 1 (EC 2.7.11.1) (Serine/threonine-protein kinase RIP) (Cell death protein RIP) (Receptor-interacting protein)	2	6.1	656	74854	6.5	Plasma Membrane	kinase	NONE	2.57E-05
Q60864	STIP1_MOUSE	STIP1	(Q60864) Stress-induced-phosphoprotein 1 (STI1) (Hsc70/Hsp90-organizing protein) (Hop) (mSTI1)	20	28.2	543	62582	6.8	Cytoplasm	other	NONE	0.000548
Q60865	GP137_MOUSE	GPIAP1	(Q60865) GPI-anchored protein p137 (p137GPI)	7	17.1	656	73548	5.4	Plasma Membrane	other	NONE	0.000188
Q60866	PTER_MOUSE	PTER	(Q60866) Phosphotriesterase-related protein (Parathion hydrolase-related protein)	8	22.9	349	39218	6.7	Unknown	enzyme	NONE	0.000273
Q60928	GGT1_MOUSE	GGT1	(Q60928) Gamma-glutamyltranspeptidase 1 precursor (EC 2.3.2.2) (Gamma-glutamyltransferase 1) (GGT 1) (CD224 antigen) [Contains: Gamma-glutamyltranspeptidase 1 heavy chain; Gamma-glutamyltranspeptidase 1 light chain]	16	24.1	568	61563	7.2	Cytoplasm	enzyme	1	0.002322
Q60930	VDAC2_MOUSE	VDAC2	(Q60930) Voltage-dependent anion-selective channel protein 2 (VDAC-2) (mVDAC2) (mVDAC6) (Outer mitochondrial membrane protein porin 2)	6	23.4	295	31733	7.5	Cytoplasm	ion channel	NONE	0.000932
Q60931	VDAC3_MOUSE	VDAC3	(Q60931) Voltage-dependent anion-selective channel protein 3 (VDAC-3) (mVDAC3) (Outer mitochondrial membrane protein porin 3)	5	20.1	283	30753	8.8	Cytoplasm	ion channel	NONE	0.002261
Q60932	VDAC1_MOUSE	VDAC1	(Q60932) Voltage-dependent anion-selective channel protein 1 (VDAC-1) (mVDAC1) (mVDAC5) (Outer mitochondrial membrane protein porin 1) (Plasmalemmal porin)	25	58.8	296	32351	8.4	Cytoplasm	ion channel	NONE	0.013995
Q60936	CABC1_MOUSE	CABC1	(Q60936) Chaperone-activity of bc1 complex-like, mitochondrial precursor (Chaperone-ABC1-like)	2	7.3	645	71743	6.5	Cytoplasm	kinase	NONE	2.61E-05
Q60949	TBCD1_MOUSE	TBC1D1	(Q60949) TBC1 domain family member 1	2	3.2	1255	142025	7.4	Nucleus	other	1	8.95E-06
Q60967	PAPS1_MOUSE	PAPSS1 (includes EG:9061)	(Q60967) Bifunctional 3'-phosphoadenosine 5'-phosphosulfate synthetase 1 (PAPS synthetase 1) (PAPSS 1) (Sulfurylase kinase 1) (SK1) (SK 1) [Includes: Sulfate adenylyltransferase (EC 2.7.7.4) (Sulfate adenylate transferase) (SAT) (ATP-sulfuryl	1	2.7	624	70794	6.8	Cytoplasm	enzyme	NONE	9E-06
Q61001	LAMA5_MOUSE	LAMA5	(Q61001) Laminin alpha-5 chain precursor	8	3.1	3718	404013	6.7	Extracellular Space	other	NONE	1.96E-05
Q61029	LAP2B_MOUSE	TMPO	(Q61029) Lamina-associated polypeptide 2 isoforms beta/delta/epsilon/gamma (Thymopoietin isoforms beta/delta/epsilon/gamma) (TP beta/delta/epsilon/gamma)	2	7.3	451	50163	9.4	Nucleus	other	1	4.98E-05
Q61033	LAP2A_MOUSE	TMPO	(Q61033) Lamina-associated polypeptide 2 isoforms alpha/zeta (Thymopoietin isoforms alpha/zeta) (TP alpha/zeta)	2	5.1	692	75200	8.3	Nucleus	other	NONE	4.87E-05
Q61035	SYH_MOUSE	HARS	(Q61035) Histidyl-tRNA synthetase (EC 6.1.1.21) (Histidine--tRNA ligase) (HisRS)	1	3.9	509	57416	5.8	Cytoplasm	enzyme	NONE	1.1E-05
Q61074	PP2CG_MOUSE	PPM1G	(Q61074) Protein phosphatase 2C isoform gamma (EC 3.1.3.16) (PP2C-gamma) (Protein phosphatase magnesium-dependent 1 gamma) (Protein phosphatase 1C) (Fibroblast growth factor-inducible protein 13) (FIN13)	2	6.3	542	58728	4.4	Nucleus	phosphatase	NONE	4.14E-05
Q61081	CDC37_MOUSE	CDC37	(Q61081) Hsp90 co-chaperone Cdc37 (Hsp90 chaperone protein kinase-targeting subunit) (p50Cdc37)	8	25.1	379	44593	5.3	Cytoplasm	other	NONE	0.000296
Q61103	REQU_MOUSE	DPF2	(Q61103) Zinc-finger protein ubi-d4 (Requiem) (Apoptosis response zinc finger protein) (D4, zinc and double PHD fingers family 2)	1	4.1	391	44230	6.5	Nucleus	other	NONE	1.44E-05
Q61133	GSTT2_MOUSE	GSTT2	(Q61133) Glutathione S-transferase theta-2 (EC 2.5.1.18) (GST class-theta-2)	2	12.8	243	27549	7.6	Cytoplasm	enzyme	NONE	6.93E-05
Q61136	PRP4B_MOUSE	PRPF4B	(Q61136) Serine/threonine-protein kinase PRP4 homolog (EC 2.7.11.1) (PRP4 pre-mRNA-processing factor 4 homolog) (Pre-mRNA protein kinase)	3	4.5	1007	116948	10.2	Nucleus	kinase	NONE	3.9E-05
Q61140	BCAR1_MOUSE	BCAR1	(Q61140) Breast cancer anti-estrogen resistance protein 1 (CRK-associated substrate) (p130cas)	1	1.8	874	94257	5.7	Plasma Membrane	other	NONE	1.28E-05
Q61146	OCLN_MOUSE	OCLN	(Q61146) Occludin	1	4.2	521	59000	6.5	Plasma Membrane	enzyme	4	2.15E-05

Q61166	MARE1_MOUSE	MAPRE1	(Q61166) Microtubule-associated protein RP/EB family member 1 (APC-binding protein EB1) (End-binding protein 1) (EB1)	8	43.8	267	29885	5.2	Cytoplasm	other	NONE	0.000399
Q61171	PRDX2_MOUSE	PRDX2	(Q61171) Peroxiredoxin-2 (EC 1.11.1.15) (Thioredoxin peroxidase 1) (Thioredoxin-dependent peroxide reductase 1) (Thiol-specific antioxidant protein) (TSA)	7	33.5	197	21647	5.4	Cytoplasm	enzyme	NONE	0.001026
Q61187	TS101_MOUSE	TSG101	(Q61187) Tumor susceptibility gene 101 protein	2	10	391	44124	6.7	Nucleus	transcription regulator	NONE	2.87E-05
Q61191	HCFC1_MOUSE	HCFC1	(Q61191) Host cell factor (HCF) (HCF-1) (C1 factor) [Contains: HCF N-terminal chain 1; HCF N-terminal chain 2; HCF N-terminal chain 3; HCF N-terminal chain 4; HCF N-terminal chain 5; HCF N-terminal chain 6; HCF C-terminal chain 1; HCF C-termi	2	2.9	2045	210535	7.3	Nucleus	transcription regulator	NONE	8.23E-06
Q61206	PA1B2_MOUSE	PAFAH1B2	(Q61206) Platelet-activating factor acetylhydrolase IB subunit beta (EC 3.1.1.47) (PAF acetylhydrolase 30 kDa subunit) (PAF-AH 30 kDa subunit) (PAF-AH subunit beta) (PAFAH subunit beta)	3	12.2	229	25492	6.2	Cytoplasm	enzyme	NONE	0.000221
Q61207	SAP_MOUSE	PSAP	(Q61207) Sulfated glycoprotein 1 precursor (SGP-1) (Prosaposin)	3	8.8	557	61422	5.2	Extracellular Space	other	1	5.04E-05
Q61233	PLSL_MOUSE	LCP1	(Q61233) Plastin-2 (L-plastin) (Lymphocyte cytosolic protein 1) (LCP-1) (65 kDa macrophage protein) (pp65)	2	5.8	626	70018	5.3	Cytoplasm	other	NONE	2.69E-05
Q61249	IGBP1_MOUSE	IGBP1	(Q61249) Immunoglobulin-binding protein 1 (CD79a-binding protein 1) (Alpha4 phosphoprotein) (Lymphocyte signal transduction molecule alpha 4) (p52)	6	23.2	340	38971	6.2	Cytoplasm	phosphatase	NONE	0.000132
Q61292	LAMB2_MOUSE	LAMB2	(Q61292) Laminin beta-2 chain precursor (S-laminin) (S-LAM)	5	4.9	1799	196352	6.7	Extracellular Space	enzyme	1	2.81E-05
Q61316	HSP74_MOUSE	HSPA4	(Q61316) Heat shock 70 kDa protein 4 (Heat shock 70-related protein APG-2)	12	18.8	841	94133	5.2	Cytoplasm	other	NONE	0.0002
Q61329	ATBF1_MOUSE	ATBF1 (includes EG:463)	(Q61329) Alpha-fetoprotein enhancer-binding protein (AT motif-binding factor) (AT-binding transcription factor 1)	2	0.9	3726	406573	6.2	Nucleus	transcription regulator	NONE	6.03E-06
Q61335	BAP31_MOUSE	BCAP31	(Q61335) B-cell receptor-associated protein 31 (BCR-associated protein Bap31) (p28 Bap31)	5	13.5	244	27791	8.7	Cytoplasm	transporter	3	0.00023
Q61337	BAD_MOUSE	BAD	(Q61337) Bcl2 antagonist of cell death (BAD) (Bcl-2-binding component 6) (Bcl-xL/Bcl-2-associated death promoter)	1	14.2	204	22080	9.2	Cytoplasm	other	NONE	2.75E-05
Q61387	COX7R_MOUSE	COX7A2L	(Q61387) Cytochrome c oxidase subunit VIIa-related protein, mitochondrial precursor (Silica-induced gene 81 protein) (SIG-81)	2	39.6	111	12399	9.6	Cytoplasm	enzyme	1	0.000152
Q61390	TCPW_MOUSE	CCT6B	(Q61390) T-complex protein 1 subunit zeta-2 (TCP-1-zeta-2) (CCT-zeta-2) (Cctz-2)	2	5.8	530	58054	7.4	Cytoplasm	transporter	NONE	0.000106
Q61391	NEP_MOUSE	MME	(Q61391) Neprilysin (EC 3.4.24.11) (Neutral endopeptidase) (NEP) (Enkephalinase) (Neutral endopeptidase 24.11) (Atriopeptidase) (CD10 antigen)	10	17	749	85571	5.8	Plasma Membrane	peptidase	1	0.000112
Q61411	RASH_MOUSE	HRAS	(Q61411) GTPase HRas precursor (Transforming protein p21) (p21ras) (H-Ras-1) (c-H-ras)	1	5.8	189	21348	5.3	Plasma Membrane	enzyme	NONE	2.97E-05
Q61425	HCDH_MOUSE	HADH	(Q61425) Short chain 3-hydroxyacyl-CoA dehydrogenase, mitochondrial precursor (EC 1.1.1.35) (HCDH) (Medium and short chain L-3-hydroxyacyl-coenzyme A dehydrogenase)	23	43.9	314	34464	8.6	Cytoplasm	enzyme	NONE	0.002038
Q61464	ZN638_MOUSE	ZNF638	(Q61464) Zinc finger protein 638 (Nuclear protein 220) (Zinc-finger matrin-like protein)	1	1.1	1960	218132	6.9	Nucleus	other	NONE	5.73E-06
Q61503	5NTD_MOUSE	NT5E	(Q61503) 5'-nucleotidase precursor (EC 3.1.3.5) (Ecto-5'-nucleotidase) (5'-NT) (CD73 antigen)	9	23.1	576	63864	6.7	Plasma Membrane	phosphatase	2	0.000136
Q61545	EWS_MOUSE	EWSR1	(Q61545) RNA-binding protein EWS	4	12.8	655	68418	9.3	Nucleus	other	NONE	8.57E-05
Q61553	FSCN1_MOUSE	FSCN1	(Q61553) Fascin (Singed-like protein)	1	3.3	492	54274	6.7	Cytoplasm	other	NONE	2.28E-05
Q61581	IBP7_MOUSE	IGFBP7	(Q61581) Insulin-like growth factor-binding protein 7 precursor (IGFBP-7) (IBP-7) (IGF-binding protein 7) (MAC25 protein)	4	21	281	28969	8.3	Extracellular Space	transporter	NONE	0.00014
Q61584	FXR1_MOUSE	FXR1	(Q61584) Fragile X mental retardation syndrome-related protein 1 (mFxr1p)	5	9.5	677	76222	7	Cytoplasm	other	NONE	8.29E-05

Q61592	GAS6_MOUSE	GAS6	(Q61592) Growth-arrest-specific protein 6 precursor (GAS-6)	1	3.4	674	74610	5.5	Extracellular Space	growth factor	NONE	8.33E-06
Q61595	KTN1_MOUSE	KTN1	(Q61595) Kinectin	3	5.3	1327	152592	5.9	Cytoplasm	other	1	7.19E-05
Q61598	GDIB_MOUSE	GDI2	(Q61598) Rab GDP dissociation inhibitor beta (Rab GDI beta) (Guanosine diphosphate dissociation inhibitor 2) (GDI-2) (GDI-3)	12	28.8	445	50537	6.2	Cytoplasm	other	NONE	0.000605
Q61599	GDIS_MOUSE	ARHGDIB	(Q61599) Rho GDP-dissociation inhibitor 2 (Rho GDI 2) (Rho-GDI beta) (D4)	7	40.2	199	22720	5.1	Cytoplasm	other	NONE	0.000621
Q61655	DD19A_MOUSE	DDX19A	(Q61655) ATP-dependent RNA helicase DDX19A (EC 3.6.1.-) (DEAD box protein 19A) (DEAD box RNA helicase DEAD5) (mDEAD5) (Eukaryotic translation initiation factor 4A-related sequence 1)	1	5.2	478	53889	6.7	Nucleus	enzyme	NONE	2.35E-05
Q61656	DDX5_MOUSE	DDX5	(Q61656) Probable ATP-dependent RNA helicase DDX5 (EC 3.6.1.-) (DEAD box protein 5) (RNA helicase p68) (DEAD box RNA helicase DEAD1) (mDEAD1)	6	10.6	614	69320	8.9	Nucleus	enzyme	NONE	9.14E-05
Q61686	CBX5_MOUSE	CBX5	(Q61686) Chromobox protein homolog 5 (Heterochromatin protein 1 homolog alpha) (HP1 alpha)	2	15.2	191	22186	5.9	Nucleus	other	NONE	0.000206
Q61687	ATRX_MOUSE	ATRX	(Q61687) Transcriptional regulator ATRX (EC 3.6.1.-) (ATP-dependent helicase ATRX) (X-linked nuclear protein) (Heterochromatin protein 2) (HP1 alpha-interacting protein) (HP1-BP38 protein)	2	1.7	2476	278602	6.7	Nucleus	transcription regulator	NONE	6.8E-06
Q61696	HS70A_MOUSE	HSPA1A	(Q61696) Heat shock 70 kDa protein 1A (Heat shock 70 kDa protein 3) (HSP70.3) (Hsp68)	5	12.6	641	70079	5.7	Cytoplasm	other	NONE	0.000123
Q61699	HS105_MOUSE	HSPH1	(Q61699) Heat-shock protein 105 kDa (Heat shock-related 100 kDa protein E7I) (HSP-E7I) (Heat shock 110 kDa protein) (42 degrees C-HSP)	8	15	858	96407	5.5	Cytoplasm	other	NONE	6.54E-05
Q61733	RT31_MOUSE	MRPS31	(Q61733) 28S ribosomal protein S31, mitochondrial precursor (S31mt) (MRP-S31) (Imogen 38)	6	18	384	43881	8.5	Cytoplasm	other	NONE	0.000219
Q61735	CD47_MOUSE	CD47	(Q61735) Leukocyte surface antigen CD47 precursor (Integrin-associated protein) (IAP)	2	8.9	303	33098	8.6	Plasma Membrane	other	6	3.71E-05
Q61739	ITA6_MOUSE	ITGA6	(Q61739) Integrin alpha-6 precursor (VLA-6) (CD49f antigen) [Contains: Integrin alpha-6 heavy chain; Integrin alpha-6 light chain]	6	8.3	1091	122148	7	Plasma Membrane	other	2	8.23E-05
Q61767	3BHS4_MOUSE	HSD3B4	(Q61767) 3 beta-hydroxysteroid dehydrogenase type 4 (3 beta-hydroxysteroid dehydrogenase type IV) (3Beta-HSD IV) (NADPH-dependent 3-beta-hydroxy-delta(5)-steroid dehydrogenase) (EC 1.1.1.-) (3-beta-hydroxy-5-ene steroid dehydrogenase) (Proges	1	3.2	372	41635	8.6	Cytoplasm	enzyme	NONE	3.02E-05
Q61768	KINH_MOUSE	KIF5B	(Q61768) Kinesin heavy chain (Ubiquitous kinesin heavy chain) (UKHC)	9	14.1	963	109549	6.3	Cytoplasm	other	NONE	9.91E-05
Q61792	LASP1_MOUSE	LASP1	(Q61792) LIM and SH3 domain protein 1 (LASP-1) (MLN 50)	10	22.4	263	29994	7	Cytoplasm	transporter	NONE	0.000555
Q61820	RANT_MOUSE	RASL2-9	(Q61820) GTP-binding nuclear protein Ran, testis-specific isoform	3	13.4	216	24452	6.5	Nucleus	enzyme	NONE	0.00013
Q61838	A2MG_MOUSE	PZP	(Q61838) Alpha-2-macroglobulin precursor (Alpha-2-M) [Contains: Alpha-2-macroglobulin 165 kDa subunit; Alpha-2-macroglobulin 35 kDa subunit]	2	2.4	1495	165827	6.7	Extracellular Space	other	NONE	7.51E-06
Q61847	MEP1B_MOUSE	MEP1B	(Q61847) Meprin A subunit beta precursor (EC 3.4.24.18) (Endopeptidase-2)	6	9.2	704	79549	5.9	Plasma Membrane	peptidase	1	0.000167
Q61879	MYH10_MOUSE	MYH10	(Q61879) Myosin-10 (Myosin heavy chain, nonmuscle IIb) (Nonmuscle myosin heavy chain IIb) (NMMHC II-b) (NMMHC-IIB) (Cellular myosin heavy chain, type B) (Nonmuscle myosin heavy chain-B) (NMMHC-B)	13	10.1	1976	228994	5.5	Cytoplasm	other	NONE	5.97E-05
Q61937	NPM_MOUSE	NPM1 (includes EG:18148)	(Q61937) Nucleophosmin (NPM) (Nucleolar phosphoprotein B23) (Numatrin) (Nucleolar protein NO38)	4	15.1	292	32560	4.8	Nucleus	transcription regulator	NONE	0.000192
Q61941	NNTM_MOUSE	NNT	(Q61941) NAD(P) transhydrogenase, mitochondrial precursor (EC 1.6.1.2) (Pyridine nucleotide transhydrogenase) (Nicotinamide nucleotide transhydrogenase)	11	13	1086	113838	7.6	Cytoplasm	enzyme	15	0.000109
Q61990	PCBP2_MOUSE	PCBP2	(Q61990) Poly(rC)-binding protein 2 (Alpha-CP2) (Putative heterogeneous nuclear ribonucleoprotein X) (hnRNP X) (CTBP) (CBP)	2	9.9	362	38222	6.8	Nucleus	other	NONE	7.75E-05

Q62048	PEA15_MOUSE	PEA15	(Q62048) Astrocytic phosphoprotein PEA-15	2	20.8	130	15054	5	Cytoplasm	transporter	NONE	0.00013
Q62093	SFRS2_MOUSE	SFRS2	(Q62093) Splicing factor, arginine/serine-rich 2 (Splicing factor SC35) (SC-35) (Splicing component, 35 kDa) (Protein PR264) (Putative myelin regulatory factor 1) (MRF-1)	5	11.4	220	25345	11.9	Nucleus	transcription regulator	NONE	0.000383
Q62165	DAG1_MOUSE	DAG1	(Q62165) Dystroglycan precursor (Dystrophin-associated glycoprotein 1) [Contains: Alpha-dystroglycan (Alpha-DG); Beta-dystroglycan (Beta-DG)]	2	2	893	96905	8.4	Plasma Membrane	transmembrane receptor	2	2.51E-05
Q62167	DDX3X_MOUSE	DDX3X	(Q62167) ATP-dependent RNA helicase DDX3X (EC 3.6.1.-) (DEAD box protein 3, X-chromosomal) (DEAD box RNA helicase DEAD3) (mDEAD3) (Embryonic RNA helicase) (D1Pas1-related sequence 2)	9	17.1	661	72970	7.2	Nucleus	enzyme	NONE	0.000195
Q62181	SEM3C_MOUSE	SEMA3C	(Q62181) Semaphorin-3C precursor (Semaphorin E) (Sema E)	2	4.7	751	85260	8.6	Extracellular Space	other	1	1.49E-05
Q62186	SSRD_MOUSE	SSR4	(Q62186) Translocon-associated protein subunit delta precursor (TRAP-delta) (Signal sequence receptor subunit delta) (SSR-delta)	2	15.1	172	18937	5.8	Cytoplasm	other	2	0.000131
Q62189	SNRPA_MOUSE	SNRPA	(Q62189) U1 small nuclear ribonucleoprotein A (U1 snRNP protein A) (U1A protein) (U1-A)	2	14.7	286	31704	9.8	Nucleus	other	NONE	7.85E-05
Q62203	SF3A2_MOUSE	SF3A2	(Q62203) Splicing factor 3A subunit 2 (Spliceosome-associated protein 62) (SAP 62) (SF3a66)	2	7.4	475	49911	9.5	Nucleus	other	NONE	3.55E-05
Q62241	RU1C_MOUSE	SNRPC	(Q62241) U1 small nuclear ribonucleoprotein C (U1 snRNP protein C) (U1C protein) (U1-C)	2	24.5	159	17364	9.7	Nucleus	other	NONE	0.000247
Q62252	SP17_MOUSE	SPA17	(Q62252) Sperm surface protein Sp17 (Sperm autoantigenic protein 17)	2	22.8	149	17296	4.8	Plasma Membrane	other	NONE	0.000151
Q62261	SPTB2_MOUSE	SPTBN1	(Q62261) Spectrin beta chain, brain 1 (Spectrin, non-erythroid beta chain 1) (Beta-II spectrin) (Fodrin beta chain)	38	23.7	2363	274221	5.6	Plasma Membrane	other	NONE	0.000171
Q62312	TGFR2_MOUSE	TGFBR2	(Q62312) TGF-beta receptor type-2 precursor (EC 2.7.11.30) (TGF-beta receptor type II) (TGFR-2) (TGF-beta type II receptor)	1	3.4	592	67122	6.3	Plasma Membrane	kinase	1	1.9E-05
Q62318	TIF1B_MOUSE	TRIM28	(Q62318) Transcription intermediary factor 1-beta (TIF1-beta) (Tripartite motif-containing protein 28) (KRAB-A-interacting protein) (KRIP-1)	4	5.8	833	88716	5.8	Nucleus	transcription regulator	NONE	5.39E-05
Q62351	TFR1_MOUSE	TFRC	(Q62351) Transferrin receptor protein 1 (TfR1) (TR) (TfR) (Trfr) (CD71 antigen)	2	5.1	763	85731	6.6	Plasma Membrane	transporter	1	1.47E-05
Q62376	RU17_MOUSE	SNRP70	(Q62376) U1 small nuclear ribonucleoprotein 70 kDa (U1 SNRNP 70 kDa) (snRNP70)	4	6	448	51992	9.9	Nucleus	other	NONE	8.77E-05
Q62393	TPD52_MOUSE	TPD52	(Q62393) Tumor protein D52 (mD52)	12	48.6	185	20059	4.9	Cytoplasm	other	NONE	0.001426
Q62394	ZN185_MOUSE	ZNF185	(Q62394) Zinc finger protein 185 (LIM-domain protein Zfp185) (P1-A)	1	11.4	352	38322	5.1	Nucleus	other	NONE	3.19E-05
Q62417	SRBS1_MOUSE	SORBS1	(Q62417) Sorbin and SH3 domain-containing protein 1 (Ponsin) (c-Cbl-associated protein) (CAP) (SH3 domain protein 5) (SH3P12)	11	10.9	1290	143070	8.2	Plasma Membrane	other	NONE	0.0001
Q62418	DBNL_MOUSE	DBNL	(Q62418) Drebrin-like protein (SH3 domain-containing protein 7) (Actin-binding protein 1)	6	22	436	48700	4.9	Cytoplasm	other	NONE	0.000154
Q62420	SH3G2_MOUSE	SH3GL2	(Q62420) SH3-containing GRB2-like protein 2 (EC 2.3.1.-) (SH3 domain protein 2A) (Endophilin 1) (SH3p4)	1	5.1	352	39877	5.5	Plasma Membrane	enzyme	NONE	1.59E-05
Q62422	OSTF1_MOUSE	OSTF1	(Q62422) Osteoclast-stimulating factor 1 (SH3 domain protein 3)	3	26	215	23783	5.7	Nucleus	transcription regulator	NONE	0.000339
Q62425	NDUA4_MOUSE	NDUFA4	(Q62425) NADH dehydrogenase [ubiquinone] 1 alpha subcomplex subunit 4 (EC 1.6.5.3) (EC 1.6.99.3) (NADH-ubiquinone oxidoreductase MLRQ subunit) (Complex I-MLRQ) (CI-MLRQ)	12	59.8	82	9327	9.5	Cytoplasm	enzyme	NONE	0.006708
Q62426	CYTB_MOUSE	CSTB	(Q62426) Cystatin B (Stefin B)	3	50	98	11046	7.4	Cytoplasm	other	NONE	0.000401
Q62433	NDRG1_MOUSE	NDRG1	(Q62433) Protein NDRG1 (N-myc downstream-regulated gene 1 protein) (Protein Ndr1)	17	32.2	394	43009	6.1	Nucleus	kinase	NONE	0.000826
Q62446	FKBP3_MOUSE	FKBP3	(Q62446) FK506-binding protein 3 (EC 5.2.1.8) (Peptidyl-prolyl cis-trans isomerase) (PPIase) (Rotamase) (25 kDa FKBP) (FKBP-25) (Rapamycin-selective 25 kDa immunophilin)	10	35.7	224	25148	9.3	Nucleus	enzyme	NONE	0.000601

Q62448	IF4G2_MOUSE	EIF4G2 (includes EG:1982)	(Q62448) Eukaryotic translation initiation factor 4 gamma 2 (eIF-4-gamma 2) (eIF-4G 2) (eIF4G 2) (p97) (Novel APOBEC-1 target 1) (Translation repressor NAT1)	4	10.8	906	102105	7.1	Cytoplasm	translation regulator	NONE	3.1E-05
Q62468	VILI_MOUSE	VIL1	(Q62468) Villin-1	31	42	826	92670	6	Cytoplasm	other	NONE	0.0007
Q62470	ITA3_MOUSE	ITGA3	(Q62470) Integrin alpha-3 precursor (Galactoprotein B3) (GAPB3) (VLA-3 alpha chain) (CD49c antigen) [Contains: Integrin alpha-3 heavy chain; Integrin alpha-3 light chain]	6	8.3	1053	116745	6.6	Plasma Membrane	other	2	6.4E-05
Q62523	ZYX_MOUSE	ZYX	(Q62523) Zyxin	2	3.4	564	60790	6.9	Plasma Membrane	other	NONE	3.98E-05
Q63829	COMD3_MOUSE	COMMD3	(Q63829) COMM domain-containing protein 3 (Bmi-1 upstream gene protein) (Bup protein)	3	22.1	195	22037	5.6	Unknown	other	NONE	0.000173
Q63850	NUP62_MOUSE	NUP62	(Q63850) Nuclear pore glycoprotein p62 (62 kDa nucleoporin)	1	2.7	526	53255	5.3	Nucleus	transporter	NONE	2.13E-05
Q63886	UD11_MOUSE	UGT1A3	(Q63886) UDP-glucuronosyltransferase 1-1 precursor (EC 2.4.1.17) (UDPGT) (UGT1*1) (UGT1-01) (UGT1.1) (UGT1A1) (UGTBR1)	6	9.5	535	60124	8.6	Cytoplasm	enzyme	2	0.000231
Q63918	SDPR_MOUSE	SDPR	(Q63918) Serum deprivation-response protein (Phosphatidylserine-binding protein)	2	4.3	417	46633	5.2	Plasma Membrane	other	NONE	5.38E-05
Q63ZW7	INADL_MOUSE	INADL (includes EG:10207)	(Q63ZW7) InaD-like protein (Inadl protein) (Pals1-associated tight junction protein) (Protein associated to tight junctions) (Channel-interacting PDZ domain-containing protein)	2	2.5	1834	198515	5	Plasma Membrane	other	NONE	9.18E-06
Q64010	CRK_MOUSE	CRK	(Q64010) Proto-oncogene C-crk (P38) (Adapter molecule crk)	5	25.3	304	33815	5.6	Cytoplasm	other	NONE	0.000295
Q64012	RALY_MOUSE	RALY	(Q64012) RNA-binding protein Raly (hnRNP associated with lethal yellow protein) (Maternally expressed hnRNP C-related protein)	2	10.6	312	33158	8.8	Nucleus	other	NONE	7.2E-05
Q64105	SPRE_MOUSE	SPR	(Q64105) Sepiapterin reductase (EC 1.1.1.153) (SPR)	9	41.8	261	27883	5.7	Cytoplasm	enzyme	NONE	0.000581
Q64152	BTF3_MOUSE	BTF3	(Q64152) Transcription factor BTF3 (RNA polymerase B transcription factor 3)	5	36.8	204	22031	9.5	Nucleus	transcription regulator	NONE	0.000633
Q64176	EST22_MOUSE	ES22	(Q64176) Liver carboxylesterase 22 precursor (EC 3.1.1.1) (Egasyn) (Esterase-22) (Es-22)	2	7.1	562	61582	6.1	Cytoplasm	enzyme	1	6.99E-05
Q64191	ASPG_MOUSE	AGA	(Q64191) N(4)-(beta-N-acetylglucosaminyl)-L-asparaginase precursor (EC 3.5.1.26) (Glycosylasparaginase) (Aspartylglucosaminidase) (N4-(N-acetyl-beta-glucosaminyl)-L-asparagine amidase) (AGA) [Contains: Glycosylasparaginase alpha chain; Glycosy	6	21.7	346	37022	6.4	Cytoplasm	enzyme	1	0.000195
Q64213	SF01_MOUSE	SF1	(Q64213) Splicing factor 1 (Zinc finger protein 162) (Transcription factor ZFM1) (mZFM) (Zinc finger gene in MEN1 locus) (Mammalian branch point-binding protein mBBP) (BBP) (CW17)	7	14.1	652	70277	8.8	Nucleus	transcription regulator	NONE	8.61E-05
Q64314	CD34_MOUSE	CD34	(Q64314) Hematopoietic progenitor cell antigen CD34 precursor	2	7.6	382	40983	5.3	Plasma Membrane	other	2	4.41E-05
Q64324	STXB2_MOUSE	STXBP2	(Q64324) Syntaxin-binding protein 2 (UNC-18 homolog 2) (UNC-18B) (MUSEC1)	2	5.9	593	66358	6.7	Unknown	transporter	NONE	5.68E-05
Q64331	MYO6_MOUSE	MYO6	(Q64331) Myosin-6 (Myosin VI)	12	11.9	1265	146409	8.8	Cytoplasm	other	NONE	0.000102
Q64374	RGN_MOUSE	RGN	(Q64374) Regucalcin (RC) (Senescence marker protein 30) (SMP-30)	1	5.7	299	33407	5.3	Nucleus	other	NONE	1.88E-05
Q64378	FKBP5_MOUSE	FKBP5	(Q64378) FK506-binding protein 5 (EC 5.2.1.8) (Peptidyl-prolyl cis-trans isomerase) (PPIase) (Rotamase) (51 kDa FK506-binding protein) (FKBP-51)	3	6.4	456	50966	7.8	Nucleus	enzyme	NONE	9.85E-05
Q64433	CH10_MOUSE	HSPE1	(Q64433) 10 kDa heat shock protein, mitochondrial (Hsp10) (10 kDa chaperonin) (CPN10)	17	79.2	101	10831	8.3	Cytoplasm	enzyme	NONE	0.017284
Q64441	CP24A_MOUSE	CYP24A1	(Q64441) Cytochrome P450 24A1, mitochondrial precursor (EC 1.14.-.-) (P450-CC24) (Vitamin D(3) 24-hydroxylase) (1,25-dihydroxyvitamin D(3) 24-hydroxylase) (24-OHase)	2	6.6	514	59453	9	Cytoplasm	enzyme	NONE	2.18E-05
Q64442	DHSO_MOUSE	SORD	(Q64442) Sorbitol dehydrogenase (EC 1.1.1.14) (L-iditol 2-dehydrogenase) (Fragment)	19	44.3	375	40091	7	Unknown	enzyme	NONE	0.001332
Q64444	CAH4_MOUSE	CA4	(Q64444) Carbonic anhydrase 4 precursor (EC 4.2.1.1) (Carbonic anhydrase IV) (Carbonate dehydratase IV) (CA-IV)	3	17	305	34351	8.2	Plasma Membrane	enzyme	NONE	7.36E-05

Q64462	CP4B1_MOUSE	CYP4B1	(Q64462) Cytochrome P450 4B1 (EC 1.14.14.1) (CYPIVB1)	5	13.3	511	58900	8.4	Cytoplasm	enzyme	1	0.000187
Q64467	G3PT_MOUSE	GAPDHS	(Q64467) Glyceraldehyde-3-phosphate dehydrogenase, testis-specific (EC 1.2.1.12) (Spermatogenic cell-specific glyceraldehyde 3-phosphate dehydrogenase 2) (GAPDH-2)	2	4.8	440	47657	7.9	Cytoplasm	enzyme	NONE	0.000102
Q64475	H2B1B_MOUSE	HIST1H2BB	(Q64475) Histone H2B type 1-B (h2B-143)	10	21.6	125	13821	10.3	Nucleus	other	NONE	0.018231
Q64512	PTN13_MOUSE	PTPN13	(Q64512) Tyrosine-protein phosphatase non-receptor type 13 (EC 3.1.3.48) (Protein tyrosine phosphatase PTP-BL) (Protein-tyrosine phosphatase RIP) (protein tyrosine phosphatase DPZPTP) (PTP36)	4	2.6	2453	270332	6.4	Cytoplasm	phosphatase	NONE	1.6E-05
Q64516	GLPK_MOUSE	GK	(Q64516) Glycerol kinase (EC 2.7.1.30) (ATP:glycerol 3-phosphotransferase) (Glycerokinase) (GK)	5	8.4	524	57458	5.6	Cytoplasm	kinase	NONE	0.000161
Q64520	KGUA_MOUSE	GUK1	(Q64520) Guanylate kinase (EC 2.7.4.8) (GMP kinase)	3	19.8	197	21787	6.5	Cytoplasm	kinase	NONE	0.000256
Q64522	H2A2B_MOUSE	HIST2H2AB	(Q64522) Histone H2A type 2-B (H2a-613A)	4	23.3	129	13882	10.9	Nucleus	other	NONE	0.003263
Q64669	NQO1_MOUSE	NQO1	(Q64669) NAD(P)H dehydrogenase [quinone] 1 (EC 1.6.5.2) (Quinone reductase 1) (NAD(P)H:quinone oxidoreductase 1) (QR1) (DT-diaphorase) (DTD) (Azoreductase) (Phylloquinone reductase) (Menadione reductase)	1	4.8	273	30828	8.7	Cytoplasm	enzyme	NONE	4.11E-05
Q64704	STX3_MOUSE	STX3	(Q64704) Syntaxin-3	6	18.3	289	33243	5.4	Plasma Membrane	transporter	1	0.000272
Q64727	VINC_MOUSE	VCL	(Q64727) Vinculin (Metavinculin)	12	14.6	1065	116586	6	Plasma Membrane	enzyme	NONE	0.000158
Q64737	PUR2_MOUSE	GART	(Q64737) Trifunctional purine biosynthetic protein adenosine-3 [Includes: Phosphoribosylamine--glycine ligase (EC 6.3.4.13) (GARS) (Glycinamide ribonucleotide synthetase) (Phosphoribosylglycinamide synthetase); Phosphoribosylformylglycinamidin	1	2.6	1010	107395	6.7	Cytoplasm	enzyme	NONE	1.11E-05
Q64739	COBA2_MOUSE	COL11A2	(Q64739) Collagen alpha-2(XI) chain precursor	2	2.2	1736	171535	6.5	Extracellular Space	other	NONE	1.29E-05
Q66JS6	IF31_MOUSE	EIF3S1	(Q66JS6) Eukaryotic translation initiation factor 3 subunit 1 (eIF-3 alpha)	3	15.6	263	29486	4.8	Cytoplasm	translation regulator	NONE	0.000107
Q68ED3	PAD5_MOUSE	PAPD5	(Q68ED3) PAP-associated domain-containing protein 5 (EC 2.7.7.-) (Topoisomerase-related function protein 4-2) (TRF4-2)	3	7	633	69704	8.8	Nucleus	enzyme	NONE	2.66E-05
Q68EF0	RAB3I_MOUSE	RAB3IP	(Q68EF0) RAB3A-interacting protein (Rabin-3) (SSX2-interacting protein)	2	7.5	428	47134	6.7	Cytoplasm	other	NONE	7.87E-05
Q68FD5	CLH_MOUSE	CLTC	(Q68FD5) Clathrin heavy chain	11	10	1675	191555	5.7	Plasma Membrane	other	NONE	6.7E-05
Q68FE8	SUHW4_MOUSE	SUHW4 (includes EG:54816)	(Q68FE8) Suppressor of hairy wing homolog 4	1	2.1	974	107836	6.8	Unknown	other	NONE	1.15E-05
Q68FH0	PKP4_MOUSE	PKP4	(Q68FH0) Plakophilin-4 (Armadillo-related protein)	1	2.7	1190	131551	8.9	Plasma Membrane	other	NONE	4.72E-06
Q68FL4	SAHH3_MOUSE	KIAA0828	(Q68FL4) Putative adenosylhomocysteinase 3 (EC 3.3.1.1) (S-adenosyl-L-homocysteine hydrolase 3) (AdoHcyase 3)	8	12.6	613	66899	7.4	Unknown	enzyme	NONE	0.000229
Q6A068	CDC5L_MOUSE	CDC5L	(Q6A068) Cell division cycle 5-related protein (Cdc5-like protein)	4	5.1	802	92190	8	Nucleus	other	NONE	4.2E-05
Q6GSS7	H2A2A_MOUSE	HIST2H2AA2	(Q6GSS7) Histone H2A type 2-A (H2A.2) (H2a-614) (H2a-615)	7	56.6	129	13964	10.9	Unknown	other	NONE	0.005265
Q6IRU2	TPM4_MOUSE	TPM4	(Q6IRU2) Tropomyosin alpha-4 chain (Tropomyosin-4)	12	33.2	247	28337	4.7	Cytoplasm	other	NONE	0.000909
Q6IRU5	CLCB_MOUSE	CLTB	(Q6IRU5) Clathrin light chain B (Lcb)	3	11.4	229	25172	4.6	Plasma Membrane	other	NONE	0.000196
Q6KAR6	EXOC3_MOUSE	EXOC3	(Q6KAR6) Exocyst complex component 3 (Exocyst complex component Sec6)	1	2.4	755	86455	6.2	Plasma Membrane	transporter	NONE	1.49E-05
Q6NSR8	PEPL1_MOUSE	NPEPL1	(Q6NSR8) Probable aminopeptidase NPEPL1 (EC 3.4.11.-) (Aminopeptidase-like 1)	2	8.8	524	55940	6.9	Unknown	peptidase	NONE	3.21E-05
Q6NVF9	CPSF6_MOUSE	CPSF6	(Q6NVF9) Cleavage and polyadenylation specificity factor 6	1	4.5	551	59153	7.2	Nucleus	other	NONE	5.09E-05
Q6NXW6	RAD17_MOUSE	RAD17	(Q6NXW6) Cell cycle checkpoint protein RAD17	2	3.1	688	77391	6.1	Nucleus	other	NONE	1.63E-05
Q6NZB0	DNJC8_MOUSE	DNAJC8	(Q6NZB0) DnaJ homolog subfamily C member 8	2	18.2	253	29813	9.1	Nucleus	other	NONE	0.000177
Q6NZF1	ZC11A_MOUSE	ZC3H11A	(Q6NZF1) Zinc finger CCCH domain-containing protein 11A	3	7.3	792	86492	8.1	Unknown	other	NONE	2.83E-05
Q6NZJ6	IF4G1_MOUSE	EIF4G1	(Q6NZJ6) Eukaryotic translation initiation factor 4 gamma 1 (eIF-4-gamma 1) (eIF-4G1) (eIF-4G 1)	11	10.3	1600	176076	5.4	Cytoplasm	translation regulator	NONE	6.31E-05

Q6P1B1	XPP1_MOUSE	XPNPEP1	(Q6P1B1) Xaa-Pro aminopeptidase 1 (EC 3.4.11.9) (X-Pro aminopeptidase 1) (X-prolyl aminopeptidase 1, soluble) (Cytosolic aminopeptidase P) (Soluble aminopeptidase P) (sAmp) (Aminoacylproline aminopeptidase)	3	9.8	623	69591	5.5	Cytoplasm	peptidase	NONE	4.5E-05
Q6P542	ABCF1_MOUSE	ABCF1	(Q6P542) ATP-binding cassette sub-family F member 1	1	2.2	837	94945	6.5	Cytoplasm	transporter	NONE	1.34E-05
Q6P5E6	GGA2_MOUSE	GGA2	(Q6P5E6) ADP-ribosylation factor-binding protein GGA2 (Golgi-localized, gamma ear-containing, ARF-binding protein 2) (Gamma-adaptin-related protein 2)	1	4.5	603	66049	7.3	Cytoplasm	transporter	NONE	1.86E-05
Q6P8I4	PCNP_MOUSE	PCNP	(Q6P8I4) PEST-containing nuclear protein (PCNP)	2	15.2	178	18963	7.5	Nucleus	other	NONE	9.46E-05
Q6P8J7	KCRS_MOUSE	CKMT2	(Q6P8J7) Creatine kinase, sarcomeric mitochondrial precursor (EC 2.7.3.2) (S-MtCK)	2	6	419	47473	8.4	Cytoplasm	kinase	NONE	6.7E-05
Q6P8X1	SNX6_MOUSE	SNX6	(Q6P8X1) Sorting nexin-6	10	26.6	406	46635	6	Cytoplasm	transporter	NONE	0.000263
Q6P9J9	TM16F_MOUSE	TMEM16F	(Q6P9J9) Transmembrane protein 16F	2	2.3	911	106255	6.7	Unknown	other	8	3.08E-05
Q6P9K8	CSKI1_MOUSE	CASKIN1	(Q6P9K8) Caskin-1 (CASK-interacting protein 1)	2	1.7	1431	150495	9.2	Nucleus	transcription regulator	NONE	7.85E-06
Q6P9R2	OXSR1_MOUSE	OXSR1	(Q6P9R2) Serine/threonine-protein kinase OSR1 (EC 2.7.11.1) (Oxidative stress-responsive 1 protein)	2	7.4	527	58214	6.4	Nucleus	kinase	NONE	3.2E-05
Q6PAM1	TXLNA_MOUSE	TXLNA	(Q6PAM1) Alpha-taxilin	2	8.7	554	62369	6.7	Extracellular Space	cytokine	NONE	3.04E-05
Q6PB93	GALT2_MOUSE	GALNT2	(Q6PB93) Polypeptide N-acetylgalactosaminyltransferase 2 (EC 2.4.1.41) (Protein-UDP acetylgalactosaminyltransferase 2) (UDP-GalNAc:polypeptide N-acetylgalactosaminyltransferase 2) (Polypeptide GalNAc transferase 2) (GalNAc-T2) (pp-GaNTase 2)	1	3.5	570	64515	8.5	Cytoplasm	enzyme	1	9.85E-06
Q6PDG5	SMRC2_MOUSE	SMARCC2	(Q6PDG5) SWI/SNF-related matrix-associated actin-dependent regulator of chromatin subfamily C member 2 (SWI/SNF complex 170 kDa subunit) (BRG1-associated factor 170)	2	2.8	1213	132604	5.6	Nucleus	transcription regulator	NONE	1.39E-05
Q6PDM2	SFRS1_MOUSE	SFRS1	(Q6PDM2) Splicing factor, arginine/serine-rich 1	8	29.6	247	27613	10.4	Nucleus	other	NONE	0.000409
Q6PDN3	MYLK_MOUSE	MYLK	(Q6PDN3) Myosin light chain kinase, smooth muscle (EC 2.7.11.18) (MLCK) (Telokin) (Kinase-related protein) (KRP)	7	4.9	1941	212924	6.2	Cytoplasm	kinase	1	3.18E-05
Q6PDQ2	CHD4_MOUSE	CHD4	(Q6PDQ2) Chromodomain helicase-DNA-binding protein 4 (CHD-4)	3	2.8	1915	217749	5.8	Nucleus	enzyme	NONE	8.79E-06
Q6PEM6	GRAM3_MOUSE	GRAMD3	(Q6PEM6) GRAM domain-containing protein 3	3	5.2	445	49287	8.1	Unknown	other	1	6.31E-05
Q6PER3	MARE3_MOUSE	MAPRE3	(Q6PER3) Microtubule-associated protein RP/EB family member 3 (End-binding protein 3) (EB3) (EB1 protein family member 3) (EBF3) (RP3)	2	6.4	281	31966	5.5	Cytoplasm	enzyme	NONE	7.99E-05
Q6PEV3	WIRE_MOUSE	WIPF2	(Q6PEV3) WIP-related protein (WASP-interacting protein-related protein)	1	4.1	440	46298	11	Unknown	other	NONE	1.28E-05
Q6PFR5	TRA2A_MOUSE	TRA2A	(Q6PFR5) Transformer-2 protein homolog (TRA-2 alpha)	1	5	281	32316	11.3	Nucleus	other	NONE	4E-05
Q6PGK7	CHSTA_MOUSE	CHST10	(Q6PGK7) Carbohydrate sulfotransferase 10 (EC 2.8.2.-) (HNK-1 sulfotransferase) (HNK1ST) (HNK-1ST)	1	6.7	356	42055	9	Cytoplasm	enzyme	1	1.58E-05
Q6PNC0	DMXL1_MOUSE	DMXL1	(Q6PNC0) Protein DmX-like 1 (X-like 1 protein)	3	1.7	3013	336009	6.4	Unknown	other	NONE	5.59E-06
Q6R891	NEB2_MOUSE	PPP1R9B	(Q6R891) Neurabin-2 (Neurabin-II) (Spinophilin) (Protein phosphatase 1 regulatory subunit 9B)	3	6.6	817	89520	4.9	Cytoplasm	other	NONE	2.75E-05
Q6WVG3	KCD12_MOUSE	KCTD12	(Q6WVG3) Potassium channel tetramerization domain-containing protein 12 (Pfetin) (Predominantly fetal expressed T1 domain)	2	8.9	327	35892	5.8	Unknown	ion channel	NONE	8.58E-05
Q6Y685	TACC1_MOUSE	TACC1	(Q6Y685) Transforming acidic coiled-coil-containing protein 1	1	1.4	774	83952	5	Nucleus	other	NONE	7.25E-06
Q6ZQ38	CAND1_MOUSE	CAND1	(Q6ZQ38) Cullin-associated NEDD8-dissociated protein 1 (Cullin-associated and neddylation-dissociated protein 1) (p120 CAND1)	2	1.9	1230	136331	5.8	Cytoplasm	transcription regulator	NONE	2.28E-05
Q6ZQI3	K0152_MOUSE	KIAA0152	(Q6ZQI3) Protein KIAA0152 precursor	7	32.3	291	32342	6	Plasma Membrane	other	3	0.000617
Q6ZWM4	LSM8_MOUSE	LSM8	(Q6ZWM4) U6 snRNA-associated Sm-like protein LSm8	1	16.8	95	10271	4.5	Nucleus	other	NONE	0.000118
Q6ZWN5	RS9_MOUSE	RPS9	(Q6ZWN5) 40S ribosomal protein S9	6	26.4	193	22460	10.7	Cytoplasm	other	NONE	0.000698
Q6ZWV3	RL10_MOUSE	RPL10	(Q6ZWV3) 60S ribosomal protein L10 (QM protein homolog)	6	11.7	213	24473	10.1	Cytoplasm	other	NONE	0.000343

Q6ZWX6	IF2A_MOUSE	EIF2S1	(Q6ZWX6) Eukaryotic translation initiation factor 2 subunit 1 (Eukaryotic translation initiation factor 2 subunit alpha) (eIF-2-alpha) (EIF-2alpha) (EIF-2A)	8	31.2	314	35977	5.1	Cytoplasm	translation regulator	NONE	0.000375
Q6ZWY3	RS27L_MOUSE	RPS27L	(Q6ZWY3) 40S ribosomal protein S27-like protein	3	28.9	83	9346	9.5	Unknown	other	NONE	0.000473
Q78IK4	CX033_MOUSE	FAM121A	(Q78IK4) Protein CXorf33 homolog precursor	12	47.9	265	29261	9.3	Extracellular Space	other	NONE	0.000763
Q78JT3	3HAO_MOUSE	HAAO	(Q78JT3) 3-hydroxyanthranilate 3,4-dioxygenase (EC 1.13.11.6) (3-HAO) (3-hydroxyanthranilic acid dioxygenase) (3-hydroxyanthranilate oxygenase)	7	28	286	32804	6.5	Cytoplasm	enzyme	NONE	0.000353
Q78PY7	SND1_MOUSE	SND1	(Q78PY7) Staphylococcal nuclease domain-containing protein 1 (p100 co-activator) (100 kDa coactivator)	6	11.3	910	102088	7.4	Nucleus	enzyme	NONE	8.02E-05
Q78ZA7	NP1L4_MOUSE	NAP1L4	(Q78ZA7) Nucleosome assembly protein 1-like 4	7	20.3	375	42679	4.7	Nucleus	other	NONE	0.000284
Q791V5	MTCH2_MOUSE	MTCH2	(Q791V5) Mitochondrial carrier homolog 2	1	6.9	303	33499	8.3	Cytoplasm	other	3	3.71E-05
Q7M6Y3	PICA_MOUSE	PICALM	(Q7M6Y3) Phosphatidylinositol-binding clathrin assembly protein (Clathrin assembly lymphoid myeloid leukemia) (CALM)	7	17.9	660	71543	7.9	Cytoplasm	other	NONE	0.00034
Q7TMF3	NDUAC_MOUSE	NDUFA12	(Q7TMF3) NADH dehydrogenase [ubiquinone] 1 alpha subcomplex subunit 12 (EC 1.6.5.3) (EC 1.6.99.3) (NADH-ubiquinone oxidoreductase subunit B17.2) (Complex I-B17.2) (CI-B17.2) (CIB17.2)	7	51.7	145	17086	9.4	Cytoplasm	enzyme	NONE	0.000542
Q7TMK6	HOOK2_MOUSE	HOOK2	(Q7TMK6) Hook homolog 2	2	4.9	716	83384	5.4	Cytoplasm	other	NONE	1.57E-05
Q7TMK9	HNRPQ_MOUSE	SYNCRIP	(Q7TMK9) Heterogeneous nuclear ribonucleoprotein Q (hnRNP Q) (hnRNP-Q) (Synaptotagmin-binding, cytoplasmic RNA-interacting protein) (Glycine- and tyrosine-rich RNA-binding protein) (GRY-RBP) (NS1-associated protein 1) (pp68)	6	12.4	623	69633	8.6	Nucleus	other	NONE	0.000117
Q7TMS5	ABCG2_MOUSE	ABCG2	(Q7TMS5) ATP-binding cassette sub-family G member 2 (Breast cancer resistance protein 1 homolog)	6	13.2	657	72978	8.5	Plasma Membrane	transporter	6	0.000137
Q7TMY8	HUWE1_MOUSE	HUWE1	(Q7TMY8) HECT, UBA and WWE domain-containing protein 1 (EC 6.3.2.-) (E3 ubiquitin protein ligase URE-B1) (E3Histone)	3	1.4	4377	482668	5.2	Nucleus	transcription regulator	NONE	7.69E-06
Q7TNC4	LC7L2_MOUSE	LUC7L2	(Q7TNC4) Putative RNA-binding protein Luc7-like 2 (CGI-74 homolog)	4	13	392	46583	10.1	Unknown	other	NONE	0.000115
Q7TND5	RPF1_MOUSE	BXDC5	(Q7TND5) Ribosome production factor 1 (Ribosome biogenesis protein RPF1) (Brix domain-containing protein 5)	2	7.2	349	40037	10	Nucleus	other	NONE	6.43E-05
Q7TNE1	CG010_MOUSE	C7ORF10	(Q7TNE1) Protein C7orf10 homolog	3	8.9	436	47674	8.7	Unknown	other	NONE	0.000103
Q7TNV0	DEK_MOUSE	DEK	(Q7TNV0) Protein DEK	3	6.8	380	43159	6.9	Nucleus	transcription regulator	NONE	7.39E-05
Q7TPR4	ACTN1_MOUSE	ACTN1	(Q7TPR4) Alpha-actinin-1 (Alpha-actinin cytoskeletal isoform) (Non-muscle alpha-actinin-1) (F-actin cross linking protein)	6	7.1	892	103068	5.4	Cytoplasm	other	NONE	0.000101
Q7TPV4	MBB1A_MOUSE	MYBBP1A	(Q7TPV4) Myb-binding protein 1A (Myb-binding protein of 160 kDa)	4	4.8	1344	152036	9	Nucleus	transcription regulator	NONE	3.34E-05
Q7TQD2	P25A_MOUSE	TPPP	(Q7TQD2) Tubulin polymerization-promoting protein (TPPP)	1	7.3	218	23575	9.4	Cytoplasm	other	NONE	7.72E-05
Q7TQG1	PKHA6_MOUSE	PLEKHA6	(Q7TQG1) Pleckstrin homology domain-containing family A member 6 (Phosphoinositol 3-phosphate-binding protein 3) (PEPP-3)	5	6.4	1173	131427	9	Unknown	other	NONE	3.35E-05
Q7TQH0	ATX2L_MOUSE	ATXN2L	(Q7TQH0) Ataxin-2-like protein	2	3.9	1049	110649	8.9	Unknown	other	NONE	1.61E-05
Q7TQI3	OTUB1_MOUSE	OTUB1	(Q7TQI3) Ubiquitin thioesterase protein OTUB1 (EC 3.4.-.-) (Otubain 1) (OTU domain-containing ubiquitin aldehyde-binding protein 1) (Ubiquitin-specific-processing protease OTUB1) (Deubiquitinating enzyme OTUB1)	3	17	271	31270	4.9	Unknown	other	NONE	0.000269
Q7TSI3	SAPS1_MOUSE	SAPS1	(Q7TSI3) SAPS domain family member 1	1	2.6	856	94527	4.6	Unknown	other	NONE	6.56E-06
Q7TT50	MRCKB_MOUSE	CDC42BPB	(Q7TT50) Serine/threonine-protein kinase MRCK beta (EC 2.7.11.1) (CDC42-binding protein kinase beta) (Myotonic dystrophy kinase-related CDC42-binding kinase beta) (Myotonic dystrophy protein kinase-like beta) (MRCK beta) (DMPK-like beta)	2	2.9	1713	194779	6.5	Cytoplasm	kinase	NONE	9.83E-06
Q80ST9	CF152_MOUSE	C6ORF152	(Q80ST9) Protein C6orf152 homolog	2	3.4	704	80162	8.2	Unknown	other	NONE	6.38E-05
Q80TP3	EDD1_MOUSE	EDD1	(Q80TP3) Ubiquitin-protein ligase EDD1 (EC 6.3.2.-) (Hyperplastic discs protein homolog)	1	1.2	2792	308351	5.9	Nucleus	enzyme	NONE	6.03E-06

Q80U19	DAAM2_MOUSE	DAAM2	(Q80U19) Disheveled-associated activator of morphogenesis 2	2	2.7	1068	123535	6.7	Unknown	other	NONE	1.05E-05
Q80U78	PUM1_MOUSE	PUM1	(Q80U78) Pumilio homolog 1	1	1.6	1189	126619	6.9	Cytoplasm	other	NONE	9.44E-06
Q80U87	UBP8_MOUSE	USP8	(Q80U87) Ubiquitin carboxyl-terminal hydrolase 8 (EC 3.1.2.15) (Ubiquitin thioesterase 8) (Ubiquitin-specific-processing protease 8) (Deubiquitinating enzyme 8) (mUBPy)	2	3.1	1080	122610	8.5	Cytoplasm	peptidase	NONE	2.6E-05
Q80UG5	SEPT9_MOUSE	SPET9	(Q80UG5) Septin-9 (SL3-3 integration site 1 protein)	4	8.2	583	65575	8.9	Cytoplasm	enzyme	NONE	8.67E-05
Q80UU9	PGRC2_MOUSE	PGRMC2	(Q80UU9) Membrane-associated progesterone receptor component 2 (Fragment)	3	22	214	23061	5.1	Nucleus	ligand-dependent nuclear receptor	1	0.000472
Q80V42	CBPM_MOUSE	CPM	(Q80V42) Carboxypeptidase M precursor (EC 3.4.17.12)	3	9.9	443	50556	7.8	Plasma Membrane	peptidase	2	3.8E-05
Q80VC9	K1543_MOUSE	KIAA1543	(Q80VC9) Protein KIAA1543	3	6.9	1252	135175	8.4	Unknown	other	NONE	3.14E-05
Q80VD1	FA98B_MOUSE	FAM98B	(Q80VD1) Protein FAM98B	1	3.7	429	45349	8.5	Unknown	other	NONE	2.62E-05
Q80VJ2	SRA1_MOUSE	SRA1 (includes EG:10011)	(Q80VJ2) Steroid receptor RNA activator 1 (Steroid receptor RNA activator protein) (SRAP)	2	17.3	220	24325	6.6	Nucleus	transcription regulator	NONE	5.1E-05
Q80VJ3	RCL_MOUSE	C6ORF108	(Q80VJ3) c-Myc-responsive protein Rcl	2	8.1	173	18935	5.1	Nucleus	other	NONE	0.000162
Q80VP1	EPN1_MOUSE	EPN1	(Q80VP1) Epsin-1 (EPS-15-interacting protein 1) (Intersectin-EH-binding protein 1) (Ibp1)	2	9.2	566	59165	4.7	Plasma Membrane	other	NONE	2.98E-05
Q80W68	KIRR1_MOUSE	KIRREL	(Q80W68) Kin of IRRE-like protein 1 precursor (Kin of irregular chiasm-like protein 1) (Nephrin-like protein 1)	1	2	789	87176	5.9	Plasma Membrane	other	3	7.11E-06
Q80WC7	HRBL_MOUSE	HRBL	(Q80WC7) HIV-1 Rev-binding protein-like protein (Rev/Rex activation domain-binding protein related) (RAB-R)	2	9.6	479	48968	9.1	Nucleus	other	NONE	7.03E-05
Q80WJ7	LYRIC_MOUSE	MTDH	(Q80WJ7) Protein LYRIC (Lysine-rich CEACAM1 co-isolated protein) (3D3/LYRIC) (Metastasis adhesion protein) (Metadherin)	6	16.2	579	63846	9.3	Cytoplasm	other	1	0.000136
Q80WT5	AFTIN_MOUSE	AFTPH	(Q80WT5) Aftiphilin	3	7.6	931	101130	4.5	Cytoplasm	other	NONE	2.41E-05
Q80WW9	CT116_MOUSE	C20ORF116	(Q80WW9) Uncharacterized protein C20orf116 homolog precursor	1	5.4	315	35977	5.3	Extracellular Space	other	1	8.91E-05
Q80X50	UBP2L_MOUSE	UBAP2L	(Q80X50) Ubiquitin-associated protein 2-like	11	16	1107	116799	7.1	Unknown	other	NONE	9.63E-05
Q80X80	TMM24_MOUSE	TMEM24	(Q80X80) Transmembrane protein 24	2	5.4	706	76357	7.2	Unknown	other	1	2.39E-05
Q80X90	FLNB_MOUSE	FLNB	(Q80X90) Filamin-B (FLN-B) (Beta-filamin) (Actin-binding-like protein) (ABP-280-like protein)	14	9.6	2602	277750	5.7	Cytoplasm	other	NONE	4.75E-05
Q80XI3	IF4G3_MOUSE	EIF4G3	(Q80XI3) Eukaryotic translation initiation factor 4 gamma 3 (eIF-4-gamma 3) (eIF-4G 3) (eIF4G 3) (eIF-4-gamma II) (eIF4GII)	6	5.5	1579	174889	5.5	Cytoplasm	translation regulator	NONE	4.27E-05
Q80XI4	PI52B_MOUSE	PIP5K2B	(Q80XI4) Phosphatidylinositol-4-phosphate 5-kinase type-2 beta (EC 2.7.1.68) (Phosphatidylinositol-4-phosphate 5-kinase type II beta) (1-phosphatidylinositol-4-phosphate 5-kinase 2-beta) (PtdIns(4)P-5-kinase isoform 2-beta) (PIP5KII-beta) (Di	1	4.1	416	47319	7.3	Cytoplasm	kinase	NONE	1.35E-05
Q80XN0	BDH_MOUSE	BDH1	(Q80XN0) D-beta-hydroxybutyrate dehydrogenase, mitochondrial precursor (EC 1.1.1.30) (BDH) (3-hydroxybutyrate dehydrogenase)	11	24.8	343	38285	9	Cytoplasm	enzyme	NONE	0.000753
Q80Y14	GLRX5_MOUSE	GLRX5	(Q80Y14) Glutaredoxin-related protein 5	3	12.5	152	16292	6.5	Unknown	other	NONE	0.000222
Q80YR5	SAFB2_MOUSE	SAFB2	(Q80YR5) Scaffold attachment factor B2	3	3.5	991	111839	6.4	Unknown	other	NONE	4.53E-05
Q80ZI6	LRSM1_MOUSE	LRSAM1	(Q80ZI6) Ubiquitin ligase protein LRSAM1 (EC 6.3.2.-) (Leucine-rich repeat and sterile alpha motif-containing protein 1) (Tsg101-associated ligase)	2	3.6	727	83977	6.1	Cytoplasm	other	NONE	1.54E-05
Q80ZM8	CRLS1_MOUSE	CRLS1	(Q80ZM8) Cardiolipin synthetase (EC 2.7.8.-) (Cardiolipin synthase) (CLS)	2	6.6	303	32502	9.9	Cytoplasm	enzyme	7	5.56E-05
Q80ZX8	SPAG1_MOUSE	SPAG1	(Q80ZX8) Sperm-associated antigen 1 (Infertility-related sperm protein Spag-1) (TPR-containing protein involved in spermatogenesis) (TPIS)	2	2.9	901	100670	5.6	Cytoplasm	other	NONE	1.25E-05
Q810V0	MPP10_MOUSE	MPHOSPH10	(Q810V0) U3 small nucleolar ribonucleoprotein protein MPP10 (M phase phosphoprotein 10)	1	4.7	681	78675	4.9	Nucleus	other	NONE	8.24E-06
Q811D0	DLG1_MOUSE	DLG1	(Q811D0) Disks large homolog 1 (Synapse-associated protein 97) (SAP-97) (Embryo-dlg/synapse-associated protein 97) (E-dlg/SAP97)	4	7	905	100120	5.8	Plasma Membrane	kinase	NONE	4.34E-05

Q8BFR5	EFTU_MOUSE	TUFM	(Q8BFR5) Elongation factor Tu, mitochondrial precursor	27	44.9	452	49508	7.6	Cytoplasm	translation regulator	NONE	0.001056
Q8BFW7	LPP_MOUSE	LPP (includes EG:4026)	(Q8BFW7) Lipoma-preferred partner homolog	11	25.1	613	65891	7.4	Nucleus	other	NONE	0.000238
Q8BFZ9	SPFH2_MOUSE	SPFH2	(Q8BFZ9) SPFH domain-containing protein 2 precursor	10	29.4	340	37873	5.5	Plasma Membrane	other	1	0.000347
Q8BG05	ROA3_MOUSE	HNRPA3	(Q8BG05) Heterogeneous nuclear ribonucleoprotein A3 (hnRNP A3)	17	33.5	379	39652	9	Nucleus	other	NONE	0.001214
Q8BG32	PSD11_MOUSE	PSMD11	(Q8BG32) 26S proteasome non-ATPase regulatory subunit 11 (26S proteasome regulatory subunit S9) (26S proteasome regulatory subunit p44.5)	1	3.1	421	47306	6.5	Cytoplasm	other	NONE	1.33E-05
Q8BG81	PDIP3_MOUSE	POLDIP3	(Q8BG81) Polymerase delta-interacting protein 3	2	7.9	420	46133	10.1	Nucleus	other	NONE	2.67E-05
Q8BGD9	IF4B_MOUSE	EIF4B	(Q8BGD9) Eukaryotic translation initiation factor 4B (eIF-4B)	7	11.3	611	68840	5.7	Cytoplasm	translation regulator	NONE	0.000469
Q8BGN3	ENPP6_MOUSE	ENPP6	(Q8BGN3) Ectonucleotide pyrophosphatase/phosphodiesterase 6 precursor (EC 3.1.-.-) (E-NPP6) (NPP-6) [Contains: Ectonucleotide pyrophosphatase/phosphodiesterase 6 soluble form]	1	4.5	440	50618	7.3	Cytoplasm	enzyme	NONE	1.28E-05
Q8BGR2	LRC8D_MOUSE	LRRC8D	(Q8BGR2) Leucine-rich repeat-containing protein 8D	1	2.3	859	98113	7.4	Plasma Membrane	G-protein coupled receptor	3	1.31E-05
Q8BGS2	BOLA2_MOUSE	BOLA2	(Q8BGS2) BolA-like protein 2	1	22.1	86	10215	6.2	Unknown	other	NONE	0.000131
Q8BH43	WASF2_MOUSE	WASF2	(Q8BH43) Wiskott-Aldrich syndrome protein family member 2 (WASP-family protein member 2) (Protein WAVE-2)	2	6.4	497	54074	5.5	Cytoplasm	other	NONE	3.39E-05
Q8BH59	CMC1_MOUSE	SLC25A12 (includes EG:8604)	(Q8BH59) Calcium-binding mitochondrial carrier protein Aralar1 (Mitochondrial aspartate glutamate carrier 1) (Solute carrier family 25 member 12)	6	13.3	677	74570	8.3	Cytoplasm	transporter	NONE	9.12E-05
Q8BH95	ECHM_MOUSE	ECHS1	(Q8BH95) Enoyl-CoA hydratase, mitochondrial precursor (EC 4.2.1.17) (Short chain enoyl-CoA hydratase) (SCEH) (Enoyl-CoA hydratase 1)	18	53.8	290	31474	8.5	Cytoplasm	enzyme	NONE	0.001761
Q8BHJ5	TBL1R_MOUSE	TBL1XR1	(Q8BHJ5) F-box-like/WD repeat protein TBL1XR1 (Transducin beta-like 1X-related protein 1) (Nuclear receptor corepressor/HDAC3 complex subunit TBLR1) (TBL1-related protein 1)	1	4.5	514	55661	5.6	Plasma Membrane	enzyme	NONE	2.18E-05
Q8BHL8	PSMF1_MOUSE	PSMF1	(Q8BHL8) Proteasome inhibitor PI31 subunit	1	8.5	271	29664	5.3	Cytoplasm	other	NONE	2.07E-05
Q8BHN3	GANAB_MOUSE	GANAB	(Q8BHN3) Neutral alpha-glucosidase AB precursor (EC 3.2.1.84) (Glucosidase II subunit alpha) (Alpha glucosidase 2)	3	4.3	944	106911	6.1	Cytoplasm	enzyme	2	2.97E-05
Q8BHZ4	ZN592_MOUSE	ZNF592	(Q8BHZ4) Zinc finger protein 592 (Zfp-592)	1	1.7	1262	137534	8	Nucleus	other	NONE	4.45E-06
Q8BIQ5	CSTF2_MOUSE	CSTF2	(Q8BIQ5) Cleavage stimulation factor 64 kDa subunit (CSTF 64 kDa subunit) (CF-1 64 kDa subunit) (CstF-64)	1	4.8	580	61341	6.8	Nucleus	other	NONE	3.87E-05
Q8BJ64	CHDH_MOUSE	CHDH	(Q8BJ64) Choline dehydrogenase, mitochondrial precursor (EC 1.1.99.1) (CHD) (CDH)	9	15.1	596	66415	8.5	Cytoplasm	enzyme	NONE	0.000235
Q8BJF9	CHM2B_MOUSE	CHMP2B	(Q8BJF9) Charged multivesicular body protein 2b (Chromatin-modifying protein 2b) (CHMP2b)	2	11.3	213	23935	8.8	Cytoplasm	other	NONE	0.000105
Q8BJU0	SGTA_MOUSE	SGTA	(Q8BJU0) Small glutamine-rich tetratricopeptide repeat-containing protein A	3	9.5	315	34322	5.1	Cytoplasm	other	NONE	0.000214
Q8BJZ4	RT35_MOUSE	MRPS35	(Q8BJZ4) 28S ribosomal protein S35, mitochondrial precursor (S35mt) (MRP-S35)	3	17.8	320	35975	8.6	Cytoplasm	other	NONE	0.000105
Q8BK62	OLFL3_MOUSE	OLFML3	(Q8BK62) Olfactomedin-like protein 3 precursor	2	9.6	406	45798	6.4	Extracellular Space	other	NONE	8.3E-05
Q8BK64	AHSA1_MOUSE	AHSA1	(Q8BK64) Activator of 90 kDa heat shock protein ATPase homolog 1 (AHA1)	2	14.2	338	38117	5.5	Unknown	other	NONE	3.32E-05
Q8BKX1	BAIP2_MOUSE	BAIAP2	(Q8BKX1) Brain-specific angiogenesis inhibitor 1-associated protein 2 (BAI1-associated protein 2) (BAI-associated protein 2) (Insulin receptor substrate p53) (IRSp53) (Insulin receptor substrate protein of 53 kDa) (Insulin receptor tyrosine k	4	14.4	535	59237	9	Plasma Membrane	kinase	NONE	6.3E-05
Q8BKZ9	ODPX_MOUSE	PDHX	(Q8BKZ9) Pyruvate dehydrogenase protein X component, mitochondrial precursor (Dihydrolipoamide dehydrogenase-binding protein of pyruvate dehydrogenase complex) (Lipoyl-containing pyruvate dehydrogenase complex component X)	3	6.8	501	53999	7.8	Cytoplasm	enzyme	NONE	7.84E-05

Q8BL66	EEA1_MOUSE	EEA1	(Q8BL66) Early endosome antigen 1	15	15.6	1411	160914	5.8	Cytoplasm	other	NONE	0.000115
Q8BL97	SFRS7_MOUSE	SFRS7	(Q8BL97) Splicing factor, arginine/serine-rich 7	2	9.7	267	30818	11.9	Nucleus	other	NONE	8.41E-05
Q8BM72	STCH_MOUSE	STCH	(Q8BM72) Stress 70 protein chaperone microsome-associated 60 kDa protein precursor (Microsomal stress 70 protein ATPase core)	1	2.8	471	51709	5.6	Cytoplasm	other	NONE	2.38E-05
Q8BMB3	IF4E3_MOUSE	EIF4E2	(Q8BMB3) Eukaryotic translation initiation factor 4E type 3 (eIF4E type 3) (eIF-4E type 3) (mRNA cap-binding protein type 3) (Eukaryotic translation initiation factor 4E-like 3) (eIF4E-like protein 4E-LP)	1	6.5	245	28263	8.9	Cytoplasm	translation regulator	NONE	4.58E-05
Q8BMI0	FBX38_MOUSE	FBXO38	(Q8BMI0) F-box only protein 38 (Modulator of KLF7 activity) (MoKA)	1	1.6	1194	133928	6.1	Nucleus	other	NONE	4.7E-06
Q8BMK1	METL2_MOUSE	METTL2B	(Q8BMK1) Methyltransferase-like protein 2 (EC 2.1.1.-)	2	9	389	43912	6.2	Unknown	enzyme	1	2.89E-05
Q8BMP6	GCP60_MOUSE	ACBD3	(Q8BMP6) Golgi resident protein GCP60 (Acyl-CoA-binding domain-containing protein 3) (Golgi phosphoprotein 1) (GOLPH1) (Golgi complex-associated protein 1) (GOCAP1) (PBR- and PKA-associated protein 7) (Peripheral benzodiazepine receptor-assoc	2	8	524	60050	5.1	Cytoplasm	other	NONE	6.43E-05
Q8BMS4	COQ3_MOUSE	COQ3	(Q8BMS4) Hexaprenyldihydroxybenzoate methyltransferase, mitochondrial precursor (EC 2.1.1.114) (Dihydroxyhexaprenylbenzoate methyltransferase) (3,4-dihydroxy-5-hexaprenylbenzoate methyltransferase) (DHHB methyltransferase) (DHHB-MT) (DHHB-MTas	3	13	370	40957	7.9	Cytoplasm	enzyme	NONE	0.000106
Q8BN82	S17A5_MOUSE	SLC17A5	(Q8BN82) Sialin (Solute carrier family 17 member 5) (Sodium/sialic acid cotransporter)	1	4.2	495	54369	7.7	Plasma Membrane	transporter	10	1.13E-05
Q8BP40	PPA6_MOUSE	ACP6	(Q8BP40) Lysophosphatidic acid phosphatase type 6 precursor (EC 3.1.3.2) (Acid phosphatase 6, lysophosphatidic) (Acid phosphatase-like protein 1) (PACPL1)	6	17.7	418	47625	7.7	Unknown	phosphatase	NONE	0.000269
Q8BP47	SYNC_MOUSE	NARS	(Q8BP47) Asparaginyl-tRNA synthetase, cytoplasmic (EC 6.1.1.22) (Asparagine--tRNA ligase) (AsnRS)	2	9	547	63066	6	Cytoplasm	enzyme	NONE	3.08E-05
Q8BP67	RL24_MOUSE	RPL24	(Q8BP67) 60S ribosomal protein L24	5	26.8	157	17779	11.3	Cytoplasm	other	NONE	0.000572
Q8BP92	RCN2_MOUSE	RCN2	(Q8BP92) Reticulocalbin-2 precursor (Taipoxin-associated calcium-binding protein 49) (TCBP-49)	3	11.9	320	37271	4.4	Cytoplasm	other	1	7.02E-05
Q8BQZ5	CPSF4_MOUSE	CPSF4	(Q8BQZ5) Cleavage and polyadenylation specificity factor, 30 kDa subunit (CPSF 30 kDa subunit) (Clipper homolog) (Clipper/CPSF 30K)	1	10.4	211	23653	8.5	Nucleus	other	NONE	5.32E-05
Q8BRF7	SCFD1_MOUSE	SCFD1	(Q8BRF7) Sec1 family domain-containing protein 1 (Syntaxin-binding protein 1-like 2)	2	5.9	639	72323	6.4	Unknown	transporter	NONE	3.51E-05
Q8BTE0	CF057_MOUSE	C6ORF57	(Q8BTE0) Uncharacterized protein C6orf57 homolog precursor	1	25	104	11889	9.5	Unknown	other	NONE	5.4E-05
Q8BTM8	FLNA_MOUSE	FLNA (includes EG:2316)	(Q8BTM8) Filamin-A (Alpha-filamin) (Filamin-1) (Endothelial actin-binding protein) (Actin-binding protein 280) (ABP-280) (Nonmuscle filamin)	12	8.1	2646	281060	6	Cytoplasm	other	NONE	5.73E-05
Q8BU11	CN092_MOUSE	KIAA0737	(Q8BU11) Epidermal Langerhans cell protein LCP1	1	3.2	619	65991	5	Nucleus	other	NONE	1.81E-05
Q8BUK6	HOOK3_MOUSE	HOOK3	(Q8BUK6) Hook homolog 3	3	7.9	718	83218	5.2	Cytoplasm	other	NONE	2.35E-05
Q8BVE3	VATH_MOUSE	ATP6V1H (includes EG:51606)	(Q8BVE3) Vacuolar ATP synthase subunit H (EC 3.6.3.14) (V-ATPase H subunit) (Vacuolar proton pump H subunit)	5	13.7	483	55855	6.6	Cytoplasm	transporter	NONE	0.000139
Q8BVI4	DHPR_MOUSE	QDPR	(Q8BVI4) Dihydropteridine reductase (EC 1.5.1.34) (HDHPR) (Quinoid dihydropteridine reductase)	14	36.1	241	25570	7.8	Unknown	enzyme	NONE	0.001025
Q8BVU5	NUDT9_MOUSE	NUDT9	(Q8BVU5) ADP-ribose pyrophosphatase, mitochondrial precursor (EC 3.6.1.13) (ADP-ribose diphosphatase) (Adenosine diphosphoribose pyrophosphatase) (ADPR-PPase) (ADP-ribose phosphohydrolase) (Nucleoside diphosphate-linked moiety X motif 9) (Nud	3	7.7	350	38604	6.8	Cytoplasm	phosphatase	NONE	6.42E-05
Q8BWF0	SSDH_MOUSE	ALDH5A1	(Q8BWF0) Succinate semialdehyde dehydrogenase, mitochondrial precursor (EC 1.2.1.24) (NAD(+)-dependent succinic semialdehyde dehydrogenase)	7	22.9	523	55968	8.3	Cytoplasm	enzyme	NONE	0.000161

Q8BWM0	PGES2_MOUSE	PTGES2	(Q8BWM0) Prostaglandin E synthase 2 (EC 5.3.99.3) (Microsomal prostaglandin E synthase 2) (mPGES-2) (GATE-binding factor 1) (GBF-1) [Contains: Prostaglandin E synthase 2 truncated form]	6	23.2	384	43308	9	Cytoplasm	transcription regulator	NONE	0.000336
Q8BWT1	THIM_MOUSE	ACAA2	(Q8BWT1) 3-ketoacyl-CoA thiolase, mitochondrial (EC 2.3.1.16) (Beta-ketothiolase) (Acetyl-CoA acyltransferase) (Mitochondrial 3-oxoacyl-CoA thiolase)	44	67.3	397	41858	8.1	Cytoplasm	enzyme	NONE	0.004326
Q8BWW4	LARP4_MOUSE	LARP4	(Q8BWW4) La-related protein 4 (La ribonucleoprotein domain family member 4)	2	5.3	719	79763	6.4	Unknown	other	NONE	1.56E-05
Q8BX94	OSBL2_MOUSE	OSBPL2	(Q8BX94) Oxysterol-binding protein-related protein 2 (OSBP-related protein 2) (ORP-2)	1	5.6	484	55385	6.2	Cytoplasm	other	NONE	1.16E-05
Q8BXK9	CLIC5_MOUSE	CLIC5	(Q8BXK9) Chloride intracellular channel protein 5	9	36.7	251	28287	5.9	Cytoplasm	ion channel	NONE	0.001364
Q8BXR9	OSBL6_MOUSE	OSBPL6	(Q8BXR9) Oxysterol-binding protein-related protein 6 (OSBP-related protein 6) (ORP-6)	2	2.2	959	108920	7.3	Cytoplasm	other	NONE	1.76E-05
Q8BY87	UBP47_MOUSE	USP47	(Q8BY87) Ubiquitin carboxyl-terminal hydrolase 47 (EC 3.1.2.15) (Ubiquitin thioesterase 47) (Ubiquitin-specific-processing protease 47) (Deubiquitinating enzyme 47)	1	1.4	1376	157455	5.1	Unknown	peptidase	NONE	4.08E-06
Q8BYC6	TAOK3_MOUSE	TAOK3	(Q8BYC6) Serine/threonine-protein kinase TAO3 (EC 2.7.11.1) (Thousand and one amino acid protein 3)	1	2	898	105336	7.3	Cytoplasm	kinase	NONE	1.25E-05
Q8BYH8	CHD9_MOUSE	CHD9 (includes EG:80205)	(Q8BYH8) Chromodomain-helicase-DNA-binding protein 9 (EC 3.6.1.-) (ATP-dependent helicase CHD9) (CHD-9) (Peroxisomal proliferator-activated receptor A-interacting complex 320 kDa protein) (PPAR-alpha-interacting complex protein 320 kDa)	2	1	2885	323860	7.5	Unknown	other	1	3.89E-06
Q8BYU6	TOIP2_MOUSE	TOR1AIP2	(Q8BYU6) Torsin-1A-interacting protein 2	1	3.6	502	54496	4.9	Cytoplasm	other	1	1.12E-05
Q8BZN6	DOC10_MOUSE	DOCK10	(Q8BZN6) Dedicator of cytokinesis protein 10 (Fragment)	2	1.9	1816	207486	8	Unknown	other	NONE	6.18E-06
Q8C0D4	RHG12_MOUSE	ARHGAP12	(Q8C0D4) Rho-GTPase-activating protein 12	3	6.3	838	95352	7.6	Unknown	other	NONE	5.36E-05
Q8C0I1	ADAS_MOUSE	AGPS	(Q8C0I1) Alkyldihydroxyacetonephosphate synthase, peroxisomal precursor (EC 2.5.1.26) (Alkyl-DHAP synthase) (Alkylglycerone-phosphate synthase)	7	14.1	645	71684	7.5	Cytoplasm	enzyme	NONE	0.000157
Q8C0T5	SI1L1_MOUSE	SIPA1L1	(Q8C0T5) Signal-induced proliferation-associated 1-like protein 1	1	1.6	1782	197030	8.1	Cytoplasm	other	NONE	3.15E-06
Q8C1B7	SEP11_MOUSE	SPET11	(Q8C1B7) Septin-11	7	14	430	49564	6.7	Nucleus	other	NONE	0.000131
Q8C1D8	IWS1_MOUSE	IWS1	(Q8C1D8) IWS1 homolog (IWS1-like protein)	2	4	766	85248	4.7	Nucleus	enzyme	NONE	1.47E-05
Q8C2Q3	RBM14_MOUSE	RBM14 (includes EG:10432)	(Q8C2Q3) RNA-binding protein 14 (RNA-binding motif protein 14)	4	8.2	669	69449	9.7	Nucleus	transcription regulator	NONE	7.55E-05
Q8C570	RAE1L_MOUSE	RAE1	(Q8C570) mRNA-associated protein mrnp 41 (Rae1 protein homolog)	1	5.4	368	40965	7.8	Nucleus	other	NONE	1.53E-05
Q8C5Q4	GRSF1_MOUSE	GRSF1	(Q8C5Q4) G-rich sequence factor 1 (GRSF-1)	6	19.2	479	53076	6.7	Cytoplasm	other	NONE	8.2E-05
Q8C5W0	CLMN_MOUSE	CLMN	(Q8C5W0) Calmin	3	5.5	1052	117227	5	Cytoplasm	other	2	3.2E-05
Q8C7E9	CSTFT_MOUSE	CSTF2T	(Q8C7E9) Cleavage stimulation factor 64 kDa subunit, tau variant (CSTF 64 kDa subunit, tau variant) (CF-1 64 kDa subunit, tau variant) (TauCstF-64)	2	8.1	632	65862	7.3	Nucleus	other	NONE	2.66E-05
Q8C7Q4	RBM4_MOUSE	RBM4	(Q8C7Q4) RNA-binding protein 4 (RNA-binding motif protein 4) (RNA-binding motif protein 4a) (Lark homolog) (mLark)	1	4.4	361	40045	7.1	Nucleus	other	NONE	1.55E-05
Q8C7V3	UTP15_MOUSE	UTP15	(Q8C7V3) U3 small nucleolar RNA-associated protein 15 homolog (Src-associated protein SAW)	1	1.9	528	59375	8.8	Nucleus	other	NONE	2.13E-05
Q8C8U0	LIPB1_MOUSE	PPFIBP1	(Q8C8U0) Liprin-beta-1 (Protein tyrosine phosphatase receptor type f polypeptide-interacting protein-binding protein 1) (PTPRF-interacting protein-binding protein 1)	2	4.3	969	108540	5.5	Plasma Membrane	other	NONE	1.16E-05
Q8CAQ8	IMMT_MOUSE	IMMT	(Q8CAQ8) Mitochondrial inner membrane protein (Mitofilin)	23	31.4	757	83900	6.6	Cytoplasm	other	NONE	0.000334
Q8CAY6	THIC_MOUSE	ACAT2	(Q8CAY6) Acetyl-CoA acetyltransferase, cytosolic (EC 2.3.1.9) (Cytosolic acetoacetyl-CoA thiolase)	8	23.9	397	41298	7.5	Cytoplasm	enzyme	NONE	0.000212

Q8CBW3	ABI1_MOUSE	ABI1	(Q8CBW3) Abl interactor 1 (Abelson interactor 1) (Abi-1) (Spectrin SH3 domain-binding protein 1) (Eps8 SH3 domain-binding protein) (Eps8-binding protein) (e3B1) (Ablphilin-1)	2	5.6	480	52156	7.6	Cytoplasm	other	NONE	5.85E-05
Q8CBY8	DCTN4_MOUSE	DCTN4	(Q8CBY8) Dynactin subunit 4 (Dynactin subunit p62)	1	7.1	467	53057	7.7	Nucleus	other	NONE	1.2E-05
Q8CC35	SYNPO_MOUSE	SYNPO	(Q8CC35) Synaptopodin	4	7.1	929	99552	9.4	Cytoplasm	other	NONE	5.44E-05
Q8CCK0	H2AW_MOUSE	H2AFY2	(Q8CCK0) Core histone macro-H2A.2 (Histone macroH2A2) (mH2A2)	9	21.3	371	39961	9.7	Nucleus	other	NONE	0.000393
Q8CCS6	PABP2_MOUSE	PABPN1	(Q8CCS6) Polyadenylate-binding protein 2 (Poly(A)-binding protein 2) (Poly(A)-binding protein II) (PABII) (Polyadenylate-binding nuclear protein 1) (Nuclear poly(A)-binding protein 1)	2	21.6	301	32165	5.2	Nucleus	other	NONE	3.73E-05
Q8CDN6	TXNL1_MOUSE	TXNL1	(Q8CDN6) Thioredoxin-like protein 1 (32 kDa thioredoxin-related protein)	9	33	288	32106	5	Cytoplasm	enzyme	NONE	0.000565
Q8CEI1	BOLA3_MOUSE	BOLA3	(Q8CEI1) BolA-like protein 3	1	15.5	110	12228	9	Unknown	other	NONE	0.000102
Q8CFE3	RCOR1_MOUSE	RCOR1	(Q8CFE3) REST corepressor 1 (Protein CoREST)	1	3.4	477	52500	7	Nucleus	transcription regulator	NONE	2.35E-05
Q8CFX1	G6PE_MOUSE	H6PD	(Q8CFX1) GDH/6PGL endoplasmic bifunctional protein precursor [Includes: Glucose 1-dehydrogenase (EC 1.1.1.47) (Hexose-6-phosphate dehydrogenase); 6-phosphogluconolactonase (EC 3.1.1.31) (6PGL)]	2	3.5	789	88911	6.9	Cytoplasm	enzyme	NONE	1.42E-05
Q8CG76	ARK72_MOUSE	AKR7A2	(Q8CG76) Aflatoxin B1 aldehyde reductase member 2 (EC 1.-.-.-)	9	34.1	367	40598	8.1	Cytoplasm	enzyme	NONE	0.001025
Q8CGC7	SYEP_MOUSE	EPRS	(Q8CGC7) Bifunctional aminoacyl-tRNA synthetase [Includes: Glutamyl-tRNA synthetase (EC 6.1.1.17) (Glutamate--tRNA ligase); Prolyl-tRNA synthetase (EC 6.1.1.15) (Proline--tRNA ligase)]	5	3.9	1512	169936	7.6	Cytoplasm	enzyme	NONE	3.34E-05
Q8CGP5	H2A1F_MOUSE	HIST1H2AE (includes EG:3012)	(Q8CGP5) Histone H2A type 1-F	11	36.4	129	14030	11.1	Nucleus	other	NONE	0.018928
Q8CH18	CCAR1_MOUSE	CCAR1	(Q8CH18) Cell division cycle and apoptosis regulator protein 1 (Cell cycle and apoptosis regulatory protein 1) (CARP-1)	1	1.5	1146	132060	5.8	Nucleus	other	NONE	4.9E-06
Q8CHC4	SYNJ1_MOUSE	SYNJ1	(Q8CHC4) Synaptojanin-1 (EC 3.1.3.36) (Synaptic inositol-1,4,5-trisphosphate 5-phosphatase 1)	1	1.8	1574	172616	6.9	Cytoplasm	phosphatase	NONE	3.57E-06
Q8CHT0	AL4A1_MOUSE	ALDH4A1	(Q8CHT0) Delta-1-pyrroline-5-carboxylate dehydrogenase, mitochondrial precursor (EC 1.5.1.12) (P5C dehydrogenase) (Aldehyde dehydrogenase 4A1)	14	27.2	562	61811	8.4	Cytoplasm	enzyme	NONE	0.001908
Q8CI51	PDLI5_MOUSE	PDLIM5	(Q8CI51) PDZ and LIM domain protein 5 (Enigma homolog) (Enigma-like PDZ and LIM domains protein)	9	15.8	590	63198	8.2	Cytoplasm	other	NONE	0.000171
Q8CI85	CAH12_MOUSE	CA12	(Q8CI85) Carbonic anhydrase 12 precursor (EC 4.2.1.1) (Carbonic anhydrase XII) (Carbonate dehydratase XII) (CA-XII)	3	12.4	354	39695	8	Plasma Membrane	enzyme	2	0.000127
Q8CIB5	PKHC1_MOUSE	PLEKHC1	(Q8CIB5) Pleckstrin homology domain-containing family C member 1	1	7.1	680	77800	6.7	Cytoplasm	other	NONE	1.65E-05
Q8CIF4	BTD_MOUSE	BTD	(Q8CIF4) Biotinidase precursor (EC 3.5.1.12)	1	2.7	524	58601	5.8	Extracellular Space	enzyme	NONE	3.21E-05
Q8CIN4	PAK2_MOUSE	PAK2	(Q8CIN4) Serine/threonine-protein kinase PAK 2 (EC 2.7.11.1) (p21-activated kinase 2) (PAK-2)	5	13.2	524	57930	5.8	Cytoplasm	kinase	NONE	7.5E-05
Q8CJ96	RASF8_MOUSE	RASSF8	(Q8CJ96) Ras association domain-containing protein 8 (Carcinoma-associated protein HOJ-1 homolog)	2	5.7	419	48103	6	Unknown	other	NONE	4.02E-05
Q8JZN5	ACAD9_MOUSE	ACAD9	(Q8JZN5) Acyl-CoA dehydrogenase family member 9, mitochondrial precursor (EC 1.3.99.-) (ACAD-9)	2	6.4	625	68707	7.9	Cytoplasm	enzyme	NONE	8.98E-05
Q8JZQ9	IF39_MOUSE	EIF3S9	(Q8JZQ9) Eukaryotic translation initiation factor 3 subunit 9 (eIF-3 eta) (eIF3 p116)	5	13.6	803	91370	5	Cytoplasm	translation regulator	NONE	9.79E-05
Q8JZV9	DHRS6_MOUSE	BDH2	(Q8JZV9) Dehydrogenase/reductase SDR family member 6 precursor (EC 1.1.-.-)	8	24.9	245	26753	8	Extracellular Space	enzyme	NONE	0.000779
Q8JZX4	SPF45_MOUSE	RBM17	(Q8JZX4) Splicing factor 45 (45 kDa-splicing factor) (RNA-binding motif protein 17)	1	4.2	405	45304	5.8	Nucleus	other	NONE	1.39E-05
Q8K010	OPLA_MOUSE	OPLAH	(Q8K010) 5-oxoprolinase (EC 3.5.2.9) (5-oxo-L-prolinase) (Pyroglutamase) (5-OPase)	4	5.2	1288	137611	6.3	Unknown	enzyme	NONE	2.61E-05
Q8K072	REEP4_MOUSE	REEP4	(Q8K072) Receptor expression-enhancing protein 4	1	8.6	257	29691	9.8	Unknown	other	2	6.55E-05
Q8K0D5	EFG1_MOUSE	GFM1	(Q8K0D5) Elongation factor G 1, mitochondrial precursor (mEF-G 1) (Elongation factor G1)	4	8.3	751	83550	6.9	Cytoplasm	translation regulator	1	4.48E-05

Q8K0H5	TAF10_MOUSE	TAF10	(Q8K0H5) Transcription initiation factor TFIID subunit 10 (Transcription initiation factor TFIID 30 kDa subunit) (TAF(II)30) (TAFII-30) (mTAFII30)	1	11.5	218	21841	6.5	Nucleus	transcription regulator	NONE	5.15E-05
Q8K0Q5	RHG18_MOUSE	ARHGAP18	(Q8K0Q5) Rho-GTPase-activating protein 18	3	8.7	663	74930	6.7	Unknown	other	NONE	5.08E-05
Q8K0Z7	CCD44_MOUSE	CCDC44	(Q8K0Z7) Coiled-coil domain-containing protein 44	2	7.5	294	32314	8.1	Unknown	other	NONE	9.55E-05
Q8K157	GALM_MOUSE	GALM	(Q8K157) Aldose 1-epimerase (EC 5.1.3.3) (Galactose mutarotase)	4	21.1	342	37799	6.7	Cytoplasm	enzyme	NONE	9.85E-05
Q8K183	PDXK_MOUSE	PDXK	(Q8K183) Pyridoxal kinase (EC 2.7.1.35) (Pyridoxine kinase)	6	18.3	312	35015	6.3	Cytoplasm	kinase	NONE	0.000252
Q8K190	CF064_MOUSE	C6ORF64	(Q8K190) Protein C6orf64 homolog	1	10.6	188	20704	8.6	Unknown	other	1	5.97E-05
Q8K1I3	SPP24_MOUSE	SPP2	(Q8K1I3) Secreted phosphoprotein 24 precursor (Spp-24) (Secreted phosphoprotein 2)	1	5.9	203	23136	8.4	Extracellular Space	other	NONE	5.53E-05
Q8K1M6	DNM1L_MOUSE	DNM1L	(Q8K1M6) Dynamin-1-like protein (EC 3.6.5.5) (Dynamin-related protein 1) (Dynamin family member proline-rich carboxyl-terminal domain less) (Dymple)	4	10.1	742	82658	7.1	Cytoplasm	enzyme	NONE	4.54E-05
Q8K1N2	PHLB2_MOUSE	PHLDB2	(Q8K1N2) Pleckstrin homology-like domain family B member 2 (Protein LL5-beta)	4	5.8	1249	141485	7.7	Cytoplasm	other	NONE	2.7E-05
Q8K1Z0	COQ9_MOUSE	COQ9	(Q8K1Z0) Ubiquinone biosynthesis protein COQ9, mitochondrial precursor	6	22.4	313	35083	5.9	Cytoplasm	other	NONE	0.000843
Q8K202	RPF53_MOUSE	POLR1E	(Q8K202) DNA-directed RNA polymerase I-associated factor 53 kDa subunit (EC 2.7.7.6) (RNA polymerase I-associated factor 1)	1	3.3	482	54034	8.2	Nucleus	enzyme	NONE	6.99E-05
Q8K215	CF149_MOUSE	LYRM4	(Q8K215) Protein C6orf149 homolog	2	18.7	91	10855	10.1	Unknown	other	NONE	0.000247
Q8K284	TF3C1_MOUSE	GTF3C1	(Q8K284) General transcription factor 3C polypeptide 1 (Transcription factor IIIC-subunit alpha) (TF3C-alpha) (TFIIIC 220 kDa subunit) (TFIIIC220) (TFIIIC box B-binding subunit)	2	1.3	2101	237474	7.3	Nucleus	transcription regulator	2	5.34E-06
Q8K296	MTMR3_MOUSE	MTMR3	(Q8K296) Myotubularin-related protein 3 (EC 3.1.3.48)	2	2.7	1075	120226	5.8	Cytoplasm	phosphatase	NONE	1.04E-05
Q8K2B3	DHSA_MOUSE	SDHA	(Q8K2B3) Succinate dehydrogenase [ubiquinone] flavoprotein subunit, mitochondrial precursor (EC 1.3.5.1) (Fp) (Flavoprotein subunit of complex II)	21	30.9	664	72585	7.4	Cytoplasm	enzyme	NONE	0.001217
Q8K2H2	OTU6B_MOUSE	OTUD6B	(Q8K2H2) OTU domain-containing protein 6B	1	4.4	294	33758	5.5	Unknown	other	NONE	3.82E-05
Q8K2I3	FMO2_MOUSE	FMO2	(Q8K2I3) Dimethylaniline monooxygenase [N-oxide-forming] 2 (EC 1.14.13.8) (Pulmonary flavin-containing monooxygenase 2) (FMO 2) (Dimethylaniline oxidase 2)	1	2.8	534	60843	8.5	Cytoplasm	enzyme	2	1.05E-05
Q8K2K6	NUPL_MOUSE	HRB	(Q8K2K6) Nucleoporin-like protein RIP (HIV-1 Rev-binding protein homolog)	1	4.1	561	58043	8.6	Nucleus	other	NONE	2E-05
Q8K2Y7	RM47_MOUSE	MRPL47	(Q8K2Y7) 39S ribosomal protein L47, mitochondrial precursor (L47mt) (MRP-L47)	4	13.9	252	29726	10.2	Cytoplasm	other	NONE	0.000156
Q8K310	MATR3_MOUSE	MATR3	(Q8K310) Matrin-3	19	25.3	846	94630	6.3	Nucleus	other	NONE	0.000332
Q8K330	SSH3_MOUSE	SSH3	(Q8K330) Protein phosphatase Slingshot homolog 3 (EC 3.1.3.48) (EC 3.1.3.16) (SSH-3L) (mSSH-3L)	2	5.9	649	72227	5.6	Unknown	phosphatase	NONE	1.73E-05
Q8K3A0	HSC20_MOUSE	HSCB	(Q8K3A0) Co-chaperone protein HscB, mitochondrial precursor (Hsc20)	2	15.8	234	26645	7.4	Cytoplasm	other	NONE	7.2E-05
Q8K3G5	VRK3_MOUSE	VRK3	(Q8K3G5) Serine/threonine-protein kinase VRK3 (EC 2.7.11.1) (Vaccinia-related kinase 3)	2	7.5	453	50830	8.6	Unknown	kinase	NONE	4.96E-05
Q8K3J1	NUIM_MOUSE	NDUFS8	(Q8K3J1) NADH-ubiquinone oxidoreductase 23 kDa subunit, mitochondrial precursor (EC 1.6.5.3) (EC 1.6.99.3) (Complex I-23KD) (CI-23KD) (TYKY subunit)	12	33.5	212	24038	6.2	Cytoplasm	enzyme	NONE	0.001139
Q8K3J9	GPC5C_MOUSE	GPRC5C	(Q8K3J9) G-protein coupled receptor family C group 5 member C precursor (Retinoic acid-induced gene 3 protein) (RAIG-3)	3	7.5	440	48422	7.6	Plasma Membrane	G-protein coupled receptor	8	5.1E-05
Q8K3X6	ANS4B_MOUSE	ANKS4B	(Q8K3X6) Ankyrin repeat and SAM domain-containing protein 4B (Harmonin-interacting ankyrin repeat-containing protein) (Harp)	2	7.8	423	47975	5	Nucleus	transcription regulator	NONE	7.96E-05
Q8K3Z9	PO121_MOUSE	POM121	(Q8K3Z9) Nuclear envelope pore membrane protein POM 121 (Pore membrane protein of 121 kDa)	1	2.1	1200	121022	10.2	Nucleus	other	1	4.68E-06
Q8K4G5	ABLM1_MOUSE	ABLIM1	(Q8K4G5) Actin-binding LIM protein 1 (Actin-binding LIM protein family member 1) (abLIM-1)	2	3.8	861	96805	8.6	Cytoplasm	other	NONE	3.26E-05
Q8K4L3	SVIL_MOUSE	SVIL	(Q8K4L3) Supervillin (Archvillin) (p205/p250)	5	4.1	2170	243159	6.9	Plasma Membrane	other	NONE	2.07E-05
Q8K4M5	COMD1_MOUSE	COMMD1 (includes EG:150684)	(Q8K4M5) COMM domain-containing protein 1 (Protein Murr1)	3	19.1	188	20996	7.6	Nucleus	transporter	NONE	0.000328

Q8K4Z5	SF3A1_MOUSE	SF3A1	(Q8K4Z5) Splicing factor 3 subunit 1 (SF3a120)	4	6.8	791	88545	5.2	Nucleus	other	NONE	4.26E-05
Q8QZT1	THIL_MOUSE	ACAT1	(Q8QZT1) Acetyl-CoA acetyltransferase, mitochondrial precursor (EC 2.3.1.9) (Acetoacetyl-CoA thiolase)	38	60.4	424	44816	8.5	Cytoplasm	enzyme	NONE	0.005322
Q8R001	MARE2_MOUSE	MAPRE2	(Q8R001) Microtubule-associated protein RP/EB family member 2 (APC-binding protein EB2) (End-binding protein 2) (EB2)	4	15.3	326	36946	5.4	Cytoplasm	other	NONE	0.000224
Q8R016	BLMH_MOUSE	BLMH	(Q8R016) Bleomycin hydrolase (EC 3.4.22.40) (BLM hydrolase) (BMH) (BH)	2	7.5	455	52511	6.5	Cytoplasm	peptidase	NONE	2.47E-05
Q8R035	ICT1_MOUSE	ICT1	(Q8R035) Immature colon carcinoma transcript 1 protein precursor	4	13.6	206	23477	10.2	Unknown	other	NONE	0.000163
Q8R081	HNRPL_MOUSE	HNRPL	(Q8R081) Heterogeneous nuclear ribonucleoprotein L (hnRNP L)	9	19.8	555	60123	7.1	Nucleus	other	NONE	0.000172
Q8R086	SUOX_MOUSE	SUOX	(Q8R086) Sulfite oxidase, mitochondrial precursor (EC 1.8.3.1)	5	24.6	488	54048	6.1	Cytoplasm	enzyme	NONE	0.000115
Q8R0F8	FAHD1_MOUSE	FAHD1	(Q8R0F8) Fumarylacetoacetate hydrolase domain-containing protein 1 (EC 3.-.-.-)	7	30.4	227	25158	7.7	Unknown	enzyme	NONE	0.002102
Q8R0P4	CK067_MOUSE	C11ORF67	(Q8R0P4) Protein C11orf67 homolog	2	32	122	13244	8	Unknown	other	NONE	0.000184
Q8R0W0	EPIPL_MOUSE	EPPK1	(Q8R0W0) Epiplakin	2	0.9	6548	724679	6.1	Cytoplasm	other	NONE	1.71E-06
Q8R0Y6	FTHFD_MOUSE	ALDH1L1	(Q8R0Y6) 10-formyltetrahydrofolate dehydrogenase (EC 1.5.1.6) (10-FTHFDH) (Aldehyde dehydrogenase 1 family member L1)	17	22.7	902	98709	5.9	Cytoplasm	enzyme	NONE	0.000193
Q8R151	ZNFX1_MOUSE	ZNFX1	(Q8R151) NFX1-type zinc finger-containing protein 1	2	1.2	1909	218866	7.5	Nucleus	transcription regulator	NONE	5.88E-06
Q8R164	BPHL_MOUSE	BPHL	(Q8R164) Valacyclovir hydrolase precursor (EC 3.1.-.-) (VACVase) (Biphenyl hydrolase-like protein)	15	30.9	291	32851	8.9	Cytoplasm	enzyme	NONE	0.000868
Q8R180	ERO1A_MOUSE	ERO1L	(Q8R180) ERO1-like protein alpha precursor (EC 1.8.4.-) (ERO1-Lalpha) (Oxidoreductin-1-Lalpha) (Endoplasmic oxidoreductin-1-like protein) (ERO1-L)	1	3	464	54038	6.5	Cytoplasm	enzyme	1	2.42E-05
Q8R1G6	PDLI2_MOUSE	PDLIM2	(Q8R1G6) PDZ and LIM domain protein 2 (PDZ-LIM protein mystique)	5	28.7	349	37703	8.7	Cytoplasm	other	NONE	0.000161
Q8R1I1	UCR10_MOUSE	UCRC	(Q8R1I1) Ubiquinol-cytochrome c reductase complex 7.2 kDa protein (EC 1.10.2.2) (Cytochrome C1, nonheme 7 kDa protein) (Complex III subunit X) (7.2 kDa cytochrome c1-associated protein subunit)	3	37.5	64	7446	9.2	Cytoplasm	enzyme	NONE	0.000702
Q8R1N0	CCD16_MOUSE	CCDC16	(Q8R1N0) Coiled-coil domain-containing protein 16 (Ovus mutant candidate gene 1 protein)	1	6.1	363	40658	5.4	Nucleus	other	NONE	6.19E-05
Q8R1Q8	DC1L1_MOUSE	DYNC1LI1	(Q8R1Q8) Cytoplasmic dynein 1 light intermediate chain 1 (Dynein light intermediate chain 1, cytosolic) (Dynein light chain A) (DLC-A)	3	9.6	523	56614	6.4	Cytoplasm	other	NONE	0.00015
Q8R1U2	CGRE1_MOUSE	CGREF1	(Q8R1U2) Cell growth regulator with EF hand domain 1 (Cell growth regulatory gene 11 protein)	5	28.5	281	30847	4.3	Unknown	other	NONE	0.00016
Q8R1V4	TMED4_MOUSE	TMED4	(Q8R1V4) Transmembrane emp24 domain-containing protein 4 precursor (p26)	3	8.4	227	26022	8.2	Cytoplasm	transporter	2	0.000173
Q8R2V5	CENA2_MOUSE	CENTA2	(Q8R2V5) Centaurin-alpha 2	1	4.5	381	43989	9.1	Unknown	other	NONE	1.47E-05
Q8R2Y8	PTH2_MOUSE	PTRH2	(Q8R2Y8) Peptidyl-tRNA hydrolase 2, mitochondrial precursor (EC 3.1.1.29) (PTH 2)	3	22.1	181	19527	7.4	Cytoplasm	enzyme	NONE	0.000186
Q8R311	CTGE5_MOUSE	CTAGE5	(Q8R311) Cutaneous T-cell lymphoma-associated antigen 5 homolog (cTAGE-5 protein) (Meningioma-expressed antigen 6)	3	4.9	779	87719	5.1	Cytoplasm	enzyme	1	3.6E-05
Q8R317	UBQL1_MOUSE	UBQLN1	(Q8R317) Ubiquilin-1 (Protein linking IAP with cytoskeleton 1) (PLIC-1)	4	8.4	582	61976	4.9	Cytoplasm	other	NONE	8.68E-05
Q8R361	RFIP5_MOUSE	RAB11FIP5	(Q8R361) Rab11 family-interacting protein 5 (Rab11-FIP5) (Rab11-interacting protein Rip11)	9	17.1	645	69553	9.1	Cytoplasm	other	NONE	0.000131
Q8R3D1	TBC13_MOUSE	TBC1D13 (includes EG:54662)	(Q8R3D1) TBC1 domain family member 13	2	8.5	400	46453	5.4	Unknown	other	NONE	2.81E-05
Q8R3F5	FABD_MOUSE	MCAT	(Q8R3F5) Malonyl CoA-acyl carrier protein transacylase, mitochondrial precursor (EC 2.3.1.39) (MCT) (Mitochondrial malonyltransferase)	1	3.9	381	41928	8.1	Cytoplasm	enzyme	NONE	1.47E-05
Q8R3G1	PP1R8_MOUSE	PPP1R8	(Q8R3G1) Nuclear inhibitor of protein phosphatase 1 (NIPP-1) (Protein phosphatase 1 regulatory inhibitor subunit 8)	2	10.3	351	38528	7.4	Nucleus	phosphatase	NONE	9.6E-05
Q8R3N1	NOP14_MOUSE	C4ORF9	(Q8R3N1) Probable nucleolar complex protein 14	2	3.3	860	98774	7.8	Nucleus	other	NONE	1.31E-05
Q8R3P0	ACY2_MOUSE	ASPA	(Q8R3P0) Aspartoacylase (EC 3.5.1.15) (Aminoacylase-2) (ACY-2)	6	14.1	312	35345	6.6	Unknown	enzyme	NONE	0.000216

Q8R3Q6	CCD58_MOUSE	CCDC58	(Q8R3Q6) Coiled-coil domain-containing protein 58	2	19.4	144	16665	8.2	Unknown	other	NONE	0.000195
Q8R420	ABCA3_MOUSE	ABCA3	(Q8R420) ATP-binding cassette sub-family A member 3	1	1.2	1704	192000	7.1	Plasma Membrane	transporter	11	9.88E-06
Q8R4H2	ARHGC_MOUSE	ARHGEF12	(Q8R4H2) Rho guanine nucleotide exchange factor 12 (Leukemia-associated RhoGEF)	4	4.1	1543	172321	5.7	Cytoplasm	other	NONE	2.18E-05
Q8R4Y4	STAB1_MOUSE	STAB1	(Q8R4Y4) Stabilin-1 precursor (FEEL-1 protein)	2	1.1	2571	276255	6.7	Plasma Membrane	transporter	3	4.37E-06
Q8R5F7	IFIH1_MOUSE	IFIH1	(Q8R5F7) Interferon-induced helicase C domain-containing protein 1 (EC 3.6.1.-) (Interferon induced with helicase C domain protein 1) (Helicase with 2 CARD domains) (Helicard) (Melanoma differentiation-associated protein 5) (MDA-5)	1	1.7	1025	115971	6.2	Nucleus	enzyme	NONE	1.1E-05
Q8R5H6	WASF1_MOUSE	WASF1	(Q8R5H6) Wiskott-Aldrich syndrome protein family member 1 (WASP-family protein member 1) (Protein WAVE-1)	1	2.3	559	61509	6.4	Nucleus	other	NONE	1E-05
Q8VBT0	TXND1_MOUSE	TXNDC1	(Q8VBT0) Thioredoxin domain-containing protein 1 precursor	1	4.3	278	31396	5.3	Cytoplasm	enzyme	3	8.08E-05
Q8VC30	DAK_MOUSE	DAK	(Q8VC30) Dihydroxyacetone kinase (EC 2.7.1.29) (Glycerone kinase) (DHA kinase)	7	19.6	578	59691	6.9	Unknown	other	NONE	0.000136
Q8VCC2	EST1_MOUSE	CES1 (includes EG:12623)	(Q8VCC2) Liver carboxylesterase 1 precursor (EC 3.1.1.1) (Acyl coenzyme A:cholesterol acyltransferase) (ES-x)	1	2.1	565	62680	6	Cytoplasm	enzyme	NONE	4.97E-05
Q8VCF0	MAVS_MOUSE	KIAA1271	(Q8VCF0) Mitochondrial antiviral signaling protein (Interferon-beta promoter stimulator protein 1) (IPS-1) (Virus-induced signaling adapter) (CARD adapter inducing interferon-beta) (Cardif)	2	8.9	503	53399	6.4	Cytoplasm	other	2	3.35E-05
Q8VCH0	THIKB_MOUSE	ACAA1B	(Q8VCH0) 3-ketoacyl-CoA thiolase B, peroxisomal precursor (EC 2.3.1.16) (Beta-ketothiolase B) (Acetyl-CoA acyltransferase B) (Peroxisomal 3-oxoacyl-CoA thiolase B)	7	19.1	424	43995	8.5	Cytoplasm	enzyme	NONE	0.000384
Q8VCH8	UBXD2_MOUSE	UBXD2	(Q8VCH8) UBX domain-containing protein 2	2	4.9	506	56472	6.6	Unknown	other	NONE	4.44E-05
Q8VCI5	PEX19_MOUSE	PEX19	(Q8VCI5) Peroxisomal biogenesis factor 19 (Peroxin-19) (Peroxisomal farnesylated protein) (PxF)	3	18.4	299	32733	4.3	Cytoplasm	other	NONE	0.0003
Q8VCM7	FIBG_MOUSE	FGG	(Q8VCM7) Fibrinogen gamma chain precursor	1	5.3	436	49391	5.9	Extracellular Space	other	1	1.29E-05
Q8VCM8	NCLN_MOUSE	NCLN	(Q8VCM8) Nicalin precursor (Nicastrin-like protein)	2	4.6	563	62908	6.5	Cytoplasm	peptidase	2	1.99E-05
Q8VCN5	CGL_MOUSE	CTH	(Q8VCN5) Cystathionine gamma-lyase (EC 4.4.1.1) (Gamma-cystathionase)	2	11.1	398	43567	7.6	Cytoplasm	enzyme	NONE	8.46E-05
Q8VCR7	AB14B_MOUSE	ABHD14B	(Q8VCR7) Abhydrolase domain-containing protein 14B (CCG1-interacting factor B)	6	27.1	210	22451	6.3	Unknown	enzyme	NONE	0.000962
Q8VCT4	CES3_MOUSE	CES1 (includes EG:1066)	(Q8VCT4) Carboxylesterase 3 precursor (EC 3.1.1.1) (Triacylglycerol hydrolase) (TGH)	11	29.9	565	61788	6.6	Cytoplasm	enzyme	NONE	0.000725
Q8VD04	GRAP1_MOUSE	GRIPAP1	(Q8VD04) GRIP1-associated protein 1 (GRASP-1) (HCMV-interacting protein)	2	1.6	806	92715	5.2	Plasma Membrane	other	NONE	2.09E-05
Q8VD63	TSYL4_MOUSE	TSPYL4	(Q8VD63) Testis-specific Y-encoded-like protein 4 (TSPY-like 4)	2	10.6	406	44811	7	Unknown	other	NONE	9.68E-05
Q8VDC0	SYLM_MOUSE	LARS2	(Q8VDC0) Probable leucyl-tRNA synthetase, mitochondrial precursor (EC 6.1.1.4) (Leucine--tRNA ligase) (LeuRS)	3	3.8	902	101480	8.2	Cytoplasm	enzyme	NONE	4.36E-05
Q8VDD5	MYH9_MOUSE	MYH9	(Q8VDD5) Myosin-9 (Myosin heavy chain, nonmuscle IIa) (Nonmuscle myosin heavy chain IIa) (NMMHC II-a) (NMMHC-IIA) (Cellular myosin heavy chain, type A) (Nonmuscle myosin heavy chain-A) (NMMHC-A)	37	24.7	1959	226224	5.7	Cytoplasm	enzyme	NONE	0.000192
Q8VDJ3	VIGLN_MOUSE	HDLBP	(Q8VDJ3) Vigilin (High density lipoprotein-binding protein) (HDL-binding protein)	14	12	1268	141742	6.9	Nucleus	transporter	NONE	0.000137
Q8VDK1	NIT1_MOUSE	NIT1	(Q8VDK1) Nitrilase homolog 1 (EC 3.5.-.-)	6	15.2	323	35695	7.9	Unknown	enzyme	NONE	0.000278
Q8VDM4	PSD2_MOUSE	PSMD2	(Q8VDM4) 26S proteasome non-ATPase regulatory subunit 2 (26S proteasome regulatory subunit RPN1) (26S proteasome regulatory subunit S2) (26S proteasome subunit p97)	3	6.4	908	100203	5.2	Cytoplasm	other	NONE	3.09E-05
Q8VDM6	HNRL1_MOUSE	HNRPUL1	(Q8VDM6) Heterogeneous nuclear ribonucleoprotein U-like protein 1	3	4.7	859	96002	6.6	Nucleus	other	NONE	2.61E-05
Q8VDN2	AT1A1_MOUSE	ATP1A1	(Q8VDN2) Sodium/potassium-transporting ATPase alpha-1 chain precursor (EC 3.6.3.9) (Sodium pump 1) (Na+/K+ ATPase 1)	23	24.5	1023	112982	5.5	Plasma Membrane	transporter	8	0.000966
Q8VDP4	K1967_MOUSE	KIAA1967	(Q8VDP4) Protein KIAA1967 homolog	3	49 6	922	103002	5.3	Unknown	peptidase	NONE	3.04E-05

Q8VDQ1	ZADH1_MOUSE	ZADH1	(Q8VDQ1) Zinc-binding alcohol dehydrogenase domain-containing protein 1 (EC 1.-.-.-)	3	8.8	351	38054	5.5	Cytoplasm	enzyme	NONE	0.000128
Q8VDW0	DDX39_MOUSE	DDX39	(Q8VDW0) ATP-dependent RNA helicase DDX39 (EC 3.6.1.-) (DEAD box protein 39)	1	2.3	427	49067	5.7	Nucleus	enzyme	NONE	5.26E-05
Q8VDZ4	ZDHC5_MOUSE	ZDHHC5 (includes EG:25921)	(Q8VDZ4) Probable palmitoyltransferase ZDHHC5 (EC 2.3.1.-) (Zinc finger DHHC domain-containing protein 5) (DHHC-5)	2	5.2	715	77501	9	Nucleus	other	4	2.36E-05
Q8VE04	MOL2B_MOUSE	MOBKL2B	(Q8VE04) Mps one binder kinase activator-like 2B (Mob1 homolog 2b)	1	5.6	216	25519	8.4	Unknown	other	NONE	5.2E-05
Q8VE22	RT23_MOUSE	MRPS23	(Q8VE22) Mitochondrial ribosomal protein S23 (S23mt) (MRP-S23)	7	46.9	177	20348	8.6	Cytoplasm	other	NONE	0.000729
Q8VE37	RCC1_MOUSE	RCC1 (includes EG:1104)	(Q8VE37) Regulator of chromosome condensation (Chromosome condensation protein 1)	3	15	421	44931	8.1	Nucleus	other	NONE	0.000267
Q8VE47	UE1D1_MOUSE	UBE1DC1	(Q8VE47) Ubiquitin-activating enzyme E1 domain-containing protein 1 (UFM1-activating enzyme)	3	5.7	403	44786	5	Unknown	enzyme	NONE	4.18E-05
Q8VE70	PDC10_MOUSE	PDCD10	(Q8VE70) Programmed cell death protein 10 (TF-1 cell apoptosis-related protein 15)	1	7.1	212	24716	8.2	Unknown	other	NONE	2.65E-05
Q8VE92	RBM4B_MOUSE	RBM4B	(Q8VE92) RNA-binding protein 4B (RNA-binding motif protein 4B) (RNA-binding protein 30) (RNA-binding motif protein 30)	1	4.5	357	39991	6.7	Nucleus	other	NONE	3.14E-05
Q8VEE0	RPE_MOUSE	RPE	(Q8VEE0) Ribulose-phosphate 3-epimerase (EC 5.1.3.1) (Ribulose-5-phosphate-epimerase)	1	9.2	228	24945	5.4	Unknown	enzyme	NONE	2.46E-05
Q8VEE1	LMCD1_MOUSE	LMCD1	(Q8VEE1) LIM and cysteine-rich domains protein 1	3	10.7	365	40996	7.9	Cytoplasm	transcription regulator	NONE	6.15E-05
Q8VEH3	ARL8A_MOUSE	ARL8A	(Q8VEH3) ADP-ribosylation factor-like protein 8A (ADP-ribosylation factor-like protein 10B) (Novel small G protein indispensable for equal chromosome segregation 2)	2	20.4	186	21390	7.8	Unknown	enzyme	NONE	0.000121
Q8VEJ9	VPS4A_MOUSE	VPS4A	(Q8VEJ9) Vacuolar sorting protein 4a	2	7.8	437	48907	7.8	Cytoplasm	other	NONE	2.57E-05
Q8VEM8	MPCP_MOUSE	SLC25A3	(Q8VEM8) Phosphate carrier protein, mitochondrial precursor (PTP) (Solute carrier family 25 member 3)	10	17.1	357	39632	9.3	Cytoplasm	transporter	2	0.000692
Q8VH51	RNPC2_MOUSE	RBM39	(Q8VH51) RNA-binding region-containing protein 2 (Coactivator of activating protein 1 and estrogen receptors) (Coactivator of AP-1 and ERs) (Transcription coactivator CAPER)	2	6	530	59494	10.1	Nucleus	transcription regulator	NONE	5.3E-05
Q8VHE0	SEC63_MOUSE	SEC63	(Q8VHE0) Translocation protein SEC63 homolog	1	2.1	759	87711	5.4	Cytoplasm	transporter	3	7.4E-06
Q8VHE6	DYH5_MOUSE	DNAH5	(Q8VHE6) Ciliary dynein heavy chain 5 (Axonemal beta dynein heavy chain 5) (Mdnah5)	2	0.5	4621	527508	6.1	Unknown	enzyme	NONE	2.43E-06
Q8VHF2	MUCDL_MOUSE	MUCDHL	(Q8VHF2) Mucin and cadherin-like protein precursor (Mu protocadherin)	1	1.6	831	88208	5.1	Plasma Membrane	other	2	6.75E-06
Q8VHR5	P66B_MOUSE	GATAD2B	(Q8VHR5) Transcriptional repressor p66 beta (p66/p68) (GATA zinc finger domain-containing protein 2B)	1	3.4	594	65411	9.7	Nucleus	transcription regulator	NONE	9.45E-06
Q8VI36	PAXI_MOUSE	PXN	(Q8VI36) Paxillin	8	21	591	64476	6	Cytoplasm	other	NONE	0.000152
Q8VIJ6	SFPQ_MOUSE	SFPQ	(Q8VIJ6) Splicing factor, proline- and glutamine-rich (Polypyrimidine tract-binding protein-associated-splicing factor) (PTB-associated-splicing factor) (PSF) (DNA-binding p52/p100 complex, 100 kDa subunit)	3	8.3	699	75442	9.4	Nucleus	other	NONE	5.62E-05
Q8VIM4	BSND_MOUSE	BSND	(Q8VIM4) Barttin	7	24.1	307	33814	4.6	Plasma Membrane	ion channel	2	0.000256
Q91V41	RAB14_MOUSE	RAB14	(Q91V41) Ras-related protein Rab-14	7	27.6	214	23766	6.2	Cytoplasm	enzyme	NONE	0.000446
Q91V76	CK054_MOUSE	C11ORF54	(Q91V76) Ester hydrolase C11orf54 homolog (EC 3.1.-.-)	7	21	315	34996	6.3	Unknown	other	NONE	0.000428
Q91V92	ACLY_MOUSE	ACLY	(Q91V92) ATP-citrate synthase (EC 2.3.3.8) (ATP-citrate (pro-S-)-lyase) (Citrate cleavage enzyme)	2	2.1	1091	119728	7.4	Cytoplasm	enzyme	NONE	1.54E-05
Q91VA6	PDIP2_MOUSE	POLDIP2	(Q91VA6) Polymerase delta-interacting protein 2	2	12	368	41870	8.6	Nucleus	other	NONE	4.58E-05
Q91VC3	DDX48_MOUSE	EIF4A3	(Q91VC3) Probable ATP-dependent RNA helicase DDX48 (EC 3.6.1.-) (DEAD box protein 48)	2	3.9	410	46709	6.7	Nucleus	enzyme	NONE	8.21E-05
Q91VC4	PLVAP_MOUSE	PLVAP	(Q91VC4) Plasmalemma vesicle-associated protein (Plasmalemma vesicle protein 1) (PV-1) (MECA-32 antigen)	6	13.9	438	49933	8.4	Plasma Membrane	other	1	0.000205
Q91VD9	NUAM_MOUSE	NDUFS1	(Q91VD9) NADH-ubiquinone oxidoreductase 75 kDa subunit, mitochondrial precursor (EC 1.6.5.3) (EC 1.6.99.3) (Complex I-75Kd) (CI-75Kd)	13	22.4	727	79749	5.7	Cytoplasm	enzyme	NONE	0.000232
Q91VH2	SNX9_MOUSE	SNX9	(Q91VH2) Sorting nexin-9	2	506.2	595	66546	5.5	Cytoplasm	transporter	NONE	5.66E-05

Q91VH6	CB004_MOUSE	C2ORF4	(Q91VH6) Protein C2orf4 homolog	1	10.4	297	33692	7.2	Unknown	other	NONE	3.78E-05
Q91VI7	RINI_MOUSE	RNH1	(Q91VI7) Ribonuclease inhibitor (Ribonuclease/angiogenin inhibitor 1)	3	12.5	456	49817	4.8	Cytoplasm	other	NONE	8.62E-05
Q91VJ5	PQBP1_MOUSE	PQBP1	(Q91VJ5) Polyglutamine-binding protein 1 (Polyglutamine tract-binding protein 1) (PQBP-1) (38 kDa nuclear protein containing a WW domain) (Npw38)	1	6.8	263	30597	6.2	Nucleus	transcription regulator	NONE	4.27E-05
Q91VL8	TE2IP_MOUSE	TERF2IP	(Q91VL8) Telomeric repeat-binding factor 2-interacting protein 1 (TRF2-interacting telomeric protein Rap1)	2	11.2	393	43353	4.8	Nucleus	other	NONE	2.86E-05
Q91VM9	IPYR2_MOUSE	PPA2	(Q91VM9) Inorganic pyrophosphatase 2, mitochondrial precursor (EC 3.6.1.1) (PPase 2)	11	35.2	330	38115	7	Cytoplasm	enzyme	NONE	0.000391
Q91VN4	CHCH6_MOUSE	CHCHD6	(Q91VN4) Coiled-coil-helix-coiled-coil-helix domain-containing protein 6	4	9.5	273	29799	8.2	Unknown	other	NONE	0.000164
Q91VR2	ATPG_MOUSE	ATP5C1	(Q91VR2) ATP synthase gamma chain, mitochondrial precursor (EC 3.6.3.14)	9	21.1	298	32886	9	Cytoplasm	transporter	NONE	0.003673
Q91VR5	DDX1_MOUSE	DDX1	(Q91VR5) ATP-dependent RNA helicase DDX1 (EC 3.6.1.-) (DEAD box protein 1)	1	2.8	740	82500	7.2	Nucleus	enzyme	NONE	3.79E-05
Q91VS8	FARP2_MOUSE	FARP2	(Q91VS8) FERM, RhoGEF and pleckstrin domain-containing protein 2 (FERM domain including RhoGEF) (FIR)	2	3	1065	121297	8.4	Cytoplasm	other	NONE	1.58E-05
Q91VW3	SH3L3_MOUSE	SH3BGRL3	(Q91VW3) SH3 domain-binding glutamic acid-rich-like protein 3	4	21.5	93	10477	5.1	Nucleus	other	NONE	0.000362
Q91VW5	GOGA4_MOUSE	GOLGA4	(Q91VW5) Golgin subfamily A member 4 (tGolgin-1)	4	3.1	2238	257561	5.4	Cytoplasm	other	NONE	1.76E-05
Q91W39	NCOA5_MOUSE	NCOA5	(Q91W39) Nuclear receptor coactivator 5 (NCoA-5) (Coactivator independent of AF-2) (CIA)	3	9	579	65319	9.8	Nucleus	other	NONE	4.85E-05
Q91W50	CSDE1_MOUSE	CSDE1	(Q91W50) Cold shock domain-containing protein E1	1	1.5	798	88791	6.4	Cytoplasm	enzyme	NONE	2.11E-05
Q91WD2	TRPV6_MOUSE	TRPV6	(Q91WD2) Transient receptor potential cation channel subfamily V member 6 (TrpV6) (Epithelial calcium channel 2) (ECaC2) (Calcium transport protein 1) (CaT1)	2	5.6	727	83195	6.8	Plasma Membrane	ion channel	5	1.54E-05
Q91WD5	NUCM_MOUSE	NDUFS2	(Q91WD5) NADH-ubiquinone oxidoreductase 49 kDa subunit, mitochondrial precursor (EC 1.6.5.3) (EC 1.6.99.3) (Complex I-49KD) (CI-49KD)	4	15.8	463	52626	7	Cytoplasm	enzyme	NONE	7.27E-05
Q91WJ8	FUBP1_MOUSE	FUBP1	(Q91WJ8) Far upstream element-binding protein 1 (FUSE-binding protein 1) (FBP)	5	12.1	651	68540	7.9	Nucleus	transcription regulator	NONE	6.9E-05
Q91WK0	LRRF2_MOUSE	LRRFIP2 (includes EG:9209)	(Q91WK0) Leucine-rich repeat flightless-interacting protein 2 (LRR FLI-II-interacting protein 2)	3	10.8	415	47148	5.7	Unknown	other	NONE	5.41E-05
Q91WS0	CJ070_MOUSE	ZCD1	(Q91WS0) Protein C10orf70 homolog	9	46.3	108	12097	9.1	Cytoplasm	other	1	0.001143
Q91WS2	NALP6_MOUSE	NLRP6	(Q91WS2) NACHT, LRR and PYD-containing protein 6 (PYRIN-containing APAF1-like protein 5-like)	1	1.8	843	94592	8.2	Plasma Membrane	G-protein coupled receptor	1	3.33E-05
Q91WU5	AS3MT_MOUSE	AS3MT	(Q91WU5) Arsenite methyltransferase (EC 2.1.1.137) (S-adenosyl-L-methionine:arsenic(III) methyltransferase) (Methylarsonite methyltransferase)	7	25.5	376	41793	6.1	Cytoplasm	enzyme	NONE	0.000284
Q91X17	UROM_MOUSE	UMOD	(Q91X17) Uromodulin precursor (Tamm-Horsfall urinary glycoprotein) (THP)	2	5.6	642	70845	4.9	Extracellular Space	other	2	2.62E-05
Q91X52	DCXR_MOUSE	DCXR	(Q91X52) L-xylulose reductase (EC 1.1.1.10) (XR) (Dicarbonyl/L-xylulose reductase)	4	21.3	244	25746	7.3	Cytoplasm	enzyme	NONE	0.000207
Q91X72	HEMO_MOUSE	HPX	(Q91X72) Hemopexin precursor	2	5.2	460	51341	7.8	Extracellular Space	transporter	1	2.44E-05
Q91X78	SPFH1_MOUSE	SPFH1	(Q91X78) SPFH domain-containing protein 1 precursor (KE04 protein homolog)	2	7.8	346	38937	7.2	Plasma Membrane	other	1	0.00013
Q91XE4	ACY3_MOUSE	ACY3	(Q91XE4) Aspartoacylase-2 (EC 3.5.1.15) (Aminoacylase-3) (ACY-3) (Acylase III) (Hepatitis C virus core-binding protein 1) (HCBP1)	12	54.1	318	35286	5.5	Unknown	enzyme	NONE	0.001359
Q91XF0	PNPO_MOUSE	PNPO	(Q91XF0) Pyridoxine-5'-phosphate oxidase (EC 1.4.3.5) (Pyridoxamine-phosphate oxidase)	2	14.9	261	30114	8.2	Unknown	enzyme	NONE	0.000108
Q91XV3	BASP_MOUSE	BASP1	(Q91XV3) Brain acid soluble protein 1 (BASP1 protein) (Neuronal axonal membrane protein NAP-22) (22 kDa neuronal tissue-enriched acidic protein)	4	38.7	225	21955	4.5	Plasma Membrane	other	NONE	0.000599
Q91Y97	ALDOB_MOUSE	ALDOB	(Q91Y97) Fructose-bisphosphate aldolase B (EC 4.1.2.13) (Liver-type aldolase) (Aldolase 2)	25	38	363	39376	8.3	Cytoplasm	enzyme	NONE	0.010298
Q91YD6	VILL_MOUSE	VILL	(Q91YD6) Villin-like protein (EF-6)	2	1.9	859	96509	6.3	Unknown	other	NONE	1.31E-05

Q91YD9	WASL_MOUSE	WASL	(Q91YD9) Neural Wiskott-Aldrich syndrome protein (N-WASP)	2	5.8	501	54274	7.9	Cytoplasm	other	NONE	3.36E-05
Q91YE8	SYNP2_MOUSE	SYNPO2	(Q91YE8) Synaptopodin-2 (Myopodin)	1	3.3	1087	116527	7.4	Cytoplasm	other	NONE	5.16E-06
Q91YI0	ARLY_MOUSE	ASL	(Q91YI0) Argininosuccinate lyase (EC 4.3.2.1) (Arginosuccinase) (ASAL)	8	20.3	464	51739	7	Cytoplasm	enzyme	NONE	0.000351
Q91YJ2	SNX4_MOUSE	SNX4	(Q91YJ2) Sorting nexin-4	7	12.9	450	51778	5.8	Cytoplasm	transporter	NONE	0.00015
Q91YJ5	IF2M_MOUSE	MTIF2	(Q91YJ5) Translation initiation factor IF-2, mitochondrial precursor (IF-2Mt) (IF-2(Mt))	1	2.3	727	81347	7.1	Cytoplasm	translation regulator	NONE	1.54E-05
Q91YN0	CL004_MOUSE	C12ORF4	(Q91YN0) Protein C12orf4 homolog	2	5.8	552	63644	6.2	Unknown	other	NONE	2.03E-05
Q91YP0	L2HDH_MOUSE	L2HGDH	(Q91YP0) L-2-hydroxyglutarate dehydrogenase, mitochondrial precursor (EC 1.1.99.2) (Duranin)	4	11.4	464	50899	8.3	Unknown	enzyme	NONE	9.68E-05
Q91YQ5	RIB1_MOUSE	RPN1	(Q91YQ5) Dolichyl-diphosphooligosaccharide--protein glycosyltransferase 67 kDa subunit precursor (EC 2.4.1.119) (Ribophorin I) (RPN-I)	8	18.8	608	68528	6.5	Cytoplasm	enzyme	2	0.000194
Q91YR1	TWF1_MOUSE	TWF1	(Q91YR1) Twinfilin-1 (Protein A6)	6	20	350	40079	6.7	Cytoplasm	kinase	NONE	0.000337
Q91YR7	PRP6_MOUSE	PRPF6	(Q91YR7) Pre-mRNA-processing factor 6 homolog (U5 snRNP-associated 102 kDa protein) (U5-102 kDa protein)	2	3.3	941	106722	8.1	Nucleus	transcription regulator	NONE	2.98E-05
Q91YR9	LTB4D_MOUSE	LTB4DH	(Q91YR9) NADP-dependent leukotriene B4 12-hydroxydehydrogenase (EC 1.3.1.74) (15-oxoprostaglandin 13-reductase) (EC 1.3.1.48)	2	7.3	329	35560	7.9	Cytoplasm	enzyme	NONE	5.12E-05
Q91YT0	NUBM_MOUSE	NDUFV1	(Q91YT0) NADH-ubiquinone oxidoreductase 51 kDa subunit, mitochondrial precursor (EC 1.6.5.3) (EC 1.6.99.3) (Complex I-51KD) (CI-51KD) (NADH dehydrogenase flavoprotein 1)	7	13.6	464	50834	8.2	Cytoplasm	enzyme	NONE	0.000327
Q91YW3	DNJC3_MOUSE	DNAJC3	(Q91YW3) DnaJ homolog subfamily C member 3 (Interferon-induced, double-stranded RNA-activated protein kinase inhibitor) (Protein kinase inhibitor p58) (Protein kinase inhibitor of 58 kDa)	5	19.2	504	57464	5.8	Cytoplasm	other	NONE	0.000123
Q91Z38	TTC1_MOUSE	TTC1	(Q91Z38) Tetratricopeptide repeat protein 1 (TPR repeat protein 1)	1	6.2	292	33263	5	Unknown	other	NONE	7.69E-05
Q91Z53	GRHPR_MOUSE	GRHPR	(Q91Z53) Glyoxylate reductase/hydroxypyruvate reductase (EC 1.1.1.79)	4	24.1	328	35329	7.6	Cytoplasm	enzyme	NONE	6.85E-05
Q91ZA3	PCCA_MOUSE	PCCA	(Q91ZA3) Propionyl-CoA carboxylase alpha chain, mitochondrial precursor (EC 6.4.1.3) (PCCase subunit alpha) (Propanoyl-CoA:carbon dioxide ligase subunit alpha)	16	24.3	724	79922	7.2	Cytoplasm	enzyme	NONE	0.00024
Q91ZJ5	UGPA2_MOUSE	UGP2	(Q91ZJ5) UTP--glucose-1-phosphate uridylyltransferase 2 (EC 2.7.7.9) (UDP-glucose pyrophosphorylase 2) (UDPGP 2) (UGPase 2)	3	8.3	507	56848	7.6	Cytoplasm	enzyme	NONE	5.54E-05
Q91ZR1	RAB4B_MOUSE	RAB4B	(Q91ZR1) Ras-related protein Rab-4B	1	6.1	213	23629	6	Plasma Membrane	enzyme	NONE	2.64E-05
Q91ZR2	SNX18_MOUSE	SNAG1	(Q91ZR2) Sorting nexin-18 (Sorting nexin-associated Golgi protein 1)	3	5.7	614	67904	6.7	Cytoplasm	transporter	NONE	5.49E-05
Q91ZU6	BPA1_MOUSE	DST	(Q91ZU6) Bullous pemphigoid antigen 1, isoforms 1/2/3/4 (BPA) (Hemidesmosomal plaque protein) (Dystonia musculorum protein) (Dystonin)	5	1	7389	833654	5.3	Plasma Membrane	other	NONE	4.56E-06
Q91ZU8	BPAEA_MOUSE	DST	(Q91ZU8) Bullous pemphigoid antigen 1, isoform 5 (BPA) (Hemidesmosomal plaque protein) (Dystonia musculorum protein) (Dystonin)	3	1	2611	301691	6.5	Plasma Membrane	other	NONE	1.07E-05
Q91ZV0	MIA2_MOUSE	MIA2	(Q91ZV0) Melanoma inhibitory activity protein 2 precursor	1	4.3	517	57777	4.1	Extracellular Space	other	1	1.09E-05
Q920Q6	MSI2H_MOUSE	MSI2	(Q920Q6) RNA-binding protein Musashi homolog 2 (Musashi-2)	1	7.8	346	36939	8.5	Cytoplasm	other	NONE	1.62E-05
Q921F2	TADBP_MOUSE	TARDBP	(Q921F2) TAR DNA-binding protein 43 (TDP-43)	4	12.3	414	44548	6.7	Nucleus	transcription regulator	NONE	0.000149
Q921G7	ETFD_MOUSE	ETFDH	(Q921G7) Electron transfer flavoprotein-ubiquinone oxidoreductase, mitochondrial precursor (EC 1.5.5.1) (ETF-QO) (ETF-ubiquinone oxidoreductase) (ETF dehydrogenase) (Electron-transferring-flavoprotein dehydrogenase)	4	11.9	616	68091	7.6	Cytoplasm	enzyme	NONE	0.000164
Q921H8	THIKA_MOUSE	ACAA1	(Q921H8) 3-ketoacyl-CoA thiolase A, peroxisomal precursor (EC 2.3.1.16) (Beta-ketothiolase A) (Acetyl-CoA acyltransferase A) (Peroxisomal 3-oxoacyl-CoA thiolase A)	21	48.1	424	43953	8.4	Cytoplasm	enzyme	NONE	0.001337
Q921I1	TRFE_MOUSE	TF	(Q921I1) Serotransferrin precursor (Transferrin) (Siderophilin) (Beta-1-metal-binding globulin)	11	15.9	697	76724	7.2	Extracellular Space	other	1	0.00025

Q921T2	TOIP1_MOUSE	TOR1AIP1	(Q921T2) Torsin-1A-interacting protein 1 (Lamina-associated polypeptide 1B)	2	6.2	520	58415	7.1	Nucleus	other	1	2.16E-05
Q922B1	LRP16_MOUSE	LRP16	(Q922B1) Protein LRP16	2	7.1	323	35295	8.9	Unknown	other	NONE	3.48E-05
Q922B2	SYD_MOUSE	DARS	(Q922B2) Aspartyl-tRNA synthetase (EC 6.1.1.12) (Aspartate--tRNA ligase) (AspRS)	4	11.2	501	57117	6.5	Cytoplasm	enzyme	NONE	4.48E-05
Q922D8	C1TC_MOUSE	MTHFD1	(Q922D8) C-1-tetrahydrofolate synthase, cytoplasmic (C1-THF synthase) [Includes: Methylenetetrahydrofolate dehydrogenase (EC 1.5.1.5); Methenyltetrahydrofolate cyclohydrolase (EC 3.5.4.9); Formyltetrahydrofolate synthetase (EC 6.3.4.3)]	7	10	934	101124	7.1	Cytoplasm	enzyme	NONE	8.41E-05
Q922E6	K0971_MOUSE	FASTKD2	(Q922E6) Protein KIAA0971 homolog	2	5.1	689	78948	8.9	Unknown	other	NONE	2.44E-05
Q922F4	TBB6_MOUSE	TUBB6	(Q922F4) Tubulin beta-6 chain	2	4.9	447	50090	4.9	Unknown	other	NONE	6.28E-05
Q922H2	PDK3_MOUSE	PDK3	(Q922H2) [Pyruvate dehydrogenase [lipoamide]] kinase isozyme 3, mitochondrial precursor (EC 2.7.11.2) (Pyruvate dehydrogenase kinase isoform 3)	2	8.9	415	47923	8.8	Cytoplasm	kinase	NONE	4.06E-05
Q922Q8	LRC59_MOUSE	LRRC59	(Q922Q8) Leucine-rich repeat-containing protein 59	6	25.4	307	34877	9.5	Unknown	other	1	0.00053
Q922R8	PDIA6_MOUSE	PDIA6	(Q922R8) Protein disulfide-isomerase A6 precursor (EC 5.3.4.1) (Thioredoxin domain-containing protein 7)	8	32.3	440	48100	5.1	Cytoplasm	enzyme	1	0.000523
Q922U1	PRPF3_MOUSE	PRPF3	(Q922U1) U4/U6 small nuclear ribonucleoprotein Prp3 (Pre-mRNA-splicing factor 3)	4	10	683	77455	9.5	Nucleus	other	NONE	8.22E-05
Q922Y1	U33K_MOUSE	LOC51035	(Q922Y1) UBA/UBX 33.3 kDa protein	3	23.6	297	33573	5.3	Unknown	other	NONE	0.000132
Q923D2	BLVRB_MOUSE	BLVRB	(Q923D2) Flavin reductase (EC 1.5.1.30) (FR) (NADPH-dependent diaphorase) (NADPH-flavin reductase) (FLR) (Biliverdin reductase B) (EC 1.3.1.24) (BVR-B) (Biliverdin-IX beta-reductase)	3	22.4	205	22066	7	Cytoplasm	enzyme	NONE	8.21E-05
Q923D5	WBP11_MOUSE	WBP11	(Q923D5) WW domain-binding protein 11 (WBP-11)	3	6.6	641	69875	8.4	Nucleus	phosphatase	NONE	2.63E-05
Q923I7	SC5A2_MOUSE	SLC5A2	(Q923I7) Sodium/glucose cotransporter 2 (Na(+)/glucose cotransporter 2) (Low affinity sodium-glucose cotransporter)	2	4.9	670	73008	7.7	Plasma Membrane	transporter	14	3.35E-05
Q924A2	CIC_MOUSE	CIC	(Q924A2) Protein capicua homolog	1	1.7	1606	163894	8.6	Nucleus	transcription regulator	NONE	3.5E-06
Q924D0	RT4I1_MOUSE	RTN4IP1	(Q924D0) Reticulon-4-interacting protein 1, mitochondrial precursor (NOGO-interacting mitochondrial protein)	1	4.8	396	43371	9.2	Cytoplasm	enzyme	NONE	2.83E-05
Q924M7	MANA_MOUSE	MPI	(Q924M7) Mannose-6-phosphate isomerase (EC 5.3.1.8) (Phosphomannose isomerase) (PMI) (Phosphohexomutase)	1	6.4	423	46575	6	Cytoplasm	enzyme	NONE	2.65E-05
Q924N4	S12A6_MOUSE	SLC12A6	(Q924N4) Solute carrier family 12 member 6 (Electroneutral potassium-chloride cotransporter 3) (K-Cl cotransporter 3)	1	1.6	1150	127497	7.1	Plasma Membrane	transporter	13	9.76E-06
Q924T2	RT02_MOUSE	MRPS2	(Q924T2) Mitochondrial 28S ribosomal protein S2 (S2mt) (MRP-S2)	1	7.2	291	32313	9.1	Cytoplasm	other	NONE	7.72E-05
Q925B0	PAWR_MOUSE	PAWR	(Q925B0) PRKC apoptosis WT1 regulator protein (Prostate apoptosis response 4 protein) (Par-4) (Fragment)	1	16.8	197	22686	5.5	Nucleus	transcription regulator	NONE	5.7E-05
Q925F2	ESAM_MOUSE	ESAM	(Q925F2) Endothelial cell-selective adhesion molecule precursor	1	5.8	394	41810	9.3	Plasma Membrane	other	3	2.85E-05
Q925H1	TRPS1_MOUSE	TRPS1	(Q925H1) Zinc finger transcription factor Trps1	1	1.5	1281	141034	7.6	Nucleus	transcription regulator	NONE	1.31E-05
Q925I1	ATAD3_MOUSE	ATAD3A	(Q925I1) ATPase family AAA domain-containing protein 3 (AAA-ATPase TOB3)	5	10.5	591	66742	9.3	Nucleus	other	NONE	8.55E-05
Q93092	TALDO_MOUSE	TALDO1	(Q93092) Transaldolase (EC 2.2.1.2)	11	25.5	337	37387	7	Cytoplasm	enzyme	NONE	0.000466
Q99020	ROAA_MOUSE	HNRPAB	(Q99020) Heterogeneous nuclear ribonucleoprotein A/B (hnRNP A/B) (CArG-binding factor-A) (CBF-A)	5	19.3	285	30831	7.9	Nucleus	enzyme	NONE	0.000571
Q99J21	MCLN1_MOUSE	MCOLN1	(Q99J21) Mucolipin-1 (Mucolipidin)	1	4.8	580	65506	7.6	Cytoplasm	ion channel	6	1.94E-05
Q99J36	THUM1_MOUSE	THUMPD1	(Q99J36) THUMP domain-containing protein 1	1	4.3	350	38885	6.1	Unknown	other	NONE	3.21E-05
Q99J94	SO1A6_MOUSE	SLCO1A6	(Q99J94) Solute carrier organic anion transporter family member 1A6 (Solute carrier family 21 member 13) (Kidney-specific organic anion-transporting polypeptide 5) (OATP-5)	2	4.8	670	74145	8	Plasma Membrane	transporter	11	1.68E-05
Q99J99	THTM_MOUSE	MPST	(Q99J99) 3-mercaptopyruvate sulfurtransferase (EC 2.8.1.2) (MST)	11	35.5	296	32892	6.6	Cytoplasm	enzyme	NONE	0.000569
Q99JB2	STML2_MOUSE	STOML2	(Q99JB2) Stomatin-like protein 2 (SLP-2)	9	32.6	353	38385	8.9	Plasma Membrane	other	NONE	0.000541
Q99JB7	AMNLS_MOUSE	AMN	(Q99JB7) Amnionless protein precursor	2	53 5	458	48696	5.6	Plasma Membrane	other	2	4.9E-05

Q99JB8	PACN3_MOUSE	PACSIN3	(Q99JB8) Protein kinase C and casein kinase II substrate protein 3	5	18.6	424	48585	6.1	Cytoplasm	other	NONE	0.000132
Q99JF8	PSIP1_MOUSE	PSIP1	(Q99JF8) PC4 and SFRS1-interacting protein (Lens epithelium-derived growth factor) (mLEDGF)	2	2.1	528	59697	9.1	Nucleus	other	NONE	3.19E-05
Q99JG3	ANX13_MOUSE	ANXA13	(Q99JG3) Annexin A13 (Annexin XIII)	7	30.7	316	35791	6	Plasma Membrane	other	NONE	0.000266
Q99JP6	HOME3_MOUSE	HOMER3	(Q99JP6) Homer protein homolog 3 (Homer-3)	2	10.1	356	39678	5.4	Plasma Membrane	other	NONE	4.73E-05
Q99JR1	SFXN1_MOUSE	SFXN1	(Q99JR1) Sideroflexin-1	8	25.9	321	35518	9.2	Cytoplasm	transporter	4	0.000577
Q99JR5	TINAL_MOUSE	TINAGL1	(Q99JR5) Tubulointerstitial nephritis antigen-like precursor (Androgen-regulated gene 1 protein) (Adrenocortical zonation factor 1) (AZ-1) (Tubulointerstitial nephritis antigen-related protein) (TARP)	5	19.5	466	52665	6.8	Extracellular Space	transporter	1	0.000157
Q99JR6	NMNA3_MOUSE	NMNAT3 (includes EG:349565)	(Q99JR6) Nicotinamide mononucleotide adenylyltransferase 3 (EC 2.7.7.1) (NMN adenylyltransferase 3)	3	13.5	245	27703	8.5	Cytoplasm	enzyme	NONE	9.16E-05
Q99JT2	MST4_MOUSE	MASK	(Q99JT2) Serine/threonine-protein kinase MST4 (EC 2.7.11.1) (STE20-like kinase MST4) (MST-4) (Mammalian STE20-like protein kinase 4)	5	12.3	416	46614	5.2	Nucleus	kinase	NONE	6.75E-05
Q99JX3	GORS2_MOUSE	GORASP2	(Q99JX3) Golgi reassembly-stacking protein 2 (GRS2) (Golgi reassembly-stacking protein of 55 kDa) (GRASP55)	2	5.3	450	46907	4.8	Cytoplasm	other	NONE	2.49E-05
Q99JX7	NXF1_MOUSE	NXF1	(Q99JX7) Nuclear RNA export factor 1 (Tip-associating protein) (Tip-associated protein) (mRNA export factor TAP)	1	2.9	618	70301	8.6	Nucleus	transporter	NONE	2.72E-05
Q99JY0	ECHB_MOUSE	HADHB	(Q99JY0) Trifunctional enzyme subunit beta, mitochondrial precursor (TP-beta) [Includes: 3-ketoacyl-CoA thiolase (EC 2.3.1.16) (Acetyl-CoA acyltransferase) (Beta-ketothiolase)]	12	25.1	475	51386	9.4	Cytoplasm	enzyme	NONE	0.000532
Q99JY3	GIMA4_MOUSE	GIMAP4	(Q99JY3) GTPase, IMAP family member 4 (Immunity-associated protein 4) (Immunity-associated nucleotide 1 protein)	3	26.5	219	24554	7	Unknown	other	NONE	0.000179
Q99JY9	ARP3_MOUSE	ACTR3	(Q99JY9) Actin-like protein 3 (Actin-related protein 3)	2	7.7	417	47226	5.9	Plasma Membrane	other	1	4.04E-05
Q99K30	ES8L2_MOUSE	EPS8L2	(Q99K30) Epidermal growth factor receptor kinase substrate 8-like protein 2 (Epidermal growth factor receptor pathway substrate 8-related protein 2) (EPS8-like protein 2)	9	17.6	729	82229	7.2	Unknown	other	NONE	0.000139
Q99K41	EMIL1_MOUSE	EMILIN1	(Q99K41) EMILIN-1 precursor (Elastin microfibril interface-located protein 1) (Elastin microfibril interfacer 1)	2	4.7	1017	107585	5.3	Extracellular Space	other	NONE	1.1E-05
Q99K48	NONO_MOUSE	NONO	(Q99K48) Non-POU domain-containing octamer-binding protein (NonO protein)	4	8	473	54541	8.9	Nucleus	other	NONE	9.49E-05
Q99K51	PLST_MOUSE	PLS3	(Q99K51) Plastin-3 (T-plastin)	4	6.7	627	70367	5.8	Cytoplasm	other	NONE	5.37E-05
Q99K67	AASS_MOUSE	AASS	(Q99K67) Alpha-aminoadipic semialdehyde synthase, mitochondrial precursor (LKR/SDH) [Includes: Lysine ketoglutarate reductase (EC 1.5.1.8) (LOR) (LKR); Saccharopine dehydrogenase (EC 1.5.1.9) (SDH)]	11	20.4	926	102975	6.9	Cytoplasm	enzyme	NONE	0.000139
Q99KB8	GLO2_MOUSE	HAGH	(Q99KB8) Hydroxyacylglutathione hydrolase (EC 3.1.2.6) (Glyoxalase II) (Glx II)	10	21.2	260	28901	7	Cytoplasm	enzyme	NONE	0.000648
Q99KC8	LHR2A_MOUSE	LOH11CR2A	(Q99KC8) Loss of heterozygosity 11 chromosomal region 2 gene A protein homolog	2	3	793	87143	6.6	Unknown	other	NONE	4.25E-05
Q99KF1	TMED9_MOUSE	TMED9	(Q99KF1) Transmembrane emp24 domain-containing protein 9 precursor (Glycoprotein 25L2)	2	13.6	214	25010	7.2	Cytoplasm	transporter	1	0.000157
Q99KI0	ACON_MOUSE	ACO2	(Q99KI0) Aconitate hydratase, mitochondrial precursor (EC 4.2.1.3) (Citrate hydro-lyase) (Aconitase)	37	36	780	85464	7.9	Cytoplasm	enzyme	NONE	0.002008
Q99KJ8	DCTN2_MOUSE	DCTN2	(Q99KJ8) Dynactin subunit 2 (Dynactin complex 50 kDa subunit) (50 kDa dynein-associated polypeptide) (p50 dynamitin) (DCTN-50) (Growth cone membrane protein 23-48K) (GMP23-48K)	9	28.7	401	43986	5.3	Cytoplasm	other	NONE	0.000308
Q99KN9	EPN4_MOUSE	CLINT1	(Q99KN9) Epsin-4 (Epsin-related protein) (EpsinR) (Enthoprotin)	11	24.2	631	68513	6.3	Cytoplasm	other	1	0.000178
Q99KP3	CRYL1_MOUSE	CRYL1	(Q99KP3) Lambda-crystallin homolog	13	37.7	318	35078	5.9	Unknown	other	NONE	0.000794
Q99KP6	PRP19_MOUSE	PRPF19	(Q99KP6) Pre-mRNA-splicing factor 19 (PRP19/PSO4 homolog) (Nuclear matrix protein 200) (Nuclear matrix protein SNEV)	2	14.1	504	55239	6.6	Nucleus	other	NONE	3.34E-05

Q99KQ4	NAMPT_MOUSE	PBEF1	(Q99KQ4) Nicotinamide phosphoribosyltransferase (EC 2.4.2.12) (NAmPRTase) (Nampt) (Pre-B-cell colony-enhancing factor 1 homolog) (PBEF) (Visfatin)	7	24.4	491	55447	7.2	Extracellular Space	cytokine	NONE	0.000217
Q99KR7	PPIF_MOUSE	PPIF	(Q99KR7) Peptidyl-prolyl cis-trans isomerase, mitochondrial precursor (EC 5.2.1.8) (PPIase) (Rotamase) (Cyclophilin F)	4	18.9	206	21737	9.2	Cytoplasm	enzyme	NONE	0.000354
Q99KV1	DNJBB_MOUSE	DNAJB11	(Q99KV1) DnaJ homolog subfamily B member 11 precursor	5	24	358	40555	6.3	Cytoplasm	other	1	0.000141
Q99KW3	TARA_MOUSE	TRIOBP	(Q99KW3) TRIO and F-actin-binding protein (Protein Tara) (Trio-associated repeat on actin)	1	0.9	2014	223366	8.1	Nucleus	other	NONE	2.79E-06
Q99KY4	GAK_MOUSE	GAK	(Q99KY4) Cyclin G-associated kinase (EC 2.7.11.1)	1	2.9	1305	143640	5.7	Nucleus	kinase	2	4.3E-06
Q99L04	DHRS1_MOUSE	DHRS1	(Q99L04) Dehydrogenase/reductase SDR family member 1 (EC 1.1.-.-)	2	8.9	313	34005	8.4	Unknown	enzyme	NONE	7.17E-05
Q99L13	3HIDH_MOUSE	HIBADH	(Q99L13) 3-hydroxyisobutyrate dehydrogenase, mitochondrial precursor (EC 1.1.1.31) (HIBADH)	42	48.4	335	35440	8.1	Cytoplasm	enzyme	NONE	0.002714
Q99L45	IF2B_MOUSE	EIF2S2	(Q99L45) Eukaryotic translation initiation factor 2 subunit 2 (Eukaryotic translation initiation factor 2 subunit beta) (eIF-2-beta)	11	32.6	331	38092	5.8	Cytoplasm	translation regulator	NONE	0.000458
Q99L47	F10A1_MOUSE	ST13	(Q99L47) Hsc70-interacting protein (Hip) (Protein ST13 homolog) (Protein FAM10A1)	9	18.3	371	41656	5.3	Cytoplasm	other	NONE	0.000439
Q99LB2	DHRS4_MOUSE	DHRS4	(Q99LB2) Dehydrogenase/reductase SDR family member 4 (EC 1.1.1.184) (NADPH-dependent carbonyl reductase/NADP-retinol dehydrogenase) (CR) (PHCR) (Peroxisomal short-chain alcohol dehydrogenase) (NADPH-dependent retinol dehydrogenase/reductase)	6	14.2	260	27754	9.1	Cytoplasm	enzyme	NONE	0.000281
Q99LB7	SARDH_MOUSE	SARDH	(Q99LB7) Sarcosine dehydrogenase, mitochondrial precursor (EC 1.5.99.1) (SarDH)	5	7.3	919	101682	6.7	Cytoplasm	enzyme	NONE	5.5E-05
Q99LC3	NDUAA_MOUSE	NDUFA10 (includes EG:4705)	(Q99LC3) NADH dehydrogenase [ubiquinone] 1 alpha subcomplex subunit 10, mitochondrial precursor (EC 1.6.5.3) (EC 1.6.99.3) (NADH-ubiquinone oxidoreductase 42 kDa subunit) (Complex I-42KD) (CI-42KD)	9	29	355	40603	7.8	Cytoplasm	enzyme	NONE	0.000379
Q99LC5	ETFA_MOUSE	ETFA	(Q99LC5) Electron transfer flavoprotein subunit alpha, mitochondrial precursor (Alpha-ETF)	24	55.9	333	35039	8.4	Cytoplasm	transporter	NONE	0.013333
Q99LD8	DDAH2_MOUSE	DDAH2	(Q99LD8) NG,NG-dimethylarginine dimethylaminohydrolase 2 (EC 3.5.3.18) (Dimethylargininase-2) (Dimethylarginine dimethylaminohydrolase 2) (DDAHII)	1	8.1	285	29646	6	Unknown	enzyme	NONE	3.94E-05
Q99LG1	TMM51_MOUSE	TMEM51	(Q99LG1) Transmembrane protein 51	2	17.7	249	27398	7	Unknown	other	2	6.76E-05
Q99LI7	CSTF3_MOUSE	CSTF3	(Q99LI7) Cleavage stimulation factor 77 kDa subunit (CSTF 77 kDa subunit) (CF-1 77 kDa subunit) (CstF-77)	2	3.3	717	82877	8.1	Nucleus	other	NONE	1.57E-05
Q99LJ1	FUCO_MOUSE	FUCA1	(Q99LJ1) Tissue alpha-L-fucosidase precursor (EC 3.2.1.51) (Alpha-L-fucosidase I) (Alpha-L-fucoside fucohydrolase)	2	7.5	452	52281	7	Cytoplasm	enzyme	1	3.73E-05
Q99LP6	GRPE1_MOUSE	GRPEL1	(Q99LP6) GrpE protein homolog 1, mitochondrial precursor (Mt-GrpE#1)	8	38.7	217	24307	8.4	Cytoplasm	other	NONE	0.000698
Q99LS3	SERB_MOUSE	PSPH	(Q99LS3) Phosphoserine phosphatase (EC 3.1.3.3) (PSP) (O-phosphoserine phosphohydrolase) (PSPase)	2	12.4	225	25096	6.1	Unknown	phosphatase	NONE	4.99E-05
Q99LT0	DPY30_MOUSE	LOC84661	(Q99LT0) Dpy-30-like protein	4	36.4	99	11213	4.9	Nucleus	other	NONE	0.001531
Q99LX0	PARK7_MOUSE	PARK7	(Q99LX0) Protein DJ-1	13	56.6	189	20021	6.8	Nucleus	other	NONE	0.001129
Q99LY9	NIPM_MOUSE	NDUFS5	(Q99LY9) NADH-ubiquinone oxidoreductase 15 kDa subunit (EC 1.6.5.3) (EC 1.6.99.3) (Complex I-15 kDa) (CI-15 kDa)	5	26.7	105	12516	8.9	Cytoplasm	enzyme	NONE	0.001497
Q99M04	LIAS_MOUSE	LIAS	(Q99M04) Lipoic acid synthetase, mitochondrial precursor (Lip-syn) (Lipoate synthase) (mLIP1)	1	4.6	373	41879	8.9	Cytoplasm	enzyme	NONE	1.5E-05
Q99M28	RNPS1_MOUSE	RNPS1	(Q99M28) RNA-binding protein with serine-rich domain 1	1	4.9	305	34208	11.8	Nucleus	other	NONE	1.84E-05
Q99M87	DNJA3_MOUSE	DNAJA3	(Q99M87) DnaJ homolog subfamily A member 3, mitochondrial precursor (Tumorous imaginal discs protein Tid56 homolog) (DnaJ protein Tid-1) (mTid-1)	6	15.6	480	52443	9.2	Cytoplasm	other	NONE	0.000152
Q99MI6	GIMA3_MOUSE	GIMAP3	(Q99MI6) GTPase, IMAP family member 3 (Immunity-associated nucleotide 4 protein)	1	6.3	301	34179	7.1	Cytoplasm	other	1	0.000168

Q99MN9	PCCB_MOUSE	PCCB	(Q99MN9) Propionyl-CoA carboxylase beta chain, mitochondrial precursor (EC 6.4.1.3) (PCCase subunit beta) (Propanoyl-CoA:carbon dioxide ligase subunit beta)	7	21.1	541	58394	7.5	Cytoplasm	enzyme	NONE	0.000104
Q99MR6	ARS2_MOUSE	ARS2	(Q99MR6) Arsenite-resistance protein 2	1	2.9	875	100452	6	Nucleus	other	NONE	1.92E-05
Q99MR8	MCCA_MOUSE	MCCC1	(Q99MR8) Methylcrotonoyl-CoA carboxylase subunit alpha, mitochondrial precursor (EC 6.4.1.4) (3-methylcrotonyl-CoA carboxylase 1) (MCCase alpha subunit) (3-methylcrotonyl-CoA:carbon dioxide ligase subunit alpha) (3-methylcrotonyl-CoA carboxyla	13	24.1	717	79344	7.8	Cytoplasm	enzyme	NONE	0.000196
Q99MZ7	PECR_MOUSE	PECR	(Q99MZ7) Peroxisomal trans-2-enoyl-CoA reductase (EC 1.3.1.38)	9	31.7	303	32410	8.3	Cytoplasm	enzyme	NONE	0.000482
Q99N23	CAH15_MOUSE	CAR15	(Q99N23) Carbonic anhydrase 15 precursor (EC 4.2.1.1) (Carbonic anhydrase XV) (Carbonate dehydratase XV) (CA-XV)	1	9.9	324	35482	6.8	Extracellular Space	enzyme	1	3.46E-05
Q99N84	RT18B_MOUSE	MRPS18B	(Q99N84) 28S ribosomal protein S18b, mitochondrial precursor (MRP-S18-b) (Mrps18b) (MRP-S18-2)	1	8.7	254	28703	8.4	Cytoplasm	other	NONE	8.84E-05
Q99N92	RM27_MOUSE	MRPL27	(Q99N92) Mitochondrial 39S ribosomal protein L27 (L27mt) (MRP-L27)	2	18.9	148	15945	10.2	Cytoplasm	other	NONE	0.000114
Q99N93	RM16_MOUSE	MRPL16	(Q99N93) 39S ribosomal protein L16, mitochondrial precursor (L16mt) (MRP-L16)	1	6.4	251	28804	10.3	Unknown	other	NONE	8.95E-05
Q99NB1	ACS2L_MOUSE	ACSS1	(Q99NB1) Acetyl-coenzyme A synthetase 2-like, mitochondrial precursor (EC 6.2.1.1) (Acetate--CoA ligase 2) (Acetyl-CoA synthetase 2) (AceCS2) (Acyl-CoA synthetase short-chain family member 1)	2	3.7	682	74623	7	Cytoplasm	enzyme	1	9.88E-05
Q99NB9	SF3B1_MOUSE	SF3B1	(Q99NB9) Splicing factor 3B subunit 1 (Spliceosome-associated protein 155) (SAP 155) (SF3b155) (Pre-mRNA-splicing factor SF3b 155 kDa subunit)	2	2.5	1304	145816	7.1	Nucleus	other	NONE	1.72E-05
Q99NH2	PARD3_MOUSE	PARD3	(Q99NH2) Partitioning-defective 3 homolog (PARD-3) (PAR-3) (Atypical PKC isotype-specific-interacting protein) (ASIP) (Ephrin-interacting protein) (PHIP)	2	2.8	1333	149060	7.8	Plasma Membrane	other	NONE	1.26E-05
Q99PG0	AAAD_MOUSE	AADAC	(Q99PG0) Arylacetamide deacetylase (EC 3.1.1.-) (AADAC)	2	10.1	397	45119	7.5	Cytoplasm	enzyme	NONE	5.66E-05
Q99PG2	OGFR_MOUSE	OGFR	(Q99PG2) Opioid growth factor receptor (OGFr) (Zeta-type opioid receptor)	5	12.5	633	70679	4.8	Plasma Membrane	G-protein coupled receptor	NONE	7.09E-05
Q99PL5	RRBP1_MOUSE	RRBP1	(Q99PL5) Ribosome-binding protein 1 (Ribosome receptor protein) (mRRp)	22	17.8	1605	172878	9.3	Cytoplasm	transporter	1	0.000143
Q99PT1	GDIR_MOUSE	ARHGDIA	(Q99PT1) Rho GDP-dissociation inhibitor 1 (Rho GDI 1) (Rho-GDI alpha) (GDI-1)	14	34.5	203	23276	5.2	Cytoplasm	other	NONE	0.001493
Q99PU8	DHX30_MOUSE	DHX30	(Q99PU8) Putative ATP-dependent RNA helicase DHX30 (EC 3.6.1.-) (DEAH box protein 30)	3	3.8	1217	136668	8.7	Nucleus	enzyme	1	1.38E-05
Q99PW8	KIF17_MOUSE	KIF17	(Q99PW8) Kinesin-like protein KIF17 (MmKIF17)	2	2.9	1038	116373	5.3	Cytoplasm	transporter	NONE	1.08E-05
Q9BCZ4	SELS_MOUSE	SELS	(Q9BCZ4) Selenoprotein S (VCP-interacting membrane protein) (Minor histocompatibility antigen H47)	2	16.8	190	21462	9.3	Cytoplasm	other	1	0.000177
Q9CPP6	NDUA5_MOUSE	NDUFA5	(Q9CPP6) NADH dehydrogenase [ubiquinone] 1 alpha subcomplex subunit 5 (EC 1.6.5.3) (EC 1.6.99.3) (NADH-ubiquinone oxidoreductase 13 kDa-B subunit) (Complex I-13Kd-B) (CI-13Kd-B) (Complex I subunit B13)	8	27.8	115	13229	8.1	Cytoplasm	enzyme	NONE	0.001415
Q9CPQ1	COX6C_MOUSE	COX6C (includes EG:1345)	(Q9CPQ1) Cytochrome c oxidase polypeptide VIc (EC 1.9.3.1)	7	29.3	75	8338	10.1	Cytoplasm	enzyme	1	0.001871
Q9CPQ3	TOM22_MOUSE	RNF13	(Q9CPQ3) Mitochondrial import receptor subunit TOM22 homolog (Translocase of outer membrane 22 kDa subunit homolog)	4	24.8	141	15406	4.3	Nucleus	other	1	0.000518
Q9CPQ8	ATP5L_MOUSE	ATP5L	(Q9CPQ8) ATP synthase subunit g, mitochondrial (EC 3.6.3.14) (ATPase subunit g)	6	47.6	103	11424	9.7	Cytoplasm	transporter	1	0.000817
Q9CPR4	RL17_MOUSE	RPL17	(Q9CPR4) 60S ribosomal protein L17 (L23)	7	26.2	183	21292	10.2	Cytoplasm	other	NONE	0.000644
Q9CPT4	CS010_MOUSE	C19ORF10	(Q9CPT4) Uncharacterized protein C19orf10 homolog precursor (Stromal cell-derived growth factor SF20) (Interleukin-25) (IL-25)	2	14.5	166	17982	6.8	Extracellular Space	cytokine	1	0.000135

Q9CPU0	LGUL_MOUSE	GLO1	(Q9CPU0) Lactoylglutathione lyase (EC 4.4.1.5) (Methylglyoxalase) (Aldoketomutase) (Glyoxalase I) (Glx I) (Ketone-aldehyde mutase) (S-D-lactoylglutathione methylglyoxal lyase)	3	19.7	183	20678	5.5	Cytoplasm	enzyme	NONE	0.000399
Q9CPV3	RT32_MOUSE	MRPL42 (includes EG:28977)	(Q9CPV3) Mitochondrial 28S ribosomal protein S32 (S32mt) (MRP-S32)	1	9.2	142	16493	9.2	Cytoplasm	other	NONE	3.95E-05
Q9CPW4	ARPC5_MOUSE	ARPC5	(Q9CPW4) Actin-related protein 2/3 complex subunit 5 (ARP2/3 complex 16 kDa subunit) (p16-ARC)	3	23.3	150	16157	5.7	Cytoplasm	other	NONE	0.000225
Q9CPX6	ATG3_MOUSE	ATG3	(Q9CPX6) Autophagy-related protein 3 (APG3-like)	3	13.4	314	35796	4.7	Cytoplasm	enzyme	NONE	8.94E-05
Q9CPX7	RT16_MOUSE	MRPS16 (includes EG:51021)	(Q9CPX7) 28S ribosomal protein S16, mitochondrial precursor (S16mt) (MRP-S16)	2	28.1	135	15192	9.7	Cytoplasm	other	NONE	0.000125
Q9CPY7	AMPL_MOUSE	LAP3	(Q9CPY7) Cytosol aminopeptidase (EC 3.4.11.1) (Leucine aminopeptidase) (LAP) (Leucyl aminopeptidase) (Proline aminopeptidase) (EC 3.4.11.5) (Prolyl aminopeptidase)	34	49.1	487	52619	7	Cytoplasm	peptidase	NONE	0.001245
Q9CQ01	RNT2_MOUSE	RNASET2	(Q9CQ01) Ribonuclease T2 precursor (EC 3.1.27.-) (Ribonuclease 6)	2	8.1	259	29609	6.4	Extracellular Space	enzyme	1	0.000238
Q9CQ10	CHMP3_MOUSE	VPS24	(Q9CQ10) Charged multivesicular body protein 3 (Chromatin-modifying protein 3) (Vacuolar protein sorting 24)	2	7.2	223	25088	5.1	Cytoplasm	other	NONE	0.000101
Q9CQ19	MLRN_MOUSE	MYL9	(Q9CQ19) Myosin regulatory light chain 2, smooth muscle isoform (Myosin RLC) (Myosin regulatory light chain 9)	9	35.1	171	19723	4.9	Cytoplasm	other	NONE	0.000525
Q9CQ40	RM49_MOUSE	MRPL49	(Q9CQ40) Mitochondrial 39S ribosomal protein L49 (L49mt) (MRP-L49)	3	21.7	166	19133	9.5	Cytoplasm	enzyme	NONE	0.000203
Q9CQ45	NENF_MOUSE	NENF	(Q9CQ45) Neudesin precursor (Neuron-derived neurotrophic factor) (Secreted protein of unknown function) (SPUF protein)	2	10.5	171	18904	5.3	Extracellular Space	growth factor	1	0.000131
Q9CQ48	NUDC2_MOUSE	NUDCD2	(Q9CQ48) NudC domain-containing protein 2	1	13.4	157	17660	5.1	Unknown	other	NONE	7.15E-05
Q9CQ60	6PGL_MOUSE	PGLS	(Q9CQ60) 6-phosphogluconolactonase (EC 3.1.1.31) (6PGL)	6	35.4	257	27254	5.8	Cytoplasm	enzyme	NONE	0.000328
Q9CQ62	DECR_MOUSE	DECR1	(Q9CQ62) 2,4-dienoyl-CoA reductase, mitochondrial precursor (EC 1.3.1.34) (2,4-dienoyl-CoA reductase [NADPH]) (4-enoyl-CoA reductase [NADPH])	11	29.3	335	36214	8.9	Cytoplasm	enzyme	NONE	0.000519
Q9CQ65	MTAP_MOUSE	MTAP (includes EG:4507)	(Q9CQ65) S-methyl-5-thioadenosine phosphorylase (EC 2.4.2.28) (5'-methylthioadenosine phosphorylase) (MTA phosphorylase) (MTAPase)	3	12.4	283	31062	7.1	Nucleus	enzyme	NONE	0.000159
Q9CQ69	UCRQ_MOUSE	UQCRQ	(Q9CQ69) Ubiquinol-cytochrome c reductase complex ubiquinone-binding protein QP-C (EC 1.10.2.2) (Ubiquinol-cytochrome c reductase complex 9.5 kDa protein) (Complex III subunit VII)	5	29.6	81	9637	10.3	Cytoplasm	enzyme	NONE	0.002911
Q9CQ75	NDUA2_MOUSE	NDUFA2	(Q9CQ75) NADH dehydrogenase [ubiquinone] 1 alpha subcomplex subunit 2 (EC 1.6.5.3) (EC 1.6.99.3) (NADH-ubiquinone oxidoreductase B8 subunit) (Complex I-B8) (CI-B8)	4	39.8	98	10784	10	Cytoplasm	enzyme	NONE	0.000802
Q9CQ85	TIM22_MOUSE	TIMM22	(Q9CQ85) Mitochondrial import inner membrane translocase subunit Tim22	2	15.5	194	20114	8.2	Cytoplasm	transporter	1	0.000116
Q9CQ89	CUTA_MOUSE	CUTA	(Q9CQ89) Protein CutA precursor (Brain acetylcholinesterase putative membrane anchor)	1	7.9	177	18865	6.8	Unknown	other	NONE	0.000254
Q9CQ92	FIS1_MOUSE	FIS1	(Q9CQ92) Mitochondrial fission 1 protein (Fis1 homolog) (Tetratricopeptide repeat protein 11) (TPR repeat protein 11)	3	18.4	152	17009	8.5	Cytoplasm	other	1	0.000185
Q9CQA3	DHSB_MOUSE	SDHB	(Q9CQA3) Succinate dehydrogenase [ubiquinone] iron-sulfur protein, mitochondrial precursor (EC 1.3.5.1) (Ip) (Iron-sulfur subunit of complex II)	15	37.9	282	31814	8.7	Cytoplasm	enzyme	NONE	0.001254
Q9CQA5	MED4_MOUSE	MED4	(Q9CQA5) Mediator complex subunit 4 (Mediator of RNA polymerase II transcription subunit 4)	2	11.5	270	29781	5	Nucleus	transcription regulator	NONE	6.24E-05
Q9CQA6	CHCH1_MOUSE	CHCHD1	(Q9CQA6) Coiled-coil-helix-coiled-coil-helix domain-containing protein 1	1	16.9	118	13608	10.2	Nucleus	other	NONE	9.51E-05
Q9CQC7	NDUB4_MOUSE	NDUFB4	(Q9CQC7) NADH dehydrogenase [ubiquinone] 1 beta subcomplex subunit 4 (EC 1.6.5.3) (EC 1.6.99.3) (NADH-ubiquinone oxidoreductase B15 subunit) (Complex I-B15) (CI-B15)	4	30.5	128	14950	9.9	Cytoplasm	enzyme	1	0.000965
Q9CQC9	SAR1B_MOUSE	SAR1B	(Q9CQC9) GTP-binding protein SAR1b	2	22.7	198	22382	6.1	Unknown	enzyme	NONE	8.5E-05

Q9CQD1	RAB5A_MOUSE	RAB5A	(Q9CQD1) Ras-related protein Rab-5A	3	10.2	215	23599	8.1	Cytoplasm	enzyme	NONE	0.000183
Q9CQE8	CN166_MOUSE	C14ORF166	(Q9CQE8) Protein C14orf166 homolog	5	28.3	244	28152	6.9	Nucleus	other	NONE	0.000207
Q9CQF0	RM11_MOUSE	MRPL11	(Q9CQF0) 39S ribosomal protein L11, mitochondrial precursor (L11mt) (MRP-L11)	3	27.1	192	20680	9.7	Cytoplasm	other	NONE	0.000175
Q9CQF3	CPSF5_MOUSE	NUDT21	(Q9CQF3) Cleavage and polyadenylation specificity factor 5 (Nucleoside diphosphate-linked moiety X motif 21) (Nudix motif 21)	3	29.5	227	26240	8.8	Nucleus	other	NONE	7.42E-05
Q9CQF4	CF203_MOUSE	C6ORF203	(Q9CQF4) Protein C6orf203 homolog	4	22.1	240	27847	9.4	Unknown	other	NONE	0.000117
Q9CQF9	PCYOX_MOUSE	PCYOX1	(Q9CQF9) Prenylcysteine oxidase precursor (EC 1.8.3.5)	3	12.5	505	56495	6.9	Cytoplasm	enzyme	1	7.78E-05
Q9CQH0	PDZ1I_MOUSE	PDZK1IP1	(Q9CQH0) PDZK1-interacting protein 1 (17 kDa membrane-associated protein)	5	16.7	114	12298	4.8	Extracellular Space	other	2	0.001379
Q9CQH3	NDUB5_MOUSE	NDUFB5	(Q9CQH3) NADH dehydrogenase [ubiquinone] 1 beta subcomplex subunit 5, mitochondrial precursor (EC 1.6.5.3) (EC 1.6.99.3) (NADH-ubiquinone oxidoreductase SGDH subunit) (Complex I-SGDH) (CI-SGDH)	8	26.5	189	21710	9.4	Cytoplasm	enzyme	1	0.000624
Q9CQI3	GMFB_MOUSE	GMFB	(Q9CQI3) Glia maturation factor beta (GMF-beta)	2	14.2	141	16592	5.2	Cytoplasm	growth factor	NONE	0.000119
Q9CQI6	COTL1_MOUSE	COTL1	(Q9CQI6) Coactosin-like protein	4	18.4	141	15813	5.4	Cytoplasm	other	NONE	0.000438
Q9CQJ6	DENR_MOUSE	DENR (includes EG:8562)	(Q9CQJ6) Density-regulated protein (DRP)	2	13.1	198	22166	5.3	Unknown	other	NONE	8.5E-05
Q9CQJ8	NDUB9_MOUSE	NDUFB9	(Q9CQJ8) NADH dehydrogenase [ubiquinone] 1 beta subcomplex subunit 9 (EC 1.6.5.3) (EC 1.6.99.3) (NADH-ubiquinone oxidoreductase B22 subunit) (Complex I-B22) (CI-B22)	11	52.8	178	21853	7.8	Cytoplasm	enzyme	NONE	0.001198
Q9CQM5	TXNL5_MOUSE	TXNL5	(Q9CQM5) Thioredoxin-like protein 5 (14 kDa thioredoxin-related protein) (TRP14) (Protein 42-9-9)	5	46.3	123	14015	4.8	Cytoplasm	enzyme	NONE	0.000639
Q9CQM9	TXNL2_MOUSE	TXNL2	(Q9CQM9) Thioredoxin-like protein 2 (PKC-interacting cousin of thioredoxin) (PKC-theta-interacting protein) (PKCq-interacting protein)	3	11.3	337	37778	5.6	Cytoplasm	enzyme	NONE	0.000167
Q9CQN1	TRAP1_MOUSE	TRAP1	(Q9CQN1) Heat shock protein 75 kDa, mitochondrial precursor (HSP 75) (Tumor necrosis factor type 1 receptor-associated protein) (TRAP-1) (TNFR-associated protein 1)	6	13	706	80209	6.7	Cytoplasm	enzyme	NONE	8.75E-05
Q9CQP0	RM33_MOUSE	MRPL33	(Q9CQP0) Mitochondrial 39S ribosomal protein L33 (L33mt) (MRP-L33)	1	20	65	7416	10.9	Unknown	other	NONE	8.64E-05
Q9CQQ7	AT5F1_MOUSE	ATP5F1	(Q9CQQ7) ATP synthase B chain, mitochondrial precursor (EC 3.6.3.14)	10	31.6	256	28949	9.1	Cytoplasm	transporter	NONE	0.000745
Q9CQR2	RS21_MOUSE	RPS21	(Q9CQR2) 40S ribosomal protein S21	5	47	83	9141	8.5	Cytoplasm	other	NONE	0.001488
Q9CQR4	THEM2_MOUSE	THEM2	(Q9CQR4) Thioesterase superfamily member 2	7	31.4	140	15183	8.8	Unknown	enzyme	NONE	0.001123
Q9CQU0	TXD12_MOUSE	TXNDC12	(Q9CQU0) Thioredoxin domain-containing protein 12 precursor (EC 1.8.4.2) (Thioredoxin-like protein p19) (Endoplasmic reticulum protein ERp19)	1	8.8	170	19049	5.3	Cytoplasm	enzyme	NONE	0.000165
Q9CQV1	TIM16_MOUSE	MAGMAS	(Q9CQV1) Mitochondrial import inner membrane translocase subunit TIM16 (Mitochondria-associated granulocyte macrophage CSF signaling molecule)	3	36	125	13785	9.6	Cytoplasm	other	1	0.000404
Q9CQV7	TIM14_MOUSE	DNAJC19	(Q9CQV7) Mitochondrial import inner membrane translocase subunit TIM14 (DnaJ homolog subfamily C member 19)	1	15.7	115	12305	10.1	Cytoplasm	other	NONE	9.76E-05
Q9CQV8	1433B_MOUSE	YWHAB	(Q9CQV8) 14-3-3 protein beta/alpha (Protein kinase C inhibitor protein 1) (KCIP-1)	7	38	245	27955	4.8	Cytoplasm	other	NONE	0.000367
Q9CQX2	CYB5B_MOUSE	CYB5B	(Q9CQX2) Cytochrome b5 type B precursor (Cytochrome b5 outer mitochondrial membrane isoform)	10	54.1	146	16318	4.9	Cytoplasm	enzyme	1	0.001115
Q9CQX8	RT36_MOUSE	MRPS36	(Q9CQX8) Mitochondrial 28S ribosomal protein S36 (S36mt) (MRP-S36)	14	57.8	102	11101	10	Cytoplasm	other	NONE	0.002641
Q9CQY5	IAG2_MOUSE	DKFZP564K142	(Q9CQY5) Implantation-associated protein precursor (IAP) (Magnesium transporter protein 1) (MagT1)	2	10.1	335	37970	9.8	Extracellular Space	enzyme	5	3.35E-05
Q9CQZ5	NDUA6_MOUSE	NDUFA6	(Q9CQZ5) NADH dehydrogenase [ubiquinone] 1 alpha subcomplex subunit 6 (EC 1.6.5.3) (EC 1.6.99.3) (NADH-ubiquinone oxidoreductase B14 subunit) (Complex I-B14) (CI-B14)	3	25.2	131	15283	10.1	Cytoplasm	enzyme	NONE	0.000214

Q9CQZ6	NDUB3_MOUSE	NDUFB3	(Q9CQZ6) NADH dehydrogenase [ubiquinone] 1 beta subcomplex subunit 3 (EC 1.6.5.3) (EC 1.6.99.3) (NADH-ubiquinone oxidoreductase B12 subunit) (Complex I-B12) (CI-B12)	3	23.1	104	11692	9	Cytoplasm	enzyme	NONE	0.000324
Q9CR00	PSD9_MOUSE	PSMD9	(Q9CR00) 26S proteasome non-ATPase regulatory subunit 9 (26S proteasome regulatory subunit p27)	2	9.9	222	24720	6.4	Cytoplasm	transcription regulator	NONE	7.59E-05
Q9CR09	UFC1_MOUSE	UFC1	(Q9CR09) Ufm1-conjugating enzyme 1 (Ubiquitin-fold modifier-conjugating enzyme 1)	2	6	167	19481	7.4	Unknown	enzyme	1	0.000235
Q9CR16	PPID_MOUSE	PPID	(Q9CR16) 40 kDa peptidyl-prolyl cis-trans isomerase (EC 5.2.1.8) (PPIase) (Rotamase) (Cyclophilin-40) (CYP-40)	7	33.1	369	40611	7.4	Cytoplasm	enzyme	NONE	0.000137
Q9CR21	ACPM_MOUSE	NDUFAB1	(Q9CR21) Acyl carrier protein, mitochondrial precursor (ACP) (NADH-ubiquinone oxidoreductase 9.6 kDa subunit) (CI-SDAP)	5	20.5	156	17370	5.2	Cytoplasm	enzyme	NONE	0.000648
Q9CR23	TMEM9_MOUSE	TMEM9	(Q9CR23) Transmembrane protein 9 precursor	2	7.1	183	20633	7	Cytoplasm	other	2	9.2E-05
Q9CR24	NUDT8_MOUSE	NUDT8	(Q9CR24) Nucleoside diphosphate-linked moiety X motif 8, mitochondrial precursor (EC 3.6.1.-) (Nudix motif 8)	2	6.2	210	23253	6.5	Cytoplasm	other	1	5.35E-05
Q9CR26	CF055_MOUSE	C6ORF55	(Q9CR26) Protein C6orf55 homolog	2	11.3	309	33913	6.1	Unknown	other	NONE	3.63E-05
Q9CR51	VATG1_MOUSE	ATP6V1G1	(Q9CR51) Vacuolar ATP synthase subunit G 1 (EC 3.6.3.14) (V-ATPase G subunit 1) (Vacuolar proton pump G subunit 1) (V-ATPase 13 kDa subunit 1)	5	39.3	117	13593	8	Cytoplasm	transporter	NONE	0.000672
Q9CR57	RL14_MOUSE	RPL14	(Q9CR57) 60S ribosomal protein L14	2	11.1	216	23433	11	Cytoplasm	other	NONE	7.8E-05
Q9CR59	G45IP_MOUSE	GADD45GIP 1	(Q9CR59) Growth arrest and DNA-damage-inducible proteins-interacting protein 1	3	24.3	222	25820	10.3	Nucleus	other	NONE	0.000126
Q9CR61	NDUB7_MOUSE	NDUFB7	(Q9CR61) NADH dehydrogenase [ubiquinone] 1 beta subcomplex subunit 7 (EC 1.6.5.3) (EC 1.6.99.3) (NADH-ubiquinone oxidoreductase B18 subunit) (Complex I-B18) (CI-B18)	6	53.7	136	16200	8.2	Cytoplasm	enzyme	NONE	0.002889
Q9CR62	M2OM_MOUSE	SLC25A11	(Q9CR62) Mitochondrial 2-oxoglutarate/malate carrier protein (OGCP) (Solute carrier family 25 member 11)	4	16.9	313	34024	9.9	Cytoplasm	transporter	NONE	0.000646
Q9CR68	UCRI_MOUSE	UQCRFS1	(Q9CR68) Ubiquinol-cytochrome c reductase iron-sulfur subunit, mitochondrial precursor (EC 1.10.2.2) (Rieske iron-sulfur protein) (RISP)	9	43.1	274	29368	8.7	Cytoplasm	enzyme	NONE	0.001598
Q9CR88	RT14_MOUSE	MRPS14	(Q9CR88) Mitochondrial 28S ribosomal protein S14 (S14mt) (MRP-S14)	2	11.7	128	14920	11.4	Cytoplasm	other	NONE	0.000132
Q9CR95	NECP1_MOUSE	NECAP1	(Q9CR95) Adaptin ear-binding coat-associated protein 1 (NECAP-1)	3	16	275	29639	6.4	Plasma Membrane	other	NONE	0.000122
Q9CRB2	NOLA2_MOUSE	NOLA2	(Q9CRB2) H/ACA ribonucleoprotein complex subunit 2 (Nucleolar protein family A member 2) (snoRNP protein NHP2)	2	23.5	153	17247	8.4	Nucleus	other	NONE	0.000183
Q9CRB9	CHCH3_MOUSE	CHCHD3	(Q9CRB9) Coiled-coil-helix-coiled-coil-helix domain-containing protein 3	12	34.8	227	26335	8.4	Cytoplasm	other	NONE	0.000593
Q9CRC3	U235_MOUSE	C15ORF40	(Q9CRC3) UPF0235 protein C15orf40 homolog	2	16.7	126	13189	9.1	Unknown	other	NONE	8.91E-05
Q9CRD2	T103_MOUSE	TTC35	(Q9CRD2) Tetratricopeptide repeat protein KIAA0103	3	11.1	297	34935	6.8	Nucleus	other	NONE	0.000113
Q9CSN1	SNW1_MOUSE	SNW1	(Q9CSN1) SNW domain-containing protein 1 (Nuclear protein SkiP) (Ski-interacting protein)	3	7.3	536	61476	9.5	Nucleus	transcription regulator	NONE	5.24E-05
Q9CSU0	CT077_MOUSE	C20ORF77	(Q9CSU0) Uncharacterized protein C20orf77 homolog	3	12.3	326	36884	6	Unknown	other	NONE	8.61E-05
Q9CT10	RANB3_MOUSE	RANBP3 (includes EG:8498)	(Q9CT10) Ran-binding protein 3 (RanBP3)	4	11	491	52573	5.1	Nucleus	other	NONE	0.000137
Q9CU62	SMC1A_MOUSE	SMC1A	(Q9CU62) Structural maintenance of chromosome 1-like 1 protein (SMC1alpha protein) (Chromosome segregation protein SmcB) (Sb1.8)	3	4.1	1233	143215	7.4	Nucleus	transporter	NONE	4.1E-05
Q9CVB6	ARPC2_MOUSE	ARPC2	(Q9CVB6) Actin-related protein 2/3 complex subunit 2 (ARP2/3 complex 34 kDa subunit) (p34-ARC) (Fragment)	2	15.7	191	22165	7.6	Cytoplasm	other	NONE	5.88E-05
Q9CWH6	PSA7L_MOUSE	PSMA8	(Q9CWH6) Proteasome subunit alpha type 7-like (EC 3.4.25.1)	5	22.4	250	27866	8.7	Cytoplasm	peptidase	NONE	0.000269

Q9CWJ9	PUR9_MOUSE	ATIC	(Q9CWJ9) Bifunctional purine biosynthesis protein PURH [Includes: Phosphoribosylaminoimidazolecarboxamide formyltransferase (EC 2.1.2.3) (AICAR transformylase); IMP cyclohydrolase (EC 3.5.4.10) (Inosinicase) (IMP synthetase) (ATIC)]	1	2.9	592	64157	6.8	Unknown	enzyme	NONE	9.48E-06
Q9CWK8	SNX2_MOUSE	SNX2	(Q9CWK8) Sorting nexin-2	10	20.4	519	58471	5.1	Cytoplasm	transporter	NONE	0.000281
Q9CWS0	DDAH1_MOUSE	DDAH1	(Q9CWS0) NG,NG-dimethylarginine dimethylaminohydrolase 1 (EC 3.5.3.18) (Dimethylargininase-1) (Dimethylarginine dimethylaminohydrolase 1) (DDAHI) (DDAH-1)	16	51.1	284	31250	6	Cytoplasm	enzyme	NONE	0.001265
Q9CWW6	PIN4_MOUSE	PIN4	(Q9CWW6) Peptidyl-prolyl cis-trans isomerase NIMA-interacting 4 (EC 5.2.1.8) (Rotamase Pin4) (PPIase Pin4)	1	22.9	131	13815	9.8	Nucleus	enzyme	NONE	4.28E-05
Q9CWZ3	RBM8A_MOUSE	RBM8A	(Q9CWZ3) RNA-binding protein 8A (RNA-binding motif protein 8A) (Ribonucleoprotein RBM8A)	3	10.9	174	19889	5.7	Nucleus	other	NONE	0.000645
Q9CWZ7	SNAG_MOUSE	NAPG	(Q9CWZ7) Gamma-soluble NSF attachment protein (SNAP-gamma) (N-ethylmaleimide-sensitive factor attachment protein, gamma)	5	23.4	312	34732	5.4	Cytoplasm	transporter	NONE	0.00018
Q9CX00	K0174_MOUSE	KIAA0174	(Q9CX00) Protein KIAA0174	2	14.4	362	39468	5.4	Unknown	other	NONE	7.75E-05
Q9CX34	SUGT1_MOUSE	SUGT1	(Q9CX34) Suppressor of G2 allele of SKP1 homolog	2	11.3	335	38028	5.4	Unknown	other	NONE	0.000117
Q9CXI0	COQ5_MOUSE	COQ5	(Q9CXI0) Ubiquinone biosynthesis methyltransferase COQ5, mitochondrial precursor (EC 2.1.1.-)	5	20.8	327	37336	7.5	Cytoplasm	enzyme	NONE	0.000137
Q9CXI5	ARMET_MOUSE	ARMET	(Q9CXI5) ARMET protein precursor	1	9.5	179	20374	8.1	Extracellular Space	other	NONE	6.27E-05
Q9CXN7	MAWB1_MOUSE	3110049J23 RIK	(Q9CXN7) Probable isomerase MAWBP-1 (EC 5.1.-.-)	4	16.3	288	31983	5.3	Unknown	other	NONE	0.000448
Q9CXS4	PRR6_MOUSE	PRR6	(Q9CXS4) Proline-rich protein 6	5	27	252	27541	9.8	Nucleus	other	NONE	0.00029
Q9CXU9	EIF1B_MOUSE	EIF1B	(Q9CXU9) Eukaryotic translation initiation factor 1b (eIF1b)	6	29.2	113	12824	7.4	Cytoplasm	translation regulator	NONE	0.000546
Q9CXW2	RT22_MOUSE	MRPS22	(Q9CXW2) Mitochondrial 28S ribosomal protein S22 (S22mt) (MRP-S22)	5	17.5	359	41192	8.6	Cytoplasm	other	NONE	0.000156
Q9CXW3	CYBP_MOUSE	CACYBP	(Q9CXW3) Calcyclin-binding protein (CacyBP) (Siah-interacting protein)	2	17.5	229	26510	7.9	Nucleus	other	NONE	9.8E-05
Q9CXW4	RL11_MOUSE	RPL11	(Q9CXW4) 60S ribosomal protein L11	3	28.8	177	20121	9.6	Cytoplasm	other	NONE	0.001205
Q9CXZ1	NUYM_MOUSE	NDUFS4	(Q9CXZ1) NADH-ubiquinone oxidoreductase 18 kDa subunit, mitochondrial precursor (EC 1.6.5.3) (EC 1.6.99.3) (Complex I-18 kDa) (CI-18 kDa) (Complex I-AQDQ) (CI-AQDQ)	16	45.7	175	19785	10	Cytoplasm	enzyme	NONE	0.002919
Q9CY16	RT28_MOUSE	MRPS28 (includes EG:28957)	(Q9CY16) Mitochondrial 28S ribosomal protein S28 (S28mt) (MRP-S28)	3	8.1	186	20520	8.9	Cytoplasm	other	NONE	0.000181
Q9CY18	SNX7_MOUSE	SNX7	(Q9CY18) Sorting nexin-7	3	8.3	387	45000	5.1	Unknown	transporter	NONE	7.25E-05
Q9CY50	SSRA_MOUSE	SSR1	(Q9CY50) Translocon-associated protein subunit alpha precursor (TRAP-alpha) (Signal sequence receptor subunit alpha) (SSR-alpha)	2	14.3	286	32065	4.5	Cytoplasm	other	2	5.89E-05
Q9CY58	PAIRB_MOUSE	SERBP1	(Q9CY58) Plasminogen activator inhibitor 1 RNA-binding protein (PAI1 RNA-binding protein 1) (PAI-RBP1) (SERPINE1 mRNA-binding protein 1)	5	17	407	44714	8.5	Nucleus	other	NONE	0.000124
Q9CY64	BIEA_MOUSE	BLVRA	(Q9CY64) Biliverdin reductase A precursor (EC 1.3.1.24) (Biliverdin-IX alpha-reductase) (BVR A)	8	27.5	295	33525	7	Cytoplasm	enzyme	NONE	0.000266
Q9CYG7	OM34_MOUSE	TOMM34	(Q9CYG7) Mitochondrial import receptor subunit TOM34 (Translocase of outer membrane 34 kDa subunit)	3	11.7	309	34278	9.1	Cytoplasm	other	NONE	7.27E-05
Q9CYH2	CJ058_MOUSE	C10ORF58	(Q9CYH2) Protein C10orf58 homolog precursor	3	20.2	218	24395	9.2	Extracellular Space	other	1	0.000283
Q9CYN2	SPCS2_MOUSE	SPCS2	(Q9CYN2) Signal peptidase complex subunit 2 (EC 3.4.-.-) (Microsomal signal peptidase 25 kDa subunit) (SPase 25 kDa subunit)	1	8.4	226	24978	8.6	Cytoplasm	other	2	2.48E-05
Q9CYR0	SSB_MOUSE	SSBP1	(Q9CYR0) Single-stranded DNA-binding protein, mitochondrial precursor (Mt-SSB) (MtSSB)	4	22.4	152	17319	9.9	Cytoplasm	other	NONE	0.000591
Q9CYZ2	TPD54_MOUSE	TPD52L2	(Q9CYZ2) Tumor protein D54 (Tumor protein D52-like 2)	9	44.5	220	24043	6.1	Cytoplasm	other	NONE	0.000383
Q9CZ13	UQCR1_MOUSE	UQCRC1	(Q9CZ13) Ubiquinol-cytochrome-c reductase complex core protein I, mitochondrial precursor (EC 1.10.2.2)	18	42.3	480	52769	6.1	Cytoplasm	enzyme	NONE	0.000889
Q9CZ30	PTD4_MOUSE	GTPBP9	(Q9CZ30) Putative GTP-binding protein PTD004 homolog	1	3.8	396	44730	7.8	Unknown	other	NONE	5.67E-05

Q9CZ44	NSF1C_MOUSE	STXBP3	(Q9CZ44) NSFL1 cofactor p47 (p97 cofactor p47)	10	30	370	40710	5.1	Plasma Membrane	transporter	NONE	0.000607
Q9CZG9	PDK11_MOUSE	PDZD11	(Q9CZG9) PDZ domain-containing protein 11	3	44.3	140	16182	7.1	Extracellular Space	other	NONE	0.000281
Q9CZL5	PHS2_MOUSE	PCBD2	(Q9CZL5) Pterin-4-alpha-carbinolamine dehydratase 2 (EC 4.2.1.96) (PHS 2) (4-alpha-hydroxy-tetrahydropterin dehydratase 2) (DcoH-like protein DCoHm) (Dimerization cofactor of hepatocyte nuclear factor 1 from muscle) (HNF1-alpha dimerization co	3	23.3	103	11740	6.8	Unknown	enzyme	NONE	0.000272
Q9CZM2	RL15_MOUSE	RPL15	(Q9CZM2) 60S ribosomal protein L15	4	20.2	203	24015	11.6	Cytoplasm	other	NONE	0.000636
Q9CZR8	EFTS_MOUSE	TSFM	(Q9CZR8) Elongation factor Ts, mitochondrial precursor (EF-Ts) (EF-TsMt)	9	35.8	324	35334	7.1	Cytoplasm	translation regulator	NONE	0.000468
Q9CZU6	CISY_MOUSE	CS	(Q9CZU6) Citrate synthase, mitochondrial precursor (EC 2.3.3.1)	4	13.8	464	51737	8.6	Cytoplasm	enzyme	NONE	0.000169
Q9CZW5	TOM70_MOUSE	TOMM70A	(Q9CZW5) Mitochondrial precursor proteins import receptor (Translocase of outer membrane TOM70)	4	8.7	611	67521	7.3	Cytoplasm	other	NONE	7.35E-05
Q9CZX8	RS19_MOUSE	RPS19	(Q9CZX8) 40S ribosomal protein S19	10	37.5	144	15954	10.4	Cytoplasm	other	NONE	0.003079
Q9CZY3	UB2V1_MOUSE	UBE2V1	(Q9CZY3) Ubiquitin-conjugating enzyme E2 variant 1 (UEV-1) (CROC-1)	4	38.8	147	16355	8	Nucleus	transcription regulator	NONE	0.000726
Q9D020	5NT3_MOUSE	NT5C3	(Q9D020) Cytosolic 5'-nucleotidase III (EC 3.1.3.5) (cN-III) (Pyrimidine 5'-nucleotidase 1) (P5'N-1) (P5N-1) (PN-I) (Lupin)	2	4.4	297	33790	5.7	Cytoplasm	phosphatase	NONE	3.78E-05
Q9D023	BR44_MOUSE	BRP44	(Q9D023) Brain protein 44	7	22.8	127	14286	10.6	Plasma Membrane	other	NONE	0.000751
Q9D024	CC47_MOUSE	CCDC47	(Q9D024) Coiled-coil domain-containing protein 47 precursor (Adipocyte-specific protein 4)	8	17.6	483	55844	4.8	Extracellular Space	other	1	0.000302
Q9D051	ODPB_MOUSE	PDHB	(Q9D051) Pyruvate dehydrogenase E1 component subunit beta, mitochondrial precursor (EC 1.2.4.1) (PDHE1-B)	14	32.6	359	38937	6.9	Cytoplasm	enzyme	NONE	0.001048
Q9D0E1	HNRPM_MOUSE	HNRPM	(Q9D0E1) Heterogeneous nuclear ribonucleoprotein M (hnRNP M)	13	20.9	728	77517	8.6	Plasma Membrane	transmembrane receptor	NONE	0.000239
Q9D0F3	LMAN1_MOUSE	LMAN1	(Q9D0F3) ERGIC-53 protein precursor (ER-Golgi intermediate compartment 53 kDa protein) (Lectin, mannose-binding 1) (p58)	2	6	517	57789	6.3	Cytoplasm	other	1	2.17E-05
Q9D0F9	PGM1_MOUSE	PGM1	(Q9D0F9) Phosphoglucomutase-1 (EC 5.4.2.2) (Glucose phosphomutase 1) (PGM 1)	5	10.5	561	61386	6.8	Cytoplasm	enzyme	NONE	0.0001
Q9D0I4	STX17_MOUSE	STX17	(Q9D0I4) Syntaxin-17	1	8.6	301	33221	6.7	Plasma Membrane	other	2	1.86E-05
Q9D0I9	SYR_MOUSE	RARS	(Q9D0I9) Arginyl-tRNA synthetase (EC 6.1.1.19) (Arginine--tRNA ligase) (ArgRS)	1	3	660	75674	7.6	Cytoplasm	enzyme	NONE	4.25E-05
Q9D0J8	PTMS_MOUSE	PTMS	(Q9D0J8) Parathymosin	1	11	100	11299	4.2	Nucleus	other	NONE	0.000393
Q9D0K2	SCOT_MOUSE	OXCT1	(Q9D0K2) Succinyl-CoA:3-ketoacid-coenzyme A transferase 1, mitochondrial precursor (EC 2.8.3.5) (Somatic-type succinyl CoA:3-oxoacid CoA-transferase) (Scot-S)	18	35.6	520	55989	8.5	Cytoplasm	enzyme	NONE	0.003724
Q9D0M1	KPRA_MOUSE	PRPSAP1	(Q9D0M1) Phosphoribosyl pyrophosphate synthetase-associated protein 1 (PRPP synthetase-associated protein 1) (39 kDa phosphoribosypyrophosphate synthetase-associated protein) (PAP39)	3	14.9	356	39432	7.2	Unknown	other	NONE	0.000142
Q9D0M3	CY1_MOUSE	CYC1	(Q9D0M3) Cytochrome c1, heme protein, mitochondrial precursor (Cytochrome c-1)	9	38.2	325	35328	9.2	Cytoplasm	enzyme	NONE	0.000743
Q9D0M5	DYL2_MOUSE	DYNLL2	(Q9D0M5) Dynein light chain 2, cytoplasmic (Dynein light chain LC8-type 2) (8 kDa dynein light chain) (DLC8) (DLC8b)	3	14.6	89	10350	7.4	Cytoplasm	other	NONE	0.000441
Q9D0Q7	RM45_MOUSE	MRPL45	(Q9D0Q7) 39S ribosomal protein L45, mitochondrial precursor (L45mt) (MRP-L45)	2	8.5	306	35411	9.2	Cytoplasm	other	NONE	3.67E-05
Q9D0R4	DDX56_MOUSE	DDX56	(Q9D0R4) Probable ATP-dependent RNA helicase DDX56 (EC 3.6.1.-) (DEAD box protein 56) (ATP-dependent 61 kDa nucleolar RNA helicase)	1	3.5	546	61212	9.2	Nucleus	enzyme	NONE	3.08E-05
Q9D0S9	HINT2_MOUSE	HINT2	(Q9D0S9) Histidine triad nucleotide-binding protein 2 (EC 3.-.-.-) (HINT-2) (HINT-3)	5	29.4	163	17320	9.8	Cytoplasm	other	NONE	0.004167
Q9D0W5	PPIL1_MOUSE	PPIL1	(Q9D0W5) Peptidyl-prolyl cis-trans isomerase-like 1 (EC 5.2.1.8) (PPIase) (Rotamase)	2	20.5	166	18237	8	Plasma Membrane	enzyme	NONE	0.000135
Q9D110	MTHFS_MOUSE	MTHFS	(Q9D110) 5-formyltetrahydrofolate cyclo-ligase (EC 6.3.3.2) (5,10-methenyl-tetrahydrofolate synthetase) (Methenyl-THF synthetase) (MTHFS)	1	6.9	202	23070	7	Cytoplasm	enzyme	NONE	5.56E-05
Q9D172	ES1_MOUSE	C21ORF33	(Q9D172) ES1 protein homolog, mitochondrial precursor	18	45.5	266	28090	8.8	Cytoplasm	other	NONE	0.003376

Q9D187	FA96B_MOUSE	FAM96B	(Q9D187) Protein FAM96B	1	13.5	163	17667	5	Unknown	other	NONE	6.89E-05
Q9D1A2	CPGL1_MOUSE	CNDP2	(Q9D1A2) Cytosolic nonspecific dipeptidase (Glutamate carboxypeptidase-like protein 1) (CNDP dipeptidase 2)	23	36	475	52767	5.7	Cytoplasm	peptidase	NONE	0.001194
Q9D1B9	RM28_MOUSE	MRPL28	(Q9D1B9) 39S ribosomal protein L28, mitochondrial precursor (L28mt) (MRP-L28)	3	13.2	257	30170	9.3	Cytoplasm	other	NONE	0.000109
Q9D1D4	TMEDA_MOUSE	TMED10	(Q9D1D4) Transmembrane emp24 domain-containing protein 10 precursor (Transmembrane protein Tmp21) (21 kDa transmembrane-trafficking protein)	9	26.9	219	24911	6.7	Cytoplasm	transporter	2	0.000615
Q9D1G1	RAB1B_MOUSE	RAB1B	(Q9D1G1) Ras-related protein Rab-1B	5	20.9	201	22187	5.7	Cytoplasm	enzyme	NONE	0.000642
Q9D1H6	CF066_MOUSE	C6ORF66	(Q9D1H6) UPF0240 protein C6orf66 homolog	1	9.2	173	20082	9.4	Unknown	other	NONE	6.49E-05
Q9D1I5	MCEE_MOUSE	MCEE	(Q9D1I5) Methylmalonyl-CoA epimerase, mitochondrial precursor (EC 5.1.99.1) (DL-methylmalonyl-CoA racemase)	5	34.3	178	19017	9.1	Cytoplasm	enzyme	NONE	0.001167
Q9D1J1	NECP2_MOUSE	NECAP2	(Q9D1J1) Adaptin ear-binding coat-associated protein 2 (NECAP-2)	4	22.6	266	28598	8	Cytoplasm	other	NONE	0.000148
Q9D1J3	HCC1_MOUSE	CIP29	(Q9D1J3) Nuclear protein Hcc-1	3	18.7	209	23401	6.7	Nucleus	other	NONE	0.000161
Q9D1K2	VATF_MOUSE	ATP6V1F	(Q9D1K2) Vacuolar ATP synthase subunit F (EC 3.6.3.14) (V-ATPase F subunit) (Vacuolar proton pump F subunit) (V-ATPase 14 kDa subunit)	5	64.7	119	13370	5.8	Cytoplasm	transporter	NONE	0.000943
Q9D1L0	CHCH2_MOUSE	CHCHD2	(Q9D1L0) Coiled-coil-helix-coiled-coil-helix domain-containing protein 2	2	15.7	153	15661	9.6	Unknown	other	NONE	0.000147
Q9D1L9	XIP_MOUSE	HBXIP	(Q9D1L9) Hepatitis B virus X-interacting protein homolog (HBX-interacting protein) (HBV X-interacting protein)	2	22	91	9642	4.9	Cytoplasm	other	NONE	0.000308
Q9D1M7	FKB11_MOUSE	FKBP11	(Q9D1M7) FK506-binding protein 11 precursor (EC 5.2.1.8) (Peptidyl-prolyl cis-trans isomerase) (PPIase) (Rotamase) (19 kDa FK506-binding protein) (FKBP-19)	1	9	201	22137	9.4	Cytoplasm	enzyme	2	2.79E-05
Q9D1P0	RM13_MOUSE	MRPL13	(Q9D1P0) 39S ribosomal protein L13, mitochondrial (L13mt) (MRP-L13)	5	29.8	178	20677	9.4	Cytoplasm	other	NONE	0.000441
Q9D1Q6	TXND4_MOUSE	TXNDC4	(Q9D1Q6) Thioredoxin domain-containing protein 4 precursor (Endoplasmic reticulum resident protein ERp44)	6	12.1	406	46853	5.3	Cytoplasm	enzyme	NONE	0.000152
Q9D1X0	NOL3_MOUSE	NOL3	(Q9D1X0) Nucleolar protein 3	1	8.6	220	24568	4.1	Nucleus	other	NONE	5.1E-05
Q9D273	MMAB_MOUSE	MMAB	(Q9D273) Cob(I)yrinic acid a,c-diamide adenosyltransferase, mitochondrial precursor (EC 2.5.1.17) (Cob(I)alamin adenosyltransferase) (Methylmalonic aciduria type B homolog)	2	12.7	237	26273	9.2	Cytoplasm	enzyme	NONE	7.11E-05
Q9D2G2	ODO2_MOUSE	DLST	(Q9D2G2) Dihydrolipoyllysine-residue succinyltransferase component of 2-oxoglutarate dehydrogenase complex, mitochondrial precursor (EC 2.3.1.61) (Dihydrolipoamide succinyltransferase component of 2-oxoglutarate dehydrogenase complex) (E2) (E2	8	20.9	454	48995	9	Cytoplasm	enzyme	NONE	0.000433
Q9D2M8	UB2V2_MOUSE	UBE2V2 (includes EG:7336)	(Q9D2M8) Ubiquitin-conjugating enzyme E2 variant 2 (Ubc-like protein MMS2)	1	11.1	144	16236	8	Cytoplasm	enzyme	NONE	0.000117
Q9D2R8	RT33_MOUSE	MRPS33	(Q9D2R8) Mitochondrial 28S ribosomal protein S33 (S33mt) (MRP-S33) (Ganglioside-induced differentiation-associated-protein 3)	1	11.3	106	12459	10.3	Cytoplasm	other	NONE	0.000159
Q9D338	RM19_MOUSE	MRPL19	(Q9D338) 39S ribosomal protein L19, mitochondrial precursor (L19mt) (MRP-L19)	3	11	292	33578	9.4	Cytoplasm	other	NONE	0.000192
Q9D358	PPAC_MOUSE	ACP1	(Q9D358) Low molecular weight phosphotyrosine protein phosphatase (EC 3.1.3.48) (LMW-PTP) (Low molecular weight cytosolic acid phosphatase) (EC 3.1.3.2) (PTPase)	2	18.5	157	18061	6.8	Cytoplasm	phosphatase	NONE	7.15E-05
Q9D3D9	ATPD_MOUSE	ATP5D	(Q9D3D9) ATP synthase delta chain, mitochondrial precursor (EC 3.6.3.14)	2	13.7	168	17600	5.1	Cytoplasm	transporter	NONE	0.000768
Q9D4J1	EFHD1_MOUSE	EFHD1	(Q9D4J1) EF-hand domain-containing protein 1 (Swiprosin-2)	1	7.1	240	27000	6	Unknown	other	NONE	2.34E-05
Q9D554	SF3A3_MOUSE	SF3A3	(Q9D554) Splicing factor 3A subunit 3 (Spliceosome-associated protein 61) (SAP 61) (SF3a60)	4	14	501	58842	5.3	Nucleus	other	NONE	6.72E-05
Q9D5V6	SYAP1_MOUSE	SYAP1	(Q9D5V6) Synapse-associated protein 1	3	13.2	365	41350	4.5	Nucleus	other	NONE	0.000123
Q9D662	SC23B_MOUSE	SEC23B	(Q9D662) Protein transport protein Sec23B (SEC23-related protein B)	1	3.4	767	86437	7	Cytoplasm	transporter	NONE	7.32E-06

Q9D666	UN84A_MOUSE	UNC84A	(Q9D666) Sad1/unc-84 protein-like 1 (Unc-84 homolog A)	1	1.8	913	101976	6.8	Nucleus	other	2	1.23E-05
Q9D687	S6A19_MOUSE	SLC6A19	(Q9D687) Sodium-dependent neutral amino acid transporter B(0) (System B(0) neutral amino acid transporter) (B(0)AT1) (Solute carrier family 6 member 19)	2	3.2	634	71367	5.1	Unknown	transporter	12	5.31E-05
Q9D6J5	NDUB8_MOUSE	NDUFB8	(Q9D6J5) NADH dehydrogenase [ubiquinone] 1 beta subcomplex subunit 8, mitochondrial precursor (EC 1.6.5.3) (EC 1.6.99.3) (NADH-ubiquinone oxidoreductase ASHI subunit) (Complex I-ASHI) (CI-ASHI)	10	27.4	186	21876	6.6	Cytoplasm	enzyme	NONE	0.001177
Q9D6J6	NUHM_MOUSE	NDUFV2	(Q9D6J6) NADH-ubiquinone oxidoreductase 24 kDa subunit, mitochondrial precursor (EC 1.6.5.3) (EC 1.6.99.3)	10	43.5	248	27315	7.4	Cytoplasm	enzyme	NONE	0.000883
Q9D6R2	IDH3A_MOUSE	IDH3A	(Q9D6R2) Isocitrate dehydrogenase [NAD] subunit alpha, mitochondrial precursor (EC 1.1.1.41) (Isocitric dehydrogenase) (NAD(+)-specific ICDH)	13	24.9	366	39639	6.7	Cytoplasm	enzyme	NONE	0.00046
Q9D6S7	RRFM_MOUSE	MRRF (includes EG:67871)	(Q9D6S7) Ribosome recycling factor, mitochondrial precursor	3	22.1	262	29051	9.8	Cytoplasm	other	NONE	0.00015
Q9D6V8	PAIP2_MOUSE	PAIP2	(Q9D6V8) Polyadenylate-binding protein-interacting protein 2 (Poly(A)-binding protein-interacting protein 2) (PABP-interacting protein 2)	1	24.2	124	14700	4.1	Cytoplasm	translation regulator	NONE	0.000181
Q9D6Y7	MSRA_MOUSE	MSRA	(Q9D6Y7) Peptide methionine sulfoxide reductase (EC 1.8.4.6) (Protein-methionine-S-oxide reductase) (PMSR) (Peptide Met(O) reductase)	11	35.6	233	25988	8.4	Cytoplasm	enzyme	NONE	0.000964
Q9D6Y9	GLGB_MOUSE	GBE1	(Q9D6Y9) 1,4-alpha-glucan branching enzyme (EC 2.4.1.18) (Glycogen branching enzyme) (Brancher enzyme)	1	2.4	702	80364	6.4	Cytoplasm	enzyme	NONE	8E-06
Q9D6Z1	NOP56_MOUSE	NOL5A	(Q9D6Z1) Nucleolar protein Nop56 (Nucleolar protein 5A)	5	13.8	580	64464	9.1	Nucleus	other	1	9.68E-05
Q9D7A8	ARMC1_MOUSE	ARMC1	(Q9D7A8) Armadillo repeat-containing protein 1	2	11.7	282	31247	5.6	Unknown	other	NONE	5.97E-05
Q9D7B6	ACAD8_MOUSE	ACAD8	(Q9D7B6) Acyl-CoA dehydrogenase family member 8, mitochondrial precursor (EC 1.3.99.-) (ACAD-8) (Isobutyryl-CoA dehydrogenase)	4	11.1	413	45020	8.1	Cytoplasm	enzyme	NONE	0.000136
Q9D7N3	RT09_MOUSE	MRPS9	(Q9D7N3) 28S ribosomal protein S9, mitochondrial precursor (S9mt) (MRP-S9)	2	5.9	390	44886	8.7	Cytoplasm	other	NONE	2.88E-05
Q9D7N9	APMAP_MOUSE	C20ORF3	(Q9D7N9) Adipocyte plasma membrane-associated protein (Protein DD16)	4	13.7	415	46434	6.3	Plasma Membrane	other	1	8.12E-05
Q9D7P6	NIFUN_MOUSE	ISCU	(Q9D7P6) NifU-like N-terminal domain-containing protein, mitochondrial precursor (NifU-like protein) (Iron-sulfur cluster assembly enzyme ISCU)	3	31	168	18098	9.3	Cytoplasm	other	NONE	0.000301
Q9D7S9	CHMP5_MOUSE	CHMP5	(Q9D7S9) Charged multivesicular body protein 5 (Chromatin-modifying protein 5) (SNF7 domain-containing protein 2)	1	11	219	24576	4.8	Cytoplasm	other	NONE	5.13E-05
Q9D7W5	MED8_MOUSE	MED8	(Q9D7W5) Mediator of RNA polymerase II transcription subunit 8 homolog (Activator-recruited cofactor 32 kDa component) (ARC32)	1	6	268	29199	7	Nucleus	other	NONE	2.09E-05
Q9D7X3	DUS3_MOUSE	DUSP3	(Q9D7X3) Dual specificity protein phosphatase 3 (EC 3.1.3.48) (EC 3.1.3.16) (T-DSP11)	3	16.2	185	20472	6.5	Cytoplasm	phosphatase	NONE	0.000121
Q9D819	IPYR_MOUSE	PPA1	(Q9D819) Inorganic pyrophosphatase (EC 3.6.1.1) (Pyrophosphate phospho-hydrolase) (PPase)	7	31.1	289	32667	5.6	Cytoplasm	enzyme	NONE	0.000291
Q9D824	FIP1_MOUSE	FIP1L1	(Q9D824) Pre-mRNA 3'-end-processing factor FIP1 (FIP1-like 1)	1	3.3	581	64959	5.8	Nucleus	other	NONE	2.9E-05
Q9D826	SOX_MOUSE	PIPOX	(Q9D826) Peroxisomal sarcosine oxidase (EC 1.5.3.1) (PSO) (L-pipecolate oxidase) (EC 1.5.3.7) (L-pipecolic acid oxidase)	4	9.2	390	43847	7.6	Cytoplasm	enzyme	NONE	0.000101
Q9D832	DNJB4_MOUSE	DNAJB4	(Q9D832) DnaJ homolog subfamily B member 4	1	4.5	337	37782	8.6	Unknown	other	NONE	1.67E-05
Q9D855	UCR6_MOUSE	UQCRB (includes EG:67530)	(Q9D855) Ubiquinol-cytochrome c reductase complex 14 kDa protein (EC 1.10.2.2) (Complex III subunit VI)	20	57.3	110	13396	9.1	Cytoplasm	enzyme	NONE	0.013063
Q9D880	TIM50_MOUSE	TIMM50	(Q9D880) Import inner membrane translocase subunit TIM50, mitochondrial precursor	2	7.9	353	39776	8.1	Cytoplasm	phosphatase	NONE	6.36E-05
Q9D8B3	CHM4B_MOUSE	CHMP4B	(Q9D8B3) Charged multivesicular body protein 4b (Chromatin-modifying protein 4b) (CHMP4b)	2	13.4	224	24936	4.8	Cytoplasm	other	NONE	0.00015

Q9D8B4	NDUAB_MOUSE	NDUFA11 (includes EG:69875)	(Q9D8B4) NADH dehydrogenase [ubiquinone] 1 alpha subcomplex subunit 11 (EC 1.6.5.3) (EC 1.6.99.3) (NADH-ubiquinone oxidoreductase subunit B14.7) (Complex I-B14.7) (CI-B14.7)	3	9.9	141	14982	8.4	Cytoplasm	enzyme	NONE	0.000199
Q9D8C4	IN35_MOUSE	IFI35	(Q9D8C4) Interferon-induced 35 kDa protein homolog (IFP 35)	1	6	285	31744	5.9	Nucleus	other	NONE	1.97E-05
Q9D8E6	RL4_MOUSE	RPL4	(Q9D8E6) 60S ribosomal protein L4 (L1)	15	30.4	418	47023	11	Cytoplasm	enzyme	NONE	0.000483
Q9D8N0	EF1G_MOUSE	EEF1G	(Q9D8N0) Elongation factor 1-gamma (EF-1-gamma) (eEF-1B gamma)	2	5.3	436	49930	6.7	Cytoplasm	translation regulator	NONE	5.15E-05
Q9D8S3	ARFG3_MOUSE	ARFGAP3	(Q9D8S3) ADP-ribosylation factor GTPase-activating protein 3 (ARF GAP 3)	2	6.5	525	57614	8.5	Cytoplasm	transporter	NONE	5.35E-05
Q9D8S4	ORN_MOUSE	REXO2	(Q9D8S4) Oligoribonuclease, mitochondrial precursor (EC 3.1.-.-) (Small fragment nuclease) (RNA exonuclease 2 homolog)	6	30	237	26739	7.2	Cytoplasm	enzyme	NONE	0.000213
Q9D8S9	BOLA1_MOUSE	BOLA1	(Q9D8S9) BolA-like protein 1	5	51.1	137	14379	8.8	Unknown	other	NONE	0.000451
Q9D8U8	SNX5_MOUSE	SNX5	(Q9D8U8) Sorting nexin-5	7	21.5	404	46797	6.6	Cytoplasm	transporter	NONE	0.000139
Q9D8W5	PSD12_MOUSE	PSMD12	(Q9D8W5) 26S proteasome non-ATPase regulatory subunit 12 (26S proteasome regulatory subunit p55)	2	6.6	455	52746	7.1	Cytoplasm	other	NONE	4.93E-05
Q9D8Y0	EFHD2_MOUSE	EFHD2	(Q9D8Y0) EF-hand domain-containing protein 2 (Swiprosin-1)	4	11.7	240	26791	5.1	Unknown	other	NONE	0.000117
Q9D8Z2	TRIA1_MOUSE	TRIAP1	(Q9D8Z2) TP53-regulated inhibitor of apoptosis 1 (p53-inducible cell-survival factor) (p53CSV) (Protein 15E1.1) (WF-1)	1	18.4	76	8756	5.5	Cytoplasm	other	NONE	0.000148
Q9D903	EBP2_MOUSE	EBNA1BP2	(Q9D903) Probable rRNA-processing protein EBP2	2	13.7	306	34703	10.1	Nucleus	other	NONE	5.5E-05
Q9D939	ST1C2_MOUSE	SULT1C1	(Q9D939) Sulfotransferase 1C2 (EC 2.8.2.-)	8	24.7	296	34953	7.8	Cytoplasm	enzyme	NONE	0.000569
Q9D958	SPCS1_MOUSE	SPCS1 (includes EG:28972)	(Q9D958) Signal peptidase complex subunit 1 (EC 3.4.-.-) (Microsomal signal peptidase 12 kDa subunit) (SPase 12 kDa subunit)	2	17.6	102	11777	9.3	Cytoplasm	peptidase	2	0.00011
Q9D964	GATM_MOUSE	GATM	(Q9D964) Glycine amidinotransferase, mitochondrial precursor (EC 2.1.4.1) (L-arginine:glycine amidinotransferase) (Transamidinase) (AT)	14	39.5	423	48297	7.9	Cytoplasm	enzyme	NONE	0.000398
Q9D967	MGDP1_MOUSE	MGC5987	(Q9D967) Magnesium-dependent phosphatase 1 (EC 3.1.3.-) (EC 3.1.3.48) (MDP-1)	4	41.5	164	18582	6.8	Unknown	other	NONE	0.000513
Q9D9B4	CJ011_MOUSE	C10ORF11	(Q9D9B4) Protein C10orf11 homolog	1	10.9	229	25989	6.4	Unknown	other	NONE	2.45E-05
Q9D9K3	AVEN_MOUSE	AVEN	(Q9D9K3) Cell death regulator Aven	2	14.9	342	37195	5	Cytoplasm	ion channel	NONE	6.57E-05
Q9DAK9	PHP14_MOUSE	PHPT1	(Q9DAK9) 14 kDa phosphohistidine phosphatase (EC 3.1.3.-) (Phosphohistidine phosphatase 1)	5	50	124	13997	5.5	Cytoplasm	phosphatase	NONE	0.000317
Q9DAR7	DCPS_MOUSE	DCPS	(Q9DAR7) Scavenger mRNA decapping enzyme DcpS (EC 3.-.-.-) (DCS-1) (Hint-related 7meGMP-directed hydrolase) (Histidine triad protein member 5) (HINT-5)	4	15.4	338	38988	6.5	Nucleus	enzyme	NONE	0.000183
Q9DAW9	CNN3_MOUSE	CNN3	(Q9DAW9) Calponin-3 (Calponin, acidic isoform)	5	17.9	330	36429	5.7	Cytoplasm	other	NONE	0.000272
Q9DB05	SNAA_MOUSE	NAPA	(Q9DB05) Alpha-soluble NSF attachment protein (SNAP-alpha) (N-ethylmaleimide-sensitive factor attachment protein, alpha)	3	13.2	295	33190	5.4	Cytoplasm	transporter	NONE	7.61E-05
Q9DB15	RM12_MOUSE	MRPL12	(Q9DB15) 39S ribosomal protein L12, mitochondrial precursor (L12mt) (MRP-L12)	7	39.8	201	21708	9.3	Cytoplasm	other	NONE	0.000586
Q9DB20	ATPO_MOUSE	ATP5O	(Q9DB20) ATP synthase O subunit, mitochondrial precursor (EC 3.6.3.14) (Oligomycin sensitivity conferral protein) (OSCP)	24	63.4	213	23364	10	Cytoplasm	transporter	NONE	0.0078
Q9DB25	ALG5_MOUSE	ALG5	(Q9DB25) Dolichyl-phosphate beta-glucosyltransferase (EC 2.4.1.117) (DolP-glucosyltransferase)	2	11.1	324	36791	8.6	Cytoplasm	enzyme	1	6.93E-05
Q9DB30	PHKG2_MOUSE	PHKG2	(Q9DB30) Phosphorylase b kinase gamma catalytic chain, testis/liver isoform (EC 2.7.11.19) (PHK-gamma-T) (Phosphorylase kinase subunit gamma 2)	1	2.7	406	46519	6.4	Unknown	kinase	NONE	1.38E-05
Q9DB34	CHM2A_MOUSE	CHMP2A	(Q9DB34) Charged multivesicular body protein 2a (Chromatin-modifying protein 2a) (CHMP2a) (Vacuolar protein sorting 2) (mVps2)	2	12.2	222	25134	6	Cytoplasm	other	NONE	7.59E-05
Q9DB77	UQCR2_MOUSE	UQCRC2	(Q9DB77) Ubiquinol-cytochrome-c reductase complex core protein 2, mitochondrial precursor (EC 1.10.2.2) (Complex III subunit II)	18	32.7	453	48235	9.3	Cytoplasm	enzyme	NONE	0.000719
Q9DBC0	SELO_MOUSE	SELO	(Q9DBC0) Selenoprotein O	2	6.1	667	74131	5.7	Unknown	enzyme	NONE	2.52E-05
Q9DBE0	CSAD_MOUSE	CSAD	(Q9DBE0) Cysteine sulfinic acid decarboxylase (EC 4.1.1.29) (Sulfinoalanine decarboxylase) (Cysteine-sulfinate decarboxylase)	3	9.9	493	55145	6.6	Unknown	enzyme	NONE	7.97E-05

Q9DBF1	AL7A1_MOUSE	ALDH7A1	(Q9DBF1) Aldehyde dehydrogenase family 7 member A1 (EC 1.2.1.3) (Antiquitin-1)	6	13.3	510	55514	6.4	Unknown	enzyme	2	0.000154
Q9DBG5	M6PBP_MOUSE	M6PRBP1	(Q9DBG5) Mannose-6-phosphate receptor-binding protein 1 (Cargo selection protein TIP47)	8	33.6	437	47262	5.6	Cytoplasm	other	NONE	0.000244
Q9DBG6	RPN2_MOUSE	RPN2	(Q9DBG6) Dolichyl-diphosphooligosaccharide--protein glycosyltransferase 63 kDa subunit precursor (EC 2.4.1.119) (Ribophorin II) (RPN-II)	2	5.4	631	69063	5.8	Cytoplasm	enzyme	5	1.78E-05
Q9DBG9	TX1B3_MOUSE	TAX1BP3	(Q9DBG9) Tax1-binding protein 3 (Tax interaction protein 1) (TIP-1)	1	13.7	124	13723	8.5	Nucleus	transcription regulator	NONE	9.05E-05
Q9DBH5	LMAN2_MOUSE	LMAN2	(Q9DBH5) Vesicular integral-membrane protein VIP36 precursor (Lectin, mannose-binding 2)	3	14	358	40416	7	Cytoplasm	transporter	2	6.27E-05
Q9DBJ1	PGAM1_MOUSE	PGAM1	(Q9DBJ1) Phosphoglycerate mutase 1 (EC 5.4.2.1) (EC 5.4.2.4) (EC 3.1.3.13) (Phosphoglycerate mutase isozyme B) (PGAM-B) (BPG-dependent PGAM 1)	21	59.3	253	28701	7.2	Cytoplasm	phosphatase	NONE	0.001575
Q9DBK0	ACO12_MOUSE	ACOT12	(Q9DBK0) Acyl-coenzyme A thioesterase 12 (EC 3.1.2.1) (Acyl-CoA thioesterase 12) (Acyl-CoA thioester hydrolase 12) (Cytoplasmic acetyl-CoA hydrolase 1) (CACH-1) (mCACH-1)	1	4.1	556	61762	7.2	Cytoplasm	enzyme	NONE	1.01E-05
Q9DBL1	ACDSB_MOUSE	ACADSB	(Q9DBL1) Short/branched chain specific acyl-CoA dehydrogenase, mitochondrial precursor (EC 1.3.99.-) (SBCAD) (2-methyl branched chain acyl-CoA dehydrogenase) (2-MEBCAD) (2-methylbutyryl-coenzyme A dehydrogenase) (2-methylbutyryl-CoA dehydroge	9	20.8	432	47874	7.9	Cytoplasm	enzyme	NONE	0.00026
Q9DBL7	COASY_MOUSE	COASY	(Q9DBL7) Bifunctional coenzyme A synthase (CoA synthase) [Includes: Phosphopantetheine adenylyltransferase (EC 2.7.7.3) (Pantetheine-phosphate adenylyltransferase) (PPAT) (Dephospho-CoA pyrophosphorylase); Dephospho-CoA kinase (EC 2.7.1.24) (	4	18.7	563	62023	7.1	Cytoplasm	kinase	NONE	4.99E-05
Q9DBM2	ECHP_MOUSE	EHHADH	(Q9DBM2) Peroxisomal bifunctional enzyme (PBE) (PBFE) [Includes: Enoyl-CoA hydratase (EC 4.2.1.17); 3,2-trans-enoyl-CoA isomerase (EC 5.3.3.8); 3-hydroxyacyl-CoA dehydrogenase (EC 1.1.1.35)]	24	39.5	717	78112	9.2	Cytoplasm	enzyme	NONE	0.00047
Q9DBP5	KCY_MOUSE	CMPK	(Q9DBP5) UMP-CMP kinase (EC 2.7.4.14) (Cytidylate kinase) (Deoxycytidylate kinase) (Cytidine monophosphate kinase) (Uridine monophosphate/cytidine monophosphate kinase) (UMP/CMP kinase) (UMP/CMPK) (Uridine monophosphate kinase)	11	35.2	196	22165	5.8	Nucleus	kinase	NONE	0.001632
Q9DBR0	AKAP8_MOUSE	AKAP8	(Q9DBR0) A-kinase anchor protein 8 (A-kinase anchor protein 95 kDa) (AKAP 95)	3	9	687	76294	5.1	Nucleus	other	NONE	4.09E-05
Q9DBR7	MYPT1_MOUSE	PPP1R12A	(Q9DBR7) Protein phosphatase 1 regulatory subunit 12A (Myosin phosphatase-targeting subunit 1) (Myosin phosphatase target subunit 1)	13	15.5	1004	111809	5.6	Cytoplasm	phosphatase	NONE	0.000123
Q9DBS5	KLC4_MOUSE	KLC4	(Q9DBS5) Kinesin light chain 4 (KLC 4) (Kinesin-like protein 8)	3	8.4	619	68613	6.1	Unknown	other	NONE	3.63E-05
Q9DBS9	OSBL3_MOUSE	OSBPL3	(Q9DBS9) Oxysterol-binding protein-related protein 3 (OSBP-related protein 3) (ORP-3)	7	10.4	855	96967	6.5	Cytoplasm	other	NONE	8.53E-05
Q9DBT9	M2GD_MOUSE	DMGDH	(Q9DBT9) Dimethylglycine dehydrogenase, mitochondrial precursor (EC 1.5.99.2) (ME2GLYDH)	5	9.1	869	97255	7.9	Cytoplasm	enzyme	NONE	4.52E-05
Q9DBU3	RIOK3_MOUSE	RIOK3	(Q9DBU3) Serine/threonine-protein kinase RIO3 (EC 2.7.11.1) (RIO kinase 3)	2	4	519	58691	5.7	Unknown	kinase	NONE	6.49E-05
Q9DBX2	PHLP_MOUSE	PDCL	(Q9DBX2) Phosducin-like protein (PHLP)	1	8.6	301	34407	4.9	Cytoplasm	enzyme	NONE	1.86E-05
Q9DBZ5	IF3C_MOUSE	EIF3S12	(Q9DBZ5) Eukaryotic translation initiation factor 3 subunit 12 (eIF-3 p25) (eIF3k)	2	6.9	218	25087	4.9	Cytoplasm	translation regulator	NONE	5.15E-05
Q9DC16	ERGI1_MOUSE	ERGIC1	(Q9DC16) Endoplasmic reticulum-Golgi intermediate compartment protein 1 (ER-Golgi intermediate compartment 32 kDa protein) (ERGIC-32)	2	13.1	290	32562	7.1	Cytoplasm	other	1	5.81E-05
Q9DC50	OCTC_MOUSE	CROT	(Q9DC50) Peroxisomal carnitine O-octanoyltransferase (EC 2.3.1.137) (COT)	2	7.4	612	70264	6.7	Cytoplasm	enzyme	NONE	6.42E-05
Q9DC51	GNAI3_MOUSE	GNAI3	(Q9DC51) Guanine nucleotide-binding protein G(k) subunit alpha (G(i) alpha-3)	1	4.2	353	40407	5.7	Cytoplasm	enzyme	NONE	3.18E-05

Q9DC69	NDUA9_MOUSE	NDUFA9 (includes EG:4704)	(Q9DC69) NADH dehydrogenase [ubiquinone] 1 alpha subcomplex subunit 9, mitochondrial precursor (EC 1.6.5.3) (EC 1.6.99.3) (NADH-ubiquinone oxidoreductase 39 kDa subunit) (Complex I-39KD) (CI-39KD)	5	13	377	42509	9.7	Cytoplasm	enzyme	NONE	0.000402
Q9DC70	NUKM_MOUSE	NDUFS7	(Q9DC70) NADH-ubiquinone oxidoreductase 20 kDa subunit, mitochondrial precursor (EC 1.6.5.3) (EC 1.6.99.3) (Complex I-20KD) (CI-20KD) (PSST subunit)	6	37.5	224	24683	9.9	Cytoplasm	enzyme	NONE	0.000401
Q9DC71	RT15_MOUSE	MRPS15	(Q9DC71) 28S ribosomal protein S15, mitochondrial precursor (S15mt) (MRP-S15)	2	10.5	258	29464	10.1	Cytoplasm	other	NONE	6.53E-05
Q9DCA2	RT11_MOUSE	MRPS11	(Q9DCA2) 28S ribosomal protein S11, mitochondrial precursor (S11mt) (MRP-S11)	4	24.6	191	20208	10.8	Cytoplasm	other	NONE	0.000176
Q9DCE5	PK1IP_MOUSE	PAK1IP1	(Q9DCE5) p21-activated protein kinase-interacting protein 1 (PAK1-interacting protein 1) (Putative PAK inhibitor Skb15)	1	5.5	382	42116	8.5	Nucleus	other	NONE	4.41E-05
Q9DCG6	MAWB2_MOUSE	PBLD	(Q9DCG6) Probable isomerase MAWBP-2 (EC 5.1.-.-)	2	14.2	288	32048	7	Unknown	enzyme	NONE	0.000331
Q9DCG9	U315_MOUSE	HSPC152	(Q9DCG9) UPF0315 protein	2	20	125	14141	5.3	Unknown	other	NONE	0.00018
Q9DCJ5	NDUA8_MOUSE	NDUFA8	(Q9DCJ5) NADH dehydrogenase [ubiquinone] 1 alpha subcomplex subunit 8 (EC 1.6.5.3) (EC 1.6.99.3) (NADH-ubiquinone oxidoreductase 19 kDa subunit) (Complex I-19KD) (CI-19KD) (Complex I-PGIV) (CI-PGIV)	13	63.2	171	19861	8.5	Cytoplasm	enzyme	NONE	0.00581
Q9DCJ7	AKIP_MOUSE	AURKAIP1	(Q9DCJ7) Aurora kinase A-interacting protein (AURKA-interacting protein)	1	7	200	23328	10.8	Nucleus	enzyme	NONE	2.81E-05
Q9DCM0	ETHE1_MOUSE	ETHE1	(Q9DCM0) ETHE1 protein, mitochondrial precursor (EC 3.-.-.-) (Ethylmalonic encephalopathy protein 1 homolog) (Hepatoma subtracted clone one protein)	9	29.9	254	27739	7.2	Cytoplasm	other	NONE	0.001591
Q9DCM2	GSTK1_MOUSE	GSTK1	(Q9DCM2) Glutathione S-transferase kappa 1 (EC 2.5.1.18) (GST 13-13) (Glutathione S-transferase subunit 13) (GST class-kappa) (GSTK1-1) (mGSTK1)	5	28.4	225	25573	8.9	Cytoplasm	enzyme	NONE	0.000225
Q9DCN1	NUD12_MOUSE	NUDT12	(Q9DCN1) Peroxisomal NADH pyrophosphatase NUDT12 (EC 3.6.1.22) (Nucleoside diphosphate-linked moiety X motif 12) (Nudix motif 12)	2	10	462	51511	7.1	Cytoplasm	phosphatase	NONE	2.43E-05
Q9DCN2	NCB5R_MOUSE	CYB5R3	(Q9DCN2) NADH-cytochrome b5 reductase (EC 1.6.2.2) (B5R) (Diaphorase-1) (Cytochrome b5 reductase 3) [Contains: NADH-cytochrome b5 reductase membrane-bound form; NADH-cytochrome b5 reductase soluble form]	3	14.7	300	33996	8.4	Cytoplasm	enzyme	1	0.000112
Q9DCS3	MECR_MOUSE	MECR	(Q9DCS3) Trans-2-enoyl-CoA reductase, mitochondrial precursor (EC 1.3.1.38)	10	26.8	373	40343	9.1	Cytoplasm	enzyme	NONE	0.000346
Q9DCS9	NDUBA_MOUSE	NDUFB10 (includes EG:4716)	(Q9DCS9) NADH dehydrogenase [ubiquinone] 1 beta subcomplex subunit 10 (EC 1.6.5.3) (EC 1.6.99.3) (NADH-ubiquinone oxidoreductase PDSW subunit) (Complex I-PDSW) (CI-PDSW)	16	56.6	175	20893	8	Cytoplasm	enzyme	NONE	0.006704
Q9DCT2	NUGM_MOUSE	NDUFS3	(Q9DCT2) NADH-ubiquinone oxidoreductase 30 kDa subunit, mitochondrial precursor (EC 1.6.5.3) (EC 1.6.99.3) (Complex I-30KD) (CI-30KD)	23	53.2	263	30207	6.9	Cytoplasm	enzyme	NONE	0.001942
Q9DCT5	SDF2_MOUSE	SDF2	(Q9DCT5) Stromal cell-derived factor 2 precursor (SDF-2)	6	25.6	211	23159	7.3	Extracellular Space	enzyme	1	0.000346
Q9DCT8	CRIP2_MOUSE	CRIP2	(Q9DCT8) Cysteine-rich protein 2 (CRP2) (Heart LIM protein)	4	38.5	208	22727	8.6	Unknown	other	NONE	0.000351
Q9DCV4	FA82B_MOUSE	FAM82B	(Q9DCV4) Protein FAM82B	5	16.7	305	35000	8.7	Unknown	other	NONE	0.000184
Q9DCW4	ETFB_MOUSE	ETFB	(Q9DCW4) Electron transfer flavoprotein subunit beta (Beta-ETF)	20	61.4	254	27492	8.1	Cytoplasm	transporter	NONE	0.007492
Q9DCX2	ATP5H_MOUSE	ATP5H (includes EG:10476)	(Q9DCX2) ATP synthase D chain, mitochondrial (EC 3.6.3.14)	25	78.1	160	18618	5.7	Cytoplasm	transporter	NONE	0.008946
Q9DCX8	IYD1_MOUSE	IYD	(Q9DCX8) Iodotyrosine dehalogenase 1 precursor (EC 1.-.-.-) (IYD-1)	4	9.5	285	32814	6.4	Unknown	other	1	7.88E-05
Q9DCZ1	GMPR1_MOUSE	GMPR	(Q9DCZ1) GMP reductase 1 (EC 1.7.1.7) (Guanosine 5'-monophosphate oxidoreductase 1) (Guanosine monophosphate reductase 1)	2	3.2	345	37482	7.1	Cytoplasm	enzyme	NONE	9.76E-05

Q9EP71	RAI14_MOUSE	RAI14	(Q9EP71) Ankycorbin (Ankyrin repeat and coiled-coil structure-containing protein) (Retinoic acid-induced protein 14) (Novel retinal pigment epithelial cell protein) (p125)	6	11.5	979	108852	6.3	Nucleus	transcription regulator	NONE	5.73E-05
Q9EP72	CO024_MOUSE	C15ORF24	(Q9EP72) Uncharacterized protein C15orf24 homolog precursor	1	6.6	241	26310	9.2	Unknown	other	2	9.32E-05
Q9EP89	LACTB_MOUSE	LACTB	(Q9EP89) Serine beta-lactamase-like protein LACTB	6	15.8	551	60706	8.9	Cytoplasm	other	NONE	0.000122
Q9EPJ9	ARFG1_MOUSE	ARFGAP1	(Q9EPJ9) ADP-ribosylation factor GTPase-activating protein 1 (ADP-ribosylation factor 1 GTPase-activating protein) (ARF1 GAP) (ARF1-directed GTPase-activating protein) (GAP protein)	3	14.5	414	45288	5.6	Cytoplasm	transporter	NONE	0.000108
Q9EPK8	TRPV4_MOUSE	TRPV4	(Q9EPK8) Transient receptor potential cation channel subfamily V member 4 (TrpV4) (osm-9-like TRP channel 4) (OTRPC4) (Vanilloid receptor-like channel 2) (Vanilloid receptor-like protein 2) (Vanilloid receptor-related osmotically-activated ch	2	4.2	871	98027	7.3	Plasma Membrane	ion channel	6	1.29E-05
Q9EPL9	ACOX3_MOUSE	ACOX3	(Q9EPL9) Acyl-coenzyme A oxidase 3, peroxisomal (EC 1.3.3.6) (Pristanoyl-CoA oxidase) (Branched-chain acyl-CoA oxidase) (BRCACox)	4	7.3	700	78539	7.4	Cytoplasm	enzyme	NONE	4.81E-05
Q9EQ06	DHRS8_MOUSE	HSD17B11	(Q9EQ06) Dehydrogenase/reductase SDR family member 8 precursor (EC 1.1.1.-) (17-beta-hydroxysteroid dehydrogenase 11) (17-beta-HSD 11) (17-beta-HSD XI) (17betaHSDXI) (17bHSD11) (17betaHSD11)	4	15.1	298	32881	8.7	Cytoplasm	enzyme	2	0.000207
Q9EQG7	ENPP5_MOUSE	ENPP5	(Q9EQG7) Ectonucleotide pyrophosphatase/phosphodiesterase 5 precursor (EC 3.1.-.-) (E-NPP5) (NPP-5)	1	4.4	477	54415	5.9	Extracellular Space	enzyme	2	1.18E-05
Q9EQG9	C43BP_MOUSE	COL4A3BP	(Q9EQG9) Goodpasture antigen-binding protein (EC 2.7.11.9) (GPBP) (Collagen type IV alpha-3-binding protein) (StAR-related lipid transfer protein 11) (StARD11) (START domain-containing protein 11)	2	4.3	624	71111	5.4	Cytoplasm	kinase	NONE	1.8E-05
Q9EQH3	VPS35_MOUSE	VPS35	(Q9EQH3) Vacuolar protein sorting 35 (Vesicle protein sorting 35) (Maternal-embryonic 3)	2	3.4	796	91713	5.4	Cytoplasm	transporter	NONE	1.41E-05
Q9EQI8	RM46_MOUSE	MRPL46	(Q9EQI8) 39S ribosomal protein L46, mitochondrial precursor (L46mt) (MRP-L46)	2	12.7	283	32132	7.4	Cytoplasm	other	NONE	7.93E-05
Q9EQN3	T22D4_MOUSE	TSC22D4	(Q9EQN3) TSC22 domain family protein 4 (TSC22-related-inducible leucine zipper protein 2)	1	4.4	387	39988	8.1	Nucleus	transcription regulator	NONE	2.9E-05
Q9EQP2	EHD4_MOUSE	EHD4	(Q9EQP2) EH-domain-containing protein 4 (mPAST2)	6	17	541	61481	6.8	Plasma Membrane	enzyme	NONE	0.000135
Q9EQS3	MYCBP_MOUSE	MYCBP	(Q9EQS3) C-Myc-binding protein (Associate of Myc 1) (AMY-1)	2	30.4	102	11824	5.9	Nucleus	transcription regulator	NONE	0.00011
Q9EQU5	SET_MOUSE	SET	(Q9EQU5) Protein SET (Phosphatase 2A inhibitor I2PP2A) (I-2PP2A) (Template-activating factor I) (TAF-I)	5	22.5	289	33378	4.3	Nucleus	phosphatase	NONE	0.000291
Q9ER00	STX12_MOUSE	STX12	(Q9ER00) Syntaxin-12	4	20.4	274	31195	5.4	Plasma Membrane	other	1	0.000184
Q9ER35	FN3K_MOUSE	FN3K	(Q9ER35) Fructosamine-3-kinase (EC 2.7.1.-)	1	4.5	309	35032	8.4	Unknown	kinase	NONE	1.82E-05
Q9ER88	RT29_MOUSE	DAP3	(Q9ER88) Mitochondrial 28S ribosomal protein S29 (S29mt) (MRP-S29) (Death-associated protein 3) (DAP-3)	2	7.2	391	44699	8.9	Cytoplasm	other	NONE	2.87E-05
Q9ERB0	SNP29_MOUSE	SNAP29	(Q9ERB0) Synaptosomal-associated protein 29 (SNAP-29) (Vesicle-membrane fusion protein SNAP-29) (Soluble 29 kDa NSF attachment protein) (Golgi SNARE of 32 kDa) (Gs32)	4	21.5	260	29572	5.4	Cytoplasm	transporter	NONE	0.000216
Q9ERD7	TBB3_MOUSE	TUBB3	(Q9ERD7) Tubulin beta-3 chain	2	3.3	450	50419	4.9	Cytoplasm	other	NONE	9.98E-05
Q9ERE7	MESD2_MOUSE	MESDC2	(Q9ERE7) Mesoderm development candidate 2	2	12.9	224	25207	6.3	Unknown	other	1	7.52E-05
Q9ERG0	LIMA1_MOUSE	LIMA1	(Q9ERG0) LIM domain and actin-binding protein 1 (Epithelial protein lost in neoplasm) (mEPLIN)	8	14.1	753	84090	6.6	Cytoplasm	other	NONE	8.95E-05
Q9ERG2	STRN3_MOUSE	STRN3	(Q9ERG2) Striatin-3 (Cell-cycle autoantigen SG2NA) (S/G2 antigen)	1	3.3	796	87150	5.3	Nucleus	other	NONE	7.05E-06
Q9ERI6	RDH14_MOUSE	RDH14	(Q9ERI6) Retinol dehydrogenase 14 (EC 1.1.1.-) (Alcohol dehydrogenase PAN2)	2	7.8	334	36366	8.2	Cytoplasm	enzyme	1	5.04E-05
Q9ERR7	SEP15_MOUSE	SEP15	(Q9ERR7) 15 kDa selenoprotein precursor	3	25.3	162	17731	5.4	Cytoplasm	enzyme	NONE	0.000346

Q9ERS2	NDUAD_MOUSE	NDUFA13	(Q9ERS2) NADH dehydrogenase [ubiquinone] 1 alpha subcomplex subunit 13 (EC 1.6.5.3) (EC 1.6.99.3) (NADH-ubiquinone oxidoreductase B16.6 subunit) (Complex I-B16.6) (CI-B16.6) (Gene associated with retinoic-interferon-induced mortality 19 prote	15	50.3	143	16728	9.5	Cytoplasm	enzyme	1	0.001727
Q9ERT9	IPP1_MOUSE	PPP1R1A	(Q9ERT9) Protein phosphatase inhibitor 1 (IPP-1) (I-1)	2	35.7	171	18718	5.3	Cytoplasm	phosphatase	NONE	0.000197
Q9ERU9	RBP2_MOUSE	RANBP2	(Q9ERU9) Ran-binding protein 2 (RanBP2)	7	4.2	3053	341092	6.2	Nucleus	enzyme	NONE	5.33E-05
Q9ESD7	DYSF_MOUSE	DYSF	(Q9ESD7) Dysferlin (Dystrophy-associated fer-1-like protein) (Fer-1-like protein 1)	1	0.9	2083	237127	5.8	Plasma Membrane	other	1	2.69E-06
Q9ESG4	TMM27_MOUSE	TMEM27	(Q9ESG4) Collectrin precursor (Transmembrane protein 27)	4	23	222	25070	5.8	Plasma Membrane	other	2	0.000152
Q9ESU6	BRD4_MOUSE	BRD4	(Q9ESU6) Bromodomain-containing protein 4 (Mitotic chromosome-associated protein) (MCAP)	2	3.3	1400	155923	9.2	Nucleus	kinase	NONE	8.02E-06
Q9ESW8	PGPI_MOUSE	PGPEP1	(Q9ESW8) Pyroglutamyl-peptidase 1 (EC 3.4.19.3) (Pyroglutamyl-peptidase I) (Pyrrolidone-carboxylate peptidase) (5-oxoprolyl-peptidase) (PGP-I)	1	11	209	22934	5.4	Cytoplasm	peptidase	NONE	0.000107
Q9ESX5	DKC1_MOUSE	BC068171	(Q9ESX5) H/ACA ribonucleoprotein complex subunit 4 (EC 5.4.99.-) (Dyskerin) (Nucleolar protein family A member 4) (snoRNP protein DKC1) (Nopp140-associated protein of 57 kDa) (Nucleolar protein NAP57)	2	5.3	508	57371	9.2	Nucleus	other	NONE	4.42E-05
Q9ESZ8	GTF2I_MOUSE	GTF2I	(Q9ESZ8) General transcription factor II-I (GTFII-I) (TFII-I) (Bruton tyrosine kinase-associated protein 135) (BTK-associated protein 135) (BAP-135)	5	7.8	998	112265	6.6	Nucleus	transcription regulator	NONE	3.37E-05
Q9ET22	DPP2_MOUSE	DPP7	(Q9ET22) Dipeptidyl-peptidase 2 precursor (EC 3.4.14.2) (Dipeptidyl-peptidase II) (DPP II) (Dipeptidyl aminopeptidase II) (Quiescent cell proline dipeptidase) (Dipeptidyl peptidase 7)	2	4.5	506	56270	5.4	Cytoplasm	peptidase	1	2.22E-05
Q9JHI5	IVD_MOUSE	IVD	(Q9JHI5) Isovaleryl-CoA dehydrogenase, mitochondrial precursor (EC 1.3.99.10) (IVD)	13	26.2	424	46325	8.3	Cytoplasm	enzyme	NONE	0.00045
Q9JHJ0	TMOD3_MOUSE	TMOD3	(Q9JHJ0) Tropomodulin-3 (Ubiquitous tropomodulin) (U-Tmod)	6	24.1	352	39503	5.1	Cytoplasm	other	NONE	0.000191
Q9JHL1	NHRF2_MOUSE	SLC9A3R2	(Q9JHL1) Na(+)/H(+) exchange regulatory cofactor NHE-RF2 (NHERF-2) (Tyrosine kinase activator protein 1) (TKA-1) (SRY-interacting protein 1) (SIP-1) (Solute carrier family 9 isoform A3 regulatory factor 2) (NHE3 kinase A regulatory protein E3	6	13.9	337	37393	7.6	Plasma Membrane	transporter	NONE	0.000283
Q9JHS4	CLPX_MOUSE	CLPX	(Q9JHS4) ATP-dependent Clp protease ATP-binding subunit ClpX-like, mitochondrial precursor	13	28.2	634	69314	7.9	Cytoplasm	enzyme	NONE	0.000212
Q9JHU4	DYHC_MOUSE	DYNC1H1	(Q9JHU4) Dynein heavy chain, cytosolic (DYHC) (Cytoplasmic dynein heavy chain 1) (DHC1) (Dynein heavy chain 1, cytoplasmic 1)	13	3.7	4644	532030	6.4	Cytoplasm	peptidase	NONE	2.3E-05
Q9JHW4	SELB_MOUSE	EEFSEC	(Q9JHW4) Selenocysteine-specific elongation factor (Elongation factor sec) (Eukaryotic elongation factor, selenocysteine-tRNA-specific) (mSelB)	2	6.9	583	63417	8.3	Cytoplasm	translation regulator	NONE	7.7E-05
Q9JI13	SAS10_MOUSE	SAS10	(Q9JI13) Something about silencing protein 10 (Disrupter of silencing SAS10) (Charged amino acid-rich leucine zipper 1) (Crl-1)	1	3.2	469	53399	5.5	Nucleus	other	NONE	2.39E-05
Q9JI33	NET4_MOUSE	NTN4	(Q9JI33) Netrin-4 precursor (Beta-netrin)	1	2.4	628	69897	8.2	Extracellular Space	other	NONE	1.79E-05
Q9JIF7	COPB_MOUSE	COPB1	(Q9JIF7) Coatomer subunit beta (Beta-coat protein) (Beta-COP)	3	3.5	953	107066	6	Cytoplasm	transporter	NONE	1.77E-05
Q9JII5	DAZP1_MOUSE	DAZAP1	(Q9JII5) DAZ-associated protein 1 (Deleted in azoospermia-associated protein 1)	2	8.4	406	43214	8.6	Unknown	other	NONE	8.3E-05
Q9JII6	AK1A1_MOUSE	AKR1A1	(Q9JII6) Alcohol dehydrogenase [NADP+] (EC 1.1.1.2) (Aldehyde reductase) (Aldo-keto reductase family 1 member A1)	26	57.7	324	36456	7.4	Unknown	enzyme	NONE	0.005388
Q9JIK5	DDX21_MOUSE	DDX21	(Q9JIK5) Nucleolar RNA helicase 2 (EC 3.6.1.-) (Nucleolar RNA helicase II) (Nucleolar RNA helicase Gu) (RH II/Gu) (Gu-alpha) (DEAD box protein 21)	4	5.9	851	93582	9.1	Nucleus	enzyme	NONE	4.62E-05
Q9JIK9	RT34_MOUSE	MRPS34	(Q9JIK9) Mitochondrial 28S ribosomal protein S34 (S34mt) (MRP-S34) (T-complex expressed gene 2 protein)	3	19.3	218	25827	10.4	Cytoplasm	other	NONE	0.000232

Q9JIL4	PDZK1_MOUSE	PDZK1	(Q9JIL4) PDZ domain-containing protein 1 (CFTR-associated protein of 70 kDa) (Na/Pi cotransporter C-terminal-associated protein) (NaPi-Cap1) (Na(+)/H(+) exchanger regulatory factor 3) (Sodium-hydrogen exchanger regulatory factor 3)	55	75.1	519	56499	5.4	Plasma Membrane	transporter	NONE	0.005235
Q9JIL5	TULP4_MOUSE	TULP4	(Q9JIL5) Tubby-like protein 4 (Tubby superfamily protein)	2	1.8	1547	169638	7.5	Cytoplasm	transcription regulator	NONE	7.26E-06
Q9JIQ3	DBLOH_MOUSE	DIABLO	(Q9JIQ3) Diablo homolog, mitochondrial precursor (Second mitochondria-derived activator of caspase) (Smac protein) (Direct IAP-binding protein with low pI)	1	5.5	237	26829	6.5	Cytoplasm	other	NONE	2.37E-05
Q9JIW9	RALB_MOUSE	RALB	(Q9JIW9) Ras-related protein Ral-B	3	15.5	206	23349	6.6	Cytoplasm	enzyme	NONE	0.000191
Q9JIX8	ACINU_MOUSE	ACIN1	(Q9JIX8) Apoptotic chromatin condensation inducer in the nucleus (Acinus)	3	4.4	1338	150691	5.9	Nucleus	enzyme	NONE	2.1E-05
Q9JIY5	HTRA2_MOUSE	HTRA2	(Q9JIY5) Serine protease HTRA2, mitochondrial precursor (EC 3.4.21.-) (High temperature requirement protein A2) (HtrA2) (Omi stress-regulated endoprotease) (Serine proteinase OMI)	1	5.7	458	49348	9.6	Cytoplasm	peptidase	NONE	2.45E-05
Q9JJ59	ABCB9_MOUSE	ABCB9	(Q9JJ59) ATP-binding cassette sub-family B member 9 precursor (ATP-binding cassette transporter 9) (ABC transporter 9 protein) (TAP-like protein) (TAPL) (mABCB9)	2	3.4	762	83963	7.6	Cytoplasm	transporter	8	1.47E-05
Q9JJG0	TACC2_MOUSE	TACC2	(Q9JJG0) Transforming acidic coiled-coil-containing protein 2	1	2.6	1035	112716	5	Nucleus	other	NONE	1.08E-05
Q9JJI8	RL38_MOUSE	RPL38 (includes EG:6169)	(Q9JJI8) 60S ribosomal protein L38	3	18.8	69	8073	10.1	Cytoplasm	other	NONE	0.00179
Q9JJL8	SYSM_MOUSE	SARS2	(Q9JJL8) Seryl-tRNA synthetase, mitochondrial precursor (EC 6.1.1.11) (Serine--tRNA ligase) (SerRSmt)	3	11.4	518	58302	7.9	Cytoplasm	enzyme	NONE	8.67E-05
Q9JJR7	ASCL3_MOUSE	ASCL3 (includes EG:56676)	(Q9JJR7) Achaete-scute homolog 3 (bHLH transcriptional regulator Sgn-1) (Mash-3)	1	10.3	174	20245	8.4	Nucleus	transcription regulator	NONE	3.23E-05
Q9JJR8	TMM9B_MOUSE	TMEM9B	(Q9JJR8) Transmembrane protein 9B precursor	2	25.6	199	22607	8.2	Plasma Membrane	other	2	5.64E-05
Q9JJU8	SH3L1_MOUSE	SH3BGRL	(Q9JJU8) SH3 domain-binding glutamic acid-rich-like protein	1	14	114	12811	4.9	Unknown	other	NONE	0.000197
Q9JJW6	REFP2_MOUSE	REFBP2	(Q9JJW6) RNA and export factor-binding protein 2	1	5	218	23730	10	Unknown	other	NONE	5.15E-05
Q9JK38	GNA1_MOUSE	GNPNAT1	(Q9JK38) Glucosamine 6-phosphate N-acetyltransferase (EC 2.3.1.4) (Phosphoglucosamine transacetylase) (Phosphoglucosamine acetylase) (EMeg32 protein)	1	8.2	184	20791	7.7	Cytoplasm	enzyme	NONE	9.15E-05
Q9JK48	SHLB1_MOUSE	SH3GLB1	(Q9JK48) SH3 domain GRB2-like protein B1 (EC 2.3.1.-) (Endophilin B1)	1	3.6	365	40855	6	Cytoplasm	enzyme	NONE	3.08E-05
Q9JK53	PRELP_MOUSE	PRELP	(Q9JK53) Prolargin precursor (Proline-arginine-rich end leucine-rich repeat protein)	1	3.2	378	43293	9.5	Extracellular Space	other	1	2.97E-05
Q9JK81	MYG1_MOUSE	C12ORF10	(Q9JK81) Protein MYG1 (Protein Gamm1)	2	5.3	380	42723	7	Unknown	other	NONE	2.95E-05
Q9JKB1	UCHL3_MOUSE	UCHL3	(Q9JKB1) Ubiquitin carboxyl-terminal hydrolase isozyme L3 (EC 3.4.19.12) (UCH-L3) (Ubiquitin thioesterase L3)	3	19.1	230	26152	5	Cytoplasm	peptidase	NONE	0.000122
Q9JKB3	DBPA_MOUSE	CSDA	(Q9JKB3) DNA-binding protein A (Cold shock domain-containing protein A) (Y-box protein 3)	5	24.7	361	38814	9.7	Nucleus	transcription regulator	NONE	0.00028
Q9JKC8	AP3M1_MOUSE	AP3M1	(Q9JKC8) AP-3 complex subunit mu-1 (Adapter-related protein complex 3 mu-1 subunit) (Mu-adaptin 3A) (AP-3 adapter complex mu3A subunit)	1	5.5	418	46936	6.9	Cytoplasm	transporter	NONE	2.69E-05
Q9JKF1	IQGA1_MOUSE	IQGAP1 (includes EG:8826)	(Q9JKF1) Ras GTPase-activating-like protein IQGAP1	2	2.2	1657	188755	6.5	Cytoplasm	other	NONE	1.02E-05
Q9JKF6	PVRL1_MOUSE	PVRL1	(Q9JKF6) Poliovirus receptor-related protein 1 precursor (Herpes virus entry mediator C) (HveC) (Nectin-1) (CD111 antigen)	2	5.2	515	57064	6.5	Plasma Membrane	other	1	3.27E-05
Q9JKX6	NUDT5_MOUSE	NUDT5	(Q9JKX6) ADP-sugar pyrophosphatase (EC 3.6.1.13) (EC 3.6.1.-) (Nucleoside diphosphate-linked moiety X motif 5) (Nudix motif 5)	1	6	218	23984	5.5	Cytoplasm	phosphatase	NONE	2.57E-05
Q9JL35	NSBP1_MOUSE	NSBP1	(Q9JL35) Nucleosome-binding protein 1 (Nucleosome-binding protein 45) (NBP-45) (GARP45 protein)	3	8.9	406	45344	4.4	Nucleus	transcription regulator	NONE	6.91E-05

Q9JLB0	MPP6_MOUSE	MPP6	(Q9JLB0) MAGUK p55 subfamily member 6 (Protein associated with Lin-7 2) (Dlgh4 protein) (P55T protein)	2	6.5	553	62631	6.4	Plasma Membrane	kinase	NONE	5.08E-05
Q9JLB4	CUBN_MOUSE	CUBN	(Q9JLB4) Cubilin precursor (Intrinsic factor-cobalamin receptor)	5	1.9	3623	399070	5.8	Plasma Membrane	transmembrane receptor	NONE	3.56E-05
Q9JLC8	SACS_MOUSE	SACS	(Q9JLC8) Sacsin	2	0.9	3830	436758	7.1	Nucleus	other	NONE	4.4E-06
Q9JLJ2	AL9A1_MOUSE	ALDH9A1	(Q9JLJ2) 4-trimethylaminobutyraldehyde dehydrogenase (EC 1.2.1.47) (TMABADH) (Aldehyde dehydrogenase 9A1) (EC 1.2.1.3)	5	10.9	494	53515	7	Cytoplasm	enzyme	NONE	0.000159
Q9JLQ0	CD2AP_MOUSE	CD2AP	(Q9JLQ0) CD2-associated protein (Mesenchyme-to-epithelium transition protein with SH3 domains 1) (METS-1)	6	8	637	70432	6.4	Cytoplasm	other	NONE	9.69E-05
Q9JLT2	TREA_MOUSE	TREH	(Q9JLT2) Trehalase precursor (EC 3.2.1.28) (Alpha,alpha-trehalase) (Alpha,alpha-trehalose glucohydrolase)	7	18.2	576	65401	5.6	Plasma Membrane	enzyme	2	0.000185
Q9JLZ3	AUHM_MOUSE	AUH	(Q9JLZ3) Methylglutaconyl-CoA hydratase, mitochondrial precursor (EC 4.2.1.18) (AU-specific RNA-binding enoyl-CoA hydratase) (AU-binding enoyl-CoA hydratase) (muAUH)	6	18.2	314	33395	9.5	Cytoplasm	enzyme	NONE	0.000322
Q9JLZ8	SIGIR_MOUSE	SIGIRR	(Q9JLZ8) Single Ig IL-1-related receptor (Single Ig IL-1R-related molecule) (Single immunoglobulin domain-containing IL1R-related protein) (Toll/interleukin-1 receptor 8) (TIR8)	4	10.8	409	46159	5.7	Plasma Membrane	transmembrane receptor	1	0.00011
Q9JM14	NT5C_MOUSE	NT5C	(Q9JM14) 5'(3')-deoxyribonucleotidase, cytosolic type (EC 3.1.3.-) (Cytosolic 5',3'-pyrimidine nucleotidase) (Deoxy-5'-nucleotidase 1) (dNT-1)	6	38.5	200	23076	5.5	Cytoplasm	phosphatase	NONE	0.000449
Q9JM63	IRK10_MOUSE	KCNJ10	(Q9JM63) ATP-sensitive inward rectifier potassium channel 10 (Potassium channel, inwardly rectifying subfamily J member 10) (Inward rectifier K(+) channel Kir4.1)	1	5.3	379	42432	8.3	Plasma Membrane	ion channel	2	2.96E-05
Q9JM76	ARPC3_MOUSE	ARPC3	(Q9JM76) Actin-related protein 2/3 complex subunit 3 (ARP2/3 complex 21 kDa subunit) (p21-ARC)	2	6.2	177	20393	8.6	Cytoplasm	other	NONE	0.000222
Q9JMA1	UBP14_MOUSE	USP14	(Q9JMA1) Ubiquitin carboxyl-terminal hydrolase 14 (EC 3.1.2.15) (Ubiquitin thioesterase 14) (Ubiquitin-specific-processing protease 14) (Deubiquitinating enzyme 14)	4	11.8	492	55871	5.2	Cytoplasm	peptidase	NONE	6.85E-05
Q9JMB7	PIWL1_MOUSE	PIWIL1	(Q9JMB7) Piwi-like protein 1	2	1.6	862	98574	9.4	Cytoplasm	other	NONE	2.6E-05
Q9JMG1	EDF1_MOUSE	EDF1	(Q9JMG1) Endothelial differentiation-related factor 1 (EDF-1) (Multiprotein-bridging factor 1) (MBF1)	2	10.1	148	16369	10	Nucleus	transcription regulator	NONE	0.000265
Q9JMH6	TRXR1_MOUSE	TXNRD1	(Q9JMH6) Thioredoxin reductase 1, cytoplasmic (EC 1.8.1.9) (TR) (TR1)	3	11.8	499	54497	6.3	Cytoplasm	enzyme	2	5.62E-05
Q9JMH9	MY18A_MOUSE	MYO18A (includes EG:399687)	(Q9JMH9) Myosin-18A (Myosin XVIIIa) (Myosin containing PDZ domain) (Molecule associated with JAK3 N-terminus) (MAJN)	4	3.6	2035	230906	6.2	Unknown	other	NONE	1.38E-05
Q9NYQ2	HAOX2_MOUSE	HAO2 (includes EG:51179)	(Q9NYQ2) Hydroxyacid oxidase 2 (EC 1.1.3.15) (HAOX2) ((S)-2-hydroxy-acid oxidase, peroxisomal) (Medium chain alpha-hydroxy acid oxidase) (Medium-chain L-2-hydroxy acid oxidase)	7	24.1	353	38700	7.6	Cytoplasm	enzyme	NONE	0.001415
Q9QUH0	GLRX1_MOUSE	GLRX	(Q9QUH0) Glutaredoxin-1 (Thioltransferase-1) (TTase-1)	4	35.8	106	11740	8.4	Cytoplasm	enzyme	NONE	0.001589
Q9QUI0	RHOA_MOUSE	RHOA	(Q9QUI0) Transforming protein RhoA precursor	4	21.2	193	21782	6.1	Cytoplasm	enzyme	NONE	0.000175
Q9QUM9	PSA6_MOUSE	PSMA6	(Q9QUM9) Proteasome subunit alpha type 6 (EC 3.4.25.1) (Proteasome iota chain) (Macropain iota chain) (Multicatalytic endopeptidase complex iota chain)	4	10.6	246	27372	6.7	Cytoplasm	peptidase	NONE	0.000137
Q9QUR7	PIN1_MOUSE	PIN1	(Q9QUR7) Peptidyl-prolyl cis-trans isomerase NIMA-interacting 1 (EC 5.2.1.8) (Rotamase Pin1) (PPIase Pin1)	4	20.6	165	18370	8.8	Nucleus	enzyme	NONE	0.000272
Q9QWI6	SNIP_MOUSE	SNIP	(Q9QWI6) p130Cas-associated protein (p140Cap) (SNAP-25-interacting protein) (SNIP)	2	3.4	1250	134858	9.3	Cytoplasm	other	NONE	1.35E-05
Q9QWR8	NAGAB_MOUSE	NAGA	(Q9QWR8) Alpha-N-acetylgalactosaminidase precursor (EC 3.2.1.49) (Alpha-galactosidase B)	3	6.7	415	47235	6.4	Cytoplasm	enzyme	1	8.12E-05
Q9QXA5	LSM4_MOUSE	LSM4	(Q9QXA5) U6 snRNA-associated Sm-like protein LSm4	2	9.5	137	15076	10.1	Nucleus	other	NONE	0.000164

Q9QXD6	F16P1_MOUSE	FBP1	(Q9QXD6) Fructose-1,6-bisphosphatase 1 (EC 3.1.3.11) (D-fructose-1,6-bisphosphate 1-phosphohydrolase 1) (FBPase 1)	31	52.8	337	36781	6.6	Cytoplasm	phosphatase	NONE	0.008911
Q9QXL2	KI21A_MOUSE	KIF21A	(Q9QXL2) Kinesin family member 21A	5	5.3	1672	186535	6.3	Cytoplasm	other	NONE	3.02E-05
Q9QXN5	MIOX_MOUSE	MIOX	(Q9QXN5) Inositol oxygenase (EC 1.13.99.1) (Myo-inositol oxygenase) (Aldehyde reductase-like 6) (Renal-specific oxidoreductase)	2	8.1	285	33164	5.3	Cytoplasm	enzyme	NONE	9.85E-05
Q9QXS6	DREB_MOUSE	DBN1	(Q9QXS6) Drebrin (Developmentally-regulated brain protein)	1	2.3	705	77156	4.5	Cytoplasm	other	NONE	1.59E-05
Q9QXT0	MSAP_MOUSE	TMEM4	(Q9QXT0) MIR-interacting saposin-like protein precursor (Transmembrane protein 4) (Putative secreted protein ZSIG9)	4	29.7	182	20767	5.1	Plasma Membrane	other	1	0.000401
Q9QXW9	LAT2_MOUSE	SLC7A8	(Q9QXW9) Large neutral amino acids transporter small subunit 2 (L-type amino acid transporter 2)	2	4.5	531	57873	6.7	Plasma Membrane	transporter	13	3.17E-05
Q9QXX0	JAG1_MOUSE	JAG1	(Q9QXX0) Jagged-1 precursor (Jagged1) (CD339 antigen)	1	2.3	1218	134164	6.2	Extracellular Space	growth factor	2	4.61E-06
Q9QXX4	CMC2_MOUSE	SLC25A13 (includes EG:10165)	(Q9QXX4) Calcium-binding mitochondrial carrier protein Aralar2 (Mitochondrial aspartate glutamate carrier 2) (Solute carrier family 25 member 13) (Citrin)	10	24.9	676	74467	8.6	Cytoplasm	transporter	NONE	0.000349
Q9QXY6	EHD3_MOUSE	EHD3	(Q9QXY6) EH-domain-containing protein 3	3	13.8	535	60869	6.4	Cytoplasm	other	NONE	6.3E-05
Q9QXZ6	SO1A1_MOUSE	SLCO1A1	(Q9QXZ6) Solute carrier organic anion transporter family member 1A1 (Solute carrier family 21 member 1) (Sodium-independent organic anion-transporting polypeptide 1) (OATP1)	2	5.1	670	74396	8.2	Plasma Membrane	transporter	8	1.68E-05
Q9QY06	MYO9B_MOUSE	MYO9B	(Q9QY06) Myosin-9B (Myosin IXb) (Unconventional myosin-9b)	2	1.7	2114	238832	8.6	Cytoplasm	enzyme	NONE	5.31E-06
Q9QY53	NPHP1_MOUSE	NPHP1	(Q9QY53) Nephrocystin-1	1	2	687	77035	5.3	Nucleus	other	NONE	8.17E-06
Q9QY76	VAPB_MOUSE	VAPB	(Q9QY76) Vesicle-associated membrane protein-associated protein B (VAMP-associated protein B) (VAMP-associated protein 33b) (VAMP-B) (VAP-B)	4	11.6	242	26815	7.8	Plasma Membrane	other	1	0.000487
Q9QYB1	CLIC4_MOUSE	CLIC4	(Q9QYB1) Chloride intracellular channel protein 4 (mc3s5/mtCLIC)	12	40.1	252	28598	5.6	Cytoplasm	ion channel	NONE	0.000846
Q9QYB5	ADDG_MOUSE	ADD3	(Q9QYB5) Gamma-adducin (Adducin-like protein 70)	7	15.3	706	78763	5.9	Cytoplasm	other	NONE	8.75E-05
Q9QYC0	ADDA_MOUSE	ADD1	(Q9QYC0) Alpha-adducin (Erythrocyte adducin subunit alpha)	10	19.3	735	80647	5.9	Cytoplasm	other	NONE	0.000122
Q9QYE6	GOGA5_MOUSE	GOLGA5	(Q9QYE6) Golgin subfamily A member 5 (Golgin-84) (Sumiko protein) (Ret-II protein)	3	10	729	82368	6.2	Cytoplasm	kinase	1	3.08E-05
Q9QYG0	NDRG2_MOUSE	NDRG2	(Q9QYG0) Protein NDRG2 (Protein Ndr2)	1	3.8	371	40789	5.4	Cytoplasm	other	NONE	3.03E-05
Q9QYI3	DNJC7_MOUSE	DNAJC7	(Q9QYI3) DnaJ homolog subfamily C member 7 (Tetratricopeptide repeat protein 2) (TPR repeat protein 2) (MDj11)	5	12.3	494	56476	6.5	Cytoplasm	other	NONE	5.68E-05
Q9QYJ0	DNJA2_MOUSE	DNAJA2	(Q9QYJ0) DnaJ homolog subfamily A member 2 (mDj3)	2	9.2	412	45746	6.5	Nucleus	enzyme	NONE	4.09E-05
Q9QYR9	ACOT2_MOUSE	ACOT2	(Q9QYR9) Acyl-coenzyme A thioesterase 2, mitochondrial precursor (EC 3.1.2.2) (Acyl-CoA thioesterase 2) (Acyl coenzyme A thioester hydrolase) (Very-long-chain acyl-CoA thioesterase) (MTE-I)	5	13	453	49652	7.4	Cytoplasm	enzyme	NONE	0.000161
Q9QYS9	QKI_MOUSE	QKI	(Q9QYS9) Quaking protein (qkI)	4	16.1	341	37671	8.5	Nucleus	other	NONE	0.000115
Q9QYX7	PCLO_MOUSE	PCLO	(Q9QYX7) Protein piccolo (Aczonin) (Multidomain presynaptic cytomatrix protein) (Brain-derived HLMN protein)	2	0.5	5038	547578	6.4	Cytoplasm	transporter	NONE	2.23E-06
Q9QZ06	TOLIP_MOUSE	TOLLIP	(Q9QZ06) Toll-interacting protein	1	4.7	274	30345	5.2	Cytoplasm	other	NONE	2.05E-05
Q9QZ23	HIRP5_MOUSE	NFU1	(Q9QZ23) HIRA-interacting protein 5 (mHIRIP5)	2	11.6	199	22140	4.4	Unknown	other	NONE	0.000141
Q9QZ88	VPS29_MOUSE	VPS29	(Q9QZ88) Vacuolar protein sorting 29 (Vesicle protein sorting 29)	1	12.6	182	20496	6.8	Cytoplasm	transporter	NONE	3.08E-05
Q9QZD8	DIC_MOUSE	SLC25A10	(Q9QZD8) Mitochondrial dicarboxylate carrier (Solute carrier family 25 member 10)	5	16	287	31715	9.3	Cytoplasm	transporter	NONE	0.00045
Q9QZD9	IF32_MOUSE	EIF3S2 (includes EG:8668)	(Q9QZD9) Eukaryotic translation initiation factor 3 subunit 2 (eIF-3 beta) (eIF3 p36) (eIF3i) (TGF-beta receptor-interacting protein 1) (TRIP-1)	3	12.3	325	36461	5.6	Cytoplasm	translation regulator	NONE	6.91E-05
Q9QZH3	PPIE_MOUSE	PPIE	(Q9QZH3) Peptidyl-prolyl cis-trans isomerase E (EC 5.2.1.8) (PPIase E) (Rotamase E) (Cyclophilin E) (Cyclophilin 33)	2	13	301	33449	5.6	Nucleus	enzyme	NONE	5.59E-05

Q9QZM0	UBQL2_MOUSE	UBQLN2	(Q9QZM0) Ubiquilin-2 (Protein linking IAP with cytoskeleton 2) (PLIC-2) (Ubiquitin-like product Chap1/Dsk2) (DSK2 homolog) (Chap1)	1	2.4	638	67379	5.3	Nucleus	other	NONE	8.8E-06
Q9QZQ8	H2AY_MOUSE	H2AFY	(Q9QZQ8) Core histone macro-H2A.1 (Histone macroH2A1) (mH2A1) (H2A.y) (H2A/y)	9	27	371	39604	9.8	Nucleus	other	NONE	0.000454
Q9QZR0	RNF25_MOUSE	RNF25	(Q9QZR0) RING finger protein 25 (EC 6.3.2.-) (RING finger protein AO7)	1	4.4	456	51227	6.3	Nucleus	transcription regulator	NONE	1.23E-05
Q9QZS7	NPHN_MOUSE	NPHS1	(Q9QZS7) Nephrin precursor (Renal glomerulus-specific cell adhesion receptor)	2	2.9	1242	134890	5.6	Plasma Membrane	other	NONE	9.04E-06
Q9R022	DJC12_MOUSE	DNAJC12	(Q9R022) DnaJ homolog subfamily C member 12 (J domain-containing protein 1)	6	26.3	198	22853	6.2	Unknown	other	NONE	0.00034
Q9R062	GLYG_MOUSE	GYG1	(Q9R062) Glycogenin-1 (EC 2.4.1.186)	2	9.6	332	37271	5.3	Cytoplasm	enzyme	NONE	6.76E-05
Q9R0A0	PEX14_MOUSE	PEX14	(Q9R0A0) Peroxisomal membrane protein PEX14 (Peroxin-14) (Peroxisomal membrane anchor protein PEX14) (PTS1 receptor docking protein)	2	6.4	376	41208	5.1	Cytoplasm	other	NONE	4.48E-05
Q9R0C0	PLDN_MOUSE	PLDN	(Q9R0C0) Pallidin (Pallid protein) (Syntaxin 13-interacting protein)	1	11	172	19682	6.3	Cytoplasm	other	NONE	6.53E-05
Q9R0H0	ACOX1_MOUSE	ACOX1	(Q9R0H0) Acyl-coenzyme A oxidase 1, peroxisomal (EC 1.3.3.6) (Palmitoyl-CoA oxidase) (AOX)	17	37.1	661	74634	8.6	Cytoplasm	enzyme	NONE	0.000433
Q9R0M0	CELR2_MOUSE	CELSR2	(Q9R0M0) Cadherin EGF LAG seven-pass G-type receptor 2 precursor (Flamingo 1) (mFmi1)	2	1.3	2920	317593	5.7	Plasma Membrane	G-protein coupled receptor	8	7.69E-06
Q9R0P3	ESTD_MOUSE	ESD	(Q9R0P3) Esterase D (EC 3.1.1.1) (Esterase 10) (Sid 478)	9	21.3	282	31320	7.1	Cytoplasm	enzyme	NONE	0.000577
Q9R0P5	DEST_MOUSE	DSTN	(Q9R0P5) Destrin (Actin-depolymerizing factor) (ADF) (Sid 23)	3	29.9	164	18390	8	Cytoplasm	other	NONE	0.00024
Q9R0P9	UCHL1_MOUSE	UCHL1	(Q9R0P9) Ubiquitin carboxyl-terminal hydrolase isozyme L1 (EC 3.4.19.12) (EC 6.-.-.-) (UCH-L1) (Ubiquitin thioesterase L1) (Neuron cytoplasmic protein 9.5) (PGP 9.5) (PGP9.5)	4	16.1	223	24838	5.2	Cytoplasm	peptidase	NONE	0.000176
Q9R0U0	FUSIP_MOUSE	FUSIP1	(Q9R0U0) FUS-interacting serine-arginine-rich protein 1 (TLS-associated protein with Ser-Arg repeats) (TLS-associated protein with SR repeats) (TASR) (TLS-associated serine-arginine protein) (TLS-associated SR protein) (Neural-specific SR pro	3	14.9	262	31301	11.3	Nucleus	other	NONE	0.000193
Q9R0Y5	KAD1_MOUSE	AK1	(Q9R0Y5) Adenylate kinase isoenzyme 1 (EC 2.7.4.3) (ATP-AMP transphosphorylase) (AK1) (Myokinase)	7	39.2	194	21540	5.8	Cytoplasm	kinase	NONE	0.000723
Q9R112	SQRD_MOUSE	SQRDL	(Q9R112) Sulfide:quinone oxidoreductase, mitochondrial precursor (EC 1.-.-.-)	11	18.2	450	50340	9	Cytoplasm	enzyme	NONE	0.000437
Q9R190	MTA2_MOUSE	MTA2	(Q9R190) Metastasis-associated protein MTA2 (Metastasis-associated 1-like 1)	5	8.8	668	75030	9.7	Nucleus	transcription regulator	NONE	4.2E-05
Q9R1C7	PRP40_MOUSE	PRPF40A	(Q9R1C7) Pre-mRNA-processing factor 40 homolog A (Formin-binding protein 3) (Formin-binding protein 11) (FBP 11)	1	1.8	953	108481	7.7	Nucleus	other	NONE	5.89E-06
Q9R1K9	CETN2_MOUSE	CETN2	(Q9R1K9) Centrin-2 (Caltractin isoform 1)	2	9.3	172	19796	5	Nucleus	enzyme	NONE	9.79E-05
Q9R1P0	PSA4_MOUSE	PSMA4	(Q9R1P0) Proteasome subunit alpha type 4 (EC 3.4.25.1) (Proteasome component C9) (Macropain subunit C9) (Multicatalytic endopeptidase complex subunit C9) (Proteasome subunit L)	2	8	261	29471	7.7	Cytoplasm	peptidase	NONE	6.45E-05
Q9R1P1	PSB3_MOUSE	PSMB3	(Q9R1P1) Proteasome subunit beta type 3 (EC 3.4.25.1) (Proteasome theta chain) (Proteasome chain 13) (Proteasome component C10-II)	1	7.8	205	22965	6.5	Cytoplasm	peptidase	NONE	5.48E-05
Q9R1P3	PSB2_MOUSE	PSMB2	(Q9R1P3) Proteasome subunit beta type 2 (EC 3.4.25.1) (Proteasome component C7-I) (Macropain subunit C7-I) (Multicatalytic endopeptidase complex subunit C7-I)	3	13.9	201	22906	7	Cytoplasm	peptidase	NONE	0.000168
Q9R1P4	PSA1_MOUSE	PSMA1	(Q9R1P4) Proteasome subunit alpha type 1 (EC 3.4.25.1) (Proteasome component C2) (Macropain subunit C2) (Multicatalytic endopeptidase complex subunit C2) (Proteasome nu chain)	7	19.4	263	29547	6.4	Cytoplasm	peptidase	NONE	0.000341
Q9R1T2	ULE1A_MOUSE	SAE1	(Q9R1T2) Ubiquitin-like 1-activating enzyme E1A (SUMO-1-activating enzyme subunit 1)	2	8	350	38620	5.4	Cytoplasm	enzyme	NONE	6.42E-05
Q9R1T4	SEPT6_MOUSE	SPET6	(Q9R1T4) Septin-6	2	9.7	433	49488	6.4	Cytoplasm	other	NONE	6.48E-05
Q9R1Z7	PTPS_MOUSE	PTS	(Q9R1Z7) 6-pyruvoyl tetrahydrobiopterin synthase (EC 4.2.3.12) (PTPS) (PTP synthase)	1	9	144	16188	6.5	Unknown	enzyme	NONE	3.9E-05

Q9R1Z8	VINEX_MOUSE	SORBS3	(Q9R1Z8) Vinexin (Sorbin and SH3 domain-containing protein 3) (SH3-containing adapter molecule 1) (SCAM-1) (SH3 domain-containing protein SH3P3)	4	9.7	733	82349	9.2	Cytoplasm	other	NONE	3.83E-05
Q9R233	TPSN_MOUSE	TAPBP	(Q9R233) Tapasin precursor (TPSN) (TPN) (TAP-binding protein) (TAP-associated protein)	2	9.5	465	49736	8.5	Cytoplasm	transporter	1	7.24E-05
Q9R257	HEBP1_MOUSE	HEBP1	(Q9R257) Heme-binding protein 1 (p22HBP)	7	51.6	190	21053	5.3	Cytoplasm	other	NONE	0.000414
Q9WTI7	MYO1C_MOUSE	MYO1C	(Q9WTI7) Myosin Ic (Myosin I beta) (MMIb)	3	5.2	1028	118156	9.4	Cytoplasm	other	NONE	3.82E-05
Q9WTP6	KAD2_MOUSE	AK2	(Q9WTP6) Adenylate kinase isoenzyme 2, mitochondrial (EC 2.7.4.3) (ATP-AMP transphosphorylase)	18	55.8	231	25474	7.4	Cytoplasm	kinase	NONE	0.001944
Q9WTP7	KAD3_MOUSE	AK3	(Q9WTP7) GTP:AMP phosphotransferase mitochondrial (EC 2.7.4.10) (Adenylate kinase 3) (AK3) (Adenylate kinase 3 alpha-like 1)	17	63.7	226	25295	8.8	Cytoplasm	kinase	NONE	0.001515
Q9WTX5	SKP1_MOUSE	SKP1A	(Q9WTX5) S-phase kinase-associated protein 1A (Cyclin A/CDK2-associated protein p19) (p19A) (p19skp1)	5	37	162	18541	4.5	Nucleus	transcription regulator	NONE	0.00052
Q9WU28	PFD5_MOUSE	PFDN5	(Q9WU28) Prefoldin subunit 5 (C-myc-binding protein Mm-1) (Myc modulator 1) (EIG-1)	1	11.7	154	17356	6.3	Nucleus	transcription regulator	NONE	0.000146
Q9WU78	PDC6I_MOUSE	PDCD6IP	(Q9WU78) Programmed cell death 6-interacting protein (ALG-2-interacting protein X) (ALG-2-interacting protein 1) (E2F1-inducible protein) (Eig2)	2	6	869	96010	6.5	Cytoplasm	other	NONE	1.94E-05
Q9WU79	PROD_MOUSE	PRODH	(Q9WU79) Proline oxidase, mitochondrial precursor (EC 1.5.3.-) (Proline dehydrogenase)	6	18.1	497	56774	6.8	Cytoplasm	enzyme	NONE	0.000169
Q9WU84	CCS_MOUSE	CCS	(Q9WU84) Copper chaperone for superoxide dismutase (Superoxide dismutase copper chaperone)	1	8.8	274	28912	6.1	Cytoplasm	enzyme	NONE	0.000102
Q9WUD1	STUB1_MOUSE	STUB1	(Q9WUD1) STIP1 homology and U box-containing protein 1 (EC 6.3.2.-) (STIP1 homology and U-box-containing protein 1) (Carboxy terminus of Hsp70-interacting protein) (E3 ubiquitin protein ligase CHIP)	5	20.4	304	34909	6	Cytoplasm	enzyme	NONE	0.000129
Q9WUK2	IF4H_MOUSE	EIF4H	(Q9WUK2) Eukaryotic translation initiation factor 4H (eIF-4H) (Williams-Beuren syndrome chromosome region 1 protein homolog)	4	20.6	247	27210	7.2	Cytoplasm	translation regulator	NONE	0.000568
Q9WUL7	ARL3_MOUSE	ARL3	(Q9WUL7) ADP-ribosylation factor-like protein 3	2	10.4	182	20487	7.2	Unknown	other	NONE	0.000123
Q9WUM3	COR1B_MOUSE	CORO1B	(Q9WUM3) Coronin-1B (Coronin-2)	3	7	484	53912	5.8	Cytoplasm	other	NONE	6.96E-05
Q9WUM4	COR1C_MOUSE	CORO1C	(Q9WUM4) Coronin-1C (Coronin-3)	3	14.1	474	53121	7.1	Cytoplasm	other	NONE	7.11E-05
Q9WUM5	SUCA_MOUSE	SUCLG1	(Q9WUM5) Succinyl-CoA ligase [GDP-forming] alpha-chain, mitochondrial precursor (EC 6.2.1.4) (Succinyl-CoA synthetase, alpha chain) (SCS-alpha)	13	29.4	333	34994	9.4	Cytoplasm	enzyme	NONE	0.00295
Q9WUP7	UCHL5_MOUSE	UCHL5	(Q9WUP7) Ubiquitin carboxyl-terminal hydrolase isozyme L5 (EC 3.4.19.12) (UCH-L5) (Ubiquitin thioesterase L5) (Ubiquitin C-terminal hydrolase UCH37)	1	4.3	329	37617	5.3	Cytoplasm	peptidase	NONE	3.41E-05
Q9WUQ2	PREB_MOUSE	PREB	(Q9WUQ2) Prolactin regulatory element-binding protein (Mammalian guanine nucleotide exchange factor mSec12)	2	6	417	45437	8.8	Nucleus	transcription regulator	2	4.04E-05
Q9WUR2	PECI_MOUSE	PECI	(Q9WUR2) Peroxisomal 3,2-trans-enoyl-CoA isomerase (EC 5.3.3.8) (Dodecenoyl-CoA isomerase) (Delta(3),delta(2)-enoyl-CoA isomerase) (D3,D2-enoyl-CoA isomerase)	9	25.4	358	39479	8	Cytoplasm	enzyme	NONE	0.000455
Q9WUR9	KAD4_MOUSE	AK3L1	(Q9WUR9) Adenylate kinase isoenzyme 4, mitochondrial (EC 2.7.4.3) (Adenylate kinase 3-like 1) (ATP-AMP transphosphorylase)	17	57	223	25062	7.5	Cytoplasm	kinase	NONE	0.001737
Q9WUU9	MCM3A_MOUSE	MCM3AP	(Q9WUU9) 80 kDa MCM3-associated protein (GANP protein)	2	0.7	1971	217138	6.6	Nucleus	other	NONE	5.7E-06
Q9WUZ9	ENP5_MOUSE	ENTPD5	(Q9WUZ9) Ectonucleoside triphosphate diphosphohydrolase 5 precursor (EC 3.6.1.6) (NTPDase5) (Nucleoside diphosphatase) (CD39 antigen-like 4) (ER-UDPase)	3	14.1	427	47102	5.3	Cytoplasm	enzyme	2	0.000105
Q9WV27	AT1A4_MOUSE	ATP1A4	(Q9WV27) Sodium/potassium-transporting ATPase alpha-4 chain (EC 3.6.3.9) (Sodium pump 4) (Na+/K+ ATPase 4)	4	3.4	1032	114816	5.7	Plasma Membrane	transporter	9	8.16E-05
Q9WV32	ARC1B_MOUSE	ARPC1B	(Q9WV32) Actin-related protein 2/3 complex subunit 1B (ARP2/3 complex 41 kDa subunit) (p41-ARC)	2	6.2	371	41018	8.4	Cytoplasm	other	NONE	4.54E-05

Q9WV55	VAPA_MOUSE	VAPA	(Q9WV55) Vesicle-associated membrane protein-associated protein A (VAMP-associated protein A) (VAMP-A) (VAP-A) (33 kDa Vamp-associated protein) (VAP-33)	5	19.4	242	27280	8.4	Plasma Membrane	other	1	0.000394
Q9WV69	DEMA_MOUSE	EPB49	(Q9WV69) Dematin (Erythrocyte membrane protein band 4.9)	2	10.6	405	45468	8.4	Plasma Membrane	other	NONE	4.16E-05
Q9WV80	SNX1_MOUSE	SNX1	(Q9WV80) Sorting nexin-1	13	26.2	522	58952	5.2	Cytoplasm	transporter	NONE	0.000247
Q9WV85	NDK3_MOUSE	NME3	(Q9WV85) Nucleoside diphosphate kinase 3 (EC 2.7.4.6) (NDK 3) (NDP kinase 3) (Nucleoside diphosphate kinase C) (NDPKC) (nm23-M3) (DR-nm23)	4	24.9	169	19099	6.7	Unknown	kinase	1	0.000199
Q9WV92	E41L3_MOUSE	EPB41L3	(Q9WV92) Band 4.1-like protein 3 (4.1B) (Differentially expressed in adenocarcinoma of the lung protein 1) (DAL-1) (DAL1P) (mDAL-1)	7	10.4	929	103338	5.3	Plasma Membrane	other	NONE	0.000121
Q9WV96	TIM9B_MOUSE	FXC1	(Q9WV96) Mitochondrial import inner membrane translocase subunit Tim9 B (TIMM10B) (Tim10b)	1	19	100	11314	7.1	Cytoplasm	transporter	NONE	0.000225
Q9WV98	TIM9_MOUSE	TIMM9	(Q9WV98) Mitochondrial import inner membrane translocase subunit Tim9	2	16.9	89	10344	7.2	Cytoplasm	transporter	NONE	0.000441
Q9WVA2	TIM8A_MOUSE	TIMM8A	(Q9WVA2) Mitochondrial import inner membrane translocase subunit Tim8 A (Deafness dystonia protein 1 homolog)	1	23.7	97	11042	5.2	Cytoplasm	transporter	NONE	0.000116
Q9WVA4	TAGL2_MOUSE	TAGLN2	(Q9WVA4) Transgelin-2	11	35.1	211	23466	7.1	Cytoplasm	other	NONE	0.003804
Q9WVB0	RBPMS_MOUSE	RBPMS	(Q9WVB0) RNA-binding protein with multiple splicing (RBP-MS) (HEart, RRM Expressed Sequence) (Hermes)	2	14.7	197	21751	8.1	Unknown	other	NONE	5.7E-05
Q9WVE8	PACN2_MOUSE	PACSIN2	(Q9WVE8) Protein kinase C and casein kinase substrate in neurons protein 2	7	17.3	486	55833	5.2	Cytoplasm	transporter	NONE	0.000335
Q9WVK4	EHD1_MOUSE	EHD1	(Q9WVK4) EH-domain-containing protein 1 (mPAST1)	15	31.3	534	60603	6.8	Cytoplasm	other	NONE	0.000326
Q9WVL0	MAAI_MOUSE	GSTZ1	(Q9WVL0) Maleylacetoacetate isomerase (EC 5.2.1.2) (MAAI) (Glutathione S-transferase zeta 1) (EC 2.5.1.18) (GSTZ1-1)	7	38.9	216	24275	7.9	Cytoplasm	enzyme	NONE	0.000572
Q9WVL3	S12A7_MOUSE	SLC12A7	(Q9WVL3) Solute carrier family 12 member 7 (Electroneutral potassium-chloride cotransporter 4) (K-Cl cotransporter 4)	3	5.4	1083	119481	6.5	Plasma Membrane	transporter	11	4.66E-05
Q9WVM8	AADAT_MOUSE	AADAT	(Q9WVM8) Kynurenine/alpha-aminoadipate aminotransferase mitochondrial precursor (KAT/AadAT) (Kynurenine--oxoglutarate transaminase II) (EC 2.6.1.7) (Kynurenine aminotransferase II) (Kynurenine--oxoglutarate aminotransferase II) (2-aminoadipat	8	25.9	425	47598	8.2	Cytoplasm	enzyme	NONE	0.000225
Q9WVT6	CAH14_MOUSE	CA14	(Q9WVT6) Carbonic anhydrase 14 precursor (EC 4.2.1.1) (Carbonic anhydrase XIV) (Carbonate dehydratase XIV) (CA-XIV)	2	12.2	337	37505	6.4	Plasma Membrane	enzyme	2	0.000167
Q9Z0F7	SYUG_MOUSE	SNCG	(Q9Z0F7) Gamma-synuclein (Persyn)	1	18.7	123	13160	4.7	Cytoplasm	other	NONE	0.000183
Q9Z0J0	NPC2_MOUSE	NPC2	(Q9Z0J0) Epididymal secretory protein E1 precursor (Niemann Pick type C2 protein homolog) (mE1)	2	26.2	149	16442	7.7	Extracellular Space	other	1	0.000151
Q9Z0K8	VNN1_MOUSE	VNN1	(Q9Z0K8) Pantetheinase precursor (EC 3.5.1.92) (Pantetheine hydrolase) (Vascular non-inflammatory molecule 1) (Vanin-1)	2	9.4	512	57061	5.9	Plasma Membrane	enzyme	2	0.000143
Q9Z0P4	PALM_MOUSE	PALM	(Q9Z0P4) Paralemmin	5	20.1	383	41614	4.8	Plasma Membrane	other	NONE	0.00022
Q9Z0R6	ITSN2_MOUSE	ITSN2	(Q9Z0R6) Intersectin-2 (SH3 domain-containing protein 1B) (EH and SH3 domains protein 2) (EH domain and SH3 domain regulator of endocytosis 2)	3	2.8	1658	188775	8.1	Cytoplasm	other	NONE	1.02E-05
Q9Z0S1	BPNT1_MOUSE	BPNT1	(Q9Z0S1) 3'(2'),5'-bisphosphate nucleotidase 1 (EC 3.1.3.7) (Bisphosphate 3'-nucleotidase 1) (PAP-inositol-1,4-phosphatase) (PIP)	8	26	308	33196	5.8	Nucleus	phosphatase	NONE	0.000656
Q9Z0U1	ZO2_MOUSE	TJP2	(Q9Z0U1) Tight junction protein ZO-2 (Zonula occludens 2 protein) (Zona occludens 2 protein) (Tight junction protein 2)	4	4.5	1167	131280	6.8	Plasma Membrane	kinase	NONE	2.89E-05
Q9Z0X1	PDCD8_MOUSE	AIFM1	(Q9Z0X1) Programmed cell death protein 8, mitochondrial precursor (EC 1.-.-.-) (Apoptosis-inducing factor)	22	32.5	612	66766	9.2	Cytoplasm	enzyme	NONE	0.000532
Q9Z108	STAU1_MOUSE	STAU1	(Q9Z108) Double-stranded RNA-binding protein Staufen homolog 1	2	6.2	487	53925	9.5	Cytoplasm	transporter	NONE	2.31E-05

Q9Z139	ROR1_MOUSE	ROR1 (includes EG:4919)	(Q9Z139) Tyrosine-protein kinase transmembrane receptor ROR1 precursor (EC 2.7.10.1) (Neurotrophic tyrosine kinase, receptor-related 1) (mROR1)	2	2.8	937	104157	7.3	Plasma Membrane	kinase	2	4.19E-05
Q9Z1D1	IF34_MOUSE	EIF3S4	(Q9Z1D1) Eukaryotic translation initiation factor 3 subunit 4 (eIF-3 delta) (eIF3 p44) (eIF-3 RNA-binding subunit) (eIF3 p42) (Eif3p42) (eIF3g)	3	19.4	320	35638	5.9	Cytoplasm	translation regulator	NONE	7.02E-05
Q9Z1G3	VATC_MOUSE	ATP6V1C1	(Q9Z1G3) Vacuolar ATP synthase subunit C (EC 3.6.3.14) (V-ATPase C subunit) (Vacuolar proton pump C subunit)	2	4.2	381	43729	6.8	Cytoplasm	transporter	NONE	7.37E-05
Q9Z1J2	NEK4_MOUSE	NEK4	(Q9Z1J2) Serine/threonine-protein kinase Nek4 (EC 2.7.11.1) (NimA-related protein kinase 4) (Serine/threonine-protein kinase 2)	1	2.7	792	88994	7.3	Nucleus	kinase	NONE	1.42E-05
Q9Z1J3	NFS1_MOUSE	NFS1	(Q9Z1J3) Cysteine desulfurase, mitochondrial precursor (EC 2.8.1.7) (m-Nfs1)	4	9.5	451	50001	8.5	Cytoplasm	enzyme	NONE	0.000124
Q9Z1K6	ARI2_MOUSE	ARIH2	(Q9Z1K6) Protein ariadne-2 homolog (ARI-2) (Triad1 protein) (UbcM4-interacting protein 48)	1	3.5	492	57697	5.7	Nucleus	other	NONE	1.14E-05
Q9Z1M8	RED_MOUSE	IK	(Q9Z1M8) Protein Red (Protein RER) (IK factor) (Cytokine IK)	1	1.6	557	65517	6.5	Extracellular Space	cytokine	NONE	1.01E-05
Q9Z1N5	UAP56_MOUSE	BAT1	(Q9Z1N5) Spliceosome RNA helicase Bat1 (EC 3.6.1.-) (DEAD box protein UAP56) (56 kDa U2AF65-associated protein) (HLA-B-associated transcript 1)	2	4.4	428	49035	5.7	Nucleus	enzyme	NONE	5.25E-05
Q9Z1P6	NDUA7_MOUSE	NDUFA7	(Q9Z1P6) NADH dehydrogenase [ubiquinone] 1 alpha subcomplex subunit 7 (EC 1.6.5.3) (EC 1.6.99.3) (NADH-ubiquinone oxidoreductase subunit B14.5a) (Complex I-B14.5a) (CI-B14.5a)	5	48.2	112	12444	10.2	Cytoplasm	enzyme	NONE	0.000902
Q9Z1Q5	CLIC1_MOUSE	CLIC1	(Q9Z1Q5) Chloride intracellular channel protein 1 (Nuclear chloride ion channel 27) (NCC27)	6	28.8	240	26882	5.2	Nucleus	ion channel	NONE	0.000772
Q9Z1R2	BAT3_MOUSE	BAT3	(Q9Z1R2) Large proline-rich protein BAT3 (HLA-B-associated transcript 3)	1	2.7	1154	121037	5.7	Nucleus	enzyme	NONE	4.86E-06
Q9Z1T1	AP3B1_MOUSE	AP3B1	(Q9Z1T1) AP-3 complex subunit beta-1 (Adapter-related protein complex 3 beta-1 subunit) (Beta3A-adaptin) (Adaptor protein complex AP-3 beta-1 subunit) (Clathrin assembly protein complex 3 beta-1 large chain)	4	7.5	1105	122870	5.7	Cytoplasm	transporter	NONE	3.05E-05
Q9Z1X4	ILF3_MOUSE	ILF3	(Q9Z1X4) Interleukin enhancer-binding factor 3	5	13.5	898	96021	8.8	Nucleus	transcription regulator	NONE	5E-05
Q9Z1Z2	STRAP_MOUSE	STRAP	(Q9Z1Z2) Serine-threonine kinase receptor-associated protein (UNR-interacting protein)	2	8.3	351	38513	5.1	Plasma Membrane	other	NONE	6.4E-05
Q9Z204	HNRPC_MOUSE	HNRPC	(Q9Z204) Heterogeneous nuclear ribonucleoproteins C1/C2 (hnRNP C1 / hnRNP C2)	5	20.4	313	34385	5	Nucleus	other	NONE	0.000466
Q9Z277	BAZ1B_MOUSE	BAZ1B (includes EG:9031)	(Q9Z277) Bromodomain adjacent to zinc finger domain protein 1B (Williams-Beuren syndrome chromosome region 9 protein homolog) (WBRS9)	2	2.4	1479	170788	8.6	Nucleus	transcription regulator	NONE	1.52E-05
Q9Z2A0	PDPK1_MOUSE	PDPK1	(Q9Z2A0) 3-phosphoinositide-dependent protein kinase 1 (EC 2.7.11.1) (mPDK1)	1	3	559	63759	7.4	Cytoplasm	kinase	NONE	1E-05
Q9Z2A9	GGT5_MOUSE	GGTLA1	(Q9Z2A9) Gamma-glutamyltransferase 5 precursor (EC 2.3.2.2) (Gamma-glutamyltransferase-like activity 1) (Gamma-glutamyl leukotrienase) (GGL) [Contains: Gamma-glutamyltransferase 5 heavy chain; Gamma-glutamyltransferase 5 light chain]	1	3.3	573	61658	8.5	Plasma Membrane	enzyme	1	1.96E-05
Q9Z2C5	MTM1_MOUSE	MTM1	(Q9Z2C5) Myotubularin (EC 3.1.3.48)	1	2.7	603	69594	7.6	Cytoplasm	phosphatase	NONE	1.86E-05
Q9Z2D6	MECP2_MOUSE	MECP2	(Q9Z2D6) Methyl-CpG-binding protein 2 (MeCP-2 protein) (MeCP2)	11	28.7	484	52308	10	Nucleus	transcription regulator	NONE	0.000348
Q9Z2D8	MBD3_MOUSE	MBD3 (includes EG:53615)	(Q9Z2D8) Methyl-CpG-binding domain protein 3 (Methyl-CpG-binding protein MBD3)	2	10.5	285	32168	5.8	Nucleus	other	NONE	3.94E-05
Q9Z2G6	SEL1L_MOUSE	SEL1L	(Q9Z2G6) Sel-1 homolog precursor (Suppressor of lin-12-like protein) (Sel-1L)	1	2.7	790	88340	5.6	Cytoplasm	other	3	1.42E-05
Q9Z2H5	E41L1_MOUSE	EPB41L1	(Q9Z2H5) Band 4.1-like protein 1 (Neuronal protein 4.1) (4.1N)	6	6.6	879	98315	5.7	Plasma Membrane	other	NONE	0.000102
Q9Z2I0	LETM1_MOUSE	LETM1	(Q9Z2I0) Leucine zipper-EF-hand-containing transmembrane protein 1, mitochondrial precursor	21	26	738	82989	6.5	Cytoplasm	other	1	0.00038
Q9Z2I8	SUCB2_MOUSE	SUCLG2	(Q9Z2I8) Succinyl-CoA ligase [GDP-forming] beta-chain, mitochondrial precursor (EC 6.2.1.4) (Succinyl-CoA synthetase, betaG chain) (SCS-betaG) (GTP-specific succinyl-CoA synthetase subunit beta)	24	51	433	46840	7	Cytoplasm	enzyme	1	0.001478

Q9Z2I9	SUCB1_MOUSE	SUCLA2	(Q9Z2I9) Succinyl-CoA ligase [ADP-forming] beta-chain, mitochondrial precursor (EC 6.2.1.5) (Succinyl-CoA synthetase, betaA chain) (SCS-betaA) (ATP-specific succinyl-CoA synthetase subunit beta)	9	27.4	463	50114	7	Cytoplasm	enzyme	NONE	0.000412
Q9Z2J0	S23A1_MOUSE	SLC23A1	(Q9Z2J0) Solute carrier family 23 member 1 (Sodium-dependent vitamin C transporter 1) (Na(+)/L-ascorbic acid transporter 1) (Yolk sac permease-like molecule 3)	1	3.6	605	65554	7.1	Plasma Membrane	transporter	12	3.71E-05
Q9Z2M7	PMM2_MOUSE	PMM2	(Q9Z2M7) Phosphomannomutase 2 (EC 5.4.2.8) (PMM 2)	3	21.1	242	27657	6.4	Cytoplasm	enzyme	NONE	0.000116
Q9Z2Q5	RM40_MOUSE	MRPL40	(Q9Z2Q5) 39S ribosomal protein L40, mitochondrial precursor (L40mt) (MRP-40) (Nuclear localization signal-containing protein deleted in velocardiofacial syndrome homolog)	3	16.5	206	24301	9.5	Cytoplasm	other	NONE	0.000163
Q9Z2U0	PSA7_MOUSE	PSMA7	(Q9Z2U0) Proteasome subunit alpha type 7 (EC 3.4.25.1) (Proteasome subunit RC6-1)	5	20.6	248	27855	8.5	Cytoplasm	peptidase	NONE	0.000317
Q9Z2U1	PSA5_MOUSE	PSMA5	(Q9Z2U1) Proteasome subunit alpha type 5 (EC 3.4.25.1) (Proteasome zeta chain) (Macropain zeta chain) (Multicatalytic endopeptidase complex zeta chain)	4	25.7	241	26411	4.8	Cytoplasm	peptidase	NONE	0.000186
Q9Z2V4	PPCKC_MOUSE	PCK1	(Q9Z2V4) Phosphoenolpyruvate carboxykinase, cytosolic [GTP] (EC 4.1.1.32) (Phosphoenolpyruvate carboxylase) (PEPCK-C)	7	16.4	622	69355	6.6	Cytoplasm	kinase	NONE	0.000135
Q9Z2Y8	PROSC_MOUSE	PROSC	(Q9Z2Y8) Proline synthetase co-transcribed bacterial homolog protein	11	40.9	274	30049	8.3	Unknown	enzyme	NONE	0.000881
