# Supplementary material for: Comprehensive analysis of the mouse renal cortex using two-dimensional HPLC – tandem mass spectrometry
Source: Proteome Sci. 2008 May 23;6:15. doi: 10.1186/1477-5956-6-15 (PMC2412861; doi:10.1186/1477-5956-6-15)
Supplement: Additional file 4 — Renal proteins with anti-oxidant activity. List of all renal identified proteins with anti-oxidant activity. [file 1477-5956-6-15-S4.pdf]

Supplemental Table S4: Renal proteins with anti-oxidant activity

Protein name	Gene name	Uniprot accession	EC #
aminoadipate-semialdehyde synthase	Aass	Q99K67	1.5.1.7
apolipoprotein e	Apoe	P08226	---
catalase	Cat	P24270	1.11.1.6
cathepsin b	Ctsb	P10605	3.4.22.1
glutathione peroxidase 1	Gpx1	P11352	1.11.1.9
glutathione peroxidase 3	Gpx3	P46412	1.11.1.9
glutathione reductase 1	Gsr	P47791	1.8.1.7
glutathione S-transferase kappa 1	Gstk1	Q9DCM2	2.5.1.18
glutathione transferase zeta 1	Gstz1	Q9WVL0	2.5.1.18
peroxiredoxin 1	Prdx1	P35700	1.11.1.15
peroxiredoxin 2	Prdx2	Q61171	1.11.1.15
Peroxiredoxin 3	Prdx3	P20108	1.11.1.15
peroxiredoxin-6	Prdx6	O08709	1.11.1.15
peroxisomal membrane protein 20	Prdx5	P99029	1.11.1.15
phospholipid hydroperoxide glutathione peroxidase	Gpx-4	O70325	1.11.1.12
superoxide dismutase 1, soluble	Sod1	P08228	1.15.1.1
superoxide dismutase 3, extracellular	Sod3	O09164	1.15.1.1
thioredoxin reductase 1	Txnrd1	Q9JMH6	1.8.1.9
