# Supplementary material for: Comprehensive analysis of the mouse renal cortex using two-dimensional HPLC – tandem mass spectrometry
Source: Proteome Sci. 2008 May 23;6:15. doi: 10.1186/1477-5956-6-15 (PMC2412861; doi:10.1186/1477-5956-6-15)
Supplement: Additional file 5 — Renal proteins identified in published urine proteomes. List of all proteins identified in the mouse renal cortex that have been previously identified in published human urine proteomes. [file 1477-5956-6-15-S5.pdf]

Accession Number	Protein Name	Number of Identified Peptides	Subcellular Location	Protein Family	Number of Amino Acid	NSAF
O08709	(O08709) Peroxiredoxin-6 (EC 1.11.1.15) (Antioxidant protein 2) (1-Cys peroxiredoxin) (1-Cys PRX) (Acidic calcium-independent phospholipase A2) (EC 3.1.1.-) (aiPLA2) (Non-selenium glutathione peroxidase) (EC 1.11.1.7) (NSGPx)	14	Cytoplasm	enzyme	223	0.001459916
O08795	(O08795) Glucosidase 2 subunit beta precursor (Glucosidase II subunit beta) (Protein kinase C substrate, 60.1 kDa protein, heavy chain) (PKCSH) (80K-H protein)	14	Cytoplasm	enzyme	521	0.000333986
O09131	(O09131) Glutathione transferase omega-1 (EC 2.5.1.18) (GSTO 1-1) (p28)	3	Cytoplasm	enzyme	240	0.000304044
O09159	(O09159) Lysosomal alpha-mannosidase precursor (EC 3.2.1.24) (Mannosidase, alpha B) (Lysosomal acid alpha-mannosidase) (Laman) (Mannosidase alpha class 2B member 1)	2	Cytoplasm	enzyme	1013	4.43287E-05
O35215	(O35215) D-dopachrome decarboxylase (EC 4.1.1.84) (D-dopachrome tautomerase)	10	Cytoplasm	enzyme	117	0.002878527
O35639	(O35639) Annexin A3 (Annexin III) (Lipocortin III) (Placental anticoagulant protein III) (PAP-III) (35-alpha calcimedin)	4	Cytoplasm	enzyme	322	8.71604E-05
O54983	(O54983) Mu-crystallin homolog	8	Cytoplasm	enzyme	313	0.000484199
O70325	(O70325) Phospholipid hydroperoxide glutathione peroxidase, mitochondrial precursor (EC 1.11.1.12) (PHGPx) (GPX-4)	4	Cytoplasm	enzyme	197	0.000199451
O88531	(O88531) Palmitoyl-protein thioesterase 1 precursor (EC 3.1.2.22) (Palmitoyl-protein hydrolase 1)	4	Cytoplasm	enzyme	306	0.000128405
O88587	(O88587) Catechol O-methyltransferase (EC 2.1.1.6)	1	Cytoplasm	enzyme	265	4.23632E-05
O88844	(O88844) Isocitrate dehydrogenase [NADP] cytoplasmic (EC 1.1.1.42) (Cytosolic NADP-isocitrate dehydrogenase) (Oxalosuccinate decarboxylase) (IDH) (NADP(+)-specific ICDH) (IDP)	53	Cytoplasm	enzyme	414	0.007524844
P00920	(P00920) Carbonic anhydrase 2 (EC 4.2.1.1) (Carbonic anhydrase II) (Carbonate dehydratase II) (CA-II)	8	Cytoplasm	enzyme	259	0.003207501

Accession Number	Protein Name	Number of Identified Peptides	Subcellular Location	Protein Family	Number of Amino Acid	NSAF
P05064	(P05064) Fructose-bisphosphate aldolase A (EC 4.1.2.13) (Muscle-type aldolase) (Aldolase 1)	12	Cytoplasm	enzyme	363	0.001994748
P05201	(P05201) Aspartate aminotransferase, cytoplasmic (EC 2.6.1.1) (Transaminase A) (Glutamate oxaloacetate transaminase 1)	2	Cytoplasm	enzyme	412	5.44964E-05
P05202	(P05202) Aspartate aminotransferase, mitochondrial precursor (EC 2.6.1.1) (Transaminase A) (Glutamate oxaloacetate transaminase 2)	14	Cytoplasm	enzyme	430	0.000522151
P06151	(P06151) L-lactate dehydrogenase A chain (EC 1.1.1.27) (LDH-A) (LDH muscle subunit) (LDH-M)	9	Cytoplasm	enzyme	331	0.000203497
P08228	(P08228) Superoxide dismutase [Cu-Zn] (EC 1.15.1.1)	5	Cytoplasm	enzyme	153	0.005796563
P08249	(P08249) Malate dehydrogenase, mitochondrial precursor (EC 1.1.1.37)	24	Cytoplasm	enzyme	338	0.005745982
P09103	(P09103) Protein disulfide-isomerase precursor (EC 5.3.4.1) (PDI) (Prolyl 4-hydroxylase subunit beta) (Cellular thyroid hormone-binding protein) (p55) (Erp59)	33	Cytoplasm	enzyme	509	0.00235994
P09528	(P09528) Ferritin heavy chain (EC 1.16.3.1) (Ferritin H subunit)	4	Cytoplasm	enzyme	181	0.000279106
P09671	(P09671) Superoxide dismutase [Mn], mitochondrial precursor (EC 1.15.1.1)	10	Cytoplasm	enzyme	222	0.001238933
P10518	(P10518) Delta-aminolevulinic acid dehydratase (EC 4.2.1.24) (Porphobilinogen synthase) (ALADH)	5	Cytoplasm	enzyme	330	0.000204114
P10639	(P10639) Thioredoxin (ATL-derived factor) (ADF)	1	Cytoplasm	enzyme	104	0.000485751
P12265	(P12265) Beta-glucuronidase precursor (EC 3.2.1.31)	2	Cytoplasm	enzyme	648	2.59867E-05
P13707	(P13707) Glycerol-3-phosphate dehydrogenase [NAD+], cytoplasmic (EC 1.1.1.8) (GPD-C) (GPDH-C)	3	Cytoplasm	enzyme	348	0.000161297
P14152	(P14152) Malate dehydrogenase, cytoplasmic (EC 1.1.1.37) (Cytosolic malate dehydrogenase)	10	Cytoplasm	enzyme	333	0.004197203
P16125	(P16125) L-lactate dehydrogenase B chain (EC 1.1.1.27) (LDH-B) (LDH heart subunit) (LDH-H)	23	Cytoplasm	enzyme	333	0.001078799

Accession Number	Protein Name	Number of Identified Peptides	Subcellular Location	Protein Family	Number of Amino Acid	NSAF
P17742	(P17742) Peptidyl-prolyl cis-trans isomerase A (EC 5.2.1.8) (PPIase A) (Rotamase A) (Cyclophilin A) (Cyclosporin A-binding protein) (SP18)	21	Cytoplasm	enzyme	163	0.012328218
P17751	(P17751) Triosephosphate isomerase (EC 5.3.1.1) (TIM) (Triose-phosphate isomerase)	12	Cytoplasm	enzyme	248	0.003304502
P19157	(P19157) Glutathione S-transferase P 1 (EC 2.5.1.18) (GST YF-YF) (GST-piB) (GST class-pi) (Gst P1) (Preadipocyte growth factor)	11	Cytoplasm	enzyme	209	0.001584567
P20060	(P20060) Beta-hexosaminidase beta chain precursor (EC 3.2.1.52) (N-acetyl-beta-glucosaminidase) (Beta-N-acetylhexosaminidase) (Hexosaminidase B)	2	Cytoplasm	enzyme	536	3.14168E-05
P20108	(P20108) Thioredoxin-dependent peroxide reductase, mitochondrial precursor (EC 1.11.1.15) (Perioredoxin-3) (PRX III) (Antioxidant protein 1) (AOP-1) (Protein MER5)	6	Cytoplasm	enzyme	257	0.001070207
P23780	(P23780) Beta-galactosidase precursor (EC 3.2.1.23) (Lactase) (Acid beta-galactosidase)	1	Cytoplasm	enzyme	647	1.73512E-05
P24270	(P24270) Catalase (EC 1.11.1.6)	35	Cytoplasm	enzyme	526	0.001632716
P24369	(P24369) Peptidyl-prolyl cis-trans isomerase B precursor (EC 5.2.1.8) (PPIase) (Rotamase) (Cyclophilin B) (S-cyclophilin) (SCYLP) (CYP-S1)	19	Cytoplasm	enzyme	208	0.001943006
P24549	(P24549) Retinal dehydrogenase 1 (EC 1.2.1.36) (RalDH1) (RALDH 1) (Aldehyde dehydrogenase family 1 member A1) (Aldehyde dehydrogenase, cytosolic) (ALHDII) (ALDH-E1)	2	Cytoplasm	enzyme	500	7.85838E-05
P26883	(P26883) FK506-binding protein 1A (EC 5.2.1.8) (Peptidyl-prolyl cis-trans isomerase) (PPIase) (Rotamase) (12 kDa FKBP) (FKBP-12) (Immunophilin FKBP12)	3	Cytoplasm	enzyme	107	0.00062951
P28271	(P28271) Iron-responsive element-binding protein 1 (IRE-BP 1) (Iron regulatory protein 1) (IRP1) (Ferritin repressor protein) (Aconitate hydratase) (EC 4.2.1.3) (Citrate hydro-lyase) (Aconitase)	18	Cytoplasm	enzyme	889	0.000227303

Accession Number	Protein Name	Number of Identified Peptides	Subcellular Location	Protein Family	Number of Amino Acid	NSAF
P28474	(P28474) Alcohol dehydrogenase class 3 (EC 1.1.1.1) (Alcohol dehydrogenase class III) (Alcohol dehydrogenase 2) (S-(hydroxymethyl)glutathione dehydrogenase) (EC 1.1.1.284) (Glutathione-dependent formaldehyde dehydrogenase) (FDH) (FALDH) (Alcoh	8	Cytoplasm	enzyme	373	0.000165535
P30115	(P30115) Glutathione S-transferase Yc (EC 2.5.1.18) (GST class-alpha) (Ya3)	1	Cytoplasm	enzyme	220	5.10284E-05
P30412	(P30412) Peptidyl-prolyl cis-trans isomerase C (EC 5.2.1.8) (PPIase) (Rotamase) (Cyclophilin C)	1	Cytoplasm	enzyme	212	2.6477E-05
P31253	(P31253) Ubiquitin-activating enzyme E1 X (Fragment)	3	Cytoplasm	enzyme	450	7.48417E-05
P34914	(P34914) Epoxide hydrolase 2 (EC 3.3.2.3) (Soluble epoxide hydrolase) (SEH) (Epoxide hydratase) (Cytosolic epoxide hydrolase) (CEH)	9	Cytoplasm	enzyme	554	0.000344488
P35278	(P35278) Ras-related protein Rab-5C	2	Cytoplasm	enzyme	216	0.000129933
P35282	(P35282) Ras-related protein Rab-21 (Rab-12)	3	Cytoplasm	enzyme	221	0.000152393
P35505	(P35505) Fumarylacetoacetase (EC 3.7.1.2) (Fumarylacetoacetate hydrolase) (Beta-diketonase) (FAA)	9	Cytoplasm	enzyme	419	0.001125305
P35700	(P35700) Peroxiredoxin-1 (EC 1.11.1.15) (Thioredoxin peroxidase 2) (Thioredoxin-dependent peroxide reductase 2) (Osteoblast-specific factor 3) (OSF-3) (Macrophage 23 kDa stress protein)	22	Cytoplasm	enzyme	199	0.009421027
P40142	(P40142) Transketolase (EC 2.2.1.1) (TK) (P68)	17	Cytoplasm	enzyme	623	0.000432472
P45376	(P45376) Aldose reductase (EC 1.1.1.21) (AR) (Aldehyde reductase)	6	Cytoplasm	enzyme	315	0.000605861
P45952	(P45952) Medium-chain specific acyl-CoA dehydrogenase, mitochondrial precursor (EC 1.3.99.3) (MCAD)	27	Cytoplasm	enzyme	421	0.002053258
P46638	(P46638) Ras-related protein Rab-11B	2	Cytoplasm	enzyme	217	0.000155202
P47199	(P47199) Quinone oxidoreductase (EC 1.6.5.5) (NADPH:quinone reductase) (Zeta-crystallin)	17	Cytoplasm	enzyme	331	0.002272384

Accession Number	Protein Name	Number of Identified Peptides	Subcellular Location	Protein Family	Number of Amino Acid	NSAF
P47791	(P47791) Glutathione reductase, mitochondrial precursor (EC 1.8.1.7) (GR) (GRase)	2	Cytoplasm	enzyme	500	5.61313E-05
P48758	(P48758) Carbonyl reductase [NADPH] 1 (EC 1.1.1.184) (NADPH-dependent carbonyl reductase 1)	9	Cytoplasm	enzyme	276	0.000528773
P50247	(P50247) Adenosylhomocysteinase (EC 3.3.1.1) (S-adenosyl-L-homocysteine hydrolase) (AdoHcyase) (Liver copper-binding protein) (CUBP)	10	Cytoplasm	enzyme	431	0.000351634
P50431	(P50431) Serine hydroxymethyltransferase, cytosolic (EC 2.1.2.1) (Serine methylase) (Glycine hydroxymethyltransferase) (SHMT)	3	Cytoplasm	enzyme	478	4.69718E-05
P51150	(P51150) Ras-related protein Rab-7	9	Cytoplasm	enzyme	207	0.001708343
P51855	(P51855) Glutathione synthetase (EC 6.3.2.3) (Glutathione synthase) (GSH synthetase) (GSH-S)	2	Cytoplasm	enzyme	474	5.92102E-05
P53994	(P53994) Ras-related protein Rab-2A	5	Cytoplasm	enzyme	212	0.000238293
P54071	(P54071) Isocitrate dehydrogenase [NADP], mitochondrial precursor (EC 1.1.1.42) (Oxalosuccinate decarboxylase) (IDH) (NADP(+)-specific ICDH) (IDP) (ICD-M)	27	Cytoplasm	enzyme	523	0.001953325
P54818	(P54818) Galactocerebrosidase precursor (EC 3.2.1.46) (GALCERase) (Galactosylceramidase) (Galactosylceramide beta-galactosidase) (Galactocerebroside beta-galactosidase)	1	Cytoplasm	enzyme	668	2.52087E-05
P55258	(P55258) Ras-related protein Rab-8A (Oncogene c-mel)	3	Cytoplasm	enzyme	207	0.000135583
P61021	(P61021) Ras-related protein Rab-5B	1	Cytoplasm	enzyme	215	7.83227E-05
P61027	(P61027) Ras-related protein Rab-10	1	Cytoplasm	enzyme	200	0.000140328
P61089	(P61089) Ubiquitin-conjugating enzyme E2 N (EC 6.3.2.19) (Ubiquitin-protein ligase N) (Ubiquitin carrier protein N) (Ubc13) (Bendless-like ubiquitin-conjugating enzyme)	7	Cytoplasm	enzyme	152	0.000664712
P62492	(P62492) Ras-related protein Rab-11A (Rab-11)	4	Cytoplasm	enzyme	215	0.000391614
P62821	(P62821) Ras-related protein Rab-1A (YPT1-related protein)	7	Cytoplasm	enzyme	204	0.001375766
P62897	(P62897) Cytochrome c, somatic	11	Cytoplasm	enzyme	104	0.003562177

Accession Number	Protein Name	Number of Identified Peptides	Subcellular Location	Protein Family	Number of Amino Acid	NSAF
P63001	(P63001) Ras-related C3 botulinum toxin substrate 1 precursor (p21-Rac1)	6	Cytoplasm	enzyme	192	0.000380055
P63017	(P63017) Heat shock cognate 71 kDa protein (Heat shock 70 kDa protein 8)	33	Cytoplasm	enzyme	646	0.001233845
P63101	(P63101) 14-3-3 protein zeta/delta (Protein kinase C inhibitor protein 1) (KCIP-1) (SEZ-2)	16	Cytoplasm	enzyme	245	0.001809947
P68037	(P68037) Ubiquitin-conjugating enzyme E2 L3 (EC 6.3.2.19) (Ubiquitin-protein ligase L3) (Ubiquitin carrier protein L3) (UbcM4)	4	Cytoplasm	enzyme	154	0.00065608
P70699	(P70699) Lysosomal alpha-glucosidase precursor (EC 3.2.1.20) (Acid maltase)	1	Cytoplasm	enzyme	953	2.35598E-05
P84096	(P84096) Rho-related GTP-binding protein RhoG precursor (Sid 10750)	1	Cytoplasm	enzyme	191	8.81643E-05
P99029	(P99029) Peroxiredoxin-5, mitochondrial precursor (EC 1.11.1.15) (Prx-V) (Peroxisomal antioxidant enzyme) (PLP) (Thioredoxin reductase) (Thioredoxin peroxidase PMP20) (Antioxidant enzyme B166) (AOEB166) (Liver tissue 2D-page spot 2D-0014IV)	20	Cytoplasm	enzyme	210	0.009034462
Q00612	(Q00612) Glucose-6-phosphate 1-dehydrogenase X (EC 1.1.1.49) (G6PD)	2	Cytoplasm	enzyme	514	2.1841E-05
Q01853	(Q01853) Transitional endoplasmic reticulum ATPase (TER ATPase) (15S Mg(2+)-ATPase p97 subunit) (Valosin-containing protein) (VCP)	9	Cytoplasm	enzyme	805	0.000118538
Q02053	(Q02053) Ubiquitin-activating enzyme E1 1	5	Cytoplasm	enzyme	1058	3.71379E-05
Q60648	(Q60648) Ganglioside GM2 activator precursor (GM2-AP) (Cerebroside sulfate activator protein) (Shingolipid activator protein 3) (SAP-3)	4	Cytoplasm	enzyme	193	0.002297601
Q60928	(Q60928) Gamma-glutamyltranspeptidase 1 precursor (EC 2.3.2.2) (Gamma-glutamyltransferase 1) (GGT 1) (CD224 antigen) [Contains: Gamma-glutamyltranspeptidase 1 heavy chain; Gamma-glutamyltranspeptidase 1 light chain]	16	Cytoplasm	enzyme	568	0.002322333

Accession Number	Protein Name	Number of Identified Peptides	Subcellular Location	Protein Family	Number of Amino Acid	NSAF
Q61171	(Q61171) Peroxiredoxin-2 (EC 1.11.1.15) (Thioredoxin peroxidase 1) (Thioredoxin-dependent peroxide reductase 1) (Thiol-specific antioxidant protein) (TSA)	7	Cytoplasm	enzyme	197	0.001025749
Q64105	(Q64105) Sepiapterin reductase (EC 1.1.1.153) (SPR)	9	Cytoplasm	enzyme	261	0.000580668
Q64191	(Q64191) N(4)-(beta-N-acetylglucosaminyl)-L-asparaginase precursor (EC 3.5.1.26) (Glycosylasparaginase) (Aspartylglucosaminidase) (N4-(N-acetyl-beta-glucosaminyl)-L-asparagine amidase) (AGA) [Contains: Glycosylasparaginase alpha chain; Glycosy	6	Cytoplasm	enzyme	346	0.000194675
Q64433	(Q64433) 10 kDa heat shock protein, mitochondrial (Hsp10) (10 kDa chaperonin) (CPN10)	17	Cytoplasm	enzyme	101	0.017283986
Q78JT3	(Q78JT3) 3-hydroxyanthranilate 3,4-dioxygenase (EC 1.13.11.6) (3-HAO) (3-hydroxyanthranilic acid dioxygenase) (3-hydroxyanthranilate oxygenase)	7	Cytoplasm	enzyme	286	0.000353274
Q8BGN3	(Q8BGN3) Ectonucleotide pyrophosphatase/phosphodiesterase 6 precursor (EC 3.1.-.-) (E-NPP6) (NPP-6) [Contains: Ectonucleotide pyrophosphatase/phosphodiesterase 6 soluble form]	1	Cytoplasm	enzyme	440	1.27571E-05
Q8CG76	(Q8CG76) Aflatoxin B1 aldehyde reductase member 2 (EC 1.-.-.-)	9	Cytoplasm	enzyme	367	0.00102474
Q8K157	(Q8K157) Aldose 1-epimerase (EC 5.1.3.3) (Galactose mutarotase)	4	Cytoplasm	enzyme	342	9.84759E-05
Q8R0Y6	(Q8R0Y6) 10-formyltetrahydrofolate dehydrogenase (EC 1.5.1.6) (10-FTHFDH) (Aldehyde dehydrogenase 1 family member L1)	17	Cytoplasm	enzyme	902	0.000192912
Q8R180	(Q8R180) ERO1-like protein alpha precursor (EC 1.8.4.-) (ERO1-Lalpha) (Oxidoreductin-1-Lalpha) (Endoplasmic oxidoreductin-1-like protein) (ERO1-L)	1	Cytoplasm	enzyme	464	2.41945E-05

Accession Number	Protein Name	Number of Identified Peptides	Subcellular Location	Protein Family	Number of Amino Acid	NSAF
Q8VDD5	(Q8VDD5) Myosin-9 (Myosin heavy chain, nonmuscle IIa) (Nonmuscle myosin heavy chain IIa) (NMMHC II-a) (NMMHC-IIA) (Cellular myosin heavy chain, type A) (Nonmuscle myosin heavy chain-A) (NMMHC-A)	37	Cytoplasm	enzyme	1959	0.000191975
Q91V41	(Q91V41) Ras-related protein Rab-14	7	Cytoplasm	enzyme	214	0.000445903
Q91V92	(Q91V92) ATP-citrate synthase (EC 2.3.3.8) (ATP-citrate (pro-S-)-lyase) (Citrate cleavage enzyme)	2	Cytoplasm	enzyme	1091	1.54348E-05
Q91X52	(Q91X52) L-xylulose reductase (EC 1.1.1.10) (XR) (Dicarbonyl/L-xylulose reductase)	4	Cytoplasm	enzyme	244	0.000207042
Q91Y97	(Q91Y97) Fructose-bisphosphate aldolase B (EC 4.1.2.13) (Liver-type aldolase) (Aldolase 2)	25	Cytoplasm	enzyme	363	0.010298465
Q91YR9	(Q91YR9) NADP-dependent leukotriene B4 12-hydroxydehydrogenase (EC 1.3.1.74) (15-oxoprostaglandin 13-reductase) (EC 1.3.1.48)	2	Cytoplasm	enzyme	329	5.11835E-05
Q91Z53	(Q91Z53) Glyoxylate reductase/hydroxypyruvate reductase (EC 1.1.1.79)	4	Cytoplasm	enzyme	328	6.84528E-05
Q922R8	(Q922R8) Protein disulfide-isomerase A6 precursor (EC 5.3.4.1) (Thioredoxin domain-containing protein 7)	8	Cytoplasm	enzyme	440	0.000523041
Q923D2	(Q923D2) Flavin reductase (EC 1.5.1.30) (FR) (NADPH-dependent diaphorase) (NADPH-flavin reductase) (FLR) (Biliverdin reductase B) (EC 1.3.1.24) (BVR-B) (Biliverdin-IX beta-reductase)	3	Cytoplasm	enzyme	205	8.21433E-05
Q93092	(Q93092) Transaldolase (EC 2.2.1.2)	11	Cytoplasm	enzyme	337	0.000466373
Q99J99	(Q99J99) 3-mercaptopyruvate sulfurtransferase (EC 2.8.1.2) (MST)	11	Cytoplasm	enzyme	296	0.000568898
Q99KI0	(Q99KI0) Aconitate hydratase, mitochondrial precursor (EC 4.2.1.3) (Citrate hydro-lyase) (Aconitase)	37	Cytoplasm	enzyme	780	0.002007772
Q99LJ1	(Q99LJ1) Tissue alpha-L-fucosidase precursor (EC 3.2.1.51) (Alpha-L-fucosidase I) (Alpha-L-fucoside fucohydrolase)	2	Cytoplasm	enzyme	452	3.72553E-05

Accession Number	Protein Name	Number of Identified Peptides	Subcellular Location	Protein Family	Number of Amino Acid	NSAF
Q9CPU0	(Q9CPU0) Lactoylglutathione lyase (EC 4.4.1.5) (Methylglyoxalase) (Aldoketomutase) (Glyoxalase I) (Glx I) (Ketone-aldehyde mutase) (S-D-lactoylglutathione methylglyoxal lyase)	3	Cytoplasm	enzyme	183	0.000398747
Q9CQ60	(Q9CQ60) 6-phosphogluconolactonase (EC 3.1.1.31) (6PGL)	6	Cytoplasm	enzyme	257	0.000327614
Q9CQ62	(Q9CQ62) 2,4-dienoyl-CoA reductase, mitochondrial precursor (EC 1.3.1.34) (2,4-dienoyl-CoA reductase [NADPH]) (4-enoyl-CoA reductase [NADPH])	11	Cytoplasm	enzyme	335	0.000519424
Q9CQD1	(Q9CQD1) Ras-related protein Rab-5A	3	Cytoplasm	enzyme	215	0.000182753
Q9CQF9	(Q9CQF9) Prenylcysteine oxidase precursor (EC 1.8.3.5)	3	Cytoplasm	enzyme	505	7.78057E-05
Q9CQM5	(Q9CQM5) Thioredoxin-like protein 5 (14 kDa thioredoxin-related protein) (TRP14) (Protein 42-9-9)	5	Cytoplasm	enzyme	123	0.000638893
Q9CWS0	(Q9CWS0) NG,NG-dimethylarginine dimethylaminohydrolase 1 (EC 3.5.3.18) (Dimethylargininase-1) (Dimethylarginine dimethylaminohydrolase 1) (DDAHI) (DDAH-1)	16	Cytoplasm	enzyme	284	0.00126493
Q9CZU6	(Q9CZU6) Citrate synthase, mitochondrial precursor (EC 2.3.3.1)	4	Cytoplasm	enzyme	464	0.000169362
Q9D1G1	(Q9D1G1) Ras-related protein Rab-1B	5	Cytoplasm	enzyme	201	0.000642298
Q9D1Q6	(Q9D1Q6) Thioredoxin domain-containing protein 4 precursor (Endoplasmic reticulum resident protein ERp44)	6	Cytoplasm	enzyme	406	0.00015208
Q9D2G2	(Q9D2G2) Dihydrolipoyllysine-residue succinyltransferase component of 2-oxoglutarate dehydrogenase complex, mitochondrial precursor (EC 2.3.1.61) (Dihydrolipoamide succinyltransferase component of 2-oxoglutarate dehydrogenase complex) (E2) (E2	8	Cytoplasm	enzyme	454	0.00043273
Q9D6Y7	(Q9D6Y7) Peptide methionine sulfoxide reductase (EC 1.8.4.6) (Protein-methionine-S-oxide reductase) (PMSR) (Peptide Met(O) reductase)	11	Cytoplasm	enzyme	233	0.000963627

Accession Number	Protein Name	Number of Identified Peptides	Subcellular Location	Protein Family	Number of Amino Acid	NSAF
Q9D819	(Q9D819) Inorganic pyrophosphatase (EC 3.6.1.1) (Pyrophosphate phospho-hydrolase) (PPase)	7	Cytoplasm	enzyme	289	0.000291339
Q9D964	(Q9D964) Glycine amidinotransferase, mitochondrial precursor (EC 2.1.4.1) (L-arginine:glycine amidinotransferase) (Transamidinase) (AT)	14	Cytoplasm	enzyme	423	0.000398094
Q9DC51	(Q9DC51) Guanine nucleotide-binding protein G(k) subunit alpha (G(i) alpha-3)	1	Cytoplasm	enzyme	353	3.18024E-05
Q9JIW9	(Q9JIW9) Ras-related protein Ral-B	3	Cytoplasm	enzyme	206	0.000190737
Q9JLJ2	(Q9JLJ2) 4-trimethylaminobutyraldehyde dehydrogenase (EC 1.2.1.47) (TMABADH) (Aldehyde dehydrogenase 9A1) (EC 1.2.1.3)	5	Cytoplasm	enzyme	494	0.000159076
Q9QUH0	(Q9QUH0) Glutaredoxin-1 (Thioltransferase-1) (TTase-1)	4	Cytoplasm	enzyme	106	0.001588621
Q9QUI0	(Q9QUI0) Transforming protein RhoA precursor	4	Cytoplasm	enzyme	193	0.000174501
Q9QWR8	(Q9QWR8) Alpha-N-acetylgalactosaminidase precursor (EC 3.2.1.49) (Alpha-galactosidase B)	3	Cytoplasm	enzyme	415	8.11536E-05
Q9R0P3	(Q9R0P3) Esterase D (EC 3.1.1.1) (Esterase 10) (Sid 478)	9	Cytoplasm	enzyme	282	0.000577236
Q60932	(Q60932) Voltage-dependent anion-selective channel protein 1 (VDAC-1) (mVDAC1) (mVDAC5) (Outer mitochondrial membrane protein porin 1) (Plasmalemmal porin)	25	Cytoplasm	ion channel	296	0.013994891
Q9QYB1	(Q9QYB1) Chloride intracellular channel protein 4 (mc3s5/mtCLIC)	12	Cytoplasm	ion channel	252	0.000846424
P09411	(P09411) Phosphoglycerate kinase 1 (EC 2.7.2.3)	33	Cytoplasm	kinase	416	0.00375108
P52480	(P52480) Pyruvate kinase isozyme M2 (EC 2.7.1.40)	13	Cytoplasm	kinase	530	0.000444814
P97328	(P97328) Ketohexokinase (EC 2.7.1.3) (Hepatic fructokinase)	7	Cytoplasm	kinase	298	0.004595983
Q6PDN3	(Q6PDN3) Myosin light chain kinase, smooth muscle (EC 2.7.11.18) (MLCK) (Telokin) (Kinase-related protein) (KRP)	7	Cytoplasm	kinase	1941	3.18106E-05
Q8K183	(Q8K183) Pyridoxal kinase (EC 2.7.1.35) (Pyridoxine kinase)	6	Cytoplasm	kinase	312	0.000251871

Accession Number	Protein Name	Number of Identified Peptides	Subcellular Location	Protein Family	Number of Amino Acid	NSAF
Q9R0Y5	(Q9R0Y5) Adenylate kinase isoenzyme 1 (EC 2.7.4.3) (ATP-AMP transphosphorylase) (AK1) (Myokinase)	7	Cytoplasm	kinase	194	0.000723341
P07901	(P07901) Heat shock protein HSP 90-alpha (HSP 86) (Tumor-specific transplantation 86 kDa antigen) (TSTA)	41	Cytoplasm	other	732	0.002499835
P08113	(P08113) Endoplasmin precursor (Heat shock protein 90 kDa beta member 1) (94 kDa glucose-regulated protein) (GRP94) (ERP99) (Polymorphic tumor rejection antigen 1) (Tumor rejection antigen gp96)	36	Cytoplasm	other	802	0.001693737
P11499	(P11499) Heat shock protein HSP 90-beta (HSP 84) (Tumor-specific transplantation 84 kDa antigen) (TSTA)	32	Cytoplasm	other	723	0.001133495
P12658	(P12658) Calbindin (Vitamin D-dependent calcium-binding protein, avian-type) (Calbindin D28) (D-28K) (Spot 35 protein) (PCD-29)	23	Cytoplasm	other	260	0.003108809
P12815	(P12815) Programmed cell death protein 6 (Probable calcium-binding protein ALG-2) (PMP41) (ALG-257)	3	Cytoplasm	other	191	0.000146941
P14602	(P14602) Heat-shock protein beta-1 (HspB1) (Heat shock 27 kDa protein) (HSP 27) (Growth-related 25 kDa protein) (P25) (HSP25)	6	Cytoplasm	other	209	0.000349142
P17563	(P17563) Selenium-binding protein 1 (56 kDa selenium-binding protein) (SP56)	16	Cytoplasm	other	472	0.000713533
P20029	(P20029) 78 kDa glucose-regulated protein precursor (GRP 78) (Immunoglobulin heavy chain-binding protein) (BiP)	47	Cytoplasm	other	655	0.002742291
P26043	(P26043) Radixin (ESP10)	12	Cytoplasm	other	583	0.000259956
P31786	(P31786) Acyl-CoA-binding protein (ACBP) (Diazepam-binding inhibitor) (DBI) (Endozepine) (EP)	11	Cytoplasm	other	86	0.005547858
P32848	(P32848) Parvalbumin alpha	1	Cytoplasm	other	109	5.14966E-05
P35979	(P35979) 60S ribosomal protein L12	6	Cytoplasm	other	165	0.003129744
P47754	(P47754) F-actin capping protein alpha-2 subunit (CapZ alpha-2)	7	Cytoplasm	other	285	0.000275733

Accession Number	Protein Name	Number of Identified Peptides	Subcellular Location	Protein Family	Number of Amino Acid	NSAF
P47757	(P47757) F-actin capping protein subunit beta (CapZ beta)	2	Cytoplasm	other	276	0.000122025
P50396	(P50396) Rab GDP dissociation inhibitor alpha (Rab GDI alpha) (Guanosine diphosphate dissociation inhibitor 1) (GDI-1)	5	Cytoplasm	other	447	0.000288819
P52760	(P52760) Ribonuclease UK114 (EC 3.1.-.-) (Heat-responsive protein 12)	15	Cytoplasm	other	134	0.013865262
P57780	(P57780) Alpha-actinin-4 (Non-muscle alpha-actinin 4) (F-actin cross linking protein)	17	Cytoplasm	other	912	0.000209261
P60710	(P60710) Actin, cytoplasmic 1 (Beta-actin)	16	Cytoplasm	other	375	0.00423604
P61982	(P61982) 14-3-3 protein gamma	2	Cytoplasm	other	246	0.000159723
P62259	(P62259) 14-3-3 protein epsilon (14-3-3E)	11	Cytoplasm	other	255	0.001298724
P62737	(P62737) Actin, aortic smooth muscle (Alpha-actin-2)	41	Cytoplasm	other	377	0.002948008
P62889	(P62889) 60S ribosomal protein L30	3	Cytoplasm	other	114	0.000443142
P62908	(P62908) 40S ribosomal protein S3	7	Cytoplasm	other	243	0.000785376
P62962	(P62962) Profilin-1 (Profilin I)	5	Cytoplasm	other	139	0.001655671
P62983	(P62983) 40S ribosomal protein S27a	3	Cytoplasm	other	80	0.000701641
P62991	(P62991) Ubiquitin	4	Cytoplasm	other	76	0.000590855
P68254	(P68254) 14-3-3 protein theta (14-3-3 protein tau)	5	Cytoplasm	other	245	0.00032075
P68372	(P68372) Tubulin beta-2C chain	34	Cytoplasm	other	445	0.004427433
P70296	(P70296) Phosphatidylethanolamine-binding protein 1 (PEBP-1) (HCNPpp) [Contains: Hippocampal cholinergic neurostimulating peptide (HCNP)]	11	Cytoplasm	other	186	0.001539083
P70444	(P70444) BH3-interacting domain death agonist (BID) (p22 BID) [Contains: BH3-interacting domain death agonist p15 (p15 BID); BH3-interacting domain death agonist p13 (p13 BID); BH3-interacting domain death agonist p11 (p11 BID)]	4	Cytoplasm	other	195	0.000287853
Q02819	(Q02819) Nucleobindin-1 precursor (CALNUC)	18	Cytoplasm	other	459	0.000415787
Q61233	(Q61233) Plastin-2 (L-plastin) (Lymphocyte cytosolic protein 1) (LCP-1) (65 kDa macrophage protein) (pp65)	2	Cytoplasm	other	626	2.69E-05

Accession Number	Protein Name	Number of Identified Peptides	Subcellular Location	Protein Family	Number of Amino Acid	NSAF
Q61316	(Q61316) Heat shock 70 kDa protein 4 (Heat shock 70-related protein APG-2)	12	Cytoplasm	other	841	0.00020023
Q61598	(Q61598) Rab GDP dissociation inhibitor beta (Rab GDI beta) (Guanosine diphosphate dissociation inhibitor 2) (GDI-2) (GDI-3)	12	Cytoplasm	other	445	0.000605461
Q61599	(Q61599) Rho GDP-dissociation inhibitor 2 (Rho GDI 2) (Rho-GDI beta) (D4)	7	Cytoplasm	other	199	0.000620547
Q61696	(Q61696) Heat shock 70 kDa protein 1A (Heat shock 70 kDa protein 3) (HSP70.3) (Hsp68)	5	Cytoplasm	other	641	0.000122596
Q62426	(Q62426) Cystatin B (Stefin B)	3	Cytoplasm	other	98	0.000400938
Q6ZWN5	(Q6ZWN5) 40S ribosomal protein S9	6	Cytoplasm	other	193	0.000698005
Q7TPR4	(Q7TPR4) Alpha-actinin-1 (Alpha-actinin cytoskeletal isoform) (Non-muscle alpha-actinin-1) (F-actin cross linking protein)	6	Cytoplasm	other	892	0.000100684
Q80X90	(Q80X90) Filamin-B (FLN-B) (Beta-filamin) (Actin-binding-like protein) (ABP-280-like protein)	14	Cytoplasm	other	2602	4.74592E-05
Q8BJF9	(Q8BJF9) Charged multivesicular body protein 2b (Chromatin-modifying protein 2b) (CHMP2b)	2	Cytoplasm	other	213	0.000105411
Q8VEJ9	(Q8VEJ9) Vacuolar sorting protein 4a	2	Cytoplasm	other	437	2.56894E-05
Q91VI7	(Q91VI7) Ribonuclease inhibitor (Ribonuclease/angiogenin inhibitor 1)	3	Cytoplasm	other	456	8.61664E-05
Q99JB8	(Q99JB8) Protein kinase C and casein kinase II substrate protein 3	5	Cytoplasm	other	424	0.000132385
Q99K51	(Q99K51) Plastin-3 (T-plastin)	4	Cytoplasm	other	627	5.37141E-05
Q99KJ8	(Q99KJ8) Dynactin subunit 2 (Dynactin complex 50 kDa subunit) (50 kDa dynein-associated polypeptide) (p50 dynamitin) (DCTN-50) (Growth cone membrane protein 23-48K) (GMP23-48K)	9	Cytoplasm	other	401	0.000307952
Q99L47	(Q99L47) Hsc70-interacting protein (Hip) (Protein ST13 homolog) (Protein FAM10A1)	9	Cytoplasm	other	371	0.000438762
Q99PT1	(Q99PT1) Rho GDP-dissociation inhibitor 1 (Rho GDI 1) (Rho-GDI alpha) (GDI-1)	14	Cytoplasm	other	203	0.001493147

Accession Number	Protein Name	Number of Identified Peptides	Subcellular Location	Protein Family	Number of Amino Acid	NSAF
Q9BCZ4	(Q9BCZ4) Selenoprotein S (VCP-interacting membrane protein) (Minor histocompatibility antigen H47)	2	Cytoplasm	other	190	0.000177257
Q9CQ10	(Q9CQ10) Charged multivesicular body protein 3 (Chromatin-modifying protein 3) (Vacuolar protein sorting 24)	2	Cytoplasm	other	223	0.000100684
Q9CQI6	(Q9CQI6) Coactosin-like protein	4	Cytoplasm	other	141	0.000437904
Q9CQV8	(Q9CQV8) 14-3-3 protein beta/alpha (Protein kinase C inhibitor protein 1) (KCIP-1)	7	Cytoplasm	other	245	0.000366572
Q9D7S9	(Q9D7S9) Charged multivesicular body protein 5 (Chromatin-modifying protein 5) (SNF7 domain-containing protein 2)	1	Cytoplasm	other	219	5.12614E-05
Q9D8B3	(Q9D8B3) Charged multivesicular body protein 4b (Chromatin-modifying protein 4b) (CHMP4b)	2	Cytoplasm	other	224	0.000150352
Q9DB34	(Q9DB34) Charged multivesicular body protein 2a (Chromatin-modifying protein 2a) (CHMP2a) (Vacuolar protein sorting 2) (mVps2)	2	Cytoplasm	other	222	7.58531E-05
Q9JLQ0	(Q9JLQ0) CD2-associated protein (Mesenchyme-to-epithelium transition protein with SH3 domains 1) (METS-1)	6	Cytoplasm	other	637	9.693E-05
Q9JM76	(Q9JM76) Actin-related protein 2/3 complex subunit 3 (ARP2/3 complex 21 kDa subunit) (p21-ARC)	2	Cytoplasm	other	177	0.000221988
Q9QXY6	(Q9QXY6) EH-domain-containing protein 3	3	Cytoplasm	other	535	6.2951E-05
Q9QZ06	(Q9QZ06) Toll-interacting protein	1	Cytoplasm	other	274	2.04859E-05
Q9R0P5	(Q9R0P5) Destrin (Actin-depolymerizing factor) (ADF) (Sid 23)	3	Cytoplasm	other	164	0.000239585
Q9R257	(Q9R257) Heme-binding protein 1 (p22HBP)	7	Cytoplasm	other	190	0.000413599
Q9WTI7	(Q9WTI7) Myosin Ic (Myosin I beta) (MMIb)	3	Cytoplasm	other	1028	3.82217E-05
Q9WU78	(Q9WU78) Programmed cell death 6-interacting protein (ALG-2-interacting protein X) (ALG-2-interacting protein 1) (E2F1-inducible protein) (Eig2)	2	Cytoplasm	other	869	1.93779E-05
Q9WVA4	(Q9WVA4) Transgelin-2	11	Cytoplasm	other	211	0.003804157
Q9WVK4	(Q9WVK4) EH-domain-containing protein 1 (mPAST1)	15	Cytoplasm	other	534	0.000325856

Accession Number	Protein Name	Number of Identified Peptides	Subcellular Location	Protein Family	Number of Amino Acid	NSAF
Q9Z0F7	(Q9Z0F7) Gamma-synuclein (Persyn)	1	Cytoplasm	other	123	0.000182541
O09061	(O09061) Proteasome subunit beta type 1 (EC 3.4.25.1) (Proteasome component C5) (Macropain subunit C5) (Multicatalytic endopeptidase complex subunit C5) (Proteasome gamma chain)	8	Cytoplasm	peptidase	240	0.000491149
O55234	(O55234) Proteasome subunit beta type 5 precursor (EC 3.4.25.1) (Proteasome epsilon chain) (Macropain epsilon chain) (Multicatalytic endopeptidase complex epsilon chain) (Proteasome subunit X) (Proteasome chain 6)	2	Cytoplasm	peptidase	209	8.05712E-05
O70435	(O70435) Proteasome subunit alpha type 3 (EC 3.4.25.1) (Proteasome component C8) (Macropain subunit C8) (Multicatalytic endopeptidase complex subunit C8) (Proteasome subunit K)	4	Cytoplasm	peptidase	254	0.000154692
O88456	(O88456) Calpain small subunit 1 (CSS1) (Calcium-dependent protease small subunit 1) (Calcium-dependent protease small subunit) (CDPS) (Calpain regulatory subunit) (Calcium-activated neutral proteinase small subunit) (CANP small subunit)	4	Cytoplasm	peptidase	269	0.000166933
O89017	(O89017) Legumain precursor (EC 3.4.22.34) (Asparaginyl endopeptidase) (Protease, cysteine 1)	4	Cytoplasm	peptidase	435	0.000154845
O89023	(O89023) Tripeptidyl-peptidase 1 precursor (EC 3.4.14.9) (Tripeptidyl-peptidase I) (TPP-I) (Tripeptidyl aminopeptidase) (Lysosomal pepstatin insensitive protease) (LPIC)	2	Cytoplasm	peptidase	562	5.99266E-05
P10605	(P10605) Cathepsin B precursor (EC 3.4.22.1) (Cathepsin B1) [Contains: Cathepsin B light chain; Cathepsin B heavy chain]	3	Cytoplasm	peptidase	339	9.93474E-05
P18242	(P18242) Cathepsin D precursor (EC 3.4.23.5)	13	Cytoplasm	peptidase	410	0.00049286
P27773	(P27773) Protein disulfide-isomerase A3 precursor (EC 5.3.4.1) (Disulfide isomerase ER-60) (ERp60) (58 kDa microsomal protein) (p58) (ERp57)	37	Cytoplasm	peptidase	504	0.002338803

Accession Number	Protein Name	Number of Identified Peptides	Subcellular Location	Protein Family	Number of Amino Acid	NSAF
P31428	(P31428) Dipeptidase 1 precursor (EC 3.4.13.19) (Microsomal dipeptidase) (Renal dipeptidase) (Membrane-bound dipeptidase 1) (MBD-1)	6	Cytoplasm	peptidase	410	0.000301192
P49722	(P49722) Proteasome subunit alpha type 2 (EC 3.4.25.1) (Proteasome component C3) (Macropain subunit C3) (Multicatalytic endopeptidase complex subunit C3)	6	Cytoplasm	peptidase	233	0.000505904
P49935	(P49935) Cathepsin H precursor (EC 3.4.22.16) (Cathepsin B3) (Cathepsin BA) [Contains: Cathepsin H mini chain; Cathepsin H heavy chain; Cathepsin H light chain]	4	Cytoplasm	peptidase	333	0.000219131
P56399	(P56399) Ubiquitin carboxyl-terminal hydrolase 5 (EC 3.1.2.15) (Ubiquitin thioesterase 5) (Ubiquitin-specific-processing protease 5) (Deubiquitinating enzyme 5) (Isopeptidase T)	1	Cytoplasm	peptidase	858	1.30842E-05
P99026	(P99026) Proteasome subunit beta type 4 precursor (EC 3.4.25.1) (Proteasome beta chain) (Macropain beta chain) (Multicatalytic endopeptidase complex beta chain) (Proteasome chain 3)	5	Cytoplasm	peptidase	264	0.000212618
Q60692	(Q60692) Proteasome subunit beta type 6 precursor (EC 3.4.25.1) (Proteasome delta chain) (Macropain delta chain) (Multicatalytic endopeptidase complex delta chain) (Proteasome subunit Y)	1	Cytoplasm	peptidase	238	4.71691E-05
Q8R016	(Q8R016) Bleomycin hydrolase (EC 3.4.22.40) (BLM hydrolase) (BMH) (BH)	2	Cytoplasm	peptidase	455	2.46731E-05
Q9CPY7	(Q9CPY7) Cytosol aminopeptidase (EC 3.4.11.1) (Leucine aminopeptidase) (LAP) (Leucyl aminopeptidase) (Proline aminopeptidase) (EC 3.4.11.5) (Prolyl aminopeptidase)	34	Cytoplasm	peptidase	487	0.0012448
Q9D1A2	(Q9D1A2) Cytosolic nonspecific dipeptidase (Glutamate carboxypeptidase-like protein 1) (CNDP dipeptidase 2)	23	Cytoplasm	peptidase	475	0.001193528

Accession Number	Protein Name	Number of Identified Peptides	Subcellular Location	Protein Family	Number of Amino Acid	NSAF
Q9ET22	(Q9ET22) Dipeptidyl-peptidase 2 precursor (EC 3.4.14.2) (Dipeptidyl-peptidase II) (DPP II) (Dipeptidyl aminopeptidase II) (Quiescent cell proline dipeptidase) (Dipeptidyl peptidase 7)	2	Cytoplasm	peptidase	506	2.21863E-05
Q9QUM9	(Q9QUM9) Proteasome subunit alpha type 6 (EC 3.4.25.1) (Proteasome iota chain) (Macropain iota chain) (Multicatalytic endopeptidase complex iota chain)	4	Cytoplasm	peptidase	246	0.000136906
Q9R1P1	(Q9R1P1) Proteasome subunit beta type 3 (EC 3.4.25.1) (Proteasome theta chain) (Proteasome chain 13) (Proteasome component C10-II)	1	Cytoplasm	peptidase	205	5.47622E-05
Q9R1P3	(Q9R1P3) Proteasome subunit beta type 2 (EC 3.4.25.1) (Proteasome component C7-I) (Macropain subunit C7-I) (Multicatalytic endopeptidase complex subunit C7-I)	3	Cytoplasm	peptidase	201	0.000167556
Q9Z2U0	(Q9Z2U0) Proteasome subunit alpha type 7 (EC 3.4.25.1) (Proteasome subunit RC6-1)	5	Cytoplasm	peptidase	248	0.00031687
O70250	(O70250) Phosphoglycerate mutase 2 (EC 5.4.2.1) (EC 5.4.2.4) (EC 3.1.3.13) (Phosphoglycerate mutase isozyme M) (PGAM-M) (BPG-dependent PGAM 2) (Muscle-specific phosphoglycerate mutase)	3	Cytoplasm	phosphatase	252	0.000133646
Q9DAK9	(Q9DAK9) 14 kDa phosphohistidine phosphatase (EC 3.1.3.-) (Phosphohistidine phosphatase 1)	5	Cytoplasm	phosphatase	124	0.00031687
Q9DBJ1	(Q9DBJ1) Phosphoglycerate mutase 1 (EC 5.4.2.1) (EC 5.4.2.4) (EC 3.1.3.13) (Phosphoglycerate mutase isozyme B) (PGAM-B) (BPG-dependent PGAM 1)	21	Cytoplasm	phosphatase	253	0.001575225
Q9JM14	(Q9JM14) 5'(3')-deoxyribonucleotidase, cytosolic type (EC 3.1.3.-) (Cytosolic 5',3'-pyrimidine nucleotidase) (Deoxy-5'-nucleotidase 1) (dNT-1)	6	Cytoplasm	phosphatase	200	0.00044905
Q9QXD6	(Q9QXD6) Fructose-1,6-bisphosphatase 1 (EC 3.1.3.11) (D-fructose-1,6-bisphosphate 1-phosphohydrolase 1) (FBPase 1)	31	Cytoplasm	phosphatase	337	0.008911048

Accession Number	Protein Name	Number of Identified Peptides	Subcellular Location	Protein Family	Number of Amino Acid	NSAF
P14211	(P14211) Calreticulin precursor (CRP55) (Calregulin) (HACBP) (ERp60)	20	Cytoplasm	transcription regulator	416	0.002509715
P17182	(P17182) Alpha-enolase (EC 4.2.1.11) (2-phospho-D-glycerate hydro-lyase) (Non-neural enolase) (NNE) (Enolase 1)	47	Cytoplasm	transcription regulator	433	0.010915134
Q6ZQ38	(Q6ZQ38) Cullin-associated NEDD8-dissociated protein 1 (Cullin-associated and neddylation-dissociated protein 1) (p120 CAND1)	2	Cytoplasm	transcription regulator	1230	2.28176E-05
O55135	(O55135) Eukaryotic translation initiation factor 6 (eIF-6) (B4 integrin interactor) (CAB) (p27(BBP))	2	Cytoplasm	translation regulator	245	9.16429E-05
P10126	(P10126) Elongation factor 1-alpha 1 (EF-1-alpha-1) (Elongation factor 1 A-1) (eEF1A-1) (Elongation factor Tu) (EF-Tu)	25	Cytoplasm	translation regulator	462	0.003365446
P58252	(P58252) Elongation factor 2 (EF-2)	5	Cytoplasm	translation regulator	857	7.85969E-05
P63242	(P63242) Eukaryotic translation initiation factor 5A-1 (eIF-5A-1) (eIF-5A1) (Eukaryotic initiation factor 5A isoform 1) (eIF-5A) (eIF-4D)	9	Cytoplasm	translation regulator	153	0.004879385
O08997	(O08997) Copper transport protein ATOX1 (Metal transport protein ATX1)	2	Cytoplasm	transporter	68	0.00041273
P01942	(P01942) Hemoglobin subunit alpha (Hemoglobin alpha chain) (Alpha-globin)	6	Cytoplasm	transporter	141	0.00473732
P02088	(P02088) Hemoglobin beta-1 subunit (Hemoglobin beta-1 chain) (Beta-1-globin) (Hemoglobin beta-major chain)	5	Cytoplasm	transporter	146	0.007458539
P04117	(P04117) Fatty acid-binding protein, adipocyte (AFABP) (Adipocyte lipid-binding protein) (ALBP) (A-FABP) (P2 adipocyte protein) (Myelin P2 protein homolog) (3T3-L1 lipid-binding protein) (422 protein) (P15)	9	Cytoplasm	transporter	131	0.001842477
P11404	(P11404) Fatty acid-binding protein, heart (H-FABP) (Heart-type fatty acid-binding protein) (Mammary-derived growth inhibitor) (MDGI)	7	Cytoplasm	transporter	132	0.000595332

Accession Number	Protein Name	Number of Identified Peptides	Subcellular Location	Protein Family	Number of Amino Acid	NSAF
P12710	(P12710) Fatty acid-binding protein, liver (L-FABP) (14 kDa selenium-binding protein)	2	Cytoplasm	transporter	127	8.83957E-05
P46467	(P46467) Vacuolar sorting protein 4b (SKD1 protein)	2	Cytoplasm	transporter	444	6.32109E-05
P50516	(P50516) Vacuolar ATP synthase catalytic subunit A, ubiquitous isoform (EC 3.6.3.14) (V-ATPase subunit A 1) (Vacuolar proton pump alpha subunit 1) (V-ATPase 69 kDa subunit 1)	19	Cytoplasm	transporter	617	0.000454873
P50518	(P50518) Vacuolar ATP synthase subunit E (EC 3.6.3.14) (V-ATPase E subunit) (Vacuolar proton pump E subunit) (V-ATPase 31 kDa subunit) (P31)	19	Cytoplasm	transporter	228	0.002437279
P56480	(P56480) ATP synthase beta chain, mitochondrial precursor (EC 3.6.3.14)	57	Cytoplasm	transporter	529	0.007088032
P84078	(P84078) ADP-ribosylation factor 1	2	Cytoplasm	transporter	180	9.35521E-05
Q03265	(Q03265) ATP synthase alpha chain, mitochondrial precursor (EC 3.6.3.14)	44	Cytoplasm	transporter	553	0.006445453
Q9CR51	(Q9CR51) Vacuolar ATP synthase subunit G 1 (EC 3.6.3.14) (V-ATPase G subunit 1) (Vacuolar proton pump G subunit 1) (V-ATPase 13 kDa subunit 1)	5	Cytoplasm	transporter	117	0.000671656
Q9DBH5	(Q9DBH5) Vesicular integral-membrane protein VIP36 precursor (Lectin, mannose-binding 2)	3	Cytoplasm	transporter	358	6.27165E-05
Q9WVE8	(Q9WVE8) Protein kinase C and casein kinase substrate in neurons protein 2	7	Cytoplasm	transporter	486	0.00033494
Q9Z1G3	(Q9Z1G3) Vacuolar ATP synthase subunit C (EC 3.6.3.14) (V-ATPase C subunit) (Vacuolar proton pump C subunit)	2	Cytoplasm	transporter	381	7.36631E-05
P10923	(P10923) Osteopontin precursor (Bone sialoprotein-1) (Secreted phosphoprotein 1) (SPP-1) (Minopontin) (Early T-lymphocyte activation 1 protein) (2AR) (Calcium oxalate crystal growth inhibitor protein)	2	Extracellular Space	cytokine	294	7.63691E-05
P34884	(P34884) Macrophage migration inhibitory factor (MIF) (Phenylpyruvate tautomerase) (EC 5.3.2.1) (Glycosylation-inhibiting factor) (GIF) (Delayed early response protein 6) (DER6)	2	Extracellular Space	cytokine	114	0.003594371

Accession Number	Protein Name	Number of Identified Peptides	Subcellular Location	Protein Family	Number of Amino Acid	NSAF
Q9CPT4	(Q9CPT4) Uncharacterized protein C19orf10 homolog precursor (Stromal cell-derived growth factor SF20) (Interleukin-25) (IL-25)	2	Extracellular Space	cytokine	166	0.000135256
O09164	(O09164) Extracellular superoxide dismutase [Cu-Zn] precursor (EC 1.15.1.1) (EC-SOD)	2	Extracellular Space	enzyme	251	8.94522E-05
P06745	(P06745) Glucose-6-phosphate isomerase (EC 5.3.1.9) (GPI) (Phosphoglucose isomerase) (PGI) (Phosphohexose isomerase) (PHI) (Neuroleukin) (NLK)	13	Extracellular Space	enzyme	557	0.000403097
P08905	(P08905) Lysozyme C type M precursor (EC 3.2.1.17) (1,4-beta-N-acetylmuramidase C)	6	Extracellular Space	enzyme	148	0.001175723
P46412	(P46412) Glutathione peroxidase 3 precursor (EC 1.11.1.9) (GSHPx-3) (GPx-3) (Plasma glutathione peroxidase) (GSHPx-P)	12	Extracellular Space	enzyme	226	0.000769942
Q61292	(Q61292) Laminin beta-2 chain precursor (S-laminin) (S-LAM)	5	Extracellular Space	enzyme	1799	2.80812E-05
Q8CIF4	(Q8CIF4) Biotinidase precursor (EC 3.5.1.12)	1	Extracellular Space	enzyme	524	3.21362E-05
Q8JZV9	(Q8JZV9) Dehydrogenase/reductase SDR family member 6 precursor (EC 1.1.-.-)	8	Extracellular Space	enzyme	245	0.000778965
Q9CQ01	(Q9CQ01) Ribonuclease T2 precursor (EC 3.1.27.-) (Ribonuclease 6)	2	Extracellular Space	enzyme	259	0.000238395
P01132	(P01132) Pro-epidermal growth factor precursor (EGF) [Contains: Epidermal growth factor]	2	Extracellular Space	growth factor	1217	9.22453E-06
Q61592	(Q61592) Growth-arrest-specific protein 6 precursor (GAS-6)	1	Extracellular Space	growth factor	674	8.32808E-06
Q9CQ45	(Q9CQ45) Neudesin precursor (Neuron-derived neurotrophic factor) (Secreted protein of unknown function) (SPUF protein)	2	Extracellular Space	growth factor	171	0.000131301
O09051	(O09051) Guanylate cyclase activator 2B precursor [Contains: Uroguanylin (UGN)]	1	Extracellular Space	other	106	5.2954E-05
O35206	(O35206) Collagen alpha-1(XV) chain precursor [Contains: Endostatin (Endostatin-XV)]	2	Extracellular Space	other	1367	8.21233E-06

Accession Number	Protein Name	Number of Identified Peptides	Subcellular Location	Protein Family	Number of Amino Acid	NSAF
P01029	(P01029) Complement C4-B precursor [Contains: Complement C4 beta chain; Complement C4 alpha chain; C4a anaphylatoxin; Complement C4 gamma chain]	1	Extracellular Space	other	1738	6.45929E-06
P02468	(P02468) Laminin gamma-1 chain precursor (Laminin B2 chain)	8	Extracellular Space	other	1607	5.23938E-05
P06683	(P06683) Complement component C9 precursor	2	Extracellular Space	other	548	2.04859E-05
P06909	(P06909) Complement factor H precursor (Protein beta-1-H)	1	Extracellular Space	other	1234	4.54873E-06
P07758	(P07758) Alpha-1-antitrypsin 1-1 precursor (Serine protease inhibitor 1-1) (Alpha-1 protease inhibitor 1) (Alpha-1-antiproteinase) (AAT)	4	Extracellular Space	other	413	0.000163093
P10493	(P10493) Nidogen-1 precursor (Entactin)	10	Extracellular Space	other	1245	0.00012173
P13020	(P13020) Gelsolin precursor (Actin-depolymerizing factor) (ADF) (Brevin)	4	Extracellular Space	other	780	5.75705E-05
P16110	(P16110) Galectin-3 (Galactose-specific lectin 3) (Mac-2 antigen) (IgE-binding protein) (35 kDa lectin) (Carbohydrate-binding protein 35) (CBP 35) (Laminin-binding protein) (Lectin L-29) (L-34 galactoside-binding lectin)	2	Extracellular Space	other	263	8.53708E-05
P21460	(P21460) Cystatin C precursor (Cystatin 3)	1	Extracellular Space	other	140	4.00938E-05
P22599	(P22599) Alpha-1-antitrypsin 1-2 precursor (Serine protease inhibitor 1-2) (Alpha-1 protease inhibitor 2) (Alpha-1-antiproteinase) (AAT)	3	Extracellular Space	other	413	4.07733E-05
P26339	(P26339) Chromogranin A precursor (CgA) [Contains: Pancreastatin; Beta-granin; WE-14]	2	Extracellular Space	other	463	2.42468E-05
P28653	(P28653) Biglycan precursor (Bone/cartilage proteoglycan I) (PG-S1)	2	Extracellular Space	other	369	4.56352E-05
P29699	(P29699) Alpha-2-HS-glycoprotein precursor (Fetuin-A) (Countertrypin)	1	Extracellular Space	other	345	3.25399E-05
P32261	(P32261) Antithrombin-III precursor (ATIII)	1	Extracellular Space	other	465	1.20712E-05
P39061	(P39061) Collagen alpha-1(XVIII) chain precursor [Contains: Endostatin]	4	Extracellular Space	other	1774	3.48052E-05

Accession Number	Protein Name	Number of Identified Peptides	Subcellular Location	Protein Family	Number of Amino Acid	NSAF
P51885	(P51885) Lumican precursor (Keratan sulfate proteoglycan lumican) (KSPG lumican)	2	Extracellular Space	other	338	0.000132855
P97298	(P97298) Pigment epithelium-derived factor precursor (PEDF) (Stromal cell-derived factor 3) (SDF-3) (Caspin)	2	Extracellular Space	other	417	6.73037E-05
Q00898	(Q00898) Alpha-1-antitrypsin 1-5 precursor (Serine protease inhibitor 1-5) (Alpha-1 protease inhibitor 5)	2	Extracellular Space	other	413	5.43644E-05
Q02788	(Q02788) Collagen alpha-2(VI) chain precursor	2	Extracellular Space	other	1029	1.63648E-05
Q04857	(Q04857) Collagen alpha-1(VI) chain precursor	4	Extracellular Space	other	1025	4.38098E-05
Q06890	(Q06890) Clusterin precursor (Sulfated glycoprotein 2) (SGP-2) (Clustrin) (Apolipoprotein J) (Apo-J) [Contains: Clusterin beta chain; Clusterin alpha chain]	2	Extracellular Space	other	448	5.01172E-05
Q61001	(Q61001) Laminin alpha-5 chain precursor	8	Extracellular Space	other	3718	1.96263E-05
Q61207	(Q61207) Sulfated glycoprotein 1 precursor (SGP-1) (Prosaposin)	3	Extracellular Space	other	557	5.03871E-05
Q8VCM7	(Q8VCM7) Fibrinogen gamma chain precursor	1	Extracellular Space	other	436	1.28741E-05
Q91X17	(Q91X17) Uromodulin precursor (Tamm-Horsfall urinary glycoprotein) (THP)	2	Extracellular Space	other	642	2.62296E-05
Q921I1	(Q921I1) Serotransferrin precursor (Transferrin) (Siderophilin) (Beta-1-metal-binding globulin)	11	Extracellular Space	other	697	0.000249651
Q9CQH0	(Q9CQH0) PDZK1-interacting protein 1 (17 kDa membrane-associated protein)	5	Extracellular Space	other	114	0.001378663
Q9Z0J0	(Q9Z0J0) Epididymal secretory protein E1 precursor (Niemann Pick type C2 protein homolog) (mE1)	2	Extracellular Space	other	149	0.000150688
O09043	(O09043) Napsin-A precursor (EC 3.4.23.-) (Kidney-derived aspartic protease-like protein) (KDAP-1) (KAP)	3	Extracellular Space	peptidase	419	0.000133965
P01027	(P01027) Complement C3 precursor (HSE-MSF) [Contains: Complement C3 beta chain; Complement C3 alpha chain; C3a anaphylatoxin; Complement C3b alpha' chain; Complement C3c fragment; Complement C3dg fragment; Complement C3g fragment; Complement C3	3	Extracellular Space	peptidase	1663	2.70024E-05

Accession Number	Protein Name	Number of Identified Peptides	Subcellular Location	Protein Family	Number of Amino Acid	NSAF
P04186	(P04186) Complement factor B precursor (EC 3.4.21.47) (C3/C5 convertase) [Contains: Complement factor B Ba fragment; Complement factor B Bb fragment]	2	Extracellular Space	peptidase	761	2.2128E-05
O88968	(O88968) Transcobalamin-2 precursor (Transcobalamin II) (TCII) (TC II)	1	Extracellular Space	transporter	430	6.52689E-05
P07309	(P07309) Transthyretin precursor (Prealbumin)	2	Extracellular Space	transporter	147	0.000801875
P07724	(P07724) Serum albumin precursor	14	Extracellular Space	transporter	608	0.001024765
P08226	(P08226) Apolipoprotein E precursor (Apo-E)	2	Extracellular Space	transporter	311	5.41459E-05
P21614	(P21614) Vitamin D-binding protein precursor (DBP) (Group-specific component) (Gc-globulin) (VDB)	2	Extracellular Space	transporter	476	5.89614E-05
Q00724	(Q00724) Plasma retinol-binding protein precursor (PRBP) (RBP)	1	Extracellular Space	transporter	201	5.5852E-05
Q61581	(Q61581) Insulin-like growth factor-binding protein 7 precursor (IGFBP-7) (IBP-7) (IGF-binding protein 7) (MAC25 protein)	4	Extracellular Space	transporter	281	0.000139829
Q91X72	(Q91X72) Hemopexin precursor	2	Extracellular Space	transporter	460	2.44049E-05
Q99JR5	(Q99JR5) Tubulointerstitial nephritis antigen-like precursor (Androgen-regulated gene 1 protein) (Adrenocortical zonation factor 1) (AZ-1) (Tubulointerstitial nephritis antigen-related protein) (TARP)	5	Extracellular Space	transporter	466	0.000156589
O70475	(O70475) UDP-glucose 6-dehydrogenase (EC 1.1.1.22) (UDP-Glc dehydrogenase) (UDP-GlcDH) (UDPGDH)	4	Nucleus	enzyme	493	5.69283E-05
P17225	(P17225) Polypyrimidine tract-binding protein 1 (PTB) (Heterogeneous nuclear ribonucleoprotein I) (hnRNP I)	7	Nucleus	enzyme	527	0.000543206
P23492	(P23492) Purine nucleoside phosphorylase (EC 2.4.2.1) (Inosine phosphorylase) (PNP)	3	Nucleus	enzyme	289	0.000116536
P29595	(P29595) NEDD8 precursor (Ubiquitin-like protein Nedd8) (Neddylin) (Neural precursor cell expressed developmentally down-regulated protein 8)	3	Nucleus	enzyme	81	0.000485085

Accession Number	Protein Name	Number of Identified Peptides	Subcellular Location	Protein Family	Number of Amino Acid	NSAF
P62827	(P62827) GTP-binding nuclear protein Ran (GTPase Ran) (Ras-like protein TC4)	2	Nucleus	enzyme	215	0.000652689
Q9Z1Q5	(Q9Z1Q5) Chloride intracellular channel protein 1 (Nuclear chloride ion channel 27) (NCC27)	6	Nucleus	ion channel	240	0.000771805
Q9DBP5	(Q9DBP5) UMP-CMP kinase (EC 2.7.4.14) (Cytidylate kinase) (Deoxycytidylate kinase) (Cytidine monophosphate kinase) (Uridine monophosphate/cytidine monophosphate kinase) (UMP/CMP kinase) (UMP/CMPK) (Uridine monophosphate kinase)	11	Nucleus	kinase	196	0.001632389
O54962	(O54962) Barrier-to-autointegration factor (Breakpoint cluster region protein 1) (LAP2-binding protein 1)	4	Nucleus	other	89	0.000693757
O89086	(O89086) Putative RNA-binding protein 3 (RNA-binding motif protein 3)	4	Nucleus	other	153	0.000586994
P18760	(P18760) Cofilin-1 (Cofilin, non-muscle isoform)	6	Nucleus	other	165	0.005034805
P23927	(P23927) Alpha crystallin B chain (Alpha(B)-crystallin) (P23)	2	Nucleus	other	175	0.000160375
P43276	(P43276) Histone H1.5 (H1 VAR.5) (H1b)	3	Nucleus	other	222	0.000278128
P45591	(P45591) Cofilin-2 (Cofilin, muscle isoform)	3	Nucleus	other	166	0.000304326
P48678	(P48678) Lamin-A/C	25	Nucleus	other	665	0.000582415
P62315	(P62315) Small nuclear ribonucleoprotein Sm D1 (snRNP core protein D1) (Sm-D1) (Sm-D autoantigen)	4	Nucleus	other	119	0.001462243
P97384	(P97384) Annexin A11 (Annexin XI) (Calcyclin-associated annexin 50) (CAP-50)	1	Nucleus	other	503	1.11593E-05
Q3THW5	(Q3THW5) Histone H2AV (H2A.F/Z)	4	Nucleus	other	127	0.007513635
Q78ZA7	(Q78ZA7) Nucleosome assembly protein 1-like 4	7	Nucleus	other	375	0.000284398
Q80VJ3	(Q80VJ3) c-Myc-responsive protein Rcl	2	Nucleus	other	173	0.000162229
Q91VW3	(Q91VW3) SH3 domain-binding glutamic acid-rich-like protein 3	4	Nucleus	other	93	0.000362137
Q99K48	(Q99K48) Non-POU domain-containing octamer-binding protein (NonO protein)	4	Nucleus	other	473	9.49366E-05
Q99LX0	(Q99LX0) Protein DJ-1	13	Nucleus	other	189	0.001128565

Accession Number	Protein Name	Number of Identified Peptides	Subcellular Location	Protein Family	Number of Amino Acid	NSAF
Q9CPQ3	(Q9CPQ3) Mitochondrial import receptor subunit TOM22 homolog (Translocase of outer membrane 22 kDa subunit homolog)	4	Nucleus	other	141	0.000517522
Q9QZM0	(Q9QZM0) Ubiquilin-2 (Protein linking IAP with cytoskeleton 2) (PLIC-2) (Ubiquitin-like product Chap1/Dsk2) (DSK2 homolog) (Chap1)	1	Nucleus	other	638	8.798E-06
Q9Z204	(Q9Z204) Heterogeneous nuclear ribonucleoproteins C1/C2 (hnRNP C1 / hnRNP C2)	5	Nucleus	other	313	0.000466266
Q3UM45	(Q3UM45) Protein phosphatase 1 regulatory subunit 7 (Protein phosphatase 1 regulatory subunit 22)	2	Nucleus	phosphatase	361	7.77441E-05
Q9Z0S1	(Q9Z0S1) 3'(2'),5'-bisphosphate nucleotidase 1 (EC 3.1.3.7) (Bisphosphate 3'-nucleotidase 1) (PAP-inositol-1,4-phosphatase) (PIP)	8	Nucleus	phosphatase	308	0.00065608
P56959	(P56959) RNA-binding protein FUS (Pigpen protein)	10	Nucleus	transcription regulator	518	0.000270904
P61458	(P61458) Pterin-4-alpha-carbinolamine dehydratase (EC 4.2.1.96) (PHS) (4-alpha-hydroxy-tetrahydropterin dehydratase) (Phenylalanine hydroxylase-stimulating protein) (Pterin carbinolamine dehydratase) (PCD) (Dimerization cofactor of hepatocyte n	9	Nucleus	transcription regulator	103	0.001798381
P62774	(P62774) Myotrophin (Protein V-1) (Granule cell differentiation protein)	4	Nucleus	transcription regulator	117	0.000623681
Q61187	(Q61187) Tumor susceptibility gene 101 protein	2	Nucleus	transcription regulator	391	2.87116E-05
Q8K0H5	(Q8K0H5) Transcription initiation factor TFIID subunit 10 (Transcription initiation factor TFIID 30 kDa subunit) (TAF(II)30) (TAFII-30) (mTAFII30)	1	Nucleus	transcription regulator	218	5.14966E-05
Q9JKB3	(Q9JKB3) DNA-binding protein A (Cold shock domain-containing protein A) (Y-box protein 3)	5	Nucleus	transcription regulator	361	0.000279879
Q9WTX5	(Q9WTX5) S-phase kinase-associated protein 1A (Cyclin A/CDK2-associated protein p19) (p19A) (p19skp1)	5	Nucleus	transcription regulator	162	0.000519734

Accession Number	Protein Name	Number of Identified Peptides	Subcellular Location	Protein Family	Number of Amino Acid	NSAF
P70168	(P70168) Importin beta-1 subunit (Karyopherin beta-1 subunit) (Nuclear factor P97) (Pore targeting complex 97 kDa subunit) (PTAC97) (SCG)	2	Nucleus	transporter	876	2.56307E-05
O88338	(O88338) Cadherin-16 precursor (Kidney-specific cadherin) (Ksp-cadherin)	18	Plasma Membrane	enzyme	830	0.000987369
P08752	(P08752) Guanine nucleotide-binding protein G(i), alpha-2 subunit (Adenylate cyclase-inhibiting G alpha protein)	1	Plasma Membrane	enzyme	354	3.17126E-05
P11276	(P11276) Fibronectin precursor (FN)	2	Plasma Membrane	enzyme	2477	4.5322E-06
P17047	(P17047) Lysosome-associated membrane glycoprotein 2 precursor (LAMP-2) (Lysosomal membrane glycoprotein type B) (LGP-B) (CD107b antigen)	1	Plasma Membrane	enzyme	415	8.11536E-05
P21278	(P21278) Guanine nucleotide-binding protein alpha-11 subunit	2	Plasma Membrane	enzyme	359	3.12709E-05
P21279	(P21279) Guanine nucleotide-binding protein G(q) subunit alpha (Guanine nucleotide-binding protein alpha-q)	1	Plasma Membrane	enzyme	353	1.59012E-05
P27601	(P27601) Guanine nucleotide-binding protein alpha-13 subunit (G alpha-13)	2	Plasma Membrane	enzyme	377	4.46668E-05
P62071	(P62071) Ras-related protein R-Ras2	4	Plasma Membrane	enzyme	204	0.000192607
P63094	(P63094) Guanine nucleotide-binding protein G(s) subunit alpha (Adenylate cyclase-stimulating G alpha protein)	2	Plasma Membrane	enzyme	394	7.12326E-05
Q61411	(Q61411) GTPase HRas precursor (Transforming protein p21) (p21ras) (H-Ras-1) (c-H-ras)	1	Plasma Membrane	enzyme	189	2.96991E-05
Q64444	(Q64444) Carbonic anhydrase 4 precursor (EC 4.2.1.1) (Carbonic anhydrase IV) (Carbonate dehydratase IV) (CA-IV)	3	Plasma Membrane	enzyme	305	7.36148E-05
Q64727	(Q64727) Vinculin (Metavinculin)	12	Plasma Membrane	enzyme	1065	0.000158116
Q9EQP2	(Q9EQP2) EH-domain-containing protein 4 (mPAST2)	6	Plasma Membrane	enzyme	541	0.000134881

Accession Number	Protein Name	Number of Identified Peptides	Subcellular Location	Protein Family	Number of Amino Acid	NSAF
Q9JLT2	(Q9JLT2) Trehalase precursor (EC 3.2.1.28) (Alpha,alpha-trehalase) (Alpha,alpha-trehalose glucohydrolase)	7	Plasma Membrane	enzyme	576	0.000185155
Q9Z0K8	(Q9Z0K8) Pantetheinase precursor (EC 3.5.1.92) (Pantetheine hydrolase) (Vascular non-inflammatory molecule 1) (Vanin-1)	2	Plasma Membrane	enzyme	512	0.000142521
Q8K3J9	(Q8K3J9) G-protein coupled receptor family C group 5 member C precursor (Retinoic acid-induced gene 3 protein) (RAIG-3)	3	Plasma Membrane	G-protein coupled receptor	440	5.10284E-05
Q04646	(Q04646) Sodium/potassium-transporting ATPase gamma chain (Sodium pump gamma chain) (Na+/K+ ATPase subunit gamma) (FXYD domain-containing ion transport regulator 2)	3	Plasma Membrane	ion channel	70	0.001202813
Q07076	(Q07076) Annexin A7 (Annexin VII) (Synexin)	1	Plasma Membrane	ion channel	463	2.42468E-05
Q8BKX1	(Q8BKX1) Brain-specific angiogenesis inhibitor 1-associated protein 2 (BAI1-associated protein 2) (BAI-associated protein 2) (Insulin receptor substrate p53) (IRSp53) (Insulin receptor substrate protein of 53 kDa) (Insulin receptor tyrosine k	4	Plasma Membrane	kinase	535	6.2951E-05
O55022	(O55022) Membrane-associated progesterone receptor component 1	5	Plasma Membrane	other	194	0.00095481
O55111	(O55111) Desmoglein-2 precursor	1	Plasma Membrane	other	1122	1.50084E-05
O70404	(O70404) Vesicle-associated membrane protein 8 (VAMP-8) (Endobrevin) (Edb)	1	Plasma Membrane	other	101	0.000111151
O89103	(O89103) Complement component C1q receptor precursor (Complement component 1 q subcomponent receptor 1) (C1qRp) (C1qR(p)) (C1q/MBL/SPA receptor) (CD93 antigen) (Cell surface antigen AA4) (Lymphocyte antigen 68)	2	Plasma Membrane	other	644	4.35802E-05
P07356	(P07356) Annexin A2 (Annexin II) (Lipocortin II) (Calpactin I heavy chain) (Chromobindin-8) (p36) (Protein I) (Placental anticoagulant protein IV) (PAP-IV)	10	Plasma Membrane	other	338	0.000332138

Accession Number	Protein Name	Number of Identified Peptides	Subcellular Location	Protein Family	Number of Amino Acid	NSAF
P09803	(P09803) Epithelial-cadherin precursor (E-cadherin) (Uvomorulin) (Cadherin-1) (ARC-1) (CD324 antigen) [Contains: E-Cad/CTF1; E-Cad/CTF2; E-Cad/CTF3]	12	Plasma Membrane	other	884	0.000222239
P10107	(P10107) Annexin A1 (Annexin I) (Lipocortin I) (Calpactin II) (Chromobindin-9) (p35) (Phospholipase A2 inhibitory protein)	4	Plasma Membrane	other	345	0.000130159
P14824	(P14824) Annexin A6 (Annexin VI) (Lipocortin VI) (P68) (P70) (Protein III) (Chromobindin-20) (67 kDa calelectrin) (Calphobindin-II) (CPB-II)	5	Plasma Membrane	other	672	7.51758E-05
P16546	(P16546) Spectrin alpha chain, brain (Spectrin, non-erythroid alpha chain) (Alpha-II spectrin) (Fodrin alpha chain) (Fragment)	34	Plasma Membrane	other	1458	0.000261792
P18572	(P18572) Basigin precursor (Basic immunoglobulin superfamily) (Membrane glycoprotein gp42) (HT7 antigen) (CD147 antigen)	4	Plasma Membrane	other	389	0.000187585
P26039	(P26039) Talin-1	37	Plasma Membrane	other	2541	0.000152423
P26040	(P26040) Ezrin (p81) (Cytovillin) (Villin-2)	23	Plasma Membrane	other	585	0.001995779
P26041	(P26041) Moesin (Membrane-organizing extension spike protein)	25	Plasma Membrane	other	576	0.001091441
P26645	(P26645) Myristoylated alanine-rich C-kinase substrate (MARCKS)	4	Plasma Membrane	other	308	0.000200469
P40124	(P40124) Adenylyl cyclase-associated protein 1 (CAP 1)	7	Plasma Membrane	other	473	0.000213607
P48036	(P48036) Annexin A5 (Annexin V) (Lipocortin V) (Endonexin II) (Calphobindin I) (CBP-I) (Placental anticoagulant protein I) (PAP-I) (PP4) (Thromboplastin inhibitor) (Vascular anticoagulant-alpha) (VAC-alpha) (Anchorin CII)	15	Plasma Membrane	other	319	0.000932589
P61161	(P61161) Actin-like protein 2 (Actin-related protein 2)	2	Plasma Membrane	other	394	7.12326E-05
P63024	(P63024) Vesicle-associated membrane protein 3 (VAMP-3) (Synaptobrevin-3) (Cellubrevin) (CEB)	1	Plasma Membrane	other	103	0.000163489
P63044	(P63044) Vesicle-associated membrane protein 2 (VAMP-2) (Synaptobrevin-2)	3	Plasma Membrane	other	115	0.000292859

Accession Number	Protein Name	Number of Identified Peptides	Subcellular Location	Protein Family	Number of Amino Acid	NSAF
P70441	(P70441) Ezrin-radixin-moesin-binding phosphoprotein 50 (EBP50) (Na(+)/H(+) exchange regulatory cofactor NHE-RF) (NHERF-1) (Regulatory cofactor of Na(+)/H(+) exchanger) (Sodium-hydrogen exchanger regulatory factor 1) (Solute carrier family 9	26	Plasma Membrane	other	354	0.001950324
P97429	(P97429) Annexin A4 (Annexin IV)	8	Plasma Membrane	other	318	0.000405981
Q08481	(Q08481) Platelet endothelial cell adhesion molecule precursor (PECAM-1) (CD31 antigen)	1	Plasma Membrane	other	727	7.72095E-06
Q60598	(Q60598) Src substrate cortactin	8	Plasma Membrane	other	546	0.00021589
Q6ZQI3	(Q6ZQI3) Protein KIAA0152 precursor	7	Plasma Membrane	other	291	0.000617251
Q80W68	(Q80W68) Kin of IRRE-like protein 1 precursor (Kin of irregular chiasm-like protein 1) (Nephrin-like protein 1)	1	Plasma Membrane	other	789	7.11423E-06
Q8VHF2	(Q8VHF2) Mucin and cadherin-like protein precursor (Mu-protocadherin)	1	Plasma Membrane	other	831	6.75467E-06
Q91XV3	(Q91XV3) Brain acid soluble protein 1 (BASP1 protein) (Neuronal axonal membrane protein NAP-22) (22 kDa neuronal tissue-enriched acidic protein)	4	Plasma Membrane	other	225	0.000598734
Q925F2	(Q925F2) Endothelial cell-selective adhesion molecule precursor	1	Plasma Membrane	other	394	2.8493E-05
Q99JY9	(Q99JY9) Actin-like protein 3 (Actin-related protein 3)	2	Plasma Membrane	other	417	4.03822E-05
Q9ESG4	(Q9ESG4) Collectrin precursor (Transmembrane protein 27)	4	Plasma Membrane	other	222	0.000151706
Q9JKF6	(Q9JKF6) Poliovirus receptor-related protein 1 precursor (Herpes virus entry mediator C) (HveC) (Nectin-1) (CD111 antigen)	2	Plasma Membrane	other	515	3.26978E-05
O35409	(O35409) Glutamate carboxypeptidase 2 (EC 3.4.17.21) (Glutamate carboxypeptidase II) (Membrane glutamate carboxypeptidase) (mGCP) (N-acetylated-alpha-linked acidic dipeptidase I) (NAALADase I) (Pteroylpoly-gamma-glutamate carboxypeptidase) (F	13	Plasma Membrane	peptidase	752	0.000298571

Accession Number	Protein Name	Number of Identified Peptides	Subcellular Location	Protein Family	Number of Amino Acid	NSAF
P09470	(P09470) Angiotensin-converting enzyme, somatic isoform precursor (EC 3.4.15.1) (Dipeptidyl carboxypeptidase I) (Kininase II) [Contains: Angiotensin-converting enzyme, somatic isoform, soluble form]	1	Plasma Membrane	peptidase	1312	1.28349E-05
P16406	(P16406) Glutamyl aminopeptidase (EC 3.4.11.7) (EAP) (Aminopeptidase A) (APA) (BP-1/6C3 antigen)	14	Plasma Membrane	peptidase	945	0.000136616
P28825	(P28825) Meprin A subunit alpha precursor (EC 3.4.24.18) (Endopeptidase-2) (MEP-1)	8	Plasma Membrane	peptidase	747	0.000308083
P28843	(P28843) Dipeptidyl peptidase 4 (EC 3.4.14.5) (Dipeptidyl peptidase IV) (DPP IV) (T-cell activation antigen CD26) (Thymocyte-activating molecule) (THAM) [Contains: Dipeptidyl peptidase 4 membrane form (Dipeptidyl peptidase IV membrane form); D	5	Plasma Membrane	peptidase	760	6.64712E-05
P97449	(P97449) Aminopeptidase N (EC 3.4.11.2) (mAPN) (Alanyl aminopeptidase) (Microsomal aminopeptidase) (Aminopeptidase M) (Membrane protein p161) (CD13 antigen)	27	Plasma Membrane	peptidase	965	0.000860873
Q08509	(Q08509) Epidermal growth factor receptor kinase substrate 8	2	Plasma Membrane	peptidase	821	1.36739E-05
Q61391	(Q61391) Neprilysin (EC 3.4.24.11) (Neutral endopeptidase) (NEP) (Enkephalinase) (Neutral endopeptidase 24.11) (Atriopeptidase) (CD10 antigen)	10	Plasma Membrane	peptidase	749	0.000112412
Q80V42	(Q80V42) Carboxypeptidase M precursor (EC 3.4.17.12)	3	Plasma Membrane	peptidase	443	3.80121E-05
P09242	(P09242) Alkaline phosphatase, tissue-nonspecific isozyme precursor (EC 3.1.3.1) (AP-TNAP) (TNSALP)	11	Plasma Membrane	phosphatase	524	0.000235666
P35822	(P35822) Receptor-type tyrosine-protein phosphatase kappa precursor (EC 3.1.3.48) (Protein-tyrosine phosphatase kappa) (R-PTP-kappa)	4	Plasma Membrane	phosphatase	1457	2.31151E-05
P97798	(P97798) Neogenin precursor	1	Plasma Membrane	transcription regulator	1493	7.51926E-06

Accession Number	Protein Name	Number of Identified Peptides	Subcellular Location	Protein Family	Number of Amino Acid	NSAF
P01902	(P01902) H-2 class I histocompatibility antigen, K-D alpha chain precursor (H-2K(D))	4	Plasma Membrane	transmembrane receptor	368	9.15184E-05
Q07113	(Q07113) Cation-independent mannose-6-phosphate receptor precursor (CI Man-6-P receptor) (CI-MPR) (M6PR) (Insulin-like growth factor 2 receptor) (Insulin-like growth factor II receptor) (IGF-II receptor) (M6P/IGF2 receptor) (M6P/IGF2R) (300 kD	1	Plasma Membrane	transmembrane receptor	2483	4.52125E-06
Q62165	(Q62165) Dystroglycan precursor (Dystrophin-associated glycoprotein 1) [Contains: Alpha-dystroglycan (Alpha-DG); Beta-dystroglycan (Beta-DG)]	2	Plasma Membrane	transmembrane receptor	893	2.51428E-05
Q9D0E1	(Q9D0E1) Heterogeneous nuclear ribonucleoprotein M (hnRNP M)	13	Plasma Membrane	transmembrane receptor	728	0.000239021
Q9JLB4	(Q9JLB4) Cubilin precursor (Intrinsic factor-cobalamin receptor)	5	Plasma Membrane	transmembrane receptor	3623	3.5634E-05
P10852	(P10852) 4F2 cell-surface antigen heavy chain (4F2hc)	8	Plasma Membrane	transporter	526	0.000224098
P14094	(P14094) Sodium/potassium-transporting ATPase subunit beta-1 (Sodium/potassium-dependent ATPase beta-1 subunit)	14	Plasma Membrane	transporter	304	0.001237104
P35846	(P35846) Folate receptor alpha precursor (FR-alpha) (Folate receptor 1) (Folate-binding protein 1)	3	Plasma Membrane	transporter	255	0.000154086
P55012	(P55012) Solute carrier family 12 member 2 (Bumetanide-sensitive sodium-(potassium)-chloride cotransporter 1) (Basolateral Na-K-Cl symporter)	1	Plasma Membrane	transporter	1205	4.6582E-06
P55014	(P55014) Solute carrier family 12 member 1 (Bumetanide-sensitive sodium-(potassium)-chloride cotransporter 2) (BSC1) (Kidney-specific Na-K-Cl symporter)	5	Plasma Membrane	transporter	1095	4.61353E-05
P59158	(P59158) Solute carrier family 12 member 3 (Thiazide-sensitive sodium-chloride cotransporter) (Na-Cl symporter)	1	Plasma Membrane	transporter	1002	1.12038E-05
P70414	(P70414) Sodium/calcium exchanger 1 precursor (Na(+)/Ca(2+)-exchange protein 1)	3	Plasma Membrane	transporter	970	4.05071E-05

Accession Number	Protein Name	Number of Identified Peptides	Subcellular Location	Protein Family	Number of Amino Acid	NSAF
Q02013	(Q02013) Aquaporin-1 (AQP-1) (Aquaporin-CHIP) (Water channel protein for red blood cells and kidney proximal tubule) (Early response protein DER2)	1	Plasma Membrane	transporter	268	0.000104723
Q8VDN2	(Q8VDN2) Sodium/potassium-transporting ATPase alpha-1 chain precursor (EC 3.6.3.9) (Sodium pump 1) (Na+/K+ ATPase 1)	23	Plasma Membrane	transporter	1023	0.000965699
Q64442	(Q64442) Sorbitol dehydrogenase (EC 1.1.1.14) (L-iditol 2-dehydrogenase) (Fragment)	19	Unknown	enzyme	375	0.001332182
Q8BVI4	(Q8BVI4) Dihydropteridine reductase (EC 1.5.1.34) (HDHPR) (Quinoid dihydropteridine reductase)	14	Unknown	enzyme	241	0.001024803
Q8VCR7	(Q8VCR7) Abhydrolase domain-containing protein 14B (CCG1-interacting factor B)	6	Unknown	enzyme	210	0.00096225
Q91XE4	(Q91XE4) Aspartoacylase-2 (EC 3.5.1.15) (Aminoacylase-3) (ACY-3) (Acylase III) (Hepatitis C virus core-binding protein 1) (HCBP1)	12	Unknown	enzyme	318	0.001359153
Q91XF0	(Q91XF0) Pyridoxine-5'-phosphate oxidase (EC 1.4.3.5) (Pyridoxamine-phosphate oxidase)	2	Unknown	enzyme	261	0.000107531
Q99LD8	(Q99LD8) NG,NG-dimethylarginine dimethylaminohydrolase 2 (EC 3.5.3.18) (Dimethylargininase-2) (Dimethylarginine dimethylaminohydrolase 2) (DDAHII)	1	Unknown	enzyme	285	3.93904E-05
Q9DBF1	(Q9DBF1) Aldehyde dehydrogenase family 7 member A1 (EC 1.2.1.3) (Antiquitin-1)	6	Unknown	enzyme	510	0.000154086
Q9JII6	(Q9JII6) Alcohol dehydrogenase [NADP+] (EC 1.1.1.2) (Aldehyde reductase) (Aldo-keto reductase family 1 member A1)	26	Unknown	enzyme	324	0.005387909
P59999	(P59999) Actin-related protein 2/3 complex subunit 4 (ARP2/3 complex 20 kDa subunit) (p20-ARC)	2	Unknown	other	167	0.000168058
Q8VC30	(Q8VC30) Dihydroxyacetone kinase (EC 2.7.1.29) (Glycerone kinase) (DHA kinase)	7	Unknown	other	578	0.000135958
Q8VE70	(Q8VE70) Programmed cell death protein 10 (TF-1 cell apoptosis-related protein 15)	1	Unknown	other	212	2.6477E-05
Q91V76	(Q91V76) Ester hydrolase C11orf54 homolog (EC 3.1.-.-)	7	Unknown	other	315	0.000427667

Accession Number	Protein Name	Number of Identified Peptides	Subcellular Location	Protein Family	Number of Amino Acid	NSAF
Q99K30	(Q99K30) Epidermal growth factor receptor kinase substrate 8-like protein 2 (Epidermal growth factor receptor pathway substrate 8-related protein 2) (EPS8-like protein 2)	9	Unknown	other	729	0.000138596
Q99KP3	(Q99KP3) Lambda-crystallin homolog	13	Unknown	other	318	0.00079431
Q9CQ89	(Q9CQ89) Protein CutA precursor (Brain acetylcholinesterase putative membrane anchor)	1	Unknown	other	177	0.000253701
Q9CR26	(Q9CR26) Protein C6orf55 homolog	2	Unknown	other	309	3.63309E-05
Q9CX00	(Q9CX00) Protein KIAA0174	2	Unknown	other	362	7.75294E-05
Q9D4J1	(Q9D4J1) EF-hand domain-containing protein 1 (Swiprosin-2)	1	Unknown	other	240	2.3388E-05
Q9JJU8	(Q9JJU8) SH3 domain-binding glutamic acid-rich-like protein	1	Unknown	other	114	0.000196952
Q9WUL7	(Q9WUL7) ADP-ribosylation factor-like protein 3	2	Unknown	other	182	0.000123365
Q9D687	(Q9D687) Sodium-dependent neutral amino acid transporter B(0) (System B(0) neutral amino acid transporter) (B(0)AT1) (Solute carrier family 6 member 19)	2	Unknown	transporter	634	5.31211E-05
